# Spherical analyzers and monochromators for resonant inelastic hard X-ray scattering: a compilation of crystals and reflections

**DOI:** 10.1107/S0909049512043154

**Published:** 2012-11-10

**Authors:** Thomas Gog, Diego M. Casa, Ayman H. Said, Mary H. Upton, Jungho Kim, Ivan Kuzmenko, XianRong Huang, Ruben Khachatryan

**Affiliations:** a Advanced Photon Source, Argonne National Laboratory, 9700 South Cass Avenue, Argonne, IL 60439, USA

**Keywords:** resonant inelastic X-ray scattering (RIXS), spherical diced analyzers

## Abstract

Resonant inelastic X-ray scattering (RIXS) experiments require special sets of near-backscattering spherical diced analyzers and high-resolution monochromators for every distinct absorption-edge energy and emission line. For the purpose of aiding the design and planning of efficient RIXS experiments, comprehensive lists of suitable analyzer reflections for silicon, germanium, α-quartz, sapphire and lithium niobate crystals were compiled for a multitude of absorption edges and emission lines. Analyzers made from lithium niobate, sapphire or α-quartz offer many choices of reflections with intrinsic resolutions currently unattainable from silicon or germanium. In some cases these materials offer higher intensities at comparable resolutions. While lithium niobate, sapphire or α-quartz analyzers are still in an early stage of development, the present compilation can serve as a computational basis for assessing expected and actual performance. With regard to high-resolution monochromators, bandpass and throughput calculations for combinations of double-crystal, high-heat-load and near-backscattering high-resolution channel-cuts were assembled. The compilation of these analyzer and monochromator data is publicly available on a website.

## Abstract 2

Resonant inelastic X-ray scattering (RIXS) experiments require special sets of near-backscattering spherical diced analyzers and high-resolution monochromators for every distinct absorption-edge energy or emission line. For the purpose of aiding the design and planning of efficient RIXS experiments, a compilation of suitable crystal materials and viable reflections for hard X-rays, together with energy resolution and throughput information, is presented.

## 1. Introduction   

With the advent of third-generation synchrotron radiation sources, resonant inelastic X-ray scattering (RIXS) has become a popular technique to study collective electron phenomena in materials of great scientific and technological significance. Near an absorption edge, the technique provides resonant enhancements of inelastic scattering signals and makes measurements feasible that would not yield enough intensity in a non-resonant mode. Most noteworthy, excitation spectra of transition metal oxides with their vast collection of novel and important properties, such as high-*T*_c_ superconductivity and multi-ferroic behavior, have been measured and interpreted very successfully with RIXS. For a comprehensive overview of the field, see a recent review article (Ament *et al.*, 2011 ▶) and references therein.

Owing to its resonant character, one of the major technical challenges for RIXS measurements is the selection of analyzers and monochromators that provide the desired resolution and intensity at a specific absorption-edge or emission-line energy. Good energy resolution requires near-backscattering analyzer reflections combined with a matched monochromator bandpass. In order to assist with an appropriate selection of these, comprehensive tables for suitable analyzer reflections in various materials are presented here. In addition, bandpass and throughput calculations for a particular monochromator concept are tabulated.

**Figure 1:**
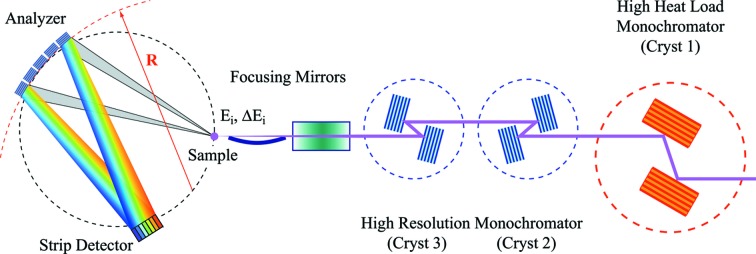
Schematic layout of a typical RIXS experiment. The incident beam is first monochromated by a high-heat-load monochromator (Cryst 1). The beam is then passed through one or two channel-cut crystals (Cryst 2 and Cryst 3) and focused onto the sample. X-ray photons with energy *E*_i_ and bandpass Δ*E*_i_ are scattered from the sample and then reflected by a diced spherical analyzer towards the detector.

**Figure 2:**
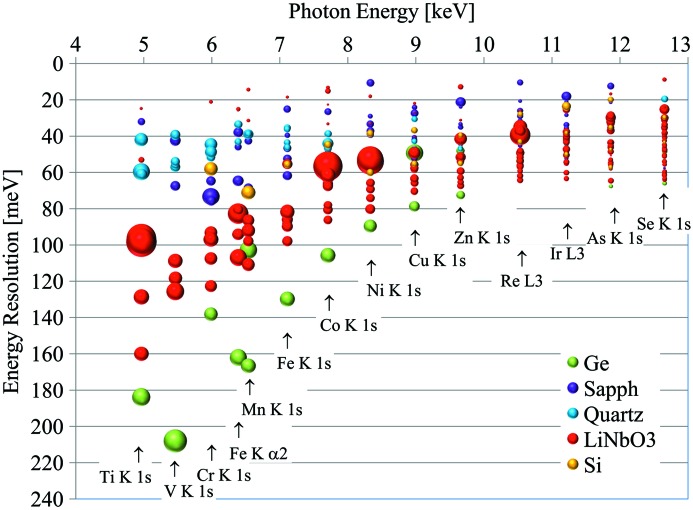
Partial map of the analyzer near-backscattering reflections. For the various relevant energies the intrinsic energy resolution is shown. The area of the markers is proportional to the integrated reflectivity.

In a typical RIXS experimental set-up (Gog *et al.*, 2009 ▶; Schwoerer-Böhning *et al.*, 1998 ▶), an incident monochromatic X-ray beam with an energy bandpass in the meV range is prepared by a succession of high-heat-load and high-resolution monochromators and micro-focused onto the sample by a set of focusing mirrors. Scattered radiation from the sample is collected by a diced spherically shaped crystal analyzer in near-backscattering configuration and redirected to a position-sensitive ‘strip’ detector (Huotari *et al.*, 2006 ▶). Sample, analyzer and detector are arranged in Rowland geometry. A schematic representation of this set-up is shown in Fig. 1 ▶. The overall energy resolution, Δ*E*_tot_, of such a configuration is given by a convolution of all its resolution elements. For near-Gaussian characteristics this convolution can be approximated by a square sum of the incident bandpass, Δ*E*_i_, corresponding to the selected monochromator combination, the intrinsic analyzer resolution, Δ*E*_a_, and geometric factors, Δ*E*_g_,
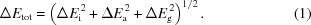
A spherical RIXS analyzer typically consists of a flat wafer of an ideal crystal material, bonded to a glass or plastic substrate, diced into square pixels of millimeter size and bent into a spherical shape of radius *R*. Overall, the analyzer is thus an assembly of many flat unstrained crystallites tangent to a spherical surface. Ignoring possible deviations from a perfect spherical shape (figure errors), the intrinsic analyzer resolution, Δ*E*_a_, is determined by the incident energy, *E*_i_, the angular reflection (Darwin) width, *W*, of the crystal reflection and the Bragg angle, Θ_B_. Namely,
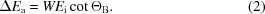
It is apparent that the energy resolution of such an analyzer is best for reflections with a small Darwin width and near-backscattering conditions, where the Bragg angle is close to 90° so that its cotangent approaches zero. The task is thus to identify near-backscattering crystal reflections for every absorption edge and emission line of interest in RIXS, yielding the best resolution at reasonable reflectivities. In the past, silicon and germanium were the preferred choices for spherical analyzers since these materials yield nearly perfect crystals. However, with advances in crystal growth, other materials are becoming viable, such as lithium niobate (LiNbO_3_), sapphire (Al_2_O_3_) and α-quartz (SiO_2_). These materials have crystal structures of lower symmetry and thus offer many more possible reflections than silicon or germanium, with numerous choices of intrinsic resolution and throughput. Fig. 2 ▶ displays a partial map of reflections for the various crystal materials in the energy *versus* intrinsic resolution plane, with the size of the symbols proportional to the integrated reflectivity. It is quite apparent that lithium niobate and sapphire offer both high-throughput as well as high-resolution reflections for the whole spectrum of energies. The fabrication of associated analyzers needs to be pursued to advance the technique.

The geometric term, Δ*E*_g_, arises from the fact that both the spatial resolution of the detector and the beam footprint on the sample are not zero but of finite extent. These spatial extensions subtend angles, ΔΘ, which in turn translate into an energy spread, Δ*E*,
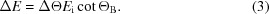
For the detector portion, ΔΘ = *p*/2*R*, where *p* is the size of a detector element and *R* is the diameter of the Rowland circle. For the footprint, ΔΘ = *s*/*R*, where *s* is the size of beam on the sample projected towards the analyzer. The present calculations only lists the detector portion, since *p* and *R* are constant for a given experimental set-up. In contrast, *s* depends on the focusing and the orientation of the sample, which may vary throughout the measurement.

The observed energy resolution of a RIXS set-up may contain additional contributions arising from imperfections of the instrument or its performance. These contributions are not addressed in the current document.

Comprehensive lists of crystal reflections in silicon, germanium, lithium niobate, sapphire and α-quartz were compiled for a multitude of absorption edges and emission lines of interest in RIXS, together with auxiliary information and geometric factors. In the same vein, data for near-backscattering silicon channel-cut crystals as one appropriate choice for high-resolution monochromators were assembled. For this monochromator concept the large angular acceptance associated with near-backscattering reflections offers optimal throughput for the incident X-ray beam with bandpass choices matched to the intrinsic resolution of selected analyzers. The compilation of these analyzer and monochromator data are made available on a publicly accessible website described in §3 and §4, respectively.

## 2. Dynamical diffraction calculations   

In this article, dynamical diffraction calculations for both analyzer and monochromator crystals are based on a formulation by Authier (2001 ▶), and were executed using the software package *Mathematica* (Wolfram, 2009 ▶). The reflectivity *I*_R_ of a crystal reflection is described as
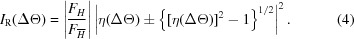
In this expression, η(ΔΘ) is a generalized angular parameter, itself a function of the deviation ΔΘ of the angle of incidence from the Bragg angle, Θ_B_. For a symmetric reflection and photon polarization perpendicular to the diffraction plane (σ-polarization), η is given by 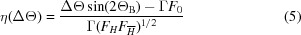
with Γ = *r*_e_λ^2^/π*V*. Here *r*_e_ is the classical electron radius, λ the wavelength and *V* the volume of the crystal unit cell. *F*_0_ and *F*_*H*_ are unit-cell structure factors associated with reciprocal lattice vectors **0** and **H**, respectively. These in turn can be written as
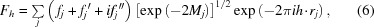
where the sum extends over all atoms of the unit cell and consists of atomic scattering factors *f*_*j*_, their anomalous dispersion corrections 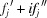, a Debye–Waller factor exp(−2*M*_*j*_) and a geometric component.

For the purpose of the present tables, structure factors were determined for room temperature (RT = 293.15 K) to reflect realistic operating conditions in an experiment. Calculations of *f*_*j*_ follow the algorithm used in the software package *XOP* (del Rio & Dejus, 2004 ▶). A Waasmaier & Kirfel-like parametrization is employed (Waasmaier & Kirfel, 1995 ▶), as evaluated by Kissel (2000 ▶) using modified relativistic form factors. Dispersion corrections 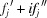 were taken from the database at the Center for X-ray Optics (CXRO) at Lawrence Berkeley National Laboratory (Henke *et al.*, 1993 ▶). Debye–Waller factors in the form of (see Als-Nielsen & McMorrow, 2001 ▶, or other text books on X-ray physics)
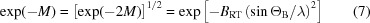
are considered, where (sinΘ_B_/λ) is proportional to the momentum transfer and mean-square atomic vibrational amplitudes at room temperature, *B*_RT_, were derived from X-ray diffraction measurements. In particular, the vibrational amplitudes and associated Debye temperatures, Θ_D_, assembled in Table 1 ▶, were used. For the geometric portion of the structure factors, some crystallographic data from *XOP* were used.

An important parameter in the present context is the Darwin width of a reflection. It is given by
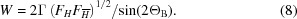



## 3. Spherical analyzer tables   

**Figure 3:**
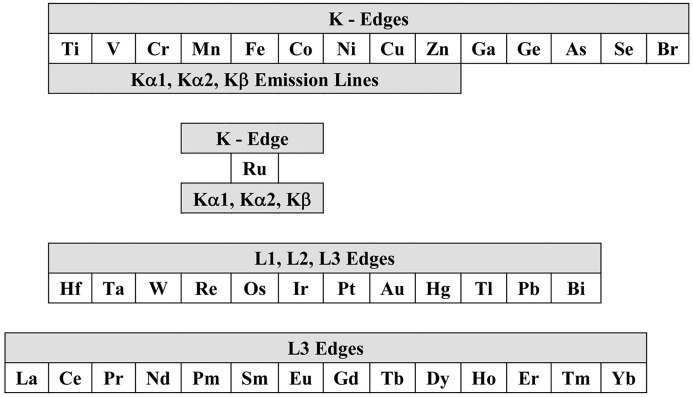
Absorption edges and emission lines for a selection of chemical elements included in the present data compilation.

The tables for the spherical analyzer are located at http://www.aps.anl.gov/Sectors/Sector30/AnalyzerAtlas/AnalyzerAtlas.html and constitute a compilation of near-backscattering reflections in silicon, germanium, lithium niobate (LiNbO_3_), sapphire (Al_2_O_3_) and quartz (SiO_2_) for Bragg angles in the range 70° to 90°.^1^ Absorption edges and emission lines for a selection of chemical elements of interest in RIXS, which are included in the present compilation, are indicated in Fig. 3 ▶.

**Figure 4:**
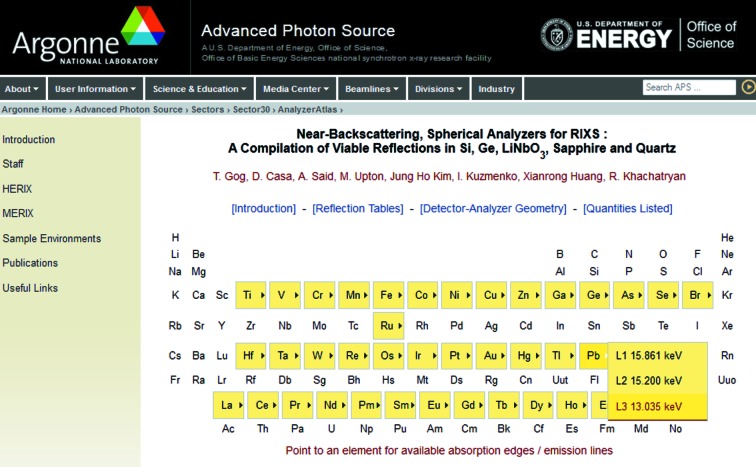
Partial screenshot of the top-level spherical analyzer compilation. Pointing to an element highlighted in yellow will open a menu with available absorption-edge or emission-line data.

Partial screenshots for the top-level menu and an example of a listing for the Cu *K*-edge are shown in Figs. 4 ▶ and 5 ▶. These tables are divided into two groups of crystals with Si and Ge at the top (highlighted in yellow), lithium niobate, sapphire and quartz at the bottom. Within these two groups reflections are arranged from top to bottom by strength, according to the integrated reflectivity 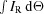. For lithium niobate, sapphire and quartz all equivalent reflections are listed, while for Si and Ge equivalent reflections are only listed if their indices are not simple permutations or inversions of the parent reflection. The following quantities are included in the tables:

*Backscattering energy*, *E*_B_ (keV). X-ray energy for which the incident beam is reflected at a Bragg angle of Θ_B_ = 90°. It is given by
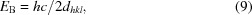
where *h* is Planck’s constant, *c* is the speed of light and *d*_*hkl*_ is the diffraction plane spacing. These are the lowest-energy photons that can be reflected by a particular analyzer.

*Integrated reflectivity*, 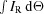 (µrad). As a measure of the reflection strength this quantity represents a numerical integration of the dynamical reflectivity over the entire rocking curve.

*Angular reflection (Darwin) width*, *W* (µrad). Intrinsic, dynamical (Darwin) width associated with the symmetric reflection.

*Change in energy with angle*, d*E*/dΘ = *E*_i_cotΘ (meV µrad^−1^). From the differential Bragg law, this quantity serves as the conversion factor from angular to energy width in convenient units and is included for guidance.

*Intrinsic energy resolution*, Δ*E*_a_ (meV). Energy resolution of the analyzer reflection owing to its intrinsic (Darwin) width, Δ*E*_a_ = *W* *E*_i_cotΘ_B_.

*Geometric contribution*, Δ*E*_g_ (meV). Geometric contribution to the energy resolution, based on the analyzer radius *R* and the detector pitch *p*: Δ*E*_g_ = *E*_i_cot(Θ_B_)*p*/2*R*. For the present tables the detector pitch is assumed to be *p* = 50 µm (Dectris ‘Mythen’ detector) while the analyzer radius is *R* = 2 m.

*Combined intrinsic and geometric energy resolution*, Δ*E*_t_ (meV). Δ*E*_t_ = (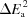 + 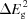)^1/2^.

## 4. High-resolution monochromator tables   

**Figure 6:**
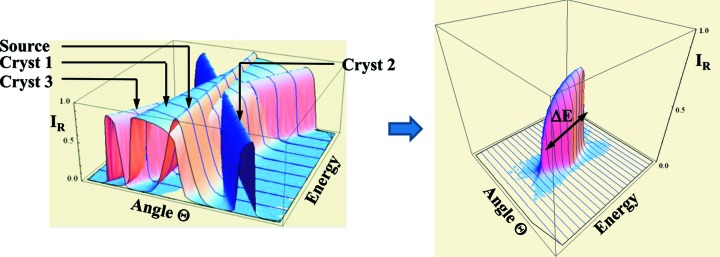
The product of a typical Gaussian source distribution and crystal reflectivities (left panel) leads to a reflectivity profile (right panel), which is the basis for the bandpass and throughput calculations.

The tables for high-resolution channel-cut monochromator crystals are located at http://www.aps.anl.gov/Sectors/Sector30/AnalyzerAtlas/MonoAtlas.html and were assembled for combinations of a Si or diamond high-heat-load monochromator followed by one or two pairs of high-resolution Si channel-cut crystals, as indicated in Fig. 1 ▶.^2^ The rationale for this crystal arrangement is inspired by the fact that the angular acceptance of the high-resolution portion is proportional to 1/sin(2Θ_B_) [equation (5)]. This term becomes large for near-backscattering conditions and thus guarantees an optimal throughput, while many choices of reflections arise to closely match the bandpass to the analyzer resolution. The bandpass and throughput data were calculated by multiplying a Gaussian X-ray source distribution with all pertinent dynamical crystal reflectivities as shown in Fig. 6 ▶. The source distribution is given by
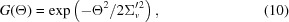
where
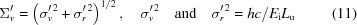
are the vertical combined, electron and photon beam divergences, respectively. For a typical undulator beamline at the Advanced Photon Source (APS), 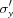 = 3.3 µrad and the undulator length *L*_u_ is 4.8 m.

**Figure 7:**
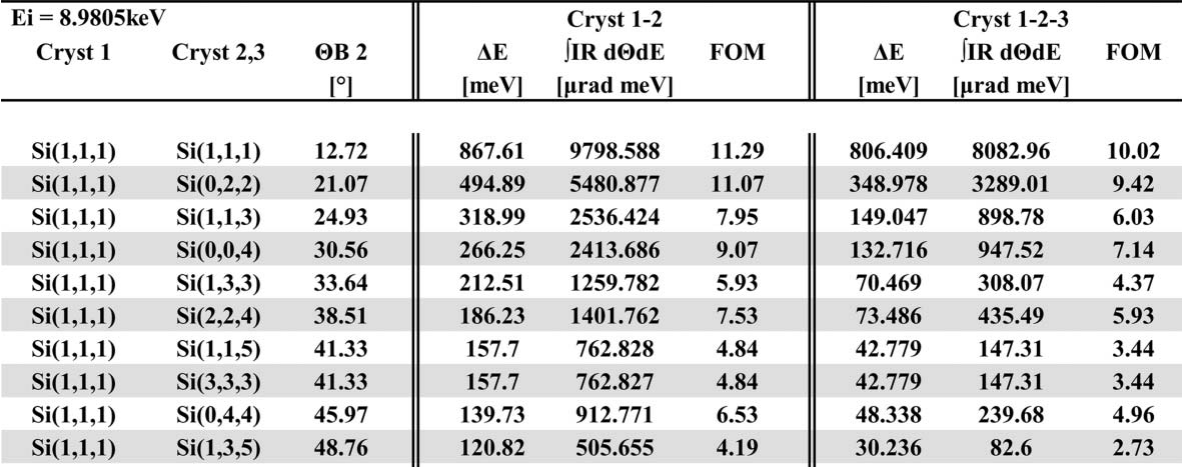
Partial screenshot of a monochromator listing for an absorption-edge energy of 8.9805 keV (Cu *K*-edge).

The intensity profile resulting from the product is numerically integrated over angle and energy. A partial screenshot of a listing at the Cu *K*-edge is shown in Fig. 7 ▶. The following quantities are included in the tables:

*Overall energy resolution*, Δ*E* (meV), for the entire four- or six-bounce combination of crystals (FWHM).

*Integrated throughput*, 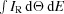 (µrad meV).

*Figure-of-merit (FOM)* (µrad), ratio of throughput per energy resolution.

While parts of the calculations presented here involve parameters specific to a particular beamline or facility, the resulting tables are general enough to be a useful guide for RIXS beamlines everywhere. In the spherical analyzer section only the last two columns contain such parameters. Here the term Δ*E*_g_ scales linearly with *p* and 1/2*R* as prescribed above and can easily be modified for a different geometry. In the same vein, the term Δ*E*_t_ can be expanded to include additional terms in a square-sum fashion. The monochromator tables involve source parameters specific to the APS. Nevertheless, they still provide useful guidance generally in as much as source characteristics of third-generation synchrotron sources are rather similar.

## 5. Example of the utility of the tables: 5*d* TMOs at the Ir *L*_3_ absorption edge   

For the purpose of illustrating the utility of the analyzer and monochromator tables presented here, a specific example of an optical configuration for RIXS measurements at the Ir *L*_3_ absorption edge (*E* = 11.215 keV) is discussed. In recent years there has been a rapidly increasing interest in 5*d* transition metal oxides (TMOs), in particular the iridium compounds. These materials feature a rich spectrum of exotic electronic excitations, which are expected to lead to many novel phenomena of scientific and technological significance (Kim *et al.*, 2012 ▶; Liu *et al.*, 2012 ▶). In order to observe these excitations with RIXS, an energy resolution of 30 to 40 meV or better is required, substantially exceeding the typical conditions for this technique prior to this effort.

As explained in §1, the total energy resolution of the experimental set-up shown in Fig. 1 ▶ is chiefly given by the square root of a sum of squares, consisting of monochromator bandwidth, intrinsic analyzer resolution and geometric contributions owing to the detector pitch and source size at the sample. According to the present tables, a monochromator as shown in Fig. 1 ▶ and using a combination of Si(111) followed by either one or two Si(448) channel-cut crystals has a bandwidth of Δ*E*_i_ = 15.8 meV and 8.956 meV, respectively. Furthermore, a Si(448) reflection at 11.215 keV has an intrinsic resolution of Δ*E* = 14.57 meV, and a geometric detector contribution of Δ*E*_g_ = 10.47 meV, given a Rowland circle with a diameter of 2 m and a detector pitch of 50 µm. The associated source contribution from a beam size of 50 µm is Δ*E*_s_ = 20.95 meV. Altogether, the predicted energy resolution for such an experimental RIXS set-up is then Δ*E*_tot_ = 31.8 meV and 29.0 meV for the single and double monochromator combinations, respectively.

**Figure 8:**
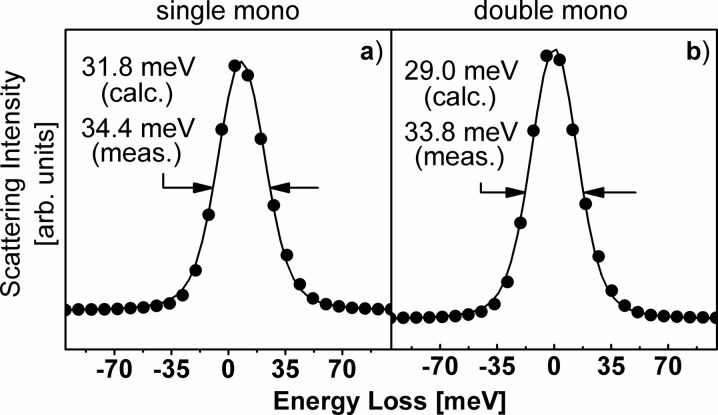
Measurements of elastic lines from a ‘standard’ scatterer, Scotch Brand #810 Magic Mending Tape. Measured FWHM and calculated values based on present tables are well within 10% of each other.

The two configurations described above have been implemented on RIXS instruments at the undulator beamlines 9-ID and 30-ID at the APS at Argonne National Laboratory. Measurements of elastic lines from a ‘standard’ scatterer, Scotch Brand #810 Magic Mending Tape, which is composed of a cellulose acetate carrier and an acrylic polymer adhesive, are shown in Fig. 8 ▶. The dots represent measured data and the line is a Voigt-function fit. The full width at half-maximum was determined to be 34.4 meV and 33.8 meV, respectively, for the single and double monochromator, well within 10% of what was predicted based on the tables.

## 6. Conclusions   

Identifying near-backscattering reflections for spherical analyzers combined with matching monochromator characteristics, yielding a required energy-resolution with optimal throughput, has been a daunting challenge for RIXS experiments. The present compilation of analyzer reflections and channel-cut monochromator combinations was designed to aid the selection of crystals suitable for RIXS measurements at a particular absorption edge or for a particular emission line. In addition, the inclusion of lithium niobate, sapphire and α-quartz for spherical analyzers provides a theoretical basis for the characteristics that can be expected from these unusual crystal materials and can help to assess their actual performance in an experiment.

## Supplementary Material

Spherical analyzer tables. DOI: 10.1107/S0909049512043154/ie5087sup1.pdf

High-resolution monochromator tables. DOI: 10.1107/S0909049512043154/ie5087sup2.pdf

## Figures and Tables

**Figure 5:**
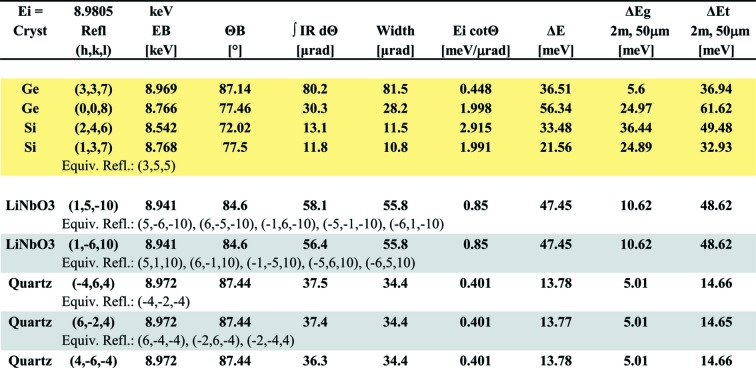
Partial screenshot of the spherical analyzer listing for an absorption-edge energy of 8.9805 keV (Cu *K*-edge).

**Table 1:** Atomic vibrational amplitudes and Debye temperatures used in the dynamical diffraction calculations The origin of these data are indicated by the references.

Crystal material	*B*_RT_ (Å^2^)	Θ_D_ (K)
Si	0.4632^*a*^	530.82
Ge	0.5661^*b*^	290.03
LiNbO_3_	Li: 0.5264^*c*^	1118.44
	Nb: 0.4174	298.90
	O: 0.5738	643.27
Al_2_O_3_	Al: 0.1921^*d*^	897.40
	O: 0.2271	1122.51
SiO_2_	Si: 0.4874^*e*^	516.38
	O: 0.9949	476.16

^*a*^Deutsch & Hart (1985 ▶). ^*b*^Deutsch *et al.* (1990 ▶). ^*c*^Etschmann & Ishizawa (2001 ▶). ^*d*^Kirfel & Eichhorn (1990 ▶). ^*e*^LePage *et al.* (1980 ▶).

## Footnotes

1 The spherical analyzer tables are available in pdf format from the IUCr electronic archives (Reference: IE5087). Services for accessing these data are described at the back of the journal.

2 The high-resolution monochromator tables are available in pdf format from the IUCr electronic archives (Reference: IE5087). Services for accessing these data are described at the back of the journal.
