# Supplementary material for: Spherical analyzers and monochromators for resonant inelastic hard X-ray scattering: a compilation of crystals and reflections
Source: J Synchrotron Radiat. 2012 Nov 10;20(Pt 1):74–9. doi: 10.1107/S0909049512043154 (PMC3943536; doi:10.1107/S0909049512043154)
Supplement: Supplementary file 2 [file s-20-00074-sup2.pdf]

Argonne NATIONAL LABORATORY

Advanced Photon Source
A U.S. Department of Energy, Office of Science,
Office of Basic Energy Sciences national synchrotron x-ray research facility

U.S. DEPARTMENT OF ENERGY | Office of Science

About ▾ | User Information ▾ | Science & Education ▾ | Media Center ▾ | Beamlines ▾ | Divisions ▾ | Industry | Search APS ...

Argonne Home › Advanced Photon Source › Sectors › Sector30 › AnalyzerAtlas ›

- Introduction
- Staff
- HERIX
- MERIX
- Sample Environments
- Publications
- Useful Links

High-Resolution Si Channel-Cut Monochromators for RIXS : A Compilation of Viable Crystal Combinations

T. Gog, D. Casa, A. Said, M. Upton, Jung Ho Kim, I. Kuzmenko, Xianrong Huang, R. Khachatryan

[Introduction] - [Tables] - [Monochromator Geometry] - [Quantities Listed]

H																	He
Li	Be											B	C	N	O	F	Ne
Na	Mg											Al	Si	P	S	Cl	Ar
K	Ca	Sc	Ti	V	Cr	Mn	Fe	Co	Ni	Cu	Zn	Ga	Ge	As	Se	Br	Kr
Rb	Sr	Y	Zr	Nb	Mo	Tc	Ru	Rh	Pd	Ag	Cd	In	Sn	Sb	Te	I	Xe
Cs	Ba	Lu	Hf	Ta	W	Re	Os	Ir	Pt	Au	Hg	Tl	Pb	Bi	Po	At	Rn
Fr	Ra	Lr	Rf	Db	Sg	Bh	Hs	Mt	Ds	Rg	Cn	Uut	Fl	Uup	Lv	Uus	Uuo
		La	Ce	Pr	Nd	Pm	Sm	Eu	Gd	Tb	Dy	Ho	Er	Tm	Yb		
		Ac	Th	Pa	U	Np	Pu	Am	Cm	Bk	Cf	Es	Fm	Md	No		

Point to an Element for available Absorption Edges / Emission Lines

contact: Thomas Gog, last updated: 09.October.2012

The Advanced Photon Source is an Office of Science User Facility operated for the U.S. Department of Energy Office of Science by Argonne National Laboratory.

UChicago Argonne LLC | Privacy & Security Notice | Contact Us | Site Map

Introduction

The tables presented here were assembled for combinations of a Si- or Diamond high-heat-load monochromator followed by one or two pairs of high-resolution Si channel-cut crystals, as indicated under [Monochromator Geometry]. The rationale for this crystal arrangement is inspired by the fact that the angular acceptance of the high-resolution portion is proportional to $1/\sin(2\ \Theta_B)$. This term becomes large for near-backscattering conditions and thus guarantees an optimal throughput, while many choices of reflections arise to closely match the band pass to the analyzer resolution. The band pass and throughput data were calculated by multiplying a Gaussian x-ray source distribution with all pertinent dynamical crystal reflectivities as shown under [Tables] and numerically integrating over angle and energy.
The monochromator tables involve source parameters specific to the APS. Nevertheless, they still provide useful guidance generally in as much as source characteristics of 3rd generation synchrotron sources are rather similar.

Tables

The bandpass- and throughput data in the monochromator tables were compiled by superposing a Gaussian source distribution (see below) and all pertinent dynamical crystal reflectivities. The resulting intensity profile is then numerically integrated over angle and energy.

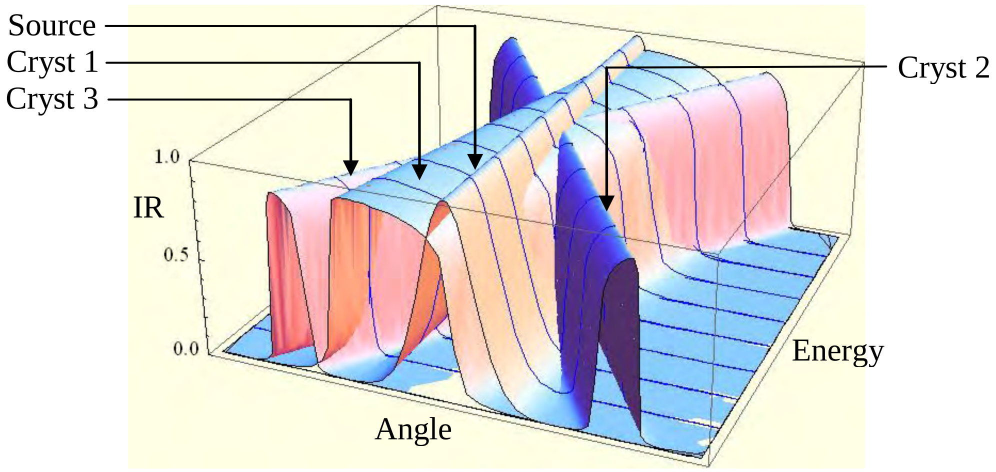

Source distribution and crystal reflectivities as functions of angle and energy

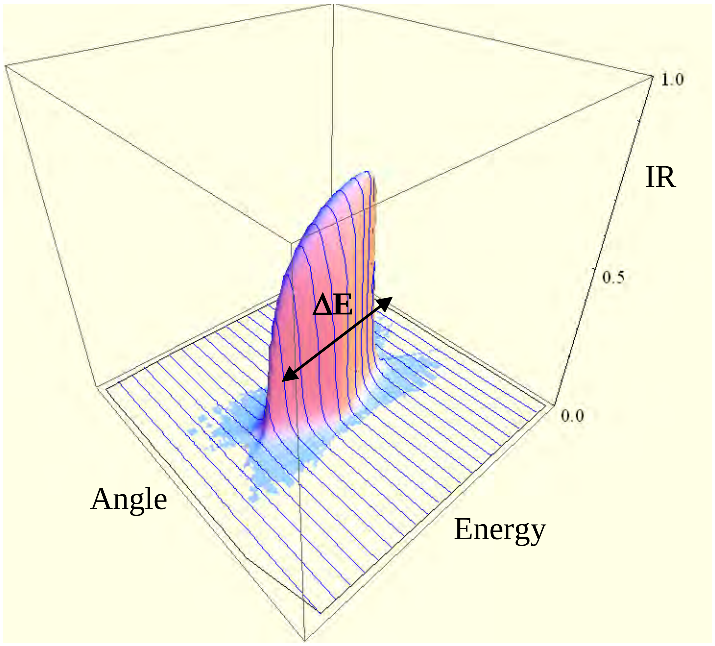

Intensity profile as a function of angle and energy, resulting from superposition of source distribution and crystal reflectivities

Source Parameters

Monochromator tables are based on a typical vertical source distibution for an undulator beamline at the Advanced Photon Source of Argonne Nat'l Laboratory.

Pertinent parameters are

Undulator Length:

$$L_u = 4.8m$$

Electron beam divergence, vertical:

$$\sigma_y^' = 3.3\mu rad$$

Photon beam divergence, vertical, incident energy E_i :

$$\sigma_r^' = \sqrt{hc / E_i L_u}$$

Combined beam divergence, vertical:

$$\Sigma_y^' = \sqrt{\sigma_y^{'2} + \sigma_r^{'2}}$$

Gaussian angular source distribution:

$$G(\theta) = e^{-\theta^2 / 2\Sigma_y^2}$$

Monochromator Geometry

Monochromator assemblies consisting of two combinations of channel-cut crystals are considered:

1. One pair of Si(1,1,1) or Diamond(1,1,1) crystals followed by one pair of Si(h,k,l) crystals in dispersive configuration
2. One pair of Si(1,1,1) or Diamond(1,1,1) crystals followed by two pairs of Si(h,k,l) in dispersive configuration

Crystals can be realized as monolithic or virtual channel-cuts.

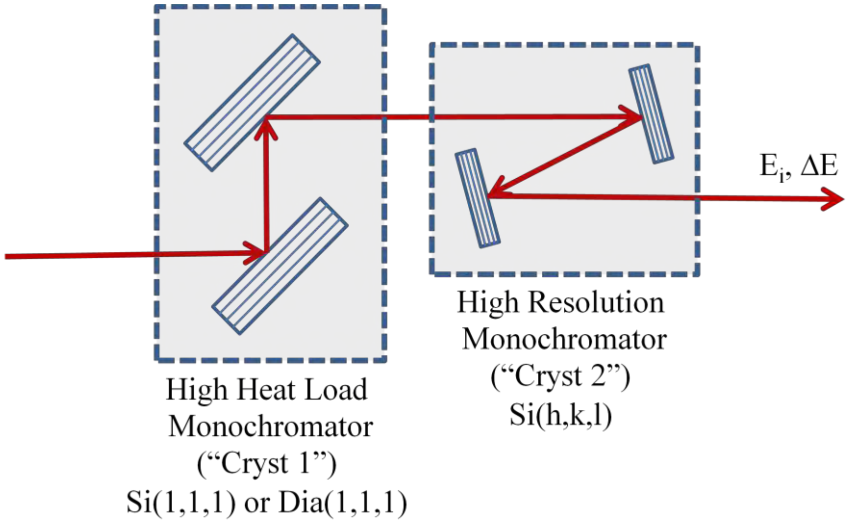

Monochromator assembly consisting of two pairs of crystals

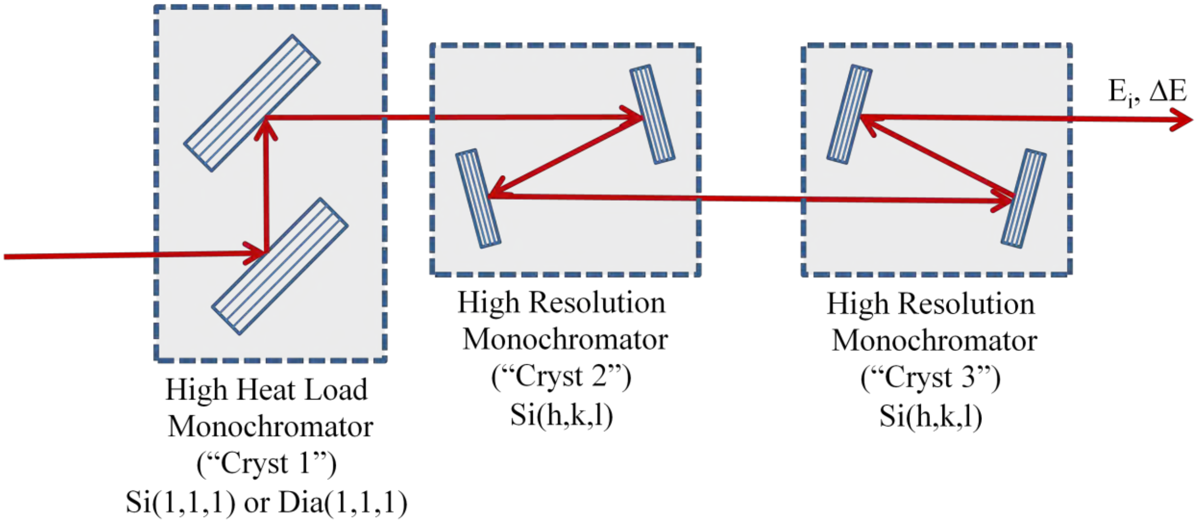

Monochromator assembly consisting of three pairs of crystals

Quantities Listed in the Tables

Cryst 1
Type of first crystal pair, corresponding to the high-heat-load portion of the monochromator assembly. Types considered are Si(1,1,1) and Diamond(1,1,1)

Cryst 2, 3
Type of second and third crystal pair, corresponding to the high-resolution portion of the monochromator assembly.

BraggAngle Θ_B 2
Bragg angle of the second and third pair of crystals

Bandpass ΔE
Energy bandpass of the monochromator assembly, as determined by numerically integrating the superposition of source distribution and dynamical reflectivities in angle, yielding and an overall energy profile.

Integrated Reflectivity, $\int I_R \, d\Theta dE$

As a measure of photon throughput the superposition of source distribution and dynamical reflectivities is numerically integrated over angle and energy.

FOM
Figure-of-merit defined as ratio of Integrated Reflectivity / Bandpass

Ei = 4.966 keV			Cryst 1-2			Cryst 1-2-3		
Cryst 1	Cryst 2,3	ΘB 2 [°]	ΔE [meV]	∫IR dΘdE [µrad meV]	FOM	ΔE [meV]	∫IR dΘdE [µrad meV]	FOM
Si(1,1,1)	Si(1,1,1)	23.46	457.56	4435.187	9.69	400.066	2812.93	7.03
Si(1,1,1)	Si(0,2,2)	40.55	250.71	2623.213	10.46	178.09	1476.93	8.29
Si(1,1,1)	Si(1,1,3)	49.67	132.12	1026.816	7.77	82.295	371.74	4.52
Si(1,1,1)	Si(0,0,4)	66.84	107.22	1073.033	10.01	74.93	560.55	7.48
Dia(1,1,1)	Si(1,1,1)	23.46	289.29	3638.412	12.58	279.845	2377.25	8.49
Dia(1,1,1)	Si(0,2,2)	40.55	215.48	2965.207	13.76	183.845	2013.21	10.95
Dia(1,1,1)	Si(1,1,3)	49.67	127.05	1350.261	10.63	83.175	530.03	6.37
Dia(1,1,1)	Si(0,0,4)	66.84	106.91	1480.716	13.85	76.014	802.7	10.56

Ei = 5.465 keV			Cryst 1-2			Cryst 1-2-3		
Cryst 1	Cryst 2,3	ΘB 2 [°]	ΔE [meV]	∫IR dΘdE [µrad meV]	FOM	ΔE [meV]	∫IR dΘdE [µrad meV]	FOM
Si(1,1,1)	Si(1,1,1)	21.21	515.54	5248.741	10.18	364.993	3532.59	9.68
Si(1,1,1)	Si(0,2,2)	36.21	284.73	3065.136	10.76	206.525	1746.51	8.46
Si(1,1,1)	Si(1,1,3)	43.85	153.62	1247.406	8.12	91.552	450.16	4.92
Si(1,1,1)	Si(0,0,4)	56.66	126.18	1270.959	10.07	84.234	611.44	7.26
Si(1,1,1)	Si(1,3,3)	65.56	74.06	583.52	7.88	45.747	204.89	4.48
Dia(1,1,1)	Si(1,1,1)	21.21	319	4092.182	12.83	312.145	2858.4	9.16
Dia(1,1,1)	Si(0,2,2)	36.21	236.73	3205.606	13.54	208.107	2261.78	10.87
Dia(1,1,1)	Si(1,1,3)	43.85	143.04	1500.676	10.49	92.545	608.82	6.58
Dia(1,1,1)	Si(0,0,4)	56.66	122.12	1608.522	13.17	84.879	839.76	9.89
Dia(1,1,1)	Si(1,3,3)	65.56	73.11	767.868	10.5	46.078	278.83	6.05

Ei = 5.989 keV			Cryst 1-2			Cryst 1-2-3		
Cryst 1	Cryst 2,3	ΘB 2 [°]	ΔE [meV]	∫IR dΘdE [μrad meV]	FOM	ΔE [meV]	∫IR dΘdE [μrad meV]	FOM
Si(1,1,1)	Si(1,1,1)	19.28	574.11	6059.982	10.56	515.597	4283.16	8.31
Si(1,1,1)	Si(0,2,2)	32.62	318.68	3503.538	10.99	231.072	2033.27	8.8
Si(1,1,1)	Si(1,1,3)	39.21	176.8	1472.468	8.33	100.884	530.12	5.25
Si(1,1,1)	Si(0,0,4)	49.67	145.58	1473.936	10.12	91.697	679.55	7.41
Si(1,1,1)	Si(1,3,3)	56.18	92.74	697.779	7.52	48.594	214.85	4.42
Si(1,1,1)	Si(2,2,4)	69.02	81.08	830.658	10.25	54.948	405.53	7.38
Si(1,1,1)	Si(3,3,3)	82.03	46.11	407.017	8.83	33.86	188.4	5.56
Dia(1,1,1)	Si(1,1,1)	19.28	349.7	4526.986	12.95	344.599	3330.11	9.66
Dia(1,1,1)	Si(0,2,2)	32.62	257.8	3434.086	13.32	231.651	2523.95	10.9
Dia(1,1,1)	Si(1,1,3)	39.21	159.06	1643.564	10.33	100.847	694.08	6.88
Dia(1,1,1)	Si(0,0,4)	49.67	136.77	1724.278	12.61	92.59	878.95	9.49
Dia(1,1,1)	Si(1,3,3)	56.18	89.68	848.757	9.46	48.838	279.73	5.73
Dia(1,1,1)	Si(2,2,4)	69.02	79.77	1037.509	13.01	55.288	527.12	9.53
Dia(1,1,1)	Si(3,3,3)	82.03	45.98	521.117	11.33	33.953	244.42	7.2

Ei = 6.539 keV			Cryst 1-2			Cryst 1-2-3		
Cryst 1	Cryst 2,3	ΘB 2 [°]	ΔE [meV]	∫IR dΘdE [µrad meV]	FOM	ΔE [meV]	∫IR dΘdE [µrad meV]	FOM
Si(1,1,1)	Si(1,1,1)	17.6	633.67	6858.493	10.82	577.671	5049.09	8.74
Si(1,1,1)	Si(0,2,2)	29.59	352.95	3935.881	11.15	255.517	2313.87	9.06
Si(1,1,1)	Si(1,1,3)	35.38	201.76	1697.781	8.41	109.768	613.81	5.59
Si(1,1,1)	Si(0,0,4)	44.29	166.47	1674.309	10.06	100.747	739.2	7.34
Si(1,1,1)	Si(1,3,3)	49.54	113.47	813.503	7.17	52.959	230.68	4.36
Si(1,1,1)	Si(2,2,4)	58.78	98.01	950.379	9.7	57.637	393.21	6.82
Si(1,1,1)	Si(3,3,3)	65.1	65.38	478.987	7.33	32.617	139.2	4.27
Si(1,1,1)	Si(0,4,4)	80.92	56.71	607.893	10.72	42.146	347.11	8.24
Dia(1,1,1)	Si(1,1,1)	17.6	381.44	4940.407	12.95	378.276	3803.1	10.05
Dia(1,1,1)	Si(0,2,2)	29.59	277.08	3658.421	13.2	252.866	2785.54	11.02
Dia(1,1,1)	Si(1,1,3)	35.38	175.75	1769.628	10.07	109.817	764.88	6.97
Dia(1,1,1)	Si(0,0,4)	44.29	151.87	1829.747	12.05	100.741	921.58	9.15
Dia(1,1,1)	Si(1,3,3)	49.54	106.77	924.849	8.66	52.709	289.51	5.49
Dia(1,1,1)	Si(2,2,4)	58.78	93.79	1105.661	11.79	57.989	488.23	8.42
Dia(1,1,1)	Si(3,3,3)	65.1	63.51	568.916	8.96	32.78	173.99	5.31
Dia(1,1,1)	Si(0,4,4)	80.92	56.32	740.287	13.14	42.19	431.69	10.23

Ei = 7.112 keV			Cryst 1-2			Cryst 1-2-3		
Cryst 1	Cryst 2,3	ΘB 2 [°]	ΔE [meV]	∫IR dΘdE [µrad meV]	FOM	ΔE [meV]	∫IR dΘdE [µrad meV]	FOM
Si(1,1,1)	Si(1,1,1)	16.14	693.87	7633.501	11	639.45	5809.21	9.08
Si(1,1,1)	Si(0,2,2)	27	387.01	4351.48	11.24	278.105	2586.21	9.3
Si(1,1,1)	Si(1,1,3)	32.16	228.35	1918.353	8.4	119.172	686.78	5.76
Si(1,1,1)	Si(0,0,4)	39.94	188.91	1870.231	9.9	108.026	805.49	7.46
Si(1,1,1)	Si(1,3,3)	44.39	135.71	930.279	6.85	58.032	250.89	4.32
Si(1,1,1)	Si(2,2,4)	51.84	117.21	1070.202	9.13	61.226	397.42	6.49
Si(1,1,1)	Si(3,3,3)	56.51	87.15	551.603	6.33	35.352	132.71	3.75
Si(1,1,1)	Si(0,4,4)	65.22	73.69	686.78	9.32	40.773	265.03	6.5
Si(1,1,1)	Si(1,3,5)	71.71	48.79	358.653	7.35	24.258	103.57	4.27
Dia(1,1,1)	Si(1,1,1)	16.14	414.29	5325.07	12.85	412.176	4254.31	10.32
Dia(1,1,1)	Si(0,2,2)	27	296.77	3857.626	13	273.707	3020.17	11.03
Dia(1,1,1)	Si(1,1,3)	32.16	192.6	1884.621	9.78	118.44	828.1	6.99
Dia(1,1,1)	Si(0,0,4)	39.94	167.12	1927.931	11.54	108.55	970.04	8.94
Dia(1,1,1)	Si(1,3,3)	44.39	124.23	992.013	7.99	57.754	303.21	5.25
Dia(1,1,1)	Si(2,2,4)	51.84	109.28	1167.903	10.69	60.98	481.89	7.9
Dia(1,1,1)	Si(3,3,3)	56.51	83.01	614.491	7.4	35.248	161.62	4.59
Dia(1,1,1)	Si(0,4,4)	65.22	70.48	779.76	11.06	40.775	320.1	7.85
Dia(1,1,1)	Si(1,3,5)	71.71	47.2	414.011	8.77	23.712	127.56	5.38

Ei = 7.709 keV			Cryst 1-2			Cryst 1-2-3		
Cryst 1	Cryst 2,3	ΘB 2 [°]	ΔE [meV]	∫IR dΘdE [µrad meV]	FOM	ΔE [meV]	∫IR dΘdE [µrad meV]	FOM
Si(1,1,1)	Si(1,1,1)	14.86	752.91	8385.419	11.14	699.541	6561.21	9.38
Si(1,1,1)	Si(0,2,2)	24.76	422.63	4753.988	11.25	300.043	2834.48	9.45
Si(1,1,1)	Si(1,1,3)	29.41	256.79	2131.818	8.3	128.839	762.45	5.92
Si(1,1,1)	Si(0,0,4)	36.32	212.99	2059.542	9.67	114.4	865.86	7.57
Si(1,1,1)	Si(1,3,3)	40.2	159.48	1043.489	6.54	61.683	271.53	4.4
Si(1,1,1)	Si(2,2,4)	46.5	138.69	1181.876	8.52	65.44	403.4	6.16
Si(1,1,1)	Si(3,3,3)	50.3	109.49	623.465	5.69	37.329	135.69	3.63
Si(1,1,1)	Si(0,4,4)	56.89	94.86	763.191	8.05	42.648	242.95	5.7
Si(1,1,1)	Si(1,3,5)	61.16	73.22	407.923	5.57	25.468	85.98	3.38
Si(1,1,1)	Si(0,2,6)	69.46	59.38	526.787	8.87	30.715	189.67	6.18
Si(1,1,1)	Si(3,3,5)	76.15	38.18	281.96	7.39	18.872	82.39	4.37
Dia(1,1,1)	Si(1,1,1)	14.86	447.84	5682.159	12.69	2311.84	4706.71	2.04
Dia(1,1,1)	Si(0,2,2)	24.76	315.42	4042.528	12.82	296.028	3235.89	10.93
Dia(1,1,1)	Si(1,1,3)	29.41	209.75	1994.335	9.51	127.804	889.97	6.96
Dia(1,1,1)	Si(0,0,4)	36.32	183.02	2011.12	10.99	116.076	1013.99	8.74
Dia(1,1,1)	Si(1,3,3)	40.2	141.58	1056.141	7.46	61.905	319.93	5.17
Dia(1,1,1)	Si(2,2,4)	46.5	125.37	1223.153	9.76	65.371	475.4	7.27
Dia(1,1,1)	Si(3,3,3)	50.3	102.02	655.216	6.42	37.273	161.45	4.33
Dia(1,1,1)	Si(0,4,4)	56.89	88.02	819.368	9.31	43.108	285.47	6.62
Dia(1,1,1)	Si(1,3,5)	61.16	70	443.023	6.33	25.271	101.12	4
Dia(1,1,1)	Si(0,2,6)	69.46	56.49	581.629	10.3	30.712	223.01	7.26
Dia(1,1,1)	Si(3,3,5)	76.15	36.64	315.472	8.61	18.834	97.41	5.17

Ei = 8.333 keV			Cryst 1-2			Cryst 1-2-3		
Cryst 1	Cryst 2,3	ΘB 2 [°]	ΔE [meV]	∫IR dΘdE [µrad meV]	FOM	ΔE [meV]	∫IR dΘdE [µrad meV]	FOM
Si(1,1,1)	Si(1,1,1)	13.72	804.51	9133.581	11.35	750.019	7332.09	9.78
Si(1,1,1)	Si(0,2,2)	22.79	458.55	5105.405	11.13	323.973	3075.87	9.49
Si(1,1,1)	Si(1,1,3)	27.02	287.09	2339.362	8.15	139.319	832.19	5.97
Si(1,1,1)	Si(0,0,4)	33.22	238.89	2239.722	9.38	122.389	919.56	7.51
Si(1,1,1)	Si(1,3,3)	36.66	185.16	1154.2	6.23	66.306	290.34	4.38
Si(1,1,1)	Si(2,2,4)	42.15	161.62	1294.739	8.01	70.149	419.82	5.98
Si(1,1,1)	Si(3,3,3)	45.38	132.89	694.365	5.23	39.765	140.95	3.54
Si(1,1,1)	Si(0,4,4)	50.79	116.56	840.414	7.21	45.724	238.88	5.22
Si(1,1,1)	Si(1,3,5)	54.13	96.72	456.694	4.72	27.315	81.49	2.98
Si(1,1,1)	Si(0,2,6)	60.03	82.66	581.48	7.03	32.294	158.55	4.91
Si(1,1,1)	Si(3,3,5)	63.93	66.33	317.45	4.79	19.409	57.5	2.96
Si(1,1,1)	Si(4,4,4)	71.63	51.68	419.679	8.12	24.161	133.58	5.53
Si(1,1,1)	Si(1,1,7)	78.02	32.95	228.973	6.95	15.181	61.87	4.08
Dia(1,1,1)	Si(1,1,1)	13.72	483.35	6014.508	12.44	2153.54	5101.33	2.37
Dia(1,1,1)	Si(0,2,2)	22.79	338.9	4186.987	12.35	317.618	3422.28	10.77
Dia(1,1,1)	Si(1,1,3)	27.02	227.9	2094.649	9.19	138.372	954.47	6.9
Dia(1,1,1)	Si(0,0,4)	33.22	199.28	2086.71	10.47	123.502	1053.77	8.53
Dia(1,1,1)	Si(1,3,3)	36.66	159.1	1110.516	6.98	65.935	335.97	5.1
Dia(1,1,1)	Si(2,2,4)	42.15	141.68	1278.961	9.03	70.844	485.04	6.85
Dia(1,1,1)	Si(3,3,3)	45.38	120.66	695.396	5.76	40.206	160.1	3.98
Dia(1,1,1)	Si(0,4,4)	50.79	105.81	856.851	8.1	45.36	276.25	6.09
Dia(1,1,1)	Si(1,3,5)	54.13	90.99	471.015	5.18	27.442	93.71	3.41
Dia(1,1,1)	Si(0,2,6)	60.03	76.83	607.845	7.91	31.693	184.59	5.82
Dia(1,1,1)	Si(3,3,5)	63.93	63.38	335.339	5.29	19.739	65.27	3.31
Dia(1,1,1)	Si(4,4,4)	71.63	48.71	449.395	9.23	24.219	154.24	6.37
Dia(1,1,1)	Si(1,1,7)	78.02	31.33	248.051	7.92	15.267	71.34	4.67

Ei = 8.9805 keV			Cryst 1-2			Cryst 1-2-3		
Cryst 1	Cryst 2,3	ΘB 2 [°]	ΔE [meV]	∫IR dΘdE [μrad meV]	FOM	ΔE [meV]	∫IR dΘdE [μrad meV]	FOM
Si(1,1,1)	Si(1,1,1)	12.72	867.61	9798.588	11.29	806.409	8082.96	10.02
Si(1,1,1)	Si(0,2,2)	21.07	494.89	5480.877	11.07	348.978	3289.01	9.42
Si(1,1,1)	Si(1,1,3)	24.93	318.99	2536.424	7.95	149.047	898.78	6.03
Si(1,1,1)	Si(0,0,4)	30.56	266.25	2413.686	9.07	132.716	947.52	7.14
Si(1,1,1)	Si(1,3,3)	33.64	212.51	1259.782	5.93	70.469	308.07	4.37
Si(1,1,1)	Si(2,2,4)	38.51	186.23	1401.762	7.53	73.486	435.49	5.93
Si(1,1,1)	Si(3,3,3)	41.33	157.7	762.827	4.84	42.779	147.31	3.44
Si(1,1,1)	Si(0,4,4)	45.97	139.73	912.771	6.53	48.338	239.68	4.96
Si(1,1,1)	Si(1,3,5)	48.76	120.82	505.655	4.19	30.236	82.6	2.73
Si(1,1,1)	Si(0,2,6)	53.5	105.96	633.534	5.98	33.91	148.2	4.37
Si(1,1,1)	Si(3,3,5)	56.46	90.87	352.678	3.88	21.791	53.1	2.44
Si(1,1,1)	Si(4,4,4)	61.71	77.04	458.71	5.95	25.056	104.55	4.17
Si(1,1,1)	Si(1,1,7)	65.19	64.02	255.341	3.99	16.35	38.51	2.36
Si(1,1,1)	Si(1,5,5)	65.19	64.02	255.341	3.99	16.35	38.51	2.36
Si(1,1,1)	Si(2,4,6)	72.02	49.19	342.831	6.97	19.568	93.76	4.79
Si(1,1,1)	Si(1,3,7)	77.5	33.15	189.741	5.72	12.384	41.63	3.36
Dia(1,1,1)	Si(1,1,1)	12.72	520.9	6308.664	12.11	2364.68	5451.96	2.31
Dia(1,1,1)	Si(0,2,2)	21.07	360.86	4322.443	11.98	336.953	3606.74	10.7
Dia(1,1,1)	Si(1,1,3)	24.93	246.88	2179.5	8.83	150.766	1024.05	6.79
Dia(1,1,1)	Si(0,0,4)	30.56	215.65	2160.168	10.02	133.006	1083.34	8.15
Dia(1,1,1)	Si(1,3,3)	33.64	176.86	1160.763	6.56	70.566	349.34	4.95
Dia(1,1,1)	Si(2,2,4)	38.51	158.7	1320.537	8.32	74.658	496.32	6.65
Dia(1,1,1)	Si(3,3,3)	41.33	138.93	730.563	5.26	43.525	164.91	3.79
Dia(1,1,1)	Si(0,4,4)	45.97	123.38	889.884	7.21	48.266	271.25	5.62
Dia(1,1,1)	Si(1,3,5)	48.76	110.08	496.367	4.51	30.31	93.46	3.08
Dia(1,1,1)	Si(0,2,6)	53.5	96.32	631.404	6.56	33.804	167.04	4.94
Dia(1,1,1)	Si(3,3,5)	56.46	85.51	354.09	4.14	21.901	59.82	2.73
Dia(1,1,1)	Si(4,4,4)	61.71	71.86	466.721	6.49	25.244	119.24	4.72
Dia(1,1,1)	Si(1,1,7)	65.19	61.17	261.662	4.28	16.407	43.47	2.65
Dia(1,1,1)	Si(1,5,5)	65.19	61.17	261.662	4.28	16.407	43.47	2.65
Dia(1,1,1)	Si(2,4,6)	72.02	45.99	356.46	7.75	18.994	107.29	5.65
Dia(1,1,1)	Si(1,3,7)	77.5	31.36	199.138	6.35	12.235	47.57	3.89

Ei = 9.659 keV				Cryst 1-2			Cryst 1-2-3	
Cryst 1	Cryst 2,3	ΘB 2 [°]	ΔE [meV]	∫IR dΘdE [µrad meV]	FOM	ΔE [meV]	∫IR dΘdE [µrad meV]	FOM
Si(1,1,1)	Si(1,1,1)	11.81	926.31	10467.621	11.3	873.374	8733.63	10
Si(1,1,1)	Si(0,2,2)	19.53	532.59	5824.433	10.94	371.883	3490.39	9.39
Si(1,1,1)	Si(1,1,3)	23.08	352.7	2716.878	7.7	158.497	957.17	6.04
Si(1,1,1)	Si(0,0,4)	28.21	295.4	2578.904	8.73	142.023	999.58	7.04
Si(1,1,1)	Si(1,3,3)	31	241.97	1361.782	5.63	75.515	325.04	4.3
Si(1,1,1)	Si(2,2,4)	35.38	212.38	1504.571	7.08	79.685	453.17	5.69
Si(1,1,1)	Si(3,3,3)	37.88	184.32	829.209	4.5	47.394	153.64	3.24
Si(1,1,1)	Si(0,4,4)	41.95	164.56	983.858	5.98	51.209	238.11	4.65
Si(1,1,1)	Si(1,3,5)	44.36	145.36	551.501	3.79	31.93	86.63	2.71
Si(1,1,1)	Si(0,2,6)	48.37	129.75	685.297	5.28	36.274	143.48	3.96
Si(1,1,1)	Si(3,3,5)	50.8	116.39	387.521	3.33	23.166	52.61	2.27
Si(1,1,1)	Si(4,4,4)	54.96	101.96	497.488	4.88	26.932	96.08	3.57
Si(1,1,1)	Si(1,1,7)	57.56	89.78	281.582	3.14	17.267	35.18	2.04
Si(1,1,1)	Si(2,4,6)	62.17	76.81	373.135	4.86	20.892	69.28	3.32
Si(1,1,1)	Si(1,3,7)	65.19	66.16	210.706	3.18	13.485	26.38	1.96
Si(1,1,1)	Si(0,0,8)	70.98	51.67	285.748	5.53	16.205	61	3.76
Si(1,1,1)	Si(3,3,7)	75.31	38.39	160.227	4.17	10.36	25.56	2.47
Dia(1,1,1)	Si(1,1,1)	11.81	560.16	6593.678	11.77	2593.36	5793.26	2.23
Dia(1,1,1)	Si(0,2,2)	19.53	382.36	4456.736	11.66	354.159	3787.86	10.7
Dia(1,1,1)	Si(1,1,3)	23.08	266.03	2255.482	8.48	157.226	1066.93	6.79
Dia(1,1,1)	Si(0,0,4)	28.21	233.73	2228.414	9.53	142.379	1108.03	7.78
Dia(1,1,1)	Si(1,3,3)	31	194.88	1210.22	6.21	75.412	361.87	4.8
Dia(1,1,1)	Si(2,2,4)	35.38	175.81	1360.371	7.74	75.013	512.71	6.84
Dia(1,1,1)	Si(3,3,3)	37.88	157.03	760.455	4.84	47.035	171.67	3.65
Dia(1,1,1)	Si(0,4,4)	41.95	140.96	919.637	6.52	51.558	267.63	5.19
Dia(1,1,1)	Si(1,3,5)	44.36	129.34	521.289	4.03	32.292	96.1	2.98
Dia(1,1,1)	Si(0,2,6)	48.37	115.11	655.606	5.7	36.038	159.66	4.43
Dia(1,1,1)	Si(3,3,5)	50.8	105.72	372.746	3.53	22.894	59.63	2.6
Dia(1,1,1)	Si(4,4,4)	54.96	92.71	484.747	5.23	27.267	105.78	3.88
Dia(1,1,1)	Si(1,1,7)	57.56	84.2	274.861	3.26	17.09	39.9	2.33
Dia(1,1,1)	Si(2,4,6)	62.17	71.31	369.097	5.18	20.873	77.34	3.71
Dia(1,1,1)	Si(1,3,7)	65.19	62.77	208.986	3.33	13.256	30.01	2.26
Dia(1,1,1)	Si(0,0,8)	70.98	48.07	287.689	5.98	16.122	67.83	4.21
Dia(1,1,1)	Si(3,3,7)	75.31	36.53	162.499	4.45	10.549	28.08	2.66

Ei = 10.367 keV			Cryst 1-2			Cryst 1-2-3		
Cryst 1	Cryst 2,3	ΘB 2 [°]	ΔE [meV]	∫IR dΘdE [µrad meV]	FOM	ΔE [meV]	∫IR dΘdE [µrad meV]	FOM
Si(1,1,1)	Si(1,1,1)	10.99	986.08	11072.013	11.23	935.95	9416.48	10.06
Si(1,1,1)	Si(0,2,2)	18.14	571.32	6148.675	10.76	393.739	3698.7	9.39
Si(1,1,1)	Si(1,1,3)	21.42	388.41	2896.581	7.46	167.95	1023.32	6.09
Si(1,1,1)	Si(0,0,4)	26.13	326.21	2735.958	8.39	151.079	1050.78	6.96
Si(1,1,1)	Si(1,3,3)	28.68	273.03	1459.6	5.35	80.631	339.19	4.21
Si(1,1,1)	Si(2,2,4)	32.64	240.31	1601.205	6.66	83.327	463.17	5.56
Si(1,1,1)	Si(3,3,3)	34.9	213.25	893.051	4.19	50.596	162.31	3.21
Si(1,1,1)	Si(0,4,4)	38.52	190.93	1050.982	5.5	54.896	241.18	4.39
Si(1,1,1)	Si(1,3,5)	40.65	171.78	596.68	3.47	33.946	90.61	2.67
Si(1,1,1)	Si(0,2,6)	44.14	154.79	735.17	4.75	39.71	144.36	3.64
Si(1,1,1)	Si(3,3,5)	46.22	140.67	420.42	2.99	24.544	52.98	2.16
Si(1,1,1)	Si(4,4,4)	49.71	126.67	535.284	4.23	29.829	93.9	3.15
Si(1,1,1)	Si(1,1,7)	51.84	115.15	307.04	2.67	18.417	33.05	1.79
Si(1,1,1)	Si(2,4,6)	55.48	102.73	402.152	3.91	22.638	64.77	2.86
Si(1,1,1)	Si(1,3,7)	57.75	92.38	229.977	2.49	14.232	22.76	1.6
Si(1,1,1)	Si(0,0,8)	61.74	80.33	308.994	3.85	17.881	48.22	2.7
Si(1,1,1)	Si(3,3,7)	64.32	70.56	176.055	2.5	11.244	17.26	1.53
Si(1,1,1)	Si(0,6,6)	69.11	57.87	240.565	4.16	14.347	40.1	2.8
Si(1,1,1)	Si(5,5,5)	72.46	47.13	137.323	2.91	9.132	15.89	1.74
Si(1,1,1)	Si(0,4,8)	80	29.19	192.475	6.59	11.474	50.85	4.43
Dia(1,1,1)	Si(1,1,1)	10.99	599.91	6881.756	11.47	2843.04	6123.53	2.15
Dia(1,1,1)	Si(0,2,2)	18.14	402.92	4596.523	11.41	382.909	3933.23	10.27
Dia(1,1,1)	Si(1,1,3)	21.42	286.17	2323.432	8.12	166.033	1113.5	6.71
Dia(1,1,1)	Si(0,0,4)	26.13	251.93	2288.041	9.08	148.878	1153.51	7.75
Dia(1,1,1)	Si(1,3,3)	28.68	213.74	1253.657	5.87	80.475	372.86	4.63
Dia(1,1,1)	Si(2,2,4)	32.64	193.87	1396.57	7.2	84.096	511.28	6.08
Dia(1,1,1)	Si(3,3,3)	34.9	175.06	791.153	4.52	50.521	178.03	3.52
Dia(1,1,1)	Si(0,4,4)	38.52	158.64	945.74	5.96	54.721	264.58	4.84
Dia(1,1,1)	Si(1,3,5)	40.65	147.98	542.037	3.66	33.889	99.02	2.92
Dia(1,1,1)	Si(0,2,6)	44.14	133.41	675.773	5.07	39.501	158.87	4.02
Dia(1,1,1)	Si(3,3,5)	46.22	124.92	389.533	3.12	24.481	58.04	2.37
Dia(1,1,1)	Si(4,4,4)	49.71	112.18	501.54	4.47	29.831	102.77	3.44
Dia(1,1,1)	Si(1,1,7)	51.84	105.06	288.643	2.75	18.338	36.56	1.99
Dia(1,1,1)	Si(2,4,6)	55.48	92.72	381.938	4.12	22.706	71.16	3.13
Dia(1,1,1)	Si(1,3,7)	57.75	86.08	219.402	2.55	14.224	25.2	1.77
Dia(1,1,1)	Si(0,0,8)	61.74	73.94	297.497	4.02	17.9	52.8	2.95
Dia(1,1,1)	Si(3,3,7)	64.32	67.39	170.377	2.53	11.234	19.11	1.7
Dia(1,1,1)	Si(0,6,6)	69.11	53.52	236.391	4.42	14.218	44.29	3.11
Dia(1,1,1)	Si(5,5,5)	72.46	44.75	134.574	3.01	9.005	17.8	1.98
Dia(1,1,1)	Si(0,4,8)	80	26.66	191.593	7.19	11.417	55.86	4.89

Ei = 11.103 keV			Cryst 1-2			Cryst 1-2-3		
Cryst 1	Cryst 2,3	ΘB 2 [°]	ΔE [meV]	∫IR dΘdE [µrad meV]	FOM	ΔE [meV]	∫IR dΘdE [µrad meV]	FOM
Si(1,1,1)	Si(1,1,1)	10.26	1047.41	11643.915	11.12	997.923	10032.26	10.05
Si(1,1,1)	Si(0,2,2)	16.9	611.39	6449.886	10.55	416.775	3885.03	9.32
Si(1,1,1)	Si(1,1,3)	19.94	425.56	3064.943	7.2	177.517	1073.12	6.05
Si(1,1,1)	Si(0,0,4)	24.28	358.74	2879.313	8.03	159.11	1085.33	6.82
Si(1,1,1)	Si(1,3,3)	26.62	305.07	1551.44	5.09	86.839	355.62	4.1
Si(1,1,1)	Si(2,2,4)	30.24	269.9	1694.85	6.28	89.345	472.5	5.29
Si(1,1,1)	Si(3,3,3)	32.29	243.56	953.961	3.92	53.925	170.46	3.16
Si(1,1,1)	Si(0,4,4)	35.56	218.73	1115.303	5.1	59.356	246.21	4.15
Si(1,1,1)	Si(1,3,5)	37.46	199.78	638.302	3.2	36.224	91.5	2.53
Si(1,1,1)	Si(0,2,6)	40.56	181.55	782.292	4.31	42.55	147.73	3.47
Si(1,1,1)	Si(3,3,5)	42.39	167.34	452.466	2.7	26.189	52.28	2
Si(1,1,1)	Si(4,4,4)	45.42	152.55	571.9	3.75	31.191	94.05	3.02
Si(1,1,1)	Si(1,1,7)	47.24	140.77	331.395	2.35	19.873	32.26	1.62
Si(1,1,1)	Si(2,4,6)	50.29	127.99	430.404	3.36	23.702	63.24	2.67
Si(1,1,1)	Si(1,3,7)	52.15	118.07	249.413	2.11	15.875	20.89	1.32
Si(1,1,1)	Si(0,0,8)	55.33	106.55	331.259	3.11	18.664	43.98	2.36
Si(1,1,1)	Si(3,3,7)	57.3	98.44	190.935	1.94	12.894	15.34	1.19
Si(1,1,1)	Si(0,6,6)	60.73	86.71	260.188	3	14.902	32.87	2.21
Si(1,1,1)	Si(5,5,5)	62.91	77.91	149.45	1.92	10.32	11.59	1.12
Si(1,1,1)	Si(5,5,5)	62.91	77.91	149.45	1.92	10.32	11.59	1.12
Si(1,1,1)	Si(0,4,8)	66.86	66.04	207.031	3.13	12.34	27.08	2.19
Si(1,1,1)	Si(1,1,9)	69.49	57.17	118.462	2.07	8.005	10	1.25
Si(1,1,1)	Si(4,6,6)	74.67	43.17	167.007	3.87	10.306	26.21	2.54
Si(1,1,1)	Si(1,3,9)	78.72	31.28	95.171	3.04	6.591	11.8	1.79
Dia(1,1,1)	Si(1,1,1)	10.26	640.38	7150.177	11.17	3624.93	6480.39	1.79
Dia(1,1,1)	Si(0,2,2)	16.9	426.91	4714.534	11.04	403.89	4098.11	10.15
Dia(1,1,1)	Si(1,1,3)	19.94	306.41	2393.752	7.81	178.384	1168.98	6.55
Dia(1,1,1)	Si(0,0,4)	24.28	270.9	2337.946	8.63	155.201	1170.26	7.54
Dia(1,1,1)	Si(1,3,3)	26.62	233.24	1289.484	5.53	86.23	385.73	4.47
Dia(1,1,1)	Si(2,2,4)	30.24	211.64	1438.231	6.8	88.431	512.33	5.79
Dia(1,1,1)	Si(3,3,3)	32.29	193.13	819.112	4.24	54.095	185.01	3.42
Dia(1,1,1)	Si(0,4,4)	35.56	176.15	973.282	5.53	58.93	269.74	4.58
Dia(1,1,1)	Si(1,3,5)	37.46	166.28	563.564	3.39	35.763	102.69	2.87
Dia(1,1,1)	Si(0,2,6)	40.56	151.38	693.162	4.58	42.077	161.81	3.85
Dia(1,1,1)	Si(3,3,5)	42.39	143.61	405.214	2.82	26.175	57.12	2.18
Dia(1,1,1)	Si(4,4,4)	45.42	131.13	515.717	3.93	31.313	102.08	3.26
Dia(1,1,1)	Si(1,1,7)	47.24	123.88	300.224	2.42	19.902	35.7	1.79
Dia(1,1,1)	Si(2,4,6)	50.29	112.53	394.53	3.51	23.598	68.7	2.91
Dia(1,1,1)	Si(1,3,7)	52.15	106.93	229.234	2.14	15.896	22.76	1.43
Dia(1,1,1)	Si(0,0,8)	55.33	95.18	303.35	3.19	18.91	47.67	2.52
Dia(1,1,1)	Si(3,3,7)	57.3	90.93	178.959	1.97	12.911	16.19	1.25
Dia(1,1,1)	Si(0,6,6)	60.73	78.54	243.991	3.11	14.892	36.25	2.43
Dia(1,1,1)	Si(5,5,5)	62.91	73.73	141.524	1.92	10.334	12.22	1.18
Dia(1,1,1)	Si(5,5,5)	62.91	73.73	141.523	1.92	10.334	12.22	1.18
Dia(1,1,1)	Si(0,4,8)	66.86	60.95	196.235	3.22	12.112	30.22	2.5
Dia(1,1,1)	Si(1,1,9)	69.49	54.71	113.227	2.07	8.071	10.15	1.26
Dia(1,1,1)	Si(4,6,6)	74.67	39.8	161.022	4.05	10.208	28.66	2.81
Dia(1,1,1)	Si(1,3,9)	78.72	29.17	92.105	3.16	6.669	12.72	1.91

Ei = 11.867 keV			Cryst 1-2			Cryst 1-2-3		
Cryst 1	Cryst 2,3	ΘB 2 [°]	ΔE [meV]	∫IR dΘdE [μrad meV]	FOM	ΔE [meV]	∫IR dΘdE [μrad meV]	FOM
Si(1,1,1)	Si(1,1,1)	9.59	1110.7	12168.366	10.96	1061.73	10611.36	9.99
Si(1,1,1)	Si(0,2,2)	15.79	653.22	6726.313	10.3	440.822	4070.42	9.23
Si(1,1,1)	Si(1,1,3)	18.6	464.62	3224.3	6.94	187.678	1122.57	5.98
Si(1,1,1)	Si(0,0,4)	22.63	392.44	3013.737	7.68	167.976	1132.57	6.74
Si(1,1,1)	Si(1,3,3)	24.79	339.65	1638.282	4.82	93.041	372.21	4
Si(1,1,1)	Si(2,2,4)	28.11	300.82	1782.2	5.92	95.255	482.02	5.06
Si(1,1,1)	Si(3,3,3)	29.99	274.51	1009.446	3.68	57.172	177.27	3.1
Si(1,1,1)	Si(3,3,3)	29.99	274.51	1009.446	3.68	57.172	177.27	3.1
Si(1,1,1)	Si(0,4,4)	32.96	247.94	1175.776	4.74	63.344	254.57	4.02
Si(1,1,1)	Si(1,3,5)	34.68	229.27	680.742	2.97	38.8	94.83	2.44
Si(1,1,1)	Si(0,2,6)	37.47	209.68	825.048	3.93	44.636	150.53	3.37
Si(1,1,1)	Si(3,3,5)	39.1	195.63	482.617	2.47	28.154	51.89	1.84
Si(1,1,1)	Si(4,4,4)	41.79	179.2	604.148	3.37	33.341	93.15	2.79
Si(1,1,1)	Si(1,1,7)	43.39	167.75	355.065	2.12	22.236	33.66	1.51
Si(1,1,1)	Si(2,4,6)	46.04	154.39	457.038	2.96	25.132	60.72	2.42
Si(1,1,1)	Si(1,3,7)	47.63	144.44	266.946	1.85	71.427	22.19	0.31
Si(1,1,1)	Si(0,0,8)	50.31	132.57	353.063	2.66	19.871	40.05	2.02
Si(1,1,1)	Si(3,3,7)	51.94	124.08	206.84	1.67	60.913	16.5	0.27
Si(1,1,1)	Si(0,6,6)	54.7	112.89	277.533	2.46	16.064	28.29	1.76
Si(1,1,1)	Si(5,5,5)	56.41	105.2	161.933	1.54	51.295	12.73	0.25
Si(1,1,1)	Si(0,4,8)	59.35	94.44	221.416	2.34	13.097	21.26	1.62
Si(1,1,1)	Si(1,1,9)	61.2	87.23	128.864	1.48	42.421	9.48	0.22
Si(1,1,1)	Si(4,6,6)	64.46	76.34	179.188	2.35	10.781	17.07	1.58
Si(1,1,1)	Si(1,3,9)	66.57	68.63	103.461	1.51	33.598	7.63	0.23
Si(1,1,1)	Si(4,4,8)	70.46	56.85	146.583	2.58	8.904	15.62	1.75
Si(1,1,1)	Si(1,7,7)	73.15	48.19	84.059	1.74	6.365	5.88	0.92
Si(1,1,1)	Si(0,2,10)	78.79	32.59	121.111	3.72	7.578	18.99	2.51
Si(1,1,1)	Si(1,5,9)	84.25	17.03	69.144	4.06	4.935	11.35	2.3
Si(1,1,1)	Si(3,7,7)	84.25	17.03	69.144	4.06	4.935	11.35	2.3

Ei =	11.867	keV						
Cryst 1	Cryst 2,3	ΘB 2	ΔE	∫IR dΘdE	FOM	ΔE	∫IR dΘdE	FOM
		[°]	[meV]	[μrad meV]		[meV]	[μrad meV]	
Dia(1,1,1)	Si(1,1,1)	9.59	682.61	7405.056	10.85	682.593	6823.89	10
Dia(1,1,1)	Si(0,2,2)	15.79	450.32	4827.738	10.72	423.456	4235.84	10
Dia(1,1,1)	Si(1,1,3)	18.6	327.14	2458.654	7.52	190.316	1216.75	6.39
Dia(1,1,1)	Si(0,0,4)	22.63	290.62	2376.402	8.18	167.922	1216.65	7.25
Dia(1,1,1)	Si(1,3,3)	24.79	253.39	1319.211	5.21	92.251	401.5	4.35
Dia(1,1,1)	Si(2,2,4)	28.11	230.32	1468.104	6.37	95.086	519.95	5.47
Dia(1,1,1)	Si(3,3,3)	29.99	211.61	844.015	3.99	56.851	190.8	3.36
Dia(1,1,1)	Si(3,3,3)	29.99	211.61	844.013	3.99	56.852	190.8	3.36
Dia(1,1,1)	Si(0,4,4)	32.96	193.94	998.948	5.15	63.566	272.71	4.29
Dia(1,1,1)	Si(1,3,5)	34.68	184.06	581.997	3.16	38.511	102.08	2.65
Dia(1,1,1)	Si(0,2,6)	37.47	168.66	711.661	4.22	44.765	161.07	3.6
Dia(1,1,1)	Si(3,3,5)	39.1	161.83	421.733	2.61	28.261	56.41	2
Dia(1,1,1)	Si(4,4,4)	41.79	149.08	528.997	3.55	32.953	100.13	3.04
Dia(1,1,1)	Si(1,1,7)	43.39	141.73	312.361	2.2	22.245	35.07	1.58
Dia(1,1,1)	Si(2,4,6)	46.04	131.52	405.131	3.08	25.124	66.53	2.65
Dia(1,1,1)	Si(1,3,7)	47.63	125.67	238.286	1.9	71.943	24.24	0.34
Dia(1,1,1)	Si(0,0,8)	50.31	114.95	315.725	2.75	20.05	43.98	2.19
Dia(1,1,1)	Si(3,3,7)	51.94	111.06	185.443	1.67	60.794	17.31	0.28
Dia(1,1,1)	Si(0,6,6)	54.7	99.77	251.143	2.52	15.996	30.37	1.9
Dia(1,1,1)	Si(5,5,5)	56.41	96.55	147.196	1.52	51.29	13.21	0.26
Dia(1,1,1)	Si(0,4,8)	59.35	85.05	202.808	2.38	13.139	23.2	1.77
Dia(1,1,1)	Si(1,1,9)	61.2	81.25	118.43	1.46	42.401	10.08	0.24
Dia(1,1,1)	Si(4,6,6)	64.46	69.78	165.636	2.37	193.432	18.6	0.1
Dia(1,1,1)	Si(1,3,9)	66.57	64.92	96.432	1.49	167.852	7.74	0.05
Dia(1,1,1)	Si(4,4,8)	70.46	52.34	137.04	2.62	8.943	16.75	1.87
Dia(1,1,1)	Si(1,7,7)	73.15	45.8	79.157	1.73	120.988	6.32	0.05
Dia(1,1,1)	Si(0,2,10)	78.79	29.55	114.697	3.88	7.662	20.15	2.63
Dia(1,1,1)	Si(1,5,9)	84.25	15.32	65.998	4.31	4.93	12.21	2.48
Dia(1,1,1)	Si(3,7,7)	84.25	15.32	65.998	4.31	4.93	12.21	2.48

Ei = 12.658 keV			Cryst 1-2			Cryst 1-2-3		
Cryst 1	Cryst 2,3	ΘB 2 [°]	ΔE [meV]	∫IR dΘdE [μrad meV]	FOM	ΔE [meV]	∫IR dΘdE [μrad meV]	FOM
Si(1,1,1)	Si(1,1,1)	8.99	1174.75	12647.006	10.77	1123.2	11152.93	9.93
Si(1,1,1)	Si(0,2,2)	14.78	696.08	6965.16	10.01	464.28	4219.64	9.09
Si(1,1,1)	Si(1,1,3)	17.4	504.96	3370.578	6.67	200.657	1173.49	5.85
Si(1,1,1)	Si(0,0,4)	21.14	427.93	3148.9	7.36	175.028	1155.2	6.6
Si(1,1,1)	Si(1,3,3)	23.15	374.53	1720.014	4.59	99.328	389.28	3.92
Si(1,1,1)	Si(2,2,4)	26.22	332.73	1859.614	5.59	99.296	494.27	4.98
Si(1,1,1)	Si(3,3,3)	27.94	308.15	1065.266	3.46	61.148	185.26	3.03
Si(1,1,1)	Si(0,4,4)	30.67	278.12	1232.641	4.43	66.908	264.65	3.96
Si(1,1,1)	Si(1,3,5)	32.24	260.35	719.622	2.76	40.985	95.63	2.33
Si(1,1,1)	Si(0,2,6)	34.77	239.53	870.319	3.63	48.266	153.89	3.19
Si(1,1,1)	Si(3,3,5)	36.25	225.06	512.172	2.28	30.634	52.54	1.71
Si(1,1,1)	Si(4,4,4)	38.66	207.4	638.051	3.08	34.91	94.29	2.7
Si(1,1,1)	Si(1,1,7)	40.09	196.43	377.221	1.92	97.351	36.18	0.37
Si(1,1,1)	Si(1,5,5)	40.09	196.43	377.221	1.92	97.351	36.18	0.37
Si(1,1,1)	Si(2,4,6)	42.44	182.01	482.286	2.65	26.988	57.32	2.12
Si(1,1,1)	Si(1,3,7)	43.84	172.28	286.033	1.66	84.115	24.76	0.29
Si(1,1,1)	Si(0,0,8)	46.17	159.46	373.255	2.34	21.602	37.59	1.74
Si(1,1,1)	Si(3,3,7)	47.57	150.79	221.081	1.47	72.97	18.01	0.25
Si(1,1,1)	Si(0,6,6)	49.92	139.62	293.696	2.1	34.568	28.82	0.83
Si(1,1,1)	Si(5,5,5)	51.35	132.34	173.731	1.31	63.454	13.05	0.21
Si(1,1,1)	Si(0,4,8)	53.76	121.5	234.413	1.93	59.94	21.71	0.36
Si(1,1,1)	Si(1,1,9)	55.24	114.31	138.002	1.21	54.9	10.31	0.19
Si(1,1,1)	Si(4,6,6)	57.77	104.28	190.165	1.82	51.331	15.79	0.31
Si(1,1,1)	Si(1,3,9)	59.34	97.65	111.914	1.15	46.745	7.91	0.17
Si(1,1,1)	Si(4,4,8)	62.07	87.79	156.332	1.78	43.119	12.55	0.29
Si(1,1,1)	Si(5,5,7)	63.8	81.15	91.216	1.12	38.84	6.08	0.16
Si(1,1,1)	Si(1,7,7)	63.8	81.14	91.217	1.12	38.84	6.08	0.16
Si(1,1,1)	Si(0,2,10)	66.87	70.88	129.061	1.82	34.97	10.74	0.31
Si(1,1,1)	Si(1,5,9)	68.87	63.87	74.799	1.17	30.712	4.85	0.16
Si(1,1,1)	Si(3,5,9)	75.25	43.86	62.226	1.42	21.373	3.99	0.19
Si(1,1,1)	Si(2,4,10)	81.05	26.92	91.302	3.39	5.942	13.4	2.25

Ei = 12.658 keV			Cryst 1-2			Cryst 1-2-3		
Cryst 1	Cryst 2,3	ΘB 2 [°]	ΔE [meV]	∫IR dΘdE [μrad meV]	FOM	ΔE [meV]	∫IR dΘdE [μrad meV]	FOM
Dia(1,1,1)	Si(1,1,1)	8.99	725.69	7648.913	10.54	721.627	7037.64	9.75
Dia(1,1,1)	Si(0,2,2)	14.78	475.24	4929.549	10.37	447.259	4372.55	9.78
Dia(1,1,1)	Si(1,1,3)	17.4	349.15	2516.162	7.21	203.949	1269.27	6.22
Dia(1,1,1)	Si(0,0,4)	21.14	311.27	2419.176	7.77	178.133	1248.28	7.01
Dia(1,1,1)	Si(1,3,3)	23.15	273.5	1349.219	4.93	98.33	417.75	4.25
Dia(1,1,1)	Si(2,2,4)	26.22	249.75	1491.204	5.97	101.325	523.18	5.16
Dia(1,1,1)	Si(3,3,3)	27.94	230.54	863.239	3.74	60.58	195.64	3.23
Dia(1,1,1)	Si(0,4,4)	30.67	212.76	1018.512	4.79	67.442	278.03	4.12
Dia(1,1,1)	Si(1,3,5)	32.24	202.06	597.034	2.95	41.706	101.16	2.43
Dia(1,1,1)	Si(0,2,6)	34.77	186.5	729.519	3.91	47.565	163.9	3.45
Dia(1,1,1)	Si(3,3,5)	36.25	179.68	434.474	2.42	30.668	56.31	1.84
Dia(1,1,1)	Si(4,4,4)	38.66	166.71	543.589	3.26	35.076	100.83	2.87
Dia(1,1,1)	Si(1,1,7)	40.09	159.64	324.834	2.03	97.733	36.95	0.38
Dia(1,1,1)	Si(1,5,5)	40.09	159.64	324.835	2.03	97.733	36.95	0.38
Dia(1,1,1)	Si(2,4,6)	42.44	149.29	416.931	2.79	26.996	62.19	2.3
Dia(1,1,1)	Si(1,3,7)	43.84	142.55	247.934	1.74	83.886	25.69	0.31
Dia(1,1,1)	Si(0,0,8)	46.17	133.34	326	2.44	21.607	40.61	1.88
Dia(1,1,1)	Si(3,3,7)	47.57	128.43	192.82	1.5	72.951	19.38	0.27
Dia(1,1,1)	Si(0,6,6)	49.92	118.62	258.071	2.18	34.526	30.07	0.87
Dia(1,1,1)	Si(5,5,5)	51.35	116.12	152.875	1.32	63.364	14.77	0.23
Dia(1,1,1)	Si(0,4,8)	53.76	105.05	207.919	1.98	60.019	22.21	0.37
Dia(1,1,1)	Si(1,1,9)	55.24	103.02	122.876	1.19	97.819	10.47	0.11
Dia(1,1,1)	Si(4,6,6)	57.77	92.5	169.958	1.84	256.896	17.38	0.07
Dia(1,1,1)	Si(1,3,9)	59.34	89.34	100.062	1.12	83.442	8.36	0.1
Dia(1,1,1)	Si(4,4,8)	62.07	78.72	140.634	1.79	216.528	13.79	0.06
Dia(1,1,1)	Si(1,7,7)	63.8	75.03	82.462	1.1	69.452	6.61	0.1
Dia(1,1,1)	Si(5,5,7)	63.8	75.03	82.461	1.1	69.452	6.61	0.1
Dia(1,1,1)	Si(0,2,10)	66.87	64.59	117.838	1.82	63.559	10.91	0.17
Dia(1,1,1)	Si(1,5,9)	68.87	59.58	68.541	1.15	55.17	5.25	0.1
Dia(1,1,1)	Si(3,5,9)	75.25	40.99	57.531	1.4	38.747	4.33	0.11
Dia(1,1,1)	Si(2,4,10)	81.05	24.06	84.798	3.52	5.964	14.2	2.38

Ei = 13.474 keV			Cryst 1-2			Cryst 1-2-3		
Cryst 1	Cryst 2,3	ΘB 2 [°]	ΔE [meV]	∫IR dΘdE [μrad meV]	FOM	ΔE [meV]	∫IR dΘdE [μrad meV]	FOM
Si(1,1,1)	Si(1,1,1)	8.44	1237.16	13099.904	10.59	1176.57	11702.07	9.95
Si(1,1,1)	Si(0,2,2)	13.86	739.64	7215.816	9.76	490.334	4360.64	8.89
Si(1,1,1)	Si(1,1,3)	16.32	544.6	3498.149	6.42	211.182	1204.87	5.71
Si(1,1,1)	Si(0,0,4)	19.81	464.59	3268.6	7.04	188.418	1189.74	6.31
Si(1,1,1)	Si(1,3,3)	21.67	410.83	1797.208	4.37	104.737	404.38	3.86
Si(1,1,1)	Si(2,2,4)	24.52	366.01	1936.816	5.29	107.034	503.84	4.71
Si(1,1,1)	Si(1,1,5)	26.12	343.14	1114.98	3.25	64.053	189	2.95
Si(1,1,1)	Si(0,4,4)	28.63	308.3	1281.828	4.16	70.61	273.98	3.88
Si(1,1,1)	Si(1,3,5)	30.08	292.67	754.87	2.58	44.017	97.02	2.2
Si(1,1,1)	Si(0,2,6)	32.4	269.51	909.169	3.37	50.194	154.6	3.08
Si(1,1,1)	Si(3,3,5)	33.75	254.98	539.024	2.11	33.978	55.32	1.63
Si(1,1,1)	Si(4,4,4)	35.94	235.96	668.674	2.83	37.229	92.98	2.5
Si(1,1,1)	Si(1,1,7)	37.23	226.1	399.254	1.77	111.256	37.4	0.34
Si(1,1,1)	Si(1,5,5)	37.23	226.1	399.253	1.77	111.256	37.4	0.34
Si(1,1,1)	Si(2,4,6)	39.34	209.68	507.273	2.42	28.952	55.7	1.92
Si(1,1,1)	Si(1,3,7)	40.59	200.97	303.196	1.51	97.222	27.25	0.28
Si(1,1,1)	Si(3,5,5)	40.59	200.97	303.197	1.51	97.222	27.25	0.28
Si(1,1,1)	Si(0,0,8)	42.67	187.07	392.562	2.1	46.204	39.9	0.86
Si(1,1,1)	Si(3,3,7)	43.9	178.78	235.097	1.32	85.756	19.53	0.23
Si(1,1,1)	Si(0,6,6)	45.96	166.83	309.689	1.86	82.325	30.47	0.37
Si(1,1,1)	Si(1,5,7)	47.19	159.01	185.647	1.17	75.945	14.99	0.2
Si(1,1,1)	Si(0,4,8)	49.26	148.61	248.399	1.67	72.322	22.47	0.31
Si(1,1,1)	Si(1,1,9)	50.51	141.26	148.192	1.05	67.165	11.42	0.17
Si(1,1,1)	Si(4,6,6)	52.63	131.61	201.765	1.53	63.625	17.67	0.28
Si(1,1,1)	Si(1,3,9)	53.91	124.48	119.624	0.96	59.391	9.83	0.17
Si(1,1,1)	Si(4,4,8)	56.1	115.41	165.529	1.43	55.726	14.53	0.26
Si(1,1,1)	Si(1,7,7)	57.45	109.88	97.916	0.89	51.965	7.51	0.14
Si(1,1,1)	Si(0,2,10)	59.76	100.27	137.415	1.37	48.377	10.59	0.22
Si(1,1,1)	Si(2,6,8)	59.76	100.27	137.415	1.37	48.377	10.59	0.22
Si(1,1,1)	Si(1,5,9)	61.2	94.22	80.674	0.86	44.715	5.83	0.13
Si(1,1,1)	Si(3,5,9)	65.29	79.13	66.952	0.85	37.403	4.72	0.13
Si(1,1,1)	Si(2,4,10)	68.13	69.38	97.136	1.4	33.455	7.63	0.23
Si(1,1,1)	Si(1,1,11)	69.97	62.67	56.312	0.9	29.693	3.91	0.13
Si(1,1,1)	Si(0,8,8)	73.42	51.52	82.204	1.6	25.169	6.22	0.25
Si(1,1,1)	Si(1,3,11)	75.84	43.43	47.51	1.09	20.917	2.99	0.14
Si(1,1,1)	Si(0,6,10)	81.09	27.54	70.672	2.57	4.737	7.89	1.67
Si(1,1,1)	Si(6,6,8)	81.09	27.54	70.672	2.57	4.737	7.89	1.67
Si(1,1,1)	Si(3,3,11)	87.16	9.2	40.417	4.39	3.071	7.63	2.48
Si(1,1,1)	Si(3,7,9)	87.16	9.2	40.417	4.39	3.071	7.63	2.48
Dia(1,1,1)	Si(1,1,1)	8.44	769.52	7884.561	10.25	771.985	7345.36	9.51
Dia(1,1,1)	Si(0,2,2)	13.86	501.4	5018.322	10.01	475.475	4463.33	9.39
Dia(1,1,1)	Si(1,1,3)	16.32	371.4	2563.648	6.9	211.831	1277.73	6.03
Dia(1,1,1)	Si(0,0,4)	19.81	330.82	2464.759	7.45	185.827	1261.95	6.79
Dia(1,1,1)	Si(1,3,3)	21.67	293.98	1380.862	4.7	104.618	433.29	4.14
Dia(1,1,1)	Si(2,2,4)	24.52	269.36	1512.216	5.61	104.697	539.44	5.15
Dia(1,1,1)	Si(1,1,5)	26.12	249.77	882.842	3.53	65.284	202.66	3.1
Dia(1,1,1)	Si(0,4,4)	28.63	231.34	1034.254	4.47	71.848	284.89	3.97
Dia(1,1,1)	Si(1,3,5)	30.08	220.24	608.512	2.76	43.948	103.85	2.36
Dia(1,1,1)	Si(0,2,6)	32.4	203.78	737.772	3.62	50.725	164.44	3.24
Dia(1,1,1)	Si(3,3,5)	33.75	196.52	443.046	2.25	33.979	59.4	1.75
Dia(1,1,1)	Si(4,4,4)	35.94	182.6	548.759	3.01	36.895	98.37	2.67

**Dia(1,1,1)**	**Si(1,1,7)**	**37.23**	**177.92**	**335.036**	**1.88**	**111.51**	**39.93**	**0.36**
**Dia(1,1,1)**	**Si(1,5,5)**	**37.23**	**177.92**	**335.036**	**1.88**	**111.511**	**39.94**	**0.36**
**Dia(1,1,1)**	**Si(2,4,6)**	**39.34**	**166.99**	**427.416**	**2.56**	**29.18**	**60.08**	**2.06**
**Dia(1,1,1)**	**Si(1,3,7)**	**40.59**	**159.42**	**255.949**	**1.61**	**97.14**	**28.2**	**0.29**
**Dia(1,1,1)**	**Si(3,5,5)**	**40.59**	**159.42**	**255.975**	**1.61**	**97.141**	**28.2**	**0.29**
**Dia(1,1,1)**	**Si(0,0,8)**	**42.67**	**151.32**	**335.498**	**2.22**	**46.08**	**42.1**	**0.91**
**Dia(1,1,1)**	**Si(3,3,7)**	**43.9**	**144.2**	**199.966**	**1.39**	**85.533**	**22.69**	**0.27**
**Dia(1,1,1)**	**Si(0,6,6)**	**45.96**	**136.25**	**266.41**	**1.96**	**81.966**	**30.88**	**0.38**
**Dia(1,1,1)**	**Si(1,5,7)**	**47.19**	**131.92**	**159.283**	**1.21**	**135.233**	**15.91**	**0.12**
**Dia(1,1,1)**	**Si(0,4,8)**	**49.26**	**123.22**	**214.028**	**1.74**	**360.256**	**23.13**	**0.06**
**Dia(1,1,1)**	**Si(1,1,9)**	**50.51**	**120.84**	**128.425**	**1.06**	**119.649**	**12.26**	**0.1**
**Dia(1,1,1)**	**Si(4,6,6)**	**52.63**	**111.16**	**175.198**	**1.58**	**114.587**	**18.25**	**0.16**
**Dia(1,1,1)**	**Si(1,3,9)**	**53.91**	**109.84**	**104.523**	**0.95**	**105.593**	**10.16**	**0.1**
**Dia(1,1,1)**	**Si(4,4,8)**	**56.1**	**99.61**	**144.45**	**1.45**	**100.428**	**14.76**	**0.15**
**Dia(1,1,1)**	**Si(1,7,7)**	**57.45**	**98.13**	**86.198**	**0.88**	**92.432**	**7.32**	**0.08**
**Dia(1,1,1)**	**Si(0,2,10)**	**59.76**	**88.08**	**120.556**	**1.37**	**86.99**	**12.13**	**0.14**
**Dia(1,1,1)**	**Si(2,6,8)**	**59.76**	**88.08**	**120.556**	**1.37**	**86.99**	**12.13**	**0.14**
**Dia(1,1,1)**	**Si(1,5,9)**	**61.2**	**85.65**	**71.583**	**0.84**	**220.516**	**5.39**	**0.02**
**Dia(1,1,1)**	**Si(3,5,9)**	**65.29**	**72.28**	**59.815**	**0.83**	**37.439**	**4.6**	**0.12**
**Dia(1,1,1)**	**Si(2,4,10)**	**68.13**	**62.41**	**86.428**	**1.38**	**60.354**	**7.96**	**0.13**
**Dia(1,1,1)**	**Si(1,1,11)**	**69.97**	**57.58**	**50.402**	**0.88**	**29.675**	**4.09**	**0.14**
**Dia(1,1,1)**	**Si(0,8,8)**	**73.42**	**46.63**	**74.318**	**1.59**	**25.231**	**6.55**	**0.26**
**Dia(1,1,1)**	**Si(1,3,11)**	**75.84**	**40.27**	**43.006**	**1.07**	**20.924**	**3.27**	**0.16**
**Dia(1,1,1)**	**Si(0,6,10)**	**81.09**	**24.42**	**64.395**	**2.64**	**4.708**	**8.52**	**1.81**
**Dia(1,1,1)**	**Si(6,6,8)**	**81.09**	**24.42**	**64.395**	**2.64**	**4.708**	**8.52**	**1.81**
**Dia(1,1,1)**	**Si(3,3,11)**	**87.16**	**8**	**37.141**	**4.64**	**3.099**	**8.06**	**2.6**
**Dia(1,1,1)**	**Si(3,7,9)**	**87.16**	**8**	**37.141**	**4.64**	**3.099**	**8.06**	**2.6**

Ei = 22.117 keV			Cryst 1-2			Cryst 1-2-3		
Cryst 1	Cryst 2,3	ΘB 2 [°]	ΔE [meV]	∫IR dΘdE [µrad meV]	FOM	ΔE [meV]	∫IR dΘdE [µrad meV]	FOM
Si(1,1,1)	Si(1,1,1)	5.13	1845.09	16256.184	8.81	1791.41	15078.83	8.42
Si(1,1,1)	Si(0,2,2)	8.39	1200.87	8705.51	7.25	720.795	5090.24	7.06
Si(1,1,1)	Si(1,1,3)	9.86	984.25	4317.733	4.39	347.909	1461.24	4.2
Si(1,1,1)	Si(0,0,4)	11.91	852.56	3993.464	4.68	301.073	1359.71	4.52
Si(1,1,1)	Si(1,3,3)	13	783.54	2271.026	2.9	172.345	485.26	2.82
Si(1,1,1)	Si(2,2,4)	14.65	708.8	2422.158	3.42	177.869	596.17	3.35
Si(1,1,1)	Si(1,1,5)	15.56	675.24	1441.42	2.13	215.967	212.76	0.99
Si(1,1,1)	Si(0,4,4)	16.97	628.36	1636.129	2.6	117.488	287.9	2.45
Si(1,1,1)	Si(1,3,5)	17.78	600.65	995.887	1.66	369.407	132.67	0.36
Si(1,1,1)	Si(0,2,6)	19.05	567.76	1174.095	2.07	354.66	161.87	0.46
Si(1,1,1)	Si(3,3,5)	19.78	541.37	719.131	1.33	322.913	88.22	0.27
Si(1,1,1)	Si(4,4,4)	20.95	516.35	868.921	1.68	311.28	118.22	0.38
Si(1,1,1)	Si(1,1,7)	21.63	505.94	542.249	1.07	1436.96	68.42	0.05
Si(1,1,1)	Si(1,3,7)	23.35	480.98	420.363	0.87	1311.7	52.19	0.04
Si(1,1,1)	Si(3,5,5)	23.35	480.98	420.359	0.87	1311.69	52.19	0.04
Si(1,1,1)	Si(2,4,6)	22.72	478.2	665.618	1.39	498.149	89.41	0.18
Si(1,1,1)	Si(3,3,7)	24.99	457.67	332.47	0.73	424.545	37.14	0.09
Si(1,1,1)	Si(0,0,8)	24.39	453.98	520.647	1.15	1066.99	66.96	0.06
Si(1,1,1)	Si(0,6,6)	25.97	434.22	419.072	0.97	473.794	54.25	0.11
Si(1,1,1)	Si(1,5,7)	26.55	432.8	265.302	0.61	399.905	30.4	0.08
Si(1,1,1)	Si(5,5,5)	26.55	432.8	265.3	0.61	399.905	30.4	0.08
Si(1,1,1)	Si(0,4,8)	27.49	415.35	340.101	0.82	386.521	43.45	0.11
Si(1,1,1)	Si(1,1,9)	28.05	407.01	215.046	0.53	374.687	21.66	0.06
Si(1,1,1)	Si(4,6,6)	28.96	393.31	279.845	0.71	359.506	31.47	0.09
Si(1,1,1)	Si(1,3,9)	29.49	384.38	175.702	0.46	352.29	19.77	0.06
Si(1,1,1)	Si(4,4,8)	30.38	372.31	231.346	0.62	341.651	28	0.08
Si(1,1,1)	Si(1,7,7)	30.9	361.63	146.236	0.4	332.17	16.81	0.05
Si(1,1,1)	Si(5,5,7)	30.9	361.62	146.237	0.4	332.171	16.81	0.05
Si(1,1,1)	Si(0,2,10)	31.76	352.16	193.796	0.55	322.956	18.76	0.06
Si(1,1,1)	Si(1,5,9)	32.27	343.43	121.749	0.35	314.325	14.57	0.05
Si(1,1,1)	Si(3,5,9)	33.6	325.77	102.926	0.32	696.819	12.34	0.02
Si(1,1,1)	Si(2,4,10)	34.43	315.62	138.112	0.44	290.579	15.49	0.05
Si(1,1,1)	Si(1,1,11)	34.92	308.94	87.54	0.28	781.165	2.82	0
Si(1,1,1)	Si(0,8,8)	35.72	302.05	118.435	0.39	762.702	13.03	0.02
Si(1,1,1)	Si(1,3,11)	36.21	294.82	75.158	0.25	743.83	0.46	0
Si(1,1,1)	Si(0,6,10)	37	287.73	102.817	0.36	726.544	12.39	0.02
Si(1,1,1)	Si(3,3,11)	37.48	280.74	64.285	0.23	709.207	0.13	0
Si(1,1,1)	Si(0,0,12)	38.27	275.14	89.694	0.33	692.93	10.88	0.02
Si(1,1,1)	Si(1,5,11)	38.74	267.68	55.946	0.21	135.754	0.05	0
Si(1,1,1)	Si(2,2,12)	39.52	260.4	77.596	0.3	136.804	9.29	0.07
Si(1,1,1)	Si(3,5,11)	39.98	256.94	48.362	0.19	128.344	0.02	0
Si(1,1,1)	Si(0,4,12)	40.75	252.05	68.157	0.27	202.849	0.97	0
Si(1,1,1)	Si(1,9,9)	41.22	244.01	41.762	0.17	122.775	0.01	0
Si(1,1,1)	Si(1,1,13)	42.44	243.47	29.67	0.12	118.24	0.01	0
Si(1,1,1)	Si(2,8,10)	41.98	241.37	61.198	0.25	125.333	0.28	0
Si(1,1,1)	Si(4,4,12)	43.21	230.84	53.125	0.23	118.709	0.11	0
Si(1,1,1)	Si(3,3,13)	44.89	221.29	14.073	0.06	110.176	0.01	0
Si(1,1,1)	Si(2,6,12)	44.43	218.51	47.291	0.22	111.324	0.05	0
Si(1,1,1)	Si(8,8,8)	45.65	213.39	42.13	0.2	105.521	0.03	0
Si(1,1,1)	Si(1,5,13)	46.11	210.6	12.286	0.06	107.912	0	0
Si(1,1,1)	Si(1,9,11)	47.33	204.76	11.104	0.05	104.39	0	0
Si(1,1,1)	Si(0,2,14)	46.88	201.77	37.584	0.19	100.593	0.02	0

**Si(1,1,1)**	**Si(7,9,9)**	**48.56**	**194.82**	**9.817**	**0.05**	**100.471**	**0**	**0**
**Si(1,1,1)**	**Si(3,9,11)**	**48.56**	**193.92**	**9.816**	**0.05**	**100.48**	**0**	**0**
**Si(1,1,1)**	**Si(0,8,12)**	**48.1**	**192.27**	**33.5**	**0.17**	**96.082**	**0.01**	**0**
**Si(1,1,1)**	**Si(1,7,13)**	**49.8**	**187.43**	**8.627**	**0.05**	**96.56**	**0**	**0**
**Si(1,1,1)**	**Si(5,5,13)**	**49.8**	**187.39**	**8.624**	**0.05**	**96.58**	**0**	**0**
**Si(1,1,1)**	**Si(4,8,12)**	**50.57**	**180.71**	**20.35**	**0.11**	**89.01**	**0.01**	**0**
**Si(1,1,1)**	**Si(1,1,15)**	**51.04**	**179.96**	**7.926**	**0.04**	**92.64**	**0**	**0**
**Si(1,1,1)**	**Si(0,6,14)**	**51.82**	**174.53**	**13.369**	**0.08**	**85.254**	**0.01**	**0**
**Si(1,1,1)**	**Si(1,3,15)**	**52.29**	**171.53**	**6.853**	**0.04**	**88.794**	**0**	**0**
**Si(1,1,1)**	**Si(1,11,11)**	**53.56**	**164.36**	**6.019**	**0.04**	**374.491**	**0**	**0**
**Si(1,1,1)**	**Si(2,10,12)**	**54.37**	**159.76**	**9.896**	**0.06**	**79.401**	**0**	**0**
**Si(1,1,1)**	**Si(4,6,14)**	**54.37**	**159.76**	**9.896**	**0.06**	**79.401**	**0**	**0**
**Si(1,1,1)**	**Si(1,9,13)**	**54.85**	**157.22**	**5.371**	**0.03**	**356.917**	**0**	**0**
**Si(1,1,1)**	**Si(1,5,15)**	**54.85**	**157.15**	**5.372**	**0.03**	**356.953**	**0**	**0**
**Si(1,1,1)**	**Si(2,4,14)**	**49.33**	**154.02**	**27.204**	**0.18**	**92.315**	**0.01**	**0**
**Si(1,1,1)**	**Si(0,0,16)**	**55.66**	**151.63**	**8.9**	**0.06**	**76.307**	**0**	**0**
**Si(1,1,1)**	**Si(1,3,13)**	**43.67**	**151.43**	**18.835**	**0.12**	**113.778**	**0.01**	**0**
**Si(1,1,1)**	**Si(3,5,15)**	**56.16**	**150.12**	**4.996**	**0.03**	**322.812**	**0**	**0**
**Si(1,1,1)**	**Si(2,2,16)**	**56.99**	**146.17**	**8.218**	**0.06**	**73.016**	**0**	**0**
**Si(1,1,1)**	**Si(0,4,16)**	**58.34**	**142**	**7.655**	**0.05**	**69.607**	**0**	**0**
**Si(1,1,1)**	**Si(7,7,13)**	**57.49**	**141.23**	**4.552**	**0.03**	**339.996**	**0**	**0**
**Si(1,1,1)**	**Si(5,11,11)**	**57.49**	**141.1**	**4.552**	**0.03**	**339.988**	**0**	**0**
**Si(1,1,1)**	**Si(6,10,12)**	**59.72**	**135.7**	**7.088**	**0.05**	**66.089**	**0**	**0**
**Si(1,1,1)**	**Si(1,7,15)**	**58.85**	**130.41**	**4.339**	**0.03**	**116.594**	**0**	**0**
**Si(1,1,1)**	**Si(0,12,12)**	**61.14**	**128.67**	**6.496**	**0.05**	**62.503**	**0**	**0**
**Si(1,1,1)**	**Si(3,7,15)**	**60.25**	**124.21**	**3.929**	**0.03**	**110.246**	**0**	**0**
**Si(1,1,1)**	**Si(9,9,11)**	**60.25**	**124.2**	**3.928**	**0.03**	**110.316**	**0**	**0**
**Si(1,1,1)**	**Si(1,1,17)**	**61.69**	**121.16**	**3.636**	**0.03**	**104.093**	**0**	**0**
**Si(1,1,1)**	**Si(0,10,14)**	**62.61**	**120.94**	**5.998**	**0.05**	**58.824**	**0**	**0**
**Si(1,1,1)**	**Si(3,11,13)**	**63.18**	**114.36**	**3.307**	**0.03**	**97.783**	**0**	**0**
**Si(1,1,1)**	**Si(7,9,13)**	**63.18**	**114.35**	**3.307**	**0.03**	**97.777**	**0**	**0**
**Si(1,1,1)**	**Si(1,3,17)**	**63.18**	**114.34**	**3.308**	**0.03**	**97.795**	**0**	**0**
**Si(1,1,1)**	**Si(4,12,12)**	**64.14**	**112.96**	**5.555**	**0.05**	**55.048**	**0**	**0**
**Si(1,1,1)**	**Si(1,9,15)**	**64.73**	**107.96**	**3.015**	**0.03**	**91.353**	**0**	**0**
**Si(1,1,1)**	**Si(4,10,14)**	**65.73**	**106.75**	**5.138**	**0.05**	**51.28**	**0**	**0**
**Si(1,1,1)**	**Si(1,5,17)**	**66.34**	**101.08**	**2.773**	**0.03**	**84.734**	**0**	**0**
**Si(1,1,1)**	**Si(0,8,16)**	**67.4**	**100.24**	**4.753**	**0.05**	**47.378**	**0**	**0**
**Si(1,1,1)**	**Si(0,2,18)**	**69.18**	**95.6**	**4.359**	**0.05**	**43.158**	**0**	**0**
**Si(1,1,1)**	**Si(7,7,15)**	**68.05**	**93.85**	**2.557**	**0.03**	**77.953**	**0**	**0**
**Si(1,1,1)**	**Si(3,5,17)**	**68.05**	**93.78**	**2.557**	**0.03**	**77.938**	**0**	**0**
**Si(1,1,1)**	**Si(5,9,15)**	**69.88**	**86.14**	**2.37**	**0.03**	**195.906**	**0**	**0**
**Si(1,1,1)**	**Si(4,8,16)**	**71.09**	**85.48**	**3.949**	**0.05**	**38.777**	**0**	**0**
**Si(1,1,1)**	**Si(1,7,17)**	**71.85**	**77.07**	**2.164**	**0.03**	**175.264**	**0**	**0**
**Si(1,1,1)**	**Si(7,11,13)**	**71.85**	**77.01**	**2.163**	**0.03**	**175.281**	**0**	**0**
**Si(1,1,1)**	**Si(2,4,18)**	**73.18**	**76.14**	**3.724**	**0.05**	**33.705**	**0**	**0**
**Si(1,1,1)**	**Si(3,7,17)**	**74.02**	**72.29**	**1.914**	**0.03**	**32.662**	**0**	**0**
**Si(1,1,1)**	**Si(1,11,15)**	**74.02**	**72.26**	**1.914**	**0.03**	**32.671**	**0**	**0**
**Si(1,1,1)**	**Si(8,12,12)**	**75.53**	**65.26**	**4.059**	**0.06**	**28.144**	**0**	**0**
**Si(1,1,1)**	**Si(3,11,15)**	**76.51**	**61.62**	**1.901**	**0.03**	**27.456**	**0**	**0**
**Si(1,1,1)**	**Si(1,1,19)**	**79.51**	**47.23**	**1.751**	**0.04**	**20.396**	**0**	**0**
**Si(1,1,1)**	**Si(0,6,18)**	**78.3**	**47.14**	**6.282**	**0.13**	**22.521**	**0**	**0**
**Si(1,1,1)**	**Si(1,3,19)**	**83.76**	**26.45**	**3.223**	**0.12**	**12.061**	**0.01**	**0**
**Dia(1,1,1)**	**Si(1,3,3)**	**13**	**1856.65**	**1545.015**	**0.83**	**170.377**	**484.57**	**2.84**
**Dia(1,1,1)**	**Si(1,1,1)**	**5.13**	**1235.64**	**9111.931**	**7.37**	**8111.94**	**8884.82**	**1.1**
**Dia(1,1,1)**	**Si(0,2,2)**	**8.39**	**765.52**	**5543.283**	**7.24**	**7337.61**	**5143.77**	**0.7**

Dia(1,1,1)	Si(1,1,3)	9.86	615.95	2821.179	4.58	344.215	1486.04	4.32
Dia(1,1,1)	Si(0,0,4)	11.91	554.04	2667.207	4.81	301.405	1375.73	4.56
Dia(1,1,1)	Si(2,2,4)	14.65	464.94	1663.135	3.58	175.154	610.69	3.49
Dia(1,1,1)	Si(1,1,5)	15.56	447.19	987.368	2.21	215.677	215.63	1
Dia(1,1,1)	Si(0,4,4)	16.97	415.22	1120.895	2.7	115.305	283.45	2.46
Dia(1,1,1)	Si(1,3,5)	17.78	403.54	692.396	1.72	1822.67	135.96	0.07
Dia(1,1,1)	Si(0,2,6)	19.05	379.42	815.027	2.15	564.823	167.86	0.3
Dia(1,1,1)	Si(3,3,5)	19.78	370.99	516.545	1.39	651.689	94.95	0.15
Dia(1,1,1)	Si(4,4,4)	20.95	350.85	621.1	1.77	302.271	119.15	0.39
Dia(1,1,1)	Si(1,1,7)	21.63	334.66	383.768	1.15	520.19	67.93	0.13
Dia(1,1,1)	Si(2,4,6)	22.72	323.49	479.15	1.48	271.528	85.11	0.31
Dia(1,1,1)	Si(1,3,7)	23.35	306.93	293.037	0.95	256.488	47.08	0.18
Dia(1,1,1)	Si(3,5,5)	23.35	306.93	293.037	0.95	256.487	47.07	0.18
Dia(1,1,1)	Si(0,0,8)	24.39	298.68	374.827	1.25	459.552	63.6	0.14
Dia(1,1,1)	Si(3,3,7)	24.99	283.5	230.408	0.81	235.846	40.52	0.17
Dia(1,1,1)	Si(0,6,6)	25.97	275.78	294.39	1.07	230.76	53.87	0.23
Dia(1,1,1)	Si(1,5,7)	26.55	265.29	184.803	0.7	220.781	30.09	0.14
Dia(1,1,1)	Si(5,5,5)	26.55	265.28	184.8	0.7	220.781	30.09	0.14
Dia(1,1,1)	Si(0,4,8)	27.49	257.29	238.227	0.93	212.966	43.05	0.2
Dia(1,1,1)	Si(1,1,9)	28.05	249.51	150.071	0.6	206.88	23.57	0.11
Dia(1,1,1)	Si(4,6,6)	28.96	247.65	200.169	0.81	198.934	34.4	0.17
Dia(1,1,1)	Si(1,3,9)	29.49	238.95	125.173	0.52	193.242	22.01	0.11
Dia(1,1,1)	Si(4,4,8)	30.38	235.68	167.281	0.71	188.318	26.46	0.14
Dia(1,1,1)	Si(1,7,7)	30.9	229.71	105.148	0.46	182.859	14.34	0.08
Dia(1,1,1)	Si(5,5,7)	30.9	229.71	105.151	0.46	182.854	14.34	0.08
Dia(1,1,1)	Si(0,2,10)	31.76	226.33	141.342	0.62	178.522	20.89	0.12
Dia(1,1,1)	Si(1,5,9)	32.27	222.93	89.242	0.4	174.353	10.98	0.06
Dia(1,1,1)	Si(3,5,9)	33.6	218.18	77.331	0.35	165.855	12.9	0.08
Dia(1,1,1)	Si(1,3,11)	36.21	212.41	57.708	0.27	148.779	8.09	0.05
Dia(1,1,1)	Si(2,4,10)	34.43	211.73	103.487	0.49	160.845	18	0.11
Dia(1,1,1)	Si(3,3,11)	37.48	210.48	50.752	0.24	141.934	7.21	0.05
Dia(1,1,1)	Si(0,8,8)	35.72	208.38	91.195	0.44	153.131	10.47	0.07
Dia(1,1,1)	Si(1,5,11)	38.74	205.59	45.256	0.22	135.384	6.53	0.05
Dia(1,1,1)	Si(0,6,10)	37	205.16	79.934	0.39	147.276	9.28	0.06
Dia(1,1,1)	Si(0,0,12)	38.27	203.47	70.536	0.35	140.933	10.34	0.07
Dia(1,1,1)	Si(3,5,11)	39.98	201.33	40.393	0.2	166.407	2.93	0.02
Dia(1,1,1)	Si(1,1,11)	34.92	200.91	64.273	0.32	156.376	7.48	0.05
Dia(1,1,1)	Si(2,2,12)	39.52	199.28	62.238	0.31	133.885	6	0.04
Dia(1,1,1)	Si(1,9,9)	41.22	193.9	35.197	0.18	191.848	0.62	0
Dia(1,1,1)	Si(0,4,12)	40.75	193.49	55.549	0.29	127.272	7.18	0.06
Dia(1,1,1)	Si(2,8,10)	41.98	188.76	49.164	0.26	121.803	6.61	0.05
Dia(1,1,1)	Si(1,1,13)	42.44	186.65	31.751	0.17	188.256	0.19	0
Dia(1,1,1)	Si(4,4,12)	43.21	181.72	44.457	0.24	116.925	6.03	0.05
Dia(1,1,1)	Si(1,3,13)	43.67	180.35	27.771	0.15	183.762	0.08	0
Dia(1,1,1)	Si(2,6,12)	44.43	174.9	40.042	0.23	111.711	5.5	0.05
Dia(1,1,1)	Si(3,3,13)	44.89	172.73	24.787	0.14	110.98	0.04	0
Dia(1,1,1)	Si(8,8,8)	45.65	168.64	35.672	0.21	136.884	5.03	0.04
Dia(1,1,1)	Si(1,5,13)	46.11	166.92	21.879	0.13	106.667	0.02	0
Dia(1,1,1)	Si(0,2,14)	46.88	160.8	32.085	0.2	156.89	1.71	0.01
Dia(1,1,1)	Si(1,9,11)	47.33	157.15	18.113	0.12	102.591	0.01	0
Dia(1,1,1)	Si(0,8,12)	48.1	154.32	28.483	0.18	127.399	0.56	0
Dia(1,1,1)	Si(3,9,11)	48.56	148.49	16.231	0.11	98.475	0.01	0
Dia(1,1,1)	Si(7,9,9)	48.56	148.48	16.236	0.11	98.418	0.01	0
Dia(1,1,1)	Si(1,7,13)	49.8	147.27	10.196	0.07	94.391	0.01	0
Dia(1,1,1)	Si(5,5,13)	49.8	147.26	10.194	0.07	94.383	0.01	0
Dia(1,1,1)	Si(2,4,14)	49.33	146.76	25.26	0.17	145.852	0.23	0

Dia(1,1,1)	Si(4,8,12)	50.57	141.88	23.321	0.16	90.357	0.12	0
Dia(1,1,1)	Si(0,6,14)	51.82	135.48	20.815	0.15	137.033	0.06	0
Dia(1,1,1)	Si(1,11,11)	53.56	130.23	5.387	0.04	82.574	0	0
Dia(1,1,1)	Si(1,9,13)	54.85	125.3	4.826	0.04	78.524	0	0
Dia(1,1,1)	Si(1,5,15)	54.85	125.28	4.821	0.04	78.537	0	0
Dia(1,1,1)	Si(2,10,12)	54.37	122.97	17.294	0.14	78.972	0.03	0
Dia(1,1,1)	Si(4,6,14)	54.37	122.97	17.294	0.14	78.972	0.03	0
Dia(1,1,1)	Si(3,5,15)	56.16	119.44	4.305	0.04	74.692	0	0
Dia(1,1,1)	Si(0,0,16)	55.66	117.25	15.54	0.13	75.47	0.02	0
Dia(1,1,1)	Si(7,7,13)	57.49	113.55	3.878	0.03	70.943	0	0
Dia(1,1,1)	Si(5,11,11)	57.49	113.52	3.877	0.03	70.938	0	0
Dia(1,1,1)	Si(2,2,16)	56.99	111.9	14.03	0.13	71.909	0.01	0
Dia(1,1,1)	Si(0,4,16)	58.34	107.09	12.972	0.12	68.36	0.01	0
Dia(1,1,1)	Si(1,7,15)	58.85	105.83	3.67	0.03	67.218	0	0
Dia(1,1,1)	Si(6,10,12)	59.72	100.89	11.166	0.11	64.743	0.01	0
Dia(1,1,1)	Si(9,9,11)	60.25	100.41	3.296	0.03	63.539	0	0
Dia(1,1,1)	Si(3,7,15)	60.25	100.31	3.297	0.03	63.512	0	0
Dia(1,1,1)	Si(0,12,12)	61.14	95.26	10.14	0.11	61.116	0.01	0
Dia(1,1,1)	Si(1,1,17)	61.69	94.73	2.979	0.03	59.827	0	0
Dia(1,1,1)	Si(0,10,14)	62.61	89.63	9.231	0.1	57.442	0.01	0
Dia(1,1,1)	Si(1,3,17)	63.18	89.06	2.703	0.03	56.157	0	0
Dia(1,1,1)	Si(3,11,13)	63.18	89.06	2.698	0.03	56.147	0	0
Dia(1,1,1)	Si(7,9,13)	63.18	88.99	2.703	0.03	56.156	0	0
Dia(1,1,1)	Si(4,12,12)	64.14	83.62	8.511	0.1	53.742	0.01	0
Dia(1,1,1)	Si(1,9,15)	64.73	83.18	2.462	0.03	52.42	0	0
Dia(1,1,1)	Si(4,10,14)	65.73	77.86	7.798	0.1	49.971	0.01	0
Dia(1,1,1)	Si(1,5,17)	66.34	77.09	2.257	0.03	48.582	0	0
Dia(1,1,1)	Si(0,8,16)	67.4	72.08	7.194	0.1	46.103	0.01	0
Dia(1,1,1)	Si(3,5,17)	68.05	70.88	2.083	0.03	44.702	0	0
Dia(1,1,1)	Si(7,7,15)	68.05	70.84	2.087	0.03	44.699	0	0
Dia(1,1,1)	Si(0,2,18)	69.18	65.73	7.065	0.11	42.096	0.01	0
Dia(1,1,1)	Si(5,9,15)	69.88	64.4	1.898	0.03	40.639	0	0
Dia(1,1,1)	Si(4,8,16)	71.09	59.33	6.405	0.11	37.835	0.01	0
Dia(1,1,1)	Si(1,1,15)	51.04	59.09	8.177	0.14	90.389	0.01	0
Dia(1,1,1)	Si(7,11,13)	71.85	58.41	1.705	0.03	36.35	0	0
Dia(1,1,1)	Si(1,7,17)	71.85	58.15	1.707	0.03	36.36	0	0
Dia(1,1,1)	Si(1,3,15)	52.29	55.66	6.551	0.12	86.491	0	0
Dia(1,1,1)	Si(2,4,18)	73.18	51.92	5.909	0.11	33.503	0.01	0
Dia(1,1,1)	Si(3,7,17)	74.02	51.2	1.645	0.03	31.718	0	0
Dia(1,1,1)	Si(1,11,15)	74.02	51.19	1.648	0.03	31.705	0	0
Dia(1,1,1)	Si(8,12,12)	75.53	44.75	5.385	0.12	44.061	0.03	0
Dia(1,1,1)	Si(3,11,15)	76.51	43.72	2.111	0.05	26.535	0	0
Dia(1,1,1)	Si(0,6,18)	78.3	35.82	5.03	0.14	34.774	0.16	0
Dia(1,1,1)	Si(1,1,19)	79.51	31.96	2.769	0.09	20.344	0.01	0
Dia(1,1,1)	Si(1,3,19)	83.76	18.96	2.506	0.13	12.1	0.18	0.01

Ei = 11.271 keV			Cryst 1-2			Cryst 1-2-3		
Cryst 1	Cryst 2,3	ΘB 2 [°]	ΔE [meV]	∫IR dΘdE [µrad meV]	FOM	ΔE [meV]	∫IR dΘdE [µrad meV]	FOM
Si(1,1,1)	Si(1,1,1)	10.1	1061.33	11765.691	11.09	4213.99	10166.31	2.41
Si(1,1,1)	Si(0,2,2)	16.65	620.9	6517.94	10.5	420.427	3936.25	9.36
Si(1,1,1)	Si(1,1,3)	19.63	434.26	3102.123	7.14	182.507	1076.73	5.9
Si(1,1,1)	Si(0,0,4)	23.9	365.87	2909.729	7.95	159.6	1081.72	6.78
Si(1,1,1)	Si(1,3,3)	26.2	312.8	1572.295	5.03	89.149	379.13	4.25
Si(1,1,1)	Si(2,2,4)	29.74	276.73	1714.548	6.2	94.293	489.61	5.19
Si(1,1,1)	Si(1,1,5)	31.75	249.95	967.038	3.87	54.39	168.53	3.1
Si(1,1,1)	Si(0,4,4)	34.95	225.11	1128.831	5.01	60.796	261	4.29
Si(1,1,1)	Si(1,3,5)	36.81	206.08	649.297	3.15	38.451	86.89	2.26
Si(1,1,1)	Si(0,2,6)	39.83	187.64	792.1	4.22	42.538	148.43	3.49
Si(1,1,1)	Si(3,3,5)	41.61	173.56	459.236	2.65	117.174	54.35	0.46
Si(1,1,1)	Si(4,4,4)	44.56	158.34	579.88	3.66	31.714	89.97	2.84
Si(1,1,1)	Si(1,1,7)	46.32	146.8	336.86	2.29	97.254	37.99	0.39
Si(1,1,1)	Si(2,4,6)	49.28	133.08	435.854	3.28	24.828	57.43	2.31
Si(1,1,1)	Si(1,3,7)	51.07	124.01	253.113	2.04	81.24	27.62	0.34
Si(1,1,1)	Si(0,0,8)	54.11	112.84	336.58	2.98	20.281	41.62	2.05
Si(1,1,1)	Si(3,3,7)	55.99	103.51	194.979	1.88	30.521	19.2	0.63
Si(1,1,1)	Si(0,6,6)	59.24	92.23	263.997	2.86	31.394	31.1	0.99
Si(1,1,1)	Si(1,5,7)	61.29	83.99	152.405	1.81	101.067	14.38	0.14
Si(1,1,1)	Si(0,4,8)	64.93	73.04	210.581	2.88	254.114	24.41	0.1
Si(1,1,1)	Si(1,1,9)	67.31	64.39	120.647	1.87	78.796	10.94	0.14
Si(1,1,1)	Si(4,6,6)	71.81	51.55	169.846	3.29	183.238	22.4	0.12
Si(1,1,1)	Si(1,3,9)	75.03	41.51	96.976	2.34	52.61	9.07	0.17
Si(1,1,1)	Si(4,4,8)	82.87	21.68	138.853	6.41	8.587	35.95	4.19
Dia(1,1,1)	Si(1,1,1)	10.1	649.88	7212.276	11.1	4290.6	6534.99	1.52
Dia(1,1,1)	Si(0,2,2)	16.65	432.11	4742.206	10.97	407.116	4144.14	10.18
Dia(1,1,1)	Si(1,1,3)	19.63	311.81	2412.019	7.74	182.666	1168.35	6.4
Dia(1,1,1)	Si(0,0,4)	23.9	275.2	2348.103	8.53	158.211	1178.89	7.45
Dia(1,1,1)	Si(1,3,3)	26.2	237.64	1297.345	5.46	88.459	410.42	4.64
Dia(1,1,1)	Si(2,2,4)	29.74	215.68	1443.995	6.7	93.209	532.01	5.71
Dia(1,1,1)	Si(1,1,5)	31.75	197.25	825.213	4.18	55.114	185.15	3.36
Dia(1,1,1)	Si(0,4,4)	34.95	179.98	978.907	5.44	60.553	285.2	4.71
Dia(1,1,1)	Si(1,3,5)	36.81	169.45	565.935	3.34	730.863	97.49	0.13
Dia(1,1,1)	Si(0,2,6)	39.83	155.37	696.802	4.48	42.822	164.65	3.84
Dia(1,1,1)	Si(3,3,5)	41.61	147.49	408.364	2.77	219.638	59.25	0.27
Dia(1,1,1)	Si(4,4,4)	44.56	135.14	518.564	3.84	205.823	98.17	0.48
Dia(1,1,1)	Si(1,1,7)	46.32	127.92	302.537	2.37	182.965	41.55	0.23
Dia(1,1,1)	Si(2,4,6)	49.28	116.88	396.944	3.4	393.451	63.97	0.16
Dia(1,1,1)	Si(1,3,7)	51.07	111.95	230.766	2.06	171.759	28.42	0.17
Dia(1,1,1)	Si(0,0,8)	54.11	99.85	310.069	3.11	20.264	46.64	2.3
Dia(1,1,1)	Si(3,3,7)	55.99	95.72	180.606	1.89	128.204	20.78	0.16
Dia(1,1,1)	Si(0,6,6)	59.24	83.35	245.567	2.95	31.426	33.8	1.08
Dia(1,1,1)	Si(1,5,7)	61.29	78.91	142.807	1.81	236.526	15.57	0.07
Dia(1,1,1)	Si(0,4,8)	64.93	66.81	198.124	2.97	13.155	27.27	2.07
Dia(1,1,1)	Si(1,1,9)	67.31	61.34	114.705	1.87	42.116	11.82	0.28
Dia(1,1,1)	Si(4,6,6)	71.81	47.57	161.855	3.4	10.282	24.12	2.35
Dia(1,1,1)	Si(1,3,9)	75.03	39.45	92.845	2.35	14.139	9.88	0.7
Dia(1,1,1)	Si(4,4,8)	82.87	19.56	134.968	6.9	8.599	38.98	4.53

Ei = 10.739 keV			Cryst 1-2			Cryst 1-2-3		
Cryst 1	Cryst 2,3	ΘB 2 [°]	ΔE [meV]	∫IR dΘdE [μrad meV]	FOM	ΔE [meV]	∫IR dΘdE [μrad meV]	FOM
Si(1,1,1)	Si(1,1,1)	10.61	1017.21	11368.608	11.18	4136.75	9712.91	2.35
Si(1,1,1)	Si(0,2,2)	17.5	591.87	6305.916	10.65	404.842	3787.4	9.36
Si(1,1,1)	Si(1,1,3)	20.64	407.21	2984.707	7.33	173.516	1036.26	5.97
Si(1,1,1)	Si(0,0,4)	25.16	342.61	2812.359	8.21	152.302	1051.77	6.91
Si(1,1,1)	Si(1,3,3)	27.6	289.51	1507.918	5.21	85.215	366.79	4.3
Si(1,1,1)	Si(2,2,4)	31.38	255.19	1649.251	6.46	89.366	479.73	5.37
Si(1,1,1)	Si(1,1,5)	33.52	228.37	924.352	4.05	52.232	167.97	3.22
Si(1,1,1)	Si(0,4,4)	36.96	204.94	1083.558	5.29	58.61	257.25	4.39
Si(1,1,1)	Si(1,3,5)	38.96	186	618.889	3.33	35.748	85.26	2.39
Si(1,1,1)	Si(0,2,6)	42.24	168.43	759.729	4.51	40.599	154.32	3.8
Si(1,1,1)	Si(3,3,5)	44.19	154.31	436.574	2.83	27.792	51.12	1.84
Si(1,1,1)	Si(4,4,4)	47.43	139.71	554.964	3.97	30.379	92.69	3.05
Si(1,1,1)	Si(1,1,7)	49.38	128.04	319.378	2.49	85.957	35.01	0.41
Si(1,1,1)	Si(2,4,6)	52.69	115.51	416.399	3.6	23.096	61.4	2.66
Si(1,1,1)	Si(1,3,7)	54.73	105.51	240.006	2.27	70.18	26.36	0.38
Si(1,1,1)	Si(3,5,5)	54.73	105.51	240.006	2.27	70.18	26.36	0.38
Si(1,1,1)	Si(0,0,8)	58.25	93.67	320.44	3.42	18.298	44.33	2.42
Si(1,1,1)	Si(3,3,7)	60.46	84.69	183.898	2.17	56.136	18.86	0.34
Si(1,1,1)	Si(0,6,6)	64.41	72.81	250.985	3.45	14.602	34.86	2.39
Si(1,1,1)	Si(1,5,7)	67	63.6	143.398	2.25	182.608	14.23	0.08
Si(1,1,1)	Si(5,5,5)	67	63.6	143.398	2.25	182.609	14.23	0.08
Si(1,1,1)	Si(0,4,8)	71.93	50.42	199.867	3.96	182.383	32.37	0.18
Si(1,1,1)	Si(1,1,9)	75.55	39.22	113.502	2.89	116.267	12.71	0.11
Si(1,1,1)	Si(4,6,6)	85.62	17.32	161.554	9.33	10.475	68.44	6.53
Dia(1,1,1)	Si(1,1,1)	10.61	620.38	7018.549	11.31	4779.57	6356.71	1.33
Dia(1,1,1)	Si(0,2,2)	17.5	415.81	4651.613	11.19	392.069	4015.67	10.24
Dia(1,1,1)	Si(1,1,3)	20.64	296.33	2359.041	7.96	173.893	1130.9	6.5
Dia(1,1,1)	Si(0,0,4)	25.16	261.28	2314.079	8.86	151.667	1168.37	7.7
Dia(1,1,1)	Si(1,3,3)	27.6	223.7	1270.548	5.68	84.966	400.64	4.72
Dia(1,1,1)	Si(2,2,4)	31.38	202.58	1419.373	7.01	89.975	523.24	5.82
Dia(1,1,1)	Si(1,1,5)	33.52	182.59	802.751	4.4	51.834	181.28	3.5
Dia(1,1,1)	Si(0,4,4)	36.96	167.58	960.204	5.73	58.138	283.02	4.87
Dia(1,1,1)	Si(1,3,5)	38.96	157.24	551.696	3.51	35.717	94.19	2.64
Dia(1,1,1)	Si(0,2,6)	42.24	142.62	684.186	4.8	40.965	164.66	4.02
Dia(1,1,1)	Si(3,3,5)	44.19	134.4	397.358	2.96	448.912	56.44	0.13
Dia(1,1,1)	Si(4,4,4)	47.43	121.96	509.35	4.18	417.272	100.49	0.24
Dia(1,1,1)	Si(1,1,7)	49.38	115.05	294.96	2.56	161.352	38.59	0.24
Dia(1,1,1)	Si(2,4,6)	52.69	103.14	388.458	3.77	341.035	67.11	0.2
Dia(1,1,1)	Si(1,3,7)	54.73	97.54	225.258	2.31	302.09	26.65	0.09
Dia(1,1,1)	Si(3,5,5)	54.73	97.54	225.258	2.31	302.091	26.65	0.09
Dia(1,1,1)	Si(0,0,8)	58.25	85.28	302.549	3.55	335.121	47.8	0.14
Dia(1,1,1)	Si(3,3,7)	60.46	79.63	174.841	2.2	290.49	20.02	0.07
Dia(1,1,1)	Si(0,6,6)	64.41	67.09	240.2	3.58	14.551	37.32	2.56
Dia(1,1,1)	Si(1,5,7)	67	60.91	138.058	2.27	42.492	15.22	0.36
Dia(1,1,1)	Si(5,5,5)	67	60.91	138.058	2.27	42.492	15.22	0.36
Dia(1,1,1)	Si(0,4,8)	71.93	46.55	193.794	4.16	11.861	35.53	3
Dia(1,1,1)	Si(1,1,9)	75.55	37.18	110.657	2.98	7.713	14.4	1.87
Dia(1,1,1)	Si(4,6,6)	85.62	16.54	160.251	9.69	10.41	74.71	7.18

Ei = 9.561 keV				Cryst 1-2			Cryst 1-2-3	
Cryst 1	Cryst 2,3	ΘB 2	ΔE	∫IR dΘdE	FOM	ΔE	∫IR dΘdE	FOM
		[°]	[meV]	[μrad meV]		[meV]	[μrad meV]	
Si(1,1,1)	Si(1,1,1)	11.93	917.81	10366.049	11.29	3561.11	8657.45	2.43
Si(1,1,1)	Si(0,2,2)	19.74	527.28	5776.871	10.96	366.454	3429.02	9.36
Si(1,1,1)	Si(1,1,3)	23.33	347.76	2690.343	7.74	155.584	946.7	6.08
Si(1,1,1)	Si(0,0,4)	28.52	291.18	2556.794	8.78	138.401	1001.78	7.24
Si(1,1,1)	Si(1,3,3)	31.36	237.67	1347.269	5.67	77.533	333.32	4.3
Si(1,1,1)	Si(2,2,4)	35.79	209.1	1489.214	7.12	79.679	443.04	5.56
Si(1,1,1)	Si(1,1,5)	38.34	180.36	820.465	4.55	46.68	157.45	3.37
Si(1,1,1)	Si(0,4,4)	42.48	160.84	973.921	6.06	53.581	245.45	4.58
Si(1,1,1)	Si(1,3,5)	44.93	141.75	544.886	3.84	31.447	85.68	2.72
Si(1,1,1)	Si(0,2,6)	49.03	126.42	678.229	5.36	37.3	151.39	4.06
Si(1,1,1)	Si(3,3,5)	51.52	111.82	382.549	3.42	22.897	50.45	2.2
Si(1,1,1)	Si(4,4,4)	55.8	98.57	492.511	5	27.422	101.03	3.68
Si(1,1,1)	Si(1,1,7)	58.49	86.29	277.727	3.22	17.216	33.88	1.97
Si(1,1,1)	Si(1,5,5)	58.49	86.29	277.727	3.22	17.216	33.88	1.97
Si(1,1,1)	Si(2,4,6)	63.3	73.32	368.509	5.03	20.781	77.3	3.72
Si(1,1,1)	Si(1,3,7)	66.49	61.65	207.264	3.36	13.186	26.92	2.04
Si(1,1,1)	Si(0,0,8)	72.76	47.05	282.394	6	16.685	66.39	3.98
Si(1,1,1)	Si(3,3,7)	77.74	32.51	157.869	4.86	10.839	29.8	2.75
Dia(1,1,1)	Si(1,1,1)	11.93	554.45	6554.717	11.82	4105.22	5817.07	1.42
Dia(1,1,1)	Si(0,2,2)	19.74	379.3	4437.237	11.7	2925.58	3739.83	1.28
Dia(1,1,1)	Si(1,1,3)	23.33	263.3	2246.258	8.53	154.541	1043.16	6.75
Dia(1,1,1)	Si(0,0,4)	28.52	230.96	2219.742	9.61	138.724	1118.71	8.06
Dia(1,1,1)	Si(1,3,3)	31.36	192.39	1204.429	6.26	77.139	370.92	4.81
Dia(1,1,1)	Si(2,2,4)	35.79	173.28	1354.057	7.81	77.661	506.92	6.53
Dia(1,1,1)	Si(1,1,5)	38.34	153.85	755.204	4.91	46.823	178.29	3.81
Dia(1,1,1)	Si(0,4,4)	42.48	138.49	916.076	6.61	53.721	274.21	5.1
Dia(1,1,1)	Si(1,3,5)	44.93	126.66	517.927	4.09	31.816	95.71	3.01
Dia(1,1,1)	Si(0,2,6)	49.03	112.52	652.164	5.8	37.707	168.2	4.46
Dia(1,1,1)	Si(3,3,5)	51.52	102.91	370.02	3.6	334.3	58.54	0.18
Dia(1,1,1)	Si(4,4,4)	55.8	89.72	480.763	5.36	27.785	112.26	4.04
Dia(1,1,1)	Si(1,1,7)	58.49	81.19	273.646	3.37	254.671	39.07	0.15
Dia(1,1,1)	Si(1,5,5)	58.49	81.19	273.646	3.37	254.671	39.07	0.15
Dia(1,1,1)	Si(2,4,6)	63.3	67.99	367.181	5.4	21.213	84.48	3.98
Dia(1,1,1)	Si(1,3,7)	66.49	58.97	207.608	3.52	13.153	30.58	2.32
Dia(1,1,1)	Si(0,0,8)	72.76	43.7	286.657	6.56	16.715	74.13	4.44
Dia(1,1,1)	Si(3,3,7)	77.74	30.67	161.578	5.27	10.81	33.39	3.09

Ei = 11.682 keV			Cryst 1-2			Cryst 1-2-3		
Cryst 1	Cryst 2,3	ΘB 2 [°]	ΔE [meV]	∫IR dΘdE [µrad meV]	FOM	ΔE [meV]	∫IR dΘdE [µrad meV]	FOM
Si(1,1,1)	Si(1,1,1)	9.74	1095.47	12047.402	11	1048.09	10496.23	10.01
Si(1,1,1)	Si(0,2,2)	16.04	643.19	6665.358	10.36	434.508	4024.17	9.26
Si(1,1,1)	Si(1,1,3)	18.91	455.15	3187.391	7	184.513	1109.05	6.01
Si(1,1,1)	Si(0,0,4)	23.01	383.98	2982.313	7.77	165.604	1123.03	6.78
Si(1,1,1)	Si(1,3,3)	25.21	331.36	1618.903	4.89	91.436	368.05	4.03
Si(1,1,1)	Si(2,2,4)	28.6	293.37	1761.001	6	93.717	479.5	5.12
Si(1,1,1)	Si(3,3,3)	30.51	266.92	997.642	3.74	56.323	175.46	3.12
Si(1,1,1)	Si(0,4,4)	33.55	240.94	1160.832	4.82	62.399	252.41	4.05
Si(1,1,1)	Si(1,3,5)	35.31	222.1	670.984	3.02	38.193	94.2	2.47
Si(1,1,1)	Si(0,2,6)	38.17	202.68	816.25	4.03	44.268	150.75	3.41
Si(1,1,1)	Si(3,3,5)	39.85	188.99	475.481	2.52	27.787	52.35	1.88
Si(1,1,1)	Si(4,4,4)	42.61	172.77	597.096	3.46	32.509	93.32	2.87
Si(1,1,1)	Si(1,1,7)	44.25	161.32	349.251	2.17	21.549	33.88	1.57
Si(1,1,1)	Si(2,4,6)	46.99	147.98	451.335	3.05	24.746	60.99	2.46
Si(1,1,1)	Si(1,3,7)	48.64	137.91	263.141	1.91	33.831	21.69	0.64
Si(1,1,1)	Si(0,0,8)	51.41	126.42	347.727	2.75	19.761	40.87	2.07
Si(1,1,1)	Si(3,3,7)	53.11	117.75	203.137	1.73	57.982	15.99	0.28
Si(1,1,1)	Si(0,6,6)	56.01	106.74	273.399	2.56	15.717	29.01	1.85
Si(1,1,1)	Si(5,5,5)	57.8	98.68	158.958	1.61	48.429	12.05	0.25
Si(1,1,1)	Si(0,4,8)	60.92	87.92	218.105	2.48	12.843	22.13	1.72
Si(1,1,1)	Si(1,1,9)	62.9	80.22	126.298	1.57	39.358	9.16	0.23
Si(1,1,1)	Si(4,6,6)	66.43	69.32	176.426	2.55	10.555	18.27	1.73
Si(1,1,1)	Si(1,3,9)	68.76	61.13	101.378	1.66	14.898	6.95	0.47
Si(1,1,1)	Si(4,4,8)	73.21	48.27	144.207	2.99	8.795	18.3	2.08
Si(1,1,1)	Si(1,7,7)	76.46	38.28	82.502	2.16	5.771	7.1	1.23
Si(1,1,1)	Si(0,2,10)	85.17	16.01	119.496	7.47	7.419	38.1	5.14

Ei = 11.682 keV			Cryst 1-2			Cryst 1-2-3		
Cryst 1	Cryst 2,3	ΘB 2 [°]	ΔE [meV]	∫IR dΘdE [μrad meV]	FOM	ΔE [meV]	∫IR dΘdE [μrad meV]	FOM
Dia(1,1,1)	Si(1,1,1)	9.74	672.53	7346.487	10.92	3876.41	6750.29	1.74
Dia(1,1,1)	Si(0,2,2)	16.04	444.27	4801.308	10.81	418.488	4200.85	10.04
Dia(1,1,1)	Si(1,1,3)	18.91	322.28	2440.351	7.57	187.076	1203.26	6.43
Dia(1,1,1)	Si(0,0,4)	23.01	285.87	2368.574	8.29	164.985	1213.02	7.35
Dia(1,1,1)	Si(1,3,3)	25.21	248.26	1311.104	5.28	90.902	398.18	4.38
Dia(1,1,1)	Si(2,2,4)	28.6	225.79	1461.376	6.47	93.339	519.52	5.57
Dia(1,1,1)	Si(3,3,3)	30.51	207.26	838.418	4.05	56.226	190.23	3.38
Dia(1,1,1)	Si(0,4,4)	33.55	189.69	992.949	5.23	62.856	270.99	4.31
Dia(1,1,1)	Si(1,3,5)	35.31	179.71	577.717	3.21	37.65	102.83	2.73
Dia(1,1,1)	Si(0,2,6)	38.17	164.81	707.285	4.29	44.362	162.62	3.67
Dia(1,1,1)	Si(3,3,5)	39.85	157.57	418.076	2.65	27.642	56.22	2.03
Dia(1,1,1)	Si(4,4,4)	42.61	144.94	525.476	3.63	32.73	100.93	3.08
Dia(1,1,1)	Si(1,1,7)	44.25	137.74	309.629	2.25	21.574	35.07	1.63
Dia(1,1,1)	Si(2,4,6)	46.99	126.52	402.228	3.18	25.248	66.13	2.62
Dia(1,1,1)	Si(1,3,7)	48.64	120.72	234.913	1.95	33.783	23.85	0.71
Dia(1,1,1)	Si(0,0,8)	51.41	110.34	313.673	2.84	19.566	44.4	2.27
Dia(1,1,1)	Si(3,3,7)	53.11	106.06	183.752	1.73	57.979	16.97	0.29
Dia(1,1,1)	Si(0,6,6)	56.01	95.03	249.708	2.63	15.724	31.4	2
Dia(1,1,1)	Si(5,5,5)	57.8	91.48	146.142	1.6	48.468	12.69	0.26
Dia(1,1,1)	Si(0,4,8)	60.92	79.79	201.397	2.52	12.778	23.78	1.86
Dia(1,1,1)	Si(1,1,9)	62.9	75.46	117.403	1.56	39.456	9.73	0.25
Dia(1,1,1)	Si(4,6,6)	66.43	63.5	164.701	2.59	10.53	19.9	1.89
Dia(1,1,1)	Si(1,3,9)	68.76	58.02	95.462	1.65	151.243	7.59	0.05
Dia(1,1,1)	Si(4,4,8)	73.21	44.37	135.842	3.06	105.188	19.76	0.19
Dia(1,1,1)	Si(1,7,7)	76.46	36.09	78.29	2.17	97.736	7.68	0.08
Dia(1,1,1)	Si(0,2,10)	85.17	14.5	114.894	7.93	7.573	40.61	5.36

Ei = 11.136 keV			Cryst 1-2			Cryst 1-2-3		
Cryst 1	Cryst 2,3	ΘB 2 [°]	ΔE [meV]	∫IR dΘdE [μrad meV]	FOM	ΔE [meV]	∫IR dΘdE [μrad meV]	FOM
Si(1,1,1)	Si(1,1,1)	10.23	1050.13	11667.901	11.11	1000.67	10056.45	10.05
Si(1,1,1)	Si(0,2,2)	16.85	613.49	6465.547	10.54	417.489	3890.94	9.32
Si(1,1,1)	Si(1,1,3)	19.87	427.29	3072.474	7.19	177.778	1074.46	6.04
Si(1,1,1)	Si(0,0,4)	24.2	360.19	2885.764	8.01	159.416	1086.51	6.82
Si(1,1,1)	Si(1,3,3)	26.54	306.44	1555.237	5.08	87.135	356.37	4.09
Si(1,1,1)	Si(2,2,4)	30.14	271.26	1698.537	6.26	89.675	473.08	5.28
Si(1,1,1)	Si(3,3,3)	32.18	244.92	956.41	3.91	54.055	170.66	3.16
Si(1,1,1)	Si(0,4,4)	35.44	220	1116.863	5.08	59.51	246.36	4.14
Si(1,1,1)	Si(1,3,5)	37.33	200.94	640.875	3.19	36.333	91.65	2.52
Si(1,1,1)	Si(0,2,6)	40.41	182.79	783.533	4.29	42.573	147.7	3.47
Si(1,1,1)	Si(3,3,5)	42.23	168.38	453.667	2.69	26.277	52.29	1.99
Si(1,1,1)	Si(4,4,4)	45.25	153.55	572.855	3.73	31.266	94.09	3.01
Si(1,1,1)	Si(1,1,7)	47.05	141.72	332.353	2.35	19.972	32.24	1.61
Si(1,1,1)	Si(2,4,6)	50.09	129.22	431.714	3.34	23.83	62.98	2.64
Si(1,1,1)	Si(1,3,7)	51.94	119.26	250.172	2.1	15.966	21.35	1.34
Si(1,1,1)	Si(0,0,8)	55.09	107.67	332.264	3.09	18.684	43.81	2.34
Si(1,1,1)	Si(3,3,7)	57.03	98.79	191.821	1.94	13.013	15.27	1.17
Si(1,1,1)	Si(0,6,6)	60.43	87.99	260.986	2.97	14.954	32.7	2.19
Si(1,1,1)	Si(5,5,5)	62.58	79.74	149.763	1.88	10.441	11.49	1.1
Si(1,1,1)	Si(0,4,8)	66.46	67.33	207.466	3.08	12.305	26.67	2.17
Si(1,1,1)	Si(1,1,9)	69.04	58.68	118.885	2.03	8.098	9.71	1.2
Si(1,1,1)	Si(3,5,7)	69.04	58.68	118.885	2.03	8.098	9.71	1.2
Si(1,1,1)	Si(4,6,6)	74.06	44.98	167.798	3.73	10.303	25.58	2.48
Si(1,1,1)	Si(1,3,9)	77.9	33.45	95.451	2.85	6.625	11.04	1.67
Dia(1,1,1)	Si(1,1,1)	10.23	642.37	7163.674	11.15	3638.87	6495.51	1.79
Dia(1,1,1)	Si(0,2,2)	16.85	427.99	4720.88	11.03	404.831	4103.36	10.14
Dia(1,1,1)	Si(1,1,3)	19.87	307.28	2396.517	7.8	178.934	1171.28	6.55
Dia(1,1,1)	Si(0,0,4)	24.2	271.73	2340.52	8.61	155.305	1170.37	7.54
Dia(1,1,1)	Si(1,3,3)	26.54	234.11	1290.924	5.51	86.507	386.43	4.47
Dia(1,1,1)	Si(2,2,4)	30.14	212.4	1439.223	6.78	89.039	515.47	5.79
Dia(1,1,1)	Si(3,3,3)	32.18	193.31	819.039	4.24	54.268	185.33	3.42
Dia(1,1,1)	Si(0,4,4)	35.44	176.92	974.497	5.51	59.05	269.71	4.57
Dia(1,1,1)	Si(1,3,5)	37.33	166.48	562.643	3.38	35.856	102.58	2.86
Dia(1,1,1)	Si(0,2,6)	40.41	152.18	693.549	4.56	42.242	162.03	3.84
Dia(1,1,1)	Si(3,3,5)	42.23	144.32	405.696	2.81	26.219	56.98	2.17
Dia(1,1,1)	Si(4,4,4)	45.25	131.93	516.295	3.91	31.382	102.04	3.25
Dia(1,1,1)	Si(1,1,7)	47.05	124.66	300.738	2.41	19.983	35.65	1.78
Dia(1,1,1)	Si(2,4,6)	50.09	113.48	394.993	3.48	23.652	68.55	2.9
Dia(1,1,1)	Si(1,3,7)	51.94	107.74	229.457	2.13	16.006	22.66	1.42
Dia(1,1,1)	Si(0,0,8)	55.09	96.26	306.264	3.18	18.996	47.41	2.5
Dia(1,1,1)	Si(3,3,7)	57.03	91.83	179.282	1.95	13.025	16.19	1.24
Dia(1,1,1)	Si(0,6,6)	60.43	79.46	244.166	3.07	14.793	36.36	2.46
Dia(1,1,1)	Si(5,5,5)	62.58	74.8	141.721	1.89	10.452	12.19	1.17
Dia(1,1,1)	Si(0,4,8)	66.46	62.1	196.518	3.16	12.096	29.96	2.48
Dia(1,1,1)	Si(1,1,9)	69.04	56.08	113.533	2.02	8.153	9.85	1.21
Dia(1,1,1)	Si(3,5,7)	69.04	56.08	113.533	2.02	8.153	9.85	1.21
Dia(1,1,1)	Si(4,6,6)	74.06	41.43	161.204	3.89	10.313	27.8	2.7
Dia(1,1,1)	Si(1,3,9)	77.9	31.35	92.127	2.94	6.504	12.27	1.89

Ei = 9.881 keV				Cryst 1-2			Cryst 1-2-3	
Cryst 1	Cryst 2,3	ΘB 2	ΔE	∫IR dΘdE	FOM	ΔE	∫IR dΘdE	FOM
		[°]	[meV]	[μrad meV]		[meV]	[μrad meV]	
Si(1,1,1)	Si(1,1,1)	11.54	945.2	10667.053	11.29	893.331	8951.08	10.02
Si(1,1,1)	Si(0,2,2)	19.07	544.97	5930.184	10.88	379.447	3560.53	9.38
Si(1,1,1)	Si(1,1,3)	22.53	363.74	2774.273	7.63	161.202	977.49	6.06
Si(1,1,1)	Si(0,0,4)	27.52	304.63	2637.512	8.66	145.314	1017.06	7
Si(1,1,1)	Si(1,3,3)	30.23	251.63	1394.024	5.54	77.204	330.3	4.28
Si(1,1,1)	Si(2,2,4)	34.47	221.3	1535.314	6.94	80.446	454.48	5.65
Si(1,1,1)	Si(3,3,3)	36.89	193.05	849.618	4.4	48.512	156.45	3.22
Si(1,1,1)	Si(0,4,4)	40.8	173.05	1003.186	5.8	52.444	239.75	4.57
Si(1,1,1)	Si(1,3,5)	43.11	153.54	566.024	3.69	32.145	88.48	2.75
Si(1,1,1)	Si(0,2,6)	46.94	137.67	701.351	5.09	36.635	145.71	3.98
Si(1,1,1)	Si(3,3,5)	49.25	123.71	398.167	3.22	23.419	52.65	2.25
Si(1,1,1)	Si(4,4,4)	53.16	109.67	510.659	4.66	27.783	95.66	3.44
Si(1,1,1)	Si(1,1,7)	55.59	97.82	289.497	2.96	17.622	34.58	1.96
Si(1,1,1)	Si(2,4,6)	59.82	85.58	382.113	4.46	21.706	67.33	3.1
Si(1,1,1)	Si(1,3,7)	62.54	74.44	216.68	2.91	13.628	25.08	1.84
Si(1,1,1)	Si(0,0,8)	67.54	61.33	293.082	4.78	16.318	54.8	3.36
Si(1,1,1)	Si(3,3,7)	71.01	49.94	165.462	3.31	10.889	21.55	1.98
Si(1,1,1)	Si(0,6,6)	78.58	32.85	229.182	6.98	13.683	63.13	4.61
Dia(1,1,1)	Si(1,1,1)	11.54	572.65	6688.881	11.68	2670.02	5888.94	2.21
Dia(1,1,1)	Si(0,2,2)	19.07	389.18	4500.215	11.56	364.124	3840.76	10.55
Dia(1,1,1)	Si(1,1,3)	22.53	271.96	2275.716	8.37	159.003	1079.65	6.79
Dia(1,1,1)	Si(0,0,4)	27.52	239.51	2249.471	9.39	144.049	1125.8	7.82
Dia(1,1,1)	Si(1,3,3)	30.23	200.36	1224.456	6.11	76.975	364.98	4.74
Dia(1,1,1)	Si(2,2,4)	34.47	180.99	1372.252	7.58	76.503	511	6.68
Dia(1,1,1)	Si(3,3,3)	36.89	162.78	770.177	4.73	48.219	174.16	3.61
Dia(1,1,1)	Si(0,4,4)	40.8	146.74	925.607	6.31	52.421	266.13	5.08
Dia(1,1,1)	Si(1,3,5)	43.11	135.31	528.063	3.9	32.663	96.73	2.96
Dia(1,1,1)	Si(0,2,6)	46.94	120.9	662.244	5.48	37.064	159.88	4.31
Dia(1,1,1)	Si(3,3,5)	49.25	112.05	374.861	3.35	23.166	60.03	2.59
Dia(1,1,1)	Si(4,4,4)	53.16	99.06	490.394	4.95	28.005	105.11	3.75
Dia(1,1,1)	Si(1,1,7)	55.59	91.33	280.029	3.07	17.66	38.17	2.16
Dia(1,1,1)	Si(2,4,6)	59.82	78.38	373.012	4.76	21.543	75.3	3.5
Dia(1,1,1)	Si(1,3,7)	62.54	70.74	212.42	3	13.613	27.88	2.05
Dia(1,1,1)	Si(0,0,8)	67.54	57.05	290.9	5.1	16.572	60.03	3.62
Dia(1,1,1)	Si(3,3,7)	71.01	47.67	164.796	3.46	10.944	23.79	2.17
Dia(1,1,1)	Si(0,6,6)	78.58	30.32	232.052	7.65	13.577	70.23	5.17

Ei = 12.1 keV			Cryst 1-2			Cryst 1-2-3		
Cryst 1	Cryst 2,3	ΘB 2 [°]	ΔE [meV]	∫IR dΘdE [μrad meV]	FOM	ΔE [meV]	∫IR dΘdE [μrad meV]	FOM
Si(1,1,1)	Si(1,1,1)	9.4	1129.72	12313.505	10.9	1079.25	10765.87	9.98
Si(1,1,1)	Si(0,2,2)	15.47	665.87	6802.386	10.22	447.167	4116.38	9.21
Si(1,1,1)	Si(1,1,3)	18.23	476.53	3268.109	6.86	191.233	1137.69	5.95
Si(1,1,1)	Si(0,0,4)	22.17	403.06	3056.931	7.58	170.574	1142.34	6.7
Si(1,1,1)	Si(1,3,3)	24.28	350	1662.457	4.75	95.139	377.82	3.97
Si(1,1,1)	Si(2,2,4)	27.53	310.35	1806.258	5.82	96.907	483.7	4.99
Si(1,1,1)	Si(1,1,5)	29.35	284.5	1027.554	3.61	58.302	179.67	3.08
Si(1,1,1)	Si(0,4,4)	32.25	256.4	1193.4	4.65	64.541	256.28	3.97
Si(1,1,1)	Si(1,3,5)	33.92	238.52	692.896	2.9	39.385	95.09	2.41
Si(1,1,1)	Si(0,2,6)	36.63	218.12	839.697	3.85	45.832	151.1	3.3
Si(1,1,1)	Si(3,3,5)	38.21	204.28	491.922	2.41	28.702	51.55	1.8
Si(1,1,1)	Si(4,4,4)	40.81	187.5	615.079	3.28	34.017	93.48	2.75
Si(1,1,1)	Si(1,1,7)	42.35	176.07	361.53	2.05	23.143	33.21	1.44
Si(1,1,1)	Si(1,5,5)	42.35	176.07	361.531	2.05	23.143	33.21	1.44
Si(1,1,1)	Si(2,4,6)	44.9	162.46	464.956	2.86	25.734	60.5	2.35
Si(1,1,1)	Si(1,3,7)	46.44	152.76	272.237	1.78	75.316	22.92	0.3
Si(1,1,1)	Si(0,0,8)	49	140.41	358.976	2.56	20.287	39.05	1.92
Si(1,1,1)	Si(3,3,7)	50.55	131.83	211.272	1.6	64.413	17.14	0.27
Si(1,1,1)	Si(0,6,6)	53.17	120.91	282.425	2.34	16.505	27.81	1.68
Si(1,1,1)	Si(1,5,7)	54.78	113	165.801	1.47	54.875	11.94	0.22
Si(1,1,1)	Si(5,5,5)	54.78	113	165.801	1.47	54.876	11.94	0.22
Si(1,1,1)	Si(0,4,8)	57.54	102.53	225.497	2.2	13.688	20.82	1.52
Si(1,1,1)	Si(1,1,9)	59.25	95.17	131.669	1.38	46.117	9.58	0.21
Si(1,1,1)	Si(4,6,6)	62.24	84.71	182.491	2.15	11.31	16.82	1.49
Si(1,1,1)	Si(1,3,9)	64.14	77.68	106.068	1.37	37.666	6.98	0.19
Si(1,1,1)	Si(4,4,8)	67.56	66.76	149.363	2.24	9.228	13.57	1.47
Si(1,1,1)	Si(1,7,7)	69.82	59.01	86.12	1.46	28.849	6.2	0.22
Si(1,1,1)	Si(0,2,10)	74.16	46.15	122.908	2.66	7.662	13.99	1.83
Si(1,1,1)	Si(1,5,9)	77.37	36.31	70.786	1.95	5.131	5.56	1.08
Dia(1,1,1)	Si(1,1,1)	9.4	694.98	7477.616	10.76	697.349	6875.93	9.86
Dia(1,1,1)	Si(0,2,2)	15.47	457.77	4859.134	10.61	430.756	4284.36	9.95
Dia(1,1,1)	Si(1,1,3)	18.23	334.28	2478.084	7.41	194.493	1233.86	6.34
Dia(1,1,1)	Si(0,0,4)	22.17	296.65	2389.582	8.06	171.295	1226.36	7.16
Dia(1,1,1)	Si(1,3,3)	24.28	259.47	1328.509	5.12	93.834	405.83	4.32
Dia(1,1,1)	Si(2,2,4)	27.53	236.01	1475.496	6.25	96.809	518.72	5.36
Dia(1,1,1)	Si(1,1,5)	29.35	216.49	848.466	3.92	57.865	191.95	3.32
Dia(1,1,1)	Si(0,4,4)	32.25	199.28	1004.708	5.04	65.283	275.5	4.22
Dia(1,1,1)	Si(1,3,5)	33.92	188.44	584.759	3.1	39.455	100.71	2.55
Dia(1,1,1)	Si(0,2,6)	36.63	172.58	714.93	4.14	45.238	162.27	3.59
Dia(1,1,1)	Si(3,3,5)	38.21	167.21	426.01	2.55	29.011	56.56	1.95
Dia(1,1,1)	Si(4,4,4)	40.81	154.02	533.488	3.46	33.394	100.75	3.02
Dia(1,1,1)	Si(1,1,7)	42.35	147.2	316.422	2.15	23.152	35.01	1.51
Dia(1,1,1)	Si(1,5,5)	42.35	147.2	316.42	2.15	23.152	35.01	1.51
Dia(1,1,1)	Si(2,4,6)	44.9	136.94	409.308	2.99	25.497	64.88	2.54
Dia(1,1,1)	Si(1,3,7)	46.44	130.68	241.1	1.84	75.412	24.66	0.33
Dia(1,1,1)	Si(0,0,8)	49	120.58	318.87	2.64	20.398	42.33	2.08
Dia(1,1,1)	Si(3,3,7)	50.55	116.01	187.341	1.61	64.412	17.87	0.28
Dia(1,1,1)	Si(0,6,6)	53.17	105.45	252.82	2.4	16.46	29.9	1.82
Dia(1,1,1)	Si(1,5,7)	54.78	102.12	148.445	1.45	54.848	13.74	0.25
Dia(1,1,1)	Si(5,5,5)	54.78	102.12	148.447	1.45	54.848	13.74	0.25
Dia(1,1,1)	Si(0,4,8)	57.54	91.3	203.65	2.23	13.684	22.49	1.64

**Dia(1,1,1)**	**Si(1,1,9)**	**59.25**	**88.15**	**119.724**	**1.36**	**229.347**	**10.88**	**0.05**
**Dia(1,1,1)**	**Si(4,6,6)**	**62.24**	**76.87**	**167.337**	**2.18**	**212.058**	**17.12**	**0.08**
**Dia(1,1,1)**	**Si(1,3,9)**	**64.14**	**72.67**	**97.46**	**1.34**	**67.585**	**8.44**	**0.12**
**Dia(1,1,1)**	**Si(4,4,8)**	**67.56**	**61.18**	**137.981**	**2.26**	**156.58**	**14.67**	**0.09**
**Dia(1,1,1)**	**Si(1,7,7)**	**69.82**	**55.72**	**80.168**	**1.44**	**52.268**	**6.27**	**0.12**
**Dia(1,1,1)**	**Si(0,2,10)**	**74.16**	**42.37**	**115.501**	**2.73**	**7.614**	**15.46**	**2.03**
**Dia(1,1,1)**	**Si(1,5,9)**	**77.37**	**34.06**	**66.483**	**1.95**	**91.93**	**5.98**	**0.07**

Ei = 11.544 keV			Cryst 1-2			Cryst 1-2-3		
Cryst 1	Cryst 2,3	ΘB 2 [°]	ΔE [meV]	∫IR dΘdE [μrad meV]	FOM	ΔE [meV]	∫IR dΘdE [μrad meV]	FOM
Si(1,1,1)	Si(1,1,1)	9.86	1084.07	11953.658	11.03	1035.24	10375.47	10.02
Si(1,1,1)	Si(0,2,2)	16.24	635.85	6614.474	10.4	429.944	3990.88	9.28
Si(1,1,1)	Si(1,1,3)	19.14	448.12	3159.956	7.05	182.629	1100.26	6.02
Si(1,1,1)	Si(0,0,4)	23.3	378.22	2960.001	7.83	163.633	1111.26	6.79
Si(1,1,1)	Si(1,3,3)	25.53	324.95	1603.38	4.93	90.42	365.12	4.04
Si(1,1,1)	Si(2,2,4)	28.97	287.8	1746.267	6.07	92.562	477.21	5.16
Si(1,1,1)	Si(1,1,5)	30.92	261.27	987.605	3.78	55.659	174.03	3.13
Si(1,1,1)	Si(0,4,4)	34.01	235.55	1150.479	4.88	61.807	251.22	4.06
Si(1,1,1)	Si(1,3,5)	35.8	216.63	663.712	3.06	37.745	93.6	2.48
Si(1,1,1)	Si(0,2,6)	38.71	196.84	808.671	4.11	43.596	149.48	3.43
Si(1,1,1)	Si(3,3,5)	40.42	183.64	470.249	2.56	27.434	52.44	1.91
Si(1,1,1)	Si(4,4,4)	43.24	167.94	590.97	3.52	32.095	93.82	2.92
Si(1,1,1)	Si(1,1,7)	44.92	156.77	344.781	2.2	21.094	33.11	1.57
Si(1,1,1)	Si(2,4,6)	47.73	143.17	446.206	3.12	24.552	61.33	2.5
Si(1,1,1)	Si(1,3,7)	49.42	133.42	259.378	1.94	32.982	21.89	0.66
Si(1,1,1)	Si(0,0,8)	52.28	121.75	344.232	2.83	19.434	41.9	2.16
Si(1,1,1)	Si(3,3,7)	54.03	113.17	200.56	1.77	55.774	15.69	0.28
Si(1,1,1)	Si(0,6,6)	57.04	101.65	270.16	2.66	15.485	29.74	1.92
Si(1,1,1)	Si(1,5,7)	58.9	94.01	156.969	1.67	46.19	11.76	0.25
Si(1,1,1)	Si(5,5,5)	58.9	94.01	156.968	1.67	46.191	11.76	0.25
Si(1,1,1)	Si(0,4,8)	62.18	82.87	215.606	2.6	12.61	22.97	1.82
Si(1,1,1)	Si(1,1,9)	64.27	75.39	124.536	1.65	37.019	8.94	0.24
Si(1,1,1)	Si(4,6,6)	68.06	63.61	174.282	2.74	10.444	20.04	1.92
Si(1,1,1)	Si(1,3,9)	70.6	54.92	99.843	1.82	7.306	7.16	0.98
Si(1,1,1)	Si(4,4,8)	75.65	41.01	142.467	3.47	8.904	20.57	2.31
Si(1,1,1)	Si(1,7,7)	79.68	29.09	81.222	2.79	5.67	9.44	1.67
Dia(1,1,1)	Si(1,1,1)	9.86	664.78	7302.465	10.98	3814.66	6686.44	1.75
Dia(1,1,1)	Si(0,2,2)	16.24	440.36	4781.736	10.86	416.304	4182.87	10.05
Dia(1,1,1)	Si(1,1,3)	19.14	318.55	2428.797	7.62	184.891	1194.93	6.46
Dia(1,1,1)	Si(0,0,4)	23.3	282	2361.105	8.37	162.104	1203.54	7.42
Dia(1,1,1)	Si(1,3,3)	25.53	244.83	1306.578	5.34	89.902	395.39	4.4
Dia(1,1,1)	Si(2,2,4)	28.97	222.44	1455.634	6.54	92.212	518.59	5.62
Dia(1,1,1)	Si(1,1,5)	30.92	203.31	832.89	4.1	55.797	189.76	3.4
Dia(1,1,1)	Si(0,4,4)	34.01	186.64	985.514	5.28	61.751	271.01	4.39
Dia(1,1,1)	Si(1,3,5)	35.8	176.34	574.192	3.26	37.026	103.16	2.79
Dia(1,1,1)	Si(0,2,6)	38.71	161.54	704.715	4.36	43.395	162.29	3.74
Dia(1,1,1)	Si(3,3,5)	40.42	154.24	414.817	2.69	27.226	56.15	2.06
Dia(1,1,1)	Si(4,4,4)	43.24	141.77	523.822	3.7	32.656	102.15	3.13
Dia(1,1,1)	Si(1,1,7)	44.92	134.5	307.328	2.28	21.179	33.43	1.58
Dia(1,1,1)	Si(2,4,6)	47.73	123.72	400.755	3.24	24.557	68.49	2.79
Dia(1,1,1)	Si(1,3,7)	49.42	117.49	233.737	1.99	32.924	23.56	0.72
Dia(1,1,1)	Si(0,0,8)	52.28	106.98	312.841	2.92	19.373	44.6	2.3
Dia(1,1,1)	Si(3,3,7)	54.03	102.78	182.599	1.78	55.909	16.82	0.3
Dia(1,1,1)	Si(0,6,6)	57.04	91.32	248.306	2.72	15.604	32.64	2.09
Dia(1,1,1)	Si(1,5,7)	58.9	87.48	145.08	1.66	46.302	12.48	0.27
Dia(1,1,1)	Si(5,5,5)	58.9	87.48	145.08	1.66	46.302	12.48	0.27
Dia(1,1,1)	Si(0,4,8)	62.18	75.59	200.462	2.65	12.649	25.01	1.98
Dia(1,1,1)	Si(1,1,9)	64.27	70.86	116.329	1.64	37.085	9.55	0.26
Dia(1,1,1)	Si(4,6,6)	68.06	58.52	163.653	2.8	10.386	21.83	2.1
Dia(1,1,1)	Si(1,3,9)	70.6	52.46	94.519	1.8	138.015	7.7	0.06
Dia(1,1,1)	Si(4,4,8)	75.65	37.45	135.382	3.62	8.946	22.1	2.47

**Dia(1,1,1)**	**Si(1,7,7)**	**79.68**	**27.01**	**77.69**	**2.88**	**5.726**	**10.07**	**1.76**

Ei = 10.207 keV				Cryst 1-2			Cryst 1-2-3	
Cryst 1	Cryst 2,3	ΘB 2 [°]	ΔE [meV]	∫IR dΘdE [µrad meV]	FOM	ΔE [meV]	∫IR dΘdE [µrad meV]	FOM
Si(1,1,1)	Si(1,1,1)	11.17	972.62	10938.969	11.25	922.042	9269.09	10.05
Si(1,1,1)	Si(0,2,2)	18.44	562.62	6074.683	10.8	390.096	3647.4	9.35
Si(1,1,1)	Si(1,1,3)	21.77	380.49	2857.764	7.51	165.585	1009.64	6.1
Si(1,1,1)	Si(0,0,4)	26.57	319.29	2702.458	8.46	149.68	1042.4	6.96
Si(1,1,1)	Si(1,3,3)	29.17	265.98	1438.306	5.41	79.41	335.64	4.23
Si(1,1,1)	Si(2,2,4)	33.22	234.2	1580.213	6.75	82.832	462.75	5.59
Si(1,1,1)	Si(1,1,5)	35.53	206.31	878.669	4.26	49.845	160.37	3.22
Si(1,1,1)	Si(0,4,4)	39.24	184.83	1036.477	5.61	53.903	239.94	4.45
Si(1,1,1)	Si(1,3,5)	41.42	165.85	587.035	3.54	33.343	89.97	2.7
Si(1,1,1)	Si(0,2,6)	45.01	149.13	723.476	4.85	38.98	143.45	3.68
Si(1,1,1)	Si(3,3,5)	47.17	135.03	412.969	3.06	24.173	52.97	2.19
Si(1,1,1)	Si(4,4,4)	50.78	121.27	527.422	4.35	29.241	94.06	3.22
Si(1,1,1)	Si(1,1,7)	53	109.41	300.926	2.75	18.118	33.3	1.84
Si(1,1,1)	Si(2,4,6)	56.81	96.92	395.715	4.08	22.616	65.59	2.9
Si(1,1,1)	Si(1,3,7)	59.2	86.67	225.546	2.6	14.018	23.27	1.66
Si(1,1,1)	Si(3,5,5)	59.2	86.67	225.546	2.6	14.018	23.27	1.66
Si(1,1,1)	Si(0,0,8)	63.46	74.3	303.607	4.09	17.724	49.73	2.81
Si(1,1,1)	Si(3,3,7)	66.26	64.28	172.772	2.69	11.073	18.34	1.66
Si(1,1,1)	Si(0,6,6)	71.61	50.44	237.647	4.71	13.475	44.01	3.27
Si(1,1,1)	Si(1,5,7)	75.57	38.41	134.254	3.5	9.128	18.33	2.01
Dia(1,1,1)	Si(1,1,1)	11.17	590.89	6817.689	11.54	2784.94	6083.47	2.18
Dia(1,1,1)	Si(0,2,2)	18.44	399.43	4560.948	11.42	374.578	3920.92	10.47
Dia(1,1,1)	Si(1,1,3)	21.77	281.59	2308.877	8.2	163.655	1102.46	6.74
Dia(1,1,1)	Si(0,0,4)	26.57	247.99	2277.089	9.18	147.217	1147.55	7.79
Dia(1,1,1)	Si(1,3,3)	29.17	209.34	1244.781	5.95	79.253	370.01	4.67
Dia(1,1,1)	Si(2,2,4)	33.22	189.39	1390.347	7.34	82.248	515.26	6.26
Dia(1,1,1)	Si(1,1,5)	35.53	170.99	784.261	4.59	49.748	176.98	3.56
Dia(1,1,1)	Si(0,4,4)	39.24	154.69	940.089	6.08	53.827	263.66	4.9
Dia(1,1,1)	Si(1,3,5)	41.42	143.89	537.261	3.73	33.491	97.83	2.92
Dia(1,1,1)	Si(0,2,6)	45.01	129.41	671.913	5.19	39.012	158.68	4.07
Dia(1,1,1)	Si(3,3,5)	47.17	121.53	385.505	3.17	23.953	58.77	2.45
Dia(1,1,1)	Si(4,4,4)	50.78	107.99	498.008	4.61	29.263	103.78	3.55
Dia(1,1,1)	Si(1,1,7)	53	100.55	286.535	2.85	18.135	37.13	2.05
Dia(1,1,1)	Si(2,4,6)	56.81	88.35	379.573	4.3	22.72	72.19	3.18
Dia(1,1,1)	Si(1,3,7)	59.2	81.63	217.432	2.66	13.967	25.81	1.85
Dia(1,1,1)	Si(3,5,5)	59.2	81.63	217.432	2.66	13.968	25.81	1.85
Dia(1,1,1)	Si(0,0,8)	63.46	68.26	294.962	4.32	17.8	54.74	3.08
Dia(1,1,1)	Si(3,3,7)	66.26	61.42	168.456	2.74	11.111	20.21	1.82
Dia(1,1,1)	Si(0,6,6)	71.61	46.83	234.873	5.02	13.576	48.15	3.55
Dia(1,1,1)	Si(1,5,7)	75.57	36.39	133.459	3.67	9.136	20.18	2.21

Ei = 12.527 keV			Cryst 1-2			Cryst 1-2-3		
Cryst 1	Cryst 2,3	ΘB 2 [°]	ΔE [meV]	∫IR dΘdE [μrad meV]	FOM	ΔE [meV]	∫IR dΘdE [μrad meV]	FOM
Si(1,1,1)	Si(1,1,1)	9.08	1164.65	12574.762	10.8	1113.03	11064.84	9.94
Si(1,1,1)	Si(0,2,2)	14.93	689.26	6926.801	10.05	461.767	4197.19	9.09
Si(1,1,1)	Si(1,1,3)	17.59	497.87	3345.943	6.72	198.943	1166.71	5.86
Si(1,1,1)	Si(0,0,4)	21.38	422.42	3127.439	7.4	174.546	1155.44	6.62
Si(1,1,1)	Si(1,3,3)	23.4	368.87	1707.088	4.63	98.256	386.36	3.93
Si(1,1,1)	Si(2,2,4)	26.51	327.51	1849.619	5.65	98.318	494.28	5.03
Si(1,1,1)	Si(3,3,3)	28.26	303.53	1057.614	3.48	60.547	184.17	3.04
Si(1,1,1)	Si(0,4,4)	31.03	273.03	1223.591	4.48	66.407	263.64	3.97
Si(1,1,1)	Si(1,3,5)	32.62	254.81	712.726	2.8	40.509	95.29	2.35
Si(1,1,1)	Si(0,2,6)	35.19	233.99	862.55	3.69	47.578	152.82	3.21
Si(1,1,1)	Si(3,3,5)	36.69	220.32	507.598	2.3	30.135	52.17	1.73
Si(1,1,1)	Si(4,4,4)	39.15	202.41	632.633	3.13	34.764	95.9	2.76
Si(1,1,1)	Si(1,1,7)	40.6	191.78	373.291	1.95	46.653	35.86	0.77
Si(1,1,1)	Si(2,4,6)	42.99	177.32	478.269	2.7	26.811	58.77	2.19
Si(1,1,1)	Si(1,3,7)	44.42	167.76	283.319	1.69	82.071	24.33	0.3
Si(1,1,1)	Si(0,0,8)	46.8	154.89	369.725	2.39	21.319	37.91	1.78
Si(1,1,1)	Si(3,3,7)	48.23	146.22	218.766	1.5	70.934	18.53	0.26
Si(1,1,1)	Si(0,6,6)	50.64	135.26	290.961	2.15	17.789	27.8	1.56
Si(1,1,1)	Si(5,5,5)	52.1	127.55	171.752	1.35	61.422	13.03	0.21
Si(1,1,1)	Si(0,4,8)	54.59	116.99	232.838	1.99	28.589	20.9	0.73
Si(1,1,1)	Si(1,1,9)	56.11	109.7	137.192	1.25	52.895	9.7	0.18
Si(1,1,1)	Si(4,6,6)	58.73	99.99	187.995	1.88	49.303	16.44	0.33
Si(1,1,1)	Si(1,3,9)	60.37	92.86	110.025	1.18	44.706	7.55	0.17
Si(1,1,1)	Si(4,4,8)	63.22	83.08	154.102	1.85	41.016	13.09	0.32
Si(1,1,1)	Si(1,7,7)	65.04	76.38	90.094	1.18	36.657	5.93	0.16
Si(1,1,1)	Si(0,2,10)	68.32	65.64	128.104	1.95	16.324	10.88	0.67
Si(1,1,1)	Si(1,5,9)	70.48	58.24	73.986	1.27	28.174	4.66	0.17
Si(1,1,1)	Si(3,5,9)	77.73	35.94	61.355	1.71	4.779	4.41	0.92
Si(1,1,1)	Si(2,4,10)	86.52	12.14	90.365	7.44	5.99	29.09	4.86

Ei = 12.527 keV			Cryst 1-2			Cryst 1-2-3		
Cryst 1	Cryst 2,3	ΘB 2	ΔE	∫IR dΘdE	FOM	ΔE	∫IR dΘdE	FOM
		[°]	[meV]	[µrad meV]		[meV]	[µrad meV]	
Dia(1,1,1)	Si(1,1,1)	9.08	718.48	7610.067	10.59	714.792	6992.81	9.78
Dia(1,1,1)	Si(0,2,2)	14.93	471.2	4913.875	10.43	443.551	4354.86	9.82
Dia(1,1,1)	Si(1,1,3)	17.59	345.8	2506.021	7.25	202.182	1263.58	6.25
Dia(1,1,1)	Si(0,0,4)	21.38	307.4	2410.011	7.84	177.059	1247.09	7.04
Dia(1,1,1)	Si(1,3,3)	23.4	270.27	1342.955	4.97	97.272	414.97	4.27
Dia(1,1,1)	Si(2,2,4)	26.51	246.51	1487.531	6.03	100.151	521.3	5.21
Dia(1,1,1)	Si(3,3,3)	28.26	226.9	858.658	3.78	59.936	194.76	3.25
Dia(1,1,1)	Si(0,4,4)	31.03	209.48	1015.99	4.85	66.692	277.19	4.16
Dia(1,1,1)	Si(1,3,5)	32.62	199.07	594.314	2.99	41.157	100.89	2.45
Dia(1,1,1)	Si(0,2,6)	35.19	183.58	726.57	3.96	47.1	164.16	3.49
Dia(1,1,1)	Si(3,3,5)	36.69	176.85	432.779	2.45	30.224	56.21	1.86
Dia(1,1,1)	Si(4,4,4)	39.15	163.91	541.881	3.31	34.721	101.06	2.91
Dia(1,1,1)	Si(1,1,7)	40.6	156.87	321.976	2.05	46.821	36.49	0.78
Dia(1,1,1)	Si(2,4,6)	42.99	146.63	415.491	2.83	26.675	61.98	2.32
Dia(1,1,1)	Si(1,3,7)	44.42	139.61	246.147	1.76	81.858	25.38	0.31
Dia(1,1,1)	Si(0,0,8)	46.8	130.35	323.997	2.49	21.205	40.59	1.91
Dia(1,1,1)	Si(3,3,7)	48.23	125.77	191.737	1.52	70.976	19.03	0.27
Dia(1,1,1)	Si(0,6,6)	50.64	115.73	256.86	2.22	17.818	28.73	1.61
Dia(1,1,1)	Si(5,5,5)	52.1	112.61	151.479	1.35	61.363	14.86	0.24
Dia(1,1,1)	Si(0,4,8)	54.59	101.67	206.244	2.03	28.556	21.55	0.75
Dia(1,1,1)	Si(1,1,9)	56.11	99.53	121.777	1.22	94.287	10.63	0.11
Dia(1,1,1)	Si(4,6,6)	58.73	88.95	169.131	1.9	246.847	17.21	0.07
Dia(1,1,1)	Si(1,3,9)	60.37	85.85	99.606	1.16	79.799	7.96	0.1
Dia(1,1,1)	Si(4,4,8)	63.22	74.89	140.057	1.87	206.041	13.69	0.07
Dia(1,1,1)	Si(1,7,7)	65.04	70.98	81.925	1.15	65.619	6.43	0.1
Dia(1,1,1)	Si(0,2,10)	68.32	60.04	117.472	1.96	22.672	11.36	0.5
Dia(1,1,1)	Si(1,5,9)	70.48	54.62	68.146	1.25	50.828	5.55	0.11
Dia(1,1,1)	Si(3,5,9)	77.73	33.59	57.04	1.7	4.796	4.41	0.92
Dia(1,1,1)	Si(2,4,10)	86.52	10.93	84.963	7.77	5.971	31.15	5.22

Ei = 11.959 keV			Cryst 1-2			Cryst 1-2-3		
Cryst 1	Cryst 2,3	ΘB 2 [°]	ΔE [meV]	∫IR dΘdE [µrad meV]	FOM	ΔE [meV]	∫IR dΘdE [µrad meV]	FOM
Si(1,1,1)	Si(1,1,1)	9.52	1118.25	12227.826	10.93	1068.44	10671.45	9.99
Si(1,1,1)	Si(0,2,2)	15.66	658.48	6757.539	10.26	443.766	4091.68	9.22
Si(1,1,1)	Si(1,1,3)	18.46	469.34	3242.016	6.91	189.072	1128.53	5.97
Si(1,1,1)	Si(0,0,4)	22.44	396.71	3030.246	7.64	169.45	1138.56	6.72
Si(1,1,1)	Si(1,3,3)	24.58	343.87	1647.965	4.79	93.845	374.3	3.99
Si(1,1,1)	Si(2,2,4)	27.88	304.49	1790.923	5.88	96.076	483.33	5.03
Si(1,1,1)	Si(3,3,3)	29.73	278.84	1017.848	3.65	57.583	178.17	3.09
Si(1,1,1)	Si(0,4,4)	32.68	251.61	1182.7	4.7	63.87	255.54	4
Si(1,1,1)	Si(1,3,5)	34.38	232.94	685.714	2.94	39.062	95.03	2.43
Si(1,1,1)	Si(0,2,6)	37.13	212.86	831.561	3.91	45.217	150.99	3.34
Si(1,1,1)	Si(3,3,5)	38.75	198.94	486.515	2.45	28.366	51.68	1.82
Si(1,1,1)	Si(4,4,4)	41.4	182.22	608.822	3.34	33.49	93.84	2.8
Si(1,1,1)	Si(1,1,7)	42.97	170.92	357.435	2.09	22.59	33.52	1.48
Si(1,1,1)	Si(2,4,6)	45.58	157.56	460.234	2.92	25.368	60.65	2.39
Si(1,1,1)	Si(1,3,7)	47.15	148.49	269.336	1.81	72.939	22.47	0.31
Si(1,1,1)	Si(3,5,5)	47.15	148.49	269.336	1.81	72.939	22.47	0.31
Si(1,1,1)	Si(0,0,8)	49.78	135.59	354.91	2.62	20	39.58	1.98
Si(1,1,1)	Si(3,3,7)	51.38	126.69	208.686	1.65	62.3	16.76	0.27
Si(1,1,1)	Si(0,6,6)	54.09	116.14	279.407	2.41	16.257	28.06	1.73
Si(1,1,1)	Si(5,5,5)	55.75	108.33	163.6	1.51	52.7	12.71	0.24
Si(1,1,1)	Si(0,4,8)	58.62	97.1	222.584	2.29	13.299	21.03	1.58
Si(1,1,1)	Si(1,1,9)	60.41	90.32	129.93	1.44	43.896	8.91	0.2
Si(1,1,1)	Si(4,6,6)	63.56	79.69	180.492	2.26	10.939	16.63	1.52
Si(1,1,1)	Si(1,3,9)	65.57	72.36	104.487	1.44	35.255	7.54	0.21
Si(1,1,1)	Si(4,4,8)	69.26	60.88	147.778	2.43	9.044	14.57	1.61
Si(1,1,1)	Si(1,7,7)	71.75	52.64	84.828	1.61	12.833	5.67	0.44
Si(1,1,1)	Si(0,2,10)	76.75	38.51	122.203	3.17	7.654	16.53	2.16
Si(1,1,1)	Si(1,5,9)	80.86	26.26	69.759	2.66	4.99	7.75	1.55
Si(1,1,1)	Si(3,7,7)	80.86	26.26	69.759	2.66	4.99	7.75	1.55

Ei = 11.959 keV			Cryst 1-2			Cryst 1-2-3		
Cryst 1	Cryst 2,3	ΘB 2 [°]	ΔE [meV]	∫IR dΘdE [μrad meV]	FOM	ΔE [meV]	∫IR dΘdE [μrad meV]	FOM
Dia(1,1,1)	Si(1,1,1)	9.52	687.46	7431.556	10.81	685.819	6853.42	9.99
Dia(1,1,1)	Si(0,2,2)	15.66	453.38	4841.985	10.68	425.955	4257.31	9.99
Dia(1,1,1)	Si(1,1,3)	18.46	329.89	2466.963	7.48	191.817	1222.77	6.37
Dia(1,1,1)	Si(0,0,4)	22.44	292.97	2380.201	8.12	169.507	1218.2	7.19
Dia(1,1,1)	Si(1,3,3)	24.58	255.72	1322.296	5.17	92.868	403.14	4.34
Dia(1,1,1)	Si(2,2,4)	27.88	232.66	1470.994	6.32	95.923	520.38	5.43
Dia(1,1,1)	Si(3,3,3)	29.73	213.62	846.082	3.96	57.219	191.18	3.34
Dia(1,1,1)	Si(0,4,4)	32.68	196.21	1001.061	5.1	64.152	273.53	4.26
Dia(1,1,1)	Si(1,3,5)	34.38	185.94	583.388	3.14	38.913	101.47	2.61
Dia(1,1,1)	Si(0,2,6)	37.13	169.9	711.942	4.19	44.762	161.79	3.61
Dia(1,1,1)	Si(3,3,5)	38.75	163.82	423.455	2.58	28.567	56.5	1.98
Dia(1,1,1)	Si(4,4,4)	41.4	150.55	530.23	3.52	33.174	99.99	3.01
Dia(1,1,1)	Si(1,1,7)	42.97	143.79	313.814	2.18	22.597	35.06	1.55
Dia(1,1,1)	Si(2,4,6)	45.58	133.67	406.925	3.04	25.186	65.67	2.61
Dia(1,1,1)	Si(1,3,7)	47.15	127.31	238.76	1.88	73.323	24.42	0.33
Dia(1,1,1)	Si(3,5,5)	47.15	127.31	238.759	1.88	73.323	24.42	0.33
Dia(1,1,1)	Si(0,0,8)	49.78	117.15	317.091	2.71	20.285	43.59	2.15
Dia(1,1,1)	Si(3,3,7)	51.38	113.4	186.38	1.64	62.223	17.52	0.28
Dia(1,1,1)	Si(0,6,6)	54.09	102.01	251.542	2.47	16.141	29.87	1.85
Dia(1,1,1)	Si(5,5,5)	55.75	99.05	147.82	1.49	52.693	13.42	0.25
Dia(1,1,1)	Si(0,4,8)	58.62	87.67	203.035	2.32	13.306	22.73	1.71
Dia(1,1,1)	Si(1,1,9)	60.41	83.94	118.905	1.42	218.212	10.49	0.05
Dia(1,1,1)	Si(4,6,6)	63.56	72.67	166.337	2.29	200.521	18.07	0.09
Dia(1,1,1)	Si(1,3,9)	65.57	68.09	96.859	1.42	63.372	7.88	0.12
Dia(1,1,1)	Si(4,4,8)	69.26	55.97	137.485	2.46	9.02	15.79	1.75
Dia(1,1,1)	Si(1,7,7)	71.75	49.92	79.627	1.6	109.614	6.21	0.06
Dia(1,1,1)	Si(0,2,10)	76.75	35.13	115.075	3.28	7.696	17.61	2.29
Dia(1,1,1)	Si(1,5,9)	80.86	24.06	66.003	2.74	4.925	8.48	1.72
Dia(1,1,1)	Si(3,7,7)	80.86	24.06	66.003	2.74	4.925	8.48	1.72

Ei = 10.535 keV			Cryst 1-2			Cryst 1-2-3		
Cryst 1	Cryst 2,3	ΘB 2 [°]	ΔE [meV]	∫IR dΘdE [µrad meV]	FOM	ΔE [meV]	∫IR dΘdE [µrad meV]	FOM
Si(1,1,1)	Si(1,1,1)	10.82	1000.15	11207.187	11.21	950.054	9560.39	10.06
Si(1,1,1)	Si(0,2,2)	17.85	580.75	6221.389	10.71	399.45	3747.95	9.38
Si(1,1,1)	Si(1,1,3)	21.06	396.94	2936.981	7.4	170.31	1036.8	6.09
Si(1,1,1)	Si(0,0,4)	25.68	333.71	2771.061	8.3	153.161	1063.24	6.94
Si(1,1,1)	Si(1,3,3)	28.18	280.45	1481.736	5.28	81.991	343.03	4.18
Si(1,1,1)	Si(2,2,4)	32.06	247.16	1623.511	6.57	84.272	464.86	5.52
Si(1,1,1)	Si(3,3,3)	34.26	220.14	907.489	4.12	51.422	164.35	3.2
Si(1,1,1)	Si(0,4,4)	37.8	197.22	1065.953	5.4	56.067	241.8	4.31
Si(1,1,1)	Si(1,3,5)	39.87	178.14	606.895	3.41	34.477	90.83	2.63
Si(1,1,1)	Si(0,2,6)	43.26	161.04	746.174	4.63	39.683	146.79	3.7
Si(1,1,1)	Si(3,3,5)	45.27	146.81	427.998	2.92	24.85	52.7	2.12
Si(1,1,1)	Si(4,4,4)	48.65	132.78	543.895	4.1	30.117	93.25	3.1
Si(1,1,1)	Si(1,1,7)	50.69	120.77	312.625	2.59	18.741	32.9	1.76
Si(1,1,1)	Si(2,4,6)	54.17	108.5	408.646	3.77	22.962	64.7	2.82
Si(1,1,1)	Si(1,3,7)	56.33	98.41	234.597	2.38	14.472	22.24	1.54
Si(1,1,1)	Si(0,0,8)	60.09	86.36	314.017	3.64	18.019	47.87	2.66
Si(1,1,1)	Si(3,3,7)	62.48	77.02	179.565	2.33	11.375	16.94	1.49
Si(1,1,1)	Si(0,6,6)	66.83	64.76	246.11	3.8	14.642	37.82	2.58
Si(1,1,1)	Si(5,5,5)	69.77	54.72	140.01	2.56	9.168	14.14	1.54
Si(1,1,1)	Si(0,4,8)	75.72	39.78	195.672	4.92	11.425	37.46	3.28
Si(1,1,1)	Si(1,1,9)	80.79	25.43	110.62	4.35	7.473	18.68	2.5
Dia(1,1,1)	Si(1,1,1)	10.82	609.3	6947.761	11.4	2905.99	6184.38	2.13
Dia(1,1,1)	Si(0,2,2)	17.85	408.22	4614.56	11.3	387.236	3991.23	10.31
Dia(1,1,1)	Si(1,1,3)	21.06	290.8	2339.543	8.05	168.703	1125.71	6.67
Dia(1,1,1)	Si(0,0,4)	25.68	256.58	2300.633	8.97	151.081	1162.19	7.69
Dia(1,1,1)	Si(1,3,3)	28.18	218.21	1263.094	5.79	81.799	375.96	4.6
Dia(1,1,1)	Si(2,2,4)	32.06	197.34	1407.02	7.13	84.96	511.07	6.02
Dia(1,1,1)	Si(3,3,3)	34.26	178.99	796.903	4.45	51.387	179.2	3.49
Dia(1,1,1)	Si(0,4,4)	37.8	161.54	948.732	5.87	55.591	265.6	4.78
Dia(1,1,1)	Si(1,3,5)	39.87	152.21	546.932	3.59	34.482	99.79	2.89
Dia(1,1,1)	Si(0,2,6)	43.26	137.56	679.169	4.94	40.234	159.58	3.97
Dia(1,1,1)	Si(3,3,5)	45.27	129.17	392.863	3.04	25.023	57.43	2.3
Dia(1,1,1)	Si(4,4,4)	48.65	116.69	505.183	4.33	30.106	102.86	3.42
Dia(1,1,1)	Si(1,1,7)	50.69	109.62	291.885	2.66	18.646	36.21	1.94
Dia(1,1,1)	Si(2,4,6)	54.17	97.46	385.128	3.95	22.862	70.47	3.08
Dia(1,1,1)	Si(1,3,7)	56.33	91.67	222.714	2.43	14.494	24.8	1.71
Dia(1,1,1)	Si(0,0,8)	60.09	78.96	299.218	3.79	18.069	51.89	2.87
Dia(1,1,1)	Si(3,3,7)	62.48	73.22	172.508	2.36	11.423	18.26	1.6
Dia(1,1,1)	Si(0,6,6)	66.83	59.94	237.807	3.97	14.63	41.25	2.82
Dia(1,1,1)	Si(5,5,5)	69.77	52.48	136.004	2.59	9.177	15.49	1.69
Dia(1,1,1)	Si(0,4,8)	75.72	36.65	192.078	5.24	11.414	41.04	3.6
Dia(1,1,1)	Si(1,1,9)	80.79	23.64	109.784	4.64	7.433	20.51	2.76

Ei = 12.968 keV			Cryst 1-2			Cryst 1-2-3		
Cryst 1	Cryst 2,3	ΘB 2 [°]	ΔE [meV]	∫IR dΘdE [μrad meV]	FOM	ΔE [meV]	∫IR dΘdE [μrad meV]	FOM
Si(1,1,1)	Si(1,1,1)	8.77	1198.85	12824.516	10.7	1136.16	11369.18	10.01
Si(1,1,1)	Si(0,2,2)	14.42	713.07	7063.394	9.91	475.422	4278.13	9
Si(1,1,1)	Si(1,1,3)	16.97	520.38	3421.266	6.57	203.911	1183.46	5.8
Si(1,1,1)	Si(0,0,4)	20.61	441.92	3197.964	7.24	178.876	1163.66	6.51
Si(1,1,1)	Si(1,3,3)	22.56	388.37	1748.117	4.5	101.851	396.16	3.89
Si(1,1,1)	Si(2,2,4)	25.54	345.69	1891.214	5.47	101.778	497.21	4.89
Si(1,1,1)	Si(1,1,5)	27.22	321.43	1084.523	3.37	62.29	186.99	3
Si(1,1,1)	Si(0,4,4)	29.86	289.97	1253.995	4.32	68.354	267.44	3.91
Si(1,1,1)	Si(1,3,5)	31.38	272.69	733.225	2.69	42.198	96.56	2.29
Si(1,1,1)	Si(0,2,6)	33.83	250.75	885.252	3.53	48.531	153.09	3.15
Si(1,1,1)	Si(3,3,5)	35.25	236.86	523.092	2.21	31.872	53.54	1.68
Si(1,1,1)	Si(4,4,4)	37.58	218.29	650.418	2.98	35.686	92.99	2.61
Si(1,1,1)	Si(1,1,7)	38.95	207.87	386.21	1.86	102.678	36.84	0.36
Si(1,1,1)	Si(2,4,6)	41.2	192.38	492.317	2.56	27.719	56.94	2.05
Si(1,1,1)	Si(1,3,7)	42.54	182.65	292.312	1.6	89.021	25.76	0.29
Si(1,1,1)	Si(3,5,5)	42.54	182.65	292.314	1.6	89.021	25.76	0.29
Si(1,1,1)	Si(0,0,8)	44.76	170.17	380.824	2.24	22.574	38.21	1.69
Si(1,1,1)	Si(3,3,7)	46.09	161.21	226.64	1.41	77.8	17.59	0.23
Si(1,1,1)	Si(0,6,6)	48.32	149.85	299.357	2	73.974	28.71	0.39
Si(1,1,1)	Si(1,5,7)	49.67	142.01	178.194	1.25	68.309	14.16	0.21
Si(1,1,1)	Si(5,5,5)	49.67	142.01	178.193	1.25	68.309	14.16	0.21
Si(1,1,1)	Si(0,4,8)	51.93	131.73	239.974	1.82	64.782	20.79	0.32
Si(1,1,1)	Si(1,1,9)	53.31	124.84	142.418	1.14	59.552	10.62	0.18
Si(1,1,1)	Si(4,6,6)	55.66	115.05	195.063	1.7	56.245	16.63	0.3
Si(1,1,1)	Si(1,3,9)	57.1	109.21	114.906	1.05	51.521	8.34	0.16
Si(1,1,1)	Si(4,4,8)	59.59	98.74	160.119	1.62	48.125	13.29	0.28
Si(1,1,1)	Si(1,7,7)	61.14	92.27	93.812	1.02	43.857	6.85	0.16
Si(1,1,1)	Si(0,2,10)	63.85	82.44	132.524	1.61	40.316	10.67	0.26
Si(1,1,1)	Si(1,5,9)	65.57	76.17	77.226	1.01	36.215	5.38	0.15
Si(1,1,1)	Si(3,5,9)	70.72	58.8	64.186	1.09	28.126	4.05	0.14
Si(1,1,1)	Si(2,4,10)	74.63	46.57	93.394	2.01	6.245	7.8	1.25
Si(1,1,1)	Si(1,1,11)	77.47	37.47	53.665	1.43	18.403	3.43	0.19
Si(1,1,1)	Si(0,8,8)	84.77	16.44	79.393	4.83	5.12	16.17	3.16
Dia(1,1,1)	Si(1,1,1)	8.77	742.43	7738.503	10.42	741.366	7165.09	9.66
Dia(1,1,1)	Si(0,2,2)	14.42	484.65	4956.181	10.23	460.729	4387.88	9.52
Dia(1,1,1)	Si(1,1,3)	16.97	358.34	2538.916	7.09	202.766	1253.17	6.18
Dia(1,1,1)	Si(0,0,4)	20.61	318.18	2436.309	7.66	179.714	1246.14	6.93
Dia(1,1,1)	Si(1,3,3)	22.56	281.37	1361.306	4.84	100.976	424.46	4.2
Dia(1,1,1)	Si(2,2,4)	25.54	257.26	1499.146	5.83	103.586	525.67	5.07
Dia(1,1,1)	Si(1,1,5)	27.22	237.77	869.89	3.66	62.315	198.23	3.18
Dia(1,1,1)	Si(0,4,4)	29.86	219.61	1026.233	4.67	69.067	280.32	4.06
Dia(1,1,1)	Si(1,3,5)	31.38	208.72	601.256	2.88	42.502	105.57	2.48
Dia(1,1,1)	Si(0,2,6)	33.83	193.16	734.595	3.8	48.857	163.88	3.35
Dia(1,1,1)	Si(3,3,5)	35.25	186.62	438.893	2.35	31.823	56.8	1.79
Dia(1,1,1)	Si(4,4,4)	37.58	173.16	548.081	3.17	35.77	101.21	2.83
Dia(1,1,1)	Si(1,1,7)	38.95	166.54	329.141	1.98	102.875	38.07	0.37
Dia(1,1,1)	Si(2,4,6)	41.2	156.27	421.4	2.7	27.884	61.89	2.22
Dia(1,1,1)	Si(1,3,7)	42.54	148.84	251.088	1.69	88.855	26.57	0.3
Dia(1,1,1)	Si(3,5,5)	42.54	148.84	251.086	1.69	88.855	26.57	0.3
Dia(1,1,1)	Si(0,0,8)	44.76	140.25	329.64	2.35	22.605	40.76	1.8
Dia(1,1,1)	Si(3,3,7)	46.09	134.57	195.658	1.45	77.702	20.54	0.26

**Dia(1,1,1)**	**Si(0,6,6)**	**48.32**	**125.3**	**261.005**	**2.08**	**36.465**	**29.49**	**0.81**
**Dia(1,1,1)**	**Si(1,5,7)**	**49.67**	**122.33**	**155.542**	**1.27**	**285.243**	**14.88**	**0.05**
**Dia(1,1,1)**	**Si(5,5,5)**	**49.67**	**122.33**	**155.543**	**1.27**	**285.242**	**14.88**	**0.05**
**Dia(1,1,1)**	**Si(0,4,8)**	**51.93**	**112.35**	**210.443**	**1.87**	**64.827**	**22.85**	**0.35**
**Dia(1,1,1)**	**Si(1,1,9)**	**53.31**	**109.83**	**124.946**	**1.14**	**106.135**	**11**	**0.1**
**Dia(1,1,1)**	**Si(4,6,6)**	**55.66**	**99.47**	**171.235**	**1.72**	**280.321**	**17.38**	**0.06**
**Dia(1,1,1)**	**Si(1,3,9)**	**57.1**	**97.64**	**101.729**	**1.04**	**91.905**	**8.53**	**0.09**
**Dia(1,1,1)**	**Si(4,4,8)**	**59.59**	**87.68**	**142.005**	**1.62**	**86.902**	**13.73**	**0.16**
**Dia(1,1,1)**	**Si(1,7,7)**	**61.14**	**84.45**	**83.853**	**0.99**	**78.274**	**6.72**	**0.09**
**Dia(1,1,1)**	**Si(0,2,10)**	**63.85**	**74.54**	**118.884**	**1.59**	**72.996**	**11.08**	**0.15**
**Dia(1,1,1)**	**Si(1,5,9)**	**65.57**	**70.14**	**69.257**	**0.99**	**180.167**	**5.34**	**0.03**
**Dia(1,1,1)**	**Si(3,5,9)**	**70.72**	**54.59**	**58.259**	**1.07**	**50.445**	**4.34**	**0.09**
**Dia(1,1,1)**	**Si(2,4,10)**	**74.63**	**42.4**	**85.78**	**2.02**	**6.24**	**8.42**	**1.35**
**Dia(1,1,1)**	**Si(1,1,11)**	**77.47**	**34.85**	**49.307**	**1.41**	**18.438**	**3.65**	**0.2**
**Dia(1,1,1)**	**Si(0,8,8)**	**84.77**	**14.38**	**73.713**	**5.13**	**4.984**	**17.52**	**3.52**

Ei = 12.385 keV			Cryst 1-2			Cryst 1-2-3		
Cryst 1	Cryst 2,3	ΘB 2 [°]	ΔE [meV]	∫IR dΘdE [µrad meV]	FOM	ΔE [meV]	∫IR dΘdE [µrad meV]	FOM
Si(1,1,1)	Si(1,1,1)	9.19	1153.08	12490.876	10.83	1102.78	10972.07	9.95
Si(1,1,1)	Si(0,2,2)	15.11	681.96	6881.508	10.09	456.561	4165.39	9.12
Si(1,1,1)	Si(1,1,3)	17.8	491.13	3321.585	6.76	196.975	1160.72	5.89
Si(1,1,1)	Si(0,0,4)	21.63	415.27	3102.641	7.47	173.705	1155	6.65
Si(1,1,1)	Si(1,3,3)	23.69	362.23	1691.771	4.67	97.153	383.39	3.95
Si(1,1,1)	Si(2,2,4)	26.84	321.7	1832.583	5.7	98.002	489.58	5
Si(1,1,1)	Si(1,1,5)	28.61	298.16	1047.749	3.51	59.834	182.8	3.06
Si(1,1,1)	Si(3,3,3)	28.61	298.16	1047.749	3.51	59.834	182.8	3.06
Si(1,1,1)	Si(0,4,4)	31.42	267.73	1213.855	4.53	65.546	261.83	3.99
Si(1,1,1)	Si(1,3,5)	33.04	249.75	706.629	2.83	40.048	95.04	2.37
Si(1,1,1)	Si(0,2,6)	35.65	228.7	855.178	3.74	47.083	152.31	3.23
Si(1,1,1)	Si(3,3,5)	37.18	215.07	502.463	2.34	29.62	51.85	1.75
Si(1,1,1)	Si(4,4,4)	39.68	199.45	624.22	3.13	34.397	96.66	2.81
Si(1,1,1)	Si(1,1,7)	41.16	186.91	369.217	1.98	45.75	34.21	0.75
Si(1,1,1)	Si(2,4,6)	43.61	172.04	474	2.76	26.534	59.99	2.26
Si(1,1,1)	Si(1,3,7)	45.07	162.65	279.791	1.72	79.869	23.86	0.3
Si(1,1,1)	Si(0,0,8)	47.5	149.95	366.248	2.44	21.06	38.45	1.83
Si(1,1,1)	Si(3,3,7)	48.97	142.05	216.17	1.52	68.754	17.86	0.26
Si(1,1,1)	Si(0,6,6)	51.45	130.36	288.229	2.21	17.303	27.66	1.6
Si(1,1,1)	Si(1,5,7)	52.95	122.41	169.729	1.39	59.237	13.01	0.22
Si(1,1,1)	Si(0,4,8)	55.52	112.11	230.515	2.06	27.76	21.09	0.76
Si(1,1,1)	Si(1,1,9)	57.1	105.37	135.345	1.28	50.644	9.57	0.19
Si(1,1,1)	Si(4,6,6)	59.83	95.15	186.666	1.96	23.237	16.69	0.72
Si(1,1,1)	Si(1,3,9)	61.54	87.56	108.903	1.24	42.436	7.4	0.17
Si(1,1,1)	Si(4,4,8)	64.56	77.7	153.004	1.97	19.265	12.76	0.66
Si(1,1,1)	Si(1,7,7)	66.49	70.83	88.819	1.25	34.17	5.95	0.17
Si(1,1,1)	Si(0,2,10)	70.03	59.63	126.634	2.12	8.07	11	1.36
Si(1,1,1)	Si(1,5,9)	72.43	51.71	72.923	1.41	25.221	5.15	0.2
Si(1,1,1)	Si(3,5,9)	81.24	25.58	60.517	2.37	4.415	6	1.36
Dia(1,1,1)	Si(1,1,1)	9.19	708.49	7548.263	10.65	707.786	6946.76	9.81
Dia(1,1,1)	Si(0,2,2)	15.11	466.7	4897.879	10.49	440.835	4333.91	9.83
Dia(1,1,1)	Si(1,1,3)	17.8	341.86	2497.992	7.31	199.619	1254.47	6.28
Dia(1,1,1)	Si(0,0,4)	21.63	303.84	2404.492	7.91	175.164	1240.45	7.08
Dia(1,1,1)	Si(1,3,3)	23.69	266.77	1339.104	5.02	96.116	411.93	4.29
Dia(1,1,1)	Si(2,2,4)	26.84	243.03	1484.419	6.11	99.126	520.6	5.25
Dia(1,1,1)	Si(1,1,5)	28.61	224.11	856.705	3.82	59.257	193.89	3.27
Dia(1,1,1)	Si(3,3,3)	28.61	224.11	856.706	3.82	59.257	193.89	3.27
Dia(1,1,1)	Si(0,4,4)	31.42	205.96	1012.722	4.92	66.335	275.53	4.15
Dia(1,1,1)	Si(1,3,5)	33.04	195.8	591.662	3.02	40.563	100.62	2.48
Dia(1,1,1)	Si(0,2,6)	35.65	180.57	723.175	4	46.401	163.52	3.52
Dia(1,1,1)	Si(3,3,5)	37.18	173.69	430.432	2.48	29.783	56.23	1.89
Dia(1,1,1)	Si(4,4,4)	39.68	160.59	539.796	3.36	34.348	101.13	2.94
Dia(1,1,1)	Si(1,1,7)	41.16	153.33	320.564	2.09	45.865	35.44	0.77
Dia(1,1,1)	Si(2,4,6)	43.61	143.57	414.208	2.89	26.315	64.21	2.44
Dia(1,1,1)	Si(1,3,7)	45.07	136.55	244.258	1.79	79.683	25.09	0.31
Dia(1,1,1)	Si(0,0,8)	47.5	127.17	322.532	2.54	20.814	40.74	1.96
Dia(1,1,1)	Si(3,3,7)	48.97	122.47	190.116	1.55	68.803	18.64	0.27
Dia(1,1,1)	Si(0,6,6)	51.45	112.42	255.393	2.27	17.339	29.65	1.71
Dia(1,1,1)	Si(1,5,7)	52.95	109.77	150.954	1.38	59.195	14.87	0.25
Dia(1,1,1)	Si(0,4,8)	55.52	98.23	205.723	2.09	27.823	21.96	0.79
Dia(1,1,1)	Si(1,1,9)	57.1	95.8	121.274	1.27	90.402	10.52	0.12

Dia(1,1,1)	Si(4,6,6)	59.83	85.24	168.303	1.97	235.788	16.71	0.07
Dia(1,1,1)	Si(1,3,9)	61.54	81.44	98.932	1.21	75.848	7.91	0.1
Dia(1,1,1)	Si(4,4,8)	64.56	70.76	139.653	1.97	194.388	13.54	0.07
Dia(1,1,1)	Si(1,7,7)	66.49	66.23	81.404	1.23	61.338	6.6	0.11
Dia(1,1,1)	Si(0,2,10)	70.03	54.4	116.553	2.14	149.913	11.96	0.08
Dia(1,1,1)	Si(1,5,9)	72.43	48.77	67.684	1.39	45.717	5.18	0.11
Dia(1,1,1)	Si(3,5,9)	81.24	23.49	56.623	2.41	4.357	6.55	1.5

Ei = 10.871 keV			Cryst 1-2			Cryst 1-2-3		
Cryst 1	Cryst 2,3	ΘB 2	ΔE	∫IR dΘdE	FOM	ΔE	∫IR dΘdE	FOM
		[°]	[meV]	[µrad meV]		[meV]	[µrad meV]	
Si(1,1,1)	Si(1,1,1)	10.48	1028.23	11470.143	11.16	977.374	9826.51	10.05
Si(1,1,1)	Si(0,2,2)	17.28	599.09	6369.162	10.63	409.522	3832.81	9.36
Si(1,1,1)	Si(1,1,3)	20.38	413.92	3013.955	7.28	175.536	1063.8	6.06
Si(1,1,1)	Si(0,0,4)	24.83	348.39	2838.748	8.15	156.996	1079.12	6.87
Si(1,1,1)	Si(1,3,3)	27.24	295.28	1523.87	5.16	84.816	350.37	4.13
Si(1,1,1)	Si(2,2,4)	30.96	260.6	1666.274	6.39	87.368	473.72	5.42
Si(1,1,1)	Si(3,3,3)	33.07	234.1	934.277	3.99	53.137	168.48	3.17
Si(1,1,1)	Si(0,4,4)	36.44	210.07	1095.957	5.22	58.081	244	4.2
Si(1,1,1)	Si(1,3,5)	38.4	190.95	626.363	3.28	35.561	90.64	2.55
Si(1,1,1)	Si(0,2,6)	41.61	173.05	768.712	4.44	40.976	148.64	3.63
Si(1,1,1)	Si(3,3,5)	43.51	158.96	442.754	2.79	25.636	51.93	2.03
Si(1,1,1)	Si(4,4,4)	46.67	144.57	560.828	3.88	30.916	94.17	3.05
Si(1,1,1)	Si(1,1,7)	48.58	132.7	323.821	2.44	19.321	32.39	1.68
Si(1,1,1)	Si(1,5,5)	48.58	132.7	323.821	2.44	19.321	32.39	1.68
Si(1,1,1)	Si(2,4,6)	51.79	120.02	421.681	3.51	23.787	61.82	2.6
Si(1,1,1)	Si(1,3,7)	53.76	110.13	243.482	2.21	15.184	22.2	1.46
Si(1,1,1)	Si(3,5,5)	53.76	110.13	243.481	2.21	15.184	22.21	1.46
Si(1,1,1)	Si(0,0,8)	57.14	98.47	324.446	3.29	18.58	45.06	2.43
Si(1,1,1)	Si(3,3,7)	59.26	89.3	186.731	2.09	12.173	15.21	1.25
Si(1,1,1)	Si(0,6,6)	62.99	77.81	254.339	3.27	14.763	34.92	2.37
Si(1,1,1)	Si(5,5,5)	65.41	68.98	145.621	2.11	9.615	12.4	1.29
Si(1,1,1)	Si(0,4,8)	69.91	56.36	202.611	3.6	12.215	29.83	2.44
Si(1,1,1)	Si(1,1,9)	73.06	46.29	115.434	2.49	7.735	11.38	1.47
Si(1,1,1)	Si(4,6,6)	80.06	28.74	163.514	5.69	9.711	37.29	3.84
Dia(1,1,1)	Si(1,1,1)	10.48	627.67	7067.796	11.26	3527.85	6374.44	1.81
Dia(1,1,1)	Si(0,2,2)	17.28	419.8	4678.25	11.14	397.776	4052.96	10.19
Dia(1,1,1)	Si(1,1,3)	20.38	300.06	2372.445	7.91	174.387	1151.17	6.6
Dia(1,1,1)	Si(0,0,4)	24.83	264.96	2324.432	8.77	154.184	1172.35	7.6
Dia(1,1,1)	Si(1,3,3)	27.24	227.2	1279.196	5.63	84.414	381.1	4.51
Dia(1,1,1)	Si(2,2,4)	30.96	205.76	1425.726	6.93	87.405	514.03	5.88
Dia(1,1,1)	Si(3,3,3)	33.07	187.52	810.409	4.32	52.959	182.7	3.45
Dia(1,1,1)	Si(0,4,4)	36.44	170.61	964.624	5.65	57.544	268.39	4.66
Dia(1,1,1)	Si(1,3,5)	38.4	160.01	555.082	3.47	35.582	100.9	2.84
Dia(1,1,1)	Si(0,2,6)	41.61	145.92	686.811	4.71	41.253	159.44	3.86
Dia(1,1,1)	Si(3,3,5)	43.51	137.8	399.866	2.9	25.768	57.79	2.24
Dia(1,1,1)	Si(4,4,4)	46.67	125.36	510.117	4.07	30.603	101.68	3.32
Dia(1,1,1)	Si(1,1,7)	48.58	117.57	296.489	2.52	19.364	35.66	1.84
Dia(1,1,1)	Si(1,5,5)	48.58	117.57	296.49	2.52	19.364	35.66	1.84
Dia(1,1,1)	Si(2,4,6)	51.79	106.68	391.358	3.67	23.442	69.35	2.96
Dia(1,1,1)	Si(3,5,5)	53.76	101.34	226.769	2.24	15.175	23.44	1.54
Dia(1,1,1)	Si(1,3,7)	53.76	101.33	226.768	2.24	15.175	23.44	1.54
Dia(1,1,1)	Si(0,0,8)	57.14	88.97	304.714	3.42	18.229	49.61	2.72
Dia(1,1,1)	Si(3,3,7)	59.26	83.66	176.252	2.11	12.175	16.55	1.36
Dia(1,1,1)	Si(0,6,6)	62.99	71.48	241.374	3.38	14.679	38.17	2.6
Dia(1,1,1)	Si(5,5,5)	65.41	65.8	139.369	2.12	9.697	12.63	1.3
Dia(1,1,1)	Si(0,4,8)	69.91	52.1	194.668	3.74	12.245	32.43	2.65
Dia(1,1,1)	Si(1,1,9)	73.06	44.15	111.499	2.53	7.729	12.46	1.61
Dia(1,1,1)	Si(4,6,6)	80.06	26.13	160.072	6.13	9.619	40.56	4.22

Ei = 13.419 keV			Cryst 1-2			Cryst 1-2-3		
Cryst 1	Cryst 2,3	ΘB 2 [°]	ΔE [meV]	∫IR dΘdE [μrad meV]	FOM	ΔE [meV]	∫IR dΘdE [μrad meV]	FOM
Si(1,1,1)	Si(1,1,1)	8.47	1233.03	13070.106	10.6	1173.88	11672.51	9.94
Si(1,1,1)	Si(0,2,2)	13.92	736.36	7201.116	9.78	488.384	4348.06	8.9
Si(1,1,1)	Si(1,1,3)	16.39	543.33	3494.387	6.43	210.422	1202.74	5.72
Si(1,1,1)	Si(0,0,4)	19.89	462.31	3262.298	7.06	186.547	1183.68	6.35
Si(1,1,1)	Si(1,3,3)	21.76	407.95	1790.272	4.39	104.335	403.28	3.87
Si(1,1,1)	Si(2,2,4)	24.63	363.71	1931.918	5.31	106.263	503.26	4.74
Si(1,1,1)	Si(3,3,3)	26.23	340.95	1111.529	3.26	63.806	188.69	2.96
Si(1,1,1)	Si(0,4,4)	28.76	306.75	1279.91	4.17	70.19	273.46	3.9
Si(1,1,1)	Si(1,3,5)	30.21	290.32	752.506	2.59	43.777	96.97	2.22
Si(1,1,1)	Si(0,2,6)	32.55	267.47	906.16	3.39	50.002	154.41	3.09
Si(1,1,1)	Si(3,3,5)	33.9	252.93	537.497	2.13	33.74	55.11	1.63
Si(1,1,1)	Si(4,4,4)	36.11	233.77	666.059	2.85	37.06	93.04	2.51
Si(1,1,1)	Si(1,1,7)	37.41	224.12	398.078	1.78	110.285	37.38	0.34
Si(1,1,1)	Si(2,4,6)	39.53	207.77	505.805	2.43	28.779	55.72	1.94
Si(1,1,1)	Si(1,3,7)	40.8	198.84	301.902	1.52	96.321	27.1	0.28
Si(1,1,1)	Si(0,0,8)	42.88	184.93	391.085	2.11	45.883	39.76	0.87
Si(1,1,1)	Si(3,3,7)	44.13	176.86	234.207	1.32	84.88	19.51	0.23
Si(1,1,1)	Si(0,6,6)	46.2	167.37	307.014	1.83	81.361	30.43	0.37
Si(1,1,1)	Si(5,5,5)	47.45	157.05	184.741	1.18	75.12	14.9	0.2
Si(1,1,1)	Si(0,4,8)	49.54	146.92	247.416	1.68	71.518	22.32	0.31
Si(1,1,1)	Si(1,1,9)	50.8	138.52	147.378	1.06	66.319	11.33	0.17
Si(1,1,1)	Si(4,6,6)	52.93	130.01	201.205	1.55	62.804	17.6	0.28
Si(1,1,1)	Si(1,3,9)	54.24	123.28	119.328	0.97	58.517	9.77	0.17
Si(1,1,1)	Si(4,4,8)	56.45	114.14	165.157	1.45	54.891	14.19	0.26
Si(1,1,1)	Si(1,7,7)	57.82	107.77	97.398	0.9	51.092	7.5	0.15
Si(1,1,1)	Si(5,5,7)	57.82	107.77	97.399	0.9	51.092	7.5	0.15
Si(1,1,1)	Si(0,2,10)	60.17	98.44	136.925	1.39	47.466	10.61	0.22
Si(1,1,1)	Si(1,5,9)	61.63	92.59	80.278	0.87	43.809	5.85	0.13
Si(1,1,1)	Si(3,5,9)	65.81	76.86	66.787	0.87	36.42	4.73	0.13
Si(1,1,1)	Si(2,4,10)	68.72	66.92	96.669	1.44	32.461	7.56	0.23
Si(1,1,1)	Si(1,1,11)	70.63	60.87	56.015	0.92	28.623	3.87	0.14
Si(1,1,1)	Si(0,8,8)	74.23	48.76	82.021	1.68	23.983	6.21	0.26
Si(1,1,1)	Si(1,3,11)	76.8	40.42	47.267	1.17	19.515	2.92	0.15
Si(1,1,1)	Si(0,6,10)	82.74	22.48	70.419	3.13	4.722	9.73	2.06

Ei = 13.419 keV			Cryst 1-2			Cryst 1-2-3		
Cryst 1	Cryst 2,3	ΘB 2 [°]	ΔE [meV]	∫IR dΘdE [μrad meV]	FOM	ΔE [meV]	∫IR dΘdE [μrad meV]	FOM
Dia(1,1,1)	Si(1,1,1)	8.47	766.58	7870.618	10.27	769.077	7328.81	9.53
Dia(1,1,1)	Si(0,2,2)	13.92	499.61	5013.143	10.03	475.64	4471.62	9.4
Dia(1,1,1)	Si(1,1,3)	16.39	370.98	2564.67	6.91	209.557	1269.77	6.06
Dia(1,1,1)	Si(0,0,4)	19.89	329.41	2461.353	7.47	185.053	1259.88	6.81
Dia(1,1,1)	Si(1,3,3)	21.76	292.52	1378.551	4.71	104.338	432.62	4.15
Dia(1,1,1)	Si(2,2,4)	24.63	268.05	1511.014	5.64	103.969	539.35	5.19
Dia(1,1,1)	Si(3,3,3)	26.23	248.51	881.465	3.55	64.982	202.25	3.11
Dia(1,1,1)	Si(0,4,4)	28.76	230.04	1033.204	4.49	71.744	284.94	3.97
Dia(1,1,1)	Si(1,3,5)	30.21	218.97	607.817	2.78	43.77	104.11	2.38
Dia(1,1,1)	Si(0,2,6)	32.55	202.59	737.269	3.64	50.747	165.03	3.25
Dia(1,1,1)	Si(3,3,5)	33.9	196.43	443.997	2.26	33.731	59.18	1.75
Dia(1,1,1)	Si(4,4,4)	36.11	181.61	548.365	3.02	36.82	98.94	2.69
Dia(1,1,1)	Si(1,1,7)	37.41	176.68	334.32	1.89	110.56	39.73	0.36
Dia(1,1,1)	Si(2,4,6)	39.53	165.83	426.844	2.57	29.051	60.41	2.08
Dia(1,1,1)	Si(1,3,7)	40.8	158.35	255.432	1.61	96.24	28.02	0.29
Dia(1,1,1)	Si(0,0,8)	42.88	150.04	334.757	2.23	45.741	42.04	0.92
Dia(1,1,1)	Si(3,3,7)	44.13	143.43	199.481	1.39	84.675	22.64	0.27
Dia(1,1,1)	Si(0,6,6)	46.2	135.11	265.606	1.97	81.172	30.72	0.38
Dia(1,1,1)	Si(5,5,5)	47.45	130.03	158.423	1.22	133.74	15.91	0.12
Dia(1,1,1)	Si(0,4,8)	49.54	121.93	213.838	1.75	356.437	23.13	0.06
Dia(1,1,1)	Si(1,1,9)	50.8	119.37	127.859	1.07	118.179	12.15	0.1
Dia(1,1,1)	Si(4,6,6)	52.93	110.13	174.763	1.59	113.135	18.12	0.16
Dia(1,1,1)	Si(1,3,9)	54.24	108.88	104.479	0.96	104.106	10.14	0.1
Dia(1,1,1)	Si(4,4,8)	56.45	98.22	143.969	1.47	98.975	14.64	0.15
Dia(1,1,1)	Si(1,7,7)	57.82	96.86	85.982	0.89	90.915	7.5	0.08
Dia(1,1,1)	Si(5,5,7)	57.82	96.85	85.982	0.89	90.917	7.5	0.08
Dia(1,1,1)	Si(0,2,10)	60.17	86.96	120.564	1.39	85.507	12.02	0.14
Dia(1,1,1)	Si(1,5,9)	61.63	84.14	71.376	0.85	216.194	5.62	0.03
Dia(1,1,1)	Si(3,5,9)	65.81	70.55	59.676	0.85	36.46	4.81	0.13
Dia(1,1,1)	Si(2,4,10)	68.72	60.59	86.308	1.42	58.609	7.79	0.13
Dia(1,1,1)	Si(1,1,11)	70.63	55.48	50.128	0.9	28.606	3.58	0.13
Dia(1,1,1)	Si(0,8,8)	74.23	44.13	74.241	1.68	24.039	6.52	0.27
Dia(1,1,1)	Si(1,3,11)	76.8	37.4	42.912	1.15	19.514	3.38	0.17
Dia(1,1,1)	Si(0,6,10)	82.74	19.85	64.362	3.24	4.715	10.31	2.19

Ei = 12.824 keV				Cryst 1-2			Cryst 1-2-3	
Cryst 1	Cryst 2,3	ΘB 2 [°]	ΔE [meV]	∫IR dΘdE [μrad meV]	FOM	ΔE [meV]	∫IR dΘdE [μrad meV]	FOM
Si(1,1,1)	Si(1,1,1)	8.87	1187.81	12744.247	10.73	1133.9	11253.47	9.92
Si(1,1,1)	Si(0,2,2)	14.58	705.5	7015.732	9.94	470.388	4250.41	9.04
Si(1,1,1)	Si(1,1,3)	17.17	513.07	3398.53	6.62	202.43	1179.54	5.83
Si(1,1,1)	Si(0,0,4)	20.86	435.49	3175.394	7.29	176.245	1156.94	6.56
Si(1,1,1)	Si(1,3,3)	22.83	381.83	1734.098	4.54	101.047	393.84	3.9
Si(1,1,1)	Si(2,2,4)	25.85	339.6	1877.909	5.53	100.665	495.85	4.93
Si(1,1,1)	Si(3,3,3)	27.55	315.28	1075.676	3.41	61.828	186.42	3.02
Si(1,1,1)	Si(0,4,4)	30.23	284.24	1243.692	4.38	67.61	266	3.93
Si(1,1,1)	Si(1,3,5)	31.77	267.01	726.883	2.72	41.629	96.14	2.31
Si(1,1,1)	Si(0,2,6)	34.26	245.63	878.417	3.58	48.253	153.17	3.17
Si(1,1,1)	Si(3,3,5)	35.71	231.46	517.969	2.24	31.289	53.06	1.7
Si(1,1,1)	Si(4,4,4)	38.07	213.14	644.856	3.03	35.491	92.02	2.59
Si(1,1,1)	Si(1,1,7)	39.47	202.52	382.069	1.89	100.216	36.56	0.36
Si(1,1,1)	Si(2,4,6)	41.77	186.36	487.871	2.62	27.348	57.15	2.09
Si(1,1,1)	Si(1,3,7)	43.13	177.7	289.621	1.63	86.729	25.3	0.29
Si(1,1,1)	Si(0,0,8)	45.4	165.06	376.961	2.28	22.092	37.64	1.7
Si(1,1,1)	Si(3,3,7)	46.77	154.98	223.443	1.44	75.562	17.1	0.23
Si(1,1,1)	Si(0,6,6)	49.05	144.99	297.065	2.05	35.508	29.36	0.83
Si(1,1,1)	Si(5,5,5)	50.43	137.83	176.03	1.28	66.046	13.93	0.21
Si(1,1,1)	Si(0,4,8)	52.76	127.05	237.101	1.87	62.49	21.47	0.34
Si(1,1,1)	Si(1,1,9)	54.18	119.73	140.518	1.17	57.392	10.5	0.18
Si(1,1,1)	Si(4,6,6)	56.61	109.87	193.132	1.76	53.953	16.28	0.3
Si(1,1,1)	Si(1,3,9)	58.11	103.33	113.672	1.1	49.315	7.99	0.16
Si(1,1,1)	Si(4,4,8)	60.7	93.67	158.359	1.69	45.84	13	0.28
Si(1,1,1)	Si(1,7,7)	62.33	86.17	92.409	1.07	41.552	6.35	0.15
Si(1,1,1)	Si(0,2,10)	65.19	77.91	130.901	1.68	37.799	10.42	0.28
Si(1,1,1)	Si(1,5,9)	67.03	70.55	76.214	1.08	33.71	4.96	0.15
Si(1,1,1)	Si(3,7,7)	67.03	70.55	76.214	1.08	33.71	4.96	0.15
Si(1,1,1)	Si(3,5,9)	72.65	52.2	63.248	1.21	25.186	3.85	0.15
Si(1,1,1)	Si(2,4,10)	77.17	38.67	92.488	2.39	5.925	9.44	1.59
Si(1,1,1)	Si(1,1,11)	80.81	27.33	52.92	1.94	3.965	4.2	1.06
Si(1,1,1)	Si(5,7,7)	80.81	27.33	52.92	1.94	3.965	4.2	1.06

Ei = 12.824 keV			Cryst 1-2			Cryst 1-2-3		
Cryst 1	Cryst 2,3	ΘB 2 [°]	ΔE [meV]	∫IR dΘdE [μrad meV]	FOM	ΔE [meV]	∫IR dΘdE [μrad meV]	FOM
Dia(1,1,1)	Si(1,1,1)	8.87	734.48	7695.209	10.48	732.773	7107.21	9.7
Dia(1,1,1)	Si(0,2,2)	14.58	480.63	4948.085	10.29	452.59	4396.49	9.71
Dia(1,1,1)	Si(1,1,3)	17.17	354.35	2529.496	7.14	201.367	1250.4	6.21
Dia(1,1,1)	Si(0,0,4)	20.86	314.72	2429.221	7.72	179.007	1247.17	6.97
Dia(1,1,1)	Si(1,3,3)	22.83	277.35	1355.659	4.89	99.736	421.36	4.22
Dia(1,1,1)	Si(2,2,4)	25.85	254.01	1493.782	5.88	102.719	525.31	5.11
Dia(1,1,1)	Si(3,3,3)	27.55	233.94	865.633	3.7	61.481	196.96	3.2
Dia(1,1,1)	Si(0,4,4)	30.23	216.21	1022.97	4.73	68.054	278.59	4.09
Dia(1,1,1)	Si(1,3,5)	31.77	205.81	599.525	2.91	41.972	105.45	2.51
Dia(1,1,1)	Si(0,2,6)	34.26	190.04	732.726	3.86	48.246	163.72	3.39
Dia(1,1,1)	Si(3,3,5)	35.71	183.53	436.573	2.38	31.266	56.52	1.81
Dia(1,1,1)	Si(4,4,4)	38.07	170.31	546.362	3.21	35.563	100.56	2.83
Dia(1,1,1)	Si(1,1,7)	39.47	163.22	327.078	2	100.472	37.55	0.37
Dia(1,1,1)	Si(2,4,6)	41.77	153.97	418.628	2.72	27.492	62.12	2.26
Dia(1,1,1)	Si(1,3,7)	43.13	145.92	249.531	1.71	86.516	26.15	0.3
Dia(1,1,1)	Si(0,0,8)	45.4	136.99	327.919	2.39	22.146	40.72	1.84
Dia(1,1,1)	Si(3,3,7)	46.77	131.62	194.207	1.48	75.484	20.15	0.27
Dia(1,1,1)	Si(0,6,6)	49.05	122.13	259.669	2.13	35.51	29.94	0.84
Dia(1,1,1)	Si(5,5,5)	50.43	119.06	154.237	1.3	327.082	14.51	0.04
Dia(1,1,1)	Si(0,4,8)	52.76	109.21	209.13	1.91	62.702	22.57	0.36
Dia(1,1,1)	Si(1,1,9)	54.18	107.16	124.38	1.16	102.285	11.13	0.11
Dia(1,1,1)	Si(4,6,6)	56.61	95.94	169.316	1.76	269.49	17.49	0.06
Dia(1,1,1)	Si(1,3,9)	58.11	94.15	101.007	1.07	87.976	8.41	0.1
Dia(1,1,1)	Si(4,4,8)	60.7	83.78	141.273	1.69	229.546	13.7	0.06
Dia(1,1,1)	Si(1,7,7)	62.33	80.41	82.81	1.03	74.22	6.6	0.09
Dia(1,1,1)	Si(0,2,10)	65.19	69.94	118.331	1.69	68.753	10.98	0.16
Dia(1,1,1)	Si(1,5,9)	67.03	65.32	68.9	1.05	60.405	5.34	0.09
Dia(1,1,1)	Si(3,7,7)	67.03	65.32	68.901	1.05	60.404	5.34	0.09
Dia(1,1,1)	Si(3,5,9)	72.65	48.73	57.942	1.19	45.332	4.61	0.1
Dia(1,1,1)	Si(2,4,10)	77.17	34.83	85.067	2.44	5.905	10.21	1.73
Dia(1,1,1)	Si(1,1,11)	80.81	25.08	49.024	1.95	3.968	4.49	1.13
Dia(1,1,1)	Si(5,7,7)	80.81	25.08	49.024	1.95	3.968	4.49	1.13

Ei = 11.215 keV			Cryst 1-2			Cryst 1-2-3		
Cryst 1	Cryst 2,3	ΘB 2 [°]	ΔE [meV]	∫IR dΘdE [µrad meV]	FOM	ΔE [meV]	∫IR dΘdE [µrad meV]	FOM
Si(1,1,1)	Si(1,1,1)	10.15	1056.68	11724.748	11.1	1007.24	10114.26	10.04
Si(1,1,1)	Si(0,2,2)	16.73	617.77	6487.511	10.5	419.215	3908.51	9.32
Si(1,1,1)	Si(1,1,3)	19.73	431.42	3090.114	7.16	178.545	1077.79	6.04
Si(1,1,1)	Si(0,0,4)	24.02	363.43	2900.106	7.98	160.164	1092.56	6.82
Si(1,1,1)	Si(1,3,3)	26.34	310.46	1565.379	5.04	87.762	358.15	4.08
Si(1,1,1)	Si(2,2,4)	29.91	274.51	1707.235	6.22	90.326	473.85	5.25
Si(1,1,1)	Si(3,3,3)	31.93	248.2	963.308	3.88	54.265	171.15	3.15
Si(1,1,1)	Si(0,4,4)	35.15	222.99	1123.531	5.04	59.851	246.91	4.13
Si(1,1,1)	Si(1,3,5)	37.02	203.92	645.662	3.17	36.594	92.03	2.51
Si(1,1,1)	Si(0,2,6)	40.07	185.5	788.494	4.25	42.756	147.91	3.46
Si(1,1,1)	Si(3,3,5)	41.87	170.92	456.529	2.67	26.483	52.3	1.97
Si(1,1,1)	Si(4,4,4)	44.84	156.41	577.089	3.69	31.469	94.23	2.99
Si(1,1,1)	Si(1,1,7)	46.62	144.85	335.073	2.31	20.167	32.18	1.6
Si(1,1,1)	Si(2,4,6)	49.61	131.96	434.58	3.29	24.234	62.13	2.56
Si(1,1,1)	Si(1,3,7)	51.42	122.2	252.102	2.06	16.214	22.01	1.36
Si(1,1,1)	Si(0,0,8)	54.51	110.45	334.524	3.03	18.763	43.42	2.31
Si(1,1,1)	Si(3,3,7)	56.42	101.46	193.841	1.91	25.311	15.07	0.6
Si(1,1,1)	Si(0,6,6)	59.73	90.33	262.496	2.91	15.065	32.24	2.14
Si(1,1,1)	Si(5,5,5)	61.81	81.99	151.184	1.84	20.453	11.22	0.55
Si(1,1,1)	Si(0,4,8)	65.55	70.83	209.46	2.96	12.252	25.86	2.11
Si(1,1,1)	Si(1,1,9)	68.01	61.96	119.939	1.94	8.364	8.99	1.07
Si(1,1,1)	Si(4,6,6)	72.7	48.94	169.076	3.46	10.268	24.27	2.36
Si(1,1,1)	Si(1,3,9)	76.14	38.42	96.457	2.51	6.624	9.7	1.46
Si(1,1,1)	Si(4,4,8)	85.73	15.8	138.211	8.75	8.956	53.62	5.99
Dia(1,1,1)	Si(1,1,1)	10.15	646.69	7192.006	11.12	3672.38	6532.17	1.78
Dia(1,1,1)	Si(0,2,2)	16.73	430.33	4732.955	11	407.809	4121.45	10.11
Dia(1,1,1)	Si(1,1,3)	19.73	309.13	2402.16	7.77	180.244	1176.78	6.53
Dia(1,1,1)	Si(0,0,4)	24.02	273.93	2345.252	8.56	157.573	1191.37	7.56
Dia(1,1,1)	Si(1,3,3)	26.34	236.12	1294.734	5.48	87.179	388.18	4.45
Dia(1,1,1)	Si(2,2,4)	29.91	214.37	1441.939	6.73	89.486	515.3	5.76
Dia(1,1,1)	Si(3,3,3)	31.93	195.87	823.057	4.2	54.679	186.1	3.4
Dia(1,1,1)	Si(0,4,4)	35.15	178.61	976.632	5.47	59.35	269.86	4.55
Dia(1,1,1)	Si(1,3,5)	37.02	168.57	565.267	3.35	36.052	102.35	2.84
Dia(1,1,1)	Si(0,2,6)	40.07	154	694.992	4.51	42.618	162.49	3.81
Dia(1,1,1)	Si(3,3,5)	41.87	146.1	407.147	2.79	26.348	56.66	2.15
Dia(1,1,1)	Si(4,4,4)	44.84	133.79	517.466	3.87	31.495	101.88	3.23
Dia(1,1,1)	Si(1,1,7)	46.62	126.74	301.826	2.38	20.188	35.7	1.77
Dia(1,1,1)	Si(2,4,6)	49.61	115.53	396.535	3.43	23.822	68.27	2.87
Dia(1,1,1)	Si(1,3,7)	51.42	110.19	230.423	2.09	16.26	22.75	1.4
Dia(1,1,1)	Si(0,0,8)	54.51	98.31	309.02	3.14	18.829	48.04	2.55
Dia(1,1,1)	Si(3,3,7)	56.42	94.12	179.998	1.91	25.305	16.24	0.64
Dia(1,1,1)	Si(0,6,6)	59.73	81.96	245.147	2.99	14.933	35.33	2.37
Dia(1,1,1)	Si(5,5,5)	61.81	77.31	142.354	1.84	20.46	12.17	0.6
Dia(1,1,1)	Si(0,4,8)	65.55	64.94	197.516	3.04	12.342	27.92	2.26
Dia(1,1,1)	Si(1,1,9)	68.01	59.22	114.188	1.93	8.361	9.82	1.17
Dia(1,1,1)	Si(4,6,6)	72.7	45.09	161.481	3.58	10.385	26.17	2.52
Dia(1,1,1)	Si(1,3,9)	76.14	36.26	92.567	2.55	6.584	10.77	1.64
Dia(1,1,1)	Si(4,4,8)	85.73	14.78	135.073	9.14	8.856	58.33	6.59

Ei = 13.88 keV				Cryst 1-2			Cryst 1-2-3	
Cryst 1	Cryst 2,3	ΘB 2 [°]	ΔE [meV]	∫IR dΘdE [µrad meV]	FOM	ΔE [meV]	∫IR dΘdE [µrad meV]	FOM
Si(1,1,1)	Si(1,1,1)	8.19	1267.21	13315.129	10.51	1203.26	11944.88	9.93
Si(1,1,1)	Si(0,2,2)	13.45	761.94	7325.455	9.61	501.06	4420.41	8.82
Si(1,1,1)	Si(1,1,3)	15.83	566.59	3562.859	6.29	217.159	1214.29	5.59
Si(1,1,1)	Si(0,0,4)	19.2	483.03	3327.988	6.89	194.585	1210.27	6.22
Si(1,1,1)	Si(1,3,3)	21.01	429.17	1831.891	4.27	108.159	413.33	3.82
Si(1,1,1)	Si(2,2,4)	23.76	382.88	1973.382	5.15	110.555	508.19	4.6
Si(1,1,1)	Si(1,1,5)	25.3	358.92	1138.784	3.17	65.999	191.66	2.9
Si(1,1,1)	Si(0,4,4)	27.72	324.13	1307.824	4.03	72.411	272.49	3.76
Si(1,1,1)	Si(1,3,5)	29.11	308.92	770.709	2.49	45.664	97.66	2.14
Si(1,1,1)	Si(0,2,6)	31.34	284.69	926.803	3.26	51.92	156.73	3.02
Si(1,1,1)	Si(3,3,5)	32.63	270.39	550.574	2.04	67.402	57.1	0.85
Si(1,1,1)	Si(4,4,4)	34.73	250.49	681.49	2.72	38.735	93.04	2.4
Si(1,1,1)	Si(1,1,7)	35.96	240.99	409.264	1.7	118.471	37.86	0.32
Si(1,1,1)	Si(1,5,5)	35.96	240.99	409.262	1.7	118.471	37.86	0.32
Si(1,1,1)	Si(2,4,6)	37.98	223.71	517.446	2.31	30.38	55.7	1.83
Si(1,1,1)	Si(1,3,7)	39.17	215.93	311.182	1.44	103.924	29.03	0.28
Si(1,1,1)	Si(0,0,8)	41.14	201.09	401.251	2	99.443	40.95	0.41
Si(1,1,1)	Si(3,3,7)	42.31	193.31	241.897	1.25	92.367	19.8	0.21
Si(1,1,1)	Si(0,6,6)	44.25	180.8	316.278	1.75	88.453	29.95	0.34
Si(1,1,1)	Si(1,5,7)	45.41	172.13	190.52	1.11	82.097	15.49	0.19
Si(1,1,1)	Si(0,4,8)	47.35	162.06	255.007	1.57	78.371	23.46	0.3
Si(1,1,1)	Si(1,1,9)	48.52	154.24	152.719	0.99	73.604	12.18	0.17
Si(1,1,1)	Si(4,6,6)	50.48	145.22	206.998	1.43	69.681	18.8	0.27
Si(1,1,1)	Si(1,3,9)	51.67	139.04	123.716	0.89	65.661	10.17	0.15
Si(1,1,1)	Si(4,4,8)	53.68	128.27	169.941	1.32	62.035	14.38	0.23
Si(1,1,1)	Si(1,7,7)	54.91	122.79	101.198	0.82	58.186	7.3	0.13
Si(1,1,1)	Si(0,2,10)	57	114.29	141.048	1.23	54.482	11.34	0.21
Si(1,1,1)	Si(1,5,9)	58.28	107.81	83.438	0.77	51.126	5.67	0.11
Si(1,1,1)	Si(3,7,7)	58.28	107.81	83.438	0.77	51.126	5.67	0.11
Si(1,1,1)	Si(3,5,9)	61.87	94.03	69.244	0.74	44.248	4.49	0.1
Si(1,1,1)	Si(2,4,10)	64.27	84.73	99.903	1.18	40.377	7.78	0.19
Si(1,1,1)	Si(1,1,11)	65.79	78.53	58.224	0.74	37.237	3.56	0.1
Si(1,1,1)	Si(5,7,7)	65.79	78.53	58.224	0.74	37.237	3.56	0.1
Si(1,1,1)	Si(0,8,8)	68.5	69.26	84.904	1.23	33.181	6.38	0.19
Si(1,1,1)	Si(1,3,11)	70.26	62.89	49.206	0.78	29.737	3.25	0.11
Si(1,1,1)	Si(1,7,9)	70.26	62.89	49.206	0.78	29.737	3.25	0.11
Si(1,1,1)	Si(0,6,10)	73.54	52.27	72.716	1.39	25.233	5.49	0.22
Si(1,1,1)	Si(3,3,11)	75.83	44.42	41.781	0.94	21.134	2.64	0.12
Si(1,1,1)	Si(3,7,9)	75.83	44.42	41.781	0.94	21.134	2.64	0.12
Si(1,1,1)	Si(0,0,12)	80.69	29.29	62.713	2.14	4.268	5.76	1.35
Si(1,1,1)	Si(1,5,11)	85.61	13.84	35.826	2.59	2.839	4.12	1.45
Dia(1,1,1)	Si(1,1,1)	8.19	791.9	7985.547	10.08	788.769	7444.52	9.44
Dia(1,1,1)	Si(0,2,2)	13.45	514.62	5053.488	9.82	488.998	4513.52	9.23
Dia(1,1,1)	Si(1,1,3)	15.83	383.99	2589.39	6.74	215.91	1279.45	5.93
Dia(1,1,1)	Si(0,0,4)	19.2	341.07	2486.399	7.29	192.075	1279.14	6.66
Dia(1,1,1)	Si(1,3,3)	21.01	303.89	1394.936	4.59	106.649	437.18	4.1
Dia(1,1,1)	Si(2,2,4)	23.76	278.76	1521.788	5.46	111.244	536.94	4.83
Dia(1,1,1)	Si(1,1,5)	25.3	259.15	893.584	3.45	66.812	203.48	3.05
Dia(1,1,1)	Si(0,4,4)	27.72	240.6	1041.049	4.33	72.132	292.57	4.06
Dia(1,1,1)	Si(1,3,5)	29.11	229.07	613.789	2.68	44.856	103.67	2.31
Dia(1,1,1)	Si(0,2,6)	31.34	212.86	742.924	3.49	51.393	162.53	3.16

Dia(1,1,1)	Si(3,3,5)	32.63	205.88	446.4	2.17	67.57	61.07	0.9
Dia(1,1,1)	Si(4,4,4)	34.73	191.37	553.089	2.89	38.756	96.74	2.5
Dia(1,1,1)	Si(1,1,7)	35.96	186.72	338.827	1.81	118.436	41.42	0.35
Dia(1,1,1)	Si(1,5,5)	35.96	186.72	338.826	1.81	118.436	41.42	0.35
Dia(1,1,1)	Si(2,4,6)	37.98	175.24	430.885	2.46	30.298	58.73	1.94
Dia(1,1,1)	Si(1,3,7)	39.17	167.88	259.67	1.55	103.806	29.57	0.28
Dia(1,1,1)	Si(0,0,8)	41.14	159.81	339.155	2.12	100.421	41.39	0.41
Dia(1,1,1)	Si(3,3,7)	42.31	151.5	202.328	1.34	455.75	22.87	0.05
Dia(1,1,1)	Si(0,6,6)	44.25	144.7	269.512	1.86	88.085	32.03	0.36
Dia(1,1,1)	Si(1,5,7)	45.41	138.95	161.746	1.16	146.192	16.01	0.11
Dia(1,1,1)	Si(0,4,8)	47.35	130.37	216.173	1.66	390.14	23.81	0.06
Dia(1,1,1)	Si(1,1,9)	48.52	128.21	130.741	1.02	130.582	13.06	0.1
Dia(1,1,1)	Si(4,6,6)	50.48	119.35	177.4	1.49	125.276	19.28	0.15
Dia(1,1,1)	Si(1,3,9)	51.67	117.43	106.602	0.91	116.536	9.77	0.08
Dia(1,1,1)	Si(4,4,8)	53.68	108.49	146.504	1.35	111.055	16.09	0.14
Dia(1,1,1)	Si(1,7,7)	54.91	107.63	88.136	0.82	103.238	7.83	0.08
Dia(1,1,1)	Si(0,2,10)	57	98.11	122.859	1.25	97.73	12.39	0.13
Dia(1,1,1)	Si(1,5,9)	58.28	96.49	73.393	0.76	251.172	6.23	0.02
Dia(1,1,1)	Si(3,7,7)	58.28	96.49	73.394	0.76	251.17	6.23	0.02
Dia(1,1,1)	Si(3,5,9)	61.87	84.49	61.478	0.73	44.097	5.02	0.11
Dia(1,1,1)	Si(2,4,10)	64.27	75.2	87.545	1.16	201.632	7.87	0.04
Dia(1,1,1)	Si(1,1,11)	65.79	71.66	51.67	0.72	37.168	4.06	0.11
Dia(1,1,1)	Si(5,7,7)	65.79	71.65	51.671	0.72	37.168	4.06	0.11
Dia(1,1,1)	Si(0,8,8)	68.5	62.02	74.92	1.21	33.216	7.21	0.22
Dia(1,1,1)	Si(1,3,11)	70.26	57.51	43.825	0.76	29.745	3.07	0.1
Dia(1,1,1)	Si(1,7,9)	70.26	57.51	43.826	0.76	29.745	3.07	0.1
Dia(1,1,1)	Si(0,6,10)	73.54	46.75	64.761	1.39	25.257	5.69	0.23
Dia(1,1,1)	Si(3,3,11)	75.83	40.77	37.573	0.92	21.129	2.61	0.12
Dia(1,1,1)	Si(3,7,9)	75.83	40.77	37.573	0.92	21.129	2.61	0.12
Dia(1,1,1)	Si(0,0,12)	80.69	25.79	56.585	2.19	4.251	6.26	1.47
Dia(1,1,1)	Si(1,5,11)	85.61	12.14	32.614	2.69	2.839	4.33	1.53

Ei = 13.273 keV			Cryst 1-2			Cryst 1-2-3		
Cryst 1	Cryst 2,3	ΘB 2 [°]	ΔE [meV]	∫IR dΘdE [µrad meV]	FOM	ΔE [meV]	∫IR dΘdE [µrad meV]	FOM
Si(1,1,1)	Si(1,1,1)	8.57	1221.96	12991.954	10.63	1163.09	11580.82	9.96
Si(1,1,1)	Si(0,2,2)	14.08	728.48	7159.385	9.83	484.614	4328.26	8.93
Si(1,1,1)	Si(1,1,3)	16.57	535.93	3471.592	6.48	208.26	1196.64	5.75
Si(1,1,1)	Si(0,0,4)	20.12	455.7	3241.573	7.11	183.28	1174.27	6.41
Si(1,1,1)	Si(1,3,3)	22.02	401.7	1776.913	4.42	103.339	400.56	3.88
Si(1,1,1)	Si(2,2,4)	24.92	358.06	1919.691	5.36	104.547	501.2	4.79
Si(1,1,1)	Si(1,1,5)	26.54	333.98	1102.973	3.3	63.217	187.94	2.97
Si(1,1,1)	Si(0,4,4)	29.11	301.19	1270.137	4.22	69.176	272	3.93
Si(1,1,1)	Si(1,3,5)	30.58	284.11	745.738	2.62	43.278	96.93	2.24
Si(1,1,1)	Si(0,2,6)	32.95	261.81	899.589	3.44	49.613	154.25	3.11
Si(1,1,1)	Si(3,3,5)	34.33	247.89	532.936	2.15	33.125	54.58	1.65
Si(1,1,1)	Si(4,4,4)	36.57	228.62	661.031	2.89	36.667	93.25	2.54
Si(1,1,1)	Si(1,1,7)	37.89	218.94	394.255	1.8	107.75	37.29	0.35
Si(1,1,1)	Si(2,4,6)	40.06	202.57	501.319	2.47	28.373	55.92	1.97
Si(1,1,1)	Si(1,3,7)	41.34	193.11	299.033	1.55	93.94	26.68	0.28
Si(1,1,1)	Si(0,0,8)	43.47	180.13	387.781	2.15	23.692	39.02	1.65
Si(1,1,1)	Si(3,3,7)	44.74	171.89	231.624	1.35	82.568	19.43	0.24
Si(1,1,1)	Si(0,6,6)	46.86	160.08	305.713	1.91	78.868	29.1	0.37
Si(1,1,1)	Si(1,5,7)	48.14	152.36	182.883	1.2	72.937	14.67	0.2
Si(1,1,1)	Si(0,4,8)	50.28	142.12	245.006	1.72	69.436	21.92	0.32
Si(1,1,1)	Si(1,1,9)	51.58	135.19	145.959	1.08	64.114	10.75	0.17
Si(1,1,1)	Si(4,6,6)	53.78	124.77	199.192	1.6	60.647	17.33	0.29
Si(1,1,1)	Si(1,3,9)	55.12	118.1	117.933	1	56.199	8.91	0.16
Si(1,1,1)	Si(4,4,8)	57.42	109.29	163.524	1.5	52.684	13.94	0.26
Si(1,1,1)	Si(1,7,7)	58.83	102.82	96.295	0.94	48.729	7.43	0.15
Si(1,1,1)	Si(0,2,10)	61.28	93.57	135.581	1.45	45.116	11.2	0.25
Si(1,1,1)	Si(1,5,9)	62.82	87.29	79.299	0.91	41.347	5.84	0.14
Si(1,1,1)	Si(3,5,9)	67.25	71.15	65.928	0.93	33.812	4.7	0.14
Si(1,1,1)	Si(2,4,10)	70.4	60.99	95.434	1.56	29.692	7.32	0.25
Si(1,1,1)	Si(1,1,11)	72.51	53.82	55.337	1.03	25.657	3.57	0.14
Si(1,1,1)	Si(0,8,8)	76.64	40.92	81.272	1.99	5.508	6.74	1.22
Si(1,1,1)	Si(1,3,11)	79.83	30.68	46.653	1.52	7.584	3.05	0.4
Si(1,1,1)	Si(1,7,9)	79.83	30.68	46.653	1.52	7.584	3.05	0.4
Dia(1,1,1)	Si(1,1,1)	8.57	758.91	7821.435	10.31	761.094	7283.47	9.57
Dia(1,1,1)	Si(0,2,2)	14.08	494.93	4998.477	10.1	471.45	4434.19	9.41
Dia(1,1,1)	Si(1,1,3)	16.57	366.48	2555.098	6.97	206.451	1259.88	6.1
Dia(1,1,1)	Si(0,0,4)	20.12	326.04	2452.88	7.52	182.844	1253.36	6.85
Dia(1,1,1)	Si(1,3,3)	22.02	289.64	1375.44	4.75	103.367	430.32	4.16
Dia(1,1,1)	Si(2,2,4)	24.92	264.73	1504.929	5.68	106.042	528.06	4.98
Dia(1,1,1)	Si(1,1,5)	26.54	245.08	877.057	3.58	64.137	201.03	3.13
Dia(1,1,1)	Si(0,4,4)	29.11	226.77	1030.265	4.54	71.008	283.77	4
Dia(1,1,1)	Si(1,3,5)	30.58	215.46	605.535	2.81	43.374	104.71	2.41
Dia(1,1,1)	Si(0,2,6)	32.95	199.55	738.048	3.7	49.935	164	3.28
Dia(1,1,1)	Si(3,3,5)	34.33	192.53	440.825	2.29	33.086	58.62	1.77
Dia(1,1,1)	Si(4,4,4)	36.57	179.28	549.424	3.06	36.911	101.24	2.74
Dia(1,1,1)	Si(1,1,7)	37.89	173.42	332.628	1.92	108.049	39.19	0.36
Dia(1,1,1)	Si(2,4,6)	40.06	162.78	424.806	2.61	28.382	60.02	2.11
Dia(1,1,1)	Si(1,3,7)	41.34	155.14	254.1	1.64	93.862	27.54	0.29
Dia(1,1,1)	Si(0,0,8)	43.47	147.1	333.756	2.27	23.677	41.84	1.77
Dia(1,1,1)	Si(3,3,7)	44.74	140.43	198.234	1.41	82.41	21.69	0.26
Dia(1,1,1)	Si(0,6,6)	46.86	131.98	264.311	2	79.144	30.3	0.38

Dia(1,1,1)	Si(1,5,7)	48.14	128.66	157.278	1.22	129.785	15.88	0.12
Dia(1,1,1)	Si(0,4,8)	50.28	118.54	212.613	1.79	345.484	23.22	0.07
Dia(1,1,1)	Si(1,1,9)	51.58	116.95	127.181	1.09	114.283	11.85	0.1
Dia(1,1,1)	Si(4,6,6)	53.78	106.81	173.899	1.63	303.073	17.78	0.06
Dia(1,1,1)	Si(1,3,9)	55.12	105.37	103.627	0.98	100.159	9.38	0.09
Dia(1,1,1)	Si(4,4,8)	57.42	94.85	143.367	1.51	95.112	14.32	0.15
Dia(1,1,1)	Si(1,7,7)	58.83	92.99	85.068	0.91	86.846	7.86	0.09
Dia(1,1,1)	Si(0,2,10)	61.28	83.06	119.817	1.44	81.531	11.57	0.14
Dia(1,1,1)	Si(1,5,9)	62.82	79.66	70.663	0.89	204.691	6.11	0.03
Dia(1,1,1)	Si(3,5,9)	67.25	65.42	59.023	0.9	33.798	4.92	0.15
Dia(1,1,1)	Si(2,4,10)	70.4	55.3	86.147	1.56	53.827	7.68	0.14
Dia(1,1,1)	Si(1,1,11)	72.51	49.79	50.018	1	25.653	3.59	0.14
Dia(1,1,1)	Si(0,8,8)	76.64	36.86	73.935	2.01	5.503	7.26	1.32
Dia(1,1,1)	Si(1,3,11)	79.83	28.34	42.657	1.51	7.593	3.28	0.43
Dia(1,1,1)	Si(1,7,9)	79.83	28.34	42.657	1.51	7.593	3.28	0.43

Ei = 11.564 keV				Cryst 1-2			Cryst 1-2-3	
Cryst 1	Cryst 2,3	ΘB 2 [°]	ΔE [meV]	∫IR dΘdE [µrad meV]	FOM	ΔE [meV]	∫IR dΘdE [µrad meV]	FOM
Si(1,1,1)	Si(1,1,1)	9.84	1085.72	11967.86	11.02	1037.93	10395.42	10.02
Si(1,1,1)	Si(0,2,2)	16.21	636.8	6622.826	10.4	430.619	3995.8	9.28
Si(1,1,1)	Si(1,1,3)	19.11	449.12	3163.952	7.04	182.893	1101.5	6.02
Si(1,1,1)	Si(0,0,4)	23.26	379.11	2963.693	7.82	164.015	1115.68	6.8
Si(1,1,1)	Si(1,3,3)	25.48	325.84	1605.587	4.93	90.573	365.53	4.04
Si(1,1,1)	Si(2,2,4)	28.92	288.6	1748.695	6.06	92.721	477.49	5.15
Si(1,1,1)	Si(1,1,5)	30.86	262.12	989.055	3.77	55.755	174.23	3.12
Si(1,1,1)	Si(0,4,4)	33.94	236.32	1151.828	4.87	61.906	251.41	4.06
Si(1,1,1)	Si(1,3,5)	35.73	217.41	664.71	3.06	37.79	93.69	2.48
Si(1,1,1)	Si(0,2,6)	38.63	198.22	810.413	4.09	43.631	149.58	3.43
Si(1,1,1)	Si(3,3,5)	40.34	184.66	471.093	2.55	27.506	52.44	1.91
Si(1,1,1)	Si(4,4,4)	43.15	168.58	592	3.51	32.13	93.74	2.92
Si(1,1,1)	Si(1,1,7)	44.82	157.14	345.644	2.2	21.161	33.11	1.56
Si(1,1,1)	Si(2,4,6)	47.62	143.88	446.914	3.11	24.603	61.25	2.49
Si(1,1,1)	Si(1,3,7)	49.3	134.1	259.9	1.94	33.102	21.87	0.66
Si(1,1,1)	Si(0,0,8)	52.15	122.42	344.786	2.82	19.492	41.74	2.14
Si(1,1,1)	Si(3,3,7)	53.9	113.88	200.838	1.76	56.091	15.73	0.28
Si(1,1,1)	Si(0,6,6)	56.88	102.57	270.6	2.64	15.505	29.62	1.91
Si(1,1,1)	Si(1,5,7)	58.74	94.6	157.297	1.66	46.513	11.8	0.25
Si(1,1,1)	Si(0,4,8)	61.99	83.69	216.026	2.58	12.639	22.84	1.81
Si(1,1,1)	Si(1,1,9)	64.06	76.05	124.631	1.64	37.354	8.97	0.24
Si(1,1,1)	Si(4,6,6)	67.81	64.37	174.181	2.71	10.443	19.78	1.89
Si(1,1,1)	Si(1,3,9)	70.32	55.91	100.131	1.79	7.392	7.03	0.95
Si(1,1,1)	Si(4,4,8)	75.27	42.19	142.751	3.38	8.829	20.21	2.29
Si(1,1,1)	Si(1,7,7)	79.15	30.54	81.375	2.66	5.688	8.97	1.58
Si(1,1,1)	Si(5,5,7)	79.15	30.54	81.375	2.66	5.688	8.97	1.58
Dia(1,1,1)	Si(1,1,1)	9.84	665.87	7308.774	10.98	3823.5	6695.76	1.75
Dia(1,1,1)	Si(0,2,2)	16.21	441.08	4785.713	10.85	417.112	4187.1	10.04
Dia(1,1,1)	Si(1,1,3)	19.11	319.06	2430.605	7.62	185.215	1196.1	6.46
Dia(1,1,1)	Si(0,0,4)	23.26	282.94	2363.909	8.35	162.491	1205.51	7.42
Dia(1,1,1)	Si(1,3,3)	25.48	245.27	1306.894	5.33	90	395.81	4.4
Dia(1,1,1)	Si(2,2,4)	28.92	223.07	1456.522	6.53	92.348	518.66	5.62
Dia(1,1,1)	Si(1,1,5)	30.86	203.74	833.514	4.09	55.864	189.85	3.4
Dia(1,1,1)	Si(0,4,4)	33.94	187.02	989.42	5.29	61.929	270.99	4.38
Dia(1,1,1)	Si(1,3,5)	35.73	176.84	574.71	3.25	37.116	103.12	2.78
Dia(1,1,1)	Si(0,2,6)	38.63	162.05	705.298	4.35	43.479	162.32	3.73
Dia(1,1,1)	Si(3,3,5)	40.34	154.68	414.745	2.68	27.288	56.16	2.06
Dia(1,1,1)	Si(4,4,4)	43.15	141.7	523.129	3.69	32.689	102	3.12
Dia(1,1,1)	Si(1,1,7)	44.82	134.95	307.638	2.28	21.183	35.13	1.66
Dia(1,1,1)	Si(2,4,6)	47.62	124.2	400.987	3.23	24.649	68.21	2.77
Dia(1,1,1)	Si(1,3,7)	49.3	117.98	233.917	1.98	33.043	23.6	0.71
Dia(1,1,1)	Si(0,0,8)	52.15	107.51	312.947	2.91	19.428	44.33	2.28
Dia(1,1,1)	Si(3,3,7)	53.9	103.23	182.816	1.77	56.213	16.84	0.3
Dia(1,1,1)	Si(0,6,6)	56.88	91.78	248.569	2.71	15.627	32.46	2.08
Dia(1,1,1)	Si(1,5,7)	58.74	88.06	145.195	1.65	46.625	12.5	0.27
Dia(1,1,1)	Si(0,4,8)	61.99	76.17	200.615	2.63	12.657	24.81	1.96
Dia(1,1,1)	Si(1,1,9)	64.06	71.34	116.45	1.63	37.431	9.56	0.26
Dia(1,1,1)	Si(4,6,6)	67.81	59.19	163.866	2.77	10.447	21.44	2.05
Dia(1,1,1)	Si(1,3,9)	70.32	53.43	94.764	1.77	140	7.62	0.05
Dia(1,1,1)	Si(4,4,8)	75.27	38.66	135.506	3.51	8.904	21.72	2.44
Dia(1,1,1)	Si(1,7,7)	79.15	28.36	77.644	2.74	5.59	9.96	1.78

**Dia(1,1,1)**	**Si(5,5,7)**	**79.15**	**28.36**	**77.645**	**2.74**	**5.59**	**9.96**	**1.78**

Ei = 14.353 keV			Cryst 1-2			Cryst 1-2-3		
Cryst 1	Cryst 2,3	ΘB 2 [°]	ΔE [meV]	∫IR dΘdE [µrad meV]	FOM	ΔE [meV]	∫IR dΘdE [µrad meV]	FOM
Si(1,1,1)	Si(1,1,1)	7.92	1302.14	13559.327	10.41	1229.45	12247.07	9.96
Si(1,1,1)	Si(0,2,2)	13	787.23	7451.582	9.47	516.155	4483.99	8.69
Si(1,1,1)	Si(1,1,3)	15.29	590.46	3628.193	6.14	224.079	1231.71	5.5
Si(1,1,1)	Si(0,0,4)	18.55	503.27	3388.449	6.73	200.63	1230.46	6.13
Si(1,1,1)	Si(1,3,3)	20.28	450.1	1873.45	4.16	111.847	421.84	3.77
Si(1,1,1)	Si(2,2,4)	22.93	401.46	2010.505	5.01	114.692	518.02	4.52
Si(1,1,1)	Si(1,1,5)	24.41	377.64	1165.3	3.09	68.476	194.95	2.85
Si(1,1,1)	Si(0,4,4)	26.74	341.85	1335.437	3.91	76.504	274.07	3.58
Si(1,1,1)	Si(1,3,5)	28.07	327.7	790.384	2.41	47.378	97.28	2.05
Si(1,1,1)	Si(0,2,6)	30.2	302.17	946.623	3.13	53.968	158.95	2.95
Si(1,1,1)	Si(3,3,5)	31.43	286.84	564.014	1.97	147.077	59.88	0.41
Si(1,1,1)	Si(4,4,4)	33.43	267.64	697.543	2.61	40.199	92.01	2.29
Si(1,1,1)	Si(1,1,7)	34.61	259.25	418.649	1.61	126.901	40.53	0.32
Si(1,1,1)	Si(2,4,6)	36.52	239.8	529.846	2.21	32.181	56.18	1.75
Si(1,1,1)	Si(1,3,7)	37.65	232.73	319.65	1.37	111.871	30.6	0.27
Si(1,1,1)	Si(3,5,5)	37.65	232.73	319.649	1.37	111.87	30.6	0.27
Si(1,1,1)	Si(0,0,8)	39.51	215.64	410.992	1.91	107.898	42.04	0.39
Si(1,1,1)	Si(3,3,7)	40.61	209.53	248.436	1.19	99.926	22.94	0.23
Si(1,1,1)	Si(0,6,6)	42.44	196.24	326.327	1.66	95.663	31.17	0.33
Si(1,1,1)	Si(1,5,7)	43.53	190.07	196.202	1.03	89.786	15.78	0.18
Si(1,1,1)	Si(0,4,8)	45.34	178.34	262.042	1.47	85.54	24.54	0.29
Si(1,1,1)	Si(1,1,9)	46.43	171.09	157.685	0.92	80.934	13.43	0.17
Si(1,1,1)	Si(4,6,6)	48.25	160.46	212.842	1.33	77.066	19.45	0.25
Si(1,1,1)	Si(1,3,9)	49.34	153.45	127.812	0.83	72.723	10.14	0.14
Si(1,1,1)	Si(4,4,8)	51.19	144.95	175.228	1.21	68.831	15.82	0.23
Si(1,1,1)	Si(1,7,7)	52.31	138.03	104.568	0.76	65.205	7.34	0.11
Si(1,1,1)	Si(5,5,7)	52.31	138.03	104.569	0.76	65.205	7.34	0.11
Si(1,1,1)	Si(0,2,10)	54.2	129.37	145.614	1.13	61.429	12.18	0.2
Si(1,1,1)	Si(1,5,9)	55.35	123.77	86.456	0.7	58.199	5.9	0.1
Si(1,1,1)	Si(3,7,7)	55.35	123.77	86.457	0.7	58.199	5.9	0.1
Si(1,1,1)	Si(3,5,9)	58.52	110.26	72.313	0.66	51.47	4.71	0.09
Si(1,1,1)	Si(2,4,10)	60.59	101.84	102.814	1.01	47.801	8.03	0.17
Si(1,1,1)	Si(1,1,11)	61.88	96.09	60.618	0.63	44.893	4.76	0.11
Si(1,1,1)	Si(0,8,8)	64.12	87.63	87.943	1	41.164	6.43	0.16
Si(1,1,1)	Si(1,3,11)	65.54	81.99	51.149	0.62	38.298	3.91	0.1
Si(1,1,1)	Si(0,6,10)	68.04	72.44	75.176	1.04	34.347	5.59	0.16
Si(1,1,1)	Si(3,3,11)	69.65	66.27	43.6	0.66	31.391	2.57	0.08
Si(1,1,1)	Si(3,7,9)	69.65	66.27	43.6	0.66	31.391	2.57	0.08
Si(1,1,1)	Si(0,0,12)	72.62	56.27	64.646	1.15	26.913	4.71	0.18
Si(1,1,1)	Si(1,5,11)	74.62	49.24	37.297	0.76	23.312	2.43	0.1
Si(1,1,1)	Si(7,7,7)	74.62	49.24	37.297	0.76	23.312	2.43	0.1
Si(1,1,1)	Si(2,2,12)	78.66	36.23	56.072	1.55	17.766	4.21	0.24
Si(1,1,1)	Si(3,5,11)	81.93	25.61	32.176	1.26	12.513	2	0.16
Dia(1,1,1)	Si(1,1,1)	7.92	817.11	8094.219	9.91	815.341	7594.47	9.31
Dia(1,1,1)	Si(0,2,2)	13	529.71	5093.041	9.61	502.73	4561.43	9.07
Dia(1,1,1)	Si(1,1,3)	15.29	396.69	2609.33	6.58	224.108	1292.91	5.77
Dia(1,1,1)	Si(0,0,4)	18.55	352.27	2506.543	7.12	195.255	1278.99	6.55
Dia(1,1,1)	Si(1,3,3)	20.28	315.34	1410.144	4.47	110.156	443.63	4.03
Dia(1,1,1)	Si(2,2,4)	22.93	289.82	1537.546	5.31	114.17	541.68	4.74
Dia(1,1,1)	Si(1,1,5)	24.41	270.15	905.323	3.35	67.997	202	2.97
Dia(1,1,1)	Si(0,4,4)	26.74	251.17	1048.65	4.18	75.594	288.67	3.82

Dia(1,1,1)	Si(1,3,5)	28.07	239.59	622.605	2.6	46.775	102.44	2.19
Dia(1,1,1)	Si(0,2,6)	30.2	222.83	750.831	3.37	53.753	165.02	3.07
Dia(1,1,1)	Si(3,3,5)	31.43	216.48	452.041	2.09	145.052	62.95	0.43
Dia(1,1,1)	Si(4,4,4)	33.43	201.39	556.132	2.76	39.83	94.08	2.36
Dia(1,1,1)	Si(1,1,7)	34.61	196.87	342.173	1.74	126.347	43.02	0.34
Dia(1,1,1)	Si(2,4,6)	36.52	184.96	434.572	2.35	32.128	59.61	1.86
Dia(1,1,1)	Si(1,3,7)	37.65	178.04	263.208	1.48	111.619	31.17	0.28
Dia(1,1,1)	Si(3,5,5)	37.65	178.04	263.204	1.48	111.619	31.16	0.28
Dia(1,1,1)	Si(0,0,8)	39.51	169.27	342.052	2.02	107.721	44.88	0.42
Dia(1,1,1)	Si(3,3,7)	40.61	161.4	205.632	1.27	177.57	22.36	0.13
Dia(1,1,1)	Si(0,6,6)	42.44	154.66	273.71	1.77	474.862	33.16	0.07
Dia(1,1,1)	Si(1,5,7)	43.53	147.45	163.867	1.11	159.179	17.43	0.11
Dia(1,1,1)	Si(0,4,8)	45.34	140.69	220.202	1.57	153.333	25.08	0.16
Dia(1,1,1)	Si(1,1,9)	46.43	135.73	132.81	0.98	143.178	14.63	0.1
Dia(1,1,1)	Si(4,6,6)	48.25	128.43	180.06	1.4	137.553	20.37	0.15
Dia(1,1,1)	Si(1,3,9)	49.34	125.95	109.008	0.87	128.919	10.52	0.08
Dia(1,1,1)	Si(4,4,8)	51.19	117.79	149.126	1.27	123.201	15.73	0.13
Dia(1,1,1)	Si(1,7,7)	52.31	115.71	89.81	0.78	115.502	7.98	0.07
Dia(1,1,1)	Si(5,5,7)	52.31	115.7	89.811	0.78	115.503	7.98	0.07
Dia(1,1,1)	Si(0,2,10)	54.2	107.54	124.949	1.16	61.324	12.82	0.21
Dia(1,1,1)	Si(1,5,9)	55.35	106.52	75.011	0.7	283.738	6.79	0.02
Dia(1,1,1)	Si(3,7,7)	55.35	106.52	75.008	0.7	283.735	6.79	0.02
Dia(1,1,1)	Si(3,5,9)	58.52	97.07	63.104	0.65	51.312	5.78	0.11
Dia(1,1,1)	Si(2,4,10)	60.59	87.5	89.38	1.02	47.91	8.55	0.18
Dia(1,1,1)	Si(1,1,11)	61.88	85.58	53.381	0.62	44.787	4.69	0.1
Dia(1,1,1)	Si(0,8,8)	64.12	76.5	76.522	1	41.165	6.78	0.16
Dia(1,1,1)	Si(1,3,11)	65.54	73.54	45.267	0.62	38.205	3.8	0.1
Dia(1,1,1)	Si(0,6,10)	68.04	64.56	65.873	1.02	34.319	5.78	0.17
Dia(1,1,1)	Si(3,3,11)	69.65	60.32	38.374	0.64	31.328	3.04	0.1
Dia(1,1,1)	Si(3,7,9)	69.65	60.32	38.374	0.64	31.329	3.04	0.1
Dia(1,1,1)	Si(0,0,12)	72.62	50.22	56.932	1.13	26.923	5.27	0.2
Dia(1,1,1)	Si(1,5,11)	74.62	44.95	33.109	0.74	23.313	2.44	0.1
Dia(1,1,1)	Si(7,7,7)	74.62	44.95	33.11	0.74	23.313	2.44	0.1
Dia(1,1,1)	Si(2,2,12)	78.66	32.15	49.922	1.55	17.802	4.37	0.25
Dia(1,1,1)	Si(3,5,11)	81.93	22.94	28.781	1.25	12.536	2.08	0.17

Ei = 13.734 keV			Cryst 1-2			Cryst 1-2-3		
Cryst 1	Cryst 2,3	ΘB 2 [°]	ΔE [meV]	∫IR dΘdE [μrad meV]	FOM	ΔE [meV]	∫IR dΘdE [μrad meV]	FOM
Si(1,1,1)	Si(1,1,1)	8.28	1256.48	13237.297	10.54	1191.51	11851.31	9.95
Si(1,1,1)	Si(0,2,2)	13.6	754.04	7300.06	9.68	497.606	4391.43	8.83
Si(1,1,1)	Si(1,1,3)	16	559.05	3540.644	6.33	215.271	1209.71	5.62
Si(1,1,1)	Si(0,0,4)	19.42	475.95	3301.394	6.94	191.71	1200.09	6.26
Si(1,1,1)	Si(1,3,3)	21.24	422.63	1822.048	4.31	107.05	410.42	3.83
Si(1,1,1)	Si(2,2,4)	24.03	377.08	1960.696	5.2	110.506	504.45	4.56
Si(1,1,1)	Si(1,1,5)	25.59	353.59	1129.15	3.19	65.271	190.61	2.92
Si(1,1,1)	Si(0,4,4)	28.04	318.81	1299.507	4.08	71.505	273.26	3.82
Si(1,1,1)	Si(1,3,5)	29.45	303.03	765.172	2.53	45.056	97.41	2.16
Si(1,1,1)	Si(0,2,6)	31.71	279.21	920.864	3.3	51.297	155.98	3.04
Si(1,1,1)	Si(3,3,5)	33.02	264.51	546.412	2.07	66.252	56.42	0.85
Si(1,1,1)	Si(4,4,4)	35.16	245.45	677.334	2.76	38.144	93.07	2.44
Si(1,1,1)	Si(1,1,7)	36.41	235.6	404.962	1.72	115.88	37.27	0.32
Si(1,1,1)	Si(2,4,6)	38.46	218.63	514.083	2.35	29.846	55.7	1.87
Si(1,1,1)	Si(1,3,7)	39.67	210.36	308.19	1.47	101.5	28.53	0.28
Si(1,1,1)	Si(3,5,5)	39.67	210.36	308.19	1.47	101.5	28.53	0.28
Si(1,1,1)	Si(0,0,8)	41.67	196.13	398.625	2.03	97.171	40.55	0.42
Si(1,1,1)	Si(3,3,7)	42.87	188.75	239.234	1.27	89.958	19.66	0.22
Si(1,1,1)	Si(0,6,6)	44.85	175.71	314.751	1.79	86.362	30.38	0.35
Si(1,1,1)	Si(1,5,7)	46.03	167.44	188.655	1.13	79.857	15.33	0.19
Si(1,1,1)	Si(0,4,8)	48.02	157.55	252.593	1.6	76.182	23.06	0.3
Si(1,1,1)	Si(1,1,9)	49.22	149.57	150.703	1.01	71.289	11.9	0.17
Si(1,1,1)	Si(4,6,6)	51.23	140.14	204.634	1.46	67.496	18.5	0.27
Si(1,1,1)	Si(1,3,9)	52.45	133.73	122.251	0.91	63.436	10.08	0.16
Si(1,1,1)	Si(4,4,8)	54.52	124.17	168.525	1.36	59.82	14.48	0.24
Si(1,1,1)	Si(1,7,7)	55.79	117.7	99.967	0.85	55.985	7.44	0.13
Si(1,1,1)	Si(5,5,7)	55.79	117.7	99.968	0.85	55.985	7.44	0.13
Si(1,1,1)	Si(0,2,10)	57.95	108.64	139.798	1.29	52.331	11.03	0.21
Si(1,1,1)	Si(1,5,9)	59.28	103.04	82.483	0.8	48.846	5.55	0.11
Si(1,1,1)	Si(3,5,9)	63.03	88.37	68.61	0.78	41.833	4.42	0.11
Si(1,1,1)	Si(2,4,10)	65.57	79.32	98.763	1.25	37.989	7.49	0.2
Si(1,1,1)	Si(1,1,11)	67.18	73.24	57.54	0.79	34.617	3.81	0.11
Si(1,1,1)	Si(0,8,8)	70.1	63.35	84.071	1.33	30.523	6.6	0.22
Si(1,1,1)	Si(1,3,11)	72.04	56.4	48.64	0.86	26.736	3.31	0.12
Si(1,1,1)	Si(1,7,9)	72.04	56.4	48.64	0.86	26.736	3.31	0.12
Si(1,1,1)	Si(0,6,10)	75.75	44.48	71.911	1.62	21.842	5.3	0.24
Si(1,1,1)	Si(3,3,11)	78.48	35.55	41.34	1.16	17.242	2.68	0.16
Si(1,1,1)	Si(0,0,12)	85.81	13.51	62.117	4.6	4.133	12.29	2.97
Dia(1,1,1)	Si(1,1,1)	8.28	783.65	7946.685	10.14	781.315	7399.21	9.47
Dia(1,1,1)	Si(0,2,2)	13.6	509.77	5040.324	9.89	485.949	4502.62	9.27
Dia(1,1,1)	Si(1,1,3)	16	379.8	2581.854	6.8	214.387	1281.72	5.98
Dia(1,1,1)	Si(0,0,4)	19.42	337.34	2479.156	7.35	189.728	1272.93	6.71
Dia(1,1,1)	Si(1,3,3)	21.24	300.29	1389.779	4.63	105.724	435.52	4.12
Dia(1,1,1)	Si(2,2,4)	24.03	275.58	1517.389	5.51	109.448	535.55	4.89
Dia(1,1,1)	Si(1,1,5)	25.59	255.76	889.557	3.48	66.401	203.63	3.07
Dia(1,1,1)	Si(0,4,4)	28.04	237.24	1038.577	4.38	71.912	288.88	4.02
Dia(1,1,1)	Si(1,3,5)	29.45	225.1	610.592	2.71	44.515	102.18	2.3
Dia(1,1,1)	Si(0,2,6)	31.71	209.27	740.301	3.54	50.994	162.75	3.19
Dia(1,1,1)	Si(3,3,5)	33.02	202.62	444.589	2.19	66.384	60.46	0.91
Dia(1,1,1)	Si(4,4,4)	35.16	188.19	551.692	2.93	37.621	96.31	2.56
Dia(1,1,1)	Si(1,1,7)	36.41	183.62	337.698	1.84	116.03	40.89	0.35

Dia(1,1,1)	Si(2,4,6)	38.46	172.38	429.351	2.49	29.832	58.89	1.97
Dia(1,1,1)	Si(1,3,7)	39.67	164.84	258.178	1.57	101.407	29.07	0.29
Dia(1,1,1)	Si(3,5,5)	39.67	164.84	258.177	1.57	101.407	29.07	0.29
Dia(1,1,1)	Si(0,0,8)	41.67	156.82	337.934	2.15	47.83	42.44	0.89
Dia(1,1,1)	Si(3,3,7)	42.87	149.12	201.492	1.35	89.587	22.86	0.26
Dia(1,1,1)	Si(0,6,6)	44.85	141.75	268.562	1.89	85.817	31.63	0.37
Dia(1,1,1)	Si(1,5,7)	46.03	136.4	160.737	1.18	142.235	15.76	0.11
Dia(1,1,1)	Si(0,4,8)	48.02	128.48	216.259	1.68	379.251	23.48	0.06
Dia(1,1,1)	Si(1,1,9)	49.22	125.41	129.987	1.04	126.622	12.77	0.1
Dia(1,1,1)	Si(4,6,6)	51.23	116.32	176.411	1.52	121.441	18.91	0.16
Dia(1,1,1)	Si(1,3,9)	52.45	115.42	106.121	0.92	112.541	10.18	0.09
Dia(1,1,1)	Si(4,4,8)	54.52	105.58	145.921	1.38	107.253	15.55	0.14
Dia(1,1,1)	Si(1,7,7)	55.79	104.8	87.384	0.83	99.402	7.69	0.08
Dia(1,1,1)	Si(5,5,7)	55.79	104.8	87.385	0.83	99.404	7.69	0.08
Dia(1,1,1)	Si(0,2,10)	57.95	94.25	121.585	1.29	93.906	12.81	0.14
Dia(1,1,1)	Si(1,5,9)	59.28	92.83	72.821	0.78	240.373	6.11	0.03
Dia(1,1,1)	Si(3,5,9)	63.03	80.34	60.927	0.76	41.799	4.9	0.12
Dia(1,1,1)	Si(2,4,10)	65.57	70.73	87.198	1.23	68.208	8.52	0.12
Dia(1,1,1)	Si(1,1,11)	67.18	66.92	51.132	0.76	34.618	3.97	0.11
Dia(1,1,1)	Si(0,8,8)	70.1	56.85	74.62	1.31	30.491	6.82	0.22
Dia(1,1,1)	Si(1,3,11)	72.04	51.75	43.439	0.84	26.721	3.61	0.14
Dia(1,1,1)	Si(1,7,9)	72.04	51.75	43.439	0.84	26.721	3.61	0.14
Dia(1,1,1)	Si(0,6,10)	75.75	39.99	64.647	1.62	21.886	5.69	0.26
Dia(1,1,1)	Si(3,3,11)	78.48	32.88	37.326	1.14	17.254	2.93	0.17
Dia(1,1,1)	Si(0,0,12)	85.81	11.68	56.643	4.85	4.017	13.24	3.3

Ei = 11.919 keV			Cryst 1-2			Cryst 1-2-3		
Cryst 1	Cryst 2,3	ΘB 2 [°]	ΔE [meV]	∫IR dΘdE [µrad meV]	FOM	ΔE [meV]	∫IR dΘdE [µrad meV]	FOM
Si(1,1,1)	Si(1,1,1)	9.55	1114.5	12200.096	10.95	1065.5	10644.92	9.99
Si(1,1,1)	Si(0,2,2)	15.72	656.31	6744.667	10.28	442.492	4082.51	9.23
Si(1,1,1)	Si(1,1,3)	18.52	467.28	3234.445	6.92	188.471	1125.94	5.97
Si(1,1,1)	Si(0,0,4)	22.52	394.94	3023.395	7.66	168.94	1136.41	6.73
Si(1,1,1)	Si(1,3,3)	24.67	341.98	1643.53	4.81	93.501	373.39	3.99
Si(1,1,1)	Si(2,2,4)	27.98	302.81	1786.758	5.9	95.743	482.78	5.04
Si(1,1,1)	Si(1,1,5)	29.84	277.19	1015.214	3.66	57.391	177.77	3.1
Si(1,1,1)	Si(0,4,4)	32.8	250.02	1179.983	4.72	63.625	255.09	4.01
Si(1,1,1)	Si(1,3,5)	34.51	231.35	683.499	2.95	38.952	94.96	2.44
Si(1,1,1)	Si(0,2,6)	37.28	211.16	828.742	3.92	44.812	150.56	3.36
Si(1,1,1)	Si(3,3,5)	38.9	197.42	484.751	2.46	28.281	51.76	1.83
Si(1,1,1)	Si(4,4,4)	41.57	180.79	606.861	3.36	33.516	93.12	2.78
Si(1,1,1)	Si(1,1,7)	43.15	169.38	356.28	2.1	22.436	33.59	1.5
Si(1,1,1)	Si(1,5,5)	43.15	169.38	356.281	2.1	22.436	33.59	1.5
Si(1,1,1)	Si(2,4,6)	45.78	156.2	458.788	2.94	25.265	60.67	2.4
Si(1,1,1)	Si(1,3,7)	47.36	146.39	268.727	1.84	72.275	22.35	0.31
Si(1,1,1)	Si(0,0,8)	50.01	134.11	354.165	2.64	19.942	39.78	1.99
Si(1,1,1)	Si(3,3,7)	51.62	125.78	208.057	1.65	61.701	16.64	0.27
Si(1,1,1)	Si(0,6,6)	54.35	114.59	278.477	2.43	16.193	28.17	1.74
Si(1,1,1)	Si(1,5,7)	56.03	107.2	162.993	1.52	52.085	12.88	0.25
Si(1,1,1)	Si(5,5,5)	56.03	107.2	162.993	1.52	52.085	12.88	0.25
Si(1,1,1)	Si(0,4,8)	58.93	96.36	222.289	2.31	13.209	21.11	1.6
Si(1,1,1)	Si(1,1,9)	60.75	88.75	129.275	1.46	43.255	9.18	0.21
Si(1,1,1)	Si(4,6,6)	63.94	78.3	179.851	2.3	10.853	16.78	1.55
Si(1,1,1)	Si(1,3,9)	66	70.77	103.895	1.47	34.54	7.75	0.22
Si(1,1,1)	Si(4,4,8)	69.77	59.29	147.312	2.48	8.996	14.94	1.66
Si(1,1,1)	Si(1,7,7)	72.34	50.74	84.541	1.67	12.542	5.86	0.47
Si(1,1,1)	Si(5,5,7)	72.34	50.74	84.541	1.67	12.542	5.86	0.47
Si(1,1,1)	Si(0,2,10)	77.59	36.04	121.766	3.38	7.713	17.33	2.25
Si(1,1,1)	Si(1,5,9)	82.15	22.74	69.511	3.06	5.077	8.79	1.73
Dia(1,1,1)	Si(1,1,1)	9.55	685.36	7420.046	10.83	684.334	6838.81	9.99
Dia(1,1,1)	Si(0,2,2)	15.72	452.14	4836.38	10.7	424.931	4244.61	9.99
Dia(1,1,1)	Si(1,1,3)	18.52	328.4	2462.056	7.5	191.161	1220.12	6.38
Dia(1,1,1)	Si(0,0,4)	22.52	292.06	2378.945	8.15	168.787	1217.31	7.21
Dia(1,1,1)	Si(1,3,3)	24.67	254.62	1320.769	5.19	92.598	402.42	4.35
Dia(1,1,1)	Si(2,2,4)	27.98	231.6	1469.988	6.35	95.569	520.21	5.44
Dia(1,1,1)	Si(1,1,5)	29.84	212.74	845.003	3.97	57.053	191	3.35
Dia(1,1,1)	Si(0,4,4)	32.8	195.23	1000.723	5.13	63.899	273.17	4.28
Dia(1,1,1)	Si(1,3,5)	34.51	185.28	583	3.15	38.741	101.76	2.63
Dia(1,1,1)	Si(0,2,6)	37.28	170.23	711.279	4.18	44.876	160.81	3.58
Dia(1,1,1)	Si(3,3,5)	38.9	163.01	422.652	2.59	28.435	56.46	1.99
Dia(1,1,1)	Si(4,4,4)	41.57	150.3	529.95	3.53	33.066	100.03	3.03
Dia(1,1,1)	Si(1,1,7)	43.15	142.88	313.251	2.19	22.443	35.06	1.56
Dia(1,1,1)	Si(1,5,5)	43.15	142.88	313.251	2.19	22.443	35.06	1.56
Dia(1,1,1)	Si(2,4,6)	45.78	132.7	406.05	3.06	25.14	65.99	2.62
Dia(1,1,1)	Si(1,3,7)	47.36	126.47	238.659	1.89	72.723	24.34	0.33
Dia(1,1,1)	Si(0,0,8)	50.01	116.23	316.616	2.72	20.19	43.79	2.17
Dia(1,1,1)	Si(3,3,7)	51.62	112.23	185.824	1.66	61.601	17.43	0.28
Dia(1,1,1)	Si(0,6,6)	54.35	101.15	251.523	2.49	16.072	30.05	1.87
Dia(1,1,1)	Si(1,5,7)	56.03	97.96	147.465	1.51	52.083	13.33	0.26
Dia(1,1,1)	Si(5,5,5)	56.03	97.96	147.464	1.51	52.083	13.33	0.26

**Dia(1,1,1)**	**Si(0,4,8)**	**58.93**	**86.55**	**203.085**	**2.35**	**13.235**	**22.91**	**1.73**
**Dia(1,1,1)**	**Si(1,1,9)**	**60.75**	**82.81**	**118.794**	**1.43**	**215.031**	**10.18**	**0.05**
**Dia(1,1,1)**	**Si(4,6,6)**	**63.94**	**71.48**	**166.797**	**2.33**	**197.451**	**18.32**	**0.09**
**Dia(1,1,1)**	**Si(1,3,9)**	**66**	**66.74**	**96.637**	**1.45**	**144.919**	**7.81**	**0.05**
**Dia(1,1,1)**	**Si(4,4,8)**	**69.77**	**54.41**	**137.394**	**2.52**	**8.969**	**16.16**	**1.8**
**Dia(1,1,1)**	**Si(1,7,7)**	**72.34**	**48.18**	**79.437**	**1.65**	**106.198**	**6.24**	**0.06**
**Dia(1,1,1)**	**Si(5,5,7)**	**72.34**	**48.18**	**79.438**	**1.65**	**106.199**	**6.24**	**0.06**
**Dia(1,1,1)**	**Si(0,2,10)**	**77.59**	**32.71**	**114.797**	**3.51**	**7.747**	**18.58**	**2.4**
**Dia(1,1,1)**	**Si(1,5,9)**	**82.15**	**20.79**	**66.087**	**3.18**	**5.099**	**9.42**	**1.85**

Ei = 14.839 keV			Cryst 1-2			Cryst 1-2-3		
Cryst 1	Cryst 2,3	ΘB 2	ΔE	∫IR dΘdE	FOM	ΔE	∫IR dΘdE	FOM
		[°]	[meV]	[μrad meV]		[meV]	[μrad meV]	
Si(1,1,1)	Si(1,1,1)	7.66	1337.1	13800.293	10.32	1258.81	12500.25	9.93
Si(1,1,1)	Si(0,2,2)	12.57	812.88	7574.771	9.32	531.803	4533.05	8.52
Si(1,1,1)	Si(1,1,3)	14.78	612.42	3690.891	6.03	232.472	1250.06	5.38
Si(1,1,1)	Si(0,0,4)	17.92	525.39	3447.79	6.56	208.151	1253.34	6.02
Si(1,1,1)	Si(1,3,3)	19.59	472	1911	4.05	115.37	429.64	3.72
Si(1,1,1)	Si(2,2,4)	22.14	421.92	2049.734	4.86	117.824	525.76	4.46
Si(1,1,1)	Si(1,1,5)	23.56	396.44	1190.07	3	70.907	197.53	2.79
Si(1,1,1)	Si(0,4,4)	25.79	360.14	1364.134	3.79	77.668	282.02	3.63
Si(1,1,1)	Si(1,3,5)	27.07	347.05	809.606	2.33	49.352	96.78	1.96
Si(1,1,1)	Si(0,2,6)	29.11	320.75	967.503	3.02	55.576	159.26	2.87
Si(1,1,1)	Si(3,3,5)	30.29	305.38	576.19	1.89	156.095	62.28	0.4
Si(1,1,1)	Si(4,4,4)	32.2	285.37	712.419	2.5	40.936	90.13	2.2
Si(1,1,1)	Si(1,1,7)	33.32	274.61	430.346	1.57	135.66	42.56	0.31
Si(1,1,1)	Si(1,5,5)	33.32	274.61	430.345	1.57	135.659	42.56	0.31
Si(1,1,1)	Si(2,4,6)	35.14	254.92	540.274	2.12	64.387	57.43	0.89
Si(1,1,1)	Si(1,3,7)	36.22	248.92	328.274	1.32	120.19	32.24	0.27
Si(1,1,1)	Si(3,5,5)	36.22	248.92	328.272	1.32	120.19	32.24	0.27
Si(1,1,1)	Si(0,0,8)	37.98	232.06	420.095	1.81	115.813	44.68	0.39
Si(1,1,1)	Si(3,3,7)	39.02	226.04	254.205	1.12	107.673	24.78	0.23
Si(1,1,1)	Si(0,6,6)	40.75	212.75	333.76	1.57	103.317	32.78	0.32
Si(1,1,1)	Si(1,5,7)	41.77	205.68	202.035	0.98	97.679	17.36	0.18
Si(1,1,1)	Si(0,4,8)	43.47	194.77	268.396	1.38	93.012	25.33	0.27
Si(1,1,1)	Si(1,1,9)	44.49	187.79	162.39	0.86	88.384	14.96	0.17
Si(1,1,1)	Si(4,6,6)	46.19	176.76	218.999	1.24	84.132	18.99	0.23
Si(1,1,1)	Si(1,3,9)	47.2	169.85	131.769	0.78	79.747	10.3	0.13
Si(1,1,1)	Si(4,4,8)	48.91	160.55	180.416	1.12	75.852	16.85	0.22
Si(1,1,1)	Si(1,7,7)	49.94	155.29	108.159	0.7	72.29	7.82	0.11
Si(1,1,1)	Si(0,2,10)	51.67	147.15	149.575	1.02	68.782	12.04	0.18
Si(1,1,1)	Si(1,5,9)	52.72	140.17	90.037	0.64	65.241	6.21	0.1
Si(1,1,1)	Si(3,7,7)	52.72	140.16	90.036	0.64	65.242	6.22	0.1
Si(1,1,1)	Si(3,5,9)	55.58	125.06	75.072	0.6	58.495	5.79	0.1
Si(1,1,1)	Si(2,4,10)	57.42	117.67	106.466	0.9	55.515	8.96	0.16
Si(1,1,1)	Si(1,1,11)	58.55	111.58	63.073	0.57	52.004	4.17	0.08
Si(1,1,1)	Si(0,8,8)	60.49	104.01	90.655	0.87	49.099	7.81	0.16
Si(1,1,1)	Si(1,7,9)	61.69	98.44	53.362	0.54	45.741	3.21	0.07
Si(1,1,1)	Si(1,3,11)	61.69	98.43	53.348	0.54	45.741	3.21	0.07
Si(1,1,1)	Si(5,5,9)	61.69	98.43	53.349	0.54	45.741	3.21	0.07
Si(1,1,1)	Si(0,6,10)	63.77	90.48	77.735	0.86	42.724	6.49	0.15
Si(1,1,1)	Si(3,3,11)	65.08	84.72	45.364	0.54	39.494	2.75	0.07
Si(1,1,1)	Si(3,7,9)	65.08	84.72	45.363	0.54	39.494	2.75	0.07
Si(1,1,1)	Si(0,0,12)	67.38	76.38	66.969	0.88	36.15	5.49	0.15
Si(1,1,1)	Si(1,5,11)	68.85	71.12	38.951	0.55	33.048	2.59	0.08
Si(1,1,1)	Si(2,2,12)	71.51	61.54	58.048	0.94	29.043	4.2	0.14
Si(1,1,1)	Si(4,6,10)	71.51	61.54	58.048	0.94	29.043	4.2	0.14
Si(1,1,1)	Si(3,5,11)	73.27	55.03	33.521	0.61	25.992	1.9	0.07
Si(1,1,1)	Si(5,7,9)	73.27	55.03	33.521	0.61	25.992	1.9	0.07
Si(1,1,1)	Si(0,4,12)	76.65	43.73	50.529	1.16	20.94	3.57	0.17
Si(1,1,1)	Si(1,9,9)	79.13	35.39	29.055	0.82	16.728	1.85	0.11
Si(1,1,1)	Si(2,8,10)	85.58	14.58	44.364	3.04	3.112	6.4	2.06
Dia(1,1,1)	Si(1,1,1)	7.66	841.95	8194.721	9.73	843.642	7738.64	9.17
Dia(1,1,1)	Si(0,2,2)	12.57	545.46	5131.067	9.41	515.822	4612.33	8.94

Dia(1,1,1)	Si(1,1,3)	14.78	410.7	2629.412	6.4	228.594	1279.99	5.6
Dia(1,1,1)	Si(0,0,4)	17.92	364.91	2529.373	6.93	201.188	1286.08	6.39
Dia(1,1,1)	Si(1,3,3)	19.59	325.87	1419.373	4.36	114.45	451.35	3.94
Dia(1,1,1)	Si(2,2,4)	22.14	300.61	1552.37	5.16	118.081	549.42	4.65
Dia(1,1,1)	Si(1,1,5)	23.56	281.35	919.022	3.27	70.363	203.44	2.89
Dia(1,1,1)	Si(0,4,4)	25.79	262	1058.937	4.04	77.619	290.46	3.74
Dia(1,1,1)	Si(1,3,5)	27.07	249.62	630.817	2.53	49.119	101.67	2.07
Dia(1,1,1)	Si(0,2,6)	29.11	233.22	760.92	3.26	55.244	164.39	2.98
Dia(1,1,1)	Si(3,3,5)	30.29	226.86	457.619	2.02	154.297	64.81	0.42
Dia(1,1,1)	Si(4,4,4)	32.2	212.17	562.739	2.65	41.555	95.02	2.29
Dia(1,1,1)	Si(1,1,7)	33.32	207.35	345.597	1.67	134.787	44.43	0.33
Dia(1,1,1)	Si(1,5,5)	33.32	207.35	345.6	1.67	134.787	44.43	0.33
Dia(1,1,1)	Si(2,4,6)	35.14	194.74	437.164	2.24	64.497	61.25	0.95
Dia(1,1,1)	Si(1,3,7)	36.22	188.7	266.433	1.41	119.6	32.78	0.27
Dia(1,1,1)	Si(3,5,5)	36.22	188.7	266.434	1.41	119.6	32.78	0.27
Dia(1,1,1)	Si(0,0,8)	37.98	178.34	343.416	1.93	115.077	44.25	0.38
Dia(1,1,1)	Si(3,3,7)	39.02	171.26	208.23	1.22	191.691	25.44	0.13
Dia(1,1,1)	Si(0,6,6)	40.75	164.83	276.377	1.68	513.695	33.45	0.07
Dia(1,1,1)	Si(1,5,7)	41.77	156.58	166.125	1.06	172.682	18.55	0.11
Dia(1,1,1)	Si(0,4,8)	43.47	150.37	223.062	1.48	166.681	26.48	0.16
Dia(1,1,1)	Si(1,1,9)	44.49	143.52	134.051	0.93	156.182	14.7	0.09
Dia(1,1,1)	Si(4,6,6)	46.19	138.26	183.001	1.32	150.384	22.04	0.15
Dia(1,1,1)	Si(1,3,9)	47.2	133.41	109.963	0.82	141.507	10.7	0.08
Dia(1,1,1)	Si(4,4,8)	48.91	126.59	151.131	1.19	376.85	17.16	0.05
Dia(1,1,1)	Si(1,7,7)	49.94	123.63	91.563	0.74	352.779	8.55	0.02
Dia(1,1,1)	Si(0,2,10)	51.67	116.6	126.578	1.09	68.903	13.34	0.19
Dia(1,1,1)	Si(1,5,9)	52.72	115.24	76.273	0.66	318.017	7.75	0.02
Dia(1,1,1)	Si(3,7,7)	52.72	115.24	76.273	0.66	318.013	7.75	0.02
Dia(1,1,1)	Si(3,5,9)	55.58	106.35	64.345	0.61	58.414	6.26	0.11
Dia(1,1,1)	Si(2,4,10)	57.42	97.85	90.677	0.93	55.499	9.27	0.17
Dia(1,1,1)	Si(1,1,11)	58.55	97.21	54.616	0.56	52.05	5.02	0.1
Dia(1,1,1)	Si(0,8,8)	60.49	88.48	77.972	0.88	49.144	7.96	0.16
Dia(1,1,1)	Si(1,3,11)	61.69	87.43	46.728	0.53	45.745	4.23	0.09
Dia(1,1,1)	Si(1,7,9)	61.69	87.43	46.727	0.53	45.747	4.23	0.09
Dia(1,1,1)	Si(5,5,9)	61.69	87.43	46.726	0.53	45.747	4.23	0.09
Dia(1,1,1)	Si(0,6,10)	63.77	78.53	67.284	0.86	42.761	6.6	0.15
Dia(1,1,1)	Si(3,3,11)	65.08	76.01	39.871	0.52	39.483	3.46	0.09
Dia(1,1,1)	Si(3,7,9)	65.08	76.01	39.871	0.52	39.483	3.46	0.09
Dia(1,1,1)	Si(0,0,12)	67.38	67.34	58.259	0.87	36.19	5.65	0.16
Dia(1,1,1)	Si(1,5,11)	68.85	63.9	34.346	0.54	33.032	2.83	0.09
Dia(1,1,1)	Si(2,2,12)	71.51	54.61	50.653	0.93	29.027	4.14	0.14
Dia(1,1,1)	Si(4,6,10)	71.51	54.61	50.653	0.93	29.027	4.14	0.14
Dia(1,1,1)	Si(3,5,11)	73.27	49.84	29.399	0.59	25.954	2.26	0.09
Dia(1,1,1)	Si(5,7,9)	73.27	49.84	29.399	0.59	25.954	2.26	0.09
Dia(1,1,1)	Si(0,4,12)	76.65	38.57	44.317	1.15	20.967	4.16	0.2
Dia(1,1,1)	Si(1,9,9)	79.13	31.73	25.536	0.8	16.725	1.71	0.1
Dia(1,1,1)	Si(2,8,10)	85.58	12.42	39.596	3.19	3.151	6.61	2.1

Ei = 14.209 keV				Cryst 1-2			Cryst 1-2-3	
Cryst 1	Cryst 2,3	ΘB 2 [°]	ΔE [meV]	∫IR dΘdE [µrad meV]	FOM	ΔE [meV]	∫IR dΘdE [µrad meV]	FOM
Si(1,1,1)	Si(1,1,1)	8	1266.24	13575.633	10.72	1230.28	12142.38	9.87
Si(1,1,1)	Si(0,2,2)	13.13	779.52	7415.984	9.51	513.308	4471.38	8.71
Si(1,1,1)	Si(1,1,3)	15.45	583.13	3607.829	6.19	221.876	1226.5	5.53
Si(1,1,1)	Si(0,0,4)	18.74	497.34	3370.985	6.78	198.767	1225.11	6.16
Si(1,1,1)	Si(1,3,3)	20.5	443.85	1861.122	4.19	110.514	418.92	3.79
Si(1,1,1)	Si(2,2,4)	23.18	397.62	1994.855	5.02	113.346	514.77	4.54
Si(1,1,1)	Si(1,1,5)	24.67	372.21	1157.93	3.11	67.762	194.11	2.86
Si(1,1,1)	Si(0,4,4)	27.03	336.43	1326.722	3.94	75.405	273.19	3.62
Si(1,1,1)	Si(1,3,5)	28.38	322.31	784.338	2.43	46.897	97.64	2.08
Si(1,1,1)	Si(0,2,6)	30.54	296.85	940.734	3.17	53.288	158.3	2.97
Si(1,1,1)	Si(3,3,5)	31.79	281.23	559.512	1.99	102.931	59.17	0.57
Si(1,1,1)	Si(4,4,4)	33.82	262.9	691.841	2.63	39.996	93.11	2.33
Si(1,1,1)	Si(1,1,7)	35.01	252.49	416.477	1.65	124.326	39.93	0.32
Si(1,1,1)	Si(2,4,6)	36.95	234.71	525.939	2.24	31.604	55.9	1.77
Si(1,1,1)	Si(1,3,7)	38.1	227.59	317.113	1.39	109.436	30.13	0.28
Si(1,1,1)	Si(0,0,8)	39.99	211.87	408.711	1.93	105.145	41.76	0.4
Si(1,1,1)	Si(3,3,7)	41.11	204.67	246.903	1.21	97.689	21.1	0.22
Si(1,1,1)	Si(0,6,6)	42.97	191.81	323.638	1.69	93.434	30.7	0.33
Si(1,1,1)	Si(1,5,7)	44.08	181.4	194.35	1.07	87.402	15.35	0.18
Si(1,1,1)	Si(0,4,8)	45.93	172.26	259.123	1.5	83.348	24.27	0.29
Si(1,1,1)	Si(1,1,9)	47.04	165.63	156.327	0.94	78.718	13.13	0.17
Si(1,1,1)	Si(4,6,6)	48.9	155.78	211.372	1.36	74.799	16.8	0.22
Si(1,1,1)	Si(1,3,9)	50.02	148.49	126.427	0.85	70.6	10.21	0.14
Si(1,1,1)	Si(4,4,8)	51.91	140.67	173.454	1.23	66.742	14.54	0.22
Si(1,1,1)	Si(1,7,7)	53.06	134.73	103.668	0.77	63.087	7.38	0.12
Si(1,1,1)	Si(0,2,10)	55.01	124.81	144.727	1.16	59.334	12.05	0.2
Si(1,1,1)	Si(1,5,9)	56.2	118.97	85.638	0.72	56.104	5.92	0.11
Si(1,1,1)	Si(3,5,9)	59.48	105.06	71.05	0.68	49.336	4.65	0.09
Si(1,1,1)	Si(2,4,10)	61.64	96.3	102.178	1.06	45.592	7.85	0.17
Si(1,1,1)	Si(1,1,11)	62.99	90.82	59.752	0.66	42.681	4.28	0.1
Si(1,1,1)	Si(0,8,8)	65.35	82.11	86.53	1.05	38.827	6.58	0.17
Si(1,1,1)	Si(1,3,11)	66.85	75.99	50.525	0.66	35.923	3.01	0.08
Si(1,1,1)	Si(5,5,9)	66.85	75.99	50.524	0.66	35.924	3.01	0.08
Si(1,1,1)	Si(0,6,10)	69.53	66.76	74.391	1.11	31.749	5.31	0.17
Si(1,1,1)	Si(3,3,11)	71.28	60.18	43.108	0.72	28.533	2.64	0.09
Si(1,1,1)	Si(3,7,9)	71.28	60.17	43.108	0.72	28.533	2.64	0.09
Si(1,1,1)	Si(0,0,12)	74.58	49.27	64.12	1.3	23.845	5.05	0.21
Si(1,1,1)	Si(1,5,11)	76.9	41.66	36.927	0.89	19.733	2.4	0.12
Si(1,1,1)	Si(2,2,12)	82.06	25.28	55.679	2.2	3.837	5.37	1.4
Dia(1,1,1)	Si(1,1,1)	8	808.1	8052.23	9.96	806.676	7544.65	9.35
Dia(1,1,1)	Si(0,2,2)	13.13	525.09	5083.087	9.68	497.682	4542.67	9.13
Dia(1,1,1)	Si(1,1,3)	15.45	392.97	2602.313	6.62	222.031	1290.33	5.81
Dia(1,1,1)	Si(0,0,4)	18.74	349.29	2500.521	7.16	194.423	1279.45	6.58
Dia(1,1,1)	Si(1,3,3)	20.5	311.86	1405.69	4.51	109.033	441.59	4.05
Dia(1,1,1)	Si(2,2,4)	23.18	286.73	1532.806	5.35	113.142	539.78	4.77
Dia(1,1,1)	Si(1,1,5)	24.67	266.85	901.21	3.38	67.575	202.19	2.99
Dia(1,1,1)	Si(0,4,4)	27.03	247.96	1045.823	4.22	75.068	290.1	3.86
Dia(1,1,1)	Si(1,3,5)	28.38	236.28	619.479	2.62	46.14	102.69	2.23
Dia(1,1,1)	Si(0,2,6)	30.54	219.84	748.687	3.41	53.112	164.51	3.1
Dia(1,1,1)	Si(3,3,5)	31.79	213.23	450.524	2.11	70.245	62.4	0.89
Dia(1,1,1)	Si(4,4,4)	33.82	198.47	552.54	2.78	39.246	97.81	2.49

Dia(1,1,1)	Si(1,1,7)	35.01	193.51	340.049	1.76	123.926	42.55	0.34
Dia(1,1,1)	Si(2,4,6)	36.95	181.93	433.364	2.38	31.543	59.22	1.88
Dia(1,1,1)	Si(1,3,7)	38.1	174.92	262.242	1.5	109.226	30.68	0.28
Dia(1,1,1)	Si(0,0,8)	39.99	166.71	341.915	2.05	105.47	44.67	0.42
Dia(1,1,1)	Si(3,3,7)	41.11	158.47	204.847	1.29	173.43	22.62	0.13
Dia(1,1,1)	Si(0,6,6)	42.97	151.44	272.155	1.8	463.483	32.85	0.07
Dia(1,1,1)	Si(1,5,7)	44.08	144.91	163.314	1.13	155.194	17.1	0.11
Dia(1,1,1)	Si(0,4,8)	45.93	137.77	219.283	1.59	349.944	24.68	0.07
Dia(1,1,1)	Si(1,1,9)	47.04	133.24	131.884	0.99	139.249	13.67	0.1
Dia(1,1,1)	Si(4,6,6)	48.9	125.74	179.167	1.42	133.766	20.06	0.15
Dia(1,1,1)	Si(1,3,9)	50.02	123.44	108.283	0.88	125.161	9.49	0.08
Dia(1,1,1)	Si(4,4,8)	51.91	114.94	148.479	1.29	119.61	16.28	0.14
Dia(1,1,1)	Si(1,7,7)	53.06	114.02	89.247	0.78	261.787	8.15	0.03
Dia(1,1,1)	Si(0,2,10)	55.01	104.7	124.17	1.19	295.426	13.03	0.04
Dia(1,1,1)	Si(1,5,9)	56.2	104.09	74.574	0.72	274.162	6.61	0.02
Dia(1,1,1)	Si(3,5,9)	59.48	93.39	62.423	0.67	49.13	5.61	0.11
Dia(1,1,1)	Si(2,4,10)	61.64	83.82	88.872	1.06	45.541	8.1	0.18
Dia(1,1,1)	Si(1,1,11)	62.99	81.38	52.565	0.65	42.537	4.54	0.11
Dia(1,1,1)	Si(0,8,8)	65.35	72.8	76.081	1.05	38.789	6.78	0.17
Dia(1,1,1)	Si(1,3,11)	66.85	68.9	44.807	0.65	35.813	3.65	0.1
Dia(1,1,1)	Si(5,5,9)	66.85	68.9	44.808	0.65	35.813	3.65	0.1
Dia(1,1,1)	Si(0,6,10)	69.53	59.47	65.256	1.1	31.744	5.59	0.18
Dia(1,1,1)	Si(3,3,11)	71.28	54.94	38.234	0.7	28.502	2.87	0.1
Dia(1,1,1)	Si(3,7,9)	71.28	54.94	38.234	0.7	28.501	2.87	0.1
Dia(1,1,1)	Si(0,0,12)	74.58	44	56.647	1.29	23.856	5	0.21
Dia(1,1,1)	Si(1,5,11)	76.9	38.03	32.917	0.87	19.728	2.23	0.11
Dia(1,1,1)	Si(2,2,12)	82.06	22.14	49.971	2.26	3.819	5.79	1.52

Ei = 12.284 keV				Cryst 1-2			Cryst 1-2-3	
Cryst 1	Cryst 2,3	ΘB 2 [°]	ΔE [meV]	∫IR dΘdE [μrad meV]	FOM	ΔE [meV]	∫IR dΘdE [μrad meV]	FOM
Si(1,1,1)	Si(1,1,1)	9.26	1144.83	12429.555	10.86	1094.87	10898.83	9.95
Si(1,1,1)	Si(0,2,2)	15.24	676.53	6849.887	10.13	453.563	4148.36	9.15
Si(1,1,1)	Si(1,1,3)	17.95	485.96	3303.227	6.8	195.079	1152.2	5.91
Si(1,1,1)	Si(0,0,4)	21.82	411.3	3087.476	7.51	172.612	1150.69	6.67
Si(1,1,1)	Si(1,3,3)	23.89	357.99	1681.506	4.7	96.459	381.41	3.95
Si(1,1,1)	Si(2,2,4)	27.08	317.4	1824.088	5.75	98.348	486.07	4.94
Si(1,1,1)	Si(1,1,5)	28.87	292.24	1040.268	3.56	59.298	181.71	3.06
Si(1,1,1)	Si(0,4,4)	31.71	263.84	1207.352	4.58	64.987	260.68	4.01
Si(1,1,1)	Si(1,3,5)	33.35	245.78	701.327	2.85	39.774	94.98	2.39
Si(1,1,1)	Si(0,2,6)	35.99	224.93	850.042	3.78	46.619	151.74	3.25
Si(1,1,1)	Si(3,3,5)	37.54	211.16	498.793	2.36	29.273	51.68	1.77
Si(1,1,1)	Si(4,4,4)	40.07	193.87	622.496	3.21	34.008	96.68	2.84
Si(1,1,1)	Si(1,1,7)	41.57	182.82	366.453	2	45.134	32.73	0.73
Si(1,1,1)	Si(1,5,5)	41.57	182.82	366.453	2	45.134	32.73	0.73
Si(1,1,1)	Si(2,4,6)	44.06	169.13	471.342	2.79	26.267	60.3	2.3
Si(1,1,1)	Si(1,3,7)	45.54	159.2	277.327	1.74	78.301	23.52	0.3
Si(1,1,1)	Si(3,5,5)	45.54	159.2	277.327	1.74	78.301	23.52	0.3
Si(1,1,1)	Si(0,0,8)	48.02	145.62	362.686	2.49	20.807	38.71	1.86
Si(1,1,1)	Si(3,3,7)	49.52	138.32	214.513	1.55	67.207	17.61	0.26
Si(1,1,1)	Si(0,6,6)	52.04	127.02	285.836	2.25	16.977	27.6	1.63
Si(1,1,1)	Si(1,5,7)	53.58	119.12	168.543	1.41	57.684	12.96	0.22
Si(1,1,1)	Si(0,4,8)	56.21	108.8	228.894	2.1	14.275	21.64	1.52
Si(1,1,1)	Si(1,1,9)	57.84	101.62	134.071	1.32	49.035	9.55	0.19
Si(1,1,1)	Si(4,6,6)	60.65	91.34	185.216	2.03	22.706	16.2	0.71
Si(1,1,1)	Si(1,3,9)	62.43	84.33	107.871	1.28	40.752	7.39	0.18
Si(1,1,1)	Si(4,4,8)	65.57	73.96	151.751	2.05	9.755	13.61	1.4
Si(1,1,1)	Si(1,7,7)	67.6	66.75	87.827	1.32	32.343	5.62	0.17
Si(1,1,1)	Si(0,2,10)	71.37	55.21	125.644	2.28	7.847	11.59	1.48
Si(1,1,1)	Si(1,5,9)	73.98	46.67	72.127	1.55	11.324	4.73	0.42
Si(1,1,1)	Si(3,5,9)	85.18	14.6	59.955	4.11	4.339	10.12	2.33
Dia(1,1,1)	Si(1,1,1)	9.26	704.76	7528.078	10.68	703.258	6916.67	9.84
Dia(1,1,1)	Si(0,2,2)	15.24	463.51	4884.382	10.54	437.813	4318.21	9.86
Dia(1,1,1)	Si(1,1,3)	17.95	339.46	2492.419	7.34	198.03	1248.18	6.3
Dia(1,1,1)	Si(0,0,4)	21.82	300.93	2396.74	7.96	173.937	1236.32	7.11
Dia(1,1,1)	Si(1,3,3)	23.89	263.95	1334.585	5.06	95.283	409.71	4.3
Dia(1,1,1)	Si(2,2,4)	27.08	240.59	1481.265	6.16	98.324	520.02	5.29
Dia(1,1,1)	Si(1,1,5)	28.87	221.71	854.596	3.85	58.755	193.19	3.29
Dia(1,1,1)	Si(0,4,4)	31.71	203.79	1009.995	4.96	66.102	275.82	4.17
Dia(1,1,1)	Si(1,3,5)	33.35	192.36	586.862	3.05	40.163	100.49	2.5
Dia(1,1,1)	Si(0,2,6)	35.99	178.28	721.703	4.05	46.256	163.81	3.54
Dia(1,1,1)	Si(3,3,5)	37.54	171.54	428.94	2.5	29.507	56.35	1.91
Dia(1,1,1)	Si(4,4,4)	40.07	158.53	537.907	3.39	33.972	100.94	2.97
Dia(1,1,1)	Si(1,1,7)	41.57	151.07	319.084	2.11	45.168	35.28	0.78
Dia(1,1,1)	Si(1,5,5)	41.57	151.07	319.086	2.11	45.168	35.28	0.78
Dia(1,1,1)	Si(2,4,6)	44.06	141.05	411.95	2.92	25.965	65.06	2.51
Dia(1,1,1)	Si(1,3,7)	45.54	134.67	243.131	1.81	78.159	24.93	0.32
Dia(1,1,1)	Si(3,5,5)	45.54	134.67	243.132	1.81	78.158	24.93	0.32
Dia(1,1,1)	Si(0,0,8)	48.02	124.99	321.226	2.57	20.584	41.01	1.99
Dia(1,1,1)	Si(3,3,7)	49.52	119.99	189.006	1.58	67.252	18.36	0.27
Dia(1,1,1)	Si(0,6,6)	52.04	109.9	254.274	2.31	17.017	29.76	1.75
Dia(1,1,1)	Si(1,5,7)	53.58	107.08	150.016	1.4	57.65	14.82	0.26

**Dia(1,1,1)**	**Si(0,4,8)**	**56.21**	**95.69**	**205.008**	**2.14**	**14.305**	**22.32**	**1.56**
**Dia(1,1,1)**	**Si(1,1,9)**	**57.84**	**93.18**	**120.655**	**1.29**	**87.657**	**10.72**	**0.12**
**Dia(1,1,1)**	**Si(4,6,6)**	**60.65**	**82.48**	**168.282**	**2.04**	**227.769**	**17.03**	**0.07**
**Dia(1,1,1)**	**Si(1,3,9)**	**62.43**	**78.47**	**98.465**	**1.25**	**72.936**	**8.2**	**0.11**
**Dia(1,1,1)**	**Si(4,4,8)**	**65.57**	**67.63**	**139.242**	**2.06**	**185.849**	**14.05**	**0.08**
**Dia(1,1,1)**	**Si(1,7,7)**	**67.6**	**62.68**	**81.009**	**1.29**	**58.213**	**6.76**	**0.12**
**Dia(1,1,1)**	**Si(0,2,10)**	**71.37**	**50.57**	**116.144**	**2.3**	**139.534**	**12.56**	**0.09**
**Dia(1,1,1)**	**Si(1,5,9)**	**73.98**	**44.09**	**67.298**	**1.53**	**41.707**	**5.16**	**0.12**
**Dia(1,1,1)**	**Si(3,5,9)**	**85.18**	**12.98**	**56.662**	**4.37**	**4.338**	**10.84**	**2.5**

Ei = 15.347 keV			Cryst 1-2			Cryst 1-2-3		
Cryst 1	Cryst 2,3	ΘB 2 [°]	ΔE [meV]	∫IR dΘdE [μrad meV]	FOM	ΔE [meV]	∫IR dΘdE [μrad meV]	FOM
Si(1,1,1)	Si(1,1,1)	7.4	1373.38	14040.731	10.22	10955.7	12767.64	1.17
Si(1,1,1)	Si(0,2,2)	12.14	840.45	7697.594	9.16	535.688	4652.6	8.69
Si(1,1,1)	Si(1,1,3)	14.28	640.41	3758.954	5.87	245.671	1320.77	5.38
Si(1,1,1)	Si(0,0,4)	17.31	547.95	3502.853	6.39	216.725	1268.12	5.85
Si(1,1,1)	Si(1,3,3)	18.92	494.92	1947.422	3.93	121.428	437.31	3.6
Si(1,1,1)	Si(2,2,4)	21.37	443.03	2088.538	4.71	122.678	554.73	4.52
Si(1,1,1)	Si(1,1,5)	22.73	413.39	1212.225	2.93	152.919	196.18	1.28
Si(1,1,1)	Si(3,3,3)	22.73	413.39	1212.225	2.93	152.919	196.18	1.28
Si(1,1,1)	Si(0,4,4)	24.88	379.27	1391.795	3.67	1228.64	270.19	0.22
Si(1,1,1)	Si(1,3,5)	26.1	367.33	827.711	2.25	254.137	119.52	0.47
Si(1,1,1)	Si(0,2,6)	28.06	337.4	985.827	2.92	1243.13	156.93	0.13
Si(1,1,1)	Si(3,3,5)	29.19	323.96	591.757	1.83	399.153	84.74	0.21
Si(1,1,1)	Si(4,4,4)	31.02	303.73	727.878	2.4	893.604	109.42	0.12
Si(1,1,1)	Si(1,1,7)	32.08	291.4	440.78	1.51	346.233	59.16	0.17
Si(1,1,1)	Si(2,4,6)	33.82	273.02	552.372	2.02	340.75	76.86	0.23
Si(1,1,1)	Si(1,3,7)	34.84	266.22	336.973	1.27	306.298	40.07	0.13
Si(1,1,1)	Si(0,0,8)	36.51	248.42	429.416	1.73	302.132	56.8	0.19
Si(1,1,1)	Si(3,3,7)	37.5	243.6	262.308	1.08	272.144	34.36	0.13
Si(1,1,1)	Si(0,6,6)	39.13	229.07	341.081	1.49	268.694	45.61	0.17
Si(1,1,1)	Si(5,5,5)	40.1	223.48	207.695	0.93	245.17	24.59	0.1
Si(1,1,1)	Si(1,5,7)	40.1	223.47	207.695	0.93	245.168	24.59	0.1
Si(1,1,1)	Si(0,4,8)	41.7	210.12	274.811	1.31	241.182	36.57	0.15
Si(1,1,1)	Si(1,1,9)	42.66	204.7	166.403	0.81	223.391	20.51	0.09
Si(1,1,1)	Si(4,6,6)	44.24	193.26	224.335	1.16	217.406	28.08	0.13
Si(1,1,1)	Si(1,3,9)	45.19	187.08	135.008	0.72	203.055	13.69	0.07
Si(1,1,1)	Si(4,4,8)	46.78	176.05	184.352	1.05	196.613	21.76	0.11
Si(1,1,1)	Si(1,7,7)	47.73	171.29	111.715	0.65	184.743	11.22	0.06
Si(1,1,1)	Si(5,5,7)	47.73	171.29	111.716	0.65	184.742	11.22	0.06
Si(1,1,1)	Si(0,2,10)	49.33	161.85	153.991	0.95	179.181	17.85	0.1
Si(1,1,1)	Si(1,5,9)	50.3	155.59	92.545	0.59	168.057	10.45	0.06
Si(1,1,1)	Si(3,5,9)	52.9	141.78	77.655	0.55	152.57	6.46	0.04
Si(1,1,1)	Si(2,4,10)	54.56	133.58	109.376	0.82	147.04	12.76	0.09
Si(1,1,1)	Si(1,1,11)	55.58	128.13	65.261	0.51	137.953	6.42	0.05
Si(1,1,1)	Si(0,8,8)	57.3	120.57	93.283	0.77	132.237	9.43	0.07
Si(1,1,1)	Si(1,3,11)	58.35	115.41	54.851	0.48	344.861	5.37	0.02
Si(1,1,1)	Si(0,6,10)	60.15	107.9	79.95	0.74	117.974	7.95	0.07
Si(1,1,1)	Si(3,3,11)	61.27	102.83	47.176	0.46	306.556	4.41	0.01
Si(1,1,1)	Si(3,7,9)	61.27	102.83	47.176	0.46	306.557	4.41	0.01
Si(1,1,1)	Si(0,0,12)	63.19	95.86	68.94	0.72	103.948	6.66	0.06
Si(1,1,1)	Si(1,5,11)	64.39	89.89	40.484	0.45	268.183	3.65	0.01
Si(1,1,1)	Si(2,2,12)	66.49	81.8	59.945	0.73	89.799	5.56	0.06
Si(1,1,1)	Si(3,5,11)	67.82	75.95	35	0.46	228.64	2.98	0.01
Si(1,1,1)	Si(0,4,12)	70.18	68.13	52.341	0.77	75.024	5.36	0.07
Si(1,1,1)	Si(1,9,9)	71.73	61.73	30.265	0.49	158.772	2.3	0.01
Si(1,1,1)	Si(2,8,10)	74.58	52.05	45.939	0.88	161.763	4.53	0.03
Si(1,1,1)	Si(1,1,13)	76.55	44.98	26.371	0.59	49.418	2.18	0.04
Si(1,1,1)	Si(3,9,9)	76.55	44.98	26.371	0.59	49.418	2.18	0.04
Si(1,1,1)	Si(4,4,12)	80.65	31.17	40.192	1.29	19.851	3.72	0.19
Si(1,1,1)	Si(1,3,13)	84.31	18.87	23.042	1.22	12.155	1.84	0.15
Dia(1,1,1)	Si(1,1,1)	7.4	870.56	8303.739	9.54	4635.86	7640.49	1.65
Dia(1,1,1)	Si(0,2,2)	12.14	562.01	5164.292	9.19	521.376	4686.13	8.99

Dia(1,1,1)	Si(1,1,3)	14.28	425.55	2651.033	6.23	241.092	1367.05	5.67
Dia(1,1,1)	Si(0,0,4)	17.31	377.11	2547.826	6.76	215.346	1332.2	6.19
Dia(1,1,1)	Si(1,3,3)	18.92	338.33	1433.759	4.24	120.105	452.75	3.77
Dia(1,1,1)	Si(2,2,4)	21.37	312.49	1567.421	5.02	123.826	583.32	4.71
Dia(1,1,1)	Si(1,1,5)	22.73	292.81	929.43	3.17	152.779	203.11	1.33
Dia(1,1,1)	Si(3,3,3)	22.73	292.81	929.433	3.17	152.779	203.11	1.33
Dia(1,1,1)	Si(0,4,4)	24.88	272.83	1068.547	3.92	1447.98	271.43	0.19
Dia(1,1,1)	Si(1,3,5)	26.1	259.92	639.676	2.46	408.121	123.85	0.3
Dia(1,1,1)	Si(0,2,6)	28.06	243.87	767.814	3.15	394.14	162.66	0.41
Dia(1,1,1)	Si(3,3,5)	29.19	237.77	463.977	1.95	145.568	87.55	0.6
Dia(1,1,1)	Si(4,4,4)	31.02	222.23	571.33	2.57	1052.59	111.96	0.11
Dia(1,1,1)	Si(1,1,7)	32.08	217.91	349.207	1.6	313.133	60.35	0.19
Dia(1,1,1)	Si(2,4,6)	33.82	204.74	439.128	2.14	178.683	81.66	0.46
Dia(1,1,1)	Si(1,3,7)	34.84	200.5	270.804	1.35	871.695	45.78	0.05
Dia(1,1,1)	Si(0,0,8)	36.51	189.21	346.697	1.83	308.803	61	0.2
Dia(1,1,1)	Si(3,3,7)	37.5	182.15	210.962	1.16	149.565	34.79	0.23
Dia(1,1,1)	Si(0,6,6)	39.13	174.15	277.614	1.59	145.688	45.36	0.31
Dia(1,1,1)	Si(1,5,7)	40.1	166.52	167.706	1.01	135.502	26.26	0.19
Dia(1,1,1)	Si(5,5,5)	40.1	166.52	167.707	1.01	135.503	26.26	0.19
Dia(1,1,1)	Si(0,4,8)	41.7	160.61	225.305	1.4	132.188	37.08	0.28
Dia(1,1,1)	Si(1,1,9)	42.66	152.34	135.176	0.89	123.744	17.37	0.14
Dia(1,1,1)	Si(4,6,6)	44.24	147.54	184.122	1.25	118.782	29.55	0.25
Dia(1,1,1)	Si(1,3,9)	45.19	140.94	110.204	0.78	112.839	14.89	0.13
Dia(1,1,1)	Si(4,4,8)	46.78	135.83	152.793	1.12	108.066	22.84	0.21
Dia(1,1,1)	Si(1,7,7)	47.73	131.1	92.104	0.7	103.447	12.98	0.13
Dia(1,1,1)	Si(5,5,7)	47.73	131.1	92.104	0.7	103.447	12.98	0.13
Dia(1,1,1)	Si(0,2,10)	49.33	125.49	127.86	1.02	99.154	19.07	0.19
Dia(1,1,1)	Si(1,5,9)	50.3	121.51	77.228	0.64	62.649	11.11	0.18
Dia(1,1,1)	Si(3,5,9)	52.9	113.52	65.298	0.58	85.885	8.76	0.1
Dia(1,1,1)	Si(2,4,10)	54.56	106.88	92.149	0.86	81.564	11.54	0.14
Dia(1,1,1)	Si(1,1,11)	55.58	105.91	55.52	0.52	77.948	7.32	0.09
Dia(1,1,1)	Si(1,3,11)	58.35	98.31	47.662	0.48	70.293	5.84	0.08
Dia(1,1,1)	Si(0,8,8)	57.3	98.14	79.23	0.81	73.303	10.35	0.14
Dia(1,1,1)	Si(3,3,11)	61.27	89.59	41.045	0.46	62.842	4.74	0.08
Dia(1,1,1)	Si(3,7,9)	61.27	89.59	41.048	0.46	62.841	4.74	0.08
Dia(1,1,1)	Si(0,6,10)	60.15	89.33	68.168	0.76	65.428	8.34	0.13
Dia(1,1,1)	Si(0,0,12)	63.19	80.79	59.314	0.73	57.699	7.3	0.13
Dia(1,1,1)	Si(1,5,11)	64.39	79.35	35.303	0.44	54.923	3.94	0.07
Dia(1,1,1)	Si(2,2,12)	66.49	71.32	51.371	0.72	49.835	6.18	0.12
Dia(1,1,1)	Si(3,5,11)	67.82	67.8	30.606	0.45	46.685	3.35	0.07
Dia(1,1,1)	Si(0,4,12)	70.18	59.16	45.384	0.77	41.49	4.95	0.12
Dia(1,1,1)	Si(1,9,9)	71.73	55.41	26.591	0.48	37.738	2.85	0.08
Dia(1,1,1)	Si(2,8,10)	74.58	45.38	39.837	0.88	32.342	4.25	0.13
Dia(1,1,1)	Si(1,1,13)	76.55	40.32	23.164	0.57	27.385	2.26	0.08
Dia(1,1,1)	Si(3,9,9)	76.55	40.31	23.165	0.57	27.385	2.26	0.08
Dia(1,1,1)	Si(4,4,12)	80.65	26.68	35.082	1.31	19.612	3.95	0.2
Dia(1,1,1)	Si(1,3,13)	84.31	16.27	20.204	1.24	11.992	1.94	0.16

Ei = 14.698 keV			Cryst 1-2			Cryst 1-2-3		
Cryst 1	Cryst 2,3	ΘB 2 [°]	ΔE [meV]	∫IR dΘdE [µrad meV]	FOM	ΔE [meV]	∫IR dΘdE [µrad meV]	FOM
Si(1,1,1)	Si(1,1,1)	7.73	1327.48	13735.244	10.35	5943.85	12377.42	2.08
Si(1,1,1)	Si(0,2,2)	12.69	805.64	7541.26	9.36	517.057	4567.39	8.83
Si(1,1,1)	Si(1,1,3)	14.93	604.5	3668.584	6.07	234.923	1285.88	5.47
Si(1,1,1)	Si(0,0,4)	18.1	518.92	3431.12	6.61	208.058	1240.11	5.96
Si(1,1,1)	Si(1,3,3)	19.79	465.63	1901.191	4.08	115.482	429.27	3.72
Si(1,1,1)	Si(2,2,4)	22.36	416.26	2040.664	4.9	117.807	548.92	4.66
Si(1,1,1)	Si(1,1,5)	23.8	391.66	1183.693	3.02	75.796	188.26	2.48
Si(1,1,1)	Si(0,4,4)	26.06	354.95	1355.793	3.82	1369.08	275.07	0.2
Si(1,1,1)	Si(1,3,5)	27.35	341.37	804.444	2.36	234.466	115.56	0.49
Si(1,1,1)	Si(0,2,6)	29.42	314.7	960.394	3.05	1140.16	151.39	0.13
Si(1,1,1)	Si(3,3,5)	30.61	299.52	574.016	1.92	368.584	80.14	0.22
Si(1,1,1)	Si(4,4,4)	32.55	280.37	708.144	2.53	983.67	104.41	0.11
Si(1,1,1)	Si(1,1,7)	33.68	269.27	426.935	1.59	318.742	55.46	0.17
Si(1,1,1)	Si(1,5,5)	33.68	269.27	426.932	1.59	318.742	55.46	0.17
Si(1,1,1)	Si(2,4,6)	35.53	250.06	536.506	2.15	314.793	72.78	0.23
Si(1,1,1)	Si(1,3,7)	36.62	244.93	325.996	1.33	280.113	42.06	0.15
Si(1,1,1)	Si(3,5,5)	36.62	244.93	325.996	1.33	280.114	42.06	0.15
Si(1,1,1)	Si(0,0,8)	38.41	227.55	417.702	1.84	277.376	57.11	0.21
Si(1,1,1)	Si(3,3,7)	39.47	220.27	253.262	1.15	247.817	31.68	0.13
Si(1,1,1)	Si(0,6,6)	41.22	207.93	331.784	1.6	244.507	42.72	0.17
Si(1,1,1)	Si(1,5,7)	42.26	200.87	200.682	1	220.803	22.72	0.1
Si(1,1,1)	Si(0,4,8)	44	190.24	266.194	1.4	217.79	33.23	0.15
Si(1,1,1)	Si(1,1,9)	45.03	182.69	160.749	0.88	199.908	19.7	0.1
Si(1,1,1)	Si(4,6,6)	46.76	172.15	216.882	1.26	195.135	25.53	0.13
Si(1,1,1)	Si(1,3,9)	47.8	165.18	130.509	0.79	180.625	12.99	0.07
Si(1,1,1)	Si(4,4,8)	49.54	155.52	178.798	1.15	174.725	22.28	0.13
Si(1,1,1)	Si(1,7,7)	50.6	150.13	107.431	0.72	162.854	10.42	0.06
Si(1,1,1)	Si(5,5,7)	50.6	150.13	107.431	0.72	162.853	10.42	0.06
Si(1,1,1)	Si(0,2,10)	52.37	141.37	148.975	1.05	156.799	15.9	0.1
Si(1,1,1)	Si(1,5,9)	53.45	135.21	88.899	0.66	146.404	8.11	0.06
Si(1,1,1)	Si(3,5,9)	56.39	121.28	74.266	0.61	130.932	7.89	0.06
Si(1,1,1)	Si(2,4,10)	58.29	112.73	105.553	0.94	125.248	11.36	0.09
Si(1,1,1)	Si(1,1,11)	59.46	107.39	62.357	0.58	116.053	5.91	0.05
Si(1,1,1)	Si(0,8,8)	61.48	98.97	89.743	0.91	110.157	10.02	0.09
Si(1,1,1)	Si(1,3,11)	62.73	94.08	52.84	0.56	101.413	4.69	0.05
Si(1,1,1)	Si(0,6,10)	64.91	85.35	77.028	0.9	263.536	8.32	0.03
Si(1,1,1)	Si(3,3,11)	66.29	79.65	44.946	0.56	86.6	4.05	0.05
Si(1,1,1)	Si(0,0,12)	68.74	70.99	66.433	0.94	209.294	6.6	0.03
Si(1,1,1)	Si(1,5,11)	70.32	65.54	38.362	0.59	71.02	3.68	0.05
Si(1,1,1)	Si(2,2,12)	73.23	55.15	57.443	1.04	172.344	5.34	0.03
Si(1,1,1)	Si(3,5,11)	75.21	48.34	33.126	0.69	53.507	2.93	0.05
Si(1,1,1)	Si(5,7,9)	75.21	48.34	33.126	0.69	53.507	2.93	0.05
Si(1,1,1)	Si(0,4,12)	79.21	35.39	49.945	1.41	22.429	4.87	0.22
Si(1,1,1)	Si(1,9,9)	82.52	24.13	28.629	1.19	15.439	2.29	0.15
Dia(1,1,1)	Si(1,1,1)	7.73	835.88	8174.534	9.78	4383.68	7470.05	1.7
Dia(1,1,1)	Si(0,2,2)	12.69	540.88	5117.479	9.46	500.428	4664.97	9.32
Dia(1,1,1)	Si(1,1,3)	14.93	407.77	2637.325	6.47	237.316	1363.06	5.74
Dia(1,1,1)	Si(0,0,4)	18.1	361.33	2520.249	6.97	206.031	1308.71	6.35
Dia(1,1,1)	Si(1,3,3)	19.79	323.74	1420.317	4.39	114.307	448.62	3.92
Dia(1,1,1)	Si(2,2,4)	22.36	297.65	1550.536	5.21	118.329	576.22	4.87
Dia(1,1,1)	Si(1,1,5)	23.8	277.94	915.406	3.29	75.834	196.15	2.59

Dia(1,1,1)	Si(0,4,4)	26.06	258.66	1054.15	4.08	77.985	280.39	3.6
Dia(1,1,1)	Si(1,3,5)	27.35	246.44	628.153	2.55	376.599	123.21	0.33
Dia(1,1,1)	Si(0,2,6)	29.42	230.23	758.289	3.29	361.098	157.63	0.44
Dia(1,1,1)	Si(3,3,5)	30.61	224.13	456.425	2.04	366.091	83.58	0.23
Dia(1,1,1)	Si(4,4,4)	32.55	208.73	561.086	2.69	969.742	107.35	0.11
Dia(1,1,1)	Si(1,1,7)	33.68	204.26	344.435	1.69	294.127	58.2	0.2
Dia(1,1,1)	Si(1,5,5)	33.68	204.26	344.435	1.69	294.131	58.2	0.2
Dia(1,1,1)	Si(2,4,6)	35.53	191.89	435.909	2.27	863.74	77.58	0.09
Dia(1,1,1)	Si(1,3,7)	36.62	185.68	265.521	1.43	797.69	42.91	0.05
Dia(1,1,1)	Si(3,5,5)	36.62	185.68	265.524	1.43	797.691	42.91	0.05
Dia(1,1,1)	Si(0,0,8)	38.41	176.06	343.131	1.95	644.062	58.69	0.09
Dia(1,1,1)	Si(3,3,7)	39.47	168.49	207.472	1.23	136.195	33.51	0.25
Dia(1,1,1)	Si(0,6,6)	41.22	161.8	275.467	1.7	132.943	44.24	0.33
Dia(1,1,1)	Si(1,5,7)	42.26	153.86	165.282	1.07	122.506	24.27	0.2
Dia(1,1,1)	Si(0,4,8)	44	147.39	222.154	1.51	119.41	34.59	0.29
Dia(1,1,1)	Si(1,1,9)	45.03	141.76	133.987	0.95	111.713	19.94	0.18
Dia(1,1,1)	Si(4,6,6)	46.76	135.11	181.362	1.34	107.528	29.14	0.27
Dia(1,1,1)	Si(1,3,9)	47.8	130.39	109.642	0.84	101.109	14.18	0.14
Dia(1,1,1)	Si(4,4,8)	49.54	124.88	149.979	1.2	96.471	22.85	0.24
Dia(1,1,1)	Si(1,7,7)	50.6	121.93	91.119	0.75	91.218	11.15	0.12
Dia(1,1,1)	Si(5,5,7)	50.6	121.93	91.12	0.75	91.218	11.15	0.12
Dia(1,1,1)	Si(0,2,10)	52.37	114.11	126.278	1.11	86.978	17.39	0.2
Dia(1,1,1)	Si(1,5,9)	53.45	112.54	75.86	0.67	414.725	9.64	0.02
Dia(1,1,1)	Si(3,5,9)	56.39	104.13	64.013	0.61	80.914	8.18	0.1
Dia(1,1,1)	Si(2,4,10)	58.29	95.09	90.619	0.95	69.808	12.1	0.17
Dia(1,1,1)	Si(1,1,11)	59.46	94.2	54.224	0.58	277.965	6.6	0.02
Dia(1,1,1)	Si(0,8,8)	61.48	85.63	77.689	0.91	61.508	10.63	0.17
Dia(1,1,1)	Si(1,3,11)	62.73	83.44	46.16	0.55	57.296	5.36	0.09
Dia(1,1,1)	Si(0,6,10)	64.91	74.99	66.934	0.89	53.038	7.96	0.15
Dia(1,1,1)	Si(3,3,11)	66.29	71.34	39.355	0.55	48.777	4.4	0.09
Dia(1,1,1)	Si(0,0,12)	68.74	62.78	57.855	0.92	44.277	6.6	0.15
Dia(1,1,1)	Si(1,5,11)	70.32	58.77	33.689	0.57	39.692	3.6	0.09
Dia(1,1,1)	Si(2,2,12)	73.23	48.99	50.21	1.02	34.691	5.75	0.17
Dia(1,1,1)	Si(3,5,11)	75.21	43.7	29.282	0.67	29.611	2.43	0.08
Dia(1,1,1)	Si(5,7,9)	75.21	43.7	29.282	0.67	29.611	2.43	0.08
Dia(1,1,1)	Si(0,4,12)	79.21	30.72	44.188	1.44	22.204	4.99	0.22
Dia(1,1,1)	Si(1,9,9)	82.52	21.45	25.447	1.19	15.266	2.47	0.16

Ei = 12.658 keV			Cryst 1-2			Cryst 1-2-3		
Cryst 1	Cryst 2,3	ΘB 2 [°]	ΔE [meV]	∫IR dΘdE [μrad meV]	FOM	ΔE [meV]	∫IR dΘdE [μrad meV]	FOM
Si(1,1,1)	Si(1,1,1)	8.99	1174.75	12647.006	10.77	4862.91	11094.75	2.28
Si(1,1,1)	Si(0,2,2)	14.78	696.08	6965.16	10.01	459.72	4231.46	9.2
Si(1,1,1)	Si(1,1,3)	17.4	504.96	3370.578	6.67	204.641	1175.47	5.74
Si(1,1,1)	Si(0,0,4)	21.14	427.93	3148.9	7.36	179.132	1157.33	6.46
Si(1,1,1)	Si(1,3,3)	23.15	374.53	1720.014	4.59	98.623	400.86	4.06
Si(1,1,1)	Si(2,2,4)	26.22	332.73	1859.614	5.59	101.908	525.09	5.15
Si(1,1,1)	Si(1,1,5)	27.94	308.15	1065.266	3.46	61.759	173.94	2.82
Si(1,1,1)	Si(0,4,4)	30.67	278.12	1232.641	4.43	67.53	271.94	4.03
Si(1,1,1)	Si(1,3,5)	32.24	260.35	719.622	2.76	179.563	98.16	0.55
Si(1,1,1)	Si(0,2,6)	34.77	239.53	870.319	3.63	47.814	143.51	3
Si(1,1,1)	Si(3,3,5)	36.25	225.06	512.172	2.28	149.263	63.94	0.43
Si(1,1,1)	Si(4,4,4)	38.66	207.4	638.051	3.08	37.753	90.7	2.4
Si(1,1,1)	Si(1,1,7)	40.09	196.43	377.221	1.92	263.438	46.87	0.18
Si(1,1,1)	Si(1,5,5)	40.09	196.43	377.221	1.92	263.433	46.87	0.18
Si(1,1,1)	Si(2,4,6)	42.44	182.01	482.286	2.65	122.369	61.61	0.5
Si(1,1,1)	Si(1,3,7)	43.84	172.28	286.033	1.66	556.155	32.54	0.06
Si(1,1,1)	Si(0,0,8)	46.17	159.46	373.255	2.34	544.153	45.64	0.08
Si(1,1,1)	Si(3,3,7)	47.57	150.79	221.081	1.47	195.348	23.81	0.12
Si(1,1,1)	Si(0,6,6)	49.92	139.62	293.696	2.1	171.619	36.76	0.21
Si(1,1,1)	Si(1,5,7)	51.35	132.34	173.731	1.31	150.008	17.3	0.12
Si(1,1,1)	Si(0,4,8)	53.76	121.5	234.413	1.93	147.177	28.14	0.19
Si(1,1,1)	Si(1,1,9)	55.24	114.31	138.002	1.21	129.166	13.69	0.11
Si(1,1,1)	Si(4,6,6)	57.77	104.28	190.165	1.82	125.407	20.56	0.16
Si(1,1,1)	Si(1,3,9)	59.34	97.65	111.914	1.15	110.082	10.51	0.1
Si(1,1,1)	Si(4,4,8)	62.07	87.79	156.332	1.78	105.518	16.34	0.15
Si(1,1,1)	Si(5,5,7)	63.8	81.15	91.216	1.12	91.812	8.07	0.09
Si(1,1,1)	Si(1,7,7)	63.8	81.14	91.217	1.12	91.812	8.07	0.09
Si(1,1,1)	Si(0,2,10)	66.87	70.88	129.061	1.82	86.011	13.88	0.16
Si(1,1,1)	Si(1,5,9)	68.87	63.87	74.799	1.17	73.354	6.43	0.09
Si(1,1,1)	Si(3,5,9)	75.25	43.86	62.226	1.42	52.348	5.19	0.1
Si(1,1,1)	Si(2,4,10)	81.05	26.92	91.302	3.39	5.858	13.25	2.26
Dia(1,1,1)	Si(1,1,1)	8.99	725.69	7648.913	10.54	4973.57	6952.86	1.4
Dia(1,1,1)	Si(0,2,2)	14.78	475.24	4929.549	10.37	440.793	4393.31	9.97
Dia(1,1,1)	Si(1,1,3)	17.4	349.15	2516.162	7.21	204.317	1251.19	6.12
Dia(1,1,1)	Si(0,0,4)	21.14	311.27	2419.176	7.77	179.18	1226.28	6.84
Dia(1,1,1)	Si(1,3,3)	23.15	273.5	1349.219	4.93	99.016	434.64	4.39
Dia(1,1,1)	Si(2,2,4)	26.22	249.75	1491.204	5.97	103.674	554.58	5.35
Dia(1,1,1)	Si(1,1,5)	27.94	230.54	863.239	3.74	61.167	182.16	2.98
Dia(1,1,1)	Si(0,4,4)	30.67	212.76	1018.512	4.79	66.805	281.1	4.21
Dia(1,1,1)	Si(1,3,5)	32.24	202.06	597.034	2.95	294.001	101.54	0.35
Dia(1,1,1)	Si(0,2,6)	34.77	186.5	729.519	3.91	851.293	155.25	0.18
Dia(1,1,1)	Si(3,3,5)	36.25	179.68	434.474	2.42	251.739	68.43	0.27
Dia(1,1,1)	Si(4,4,4)	38.66	166.71	543.589	3.26	265.499	96.45	0.36
Dia(1,1,1)	Si(1,1,7)	40.09	159.64	324.834	2.03	90.771	47.76	0.53
Dia(1,1,1)	Si(1,5,5)	40.09	159.64	324.835	2.03	90.77	47.76	0.53
Dia(1,1,1)	Si(2,4,6)	42.44	149.29	416.931	2.79	631.429	66.89	0.11
Dia(1,1,1)	Si(1,3,7)	43.84	142.55	247.934	1.74	208.049	33.77	0.16
Dia(1,1,1)	Si(0,0,8)	46.17	133.34	326	2.44	547.158	49.27	0.09
Dia(1,1,1)	Si(3,3,7)	47.57	128.43	192.82	1.5	498.097	25.63	0.05
Dia(1,1,1)	Si(0,6,6)	49.92	118.62	258.071	2.18	474.802	38.35	0.08
Dia(1,1,1)	Si(1,5,7)	51.35	116.12	152.875	1.32	83.967	19.58	0.23

**Dia(1,1,1)**	**Si(0,4,8)**	**53.76**	**105.05**	**207.919**	**1.98**	**78.289**	**28.78**	**0.37**
**Dia(1,1,1)**	**Si(1,1,9)**	**55.24**	**103.02**	**122.876**	**1.19**	**72.499**	**13.89**	**0.19**
**Dia(1,1,1)**	**Si(4,6,6)**	**57.77**	**92.5**	**169.958**	**1.84**	**67.165**	**22.66**	**0.34**
**Dia(1,1,1)**	**Si(1,3,9)**	**59.34**	**89.34**	**100.062**	**1.12**	**61.777**	**11.1**	**0.18**
**Dia(1,1,1)**	**Si(4,4,8)**	**62.07**	**78.72**	**140.634**	**1.79**	**56.579**	**17.98**	**0.32**
**Dia(1,1,1)**	**Si(1,7,7)**	**63.8**	**75.03**	**82.462**	**1.1**	**51.325**	**8.78**	**0.17**
**Dia(1,1,1)**	**Si(5,5,7)**	**63.8**	**75.03**	**82.461**	**1.1**	**51.325**	**8.78**	**0.17**
**Dia(1,1,1)**	**Si(0,2,10)**	**66.87**	**64.59**	**117.838**	**1.82**	**45.786**	**14.08**	**0.31**
**Dia(1,1,1)**	**Si(1,5,9)**	**68.87**	**59.58**	**68.541**	**1.15**	**40.44**	**6.95**	**0.17**
**Dia(1,1,1)**	**Si(3,5,9)**	**75.25**	**40.99**	**57.531**	**1.4**	**28.004**	**5.65**	**0.2**
**Dia(1,1,1)**	**Si(2,4,10)**	**81.05**	**24.06**	**84.798**	**3.52**	**5.866**	**14**	**2.39**

Ei = 15.861 keV			Cryst 1-2			Cryst 1-2-3		
Cryst 1	Cryst 2,3	ΘB 2 [°]	ΔE [meV]	∫IR dΘdE [µrad meV]	FOM	ΔE [meV]	∫IR dΘdE [µrad meV]	FOM
Si(1,1,1)	Si(1,1,1)	7.16	1410.54	14237.754	10.09	1340.68	12962.19	9.67
Si(1,1,1)	Si(0,2,2)	11.74	868.2	7814.179	9	568.111	4665.01	8.21
Si(1,1,1)	Si(1,1,3)	13.81	666.15	3828.084	5.75	248.108	1273.34	5.13
Si(1,1,1)	Si(0,0,4)	16.73	571.12	3556.006	6.23	214.083	1224.21	5.72
Si(1,1,1)	Si(1,3,3)	18.28	516.92	1977.357	3.83	122.237	442	3.62
Si(1,1,1)	Si(2,2,4)	20.64	463.65	2122.08	4.58	125.519	541.55	4.31
Si(1,1,1)	Si(1,1,5)	21.96	436.18	1233.52	2.83	75.794	200.34	2.64
Si(1,1,1)	Si(0,4,4)	24.02	397.84	1416.018	3.56	84.152	286.5	3.4
Si(1,1,1)	Si(1,3,5)	25.2	387.75	845.247	2.18	54.358	97.4	1.79
Si(1,1,1)	Si(0,2,6)	27.07	355.03	1004.954	2.83	58.832	157.76	2.68
Si(1,1,1)	Si(3,3,5)	28.16	342.94	605.802	1.77	175.753	67.11	0.38
Si(1,1,1)	Si(4,4,4)	29.91	322.14	743.013	2.31	45.119	91.7	2.03
Si(1,1,1)	Si(1,1,7)	30.93	308.56	450.391	1.46	154.794	46.79	0.3
Si(1,1,1)	Si(2,4,6)	32.58	290.7	564.288	1.94	149.458	61.5	0.41
Si(1,1,1)	Si(1,3,7)	33.56	281.71	345.391	1.23	138.426	34.8	0.25
Si(1,1,1)	Si(3,5,5)	33.56	281.71	345.392	1.23	138.426	34.8	0.25
Si(1,1,1)	Si(0,0,8)	35.15	265.22	439.133	1.66	132.962	44.69	0.34
Si(1,1,1)	Si(3,3,7)	36.09	260.8	269.758	1.03	125.58	25.42	0.2
Si(1,1,1)	Si(0,6,6)	37.64	245.05	349.029	1.42	119.893	35.93	0.3
Si(1,1,1)	Si(1,5,7)	38.55	240.25	213.433	0.89	114.211	19.61	0.17
Si(1,1,1)	Si(5,5,5)	38.55	240.25	213.435	0.89	114.21	19.61	0.17
Si(1,1,1)	Si(0,4,8)	40.07	226.97	281.329	1.24	109.24	25.65	0.23
Si(1,1,1)	Si(1,1,9)	40.97	222.19	171.199	0.77	103.511	15.47	0.15
Si(1,1,1)	Si(4,6,6)	42.46	209.72	229.578	1.09	99.609	22.96	0.23
Si(1,1,1)	Si(1,3,9)	43.35	205.44	139.141	0.68	95.131	11.1	0.12
Si(1,1,1)	Si(4,4,8)	44.84	193.77	189.273	0.98	91.774	16.14	0.18
Si(1,1,1)	Si(1,7,7)	45.73	186.36	114.388	0.61	87.167	9	0.1
Si(1,1,1)	Si(5,5,7)	45.73	186.36	114.39	0.61	87.168	9.01	0.1
Si(1,1,1)	Si(0,2,10)	47.21	178.11	157.386	0.88	84.147	14.35	0.17
Si(1,1,1)	Si(1,5,9)	48.11	172.28	95.084	0.55	79.557	7.14	0.09
Si(1,1,1)	Si(3,5,9)	50.51	158.47	79.546	0.5	72.853	6.29	0.09
Si(1,1,1)	Si(2,4,10)	52.03	150.38	111.582	0.74	70.435	9.14	0.13
Si(1,1,1)	Si(1,1,11)	52.95	145.62	67.07	0.46	66.803	5.33	0.08
Si(1,1,1)	Si(0,8,8)	54.51	137.88	95.406	0.69	64.184	7.52	0.12
Si(1,1,1)	Si(1,3,11)	55.46	134.02	56.964	0.43	60.994	4.48	0.07
Si(1,1,1)	Si(0,6,10)	57.06	125.02	82.213	0.66	58.174	6.02	0.1
Si(1,1,1)	Si(3,3,11)	58.04	119.46	48.961	0.41	55.337	3.74	0.07
Si(1,1,1)	Si(0,0,12)	59.72	112.41	71.286	0.63	52.282	5.08	0.1
Si(1,1,1)	Si(1,5,11)	60.75	105.47	41.963	0.4	15.281	3.13	0.2
Si(1,1,1)	Si(7,7,7)	60.75	105.47	41.964	0.4	15.286	3.13	0.2
Si(1,1,1)	Si(2,2,12)	62.53	100.12	62.054	0.62	46.481	4.33	0.09
Si(1,1,1)	Si(3,5,11)	63.63	93.72	36.325	0.39	13.597	2.61	0.19
Si(1,1,1)	Si(0,4,12)	65.55	87.7	53.915	0.61	40.715	3.69	0.09
Si(1,1,1)	Si(1,9,9)	66.75	83.28	31.625	0.38	11.651	2.17	0.19
Si(1,1,1)	Si(2,8,10)	68.87	74.62	47.373	0.63	34.782	3.07	0.09
Si(1,1,1)	Si(1,1,13)	70.23	69.12	27.386	0.4	63.086	1.77	0.03
Si(1,1,1)	Si(4,4,12)	72.69	59.63	41.713	0.7	28.318	2.92	0.1
Si(1,1,1)	Si(1,3,13)	74.33	53.41	24.03	0.45	44.172	1.23	0.03
Si(1,1,1)	Si(3,7,11)	74.33	53.4	24.03	0.45	44.172	1.23	0.03
Si(1,1,1)	Si(2,6,12)	77.47	42.87	36.867	0.86	99.784	2.66	0.03
Si(1,1,1)	Si(3,3,13)	79.77	34.83	21.124	0.61	79.221	1.34	0.02
Si(1,1,1)	Si(8,8,8)	85.7	14.66	32.696	2.23	2.423	3.52	1.45

Dia(1,1,1)	Si(1,1,1)	7.16	898.28	8403.449	9.36	5639.39	7950.58	1.41
Dia(1,1,1)	Si(0,2,2)	11.74	578.29	5188.973	8.97	549.368	4650.81	8.47
Dia(1,1,1)	Si(1,1,3)	13.81	440.61	2654.971	6.03	245.904	1313.5	5.34
Dia(1,1,1)	Si(0,0,4)	16.73	390.53	2564.961	6.57	215.666	1301.01	6.03
Dia(1,1,1)	Si(1,3,3)	18.28	351.67	1448.852	4.12	122.587	462.56	3.77
Dia(1,1,1)	Si(2,2,4)	20.64	323.94	1580.927	4.88	126.065	564.38	4.48
Dia(1,1,1)	Si(1,1,5)	21.96	304.35	939.267	3.09	76.345	209.12	2.74
Dia(1,1,1)	Si(0,4,4)	24.02	284.3	1077.671	3.79	83.439	297.42	3.56
Dia(1,1,1)	Si(1,3,5)	25.2	269.82	648.227	2.4	54.581	101.78	1.86
Dia(1,1,1)	Si(0,2,6)	27.07	254.5	779.015	3.06	59.897	166.87	2.79
Dia(1,1,1)	Si(3,3,5)	28.16	247.6	470.27	1.9	174.93	63.57	0.36
Dia(1,1,1)	Si(4,4,4)	29.91	232.15	578.671	2.49	45.1	92.9	2.06
Dia(1,1,1)	Si(1,1,7)	30.93	229.04	354.383	1.55	153.952	46.85	0.3
Dia(1,1,1)	Si(2,4,6)	32.58	213.52	442.658	2.07	148.228	65.13	0.44
Dia(1,1,1)	Si(1,3,7)	33.56	211.75	274.21	1.29	246.208	36.07	0.15
Dia(1,1,1)	Si(3,5,5)	33.56	211.75	274.211	1.3	246.208	36.07	0.15
Dia(1,1,1)	Si(0,0,8)	35.15	199.56	349.929	1.75	660.962	48.22	0.07
Dia(1,1,1)	Si(3,3,7)	36.09	194.94	216.125	1.11	222.154	26.4	0.12
Dia(1,1,1)	Si(0,6,6)	37.64	185.13	280.664	1.52	215.296	36.25	0.17
Dia(1,1,1)	Si(1,5,7)	38.55	177.93	171.266	0.96	201.614	20.86	0.1
Dia(1,1,1)	Si(5,5,5)	38.55	177.93	171.266	0.96	201.614	20.86	0.1
Dia(1,1,1)	Si(0,4,8)	40.07	171.26	227.317	1.33	195.29	29.27	0.15
Dia(1,1,1)	Si(1,1,9)	40.97	163.16	137.435	0.84	183.766	15.67	0.09
Dia(1,1,1)	Si(4,6,6)	42.46	157.44	185.713	1.18	493.242	24.2	0.05
Dia(1,1,1)	Si(1,3,9)	43.35	150.13	111.797	0.74	463.27	11.85	0.03
Dia(1,1,1)	Si(4,4,8)	44.84	145.5	153.816	1.06	91.477	18.19	0.2
Dia(1,1,1)	Si(1,7,7)	45.73	138.56	92.308	0.67	86.598	10.47	0.12
Dia(1,1,1)	Si(5,5,7)	45.73	138.55	92.306	0.67	86.596	10.47	0.12
Dia(1,1,1)	Si(0,2,10)	47.21	134.44	128.94	0.96	83.714	15.23	0.18
Dia(1,1,1)	Si(1,5,9)	48.11	129.11	77.736	0.6	79.417	8.81	0.11
Dia(1,1,1)	Si(3,5,9)	50.51	120.55	65.637	0.54	72.752	7.22	0.1
Dia(1,1,1)	Si(2,4,10)	52.03	115.58	92.737	0.8	69.802	9.41	0.13
Dia(1,1,1)	Si(1,1,11)	52.95	111.25	55.841	0.5	66.712	4.7	0.07
Dia(1,1,1)	Si(0,8,8)	54.51	107.05	79.815	0.75	63.818	7.85	0.12
Dia(1,1,1)	Si(1,3,11)	55.46	104.66	48.003	0.46	60.871	3.66	0.06
Dia(1,1,1)	Si(0,6,10)	57.06	98.83	68.566	0.69	57.932	6.72	0.12
Dia(1,1,1)	Si(3,3,11)	58.04	97.82	41.275	0.42	55.192	4.29	0.08
Dia(1,1,1)	Si(0,0,12)	59.72	91.24	60.387	0.66	52.178	6.05	0.12
Dia(1,1,1)	Si(1,5,11)	60.75	90.67	36.156	0.4	49.561	3.49	0.07
Dia(1,1,1)	Si(7,7,7)	60.75	90.67	36.155	0.4	49.56	3.49	0.07
Dia(1,1,1)	Si(2,2,12)	62.53	82.69	52.741	0.64	46.439	5.14	0.11
Dia(1,1,1)	Si(3,5,11)	63.63	82.2	31.397	0.38	43.906	2.88	0.07
Dia(1,1,1)	Si(0,4,12)	65.55	73.21	46.178	0.63	40.674	4.55	0.11
Dia(1,1,1)	Si(1,9,9)	66.75	72.34	27.34	0.38	38.057	2.43	0.06
Dia(1,1,1)	Si(2,8,10)	68.87	63.39	40.786	0.64	34.741	3.64	0.1
Dia(1,1,1)	Si(1,1,13)	70.23	60.94	23.898	0.39	31.748	2.08	0.07
Dia(1,1,1)	Si(4,4,12)	72.69	51.7	36.037	0.7	28.303	3.09	0.11
Dia(1,1,1)	Si(1,3,13)	74.33	47.69	20.976	0.44	24.845	1.64	0.07
Dia(1,1,1)	Si(3,7,11)	74.33	47.69	20.976	0.44	24.845	1.64	0.07
Dia(1,1,1)	Si(2,6,12)	77.47	36.81	31.828	0.86	20.176	2.61	0.13
Dia(1,1,1)	Si(3,3,13)	79.77	30.71	18.394	0.6	16.381	1.15	0.07
Dia(1,1,1)	Si(8,8,8)	85.7	12.31	28.56	2.32	2.432	3.63	1.49

Ei = 15.2 keV				Cryst 1-2			Cryst 1-2-3	
Cryst 1	Cryst 2,3	ΘB 2 [°]	ΔE [meV]	∫IR dΘdE [µrad meV]	FOM	ΔE [meV]	∫IR dΘdE [µrad meV]	FOM
Si(1,1,1)	Si(1,1,1)	7.47	1363.11	13974.198	10.25	1284.33	12742.96	9.92
Si(1,1,1)	Si(0,2,2)	12.26	832.58	7663.819	9.2	544.374	4587.93	8.43
Si(1,1,1)	Si(1,1,3)	14.42	632.82	3742.031	5.91	237.934	1258.42	5.29
Si(1,1,1)	Si(0,0,4)	17.48	541.46	3487.633	6.44	211.826	1260.35	5.95
Si(1,1,1)	Si(1,3,3)	19.11	488.07	1937.049	3.97	118.659	436.72	3.68
Si(1,1,1)	Si(2,2,4)	21.59	436.67	2076.98	4.76	120.696	532.21	4.41
Si(1,1,1)	Si(1,1,5)	22.97	410.96	1208.455	2.94	72.65	198.91	2.74
Si(1,1,1)	Si(0,4,4)	25.14	373.68	1384.609	3.71	80.281	281.38	3.5
Si(1,1,1)	Si(1,3,5)	26.38	361.69	823.368	2.28	50.999	96.6	1.89
Si(1,1,1)	Si(0,2,6)	28.36	332.7	979.942	2.95	56.148	157.08	2.8
Si(1,1,1)	Si(3,3,5)	29.5	318.52	587.781	1.85	162.743	64.02	0.39
Si(1,1,1)	Si(4,4,4)	31.35	298.15	722.489	2.42	42.264	90.27	2.14
Si(1,1,1)	Si(1,1,7)	32.43	286.42	438.026	1.53	142.314	44.07	0.31
Si(1,1,1)	Si(2,4,6)	34.19	268.11	549.076	2.05	67.128	58.76	0.88
Si(1,1,1)	Si(1,3,7)	35.23	262.05	334.573	1.28	126.498	31	0.25
Si(1,1,1)	Si(3,5,5)	35.23	262.05	334.573	1.28	126.499	31	0.25
Si(1,1,1)	Si(0,0,8)	36.92	244.14	426.88	1.75	121.873	42.11	0.35
Si(1,1,1)	Si(3,3,7)	37.93	238.74	260.425	1.09	113.965	25.53	0.22
Si(1,1,1)	Si(0,6,6)	39.58	224.33	339.193	1.51	109.104	33.95	0.31
Si(1,1,1)	Si(1,5,7)	40.57	218.57	206.23	0.94	103.499	18.14	0.18
Si(1,1,1)	Si(0,4,8)	42.2	206.13	273.488	1.33	98.821	27.18	0.28
Si(1,1,1)	Si(1,1,9)	43.17	199.98	165.511	0.83	93.812	15.3	0.16
Si(1,1,1)	Si(4,6,6)	44.79	188.03	220.802	1.17	89.451	19.93	0.22
Si(1,1,1)	Si(1,3,9)	45.75	182.05	134.553	0.74	85.149	10.26	0.12
Si(1,1,1)	Si(4,4,8)	47.37	172.14	183.111	1.06	81.421	16.31	0.2
Si(1,1,1)	Si(1,7,7)	48.35	166.31	110.77	0.67	77.559	8.01	0.1
Si(1,1,1)	Si(0,2,10)	49.98	156.41	152.371	0.97	74.337	13.13	0.18
Si(1,1,1)	Si(2,6,8)	49.98	156.41	152.371	0.97	74.337	13.13	0.18
Si(1,1,1)	Si(1,5,9)	50.97	149.27	91.873	0.62	70.374	7.92	0.11
Si(1,1,1)	Si(3,7,7)	50.97	149.27	91.873	0.62	70.374	7.92	0.11
Si(1,1,1)	Si(3,5,9)	53.64	137.37	76.947	0.56	63.506	5.18	0.08
Si(1,1,1)	Si(2,4,10)	55.35	129.45	108.595	0.84	60.863	9.66	0.16
Si(1,1,1)	Si(1,1,11)	56.39	122.59	64.747	0.53	57.129	4.66	0.08
Si(1,1,1)	Si(5,7,7)	56.39	122.59	64.748	0.53	57.129	4.66	0.08
Si(1,1,1)	Si(0,8,8)	58.17	115.79	92.483	0.8	54.759	7.5	0.14
Si(1,1,1)	Si(1,7,9)	59.26	110.26	54.844	0.5	51.06	3.89	0.08
Si(1,1,1)	Si(1,3,11)	59.26	110.25	54.844	0.5	51.06	3.89	0.08
Si(1,1,1)	Si(0,6,10)	61.13	102.61	79.333	0.77	48.641	5.94	0.12
Si(1,1,1)	Si(3,3,11)	62.3	97.29	46.815	0.48	45.051	3.18	0.07
Si(1,1,1)	Si(3,7,9)	62.3	97.29	46.814	0.48	45.053	3.18	0.07
Si(1,1,1)	Si(0,0,12)	64.31	89.48	68.583	0.77	42.471	5.06	0.12
Si(1,1,1)	Si(1,5,11)	65.57	86.27	39.784	0.46	39.022	2.25	0.06
Si(1,1,1)	Si(7,7,7)	65.57	86.27	39.783	0.46	39.021	2.25	0.06
Si(1,1,1)	Si(2,2,12)	67.79	76.82	59.517	0.77	36.043	4.59	0.13
Si(1,1,1)	Si(3,5,11)	69.22	70.65	34.524	0.49	32.715	1.83	0.06
Si(1,1,1)	Si(5,7,9)	69.22	70.65	34.524	0.49	32.709	1.83	0.06
Si(1,1,1)	Si(0,4,12)	71.78	61.22	51.817	0.85	29.003	4.11	0.14
Si(1,1,1)	Si(1,9,9)	73.49	55.5	29.924	0.54	25.83	2.02	0.08
Si(1,1,1)	Si(2,8,10)	76.74	45.21	44.98	0.99	20.97	3.17	0.15
Si(1,1,1)	Si(1,1,13)	79.11	35.95	26.052	0.72	16.964	1.65	0.1
Si(1,1,1)	Si(4,4,12)	85.04	16.5	39.975	2.42	2.881	4.56	1.58

**Dia(1,1,1)**	**Si(1,1,1)**	**7.47**	**862.37**	**8276.686**	**9.6**	**862.513**	**7826.59**	**9.07**
**Dia(1,1,1)**	**Si(0,2,2)**	**12.26**	**556.56**	**5147.594**	**9.25**	**533.123**	**4627.57**	**8.68**
**Dia(1,1,1)**	**Si(1,1,3)**	**14.42**	**421.2**	**2643.542**	**6.28**	**234.653**	**1291.02**	**5.5**
**Dia(1,1,1)**	**Si(0,0,4)**	**17.48**	**372.03**	**2537.781**	**6.82**	**206.747**	**1293.24**	**6.26**
**Dia(1,1,1)**	**Si(1,3,3)**	**19.11**	**335.68**	**1434.119**	**4.27**	**117.74**	**456.98**	**3.88**
**Dia(1,1,1)**	**Si(2,2,4)**	**21.59**	**309.63**	**1553.293**	**5.02**	**121.064**	**555.36**	**4.59**
**Dia(1,1,1)**	**Si(1,1,5)**	**22.97**	**289.61**	**926.915**	**3.2**	**72.457**	**205.5**	**2.84**
**Dia(1,1,1)**	**Si(0,4,4)**	**25.14**	**271.22**	**1063.063**	**3.92**	**80.036**	**294.13**	**3.67**
**Dia(1,1,1)**	**Si(1,3,5)**	**26.38**	**256.99**	**637.397**	**2.48**	**51.004**	**100.81**	**1.98**
**Dia(1,1,1)**	**Si(0,2,6)**	**28.36**	**240.94**	**767.26**	**3.18**	**56.724**	**164.72**	**2.9**
**Dia(1,1,1)**	**Si(3,3,5)**	**29.5**	**231.87**	**458.827**	**1.98**	**161.51**	**66.26**	**0.41**
**Dia(1,1,1)**	**Si(4,4,4)**	**31.35**	**221.12**	**566.127**	**2.56**	**42.669**	**94.05**	**2.2**
**Dia(1,1,1)**	**Si(1,1,7)**	**32.43**	**215.55**	**349.043**	**1.62**	**141.325**	**45.27**	**0.32**
**Dia(1,1,1)**	**Si(2,4,6)**	**34.19**	**201.61**	**437.716**	**2.17**	**67.25**	**62.62**	**0.93**
**Dia(1,1,1)**	**Si(1,3,7)**	**35.23**	**197.17**	**269.615**	**1.37**	**125.797**	**33.96**	**0.27**
**Dia(1,1,1)**	**Si(3,5,5)**	**35.23**	**197.17**	**269.616**	**1.37**	**125.797**	**33.96**	**0.27**
**Dia(1,1,1)**	**Si(0,0,8)**	**36.92**	**186.14**	**345.131**	**1.85**	**120.665**	**45.75**	**0.38**
**Dia(1,1,1)**	**Si(3,3,7)**	**37.93**	**178.8**	**209.863**	**1.17**	**202.332**	**25.97**	**0.13**
**Dia(1,1,1)**	**Si(0,6,6)**	**39.58**	**171.98**	**277.849**	**1.62**	**542.887**	**33.92**	**0.06**
**Dia(1,1,1)**	**Si(1,5,7)**	**40.57**	**163.53**	**167.402**	**1.02**	**182.822**	**19.38**	**0.11**
**Dia(1,1,1)**	**Si(0,4,8)**	**42.2**	**157.45**	**224.411**	**1.43**	**176.647**	**27.5**	**0.16**
**Dia(1,1,1)**	**Si(1,1,9)**	**43.17**	**149.79**	**134.707**	**0.9**	**165.91**	**13.61**	**0.08**
**Dia(1,1,1)**	**Si(4,6,6)**	**44.79**	**144.71**	**183.807**	**1.27**	**160.033**	**22.57**	**0.14**
**Dia(1,1,1)**	**Si(1,3,9)**	**45.75**	**138.83**	**110.863**	**0.8**	**150.904**	**11.01**	**0.07**
**Dia(1,1,1)**	**Si(4,4,8)**	**47.37**	**133.23**	**152.148**	**1.14**	**81.579**	**16.4**	**0.2**
**Dia(1,1,1)**	**Si(1,7,7)**	**48.35**	**128.85**	**92.059**	**0.71**	**377.332**	**9.03**	**0.02**
**Dia(1,1,1)**	**Si(0,2,10)**	**49.98**	**123.07**	**126.881**	**1.03**	**74.27**	**14.04**	**0.19**
**Dia(1,1,1)**	**Si(2,6,8)**	**49.98**	**123.07**	**126.881**	**1.03**	**74.27**	**14.04**	**0.19**
**Dia(1,1,1)**	**Si(1,5,9)**	**50.97**	**119.5**	**77.046**	**0.64**	**70.144**	**8.17**	**0.12**
**Dia(1,1,1)**	**Si(3,7,7)**	**50.97**	**119.5**	**77.045**	**0.64**	**70.143**	**8.17**	**0.12**
**Dia(1,1,1)**	**Si(3,5,9)**	**53.64**	**111.56**	**65.085**	**0.58**	**63.495**	**6.51**	**0.1**
**Dia(1,1,1)**	**Si(2,4,10)**	**55.35**	**104.79**	**92.196**	**0.88**	**60.718**	**8.78**	**0.14**
**Dia(1,1,1)**	**Si(1,1,11)**	**56.39**	**104.19**	**55.391**	**0.53**	**57.073**	**5.43**	**0.1**
**Dia(1,1,1)**	**Si(5,7,7)**	**56.39**	**104.18**	**55.389**	**0.53**	**57.075**	**5.43**	**0.1**
**Dia(1,1,1)**	**Si(1,3,11)**	**59.26**	**96.01**	**47.349**	**0.49**	**51.036**	**4.37**	**0.09**
**Dia(1,1,1)**	**Si(1,7,9)**	**59.26**	**96.01**	**47.347**	**0.49**	**51.036**	**4.37**	**0.09**
**Dia(1,1,1)**	**Si(0,8,8)**	**58.17**	**95.99**	**79.072**	**0.82**	**54.324**	**7.71**	**0.14**
**Dia(1,1,1)**	**Si(0,6,10)**	**61.13**	**86.13**	**67.993**	**0.79**	**48.308**	**6.42**	**0.13**
**Dia(1,1,1)**	**Si(3,3,11)**	**62.3**	**85.47**	**40.529**	**0.47**	**45.057**	**3.56**	**0.08**
**Dia(1,1,1)**	**Si(3,7,9)**	**62.3**	**85.47**	**40.527**	**0.47**	**45.056**	**3.56**	**0.08**
**Dia(1,1,1)**	**Si(0,0,12)**	**64.31**	**77.24**	**59.176**	**0.77**	**42.322**	**5.41**	**0.13**
**Dia(1,1,1)**	**Si(1,5,11)**	**65.57**	**75.19**	**35.058**	**0.47**	**38.99**	**2.97**	**0.08**
**Dia(1,1,1)**	**Si(7,7,7)**	**65.57**	**75.13**	**35.058**	**0.47**	**38.99**	**2.97**	**0.08**
**Dia(1,1,1)**	**Si(2,2,12)**	**67.79**	**66.44**	**51.535**	**0.78**	**35.958**	**4**	**0.11**
**Dia(1,1,1)**	**Si(3,5,11)**	**69.22**	**63.26**	**30.354**	**0.48**	**32.671**	**2.5**	**0.08**
**Dia(1,1,1)**	**Si(5,7,9)**	**69.22**	**63.26**	**30.353**	**0.48**	**32.672**	**2.5**	**0.08**
**Dia(1,1,1)**	**Si(0,4,12)**	**71.78**	**53.89**	**45.05**	**0.84**	**29.028**	**4.24**	**0.15**
**Dia(1,1,1)**	**Si(1,9,9)**	**73.49**	**49.62**	**26.292**	**0.53**	**25.807**	**2.05**	**0.08**
**Dia(1,1,1)**	**Si(2,8,10)**	**76.74**	**38.51**	**39.378**	**1.02**	**20.973**	**3.47**	**0.17**
**Dia(1,1,1)**	**Si(1,1,13)**	**79.11**	**32.33**	**22.896**	**0.71**	**16.958**	**1.77**	**0.1**
**Dia(1,1,1)**	**Si(4,4,12)**	**85.04**	**14.06**	**35.346**	**2.51**	**2.887**	**4.75**	**1.64**

Ei = 13.035 keV			Cryst 1-2			Cryst 1-2-3		
Cryst 1	Cryst 2,3	ΘB 2 [°]	ΔE [meV]	∫IR dΘdE [µrad meV]	FOM	ΔE [meV]	∫IR dΘdE [µrad meV]	FOM
Si(1,1,1)	Si(1,1,1)	8.72	1203.66	12860.586	10.68	1140.48	11409.22	10
Si(1,1,1)	Si(0,2,2)	14.34	716.77	7084.727	9.88	477.829	4291.77	8.98
Si(1,1,1)	Si(1,1,3)	16.88	523.69	3433.335	6.56	204.806	1186.42	5.79
Si(1,1,1)	Si(0,0,4)	20.5	444.9	3208.468	7.21	181.027	1172.67	6.48
Si(1,1,1)	Si(1,3,3)	22.44	391.45	1754.555	4.48	102.133	397.03	3.89
Si(1,1,1)	Si(2,2,4)	25.4	348.44	1897.359	5.45	102.229	497.83	4.87
Si(1,1,1)	Si(1,1,5)	27.07	324.1	1088.705	3.36	62.47	187.17	3
Si(1,1,1)	Si(0,4,4)	29.69	292.57	1258.1	4.3	68.537	267.76	3.91
Si(1,1,1)	Si(1,3,5)	31.2	275.44	736.466	2.67	42.492	96.73	2.28
Si(1,1,1)	Si(0,2,6)	33.63	252.82	888.473	3.51	48.736	153.28	3.15
Si(1,1,1)	Si(3,3,5)	35.05	239.34	525.365	2.2	32.146	53.78	1.67
Si(1,1,1)	Si(4,4,4)	37.35	220.72	653.035	2.96	35.876	92.99	2.59
Si(1,1,1)	Si(1,1,7)	38.71	210.28	387.962	1.85	103.797	36.97	0.36
Si(1,1,1)	Si(2,4,6)	40.94	194.93	493.999	2.53	27.903	56.85	2.04
Si(1,1,1)	Si(1,3,7)	42.27	185.18	294.441	1.59	90.094	25.97	0.29
Si(1,1,1)	Si(0,0,8)	44.47	172.31	382.088	2.22	22.813	38.37	1.68
Si(1,1,1)	Si(3,3,7)	45.79	163.42	227.734	1.39	78.85	17.41	0.22
Si(1,1,1)	Si(0,6,6)	47.99	152.14	300.888	1.98	75.048	28.24	0.38
Si(1,1,1)	Si(1,5,7)	49.32	144.09	179	1.24	69.346	14.27	0.21
Si(1,1,1)	Si(5,5,5)	49.32	144.09	179.001	1.24	69.347	14.27	0.21
Si(1,1,1)	Si(0,4,8)	51.56	134.04	240.662	1.8	65.887	21.26	0.32
Si(1,1,1)	Si(1,1,9)	52.92	126.95	143.199	1.13	60.557	10.67	0.18
Si(1,1,1)	Si(4,6,6)	55.23	117.39	195.942	1.67	57.197	16.79	0.29
Si(1,1,1)	Si(1,3,9)	56.65	110.06	115.38	1.05	52.54	8.46	0.16
Si(1,1,1)	Si(4,4,8)	59.09	101.23	160.425	1.58	49.108	13.41	0.27
Si(1,1,1)	Si(1,7,7)	60.61	94.49	94.329	1	44.918	6.95	0.15
Si(1,1,1)	Si(0,2,10)	63.26	85.05	133.328	1.57	41.39	10.8	0.26
Si(1,1,1)	Si(1,5,9)	64.93	78.37	77.647	0.99	37.354	5.67	0.15
Si(1,1,1)	Si(3,5,9)	69.89	61.37	64.513	1.05	29.423	4.27	0.15
Si(1,1,1)	Si(2,4,10)	73.59	50.01	93.843	1.88	12.433	7.95	0.64
Si(1,1,1)	Si(1,1,11)	76.21	41.5	54.078	1.3	20.165	3.68	0.18
Si(1,1,1)	Si(0,8,8)	82.19	23.89	79.645	3.33	5.235	11.78	2.25
Dia(1,1,1)	Si(1,1,1)	8.72	746.22	7758.642	10.4	745.347	7190.4	9.65
Dia(1,1,1)	Si(0,2,2)	14.34	486.99	4965.089	10.2	463.584	4399.33	9.49
Dia(1,1,1)	Si(1,1,3)	16.88	360.05	2542.444	7.06	203.54	1254.78	6.16
Dia(1,1,1)	Si(0,0,4)	20.5	320.11	2440.148	7.62	180.19	1246.48	6.92
Dia(1,1,1)	Si(1,3,3)	22.44	283.24	1364.599	4.82	101.537	425.86	4.19
Dia(1,1,1)	Si(2,2,4)	25.4	258.95	1500.763	5.8	104.047	525.52	5.05
Dia(1,1,1)	Si(1,1,5)	27.07	239.36	871.593	3.64	62.713	198.86	3.17
Dia(1,1,1)	Si(0,4,4)	29.69	221.18	1027.389	4.65	69.474	281.02	4.05
Dia(1,1,1)	Si(1,3,5)	31.2	210.05	602.075	2.87	42.722	105.51	2.47
Dia(1,1,1)	Si(0,2,6)	33.63	195.29	740.213	3.79	49.1	163.92	3.34
Dia(1,1,1)	Si(3,3,5)	35.05	188.05	439.749	2.34	32.093	56.98	1.78
Dia(1,1,1)	Si(4,4,4)	37.35	174.12	547.152	3.14	35.958	101.06	2.81
Dia(1,1,1)	Si(1,1,7)	38.71	168.05	330.102	1.96	104.003	38.32	0.37
Dia(1,1,1)	Si(2,4,6)	40.94	157.53	422.11	2.68	27.975	61.46	2.2
Dia(1,1,1)	Si(1,3,7)	42.27	150.27	251.883	1.68	89.958	26.78	0.3
Dia(1,1,1)	Si(0,0,8)	44.47	141.73	330.694	2.33	22.817	41.44	1.82
Dia(1,1,1)	Si(3,3,7)	45.79	135.88	196.207	1.44	78.733	21.22	0.27
Dia(1,1,1)	Si(0,6,6)	47.99	126.56	261.39	2.07	75.827	29.13	0.38
Dia(1,1,1)	Si(1,5,7)	49.32	123.69	156.048	1.26	123.351	14.82	0.12

Dia(1,1,1)	Si(5,5,5)	49.32	123.69	156.049	1.26	123.351	14.82	0.12
Dia(1,1,1)	Si(0,4,8)	51.56	113.68	210.93	1.86	327.559	22.97	0.07
Dia(1,1,1)	Si(1,1,9)	52.92	112.06	125.764	1.12	107.926	10.94	0.1
Dia(1,1,1)	Si(4,6,6)	55.23	100.98	171.861	1.7	285.339	17.4	0.06
Dia(1,1,1)	Si(1,3,9)	56.65	99.67	102.323	1.03	93.721	8.61	0.09
Dia(1,1,1)	Si(4,4,8)	59.09	89.34	142.364	1.59	88.735	13.84	0.16
Dia(1,1,1)	Si(1,7,7)	60.61	86.33	84.014	0.97	80.181	6.8	0.08
Dia(1,1,1)	Si(0,2,10)	63.26	75.99	118.791	1.56	74.908	11.15	0.15
Dia(1,1,1)	Si(1,5,9)	64.93	72.23	69.69	0.96	185.702	5.39	0.03
Dia(1,1,1)	Si(3,5,9)	69.89	57.1	58.485	1.02	29.415	4.44	0.15
Dia(1,1,1)	Si(2,4,10)	73.59	45.52	85.64	1.88	6.546	7.83	1.2
Dia(1,1,1)	Si(1,1,11)	76.21	38.66	49.52	1.28	36.501	3.71	0.1
Dia(1,1,1)	Si(0,8,8)	82.19	21.19	73.554	3.47	5.319	12.33	2.32

Ei = 16.388 keV Cryst 1	Cryst 2,3	ΘB 2 [°]	Cryst 1-2 ΔE [meV]	Cryst 1-2 ∫IR dΘdE [μrad meV]	Cryst 1-2 FOM	Cryst 1-2-3 ΔE [meV]	Cryst 1-2-3 ∫IR dΘdE [μrad meV]	Cryst 1-2-3 FOM
Si(1,1,1)	Si(1,1,1)	6.93	1447.1	14519.829	10.03	12000.8	13228.67	1.1
Si(1,1,1)	Si(0,2,2)	11.36	896.15	7920.823	8.84	565.039	4754.88	8.42
Si(1,1,1)	Si(1,1,3)	13.36	692.28	3889.698	5.62	261.305	1364.3	5.22
Si(1,1,1)	Si(0,0,4)	16.18	594.55	3606.088	6.07	230.431	1309.8	5.68
Si(1,1,1)	Si(1,3,3)	17.67	541.32	2012.047	3.72	128.736	435.67	3.38
Si(1,1,1)	Si(2,2,4)	19.95	485.55	2153.463	4.44	131.361	565.51	4.3
Si(1,1,1)	Si(1,1,5)	21.22	457.39	1252.218	2.74	343.333	208.78	0.61
Si(1,1,1)	Si(0,4,4)	23.2	417.54	1440.044	3.45	603.841	268.29	0.44
Si(1,1,1)	Si(1,3,5)	24.33	407.68	863.539	2.12	1473	128.89	0.09
Si(1,1,1)	Si(0,2,6)	26.14	373.75	1024.117	2.74	512.787	166.45	0.32
Si(1,1,1)	Si(3,3,5)	27.18	362.79	619.065	1.71	451.652	91.52	0.2
Si(1,1,1)	Si(4,4,4)	28.85	341.13	758.049	2.22	441.562	116.74	0.26
Si(1,1,1)	Si(1,1,7)	29.83	326.5	461.05	1.41	393.012	64.91	0.17
Si(1,1,1)	Si(2,4,6)	31.41	308.5	575.156	1.86	386.215	83.59	0.22
Si(1,1,1)	Si(1,3,7)	32.34	297.94	353.581	1.19	349.191	47.65	0.14
Si(1,1,1)	Si(3,5,5)	32.34	297.94	353.58	1.19	349.191	47.65	0.14
Si(1,1,1)	Si(0,0,8)	33.86	280.6	447.163	1.59	342.925	61.54	0.18
Si(1,1,1)	Si(3,3,7)	34.76	276.14	276.949	1	312.381	35.05	0.11
Si(1,1,1)	Si(0,6,6)	36.23	259.82	356.715	1.37	345.563	49.72	0.14
Si(1,1,1)	Si(1,5,7)	37.1	256.93	219.415	0.85	285.871	27.69	0.1
Si(1,1,1)	Si(0,4,8)	38.53	240.7	287.43	1.19	773.795	38.62	0.05
Si(1,1,1)	Si(1,1,9)	39.39	239.53	176.538	0.74	261.22	19.98	0.08
Si(1,1,1)	Si(4,6,6)	40.8	225.99	235.064	1.04	699.172	32.54	0.05
Si(1,1,1)	Si(1,3,9)	41.64	220.93	143.155	0.65	239.451	15.08	0.06
Si(1,1,1)	Si(4,4,8)	43.03	210.34	193.694	0.92	262.821	23.32	0.09
Si(1,1,1)	Si(1,7,7)	43.87	203.29	117.719	0.58	220.148	14.37	0.07
Si(1,1,1)	Si(0,2,10)	45.26	195.36	160.826	0.82	506.458	20.4	0.04
Si(1,1,1)	Si(1,5,9)	46.09	192.26	97.556	0.51	227.944	11.37	0.05
Si(1,1,1)	Si(3,5,9)	48.32	175.13	81.451	0.47	520.299	9.17	0.02
Si(1,1,1)	Si(2,4,10)	49.73	167.7	114.387	0.68	181.162	12.76	0.07
Si(1,1,1)	Si(1,1,11)	50.58	163.6	68.398	0.42	479.345	7.77	0.02
Si(1,1,1)	Si(0,8,8)	52	154.32	98.036	0.64	166.327	10.34	0.06
Si(1,1,1)	Si(1,3,11)	52.86	148.31	58.825	0.4	440.685	6.54	0.01
Si(1,1,1)	Si(1,7,9)	52.86	148.31	58.824	0.4	440.681	6.54	0.01
Si(1,1,1)	Si(0,6,10)	54.32	140.28	84.173	0.6	152.382	8.87	0.06
Si(1,1,1)	Si(6,6,8)	54.32	140.28	84.173	0.6	152.382	8.87	0.06
Si(1,1,1)	Si(3,3,11)	55.2	137.31	50.475	0.37	403.815	5.49	0.01
Si(1,1,1)	Si(0,0,12)	56.7	129.4	73.085	0.56	386.9	8.59	0.02
Si(1,1,1)	Si(4,8,8)	56.7	129.4	73.085	0.56	386.9	8.59	0.02
Si(1,1,1)	Si(7,7,7)	57.62	125.67	43.543	0.35	368.17	4.58	0.01
Si(1,1,1)	Si(1,5,11)	57.62	125.66	43.543	0.35	368.172	4.58	0.01
Si(1,1,1)	Si(2,2,12)	59.17	116.47	63.403	0.54	351.029	6.45	0.02
Si(1,1,1)	Si(3,5,11)	60.13	113.7	37.796	0.33	333.228	4.03	0.01
Si(1,1,1)	Si(0,4,12)	61.77	105.29	55.482	0.53	315.684	5.08	0.02
Si(1,1,1)	Si(1,9,9)	62.78	101.44	32.852	0.32	298.425	3.39	0.01
Si(1,1,1)	Si(2,8,10)	64.53	93.43	48.68	0.52	280.231	4.45	0.02
Si(1,1,1)	Si(1,1,13)	65.62	89.64	28.646	0.32	54.564	2.84	0.05
Si(1,1,1)	Si(4,4,12)	67.52	81.46	43.037	0.53	243.867	4.28	0.02
Si(1,1,1)	Si(1,3,13)	68.73	75.57	25.009	0.33	46.939	2.36	0.05
Si(1,1,1)	Si(2,6,12)	70.87	68.56	38.083	0.56	205.386	4.2	0.02
Si(1,1,1)	Si(3,3,13)	72.26	64.65	21.842	0.34	38.694	1.91	0.05
Si(1,1,1)	Si(8,8,8)	74.82	53.32	33.767	0.63	20.673	2.86	0.14

Si(1,1,1)	Si(1,5,13)	76.56	47.07	19.385	0.41	140.658	1.25	0.01
Si(1,1,1)	Si(0,2,14)	80.07	34.71	29.919	0.86	21.781	2.79	0.13
Si(1,1,1)	Si(0,10,10)	80.07	34.71	29.919	0.86	21.781	2.79	0.13
Si(1,1,1)	Si(1,9,11)	82.92	24.54	17.114	0.7	77.668	1.38	0.02
Dia(1,1,1)	Si(1,1,1)	6.93	927.18	8502.364	9.17	5057.65	7882.64	1.56
Dia(1,1,1)	Si(0,2,2)	11.36	594.89	5215.577	8.77	558.54	4812.83	8.62
Dia(1,1,1)	Si(1,1,3)	13.36	455.97	2669.088	5.85	257.551	1409.25	5.47
Dia(1,1,1)	Si(0,0,4)	16.18	404.6	2577.68	6.37	227.119	1355.33	5.97
Dia(1,1,1)	Si(1,3,3)	17.67	363.38	1456.13	4.01	128.457	450.12	3.5
Dia(1,1,1)	Si(2,2,4)	19.95	335.41	1592.579	4.75	131.122	585.01	4.46
Dia(1,1,1)	Si(1,1,5)	21.22	316.21	947.46	3	337.17	214.71	0.64
Dia(1,1,1)	Si(0,4,4)	23.2	295.48	1086.512	3.68	1367.87	274.34	0.2
Dia(1,1,1)	Si(1,3,5)	24.33	281	655.746	2.33	460.07	135.64	0.29
Dia(1,1,1)	Si(0,2,6)	26.14	265.36	788.002	2.97	447.744	171.32	0.38
Dia(1,1,1)	Si(3,3,5)	27.18	255.48	475.385	1.86	1251.29	97.05	0.08
Dia(1,1,1)	Si(4,4,4)	28.85	242.01	585.395	2.42	229.81	118.34	0.51
Dia(1,1,1)	Si(1,1,7)	29.83	237.9	357.491	1.5	396.601	64.22	0.16
Dia(1,1,1)	Si(2,4,6)	31.41	224.69	448.744	2	203.549	87.96	0.43
Dia(1,1,1)	Si(1,3,7)	32.34	222.52	277.922	1.25	190.056	50.13	0.26
Dia(1,1,1)	Si(3,5,5)	32.34	222.52	277.922	1.25	190.056	50.13	0.26
Dia(1,1,1)	Si(0,0,8)	33.86	209.97	352.971	1.68	800.953	66.4	0.08
Dia(1,1,1)	Si(3,3,7)	34.76	207.46	220.583	1.06	171.718	34.82	0.2
Dia(1,1,1)	Si(0,6,6)	36.23	196.05	283.638	1.45	166.93	49.75	0.3
Dia(1,1,1)	Si(1,5,7)	37.1	190.28	174.954	0.92	157.216	29.31	0.19
Dia(1,1,1)	Si(0,4,8)	38.53	182.29	229.654	1.26	151.374	40.68	0.27
Dia(1,1,1)	Si(1,1,9)	39.39	175.29	140.535	0.8	144.759	21.27	0.15
Dia(1,1,1)	Si(4,6,6)	40.8	168.86	188.822	1.12	138.556	32.69	0.24
Dia(1,1,1)	Si(1,3,9)	41.64	161.39	114.477	0.71	132.285	16.8	0.13
Dia(1,1,1)	Si(4,4,8)	43.03	156.17	155.208	0.99	128.064	25.49	0.2
Dia(1,1,1)	Si(1,7,7)	43.87	149.35	94.153	0.63	197.042	14.92	0.08
Dia(1,1,1)	Si(0,2,10)	45.26	144.47	129.934	0.9	118.042	21.38	0.18
Dia(1,1,1)	Si(1,5,9)	46.09	138.6	77.93	0.56	112.635	12.22	0.11
Dia(1,1,1)	Si(3,5,9)	48.32	128.3	65.726	0.51	104.436	8.45	0.08
Dia(1,1,1)	Si(2,4,10)	49.73	124.12	93.145	0.75	99.912	13.12	0.13
Dia(1,1,1)	Si(1,1,11)	50.58	119.11	55.647	0.47	96.864	6.68	0.07
Dia(1,1,1)	Si(0,8,8)	52	115.46	80.094	0.69	92.12	11.32	0.12
Dia(1,1,1)	Si(1,3,11)	52.86	111.76	48.167	0.43	89.623	7.4	0.08
Dia(1,1,1)	Si(1,7,9)	52.86	111.76	48.166	0.43	89.622	7.4	0.08
Dia(1,1,1)	Si(0,6,10)	54.32	106.49	69.041	0.65	85.038	10.31	0.12
Dia(1,1,1)	Si(6,6,8)	54.32	106.49	69.041	0.65	85.038	10.31	0.12
Dia(1,1,1)	Si(3,3,11)	55.2	104.25	41.683	0.4	82.083	5.75	0.07
Dia(1,1,1)	Si(0,0,12)	56.7	99.21	60.437	0.61	77.752	9.15	0.12
Dia(1,1,1)	Si(4,8,8)	56.7	99.21	60.437	0.61	77.752	9.15	0.12
Dia(1,1,1)	Si(1,5,11)	57.62	97.28	36.271	0.37	74.814	4.38	0.06
Dia(1,1,1)	Si(7,7,7)	57.62	97.28	36.275	0.37	74.812	4.38	0.06
Dia(1,1,1)	Si(2,2,12)	59.17	91.55	53.068	0.58	70.673	7.7	0.11
Dia(1,1,1)	Si(3,5,11)	60.13	90.6	31.826	0.35	67.777	3.39	0.05
Dia(1,1,1)	Si(0,4,12)	61.77	83.99	46.794	0.56	63.705	6.49	0.1
Dia(1,1,1)	Si(1,9,9)	62.78	83.5	27.941	0.33	60.788	2.76	0.05
Dia(1,1,1)	Si(1,1,13)	65.62	76.06	24.564	0.32	53.68	2.41	0.04
Dia(1,1,1)	Si(2,8,10)	64.53	75.91	41.355	0.54	56.655	5.54	0.1
Dia(1,1,1)	Si(4,4,12)	67.52	67.46	36.652	0.54	49.22	4.78	0.1
Dia(1,1,1)	Si(1,3,13)	68.73	65.87	21.604	0.33	46.294	2.24	0.05
Dia(1,1,1)	Si(2,6,12)	70.87	57.15	32.502	0.57	41.264	4.1	0.1
Dia(1,1,1)	Si(3,3,13)	72.26	55	19.112	0.35	38.257	2.09	0.05

**Dia(1,1,1)**	**Si(8,8,8)**	**74.82**	**45.2**	**28.938**	**0.64**	**32.348**	**3.35**	**0.1**
**Dia(1,1,1)**	**Si(1,5,13)**	**76.56**	**40.94**	**16.798**	**0.41**	**28.623**	**1.81**	**0.06**
**Dia(1,1,1)**	**Si(0,2,14)**	**80.07**	**29.11**	**25.823**	**0.89**	**21.592**	**2.72**	**0.13**
**Dia(1,1,1)**	**Si(0,10,10)**	**80.07**	**29.11**	**25.823**	**0.89**	**21.592**	**2.72**	**0.13**
**Dia(1,1,1)**	**Si(1,9,11)**	**82.92**	**20.94**	**14.791**	**0.71**	**15.246**	**1.24**	**0.08**

Ei = 15.711 keV			Cryst 1-2			Cryst 1-2-3		
Cryst 1	Cryst 2,3	ΘB 2	ΔE	∫IR dΘdE	FOM	ΔE	∫IR dΘdE	FOM
		[°]	[meV]	[µrad meV]		[meV]	[µrad meV]	
Si(1,1,1)	Si(1,1,1)	7.23	1398.82	14165.227	10.13	11316.8	12965.53	1.15
Si(1,1,1)	Si(0,2,2)	11.86	859.61	7778.78	9.05	545.88	4692.09	8.6
Si(1,1,1)	Si(1,1,3)	13.94	658.63	3809.051	5.78	251.858	1339.86	5.32
Si(1,1,1)	Si(0,0,4)	16.89	564.35	3541.834	6.28	219.86	1277.7	5.81
Si(1,1,1)	Si(1,3,3)	18.46	511.36	1970.545	3.85	123.405	434.53	3.52
Si(1,1,1)	Si(2,2,4)	20.85	457.62	2112.762	4.62	126.626	563.15	4.45
Si(1,1,1)	Si(1,1,5)	22.18	432.04	1232.676	2.85	158.735	200.68	1.26
Si(1,1,1)	Si(0,4,4)	24.27	392.65	1409.441	3.59	1277.31	265.62	0.21
Si(1,1,1)	Si(1,3,5)	25.46	381.93	841.613	2.2	265.162	121.76	0.46
Si(1,1,1)	Si(0,2,6)	27.35	350.07	1000.023	2.86	1300.2	160.28	0.12
Si(1,1,1)	Si(3,3,5)	28.45	337.51	602.027	1.78	417.009	87.2	0.21
Si(1,1,1)	Si(4,4,4)	30.22	316.8	739.128	2.33	405.759	112.13	0.28
Si(1,1,1)	Si(1,1,7)	31.25	303.54	448.413	1.48	362.168	61.2	0.17
Si(1,1,1)	Si(2,4,6)	32.93	285.43	560.905	1.97	356.242	79.21	0.22
Si(1,1,1)	Si(1,3,7)	33.92	276.59	342.786	1.24	321.086	41.45	0.13
Si(1,1,1)	Si(0,0,8)	35.54	260.73	436.854	1.68	316.158	58.33	0.18
Si(1,1,1)	Si(3,3,7)	36.49	255.83	267.689	1.05	286.019	33.44	0.12
Si(1,1,1)	Si(0,6,6)	38.06	239.55	345.72	1.44	282.316	47.1	0.17
Si(1,1,1)	Si(1,5,7)	38.99	234.96	211.602	0.9	259.361	25.67	0.1
Si(1,1,1)	Si(0,4,8)	40.53	222.59	279.316	1.25	254.244	32.78	0.13
Si(1,1,1)	Si(1,1,9)	41.44	216.97	169.739	0.78	236.539	20.65	0.09
Si(1,1,1)	Si(4,6,6)	42.96	205.57	228.082	1.11	258.335	30.16	0.12
Si(1,1,1)	Si(1,3,9)	43.87	199.93	137.561	0.69	215.698	14.82	0.07
Si(1,1,1)	Si(4,4,8)	45.39	187.38	186.87	1	209.457	21.48	0.1
Si(1,1,1)	Si(1,7,7)	46.29	181.82	113.679	0.63	197.084	11.76	0.06
Si(1,1,1)	Si(5,5,7)	46.29	181.82	113.679	0.63	197.084	11.76	0.06
Si(1,1,1)	Si(0,2,10)	47.81	173.58	155.985	0.9	191.646	18.75	0.1
Si(1,1,1)	Si(1,5,9)	48.72	166.15	94.383	0.57	180.171	9.88	0.05
Si(1,1,1)	Si(3,5,9)	51.18	156.58	78.459	0.5	164.576	8.17	0.05
Si(1,1,1)	Si(2,4,10)	52.74	145.62	110.966	0.76	159.023	11.67	0.07
Si(1,1,1)	Si(1,1,11)	53.68	140.37	66.869	0.48	417.505	6.91	0.02
Si(1,1,1)	Si(0,8,8)	55.28	132.76	95.214	0.72	144.247	9.45	0.07
Si(1,1,1)	Si(1,3,11)	56.26	127.62	56.8	0.45	378.832	5.8	0.02
Si(1,1,1)	Si(0,6,10)	57.92	121.33	81.27	0.67	130.17	8.05	0.06
Si(1,1,1)	Si(3,3,11)	58.93	118.15	48.255	0.41	341.323	4.83	0.01
Si(1,1,1)	Si(0,0,12)	60.67	107.68	70.475	0.65	116.516	6.69	0.06
Si(1,1,1)	Si(1,5,11)	61.75	103.13	41.75	0.4	304.358	4.03	0.01
Si(1,1,1)	Si(2,2,12)	63.6	95.11	61.146	0.64	102.994	5.69	0.06
Si(1,1,1)	Si(3,5,11)	64.76	92.1	35.764	0.39	267.17	3.35	0.01
Si(1,1,1)	Si(5,7,9)	64.76	92.1	35.763	0.39	267.173	3.35	0.01
Si(1,1,1)	Si(0,4,12)	66.78	82.3	53.551	0.65	89.281	4.98	0.06
Si(1,1,1)	Si(1,9,9)	68.06	76.87	31.174	0.41	228.738	2.76	0.01
Si(1,1,1)	Si(2,8,10)	70.34	68.03	46.908	0.69	74.923	4.09	0.05
Si(1,1,1)	Si(1,1,13)	71.82	63.57	26.963	0.42	187.304	1.93	0.01
Si(1,1,1)	Si(4,4,12)	74.55	52.68	41.324	0.78	163.496	4.24	0.03
Si(1,1,1)	Si(1,3,13)	76.42	46.4	23.754	0.51	50.278	2.09	0.04
Si(1,1,1)	Si(2,6,12)	80.23	33.16	36.511	1.1	20.857	3.72	0.18
Si(1,1,1)	Si(3,3,13)	83.47	22.09	20.846	0.94	13.937	1.71	0.12
Dia(1,1,1)	Si(1,1,1)	7.23	889.87	8372.627	9.41	4781.56	7732.2	1.62
Dia(1,1,1)	Si(0,2,2)	11.86	573.7	5182.406	9.03	534.185	4735.11	8.86
Dia(1,1,1)	Si(1,1,3)	13.94	435.85	2653.34	6.09	245.662	1377.54	5.61

Dia(1,1,1)	Si(0,0,4)	16.89	386.65	2559.61	6.62	218.843	1338.79	6.12
Dia(1,1,1)	Si(1,3,3)	18.46	347.72	1444.244	4.15	123.18	453.46	3.68
Dia(1,1,1)	Si(2,2,4)	20.85	320.36	1576.304	4.92	127.166	587.98	4.62
Dia(1,1,1)	Si(1,1,5)	22.18	300.91	936.523	3.11	158.421	207.19	1.31
Dia(1,1,1)	Si(0,4,4)	24.27	280.78	1074.636	3.83	1509.15	271.33	0.18
Dia(1,1,1)	Si(1,3,5)	25.46	265.61	643.285	2.42	426.037	126.64	0.3
Dia(1,1,1)	Si(0,2,6)	27.35	251.21	773.904	3.08	412.744	165.54	0.4
Dia(1,1,1)	Si(3,3,5)	28.45	244.2	467.678	1.92	1150.15	85.22	0.07
Dia(1,1,1)	Si(4,4,4)	30.22	228.91	576.04	2.52	1103.47	114.05	0.1
Dia(1,1,1)	Si(1,1,7)	31.25	226.53	353.172	1.56	327.398	61.55	0.19
Dia(1,1,1)	Si(2,4,6)	32.93	212.12	443.022	2.09	187.111	83.9	0.45
Dia(1,1,1)	Si(1,3,7)	33.92	208.58	272.414	1.31	174.643	47.34	0.27
Dia(1,1,1)	Si(0,0,8)	35.54	196.3	348.449	1.78	319.081	62.91	0.2
Dia(1,1,1)	Si(3,3,7)	36.49	191.23	214.724	1.12	157.108	35.12	0.22
Dia(1,1,1)	Si(0,6,6)	38.06	181.92	279.777	1.54	152.848	46.62	0.31
Dia(1,1,1)	Si(1,5,7)	38.99	174.47	169.893	0.97	143.07	27.35	0.19
Dia(1,1,1)	Si(0,4,8)	40.53	168.15	226.674	1.35	138.519	38.4	0.28
Dia(1,1,1)	Si(1,1,9)	41.44	159.68	136.437	0.85	130.944	20.62	0.16
Dia(1,1,1)	Si(4,6,6)	42.96	154.74	185.346	1.2	125.641	31.95	0.25
Dia(1,1,1)	Si(1,3,9)	43.87	147.4	111.465	0.76	119.517	15.51	0.13
Dia(1,1,1)	Si(4,4,8)	45.39	142.69	153.555	1.08	115.083	23.82	0.21
Dia(1,1,1)	Si(1,7,7)	46.29	137.39	92.515	0.67	470.784	13.67	0.03
Dia(1,1,1)	Si(5,5,7)	46.29	137.39	92.509	0.67	470.783	13.67	0.03
Dia(1,1,1)	Si(0,2,10)	47.81	131.64	128.298	0.97	105.605	19.95	0.19
Dia(1,1,1)	Si(1,5,9)	48.72	126.75	77.566	0.61	87.437	11.57	0.13
Dia(1,1,1)	Si(3,5,9)	51.18	118.53	65.63	0.55	92.56	9.51	0.1
Dia(1,1,1)	Si(2,4,10)	52.74	113.22	92.96	0.82	87.913	12.57	0.14
Dia(1,1,1)	Si(1,1,11)	53.68	110.98	55.85	0.5	84.747	6.4	0.08
Dia(1,1,1)	Si(0,8,8)	55.28	104.58	79.047	0.76	79.93	10.27	0.13
Dia(1,1,1)	Si(1,3,11)	56.26	102.95	48.082	0.47	77.314	4.88	0.06
Dia(1,1,1)	Si(3,3,11)	58.93	96.7	41.658	0.43	69.799	4.82	0.07
Dia(1,1,1)	Si(0,6,10)	57.92	96.57	69.16	0.72	72.392	8.72	0.12
Dia(1,1,1)	Si(0,0,12)	60.67	88.65	60.276	0.68	64.924	7.83	0.12
Dia(1,1,1)	Si(1,5,11)	61.75	88.51	35.895	0.41	62.309	4.63	0.07
Dia(1,1,1)	Si(2,2,12)	63.6	78.93	52.436	0.66	57.364	6.63	0.12
Dia(1,1,1)	Si(3,5,11)	64.76	78.67	31.257	0.4	54.847	3.84	0.07
Dia(1,1,1)	Si(5,7,9)	64.76	78.67	31.256	0.4	54.849	3.84	0.07
Dia(1,1,1)	Si(0,4,12)	66.78	69.72	46.02	0.66	49.674	5.58	0.11
Dia(1,1,1)	Si(1,9,9)	68.06	67.94	27.025	0.4	46.885	3.23	0.07
Dia(1,1,1)	Si(2,8,10)	70.34	59.24	40.458	0.68	41.517	4.69	0.11
Dia(1,1,1)	Si(1,1,13)	71.82	55.49	23.777	0.43	38.227	2.49	0.07
Dia(1,1,1)	Si(4,4,12)	74.55	45.77	35.702	0.78	32.499	4.49	0.14
Dia(1,1,1)	Si(1,3,13)	76.42	40.9	20.662	0.51	28.032	2.09	0.07
Dia(1,1,1)	Si(2,6,12)	80.23	28.19	31.572	1.12	20.687	3.63	0.18
Dia(1,1,1)	Si(3,3,13)	83.47	18.84	18.093	0.96	13.778	1.82	0.13

Ei = 13.419 keV			Cryst 1-2			Cryst 1-2-3		
Cryst 1	Cryst 2,3	ΘB 2 [°]	ΔE [meV]	∫IR dΘdE [μrad meV]	FOM	ΔE [meV]	∫IR dΘdE [μrad meV]	FOM
Si(1,1,1)	Si(1,1,1)	8.47	1233.03	13070.106	10.6	5252.74	11580.29	2.2
Si(1,1,1)	Si(0,2,2)	13.92	736.36	7201.116	9.78	483.109	4389.69	9.09
Si(1,1,1)	Si(1,1,3)	16.39	543.33	3494.387	6.43	215.873	1222.54	5.66
Si(1,1,1)	Si(0,0,4)	19.89	462.31	3262.298	7.06	191.101	1182.04	6.19
Si(1,1,1)	Si(1,3,3)	21.76	407.95	1790.272	4.39	105.257	414.96	3.94
Si(1,1,1)	Si(2,2,4)	24.63	363.71	1931.918	5.31	108.62	537.13	4.95
Si(1,1,1)	Si(1,1,5)	26.23	340.95	1111.529	3.26	66.199	176.53	2.67
Si(1,1,1)	Si(0,4,4)	28.76	306.75	1279.91	4.17	70.64	273.02	3.86
Si(1,1,1)	Si(1,3,5)	30.21	290.32	752.506	2.59	199.466	105.22	0.53
Si(1,1,1)	Si(0,2,6)	32.55	267.47	906.16	3.39	51.321	143.45	2.8
Si(1,1,1)	Si(3,3,5)	33.9	252.93	537.497	2.13	167.831	69.83	0.42
Si(1,1,1)	Si(4,4,4)	36.11	233.77	666.059	2.85	161.185	94.67	0.59
Si(1,1,1)	Si(1,1,7)	37.41	224.12	398.078	1.78	265.287	48.94	0.18
Si(1,1,1)	Si(2,4,6)	39.53	207.77	505.805	2.43	717.487	66.32	0.09
Si(1,1,1)	Si(1,3,7)	40.8	198.84	301.902	1.52	229.777	35.83	0.16
Si(1,1,1)	Si(0,0,8)	42.88	184.93	391.085	2.11	228.514	50.78	0.22
Si(1,1,1)	Si(3,3,7)	44.13	176.86	234.207	1.32	200.965	25.89	0.13
Si(1,1,1)	Si(0,6,6)	46.2	167.37	307.014	1.83	198.593	39.79	0.2
Si(1,1,1)	Si(1,5,7)	47.45	157.05	184.741	1.18	176.162	19.81	0.11
Si(1,1,1)	Si(0,4,8)	49.54	146.92	247.416	1.68	172.81	29.38	0.17
Si(1,1,1)	Si(1,1,9)	50.8	138.52	147.378	1.06	154.877	15.07	0.1
Si(1,1,1)	Si(4,6,6)	52.93	130.01	201.205	1.55	151.186	23.26	0.15
Si(1,1,1)	Si(1,3,9)	54.24	123.28	119.328	0.97	136.303	13	0.1
Si(1,1,1)	Si(4,4,8)	56.45	114.14	165.157	1.45	131.874	18.8	0.14
Si(1,1,1)	Si(1,7,7)	57.82	107.77	97.398	0.9	119.079	9.98	0.08
Si(1,1,1)	Si(5,5,7)	57.82	107.77	97.399	0.9	119.079	9.98	0.08
Si(1,1,1)	Si(0,2,10)	60.17	98.44	136.925	1.39	113.936	14.05	0.12
Si(1,1,1)	Si(1,5,9)	61.63	92.59	80.278	0.87	102.352	7.79	0.08
Si(1,1,1)	Si(3,5,9)	65.81	76.86	66.787	0.87	85.624	6.3	0.07
Si(1,1,1)	Si(2,4,10)	68.72	66.92	96.669	1.44	88.272	9.98	0.11
Si(1,1,1)	Si(1,1,11)	70.63	60.87	56.015	0.92	68.002	5.15	0.08
Si(1,1,1)	Si(0,8,8)	74.23	48.76	82.021	1.68	58.818	8.05	0.14
Si(1,1,1)	Si(1,3,11)	76.8	40.42	47.267	1.17	23.155	3.86	0.17
Si(1,1,1)	Si(0,6,10)	82.74	22.48	70.419	3.13	4.694	9.46	2.01
Dia(1,1,1)	Si(1,1,1)	8.47	766.58	7870.618	10.27	3912.08	7147.61	1.83
Dia(1,1,1)	Si(0,2,2)	13.92	499.61	5013.143	10.03	465.504	4498.06	9.66
Dia(1,1,1)	Si(1,1,3)	16.39	370.98	2564.67	6.91	215.639	1290.11	5.98
Dia(1,1,1)	Si(0,0,4)	19.89	329.41	2461.353	7.47	189.47	1258.63	6.64
Dia(1,1,1)	Si(1,3,3)	21.76	292.52	1378.551	4.71	104.945	443.34	4.22
Dia(1,1,1)	Si(2,2,4)	24.63	268.05	1511.014	5.64	105.782	571.42	5.4
Dia(1,1,1)	Si(1,1,5)	26.23	248.51	881.465	3.55	66.184	185.85	2.81
Dia(1,1,1)	Si(0,4,4)	28.76	230.04	1033.204	4.49	71.805	285.19	3.97
Dia(1,1,1)	Si(1,3,5)	30.21	218.97	607.817	2.78	319.383	112.91	0.35
Dia(1,1,1)	Si(0,2,6)	32.55	202.59	737.269	3.64	303.303	151.26	0.5
Dia(1,1,1)	Si(3,3,5)	33.9	196.43	443.997	2.26	280.356	75.07	0.27
Dia(1,1,1)	Si(4,4,4)	36.11	181.61	548.365	3.02	687.795	101.73	0.15
Dia(1,1,1)	Si(1,1,7)	37.41	176.68	334.32	1.89	271.163	52.05	0.19
Dia(1,1,1)	Si(2,4,6)	39.53	165.83	426.844	2.57	719.34	70.69	0.1
Dia(1,1,1)	Si(1,3,7)	40.8	158.35	255.432	1.61	239.06	37.04	0.15
Dia(1,1,1)	Si(0,0,8)	42.88	150.04	334.757	2.23	631.043	53.8	0.09
Dia(1,1,1)	Si(3,3,7)	44.13	143.43	199.481	1.39	111.883	30.05	0.27

**Dia(1,1,1)**	**Si(0,6,6)**	**46.2**	**135.11**	**265.606**	**1.97**	**227.447**	**40.1**	**0.18**
**Dia(1,1,1)**	**Si(1,5,7)**	**47.45**	**130.03**	**158.423**	**1.22**	**98.412**	**21.15**	**0.21**
**Dia(1,1,1)**	**Si(0,4,8)**	**49.54**	**121.93**	**213.838**	**1.75**	**93.717**	**30.45**	**0.32**
**Dia(1,1,1)**	**Si(1,1,9)**	**50.8**	**119.37**	**127.859**	**1.07**	**87.052**	**16.17**	**0.19**
**Dia(1,1,1)**	**Si(4,6,6)**	**52.93**	**110.13**	**174.763**	**1.59**	**82.709**	**23.95**	**0.29**
**Dia(1,1,1)**	**Si(1,3,9)**	**54.24**	**108.88**	**104.479**	**0.96**	**76.765**	**13.5**	**0.18**
**Dia(1,1,1)**	**Si(4,4,8)**	**56.45**	**98.22**	**143.969**	**1.47**	**72.551**	**19.39**	**0.27**
**Dia(1,1,1)**	**Si(1,7,7)**	**57.82**	**96.86**	**85.982**	**0.89**	**67.063**	**9.98**	**0.15**
**Dia(1,1,1)**	**Si(5,5,7)**	**57.82**	**96.85**	**85.982**	**0.89**	**67.063**	**9.98**	**0.15**
**Dia(1,1,1)**	**Si(0,2,10)**	**60.17**	**86.96**	**120.564**	**1.39**	**62.623**	**15.94**	**0.25**
**Dia(1,1,1)**	**Si(1,5,9)**	**61.63**	**84.14**	**71.376**	**0.85**	**57.553**	**7.49**	**0.13**
**Dia(1,1,1)**	**Si(3,5,9)**	**65.81**	**70.55**	**59.676**	**0.85**	**47.996**	**6.4**	**0.13**
**Dia(1,1,1)**	**Si(2,4,10)**	**68.72**	**60.59**	**86.308**	**1.42**	**42.512**	**10.28**	**0.24**
**Dia(1,1,1)**	**Si(1,1,11)**	**70.63**	**55.48**	**50.128**	**0.9**	**37.905**	**4.77**	**0.13**
**Dia(1,1,1)**	**Si(0,8,8)**	**74.23**	**44.13**	**74.241**	**1.68**	**31.375**	**8.43**	**0.27**
**Dia(1,1,1)**	**Si(1,3,11)**	**76.8**	**37.4**	**42.912**	**1.15**	**25.561**	**4.46**	**0.17**
**Dia(1,1,1)**	**Si(0,6,10)**	**82.74**	**19.85**	**64.362**	**3.24**	**4.691**	**10.03**	**2.14**

Ei = 5.483 keV			Cryst 1-2			Cryst 1-2-3		
Cryst 1	Cryst 2,3	ΘB 2 [°]	ΔE [meV]	∫IR dΘdE [µrad meV]	FOM	ΔE [meV]	∫IR dΘdE [µrad meV]	FOM
Si(1,1,1)	Si(1,1,1)	21.14	517.58	5277.127	10.2	1469.45	3540.61	2.41
Si(1,1,1)	Si(0,2,2)	36.07	285.94	3080.881	10.77	207.312	1758.35	8.48
Si(1,1,1)	Si(1,1,3)	43.67	154.41	1255.214	8.13	92.153	449.5	4.88
Si(1,1,1)	Si(0,0,4)	56.38	126.83	1278.081	10.08	84.533	616.63	7.29
Si(1,1,1)	Si(1,3,3)	65.15	74.68	587.445	7.87	45.17	204.8	4.53
Dia(1,1,1)	Si(1,1,1)	21.14	320.06	4107.653	12.83	313.158	2874.6	9.18
Dia(1,1,1)	Si(0,2,2)	36.07	237.45	3212.364	13.53	208.919	2271	10.87
Dia(1,1,1)	Si(1,1,3)	43.67	143.6	1505.817	10.49	92.887	611.69	6.59
Dia(1,1,1)	Si(0,0,4)	56.38	122.7	1612.654	13.14	84.963	841	9.9
Dia(1,1,1)	Si(1,3,3)	65.15	73.67	770.692	10.46	46.698	278.46	5.96

Ei = 5.723 keV			Cryst 1-2			Cryst 1-2-3		
Cryst 1	Cryst 2,3	ΘB 2 [°]	ΔE [meV]	∫IR dΘdE [µrad meV]	FOM	ΔE [meV]	∫IR dΘdE [µrad meV]	FOM
Si(1,1,1)	Si(1,1,1)	20.21	544.61	5654.852	10.38	489.72	3923.25	8.01
Si(1,1,1)	Si(0,2,2)	34.34	301.17	3283.277	10.9	218.926	1884.51	8.61
Si(1,1,1)	Si(1,1,3)	41.41	164.93	1359.721	8.24	96.188	490.55	5.1
Si(1,1,1)	Si(0,0,4)	52.92	135.72	1373.777	10.12	87.933	648.31	7.37
Si(1,1,1)	Si(1,3,3)	60.39	83.11	640.232	7.7	47.448	208.1	4.39
Si(1,1,1)	Si(2,2,4)	77.71	73.51	770.567	10.48	53.511	431.75	8.07
Dia(1,1,1)	Si(1,1,1)	20.21	334.15	4311.802	12.9	326.707	3088.58	9.45
Dia(1,1,1)	Si(0,2,2)	34.34	247.58	3321.186	13.41	219.22	2399.98	10.95
Dia(1,1,1)	Si(1,1,3)	41.41	151.11	1572.741	10.41	96.494	651.62	6.75
Dia(1,1,1)	Si(0,0,4)	52.92	129.48	1668.698	12.89	88.675	854.37	9.63
Dia(1,1,1)	Si(1,3,3)	60.39	81.23	808.6	9.95	47.39	276.35	5.83
Dia(1,1,1)	Si(2,2,4)	77.71	73.41	1001.765	13.65	53.851	572.07	10.62

Ei = 5.964 keV			Cryst 1-2			Cryst 1-2-3		
Cryst 1	Cryst 2,3	ΘB 2 [°]	ΔE [meV]	∫IR dΘdE [µrad meV]	FOM	ΔE [meV]	∫IR dΘdE [µrad meV]	FOM
Si(1,1,1)	Si(1,1,1)	19.36	571.34	6022.11	10.54	512.832	4247.3	8.28
Si(1,1,1)	Si(0,2,2)	32.77	317.16	3483.095	10.98	229.916	2019.81	8.78
Si(1,1,1)	Si(1,1,3)	39.4	175.69	1461.847	8.32	100.632	525.9	5.23
Si(1,1,1)	Si(0,0,4)	49.96	144.67	1465.382	10.13	91.445	676.86	7.4
Si(1,1,1)	Si(1,3,3)	56.54	91.87	692.378	7.54	48.097	214.8	4.47
Si(1,1,1)	Si(2,2,4)	69.65	80.35	824.715	10.26	54.679	407.49	7.45
Si(1,1,1)	Si(3,3,3)	83.98	45.6	403.432	8.85	34.385	195.7	5.69
Dia(1,1,1)	Si(1,1,1)	19.36	348.34	4506.206	12.94	342.349	3301.16	9.64
Dia(1,1,1)	Si(0,2,2)	32.77	256.84	3427.31	13.34	230.823	2515.04	10.9
Dia(1,1,1)	Si(1,1,3)	39.4	158.55	1636.517	10.32	100.409	689.98	6.87
Dia(1,1,1)	Si(0,0,4)	49.96	136.06	1719.473	12.64	91.927	876.82	9.54
Dia(1,1,1)	Si(1,3,3)	56.54	88.86	845.981	9.52	48.869	278.74	5.7
Dia(1,1,1)	Si(2,2,4)	69.65	79.16	1034.173	13.06	55.024	530.55	9.64
Dia(1,1,1)	Si(3,3,3)	83.98	45.54	519	11.4	34.505	254.22	7.37

Ei = 6.208 keV			Cryst 1-2			Cryst 1-2-3		
Cryst 1	Cryst 2,3	ΘB 2 [°]	ΔE [meV]	∫IR dΘdE [µrad meV]	FOM	ΔE [meV]	∫IR dΘdE [µrad meV]	FOM
Si(1,1,1)	Si(1,1,1)	18.57	597.87	6385.443	10.68	541.398	4588.47	8.48
Si(1,1,1)	Si(0,2,2)	31.34	332.42	3679.497	11.07	242.272	2144.62	8.85
Si(1,1,1)	Si(1,1,3)	37.58	186.64	1563.679	8.38	104.509	564.53	5.4
Si(1,1,1)	Si(0,0,4)	47.35	153.86	1556.088	10.11	95.494	701	7.34
Si(1,1,1)	Si(1,3,3)	53.27	100.88	744.614	7.38	50.379	221.22	4.39
Si(1,1,1)	Si(2,2,4)	64.26	87.57	879.05	10.04	55.784	396.59	7.11
Si(1,1,1)	Si(3,3,3)	72.82	52.94	435.718	8.23	32.267	155.55	4.82
Dia(1,1,1)	Si(1,1,1)	18.57	361.97	4691.722	12.96	357.423	3515.38	9.84
Dia(1,1,1)	Si(0,2,2)	31.34	265.5	3528.427	13.29	239.718	2633.59	10.99
Dia(1,1,1)	Si(1,1,3)	37.58	165.92	1695.694	10.22	104.48	728.76	6.98
Dia(1,1,1)	Si(0,0,4)	47.35	142.71	1767.439	12.39	95.705	894.76	9.35
Dia(1,1,1)	Si(1,3,3)	53.27	96.57	879.872	9.11	50.236	282.67	5.63
Dia(1,1,1)	Si(2,2,4)	64.26	85.1	1067.212	12.54	55.911	509.27	9.11
Dia(1,1,1)	Si(3,3,3)	72.82	51.99	540.189	10.39	32.205	198.85	6.17

Ei = 6.459 keV				Cryst 1-2			Cryst 1-2-3	
Cryst 1	Cryst 2,3	ΘB 2 [°]	ΔE [meV]	∫IR dΘdE [µrad meV]	FOM	ΔE [meV]	∫IR dΘdE [µrad meV]	FOM
Si(1,1,1)	Si(1,1,1)	17.82	625.12	6745.575	10.79	2294.63	4929.7	2.15
Si(1,1,1)	Si(0,2,2)	29.99	348.02	3874.469	11.13	251.512	2290.13	9.11
Si(1,1,1)	Si(1,1,3)	35.88	198.1	1665.742	8.41	110.827	602.35	5.44
Si(1,1,1)	Si(0,0,4)	44.98	163.48	1645.599	10.07	98.615	723.65	7.34
Si(1,1,1)	Si(1,3,3)	50.38	110.37	797.548	7.23	52.057	230.28	4.42
Si(1,1,1)	Si(2,2,4)	59.97	95.39	932.985	9.78	56.533	390.45	6.91
Si(1,1,1)	Si(1,1,5)	66.67	62.36	468.62	7.52	32.948	142.02	4.31
Si(1,1,1)	Si(3,3,3)	66.67	62.36	468.62	7.52	32.948	142.02	4.31
Si(1,1,1)	Si(0,4,4)	88.57	52.84	585.904	11.09	39.876	381.65	9.57
Dia(1,1,1)	Si(1,1,1)	17.82	376.81	4883.229	12.96	2489.23	3744.09	1.5
Dia(1,1,1)	Si(0,2,2)	29.99	274.32	3626.907	13.22	988.265	2754.43	2.79
Dia(1,1,1)	Si(1,1,3)	35.88	173.44	1752.548	10.1	109.697	754.62	6.88
Dia(1,1,1)	Si(0,0,4)	44.98	149.73	1814.572	12.12	98.555	915.45	9.29
Dia(1,1,1)	Si(1,3,3)	50.38	104.42	913.718	8.75	52.034	290.47	5.58
Dia(1,1,1)	Si(2,2,4)	59.97	91.49	1096.965	11.99	56.488	489.68	8.67
Dia(1,1,1)	Si(1,1,5)	66.67	60.67	562.026	9.26	32.329	180.52	5.58
Dia(1,1,1)	Si(3,3,3)	66.67	60.67	562.026	9.26	32.329	180.52	5.58
Dia(1,1,1)	Si(0,4,4)	88.57	53.05	725.358	13.67	40.33	474.53	11.77

Ei = 6.716 keV			Cryst 1-2			Cryst 1-2-3		
Cryst 1	Cryst 2,3	ΘB 2 [°]	ΔE [meV]	∫IR dΘdE [µrad meV]	FOM	ΔE [meV]	∫IR dΘdE [µrad meV]	FOM
Si(1,1,1)	Si(1,1,1)	17.12	652.47	7103.217	10.89	2333.04	5284.6	2.27
Si(1,1,1)	Si(0,2,2)	28.73	363.67	4068.814	11.19	260.065	2411.22	9.27
Si(1,1,1)	Si(1,1,3)	34.31	209.86	1767.274	8.42	113.592	638.67	5.62
Si(1,1,1)	Si(0,0,4)	42.83	173.27	1736.636	10.02	102.098	759.06	7.43
Si(1,1,1)	Si(1,3,3)	47.8	120.28	851.078	7.08	54.161	236.82	4.37
Si(1,1,1)	Si(2,2,4)	56.37	103.92	987.556	9.5	58.932	392.96	6.67
Si(1,1,1)	Si(1,1,5)	62.02	72.13	501.649	6.95	33.263	136.87	4.11
Si(1,1,1)	Si(0,4,4)	74.04	61.19	632.339	10.33	40.655	305.98	7.53
Dia(1,1,1)	Si(1,1,1)	17.12	391.59	5063.611	12.93	2613.82	3958.61	1.51
Dia(1,1,1)	Si(0,2,2)	28.73	283.19	3724.609	13.15	1040.62	2866.87	2.75
Dia(1,1,1)	Si(1,1,3)	34.31	181	1806.592	9.98	112.522	786.14	6.99
Dia(1,1,1)	Si(0,0,4)	42.83	156.64	1861.801	11.89	102.652	924.97	9.01
Dia(1,1,1)	Si(1,3,3)	47.8	112.24	946.947	8.44	53.686	296.19	5.52
Dia(1,1,1)	Si(2,2,4)	56.37	98.53	1126.3	11.43	59.278	485.46	8.19
Dia(1,1,1)	Si(1,1,5)	62.02	69.69	583.476	8.37	32.954	169	5.13
Dia(1,1,1)	Si(0,4,4)	74.04	60	752.063	12.53	40.615	377.57	9.3

Ei = 6.977 keV			Cryst 1-2			Cryst 1-2-3		
Cryst 1	Cryst 2,3	ΘB 2 [°]	ΔE [meV]	∫IR dΘdE [μrad meV]	FOM	ΔE [meV]	∫IR dΘdE [μrad meV]	FOM
Si(1,1,1)	Si(1,1,1)	16.46	679.89	7455.869	10.97	2433.64	5643.39	2.32
Si(1,1,1)	Si(0,2,2)	27.56	379.46	4258.327	11.22	271.536	2535.26	9.34
Si(1,1,1)	Si(1,1,3)	32.86	222.05	1867.732	8.41	116.494	674.31	5.79
Si(1,1,1)	Si(0,0,4)	40.87	183.38	1824.112	9.95	106.509	791.04	7.43
Si(1,1,1)	Si(1,3,3)	45.49	130.44	903.702	6.93	56.109	245.26	4.37
Si(1,1,1)	Si(2,2,4)	53.27	112.83	1041.417	9.23	61.518	395.92	6.44
Si(1,1,1)	Si(1,1,5)	58.22	82.02	534.728	6.52	34.417	132.85	3.86
Si(1,1,1)	Si(3,3,3)	58.22	82.02	534.728	6.52	34.417	132.85	3.86
Si(1,1,1)	Si(0,4,4)	67.74	69.1	668.542	9.67	40.576	270.76	6.67
Si(1,1,1)	Si(1,3,5)	75.44	42.89	347.072	8.09	24.404	116.5	4.77
Dia(1,1,1)	Si(1,1,1)	16.46	406.58	5238.145	12.88	2742.17	4176.81	1.52
Dia(1,1,1)	Si(0,2,2)	27.56	292.11	3812.853	13.05	1091.84	2969.88	2.72
Dia(1,1,1)	Si(1,1,3)	32.86	188.66	1859.451	9.86	117.432	823.69	7.01
Dia(1,1,1)	Si(0,0,4)	40.87	163.66	1907.194	11.65	106.974	953.66	8.91
Dia(1,1,1)	Si(1,3,3)	45.49	120.21	976.647	8.12	55.974	299.33	5.35
Dia(1,1,1)	Si(2,2,4)	53.27	105.63	1154.474	10.93	60.974	484.64	7.95
Dia(1,1,1)	Si(1,1,5)	58.22	78.58	604.449	7.69	34.514	162.09	4.7
Dia(1,1,1)	Si(3,3,3)	58.22	78.58	604.449	7.69	34.514	162.09	4.7
Dia(1,1,1)	Si(0,4,4)	67.74	66.7	770.528	11.55	40.405	333.03	8.24
Dia(1,1,1)	Si(1,3,5)	75.44	41.7	406.928	9.76	24.518	141.88	5.79

Ei = 7.243 keV			Cryst 1-2			Cryst 1-2-3		
Cryst 1	Cryst 2,3	ΘB 2 [°]	ΔE [meV]	∫IR dΘdE [µrad meV]	FOM	ΔE [meV]	∫IR dΘdE [µrad meV]	FOM
Si(1,1,1)	Si(1,1,1)	15.84	706.33	7797.808	11.04	2533.72	5997.32	2.37
Si(1,1,1)	Si(0,2,2)	26.47	395.22	4444.164	11.24	281.131	2646.78	9.41
Si(1,1,1)	Si(1,1,3)	31.51	234.55	1966.724	8.38	120.569	705.77	5.85
Si(1,1,1)	Si(0,0,4)	39.08	194.13	1912.074	9.85	109.797	823.71	7.5
Si(1,1,1)	Si(1,3,3)	43.39	141.02	955.829	6.78	58.19	253.31	4.35
Si(1,1,1)	Si(2,2,4)	50.54	122.19	1094.596	8.96	62.745	398.47	6.35
Si(1,1,1)	Si(1,1,5)	54.97	92	567.58	6.17	36.224	132.03	3.64
Si(1,1,1)	Si(0,4,4)	63.06	78.21	704.183	9	41.752	257.61	6.17
Si(1,1,1)	Si(1,3,5)	68.8	54.4	369.537	6.79	24.208	97.08	4.01
Si(1,1,1)	Si(0,2,6)	85.35	44.5	483.767	10.87	34.535	298.96	8.66
Dia(1,1,1)	Si(1,1,1)	15.84	421.77	5406.89	12.82	2874.82	4342.09	1.51
Dia(1,1,1)	Si(0,2,2)	26.47	301.31	3898.733	12.94	1146.18	3073.53	2.68
Dia(1,1,1)	Si(1,1,3)	31.51	196.36	1910.934	9.73	122.518	858.16	7
Dia(1,1,1)	Si(0,0,4)	39.08	170.67	1948.983	11.42	111.561	981.11	8.79
Dia(1,1,1)	Si(1,3,3)	43.39	128.09	1007.188	7.86	58.39	304.54	5.22
Dia(1,1,1)	Si(2,2,4)	50.54	112.84	1180.487	10.46	62.439	482.92	7.73
Dia(1,1,1)	Si(1,1,5)	54.97	87.29	624.178	7.15	36.062	158.9	4.41
Dia(1,1,1)	Si(0,4,4)	63.06	74.35	788.611	10.61	41.476	311.89	7.52
Dia(1,1,1)	Si(1,3,5)	68.8	52.48	419.895	8	24.094	117.79	4.89
Dia(1,1,1)	Si(0,2,6)	85.35	44.32	564.922	12.75	34.483	355.41	10.31

Ei = 7.514 keV				Cryst 1-2			Cryst 1-2-3	
Cryst 1	Cryst 2,3	ΘB 2 [°]	ΔE [meV]	∫IR dΘdE [µrad meV]	FOM	ΔE [meV]	∫IR dΘdE [µrad meV]	FOM
Si(1,1,1)	Si(1,1,1)	15.25	734.18	8145.531	11.09	2641.18	6346.07	2.4
Si(1,1,1)	Si(0,2,2)	25.45	411.2	4624.928	11.25	289.999	2757.24	9.51
Si(1,1,1)	Si(1,1,3)	30.25	247.46	2063.756	8.34	125.098	747.28	5.97
Si(1,1,1)	Si(0,0,4)	37.42	205.09	1999.208	9.75	113.591	850.03	7.48
Si(1,1,1)	Si(1,3,3)	41.46	151.67	1007.584	6.64	60.526	261.63	4.32
Si(1,1,1)	Si(2,2,4)	48.09	131.89	1146.792	8.7	63.527	404.01	6.36
Si(1,1,1)	Si(1,1,5)	52.12	102.19	600.482	5.88	37.948	134.17	3.54
Si(1,1,1)	Si(0,4,4)	59.24	87.74	739.588	8.43	42.101	249.25	5.92
Si(1,1,1)	Si(1,3,5)	63.99	65.52	392.034	5.98	25.494	87.78	3.44
Si(1,1,1)	Si(0,2,6)	73.9	52.14	509.016	9.76	30.904	210.82	6.82
Si(1,1,1)	Si(3,3,5)	84.96	29.08	270.488	9.3	21.179	129.54	6.12
Dia(1,1,1)	Si(1,1,1)	15.25	437.32	5568.057	12.73	3011.82	4538.55	1.51
Dia(1,1,1)	Si(0,2,2)	25.45	310.64	3978.503	12.81	1203.93	3162.09	2.63
Dia(1,1,1)	Si(1,1,3)	30.25	204.26	1960.632	9.6	127.127	894.52	7.04
Dia(1,1,1)	Si(0,0,4)	37.42	177.84	1986.635	11.17	114.476	1000.15	8.74
Dia(1,1,1)	Si(1,3,3)	41.46	136	1036.437	7.62	60.677	310.79	5.12
Dia(1,1,1)	Si(2,2,4)	48.09	120.14	1205.047	10.03	64.659	485.4	7.51
Dia(1,1,1)	Si(1,1,5)	52.12	95.95	642.595	6.7	37.731	160.04	4.24
Dia(1,1,1)	Si(0,4,4)	59.24	82.33	807.047	9.8	41.804	294.57	7.05
Dia(1,1,1)	Si(1,3,5)	63.99	62.97	433.608	6.89	25.455	104.59	4.11
Dia(1,1,1)	Si(0,2,6)	73.9	50.3	573.245	11.4	30.921	250.87	8.11
Dia(1,1,1)	Si(3,3,5)	84.96	28.83	310.779	10.78	21.88	153.21	7

Ei = 7.79 keV			Cryst 1-2			Cryst 1-2-3		
Cryst 1	Cryst 2,3	ΘB 2 [°]	ΔE [meV]	∫IR dΘdE [µrad meV]	FOM	ΔE [meV]	∫IR dΘdE [µrad meV]	FOM
Si(1,1,1)	Si(1,1,1)	14.7	760.48	8483.84	11.16	703.052	6706.63	9.54
Si(1,1,1)	Si(0,2,2)	24.48	427.19	4804.8	11.25	301.379	2872.84	9.53
Si(1,1,1)	Si(1,1,3)	29.08	260.69	2159.828	8.28	129.125	779.21	6.03
Si(1,1,1)	Si(0,0,4)	35.88	216.59	2091.677	9.66	116.054	882.67	7.61
Si(1,1,1)	Si(1,3,3)	39.69	162.74	1058.287	6.5	63.205	270.66	4.28
Si(1,1,1)	Si(2,2,4)	45.88	141.62	1197.889	8.46	65.781	411.45	6.25
Si(1,1,1)	Si(1,1,5)	49.59	112.5	632.994	5.63	39.483	137.89	3.49
Si(1,1,1)	Si(0,4,4)	55.98	97.28	774.411	7.96	42.58	242.9	5.7
Si(1,1,1)	Si(1,3,5)	60.1	76.35	414.501	5.43	26.515	86.63	3.27
Si(1,1,1)	Si(0,2,6)	67.93	62.42	534.102	8.56	31.054	182.26	5.87
Si(1,1,1)	Si(3,3,5)	73.91	42.25	286.379	6.78	18.716	75.35	4.03
Dia(1,1,1)	Si(1,1,1)	14.7	449.43	5746.794	12.79	3153.28	4803.05	1.52
Dia(1,1,1)	Si(0,2,2)	24.48	319.75	4054.908	12.68	1265.96	3248.17	2.57
Dia(1,1,1)	Si(1,1,3)	29.08	212.11	2009.162	9.47	131.558	927.76	7.05
Dia(1,1,1)	Si(0,0,4)	35.88	185.21	2021.887	10.92	117.447	1024.65	8.72
Dia(1,1,1)	Si(1,3,3)	39.69	143.92	1064.002	7.39	63.081	318.36	5.05
Dia(1,1,1)	Si(2,2,4)	45.88	127.56	1228.689	9.63	67.267	493.19	7.33
Dia(1,1,1)	Si(1,1,5)	49.59	104.58	661.093	6.32	39.184	163.13	4.16
Dia(1,1,1)	Si(0,4,4)	55.98	90.43	824.662	9.12	42.281	288.53	6.82
Dia(1,1,1)	Si(1,3,5)	60.1	72.99	447.291	6.13	26.688	101.1	3.79
Dia(1,1,1)	Si(0,2,6)	67.93	59.19	584.708	9.88	30.905	215.26	6.97
Dia(1,1,1)	Si(3,3,5)	73.91	40.56	318.221	7.85	18.403	89.44	4.86

Ei = 8.071 keV			Cryst 1-2			Cryst 1-2-3		
Cryst 1	Cryst 2,3	ΘB 2	ΔE	∫IR dΘdE	FOM	ΔE	∫IR dΘdE	FOM
		[°]	[meV]	[µrad meV]		[meV]	[µrad meV]	
Si(1,1,1)	Si(1,1,1)	14.18	786.61	8813.653	11.2	729.577	7049.22	9.66
Si(1,1,1)	Si(0,2,2)	23.58	443.9	4947.584	11.15	311.457	2970.06	9.54
Si(1,1,1)	Si(1,1,3)	27.97	274.27	2253.902	8.22	133.16	808.86	6.07
Si(1,1,1)	Si(0,0,4)	34.45	227.92	2167.345	9.51	118.934	908.53	7.64
Si(1,1,1)	Si(1,3,3)	38.06	174.24	1108.46	6.36	65.833	280.69	4.26
Si(1,1,1)	Si(2,2,4)	43.86	151.96	1249.268	8.22	67.478	416.41	6.17
Si(1,1,1)	Si(1,1,5)	47.3	122.83	664.511	5.41	39.924	145.04	3.63
Si(1,1,1)	Si(3,3,3)	47.3	122.83	664.511	5.41	39.924	145.04	3.63
Si(1,1,1)	Si(0,4,4)	53.13	107.19	809.321	7.55	43.924	235.76	5.37
Si(1,1,1)	Si(1,3,5)	56.79	87	437.214	5.03	27.523	86.19	3.13
Si(1,1,1)	Si(0,2,6)	63.44	72.97	558.631	7.66	30.896	166.84	5.4
Si(1,1,1)	Si(3,3,5)	68.03	55.26	302.642	5.48	20.047	62.41	3.11
Si(1,1,1)	Si(4,4,4)	78.47	40.52	402.967	9.95	24.538	175.43	7.15
Dia(1,1,1)	Si(1,1,1)	14.18	469.17	5876.637	12.53	3299.22	4991.9	1.51
Dia(1,1,1)	Si(0,2,2)	23.58	329.25	4121.951	12.52	1331.93	3323.07	2.49
Dia(1,1,1)	Si(1,1,3)	27.97	220.23	2055.054	9.33	134.121	945.42	7.05
Dia(1,1,1)	Si(0,0,4)	34.45	192.48	2056.211	10.68	120.763	1048.28	8.68
Dia(1,1,1)	Si(1,3,3)	38.06	151.88	1088.591	7.17	65.59	327.06	4.99
Dia(1,1,1)	Si(2,2,4)	43.86	134.98	1254.856	9.3	66.509	476.21	7.16
Dia(1,1,1)	Si(1,1,5)	47.3	113.03	679.391	6.01	40.59	165.29	4.07
Dia(1,1,1)	Si(3,3,3)	47.3	113.03	679.391	6.01	40.59	165.29	4.07
Dia(1,1,1)	Si(0,4,4)	53.13	98.49	840.915	8.54	43.799	276.85	6.32
Dia(1,1,1)	Si(1,3,5)	56.79	82.47	460.121	5.58	28.059	98.49	3.51
Dia(1,1,1)	Si(0,2,6)	63.44	68.48	597.219	8.72	30.683	194.31	6.33
Dia(1,1,1)	Si(3,3,5)	68.03	53.06	326.854	6.16	19.935	72.87	3.66
Dia(1,1,1)	Si(4,4,4)	78.47	39.14	443.305	11.33	24.695	203.16	8.23

Ei = 8.358 keV				Cryst 1-2			Cryst 1-2-3	
Cryst 1	Cryst 2,3	ΘB 2 [°]	ΔE [meV]	∫IR dΘdE [µrad meV]	FOM	ΔE [meV]	∫IR dΘdE [µrad meV]	FOM
Si(1,1,1)	Si(1,1,1)	13.68	812.69	9139.206	11.25	754.961	7369.65	9.76
Si(1,1,1)	Si(0,2,2)	22.72	460.01	5120.857	11.13	322.37	3073.63	9.53
Si(1,1,1)	Si(1,1,3)	26.93	288.31	2347.315	8.14	137.697	840.4	6.1
Si(1,1,1)	Si(0,0,4)	33.11	239.92	2246.993	9.37	121.31	921.85	7.6
Si(1,1,1)	Si(1,3,3)	36.53	186.22	1158.447	6.22	68.515	291.39	4.25
Si(1,1,1)	Si(2,2,4)	41.99	162.62	1299.141	7.99	68.647	419.23	6.11
Si(1,1,1)	Si(1,1,5)	45.21	133.87	697.084	5.21	41.934	146.28	3.49
Si(1,1,1)	Si(3,3,3)	45.21	133.87	697.083	5.21	41.934	146.28	3.49
Si(1,1,1)	Si(0,4,4)	50.58	117.47	843.065	7.18	45.937	235.72	5.13
Si(1,1,1)	Si(1,3,5)	53.9	97.67	458.275	4.69	28.58	84.89	2.97
Si(1,1,1)	Si(0,2,6)	59.74	83.64	583.273	6.97	32.432	155.8	4.8
Si(1,1,1)	Si(3,3,5)	63.58	67.3	319.243	4.74	20.506	59.18	2.89
Si(1,1,1)	Si(4,4,4)	71.12	52.74	420.98	7.98	24.163	132.69	5.49
Si(1,1,1)	Si(1,1,7)	77.24	34.4	229.588	6.67	15.027	59.62	3.97
Dia(1,1,1)	Si(1,1,1)	13.68	484.58	6019.54	12.42	3450.34	5179.48	1.5
Dia(1,1,1)	Si(0,2,2)	22.72	339.82	4191.381	12.33	1400.08	3411.69	2.44
Dia(1,1,1)	Si(1,1,3)	26.93	228.6	2097.85	9.18	136.267	958.01	7.03
Dia(1,1,1)	Si(0,0,4)	33.11	199.92	2089.809	10.45	123.954	1064.7	8.59
Dia(1,1,1)	Si(1,3,3)	36.53	159.38	1111.074	6.97	68.132	335.92	4.93
Dia(1,1,1)	Si(2,2,4)	41.99	142.54	1277.744	8.96	68.88	474.78	6.89
Dia(1,1,1)	Si(1,1,5)	45.21	121.38	696.788	5.74	42.466	166.74	3.93
Dia(1,1,1)	Si(3,3,3)	45.21	121.38	696.787	5.74	42.466	166.74	3.93
Dia(1,1,1)	Si(0,4,4)	50.58	106.49	856.802	8.05	45.394	272.27	6
Dia(1,1,1)	Si(1,3,5)	53.9	91.76	472.021	5.14	28.453	97.67	3.43
Dia(1,1,1)	Si(0,2,6)	59.74	77.46	609.024	7.86	31.976	182.55	5.71
Dia(1,1,1)	Si(3,3,5)	63.58	64.32	336.088	5.23	20.88	67.1	3.21
Dia(1,1,1)	Si(4,4,4)	71.12	49.6	449.963	9.07	24.062	153.49	6.38
Dia(1,1,1)	Si(1,1,7)	77.24	32.76	248.582	7.59	14.902	69.11	4.64

Ei = 8.648 keV			Cryst 1-2			Cryst 1-2-3		
Cryst 1	Cryst 2,3	ΘB 2 [°]	ΔE [meV]	∫IR dΘdE [μrad meV]	FOM	ΔE [meV]	∫IR dΘdE [μrad meV]	FOM
Si(1,1,1)	Si(1,1,1)	13.22	838.58	9441.923	11.26	781.365	7694.83	9.85
Si(1,1,1)	Si(0,2,2)	21.92	476.1	5299.687	11.13	332.793	3167.34	9.52
Si(1,1,1)	Si(1,1,3)	25.96	302.59	2438.007	8.06	141.49	864.99	6.11
Si(1,1,1)	Si(0,0,4)	31.87	252.14	2323.044	9.21	126.904	952.55	7.51
Si(1,1,1)	Si(1,3,3)	35.12	198.49	1206.815	6.08	70.828	302.74	4.27
Si(1,1,1)	Si(2,2,4)	40.29	173.6	1344.617	7.75	70.999	423.84	5.97
Si(1,1,1)	Si(1,1,5)	43.3	144.51	728.903	5.04	43.267	147.91	3.42
Si(1,1,1)	Si(0,4,4)	48.3	127.78	876.572	6.86	47.588	237.1	4.98
Si(1,1,1)	Si(1,3,5)	51.34	108.33	480.209	4.43	29.125	84.59	2.9
Si(1,1,1)	Si(0,2,6)	56.59	93.99	607.491	6.46	34.288	151.97	4.43
Si(1,1,1)	Si(3,3,5)	59.94	78.75	334.169	4.24	21.09	55.56	2.63
Si(1,1,1)	Si(4,4,4)	66.13	64.64	439.157	6.79	24.882	113.93	4.58
Si(1,1,1)	Si(1,1,7)	70.49	49.4	241.596	4.89	15.719	46.49	2.96
Si(1,1,1)	Si(1,5,5)	70.49	49.4	241.596	4.89	15.719	46.49	2.96
Si(1,1,1)	Si(2,4,6)	81.01	33.04	327.679	9.92	20.256	143.59	7.09
Dia(1,1,1)	Si(1,1,1)	13.22	502.24	6160.332	12.27	3605.05	5347.72	1.48
Dia(1,1,1)	Si(0,2,2)	21.92	349.91	4255.498	12.16	1470.38	3491.66	2.37
Dia(1,1,1)	Si(1,1,3)	25.96	237.12	2138.264	9.02	140.026	976.12	6.97
Dia(1,1,1)	Si(0,0,4)	31.87	207.31	2122.052	10.24	127.329	1079.96	8.48
Dia(1,1,1)	Si(1,3,3)	35.12	167.92	1132.795	6.75	70.626	345.11	4.89
Dia(1,1,1)	Si(2,2,4)	40.29	150.11	1298.911	8.65	71.503	479.37	6.7
Dia(1,1,1)	Si(1,1,5)	43.3	129.67	713.425	5.5	43.184	169.31	3.92
Dia(1,1,1)	Si(0,4,4)	48.3	114.47	872.598	7.62	47.671	271.01	5.69
Dia(1,1,1)	Si(1,3,5)	51.34	100.63	483.595	4.81	29.258	96.9	3.31
Dia(1,1,1)	Si(0,2,6)	56.59	86.55	619.736	7.16	34.131	174.29	5.11
Dia(1,1,1)	Si(3,3,5)	59.94	74.35	344.384	4.63	21.05	63.44	3.01
Dia(1,1,1)	Si(4,4,4)	66.13	60.43	458.133	7.58	24.454	132.22	5.41
Dia(1,1,1)	Si(1,1,7)	70.49	47.41	254.618	5.37	16.021	52.15	3.25
Dia(1,1,1)	Si(1,5,5)	70.49	47.41	254.618	5.37	16.021	52.15	3.25
Dia(1,1,1)	Si(2,4,6)	81.01	31.84	351.687	11.05	20.231	163.88	8.1

Ei = 8.944 keV			Cryst 1-2			Cryst 1-2-3		
Cryst 1	Cryst 2,3	ΘB 2 [°]	ΔE [meV]	∫IR dΘdE [μrad meV]	FOM	ΔE [meV]	∫IR dΘdE [μrad meV]	FOM
Si(1,1,1)	Si(1,1,1)	12.77	864.41	9761.292	11.29	803.273	8041.96	10.01
Si(1,1,1)	Si(0,2,2)	21.16	492.8	5462.637	11.08	347.483	3277.29	9.43
Si(1,1,1)	Si(1,1,3)	25.04	317.17	2525.367	7.96	148.529	895.34	6.03
Si(1,1,1)	Si(0,0,4)	30.7	264.73	2404.284	9.08	132.157	945.07	7.15
Si(1,1,1)	Si(1,3,3)	33.8	210.97	1254.176	5.94	70.208	307.1	4.37
Si(1,1,1)	Si(2,2,4)	38.7	184.65	1396.498	7.56	73.333	435.54	5.94
Si(1,1,1)	Si(3,3,3)	41.54	156.32	759.332	4.86	42.544	146.97	3.45
Si(1,1,1)	Si(0,4,4)	46.21	138.48	908.518	6.56	48.425	239	4.94
Si(1,1,1)	Si(1,3,5)	49.03	119.12	502.742	4.22	30.146	82.46	2.74
Si(1,1,1)	Si(0,2,6)	53.82	104.62	630.905	6.03	33.783	148.21	4.39
Si(1,1,1)	Si(3,3,5)	56.81	89.53	350.702	3.92	21.687	53.09	2.45
Si(1,1,1)	Si(4,4,4)	62.15	76.05	457.099	6.01	24.965	105.14	4.21
Si(1,1,1)	Si(1,1,7)	65.7	62.41	253.787	4.07	16.252	38.79	2.39
Si(1,1,1)	Si(2,4,6)	72.75	47.56	341.217	7.17	19.662	95.36	4.85
Si(1,1,1)	Si(1,3,7)	78.6	30.8	188.563	6.12	12.415	44.72	3.6
Dia(1,1,1)	Si(1,1,1)	12.77	519.15	6294.28	12.12	2352.59	5432	2.31
Dia(1,1,1)	Si(0,2,2)	21.16	359.66	4315.12	12	335.93	3596.79	10.71
Dia(1,1,1)	Si(1,1,3)	25.04	245.74	2177.811	8.86	150.103	1020.44	6.8
Dia(1,1,1)	Si(0,0,4)	30.7	214.72	2156.215	10.04	132.465	1082.17	8.17
Dia(1,1,1)	Si(1,3,3)	33.8	175.89	1157.882	6.58	70.253	348.33	4.96
Dia(1,1,1)	Si(2,2,4)	38.7	157.74	1318.178	8.36	74.929	494.9	6.6
Dia(1,1,1)	Si(3,3,3)	41.54	137.94	728.876	5.28	43.326	164.52	3.8
Dia(1,1,1)	Si(0,4,4)	46.21	122.43	888.246	7.26	47.993	270.72	5.64
Dia(1,1,1)	Si(1,3,5)	49.03	109.16	495.441	4.54	30.173	93.38	3.09
Dia(1,1,1)	Si(0,2,6)	53.82	95.28	630.121	6.61	33.712	167.62	4.97
Dia(1,1,1)	Si(3,3,5)	56.81	84.38	353.106	4.18	21.813	59.88	2.74
Dia(1,1,1)	Si(4,4,4)	62.15	70.66	465.738	6.59	25.12	120.04	4.78
Dia(1,1,1)	Si(1,1,7)	65.7	59.74	260.92	4.37	16.254	43.96	2.7
Dia(1,1,1)	Si(2,4,6)	72.75	44.46	355.549	8	19.68	108.18	5.5
Dia(1,1,1)	Si(1,3,7)	78.6	29.09	198.716	6.83	12.162	51.21	4.21
