# Supplementary material for: Spherical analyzers and monochromators for resonant inelastic hard X-ray scattering: a compilation of crystals and reflections
Source: J Synchrotron Radiat. 2012 Nov 10;20(Pt 1):74–9. doi: 10.1107/S0909049512043154 (PMC3943536; doi:10.1107/S0909049512043154)
Supplement: Supplementary file 1 [file s-20-00074-sup1.pdf]

Argonne NATIONAL LABORATORY

Advanced Photon Source
A U.S. Department of Energy, Office of Science,
Office of Basic Energy Sciences national synchrotron x-ray research facility

U.S. DEPARTMENT OF ENERGY | Office of Science

Introduction
Staff
HERIX
MERIX
Sample Environments
Publications
Useful Links

Near-Backscattering, Spherical Analyzers for RIXS :
A Compilation of Viable Reflections in Si, Ge, $LiNbO_3$, Sapphire and Quartz

T. Gog, D. Casa, A. Said, M. Upton, Jung Ho Kim, I. Kuzmenko, Xianrong Huang, R. Khachatryan

[Introduction] - [Reflection Tables] - [Detector-Analyzer Geometry] - [Quantities Listed]

H																	He
Li	Be											B	C	N	O	F	Ne
Na	Mg											Al	Si	P	S	Cl	Ar
K	Ca	Sc	Ti	V	Cr	Mn	Fe	Co	Ni	Cu	Zn	Ga	Ge	As	Se	Br	Kr
Rb	Sr	Y	Zr	Nb	Mo	Tc	Ru	Rh	Pd	Ag	Cd	In	Sn	Sb	Te	I	Xe
Cs	Ba	Lu	Hf	Ta	W	Re	Os	Ir	Pt	Au	Hg	Tl	Pb	Bi	Po	At	Rn
Fr	Ra	Lr	Rf	Db	Sg	Bh	Hs	Mt	Ds	Rg	Cn	Uut	Fl	Uup	Lv	Uus	Uuo
		La	Ce	Pr	Nd	Pm	Sm	Eu	Gd	Tb	Dy	Ho	Er	Tm	Yb		
		Ac	Th	Pa	U	Np	Pu	Am	Cm	Bk	Cf	Es	Fm	Md	No		

Point to an element for available absorption edges / emission lines

contact: Thomas Gog, last updated: 06.October.2012

The Advanced Photon Source is an Office of Science User Facility operated for the U.S. Department of Energy Office of Science by Argonne National Laboratory.

UChicago Argonne LLC | Privacy & Security Notice | Contact Us | Site Map

Introduction

The tables presented here are a compilation of data pertinent to near-backscattering, spherical analyzers for Resonant Inelastic X-ray Scattering (RIXS) measurements. They contain viable crystal reflections for absorption edge- and emission line energies in the hard x-ray range, together with reflection strength- and resolution information. They assume a sample-analyzer-detector geometry based on a Rowland circle with a position-sensitive (strip-) detector as described under [Detector-Analyzer Geometry]. Crystal materials included are **Si**, **Ge, Lithium Niobate ($LiNbO_3$), Sapphire (Al_2O_3) and Quartz (SiO_2)**.

Structure factors involved in generating the reflection data were calculated for room temperature (RT=293.15 K), using Debye-Waller factors e^{-2M} with

$$e^{-M} = \sqrt{e^{-2M}} = e^{-B_{RT}\left(\frac{\sin\Theta}{\lambda}\right)} \qquad (0)$$

where $\sin\Theta/\lambda$ is proportional to the momentum transfer, and mean-square atomic vibrational amplitudes B_{RT} were derived from x-ray diffraction measurements. In particular, the following vibrational amplitudes B_{RT} and associated Debye temperatures Θ_D were used:

	B_{RT}	Θ_D
	[Å^2]	[K]
Si	0.4632 [3]	530.82
Ge	0.5661 [4]	290.03
$LiNbO_3$	Li: 0.5264[5]<br>Nb: 0.4174<br>O: 0.5738	1118.44<br>298.90<br>643.27
Al_2O_3	Al: 0.1921[6]<br>O: 0.2271	897.40<br>1122.51
SiO_2	Si: 0.4874[7]<br>O: 0.9949	516.38<br>476.16

<u>References</u>

3) M. Deutsch, M. Hart, Phys. Rev. B31, 3846 (1985)
4) M. Deutsch, M. Hart, Phys. Rev. B42, 1248 (1990)
5) B. Etschmann, N. Ishizawa, Powder Diffr. 16, 81 (2001)
6) A. Kirfel, K. Eichhorn, Acta Cryst. A46, 271 (1990)
7) Y. Le Page, L.D. Calvert, E.J. Gabe, J. Phys. Chem. Solids, 41, 721 (1980)

Tables

The tables are compilation of near-backscattering reflections in Si, Ge, Lithium Niobate ($LiNbO_3$), Sapphire (Al_2O_3) and Quartz (SiO_2) in the angular range of 70° to 90°. Tables are divided into two groups of crystals Si and Ge at the top (underplayed in yellow), Lithium Niobate , Sapphire and Quartz at the bottom. Within these two groups reflections are arranged from top to bottom by strength, according to the integrated reflectivity $\int I_R\, d\Theta$. For Lithium Niobate, Sapphire and Quartz all equivalent reflections are listed, while for Si and Ge equivalent reflections are only listed if their indices are not simple permutations or inversions of the parent reflection.

For the present calculations, some crystallographic data were taken from the software package "XOP" [8)], atomic scattering factors were taken from the database at the Center for X-Ray Optics (CXRO) at Lawrence Berkeley Nat'l Lab [9)].

References

[8)] Sanchez del Rio and R. J. Dejus, "Status of XOP: an x-ray optics software toolkit" SPIE Proceedings Vol. 5536 (2004) pp.171-174, http://dx.doi.org/10.1117/12.560903

[9)] B.L. Henke, E.M. Gullikson, and J.C. Davis. "X-ray interactions: photoabsorption, scattering, transmission, and reflection at E=50-30000 eV, Z=1-92", Atomic Data and Nuclear Data Tables Vol. **54** (no.2), 181-342 (July 1993).

Sample-Analyzer-Detector Geometry

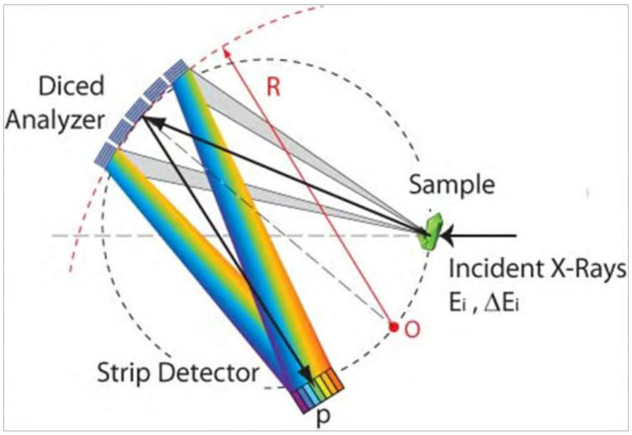

Sample-Analyzer-Detector Arrangement in Rowland Geometry

Focused, monochromatic x-rays (Energy E_i, Band Pass ΔE_i) are incident on the sample. Scattered photons are diffracted from a diced, spherical (Radius R), nearly perfect crystal analyzer. The resulting focal spot is energy dispersive in the diffraction plane of the analyzer. A position sensitive (strip-) detector (pitch p) is placed at the focal spot, recording energy loss spectra. For the present tables, the detector pitch is assumed to be p = 50 µm (Dectris “Mythen” - Detector) while the analyzer radius is R = 2m.

References

[1)] S. Huotari, F. Albergamo, G. Vanko et al., Review of Scientific Instruments **77** (5), 053102 (2006)
[2)] S. Huotari, G. Vanko, F. Albergamo et al., Journal of Synchrotron Radiation **12**, 467 (2005)

Quantities Listed in the Tables

Backscattering Energy, E_B

X-ray energy for which the incident beam is reflected at a Bragg angle of $\Theta_B = 90^o$. These are the lowest energy photons that can be reflected by the analyzer.

$$E_B = \frac{hc}{2d_{hkl}}$$

Integrated Reflectivity, $\int I_R \, d\Theta$

As a measure of the reflection strength the dynamical reflectivity is numerically integrated over the whole rocking curve.

Angular Reflection (Darwin-) Width, W

Intrinsic, dynamical (Darwin-) width associated with a symmetric reflection in a perfect crystal

Change in Energy with Angle, $dE/d\Theta = E_i \cot \Theta$

From the differential Bragg law, this quantity serves as conversion factor from angular width to energy width. It is included for guidance in units of meV/μrad

Intrinsic Energy Resolution, ΔE

Energy resolution of the analyzer reflection due to its intrinsic (Darwin-) width W

$$\Delta E = E_i \cot(\Theta_B) W$$

Geometric Contribution to the Energy Resolution, ΔE_g

Geometric Contribution to the Energy Resolution, based on the analyzer radius **R** and the detector pitch **p** :

$$\Delta E_g = E_i \cot(\Theta_B) \tfrac{p}{2R}$$

For the present tables, the detector pitch is assumed to be p = 50 μm (Dectris “Mythen” - Detector) while the analyzer radius is R = 2m.

Combined Intrinsic and Geometric Energy Resolution, ΔE_t

$$\Delta E_t = \sqrt{\Delta E^2 + \Delta E_g{}^2}$$

(Note: The total energy resolution of a RIXS setup also contains contributions from the incident energy band pass, beam size at the sample and possible others. These additional contributions are not addressed in the current document)

Symbols

E_i	Incident Photon Energy
Θ_B	Bragg (Incident) Angle
E_B	Backscattering Energy (Θ_B = 90º)
d_{hkl}	Diffraction Plane Spacing
$\int I_R \, d\Theta$	Integrated Reflectivity, Measure of Reflection Strength
W	Angular (Darwin-) Width of the Reflection
ΔE	Intrinsic Energy Resolution
ΔE_g	Geometric Contribution to the Energy Resolution
ΔE_t	Combined Intrinsic and Geometric Energy Resolution

hc = 12.39841904 keVÅ
1 Å = 0.1 nm = 10^{-10} m = 10^{-7} mm

Ei = Cryst	4.966 Refl (h,k,l)	keV EB [keV]	ΘB [°]	∫ IR dΘ [µrad]	Width [µrad]	Ei cotΘ [meV/µrad]	ΔE [meV]	ΔEg 2m, 50µm [meV]	ΔEt 2m, 50µm [meV]
**Ge**	**(1,3,3)**	**4.776**	**74.12**	**112.9**	**119.2**	**1.413**	**168.37**	**17.66**	**169.29**
**LiNbO3**	**(0,-3,6)**	**4.96**	**87.14**	**344.8**	**358.1**	**0.248**	**88.73**	**3.1**	**88.78**
	Equiv. Refl.: (3,0,6), (-3,3,6)								
**LiNbO3**	**(0,3,6)**	**4.96**	**87.14**	**329.5**	**346.7**	**0.248**	**85.91**	**3.1**	**85.96**
	Equiv. Refl.: (3,-3,6), (-3,0,6)								
**LiNbO3**	**(0,3,-6)**	**4.96**	**87.14**	**230.8**	**358.1**	**0.248**	**88.73**	**3.1**	**88.78**
	Equiv. Refl.: (3,-3,-6), (-3,0,-6)								
**LiNbO3**	**(0,-3,-6)**	**4.96**	**87.14**	**222.6**	**346.7**	**0.248**	**85.91**	**3.1**	**85.96**
	Equiv. Refl.: (3,0,-6), (-3,3,-6)								
**Quartz**	**(0,3,-2)**	**4.936**	**83.74**	**117.8**	**114**	**0.545**	**62.09**	**6.81**	**62.46**
	Equiv. Refl.: (0,-3,2), (3,0,2), (3,-3,-2), (-3,0,-2), (-3,3,2)								
**Quartz**	**(0,3,2)**	**4.936**	**83.74**	**92.9**	**95.3**	**0.545**	**51.9**	**6.81**	**52.34**
	Equiv. Refl.: (0,-3,-2), (3,0,-2), (3,-3,2), (-3,0,2), (-3,3,-2)								
**LiNbO3**	**(0,1,-10)**	**4.683**	**70.56**	**87.8**	**92**	**1.752**	**161.2**	**21.91**	**162.68**
	Equiv. Refl.: (1,-1,-10), (-1,0,-10)								
**LiNbO3**	**(2,2,0)**	**4.817**	**75.92**	**82.9**	**96.5**	**1.246**	**120.17**	**15.57**	**121.17**
	Equiv. Refl.: (2,-4,0), (4,-2,0), (-2,4,0), (-2,-2,0), (-4,2,0)								
**LiNbO3**	**(0,-1,10)**	**4.683**	**70.56**	**80.9**	**92**	**1.752**	**161.2**	**21.91**	**162.68**
	Equiv. Refl.: (1,0,10), (-1,1,10)								
**Quartz**	**(0,1,-4)**	**4.814**	**75.78**	**57.7**	**55.5**	**1.258**	**69.89**	**15.73**	**71.64**
	Equiv. Refl.: (0,-1,4)								
**Quartz**	**(1,0,4)**	**4.814**	**75.78**	**57.6**	**55.5**	**1.258**	**69.86**	**15.73**	**71.61**
	Equiv. Refl.: (1,-1,-4), (-1,0,-4), (-1,1,4)								
**LiNbO3**	**(1,1,9)**	**4.69**	**70.81**	**21.8**	**39.8**	**1.728**	**68.76**	**21.6**	**72.07**
	Equiv. Refl.: (1,1,-9), (1,-2,9), (1,-2,-9), (2,-1,9), (2,-1,-9), (-1,2,9), (-1,2,-9), (-1,-1,9), (-1,-1,-9) (-2,1,9), (-2,1,-9)								
**Sapph**	**(0,2,-8)**	**4.861**	**78.22**	**14.4**	**22.8**	**1.036**	**23.64**	**12.95**	**26.95**
	Equiv. Refl.: (0,-2,8), (2,0,8), (2,-2,-8), (-2,0,-8), (-2,2,8)								
**Quartz**	**(0,1,4)**	**4.814**	**75.78**	**11**	**15.8**	**1.258**	**19.83**	**15.73**	**25.31**
	Equiv. Refl.: (0,-1,-4), (1,0,-4), (1,-1,4), (-1,0,4), (-1,1,-4)								
**LiNbO3**	**(1,2,-7)**	**4.83**	**76.58**	**6.7**	**24**	**1.185**	**28.49**	**14.81**	**32.11**
	Equiv. Refl.: (1,-3,7), (2,1,7), (2,-3,-7), (3,-1,7), (3,-2,-7), (-1,3,-7), (-1,-2,7), (-2,3,7), (-2,-1,-7) (-3,1,-7), (-3,2,7)								

Ei =	4.511	keV						ΔEg	ΔEt
Cryst	Refl	EB	ΘB	∫ IR dΘ	Width	Ei cotΘ	ΔE	2m, 50µm	2m, 50µm
	(h,k,l)	[keV]	[°]	[µrad]	[µrad]	[meV/µrad]	[meV]	[meV]	[meV]
**Ge**	**(0,0,4)**	**4.383**	**76.32**	**256.839**	**250.54**	**1.0976**	**274.99**	**13.72**	**275.33**
	Equiv. Refl.: (0,4,0), (4,0,0)								
**Quartz**	**(0,-2,3)**	**4.509**	**88.34**	**935.641**	**852.6**	**0.131**	**111.3**	**1.63**	**111.32**
**Quartz**	**(0,2,-3)**	**4.509**	**88.34**	**935.635**	**852.6**	**0.131**	**111.3**	**1.63**	**111.32**
**Quartz**	**(-2,0,-3)**	**4.509**	**88.34**	**935.508**	**852.5**	**0.131**	**111.29**	**1.63**	**111.3**
**Quartz**	**(2,0,3)**	**4.509**	**88.34**	**935.507**	**852.5**	**0.131**	**111.29**	**1.63**	**111.3**
**Quartz**	**(-2,2,3)**	**4.509**	**88.34**	**935.499**	**852.5**	**0.131**	**111.29**	**1.63**	**111.3**
**Quartz**	**(2,-2,-3)**	**4.509**	**88.34**	**935.486**	**852.5**	**0.131**	**111.29**	**1.63**	**111.3**
**Quartz**	**(0,-2,-3)**	**4.509**	**88.34**	**265.546**	**308.2**	**0.131**	**40.23**	**1.63**	**40.27**
**Quartz**	**(0,2,3)**	**4.509**	**88.34**	**265.541**	**308.2**	**0.131**	**40.23**	**1.63**	**40.27**
**Quartz**	**(-2,2,-3)**	**4.509**	**88.34**	**265.474**	**308.1**	**0.131**	**40.22**	**1.63**	**40.25**
**Quartz**	**(-2,0,3)**	**4.509**	**88.34**	**265.454**	**308.1**	**0.131**	**40.22**	**1.63**	**40.25**
**Quartz**	**(2,-2,3)**	**4.509**	**88.34**	**265.366**	**308.1**	**0.131**	**40.22**	**1.63**	**40.25**
**Quartz**	**(2,0,-3)**	**4.509**	**88.34**	**265.357**	**308.1**	**0.131**	**40.22**	**1.63**	**40.25**
**Quartz**	**(3,-1,-2)**	**4.486**	**83.94**	**125.565**	**128.6**	**0.479**	**61.61**	**5.99**	**61.9**
**Quartz**	**(3,-2,2)**	**4.486**	**83.94**	**125.561**	**128.6**	**0.479**	**61.61**	**5.99**	**61.9**
**Quartz**	**(-2,3,-2)**	**4.486**	**83.94**	**125.556**	**128.6**	**0.479**	**61.6**	**5.99**	**61.89**
**Quartz**	**(-2,-1,2)**	**4.486**	**83.94**	**125.553**	**128.6**	**0.479**	**61.6**	**5.99**	**61.89**
**Quartz**	**(-1,3,2)**	**4.486**	**83.94**	**125.547**	**128.6**	**0.479**	**61.6**	**5.99**	**61.89**
**Quartz**	**(-1,-2,-2)**	**4.486**	**83.94**	**125.542**	**128.6**	**0.479**	**61.6**	**5.99**	**61.89**
**Quartz**	**(-3,1,2)**	**4.486**	**83.94**	**122.896**	**128.6**	**0.479**	**61.61**	**5.99**	**61.9**
**Quartz**	**(-3,2,-2)**	**4.486**	**83.94**	**122.892**	**128.6**	**0.479**	**61.61**	**5.99**	**61.9**
**Quartz**	**(1,-3,-2)**	**4.486**	**83.94**	**122.883**	**128.6**	**0.479**	**61.6**	**5.99**	**61.89**
**Quartz**	**(1,2,2)**	**4.486**	**83.94**	**122.878**	**128.6**	**0.479**	**61.6**	**5.99**	**61.89**
**Quartz**	**(2,-3,2)**	**4.486**	**83.94**	**122.871**	**128.6**	**0.479**	**61.6**	**5.99**	**61.89**
**Quartz**	**(2,1,-2)**	**4.486**	**83.94**	**122.867**	**128.6**	**0.479**	**61.6**	**5.99**	**61.89**
**Sapph**	**(1,2,-4)**	**4.415**	**78.13**	**111.203**	**109.9**	**0.948**	**104.19**	**11.85**	**104.87**
	Equiv. Refl.: (1,-3,4), (2,1,4), (2,-3,-4), (3,-1,4), (3,-2,-4), (-1,3,-4), (-1,-2,4), (-2,3,4), (-2,-1,-4) (-3,1,-4), (-3,2,4)								
**Quartz**	**(2,-3,-2)**	**4.486**	**83.94**	**79.571**	**86.7**	**0.479**	**41.54**	**5.99**	**41.96**
**Quartz**	**(2,1,2)**	**4.486**	**83.94**	**79.57**	**86.7**	**0.479**	**41.53**	**5.99**	**41.96**
**Quartz**	**(-3,1,-2)**	**4.486**	**83.94**	**79.559**	**86.7**	**0.479**	**41.53**	**5.99**	**41.96**
**Quartz**	**(-3,2,2)**	**4.486**	**83.94**	**79.547**	**86.7**	**0.479**	**41.53**	**5.99**	**41.96**
**Quartz**	**(1,2,-2)**	**4.486**	**83.94**	**79.537**	**86.7**	**0.479**	**41.52**	**5.99**	**41.95**
	Equiv. Refl.: (1,-3,2)								
**Quartz**	**(-2,3,2)**	**4.486**	**83.94**	**65.17**	**86.7**	**0.479**	**41.54**	**5.99**	**41.96**
**Quartz**	**(-2,-1,-2)**	**4.486**	**83.94**	**65.169**	**86.7**	**0.479**	**41.53**	**5.99**	**41.96**
**Quartz**	**(3,-1,2)**	**4.486**	**83.94**	**65.164**	**86.7**	**0.479**	**41.53**	**5.99**	**41.96**
**Quartz**	**(3,-2,-2)**	**4.486**	**83.94**	**65.152**	**86.7**	**0.479**	**41.53**	**5.99**	**41.96**
**Quartz**	**(-1,-2,2)**	**4.486**	**83.94**	**65.14**	**86.7**	**0.479**	**41.52**	**5.99**	**41.95**
**Quartz**	**(-1,3,-2)**	**4.486**	**83.94**	**65.139**	**86.7**	**0.479**	**41.52**	**5.99**	**41.95**
**Quartz**	**(-1,2,-3)**	**4.267**	**71.08**	**26.008**	**29**	**1.546**	**44.81**	**19.33**	**48.8**
	Equiv. Refl.: (-1,-1,3)								
**Quartz**	**(2,-1,-3)**	**4.267**	**71.08**	**26.004**	**29**	**1.546**	**44.8**	**19.33**	**48.79**
**Quartz**	**(2,-1,3)**	**4.267**	**71.08**	**25.997**	**29**	**1.546**	**44.79**	**19.33**	**48.79**
**Quartz**	**(-1,2,3)**	**4.267**	**71.08**	**25.996**	**29**	**1.546**	**44.79**	**19.33**	**48.79**
**Quartz**	**(-1,-1,-3)**	**4.267**	**71.08**	**25.995**	**29**	**1.546**	**44.79**	**19.33**	**48.79**

Ei =	4.511	keV						ΔEg	ΔEt
Cryst	Refl	EB	ΘB	∫ IR dΘ	Width	Ei cotΘ	ΔE	2m, 50µm	2m, 50µm
	(h,k,l)	[keV]	[°]	[µrad]	[µrad]	[meV/µrad]	[meV]	[meV]	[meV]
**Quartz**	**(1,1,-3)**	**4.267**	**71.08**	**22.15**	**29**	**1.546**	**44.81**	**19.33**	**48.8**
	Equiv. Refl.: (1,-2,3)								
**Quartz**	**(-2,1,3)**	**4.267**	**71.08**	**22.145**	**29**	**1.546**	**44.8**	**19.33**	**48.79**
**Quartz**	**(1,1,3)**	**4.267**	**71.08**	**22.142**	**29**	**1.546**	**44.79**	**19.33**	**48.79**
	Equiv. Refl.: (1,-2,-3)								
**Quartz**	**(-2,1,-3)**	**4.267**	**71.08**	**22.138**	**29**	**1.546**	**44.79**	**19.33**	**48.79**
**Quartz**	**(0,3,0)**	**4.371**	**75.69**	**20.522**	**28.1**	**1.151**	**32.39**	**14.39**	**35.44**
	Equiv. Refl.: (0,-3,0), (3,0,0), (3,-3,0), (-3,0,0), (-3,3,0)								
**LiNbO3**	**(1,2,5)**	**4.305**	**72.62**	**6.463**	**25.1**	**1.412**	**35.43**	**17.65**	**39.59**
	Equiv. Refl.: (1,-3,-5), (2,1,-5), (2,-3,5), (3,-1,-5), (3,-2,5), (-1,3,5), (-1,-2,-5), (-2,3,-5), (-2,-1,5) (-3,1,5), (-3,2,-5)								

Ei =	4.505	keV						ΔEg	ΔEt
**Cryst**	**Refl**	**EB**	**ΘB**	**∫IR dΘ**	**Width**	**Ei cotΘ**	**ΔE**	**2m, 50µm**	**2m, 50µm**
	**(h,k,l)**	**[keV]**	**[°]**	**[µrad]**	**[µrad]**	**[meV/µrad]**	**[meV]**	**[meV]**	**[meV]**
**Ge**	**(0,0,4)**	**4.383**	**76.64**	**263.082**	**256.74**	**1.0698**	**274.65**	**13.37**	**274.97**
	Equiv. Refl.: (0,4,0), (4,0,0)								
**Quartz**	**(3,-1,-2)**	**4.486**	**84.71**	**143.805**	**147.4**	**0.417**	**61.53**	**5.22**	**61.75**
**Quartz**	**(3,-2,2)**	**4.486**	**84.71**	**143.8**	**147.4**	**0.417**	**61.53**	**5.22**	**61.75**
**Quartz**	**(-2,3,-2)**	**4.486**	**84.71**	**143.795**	**147.4**	**0.417**	**61.52**	**5.22**	**61.74**
**Quartz**	**(-2,-1,2)**	**4.486**	**84.71**	**143.791**	**147.4**	**0.417**	**61.52**	**5.22**	**61.74**
**Quartz**	**(-1,3,2)**	**4.486**	**84.71**	**143.785**	**147.4**	**0.417**	**61.52**	**5.22**	**61.74**
**Quartz**	**(-1,-2,-2)**	**4.486**	**84.71**	**143.779**	**147.4**	**0.417**	**61.52**	**5.22**	**61.74**
**Quartz**	**(-3,1,2)**	**4.486**	**84.71**	**140.742**	**147.4**	**0.417**	**61.53**	**5.22**	**61.75**
**Quartz**	**(-3,2,-2)**	**4.486**	**84.71**	**140.737**	**147.4**	**0.417**	**61.53**	**5.22**	**61.75**
**Quartz**	**(1,-3,-2)**	**4.486**	**84.71**	**140.726**	**147.4**	**0.417**	**61.52**	**5.22**	**61.74**
**Quartz**	**(1,2,2)**	**4.486**	**84.71**	**140.721**	**147.4**	**0.417**	**61.52**	**5.22**	**61.74**
**Quartz**	**(2,-3,2)**	**4.486**	**84.71**	**140.712**	**147.4**	**0.417**	**61.52**	**5.22**	**61.74**
**Quartz**	**(2,1,-2)**	**4.486**	**84.71**	**140.709**	**147.4**	**0.417**	**61.52**	**5.22**	**61.74**
**Sapph**	**(1,2,-4)**	**4.415**	**78.5**	**114.811**	**113.5**	**0.917**	**104.06**	**11.46**	**104.69**
	Equiv. Refl.: (1,-3,4), (2,1,4), (2,-3,-4), (3,-1,4), (3,-2,-4), (-1,3,-4), (-1,-2,4), (-2,3,4), (-2,-1,-4) (-3,1,-4), (-3,2,4)								
**Quartz**	**(2,-3,-2)**	**4.486**	**84.71**	**91.12**	**99.4**	**0.417**	**41.48**	**5.22**	**41.81**
**Quartz**	**(2,1,2)**	**4.486**	**84.71**	**91.119**	**99.4**	**0.417**	**41.48**	**5.22**	**41.81**
**Quartz**	**(-3,1,-2)**	**4.486**	**84.71**	**91.107**	**99.4**	**0.417**	**41.48**	**5.22**	**41.8**
**Quartz**	**(-3,2,2)**	**4.486**	**84.71**	**91.093**	**99.4**	**0.417**	**41.47**	**5.22**	**41.8**
**Quartz**	**(1,-3,2)**	**4.486**	**84.71**	**91.082**	**99.3**	**0.417**	**41.47**	**5.22**	**41.79**
**Quartz**	**(1,2,-2)**	**4.486**	**84.71**	**91.081**	**99.3**	**0.417**	**41.47**	**5.22**	**41.79**
**Quartz**	**(-2,3,2)**	**4.486**	**84.71**	**74.595**	**99.4**	**0.417**	**41.48**	**5.22**	**41.81**
**Quartz**	**(-2,-1,-2)**	**4.486**	**84.71**	**74.594**	**99.4**	**0.417**	**41.48**	**5.22**	**41.81**
**Quartz**	**(3,-1,2)**	**4.486**	**84.71**	**74.589**	**99.4**	**0.417**	**41.48**	**5.22**	**41.8**
**Quartz**	**(3,-2,-2)**	**4.486**	**84.71**	**74.575**	**99.4**	**0.417**	**41.47**	**5.22**	**41.8**
**Quartz**	**(-1,-2,2)**	**4.486**	**84.71**	**74.561**	**99.3**	**0.417**	**41.47**	**5.22**	**41.79**
**Quartz**	**(-1,3,-2)**	**4.486**	**84.71**	**74.56**	**99.3**	**0.417**	**41.47**	**5.22**	**41.79**
**Quartz**	**(-1,-1,3)**	**4.267**	**71.3**	**26.324**	**29.4**	**1.525**	**44.75**	**19.06**	**48.64**
**Quartz**	**(-1,2,-3)**	**4.267**	**71.3**	**26.323**	**29.4**	**1.525**	**44.75**	**19.06**	**48.64**
**Quartz**	**(2,-1,-3)**	**4.267**	**71.3**	**26.319**	**29.3**	**1.525**	**44.74**	**19.06**	**48.63**
**Quartz**	**(2,-1,3)**	**4.267**	**71.3**	**26.313**	**29.3**	**1.525**	**44.74**	**19.06**	**48.63**
**Quartz**	**(-1,2,3)**	**4.267**	**71.3**	**26.311**	**29.3**	**1.525**	**44.74**	**19.06**	**48.63**
	Equiv. Refl.: (-1,-1,-3)								
**Quartz**	**(1,1,-3)**	**4.267**	**71.3**	**22.411**	**29.4**	**1.525**	**44.75**	**19.06**	**48.64**
**Quartz**	**(1,-2,3)**	**4.267**	**71.3**	**22.41**	**29.4**	**1.525**	**44.75**	**19.06**	**48.64**
**Quartz**	**(-2,1,3)**	**4.267**	**71.3**	**22.405**	**29.3**	**1.525**	**44.74**	**19.06**	**48.63**
**Quartz**	**(1,1,3)**	**4.267**	**71.3**	**22.402**	**29.3**	**1.525**	**44.74**	**19.06**	**48.63**
	Equiv. Refl.: (1,-2,-3)								
**Quartz**	**(-2,1,-3)**	**4.267**	**71.3**	**22.399**	**29.3**	**1.525**	**44.74**	**19.06**	**48.63**
**Quartz**	**(0,3,0)**	**4.371**	**75.99**	**20.959**	**28.8**	**1.124**	**32.35**	**14.05**	**35.27**
	Equiv. Refl.: (0,-3,0), (3,0,0), (3,-3,0), (-3,0,0), (-3,3,0)								
**LiNbO3**	**(1,2,5)**	**4.305**	**72.87**	**6.548**	**25.5**	**1.389**	**35.39**	**17.36**	**39.42**
	Equiv. Refl.: (1,-3,-5), (2,1,-5), (2,-3,5), (3,-1,-5), (3,-2,5), (-1,3,5), (-1,-2,-5), (-2,3,-5), (-2,-1,5) (-3,1,5), (-3,2,-5)								

Ei =	4.932	keV						ΔEg	ΔEt
**Cryst**	**Refl**	**EB**	**ΘB**	**∫ IR dΘ**	**Width**	**Ei cotΘ**	**ΔE**	**2m, 50µm**	**2m, 50µm**
	**(h,k,l)**	**[keV]**	**[°]**	**[µrad]**	**[µrad]**	**[meV/µrad]**	**[meV]**	**[meV]**	**[meV]**
**Ge**	**(1,3,3)**	**4.776**	**75.57**	**124.4891**	**131.82**	**1.2691**	**167.3**	**15.86**	**168.05**
	Equiv. Refl.: (3,1,3), (3,3,1)								
**LiNbO3**	**(2,2,0)**	**4.817**	**77.59**	**94.116**	**109.9**	**1.085**	**119.28**	**13.56**	**120.05**
	Equiv. Refl.: (2,-4,0), (4,-2,0), (-2,4,0), (-2,-2,0), (-4,2,0)								
**LiNbO3**	**(0,1,-10)**	**4.683**	**71.71**	**93.532**	**98.2**	**1.63**	**160.04**	**20.37**	**161.33**
	Equiv. Refl.: (1,-1,-10), (-1,0,-10)								
**LiNbO3**	**(0,-1,10)**	**4.683**	**71.71**	**86.115**	**98.2**	**1.63**	**160.04**	**20.37**	**161.33**
	Equiv. Refl.: (1,0,10), (-1,1,10)								
**Quartz**	**(0,-1,4)**	**4.814**	**77.43**	**65.402**	**63.2**	**1.099**	**69.43**	**13.74**	**70.77**
**Quartz**	**(0,1,-4)**	**4.814**	**77.43**	**65.401**	**63.2**	**1.099**	**69.43**	**13.74**	**70.77**
**Quartz**	**(1,0,4)**	**4.814**	**77.43**	**65.365**	**63.1**	**1.099**	**69.4**	**13.74**	**70.74**
	Equiv. Refl.: (-1,0,-4)								
**Quartz**	**(-1,1,4)**	**4.814**	**77.43**	**65.36**	**63.1**	**1.099**	**69.39**	**13.74**	**70.74**
**Quartz**	**(1,-1,-4)**	**4.814**	**77.43**	**65.359**	**63.1**	**1.099**	**69.39**	**13.74**	**70.74**
**LiNbO3**	**(1,1,9)**	**4.69**	**71.98**	**23.102**	**42.6**	**1.604**	**68.3**	**20.05**	**71.18**
	Equiv. Refl.: (1,1,-9), (1,-2,9), (1,-2,-9), (2,-1,9), (2,-1,-9), (-1,2,9), (-1,2,-9), (-1,-1,9), (-1,-1,-9) (-2,1,9), (-2,1,-9)								
**Sapph**	**(0,2,-8)**	**4.861**	**80.29**	**17.376**	**27.8**	**0.844**	**23.49**	**10.55**	**25.75**
	Equiv. Refl.: (0,-2,8), (2,0,8), (2,-2,-8), (-2,0,-8), (-2,2,8)								
**Quartz**	**(-1,0,4)**	**4.814**	**77.43**	**12.456**	**17.9**	**1.099**	**19.7**	**13.74**	**24.02**
**Quartz**	**(-1,1,-4)**	**4.814**	**77.43**	**12.453**	**17.9**	**1.099**	**19.7**	**13.74**	**24.02**
**Quartz**	**(1,0,-4)**	**4.814**	**77.43**	**12.452**	**17.9**	**1.099**	**19.7**	**13.74**	**24.02**
**Quartz**	**(1,-1,4)**	**4.814**	**77.43**	**12.447**	**17.9**	**1.099**	**19.7**	**13.74**	**24.02**
**Quartz**	**(0,-1,-4)**	**4.814**	**77.43**	**12.444**	**17.9**	**1.099**	**19.69**	**13.74**	**24.01**
**Quartz**	**(0,1,4)**	**4.814**	**77.43**	**12.443**	**17.9**	**1.099**	**19.69**	**13.74**	**24.01**
**Sapph**	**(1,2,5)**	**4.641**	**70.22**	**9.968**	**15.6**	**1.774**	**27.66**	**22.17**	**35.45**
	Equiv. Refl.: (1,-3,-5), (2,1,-5), (2,-3,5), (3,-1,-5), (3,-2,5), (-1,3,5), (-1,-2,-5), (-2,3,-5), (-2,-1,5) (-3,1,5), (-3,2,-5)								
**LiNbO3**	**(1,2,-7)**	**4.83**	**78.35**	**7.631**	**27.8**	**1.017**	**28.31**	**12.71**	**31.03**
	Equiv. Refl.: (1,-3,7), (2,1,7), (2,-3,-7), (3,-1,7), (3,-2,-7), (-1,3,-7), (-1,-2,7), (-2,3,7), (-2,-1,-7) (-3,1,-7), (-3,2,7)								

Ei =	5.465	keV						ΔEg	ΔEt
Cryst	Refl	EB	ΘB	∫IR dΘ	Width	Ei cotΘ	ΔE	2m, 50µm	2m, 50µm
	(h,k,l)	[keV]	[°]	[µrad]	[µrad]	[meV/µrad]	[meV]	[meV]	[meV]
**Ge**	**(2,2,4)**	**5.368**	**79.2**	**188.7**	**179.2**	**1.042**	**186.83**	**13.03**	**187.29**
**LiNbO3**	**(0,0,-12)**	**5.366**	**79.08**	**140.2**	**128.9**	**1.054**	**135.81**	**13.17**	**136.44**
**LiNbO3**	**(0,0,12)**	**5.366**	**79.08**	**105.9**	**128.9**	**1.054**	**135.81**	**13.17**	**136.44**
**LiNbO3**	**(1,3,4)**	**5.323**	**76.91**	**72.9**	**79.3**	**1.271**	**100.8**	**15.88**	**102.04**
	Equiv. Refl.: (3,-4,4), (4,-3,4), (-1,4,4), (-3,-1,4), (-4,1,4)								
**LiNbO3**	**(0,2,10)**	**5.266**	**74.49**	**71.1**	**74.9**	**1.517**	**113.65**	**18.96**	**115.22**
	Equiv. Refl.: (2,-2,10), (-2,0,10)								
**Sapph**	**(2,2,3)**	**5.405**	**81.47**	**70.5**	**70.8**	**0.819**	**58.04**	**10.24**	**58.93**
	Equiv. Refl.: (2,2,-3), (2,-4,3), (2,-4,-3), (4,-2,3), (4,-2,-3), (-2,4,3), (-2,4,-3), (-2,-2,3), (-2,-2,-3) (-4,2,3), (-4,2,-3)								
**LiNbO3**	**(1,-4,-4)**	**5.323**	**76.91**	**66.7**	**79.3**	**1.271**	**100.8**	**15.88**	**102.04**
	Equiv. Refl.: (3,1,-4), (4,-1,-4), (-1,-3,-4), (-3,4,-4), (-4,3,-4)								
**LiNbO3**	**(0,-2,-10)**	**5.266**	**74.49**	**64.4**	**74.9**	**1.517**	**113.65**	**18.96**	**115.22**
	Equiv. Refl.: (2,0,-10), (-2,2,-10)								
**Quartz**	**(-1,4,1)**	**5.377**	**79.7**	**50.9**	**47.4**	**0.993**	**47.06**	**12.41**	**48.66**
	Equiv. Refl.: (-1,-3,-1), (-3,4,-1), (-3,-1,1)								
**Quartz**	**(4,-1,-1)**	**5.377**	**79.7**	**50.8**	**47.4**	**0.993**	**47.05**	**12.41**	**48.66**
	Equiv. Refl.: (4,-3,1)								
**Quartz**	**(1,3,1)**	**5.377**	**79.7**	**45.4**	**47.4**	**0.993**	**47.06**	**12.41**	**48.67**
	Equiv. Refl.: (1,-4,-1), (3,1,-1), (3,-4,1), (-4,1,1), (-4,3,-1)								
**Quartz**	**(0,2,-4)**	**5.435**	**84.02**	**44.1**	**49.7**	**0.573**	**28.48**	**7.16**	**29.36**
	Equiv. Refl.: (0,-2,4), (2,0,4), (2,-2,-4), (-2,0,-4), (-2,2,4)								
**Quartz**	**(4,-1,0)**	**5.253**	**74**	**44**	**40.6**	**1.567**	**63.56**	**19.59**	**66.51**
	Equiv. Refl.: (4,-3,0), (-1,4,0), (-1,-3,0), (-3,4,0), (-3,-1,0)								
**Quartz**	**(1,3,0)**	**5.253**	**74**	**43**	**40.6**	**1.567**	**63.56**	**19.59**	**66.51**
	Equiv. Refl.: (1,-4,0), (3,1,0), (3,-4,0), (-4,1,0), (-4,3,0)								
**Quartz**	**(2,-1,4)**	**5.236**	**73.37**	**39.6**	**36**	**1.633**	**58.82**	**20.41**	**62.26**
	Equiv. Refl.: (2,-1,-4), (-1,2,4), (-1,2,-4), (-1,-1,4), (-1,-1,-4)								
**Quartz**	**(1,1,4)**	**5.236**	**73.37**	**36.5**	**36**	**1.633**	**58.84**	**20.41**	**62.28**
	Equiv. Refl.: (1,1,-4), (1,2,-3), (1,-2,4), (1,-2,-4), (1,-3,3), (2,1,3), (2,-3,-3), (-2,1,4), (-2,1,-4) (-3,1,-3), (-3,2,3)								
**Quartz**	**(3,-1,3)**	**5.167**	**71**	**35.9**	**34**	**1.882**	**64**	**23.52**	**68.19**
	Equiv. Refl.: (3,-2,-3), (-1,3,-3), (-1,-2,3), (-2,3,3), (-2,-1,-3)								
**Sapph**	**(2,2,0)**	**5.211**	**72.48**	**27.8**	**28.9**	**1.725**	**49.88**	**21.57**	**54.34**
	Equiv. Refl.: (2,-4,0), (4,-2,0), (-2,4,0), (-2,-2,0), (-4,2,0)								
**Quartz**	**(4,-2,1)**	**5.176**	**71.28**	**17.4**	**18.5**	**1.852**	**34.27**	**23.15**	**41.36**
	Equiv. Refl.: (4,-2,-1), (-2,4,1), (-2,4,-1), (-2,-2,1), (-2,-2,-1)								
**Quartz**	**(2,2,1)**	**5.176**	**71.28**	**16.5**	**18.5**	**1.852**	**34.27**	**23.15**	**41.35**
	Equiv. Refl.: (2,2,-1), (2,-4,1), (2,-4,-1), (-4,2,1), (-4,2,-1)								
**Sapph**	**(1,3,1)**	**5.445**	**85.12**	**14.9**	**28.6**	**0.466**	**13.35**	**5.83**	**14.56**
	Equiv. Refl.: (1,-4,-1), (3,1,-1), (3,-4,1), (4,-1,-1), (4,-3,1), (-1,4,1), (-1,-3,-1), (-3,4,-1), (-3,-1,1) (-4,1,1), (-4,3,-1)								
**Quartz**	**(3,-1,-3)**	**5.167**	**71**	**14.8**	**15.7**	**1.882**	**29.48**	**23.52**	**37.71**
	Equiv. Refl.: (3,-2,3), (-1,3,3), (-1,-2,-3), (-2,3,-3), (-2,-1,3)								
**Quartz**	**(1,2,3)**	**5.167**	**71**	**12.4**	**15.7**	**1.882**	**29.46**	**23.52**	**37.7**
	Equiv. Refl.: (1,-3,-3), (2,1,-3), (2,-3,3), (-3,1,3), (-3,2,-3)								
**Quartz**	**(0,2,4)**	**5.435**	**84.02**	**11.7**	**20.3**	**0.573**	**11.65**	**7.16**	**13.68**
	Equiv. Refl.: (0,-2,-4), (2,0,-4), (2,-2,4), (-2,0,4), (-2,2,-4)								
**Quartz**	**(1,3,-1)**	**5.377**	**79.7**	**11.3**	**14.3**	**0.993**	**14.18**	**12.41**	**18.84**
	Equiv. Refl.: (1,-4,1), (3,1,1), (3,-4,-1), (-4,1,-1), (-4,3,1)								

Ei =<br>Cryst	5.465<br>Refl<br>(h,k,l)	keV<br>EB<br>[keV]	ΘB<br>[°]	∫ IR dΘ<br>[µrad]	Width<br>[µrad]	Ei cotΘ<br>[meV/µrad]	ΔE<br>[meV]	ΔEg<br>2m, 50µm<br>[meV]	ΔEt<br>2m, 50µm<br>[meV]
**Sapph**	**(1,2,-7)**	**5.197**	**71.97**	**8.4**	**12.4**	**1.779**	**22.13**	**22.23**	**31.37**
	Equiv. Refl.: (1,-3,7), (2,1,7), (2,-3,-7), (3,-1,7), (3,-2,-7), (-1,3,-7), (-1,-2,7), (-2,3,7), (-2,-1,-7) (-3,1,-7), (-3,2,7)								
**Quartz**	**(4,-1,1)**	**5.377**	**79.7**	**8**	**14.3**	**0.993**	**14.18**	**12.41**	**18.84**
	Equiv. Refl.: (4,-3,-1), (-1,4,-1), (-1,-3,1), (-3,4,1), (-3,-1,-1)								
**Sapph**	**(0,3,6)**	**5.345**	**77.98**	**3.5**	**8.1**	**1.164**	**9.46**	**14.55**	**17.35**
	Equiv. Refl.: (0,3,-6), (0,-3,6), (0,-3,-6), (3,0,6), (3,0,-6), (3,-3,6), (3,-3,-6), (-3,0,6), (-3,0,-6) (-3,3,6), (-3,3,-6)								

Ei =	4.952	keV						ΔEg	ΔEt
Cryst	Refl	EB	ΘB	∫ IR dΘ	Width	Ei cotΘ	ΔE	2m, 50µm	2m, 50µm
	(h,k,l)	[keV]	[°]	[µrad]	[µrad]	[meV/µrad]	[meV]	[meV]	[meV]
**Ge**	**(1,3,3)**	**4.776**	**74.7**	**117.2406**	**123.92**	**1.3551**	**167.93**	**16.94**	**168.78**
	Equiv. Refl.: (3,1,3), (3,3,1)								
**Quartz**	**(-3,3,2)**	**4.936**	**85.45**	**162.281**	**157.1**	**0.394**	**61.92**	**4.93**	**62.11**
**Quartz**	**(-3,0,-2)**	**4.936**	**85.45**	**162.277**	**157.1**	**0.394**	**61.91**	**4.93**	**62.11**
**Quartz**	**(0,3,-2)**	**4.936**	**85.45**	**162.273**	**157.1**	**0.394**	**61.92**	**4.93**	**62.11**
	Equiv. Refl.: (0,-3,2)								
**Quartz**	**(3,-3,-2)**	**4.936**	**85.45**	**162.269**	**157.1**	**0.394**	**61.92**	**4.93**	**62.11**
**Quartz**	**(3,0,2)**	**4.936**	**85.45**	**162.265**	**157.1**	**0.394**	**61.91**	**4.93**	**62.11**
**Quartz**	**(-3,3,-2)**	**4.936**	**85.45**	**127.882**	**131.3**	**0.394**	**51.77**	**4.93**	**52.01**
**Quartz**	**(3,-3,2)**	**4.936**	**85.45**	**127.881**	**131.3**	**0.394**	**51.77**	**4.93**	**52.01**
**Quartz**	**(-3,0,2)**	**4.936**	**85.45**	**127.875**	**131.3**	**0.394**	**51.77**	**4.93**	**52**
**Quartz**	**(3,0,-2)**	**4.936**	**85.45**	**127.873**	**131.3**	**0.394**	**51.77**	**4.93**	**52**
**Quartz**	**(0,3,2)**	**4.936**	**85.45**	**127.831**	**131.3**	**0.394**	**51.76**	**4.93**	**51.99**
	Equiv. Refl.: (0,-3,-2)								
**LiNbO3**	**(0,1,-10)**	**4.683**	**71.03**	**90.052**	**94.4**	**1.703**	**160.72**	**21.28**	**162.13**
	Equiv. Refl.: (-1,0,-10)								
**LiNbO3**	**(1,-1,-10)**	**4.683**	**71.03**	**90.051**	**94.4**	**1.703**	**160.72**	**21.28**	**162.13**
**LiNbO3**	**(2,2,0)**	**4.817**	**76.58**	**86.981**	**101.4**	**1.182**	**119.8**	**14.77**	**120.71**
	Equiv. Refl.: (2,-4,0), (4,-2,0), (-2,4,0), (-2,-2,0), (-4,2,0)								
**LiNbO3**	**(0,-1,10)**	**4.683**	**71.03**	**82.956**	**94.4**	**1.703**	**160.72**	**21.28**	**162.13**
	Equiv. Refl.: (1,0,10)								
**LiNbO3**	**(-1,1,10)**	**4.683**	**71.03**	**82.955**	**94.4**	**1.703**	**160.72**	**21.28**	**162.13**
**Quartz**	**(0,-1,4)**	**4.814**	**76.43**	**60.508**	**58.3**	**1.195**	**69.7**	**14.94**	**71.28**
**Quartz**	**(0,1,-4)**	**4.814**	**76.43**	**60.507**	**58.3**	**1.195**	**69.7**	**14.94**	**71.28**
**Quartz**	**(1,0,4)**	**4.814**	**76.43**	**60.474**	**58.3**	**1.195**	**69.67**	**14.94**	**71.25**
	Equiv. Refl.: (-1,0,-4)								
**Quartz**	**(-1,1,4)**	**4.814**	**76.43**	**60.469**	**58.3**	**1.195**	**69.66**	**14.94**	**71.25**
**Quartz**	**(1,-1,-4)**	**4.814**	**76.43**	**60.468**	**58.3**	**1.195**	**69.66**	**14.94**	**71.25**
**LiNbO3**	**(1,1,9)**	**4.69**	**71.28**	**22.276**	**40.9**	**1.678**	**68.57**	**20.97**	**71.7**
	Equiv. Refl.: (1,1,-9), (1,-2,9), (1,-2,-9), (2,-1,9), (2,-1,-9), (-1,2,9), (-1,2,-9), (-1,-1,9), (-1,-1,-9) (-2,1,9), (-2,1,-9)								
**Sapph**	**(0,2,-8)**	**4.861**	**79.02**	**15.389**	**24.5**	**0.961**	**23.58**	**12.01**	**26.46**
	Equiv. Refl.: (0,-2,8), (2,0,8), (2,-2,-8), (-2,0,-8), (-2,2,8)								
**Quartz**	**(-1,0,4)**	**4.814**	**76.43**	**11.549**	**16.6**	**1.195**	**19.78**	**14.94**	**24.79**
**Quartz**	**(1,0,-4)**	**4.814**	**76.43**	**11.546**	**16.6**	**1.195**	**19.78**	**14.94**	**24.79**
	Equiv. Refl.: (-1,1,-4)								
**Quartz**	**(1,-1,4)**	**4.814**	**76.43**	**11.541**	**16.6**	**1.195**	**19.78**	**14.94**	**24.78**
**Quartz**	**(0,-1,-4)**	**4.814**	**76.43**	**11.538**	**16.5**	**1.195**	**19.77**	**14.94**	**24.78**
**Quartz**	**(0,1,4)**	**4.814**	**76.43**	**11.537**	**16.5**	**1.195**	**19.77**	**14.94**	**24.78**
**LiNbO3**	**(1,2,-7)**	**4.83**	**77.28**	**7.011**	**25.4**	**1.118**	**28.42**	**13.98**	**31.67**
	Equiv. Refl.: (1,-3,7), (2,1,7), (2,-3,-7), (3,-1,7), (3,-2,-7), (-1,3,-7), (-1,-2,7), (-2,3,7), (-2,-1,-7) (-3,1,-7), (-3,2,7)								

Ei =	4.945	keV							
Cryst	Refl	EB	ΘB	∫IR dΘ	Width	Ei cotΘ	ΔE	ΔEg	ΔEt
	(h,k,l)	[keV]	[°]	[µrad]	[µrad]	[meV/µrad]	[meV]	2m, 50µm [meV]	2m, 50µm [meV]
**Ge**	**(1,3,3)**	**4.776**	**75**	**119.6299**	**126.53**	**1.3255**	**167.71**	**16.57**	**168.52**
	Equiv. Refl.: (3,1,3), (3,3,1)								
**Quartz**	**(-3,3,2)**	**4.936**	**86.62**	**218.422**	**211.5**	**0.292**	**61.83**	**3.65**	**61.94**
**Quartz**	**(-3,0,-2)**	**4.936**	**86.62**	**218.417**	**211.5**	**0.292**	**61.83**	**3.65**	**61.94**
**Quartz**	**(0,3,-2)**	**4.936**	**86.62**	**218.412**	**211.5**	**0.292**	**61.83**	**3.65**	**61.94**
	Equiv. Refl.: (0,-3,2)								
**Quartz**	**(3,-3,-2)**	**4.936**	**86.62**	**218.406**	**211.5**	**0.292**	**61.83**	**3.65**	**61.94**
**Quartz**	**(3,0,2)**	**4.936**	**86.62**	**218.402**	**211.5**	**0.292**	**61.83**	**3.65**	**61.94**
**Quartz**	**(-3,3,-2)**	**4.936**	**86.62**	**172.111**	**176.9**	**0.292**	**51.7**	**3.65**	**51.83**
**Quartz**	**(3,-3,2)**	**4.936**	**86.62**	**172.109**	**176.9**	**0.292**	**51.7**	**3.65**	**51.83**
**Quartz**	**(-3,0,2)**	**4.936**	**86.62**	**172.102**	**176.9**	**0.292**	**51.7**	**3.65**	**51.83**
**Quartz**	**(3,0,-2)**	**4.936**	**86.62**	**172.098**	**176.9**	**0.292**	**51.7**	**3.65**	**51.83**
**Quartz**	**(0,3,2)**	**4.936**	**86.62**	**172.042**	**176.8**	**0.292**	**51.69**	**3.65**	**51.81**
	Equiv. Refl.: (0,-3,-2)								
**LiNbO3**	**(0,1,-10)**	**4.683**	**71.26**	**91.224**	**95.7**	**1.677**	**160.48**	**20.97**	**161.85**
	Equiv. Refl.: (1,-1,-10), (-1,0,-10)								
**LiNbO3**	**(2,2,0)**	**4.817**	**76.92**	**89.287**	**104.2**	**1.149**	**119.62**	**14.36**	**120.48**
	Equiv. Refl.: (-2,-2,0)								
**LiNbO3**	**(2,-4,0)**	**4.817**	**76.92**	**89.286**	**104.2**	**1.149**	**119.62**	**14.36**	**120.48**
	Equiv. Refl.: (4,-2,0), (-2,4,0), (-4,2,0)								
**LiNbO3**	**(0,-1,10)**	**4.683**	**71.26**	**84.02**	**95.7**	**1.677**	**160.48**	**20.97**	**161.85**
	Equiv. Refl.: (1,0,10), (-1,1,10)								
**Quartz**	**(0,-1,4)**	**4.814**	**76.78**	**62.093**	**59.9**	**1.162**	**69.61**	**14.53**	**71.1**
**Quartz**	**(0,1,-4)**	**4.814**	**76.78**	**62.092**	**59.9**	**1.162**	**69.61**	**14.53**	**71.1**
**Quartz**	**(1,0,4)**	**4.814**	**76.78**	**62.058**	**59.9**	**1.162**	**69.57**	**14.53**	**71.07**
	Equiv. Refl.: (-1,0,-4)								
**Quartz**	**(-1,1,4)**	**4.814**	**76.78**	**62.053**	**59.9**	**1.162**	**69.57**	**14.53**	**71.07**
**Quartz**	**(1,-1,-4)**	**4.814**	**76.78**	**62.052**	**59.9**	**1.162**	**69.57**	**14.53**	**71.07**
**LiNbO3**	**(1,1,9)**	**4.69**	**71.53**	**22.554**	**41.4**	**1.652**	**68.47**	**20.65**	**71.52**
	Equiv. Refl.: (1,1,-9), (1,-2,9), (1,-2,-9), (2,-1,9), (2,-1,-9), (-1,2,9), (-1,2,-9), (-1,-1,9), (-1,-1,-9) (-2,1,9), (-2,1,-9)								
**Sapph**	**(0,2,-8)**	**4.861**	**79.45**	**16.002**	**25.6**	**0.921**	**23.54**	**11.52**	**26.21**
	Equiv. Refl.: (0,-2,8), (2,0,8), (2,-2,-8), (-2,0,-8), (-2,2,8)								
**Quartz**	**(-1,0,4)**	**4.814**	**76.78**	**11.842**	**17**	**1.162**	**19.75**	**14.53**	**24.52**
**Quartz**	**(-1,1,-4)**	**4.814**	**76.78**	**11.84**	**17**	**1.162**	**19.75**	**14.53**	**24.52**
**Quartz**	**(1,0,-4)**	**4.814**	**76.78**	**11.839**	**17**	**1.162**	**19.75**	**14.53**	**24.52**
**Quartz**	**(1,-1,4)**	**4.814**	**76.78**	**11.834**	**17**	**1.162**	**19.75**	**14.53**	**24.52**
**Quartz**	**(0,-1,-4)**	**4.814**	**76.78**	**11.831**	**17**	**1.162**	**19.74**	**14.53**	**24.51**
**Quartz**	**(0,1,4)**	**4.814**	**76.78**	**11.83**	**17**	**1.162**	**19.74**	**14.53**	**24.51**
**LiNbO3**	**(1,2,-7)**	**4.83**	**77.64**	**7.209**	**26.2**	**1.084**	**28.38**	**13.55**	**31.44**
	Equiv. Refl.: (1,-3,7), (2,1,7), (2,-3,-7), (3,-1,7), (3,-2,-7), (-1,3,-7), (-1,-2,7), (-2,3,7), (-2,-1,-7) (-3,1,-7), (-3,2,7)								

Ei =	5.427	keV						ΔEg	ΔEt
Cryst	Refl	EB	ΘB	∫IR dΘ	Width	Ei cotΘ	ΔE	2m, 50µm	2m, 50µm
	(h,k,l)	[keV]	[°]	[µrad]	[µrad]	[meV/µrad]	[meV]	[meV]	[meV]
**Ge**	**(2,2,4)**	**5.368**	**81.56**	**242.2334**	**230.49**	**0.8054**	**185.64**	**10.07**	**185.92**
	Equiv. Refl.: (2,4,2), (4,2,2)								
**LiNbO3**	**(0,0,-12)**	**5.366**	**81.41**	**178.706**	**164.5**	**0.82**	**134.85**	**10.25**	**135.24**
**LiNbO3**	**(0,0,12)**	**5.366**	**81.41**	**134.629**	**164.5**	**0.82**	**134.85**	**10.25**	**135.24**
**Sapph**	**(2,2,3)**	**5.405**	**84.79**	**115.433**	**116.5**	**0.495**	**57.65**	**6.18**	**57.98**
	Equiv. Refl.: (2,2,-3), (2,-4,3), (2,-4,-3), (4,-2,3), (4,-2,-3), (-2,4,3), (-2,4,-3), (-2,-2,3), (-2,-2,-3) (-4,2,3), (-4,2,-3)								
**LiNbO3**	**(1,3,4)**	**5.323**	**78.77**	**85.119**	**92.8**	**1.078**	**100.08**	**13.47**	**100.98**
	Equiv. Refl.: (3,-4,4), (4,-3,4), (-1,4,4), (-3,-1,4), (-4,1,4)								
**LiNbO3**	**(0,2,10)**	**5.266**	**76.01**	**78.993**	**83.4**	**1.352**	**112.84**	**16.9**	**114.1**
	Equiv. Refl.: (2,-2,10), (-2,0,10)								
**LiNbO3**	**(1,-4,-4)**	**5.323**	**78.77**	**77.813**	**92.8**	**1.078**	**100.08**	**13.47**	**100.98**
	Equiv. Refl.: (3,1,-4), (4,-1,-4), (-1,-3,-4), (-3,4,-4), (-4,3,-4)								
**LiNbO3**	**(0,-2,-10)**	**5.266**	**76.01**	**71.497**	**83.4**	**1.352**	**112.84**	**16.9**	**114.1**
	Equiv. Refl.: (2,0,-10), (-2,2,-10)								
**Quartz**	**(-1,4,1)**	**5.377**	**82.22**	**67.446**	**63**	**0.742**	**46.74**	**9.27**	**47.65**
	Equiv. Refl.: (-1,-3,-1)								
**Quartz**	**(-3,4,-1)**	**5.377**	**82.22**	**67.444**	**63**	**0.742**	**46.74**	**9.27**	**47.65**
**Quartz**	**(-3,-1,1)**	**5.377**	**82.22**	**67.441**	**63**	**0.742**	**46.74**	**9.27**	**47.65**
**Quartz**	**(4,-1,-1)**	**5.377**	**82.22**	**67.433**	**63**	**0.742**	**46.73**	**9.27**	**47.64**
	Equiv. Refl.: (4,-3,1)								
**LiNbO3**	**(1,2,8)**	**5.131**	**71**	**62.5**	**67.2**	**1.868**	**125.63**	**23.35**	**127.78**
	Equiv. Refl.: (2,-3,8), (3,-2,8), (-1,3,8), (-2,-1,8), (-3,1,8)								
**LiNbO3**	**(1,-3,-8)**	**5.131**	**71**	**60.636**	**67.2**	**1.868**	**125.63**	**23.35**	**127.78**
	Equiv. Refl.: (2,1,-8), (3,-1,-8), (-1,-2,-8), (-2,3,-8), (-3,2,-8)								
**Quartz**	**(1,3,1)**	**5.377**	**82.22**	**60.099**	**63**	**0.742**	**46.74**	**9.27**	**47.65**
	Equiv. Refl.: (1,-4,-1)								
**Quartz**	**(3,-4,1)**	**5.377**	**82.22**	**60.098**	**63**	**0.742**	**46.74**	**9.27**	**47.65**
**Quartz**	**(3,1,-1)**	**5.377**	**82.22**	**60.094**	**63**	**0.742**	**46.74**	**9.27**	**47.65**
**Quartz**	**(-4,1,1)**	**5.377**	**82.22**	**60.087**	**63**	**0.742**	**46.73**	**9.27**	**47.64**
	Equiv. Refl.: (-4,3,-1)								
**Quartz**	**(4,-1,0)**	**5.253**	**75.46**	**48.56**	**44.9**	**1.407**	**63.13**	**17.59**	**65.53**
	Equiv. Refl.: (4,-3,0), (-1,4,0), (-1,-3,0), (-3,4,0), (-3,-1,0)								
**Quartz**	**(1,3,0)**	**5.253**	**75.46**	**47.449**	**44.9**	**1.407**	**63.13**	**17.59**	**65.53**
	Equiv. Refl.: (1,-4,0), (3,1,0), (3,-4,0), (-4,1,0), (-4,3,0)								
**Quartz**	**(-1,2,4)**	**5.236**	**74.77**	**43.358**	**39.5**	**1.478**	**58.45**	**18.47**	**61.3**
**Quartz**	**(-1,-1,-4)**	**5.236**	**74.77**	**43.357**	**39.5**	**1.478**	**58.45**	**18.47**	**61.3**
**Quartz**	**(-1,2,-4)**	**5.236**	**74.77**	**43.348**	**39.5**	**1.478**	**58.44**	**18.47**	**61.29**
**Quartz**	**(-1,-1,4)**	**5.236**	**74.77**	**43.341**	**39.5**	**1.478**	**58.43**	**18.47**	**61.28**
**Quartz**	**(2,-1,4)**	**5.236**	**74.77**	**43.332**	**39.5**	**1.478**	**58.42**	**18.47**	**61.27**
	Equiv. Refl.: (2,-1,-4)								
**Quartz**	**(1,1,4)**	**5.236**	**74.77**	**39.968**	**39.5**	**1.478**	**58.45**	**18.47**	**61.3**
	Equiv. Refl.: (1,-2,-4)								
**Quartz**	**(1,-2,4)**	**5.236**	**74.77**	**39.962**	**39.5**	**1.478**	**58.44**	**18.47**	**61.29**
**Quartz**	**(1,1,-4)**	**5.236**	**74.77**	**39.955**	**39.5**	**1.478**	**58.43**	**18.47**	**61.28**
**Quartz**	**(-2,1,4)**	**5.236**	**74.77**	**39.948**	**39.5**	**1.478**	**58.42**	**18.47**	**61.27**
	Equiv. Refl.: (-2,1,-4)								
**Quartz**	**(-3,2,3)**	**5.167**	**72.2**	**39.03**	**36.5**	**1.742**	**63.57**	**21.78**	**67.2**

Ei =	5.427	keV						ΔEg	ΔEt
Cryst	Refl	EB	ΘB	∫ IR dΘ	Width	Ei cotΘ	ΔE	2m, 50µm	2m, 50µm
	(h,k,l)	[keV]	[°]	[µrad]	[µrad]	[meV/µrad]	[meV]	[meV]	[meV]
Quartz	(-3,1,-3)	5.167	72.2	39.029	36.5	1.742	63.57	21.78	67.2
Quartz	(2,-3,-3)	5.167	72.2	39.022	36.5	1.742	63.56	21.78	67.19
Quartz	(2,1,3)	5.167	72.2	39.019	36.5	1.742	63.56	21.78	67.19
Quartz	(1,-3,3)	5.167	72.2	39.017	36.5	1.742	63.56	21.78	67.18
Quartz	(1,2,-3)	5.167	72.2	39.015	36.5	1.742	63.55	21.78	67.18
Quartz	(3,-1,3)	5.167	72.2	38.462	36.5	1.742	63.57	21.78	67.2
	Equiv. Refl.: (3,-2,-3)								
Quartz	(-2,3,3)	5.167	72.2	38.455	36.5	1.742	63.56	21.78	67.19
Quartz	(-2,-1,-3)	5.167	72.2	38.452	36.5	1.742	63.56	21.78	67.19
Quartz	(-1,3,-3)	5.167	72.2	38.449	36.5	1.742	63.56	21.78	67.18
Quartz	(-1,-2,3)	5.167	72.2	38.448	36.5	1.742	63.55	21.78	67.18
Sapph	(2,2,0)	5.211	73.8	30.074	31.4	1.577	49.55	19.71	53.32
	Equiv. Refl.: (2,-4,0), (4,-2,0), (-2,4,0), (-2,-2,0), (-4,2,0)								
Quartz	(-2,4,-1)	5.176	72.5	18.641	19.9	1.711	34.04	21.39	40.2
	Equiv. Refl.: (-2,-2,1)								
Quartz	(4,-2,1)	5.176	72.5	18.64	19.9	1.711	34.04	21.39	40.2
	Equiv. Refl.: (4,-2,-1)								
Quartz	(-2,4,1)	5.176	72.5	18.636	19.9	1.711	34.04	21.39	40.2
Quartz	(-2,-2,-1)	5.176	72.5	18.635	19.9	1.711	34.04	21.39	40.2
Quartz	(2,2,-1)	5.176	72.5	17.692	19.9	1.711	34.04	21.39	40.2
	Equiv. Refl.: (2,-4,1), (-4,2,1)								
Quartz	(-4,2,-1)	5.176	72.5	17.691	19.9	1.711	34.04	21.39	40.2
Quartz	(2,-4,-1)	5.176	72.5	17.688	19.9	1.711	34.04	21.39	40.2
Quartz	(2,2,1)	5.176	72.5	17.687	19.9	1.711	34.04	21.39	40.2
Quartz	(3,-1,-3)	5.167	72.2	15.809	16.8	1.742	29.28	21.78	36.49
Quartz	(3,-2,3)	5.167	72.2	15.808	16.8	1.742	29.28	21.78	36.49
Quartz	(-1,-2,-3)	5.167	72.2	15.794	16.8	1.742	29.26	21.78	36.47
Quartz	(-1,3,3)	5.167	72.2	15.792	16.8	1.742	29.26	21.78	36.47
	Equiv. Refl.: (-2,3,-3)								
Quartz	(-2,-1,3)	5.167	72.2	15.79	16.8	1.742	29.25	21.78	36.47
Quartz	(3,-4,-1)	5.377	82.22	14.918	19	0.742	14.09	9.27	16.86
Quartz	(3,1,1)	5.377	82.22	14.916	19	0.742	14.09	9.27	16.86
Quartz	(-4,1,-1)	5.377	82.22	14.913	19	0.742	14.08	9.27	16.86
Quartz	(-4,3,1)	5.377	82.22	14.911	19	0.742	14.08	9.27	16.86
Quartz	(1,3,-1)	5.377	82.22	14.909	19	0.742	14.08	9.27	16.86
	Equiv. Refl.: (1,-4,1)								
Quartz	(-3,1,3)	5.167	72.2	13.252	16.8	1.742	29.28	21.78	36.49
Quartz	(-3,2,-3)	5.167	72.2	13.251	16.8	1.742	29.28	21.78	36.49
Quartz	(1,2,3)	5.167	72.2	13.238	16.8	1.742	29.26	21.78	36.47
Quartz	(1,-3,-3)	5.167	72.2	13.236	16.8	1.742	29.26	21.78	36.47
Quartz	(2,-3,3)	5.167	72.2	13.235	16.8	1.742	29.26	21.78	36.47
Quartz	(2,1,-3)	5.167	72.2	13.233	16.8	1.742	29.25	21.78	36.47
Quartz	(-3,4,1)	5.377	82.22	10.552	19	0.742	14.09	9.27	16.86
Quartz	(-3,-1,-1)	5.377	82.22	10.551	19	0.742	14.09	9.27	16.86
Quartz	(4,-1,1)	5.377	82.22	10.547	19	0.742	14.08	9.27	16.86
Quartz	(4,-3,-1)	5.377	82.22	10.546	19	0.742	14.08	9.27	16.86
Quartz	(-1,4,-1)	5.377	82.22	10.544	19	0.742	14.08	9.27	16.86
	Equiv. Refl.: (-1,-3,1)								
Sapph	(1,2,-7)	5.197	73.25	9.025	13.5	1.634	21.98	20.42	30
	Equiv. Refl.: (1,-3,7), (2,1,7), (2,-3,-7), (3,-1,7), (3,-2,-7), (-1,3,-7), (-1,-2,7), (-2,3,7), (-2,-1,-7) (-3,1,-7), (-3,2,7)								
Sapph	(0,3,6)	5.345	80.04	4.183	9.9	0.953	9.39	11.92	15.17

Equiv. Refl.: (0,3,-6), (0,-3,6), (0,-3,-6), (3,0,6), (3,0,-6), (3,-3,6), (3,-3,-6), (-3,0,6), (-3,0,-6)
(-3,3,6), (-3,3,-6)

Ei = Cryst	5.989 Refl (h,k,l)	keV EB [keV]	ΘB [°]	∫IR dΘ [μrad]	Width [μrad]	Ei cotΘ [meV/μrad]	ΔE [meV]	ΔEg 2m, 50μm [meV]	ΔEt 2m, 50μm [meV]
**Ge**	**(3,3,3)**	**5.694**	**71.94**	**60.8**	**61.8**	**1.953**	**120.75**	**24.42**	**123.19**
	Equiv. Refl.: (1,1,5)								
**Si**	**(3,3,3)**	**5.931**	**82.03**	**61.9**	**61.3**	**0.839**	**51.45**	**10.48**	**52.51**
	Equiv. Refl.: (1,1,5)								
**Sapph**	**(2,2,6)**	**5.946**	**83.17**	**123**	**110**	**0.718**	**78.92**	**8.97**	**79.42**
	Equiv. Refl.: (2,2,-6), (2,-4,6), (2,-4,-6), (4,-2,6), (4,-2,-6), (-2,4,6), (-2,4,-6), (-2,-2,6), (-2,-2,-6) (-4,2,6), (-4,2,-6)								
**LiNbO3**	**(1,1,12)**	**5.882**	**79.14**	**86.5**	**84.1**	**1.149**	**96.58**	**14.36**	**97.64**
	Equiv. Refl.: (1,-2,12), (2,-1,12), (-1,2,12), (-1,-1,12), (-2,1,12)								
**Quartz**	**(0,4,-1)**	**5.94**	**82.65**	**78.1**	**70.9**	**0.773**	**54.8**	**9.66**	**55.64**
	Equiv. Refl.: (0,-4,1), (4,0,1), (4,-4,-1), (-4,0,-1), (-4,4,1)								
**LiNbO3**	**(1,1,-12)**	**5.882**	**79.14**	**70.6**	**84.1**	**1.149**	**96.58**	**14.36**	**97.64**
	Equiv. Refl.: (1,-2,-12), (2,-1,-12), (-1,2,-12), (-1,-1,-12), (-2,1,-12)								
**Quartz**	**(0,1,5)**	**5.917**	**81.12**	**70.3**	**63.1**	**0.935**	**59.01**	**11.69**	**60.16**
	Equiv. Refl.: (0,-1,-5), (1,0,-5), (1,-1,5), (-1,0,5), (-1,1,-5)								
**LiNbO3**	**(1,2,-10)**	**5.791**	**75.21**	**63.9**	**66.3**	**1.581**	**104.84**	**19.76**	**106.69**
	Equiv. Refl.: (2,-3,-10), (3,-2,-10), (-1,3,-10), (-2,-1,-10), (-3,1,-10)								
**LiNbO3**	**(1,-3,10)**	**5.791**	**75.21**	**61.5**	**66.3**	**1.581**	**104.84**	**19.76**	**106.69**
	Equiv. Refl.: (2,1,10), (3,-1,10), (-1,-2,10), (-2,3,10), (-3,2,10)								
**LiNbO3**	**(0,-4,-2)**	**5.633**	**70.16**	**59.4**	**58.4**	**2.161**	**126.13**	**27.02**	**128.99**
	Equiv. Refl.: (4,0,-2), (-4,4,-2)								
**LiNbO3**	**(0,-4,4)**	**5.842**	**77.3**	**57.2**	**62.7**	**1.35**	**84.63**	**16.87**	**86.29**
	Equiv. Refl.: (4,0,4), (-4,4,4)								
**LiNbO3**	**(0,4,-4)**	**5.842**	**77.3**	**55.5**	**62.7**	**1.35**	**84.63**	**16.87**	**86.29**
	Equiv. Refl.: (4,-4,-4), (-4,0,-4)								
**LiNbO3**	**(0,4,2)**	**5.633**	**70.16**	**54.5**	**58.4**	**2.161**	**126.13**	**27.02**	**128.99**
	Equiv. Refl.: (4,-4,2), (-4,0,2)								
**Sapph**	**(0,0,12)**	**5.727**	**73.01**	**48.7**	**44.1**	**1.83**	**80.81**	**22.88**	**83.98**
	Equiv. Refl.: (0,0,-12)								
**Quartz**	**(4,-1,-2)**	**5.732**	**73.16**	**34.3**	**30.5**	**1.812**	**55.3**	**22.65**	**59.76**
	Equiv. Refl.: (4,-3,2), (-1,4,2), (-1,-3,-2), (-3,4,-2), (-3,-1,2)								
**Quartz**	**(1,3,2)**	**5.732**	**73.16**	**32.6**	**30.5**	**1.812**	**55.29**	**22.65**	**59.75**
	Equiv. Refl.: (1,-4,-2), (3,1,-2), (3,-4,2), (-4,1,2), (-4,3,-2)								
**Sapph**	**(0,2,10)**	**5.642**	**70.4**	**32.2**	**30.1**	**2.132**	**64.14**	**26.65**	**69.46**
	Equiv. Refl.: (0,-2,-10), (2,0,-10), (2,-2,10), (-2,0,10), (-2,2,-10)								
**Sapph**	**(1,3,4)**	**5.75**	**73.77**	**26.5**	**26.4**	**1.743**	**46.09**	**21.79**	**50.98**
	Equiv. Refl.: (1,-4,-4), (3,1,-4), (3,-4,4), (4,-1,-4), (4,-3,4), (-1,4,4), (-1,-3,-4), (-3,4,-4), (-3,-1,4) (-4,1,4), (-4,3,-4)								
**Quartz**	**(3,1,2)**	**5.732**	**73.16**	**21.2**	**19.7**	**1.812**	**35.68**	**22.65**	**42.26**
	Equiv. Refl.: (3,-4,-2), (-4,1,-2), (-4,3,2)								
**Quartz**	**(1,3,-2)**	**5.732**	**73.16**	**21.1**	**19.7**	**1.812**	**35.66**	**22.65**	**42.25**
	Equiv. Refl.: (1,-4,2)								
**Quartz**	**(0,1,-5)**	**5.917**	**81.12**	**20.2**	**22.4**	**0.935**	**21**	**11.69**	**24.03**
	Equiv. Refl.: (0,-1,5), (1,0,5), (1,-1,-5), (-1,0,-5), (-1,1,5)								
**Quartz**	**(0,4,0)**	**5.828**	**76.68**	**18.5**	**19.4**	**1.418**	**27.52**	**17.72**	**32.74**
	Equiv. Refl.: (0,-4,0), (4,0,0), (4,-1,2), (4,-3,-2), (4,-4,0), (-1,4,-2), (-1,-3,2), (-3,4,2), (-3,-1,-2) (-4,0,0), (-4,4,0)								
**Quartz**	**(0,4,1)**	**5.94**	**82.65**	**11.4**	**16.2**	**0.773**	**12.48**	**9.66**	**15.78**
	Equiv. Refl.: (0,-4,-1), (4,0,-1), (4,-4,1), (-4,0,1), (-4,4,-1)								

Ei = Cryst	5.989 Refl (h,k,l)	keV EB [keV]	ΘB [°]	∫ IR dΘ [μrad]	Width [μrad]	Ei cotΘ [meV/μrad]	ΔE [meV]	ΔEg 2m, 50μm [meV]	ΔEt 2m, 50μm [meV]
**LiNbO3**	**(1,3,7)**	**5.91**	**80.71**	**10.8**	**26.3**	**0.98**	**25.74**	**12.25**	**28.51**
	Equiv. Refl.: (1,-4,-7), (3,1,-7), (3,-4,7), (4,-1,-7), (4,-3,7), (-1,4,7), (-1,-3,-7), (-3,4,-7), (-3,-1,7) (-4,1,7), (-4,3,-7)								
**Sapph**	**(1,3,-5)**	**5.926**	**81.68**	**7.2**	**12.9**	**0.875**	**11.27**	**10.94**	**15.71**
	Equiv. Refl.: (1,-4,5), (3,1,5), (3,-4,-5), (4,-1,5), (4,-3,-5), (-1,4,-5), (-1,-3,5), (-3,4,5), (-3,-1,-5) (-4,1,-5), (-4,3,5)								

Ei =	5.415	keV						ΔEg	ΔEt
**Cryst**	**Refl**	**EB**	**ΘB**	**∫IR dΘ**	**Width**	**Ei cotΘ**	**ΔE**	**2m, 50µm**	**2m, 50µm**
	**(h,k,l)**	**[keV]**	**[°]**	**[µrad]**	**[µrad]**	**[meV/µrad]**	**[meV]**	**[meV]**	**[meV]**
**Ge**	**(2,2,4)**	**5.368**	**82.46**	**271.5288**	**258.55**	**0.7166**	**185.27**	**8.96**	**185.48**
	Equiv. Refl.: (2,4,2), (4,2,2)								
**LiNbO3**	**(0,0,-12)**	**5.366**	**82.3**	**199.459**	**183.7**	**0.733**	**134.55**	**9.16**	**134.86**
**Sapph**	**(2,2,3)**	**5.405**	**86.45**	**169.315**	**171.1**	**0.336**	**57.52**	**4.2**	**57.68**
	Equiv. Refl.: (2,2,-3), (2,-4,3), (2,-4,-3), (4,-2,3), (4,-2,-3), (-2,4,3), (-2,4,-3), (-2,-2,3), (-2,-2,-3) (-4,2,3), (-4,2,-3)								
**LiNbO3**	**(0,0,12)**	**5.366**	**82.3**	**150.111**	**183.7**	**0.733**	**134.55**	**9.16**	**134.86**
**LiNbO3**	**(1,3,4)**	**5.323**	**79.42**	**90.467**	**98.8**	**1.011**	**99.85**	**12.64**	**100.64**
	Equiv. Refl.: (3,-4,4), (4,-3,4), (-3,-1,4)								
**LiNbO3**	**(-1,4,4)**	**5.323**	**79.42**	**90.466**	**98.8**	**1.011**	**99.85**	**12.64**	**100.64**
	Equiv. Refl.: (-4,1,4)								
**LiNbO3**	**(1,-4,-4)**	**5.323**	**79.42**	**82.675**	**98.8**	**1.011**	**99.85**	**12.64**	**100.64**
	Equiv. Refl.: (3,1,-4), (4,-1,-4), (-1,-3,-4), (-3,4,-4), (-4,3,-4)								
**LiNbO3**	**(0,2,10)**	**5.266**	**76.53**	**82.086**	**86.8**	**1.297**	**112.59**	**16.22**	**113.75**
	Equiv. Refl.: (2,-2,10), (-2,0,10)								
**Quartz**	**(-1,4,1)**	**5.377**	**83.21**	**77.358**	**72.3**	**0.645**	**46.64**	**8.06**	**47.33**
	Equiv. Refl.: (-1,-3,-1)								
**Quartz**	**(-3,4,-1)**	**5.377**	**83.21**	**77.356**	**72.3**	**0.645**	**46.64**	**8.06**	**47.33**
**Quartz**	**(-3,-1,1)**	**5.377**	**83.21**	**77.353**	**72.3**	**0.645**	**46.64**	**8.06**	**47.33**
**Quartz**	**(4,-1,-1)**	**5.377**	**83.21**	**77.343**	**72.3**	**0.645**	**46.63**	**8.06**	**47.32**
	Equiv. Refl.: (4,-3,1)								
**LiNbO3**	**(1,3,-2)**	**5.093**	**70.13**	**74.867**	**76.2**	**1.957**	**149.04**	**24.46**	**151.03**
	Equiv. Refl.: (3,-4,-2), (4,-3,-2), (-1,4,-2), (-3,-1,-2), (-4,1,-2)								
**LiNbO3**	**(-2,2,-10)**	**5.266**	**76.53**	**74.272**	**86.8**	**1.297**	**112.59**	**16.22**	**113.75**
**LiNbO3**	**(0,-2,-10)**	**5.266**	**76.53**	**74.271**	**86.8**	**1.297**	**112.59**	**16.22**	**113.75**
	Equiv. Refl.: (2,0,-10)								
**LiNbO3**	**(1,-4,2)**	**5.093**	**70.13**	**70.314**	**76.2**	**1.957**	**149.04**	**24.46**	**151.03**
	Equiv. Refl.: (3,1,2), (4,-1,2), (-1,-3,2), (-3,4,2), (-4,3,2)								
**Quartz**	**(1,3,1)**	**5.377**	**83.21**	**68.901**	**72.3**	**0.645**	**46.64**	**8.06**	**47.33**
**Quartz**	**(1,-4,-1)**	**5.377**	**83.21**	**68.9**	**72.3**	**0.645**	**46.64**	**8.06**	**47.33**
**Quartz**	**(3,-4,1)**	**5.377**	**83.21**	**68.899**	**72.3**	**0.645**	**46.64**	**8.06**	**47.33**
**Quartz**	**(3,1,-1)**	**5.377**	**83.21**	**68.895**	**72.3**	**0.645**	**46.64**	**8.06**	**47.33**
**Quartz**	**(-4,1,1)**	**5.377**	**83.21**	**68.886**	**72.3**	**0.645**	**46.63**	**8.06**	**47.32**
	Equiv. Refl.: (-4,3,-1)								
**LiNbO3**	**(1,2,8)**	**5.131**	**71.38**	**63.784**	**68.7**	**1.825**	**125.34**	**22.81**	**127.4**
	Equiv. Refl.: (2,-3,8), (3,-2,8), (-1,3,8), (-2,-1,8), (-3,1,8)								
**LiNbO3**	**(1,-3,-8)**	**5.131**	**71.38**	**61.876**	**68.7**	**1.825**	**125.34**	**22.81**	**127.4**
	Equiv. Refl.: (2,1,-8), (3,-1,-8), (-1,-2,-8), (-2,3,-8), (-3,2,-8)								
**Quartz**	**(4,-1,0)**	**5.253**	**75.96**	**50.325**	**46.5**	**1.354**	**62.99**	**16.93**	**65.23**
	Equiv. Refl.: (4,-3,0), (-1,4,0), (-1,-3,0), (-3,4,0), (-3,-1,0)								
**Quartz**	**(1,3,0)**	**5.253**	**75.96**	**49.169**	**46.5**	**1.354**	**62.99**	**16.93**	**65.23**
	Equiv. Refl.: (1,-4,0), (3,1,0), (3,-4,0), (-4,1,0), (-4,3,0)								
**Quartz**	**(-1,2,4)**	**5.236**	**75.24**	**44.79**	**40.9**	**1.427**	**58.32**	**17.83**	**60.99**
**Quartz**	**(-1,-1,-4)**	**5.236**	**75.24**	**44.789**	**40.9**	**1.427**	**58.32**	**17.83**	**60.99**
**Quartz**	**(-1,2,-4)**	**5.236**	**75.24**	**44.779**	**40.9**	**1.427**	**58.31**	**17.83**	**60.98**
**Quartz**	**(-1,-1,4)**	**5.236**	**75.24**	**44.772**	**40.9**	**1.427**	**58.31**	**17.83**	**60.97**
**Quartz**	**(2,-1,-4)**	**5.236**	**75.24**	**44.763**	**40.9**	**1.427**	**58.3**	**17.83**	**60.96**
**Quartz**	**(2,-1,4)**	**5.236**	**75.24**	**44.762**	**40.9**	**1.427**	**58.3**	**17.83**	**60.96**

Ei =	5.415	keV						ΔEg	ΔEt
Cryst	Refl	EB	ΘB	∫ IR dΘ	Width	Ei cotΘ	ΔE	2m, 50µm	2m, 50µm
	(h,k,l)	[keV]	[°]	[µrad]	[µrad]	[meV/µrad]	[meV]	[meV]	[meV]
Quartz	(1,1,4)	5.236	75.24	41.275	40.9	1.427	58.32	17.83	60.99
	Equiv. Refl.: (1,-2,-4)								
Quartz	(1,-2,4)	5.236	75.24	41.268	40.9	1.427	58.31	17.83	60.98
Quartz	(1,1,-4)	5.236	75.24	41.262	40.9	1.427	58.31	17.83	60.97
Quartz	(-2,1,4)	5.236	75.24	41.254	40.9	1.427	58.3	17.83	60.96
Quartz	(-2,1,-4)	5.236	75.24	41.253	40.9	1.427	58.3	17.83	60.96
Quartz	(-3,2,3)	5.167	72.6	39.959	37.4	1.697	63.44	21.21	66.89
Quartz	(-3,1,-3)	5.167	72.6	39.958	37.4	1.697	63.44	21.21	66.89
Quartz	(2,-3,-3)	5.167	72.6	39.951	37.4	1.697	63.43	21.21	66.88
Quartz	(2,1,3)	5.167	72.6	39.948	37.4	1.697	63.42	21.21	66.88
Quartz	(1,-3,3)	5.167	72.6	39.946	37.4	1.697	63.42	21.21	66.87
Quartz	(1,2,-3)	5.167	72.6	39.944	37.4	1.697	63.42	21.21	66.87
Quartz	(3,-1,3)	5.167	72.6	39.375	37.4	1.697	63.44	21.21	66.89
	Equiv. Refl.: (3,-2,-3)								
Quartz	(-2,3,3)	5.167	72.6	39.368	37.4	1.697	63.43	21.21	66.88
Quartz	(-2,-1,-3)	5.167	72.6	39.365	37.4	1.697	63.42	21.21	66.88
Quartz	(-1,3,-3)	5.167	72.6	39.362	37.4	1.697	63.42	21.21	66.87
Quartz	(-1,-2,3)	5.167	72.6	39.361	37.4	1.697	63.42	21.21	66.87
Sapph	(2,2,0)	5.211	74.24	30.936	32.4	1.528	49.44	19.1	53
	Equiv. Refl.: (2,-4,0), (4,-2,0), (-2,4,0), (-2,-2,0), (-4,2,0)								
Quartz	(4,-2,-1)	5.176	72.91	19.091	20.4	1.665	33.97	20.81	39.84
	Equiv. Refl.: (-2,4,-1), (-2,-2,1)								
Quartz	(4,-2,1)	5.176	72.91	19.09	20.4	1.665	33.97	20.81	39.84
Quartz	(-2,4,1)	5.176	72.91	19.086	20.4	1.665	33.96	20.81	39.83
	Equiv. Refl.: (-2,-2,-1)								
Quartz	(2,2,-1)	5.176	72.91	18.116	20.4	1.665	33.97	20.81	39.84
	Equiv. Refl.: (2,-4,1), (-4,2,1), (-4,2,-1)								
Quartz	(2,-4,-1)	5.176	72.91	18.112	20.4	1.665	33.96	20.81	39.83
Quartz	(2,2,1)	5.176	72.91	18.111	20.4	1.665	33.96	20.81	39.83
Quartz	(3,-4,-1)	5.377	83.21	17.096	21.8	0.645	14.06	8.06	16.2
Quartz	(3,1,1)	5.377	83.21	17.094	21.8	0.645	14.06	8.06	16.2
Quartz	(-4,1,-1)	5.377	83.21	17.091	21.8	0.645	14.05	8.06	16.2
Quartz	(-4,3,1)	5.377	83.21	17.088	21.8	0.645	14.05	8.06	16.2
Quartz	(1,3,-1)	5.377	83.21	17.086	21.8	0.645	14.05	8.06	16.2
	Equiv. Refl.: (1,-4,1)								
Quartz	(3,-1,-3)	5.167	72.6	16.179	17.2	1.697	29.22	21.21	36.1
Quartz	(3,-2,3)	5.167	72.6	16.178	17.2	1.697	29.22	21.21	36.1
Quartz	(-1,-2,-3)	5.167	72.6	16.163	17.2	1.697	29.2	21.21	36.09
Quartz	(-1,3,3)	5.167	72.6	16.161	17.2	1.697	29.19	21.21	36.08
	Equiv. Refl.: (-2,3,-3)								
Quartz	(-2,-1,3)	5.167	72.6	16.159	17.2	1.697	29.19	21.21	36.08
Quartz	(-3,1,3)	5.167	72.6	13.552	17.2	1.697	29.22	21.21	36.1
	Equiv. Refl.: (-3,2,-3)								
Quartz	(1,2,3)	5.167	72.6	13.538	17.2	1.697	29.2	21.21	36.09
Quartz	(1,-3,-3)	5.167	72.6	13.536	17.2	1.697	29.19	21.21	36.08
Quartz	(2,-3,3)	5.167	72.6	13.535	17.2	1.697	29.19	21.21	36.08
Quartz	(2,1,-3)	5.167	72.6	13.533	17.2	1.697	29.19	21.21	36.08
Quartz	(-3,4,1)	5.377	83.21	12.077	21.8	0.645	14.06	8.06	16.2
Quartz	(-3,-1,-1)	5.377	83.21	12.076	21.8	0.645	14.06	8.06	16.2
Quartz	(4,-1,1)	5.377	83.21	12.071	21.8	0.645	14.05	8.06	16.2

Ei = Cryst	5.415 Refl (h,k,l)	keV EB [keV]	ΘB [°]	∫ IR dΘ [µrad]	Width [µrad]	Ei cotΘ [meV/µrad]	ΔE [meV]	ΔEg 2m, 50µm [meV]	ΔEt 2m, 50µm [meV]
**Quartz**	**(4,-3,-1)**	**5.377**	**83.21**	**12.07**	**21.8**	**0.645**	**14.05**	**8.06**	**16.2**
**Quartz**	**(-1,4,-1)**	**5.377**	**83.21**	**12.068**	**21.8**	**0.645**	**14.05**	**8.06**	**16.2**
	Equiv. Refl.: (-1,-3,1)								
**Sapph**	**(1,2,-7)**	**5.197**	**73.68**	**9.254**	**13.8**	**1.586**	**21.93**	**19.83**	**29.56**
	Equiv. Refl.: (1,-3,7), (2,1,7), (2,-3,-7), (3,-1,7), (3,-2,-7), (-1,3,-7), (-1,-2,7), (-2,3,7), (-2,-1,-7) (-3,1,-7), (-3,2,7)								
**Sapph**	**(0,3,6)**	**5.345**	**80.79**	**4.521**	**10.7**	**0.878**	**9.37**	**10.98**	**14.44**
	Equiv. Refl.: (0,3,-6), (0,-3,6), (0,-3,-6), (3,0,6), (3,0,-6), (3,-3,6), (3,-3,-6), (-3,0,6), (-3,0,-6) (-3,3,6), (-3,3,-6)								

Ei =	5.406	keV						ΔEg	ΔEt
Cryst	Refl	EB	ΘB	∫ IR dΘ	Width	Ei cotΘ	ΔE	2m, 50µm	2m, 50µm
	(h,k,l)	[keV]	[°]	[µrad]	[µrad]	[meV/µrad]	[meV]	[meV]	[meV]
**Ge**	**(2,2,4)**	**5.368**	**83.22**	**302.1536**	**287.85**	**0.6426**	**184.99**	**8.03**	**185.16**
	Equiv. Refl.: (2,4,2), (4,2,2)								
**Sapph**	**(2,2,3)**	**5.405**	**88.69**	**460.396**	**465.6**	**0.123**	**57.43**	**1.54**	**57.45**
	Equiv. Refl.: (2,2,-3), (2,-4,3), (2,-4,-3), (4,-2,3), (4,-2,-3), (-2,4,3), (-2,4,-3), (-2,-2,3), (-2,-2,-3) (-4,2,3), (-4,2,-3)								
**LiNbO3**	**(0,0,-12)**	**5.366**	**83.04**	**220.839**	**203.4**	**0.66**	**134.32**	**8.25**	**134.58**
**LiNbO3**	**(0,0,12)**	**5.366**	**83.04**	**166.073**	**203.4**	**0.66**	**134.32**	**8.25**	**134.58**
**LiNbO3**	**(1,3,4)**	**5.323**	**79.95**	**95.219**	**104**	**0.958**	**99.68**	**11.98**	**100.39**
	Equiv. Refl.: (3,-4,4), (4,-3,4), (-1,4,4), (-3,-1,4), (-4,1,4)								
**Quartz**	**(-1,4,1)**	**5.377**	**84.06**	**88.546**	**82.8**	**0.562**	**46.56**	**7.03**	**47.09**
	Equiv. Refl.: (-1,-3,-1)								
**Quartz**	**(-3,4,-1)**	**5.377**	**84.06**	**88.544**	**82.8**	**0.562**	**46.56**	**7.03**	**47.09**
**Quartz**	**(-3,-1,1)**	**5.377**	**84.06**	**88.54**	**82.8**	**0.562**	**46.56**	**7.03**	**47.09**
**Quartz**	**(4,-1,-1)**	**5.377**	**84.06**	**88.529**	**82.8**	**0.562**	**46.56**	**7.03**	**47.08**
	Equiv. Refl.: (4,-3,1)								
**LiNbO3**	**(1,-4,-4)**	**5.323**	**79.95**	**86.998**	**104**	**0.958**	**99.68**	**11.98**	**100.39**
	Equiv. Refl.: (3,1,-4), (4,-1,-4), (-1,-3,-4), (-3,4,-4), (-4,3,-4)								
**LiNbO3**	**(0,2,10)**	**5.266**	**76.93**	**84.664**	**89.6**	**1.255**	**112.4**	**15.69**	**113.49**
	Equiv. Refl.: (2,-2,10), (-2,0,10)								
**Quartz**	**(1,3,1)**	**5.377**	**84.06**	**78.838**	**82.8**	**0.562**	**46.56**	**7.03**	**47.09**
	Equiv. Refl.: (1,-4,-1)								
**Quartz**	**(3,-4,1)**	**5.377**	**84.06**	**78.837**	**82.8**	**0.562**	**46.56**	**7.03**	**47.09**
**Quartz**	**(3,1,-1)**	**5.377**	**84.06**	**78.832**	**82.8**	**0.562**	**46.56**	**7.03**	**47.09**
**Quartz**	**(-4,1,1)**	**5.377**	**84.06**	**78.822**	**82.8**	**0.562**	**46.56**	**7.03**	**47.08**
	Equiv. Refl.: (-4,3,-1)								
**LiNbO3**	**(-2,2,-10)**	**5.266**	**76.93**	**76.584**	**89.6**	**1.255**	**112.4**	**15.69**	**113.49**
**LiNbO3**	**(0,-2,-10)**	**5.266**	**76.93**	**76.583**	**89.6**	**1.255**	**112.4**	**15.69**	**113.49**
	Equiv. Refl.: (2,0,-10)								
**LiNbO3**	**(1,3,-2)**	**5.093**	**70.4**	**75.922**	**77.3**	**1.925**	**148.79**	**24.07**	**150.72**
	Equiv. Refl.: (3,-4,-2), (4,-3,-2), (-1,4,-2), (-3,-1,-2), (-4,1,-2)								
**LiNbO3**	**(1,-4,2)**	**5.093**	**70.4**	**71.292**	**77.3**	**1.925**	**148.79**	**24.07**	**150.72**
	Equiv. Refl.: (3,1,2), (4,-1,2), (-1,-3,2), (-3,4,2), (-4,3,2)								
**LiNbO3**	**(1,2,8)**	**5.131**	**71.66**	**64.803**	**69.8**	**1.792**	**125.13**	**22.4**	**127.12**
	Equiv. Refl.: (2,-3,8), (3,-2,8), (-1,3,8), (-2,-1,8), (-3,1,8)								
**LiNbO3**	**(1,-3,-8)**	**5.131**	**71.66**	**62.859**	**69.8**	**1.792**	**125.13**	**22.4**	**127.12**
	Equiv. Refl.: (2,1,-8), (3,-1,-8), (-1,-2,-8), (-2,3,-8), (-3,2,-8)								
**Quartz**	**(4,-1,0)**	**5.253**	**76.35**	**51.784**	**47.9**	**1.313**	**62.89**	**16.41**	**65**
	Equiv. Refl.: (4,-3,0), (-1,4,0), (-1,-3,0), (-3,4,0), (-3,-1,0)								
**Quartz**	**(1,3,0)**	**5.253**	**76.35**	**50.591**	**47.9**	**1.313**	**62.89**	**16.41**	**65**
	Equiv. Refl.: (1,-4,0), (3,1,0), (3,-4,0), (-4,1,0), (-4,3,0)								
**Quartz**	**(-1,2,4)**	**5.236**	**75.61**	**45.962**	**42**	**1.387**	**58.23**	**17.34**	**60.76**
**Quartz**	**(-1,-1,-4)**	**5.236**	**75.61**	**45.961**	**42**	**1.387**	**58.23**	**17.34**	**60.76**
**Quartz**	**(-1,2,-4)**	**5.236**	**75.61**	**45.951**	**42**	**1.387**	**58.22**	**17.34**	**60.75**
**Quartz**	**(-1,-1,4)**	**5.236**	**75.61**	**45.944**	**42**	**1.387**	**58.21**	**17.34**	**60.74**
**Quartz**	**(2,-1,4)**	**5.236**	**75.61**	**45.934**	**42**	**1.387**	**58.2**	**17.34**	**60.73**
	Equiv. Refl.: (2,-1,-4)								
**Quartz**	**(1,1,4)**	**5.236**	**75.61**	**42.345**	**42**	**1.387**	**58.23**	**17.34**	**60.76**
	Equiv. Refl.: (1,-2,-4)								

Ei =	5.406	keV						ΔEg	ΔEt
Cryst	Refl	EB	ΘB	∫ IR dΘ	Width	Ei cotΘ	ΔE	2m, 50µm	2m, 50µm
	(h,k,l)	[keV]	[°]	[µrad]	[µrad]	[meV/µrad]	[meV]	[meV]	[meV]
**Quartz**	**(1,-2,4)**	**5.236**	**75.61**	**42.338**	**42**	**1.387**	**58.22**	**17.34**	**60.75**
**Quartz**	**(1,1,-4)**	**5.236**	**75.61**	**42.331**	**42**	**1.387**	**58.21**	**17.34**	**60.74**
**Quartz**	**(-2,1,4)**	**5.236**	**75.61**	**42.324**	**42**	**1.387**	**58.2**	**17.34**	**60.73**
**Quartz**	**(-2,1,-4)**	**5.236**	**75.61**	**42.323**	**42**	**1.387**	**58.2**	**17.34**	**60.73**
**Quartz**	**(-3,1,-3)**	**5.167**	**72.91**	**40.701**	**38.1**	**1.662**	**63.33**	**20.78**	**66.66**
	Equiv. Refl.: (-3,2,3)								
**Quartz**	**(2,-3,-3)**	**5.167**	**72.91**	**40.693**	**38.1**	**1.662**	**63.33**	**20.78**	**66.65**
**Quartz**	**(2,1,3)**	**5.167**	**72.91**	**40.69**	**38.1**	**1.662**	**63.32**	**20.78**	**66.64**
**Quartz**	**(1,-3,3)**	**5.167**	**72.91**	**40.688**	**38.1**	**1.662**	**63.32**	**20.78**	**66.64**
**Quartz**	**(1,2,-3)**	**5.167**	**72.91**	**40.686**	**38.1**	**1.662**	**63.32**	**20.78**	**66.64**
**Quartz**	**(3,-1,3)**	**5.167**	**72.91**	**40.105**	**38.1**	**1.662**	**63.33**	**20.78**	**66.66**
	Equiv. Refl.: (3,-2,-3)								
**Quartz**	**(-2,3,3)**	**5.167**	**72.91**	**40.098**	**38.1**	**1.662**	**63.33**	**20.78**	**66.65**
**Quartz**	**(-2,-1,-3)**	**5.167**	**72.91**	**40.095**	**38.1**	**1.662**	**63.32**	**20.78**	**66.64**
**Quartz**	**(-1,3,-3)**	**5.167**	**72.91**	**40.092**	**38.1**	**1.662**	**63.32**	**20.78**	**66.64**
**Quartz**	**(-1,-2,3)**	**5.167**	**72.91**	**40.09**	**38.1**	**1.662**	**63.32**	**20.78**	**66.64**
**Sapph**	**(2,2,0)**	**5.211**	**74.58**	**31.635**	**33.1**	**1.491**	**49.36**	**18.64**	**52.76**
	Equiv. Refl.: (2,-4,0), (4,-2,0), (-2,4,0), (-2,-2,0), (-4,2,0)								
**Quartz**	**(3,-4,-1)**	**5.377**	**84.06**	**19.556**	**25**	**0.562**	**14.03**	**7.03**	**15.69**
**Quartz**	**(3,1,1)**	**5.377**	**84.06**	**19.554**	**25**	**0.562**	**14.03**	**7.03**	**15.69**
**Quartz**	**(-4,1,-1)**	**5.377**	**84.06**	**19.55**	**25**	**0.562**	**14.03**	**7.03**	**15.69**
**Quartz**	**(-4,3,1)**	**5.377**	**84.06**	**19.547**	**25**	**0.562**	**14.03**	**7.03**	**15.69**
**Quartz**	**(1,3,-1)**	**5.377**	**84.06**	**19.545**	**25**	**0.562**	**14.03**	**7.03**	**15.69**
**Quartz**	**(1,-4,1)**	**5.377**	**84.06**	**19.544**	**25**	**0.562**	**14.03**	**7.03**	**15.69**
**Quartz**	**(-2,4,-1)**	**5.176**	**73.22**	**19.453**	**20.8**	**1.63**	**33.92**	**20.38**	**39.57**
**Quartz**	**(4,-2,-1)**	**5.176**	**73.22**	**19.452**	**20.8**	**1.63**	**33.92**	**20.38**	**39.57**
	Equiv. Refl.: (-2,-2,1)								
**Quartz**	**(4,-2,1)**	**5.176**	**73.22**	**19.451**	**20.8**	**1.63**	**33.92**	**20.38**	**39.56**
**Quartz**	**(-2,4,1)**	**5.176**	**73.22**	**19.447**	**20.8**	**1.63**	**33.91**	**20.38**	**39.56**
	Equiv. Refl.: (-2,-2,-1)								
**Quartz**	**(2,2,-1)**	**5.176**	**73.22**	**18.456**	**20.8**	**1.63**	**33.92**	**20.38**	**39.57**
	Equiv. Refl.: (2,-4,1), (-4,2,1)								
**Quartz**	**(-4,2,-1)**	**5.176**	**73.22**	**18.455**	**20.8**	**1.63**	**33.92**	**20.38**	**39.56**
**Quartz**	**(2,2,1)**	**5.176**	**73.22**	**18.451**	**20.8**	**1.63**	**33.91**	**20.38**	**39.56**
	Equiv. Refl.: (2,-4,-1)								
**Quartz**	**(3,-1,-3)**	**5.167**	**72.91**	**16.474**	**17.5**	**1.662**	**29.17**	**20.78**	**35.81**
	Equiv. Refl.: (3,-2,3)								
**Quartz**	**(-1,-2,-3)**	**5.167**	**72.91**	**16.459**	**17.5**	**1.662**	**29.15**	**20.78**	**35.8**
**Quartz**	**(-1,3,3)**	**5.167**	**72.91**	**16.456**	**17.5**	**1.662**	**29.15**	**20.78**	**35.79**
	Equiv. Refl.: (-2,3,-3)								
**Quartz**	**(-2,-1,3)**	**5.167**	**72.91**	**16.454**	**17.5**	**1.662**	**29.14**	**20.78**	**35.79**
**Quartz**	**(-3,4,1)**	**5.377**	**84.06**	**13.801**	**25**	**0.562**	**14.03**	**7.03**	**15.69**
**Quartz**	**(-3,-1,-1)**	**5.377**	**84.06**	**13.8**	**25**	**0.562**	**14.03**	**7.03**	**15.69**
**Quartz**	**(4,-1,1)**	**5.377**	**84.06**	**13.795**	**25**	**0.562**	**14.03**	**7.03**	**15.69**
**Quartz**	**(4,-3,-1)**	**5.377**	**84.06**	**13.793**	**25**	**0.562**	**14.03**	**7.03**	**15.69**
	Equiv. Refl.: (-3,1,3)								
**Quartz**	**(-3,2,-3)**	**5.167**	**72.91**	**13.792**	**17.5**	**1.662**	**29.17**	**20.78**	**35.81**
**Quartz**	**(-1,4,-1)**	**5.377**	**84.06**	**13.791**	**25**	**0.562**	**14.03**	**7.03**	**15.69**
	Equiv. Refl.: (-1,-3,1)								
**Quartz**	**(1,2,3)**	**5.167**	**72.91**	**13.778**	**17.5**	**1.662**	**29.15**	**20.78**	**35.8**

Ei = Cryst	5.406 Refl (h,k,l)	keV EB [keV]	ΘB [°]	∫ IR dΘ [µrad]	Width [µrad]	Ei cotΘ [meV/µrad]	ΔE [meV]	ΔEg 2m, 50µm [meV]	ΔEt 2m, 50µm [meV]
**Quartz**	**(1,-3,-3)**	**5.167**	**72.91**	**13.776**	**17.5**	**1.662**	**29.15**	**20.78**	**35.79**
**Quartz**	**(2,-3,3)**	**5.167**	**72.91**	**13.775**	**17.5**	**1.662**	**29.15**	**20.78**	**35.79**
**Quartz**	**(2,1,-3)**	**5.167**	**72.91**	**13.773**	**17.5**	**1.662**	**29.14**	**20.78**	**35.79**
**Sapph**	**(1,2,-7)**	**5.197**	**74**	**9.439**	**14.1**	**1.55**	**21.9**	**19.37**	**29.23**
	Equiv. Refl.: (1,-3,7), (2,1,7), (2,-3,-7), (3,-1,7), (3,-2,-7), (-1,3,-7), (-1,-2,7), (-2,3,7), (-2,-1,-7) (-3,1,-7), (-3,2,7)								
**Sapph**	**(0,3,6)**	**5.345**	**81.39**	**4.837**	**11.4**	**0.818**	**9.36**	**10.23**	**13.86**
	Equiv. Refl.: (0,3,-6), (0,-3,6), (0,-3,-6), (3,0,6), (3,0,-6), (3,-3,6), (3,-3,-6), (-3,0,6), (-3,0,-6) (-3,3,6), (-3,3,-6)								

Ei =	5.947	keV						ΔEg	ΔEt
Cryst	Refl	EB	ΘB	∫IR dΘ	Width	Ei cotΘ	ΔE	2m, 50µm	2m, 50µm
	(h,k,l)	[keV]	[°]	[µrad]	[µrad]	[meV/µrad]	[meV]	[meV]	[meV]
**Si**	**(3,3,3)**	**5.931**	**85.81**	**117.9891**	**117.35**	**0.4355**	**51.1**	**5.44**	**51.39**
	Equiv. Refl.: (1,5,1), (1,1,5), (5,1,1)								
**Ge**	**(3,3,3)**	**5.694**	**73.22**	**65.6364**	**66.92**	**1.793**	**119.98**	**22.41**	**122.06**
	Equiv. Refl.: (1,5,1), (1,1,5), (5,1,1)								
**Si**	**(2,2,4)**	**5.592**	**70.1**	**43.3955**	**39.64**	**2.1527**	**85.33**	**26.91**	**89.47**
	Equiv. Refl.: (2,4,2), (4,2,2)								
**Sapph**	**(2,2,6)**	**5.946**	**89.23**	**1095.134**	**980.8**	**0.08**	**78.39**	**1**	**78.39**
	Equiv. Refl.: (2,2,-6), (2,-4,6), (2,-4,-6), (4,-2,6), (4,-2,-6), (-2,4,6), (-2,4,-6), (-2,-2,6), (-2,-2,-6) (-4,2,6), (-4,2,-6)								
**Quartz**	**(4,-4,-1)**	**5.94**	**87.17**	**203.635**	**185.1**	**0.294**	**54.43**	**3.68**	**54.55**
**Quartz**	**(-4,4,1)**	**5.94**	**87.17**	**203.634**	**185.1**	**0.294**	**54.43**	**3.68**	**54.55**
**Quartz**	**(4,0,1)**	**5.94**	**87.17**	**203.631**	**185.1**	**0.294**	**54.43**	**3.68**	**54.55**
**Quartz**	**(-4,0,-1)**	**5.94**	**87.17**	**203.63**	**185.1**	**0.294**	**54.43**	**3.68**	**54.55**
**Quartz**	**(0,4,-1)**	**5.94**	**87.17**	**203.623**	**185.1**	**0.294**	**54.43**	**3.68**	**54.55**
	Equiv. Refl.: (0,-4,1)								
**LiNbO3**	**(1,1,12)**	**5.882**	**81.51**	**110.867**	**108**	**0.888**	**95.91**	**11.1**	**96.55**
	Equiv. Refl.: (1,-2,12), (2,-1,12), (-1,2,12), (-1,-1,12), (-2,1,12)								
**Quartz**	**(1,-1,5)**	**5.917**	**84.27**	**109.272**	**98.3**	**0.597**	**58.65**	**7.46**	**59.12**
**Quartz**	**(-1,1,-5)**	**5.917**	**84.27**	**109.271**	**98.3**	**0.597**	**58.65**	**7.46**	**59.12**
**Quartz**	**(-1,0,5)**	**5.917**	**84.27**	**109.263**	**98.3**	**0.597**	**58.65**	**7.46**	**59.12**
**Quartz**	**(1,0,-5)**	**5.917**	**84.27**	**109.262**	**98.3**	**0.597**	**58.65**	**7.46**	**59.12**
**Quartz**	**(0,1,5)**	**5.917**	**84.27**	**109.193**	**98.2**	**0.597**	**58.61**	**7.46**	**59.09**
**Quartz**	**(0,-1,-5)**	**5.917**	**84.27**	**109.192**	**98.2**	**0.597**	**58.61**	**7.46**	**59.09**
**LiNbO3**	**(1,1,-12)**	**5.882**	**81.51**	**90.338**	**108**	**0.888**	**95.91**	**11.1**	**96.55**
	Equiv. Refl.: (1,-2,-12), (2,-1,-12), (-1,2,-12), (-1,-1,-12), (-2,1,-12)								
**LiNbO3**	**(1,2,-10)**	**5.791**	**76.83**	**71.948**	**74.8**	**1.392**	**104.11**	**17.39**	**105.56**
	Equiv. Refl.: (2,-3,-10), (3,-2,-10), (-1,3,-10), (-2,-1,-10), (-3,1,-10)								
**LiNbO3**	**(1,-3,10)**	**5.791**	**76.83**	**69.177**	**74.8**	**1.392**	**104.11**	**17.39**	**105.56**
	Equiv. Refl.: (2,1,10), (3,-1,10), (-1,-2,10), (-2,3,10), (-3,2,10)								
**LiNbO3**	**(0,-4,4)**	**5.842**	**79.24**	**67.646**	**74.4**	**1.13**	**84.04**	**14.12**	**85.22**
	Equiv. Refl.: (4,0,4), (-4,4,4)								
**LiNbO3**	**(0,4,-4)**	**5.842**	**79.24**	**65.57**	**74.4**	**1.13**	**84.04**	**14.12**	**85.22**
	Equiv. Refl.: (4,-4,-4), (-4,0,-4)								
**LiNbO3**	**(0,-4,-2)**	**5.633**	**71.31**	**63.254**	**62.3**	**2.012**	**125.26**	**25.15**	**127.75**
	Equiv. Refl.: (4,0,-2), (-4,4,-2)								
**LiNbO3**	**(0,4,2)**	**5.633**	**71.31**	**57.982**	**62.3**	**2.012**	**125.26**	**25.15**	**127.75**
	Equiv. Refl.: (4,-4,2), (-4,0,2)								
**Sapph**	**(0,0,12)**	**5.727**	**74.38**	**53.204**	**48.3**	**1.662**	**80.26**	**20.78**	**82.91**
	Equiv. Refl.: (0,0,-12)								
**Quartz**	**(4,-3,2)**	**5.732**	**74.56**	**37.491**	**33.4**	**1.643**	**54.93**	**20.54**	**58.64**
**Quartz**	**(4,-1,-2)**	**5.732**	**74.56**	**37.49**	**33.4**	**1.643**	**54.93**	**20.54**	**58.64**
**Quartz**	**(-3,4,-2)**	**5.732**	**74.56**	**37.487**	**33.4**	**1.643**	**54.93**	**20.54**	**58.64**
	Equiv. Refl.: (-3,-1,2)								
**Quartz**	**(-1,-3,-2)**	**5.732**	**74.56**	**37.483**	**33.4**	**1.643**	**54.92**	**20.54**	**58.63**
**Quartz**	**(-1,4,2)**	**5.732**	**74.56**	**37.481**	**33.4**	**1.643**	**54.92**	**20.54**	**58.63**
**Quartz**	**(-4,1,2)**	**5.732**	**74.56**	**35.585**	**33.4**	**1.643**	**54.93**	**20.54**	**58.64**
	Equiv. Refl.: (-4,3,-2)								

Ei =	5.947	keV						ΔEg	ΔEt
Cryst	Refl	EB	ΘB	∫ IR dΘ	Width	Ei cotΘ	ΔE	2m, 50µm	2m, 50µm
	(h,k,l)	[keV]	[°]	[µrad]	[µrad]	[meV/µrad]	[meV]	[meV]	[meV]
**Quartz**	**(3,1,-2)**	**5.732**	**74.56**	**35.583**	**33.4**	**1.643**	**54.93**	**20.54**	**58.64**
	Equiv. Refl.: (3,-4,2)								
**Quartz**	**(1,3,2)**	**5.732**	**74.56**	**35.579**	**33.4**	**1.643**	**54.92**	**20.54**	**58.63**
**Quartz**	**(1,-4,-2)**	**5.732**	**74.56**	**35.577**	**33.4**	**1.643**	**54.92**	**20.54**	**58.63**
**Sapph**	**(0,2,10)**	**5.642**	**71.58**	**34.292**	**32.2**	**1.981**	**63.71**	**24.76**	**68.36**
	Equiv. Refl.: (0,-2,-10), (2,0,-10), (2,-2,10), (-2,0,10), (-2,2,-10)								
**Quartz**	**(-1,1,5)**	**5.917**	**84.27**	**30.866**	**34.9**	**0.597**	**20.86**	**7.46**	**22.15**
**Quartz**	**(0,1,-5)**	**5.917**	**84.27**	**30.863**	**34.9**	**0.597**	**20.86**	**7.46**	**22.15**
	Equiv. Refl.: (1,-1,-5)								
**Quartz**	**(0,-1,5)**	**5.917**	**84.27**	**30.862**	**34.9**	**0.597**	**20.86**	**7.46**	**22.15**
**Quartz**	**(-1,0,-5)**	**5.917**	**84.27**	**30.86**	**34.9**	**0.597**	**20.85**	**7.46**	**22.15**
**Quartz**	**(1,0,5)**	**5.917**	**84.27**	**30.855**	**34.9**	**0.597**	**20.85**	**7.46**	**22.15**
**Quartz**	**(0,4,1)**	**5.94**	**87.17**	**29.306**	**42.2**	**0.294**	**12.4**	**3.68**	**12.93**
	Equiv. Refl.: (0,-4,-1)								
**Quartz**	**(4,0,-1)**	**5.94**	**87.17**	**29.275**	**42.1**	**0.294**	**12.39**	**3.68**	**12.92**
	Equiv. Refl.: (4,-4,1)								
**Quartz**	**(-4,0,1)**	**5.94**	**87.17**	**29.274**	**42.1**	**0.294**	**12.39**	**3.68**	**12.92**
**Quartz**	**(-4,4,-1)**	**5.94**	**87.17**	**29.273**	**42.1**	**0.294**	**12.39**	**3.68**	**12.92**
**Sapph**	**(1,3,4)**	**5.75**	**75.23**	**29.18**	**29.2**	**1.568**	**45.78**	**19.6**	**49.8**
	Equiv. Refl.: (1,-4,-4), (3,1,-4), (3,-4,4), (4,-1,-4), (4,-3,4), (-1,4,4), (-1,-3,-4), (-3,4,-4), (-3,-1,4) (-4,1,4), (-4,3,-4)								
**Quartz**	**(3,-4,-2)**	**5.732**	**74.56**	**23.123**	**21.6**	**1.643**	**35.44**	**20.54**	**40.96**
	Equiv. Refl.: (-4,1,-2)								
**Quartz**	**(-4,3,2)**	**5.732**	**74.56**	**23.122**	**21.6**	**1.643**	**35.43**	**20.54**	**40.95**
**Quartz**	**(3,1,2)**	**5.732**	**74.56**	**23.121**	**21.6**	**1.643**	**35.43**	**20.54**	**40.95**
**Quartz**	**(1,3,-2)**	**5.732**	**74.56**	**23.113**	**21.6**	**1.643**	**35.42**	**20.54**	**40.94**
**Quartz**	**(1,-4,2)**	**5.732**	**74.56**	**23.112**	**21.6**	**1.643**	**35.42**	**20.54**	**40.94**
**Quartz**	**(0,4,0)**	**5.828**	**78.52**	**21.522**	**22.6**	**1.208**	**27.34**	**15.1**	**31.23**
	Equiv. Refl.: (0,-4,0), (4,0,0), (4,-4,0), (-4,0,0), (-4,4,0)								
**Quartz**	**(-3,4,2)**	**5.732**	**74.56**	**20.144**	**21.6**	**1.643**	**35.44**	**20.54**	**40.96**
**Quartz**	**(4,-1,2)**	**5.732**	**74.56**	**20.143**	**21.6**	**1.643**	**35.43**	**20.54**	**40.96**
**Quartz**	**(4,-3,-2)**	**5.732**	**74.56**	**20.142**	**21.6**	**1.643**	**35.43**	**20.54**	**40.95**
	Equiv. Refl.: (-3,-1,-2)								
**Quartz**	**(-1,-3,2)**	**5.732**	**74.56**	**20.134**	**21.6**	**1.643**	**35.42**	**20.54**	**40.94**
**Quartz**	**(-1,4,-2)**	**5.732**	**74.56**	**20.133**	**21.6**	**1.643**	**35.42**	**20.54**	**40.94**
**LiNbO3**	**(1,3,7)**	**5.91**	**83.64**	**15.737**	**38.6**	**0.662**	**25.57**	**8.28**	**26.88**
	Equiv. Refl.: (1,-4,-7), (3,1,-7), (3,-4,7), (4,-1,-7), (4,-3,7), (-1,4,7), (-1,-3,-7), (-3,4,-7), (-3,-1,7) (-4,1,7), (-4,3,-7)								
**Sapph**	**(1,3,-5)**	**5.926**	**85.19**	**12.227**	**22.4**	**0.501**	**11.19**	**6.26**	**12.83**
	Equiv. Refl.: (1,-4,5), (3,1,5), (3,-4,-5), (4,-1,5), (4,-3,-5), (-1,4,-5), (-1,-3,5), (-3,4,5), (-3,-1,-5) (-4,1,-5), (-4,3,5)								

Ei =	6.539	keV						ΔEg	ΔEt
**Cryst**	**Refl**	**EB**	**ΘB**	**∫ IR dΘ**	**Width**	**Ei cotΘ**	**ΔE**	**2m, 50µm**	**2m, 50µm**
	**(h,k,l)**	**[keV]**	**[°]**	**[µrad]**	**[µrad]**	**[meV/µrad]**	**[meV]**	**[meV]**	**[meV]**
**Ge**	**(1,3,5)**	**6.483**	**82.48**	**99.8**	**101.5**	**0.864**	**87.63**	**10.79**	**88.29**
**Ge**	**(0,4,4)**	**6.199**	**71.43**	**70.1**	**65**	**2.196**	**142.67**	**27.45**	**145.28**
**Si**	**(0,4,4)**	**6.457**	**80.92**	**65**	**59**	**1.046**	**61.72**	**13.07**	**63.08**
**LiNbO3**	**(0,-1,-14)**	**6.413**	**78.74**	**68.9**	**69.2**	**1.302**	**90.13**	**16.28**	**91.59**
	Equiv. Refl.: (1,0,-14), (-1,1,-14)								
**LiNbO3**	**(2,3,-4)**	**6.319**	**75.11**	**68.6**	**66.7**	**1.739**	**115.98**	**21.73**	**118**
	Equiv. Refl.: (3,-5,-4), (5,-3,-4), (-2,5,-4), (-3,-2,-4), (-5,2,-4)								
**LiNbO3**	**(2,-5,4)**	**6.319**	**75.11**	**67.5**	**66.7**	**1.739**	**115.98**	**21.73**	**118**
	Equiv. Refl.: (3,2,4), (5,-2,4), (-2,-3,4), (-3,5,4), (-5,3,4)								
**LiNbO3**	**(0,1,14)**	**6.413**	**78.74**	**65.6**	**69.2**	**1.302**	**90.13**	**16.28**	**91.59**
	Equiv. Refl.: (1,-1,14), (-1,0,14)								
**LiNbO3**	**(1,-5,0)**	**6.372**	**77.02**	**50.1**	**52.9**	**1.507**	**79.73**	**18.83**	**81.93**
	Equiv. Refl.: (4,1,0), (5,-1,0), (-1,-4,0), (-4,5,0), (-5,4,0)								
**LiNbO3**	**(1,4,0)**	**6.372**	**77.02**	**49.5**	**52.9**	**1.507**	**79.73**	**18.83**	**81.93**
	Equiv. Refl.: (4,-5,0), (5,-4,0), (-1,5,0), (-4,-1,0), (-5,1,0)								
**LiNbO3**	**(1,-4,8)**	**6.159**	**70.37**	**40.7**	**39.6**	**2.332**	**92.32**	**29.15**	**96.81**
	Equiv. Refl.: (3,1,8), (4,-1,8), (-1,-3,8), (-3,4,8), (-4,3,8)								
**Quartz**	**(5,-2,-1)**	**6.454**	**80.73**	**39.5**	**35.5**	**1.067**	**37.88**	**13.34**	**40.16**
	Equiv. Refl.: (5,-3,1), (-2,5,1), (-2,-3,-1), (-3,5,-1), (-3,-2,1)								
**Quartz**	**(2,3,1)**	**6.454**	**80.73**	**37.8**	**35.5**	**1.067**	**37.88**	**13.34**	**40.16**
	Equiv. Refl.: (2,-5,-1), (3,2,-1), (3,-5,1), (-5,2,1), (-5,3,-1)								
**LiNbO3**	**(1,3,-8)**	**6.159**	**70.37**	**35.2**	**39.6**	**2.332**	**92.32**	**29.15**	**96.81**
	Equiv. Refl.: (3,-4,-8), (4,-3,-8), (-1,4,-8), (-3,-1,-8), (-4,1,-8)								
**Sapph**	**(1,2,-10)**	**6.215**	**71.88**	**30.3**	**27.4**	**2.14**	**58.71**	**26.74**	**64.52**
	Equiv. Refl.: (1,-3,10), (2,1,10), (2,-3,-10), (3,-1,10), (3,-2,-10), (-1,3,-10), (-1,-2,10), (-2,3,10), (-2,-1,-10) (-3,1,-10), (-3,2,10)								
**Quartz**	**(2,3,0)**	**6.351**	**76.22**	**24.4**	**22.6**	**1.603**	**36.28**	**20.04**	**41.45**
	Equiv. Refl.: (2,-5,0), (3,2,0), (3,-5,0), (-5,2,0), (-5,3,0)								
**Quartz**	**(5,-2,0)**	**6.351**	**76.22**	**24.3**	**22.6**	**1.603**	**36.28**	**20.04**	**41.45**
	Equiv. Refl.: (5,-3,0), (-2,5,0), (-2,-3,0), (-3,5,0), (-3,-2,0)								
**Quartz**	**(2,-1,5)**	**6.266**	**73.38**	**19.3**	**17.3**	**1.952**	**33.78**	**24.4**	**41.67**
	Equiv. Refl.: (2,-1,-5)								
**Quartz**	**(-1,2,5)**	**6.266**	**73.38**	**19.2**	**17.3**	**1.952**	**33.77**	**24.4**	**41.66**
	Equiv. Refl.: (-1,2,-5), (-1,-1,5), (-1,-1,-5)								
**Quartz**	**(1,1,5)**	**6.266**	**73.38**	**17.6**	**17.3**	**1.952**	**33.77**	**24.4**	**41.67**
	Equiv. Refl.: (1,1,-5), (1,-2,5), (1,-2,-5), (-2,1,5), (-2,1,-5)								
**Sapph**	**(0,4,-4)**	**6.313**	**74.9**	**14.4**	**15.2**	**1.764**	**26.76**	**22.05**	**34.68**
	Equiv. Refl.: (0,-4,4), (4,0,4), (4,-4,-4), (-4,0,-4), (-4,4,4)								
**LiNbO3**	**(1,4,3)**	**6.512**	**84.76**	**12**	**30.9**	**0.599**	**18.55**	**7.49**	**20**
	Equiv. Refl.: (1,4,-3), (1,-5,3), (1,-5,-3), (4,1,3), (4,1,-3), (4,-5,3), (4,-5,-3), (5,-1,3), (5,-1,-3) (5,-4,3), (5,-4,-3), (-1,5,3), (-1,5,-3), (-1,-4,3), (-1,-4,-3), (-4,5,3), (-4,5,-3), (-4,-1,3) (-4,-1,-3), (-5,1,3), (-5,1,-3), (-5,4,3), (-5,4,-3)								
**Quartz**	**(0,4,2)**	**6.263**	**73.3**	**11.3**	**11.7**	**1.962**	**22.98**	**24.52**	**33.6**
	Equiv. Refl.: (0,-4,-2), (4,0,-2), (4,-4,2), (-4,0,2), (-4,4,-2)								
**LiNbO3**	**(2,2,9)**	**6.277**	**73.72**	**8.8**	**16.1**	**1.91**	**30.8**	**23.87**	**38.96**
	Equiv. Refl.: (2,2,-9), (2,-4,9), (2,-4,-9), (4,-2,9), (4,-2,-9), (-2,4,9), (-2,4,-9), (-2,-2,9), (-2,-2,-9) (-4,2,9), (-4,2,-9)								
**Sapph**	**(1,1,12)**	**6.292**	**74.21**	**8**	**9.9**	**1.849**	**18.33**	**23.11**	**29.5**
	Equiv. Refl.: (1,1,-12), (1,-2,12), (1,-2,-12), (2,-1,12), (2,-1,-12), (-1,2,12), (-1,2,-12), (-1,-1,12), (-1,-1,-12) (-2,1,12), (-2,1,-12)								

Ei = Cryst	6.539 Refl (h,k,l)	keV EB [keV]	ΘB [°]	∫ IR dΘ [µrad]	Width [µrad]	Ei cotΘ [meV/µrad]	ΔE [meV]	ΔEg 2m, 50µm [meV]	ΔEt 2m, 50µm [meV]
**Quartz**	**(0,3,-4)**	**6.337**	**75.72**	**7.7**	**9.1**	**1.665**	**15.1**	**20.81**	**25.71**
	Equiv. Refl.: (0,-3,4), (3,0,4), (3,-3,-4), (-3,0,-4), (-3,3,4)								
**Quartz**	**(4,-1,-3)**	**6.28**	**73.82**	**6.1**	**7.3**	**1.898**	**13.76**	**23.72**	**27.42**
	Equiv. Refl.: (4,-3,3), (-1,4,3), (-1,-3,-3), (-3,4,-3), (-3,-1,3)								
**Quartz**	**(1,3,-3)**	**6.28**	**73.82**	**6**	**6.5**	**1.898**	**12.34**	**23.72**	**26.74**
	Equiv. Refl.: (1,-4,3), (3,1,3), (3,-4,-3), (-4,1,-3), (-4,3,3)								
**Quartz**	**(1,3,3)**	**6.28**	**73.82**	**5.9**	**7.3**	**1.898**	**13.76**	**23.72**	**27.42**
	Equiv. Refl.: (1,-4,-3), (3,1,-3), (3,-4,3), (-4,1,3), (-4,3,-3)								
**Quartz**	**(0,2,-5)**	**6.433**	**79.67**	**4.8**	**7.2**	**1.192**	**8.62**	**14.9**	**17.22**
	Equiv. Refl.: (0,-2,5)								
**Quartz**	**(0,2,5)**	**6.433**	**79.67**	**4.7**	**7.1**	**1.192**	**8.52**	**14.9**	**17.17**
	Equiv. Refl.: (0,-2,-5), (2,0,5), (2,0,-5), (2,-2,5), (2,-2,-5), (-2,0,5), (-2,0,-5), (-2,2,5), (-2,2,-5)								
**Quartz**	**(4,-1,3)**	**6.28**	**73.82**	**4.3**	**6.5**	**1.898**	**12.33**	**23.72**	**26.73**
	Equiv. Refl.: (4,-3,-3), (-1,4,-3), (-1,-3,3), (-3,4,3), (-3,-1,-3)								
**Sapph**	**(1,3,7)**	**6.371**	**76.97**	**3.5**	**6.5**	**1.513**	**9.77**	**18.91**	**21.29**
	Equiv. Refl.: (1,-4,-7), (3,1,-7), (3,-4,7), (4,-1,-7), (4,-3,7), (-1,4,7), (-1,-3,-7), (-3,4,-7), (-3,-1,7) (-4,1,7), (-4,3,-7)								
**Quartz**	**(0,3,4)**	**6.337**	**75.72**	**2.9**	**4.8**	**1.665**	**7.92**	**20.81**	**22.27**
	Equiv. Refl.: (0,-3,-4), (3,0,-4), (3,-3,4), (-3,0,4), (-3,3,-4)								
**LiNbO3**	**(2,3,5)**	**6.46**	**81.1**	**1.8**	**9.2**	**1.024**	**9.38**	**12.8**	**15.87**
	Equiv. Refl.: (2,-5,-5), (3,2,-5), (3,-5,5), (5,-2,-5), (5,-3,5), (-2,5,5), (-2,-3,-5), (-3,5,-5), (-3,-2,5) (-5,2,5), (-5,3,-5)								
**Quartz**	**(0,4,-2)**	**6.263**	**73.3**	**0.9**	**2.3**	**1.962**	**4.45**	**24.52**	**24.92**
	Equiv. Refl.: (0,-4,2), (4,0,2), (4,-4,-2), (-4,0,-2), (-4,4,2)								

Ei =	5.899	keV						ΔEg	ΔEt
Cryst	Refl	EB	ΘB	∫ IR dΘ	Width	Ei cotΘ	ΔE	2m, 50µm	2m, 50µm
	(h,k,l)	[keV]	[°]	[µrad]	[µrad]	[meV/µrad]	[meV]	[meV]	[meV]
**Ge**	**(3,3,3)**	**5.694**	**74.84**	**72.8796**	**74.54**	**1.5978**	**119.11**	**19.97**	**120.77**
	Equiv. Refl.: (1,5,1), (1,1,5), (5,1,1)								
**Si**	**(2,2,4)**	**5.592**	**71.43**	**46.6722**	**42.73**	**1.9816**	**84.67**	**24.77**	**88.22**
	Equiv. Refl.: (2,4,2), (4,2,2)								
**LiNbO3**	**(1,-2,12)**	**5.882**	**85.62**	**215.835**	**210.8**	**0.451**	**95.14**	**5.64**	**95.3**
	Equiv. Refl.: (2,-1,12)								
**LiNbO3**	**(1,1,12)**	**5.882**	**85.62**	**215.834**	**210.8**	**0.451**	**95.14**	**5.64**	**95.3**
	Equiv. Refl.: (-1,2,12), (-1,-1,12), (-2,1,12)								
**LiNbO3**	**(1,1,-12)**	**5.882**	**85.62**	**175.406**	**210.8**	**0.451**	**95.14**	**5.64**	**95.3**
	Equiv. Refl.: (1,-2,-12), (2,-1,-12), (-1,2,-12), (-1,-1,-12), (-2,1,-12)								
**LiNbO3**	**(0,-4,4)**	**5.842**	**82.06**	**91.865**	**101.4**	**0.822**	**83.36**	**10.28**	**83.99**
	Equiv. Refl.: (4,0,4)								
**LiNbO3**	**(-4,4,4)**	**5.842**	**82.06**	**91.864**	**101.4**	**0.822**	**83.36**	**10.28**	**83.99**
**LiNbO3**	**(0,4,-4)**	**5.842**	**82.06**	**89.01**	**101.4**	**0.822**	**83.36**	**10.28**	**83.99**
	Equiv. Refl.: (-4,0,-4)								
**LiNbO3**	**(4,-4,-4)**	**5.842**	**82.06**	**89.009**	**101.4**	**0.822**	**83.36**	**10.28**	**83.99**
**LiNbO3**	**(1,2,-10)**	**5.791**	**79**	**86.372**	**90.1**	**1.147**	**103.28**	**14.34**	**104.27**
	Equiv. Refl.: (-1,3,-10), (-2,-1,-10), (-3,1,-10)								
**LiNbO3**	**(2,-3,-10)**	**5.791**	**79**	**86.371**	**90.1**	**1.147**	**103.28**	**14.34**	**104.27**
	Equiv. Refl.: (3,-2,-10)								
**LiNbO3**	**(1,-3,10)**	**5.791**	**79**	**83.003**	**90.1**	**1.147**	**103.28**	**14.34**	**104.27**
	Equiv. Refl.: (2,1,10), (3,-1,10), (-1,-2,10)								
**LiNbO3**	**(-2,3,10)**	**5.791**	**79**	**83.002**	**90.1**	**1.147**	**103.28**	**14.34**	**104.27**
	Equiv. Refl.: (-3,2,10)								
**LiNbO3**	**(0,-4,-2)**	**5.633**	**72.74**	**68.707**	**67.8**	**1.833**	**124.26**	**22.91**	**126.35**
	Equiv. Refl.: (4,0,-2), (-4,4,-2)								
**LiNbO3**	**(0,4,2)**	**5.633**	**72.74**	**62.908**	**67.8**	**1.833**	**124.26**	**22.91**	**126.35**
	Equiv. Refl.: (4,-4,2), (-4,0,2)								
**Sapph**	**(0,0,12)**	**5.727**	**76.15**	**60.187**	**54.8**	**1.454**	**79.63**	**18.18**	**81.68**
	Equiv. Refl.: (0,0,-12)								
**Quartz**	**(4,-1,-2)**	**5.732**	**76.35**	**42.556**	**38**	**1.433**	**54.5**	**17.91**	**57.37**
	Equiv. Refl.: (4,-3,2)								
**Quartz**	**(-3,4,-2)**	**5.732**	**76.35**	**42.552**	**38**	**1.433**	**54.5**	**17.91**	**57.37**
	Equiv. Refl.: (-3,-1,2)								
**Quartz**	**(-1,-3,-2)**	**5.732**	**76.35**	**42.548**	**38**	**1.433**	**54.49**	**17.91**	**57.36**
**Quartz**	**(-1,4,2)**	**5.732**	**76.35**	**42.545**	**38**	**1.433**	**54.49**	**17.91**	**57.36**
**Quartz**	**(-4,1,2)**	**5.732**	**76.35**	**40.363**	**38**	**1.433**	**54.5**	**17.91**	**57.37**
	Equiv. Refl.: (-4,3,-2)								
**Quartz**	**(3,1,-2)**	**5.732**	**76.35**	**40.361**	**38**	**1.433**	**54.5**	**17.91**	**57.37**
	Equiv. Refl.: (3,-4,2)								
**Quartz**	**(1,3,2)**	**5.732**	**76.35**	**40.356**	**38**	**1.433**	**54.49**	**17.91**	**57.36**
**Quartz**	**(1,-4,-2)**	**5.732**	**76.35**	**40.354**	**38**	**1.433**	**54.49**	**17.91**	**57.36**
**Sapph**	**(0,2,10)**	**5.642**	**73.03**	**37.35**	**35.1**	**1.8**	**63.23**	**22.5**	**67.11**
	Equiv. Refl.: (0,-2,-10), (2,0,-10), (2,-2,10), (-2,0,10), (-2,2,-10)								
**Sapph**	**(1,3,4)**	**5.75**	**77.11**	**33.53**	**33.7**	**1.35**	**45.43**	**16.87**	**48.47**
	Equiv. Refl.: (1,-4,-4), (3,1,-4), (3,-4,4), (4,-1,-4), (4,-3,4), (-1,4,4), (-1,-3,-4), (-3,4,-4), (-3,-1,4) (-4,1,4), (-4,3,-4)								

Ei =	5.899	keV						ΔEg	ΔEt
**Cryst**	**Refl**	**EB**	**ΘB**	**∫ IR dΘ**	**Width**	**Ei cotΘ**	**ΔE**	**2m, 50µm**	**2m, 50µm**
	**(h,k,l)**	**[keV]**	**[°]**	**[µrad]**	**[µrad]**	**[meV/µrad]**	**[meV]**	**[meV]**	**[meV]**
**Quartz**	**(0,4,0)**	**5.828**	**81.1**	**27.814**	**29.4**	**0.924**	**27.13**	**11.55**	**29.49**
	Equiv. Refl.: (0,-4,0), (4,0,0), (4,-4,0), (-4,0,0), (-4,4,0)								
**Quartz**	**(3,-4,-2)**	**5.732**	**76.35**	**26.227**	**24.5**	**1.433**	**35.16**	**17.91**	**39.46**
	Equiv. Refl.: (-4,1,-2)								
**Quartz**	**(-4,3,2)**	**5.732**	**76.35**	**26.226**	**24.5**	**1.433**	**35.16**	**17.91**	**39.46**
**Quartz**	**(3,1,2)**	**5.732**	**76.35**	**26.225**	**24.5**	**1.433**	**35.16**	**17.91**	**39.46**
**Quartz**	**(1,3,-2)**	**5.732**	**76.35**	**26.215**	**24.5**	**1.433**	**35.15**	**17.91**	**39.45**
**Quartz**	**(1,-4,2)**	**5.732**	**76.35**	**26.214**	**24.5**	**1.433**	**35.14**	**17.91**	**39.45**
**Quartz**	**(4,-1,2)**	**5.732**	**76.35**	**22.802**	**24.5**	**1.433**	**35.16**	**17.91**	**39.46**
	Equiv. Refl.: (-3,4,2)								
**Quartz**	**(4,-3,-2)**	**5.732**	**76.35**	**22.801**	**24.5**	**1.433**	**35.16**	**17.91**	**39.46**
**Quartz**	**(-3,-1,-2)**	**5.732**	**76.35**	**22.8**	**24.5**	**1.433**	**35.16**	**17.91**	**39.46**
**Quartz**	**(-1,-3,2)**	**5.732**	**76.35**	**22.791**	**24.5**	**1.433**	**35.15**	**17.91**	**39.45**
**Quartz**	**(-1,4,-2)**	**5.732**	**76.35**	**22.79**	**24.5**	**1.433**	**35.14**	**17.91**	**39.45**
**Quartz**	**(0,3,-3)**	**5.563**	**70.57**	**12.955**	**14**	**2.081**	**29.09**	**26.02**	**39.03**
	Equiv. Refl.: (0,-3,3)								
**Quartz**	**(3,0,3)**	**5.563**	**70.57**	**12.954**	**14**	**2.081**	**29.09**	**26.02**	**39.02**
	Equiv. Refl.: (-3,0,-3)								
**Quartz**	**(3,-3,-3)**	**5.563**	**70.57**	**12.953**	**14**	**2.081**	**29.09**	**26.02**	**39.02**
	Equiv. Refl.: (-3,3,3)								
**Quartz**	**(0,-3,-3)**	**5.563**	**70.57**	**7.71**	**9.5**	**2.081**	**19.69**	**26.02**	**32.62**
**Quartz**	**(0,3,3)**	**5.563**	**70.57**	**7.709**	**9.5**	**2.081**	**19.69**	**26.02**	**32.62**
**Quartz**	**(3,0,-3)**	**5.563**	**70.57**	**7.692**	**9.4**	**2.081**	**19.65**	**26.02**	**32.61**
	Equiv. Refl.: (-3,0,3)								
**Quartz**	**(3,-3,3)**	**5.563**	**70.57**	**7.691**	**9.4**	**2.081**	**19.65**	**26.02**	**32.6**
	Equiv. Refl.: (-3,3,-3)								
**Quartz**	**(-2,4,2)**	**5.544**	**70.02**	**6.298**	**7.8**	**2.144**	**16.82**	**26.81**	**31.64**
	Equiv. Refl.: (-2,-2,-2)								
**Quartz**	**(-2,4,-2)**	**5.544**	**70.02**	**6.297**	**7.8**	**2.144**	**16.81**	**26.81**	**31.64**
**Quartz**	**(-2,-2,2)**	**5.544**	**70.02**	**6.295**	**7.8**	**2.144**	**16.81**	**26.81**	**31.64**
**Quartz**	**(4,-2,2)**	**5.544**	**70.02**	**6.292**	**7.8**	**2.144**	**16.8**	**26.81**	**31.64**
**Quartz**	**(4,-2,-2)**	**5.544**	**70.02**	**6.291**	**7.8**	**2.144**	**16.8**	**26.81**	**31.64**
**Quartz**	**(2,2,2)**	**5.544**	**70.02**	**5.699**	**7.8**	**2.144**	**16.82**	**26.81**	**31.64**
	Equiv. Refl.: (2,-4,2), (2,-4,-2)								
**Quartz**	**(2,2,-2)**	**5.544**	**70.02**	**5.697**	**7.8**	**2.144**	**16.81**	**26.81**	**31.64**
**Quartz**	**(-4,2,-2)**	**5.544**	**70.02**	**5.693**	**7.8**	**2.144**	**16.8**	**26.81**	**31.64**
**Quartz**	**(-4,2,2)**	**5.544**	**70.02**	**5.692**	**7.8**	**2.144**	**16.8**	**26.81**	**31.64**

Ei =	5.888	keV						ΔEg	ΔEt
Cryst	Refl	EB	ΘB	∫IR dΘ	Width	Ei cotΘ	ΔE	2m, 50μm	2m, 50μm
	(h,k,l)	[keV]	[°]	[μrad]	[μrad]	[meV/μrad]	[meV]	[meV]	[meV]
Ge	(3,3,3)	5.694	75.25	74.8937	76.68	1.5507	118.9	19.38	120.47
	Equiv. Refl.: (1,5,1), (1,1,5), (5,1,1)								
Si	(2,2,4)	5.592	71.75	47.5324	43.54	1.9413	84.52	24.27	87.93
	Equiv. Refl.: (2,4,2), (4,2,2)								
LiNbO3	(1,-2,12)	5.882	87.37	359.592	351.2	0.27	94.96	3.38	95.02
	Equiv. Refl.: (2,-1,12)								
LiNbO3	(1,1,12)	5.882	87.37	359.591	351.2	0.27	94.96	3.38	95.02
	Equiv. Refl.: (-1,2,12), (-1,-1,12), (-2,1,12)								
LiNbO3	(-1,2,-12)	5.882	87.37	292.011	351.2	0.27	94.96	3.38	95.02
	Equiv. Refl.: (-2,1,-12)								
LiNbO3	(1,1,-12)	5.882	87.37	292.01	351.2	0.27	94.96	3.38	95.02
	Equiv. Refl.: (1,-2,-12), (2,-1,-12), (-1,-1,-12)								
LiNbO3	(0,-4,4)	5.842	82.87	102.363	113	0.736	83.21	9.2	83.71
	Equiv. Refl.: (4,0,4)								
LiNbO3	(-4,4,4)	5.842	82.87	102.362	113	0.736	83.21	9.2	83.71
LiNbO3	(0,4,-4)	5.842	82.87	99.144	113	0.736	83.21	9.2	83.71
	Equiv. Refl.: (4,-4,-4), (-4,0,-4)								
LiNbO3	(1,2,-10)	5.791	79.56	91.096	95.1	1.085	103.09	13.56	103.98
	Equiv. Refl.: (2,-3,-10), (3,-2,-10), (-1,3,-10), (-2,-1,-10), (-3,1,-10)								
LiNbO3	(1,-3,10)	5.791	79.56	87.532	95.1	1.085	103.09	13.56	103.98
	Equiv. Refl.: (2,1,10), (3,-1,10), (-1,-2,10), (-2,3,10), (-3,2,10)								
LiNbO3	(0,-4,-2)	5.633	73.09	70.169	69.3	1.79	124.03	22.38	126.03
	Equiv. Refl.: (4,0,-2), (-4,4,-2)								
LiNbO3	(0,4,2)	5.633	73.09	64.23	69.3	1.79	124.03	22.38	126.03
	Equiv. Refl.: (4,-4,2), (-4,0,2)								
Sapph	(0,0,12)	5.727	76.59	62.214	56.6	1.404	79.49	17.55	81.4
	Equiv. Refl.: (0,0,-12)								
Quartz	(4,-1,-2)	5.732	76.79	44.035	39.4	1.382	54.4	17.27	57.08
	Equiv. Refl.: (4,-3,2)								
Quartz	(-3,4,-2)	5.732	76.79	44.031	39.4	1.382	54.4	17.27	57.08
	Equiv. Refl.: (-3,-1,2)								
Quartz	(-1,-3,-2)	5.732	76.79	44.027	39.4	1.382	54.4	17.27	57.07
Quartz	(-1,4,2)	5.732	76.79	44.024	39.4	1.382	54.39	17.27	57.07
Quartz	(-4,1,2)	5.732	76.79	41.759	39.4	1.382	54.4	17.27	57.08
	Equiv. Refl.: (-4,3,-2)								
Quartz	(3,1,-2)	5.732	76.79	41.757	39.4	1.382	54.4	17.27	57.08
	Equiv. Refl.: (3,-4,2)								
Quartz	(1,3,2)	5.732	76.79	41.752	39.4	1.382	54.4	17.27	57.07
Quartz	(1,-4,-2)	5.732	76.79	41.749	39.4	1.382	54.39	17.27	57.07
Sapph	(0,2,10)	5.642	73.38	38.174	35.9	1.757	63.11	21.96	66.83
	Equiv. Refl.: (0,-2,-10), (2,0,-10), (2,-2,10), (-2,0,10), (-2,2,-10)								
Sapph	(1,3,4)	5.75	77.59	34.836	35	1.296	45.35	16.19	48.16
	Equiv. Refl.: (1,-4,-4), (3,1,-4), (3,-4,4), (4,-1,-4), (4,-3,4), (-1,4,4), (-1,-3,-4), (-3,4,-4), (-3,-1,4) (-4,1,4), (-4,3,-4)								
Quartz	(0,4,0)	5.828	81.81	30.242	32	0.847	27.08	10.59	29.08
	Equiv. Refl.: (0,-4,0), (4,0,0), (4,-4,0), (-4,0,0), (-4,4,0)								
Quartz	(3,-4,-2)	5.732	76.79	27.133	25.4	1.382	35.1	17.27	39.11
	Equiv. Refl.: (-4,1,-2)								

Ei = Cryst	5.888 Refl (h,k,l)	keV EB [keV]	ΘB [°]	∫ IR dΘ [µrad]	Width [µrad]	Ei cotΘ [meV/µrad]	ΔE [meV]	ΔEg 2m, 50µm [meV]	ΔEt 2m, 50µm [meV]
**Quartz**	**(3,1,2)**	**5.732**	**76.79**	**27.132**	**25.4**	**1.382**	**35.09**	**17.27**	**39.11**
	Equiv. Refl.: (-4,3,2)								
**Quartz**	**(1,3,-2)**	**5.732**	**76.79**	**27.122**	**25.4**	**1.382**	**35.08**	**17.27**	**39.1**
**Quartz**	**(1,-4,2)**	**5.732**	**76.79**	**27.12**	**25.4**	**1.382**	**35.08**	**17.27**	**39.1**
**Quartz**	**(4,-1,2)**	**5.732**	**76.79**	**23.579**	**25.4**	**1.382**	**35.09**	**17.27**	**39.11**
	Equiv. Refl.: (-3,4,2)								
**Quartz**	**(4,-3,-2)**	**5.732**	**76.79**	**23.578**	**25.4**	**1.382**	**35.09**	**17.27**	**39.11**
	Equiv. Refl.: (-3,-1,-2)								
**Quartz**	**(-1,-3,2)**	**5.732**	**76.79**	**23.568**	**25.4**	**1.382**	**35.08**	**17.27**	**39.1**
**Quartz**	**(-1,4,-2)**	**5.732**	**76.79**	**23.567**	**25.4**	**1.382**	**35.08**	**17.27**	**39.1**
**Quartz**	**(0,3,-3)**	**5.563**	**70.87**	**13.165**	**14.2**	**2.042**	**29.04**	**25.53**	**38.66**
	Equiv. Refl.: (0,-3,3)								
**Quartz**	**(3,0,3)**	**5.563**	**70.87**	**13.164**	**14.2**	**2.042**	**29.04**	**25.53**	**38.66**
	Equiv. Refl.: (-3,0,-3)								
**Quartz**	**(3,-3,-3)**	**5.563**	**70.87**	**13.163**	**14.2**	**2.042**	**29.03**	**25.53**	**38.66**
	Equiv. Refl.: (-3,3,3)								
**Quartz**	**(0,-3,-3)**	**5.563**	**70.87**	**7.831**	**9.6**	**2.042**	**19.65**	**25.53**	**32.21**
**Quartz**	**(0,3,3)**	**5.563**	**70.87**	**7.83**	**9.6**	**2.042**	**19.65**	**25.53**	**32.21**
**Quartz**	**(3,0,-3)**	**5.563**	**70.87**	**7.813**	**9.6**	**2.042**	**19.62**	**25.53**	**32.19**
	Equiv. Refl.: (-3,0,3)								
**Quartz**	**(3,-3,3)**	**5.563**	**70.87**	**7.812**	**9.6**	**2.042**	**19.62**	**25.53**	**32.19**
	Equiv. Refl.: (-3,3,-3)								
**Quartz**	**(-2,4,2)**	**5.544**	**70.32**	**6.391**	**8**	**2.106**	**16.78**	**26.33**	**31.22**
	Equiv. Refl.: (-2,-2,-2)								
**Quartz**	**(-2,4,-2)**	**5.544**	**70.32**	**6.39**	**8**	**2.106**	**16.78**	**26.33**	**31.22**
**Quartz**	**(-2,-2,2)**	**5.544**	**70.32**	**6.388**	**8**	**2.106**	**16.78**	**26.33**	**31.22**
**Quartz**	**(4,-2,2)**	**5.544**	**70.32**	**6.385**	**8**	**2.106**	**16.77**	**26.33**	**31.21**
	Equiv. Refl.: (4,-2,-2)								
**Quartz**	**(2,2,2)**	**5.544**	**70.32**	**5.78**	**8**	**2.106**	**16.78**	**26.33**	**31.22**
	Equiv. Refl.: (2,-4,2), (2,-4,-2)								
**Quartz**	**(2,2,-2)**	**5.544**	**70.32**	**5.779**	**8**	**2.106**	**16.78**	**26.33**	**31.22**
**Quartz**	**(-4,2,2)**	**5.544**	**70.32**	**5.774**	**8**	**2.106**	**16.77**	**26.33**	**31.21**
	Equiv. Refl.: (-4,2,-2)								

Ei =	6.49	keV						ΔEg	ΔEt
**Cryst**	**Refl**	**EB**	**ΘB**	**∫ IR dΘ**	**Width**	**Ei cotΘ**	**ΔE**	**2m, 50µm**	**2m, 50µm**
	**(h,k,l)**	**[keV]**	**[°]**	**[µrad]**	**[µrad]**	**[meV/µrad]**	**[meV]**	**[meV]**	**[meV]**
**Ge**	**(1,3,5)**	**6.483**	**87.29**	**277.533**	**282.96**	**0.3076**	**87.05**	**3.85**	**87.14**
	Equiv. Refl.: (1,5,3), (3,1,5), (3,5,1), (5,1,3), (5,3,1)								
**Si**	**(0,4,4)**	**6.457**	**84.22**	**102.5381**	**93.25**	**0.6571**	**61.28**	**8.21**	**61.82**
	Equiv. Refl.: (4,0,4), (4,4,0)								
**Ge**	**(0,4,4)**	**6.199**	**72.77**	**75.7699**	**70.4**	**2.013**	**141.71**	**25.16**	**143.93**
	Equiv. Refl.: (4,0,4), (4,4,0)								
**LiNbO3**	**(0,-1,-14)**	**6.413**	**81.17**	**88.164**	**88.7**	**1.009**	**89.48**	**12.61**	**90.36**
	Equiv. Refl.: (1,0,-14), (-1,1,-14)								
**LiNbO3**	**(0,1,14)**	**6.413**	**81.17**	**83.902**	**88.7**	**1.009**	**89.48**	**12.61**	**90.36**
	Equiv. Refl.: (1,-1,14), (-1,0,14)								
**LiNbO3**	**(2,3,-4)**	**6.319**	**76.83**	**77.804**	**75.8**	**1.518**	**115.14**	**18.98**	**116.7**
	Equiv. Refl.: (3,-5,-4), (5,-3,-4), (-2,5,-4), (-3,-2,-4), (-5,2,-4)								
**LiNbO3**	**(2,-5,4)**	**6.319**	**76.83**	**76.553**	**75.8**	**1.518**	**115.14**	**18.98**	**116.7**
	Equiv. Refl.: (3,2,4), (5,-2,4), (-2,-3,4), (-3,5,4), (-5,3,4)								
**Quartz**	**(5,-2,-1)**	**6.454**	**83.93**	**60.523**	**54.5**	**0.69**	**37.61**	**8.63**	**38.58**
	Equiv. Refl.: (5,-3,1), (-2,-3,-1)								
**Quartz**	**(-2,5,1)**	**6.454**	**83.93**	**60.522**	**54.5**	**0.69**	**37.6**	**8.63**	**38.58**
	Equiv. Refl.: (-3,5,-1), (-3,-2,1)								
**LiNbO3**	**(1,-5,0)**	**6.372**	**79.06**	**59.501**	**63.1**	**1.255**	**79.15**	**15.68**	**80.69**
	Equiv. Refl.: (4,1,0), (5,-1,0), (-1,-4,0), (-4,5,0), (-5,4,0)								
**LiNbO3**	**(1,4,0)**	**6.372**	**79.06**	**58.787**	**63.1**	**1.255**	**79.15**	**15.68**	**80.69**
	Equiv. Refl.: (4,-5,0), (5,-4,0), (-1,5,0), (-4,-1,0), (-5,1,0)								
**Quartz**	**(3,2,-1)**	**6.454**	**83.93**	**57.875**	**54.5**	**0.69**	**37.6**	**8.63**	**38.58**
	Equiv. Refl.: (3,-5,1), (-5,2,1), (-5,3,-1)								
**Quartz**	**(2,3,1)**	**6.454**	**83.93**	**57.874**	**54.5**	**0.69**	**37.61**	**8.63**	**38.58**
**Quartz**	**(2,-5,-1)**	**6.454**	**83.93**	**57.873**	**54.5**	**0.69**	**37.6**	**8.63**	**38.58**
**LiNbO3**	**(1,-4,8)**	**6.159**	**71.62**	**43.637**	**42.5**	**2.156**	**91.65**	**26.95**	**95.53**
	Equiv. Refl.: (3,1,8), (4,-1,8), (-1,-3,8), (-3,4,8), (-4,3,8)								
**LiNbO3**	**(1,3,-8)**	**6.159**	**71.62**	**37.679**	**42.5**	**2.156**	**91.65**	**26.95**	**95.53**
	Equiv. Refl.: (3,-4,-8), (4,-3,-8), (-1,4,-8), (-3,-1,-8), (-4,1,-8)								
**Sapph**	**(1,2,-10)**	**6.215**	**73.25**	**32.842**	**29.9**	**1.953**	**58.3**	**24.41**	**63.2**
	Equiv. Refl.: (1,-3,10), (2,1,10), (2,-3,-10), (3,-1,10), (3,-2,-10), (-1,3,-10), (-1,-2,10), (-2,3,10), (-2,-1,-10) (-3,1,-10), (-3,2,10)								
**LiNbO3**	**(2,3,2)**	**6.127**	**70.74**	**29.016**	**31.2**	**2.268**	**70.75**	**28.35**	**76.22**
	Equiv. Refl.: (3,-5,2), (5,-3,2), (-2,5,2), (-3,-2,2), (-5,2,2)								
**Quartz**	**(2,3,0)**	**6.351**	**78.11**	**28.078**	**26.4**	**1.366**	**36.02**	**17.07**	**39.86**
	Equiv. Refl.: (2,-5,0), (3,2,0), (3,-5,0), (-5,2,0), (-5,3,0)								
**Quartz**	**(5,-2,0)**	**6.351**	**78.11**	**27.928**	**26.4**	**1.366**	**36.02**	**17.07**	**39.86**
	Equiv. Refl.: (5,-3,0), (-2,5,0), (-2,-3,0), (-3,5,0), (-3,-2,0)								
**LiNbO3**	**(2,-5,-2)**	**6.127**	**70.74**	**26.844**	**31.2**	**2.268**	**70.75**	**28.35**	**76.22**
	Equiv. Refl.: (3,2,-2), (5,-2,-2), (-2,-3,-2), (-3,5,-2), (-5,3,-2)								
**Quartz**	**(2,-1,-5)**	**6.266**	**74.89**	**21.253**	**19.1**	**1.752**	**33.54**	**21.9**	**40.06**
**Quartz**	**(2,-1,5)**	**6.266**	**74.89**	**21.252**	**19.1**	**1.752**	**33.54**	**21.9**	**40.05**
**Quartz**	**(-1,-1,-5)**	**6.266**	**74.89**	**21.245**	**19.1**	**1.752**	**33.53**	**21.9**	**40.05**
**Quartz**	**(-1,2,5)**	**6.266**	**74.89**	**21.24**	**19.1**	**1.752**	**33.52**	**21.9**	**40.04**
**Quartz**	**(-1,2,-5)**	**6.266**	**74.89**	**21.231**	**19.1**	**1.752**	**33.51**	**21.9**	**40.03**
	Equiv. Refl.: (-1,-1,5)								
**Quartz**	**(-2,1,5)**	**6.266**	**74.89**	**19.431**	**19.1**	**1.752**	**33.54**	**21.9**	**40.06**

Ei =	6.49	keV						ΔEg	ΔEt
Cryst	Refl	EB	ΘB	∫ IR dΘ	Width	Ei cotΘ	ΔE	2m, 50µm	2m, 50µm
	(h,k,l)	[keV]	[°]	[µrad]	[µrad]	[meV/µrad]	[meV]	[meV]	[meV]
**Quartz**	**(-2,1,-5)**	**6.266**	**74.89**	**19.43**	**19.1**	**1.752**	**33.54**	**21.9**	**40.05**
**Quartz**	**(1,1,5)**	**6.266**	**74.89**	**19.424**	**19.1**	**1.752**	**33.53**	**21.9**	**40.05**
**Quartz**	**(1,-2,-5)**	**6.266**	**74.89**	**19.419**	**19.1**	**1.752**	**33.52**	**21.9**	**40.04**
**Quartz**	**(1,1,-5)**	**6.266**	**74.89**	**19.413**	**19.1**	**1.752**	**33.51**	**21.9**	**40.03**
	Equiv. Refl.: (1,-2,5)								
**Quartz**	**(2,2,3)**	**6.109**	**70.26**	**17.299**	**15.5**	**2.329**	**36.08**	**29.11**	**46.36**
	Equiv. Refl.: (2,-4,-3)								
**Quartz**	**(-4,2,-3)**	**6.109**	**70.26**	**17.298**	**15.5**	**2.329**	**36.08**	**29.11**	**46.36**
**Quartz**	**(-4,2,3)**	**6.109**	**70.26**	**17.296**	**15.5**	**2.329**	**36.08**	**29.11**	**46.36**
**Quartz**	**(2,2,-3)**	**6.109**	**70.26**	**17.294**	**15.5**	**2.329**	**36.07**	**29.11**	**46.36**
	Equiv. Refl.: (2,-4,3)								
**Quartz**	**(4,-2,3)**	**6.109**	**70.26**	**16.298**	**15.5**	**2.329**	**36.08**	**29.11**	**46.36**
**Quartz**	**(4,-2,-3)**	**6.109**	**70.26**	**16.297**	**15.5**	**2.329**	**36.08**	**29.11**	**46.36**
	Equiv. Refl.: (-2,4,3)								
**Quartz**	**(-2,-2,-3)**	**6.109**	**70.26**	**16.296**	**15.5**	**2.329**	**36.08**	**29.11**	**46.36**
**Quartz**	**(-2,4,-3)**	**6.109**	**70.26**	**16.292**	**15.5**	**2.329**	**36.07**	**29.11**	**46.36**
	Equiv. Refl.: (-2,-2,3)								
**Sapph**	**(0,4,-4)**	**6.313**	**76.6**	**16.23**	**17.2**	**1.546**	**26.58**	**19.33**	**32.86**
	Equiv. Refl.: (0,-4,4), (4,0,4), (4,-4,-4), (-4,0,-4), (-4,4,4)								
**Quartz**	**(0,4,2)**	**6.263**	**74.81**	**12.416**	**12.9**	**1.762**	**22.81**	**22.03**	**31.71**
	Equiv. Refl.: (0,-4,-2), (4,0,-2)								
**Quartz**	**(-4,0,2)**	**6.263**	**74.81**	**12.415**	**12.9**	**1.762**	**22.81**	**22.03**	**31.71**
**Quartz**	**(4,-4,2)**	**6.263**	**74.81**	**12.414**	**12.9**	**1.762**	**22.81**	**22.03**	**31.71**
	Equiv. Refl.: (-4,4,-2)								
**LiNbO3**	**(2,2,9)**	**6.277**	**75.27**	**9.615**	**17.9**	**1.706**	**30.57**	**21.32**	**37.27**
	Equiv. Refl.: (2,2,-9), (2,-4,9), (2,-4,-9), (4,-2,9), (4,-2,-9), (-2,4,9), (-2,4,-9), (-2,-2,9), (-2,-2,-9) (-4,2,9), (-4,2,-9)								
**Sapph**	**(1,1,12)**	**6.292**	**75.82**	**8.949**	**11.1**	**1.639**	**18.2**	**20.49**	**27.41**
	Equiv. Refl.: (1,1,-12), (1,-2,12), (1,-2,-12), (2,-1,12), (2,-1,-12), (-1,2,12), (-1,2,-12), (-1,-1,12), (-1,-1,-12) (-2,1,12), (-2,1,-12)								
**Quartz**	**(0,3,-4)**	**6.337**	**77.53**	**8.82**	**10.4**	**1.435**	**14.99**	**17.94**	**23.38**
	Equiv. Refl.: (0,-3,4)								
**Quartz**	**(-3,0,-4)**	**6.337**	**77.53**	**8.802**	**10.4**	**1.435**	**14.97**	**17.94**	**23.37**
**Quartz**	**(3,0,4)**	**6.337**	**77.53**	**8.801**	**10.4**	**1.435**	**14.97**	**17.94**	**23.37**
**Quartz**	**(-3,3,4)**	**6.337**	**77.53**	**8.799**	**10.4**	**1.435**	**14.97**	**17.94**	**23.37**
**Quartz**	**(3,-3,-4)**	**6.337**	**77.53**	**8.798**	**10.4**	**1.435**	**14.97**	**17.94**	**23.37**
**Quartz**	**(-3,4,-3)**	**6.28**	**75.38**	**6.72**	**8.1**	**1.693**	**13.67**	**21.16**	**25.19**
	Equiv. Refl.: (-3,-1,3)								
**Quartz**	**(4,-1,-3)**	**6.28**	**75.38**	**6.719**	**8.1**	**1.693**	**13.66**	**21.16**	**25.19**
**Quartz**	**(4,-3,3)**	**6.28**	**75.38**	**6.717**	**8.1**	**1.693**	**13.66**	**21.16**	**25.19**
**Quartz**	**(-1,4,3)**	**6.28**	**75.38**	**6.716**	**8.1**	**1.693**	**13.66**	**21.16**	**25.19**
	Equiv. Refl.: (-1,-3,-3)								
**Quartz**	**(1,3,-3)**	**6.28**	**75.38**	**6.651**	**7.2**	**1.693**	**12.25**	**21.16**	**24.45**
**Quartz**	**(1,-4,3)**	**6.28**	**75.38**	**6.649**	**7.2**	**1.693**	**12.25**	**21.16**	**24.45**
**Quartz**	**(-4,1,-3)**	**6.28**	**75.38**	**6.641**	**7.2**	**1.693**	**12.24**	**21.16**	**24.45**
**Quartz**	**(-4,3,3)**	**6.28**	**75.38**	**6.64**	**7.2**	**1.693**	**12.24**	**21.16**	**24.44**
**Quartz**	**(3,-4,-3)**	**6.28**	**75.38**	**6.632**	**7.2**	**1.693**	**12.23**	**21.16**	**24.44**
**Quartz**	**(3,1,3)**	**6.28**	**75.38**	**6.631**	**7.2**	**1.693**	**12.23**	**21.16**	**24.44**
**Quartz**	**(1,-4,-3)**	**6.28**	**75.38**	**6.495**	**8.1**	**1.693**	**13.66**	**21.16**	**25.19**
	Equiv. Refl.: (3,1,-3), (3,-4,3)								

Ei =	6.49	keV						ΔEg	ΔEt
Cryst	Refl	EB	ΘB	∫ IR dΘ	Width	Ei cotΘ	ΔE	2m, 50μm	2m, 50μm
	(h,k,l)	[keV]	[°]	[μrad]	[μrad]	[meV/μrad]	[meV]	[meV]	[meV]
Quartz	(1,3,3)	6.28	75.38	6.494	8.1	1.693	13.66	21.16	25.19
	Equiv. Refl.: (-4,1,3)								
Quartz	(-4,3,-3)	6.28	75.38	6.492	8.1	1.693	13.66	21.16	25.19
Quartz	(0,2,-5)	6.433	82.4	6.453	9.9	0.866	8.56	10.83	13.8
Quartz	(0,-2,5)	6.433	82.4	6.452	9.9	0.866	8.56	10.83	13.8
Quartz	(-2,0,-5)	6.433	82.4	6.42	9.8	0.866	8.53	10.83	13.79
Quartz	(2,0,5)	6.433	82.4	6.419	9.8	0.866	8.53	10.83	13.79
Quartz	(-2,2,5)	6.433	82.4	6.417	9.8	0.866	8.53	10.83	13.78
Quartz	(2,-2,-5)	6.433	82.4	6.416	9.8	0.866	8.53	10.83	13.78
Quartz	(-2,0,5)	6.433	82.4	6.41	9.8	0.866	8.48	10.83	13.75
Quartz	(2,0,-5)	6.433	82.4	6.407	9.8	0.866	8.48	10.83	13.75
Quartz	(-2,2,-5)	6.433	82.4	6.406	9.8	0.866	8.47	10.83	13.75
Quartz	(2,-2,5)	6.433	82.4	6.403	9.8	0.866	8.47	10.83	13.75
Quartz	(0,2,5)	6.433	82.4	6.393	9.8	0.866	8.46	10.83	13.74
	Equiv. Refl.: (0,-2,-5)								
Quartz	(-1,-3,3)	6.28	75.38	4.789	7.2	1.693	12.25	21.16	24.45
Quartz	(-1,4,-3)	6.28	75.38	4.787	7.2	1.693	12.25	21.16	24.45
Quartz	(4,-1,3)	6.28	75.38	4.782	7.2	1.693	12.24	21.16	24.45
Quartz	(4,-3,-3)	6.28	75.38	4.781	7.2	1.693	12.24	21.16	24.44
Quartz	(-3,4,3)	6.28	75.38	4.774	7.2	1.693	12.23	21.16	24.44
Quartz	(-3,-1,-3)	6.28	75.38	4.772	7.2	1.693	12.23	21.16	24.44
Sapph	(1,3,7)	6.371	78.99	4.34	7.7	1.262	9.69	15.78	18.52
	Equiv. Refl.: (1,-4,-7), (3,1,-7), (3,-4,7), (4,-1,-7), (4,-3,7), (-1,4,7), (-1,-3,-7), (-3,4,-7), (-3,-1,7) (-4,1,7), (-4,3,-7)								
Quartz	(0,-3,-4)	6.337	77.53	3.274	5.5	1.435	7.87	17.94	19.59
	Equiv. Refl.: (-3,0,4), (-3,3,-4)								
Quartz	(0,3,4)	6.337	77.53	3.273	5.5	1.435	7.87	17.94	19.59
Quartz	(3,0,-4)	6.337	77.53	3.271	5.5	1.435	7.87	17.94	19.59
Quartz	(3,-3,4)	6.337	77.53	3.269	5.5	1.435	7.87	17.94	19.59
LiNbO3	(2,3,5)	6.46	84.51	2.942	14.9	0.624	9.31	7.79	12.14
	Equiv. Refl.: (2,-5,-5), (3,2,-5), (3,-5,5), (5,-2,-5), (5,-3,5), (-2,5,5), (-2,-3,-5), (-3,5,-5), (-3,-2,5) (-5,2,5), (-5,3,-5)								
LiNbO3	(1,2,11)	6.142	71.17	2.834	8.3	2.214	18.39	27.67	33.22
	Equiv. Refl.: (1,-3,-11), (2,1,-11), (2,-3,11), (3,-1,-11), (3,-2,11), (-1,3,11), (-1,-2,-11), (-2,3,-11), (-2,-1,11) (-3,1,11), (-3,2,-11)								
Quartz	(0,4,-2)	6.263	74.81	0.978	2.5	1.762	4.42	22.03	22.47
	Equiv. Refl.: (0,-4,2), (-4,4,2)								
Quartz	(4,0,2)	6.263	74.81	0.977	2.5	1.762	4.42	22.03	22.47
	Equiv. Refl.: (4,-4,-2), (-4,0,-2)								

Ei = Cryst	7.112 Refl (h,k,l)	keV EB [keV]	ΘB [°]	∫ IR dΘ [μrad]	Width [μrad]	Ei cotΘ [meV/μrad]	ΔE [meV]	ΔEg 2m, 50μm [meV]	ΔEt 2m, 50μm [meV]
**Ge**	**(0,2,6)**	**6.93**	**77.02**	**70.7**	**65.6**	**1.639**	**107.47**	**20.49**	**109.41**
**Si**	**(1,3,5)**	**6.753**	**71.71**	**18.8**	**17.6**	**2.35**	**41.26**	**29.38**	**50.65**
**LiNbO3**	**(2,-5,-8)**	**7.038**	**81.73**	**84.9**	**81.3**	**1.034**	**84.11**	**12.92**	**85.1**
	Equiv. Refl.: (3,2,-8), (5,-2,-8), (-2,-3,-8), (-3,5,-8), (-5,3,-8)								
**LiNbO3**	**(2,3,8)**	**7.038**	**81.73**	**79.4**	**81.3**	**1.034**	**84.11**	**12.92**	**85.1**
	Equiv. Refl.: (3,-5,8), (5,-3,8), (-2,5,8), (-3,-2,8), (-5,2,8)								
**LiNbO3**	**(1,-5,-6)**	**6.914**	**76.44**	**53.2**	**51.2**	**1.715**	**87.73**	**21.44**	**90.31**
	Equiv. Refl.: (4,1,-6), (5,-1,-6), (-1,-4,-6), (-4,5,-6), (-5,4,-6)								
**LiNbO3**	**(1,4,-6)**	**6.914**	**76.44**	**52.6**	**50.4**	**1.715**	**86.42**	**21.44**	**89.04**
	Equiv. Refl.: (4,-5,-6), (5,-4,-6), (-1,5,-6), (-4,-1,-6), (-5,1,-6)								
**LiNbO3**	**(1,4,6)**	**6.914**	**76.44**	**50.2**	**51.2**	**1.715**	**87.73**	**21.44**	**90.32**
	Equiv. Refl.: (4,-5,6), (5,-4,6), (-1,5,6), (-4,-1,6), (-5,1,6)								
**LiNbO3**	**(1,-5,6)**	**6.914**	**76.44**	**49**	**50.4**	**1.715**	**86.42**	**21.44**	**89.04**
	Equiv. Refl.: (4,1,6), (5,-1,6), (-1,-4,6), (-4,5,6), (-5,4,6)								
**LiNbO3**	**(0,-2,14)**	**6.85**	**74.41**	**46.1**	**44.2**	**1.984**	**87.79**	**24.8**	**91.23**
	Equiv. Refl.: (2,0,14), (-2,2,14)								
**LiNbO3**	**(0,-3,-12)**	**6.797**	**72.88**	**44.7**	**41**	**2.191**	**89.9**	**27.39**	**93.98**
	Equiv. Refl.: (3,0,-12), (-3,3,-12)								
**LiNbO3**	**(0,3,-12)**	**6.797**	**72.88**	**43.9**	**40.3**	**2.191**	**88.3**	**27.39**	**92.45**
	Equiv. Refl.: (3,-3,-12), (-3,0,-12)								
**LiNbO3**	**(0,2,-14)**	**6.85**	**74.41**	**42.9**	**44.2**	**1.984**	**87.79**	**24.8**	**91.23**
	Equiv. Refl.: (2,-2,-14), (-2,0,-14)								
**LiNbO3**	**(1,-4,-10)**	**6.718**	**70.84**	**41.2**	**39**	**2.471**	**96.33**	**30.89**	**101.16**
	Equiv. Refl.: (3,1,-10), (4,-1,-10), (-1,-3,-10), (-3,4,-10), (-4,3,-10)								
**LiNbO3**	**(0,3,12)**	**6.797**	**72.88**	**38.1**	**41**	**2.191**	**89.9**	**27.39**	**93.98**
	Equiv. Refl.: (1,3,10), (3,-3,12), (3,-4,10), (4,-3,10), (-1,4,10), (-3,0,12), (-3,-1,10), (-4,1,10)								
**LiNbO3**	**(0,-3,12)**	**6.797**	**72.88**	**37.1**	**40.3**	**2.191**	**88.3**	**27.39**	**92.45**
	Equiv. Refl.: (3,0,12), (-3,3,12)								
**LiNbO3**	**(0,-5,2)**	**7.01**	**80.27**	**33.3**	**36.3**	**1.219**	**44.23**	**15.24**	**46.78**
	Equiv. Refl.: (5,0,2), (-5,5,2)								
**Sapph**	**(2,3,-4)**	**6.83**	**73.81**	**31.7**	**27.8**	**2.065**	**57.43**	**25.81**	**62.97**
	Equiv. Refl.: (2,-5,4), (3,2,4), (3,-5,-4), (5,-2,4), (5,-3,-4), (-2,5,-4), (-2,-3,4), (-3,5,4), (-3,-2,-4) (-5,2,-4), (-5,3,4)								
**Sapph**	**(1,4,3)**	**7.041**	**81.91**	**30.6**	**29.8**	**1.011**	**30.1**	**12.63**	**32.64**
	Equiv. Refl.: (1,4,-3), (1,-5,3), (1,-5,-3), (4,1,3), (4,1,-3), (4,-5,3), (4,-5,-3), (5,-1,3), (5,-1,-3) (5,-4,3), (5,-4,-3), (-1,5,3), (-1,5,-3), (-1,-4,3), (-1,-4,-3), (-4,5,3), (-4,5,-3), (-4,-1,3) (-4,-1,-3), (-5,1,3), (-5,1,-3), (-5,4,3), (-5,4,-3)								
**LiNbO3**	**(0,5,-2)**	**7.01**	**80.27**	**29.2**	**36.3**	**1.219**	**44.23**	**15.24**	**46.78**
	Equiv. Refl.: (5,-5,-2), (-5,0,-2)								
**Sapph**	**(0,1,14)**	**6.849**	**74.38**	**23.1**	**21.2**	**1.988**	**42.09**	**24.85**	**48.88**
	Equiv. Refl.: (0,-1,-14), (1,0,-14), (1,-1,14), (-1,0,14), (-1,1,-14), (0,1,-6), (0,-1,6), (1,0,6), (1,-1,-6) (-1,0,-6), (-1,1,6)								
**Quartz**	**(0,4,-3)**	**6.768**	**72.11**	**19.7**	**17.5**	**2.296**	**40.23**	**28.7**	**49.42**
	Equiv. Refl.: (0,-4,3), (4,0,3), (4,-4,-3), (-4,0,-3), (-4,4,3)								
**Sapph**	**(1,4,0)**	**6.894**	**75.78**	**19.1**	**18.2**	**1.802**	**32.83**	**22.53**	**39.81**
	Equiv. Refl.: (1,-5,0), (4,1,0), (4,-5,0), (5,-1,0), (5,-4,0), (-1,5,0), (-1,-4,0), (-4,5,0), (-4,-1,0) (-5,1,0), (-5,4,0)								
**Quartz**	**(5,-1,2)**	**7.06**	**83.06**	**18.3**	**18.9**	**0.866**	**16.41**	**10.82**	**19.66**
	Equiv. Refl.: (5,-4,-2), (-1,5,-2), (-1,-4,2), (-4,5,2), (-4,-1,-2)								
**Quartz**	**(1,3,-4)**	**6.975**	**78.72**	**18.2**	**16.8**	**1.418**	**23.81**	**17.72**	**29.68**
	Equiv. Refl.: (1,-4,4), (3,1,4), (3,-4,-4), (-4,1,-4), (-4,3,4)								

Ei =	7.112	keV						ΔEg	ΔEt
Cryst	Refl	EB	ΘB	∫ IR dΘ	Width	Ei cotΘ	ΔE	2m, 50µm	2m, 50µm
	(h,k,l)	[keV]	[°]	[µrad]	[µrad]	[meV/µrad]	[meV]	[meV]	[meV]
**Quartz**	**(1,4,-1)**	**6.775**	**72.28**	**18**	**15.9**	**2.273**	**36.13**	**28.41**	**45.96**
	Equiv. Refl.: (1,-5,1), (3,-1,-5), (3,-2,5), (4,1,1), (4,-5,-1), (-1,3,5), (-1,-2,-5), (-2,3,-5), (-2,-1,5) (-5,1,-1), (-5,4,1)								
**Quartz**	**(1,4,-2)**	**7.06**	**83.06**	**17.7**	**19**	**0.866**	**16.42**	**10.82**	**19.67**
	Equiv. Refl.: (1,-5,2), (4,1,2), (4,-5,-2), (-5,1,-2), (-5,4,2)								
**Quartz**	**(1,2,5)**	**6.91**	**76.32**	**17.4**	**16.5**	**1.731**	**28.55**	**21.64**	**35.83**
	Equiv. Refl.: (1,-3,-5), (2,1,-5), (2,-3,5), (-3,1,5), (-3,2,-5)								
**Quartz**	**(5,-1,1)**	**6.775**	**72.28**	**17.3**	**15.9**	**2.273**	**36.13**	**28.41**	**45.96**
	Equiv. Refl.: (5,-4,-1), (-1,5,-1), (-1,-4,1), (-4,5,1), (-4,-1,-1)								
**Quartz**	**(4,-1,4)**	**6.975**	**78.72**	**17.2**	**16.8**	**1.418**	**23.81**	**17.72**	**29.68**
	Equiv. Refl.: (4,-3,-4), (-1,4,-4), (-1,-3,4), (-3,4,4), (-3,-1,-4)								
**Sapph**	**(2,2,9)**	**6.754**	**71.73**	**16.8**	**15.9**	**2.347**	**37.26**	**29.34**	**47.43**
	Equiv. Refl.: (2,2,-9), (2,-4,9), (2,-4,-9), (4,-2,9), (4,-2,-9), (-2,4,9), (-2,4,-9), (-2,-2,9), (-2,-2,-9) (-4,2,9), (-4,2,-9)								
**Quartz**	**(5,-1,-2)**	**7.06**	**83.06**	**16.5**	**16.4**	**0.866**	**14.16**	**10.82**	**17.82**
	Equiv. Refl.: (5,-4,2), (-1,5,2), (-1,-4,-2), (-4,5,-2), (-4,-1,2)								
**Quartz**	**(2,2,4)**	**6.821**	**73.55**	**14.4**	**13.2**	**2.1**	**27.68**	**26.25**	**38.15**
	Equiv. Refl.: (2,2,-4), (2,-4,4), (2,-4,-4), (-4,2,4), (-4,2,-4)								
**Quartz**	**(0,0,6)**	**6.882**	**75.39**	**14.3**	**13.6**	**1.854**	**25.18**	**23.17**	**34.22**
	Equiv. Refl.: (0,0,-6)								
**Quartz**	**(1,4,2)**	**7.06**	**83.06**	**14**	**16.4**	**0.866**	**14.17**	**10.82**	**17.83**
	Equiv. Refl.: (1,-5,-2), (4,1,-2), (4,-5,2), (-5,1,2), (-5,4,-2)								
**Quartz**	**(4,-2,4)**	**6.821**	**73.55**	**13.8**	**13.2**	**2.1**	**27.68**	**26.25**	**38.15**
	Equiv. Refl.: (4,-2,-4), (-2,4,4), (-2,4,-4), (-2,-2,4), (-2,-2,-4)								
**Quartz**	**(0,4,3)**	**6.768**	**72.11**	**12.2**	**11.3**	**2.296**	**26.03**	**28.7**	**38.75**
	Equiv. Refl.: (0,-4,-3), (4,0,-3), (4,-4,3), (-4,0,3), (-4,4,-3)								
**Quartz**	**(1,3,4)**	**6.975**	**78.72**	**9.3**	**9.6**	**1.418**	**13.65**	**17.72**	**22.37**
	Equiv. Refl.: (1,-4,-4), (3,1,-4), (3,-4,4), (-4,1,4), (-4,3,-4)								
**Quartz**	**(4,-1,-4)**	**6.975**	**78.72**	**8.3**	**9.6**	**1.418**	**13.64**	**17.72**	**22.37**
	Equiv. Refl.: (4,-3,4), (-1,4,4), (-1,-3,-4), (-3,4,-4), (-3,-1,4)								
**Sapph**	**(2,3,5)**	**6.978**	**78.88**	**8**	**9.8**	**1.399**	**13.77**	**17.48**	**22.25**
	Equiv. Refl.: (2,-5,-5), (3,2,-5), (3,-5,5), (5,-2,-5), (5,-3,5), (-2,5,5), (-2,-3,-5), (-3,5,-5), (-3,-2,5) (-5,2,5), (-5,3,-5)								
**Quartz**	**(3,-1,5)**	**6.91**	**76.32**	**7.3**	**7.4**	**1.731**	**12.73**	**21.64**	**25.11**
	Equiv. Refl.: (3,-2,-5), (-1,3,-5), (-2,3,5), (-2,-1,-5)								
**Quartz**	**(-1,-2,5)**	**6.91**	**76.32**	**7.2**	**7.4**	**1.731**	**12.72**	**21.64**	**25.1**
**LiNbO3**	**(1,3,-11)**	**7.024**	**80.96**	**6.9**	**15.7**	**1.132**	**17.81**	**14.15**	**22.74**
	Equiv. Refl.: (1,-4,11), (3,1,11), (3,-4,-11), (4,-1,11), (4,-3,-11), (-1,4,-11), (-1,-3,11), (-3,4,11), (-3,-1,-11) (-4,1,-11), (-4,3,11)								
**Quartz**	**(1,2,-5)**	**6.91**	**76.32**	**5.7**	**7.4**	**1.731**	**12.72**	**21.64**	**25.1**
	Equiv. Refl.: (1,-3,5), (2,1,5), (2,-3,-5), (-3,1,-5), (-3,2,5)								
**Quartz**	**(5,-1,-1)**	**6.775**	**72.28**	**5.4**	**6**	**2.273**	**13.65**	**28.41**	**31.52**
	Equiv. Refl.: (5,-4,1), (-1,5,1), (-1,-4,-1), (-4,5,-1), (-4,-1,1)								
**Quartz**	**(1,4,1)**	**6.775**	**72.28**	**5.3**	**6**	**2.273**	**13.65**	**28.41**	**31.52**
	Equiv. Refl.: (1,-5,-1), (4,1,-1), (4,-5,1), (-5,1,1), (-5,4,-1)								
**Quartz**	**(2,3,-2)**	**6.752**	**71.7**	**5.2**	**5.6**	**2.351**	**13.06**	**29.39**	**32.17**
	Equiv. Refl.: (2,-5,2), (3,2,2), (3,-5,-2), (-5,2,-2), (-5,3,2)								
**Quartz**	**(5,-2,2)**	**6.752**	**71.7**	**4.9**	**5.6**	**2.351**	**13.07**	**29.39**	**32.17**
	Equiv. Refl.: (5,-3,-2), (-2,5,-2), (-2,-3,2), (-3,5,2), (-3,-2,-2)								

Ei = Cryst	7.112 Refl (h,k,l)	keV EB [keV]	ΘB [°]	∫ IR dΘ [μrad]	Width [μrad]	Ei cotΘ [meV/μrad]	ΔE [meV]	ΔEg 2m, 50μm [meV]	ΔEt 2m, 50μm [meV]
**LiNbO3**	**(1,2,-13)**	**6.88**	**75.31**	**2.7**	**7.6**	**1.864**	**14.12**	**23.3**	**27.25**
	Equiv. Refl.: (1,-3,13), (2,1,13), (2,-3,-13), (3,-1,13), (3,-2,-13), (-1,3,-13), (-1,-2,13), (-2,3,13), (-2,-1,-13) (-3,1,-13), (-3,2,13)								
**Quartz**	**(0,1,6)**	**7.035**	**81.54**	**2.4**	**4.7**	**1.058**	**4.94**	**13.22**	**14.11**
	Equiv. Refl.: (0,-1,-6), (1,0,-6), (1,-1,6), (-1,0,6), (-1,1,-6)								
**Quartz**	**(2,3,2)**	**6.752**	**71.7**	**1.8**	**2.8**	**2.351**	**6.51**	**29.39**	**30.11**
	Equiv. Refl.: (2,-5,-2), (3,2,-2), (3,-5,2), (-5,2,2), (-5,3,-2)								
**Quartz**	**(5,-2,-2)**	**6.752**	**71.7**	**1.4**	**2.8**	**2.351**	**6.5**	**29.39**	**30.1**
	Equiv. Refl.: (5,-3,2), (-2,5,2), (-2,-3,-2), (-3,5,-2), (-3,-2,2)								
**LiNbO3**	**(2,3,-7)**	**6.822**	**73.57**	**0.9**	**4.1**	**2.097**	**8.58**	**26.22**	**27.58**
	Equiv. Refl.: (2,-5,7), (3,2,7), (3,-5,-7), (5,-2,7), (5,-3,-7), (-2,5,-7), (-2,-3,7), (-3,5,7), (-3,-2,-7) (-5,2,-7), (-5,3,7)								

Ei =<br>Cryst	6.404<br>Refl<br>(h,k,l)	keV<br>EB<br>[keV]	ΘB<br>[°]	∫ IR dΘ<br>[µrad]	Width<br>[µrad]	Ei cotΘ<br>[meV/µrad]	ΔE<br>[meV]	ΔEg<br>2m, 50µm<br>[meV]	ΔEt<br>2m, 50µm<br>[meV]
**Ge**	**(0,4,4)**	**6.199**	**75.45**	**90.4**	**84.3**	**1.662**	**140.04**	**20.77**	**141.57**
**LiNbO3**	**(1,-5,0)**	**6.372**	**84.27**	**114.1**	**121.6**	**0.642**	**78.14**	**8.03**	**78.55**
	Equiv. Refl.: (4,1,0), (5,-1,0), (-1,-4,0), (-4,5,0), (-5,4,0)								
**LiNbO3**	**(1,4,0)**	**6.372**	**84.27**	**112.7**	**121.6**	**0.642**	**78.14**	**8.03**	**78.55**
	Equiv. Refl.: (4,-5,0), (5,-4,0), (-1,5,0), (-4,-1,0), (-5,1,0)								
**LiNbO3**	**(2,3,-4)**	**6.319**	**80.68**	**110.3**	**108.1**	**1.051**	**113.67**	**13.14**	**114.42**
	Equiv. Refl.: (3,-5,-4), (5,-3,-4), (-2,5,-4), (-3,-2,-4), (-5,2,-4)								
**LiNbO3**	**(2,-5,4)**	**6.319**	**80.68**	**108.5**	**108.1**	**1.051**	**113.67**	**13.14**	**114.42**
	Equiv. Refl.: (3,2,4), (5,-2,4), (-2,-3,4), (-3,5,4), (-5,3,4)								
**LiNbO3**	**(1,-4,8)**	**6.159**	**74.1**	**50.7**	**49.6**	**1.824**	**90.47**	**22.8**	**93.3**
	Equiv. Refl.: (3,1,8), (4,-1,8), (-1,-3,8), (-3,4,8), (-4,3,8)								
**Quartz**	**(2,3,0)**	**6.351**	**82.61**	**45.4**	**42.8**	**0.83**	**35.56**	**10.38**	**37.05**
	Equiv. Refl.: (2,-5,0), (3,2,0), (3,-5,0), (-5,2,0), (-5,3,0)								
**Quartz**	**(5,-2,0)**	**6.351**	**82.61**	**45.1**	**42.8**	**0.83**	**35.56**	**10.38**	**37.05**
	Equiv. Refl.: (5,-3,0), (-2,5,0), (-2,-3,0), (-3,5,0), (-3,-2,0)								
**LiNbO3**	**(1,3,-8)**	**6.159**	**74.1**	**43.7**	**49.6**	**1.824**	**90.47**	**22.8**	**93.3**
	Equiv. Refl.: (3,-4,-8), (4,-3,-8), (-1,4,-8), (-3,-1,-8), (-4,1,-8)								
**Sapph**	**(1,2,-10)**	**6.215**	**76.04**	**39.6**	**36.2**	**1.592**	**57.56**	**19.9**	**60.91**
	Equiv. Refl.: (1,-3,10), (2,1,10), (2,-3,-10), (3,-1,10), (3,-2,-10), (-1,3,-10), (-1,-2,10), (-2,3,10), (-2,-1,-10) (-3,1,-10), (-3,2,10)								
**LiNbO3**	**(2,3,2)**	**6.127**	**73.08**	**33.2**	**35.8**	**1.949**	**69.84**	**24.36**	**73.97**
	Equiv. Refl.: (3,-5,2), (5,-3,2), (-2,5,2), (-3,-2,2), (-5,2,2)								
**LiNbO3**	**(2,-5,-2)**	**6.127**	**73.08**	**30.6**	**35.8**	**1.949**	**69.84**	**24.36**	**73.97**
	Equiv. Refl.: (3,2,-2), (5,-2,-2), (-2,-3,-2), (-3,5,-2), (-5,3,-2)								
**Quartz**	**(2,-1,5)**	**6.266**	**78.07**	**26.6**	**24.5**	**1.353**	**33.11**	**16.91**	**37.18**
	Equiv. Refl.: (2,-1,-5), (-1,2,5), (-1,2,-5), (-1,-1,5), (-1,-1,-5)								
**Quartz**	**(1,1,5)**	**6.266**	**78.07**	**24.3**	**24.5**	**1.353**	**33.1**	**16.91**	**37.17**
	Equiv. Refl.: (1,1,-5), (1,-2,5), (1,-2,-5), (-2,1,5), (-2,1,-5)								
**Sapph**	**(0,4,2)**	**6.093**	**72.07**	**22.9**	**22**	**2.072**	**45.65**	**25.9**	**52.49**
	Equiv. Refl.: (0,-4,-2), (4,0,-2), (4,-4,2), (-4,0,2), (-4,4,-2)								
**Sapph**	**(0,4,-4)**	**6.313**	**80.34**	**22.2**	**24.1**	**1.09**	**26.25**	**13.62**	**29.57**
	Equiv. Refl.: (0,-4,4), (4,0,4), (4,-4,-4), (-4,0,-4), (-4,4,4)								
**Quartz**	**(2,2,3)**	**6.109**	**72.53**	**19.1**	**17.7**	**2.016**	**35.63**	**25.2**	**43.64**
	Equiv. Refl.: (2,2,-3), (2,-4,3), (2,-4,-3), (-4,2,3), (-4,2,-3)								
**Quartz**	**(4,-2,3)**	**6.109**	**72.53**	**18**	**17.7**	**2.016**	**35.63**	**25.2**	**43.64**
	Equiv. Refl.: (4,-2,-3), (-2,4,3), (-2,4,-3), (-2,-2,3), (-2,-2,-3)								
**Quartz**	**(0,4,2)**	**6.263**	**77.96**	**15.7**	**16.5**	**1.366**	**22.52**	**17.07**	**28.26**
	Equiv. Refl.: (0,-4,-2), (4,0,-2), (4,-4,2), (-4,0,2), (-4,4,-2)								
**Quartz**	**(0,3,-4)**	**6.337**	**81.7**	**13**	**15.8**	**0.935**	**14.8**	**11.68**	**18.86**
	Equiv. Refl.: (0,-3,4), (3,0,4), (3,-3,-4), (-3,0,-4), (-3,3,4)								
**LiNbO3**	**(2,2,9)**	**6.277**	**78.56**	**12.3**	**23.3**	**1.296**	**30.18**	**16.19**	**34.25**
	Equiv. Refl.: (2,2,-9), (2,-4,9), (2,-4,-9), (4,-2,9), (4,-2,-9), (-2,4,9), (-2,4,-9), (-2,-2,9), (-2,-2,-9) (-4,2,9), (-4,2,-9)								
**Sapph**	**(1,1,12)**	**6.292**	**79.29**	**11.6**	**14.8**	**1.212**	**17.96**	**15.14**	**23.5**
	Equiv. Refl.: (1,1,-12), (1,-2,12), (1,-2,-12), (2,-1,12), (2,-1,-12), (-1,2,12), (-1,2,-12), (-1,-1,12), (-1,-1,-12) (-2,1,12), (-2,1,-12)								
**Quartz**	**(4,-1,-3)**	**6.28**	**78.7**	**8.7**	**10.5**	**1.279**	**13.49**	**15.99**	**20.92**
	Equiv. Refl.: (4,-3,3), (-1,4,3), (-1,-3,-3), (-3,4,-3), (-3,-1,3)								
**Quartz**	**(1,3,-3)**	**6.28**	**78.7**	**8.6**	**9.5**	**1.279**	**12.1**	**15.99**	**20.05**
	Equiv. Refl.: (1,-4,3), (3,1,3), (3,-4,-3), (-4,1,-3), (-4,3,3)								

**Ei =**	**6.404**	**keV**						**ΔEg**	**ΔEt**
**Cryst**	**Refl (h,k,l)**	**EB [keV]**	**ΘB [°]**	**∫ IR dΘ [μrad]**	**Width [μrad]**	**Ei cotΘ [meV/μrad]**	**ΔE [meV]**	**2m, 50μm [meV]**	**2m, 50μm [meV]**
**Quartz**	**(1,3,3)**	**6.28**	**78.7**	**8.4**	**10.5**	**1.279**	**13.49**	**15.99**	**20.92**
	Equiv. Refl.: (1,-4,-3), (3,1,-3), (3,-4,3), (-4,1,3), (-4,3,-3)								
**Sapph**	**(1,3,7)**	**6.371**	**84.15**	**8.3**	**14.6**	**0.656**	**9.57**	**8.2**	**12.6**
	Equiv. Refl.: (1,-4,-7), (3,1,-7), (3,-4,7), (4,-1,-7), (4,-3,7), (-1,4,7), (-1,-3,-7), (-3,4,-7), (-3,-1,7) (-4,1,7), (-4,3,-7)								
**Quartz**	**(4,-1,3)**	**6.28**	**78.7**	**6**	**9.4**	**1.279**	**12.09**	**15.99**	**20.04**
	Equiv. Refl.: (4,-3,-3), (-1,4,-3), (-1,-3,3), (-3,4,3), (-3,-1,-3)								
**Quartz**	**(0,3,4)**	**6.337**	**81.7**	**5.5**	**8.3**	**0.935**	**7.77**	**11.68**	**14.03**
	Equiv. Refl.: (0,-3,-4), (3,0,-4), (3,-3,4), (-3,0,4), (-3,3,-4)								
**LiNbO3**	**(1,2,11)**	**6.142**	**73.57**	**3.2**	**9.6**	**1.888**	**18.15**	**23.61**	**29.78**
	Equiv. Refl.: (1,-3,-11), (2,1,-11), (2,-3,11), (3,-1,-11), (3,-2,11), (-1,3,11), (-1,-2,-11), (-2,3,-11), (-2,-1,11) (-3,1,11), (-3,2,-11)								
**Quartz**	**(0,4,-2)**	**6.263**	**77.96**	**1.2**	**3.2**	**1.366**	**4.37**	**17.07**	**17.62**
	Equiv. Refl.: (0,-4,2), (4,0,2), (4,-4,-2), (-4,0,-2), (-4,4,2)								
**LiNbO3**	**(2,3,-1)**	**6.077**	**71.62**	**1.1**	**5.2**	**2.127**	**11.09**	**26.59**	**28.81**
	Equiv. Refl.: (2,-5,1), (3,2,1), (3,-5,-1), (5,-2,1), (5,-3,-1), (-2,5,-1), (-2,-3,1), (-3,5,1), (-3,-2,-1) (-5,2,-1), (-5,3,1)								

Ei =	6.391	keV						ΔEg	ΔEt
**Cryst**	**Refl**	**EB**	**ΘB**	**∫ IR dΘ**	**Width**	**Ei cotΘ**	**ΔE**	**2m, 50μm**	**2m, 50μm**
	**(h,k,l)**	**[keV]**	**[°]**	**[μrad]**	**[μrad]**	**[meV/μrad]**	**[meV]**	**[meV]**	**[meV]**
**Ge**	**(0,4,4)**	**6.199**	**75.91**	**93.4**	**87.1**	**1.604**	**139.79**	**20.05**	**141.22**
**LiNbO3**	**(1,-5,0)**	**6.372**	**85.58**	**148.1**	**158**	**0.494**	**77.98**	**6.17**	**78.23**
	Equiv. Refl.: (4,1,0), (5,-1,0), (-1,-4,0), (-4,5,0), (-5,4,0)								
**LiNbO3**	**(1,4,0)**	**6.372**	**85.58**	**146.3**	**158**	**0.494**	**77.98**	**6.17**	**78.23**
	Equiv. Refl.: (4,-5,0), (5,-4,0), (-1,5,0), (-4,-1,0), (-5,1,0)								
**LiNbO3**	**(2,3,-4)**	**6.319**	**81.42**	**119.9**	**117.6**	**0.965**	**113.44**	**12.06**	**114.08**
	Equiv. Refl.: (3,-5,-4), (5,-3,-4), (-2,5,-4), (-3,-2,-4), (-5,2,-4)								
**LiNbO3**	**(2,-5,4)**	**6.319**	**81.42**	**117.9**	**117.6**	**0.965**	**113.44**	**12.06**	**114.08**
	Equiv. Refl.: (3,2,4), (5,-2,4), (-2,-3,4), (-3,5,4), (-5,3,4)								
**Quartz**	**(2,3,0)**	**6.351**	**83.57**	**52.2**	**49.3**	**0.72**	**35.49**	**9**	**36.62**
	Equiv. Refl.: (2,-5,0), (3,2,0), (3,-5,0), (-5,2,0), (-5,3,0)								
**LiNbO3**	**(1,-4,8)**	**6.159**	**74.51**	**52.1**	**51**	**1.771**	**90.29**	**22.13**	**92.96**
	Equiv. Refl.: (3,1,8), (4,-1,8), (-1,-3,8), (-3,4,8), (-4,3,8)								
**Quartz**	**(5,-2,0)**	**6.351**	**83.57**	**51.9**	**49.3**	**0.72**	**35.49**	**9**	**36.62**
	Equiv. Refl.: (5,-3,0), (-2,5,0), (-2,-3,0), (-3,5,0), (-3,-2,0)								
**LiNbO3**	**(1,3,-8)**	**6.159**	**74.51**	**44.9**	**51**	**1.771**	**90.29**	**22.13**	**92.96**
	Equiv. Refl.: (3,-4,-8), (4,-3,-8), (-1,4,-8), (-3,-1,-8), (-4,1,-8)								
**Sapph**	**(1,2,-10)**	**6.215**	**76.51**	**41.1**	**37.5**	**1.533**	**57.45**	**19.16**	**60.56**
	Equiv. Refl.: (1,-3,10), (2,1,10), (2,-3,-10), (3,-1,10), (3,-2,-10), (-1,3,-10), (-1,-2,10), (-2,3,10), (-2,-1,-10) (-3,1,-10), (-3,2,10)								
**LiNbO3**	**(2,3,2)**	**6.127**	**73.46**	**34**	**36.7**	**1.898**	**69.71**	**23.72**	**73.63**
	Equiv. Refl.: (3,-5,2), (5,-3,2), (-2,5,2), (-3,-2,2), (-5,2,2)								
**LiNbO3**	**(2,-5,-2)**	**6.127**	**73.46**	**31.4**	**36.7**	**1.898**	**69.71**	**23.72**	**73.63**
	Equiv. Refl.: (3,2,-2), (5,-2,-2), (-2,-3,-2), (-3,5,-2), (-5,3,-2)								
**Quartz**	**(2,-1,5)**	**6.266**	**78.64**	**28**	**25.7**	**1.284**	**33.05**	**16.05**	**36.74**
	Equiv. Refl.: (2,-1,-5), (-1,2,5), (-1,2,-5), (-1,-1,5), (-1,-1,-5)								
**Quartz**	**(1,1,5)**	**6.266**	**78.64**	**25.5**	**25.7**	**1.284**	**33.04**	**16.05**	**36.73**
	Equiv. Refl.: (1,1,-5), (1,-2,5), (1,-2,-5), (-2,1,5), (-2,1,-5)								
**Sapph**	**(0,4,-4)**	**6.313**	**81.05**	**24**	**26**	**1.006**	**26.2**	**12.57**	**29.06**
	Equiv. Refl.: (0,-4,4), (4,0,4), (4,-4,-4), (-4,0,-4), (-4,4,4)								
**Sapph**	**(0,4,2)**	**6.093**	**72.43**	**23.4**	**22.5**	**2.023**	**45.56**	**25.29**	**52.11**
	Equiv. Refl.: (0,-4,-2), (4,0,-2), (4,-4,2), (-4,0,2), (-4,4,-2)								
**Quartz**	**(2,2,3)**	**6.109**	**72.9**	**19.6**	**18.1**	**1.966**	**35.56**	**24.57**	**43.22**
	Equiv. Refl.: (2,2,-3), (2,-4,3), (2,-4,-3), (-4,2,3), (-4,2,-3)								
**Quartz**	**(4,-2,3)**	**6.109**	**72.9**	**18.4**	**18.1**	**1.966**	**35.56**	**24.57**	**43.22**
	Equiv. Refl.: (4,-2,-3), (-2,4,3), (-2,4,-3), (-2,-2,3), (-2,-2,-3)								
**Quartz**	**(0,4,2)**	**6.263**	**78.52**	**16.5**	**17.3**	**1.298**	**22.48**	**16.22**	**27.72**
	Equiv. Refl.: (0,-4,-2), (4,0,-2), (4,-4,2), (-4,0,2), (-4,4,-2)								
**Quartz**	**(0,3,-4)**	**6.337**	**82.54**	**14.5**	**17.6**	**0.837**	**14.78**	**10.47**	**18.11**
	Equiv. Refl.: (0,-3,4), (3,0,4), (3,-3,-4), (-3,0,-4), (-3,3,4)								
**LiNbO3**	**(2,2,9)**	**6.277**	**79.15**	**13**	**24.6**	**1.224**	**30.12**	**15.31**	**33.79**
	Equiv. Refl.: (2,2,-9), (2,-4,9), (2,-4,-9), (4,-2,9), (4,-2,-9), (-2,4,9), (-2,4,-9), (-2,-2,9), (-2,-2,-9) (-4,2,9), (-4,2,-9)								
**Sapph**	**(1,1,12)**	**6.292**	**79.92**	**12.3**	**15.8**	**1.136**	**17.93**	**14.2**	**22.87**
	Equiv. Refl.: (1,1,-12), (1,-2,12), (1,-2,-12), (2,-1,12), (2,-1,-12), (-1,2,12), (-1,2,-12), (-1,-1,12), (-1,-1,-12) (-2,1,12), (-2,1,-12)								
**Sapph**	**(1,3,7)**	**6.371**	**85.43**	**10.6**	**18.7**	**0.511**	**9.55**	**6.39**	**11.49**
	Equiv. Refl.: (1,-4,-7), (3,1,-7), (3,-4,7), (4,-1,-7), (4,-3,7), (-1,4,7), (-1,-3,-7), (-3,4,-7), (-3,-1,7) (-4,1,7), (-4,3,-7)								

**Ei =**	**6.391**	**keV**						**ΔEg**	**ΔEt**
**Cryst**	**Refl**	**EB**	**ΘB**	**∫ IR dΘ**	**Width**	**Ei cotΘ**	**ΔE**	**2m, 50µm**	**2m, 50µm**
	**(h,k,l)**	**[keV]**	**[°]**	**[µrad]**	**[µrad]**	**[meV/µrad]**	**[meV]**	**[meV]**	**[meV]**
**Quartz**	**(4,-1,-3)**	**6.28**	**79.3**	**9.2**	**11.2**	**1.207**	**13.47**	**15.09**	**20.23**
	Equiv. Refl.: (4,-3,3), (-1,4,3), (-1,-3,-3), (-3,4,-3), (-3,-1,3)								
**Quartz**	**(1,3,-3)**	**6.28**	**79.3**	**9.1**	**10**	**1.207**	**12.07**	**15.09**	**19.33**
	Equiv. Refl.: (1,-4,3), (3,1,3), (3,-4,-3), (-4,1,-3), (-4,3,3)								
**Quartz**	**(1,3,3)**	**6.28**	**79.3**	**8.9**	**11.2**	**1.207**	**13.47**	**15.09**	**20.23**
	Equiv. Refl.: (1,-4,-3), (3,1,-3), (3,-4,3), (-4,1,3), (-4,3,-3)								
**Quartz**	**(4,-1,3)**	**6.28**	**79.3**	**6.3**	**10**	**1.207**	**12.06**	**15.09**	**19.32**
	Equiv. Refl.: (4,-3,-3), (-1,4,-3), (-1,-3,3), (-3,4,3), (-3,-1,-3)								
**Quartz**	**(0,3,4)**	**6.337**	**82.54**	**6.2**	**9.3**	**0.837**	**7.76**	**10.47**	**13.03**
	Equiv. Refl.: (0,-3,-4), (3,0,-4), (-3,0,4), (-3,3,-4)								
**Quartz**	**(3,-3,4)**	**6.337**	**82.54**	**6.1**	**9.3**	**0.837**	**7.76**	**10.47**	**13.03**
**LiNbO3**	**(1,2,11)**	**6.142**	**73.97**	**3.3**	**9.9**	**1.836**	**18.12**	**22.95**	**29.24**
	Equiv. Refl.: (1,-3,-11), (2,1,-11), (2,-3,11), (3,-1,-11), (3,-2,11), (-1,3,11), (-1,-2,-11), (-2,3,-11), (-2,-1,11) (-3,1,11), (-3,2,-11)								
**Quartz**	**(0,4,-2)**	**6.263**	**78.52**	**1.3**	**3.4**	**1.298**	**4.36**	**16.22**	**16.8**
	Equiv. Refl.: (0,-4,2), (4,0,2), (4,-4,-2), (-4,0,-2), (-4,4,2)								
**LiNbO3**	**(2,3,-1)**	**6.077**	**71.98**	**1.1**	**5.3**	**2.079**	**11.07**	**25.99**	**28.25**
	Equiv. Refl.: (2,-5,1), (3,2,1), (3,-5,-1), (5,-2,1), (5,-3,-1), (-2,5,-1), (-2,-3,1), (-3,5,1), (-3,-2,-1) (-5,2,-1), (-5,3,1)								

Ei =	7.058	keV						ΔEg	ΔEt
Cryst	Refl	EB	ΘB	∫ IR dΘ	Width	Ei cotΘ	ΔE	2m, 50μm	2m, 50μm
	(h,k,l)	[keV]	[°]	[μrad]	[μrad]	[meV/μrad]	[meV]	[meV]	[meV]
**Ge**	**(0,2,6)**	**6.93**	**79.09**	**84.3788**	**78.44**	**1.361**	**106.77**	**17.01**	**108.11**
	Equiv. Refl.: (0,6,2), (2,0,6), (2,6,0), (6,0,2), (6,2,0)								
**Si**	**(1,3,5)**	**6.753**	**73.09**	**19.9679**	**19.09**	**2.1456**	**40.97**	**26.82**	**48.96**
	Equiv. Refl.: (1,5,3), (3,1,5), (3,5,1), (5,1,3), (5,3,1)								
**LiNbO3**	**(3,2,-8)**	**7.038**	**85.69**	**163.507**	**156.8**	**0.532**	**83.5**	**6.65**	**83.77**
	Equiv. Refl.: (-2,-3,-8), (-3,5,-8), (-5,3,-8)								
**LiNbO3**	**(2,-5,-8)**	**7.038**	**85.69**	**163.506**	**156.8**	**0.532**	**83.5**	**6.65**	**83.77**
	Equiv. Refl.: (5,-2,-8)								
**LiNbO3**	**(2,3,8)**	**7.038**	**85.69**	**152.54**	**156.8**	**0.532**	**83.5**	**6.65**	**83.77**
	Equiv. Refl.: (3,-5,8), (5,-3,8), (-3,-2,8)								
**LiNbO3**	**(-2,5,8)**	**7.038**	**85.69**	**152.539**	**156.8**	**0.532**	**83.5**	**6.65**	**83.77**
	Equiv. Refl.: (-5,2,8)								
**LiNbO3**	**(1,-5,-6)**	**6.914**	**78.4**	**62.498**	**60.1**	**1.449**	**87.1**	**18.11**	**88.96**
	Equiv. Refl.: (4,1,-6), (5,-1,-6), (-1,-4,-6), (-4,5,-6), (-5,4,-6)								
**Sapph**	**(1,4,3)**	**7.041**	**86.05**	**62.426**	**61.3**	**0.487**	**29.88**	**6.09**	**30.49**
	Equiv. Refl.: (1,4,-3), (1,-5,3), (1,-5,-3), (4,1,3), (4,1,-3), (4,-5,3), (4,-5,-3), (5,-1,3), (5,-1,-3) (5,-4,3), (5,-4,-3), (-1,5,3), (-1,5,-3), (-1,-4,3), (-1,-4,-3), (-4,5,3), (-4,5,-3), (-4,-1,3) (-4,-1,-3), (-5,1,3), (-5,1,-3), (-5,4,3), (-5,4,-3)								
**LiNbO3**	**(1,4,-6)**	**6.914**	**78.4**	**61.738**	**59.2**	**1.449**	**85.8**	**18.11**	**87.69**
	Equiv. Refl.: (4,-5,-6), (5,-4,-6), (-1,5,-6), (-4,-1,-6), (-5,1,-6)								
**LiNbO3**	**(1,4,6)**	**6.914**	**78.4**	**58.871**	**60.1**	**1.449**	**87.1**	**18.11**	**88.96**
	Equiv. Refl.: (4,-5,6), (5,-4,6), (-1,5,6), (-4,-1,6), (-5,1,6)								
**LiNbO3**	**(1,-5,6)**	**6.914**	**78.4**	**57.371**	**59.2**	**1.449**	**85.8**	**18.11**	**87.69**
	Equiv. Refl.: (4,1,6), (5,-1,6), (-1,-4,6), (-4,5,6), (-5,4,6)								
**LiNbO3**	**(0,-2,14)**	**6.85**	**76.07**	**51.796**	**49.8**	**1.751**	**87.16**	**21.89**	**89.86**
	Equiv. Refl.: (2,0,14), (-2,2,14)								
**LiNbO3**	**(0,-3,-12)**	**6.797**	**74.36**	**49.087**	**45.2**	**1.975**	**89.25**	**24.69**	**92.6**
	Equiv. Refl.: (3,0,-12), (-3,3,-12)								
**LiNbO3**	**(0,-5,2)**	**7.01**	**83.3**	**48.373**	**52.9**	**0.83**	**43.92**	**10.37**	**45.13**
	Equiv. Refl.: (5,0,2), (-5,5,2)								
**LiNbO3**	**(0,3,-12)**	**6.797**	**74.36**	**48.201**	**44.4**	**1.975**	**87.66**	**24.69**	**91.07**
	Equiv. Refl.: (3,-3,-12), (-3,0,-12)								
**LiNbO3**	**(0,2,-14)**	**6.85**	**76.07**	**48.159**	**49.8**	**1.751**	**87.16**	**21.89**	**89.86**
	Equiv. Refl.: (2,-2,-14), (-2,0,-14)								
**LiNbO3**	**(1,-4,-10)**	**6.718**	**72.14**	**44.388**	**42.1**	**2.274**	**95.63**	**28.42**	**99.76**
	Equiv. Refl.: (3,1,-10), (4,-1,-10), (-1,-3,-10), (-3,4,-10), (-4,3,-10)								
**LiNbO3**	**(0,5,-2)**	**7.01**	**83.3**	**42.365**	**52.9**	**0.83**	**43.92**	**10.37**	**45.13**
	Equiv. Refl.: (5,-5,-2), (-5,0,-2)								
**LiNbO3**	**(0,3,12)**	**6.797**	**74.36**	**41.778**	**45.2**	**1.975**	**89.25**	**24.69**	**92.6**
	Equiv. Refl.: (3,-3,12), (-3,0,12)								
**Quartz**	**(1,0,6)**	**7.035**	**85.34**	**41.619**	**40.5**	**0.576**	**23.28**	**7.19**	**24.37**
**Quartz**	**(1,-1,-6)**	**7.035**	**85.34**	**41.618**	**40.5**	**0.576**	**23.28**	**7.19**	**24.37**
	Equiv. Refl.: (-1,0,-6)								
**Quartz**	**(-1,1,6)**	**7.035**	**85.34**	**41.616**	**40.5**	**0.576**	**23.28**	**7.19**	**24.37**
**Quartz**	**(0,-1,6)**	**7.035**	**85.34**	**41.567**	**40.4**	**0.576**	**23.26**	**7.19**	**24.35**
**Quartz**	**(0,1,-6)**	**7.035**	**85.34**	**41.566**	**40.4**	**0.576**	**23.26**	**7.19**	**24.35**
**LiNbO3**	**(1,3,10)**	**6.718**	**72.14**	**40.991**	**42.1**	**2.274**	**95.63**	**28.42**	**99.76**
	Equiv. Refl.: (3,-4,10), (4,-3,10), (-1,4,10), (-3,-1,10), (-4,1,10)								

Ei =	7.058	keV						ΔEg	ΔEt
Cryst	Refl	EB	ΘB	∫ IR dΘ	Width	Ei cotΘ	ΔE	2m, 50μm	2m, 50μm
	(h,k,l)	[keV]	[°]	[μrad]	[μrad]	[meV/μrad]	[meV]	[meV]	[meV]
**LiNbO3**	**(0,-3,12)**	**6.797**	**74.36**	**40.714**	**44.4**	**1.975**	**87.66**	**24.69**	**91.07**
	Equiv. Refl.: (3,0,12), (-3,3,12)								
**Sapph**	**(2,3,-4)**	**6.83**	**75.39**	**35.283**	**31**	**1.839**	**57.02**	**22.99**	**61.48**
	Equiv. Refl.: (2,-5,4), (3,2,4), (3,-5,-4), (5,-2,4), (5,-3,-4), (-2,5,-4), (-2,-3,4), (-3,5,4), (-3,-2,-4) (-5,2,-4), (-5,3,4)								
**Sapph**	**(0,1,14)**	**6.849**	**76.03**	**25.892**	**23.8**	**1.755**	**41.79**	**21.94**	**47.2**
	Equiv. Refl.: (0,-1,-14), (1,0,-14), (1,-1,14), (-1,0,14), (-1,1,-14)								
**Quartz**	**(1,-4,4)**	**6.975**	**81.19**	**23.405**	**21.6**	**1.094**	**23.64**	**13.67**	**27.31**
**Quartz**	**(1,3,-4)**	**6.975**	**81.19**	**23.403**	**21.6**	**1.094**	**23.64**	**13.67**	**27.31**
**Quartz**	**(-4,1,-4)**	**6.975**	**81.19**	**23.402**	**21.6**	**1.094**	**23.64**	**13.67**	**27.31**
**Quartz**	**(3,-4,-4)**	**6.975**	**81.19**	**23.4**	**21.6**	**1.094**	**23.63**	**13.67**	**27.3**
**Quartz**	**(-4,3,4)**	**6.975**	**81.19**	**23.399**	**21.6**	**1.094**	**23.63**	**13.67**	**27.3**
**Quartz**	**(3,1,4)**	**6.975**	**81.19**	**23.397**	**21.6**	**1.094**	**23.63**	**13.67**	**27.3**
**Quartz**	**(4,-1,4)**	**6.975**	**81.19**	**22.008**	**21.6**	**1.094**	**23.64**	**13.67**	**27.31**
**Quartz**	**(-1,4,-4)**	**6.975**	**81.19**	**22.007**	**21.6**	**1.094**	**23.64**	**13.67**	**27.31**
**Quartz**	**(-1,-3,4)**	**6.975**	**81.19**	**22.006**	**21.6**	**1.094**	**23.64**	**13.67**	**27.31**
**Quartz**	**(4,-3,-4)**	**6.975**	**81.19**	**22.005**	**21.6**	**1.094**	**23.63**	**13.67**	**27.3**
**Quartz**	**(-3,4,4)**	**6.975**	**81.19**	**22.001**	**21.6**	**1.094**	**23.63**	**13.67**	**27.3**
**Quartz**	**(-3,-1,-4)**	**6.975**	**81.19**	**21.999**	**21.6**	**1.094**	**23.63**	**13.67**	**27.3**
**Sapph**	**(1,4,0)**	**6.894**	**77.63**	**21.972**	**21.1**	**1.548**	**32.6**	**19.35**	**37.91**
	Equiv. Refl.: (1,-5,0), (4,1,0), (4,-5,0), (5,-1,0), (5,-4,0), (-1,5,0), (-1,-4,0), (-4,5,0), (-4,-1,0) (-5,1,0), (-5,4,0)								
**Quartz**	**(0,4,-3)**	**6.768**	**73.52**	**21.502**	**19.1**	**2.088**	**39.94**	**26.1**	**47.71**
	Equiv. Refl.: (0,-4,3)								
**Quartz**	**(-4,0,-3)**	**6.768**	**73.52**	**21.495**	**19.1**	**2.088**	**39.93**	**26.1**	**47.7**
**Quartz**	**(4,0,3)**	**6.768**	**73.52**	**21.494**	**19.1**	**2.088**	**39.93**	**26.1**	**47.7**
	Equiv. Refl.: (-4,4,3)								
**Quartz**	**(4,-4,-3)**	**6.768**	**73.52**	**21.493**	**19.1**	**2.088**	**39.93**	**26.1**	**47.7**
**Quartz**	**(-1,3,5)**	**6.91**	**78.25**	**21.007**	**19.3**	**1.468**	**28.35**	**18.34**	**33.76**
**Quartz**	**(-1,-2,-5)**	**6.91**	**78.25**	**21.005**	**19.3**	**1.468**	**28.34**	**18.34**	**33.76**
**Quartz**	**(-2,3,-5)**	**6.91**	**78.25**	**20.995**	**19.3**	**1.468**	**28.34**	**18.34**	**33.76**
**Quartz**	**(3,-1,-5)**	**6.91**	**78.25**	**20.993**	**19.3**	**1.468**	**28.33**	**18.34**	**33.75**
	Equiv. Refl.: (-2,-1,5)								
**Quartz**	**(3,-2,5)**	**6.91**	**78.25**	**20.991**	**19.3**	**1.468**	**28.33**	**18.34**	**33.75**
**Quartz**	**(1,-3,-5)**	**6.91**	**78.25**	**20.367**	**19.3**	**1.468**	**28.35**	**18.34**	**33.76**
**Quartz**	**(1,2,5)**	**6.91**	**78.25**	**20.365**	**19.3**	**1.468**	**28.34**	**18.34**	**33.76**
**Quartz**	**(2,-3,5)**	**6.91**	**78.25**	**20.361**	**19.3**	**1.468**	**28.34**	**18.34**	**33.76**
**Quartz**	**(2,1,-5)**	**6.91**	**78.25**	**20.359**	**19.3**	**1.468**	**28.33**	**18.34**	**33.75**
**Quartz**	**(-3,1,5)**	**6.91**	**78.25**	**20.358**	**19.3**	**1.468**	**28.33**	**18.34**	**33.75**
**Quartz**	**(-3,2,-5)**	**6.91**	**78.25**	**20.356**	**19.3**	**1.468**	**28.33**	**18.34**	**33.75**
**Quartz**	**(4,1,1)**	**6.775**	**73.71**	**19.629**	**17.4**	**2.063**	**35.87**	**25.79**	**44.18**
	Equiv. Refl.: (4,-5,-1)								
**Quartz**	**(1,4,-1)**	**6.775**	**73.71**	**19.627**	**17.4**	**2.063**	**35.87**	**25.79**	**44.18**
**Quartz**	**(1,-5,1)**	**6.775**	**73.71**	**19.626**	**17.4**	**2.063**	**35.87**	**25.79**	**44.17**
**Quartz**	**(-5,1,-1)**	**6.775**	**73.71**	**19.625**	**17.4**	**2.063**	**35.87**	**25.79**	**44.17**
	Equiv. Refl.: (-5,4,1)								
**Quartz**	**(-4,5,1)**	**6.775**	**73.71**	**18.839**	**17.4**	**2.063**	**35.87**	**25.79**	**44.18**
	Equiv. Refl.: (-4,-1,-1)								
**Quartz**	**(-1,5,-1)**	**6.775**	**73.71**	**18.837**	**17.4**	**2.063**	**35.87**	**25.79**	**44.17**
	Equiv. Refl.: (-1,-4,1)								

Ei = Cryst	7.058 Refl (h,k,l)	keV EB [keV]	ΘB [°]	∫ IR dΘ [μrad]	Width [μrad]	Ei cotΘ [meV/μrad]	ΔE [meV]	ΔEg 2m, 50μm [meV]	ΔEt 2m, 50μm [meV]
**Quartz**	**(5,-1,1)**	**6.775**	**73.71**	**18.835**	**17.4**	**2.063**	**35.87**	**25.79**	**44.17**
	Equiv. Refl.: (5,-4,-1)								
**Sapph**	**(2,2,9)**	**6.754**	**73.11**	**18.238**	**17.3**	**2.143**	**36.98**	**26.78**	**45.66**
	Equiv. Refl.: (2,2,-9), (2,-4,9), (2,-4,-9), (4,-2,9), (4,-2,-9), (-2,4,9), (-2,4,-9), (-2,-2,9), (-2,-2,-9) (-4,2,9), (-4,2,-9)								
**Quartz**	**(0,0,6)**	**6.882**	**77.18**	**16.336**	**15.6**	**1.606**	**25**	**20.07**	**32.06**
	Equiv. Refl.: (0,0,-6)								
**Quartz**	**(2,-4,-4)**	**6.821**	**75.11**	**15.898**	**14.6**	**1.877**	**27.48**	**23.47**	**36.14**
**Quartz**	**(-4,2,4)**	**6.821**	**75.11**	**15.897**	**14.6**	**1.877**	**27.48**	**23.47**	**36.14**
**Quartz**	**(2,2,4)**	**6.821**	**75.11**	**15.895**	**14.6**	**1.877**	**27.48**	**23.47**	**36.13**
	Equiv. Refl.: (2,2,-4), (2,-4,4), (-4,2,-4)								
**Quartz**	**(-2,4,4)**	**6.821**	**75.11**	**15.249**	**14.6**	**1.877**	**27.48**	**23.47**	**36.14**
**Quartz**	**(4,-2,-4)**	**6.821**	**75.11**	**15.248**	**14.6**	**1.877**	**27.48**	**23.47**	**36.14**
**Quartz**	**(4,-2,4)**	**6.821**	**75.11**	**15.247**	**14.6**	**1.877**	**27.48**	**23.47**	**36.14**
**Quartz**	**(-2,-2,-4)**	**6.821**	**75.11**	**15.246**	**14.6**	**1.877**	**27.48**	**23.47**	**36.13**
**Quartz**	**(-2,4,-4)**	**6.821**	**75.11**	**15.245**	**14.6**	**1.877**	**27.48**	**23.47**	**36.13**
	Equiv. Refl.: (-2,-2,4)								
**Sapph**	**(1,3,-8)**	**6.633**	**70.03**	**14.231**	**13.7**	**2.565**	**35.02**	**32.07**	**47.48**
	Equiv. Refl.: (1,-4,8), (3,1,8), (3,-4,-8), (4,-1,8), (4,-3,-8), (-1,4,-8), (-1,-3,8), (-3,4,8), (-3,-1,-8) (-4,1,-8), (-4,3,8)								
**Quartz**	**(4,-4,3)**	**6.768**	**73.52**	**13.248**	**12.4**	**2.088**	**25.86**	**26.1**	**36.74**
**Quartz**	**(4,0,-3)**	**6.768**	**73.52**	**13.247**	**12.4**	**2.088**	**25.85**	**26.1**	**36.74**
**Quartz**	**(-4,4,-3)**	**6.768**	**73.52**	**13.246**	**12.4**	**2.088**	**25.86**	**26.1**	**36.74**
**Quartz**	**(-4,0,3)**	**6.768**	**73.52**	**13.245**	**12.4**	**2.088**	**25.85**	**26.1**	**36.74**
**Quartz**	**(0,4,3)**	**6.768**	**73.52**	**13.241**	**12.4**	**2.088**	**25.85**	**26.1**	**36.73**
	Equiv. Refl.: (0,-4,-3)								
**Quartz**	**(3,-4,4)**	**6.975**	**81.19**	**11.677**	**12.4**	**1.094**	**13.56**	**13.67**	**19.26**
**Quartz**	**(3,1,-4)**	**6.975**	**81.19**	**11.676**	**12.4**	**1.094**	**13.56**	**13.67**	**19.26**
**Quartz**	**(1,-4,-4)**	**6.975**	**81.19**	**11.662**	**12.4**	**1.094**	**13.55**	**13.67**	**19.25**
**Quartz**	**(1,3,4)**	**6.975**	**81.19**	**11.66**	**12.4**	**1.094**	**13.55**	**13.67**	**19.25**
**Quartz**	**(-4,3,-4)**	**6.975**	**81.19**	**11.659**	**12.4**	**1.094**	**13.55**	**13.67**	**19.25**
**Quartz**	**(-4,1,4)**	**6.975**	**81.19**	**11.657**	**12.4**	**1.094**	**13.55**	**13.67**	**19.25**
**LiNbO3**	**(1,3,-11)**	**7.024**	**84.34**	**11.187**	**25.3**	**0.699**	**17.68**	**8.74**	**19.72**
	Equiv. Refl.: (1,-4,11), (3,1,11), (3,-4,-11), (4,-1,11), (4,-3,-11), (-1,4,-11), (-1,-3,11), (-3,4,11), (-3,-1,-11) (-4,1,-11), (-4,3,11)								
**Quartz**	**(-3,4,-4)**	**6.975**	**81.19**	**10.598**	**12.4**	**1.094**	**13.56**	**13.67**	**19.26**
	Equiv. Refl.: (-3,-1,4)								
**Quartz**	**(-1,4,4)**	**6.975**	**81.19**	**10.583**	**12.4**	**1.094**	**13.55**	**13.67**	**19.25**
**Quartz**	**(4,-3,4)**	**6.975**	**81.19**	**10.581**	**12.4**	**1.094**	**13.55**	**13.67**	**19.25**
	Equiv. Refl.: (-1,-3,-4)								
**Quartz**	**(4,-1,-4)**	**6.975**	**81.19**	**10.58**	**12.4**	**1.094**	**13.55**	**13.67**	**19.25**
**Sapph**	**(2,3,5)**	**6.978**	**81.38**	**10.3**	**12.8**	**1.069**	**13.67**	**13.37**	**19.12**
	Equiv. Refl.: (2,-5,-5), (3,2,-5), (3,-5,5), (5,-2,-5), (5,-3,5), (-2,5,5), (-2,-3,-5), (-3,5,-5), (-3,-2,5) (-5,2,5), (-5,3,-5)								
**Quartz**	**(-2,-1,-5)**	**6.91**	**78.25**	**8.464**	**8.6**	**1.468**	**12.64**	**18.34**	**22.28**
**Quartz**	**(-2,3,5)**	**6.91**	**78.25**	**8.463**	**8.6**	**1.468**	**12.64**	**18.34**	**22.28**
**Quartz**	**(3,-2,-5)**	**6.91**	**78.25**	**8.462**	**8.6**	**1.468**	**12.64**	**18.34**	**22.28**
**Quartz**	**(3,-1,5)**	**6.91**	**78.25**	**8.458**	**8.6**	**1.468**	**12.64**	**18.34**	**22.28**
**Quartz**	**(-1,3,-5)**	**6.91**	**78.25**	**8.455**	**8.6**	**1.468**	**12.63**	**18.34**	**22.27**
**Quartz**	**(-1,-2,5)**	**6.91**	**78.25**	**8.453**	**8.6**	**1.468**	**12.63**	**18.34**	**22.27**

Ei = Cryst	7.058 Refl (h,k,l)	keV EB [keV]	ΘB [°]	∫ IR dΘ [µrad]	Width [µrad]	Ei cotΘ [meV/µrad]	ΔE [meV]	ΔEg 2m, 50µm [meV]	ΔEt 2m, 50µm [meV]
**Quartz**	**(2,1,5)**	**6.91**	**78.25**	**6.663**	**8.6**	**1.468**	**12.64**	**18.34**	**22.28**
	Equiv. Refl.: (2,-3,-5)								
**Quartz**	**(-3,2,5)**	**6.91**	**78.25**	**6.662**	**8.6**	**1.468**	**12.64**	**18.34**	**22.28**
**Quartz**	**(-3,1,-5)**	**6.91**	**78.25**	**6.657**	**8.6**	**1.468**	**12.64**	**18.34**	**22.28**
**Quartz**	**(1,-3,5)**	**6.91**	**78.25**	**6.654**	**8.6**	**1.468**	**12.63**	**18.34**	**22.27**
**Quartz**	**(1,2,-5)**	**6.91**	**78.25**	**6.653**	**8.6**	**1.468**	**12.63**	**18.34**	**22.27**
**Quartz**	**(-1,5,1)**	**6.775**	**73.71**	**5.864**	**6.6**	**2.063**	**13.55**	**25.79**	**29.13**
	Equiv. Refl.: (-1,-4,-1), (-4,5,-1), (-4,-1,1)								
**Quartz**	**(5,-1,-1)**	**6.775**	**73.71**	**5.862**	**6.6**	**2.063**	**13.55**	**25.79**	**29.13**
	Equiv. Refl.: (5,-4,1)								
**Quartz**	**(1,4,1)**	**6.775**	**73.71**	**5.739**	**6.6**	**2.063**	**13.55**	**25.79**	**29.13**
	Equiv. Refl.: (4,1,-1), (4,-5,1)								
**Quartz**	**(1,-5,-1)**	**6.775**	**73.71**	**5.738**	**6.6**	**2.063**	**13.55**	**25.79**	**29.13**
**Quartz**	**(-5,1,1)**	**6.775**	**73.71**	**5.737**	**6.6**	**2.063**	**13.55**	**25.79**	**29.13**
	Equiv. Refl.: (-5,4,-1)								
**Quartz**	**(3,-5,-2)**	**6.752**	**73.08**	**5.593**	**6**	**2.147**	**12.98**	**26.84**	**29.81**
**Quartz**	**(3,2,2)**	**6.752**	**73.08**	**5.592**	**6**	**2.147**	**12.98**	**26.84**	**29.81**
**Quartz**	**(2,3,-2)**	**6.752**	**73.08**	**5.59**	**6**	**2.147**	**12.97**	**26.84**	**29.81**
	Equiv. Refl.: (2,-5,2), (-5,2,-2), (-5,3,2)								
**Quartz**	**(-3,5,2)**	**6.752**	**73.08**	**5.356**	**6**	**2.147**	**12.98**	**26.84**	**29.81**
**Quartz**	**(-3,-2,-2)**	**6.752**	**73.08**	**5.355**	**6**	**2.147**	**12.98**	**26.84**	**29.81**
**Quartz**	**(5,-2,2)**	**6.752**	**73.08**	**5.354**	**6**	**2.147**	**12.97**	**26.84**	**29.81**
	Equiv. Refl.: (5,-3,-2), (-2,5,-2)								
**Quartz**	**(-2,-3,2)**	**6.752**	**73.08**	**5.353**	**6**	**2.147**	**12.97**	**26.84**	**29.81**
**Quartz**	**(0,-1,-6)**	**7.035**	**85.34**	**4.35**	**8.5**	**0.576**	**4.9**	**7.19**	**8.71**
**Quartz**	**(0,1,6)**	**7.035**	**85.34**	**4.348**	**8.5**	**0.576**	**4.9**	**7.19**	**8.71**
**Quartz**	**(1,0,-6)**	**7.035**	**85.34**	**4.298**	**8.5**	**0.576**	**4.87**	**7.19**	**8.69**
**Quartz**	**(1,-1,6)**	**7.035**	**85.34**	**4.297**	**8.5**	**0.576**	**4.87**	**7.19**	**8.69**
**Quartz**	**(-1,0,6)**	**7.035**	**85.34**	**4.294**	**8.5**	**0.576**	**4.87**	**7.19**	**8.69**
**Quartz**	**(-1,1,-6)**	**7.035**	**85.34**	**4.293**	**8.5**	**0.576**	**4.87**	**7.19**	**8.69**
**LiNbO3**	**(1,2,-13)**	**6.88**	**77.09**	**3**	**8.7**	**1.618**	**14.02**	**20.22**	**24.61**
	Equiv. Refl.: (1,-3,13), (2,1,13), (2,-3,-13), (3,-1,13), (3,-2,-13), (-1,3,-13), (-1,-2,13), (-2,3,13), (-2,-1,-13) (-3,1,-13), (-3,2,13)								
**Quartz**	**(2,3,2)**	**6.752**	**73.08**	**1.921**	**3**	**2.147**	**6.46**	**26.84**	**27.61**
	Equiv. Refl.: (2,-5,-2)								
**Quartz**	**(-5,3,-2)**	**6.752**	**73.08**	**1.919**	**3**	**2.147**	**6.46**	**26.84**	**27.6**
**Quartz**	**(3,-5,2)**	**6.752**	**73.08**	**1.918**	**3**	**2.147**	**6.45**	**26.84**	**27.6**
	Equiv. Refl.: (-5,2,2)								
**Quartz**	**(3,2,-2)**	**6.752**	**73.08**	**1.917**	**3**	**2.147**	**6.45**	**26.84**	**27.6**
**Quartz**	**(-2,5,2)**	**6.752**	**73.08**	**1.518**	**3**	**2.147**	**6.46**	**26.84**	**27.61**
	Equiv. Refl.: (-2,-3,-2)								
**Quartz**	**(5,-3,2)**	**6.752**	**73.08**	**1.516**	**3**	**2.147**	**6.46**	**26.84**	**27.6**
**Quartz**	**(5,-2,-2)**	**6.752**	**73.08**	**1.515**	**3**	**2.147**	**6.45**	**26.84**	**27.6**
	Equiv. Refl.: (-3,5,-2), (-3,-2,2)								
**LiNbO3**	**(2,3,-7)**	**6.822**	**75.13**	**0.974**	**4.5**	**1.874**	**8.52**	**23.43**	**24.93**
	Equiv. Refl.: (2,-5,7), (3,2,7), (3,-5,-7), (5,-2,7), (5,-3,-7), (-2,5,-7), (-2,-3,7), (-3,5,7), (-3,-2,-7) (-5,2,-7), (-5,3,7)								
**Quartz**	**(1,4,0)**	**6.677**	**71.08**	**0.526**	**1**	**2.419**	**2.44**	**30.24**	**30.34**
	Equiv. Refl.: (1,-5,0), (4,1,0), (4,-5,0), (-5,1,0), (-5,4,0)								
**Quartz**	**(5,-1,0)**	**6.677**	**71.08**	**0.172**	**1**	**2.419**	**2.44**	**30.24**	**30.34**
	Equiv. Refl.: (5,-4,0), (-1,5,0), (-1,-4,0), (-4,5,0), (-4,-1,0)								

Ei =	7.709	keV						ΔEg	ΔEt
Cryst	Refl	EB	ΘB	∫ IR dΘ	Width	Ei cotΘ	ΔE	2m, 50µm	2m, 50µm
	(h,k,l)	[keV]	[°]	[µrad]	[µrad]	[meV/µrad]	[meV]	[meV]	[meV]
Ge	(4,4,4)	7.592	80	67	62.2	1.36	84.55	17	86.24
Si	(3,3,5)	7.485	76.15	18.2	16.9	1.9	32.15	23.75	39.97
LiNbO3	(3,3,6)	7.707	88.79	328.6	331.2	0.163	53.98	2.04	54.02
	Equiv. Refl.: (3,-6,6), (6,-3,6), (-3,6,6), (-3,-3,6), (-6,3,6)								
LiNbO3	(3,3,-6)	7.707	88.79	311	331.2	0.163	53.98	2.04	54.02
	Equiv. Refl.: (3,-6,-6), (6,-3,-6), (-3,6,-6), (-3,-3,-6), (-6,3,-6)								
Sapph	(0,4,-10)	7.681	85.09	94.3	80.6	0.662	53.4	8.28	54.03
	Equiv. Refl.: (0,-4,10), (4,0,10), (4,-4,-10), (-4,0,-10), (-4,4,10)								
LiNbO3	(0,-2,-16)	7.676	84.72	88.9	85.5	0.712	60.91	8.91	61.56
	Equiv. Refl.: (2,0,-16), (-2,2,-16)								
LiNbO3	(0,2,16)	7.676	84.72	80.7	85.5	0.712	60.91	8.91	61.56
	Equiv. Refl.: (2,-2,16), (-2,0,16)								
Quartz	(0,5,-2)	7.638	82.2	55.5	46.6	1.057	49.22	13.21	50.97
	Equiv. Refl.: (0,-5,2), (5,0,2), (5,-5,-2), (-5,0,-2), (-5,5,2)								
LiNbO3	(2,4,4)	7.572	79.18	43.1	41.8	1.473	61.6	18.41	64.29
	Equiv. Refl.: (4,-6,4), (6,-4,4), (-2,6,4), (-4,-2,4), (-6,2,4)								
LiNbO3	(2,-6,-4)	7.572	79.18	39.5	41.8	1.473	61.6	18.41	64.29
	Equiv. Refl.: (4,2,-4), (6,-2,-4), (-2,-4,-4), (-4,6,-4), (-6,4,-4)								
LiNbO3	(2,-5,10)	7.532	77.7	38.3	37.1	1.681	62.38	21.01	65.82
	Equiv. Refl.: (3,2,10), (5,-2,10), (-2,-3,10), (-3,5,10), (-5,3,10)								
LiNbO3	(2,4,-2)	7.412	74.04	37.3	36.1	2.204	79.57	27.55	84.21
	Equiv. Refl.: (4,-6,-2), (6,-4,-2), (-2,6,-2), (-4,-2,-2), (-6,2,-2)								
LiNbO3	(2,-6,2)	7.412	74.04	37.1	36.1	2.204	79.57	27.55	84.21
	Equiv. Refl.: (4,2,2), (6,-2,2), (-2,-4,2), (-4,6,2), (-6,4,2)								
LiNbO3	(2,3,-10)	7.532	77.7	35	37.1	1.681	62.38	21.01	65.82
	Equiv. Refl.: (3,-5,-10), (5,-3,-10), (-2,5,-10), (-3,-2,-10), (-5,2,-10)								
LiNbO3	(1,-3,-14)	7.261	70.38	29.8	29	2.748	79.73	34.35	86.82
	Equiv. Refl.: (2,1,-14), (3,-1,-14), (-1,-2,-14), (-2,3,-14), (-3,2,-14)								
LiNbO3	(1,2,14)	7.261	70.38	29.6	29	2.748	79.73	34.35	86.82
	Equiv. Refl.: (2,-3,14), (3,-2,14), (-1,3,14), (-2,-1,14), (-3,1,14)								
Quartz	(3,3,1)	7.657	83.35	28.7	25.8	0.899	23.22	11.24	25.8
	Equiv. Refl.: (3,3,-1), (3,-6,1), (3,-6,-1), (-6,3,1), (-6,3,-1)								
Quartz	(0,2,6)	7.474	75.8	27.7	23.4	1.95	45.69	24.38	51.78
	Equiv. Refl.: (0,-2,-6), (2,0,-6), (2,-2,6), (-2,0,6), (-2,2,-6)								
Quartz	(2,2,5)	7.64	82.31	27.6	24.7	1.041	25.74	13.01	28.84
	Equiv. Refl.: (2,2,-5), (2,-4,5), (2,-4,-5)								
Quartz	(-4,2,5)	7.64	82.31	27.5	24.7	1.041	25.73	13.01	28.83
	Equiv. Refl.: (-4,2,-5)								
LiNbO3	(0,-1,16)	7.289	70.99	27.2	26.7	2.655	70.91	33.19	78.29
	Equiv. Refl.: (1,0,16), (-1,1,16), (6,-3,1), (6,-3,-1), (-3,6,1), (-3,6,-1), (-3,-3,1), (-3,-3,-1)								
Sapph	(1,4,6)	7.465	75.55	27.1	23.6	1.986	46.96	24.83	53.12
	Equiv. Refl.: (1,4,-6), (1,-5,6), (1,-5,-6), (4,1,6), (4,1,-6), (4,-5,6), (4,-5,-6), (5,-1,6), (5,-1,-6) (5,-4,6), (5,-4,-6), (-1,5,6), (-1,5,-6), (-1,-4,6), (-1,-4,-6), (-4,5,6), (-4,5,-6), (-4,-1,6) (-4,-1,-6), (-5,1,6), (-5,1,-6), (-5,4,6), (-5,4,-6)								
Sapph	(1,1,15)	7.619	81.23	26.9	24.9	1.19	29.64	14.87	33.16
	Equiv. Refl.: (1,1,-15), (1,-2,15), (1,-2,-15), (2,-1,15), (2,-1,-15), (-1,2,15), (-1,2,-15), (-1,-1,15), (-1,-1,-15) (-2,1,15), (-2,1,-15), (-2,4,5), (-2,4,-5), (-2,-2,5), (-2,-2,-5)								
Quartz	(4,-2,5)	7.64	82.31	26.8	24.7	1.041	25.73	13.01	28.83
	Equiv. Refl.: (4,-2,-5)								
LiNbO3	(0,1,-16)	7.289	70.99	26.3	26.7	2.655	70.91	33.19	78.29
	Equiv. Refl.: (1,-1,-16), (-1,0,-16)								

Ei =	7.709	keV						ΔEg	ΔEt
**Cryst**	**Refl**	**EB**	**ΘB**	**∫IR dΘ**	**Width**	**Ei cotΘ**	**ΔE**	**2m, 50µm**	**2m, 50µm**
	**(h,k,l)**	**[keV]**	**[°]**	**[µrad]**	**[µrad]**	**[meV/µrad]**	**[meV]**	**[meV]**	**[meV]**
**Quartz**	**(1,4,3)**	**7.511**	**77**	**26.1**	**22.4**	**1.78**	**39.83**	**22.25**	**45.62**
	Equiv. Refl.: (1,-5,-3), (4,1,-3), (4,-5,3), (-1,5,3), (-1,-4,-3), (-4,5,-3), (-4,-1,3), (-5,1,3), (-5,4,-3)								
**Quartz**	**(5,-1,-3)**	**7.511**	**77**	**26**	**22.4**	**1.78**	**39.82**	**22.25**	**45.62**
	Equiv. Refl.: (5,-4,3)								
**Sapph**	**(0,2,-14)**	**7.328**	**71.92**	**21.8**	**19**	**2.517**	**47.91**	**31.46**	**57.32**
	Equiv. Refl.: (0,-2,14), (2,0,14), (2,-2,-14), (-2,0,-14), (-2,2,14)								
**LiNbO3**	**(2,4,-5)**	**7.69**	**85.98**	**20.9**	**36.5**	**0.542**	**19.81**	**6.78**	**20.94**
	Equiv. Refl.: (2,-6,5), (4,2,5), (4,-6,-5), (6,-2,5), (6,-4,-5), (-2,6,-5), (-2,-4,5), (-4,6,5), (-4,-2,-5) (-6,2,-5), (-6,4,5)								
**Sapph**	**(0,3,12)**	**7.292**	**71.07**	**19.1**	**16.7**	**2.644**	**44.2**	**33.05**	**55.19**
	Equiv. Refl.: (0,3,-12), (0,-3,12), (0,-3,-12), (3,0,12), (3,0,-12), (3,-3,12), (3,-3,-12), (-3,0,12), (-3,0,-12) (-3,3,12), (-3,3,-12)								
**Quartz**	**(6,-3,0)**	**7.571**	**79.13**	**18.8**	**17**	**1.48**	**25.23**	**18.5**	**31.29**
	Equiv. Refl.: (-3,6,0), (-3,-3,0)								
**Quartz**	**(3,3,0)**	**7.571**	**79.13**	**18.7**	**17**	**1.48**	**25.23**	**18.5**	**31.29**
	Equiv. Refl.: (3,-6,0), (-6,3,0)								
**LiNbO3**	**(1,3,13)**	**7.677**	**84.74**	**9.3**	**20.3**	**0.71**	**14.44**	**8.87**	**16.94**
	Equiv. Refl.: (1,-4,-13), (3,1,-13), (3,-4,13), (4,-1,-13), (4,-3,13), (-1,4,13), (-1,-3,-13), (-3,4,-13), (-3,-1,13) (-4,1,13), (-4,3,-13)								
**Quartz**	**(0,4,4)**	**7.417**	**74.19**	**6.9**	**6.8**	**2.183**	**14.95**	**27.29**	**31.12**
	Equiv. Refl.: (0,-4,-4), (4,0,-4), (4,-4,4), (-4,0,4), (-4,4,-4)								
**Quartz**	**(0,2,-6)**	**7.474**	**75.8**	**6.5**	**6.6**	**1.95**	**12.78**	**24.38**	**27.52**
	Equiv. Refl.: (0,4,-4), (0,-2,6), (0,-4,4), (2,0,6), (2,-2,-6), (4,0,4), (4,-4,-4), (-2,0,-6), (-2,2,6) (-4,0,-4), (-4,4,4)								
**LiNbO3**	**(2,4,1)**	**7.371**	**72.98**	**5.8**	**9.6**	**2.36**	**22.74**	**29.5**	**37.25**
	Equiv. Refl.: (2,-6,-1), (4,2,-1), (4,-6,1), (6,-2,-1), (6,-4,1), (-2,6,1), (-2,-4,-1), (-4,6,-1), (-4,-2,1) (-6,2,1), (-6,4,-1)								
**Quartz**	**(2,-1,6)**	**7.33**	**71.96**	**5.5**	**5.5**	**2.51**	**13.91**	**31.38**	**34.32**
	Equiv. Refl.: (2,-1,-6), (-1,2,6), (-1,2,-6), (-1,-1,6), (-1,-1,-6)								
**Quartz**	**(1,1,6)**	**7.33**	**71.96**	**5.3**	**5.5**	**2.51**	**13.9**	**31.38**	**34.32**
	Equiv. Refl.: (1,1,-6), (1,-2,6), (1,-2,-6), (-2,1,6), (-2,1,-6)								
**Sapph**	**(2,3,-7)**	**7.36**	**72.69**	**4.5**	**5.2**	**2.403**	**12.45**	**30.04**	**32.52**
	Equiv. Refl.: (2,-5,7), (3,2,7), (3,-5,-7), (5,-2,7), (5,-3,-7), (-2,5,-7), (-2,-3,7), (-3,5,7), (-3,-2,-7) (-5,2,-7), (-5,3,7)								
**LiNbO3**	**(1,4,9)**	**7.537**	**77.86**	**3.4**	**8.2**	**1.658**	**13.64**	**20.73**	**24.82**
	Equiv. Refl.: (1,4,-9), (1,-5,9), (1,-5,-9), (4,1,9), (4,1,-9), (4,-5,9), (4,-5,-9), (5,-1,9), (5,-1,-9) (5,-4,9), (5,-4,-9), (-1,5,9), (-1,5,-9), (-1,-4,9), (-1,-4,-9), (-4,5,9), (-4,5,-9), (-4,-1,9) (-4,-1,-9), (-5,1,9), (-5,1,-9), (-5,4,9), (-5,4,-9)								
**Sapph**	**(1,2,-13)**	**7.372**	**72.99**	**3.2**	**4.3**	**2.358**	**10.2**	**29.48**	**31.2**
	Equiv. Refl.: (1,-3,13), (2,1,13), (2,-3,-13), (3,-1,13), (3,-2,-13), (-1,3,-13), (-1,-2,13), (-2,3,13), (-2,-1,-13) (-3,1,-13), (-3,2,13)								
**Quartz**	**(0,5,2)**	**7.638**	**82.2**	**2.8**	**4.8**	**1.057**	**5.03**	**13.21**	**14.13**
	Equiv. Refl.: (0,-5,-2), (5,0,-2), (5,-5,2), (-5,0,2), (-5,5,-2)								
**Sapph**	**(2,3,8)**	**7.588**	**79.85**	**2.6**	**4.6**	**1.381**	**6.3**	**17.26**	**18.37**
	Equiv. Refl.: (2,-5,-8), (3,2,-8), (3,-5,8), (5,-2,-8), (5,-3,8), (-2,5,8), (-2,-3,-8), (-3,5,-8), (-3,-2,8) (-5,2,8), (-5,3,-8)								
**Sapph**	**(1,3,-11)**	**7.549**	**78.31**	**2.4**	**4.1**	**1.596**	**6.52**	**19.95**	**20.98**
	Equiv. Refl.: (1,-4,11), (3,1,11), (3,-4,-11), (4,-1,11), (4,-3,-11), (-1,4,-11), (-1,-3,11), (-3,4,11), (-3,-1,-11) (-4,1,-11), (-4,3,11), (5,-1,3), (5,-4,-3), (-1,5,-3), (-1,-4,3), (-4,5,3), (-4,-1,-3)								
**Quartz**	**(1,4,-3)**	**7.511**	**77**	**2**	**3.4**	**1.78**	**6.09**	**22.25**	**23.07**
	Equiv. Refl.: (1,-5,3), (4,1,3), (4,-5,-3), (-5,1,-3), (-5,4,3)								

Ei = Cryst	7.709 Refl (h,k,l)	keV EB [keV]	ΘB [°]	∫ IR dΘ [μrad]	Width [μrad]	Ei cotΘ [meV/μrad]	ΔE [meV]	ΔEg 2m, 50μm [meV]	ΔEt 2m, 50μm [meV]
**Quartz**	**(0,5,-1)**	**7.375**	**73.06**	**0.7**	**1.5**	**2.347**	**3.64**	**29.34**	**29.57**
	Equiv. Refl.: (0,-5,1), (5,0,1), (5,-5,-1), (-5,0,-1), (-5,5,1)								
**LiNbO3**	**(3,3,3)**	**7.349**	**72.41**	**0.3**	**1.9**	**2.444**	**4.52**	**30.54**	**30.88**
	Equiv. Refl.: (3,3,-3), (3,-6,3), (3,-6,-3), (6,-3,3), (6,-3,-3), (-3,6,3), (-3,6,-3), (-3,-3,3), (-3,-3,-3) (-6,3,3), (-6,3,-3)								
**Quartz**	**(0,5,0)**	**7.285**	**70.91**	**0.2**	**0.7**	**2.668**	**1.91**	**33.35**	**33.41**
	Equiv. Refl.: (0,-5,0), (5,0,0), (5,-5,0), (-5,0,0), (-5,5,0)								

Ei =	6.93	keV						ΔEg	ΔEt
Cryst	Refl	EB	ΘB	∫ IR dΘ	Width	Ei cotΘ	ΔE	2m, 50µm	2m, 50µm
	(h,k,l)	[keV]	[°]	[µrad]	[µrad]	[meV/µrad]	[meV]	[meV]	[meV]
**Si**	**(1,3,5)**	**6.753**	**77.02**	**26.1773**	**25.2**	**1.5976**	**40.26**	**19.97**	**44.94**
	Equiv. Refl.: (1,5,3), (3,1,5), (3,5,1), (5,1,3), (5,3,1)								
**LiNbO3**	**(4,1,-6)**	**6.914**	**86.09**	**186.663**	**180.6**	**0.474**	**85.6**	**5.92**	**85.8**
	Equiv. Refl.: (-1,-4,-6), (-4,5,-6), (-5,4,-6)								
**LiNbO3**	**(1,-5,-6)**	**6.914**	**86.09**	**186.662**	**180.6**	**0.474**	**85.6**	**5.92**	**85.8**
	Equiv. Refl.: (5,-1,-6)								
**LiNbO3**	**(1,4,-6)**	**6.914**	**86.09**	**184.411**	**177.9**	**0.474**	**84.32**	**5.92**	**84.52**
	Equiv. Refl.: (-1,5,-6), (-4,-1,-6), (-5,1,-6)								
**LiNbO3**	**(4,-5,-6)**	**6.914**	**86.09**	**184.41**	**177.9**	**0.474**	**84.32**	**5.92**	**84.52**
	Equiv. Refl.: (5,-4,-6)								
**LiNbO3**	**(1,4,6)**	**6.914**	**86.09**	**175.522**	**180.6**	**0.474**	**85.6**	**5.92**	**85.8**
	Equiv. Refl.: (4,-5,6), (5,-4,6), (-4,-1,6)								
**LiNbO3**	**(-1,5,6)**	**6.914**	**86.09**	**175.521**	**180.6**	**0.474**	**85.6**	**5.92**	**85.8**
	Equiv. Refl.: (-5,1,6)								
**LiNbO3**	**(1,-5,6)**	**6.914**	**86.09**	**171.188**	**177.9**	**0.474**	**84.32**	**5.92**	**84.52**
	Equiv. Refl.: (4,1,6), (5,-1,6), (-1,-4,6), (-4,5,6), (-5,4,6)								
**LiNbO3**	**(0,-2,14)**	**6.85**	**81.3**	**83.771**	**80.8**	**1.06**	**85.65**	**13.25**	**86.67**
	Equiv. Refl.: (2,0,14), (-2,2,14)								
**LiNbO3**	**(0,2,-14)**	**6.85**	**81.3**	**77.677**	**80.8**	**1.06**	**85.65**	**13.25**	**86.67**
	Equiv. Refl.: (2,-2,-14), (-2,0,-14)								
**LiNbO3**	**(0,-3,-12)**	**6.797**	**78.75**	**68.85**	**63.6**	**1.379**	**87.7**	**17.23**	**89.38**
	Equiv. Refl.: (3,0,-12), (-3,3,-12)								
**LiNbO3**	**(0,3,-12)**	**6.797**	**78.75**	**67.6**	**62.5**	**1.379**	**86.14**	**17.23**	**87.85**
	Equiv. Refl.: (3,-3,-12), (-3,0,-12)								
**LiNbO3**	**(0,3,12)**	**6.797**	**78.75**	**58.322**	**63.6**	**1.379**	**87.7**	**17.23**	**89.38**
	Equiv. Refl.: (3,-3,12), (-3,0,12)								
**Quartz**	**(-1,3,5)**	**6.91**	**85.67**	**57.389**	**53.1**	**0.525**	**27.86**	**6.56**	**28.62**
**Quartz**	**(-1,-2,-5)**	**6.91**	**85.67**	**57.385**	**53.1**	**0.525**	**27.86**	**6.56**	**28.62**
**Quartz**	**(-2,3,-5)**	**6.91**	**85.67**	**57.357**	**53.1**	**0.525**	**27.85**	**6.56**	**28.61**
**Quartz**	**(-2,-1,5)**	**6.91**	**85.67**	**57.353**	**53**	**0.525**	**27.85**	**6.56**	**28.61**
**Quartz**	**(3,-1,-5)**	**6.91**	**85.67**	**57.351**	**53**	**0.525**	**27.85**	**6.56**	**28.61**
**Quartz**	**(3,-2,5)**	**6.91**	**85.67**	**57.345**	**53**	**0.525**	**27.84**	**6.56**	**28.61**
**LiNbO3**	**(0,-3,12)**	**6.797**	**78.75**	**56.82**	**62.5**	**1.379**	**86.14**	**17.23**	**87.85**
	Equiv. Refl.: (3,0,12)								
**LiNbO3**	**(-3,3,12)**	**6.797**	**78.75**	**56.819**	**62.5**	**1.379**	**86.14**	**17.23**	**87.85**
**LiNbO3**	**(1,-4,-10)**	**6.718**	**75.79**	**56.244**	**53.6**	**1.755**	**93.97**	**21.93**	**96.5**
	Equiv. Refl.: (3,1,-10), (4,-1,-10), (-1,-3,-10), (-3,4,-10), (-4,3,-10)								
**Quartz**	**(1,-3,-5)**	**6.91**	**85.67**	**55.584**	**53.1**	**0.525**	**27.86**	**6.56**	**28.62**
**Quartz**	**(1,2,5)**	**6.91**	**85.67**	**55.58**	**53.1**	**0.525**	**27.86**	**6.56**	**28.62**
**Quartz**	**(2,-3,5)**	**6.91**	**85.67**	**55.568**	**53.1**	**0.525**	**27.85**	**6.56**	**28.61**
**Quartz**	**(2,1,-5)**	**6.91**	**85.67**	**55.563**	**53**	**0.525**	**27.85**	**6.56**	**28.61**
**Quartz**	**(-3,1,5)**	**6.91**	**85.67**	**55.56**	**53**	**0.525**	**27.85**	**6.56**	**28.61**
**Quartz**	**(-3,2,-5)**	**6.91**	**85.67**	**55.554**	**53**	**0.525**	**27.84**	**6.56**	**28.61**
**Sapph**	**(2,3,-4)**	**6.83**	**80.25**	**53.304**	**47.1**	**1.191**	**56.03**	**14.89**	**57.98**
	Equiv. Refl.: (2,-5,4), (3,2,4), (3,-5,-4), (5,-2,4), (5,-3,-4), (-2,5,-4), (-2,-3,4), (-3,5,4), (-3,-2,-4) (-5,2,-4), (-5,3,4)								
**LiNbO3**	**(1,3,10)**	**6.718**	**75.79**	**51.818**	**53.6**	**1.755**	**93.97**	**21.93**	**96.5**
	Equiv. Refl.: (3,-4,10), (4,-3,10), (-1,4,10), (-3,-1,10), (-4,1,10)								

**Ei =**	**6.93**	**keV**						**ΔEg**	**ΔEt**
**Cryst**	**Refl**	**EB**	**ΘB**	**∫ IR dΘ**	**Width**	**Ei cotΘ**	**ΔE**	**2m, 50μm**	**2m, 50μm**
	**(h,k,l)**	**[keV]**	**[°]**	**[μrad]**	**[μrad]**	**[meV/μrad]**	**[meV]**	**[meV]**	**[meV]**
**Sapph**	**(1,4,0)**	**6.894**	**84.17**	**46.911**	**45.3**	**0.708**	**32.04**	**8.85**	**33.24**
	Equiv. Refl.: (1,-5,0), (4,1,0), (4,-5,0), (5,-1,0), (5,-4,0), (-1,5,0), (-1,-4,0), (-4,5,0), (-4,-1,0) (-5,1,0), (-5,4,0)								
**Sapph**	**(0,1,14)**	**6.849**	**81.25**	**41.637**	**38.5**	**1.067**	**41.07**	**13.33**	**43.18**
	Equiv. Refl.: (0,-1,-14), (1,0,-14), (1,-1,14), (-1,0,14), (-1,1,-14)								
**LiNbO3**	**(0,4,8)**	**6.613**	**72.61**	**41.602**	**39.6**	**2.171**	**85.93**	**27.14**	**90.11**
	Equiv. Refl.: (4,-4,8), (-4,0,8)								
**LiNbO3**	**(0,-4,-8)**	**6.613**	**72.61**	**36.253**	**39.6**	**2.171**	**85.93**	**27.14**	**90.11**
	Equiv. Refl.: (4,0,-8), (-4,4,-8)								
**Quartz**	**(0,0,6)**	**6.882**	**83.26**	**31.28**	**30**	**0.819**	**24.57**	**10.24**	**26.62**
	Equiv. Refl.: (0,0,-6)								
**Quartz**	**(0,4,-3)**	**6.768**	**77.59**	**28.785**	**25.7**	**1.525**	**39.25**	**19.07**	**43.64**
	Equiv. Refl.: (0,-4,3)								
**Quartz**	**(-4,0,-3)**	**6.768**	**77.59**	**28.775**	**25.7**	**1.525**	**39.24**	**19.07**	**43.62**
**Quartz**	**(4,0,3)**	**6.768**	**77.59**	**28.774**	**25.7**	**1.525**	**39.24**	**19.07**	**43.62**
	Equiv. Refl.: (-4,4,3)								
**Quartz**	**(4,-4,-3)**	**6.768**	**77.59**	**28.773**	**25.7**	**1.525**	**39.24**	**19.07**	**43.62**
**Quartz**	**(4,1,1)**	**6.775**	**77.84**	**26.526**	**23.6**	**1.493**	**35.25**	**18.66**	**39.89**
	Equiv. Refl.: (4,-5,-1)								
**Quartz**	**(1,4,-1)**	**6.775**	**77.84**	**26.523**	**23.6**	**1.493**	**35.25**	**18.66**	**39.88**
**Quartz**	**(1,-5,1)**	**6.775**	**77.84**	**26.522**	**23.6**	**1.493**	**35.25**	**18.66**	**39.88**
**Quartz**	**(-5,1,-1)**	**6.775**	**77.84**	**26.521**	**23.6**	**1.493**	**35.24**	**18.66**	**39.88**
	Equiv. Refl.: (-5,4,1)								
**Quartz**	**(-4,5,1)**	**6.775**	**77.84**	**25.424**	**23.6**	**1.493**	**35.25**	**18.66**	**39.89**
	Equiv. Refl.: (-4,-1,-1)								
**Quartz**	**(-1,-4,1)**	**6.775**	**77.84**	**25.422**	**23.6**	**1.493**	**35.25**	**18.66**	**39.88**
**Quartz**	**(-1,5,-1)**	**6.775**	**77.84**	**25.421**	**23.6**	**1.493**	**35.25**	**18.66**	**39.88**
**Quartz**	**(5,-1,1)**	**6.775**	**77.84**	**25.419**	**23.6**	**1.493**	**35.24**	**18.66**	**39.88**
	Equiv. Refl.: (5,-4,-1)								
**Sapph**	**(2,2,9)**	**6.754**	**77.05**	**23.899**	**22.8**	**1.594**	**36.33**	**19.92**	**41.44**
	Equiv. Refl.: (2,2,-9), (2,-4,9), (2,-4,-9), (4,-2,9), (4,-2,-9), (-2,4,9), (-2,4,-9), (-2,-2,9), (-2,-2,-9) (-4,2,9), (-4,2,-9)								
**Quartz**	**(2,-4,-4)**	**6.821**	**79.82**	**23.401**	**21.7**	**1.245**	**27**	**15.56**	**31.17**
**Quartz**	**(-4,2,4)**	**6.821**	**79.82**	**23.4**	**21.7**	**1.245**	**27**	**15.56**	**31.16**
**Quartz**	**(-4,2,-4)**	**6.821**	**79.82**	**23.398**	**21.7**	**1.245**	**27**	**15.56**	**31.16**
**Quartz**	**(2,2,4)**	**6.821**	**79.82**	**23.397**	**21.7**	**1.245**	**27**	**15.56**	**31.16**
	Equiv. Refl.: (2,2,-4), (2,-4,4)								
**Quartz**	**(-2,-1,-5)**	**6.91**	**85.67**	**22.483**	**23.7**	**0.525**	**12.42**	**6.56**	**14.05**
**Quartz**	**(-2,3,5)**	**6.91**	**85.67**	**22.48**	**23.7**	**0.525**	**12.42**	**6.56**	**14.05**
**Quartz**	**(3,-2,-5)**	**6.91**	**85.67**	**22.478**	**23.7**	**0.525**	**12.42**	**6.56**	**14.05**
**Quartz**	**(3,-1,5)**	**6.91**	**85.67**	**22.466**	**23.6**	**0.525**	**12.41**	**6.56**	**14.04**
**Quartz**	**(-1,3,-5)**	**6.91**	**85.67**	**22.458**	**23.6**	**0.525**	**12.41**	**6.56**	**14.04**
**Quartz**	**(-1,-2,5)**	**6.91**	**85.67**	**22.452**	**23.6**	**0.525**	**12.41**	**6.56**	**14.04**
**Quartz**	**(-2,4,4)**	**6.821**	**79.82**	**22.415**	**21.7**	**1.245**	**27**	**15.56**	**31.17**
**Quartz**	**(4,-2,-4)**	**6.821**	**79.82**	**22.414**	**21.7**	**1.245**	**27**	**15.56**	**31.16**
**Quartz**	**(4,-2,4)**	**6.821**	**79.82**	**22.412**	**21.7**	**1.245**	**27**	**15.56**	**31.16**
**Quartz**	**(-2,-2,-4)**	**6.821**	**79.82**	**22.411**	**21.7**	**1.245**	**27**	**15.56**	**31.16**
**Quartz**	**(-2,4,-4)**	**6.821**	**79.82**	**22.409**	**21.7**	**1.245**	**27**	**15.56**	**31.16**
	Equiv. Refl.: (-2,-2,4)								
**Quartz**	**(4,-4,3)**	**6.768**	**77.59**	**17.697**	**16.7**	**1.525**	**25.41**	**19.07**	**31.77**

Ei =<br>Cryst	6.93<br>Refl<br>(h,k,l)	keV<br>EB<br>[keV]	ΘB<br>[°]	∫ IR dΘ<br>[μrad]	Width<br>[μrad]	Ei cotΘ<br>[meV/μrad]	ΔE<br>[meV]	ΔEg<br>2m, 50μm<br>[meV]	ΔEt<br>2m, 50μm<br>[meV]
**Quartz**	**(4,0,-3)**	**6.768**	**77.59**	**17.696**	**16.7**	**1.525**	**25.41**	**19.07**	**31.77**
**Quartz**	**(-4,4,-3)**	**6.768**	**77.59**	**17.695**	**16.7**	**1.525**	**25.41**	**19.07**	**31.77**
**Quartz**	**(-4,0,3)**	**6.768**	**77.59**	**17.694**	**16.7**	**1.525**	**25.41**	**19.07**	**31.77**
**Quartz**	**(0,4,3)**	**6.768**	**77.59**	**17.689**	**16.7**	**1.525**	**25.4**	**19.07**	**31.76**
	Equiv. Refl.: (0,-4,-3)								
**Quartz**	**(2,1,5)**	**6.91**	**85.67**	**17.557**	**23.7**	**0.525**	**12.42**	**6.56**	**14.05**
**Quartz**	**(2,-3,-5)**	**6.91**	**85.67**	**17.555**	**23.7**	**0.525**	**12.42**	**6.56**	**14.05**
**Quartz**	**(-3,2,5)**	**6.91**	**85.67**	**17.553**	**23.7**	**0.525**	**12.42**	**6.56**	**14.05**
**Quartz**	**(-3,1,-5)**	**6.91**	**85.67**	**17.541**	**23.6**	**0.525**	**12.41**	**6.56**	**14.04**
**Quartz**	**(1,-3,5)**	**6.91**	**85.67**	**17.533**	**23.6**	**0.525**	**12.41**	**6.56**	**14.04**
**Quartz**	**(1,2,-5)**	**6.91**	**85.67**	**17.529**	**23.6**	**0.525**	**12.41**	**6.56**	**14.04**
**Sapph**	**(1,3,-8)**	**6.633**	**73.18**	**16.986**	**16.4**	**2.095**	**34.41**	**26.19**	**43.25**
	Equiv. Refl.: (1,-4,8), (3,1,8), (3,-4,-8), (4,-1,8), (4,-3,-8), (-1,4,-8), (-1,-3,8), (-3,4,8), (-3,-1,-8) (-4,1,-8), (-4,3,8)								
**Quartz**	**(-1,5,1)**	**6.775**	**77.84**	**7.858**	**8.9**	**1.493**	**13.32**	**18.66**	**22.93**
	Equiv. Refl.: (-1,-4,-1), (-4,5,-1), (-4,-1,1)								
**Quartz**	**(5,-1,-1)**	**6.775**	**77.84**	**7.855**	**8.9**	**1.493**	**13.32**	**18.66**	**22.93**
	Equiv. Refl.: (5,-4,1)								
**Quartz**	**(1,4,1)**	**6.775**	**77.84**	**7.685**	**8.9**	**1.493**	**13.32**	**18.66**	**22.93**
	Equiv. Refl.: (1,-5,-1), (4,1,-1), (4,-5,1)								
**Quartz**	**(-5,1,1)**	**6.775**	**77.84**	**7.682**	**8.9**	**1.493**	**13.32**	**18.66**	**22.93**
	Equiv. Refl.: (-5,4,-1)								
**Quartz**	**(3,-5,-2)**	**6.752**	**77**	**7.301**	**8**	**1.599**	**12.76**	**19.99**	**23.72**
**Quartz**	**(3,2,2)**	**6.752**	**77**	**7.3**	**8**	**1.599**	**12.76**	**19.99**	**23.72**
**Quartz**	**(-5,2,-2)**	**6.752**	**77**	**7.298**	**8**	**1.599**	**12.76**	**19.99**	**23.72**
	Equiv. Refl.: (-5,3,2)								
**Quartz**	**(2,3,-2)**	**6.752**	**77**	**7.297**	**8**	**1.599**	**12.76**	**19.99**	**23.72**
	Equiv. Refl.: (2,-5,2)								
**Quartz**	**(-3,5,2)**	**6.752**	**77**	**6.982**	**8**	**1.599**	**12.76**	**19.99**	**23.72**
	Equiv. Refl.: (-3,-2,-2)								
**Quartz**	**(5,-2,2)**	**6.752**	**77**	**6.98**	**8**	**1.599**	**12.76**	**19.99**	**23.72**
	Equiv. Refl.: (5,-3,-2), (-2,5,-2)								
**Quartz**	**(-2,-3,2)**	**6.752**	**77**	**6.979**	**8**	**1.599**	**12.76**	**19.99**	**23.72**
**Sapph**	**(2,3,-1)**	**6.575**	**71.58**	**5.813**	**7.1**	**2.308**	**16.34**	**28.85**	**33.15**
	Equiv. Refl.: (2,-5,1), (3,2,1), (3,-5,-1), (5,-2,1), (5,-3,-1), (-2,5,-1), (-2,-3,1), (-3,5,1), (-3,-2,-1) (-5,2,-1), (-5,3,1)								
**LiNbO3**	**(1,2,-13)**	**6.88**	**83.08**	**5.525**	**16.4**	**0.841**	**13.77**	**10.51**	**17.32**
	Equiv. Refl.: (1,-3,13), (2,1,13), (2,-3,-13), (3,-1,13), (3,-2,-13), (-1,3,-13), (-1,-2,13), (-2,3,13), (-2,-1,-13) (-3,1,-13), (-3,2,13)								
**Sapph**	**(1,2,11)**	**6.588**	**71.94**	**4.513**	**5.9**	**2.26**	**13.38**	**28.25**	**31.26**
	Equiv. Refl.: (1,-3,-11), (2,1,-11), (2,-3,11), (3,-1,-11), (3,-2,11), (-1,3,11), (-1,-2,-11), (-2,3,-11), (-2,-1,11) (-3,1,11), (-3,2,-11)								
**Quartz**	**(2,3,2)**	**6.752**	**77**	**2.495**	**4**	**1.599**	**6.35**	**19.99**	**20.98**
	Equiv. Refl.: (2,-5,-2)								
**Quartz**	**(-5,3,-2)**	**6.752**	**77**	**2.493**	**4**	**1.599**	**6.34**	**19.99**	**20.98**
**Quartz**	**(-5,2,2)**	**6.752**	**77**	**2.492**	**4**	**1.599**	**6.34**	**19.99**	**20.98**
**Quartz**	**(3,-5,2)**	**6.752**	**77**	**2.491**	**4**	**1.599**	**6.34**	**19.99**	**20.97**
**Quartz**	**(3,2,-2)**	**6.752**	**77**	**2.49**	**4**	**1.599**	**6.34**	**19.99**	**20.97**
**Quartz**	**(-2,-3,-2)**	**6.752**	**77**	**1.957**	**4**	**1.599**	**6.35**	**19.99**	**20.98**
**Quartz**	**(-2,5,2)**	**6.752**	**77**	**1.956**	**4**	**1.599**	**6.35**	**19.99**	**20.98**

Ei =	6.93	keV						ΔEg	ΔEt
Cryst	Refl	EB	ΘB	∫ IR dΘ	Width	Ei cotΘ	ΔE	2m, 50µm	2m, 50µm
	(h,k,l)	[keV]	[°]	[µrad]	[µrad]	[meV/µrad]	[meV]	[meV]	[meV]
**Quartz**	**(5,-3,2)**	**6.752**	**77**	**1.954**	**4**	**1.599**	**6.34**	**19.99**	**20.98**
**Quartz**	**(5,-2,-2)**	**6.752**	**77**	**1.953**	**4**	**1.599**	**6.34**	**19.99**	**20.98**
**Quartz**	**(-3,5,-2)**	**6.752**	**77**	**1.952**	**4**	**1.599**	**6.34**	**19.99**	**20.97**
	Equiv. Refl.: (-3,-2,2)								
**LiNbO3**	**(2,3,-7)**	**6.822**	**79.85**	**1.4**	**6.7**	**1.24**	**8.37**	**15.51**	**17.62**
	Equiv. Refl.: (2,-5,7), (3,2,7), (3,-5,-7), (5,-2,7), (5,-3,-7), (-2,5,-7), (-2,-3,7), (-3,5,7), (-3,-2,-7) (-5,2,-7), (-5,3,7)								
**Quartz**	**(1,4,0)**	**6.677**	**74.46**	**0.641**	**1.2**	**1.927**	**2.4**	**24.08**	**24.2**
	Equiv. Refl.: (1,-5,0), (4,1,0), (4,-5,0), (-5,1,0), (-5,4,0)								
**Quartz**	**(5,-1,0)**	**6.677**	**74.46**	**0.204**	**1.2**	**1.927**	**2.4**	**24.08**	**24.2**
	Equiv. Refl.: (5,-4,0), (-1,5,0), (-1,-4,0), (-4,5,0), (-4,-1,0)								
**Sapph**	**(2,3,2)**	**6.627**	**72.99**	**0.055**	**0.5**	**2.12**	**1.09**	**26.51**	**26.53**
	Equiv. Refl.: (2,-5,-2), (3,2,-2), (3,-5,2), (5,-2,-2), (5,-3,2), (-2,5,2), (-2,-3,-2), (-3,5,-2), (-3,-2,2) (-5,2,2), (-5,3,-2)								

Ei =	6.915	keV						ΔEg	ΔEt
**Cryst**	**Refl**	**EB**	**ΘB**	**∫IR dΘ**	**Width**	**Ei cotΘ**	**ΔE**	**2m, 50µm**	**2m, 50µm**
	**(h,k,l)**	**[keV]**	**[°]**	**[µrad]**	**[µrad]**	**[meV/µrad]**	**[meV]**	**[meV]**	**[meV]**
**Si**	**(1,3,5)**	**6.753**	**77.57**	**27.3573**	**26.36**	**1.5243**	**40.18**	**19.05**	**44.47**
	Equiv. Refl.: (1,5,3), (3,1,5), (3,5,1), (5,1,3), (5,3,1)								
**LiNbO3**	**(-4,5,-6)**	**6.914**	**88.96**	**700.879**	**678.6**	**0.126**	**85.42**	**1.57**	**85.43**
	Equiv. Refl.: (-5,4,-6)								
**LiNbO3**	**(4,1,-6)**	**6.914**	**88.96**	**700.878**	**678.6**	**0.126**	**85.42**	**1.57**	**85.43**
	Equiv. Refl.: (-1,-4,-6)								
**LiNbO3**	**(1,-5,-6)**	**6.914**	**88.96**	**700.877**	**678.6**	**0.126**	**85.42**	**1.57**	**85.43**
	Equiv. Refl.: (5,-1,-6)								
**LiNbO3**	**(1,4,-6)**	**6.914**	**88.96**	**692.442**	**668.4**	**0.126**	**84.14**	**1.57**	**84.16**
	Equiv. Refl.: (-1,5,-6), (-4,-1,-6), (-5,1,-6)								
**LiNbO3**	**(4,-5,-6)**	**6.914**	**88.96**	**692.439**	**668.4**	**0.126**	**84.14**	**1.57**	**84.16**
	Equiv. Refl.: (5,-4,-6)								
**LiNbO3**	**(4,-5,6)**	**6.914**	**88.96**	**658.908**	**678.6**	**0.126**	**85.42**	**1.57**	**85.43**
	Equiv. Refl.: (5,-4,6)								
**LiNbO3**	**(1,4,6)**	**6.914**	**88.96**	**658.907**	**678.6**	**0.126**	**85.42**	**1.57**	**85.43**
	Equiv. Refl.: (-1,5,6), (-4,-1,6), (-5,1,6)								
**LiNbO3**	**(1,-5,6)**	**6.914**	**88.96**	**642.628**	**668.4**	**0.126**	**84.14**	**1.57**	**84.16**
	Equiv. Refl.: (4,1,6), (5,-1,6), (-1,-4,6)								
**LiNbO3**	**(-4,5,6)**	**6.914**	**88.96**	**642.625**	**668.4**	**0.126**	**84.14**	**1.57**	**84.16**
	Equiv. Refl.: (-5,4,6)								
**Quartz**	**(-1,3,5)**	**6.91**	**87.87**	**116.583**	**107.9**	**0.258**	**27.8**	**3.22**	**27.99**
**Quartz**	**(-1,-2,-5)**	**6.91**	**87.87**	**116.574**	**107.9**	**0.258**	**27.8**	**3.22**	**27.99**
**Quartz**	**(-2,3,-5)**	**6.91**	**87.87**	**116.518**	**107.9**	**0.258**	**27.79**	**3.22**	**27.98**
**Quartz**	**(-2,-1,5)**	**6.91**	**87.87**	**116.509**	**107.9**	**0.258**	**27.79**	**3.22**	**27.98**
**Quartz**	**(3,-1,-5)**	**6.91**	**87.87**	**116.505**	**107.9**	**0.258**	**27.79**	**3.22**	**27.97**
**Quartz**	**(3,-2,5)**	**6.91**	**87.87**	**116.494**	**107.8**	**0.258**	**27.79**	**3.22**	**27.97**
**Quartz**	**(1,-3,-5)**	**6.91**	**87.87**	**112.903**	**107.9**	**0.258**	**27.8**	**3.22**	**27.99**
**Quartz**	**(1,2,5)**	**6.91**	**87.87**	**112.894**	**107.9**	**0.258**	**27.8**	**3.22**	**27.99**
**Quartz**	**(2,-3,5)**	**6.91**	**87.87**	**112.87**	**107.9**	**0.258**	**27.79**	**3.22**	**27.98**
**Quartz**	**(2,1,-5)**	**6.91**	**87.87**	**112.86**	**107.9**	**0.258**	**27.79**	**3.22**	**27.98**
**Quartz**	**(-3,1,5)**	**6.91**	**87.87**	**112.853**	**107.9**	**0.258**	**27.79**	**3.22**	**27.97**
**Quartz**	**(-3,2,-5)**	**6.91**	**87.87**	**112.842**	**107.8**	**0.258**	**27.79**	**3.22**	**27.97**
**LiNbO3**	**(0,-2,14)**	**6.85**	**82.16**	**92.984**	**89.8**	**0.952**	**85.47**	**11.9**	**86.3**
	Equiv. Refl.: (2,0,14)								
**LiNbO3**	**(-2,2,14)**	**6.85**	**82.16**	**92.983**	**89.8**	**0.952**	**85.47**	**11.9**	**86.3**
**LiNbO3**	**(0,2,-14)**	**6.85**	**82.16**	**86.249**	**89.8**	**0.952**	**85.47**	**11.9**	**86.3**
	Equiv. Refl.: (2,-2,-14), (-2,0,-14)								
**LiNbO3**	**(0,-3,-12)**	**6.797**	**79.39**	**73.098**	**67.6**	**1.295**	**87.52**	**16.19**	**89.01**
	Equiv. Refl.: (3,0,-12), (-3,3,-12)								
**LiNbO3**	**(0,3,-12)**	**6.797**	**79.39**	**71.772**	**66.4**	**1.295**	**85.96**	**16.19**	**87.48**
	Equiv. Refl.: (-3,0,-12)								
**LiNbO3**	**(3,-3,-12)**	**6.797**	**79.39**	**71.771**	**66.4**	**1.295**	**85.96**	**16.19**	**87.48**
**LiNbO3**	**(0,3,12)**	**6.797**	**79.39**	**61.885**	**67.6**	**1.295**	**87.52**	**16.19**	**89.01**
	Equiv. Refl.: (3,-3,12), (-3,0,12)								
**Sapph**	**(1,4,0)**	**6.894**	**85.55**	**61.487**	**59.4**	**0.539**	**31.98**	**6.73**	**32.68**
	Equiv. Refl.: (1,-5,0), (4,1,0), (4,-5,0), (5,-1,0), (5,-4,0), (-1,5,0), (-1,-4,0), (-4,5,0), (-4,-1,0) (-5,1,0), (-5,4,0)								

Ei =	6.915	keV						ΔEg	ΔEt
Cryst	Refl	EB	ΘB	∫ IR dΘ	Width	Ei cotΘ	ΔE	2m, 50µm	2m, 50µm
	(h,k,l)	[keV]	[°]	[µrad]	[µrad]	[meV/µrad]	[meV]	[meV]	[meV]
**LiNbO3**	**(0,-3,12)**	**6.797**	**79.39**	**60.29**	**66.4**	**1.295**	**85.96**	**16.19**	**87.48**
	Equiv. Refl.: (3,0,12)								
**LiNbO3**	**(-3,3,12)**	**6.797**	**79.39**	**60.289**	**66.4**	**1.295**	**85.96**	**16.19**	**87.48**
**LiNbO3**	**(1,-4,-10)**	**6.718**	**76.29**	**58.349**	**55.6**	**1.687**	**93.78**	**21.09**	**96.12**
	Equiv. Refl.: (3,1,-10), (4,-1,-10), (-1,-3,-10), (-3,4,-10), (-4,3,-10)								
**Sapph**	**(2,3,-4)**	**6.83**	**81**	**57.676**	**51.1**	**1.095**	**55.92**	**13.69**	**57.57**
	Equiv. Refl.: (2,-5,4), (3,2,4), (3,-5,-4), (5,-2,4), (5,-3,-4), (-2,5,-4), (-2,-3,4), (-3,5,4), (-3,-2,-4) (-5,2,-4), (-5,3,4)								
**LiNbO3**	**(1,3,10)**	**6.718**	**76.29**	**53.742**	**55.6**	**1.687**	**93.78**	**21.09**	**96.12**
	Equiv. Refl.: (3,-4,10), (4,-3,10), (-1,4,10), (-3,-1,10), (-4,1,10)								
**Sapph**	**(0,1,14)**	**6.849**	**82.1**	**46.148**	**42.7**	**0.96**	**40.99**	**12**	**42.71**
	Equiv. Refl.: (0,-1,-14), (1,0,-14), (1,-1,14), (-1,0,14), (-1,1,-14)								
**Quartz**	**(-2,-1,-5)**	**6.91**	**87.87**	**45.657**	**48.1**	**0.258**	**12.39**	**3.22**	**12.81**
**Quartz**	**(-2,3,5)**	**6.91**	**87.87**	**45.652**	**48.1**	**0.258**	**12.39**	**3.22**	**12.8**
**Quartz**	**(3,-2,-5)**	**6.91**	**87.87**	**45.647**	**48.1**	**0.258**	**12.39**	**3.22**	**12.8**
**Quartz**	**(3,-1,5)**	**6.91**	**87.87**	**45.623**	**48.1**	**0.258**	**12.39**	**3.22**	**12.8**
**Quartz**	**(-1,3,-5)**	**6.91**	**87.87**	**45.606**	**48.1**	**0.258**	**12.38**	**3.22**	**12.8**
**Quartz**	**(-1,-2,5)**	**6.91**	**87.87**	**45.594**	**48.1**	**0.258**	**12.38**	**3.22**	**12.79**
**LiNbO3**	**(0,4,8)**	**6.613**	**73.01**	**42.627**	**40.6**	**2.113**	**85.75**	**26.41**	**89.73**
	Equiv. Refl.: (4,-4,8), (-4,0,8)								
**Quartz**	**(0,0,-6)**	**6.882**	**84.41**	**37.741**	**36.2**	**0.677**	**24.52**	**8.46**	**25.94**
**Quartz**	**(0,0,6)**	**6.882**	**84.41**	**37.74**	**36.2**	**0.677**	**24.52**	**8.46**	**25.94**
**LiNbO3**	**(0,-4,-8)**	**6.613**	**73.01**	**37.129**	**40.6**	**2.113**	**85.75**	**26.41**	**89.73**
	Equiv. Refl.: (4,0,-8), (-4,4,-8)								
**Quartz**	**(2,1,5)**	**6.91**	**87.87**	**35.619**	**48.1**	**0.258**	**12.39**	**3.22**	**12.81**
**Quartz**	**(2,-3,-5)**	**6.91**	**87.87**	**35.616**	**48.1**	**0.258**	**12.39**	**3.22**	**12.8**
**Quartz**	**(-3,2,5)**	**6.91**	**87.87**	**35.611**	**48.1**	**0.258**	**12.39**	**3.22**	**12.8**
**Quartz**	**(-3,1,-5)**	**6.91**	**87.87**	**35.586**	**48.1**	**0.258**	**12.39**	**3.22**	**12.8**
**Quartz**	**(1,-3,5)**	**6.91**	**87.87**	**35.571**	**48.1**	**0.258**	**12.38**	**3.22**	**12.8**
**Quartz**	**(1,2,-5)**	**6.91**	**87.87**	**35.562**	**48.1**	**0.258**	**12.38**	**3.22**	**12.79**
**Quartz**	**(0,4,-3)**	**6.768**	**78.16**	**30.22**	**27**	**1.449**	**39.17**	**18.11**	**43.15**
	Equiv. Refl.: (0,-4,3)								
**Quartz**	**(-4,0,-3)**	**6.768**	**78.16**	**30.209**	**27**	**1.449**	**39.16**	**18.11**	**43.14**
**Quartz**	**(4,0,3)**	**6.768**	**78.16**	**30.208**	**27**	**1.449**	**39.16**	**18.11**	**43.14**
	Equiv. Refl.: (-4,4,3)								
**Quartz**	**(4,-4,-3)**	**6.768**	**78.16**	**30.207**	**27**	**1.449**	**39.15**	**18.11**	**43.14**
**Quartz**	**(4,1,1)**	**6.775**	**78.43**	**27.909**	**24.9**	**1.415**	**35.18**	**17.69**	**39.38**
	Equiv. Refl.: (4,-5,-1)								
**Quartz**	**(1,4,-1)**	**6.775**	**78.43**	**27.906**	**24.9**	**1.415**	**35.17**	**17.69**	**39.37**
**Quartz**	**(1,-5,1)**	**6.775**	**78.43**	**27.905**	**24.9**	**1.415**	**35.17**	**17.69**	**39.37**
**Quartz**	**(-5,1,-1)**	**6.775**	**78.43**	**27.904**	**24.9**	**1.415**	**35.17**	**17.69**	**39.37**
	Equiv. Refl.: (-5,4,1)								
**Quartz**	**(-4,5,1)**	**6.775**	**78.43**	**26.745**	**24.9**	**1.415**	**35.18**	**17.69**	**39.38**
	Equiv. Refl.: (-4,-1,-1)								
**Quartz**	**(-1,-4,1)**	**6.775**	**78.43**	**26.743**	**24.9**	**1.415**	**35.17**	**17.69**	**39.37**
**Quartz**	**(-1,5,-1)**	**6.775**	**78.43**	**26.742**	**24.9**	**1.415**	**35.17**	**17.69**	**39.37**
**Quartz**	**(5,-1,1)**	**6.775**	**78.43**	**26.74**	**24.9**	**1.415**	**35.17**	**17.69**	**39.37**
	Equiv. Refl.: (5,-4,-1)								
**Quartz**	**(2,-4,-4)**	**6.821**	**80.53**	**25.192**	**23.4**	**1.153**	**26.95**	**14.41**	**30.56**
**Quartz**	**(-4,2,4)**	**6.821**	**80.53**	**25.191**	**23.4**	**1.153**	**26.95**	**14.41**	**30.56**
**Quartz**	**(-4,2,-4)**	**6.821**	**80.53**	**25.189**	**23.4**	**1.153**	**26.94**	**14.41**	**30.56**

Ei =	6.915	keV						ΔEg	ΔEt
Cryst	Refl	EB	ΘB	∫ IR dΘ	Width	Ei cotΘ	ΔE	2m, 50µm	2m, 50µm
	(h,k,l)	[keV]	[°]	[µrad]	[µrad]	[meV/µrad]	[meV]	[meV]	[meV]
**Quartz**	**(2,2,4)**	**6.821**	**80.53**	**25.188**	**23.4**	**1.153**	**26.94**	**14.41**	**30.56**
**Quartz**	**(2,2,-4)**	**6.821**	**80.53**	**25.187**	**23.4**	**1.153**	**26.94**	**14.41**	**30.55**
	Equiv. Refl.: (2,-4,4)								
**Sapph**	**(2,2,9)**	**6.754**	**77.6**	**24.978**	**23.8**	**1.521**	**36.26**	**19.01**	**40.94**
	Equiv. Refl.: (2,2,-9), (2,-4,9), (2,-4,-9), (4,-2,9), (4,-2,-9), (-2,4,9), (-2,4,-9), (-2,-2,9), (-2,-2,-9) (-4,2,9), (-4,2,-9)								
**Quartz**	**(-2,4,4)**	**6.821**	**80.53**	**24.127**	**23.4**	**1.153**	**26.95**	**14.41**	**30.56**
**Quartz**	**(4,-2,-4)**	**6.821**	**80.53**	**24.125**	**23.4**	**1.153**	**26.95**	**14.41**	**30.56**
**Quartz**	**(4,-2,4)**	**6.821**	**80.53**	**24.123**	**23.4**	**1.153**	**26.94**	**14.41**	**30.56**
**Quartz**	**(-2,-2,-4)**	**6.821**	**80.53**	**24.122**	**23.4**	**1.153**	**26.94**	**14.41**	**30.56**
**Quartz**	**(-2,4,-4)**	**6.821**	**80.53**	**24.12**	**23.4**	**1.153**	**26.94**	**14.41**	**30.55**
	Equiv. Refl.: (-2,-2,4)								
**Quartz**	**(4,-4,3)**	**6.768**	**78.16**	**18.128**	**17.5**	**1.449**	**25.36**	**18.11**	**31.16**
**Quartz**	**(4,0,-3)**	**6.768**	**78.16**	**18.127**	**17.5**	**1.449**	**25.36**	**18.11**	**31.16**
**Quartz**	**(-4,4,-3)**	**6.768**	**78.16**	**18.126**	**17.5**	**1.449**	**25.36**	**18.11**	**31.16**
**Quartz**	**(-4,0,3)**	**6.768**	**78.16**	**18.125**	**17.5**	**1.449**	**25.36**	**18.11**	**31.16**
**Quartz**	**(0,4,3)**	**6.768**	**78.16**	**18.12**	**17.5**	**1.449**	**25.35**	**18.11**	**31.16**
	Equiv. Refl.: (0,-4,-3)								
**Sapph**	**(1,3,-8)**	**6.633**	**73.59**	**17.427**	**16.9**	**2.036**	**34.34**	**25.45**	**42.74**
	Equiv. Refl.: (1,-4,8), (3,1,8), (3,-4,-8), (4,-1,8), (4,-3,-8), (-1,4,-8), (-1,-3,8), (-3,4,8), (-3,-1,-8) (-4,1,-8), (-4,3,8)								
**Quartz**	**(-4,5,-1)**	**6.775**	**78.43**	**8.26**	**9.4**	**1.415**	**13.29**	**17.69**	**22.13**
	Equiv. Refl.: (-4,-1,1)								
**Quartz**	**(-1,5,1)**	**6.775**	**78.43**	**8.259**	**9.4**	**1.415**	**13.29**	**17.69**	**22.13**
	Equiv. Refl.: (-1,-4,-1)								
**Quartz**	**(5,-1,-1)**	**6.775**	**78.43**	**8.257**	**9.4**	**1.415**	**13.29**	**17.69**	**22.13**
	Equiv. Refl.: (5,-4,1)								
**Quartz**	**(1,4,1)**	**6.775**	**78.43**	**8.077**	**9.4**	**1.415**	**13.29**	**17.69**	**22.13**
	Equiv. Refl.: (4,1,-1), (4,-5,1)								
**Quartz**	**(1,-5,-1)**	**6.775**	**78.43**	**8.076**	**9.4**	**1.415**	**13.29**	**17.69**	**22.13**
**Quartz**	**(-5,1,1)**	**6.775**	**78.43**	**8.074**	**9.4**	**1.415**	**13.29**	**17.69**	**22.13**
	Equiv. Refl.: (-5,4,-1)								
**Quartz**	**(3,-5,-2)**	**6.752**	**77.55**	**7.626**	**8.3**	**1.526**	**12.74**	**19.08**	**22.94**
**Quartz**	**(3,2,2)**	**6.752**	**77.55**	**7.625**	**8.3**	**1.526**	**12.74**	**19.08**	**22.94**
**Quartz**	**(-5,2,-2)**	**6.752**	**77.55**	**7.623**	**8.3**	**1.526**	**12.73**	**19.08**	**22.94**
	Equiv. Refl.: (-5,3,2)								
**Quartz**	**(2,3,-2)**	**6.752**	**77.55**	**7.622**	**8.3**	**1.526**	**12.73**	**19.08**	**22.94**
	Equiv. Refl.: (2,-5,2)								
**Quartz**	**(-3,5,2)**	**6.752**	**77.55**	**7.292**	**8.3**	**1.526**	**12.74**	**19.08**	**22.94**
	Equiv. Refl.: (-3,-2,-2)								
**Quartz**	**(5,-2,2)**	**6.752**	**77.55**	**7.29**	**8.3**	**1.526**	**12.73**	**19.08**	**22.94**
	Equiv. Refl.: (5,-3,-2)								
**Quartz**	**(-2,5,-2)**	**6.752**	**77.55**	**7.289**	**8.3**	**1.526**	**12.73**	**19.08**	**22.94**
	Equiv. Refl.: (-2,-3,2)								
**LiNbO3**	**(1,2,-13)**	**6.88**	**84.2**	**6.576**	**19.6**	**0.703**	**13.74**	**8.78**	**16.31**
	Equiv. Refl.: (1,-3,13), (2,1,13), (3,-1,13), (-1,3,-13), (-1,-2,13), (-2,-1,-13), (-3,1,-13)								
**LiNbO3**	**(2,-3,-13)**	**6.88**	**84.2**	**6.575**	**19.6**	**0.703**	**13.74**	**8.78**	**16.31**
	Equiv. Refl.: (3,-2,-13), (-2,3,13), (-3,2,13)								

Ei =<br>Cryst	6.915<br>Refl<br>(h,k,l)	keV<br>EB<br>[keV]	ΘB<br>[°]	∫ IR dΘ<br>[µrad]	Width<br>[µrad]	Ei cotΘ<br>[meV/µrad]	ΔE<br>[meV]	ΔEg<br>2m, 50µm<br>[meV]	ΔEt<br>2m, 50µm<br>[meV]
**Sapph**	**(2,3,-1)**	**6.575**	**71.96**	**5.932**	**7.2**	**2.253**	**16.3**	**28.16**	**32.54**
	Equiv. Refl.: (2,-5,1), (3,2,1), (3,-5,-1), (5,-2,1), (5,-3,-1), (-2,5,-1), (-2,-3,1), (-3,5,1), (-3,-2,-1) (-5,2,-1), (-5,3,1)								
**Sapph**	**(1,2,11)**	**6.588**	**72.32**	**4.608**	**6.1**	**2.204**	**13.35**	**27.55**	**30.62**
	Equiv. Refl.: (1,-3,-11), (2,1,-11), (2,-3,11), (3,-1,-11), (3,-2,11), (-1,3,11), (-1,-2,-11), (-2,3,-11), (-2,-1,11) (-3,1,11), (-3,2,-11)								
**LiNbO3**	**(1,4,3)**	**6.512**	**70.34**	**3.237**	**7.9**	**2.471**	**19.58**	**30.89**	**36.57**
	Equiv. Refl.: (1,4,-3), (1,-5,3), (1,-5,-3), (4,1,3), (4,1,-3), (4,-5,3), (4,-5,-3), (5,-1,3), (5,-1,-3) (5,-4,3), (5,-4,-3), (-1,5,3), (-1,5,-3), (-1,-4,3), (-1,-4,-3), (-4,5,3), (-4,5,-3), (-4,-1,3) (-4,-1,-3), (-5,1,3), (-5,1,-3), (-5,4,3), (-5,4,-3)								
**Quartz**	**(2,3,2)**	**6.752**	**77.55**	**2.605**	**4.2**	**1.526**	**6.34**	**19.08**	**20.1**
	Equiv. Refl.: (2,-5,-2)								
**Quartz**	**(-5,3,-2)**	**6.752**	**77.55**	**2.602**	**4.1**	**1.526**	**6.33**	**19.08**	**20.1**
**Quartz**	**(-5,2,2)**	**6.752**	**77.55**	**2.601**	**4.1**	**1.526**	**6.33**	**19.08**	**20.1**
**Quartz**	**(3,2,-2)**	**6.752**	**77.55**	**2.6**	**4.1**	**1.526**	**6.33**	**19.08**	**20.1**
	Equiv. Refl.: (3,-5,2)								
**Quartz**	**(-2,5,2)**	**6.752**	**77.55**	**2.04**	**4.2**	**1.526**	**6.34**	**19.08**	**20.1**
	Equiv. Refl.: (-2,-3,-2)								
**Quartz**	**(5,-3,2)**	**6.752**	**77.55**	**2.038**	**4.1**	**1.526**	**6.33**	**19.08**	**20.1**
**Quartz**	**(5,-2,-2)**	**6.752**	**77.55**	**2.036**	**4.1**	**1.526**	**6.33**	**19.08**	**20.1**
	Equiv. Refl.: (-3,5,-2), (-3,-2,2)								
**LiNbO3**	**(2,3,-7)**	**6.822**	**80.57**	**1.504**	**7.3**	**1.148**	**8.35**	**14.35**	**16.61**
	Equiv. Refl.: (2,-5,7), (3,2,7), (3,-5,-7), (5,-2,7), (5,-3,-7), (-2,5,-7), (-2,-3,7), (-3,5,7), (-3,-2,-7) (-5,2,-7), (-5,3,7)								
**Quartz**	**(1,4,0)**	**6.677**	**74.92**	**0.66**	**1.3**	**1.864**	**2.39**	**23.3**	**23.42**
	Equiv. Refl.: (1,-5,0), (4,1,0), (4,-5,0), (-5,1,0), (-5,4,0)								
**Quartz**	**(5,-1,0)**	**6.677**	**74.92**	**0.209**	**1.3**	**1.864**	**2.39**	**23.3**	**23.42**
	Equiv. Refl.: (5,-4,0), (-1,5,0), (-1,-4,0), (-4,5,0), (-4,-1,0)								
**Sapph**	**(2,3,2)**	**6.627**	**73.4**	**0.056**	**0.5**	**2.062**	**1.09**	**25.77**	**25.79**
	Equiv. Refl.: (2,-5,-2), (3,2,-2), (3,-5,2), (5,-2,-2), (5,-3,2), (-2,5,2), (-2,-3,-2), (-3,5,-2), (-3,-2,2) (-5,2,2), (-5,3,-2)								

Ei =	7.649	keV							
Cryst	Refl (h,k,l)	EB [keV]	ΘB [°]	∫IR dΘ [µrad]	Width [µrad]	Ei cotΘ [meV/µrad]	ΔE [meV]	ΔEg 2m, 50µm [meV]	ΔEt 2m, 50µm [meV]
**Ge**	**(4,4,4)**	**7.592**	**82.99**	**96.0009**	**89.28**	**0.9408**	**84**	**11.76**	**84.82**
**Si**	**(0,2,6)**	**7.219**	**70.7**	**21.3457**	**18.94**	**2.6787**	**50.72**	**33.48**	**60.78**
	Equiv. Refl.: (0,6,2), (2,0,6), (2,6,0), (6,0,2), (6,2,0)								
**Si**	**(3,3,5)**	**7.485**	**78.11**	**20.8288**	**19.82**	**1.6102**	**31.91**	**20.13**	**37.73**
	Equiv. Refl.: (3,5,3), (5,3,3)								
**Quartz**	**(0,5,-2)**	**7.638**	**86.87**	**138.979**	**116.8**	**0.418**	**48.86**	**5.23**	**49.14**
	Equiv. Refl.: (0,-5,2)								
**Quartz**	**(5,0,2)**	**7.638**	**86.87**	**138.967**	**116.8**	**0.418**	**48.85**	**5.23**	**49.13**
	Equiv. Refl.: (5,-5,-2), (-5,0,-2), (-5,5,2)								
**Quartz**	**(2,2,5)**	**7.64**	**87.17**	**74.987**	**67.7**	**0.378**	**25.55**	**4.72**	**25.98**
	Equiv. Refl.: (2,-4,-5)								
**Quartz**	**(2,2,-5)**	**7.64**	**87.17**	**74.976**	**67.6**	**0.378**	**25.54**	**4.72**	**25.97**
**Quartz**	**(2,-4,5)**	**7.64**	**87.17**	**74.964**	**67.6**	**0.378**	**25.54**	**4.72**	**25.97**
**Quartz**	**(-4,2,5)**	**7.64**	**87.17**	**74.957**	**67.6**	**0.378**	**25.54**	**4.72**	**25.97**
**Quartz**	**(-4,2,-5)**	**7.64**	**87.17**	**74.95**	**67.6**	**0.378**	**25.53**	**4.72**	**25.97**
**Quartz**	**(-2,4,5)**	**7.64**	**87.17**	**73.059**	**67.7**	**0.378**	**25.55**	**4.72**	**25.98**
	Equiv. Refl.: (-2,-2,-5)								
**Quartz**	**(-2,-2,5)**	**7.64**	**87.17**	**73.048**	**67.6**	**0.378**	**25.54**	**4.72**	**25.97**
**Quartz**	**(-2,4,-5)**	**7.64**	**87.17**	**73.034**	**67.6**	**0.378**	**25.54**	**4.72**	**25.97**
**Quartz**	**(4,-2,-5)**	**7.64**	**87.17**	**73.026**	**67.6**	**0.378**	**25.54**	**4.72**	**25.97**
**Quartz**	**(4,-2,5)**	**7.64**	**87.17**	**73.02**	**67.6**	**0.378**	**25.53**	**4.72**	**25.97**
**LiNbO3**	**(2,4,4)**	**7.572**	**81.87**	**57.477**	**55.9**	**1.093**	**61.15**	**13.66**	**62.66**
	Equiv. Refl.: (4,-6,4), (6,-4,4), (-2,6,4), (-4,-2,4), (-6,2,4)								
**LiNbO3**	**(2,-6,-4)**	**7.572**	**81.87**	**52.622**	**55.9**	**1.093**	**61.15**	**13.66**	**62.66**
	Equiv. Refl.: (4,2,-4), (6,-2,-4), (-2,-4,-4), (-4,6,-4), (-6,4,-4)								
**LiNbO3**	**(2,-5,10)**	**7.532**	**79.97**	**47.124**	**45.8**	**1.353**	**61.92**	**16.91**	**64.19**
	Equiv. Refl.: (3,2,10), (5,-2,10), (-2,-3,10), (-3,5,10), (-5,3,10)								
**Sapph**	**(1,1,15)**	**7.619**	**84.91**	**46.14**	**43.2**	**0.681**	**29.42**	**8.52**	**30.63**
	Equiv. Refl.: (1,1,-15), (1,-2,15), (1,-2,-15), (2,-1,15), (2,-1,-15), (-1,2,15), (-1,2,-15), (-1,-1,15), (-1,-1,-15) (-2,1,15), (-2,1,-15)								
**LiNbO3**	**(2,3,-10)**	**7.532**	**79.97**	**42.992**	**45.8**	**1.353**	**61.92**	**16.91**	**64.19**
	Equiv. Refl.: (3,-5,-10), (5,-3,-10), (-2,5,-10), (-3,-2,-10), (-5,2,-10)								
**LiNbO3**	**(2,4,-2)**	**7.412**	**75.7**	**41.754**	**40.5**	**1.95**	**78.99**	**24.38**	**82.67**
	Equiv. Refl.: (4,-6,-2), (6,-4,-2), (-2,6,-2), (-4,-2,-2), (-6,2,-2)								
**LiNbO3**	**(2,-6,2)**	**7.412**	**75.7**	**41.556**	**40.5**	**1.95**	**78.99**	**24.38**	**82.67**
	Equiv. Refl.: (4,2,2), (6,-2,2), (-2,-4,2), (-4,6,2), (-6,4,2)								
**LiNbO3**	**(3,3,0)**	**7.225**	**70.84**	**36.813**	**35.1**	**2.658**	**93.21**	**33.23**	**98.95**
	Equiv. Refl.: (3,-6,0), (6,-3,0), (-3,6,0), (-3,-3,0), (-6,3,0)								
**Quartz**	**(2,-2,6)**	**7.474**	**77.71**	**32.153**	**27.2**	**1.667**	**45.36**	**20.84**	**49.92**
**Quartz**	**(2,0,-6)**	**7.474**	**77.71**	**32.151**	**27.2**	**1.667**	**45.36**	**20.84**	**49.92**
	Equiv. Refl.: (-2,0,6), (-2,2,-6)								
**Quartz**	**(0,2,6)**	**7.474**	**77.71**	**32.141**	**27.2**	**1.667**	**45.35**	**20.84**	**49.9**
	Equiv. Refl.: (0,-2,-6)								
**LiNbO3**	**(1,-3,-14)**	**7.261**	**71.68**	**31.975**	**31.3**	**2.532**	**79.15**	**31.65**	**85.24**
	Equiv. Refl.: (2,1,-14), (3,-1,-14), (-1,-2,-14), (-2,3,-14), (-3,2,-14)								
**LiNbO3**	**(1,2,14)**	**7.261**	**71.68**	**31.783**	**31.3**	**2.532**	**79.15**	**31.65**	**85.24**
	Equiv. Refl.: (2,-3,14), (3,-2,14), (-1,3,14), (-2,-1,14), (-3,1,14)								
**Quartz**	**(4,1,-3)**	**7.511**	**79.11**	**31.275**	**26.9**	**1.471**	**39.54**	**18.39**	**43.6**

Ei =	7.649	keV						ΔEg	ΔEt
**Cryst**	**Refl**	**EB**	**ΘB**	**∫ IR dΘ**	**Width**	**Ei cotΘ**	**ΔE**	**2m, 50µm**	**2m, 50µm**
	**(h,k,l)**	**[keV]**	**[°]**	**[µrad]**	**[µrad]**	**[meV/µrad]**	**[meV]**	**[meV]**	**[meV]**
**Quartz**	**(4,-5,3)**	**7.511**	**79.11**	**31.274**	**26.9**	**1.471**	**39.53**	**18.39**	**43.6**
**Sapph**	**(1,4,6)**	**7.465**	**77.42**	**31.27**	**27.3**	**1.708**	**46.61**	**21.34**	**51.27**
	Equiv. Refl.: (1,4,-6), (1,-5,6), (1,-5,-6), (4,1,6), (4,1,-6), (4,-5,6), (4,-5,-6), (5,-1,6), (5,-1,-6) (5,-4,6), (5,-4,-6), (-1,5,6), (-1,5,-6), (-1,-4,6), (-1,-4,-6), (-4,5,6), (-4,5,-6), (-4,-1,6) (-4,-1,-6), (-5,1,6), (-5,1,-6), (-5,4,6), (-5,4,-6)								
**Quartz**	**(1,4,3)**	**7.511**	**79.11**	**31.269**	**26.9**	**1.471**	**39.53**	**18.39**	**43.6**
**Quartz**	**(1,-5,-3)**	**7.511**	**79.11**	**31.268**	**26.9**	**1.471**	**39.53**	**18.39**	**43.6**
**Quartz**	**(-5,1,3)**	**7.511**	**79.11**	**31.263**	**26.9**	**1.471**	**39.52**	**18.39**	**43.59**
	Equiv. Refl.: (-5,4,-3)								
**Quartz**	**(-4,-1,3)**	**7.511**	**79.11**	**31.238**	**26.9**	**1.471**	**39.54**	**18.39**	**43.6**
**Quartz**	**(-4,5,-3)**	**7.511**	**79.11**	**31.236**	**26.9**	**1.471**	**39.53**	**18.39**	**43.6**
**Quartz**	**(-1,-4,-3)**	**7.511**	**79.11**	**31.232**	**26.9**	**1.471**	**39.53**	**18.39**	**43.6**
**Quartz**	**(-1,5,3)**	**7.511**	**79.11**	**31.23**	**26.9**	**1.471**	**39.53**	**18.39**	**43.6**
**Quartz**	**(5,-1,-3)**	**7.511**	**79.11**	**31.226**	**26.9**	**1.471**	**39.52**	**18.39**	**43.59**
	Equiv. Refl.: (5,-4,3)								
**LiNbO3**	**(0,-1,16)**	**7.289**	**72.34**	**29.359**	**28.9**	**2.435**	**70.39**	**30.43**	**76.69**
	Equiv. Refl.: (1,0,16), (-1,1,16)								
**LiNbO3**	**(0,1,-16)**	**7.289**	**72.34**	**28.356**	**28.9**	**2.435**	**70.39**	**30.43**	**76.69**
	Equiv. Refl.: (1,-1,-16), (-1,0,-16)								
**LiNbO3**	**(2,2,12)**	**7.211**	**70.51**	**27.898**	**26.6**	**2.707**	**72.07**	**33.83**	**79.61**
	Equiv. Refl.: (2,-4,12), (4,-2,12), (-2,-2,12)								
**LiNbO3**	**(-2,4,12)**	**7.211**	**70.51**	**27.897**	**26.6**	**2.707**	**72.07**	**33.83**	**79.61**
	Equiv. Refl.: (-4,2,12)								
**LiNbO3**	**(2,2,-12)**	**7.211**	**70.51**	**25.261**	**26.6**	**2.707**	**72.07**	**33.83**	**79.61**
	Equiv. Refl.: (2,-4,-12), (4,-2,-12), (-2,4,-12), (-2,-2,-12), (-4,2,-12)								
**Quartz**	**(6,-3,0)**	**7.571**	**81.8**	**24.959**	**22.7**	**1.103**	**25.05**	**13.78**	**28.59**
	Equiv. Refl.: (-3,6,0), (-3,-3,0)								
**Quartz**	**(3,3,0)**	**7.571**	**81.8**	**24.796**	**22.7**	**1.103**	**25.05**	**13.78**	**28.59**
	Equiv. Refl.: (3,-6,0), (-6,3,0)								
**Sapph**	**(1,3,10)**	**7.225**	**70.84**	**24.485**	**21**	**2.658**	**55.94**	**33.23**	**65.06**
	Equiv. Refl.: (1,-4,-10), (3,1,-10), (3,-4,10), (4,-1,-10), (4,-3,10), (-1,4,10), (-1,-3,-10), (-3,4,-10), (-3,-1,10) (-4,1,10), (-4,3,-10)								
**Sapph**	**(0,2,-14)**	**7.328**	**73.35**	**23.78**	**20.8**	**2.288**	**47.56**	**28.6**	**55.49**
	Equiv. Refl.: (0,-2,14), (2,0,14), (2,-2,-14), (-2,0,-14), (-2,2,14)								
**Sapph**	**(0,3,12)**	**7.292**	**72.43**	**20.403**	**18.1**	**2.422**	**43.87**	**30.28**	**53.3**
	Equiv. Refl.: (0,3,-12), (0,-3,12), (0,-3,-12), (3,0,12), (3,0,-12), (3,-3,12), (3,-3,-12), (-3,0,12), (-3,0,-12) (-3,3,12), (-3,3,-12)								
**Quartz**	**(-4,0,4)**	**7.417**	**75.86**	**7.686**	**7.7**	**1.927**	**14.86**	**24.09**	**28.3**
**Quartz**	**(4,0,-4)**	**7.417**	**75.86**	**7.685**	**7.7**	**1.927**	**14.86**	**24.09**	**28.3**
	Equiv. Refl.: (4,-4,4), (-4,4,-4)								
**Quartz**	**(0,4,4)**	**7.417**	**75.86**	**7.669**	**7.7**	**1.927**	**14.84**	**24.09**	**28.29**
	Equiv. Refl.: (0,-4,-4)								
**Quartz**	**(2,0,6)**	**7.474**	**77.71**	**7.517**	**7.6**	**1.667**	**12.71**	**20.84**	**24.4**
**Quartz**	**(2,-2,-6)**	**7.474**	**77.71**	**7.516**	**7.6**	**1.667**	**12.7**	**20.84**	**24.4**
**Quartz**	**(-2,0,-6)**	**7.474**	**77.71**	**7.513**	**7.6**	**1.667**	**12.71**	**20.84**	**24.4**
**Quartz**	**(0,5,2)**	**7.638**	**86.87**	**7.512**	**11.9**	**0.418**	**4.99**	**5.23**	**7.23**
	Equiv. Refl.: (0,-5,-2)								
**Quartz**	**(-2,2,6)**	**7.474**	**77.71**	**7.511**	**7.6**	**1.667**	**12.7**	**20.84**	**24.4**
**Quartz**	**(0,2,-6)**	**7.474**	**77.71**	**7.504**	**7.6**	**1.667**	**12.69**	**20.84**	**24.4**
	Equiv. Refl.: (0,-2,6)								

Ei =	7.649	keV						ΔEg	ΔEt
Cryst	Refl	EB	ΘB	∫ IR dΘ	Width	Ei cotΘ	ΔE	2m, 50μm	2m, 50μm
	(h,k,l)	[keV]	[°]	[μrad]	[μrad]	[meV/μrad]	[meV]	[meV]	[meV]
**Quartz**	**(5,0,-2)**	**7.638**	**86.87**	**7.486**	**11.9**	**0.418**	**4.98**	**5.23**	**7.22**
	Equiv. Refl.: (-5,0,2)								
**Quartz**	**(5,-5,2)**	**7.638**	**86.87**	**7.484**	**11.9**	**0.418**	**4.98**	**5.23**	**7.22**
**Quartz**	**(-5,5,-2)**	**7.638**	**86.87**	**7.483**	**11.9**	**0.418**	**4.98**	**5.23**	**7.22**
**Quartz**	**(4,0,4)**	**7.417**	**75.86**	**7.305**	**7.4**	**1.927**	**14.3**	**24.09**	**28.01**
	Equiv. Refl.: (-4,0,-4)								
**Quartz**	**(4,-4,-4)**	**7.417**	**75.86**	**7.303**	**7.4**	**1.927**	**14.3**	**24.09**	**28.01**
	Equiv. Refl.: (-4,4,4)								
**Quartz**	**(0,4,-4)**	**7.417**	**75.86**	**7.299**	**7.4**	**1.927**	**14.29**	**24.09**	**28.01**
	Equiv. Refl.: (0,-4,4)								
**LiNbO3**	**(2,4,1)**	**7.371**	**74.52**	**6.331**	**10.7**	**2.119**	**22.57**	**26.49**	**34.8**
	Equiv. Refl.: (2,-6,-1), (4,2,-1), (4,-6,1), (6,-2,-1), (6,-4,1), (-2,6,1), (-2,-4,-1), (-4,6,-1), (-4,-2,1) (-6,2,1), (-6,4,-1)								
**Quartz**	**(2,-1,-6)**	**7.33**	**73.4**	**6**	**6.1**	**2.28**	**13.81**	**28.5**	**31.68**
**Quartz**	**(2,-1,6)**	**7.33**	**73.4**	**5.998**	**6.1**	**2.28**	**13.81**	**28.5**	**31.67**
**Quartz**	**(-1,2,6)**	**7.33**	**73.4**	**5.994**	**6.1**	**2.28**	**13.8**	**28.5**	**31.67**
**Quartz**	**(-1,2,-6)**	**7.33**	**73.4**	**5.993**	**6.1**	**2.28**	**13.8**	**28.5**	**31.67**
	Equiv. Refl.: (-1,-1,6), (-1,-1,-6)								
**Quartz**	**(-2,1,6)**	**7.33**	**73.4**	**5.733**	**6.1**	**2.28**	**13.81**	**28.5**	**31.68**
**Quartz**	**(-2,1,-6)**	**7.33**	**73.4**	**5.73**	**6.1**	**2.28**	**13.81**	**28.5**	**31.67**
**Quartz**	**(1,1,6)**	**7.33**	**73.4**	**5.725**	**6.1**	**2.28**	**13.8**	**28.5**	**31.67**
	Equiv. Refl.: (1,-2,-6)								
**Quartz**	**(1,1,-6)**	**7.33**	**73.4**	**5.724**	**6.1**	**2.28**	**13.8**	**28.5**	**31.67**
	Equiv. Refl.: (1,-2,6)								
**Sapph**	**(2,3,-7)**	**7.36**	**74.19**	**4.884**	**5.7**	**2.166**	**12.35**	**27.07**	**29.76**
	Equiv. Refl.: (2,-5,7), (3,2,7), (3,-5,-7), (5,-2,7), (5,-3,-7), (-2,5,-7), (-2,-3,7), (-3,5,7), (-3,-2,-7) (-5,2,-7), (-5,3,7)								
**LiNbO3**	**(1,4,9)**	**7.537**	**80.16**	**4.17**	**10.2**	**1.326**	**13.54**	**16.58**	**21.4**
	Equiv. Refl.: (1,4,-9), (1,-5,9), (1,-5,-9), (4,1,9), (4,1,-9), (4,-5,9), (4,-5,-9), (5,-1,9), (5,-1,-9) (5,-4,9), (5,-4,-9), (-1,5,9), (-1,5,-9), (-1,-4,9), (-1,-4,-9), (-4,5,9), (-4,5,-9), (-4,-1,9) (-4,-1,-9), (-5,1,9), (-5,1,-9), (-5,4,9), (-5,4,-9)								
**Sapph**	**(2,3,8)**	**7.588**	**82.78**	**3.951**	**6.5**	**0.97**	**6.25**	**12.12**	**13.64**
	Equiv. Refl.: (2,-5,-8), (3,2,-8), (3,-5,8), (5,-2,-8), (5,-3,8), (-2,5,8), (-2,-3,-8), (-3,5,-8), (-3,-2,8) (-5,2,8), (-5,3,-8)								
**Sapph**	**(1,2,-13)**	**7.372**	**74.53**	**3.527**	**4.8**	**2.118**	**10.13**	**26.47**	**28.34**
	Equiv. Refl.: (1,-3,13), (2,1,13), (2,-3,-13), (3,-1,13), (3,-2,-13), (-1,3,-13), (-1,-2,13), (-2,3,13), (-2,-1,-13) (-3,1,-13), (-3,2,13)								
**Quartz**	**(3,-3,-5)**	**7.211**	**70.51**	**3.086**	**3.6**	**2.707**	**9.75**	**33.83**	**35.21**
**Quartz**	**(3,0,5)**	**7.211**	**70.51**	**3.085**	**3.6**	**2.707**	**9.75**	**33.83**	**35.21**
**Quartz**	**(0,3,-5)**	**7.211**	**70.51**	**3.083**	**3.6**	**2.707**	**9.75**	**33.83**	**35.21**
	Equiv. Refl.: (0,-3,5)								
**Quartz**	**(-3,0,-5)**	**7.211**	**70.51**	**3.082**	**3.6**	**2.707**	**9.75**	**33.83**	**35.21**
	Equiv. Refl.: (-3,3,5)								
**Sapph**	**(1,3,-11)**	**7.549**	**80.72**	**2.966**	**5.2**	**1.249**	**6.47**	**15.61**	**16.9**
	Equiv. Refl.: (1,-4,11), (3,1,11), (3,-4,-11), (4,-1,11), (4,-3,-11), (-1,4,-11), (-1,-3,11), (-3,4,11), (-3,-1,-11) (-4,1,-11), (-4,3,11)								
**Quartz**	**(-4,5,3)**	**7.511**	**79.11**	**2.892**	**4.1**	**1.471**	**6.05**	**18.39**	**19.36**
	Equiv. Refl.: (-4,-1,-3)								
**Quartz**	**(-1,5,-3)**	**7.511**	**79.11**	**2.89**	**4.1**	**1.471**	**6.04**	**18.39**	**19.36**

Ei =	7.649	keV						ΔEg	ΔEt
Cryst	Refl	EB	ΘB	∫ IR dΘ	Width	Ei cotΘ	ΔE	2m, 50µm	2m, 50µm
	(h,k,l)	[keV]	[°]	[µrad]	[µrad]	[meV/µrad]	[meV]	[meV]	[meV]
**Quartz**	**(5,-1,3)**	**7.511**	**79.11**	**2.889**	**4.1**	**1.471**	**6.04**	**18.39**	**19.36**
	Equiv. Refl.: (5,-4,-3)								
**Quartz**	**(-1,-4,3)**	**7.511**	**79.11**	**2.888**	**4.1**	**1.471**	**6.04**	**18.39**	**19.36**
**Quartz**	**(4,-5,-3)**	**7.511**	**79.11**	**2.394**	**4.1**	**1.471**	**6.05**	**18.39**	**19.36**
**Quartz**	**(1,-5,3)**	**7.511**	**79.11**	**2.393**	**4.1**	**1.471**	**6.04**	**18.39**	**19.36**
	Equiv. Refl.: (4,1,3)								
**Quartz**	**(1,4,-3)**	**7.511**	**79.11**	**2.391**	**4.1**	**1.471**	**6.04**	**18.39**	**19.36**
	Equiv. Refl.: (-5,4,3)								
**Quartz**	**(-5,1,-3)**	**7.511**	**79.11**	**2.39**	**4.1**	**1.471**	**6.04**	**18.39**	**19.36**
**Quartz**	**(3,-5,-3)**	**7.223**	**70.79**	**1.499**	**2.3**	**2.665**	**6.18**	**33.31**	**33.88**
**Quartz**	**(2,3,-3)**	**7.223**	**70.79**	**1.498**	**2.3**	**2.665**	**6.17**	**33.31**	**33.88**
	Equiv. Refl.: (2,-5,3), (3,2,3)								
**Quartz**	**(-5,2,-3)**	**7.223**	**70.79**	**1.497**	**2.3**	**2.665**	**6.17**	**33.31**	**33.88**
	Equiv. Refl.: (-5,3,3)								
**Quartz**	**(-2,5,-3)**	**7.223**	**70.79**	**1.37**	**2.3**	**2.665**	**6.18**	**33.31**	**33.88**
	Equiv. Refl.: (-3,5,3)								
**Quartz**	**(5,-2,3)**	**7.223**	**70.79**	**1.369**	**2.3**	**2.665**	**6.17**	**33.31**	**33.88**
	Equiv. Refl.: (5,-3,-3), (-2,-3,3), (-3,-2,-3)								
**Quartz**	**(5,-2,-3)**	**7.223**	**70.79**	**1.225**	**1.9**	**2.665**	**5.03**	**33.31**	**33.69**
	Equiv. Refl.: (5,-3,3)								
**Quartz**	**(-2,-3,-3)**	**7.223**	**70.79**	**1.224**	**1.9**	**2.665**	**5.03**	**33.31**	**33.69**
**Quartz**	**(-2,5,3)**	**7.223**	**70.79**	**1.223**	**1.9**	**2.665**	**5.03**	**33.31**	**33.69**
**Quartz**	**(-3,5,-3)**	**7.223**	**70.79**	**1.221**	**1.9**	**2.665**	**5.02**	**33.31**	**33.69**
	Equiv. Refl.: (-3,-2,3)								
**Quartz**	**(-5,2,3)**	**7.223**	**70.79**	**0.897**	**1.9**	**2.665**	**5.03**	**33.31**	**33.69**
	Equiv. Refl.: (-5,3,-3)								
**Quartz**	**(2,3,3)**	**7.223**	**70.79**	**0.896**	**1.9**	**2.665**	**5.03**	**33.31**	**33.69**
**Quartz**	**(2,-5,-3)**	**7.223**	**70.79**	**0.895**	**1.9**	**2.665**	**5.03**	**33.31**	**33.69**
**Quartz**	**(3,2,-3)**	**7.223**	**70.79**	**0.893**	**1.9**	**2.665**	**5.02**	**33.31**	**33.69**
	Equiv. Refl.: (3,-5,3)								
**Quartz**	**(3,-3,5)**	**7.211**	**70.51**	**0.82**	**1.6**	**2.707**	**4.33**	**33.83**	**34.11**
**Quartz**	**(3,0,-5)**	**7.211**	**70.51**	**0.819**	**1.6**	**2.707**	**4.33**	**33.83**	**34.11**
	Equiv. Refl.: (-3,3,-5)								
**Quartz**	**(-3,0,5)**	**7.211**	**70.51**	**0.818**	**1.6**	**2.707**	**4.33**	**33.83**	**34.11**
**Quartz**	**(0,3,5)**	**7.211**	**70.51**	**0.815**	**1.6**	**2.707**	**4.31**	**33.83**	**34.11**
	Equiv. Refl.: (0,-3,-5)								
**Quartz**	**(0,5,-1)**	**7.375**	**74.61**	**0.795**	**1.7**	**2.106**	**3.61**	**26.32**	**26.57**
	Equiv. Refl.: (0,-5,1), (5,0,1), (5,-5,-1), (-5,0,-1), (-5,5,1)								
**LiNbO3**	**(3,3,3)**	**7.349**	**73.89**	**0.272**	**2**	**2.209**	**4.49**	**27.61**	**27.98**
	Equiv. Refl.: (3,3,-3), (3,-6,3), (3,-6,-3), (6,-3,3), (6,-3,-3), (-3,6,3), (-3,6,-3), (-3,-3,3), (-3,-3,-3) (-6,3,3), (-6,3,-3)								
**Quartz**	**(0,5,0)**	**7.285**	**72.25**	**0.208**	**0.8**	**2.448**	**1.89**	**30.6**	**30.66**
	Equiv. Refl.: (0,-5,0), (5,0,0), (5,-5,0), (-5,0,0), (-5,5,0)								

Ei =	8.333	keV						ΔEg	ΔEt
Cryst	Refl	EB	ΘB	∫ IR dΘ	Width	Ei cotΘ	ΔE	2m, 50μm	2m, 50μm
	(h,k,l)	[keV]	[°]	[μrad]	[μrad]	[meV/μrad]	[meV]	[meV]	[meV]
**Ge**	**(2,4,6)**	**8.2**	**79.75**	**48.9**	**45.4**	**1.507**	**68.43**	**18.83**	**70.97**
**Si**	**(4,4,4)**	**7.908**	**71.63**	**16.7**	**14.7**	**2.768**	**40.75**	**34.6**	**53.46**
**Si**	**(1,1,7)**	**8.152**	**78.02**	**15.9**	**14.7**	**1.768**	**25.98**	**22.1**	**34.11**
	Equiv. Refl.: (1,5,5)								
**LiNbO3**	**(1,-5,12)**	**8.331**	**88.61**	**282**	**267.5**	**0.203**	**54.23**	**2.53**	**54.29**
	Equiv. Refl.: (4,1,12), (5,-1,12), (-1,-4,12), (-4,5,12), (-5,4,12)								
**LiNbO3**	**(1,4,12)**	**8.331**	**88.61**	**272.7**	**260**	**0.203**	**52.7**	**2.53**	**52.76**
	Equiv. Refl.: (4,-5,12), (5,-4,12), (-1,5,12), (-4,-1,12), (-5,1,12)								
**LiNbO3**	**(1,4,-12)**	**8.331**	**88.61**	**260**	**267.5**	**0.203**	**54.23**	**2.53**	**54.29**
	Equiv. Refl.: (4,-5,-12), (5,-4,-12), (-1,5,-12), (-4,-1,-12), (-5,1,-12)								
**LiNbO3**	**(1,-5,-12)**	**8.331**	**88.61**	**250.9**	**259.9**	**0.203**	**52.7**	**2.53**	**52.76**
	Equiv. Refl.: (4,1,-12), (5,-1,-12), (-1,-4,-12), (-4,5,-12), (-5,4,-12)								
**LiNbO3**	**(0,5,10)**	**8.266**	**82.75**	**53.1**	**49.2**	**1.06**	**52.11**	**13.25**	**53.77**
	Equiv. Refl.: (5,-5,10), (-5,0,10)								
**LiNbO3**	**(0,-5,-10)**	**8.266**	**82.75**	**45.3**	**49.2**	**1.06**	**52.11**	**13.25**	**53.77**
	Equiv. Refl.: (5,0,-10), (-5,5,-10)								
**LiNbO3**	**(0,0,18)**	**8.049**	**75**	**41.4**	**36.5**	**2.232**	**81.4**	**27.9**	**86.05**
**LiNbO3**	**(0,0,-18)**	**8.049**	**75**	**37.1**	**36.5**	**2.232**	**81.4**	**27.9**	**86.05**
**LiNbO3**	**(2,-6,8)**	**8.181**	**79.05**	**33.2**	**32.1**	**1.612**	**51.7**	**20.15**	**55.49**
	Equiv. Refl.: (4,2,8), (6,-2,8), (-2,-4,8), (-4,6,8), (-6,4,8)								
**Quartz**	**(3,3,3)**	**8.316**	**86.34**	**31.9**	**30**	**0.532**	**15.98**	**6.65**	**17.31**
	Equiv. Refl.: (3,3,-3), (3,-6,3), (3,-6,-3), (-6,3,3), (-6,3,-3)								
**Quartz**	**(6,-3,3)**	**8.316**	**86.34**	**31.7**	**30**	**0.532**	**15.98**	**6.65**	**17.31**
	Equiv. Refl.: (6,-3,-3), (-3,6,3), (-3,6,-3), (-3,-3,3), (-3,-3,-3)								
**LiNbO3**	**(2,4,-8)**	**8.181**	**79.05**	**30.6**	**32.1**	**1.612**	**51.7**	**20.15**	**55.49**
	Equiv. Refl.: (4,-6,-8), (6,-4,-8), (-2,6,-8), (-4,-2,-8), (-6,2,-8)								
**LiNbO3**	**(1,3,-14)**	**8.021**	**74.26**	**28.2**	**26.5**	**2.349**	**62.33**	**29.36**	**68.9**
	Equiv. Refl.: (3,-4,-14), (4,-3,-14), (-1,4,-14), (-3,-1,-14), (-4,1,-14)								
**LiNbO3**	**(1,2,-16)**	**8.045**	**74.9**	**27.3**	**26.7**	**2.249**	**60.05**	**28.11**	**66.31**
	Equiv. Refl.: (2,-3,-16), (3,-2,-16), (-1,3,-16), (-2,-1,-16), (-3,1,-16)								
**LiNbO3**	**(1,-3,16)**	**8.045**	**74.9**	**27.1**	**26.7**	**2.249**	**60.05**	**28.11**	**66.31**
	Equiv. Refl.: (2,1,16), (3,-1,16), (-1,-2,16), (-2,3,16), (-3,2,16)								
**LiNbO3**	**(1,-6,4)**	**7.946**	**72.47**	**27**	**25.5**	**2.633**	**67.24**	**32.91**	**74.86**
	Equiv. Refl.: (5,1,4), (6,-1,4), (-1,-5,4), (-5,6,4), (-6,5,4)								
**LiNbO3**	**(1,-4,14)**	**8.021**	**74.26**	**26.2**	**26.5**	**2.349**	**62.33**	**29.36**	**68.9**
	Equiv. Refl.: (3,1,14), (4,-1,14), (-1,-3,14), (-3,4,14), (-4,3,14)								
**LiNbO3**	**(1,5,-4)**	**7.946**	**72.47**	**25.9**	**25.5**	**2.633**	**67.24**	**32.91**	**74.86**
	Equiv. Refl.: (5,-6,-4), (6,-5,-4), (-1,6,-4), (-5,-1,-4), (-6,1,-4)								
**Sapph**	**(0,2,16)**	**8.208**	**80.06**	**20.5**	**18.6**	**1.46**	**27.21**	**18.25**	**32.76**
	Equiv. Refl.: (0,-2,-16), (2,0,-16), (2,4,4), (2,-2,16), (2,-6,-4), (4,2,-4), (4,-6,4), (6,-2,-4), (6,-4,4) (-2,0,16), (-2,2,-16), (-2,6,4), (-2,-4,-4), (-4,6,-4), (-4,-2,4), (-6,2,4), (-6,4,-4)								
**Quartz**	**(1,5,-1)**	**8.193**	**79.48**	**19.6**	**17.1**	**1.548**	**26.46**	**19.35**	**32.78**
	Equiv. Refl.: (1,-6,1), (5,1,1), (5,-6,-1), (-6,1,-1), (-6,5,1)								
**Quartz**	**(6,-1,1)**	**8.193**	**79.48**	**19.5**	**17.1**	**1.548**	**26.45**	**19.35**	**32.78**
	Equiv. Refl.: (6,-5,-1), (-1,6,-1), (-1,-5,1), (-5,6,1), (-5,-1,-1)								
**Sapph**	**(2,4,-5)**	**8.311**	**85.8**	**16.2**	**18.2**	**0.611**	**11.13**	**7.64**	**13.5**
	Equiv. Refl.: (2,-6,5), (4,2,5), (4,-6,-5), (6,-2,5), (6,-4,-5), (-2,6,-5), (-2,-4,5), (-4,6,5), (-4,-2,-5) (-6,2,-5), (-6,4,5)								
**Quartz**	**(2,3,4)**	**7.835**	**70.09**	**15.2**	**12.8**	**3.019**	**38.5**	**37.73**	**53.91**
	Equiv. Refl.: (2,-5,-4), (3,2,-4), (3,-5,4), (-5,2,4), (-5,3,-4)								

Ei =	8.333	keV						ΔEg	ΔEt
Cryst	Refl	EB	ΘB	∫ IR dΘ	Width	Ei cotΘ	ΔE	2m, 50mm	2m, 50mm
	(h,k,l)	[keV]	[°]	[μrad]	[μrad]	[meV/mrad]	[meV]	[meV]	[meV]
Quartz	(5,-2,-4)	7.835	70.09	15	12.8	3.019	38.49	37.73	53.9
	Equiv. Refl.: (5,-3,4), (-2,5,4), (-2,-3,-4), (-3,5,-4), (-3,-2,4)								
Sapph	(2,3,-10)	8.111	76.74	14.7	13.1	1.964	25.7	24.56	35.55
	Equiv. Refl.: (2,-5,10), (3,2,10), (3,-5,-10), (5,-2,10), (5,-3,-10), (-2,5,-10), (-2,-3,10), (-3,5,10), (-3,-2,-10) (-5,2,-10), (-5,3,10)								
Quartz	(2,4,2)	8.044	74.86	13.8	11.8	2.255	26.51	28.19	38.69
	Equiv. Refl.: (2,-6,-2), (4,2,-2), (4,-6,2), (-6,2,2), (-6,4,-2)								
Quartz	(6,-2,-2)	8.044	74.86	13.6	11.8	2.255	26.51	28.19	38.69
	Equiv. Refl.: (6,-4,2), (-2,6,2), (-2,-4,-2), (-4,6,-2), (-4,-2,2)								
Quartz	(0,1,7)	8.16	78.31	13.1	11.7	1.724	20.11	21.55	29.47
	Equiv. Refl.: (0,-1,-7), (1,0,-7), (1,-1,7), (5,-1,4), (5,-4,-4), (-1,0,7), (-1,1,-7), (-1,5,-4), (-1,-4,4) (-4,5,4), (-4,-1,-4)								
Quartz	(1,4,-4)	8.101	76.45	12.8	11.3	2.008	22.64	25.1	33.8
	Equiv. Refl.: (1,-5,4), (4,1,4), (4,-5,-4), (-5,1,-4), (-5,4,4)								
Sapph	(1,4,9)	8.123	77.11	11.9	11.3	1.908	21.49	23.84	32.1
	Equiv. Refl.: (1,4,-9), (1,-5,9), (1,-5,-9), (4,1,9), (4,1,-9), (4,-5,9), (4,-5,-9), (5,-1,9), (5,-1,-9) (5,-4,9), (5,-4,-9), (-1,5,9), (-1,5,-9), (-1,-4,9), (-1,-4,-9), (-4,5,9), (-4,5,-9), (-4,-1,9) (-4,-1,-9), (-5,1,9), (-5,1,-9), (-5,4,9), (-5,4,-9)								
Quartz	(1,2,6)	7.888	71.19	10.5	9.1	2.838	25.74	35.47	43.82
	Equiv. Refl.: (1,-3,-6), (2,1,-6), (2,-3,6), (-3,1,6), (-3,2,-6)								
Quartz	(0,3,6)	8.153	78.06	10.4	9.7	1.762	17.09	22.02	27.87
	Equiv. Refl.: (0,-3,-6), (3,0,-6), (3,-3,6), (-3,0,6), (-3,3,-6)								
Quartz	(3,-1,-6)	7.888	71.19	10.3	9.1	2.838	25.72	35.47	43.82
	Equiv. Refl.: (3,-2,6), (-1,3,6), (-1,-2,-6), (-2,3,-6), (-2,-1,6)								
LiNbO3	(2,2,15)	8.258	82.31	8.2	14.3	1.126	16.13	14.07	21.41
	Equiv. Refl.: (2,2,-15), (2,-4,15), (2,-4,-15), (4,-2,15), (4,-2,-15), (-2,4,15), (-2,4,-15), (-2,-2,15), (-2,-2,-15) (-4,2,15), (-4,2,-15)								
Quartz	(0,4,-5)	8.177	78.88	7.9	7.9	1.638	12.87	20.47	24.18
	Equiv. Refl.: (0,-4,5), (4,0,5), (4,-4,-5), (-4,0,-5), (-4,4,5)								
Quartz	(5,-2,4)	7.835	70.09	6.6	5.9	3.019	17.82	37.73	41.73
	Equiv. Refl.: (5,-3,-4), (-2,5,-4), (-2,-3,4), (-3,5,4), (-3,-2,-4)								
Quartz	(2,3,-4)	7.835	70.09	6.5	5.9	3.019	17.81	37.73	41.72
	Equiv. Refl.: (2,-5,4), (3,2,4), (3,-5,-4), (-5,2,-4), (-5,3,4)								
Quartz	(1,5,0)	8.112	76.78	6.2	5.7	1.958	11.17	24.47	26.9
	Equiv. Refl.: (1,-6,0), (5,1,0), (5,-6,0), (-6,1,0), (-6,5,0)								
Quartz	(6,-1,0)	8.112	76.78	5.2	5.7	1.958	11.17	24.47	26.9
	Equiv. Refl.: (6,-5,0), (-1,6,0), (-1,-5,0), (-5,6,0), (-5,-1,0)								
Quartz	(6,-2,2)	8.044	74.86	5.1	4.9	2.255	11.11	28.19	30.3
	Equiv. Refl.: (6,-4,-2), (-2,6,-2), (-2,-4,2), (-4,6,2), (-4,-2,-2)								
Sapph	(2,4,1)	7.975	73.14	4.8	5.1	2.525	12.86	31.56	34.08
	Equiv. Refl.: (2,-6,-1), (4,2,-1), (4,-6,1), (6,-2,-1), (6,-4,1), (-2,6,1), (-2,-4,-1), (-4,6,-1), (-4,-2,1) (-6,2,1), (-6,4,-1)								
Quartz	(2,4,-2)	8.044	74.86	4.6	4.9	2.255	11.11	28.19	30.3
	Equiv. Refl.: (2,-6,2), (4,2,2), (4,-6,-2), (-6,2,-2), (-6,4,2)								
LiNbO3	(2,4,7)	7.996	73.65	4.5	7.7	2.445	18.79	30.56	35.88
	Equiv. Refl.: (2,-6,-7), (4,2,-7), (4,-6,7), (6,-2,-7), (6,-4,7), (-2,6,7), (-2,-4,-7), (-4,6,-7), (-4,-2,7) (-6,2,7), (-6,4,-7)								
Quartz	(0,3,-6)	8.153	78.06	4.4	4.7	1.762	8.32	22.02	23.54
	Equiv. Refl.: (0,-3,6), (3,0,6), (3,-3,-6), (-3,0,-6), (-3,3,6)								
Sapph	(3,3,6)	8.325	87.53	4.1	8.7	0.36	3.12	4.5	5.48
	Equiv. Refl.: (3,3,-6), (3,-6,6), (3,-6,-6), (6,-3,6), (6,-3,-6), (-3,6,6), (-3,6,-6), (-3,-3,6), (-3,-3,-6) (-6,3,6), (-6,3,-6)								

Ei =	8.333	keV						ΔEg	ΔEt
**Cryst**	**Refl**	**EB**	**ΘB**	**∫ IR dΘ**	**Width**	**Ei cotΘ**	**ΔE**	**2m, 50mm**	**2m, 50mm**
	**(h,k,l)**	**[keV]**	**[°]**	**[μrad]**	**[μrad]**	**[meV/mrad]**	**[meV]**	**[meV]**	**[meV]**
**Quartz**	**(0,4,5)**	**8.177**	**78.88**	**4**	**4.5**	**1.638**	**7.34**	**20.47**	**21.75**
	Equiv. Refl.: (0,-4,-5), (4,0,-5), (4,-4,5), (-4,0,5), (-4,4,-5)								
**Quartz**	**(3,-1,6)**	**7.888**	**71.19**	**3.6**	**3.4**	**2.838**	**9.66**	**35.47**	**36.76**
	Equiv. Refl.: (3,-2,-6), (-1,3,-6), (-1,-2,6), (-2,3,6), (-2,-1,-6)								
**Quartz**	**(0,1,-7)**	**8.16**	**78.31**	**3.3**	**3.9**	**1.724**	**6.72**	**21.55**	**22.57**
	Equiv. Refl.: (0,-1,7), (1,0,7), (1,-1,-7), (-1,0,-7), (-1,1,7)								
**Sapph**	**(2,4,-2)**	**8.018**	**74.19**	**3.2**	**4**	**2.36**	**9.38**	**29.5**	**30.96**
	Equiv. Refl.: (2,-6,2), (4,2,2), (4,-6,-2), (6,-2,2), (6,-4,-2), (-2,6,-2), (-2,-4,2), (-4,6,2), (-4,-2,-2) (-6,2,-2), (-6,4,2)								
**Quartz**	**(1,2,-6)**	**7.888**	**71.19**	**2.7**	**3.4**	**2.838**	**9.67**	**35.47**	**36.77**
	Equiv. Refl.: (1,-3,6), (2,1,6), (2,-3,-6), (-3,1,-6), (-3,2,6)								
**Sapph**	**(1,3,13)**	**8.241**	**81.5**	**2.4**	**4.2**	**1.245**	**5.18**	**15.57**	**16.4**
	Equiv. Refl.: (1,-4,-13), (3,1,-13), (3,-4,13), (4,-1,-13), (4,-3,13), (-1,4,13), (-1,-3,-13), (-3,4,-13), (-3,-1,13) (-4,1,13), (-4,3,-13)								
**Quartz**	**(0,5,-3)**	**8.057**	**75.21**	**2.1**	**2.9**	**2.201**	**6.43**	**27.51**	**28.25**
	Equiv. Refl.: (0,-5,3), (5,0,3), (5,-5,-3), (-5,0,-3), (-5,5,3)								
**LiNbO3**	**(1,5,5)**	**8.058**	**75.25**	**1.9**	**4.8**	**2.194**	**10.43**	**27.43**	**29.35**
	Equiv. Refl.: (1,-6,-5), (5,1,-5), (5,-6,5), (6,-1,-5), (6,-5,5), (-1,6,5), (-1,-5,-5), (-5,6,-5), (-5,-1,5) (-6,1,5), (-6,5,-5)								
**Quartz**	**(1,4,4)**	**8.101**	**76.45**	**1.7**	**2.3**	**2.008**	**4.7**	**25.1**	**25.53**
	Equiv. Refl.: (1,-5,-4), (4,1,-4), (4,-5,4), (-5,1,4), (-5,4,-4)								
**Quartz**	**(5,-1,-4)**	**8.101**	**76.45**	**1.3**	**2.3**	**2.008**	**4.69**	**25.1**	**25.53**
	Equiv. Refl.: (5,-4,4), (-1,5,4), (-1,-4,-4), (-4,5,-4), (-4,-1,4)								
**Quartz**	**(6,-3,2)**	**7.911**	**71.68**	**1.1**	**1.6**	**2.759**	**4.41**	**34.49**	**34.77**
	Equiv. Refl.: (6,-3,-2), (-3,6,2), (-3,6,-2), (-3,-3,2), (-3,-3,-2)								
**Quartz**	**(3,3,2)**	**7.911**	**71.68**	**0.8**	**1.6**	**2.759**	**4.42**	**34.49**	**34.77**
	Equiv. Refl.: (3,3,-2), (3,-6,2), (3,-6,-2), (-6,3,2), (-6,3,-2)								
**Quartz**	**(6,-1,-1)**	**8.193**	**79.48**	**0.5**	**1**	**1.548**	**1.57**	**19.35**	**19.41**
	Equiv. Refl.: (6,-5,1), (-1,6,1), (-1,-5,-1), (-5,6,-1), (-5,-1,1)								
**LiNbO3**	**(3,3,9)**	**8.27**	**82.98**	**0.4**	**3.2**	**1.027**	**3.31**	**12.83**	**13.25**
	Equiv. Refl.: (3,3,-9), (3,-6,9), (3,-6,-9), (6,-3,9), (6,-3,-9), (-3,6,9), (-3,6,-9), (-3,-3,9), (-3,-3,-9) (-6,3,9), (-6,3,-9)								
**Quartz**	**(1,5,1)**	**8.193**	**79.48**	**0.2**	**1**	**1.548**	**1.57**	**19.35**	**19.41**
	Equiv. Refl.: (1,-6,-1), (5,1,-1), (5,-6,1), (-6,1,1), (-6,5,-1)								
**Sapph**	**(3,3,3)**	**7.947**	**72.5**	**0.1**	**0.5**	**2.628**	**1.36**	**32.85**	**32.87**
	Equiv. Refl.: (3,3,-3), (3,-6,3), (3,-6,-3), (6,-3,3), (6,-3,-3), (-3,6,3), (-3,6,-3), (-3,-3,3), (-3,-3,-3) (-6,3,3), (-6,3,-3)								

Ei = Cryst	7.478 Refl (h,k,l)	keV EB [keV]	ΘB [°]	∫ IR dΘ [μrad]	Width [μrad]	Ei cotΘ [meV/μrad]	ΔE [meV]	ΔEg 2m, 50μm [meV]	ΔEt 2m, 50μm [meV]
**Ge**	**(3,3,5)**	**7.186**	**73.92**	**32.7266**	**32.63**	**2.1553**	**70.33**	**26.94**	**75.31**
	Equiv. Refl.: (3,5,3), (5,3,3)								
**Si**	**(0,2,6)**	**7.219**	**74.88**	**27.5416**	**24.57**	**2.0205**	**49.65**	**25.26**	**55.7**
	Equiv. Refl.: (0,6,2), (2,0,6), (2,6,0), (6,0,2), (6,2,0)								
**Quartz**	**(2,-2,6)**	**7.474**	**88.03**	**202.701**	**172.9**	**0.257**	**44.39**	**3.21**	**44.51**
**Quartz**	**(2,0,-6)**	**7.474**	**88.03**	**202.693**	**172.9**	**0.257**	**44.39**	**3.21**	**44.51**
	Equiv. Refl.: (-2,2,-6)								
**Quartz**	**(-2,0,6)**	**7.474**	**88.03**	**202.688**	**172.9**	**0.257**	**44.39**	**3.21**	**44.51**
**Quartz**	**(0,2,6)**	**7.474**	**88.03**	**202.627**	**172.9**	**0.257**	**44.38**	**3.21**	**44.49**
**Quartz**	**(0,-2,-6)**	**7.474**	**88.03**	**202.626**	**172.9**	**0.257**	**44.38**	**3.21**	**44.49**
**Sapph**	**(1,4,6)**	**7.465**	**86.65**	**118.476**	**104.3**	**0.437**	**45.62**	**5.47**	**45.95**
	Equiv. Refl.: (1,4,-6), (1,-5,6), (1,-5,-6), (4,1,6), (4,1,-6), (4,-5,6), (4,-5,-6), (5,-1,6), (5,-1,-6) (5,-4,6), (5,-4,-6), (-1,5,6), (-1,5,-6), (-1,-4,6), (-1,-4,-6), (-4,5,6), (-4,5,-6), (-4,-1,6) (-4,-1,-6), (-5,1,6), (-5,1,-6), (-5,4,6), (-5,4,-6)								
**LiNbO3**	**(2,4,-2)**	**7.412**	**82.38**	**79.191**	**77.3**	**1.001**	**77.33**	**12.51**	**78.34**
	Equiv. Refl.: (4,-6,-2), (6,-4,-2), (-2,6,-2), (-4,-2,-2), (-6,2,-2)								
**LiNbO3**	**(2,-6,2)**	**7.412**	**82.38**	**78.803**	**77.3**	**1.001**	**77.33**	**12.51**	**78.34**
	Equiv. Refl.: (4,2,2), (6,-2,2), (-2,-4,2), (-4,6,2), (-6,4,2)								
**LiNbO3**	**(0,-5,-4)**	**7.179**	**73.74**	**51.535**	**47.6**	**2.181**	**103.85**	**27.27**	**107.36**
	Equiv. Refl.: (5,0,-4), (-5,5,-4)								
**LiNbO3**	**(0,5,4)**	**7.179**	**73.74**	**49.824**	**47.6**	**2.181**	**103.85**	**27.27**	**107.36**
	Equiv. Refl.: (5,-5,4), (-5,0,4)								
**LiNbO3**	**(3,3,0)**	**7.225**	**75.06**	**47.676**	**45.7**	**1.995**	**91.24**	**24.94**	**94.58**
	Equiv. Refl.: (-3,-3,0)								
**LiNbO3**	**(3,-6,0)**	**7.225**	**75.06**	**47.675**	**45.7**	**1.995**	**91.24**	**24.94**	**94.58**
	Equiv. Refl.: (6,-3,0), (-3,6,0), (-6,3,0)								
**Quartz**	**(2,0,6)**	**7.474**	**88.03**	**45.434**	**48.5**	**0.257**	**12.44**	**3.21**	**12.85**
**Quartz**	**(2,-2,-6)**	**7.474**	**88.03**	**45.43**	**48.5**	**0.257**	**12.44**	**3.21**	**12.85**
**Quartz**	**(-2,0,-6)**	**7.474**	**88.03**	**45.409**	**48.5**	**0.257**	**12.44**	**3.21**	**12.85**
**Quartz**	**(-2,2,6)**	**7.474**	**88.03**	**45.4**	**48.5**	**0.257**	**12.44**	**3.21**	**12.85**
**Quartz**	**(0,2,-6)**	**7.474**	**88.03**	**45.361**	**48.4**	**0.257**	**12.43**	**3.21**	**12.84**
**Quartz**	**(0,-2,6)**	**7.474**	**88.03**	**45.358**	**48.4**	**0.257**	**12.43**	**3.21**	**12.84**
**LiNbO3**	**(1,-3,-14)**	**7.261**	**76.18**	**42.764**	**42.1**	**1.84**	**77.48**	**23**	**80.83**
	Equiv. Refl.: (2,1,-14), (3,-1,-14), (-1,-2,-14), (-2,3,-14), (-3,2,-14)								
**LiNbO3**	**(1,2,14)**	**7.261**	**76.18**	**42.497**	**42.1**	**1.84**	**77.48**	**23**	**80.83**
	Equiv. Refl.: (2,-3,14), (3,-2,14), (-1,3,14), (-2,-1,14), (-3,1,14)								
**LiNbO3**	**(0,4,-10)**	**7.137**	**72.62**	**40.737**	**38.2**	**2.34**	**89.51**	**29.25**	**94.17**
	Equiv. Refl.: (4,-4,-10), (-4,0,-10)								
**LiNbO3**	**(0,-1,16)**	**7.289**	**77.08**	**40.506**	**40.2**	**1.715**	**68.92**	**21.44**	**72.18**
	Equiv. Refl.: (1,0,16)								
**LiNbO3**	**(-1,1,16)**	**7.289**	**77.08**	**40.505**	**40.2**	**1.715**	**68.92**	**21.44**	**72.18**
**LiNbO3**	**(0,1,-16)**	**7.289**	**77.08**	**39.073**	**40.2**	**1.715**	**68.92**	**21.44**	**72.18**
	Equiv. Refl.: (1,-1,-16), (-1,0,-16)								
**LiNbO3**	**(0,-4,10)**	**7.137**	**72.62**	**38.381**	**38.2**	**2.34**	**89.51**	**29.25**	**94.17**
	Equiv. Refl.: (4,0,10), (-4,4,10)								
**LiNbO3**	**(2,-4,12)**	**7.211**	**74.64**	**35.766**	**34.3**	**2.054**	**70.56**	**25.68**	**75.08**
	Equiv. Refl.: (4,-2,12)								

Ei = Cryst	7.478 Refl (h,k,l)	keV EB [keV]	ΘB [°]	∫ IR dΘ [μrad]	Width [μrad]	Ei cotΘ [meV/μrad]	ΔE [meV]	ΔEg 2m, 50μm [meV]	ΔEt 2m, 50μm [meV]
**LiNbO3**	**(2,2,12)**	**7.211**	**74.64**	**35.765**	**34.3**	**2.054**	**70.55**	**25.68**	**75.08**
	Equiv. Refl.: (-2,4,12), (-2,-2,12), (-4,2,12)								
**Sapph**	**(0,2,-14)**	**7.328**	**78.51**	**34.849**	**30.6**	**1.519**	**46.55**	**18.99**	**50.27**
	Equiv. Refl.: (0,-2,14), (2,0,14), (2,-2,-14), (-2,0,-14), (-2,2,14)								
**LiNbO3**	**(2,-5,-8)**	**7.038**	**70.25**	**34.768**	**32.8**	**2.685**	**88.2**	**33.57**	**94.37**
	Equiv. Refl.: (3,2,-8), (5,-2,-8), (-2,-3,-8), (-3,5,-8), (-5,3,-8)								
**LiNbO3**	**(2,3,8)**	**7.038**	**70.25**	**32.631**	**32.8**	**2.685**	**88.2**	**33.57**	**94.37**
	Equiv. Refl.: (3,-5,8), (5,-3,8), (-2,5,8), (-3,-2,8), (-5,2,8)								
**LiNbO3**	**(2,2,-12)**	**7.211**	**74.64**	**32.271**	**34.3**	**2.054**	**70.55**	**25.68**	**75.08**
	Equiv. Refl.: (2,-4,-12), (4,-2,-12), (-2,4,-12), (-2,-2,-12), (-4,2,-12)								
**Sapph**	**(1,3,10)**	**7.225**	**75.06**	**31.763**	**27.4**	**1.995**	**54.75**	**24.94**	**60.16**
	Equiv. Refl.: (1,-4,-10), (3,1,-10), (3,-4,10), (4,-1,-10), (4,-3,10), (-1,4,10), (-1,-3,-10), (-3,4,-10), (-3,-1,10) (-4,1,10), (-4,3,-10)								
**Sapph**	**(0,3,12)**	**7.292**	**77.2**	**28.265**	**25.3**	**1.7**	**42.92**	**21.24**	**47.89**
	Equiv. Refl.: (0,3,-12), (0,-3,12), (0,-3,-12), (3,0,12), (3,0,-12), (3,-3,12), (3,-3,-12), (-3,0,12), (-3,0,-12) (-3,3,12), (-3,3,-12)								
**Sapph**	**(0,4,8)**	**7.127**	**72.37**	**17.949**	**16.2**	**2.376**	**38.49**	**29.7**	**48.62**
	Equiv. Refl.: (0,-4,-8), (4,0,-8), (4,-4,8), (-4,0,8), (-4,4,-8)								
**Quartz**	**(-4,0,4)**	**7.417**	**82.69**	**14.518**	**15.2**	**0.959**	**14.54**	**11.99**	**18.85**
**Quartz**	**(-4,4,-4)**	**7.417**	**82.69**	**14.517**	**15.2**	**0.959**	**14.54**	**11.99**	**18.85**
**Quartz**	**(4,0,-4)**	**7.417**	**82.69**	**14.516**	**15.2**	**0.959**	**14.54**	**11.99**	**18.85**
	Equiv. Refl.: (4,-4,4)								
**Quartz**	**(0,4,4)**	**7.417**	**82.69**	**14.487**	**15.1**	**0.959**	**14.52**	**11.99**	**18.83**
	Equiv. Refl.: (0,-4,-4)								
**Quartz**	**(-4,0,-4)**	**7.417**	**82.69**	**13.777**	**14.6**	**0.959**	**13.98**	**11.99**	**18.42**
**Quartz**	**(4,0,4)**	**7.417**	**82.69**	**13.776**	**14.6**	**0.959**	**13.98**	**11.99**	**18.42**
**Quartz**	**(4,-4,-4)**	**7.417**	**82.69**	**13.774**	**14.6**	**0.959**	**13.98**	**11.99**	**18.42**
**Quartz**	**(-4,4,4)**	**7.417**	**82.69**	**13.773**	**14.6**	**0.959**	**13.98**	**11.99**	**18.42**
**Quartz**	**(0,4,-4)**	**7.417**	**82.69**	**13.767**	**14.6**	**0.959**	**13.98**	**11.99**	**18.41**
**Quartz**	**(0,-4,4)**	**7.417**	**82.69**	**13.766**	**14.6**	**0.959**	**13.98**	**11.99**	**18.41**
**Sapph**	**(1,4,3)**	**7.041**	**70.32**	**12.43**	**11.8**	**2.674**	**31.6**	**33.43**	**46**
	Equiv. Refl.: (1,4,-3), (1,-5,3), (1,-5,-3), (4,1,3), (4,1,-3), (4,-5,3), (4,-5,-3), (5,-1,3), (5,-1,-3) (5,-4,3), (5,-4,-3), (-1,5,3), (-1,5,-3), (-1,-4,3), (-1,-4,-3), (-4,5,3), (-4,5,-3), (-4,-1,3) (-4,-1,-3), (-5,1,3), (-5,1,-3), (-5,4,3), (-5,4,-3)								
**LiNbO3**	**(2,4,1)**	**7.371**	**80.31**	**9.942**	**17.3**	**1.277**	**22.08**	**15.96**	**27.25**
	Equiv. Refl.: (2,-6,-1), (4,2,-1), (4,-6,1), (6,-2,-1), (6,-4,1), (-2,6,1), (-2,-4,-1), (-4,6,-1), (-4,-2,1) (-6,2,1), (-6,4,-1)								
**Quartz**	**(1,0,6)**	**7.035**	**70.17**	**9.715**	**9.1**	**2.696**	**24.61**	**33.71**	**41.74**
**Quartz**	**(1,-1,-6)**	**7.035**	**70.17**	**9.714**	**9.1**	**2.696**	**24.61**	**33.71**	**41.73**
	Equiv. Refl.: (-1,0,-6), (-1,1,6)								
**Quartz**	**(0,1,-6)**	**7.035**	**70.17**	**9.702**	**9.1**	**2.696**	**24.59**	**33.71**	**41.72**
	Equiv. Refl.: (0,-1,6)								
**Quartz**	**(2,-1,-6)**	**7.33**	**78.59**	**8.757**	**9**	**1.509**	**13.52**	**18.87**	**23.21**
**Quartz**	**(2,-1,6)**	**7.33**	**78.59**	**8.753**	**9**	**1.509**	**13.52**	**18.87**	**23.21**
**Quartz**	**(-1,2,6)**	**7.33**	**78.59**	**8.748**	**9**	**1.509**	**13.51**	**18.87**	**23.2**
**Quartz**	**(-1,-1,6)**	**7.33**	**78.59**	**8.747**	**9**	**1.509**	**13.51**	**18.87**	**23.2**
**Quartz**	**(-1,2,-6)**	**7.33**	**78.59**	**8.746**	**8.9**	**1.509**	**13.51**	**18.87**	**23.2**
	Equiv. Refl.: (-1,-1,-6)								
**Quartz**	**(-2,1,6)**	**7.33**	**78.59**	**8.351**	**9**	**1.509**	**13.52**	**18.87**	**23.21**
**Quartz**	**(-2,1,-6)**	**7.33**	**78.59**	**8.347**	**9**	**1.509**	**13.52**	**18.87**	**23.21**
**Quartz**	**(1,-2,-6)**	**7.33**	**78.59**	**8.34**	**9**	**1.509**	**13.51**	**18.87**	**23.2**

Ei =	7.478	keV						ΔEg	ΔEt
Cryst	Refl (h,k,l)	EB [keV]	ΘB [°]	∫ IR dΘ [μrad]	Width [μrad]	Ei cotΘ [meV/μrad]	ΔE [meV]	2m, 50μm [meV]	2m, 50μm [meV]
**Quartz**	**(1,1,6)**	**7.33**	**78.59**	**8.339**	**9**	**1.509**	**13.51**	**18.87**	**23.2**
**Quartz**	**(1,1,-6)**	**7.33**	**78.59**	**8.338**	**9**	**1.509**	**13.51**	**18.87**	**23.2**
	Equiv. Refl.: (1,-2,6)								
**LiNbO3**	**(1,1,15)**	**7.127**	**72.37**	**8.171**	**12.4**	**2.376**	**29.47**	**29.7**	**41.84**
	Equiv. Refl.: (1,1,-15), (1,-2,15), (1,-2,-15), (2,-1,15), (2,-1,-15), (-1,2,15), (-1,2,-15), (-1,-1,15), (-1,-1,-15) (-2,1,15), (-2,1,-15)								
**Sapph**	**(2,3,-7)**	**7.36**	**79.79**	**7.359**	**9**	**1.346**	**12.08**	**16.83**	**20.72**
	Equiv. Refl.: (2,-5,7), (3,2,7), (3,-5,-7), (5,-2,7), (5,-3,-7), (-2,5,-7), (-2,-3,7), (-3,5,7), (-3,-2,-7) (-5,2,-7), (-5,3,7)								
**Quartz**	**(-1,5,-2)**	**7.06**	**70.75**	**6.734**	**6.6**	**2.612**	**17.23**	**32.64**	**36.91**
	Equiv. Refl.: (-1,-4,2)								
**Quartz**	**(-4,5,2)**	**7.06**	**70.75**	**6.733**	**6.6**	**2.612**	**17.23**	**32.64**	**36.91**
	Equiv. Refl.: (-4,-1,-2)								
**Quartz**	**(5,-1,2)**	**7.06**	**70.75**	**6.727**	**6.6**	**2.612**	**17.21**	**32.64**	**36.9**
	Equiv. Refl.: (5,-4,-2)								
**Quartz**	**(1,-5,2)**	**7.06**	**70.75**	**6.53**	**6.6**	**2.612**	**17.23**	**32.64**	**36.91**
**Quartz**	**(1,4,-2)**	**7.06**	**70.75**	**6.529**	**6.6**	**2.612**	**17.23**	**32.64**	**36.91**
**Quartz**	**(4,1,2)**	**7.06**	**70.75**	**6.528**	**6.6**	**2.612**	**17.23**	**32.64**	**36.91**
	Equiv. Refl.: (4,-5,-2)								
**Quartz**	**(-5,1,-2)**	**7.06**	**70.75**	**6.522**	**6.6**	**2.612**	**17.21**	**32.64**	**36.9**
	Equiv. Refl.: (-5,4,2)								
**Quartz**	**(-1,5,2)**	**7.06**	**70.75**	**5.964**	**5.7**	**2.612**	**14.85**	**32.64**	**35.86**
	Equiv. Refl.: (-1,-4,-2)								
**Quartz**	**(-4,5,-2)**	**7.06**	**70.75**	**5.961**	**5.7**	**2.612**	**14.85**	**32.64**	**35.86**
**Quartz**	**(5,-1,-2)**	**7.06**	**70.75**	**5.96**	**5.7**	**2.612**	**14.84**	**32.64**	**35.86**
	Equiv. Refl.: (5,-4,2), (-4,-1,2)								
**Sapph**	**(1,2,-13)**	**7.372**	**80.33**	**5.916**	**7.8**	**1.274**	**9.91**	**15.93**	**18.76**
	Equiv. Refl.: (1,-3,13), (2,1,13), (2,-3,-13), (3,-1,13), (3,-2,-13), (-1,3,-13), (-1,-2,13), (-2,3,13), (-2,-1,-13) (-3,1,-13), (-3,2,13)								
**Quartz**	**(1,4,2)**	**7.06**	**70.75**	**5.127**	**5.7**	**2.612**	**14.85**	**32.64**	**35.86**
	Equiv. Refl.: (1,-5,-2)								
**Quartz**	**(4,-5,2)**	**7.06**	**70.75**	**5.125**	**5.7**	**2.612**	**14.85**	**32.64**	**35.86**
**Quartz**	**(4,1,-2)**	**7.06**	**70.75**	**5.124**	**5.7**	**2.612**	**14.84**	**32.64**	**35.86**
**Quartz**	**(-5,1,2)**	**7.06**	**70.75**	**5.123**	**5.7**	**2.612**	**14.84**	**32.64**	**35.86**
	Equiv. Refl.: (-5,4,-2)								
**Quartz**	**(3,-3,-5)**	**7.211**	**74.64**	**3.931**	**4.7**	**2.054**	**9.56**	**25.68**	**27.4**
**Quartz**	**(3,0,5)**	**7.211**	**74.64**	**3.93**	**4.7**	**2.054**	**9.55**	**25.68**	**27.4**
**Quartz**	**(0,3,-5)**	**7.211**	**74.64**	**3.928**	**4.7**	**2.054**	**9.55**	**25.68**	**27.4**
**Quartz**	**(0,-3,5)**	**7.211**	**74.64**	**3.927**	**4.7**	**2.054**	**9.55**	**25.68**	**27.4**
	Equiv. Refl.: (-3,0,-5), (-3,3,5)								
**Quartz**	**(3,-5,-3)**	**7.223**	**75**	**1.911**	**3**	**2.004**	**6.04**	**25.05**	**25.77**
**Quartz**	**(2,3,-3)**	**7.223**	**75**	**1.91**	**3**	**2.004**	**6.04**	**25.05**	**25.76**
	Equiv. Refl.: (2,-5,3), (3,2,3)								
**Quartz**	**(-5,2,-3)**	**7.223**	**75**	**1.909**	**3**	**2.004**	**6.03**	**25.05**	**25.76**
	Equiv. Refl.: (-5,3,3)								
**Quartz**	**(-2,5,-3)**	**7.223**	**75**	**1.74**	**3**	**2.004**	**6.04**	**25.05**	**25.77**
**Quartz**	**(-2,-3,3)**	**7.223**	**75**	**1.739**	**3**	**2.004**	**6.04**	**25.05**	**25.76**
	Equiv. Refl.: (-3,5,3)								
**Quartz**	**(5,-2,3)**	**7.223**	**75**	**1.738**	**3**	**2.004**	**6.03**	**25.05**	**25.76**
	Equiv. Refl.: (5,-3,-3), (-3,-2,-3)								

Ei =	7.478	keV						ΔEg	ΔEt
Cryst	Refl	EB	ΘB	∫ IR dΘ	Width	Ei cotΘ	ΔE	2m, 50µm	2m, 50µm
	(h,k,l)	[keV]	[°]	[µrad]	[µrad]	[meV/µrad]	[meV]	[meV]	[meV]
**Quartz**	**(5,-2,-3)**	**7.223**	**75**	**1.564**	**2.5**	**2.004**	**4.92**	**25.05**	**25.53**
	Equiv. Refl.: (5,-3,3)								
**Quartz**	**(-2,-3,-3)**	**7.223**	**75**	**1.563**	**2.5**	**2.004**	**4.92**	**25.05**	**25.53**
**Quartz**	**(-2,5,3)**	**7.223**	**75**	**1.562**	**2.5**	**2.004**	**4.92**	**25.05**	**25.53**
**Quartz**	**(-3,5,-3)**	**7.223**	**75**	**1.559**	**2.5**	**2.004**	**4.91**	**25.05**	**25.52**
	Equiv. Refl.: (-3,-2,3)								
**Quartz**	**(5,0,1)**	**7.375**	**80.46**	**1.267**	**2.8**	**1.256**	**3.53**	**15.7**	**16.09**
	Equiv. Refl.: (-5,0,-1)								
**Quartz**	**(0,5,-1)**	**7.375**	**80.46**	**1.266**	**2.8**	**1.256**	**3.53**	**15.7**	**16.09**
	Equiv. Refl.: (0,-5,1), (5,-5,-1), (-5,5,1)								
**Quartz**	**(-5,2,3)**	**7.223**	**75**	**1.13**	**2.5**	**2.004**	**4.92**	**25.05**	**25.53**
	Equiv. Refl.: (-5,3,-3)								
**Quartz**	**(2,3,3)**	**7.223**	**75**	**1.128**	**2.5**	**2.004**	**4.92**	**25.05**	**25.53**
**Quartz**	**(2,-5,-3)**	**7.223**	**75**	**1.127**	**2.5**	**2.004**	**4.92**	**25.05**	**25.53**
**Quartz**	**(3,2,-3)**	**7.223**	**75**	**1.125**	**2.5**	**2.004**	**4.91**	**25.05**	**25.52**
	Equiv. Refl.: (3,-5,3)								
**Quartz**	**(0,1,6)**	**7.035**	**70.17**	**1.051**	**1.9**	**2.696**	**5.2**	**33.71**	**34.1**
	Equiv. Refl.: (0,-1,-6)								
**Quartz**	**(1,0,-6)**	**7.035**	**70.17**	**1.039**	**1.9**	**2.696**	**5.16**	**33.71**	**34.1**
	Equiv. Refl.: (1,-1,6)								
**Quartz**	**(-1,0,6)**	**7.035**	**70.17**	**1.038**	**1.9**	**2.696**	**5.16**	**33.71**	**34.1**
	Equiv. Refl.: (-1,1,-6)								
**Quartz**	**(3,-3,5)**	**7.211**	**74.64**	**1.031**	**2.1**	**2.054**	**4.24**	**25.68**	**26.03**
**Quartz**	**(3,0,-5)**	**7.211**	**74.64**	**1.03**	**2.1**	**2.054**	**4.24**	**25.68**	**26.03**
	Equiv. Refl.: (-3,3,-5)								
**Quartz**	**(-3,0,5)**	**7.211**	**74.64**	**1.029**	**2.1**	**2.054**	**4.24**	**25.68**	**26.03**
**Quartz**	**(0,3,5)**	**7.211**	**74.64**	**1.025**	**2.1**	**2.054**	**4.23**	**25.68**	**26.02**
	Equiv. Refl.: (0,-3,-5)								
**LiNbO3**	**(3,3,3)**	**7.349**	**79.33**	**0.398**	**3.1**	**1.409**	**4.39**	**17.61**	**18.15**
	Equiv. Refl.: (3,3,-3), (3,-6,3), (3,-6,-3), (6,-3,3), (6,-3,-3), (-3,6,3), (-3,6,-3), (-3,-3,3), (-3,-3,-3) (-6,3,3), (-6,3,-3)								
**Quartz**	**(0,5,0)**	**7.285**	**76.95**	**0.276**	**1.1**	**1.733**	**1.85**	**21.66**	**21.74**
	Equiv. Refl.: (0,-5,0), (5,0,0), (5,-5,0), (-5,0,0), (-5,5,0)								

Ei = Cryst	7.461 Refl (h,k,l)	keV EB [keV]	ΘB [°]	∫ IR dΘ [µrad]	Width [µrad]	Ei cotΘ [meV/µrad]	ΔE [meV]	ΔEg 2m, 50µm [meV]	ΔEt 2m, 50µm [meV]
**Ge**	**(3,3,5)**	**7.186**	**74.38**	**33.7246**	**33.65**	**2.0857**	**70.19**	**26.07**	**74.88**
	Equiv. Refl.: (3,5,3), (5,3,3)								
**Si**	**(0,2,6)**	**7.219**	**75.37**	**28.4965**	**25.44**	**1.9475**	**49.54**	**24.34**	**55.2**
	Equiv. Refl.: (0,6,2), (2,0,6), (2,6,0), (6,0,2), (6,2,0)								
**LiNbO3**	**(2,4,-2)**	**7.412**	**83.42**	**91.823**	**89.7**	**0.86**	**77.17**	**10.75**	**77.91**
	Equiv. Refl.: (4,-6,-2), (6,-4,-2), (-2,6,-2), (-4,-2,-2), (-6,2,-2)								
**LiNbO3**	**(2,-6,2)**	**7.412**	**83.42**	**91.371**	**89.7**	**0.86**	**77.17**	**10.75**	**77.91**
	Equiv. Refl.: (4,2,2), (6,-2,2), (-2,-4,2), (-4,6,2), (-6,4,2)								
**LiNbO3**	**(0,-5,-4)**	**7.179**	**74.19**	**53.072**	**49.1**	**2.112**	**103.62**	**26.4**	**106.93**
	Equiv. Refl.: (5,0,-4), (-5,5,-4)								
**LiNbO3**	**(0,5,4)**	**7.179**	**74.19**	**51.303**	**49.1**	**2.112**	**103.62**	**26.4**	**106.93**
	Equiv. Refl.: (5,-5,4), (-5,0,4)								
**LiNbO3**	**(3,3,0)**	**7.225**	**75.56**	**49.365**	**47.4**	**1.922**	**91.04**	**24.02**	**94.16**
	Equiv. Refl.: (3,-6,0), (6,-3,0), (-3,6,0), (-3,-3,0), (-6,3,0)								
**LiNbO3**	**(1,-3,-14)**	**7.261**	**76.72**	**44.547**	**43.9**	**1.761**	**77.32**	**22.02**	**80.39**
	Equiv. Refl.: (2,1,-14), (3,-1,-14), (-1,-2,-14), (-2,3,-14), (-3,2,-14)								
**LiNbO3**	**(1,2,14)**	**7.261**	**76.72**	**44.268**	**43.9**	**1.761**	**77.32**	**22.02**	**80.39**
	Equiv. Refl.: (2,-3,14), (3,-2,14), (-2,-1,14)								
**LiNbO3**	**(-1,3,14)**	**7.261**	**76.72**	**44.267**	**43.9**	**1.761**	**77.32**	**22.02**	**80.39**
	Equiv. Refl.: (-3,1,14)								
**LiNbO3**	**(0,-1,16)**	**7.289**	**77.66**	**42.457**	**42.1**	**1.632**	**68.77**	**20.4**	**71.73**
	Equiv. Refl.: (1,0,16)								
**LiNbO3**	**(-1,1,16)**	**7.289**	**77.66**	**42.456**	**42.1**	**1.632**	**68.77**	**20.4**	**71.73**
**LiNbO3**	**(0,4,-10)**	**7.137**	**73.04**	**41.796**	**39.3**	**2.275**	**89.32**	**28.43**	**93.74**
	Equiv. Refl.: (-4,0,-10)								
**LiNbO3**	**(4,-4,-10)**	**7.137**	**73.04**	**41.795**	**39.3**	**2.275**	**89.32**	**28.43**	**93.74**
**LiNbO3**	**(0,1,-16)**	**7.289**	**77.66**	**40.95**	**42.1**	**1.632**	**68.77**	**20.4**	**71.73**
	Equiv. Refl.: (1,-1,-16), (-1,0,-16)								
**LiNbO3**	**(0,-4,10)**	**7.137**	**73.04**	**39.37**	**39.3**	**2.275**	**89.32**	**28.43**	**93.74**
	Equiv. Refl.: (4,0,10), (-4,4,10)								
**Sapph**	**(0,2,-14)**	**7.328**	**79.18**	**37.018**	**32.6**	**1.427**	**46.45**	**17.83**	**49.75**
	Equiv. Refl.: (0,-2,14), (2,0,14), (2,-2,-14), (-2,0,-14), (-2,2,14)								
**LiNbO3**	**(2,2,12)**	**7.211**	**75.12**	**36.965**	**35.5**	**1.982**	**70.4**	**24.78**	**74.64**
	Equiv. Refl.: (2,-4,12), (4,-2,12), (-2,-2,12)								
**LiNbO3**	**(-2,4,12)**	**7.211**	**75.12**	**36.964**	**35.5**	**1.982**	**70.4**	**24.78**	**74.64**
	Equiv. Refl.: (-4,2,12)								
**LiNbO3**	**(2,-5,-8)**	**7.038**	**70.61**	**35.463**	**33.5**	**2.625**	**88.01**	**32.82**	**93.93**
	Equiv. Refl.: (3,2,-8), (5,-2,-8), (-2,-3,-8), (-3,5,-8), (-5,3,-8)								
**LiNbO3**	**(2,2,-12)**	**7.211**	**75.12**	**33.341**	**35.5**	**1.982**	**70.4**	**24.78**	**74.64**
	Equiv. Refl.: (2,-4,-12), (4,-2,-12), (-2,4,-12), (-2,-2,-12), (-4,2,-12)								
**LiNbO3**	**(2,3,8)**	**7.038**	**70.61**	**33.276**	**33.5**	**2.625**	**88.01**	**32.82**	**93.93**
	Equiv. Refl.: (3,-5,8), (5,-3,8), (-2,5,8), (-3,-2,8), (-5,2,8)								
**Sapph**	**(1,3,10)**	**7.225**	**75.56**	**32.894**	**28.4**	**1.922**	**54.63**	**24.02**	**59.68**
	Equiv. Refl.: (1,-4,-10), (3,1,-10), (3,-4,10), (4,-1,-10), (4,-3,10), (-1,4,10), (-1,-3,-10), (-3,4,-10), (-3,-1,10) (-4,1,10), (-4,3,-10)								
**Sapph**	**(0,3,12)**	**7.292**	**77.78**	**29.652**	**26.5**	**1.615**	**42.83**	**20.19**	**47.35**
	Equiv. Refl.: (0,3,-12), (0,-3,12), (0,-3,-12), (3,0,12), (3,0,-12), (3,-3,12), (3,-3,-12), (-3,0,12), (-3,0,-12) (-3,3,12), (-3,3,-12)								

Ei =	7.461	keV						ΔEg	ΔEt
**Cryst**	**Refl**	**EB**	**ΘB**	**∫ IR dΘ**	**Width**	**Ei cotΘ**	**ΔE**	**2m, 50μm**	**2m, 50μm**
	**(h,k,l)**	**[keV]**	**[°]**	**[μrad]**	**[μrad]**	**[meV/μrad]**	**[meV]**	**[meV]**	**[meV]**
**Sapph**	**(0,4,8)**	**7.127**	**72.79**	**18.398**	**16.6**	**2.311**	**38.41**	**28.89**	**48.06**
	Equiv. Refl.: (0,-4,-8), (4,0,-8), (4,-4,8), (-4,0,8), (-4,4,-8)								
**Quartz**	**(-4,0,4)**	**7.417**	**83.79**	**17.097**	**17.9**	**0.812**	**14.51**	**10.15**	**17.71**
**Quartz**	**(-4,4,-4)**	**7.417**	**83.79**	**17.096**	**17.9**	**0.812**	**14.51**	**10.15**	**17.71**
**Quartz**	**(4,0,-4)**	**7.417**	**83.79**	**17.095**	**17.9**	**0.812**	**14.51**	**10.15**	**17.71**
**Quartz**	**(4,-4,4)**	**7.417**	**83.79**	**17.094**	**17.9**	**0.812**	**14.51**	**10.15**	**17.71**
**Quartz**	**(0,4,4)**	**7.417**	**83.79**	**17.061**	**17.8**	**0.812**	**14.49**	**10.15**	**17.69**
**Quartz**	**(0,-4,-4)**	**7.417**	**83.79**	**17.06**	**17.8**	**0.812**	**14.49**	**10.15**	**17.69**
**Quartz**	**(-4,0,-4)**	**7.417**	**83.79**	**16.222**	**17.2**	**0.812**	**13.95**	**10.15**	**17.25**
**Quartz**	**(4,0,4)**	**7.417**	**83.79**	**16.221**	**17.2**	**0.812**	**13.95**	**10.15**	**17.25**
**Quartz**	**(4,-4,-4)**	**7.417**	**83.79**	**16.218**	**17.2**	**0.812**	**13.95**	**10.15**	**17.25**
**Quartz**	**(-4,4,4)**	**7.417**	**83.79**	**16.217**	**17.2**	**0.812**	**13.95**	**10.15**	**17.25**
**Quartz**	**(0,4,-4)**	**7.417**	**83.79**	**16.21**	**17.2**	**0.812**	**13.95**	**10.15**	**17.25**
**Quartz**	**(0,-4,4)**	**7.417**	**83.79**	**16.209**	**17.2**	**0.812**	**13.95**	**10.15**	**17.25**
**Sapph**	**(1,4,3)**	**7.041**	**70.69**	**12.676**	**12.1**	**2.614**	**31.53**	**32.68**	**45.41**
	Equiv. Refl.: (1,4,-3), (1,-5,3), (1,-5,-3), (4,1,3), (4,1,-3), (4,-5,3), (4,-5,-3), (5,-1,3), (5,-1,-3)<br>(5,-4,3), (5,-4,-3), (-1,5,3), (-1,5,-3), (-1,-4,3), (-1,-4,-3), (-4,5,3), (-4,5,-3), (-4,-1,3)<br>(-4,-1,-3), (-5,1,3), (-5,1,-3), (-5,4,3), (-5,4,-3)								
**LiNbO3**	**(2,4,1)**	**7.371**	**81.11**	**10.823**	**18.9**	**1.167**	**22.03**	**14.59**	**26.43**
	Equiv. Refl.: (2,-6,-1), (4,2,-1), (4,-6,1), (6,-2,-1), (6,-4,1), (-2,6,1), (-2,-4,-1), (-4,6,-1), (-4,-2,1)<br>(-6,2,1), (-6,4,-1)								
**Quartz**	**(1,0,6)**	**7.035**	**70.54**	**9.904**	**9.3**	**2.637**	**24.56**	**32.96**	**41.1**
	Equiv. Refl.: (1,-1,-6), (-1,0,-6)								
**Quartz**	**(-1,1,6)**	**7.035**	**70.54**	**9.903**	**9.3**	**2.637**	**24.56**	**32.96**	**41.1**
**Quartz**	**(0,1,-6)**	**7.035**	**70.54**	**9.892**	**9.3**	**2.637**	**24.53**	**32.96**	**41.09**
	Equiv. Refl.: (0,-1,6)								
**Quartz**	**(2,-1,-6)**	**7.33**	**79.26**	**9.303**	**9.5**	**1.416**	**13.49**	**17.7**	**22.25**
**Quartz**	**(2,-1,6)**	**7.33**	**79.26**	**9.298**	**9.5**	**1.416**	**13.49**	**17.7**	**22.25**
**Quartz**	**(-1,2,6)**	**7.33**	**79.26**	**9.293**	**9.5**	**1.416**	**13.48**	**17.7**	**22.25**
**Quartz**	**(-1,-1,6)**	**7.33**	**79.26**	**9.292**	**9.5**	**1.416**	**13.48**	**17.7**	**22.25**
	Equiv. Refl.: (-1,-1,-6)								
**Quartz**	**(-1,2,-6)**	**7.33**	**79.26**	**9.291**	**9.5**	**1.416**	**13.48**	**17.7**	**22.24**
**Quartz**	**(-2,1,6)**	**7.33**	**79.26**	**8.87**	**9.5**	**1.416**	**13.49**	**17.7**	**22.25**
**Quartz**	**(-2,1,-6)**	**7.33**	**79.26**	**8.866**	**9.5**	**1.416**	**13.49**	**17.7**	**22.25**
**Quartz**	**(1,-2,-6)**	**7.33**	**79.26**	**8.858**	**9.5**	**1.416**	**13.48**	**17.7**	**22.25**
**Quartz**	**(1,1,6)**	**7.33**	**79.26**	**8.857**	**9.5**	**1.416**	**13.48**	**17.7**	**22.25**
**Quartz**	**(1,1,-6)**	**7.33**	**79.26**	**8.856**	**9.5**	**1.416**	**13.48**	**17.7**	**22.25**
**Quartz**	**(1,-2,6)**	**7.33**	**79.26**	**8.855**	**9.5**	**1.416**	**13.48**	**17.7**	**22.24**
**LiNbO3**	**(1,1,15)**	**7.127**	**72.79**	**8.363**	**12.7**	**2.311**	**29.41**	**28.89**	**41.22**
	Equiv. Refl.: (1,1,-15), (1,-2,15), (1,-2,-15), (2,-1,15), (2,-1,-15), (-1,2,15), (-1,2,-15), (-1,-1,15), (-1,-1,-15)<br>(-2,1,15), (-2,1,-15)								
**Sapph**	**(2,3,-7)**	**7.36**	**80.55**	**7.942**	**9.7**	**1.242**	**12.06**	**15.53**	**19.66**
	Equiv. Refl.: (2,-5,7), (3,2,7), (3,-5,-7), (5,-2,7), (5,-3,-7), (-2,5,-7), (-2,-3,7), (-3,5,7), (-3,-2,-7)<br>(-5,2,-7), (-5,3,7)								
**Quartz**	**(-1,5,-2)**	**7.06**	**71.13**	**6.872**	**6.7**	**2.551**	**17.19**	**31.88**	**36.22**
**Quartz**	**(-1,-4,2)**	**7.06**	**71.13**	**6.871**	**6.7**	**2.551**	**17.19**	**31.88**	**36.22**
**Quartz**	**(-4,5,2)**	**7.06**	**71.13**	**6.87**	**6.7**	**2.551**	**17.19**	**31.88**	**36.22**
	Equiv. Refl.: (-4,-1,-2)								
**Quartz**	**(5,-1,2)**	**7.06**	**71.13**	**6.864**	**6.7**	**2.551**	**17.18**	**31.88**	**36.21**
	Equiv. Refl.: (5,-4,-2)								

Ei =<br>Cryst	7.461<br>Refl<br>(h,k,l)	keV<br>EB<br>[keV]	ΘB<br>[°]	∫ IR dΘ<br>[μrad]	Width<br>[μrad]	Ei cotΘ<br>[meV/μrad]	ΔE<br>[meV]	ΔEg<br>2m, 50μm<br>[meV]	ΔEt<br>2m, 50μm<br>[meV]
**Quartz**	**(1,4,-2)**	**7.06**	**71.13**	**6.662**	**6.7**	**2.551**	**17.19**	**31.88**	**36.22**
	Equiv. Refl.: (1,-5,2)								
**Quartz**	**(4,1,2)**	**7.06**	**71.13**	**6.661**	**6.7**	**2.551**	**17.19**	**31.88**	**36.22**
	Equiv. Refl.: (4,-5,-2)								
**Quartz**	**(-5,1,-2)**	**7.06**	**71.13**	**6.654**	**6.7**	**2.551**	**17.18**	**31.88**	**36.21**
	Equiv. Refl.: (-5,4,2)								
**Sapph**	**(1,2,-13)**	**7.372**	**81.13**	**6.332**	**8.5**	**1.165**	**9.89**	**14.56**	**17.6**
	Equiv. Refl.: (1,-3,13), (2,1,13), (2,-3,-13), (3,-1,13), (3,-2,-13), (-1,3,-13), (-1,-2,13), (-2,3,13), (-2,-1,-13) (-3,1,-13), (-3,2,13)								
**Quartz**	**(-1,5,2)**	**7.06**	**71.13**	**6.087**	**5.8**	**2.551**	**14.82**	**31.88**	**35.16**
	Equiv. Refl.: (-1,-4,-2)								
**Quartz**	**(-4,5,-2)**	**7.06**	**71.13**	**6.084**	**5.8**	**2.551**	**14.81**	**31.88**	**35.16**
**Quartz**	**(5,-1,-2)**	**7.06**	**71.13**	**6.083**	**5.8**	**2.551**	**14.81**	**31.88**	**35.15**
	Equiv. Refl.: (5,-4,2), (-4,-1,2)								
**Quartz**	**(1,4,2)**	**7.06**	**71.13**	**5.229**	**5.8**	**2.551**	**14.82**	**31.88**	**35.16**
	Equiv. Refl.: (1,-5,-2)								
**Quartz**	**(4,-5,2)**	**7.06**	**71.13**	**5.228**	**5.8**	**2.551**	**14.81**	**31.88**	**35.16**
**Quartz**	**(4,1,-2)**	**7.06**	**71.13**	**5.227**	**5.8**	**2.551**	**14.81**	**31.88**	**35.16**
**Quartz**	**(-5,1,2)**	**7.06**	**71.13**	**5.226**	**5.8**	**2.551**	**14.81**	**31.88**	**35.15**
	Equiv. Refl.: (-5,4,-2)								
**Quartz**	**(3,-3,-5)**	**7.211**	**75.12**	**4.06**	**4.8**	**1.982**	**9.54**	**24.78**	**26.55**
**Quartz**	**(3,0,5)**	**7.211**	**75.12**	**4.059**	**4.8**	**1.982**	**9.53**	**24.78**	**26.55**
**Quartz**	**(0,3,-5)**	**7.211**	**75.12**	**4.057**	**4.8**	**1.982**	**9.53**	**24.78**	**26.55**
**Quartz**	**(0,-3,5)**	**7.211**	**75.12**	**4.056**	**4.8**	**1.982**	**9.53**	**24.78**	**26.55**
	Equiv. Refl.: (-3,0,-5), (-3,3,5)								
**LiNbO3**	**(1,3,-11)**	**7.024**	**70.28**	**3.197**	**7**	**2.674**	**18.65**	**33.42**	**38.28**
	Equiv. Refl.: (1,-4,11), (3,1,11), (3,-4,-11), (4,-1,11), (4,-3,-11), (-1,4,-11), (-1,-3,11), (-3,4,11), (-3,-1,-11) (-4,1,-11), (-4,3,11)								
**Quartz**	**(3,-5,-3)**	**7.223**	**75.49**	**1.975**	**3.1**	**1.93**	**6.02**	**24.13**	**24.87**
**Quartz**	**(2,3,-3)**	**7.223**	**75.49**	**1.974**	**3.1**	**1.93**	**6.02**	**24.13**	**24.87**
	Equiv. Refl.: (2,-5,3), (3,2,3), (-5,2,-3), (-5,3,3)								
**Quartz**	**(-2,5,-3)**	**7.223**	**75.49**	**1.798**	**3.1**	**1.93**	**6.02**	**24.13**	**24.87**
**Quartz**	**(-2,-3,3)**	**7.223**	**75.49**	**1.797**	**3.1**	**1.93**	**6.02**	**24.13**	**24.87**
	Equiv. Refl.: (-3,5,3)								
**Quartz**	**(5,-2,3)**	**7.223**	**75.49**	**1.796**	**3.1**	**1.93**	**6.02**	**24.13**	**24.87**
	Equiv. Refl.: (5,-3,-3), (-3,-2,-3)								
**Quartz**	**(5,-2,-3)**	**7.223**	**75.49**	**1.617**	**2.5**	**1.93**	**4.91**	**24.13**	**24.62**
	Equiv. Refl.: (5,-3,3)								
**Quartz**	**(-2,-3,-3)**	**7.223**	**75.49**	**1.616**	**2.5**	**1.93**	**4.91**	**24.13**	**24.62**
**Quartz**	**(-2,5,3)**	**7.223**	**75.49**	**1.615**	**2.5**	**1.93**	**4.91**	**24.13**	**24.62**
**Quartz**	**(-3,5,-3)**	**7.223**	**75.49**	**1.612**	**2.5**	**1.93**	**4.9**	**24.13**	**24.62**
**Quartz**	**(-3,-2,3)**	**7.223**	**75.49**	**1.611**	**2.5**	**1.93**	**4.9**	**24.13**	**24.62**
**Quartz**	**(5,0,1)**	**7.375**	**81.28**	**1.383**	**3.1**	**1.145**	**3.53**	**14.31**	**14.74**
	Equiv. Refl.: (-5,0,-1)								
**Quartz**	**(0,5,-1)**	**7.375**	**81.28**	**1.382**	**3.1**	**1.145**	**3.52**	**14.31**	**14.74**
	Equiv. Refl.: (0,-5,1), (5,-5,-1), (-5,5,1)								
**Quartz**	**(-5,3,-3)**	**7.223**	**75.49**	**1.167**	**2.5**	**1.93**	**4.91**	**24.13**	**24.62**
**Quartz**	**(-5,2,3)**	**7.223**	**75.49**	**1.166**	**2.5**	**1.93**	**4.91**	**24.13**	**24.62**
**Quartz**	**(2,3,3)**	**7.223**	**75.49**	**1.165**	**2.5**	**1.93**	**4.91**	**24.13**	**24.62**
**Quartz**	**(2,-5,-3)**	**7.223**	**75.49**	**1.164**	**2.5**	**1.93**	**4.91**	**24.13**	**24.62**

Ei =	7.461	keV						ΔEg	ΔEt
Cryst	Refl	EB	ΘB	∫ IR dΘ	Width	Ei cotΘ	ΔE	2m, 50μm	2m, 50μm
	(h,k,l)	[keV]	[°]	[μrad]	[μrad]	[meV/μrad]	[meV]	[meV]	[meV]
**Quartz**	**(3,2,-3)**	**7.223**	**75.49**	**1.161**	**2.5**	**1.93**	**4.9**	**24.13**	**24.62**
	Equiv. Refl.: (3,-5,3)								
**Quartz**	**(0,-1,-6)**	**7.035**	**70.54**	**1.07**	**2**	**2.637**	**5.19**	**32.96**	**33.36**
**Quartz**	**(0,1,6)**	**7.035**	**70.54**	**1.069**	**2**	**2.637**	**5.19**	**32.96**	**33.36**
**Quartz**	**(3,-3,5)**	**7.211**	**75.12**	**1.064**	**2.1**	**1.982**	**4.23**	**24.78**	**25.14**
**Quartz**	**(-3,3,-5)**	**7.211**	**75.12**	**1.063**	**2.1**	**1.982**	**4.23**	**24.78**	**25.14**
**Quartz**	**(3,0,-5)**	**7.211**	**75.12**	**1.062**	**2.1**	**1.982**	**4.23**	**24.78**	**25.14**
**Quartz**	**(-3,0,5)**	**7.211**	**75.12**	**1.061**	**2.1**	**1.982**	**4.23**	**24.78**	**25.14**
**Quartz**	**(0,3,5)**	**7.211**	**75.12**	**1.058**	**2.1**	**1.982**	**4.22**	**24.78**	**25.13**
	Equiv. Refl.: (0,-3,-5), (1,0,-6)								
**Quartz**	**(1,-1,6)**	**7.035**	**70.54**	**1.057**	**2**	**2.637**	**5.15**	**32.96**	**33.36**
	Equiv. Refl.: (-1,0,6)								
**Quartz**	**(-1,1,-6)**	**7.035**	**70.54**	**1.056**	**2**	**2.637**	**5.15**	**32.96**	**33.36**
**LiNbO3**	**(3,3,3)**	**7.349**	**80.05**	**0.426**	**3.3**	**1.309**	**4.38**	**16.37**	**16.94**
	Equiv. Refl.: (3,3,-3), (3,-6,3), (3,-6,-3), (6,-3,3), (6,-3,-3), (-3,6,3), (-3,6,-3), (-3,-3,3), (-3,-3,-3) (-6,3,3), (-6,3,-3)								
**Quartz**	**(0,5,0)**	**7.285**	**77.53**	**0.288**	**1.1**	**1.65**	**1.85**	**20.63**	**20.71**
	Equiv. Refl.: (0,-5,0), (5,0,0), (5,-5,0), (-5,0,0), (-5,5,0)								

Ei =	8.265	keV						ΔEg	ΔEt
Cryst	Refl (h,k,l)	EB [keV]	ΘB [°]	∫IR dΘ [μrad]	Width [μrad]	Ei cotΘ [meV/μrad]	ΔE [meV]	2m, 50μm [meV]	2m, 50μm [meV]
**Ge**	**(2,4,6)**	**8.2**	**82.81**	**70.1227**	**65.25**	**1.0422**	**68**	**13.03**	**69.24**
	Equiv. Refl.: (2,6,4), (4,2,6), (4,6,2), (6,2,4), (6,4,2)								
**Ge**	**(1,1,7)**	**7.825**	**71.23**	**20.8113**	**20.24**	**2.8089**	**56.85**	**35.11**	**66.82**
	Equiv. Refl.: (1,5,5), (1,7,1), (5,1,5), (5,5,1), (7,1,1)								
**Si**	**(1,1,7)**	**8.152**	**80.5**	**20.0828**	**18.63**	**1.3837**	**25.78**	**17.3**	**31.05**
	Equiv. Refl.: (1,5,5), (1,7,1), (5,1,5), (5,5,1), (7,1,1)								
**Si**	**(4,4,4)**	**7.908**	**73.1**	**18.2121**	**16.11**	**2.5107**	**40.44**	**31.38**	**51.19**
**LiNbO3**	**(0,0,18)**	**8.049**	**76.88**	**47.469**	**41.9**	**1.927**	**80.78**	**24.08**	**84.29**
**LiNbO3**	**(2,-6,8)**	**8.181**	**81.84**	**44.773**	**43.3**	**1.185**	**51.31**	**14.81**	**53.41**
	Equiv. Refl.: (4,2,8), (6,-2,8), (-2,-4,8), (-4,6,8), (-6,4,8)								
**LiNbO3**	**(0,0,-18)**	**8.049**	**76.88**	**42.527**	**41.9**	**1.927**	**80.78**	**24.08**	**84.29**
**LiNbO3**	**(2,4,-8)**	**8.181**	**81.84**	**41.14**	**43.3**	**1.185**	**51.31**	**14.81**	**53.41**
	Equiv. Refl.: (4,-6,-8), (6,-4,-8), (-2,6,-8), (-4,-2,-8), (-6,2,-8)								
**LiNbO3**	**(-1,4,-14)**	**8.021**	**76.03**	**31.881**	**30.1**	**2.056**	**61.86**	**25.7**	**66.99**
	Equiv. Refl.: (-4,1,-14)								
**LiNbO3**	**(1,3,-14)**	**8.021**	**76.03**	**31.88**	**30.1**	**2.056**	**61.86**	**25.7**	**66.99**
	Equiv. Refl.: (3,-4,-14), (4,-3,-14), (-3,-1,-14)								
**LiNbO3**	**(0,5,-8)**	**7.819**	**71.09**	**31.556**	**28.6**	**2.832**	**80.86**	**35.4**	**88.27**
	Equiv. Refl.: (-5,0,-8)								
**LiNbO3**	**(5,-5,-8)**	**7.819**	**71.09**	**31.555**	**28.6**	**2.832**	**80.86**	**35.4**	**88.27**
**LiNbO3**	**(1,2,-16)**	**8.045**	**76.76**	**31.25**	**30.6**	**1.945**	**59.6**	**24.31**	**64.37**
	Equiv. Refl.: (2,-3,-16), (3,-2,-16), (-1,3,-16), (-2,-1,-16), (-3,1,-16)								
**LiNbO3**	**(1,-3,16)**	**8.045**	**76.76**	**31.03**	**30.6**	**1.945**	**59.6**	**24.31**	**64.37**
	Equiv. Refl.: (2,1,16), (3,-1,16), (-1,-2,16)								
**LiNbO3**	**(-2,3,16)**	**8.045**	**76.76**	**31.029**	**30.6**	**1.945**	**59.6**	**24.31**	**64.37**
	Equiv. Refl.: (-3,2,16)								
**Sapph**	**(0,2,16)**	**8.208**	**83.27**	**30.397**	**27.7**	**0.975**	**27**	**12.19**	**29.63**
	Equiv. Refl.: (0,-2,-16), (2,0,-16), (2,-2,16), (-2,0,16), (-2,2,-16)								
**LiNbO3**	**(1,-6,4)**	**7.946**	**74.03**	**29.715**	**28.2**	**2.366**	**66.73**	**29.57**	**72.99**
	Equiv. Refl.: (5,1,4), (6,-1,4), (-1,-5,4), (-5,6,4), (-6,5,4)								
**LiNbO3**	**(1,-4,14)**	**8.021**	**76.03**	**29.598**	**30.1**	**2.056**	**61.86**	**25.7**	**66.99**
	Equiv. Refl.: (3,1,14), (4,-1,14), (-1,-3,14), (-3,4,14), (-4,3,14)								
**LiNbO3**	**(0,-5,8)**	**7.819**	**71.09**	**29.172**	**28.6**	**2.832**	**80.86**	**35.4**	**88.27**
	Equiv. Refl.: (5,0,8), (-5,5,8)								
**LiNbO3**	**(1,5,-4)**	**7.946**	**74.03**	**28.572**	**28.2**	**2.366**	**66.73**	**29.57**	**72.99**
	Equiv. Refl.: (5,-6,-4), (6,-5,-4), (-1,6,-4), (-5,-1,-4), (-6,1,-4)								
**Sapph**	**(2,4,4)**	**8.186**	**82.09**	**28.056**	**25.2**	**1.148**	**28.98**	**14.35**	**32.34**
	Equiv. Refl.: (2,-6,-4), (4,2,-4), (4,-6,4), (6,-2,-4), (6,-4,4), (-2,6,4), (-2,-4,-4), (-4,6,-4), (-4,-2,4) (-6,2,4), (-6,4,-4)								
**Sapph**	**(3,3,0)**	**7.817**	**71.05**	**27.77**	**23**	**2.837**	**65.17**	**35.47**	**74.2**
	Equiv. Refl.: (3,-6,0), (6,-3,0), (-3,6,0), (-3,-3,0), (-6,3,0)								
**Quartz**	**(1,-6,1)**	**8.193**	**82.42**	**27.277**	**23.9**	**1.099**	**26.25**	**13.74**	**29.63**
**Quartz**	**(1,5,-1)**	**8.193**	**82.42**	**27.276**	**23.9**	**1.099**	**26.25**	**13.74**	**29.63**
**Quartz**	**(-6,1,-1)**	**8.193**	**82.42**	**27.275**	**23.9**	**1.099**	**26.25**	**13.74**	**29.63**
	Equiv. Refl.: (-6,5,1)								
**Quartz**	**(5,1,1)**	**8.193**	**82.42**	**27.273**	**23.9**	**1.099**	**26.25**	**13.74**	**29.63**
	Equiv. Refl.: (5,-6,-1)								
**Quartz**	**(-1,6,-1)**	**8.193**	**82.42**	**27.143**	**23.9**	**1.099**	**26.25**	**13.74**	**29.63**

Ei = Cryst	8.265 Refl (h,k,l)	keV EB [keV]	ΘB [°]	∫ IR dΘ [µrad]	Width [µrad]	Ei cotΘ [meV/µrad]	ΔE [meV]	ΔEg 2m, 50µm [meV]	ΔEt 2m, 50µm [meV]
**Quartz**	**(-1,-5,1)**	**8.193**	**82.42**	**27.142**	**23.9**	**1.099**	**26.25**	**13.74**	**29.63**
**Quartz**	**(6,-1,1)**	**8.193**	**82.42**	**27.141**	**23.9**	**1.099**	**26.25**	**13.74**	**29.63**
**Quartz**	**(6,-5,-1)**	**8.193**	**82.42**	**27.14**	**23.9**	**1.099**	**26.25**	**13.74**	**29.63**
**Quartz**	**(-5,6,1)**	**8.193**	**82.42**	**27.139**	**23.9**	**1.099**	**26.25**	**13.74**	**29.63**
	Equiv. Refl.: (-5,-1,-1)								
**LiNbO3**	**(2,2,15)**	**8.258**	**87.64**	**26.479**	**46.9**	**0.341**	**16.01**	**4.26**	**16.57**
	Equiv. Refl.: (2,2,-15), (2,-4,15), (2,-4,-15), (4,-2,15), (4,-2,-15), (-2,4,15), (-2,4,-15), (-2,-2,15), (-2,-2,-15) (-4,2,15), (-4,2,-15)								
**LiNbO3**	**(1,5,2)**	**7.793**	**70.55**	**22.874**	**22.2**	**2.918**	**64.72**	**36.48**	**74.29**
	Equiv. Refl.: (5,-6,2), (6,-5,2), (-1,6,2), (-5,-1,2), (-6,1,2)								
**LiNbO3**	**(1,-6,-2)**	**7.793**	**70.55**	**22.358**	**22.2**	**2.918**	**64.72**	**36.48**	**74.29**
	Equiv. Refl.: (5,1,-2), (6,-1,-2), (-1,-5,-2), (-5,6,-2), (-6,5,-2)								
**Sapph**	**(2,3,-10)**	**8.111**	**78.91**	**17.128**	**15.7**	**1.62**	**25.51**	**20.25**	**32.57**
	Equiv. Refl.: (2,-5,10), (3,2,10), (3,-5,-10), (5,-2,10), (5,-3,-10), (-2,5,-10), (-2,-3,10), (-3,5,10), (-3,-2,-10) (-5,2,-10), (-5,3,10)								
**Quartz**	**(-1,0,7)**	**8.16**	**80.87**	**16.876**	**15**	**1.329**	**19.96**	**16.61**	**25.96**
**Quartz**	**(1,0,-7)**	**8.16**	**80.87**	**16.874**	**15**	**1.329**	**19.96**	**16.61**	**25.96**
	Equiv. Refl.: (-1,1,-7)								
**Quartz**	**(0,1,7)**	**8.16**	**80.87**	**16.872**	**15**	**1.329**	**19.96**	**16.61**	**25.96**
	Equiv. Refl.: (0,-1,-7), (1,-1,7)								
**Quartz**	**(2,3,4)**	**7.835**	**71.43**	**16.389**	**13.8**	**2.776**	**38.2**	**34.7**	**51.61**
	Equiv. Refl.: (2,-5,-4)								
**Quartz**	**(3,2,-4)**	**7.835**	**71.43**	**16.387**	**13.8**	**2.776**	**38.2**	**34.7**	**51.61**
	Equiv. Refl.: (3,-5,4)								
**Quartz**	**(-5,2,4)**	**7.835**	**71.43**	**16.386**	**13.8**	**2.776**	**38.19**	**34.7**	**51.6**
	Equiv. Refl.: (-5,3,-4)								
**Quartz**	**(-2,5,4)**	**7.835**	**71.43**	**16.103**	**13.8**	**2.776**	**38.2**	**34.7**	**51.61**
	Equiv. Refl.: (-2,-3,-4), (-3,-2,4)								
**Quartz**	**(-3,5,-4)**	**7.835**	**71.43**	**16.102**	**13.8**	**2.776**	**38.19**	**34.7**	**51.61**
**Quartz**	**(5,-2,-4)**	**7.835**	**71.43**	**16.101**	**13.8**	**2.776**	**38.19**	**34.7**	**51.6**
	Equiv. Refl.: (5,-3,4)								
**Quartz**	**(4,2,-2)**	**8.044**	**76.71**	**15.8**	**13.5**	**1.952**	**26.31**	**24.4**	**35.88**
	Equiv. Refl.: (4,-6,2)								
**Quartz**	**(-6,2,2)**	**8.044**	**76.71**	**15.799**	**13.5**	**1.952**	**26.3**	**24.4**	**35.88**
	Equiv. Refl.: (-6,4,-2)								
**Quartz**	**(2,4,2)**	**8.044**	**76.71**	**15.798**	**13.5**	**1.952**	**26.3**	**24.4**	**35.88**
	Equiv. Refl.: (2,-6,-2)								
**Quartz**	**(5,-1,4)**	**8.101**	**78.57**	**15.544**	**13.4**	**1.67**	**22.46**	**20.88**	**30.67**
	Equiv. Refl.: (5,-4,-4), (-1,-4,4)								
**Quartz**	**(-1,5,-4)**	**8.101**	**78.57**	**15.543**	**13.4**	**1.67**	**22.46**	**20.88**	**30.67**
	Equiv. Refl.: (-4,-1,-4)								
**Quartz**	**(-4,5,4)**	**8.101**	**78.57**	**15.542**	**13.4**	**1.67**	**22.46**	**20.88**	**30.67**
**Quartz**	**(-4,-2,2)**	**8.044**	**76.71**	**15.497**	**13.5**	**1.952**	**26.31**	**24.4**	**35.88**
**Quartz**	**(-4,6,-2)**	**8.044**	**76.71**	**15.496**	**13.5**	**1.952**	**26.3**	**24.4**	**35.88**
**Quartz**	**(6,-2,-2)**	**8.044**	**76.71**	**15.495**	**13.5**	**1.952**	**26.3**	**24.4**	**35.88**
	Equiv. Refl.: (6,-4,2), (-2,6,2), (-2,-4,-2)								
**Quartz**	**(1,4,-4)**	**8.101**	**78.57**	**15.255**	**13.4**	**1.67**	**22.47**	**20.88**	**30.67**
	Equiv. Refl.: (-5,4,4)								
**Quartz**	**(1,-5,4)**	**8.101**	**78.57**	**15.254**	**13.4**	**1.67**	**22.46**	**20.88**	**30.67**
	Equiv. Refl.: (-5,1,-4)								

Ei =	8.265	keV						ΔEg	ΔEt
Cryst	Refl	EB	ΘB	∫ IR dΘ	Width	Ei cotΘ	ΔE	2m, 50µm	2m, 50µm
	(h,k,l)	[keV]	[°]	[µrad]	[µrad]	[meV/µrad]	[meV]	[meV]	[meV]
Quartz	(4,1,4)	8.101	78.57	15.253	13.4	1.67	22.46	20.88	30.67
Quartz	(4,-5,-4)	8.101	78.57	15.252	13.4	1.67	22.46	20.88	30.67
Sapph	(1,2,14)	7.778	70.23	14.917	13.1	2.971	38.8	37.14	53.71
	Equiv. Refl.: (1,-3,-14), (2,1,-14), (2,-3,14), (3,-1,-14), (3,-2,14), (-1,3,14), (-1,-2,-14), (-2,3,-14), (-2,-1,14) (-3,1,14), (-3,2,-14)								
Sapph	(1,4,9)	8.123	79.36	14.412	13.7	1.553	21.32	19.41	28.83
	Equiv. Refl.: (1,4,-9), (1,-5,9), (1,-5,-9), (4,1,9), (4,1,-9), (4,-5,9), (4,-5,-9), (5,-1,9), (5,-1,-9) (5,-4,9), (5,-4,-9), (-1,5,9), (-1,5,-9), (-1,-4,9), (-1,-4,-9), (-4,5,9), (-4,5,-9), (-4,-1,9) (-4,-1,-9), (-5,1,9), (-5,1,-9), (-5,4,9), (-5,4,-9)								
Quartz	(0,3,6)	8.153	80.55	13.139	12.3	1.376	16.96	17.2	24.15
	Equiv. Refl.: (0,-3,-6)								
Quartz	(3,-3,6)	8.153	80.55	13.131	12.3	1.376	16.95	17.2	24.14
	Equiv. Refl.: (-3,3,-6)								
Quartz	(3,0,-6)	8.153	80.55	13.129	12.3	1.376	16.95	17.2	24.14
	Equiv. Refl.: (-3,0,6)								
Quartz	(2,1,-6)	7.888	72.63	11.388	9.9	2.585	25.54	32.31	41.19
Quartz	(1,2,6)	7.888	72.63	11.386	9.9	2.585	25.53	32.31	41.18
	Equiv. Refl.: (2,-3,6)								
Quartz	(1,-3,-6)	7.888	72.63	11.385	9.9	2.585	25.53	32.31	41.18
Quartz	(-3,1,6)	7.888	72.63	11.379	9.9	2.585	25.52	32.31	41.17
	Equiv. Refl.: (-3,2,-6)								
Quartz	(-2,-1,6)	7.888	72.63	11.154	9.9	2.585	25.54	32.31	41.19
Quartz	(-1,-2,-6)	7.888	72.63	11.153	9.9	2.585	25.53	32.31	41.18
Quartz	(-1,3,6)	7.888	72.63	11.152	9.9	2.585	25.53	32.31	41.18
	Equiv. Refl.: (-2,3,-6)								
Quartz	(3,-1,-6)	7.888	72.63	11.145	9.9	2.585	25.52	32.31	41.17
	Equiv. Refl.: (3,-2,6)								
Quartz	(0,4,-5)	8.177	81.61	10.477	10.5	1.219	12.77	15.24	19.88
	Equiv. Refl.: (0,-4,5)								
Quartz	(4,0,5)	8.177	81.61	10.46	10.5	1.219	12.75	15.24	19.87
	Equiv. Refl.: (4,-4,-5), (-4,4,5)								
Quartz	(-4,0,-5)	8.177	81.61	10.459	10.5	1.219	12.75	15.24	19.87
Sapph	(0,1,-16)	7.783	70.34	8.104	7.7	2.952	22.61	36.9	43.28
	Equiv. Refl.: (0,-1,16), (1,0,16), (1,-1,-16), (-1,0,-16), (-1,1,16)								
Quartz	(1,5,0)	8.112	78.96	7.433	6.9	1.612	11.08	20.15	22.99
	Equiv. Refl.: (1,-6,0), (5,1,0), (5,-6,0), (-6,1,0), (-6,5,0)								
Quartz	(5,-2,4)	7.835	71.43	6.785	6.4	2.776	17.68	34.7	38.95
	Equiv. Refl.: (5,-3,-4)								
Quartz	(-2,5,-4)	7.835	71.43	6.778	6.4	2.776	17.66	34.7	38.94
	Equiv. Refl.: (-2,-3,4)								
Quartz	(-3,-2,-4)	7.835	71.43	6.776	6.4	2.776	17.66	34.7	38.94
Quartz	(-3,5,4)	7.835	71.43	6.775	6.4	2.776	17.66	34.7	38.94
Quartz	(4,2,-1)	7.795	70.57	6.709	5.9	2.915	17.18	36.43	40.28
	Equiv. Refl.: (4,-6,1)								
Quartz	(2,-6,-1)	7.795	70.57	6.708	5.9	2.915	17.17	36.43	40.28
Quartz	(2,4,1)	7.795	70.57	6.707	5.9	2.915	17.17	36.43	40.28
	Equiv. Refl.: (-6,2,1), (-6,4,-1)								
Quartz	(-5,2,-4)	7.835	71.43	6.675	6.4	2.776	17.68	34.7	38.95
	Equiv. Refl.: (-5,3,4)								

Ei =	8.265	keV						ΔEg	ΔEt
Cryst	Refl	EB	ΘB	∫ IR dΘ	Width	Ei cotΘ	ΔE	2m, 50µm	2m, 50µm
	(h,k,l)	[keV]	[°]	[µrad]	[µrad]	[meV/µrad]	[meV]	[meV]	[meV]
**Quartz**	**(2,3,-4)**	**7.835**	**71.43**	**6.668**	**6.4**	**2.776**	**17.67**	**34.7**	**38.94**
	Equiv. Refl.: (2,-5,4)								
**Quartz**	**(3,2,4)**	**7.835**	**71.43**	**6.666**	**6.4**	**2.776**	**17.66**	**34.7**	**38.94**
**Quartz**	**(3,-5,-4)**	**7.835**	**71.43**	**6.665**	**6.4**	**2.776**	**17.66**	**34.7**	**38.94**
**Quartz**	**(-4,3,-5)**	**7.777**	**70.22**	**6.469**	**5.6**	**2.972**	**16.79**	**37.15**	**40.77**
**Quartz**	**(-4,1,5)**	**7.777**	**70.22**	**6.468**	**5.6**	**2.972**	**16.79**	**37.15**	**40.77**
**Quartz**	**(1,3,5)**	**7.777**	**70.22**	**6.465**	**5.6**	**2.972**	**16.78**	**37.15**	**40.77**
	Equiv. Refl.: (1,-4,-5)								
**Quartz**	**(3,1,-5)**	**7.777**	**70.22**	**6.464**	**5.6**	**2.972**	**16.78**	**37.15**	**40.77**
**Quartz**	**(3,-4,5)**	**7.777**	**70.22**	**6.463**	**5.6**	**2.972**	**16.78**	**37.15**	**40.77**
**Quartz**	**(6,-1,0)**	**8.112**	**78.96**	**6.252**	**6.9**	**1.612**	**11.08**	**20.15**	**22.99**
	Equiv. Refl.: (6,-5,0), (-1,6,0), (-1,-5,0), (-5,6,0), (-5,-1,0)								
**Quartz**	**(-4,6,-1)**	**7.795**	**70.57**	**6.137**	**5.9**	**2.915**	**17.18**	**36.43**	**40.28**
	Equiv. Refl.: (-4,-2,1)								
**Quartz**	**(6,-2,-1)**	**7.795**	**70.57**	**6.135**	**5.9**	**2.915**	**17.17**	**36.43**	**40.28**
	Equiv. Refl.: (6,-4,1), (-2,6,1), (-2,-4,-1)								
**Quartz**	**(6,-4,-2)**	**8.044**	**76.71**	**5.856**	**5.6**	**1.952**	**11.02**	**24.4**	**26.77**
	Equiv. Refl.: (-2,6,-2)								
**Quartz**	**(6,-2,2)**	**8.044**	**76.71**	**5.855**	**5.6**	**1.952**	**11.02**	**24.4**	**26.77**
	Equiv. Refl.: (-2,-4,2)								
**Quartz**	**(-4,6,2)**	**8.044**	**76.71**	**5.851**	**5.6**	**1.952**	**11.01**	**24.4**	**26.77**
	Equiv. Refl.: (-4,-2,-2)								
**Quartz**	**(4,-3,5)**	**7.777**	**70.22**	**5.809**	**5.6**	**2.972**	**16.79**	**37.15**	**40.77**
**Quartz**	**(4,-1,-5)**	**7.777**	**70.22**	**5.808**	**5.6**	**2.972**	**16.79**	**37.15**	**40.77**
**Quartz**	**(-1,4,5)**	**7.777**	**70.22**	**5.807**	**5.6**	**2.972**	**16.78**	**37.15**	**40.77**
**Quartz**	**(-1,-3,-5)**	**7.777**	**70.22**	**5.806**	**5.6**	**2.972**	**16.78**	**37.15**	**40.77**
	Equiv. Refl.: (-3,-1,5)								
**Quartz**	**(-3,4,-5)**	**7.777**	**70.22**	**5.805**	**5.6**	**2.972**	**16.78**	**37.15**	**40.77**
**Quartz**	**(-3,3,6)**	**8.153**	**80.55**	**5.356**	**6**	**1.376**	**8.27**	**17.2**	**19.08**
**Quartz**	**(3,-3,-6)**	**8.153**	**80.55**	**5.355**	**6**	**1.376**	**8.27**	**17.2**	**19.08**
**Quartz**	**(3,0,6)**	**8.153**	**80.55**	**5.354**	**6**	**1.376**	**8.27**	**17.2**	**19.08**
	Equiv. Refl.: (-3,0,-6)								
**Quartz**	**(0,3,-6)**	**8.153**	**80.55**	**5.336**	**6**	**1.376**	**8.25**	**17.2**	**19.07**
	Equiv. Refl.: (0,-3,6)								
**Sapph**	**(2,4,1)**	**7.975**	**74.78**	**5.268**	**5.7**	**2.249**	**12.76**	**28.12**	**30.87**
	Equiv. Refl.: (2,-6,-1), (4,2,-1), (4,-6,1), (6,-2,-1), (6,-4,1), (-2,6,1), (-2,-4,-1), (-4,6,-1), (-4,-2,1) (-6,2,1), (-6,4,-1)								
**LiNbO3**	**(2,4,7)**	**7.996**	**75.34**	**5.228**	**8.6**	**2.162**	**18.64**	**27.03**	**32.83**
	Equiv. Refl.: (4,2,-7), (4,-6,7), (6,-4,7), (-2,-4,-7), (-4,6,-7), (-4,-2,7), (-6,4,-7)								
**LiNbO3**	**(2,-6,-7)**	**7.996**	**75.34**	**5.227**	**8.6**	**2.162**	**18.64**	**27.03**	**32.83**
	Equiv. Refl.: (6,-2,-7), (-2,6,7), (-6,2,7)								
**Quartz**	**(2,4,-2)**	**8.044**	**76.71**	**5.198**	**5.6**	**1.952**	**11.02**	**24.4**	**26.77**
	Equiv. Refl.: (2,-6,2), (-6,2,-2), (-6,4,2)								
**Quartz**	**(4,2,2)**	**8.044**	**76.71**	**5.194**	**5.6**	**1.952**	**11.02**	**24.4**	**26.77**
	Equiv. Refl.: (4,-6,-2)								
**Sapph**	**(1,3,13)**	**8.241**	**85.68**	**5.147**	**8.2**	**0.625**	**5.14**	**7.81**	**9.35**
	Equiv. Refl.: (1,-4,-13), (3,1,-13), (3,-4,13), (4,-1,-13), (4,-3,13), (-1,4,13), (-1,-3,-13), (-3,4,-13), (-3,-1,13) (-4,1,13), (-4,3,-13)								
**Quartz**	**(0,4,5)**	**8.177**	**81.61**	**5.08**	**6**	**1.219**	**7.28**	**15.24**	**16.89**
	Equiv. Refl.: (0,-4,-5)								
**Quartz**	**(-4,4,-5)**	**8.177**	**81.61**	**5.076**	**6**	**1.219**	**7.28**	**15.24**	**16.88**

Ei =	8.265	keV						ΔEg	ΔEt
Cryst	Refl	EB	ΘB	∫ IR dΘ	Width	Ei cotΘ	ΔE	2m, 50µm	2m, 50µm
	(h,k,l)	[keV]	[°]	[µrad]	[µrad]	[meV/µrad]	[meV]	[meV]	[meV]
**Quartz**	**(4,-4,5)**	**8.177**	**81.61**	**5.074**	**6**	**1.219**	**7.28**	**15.24**	**16.88**
**Quartz**	**(4,0,-5)**	**8.177**	**81.61**	**5.073**	**6**	**1.219**	**7.28**	**15.24**	**16.88**
	Equiv. Refl.: (-4,0,5)								
**Quartz**	**(0,1,-7)**	**8.16**	**80.87**	**4.25**	**5**	**1.329**	**6.66**	**16.61**	**17.89**
**Quartz**	**(0,-1,7)**	**8.16**	**80.87**	**4.249**	**5**	**1.329**	**6.66**	**16.61**	**17.89**
**Quartz**	**(1,-1,-7)**	**8.16**	**80.87**	**4.231**	**5**	**1.329**	**6.64**	**16.61**	**17.88**
	Equiv. Refl.: (-1,1,7)								
**Quartz**	**(-1,0,-7)**	**8.16**	**80.87**	**4.229**	**5**	**1.329**	**6.64**	**16.61**	**17.88**
**Quartz**	**(1,0,7)**	**8.16**	**80.87**	**4.228**	**5**	**1.329**	**6.64**	**16.61**	**17.88**
**Quartz**	**(-1,4,-5)**	**7.777**	**70.22**	**4.115**	**3.9**	**2.972**	**11.68**	**37.15**	**38.95**
	Equiv. Refl.: (-1,-3,5)								
**Quartz**	**(4,-1,5)**	**7.777**	**70.22**	**4.114**	**3.9**	**2.972**	**11.68**	**37.15**	**38.95**
**Quartz**	**(4,-3,-5)**	**7.777**	**70.22**	**4.113**	**3.9**	**2.972**	**11.68**	**37.15**	**38.95**
**Quartz**	**(-3,-1,-5)**	**7.777**	**70.22**	**4.111**	**3.9**	**2.972**	**11.67**	**37.15**	**38.94**
**Quartz**	**(-3,4,5)**	**7.777**	**70.22**	**4.109**	**3.9**	**2.972**	**11.67**	**37.15**	**38.94**
**Quartz**	**(1,3,-5)**	**7.777**	**70.22**	**3.934**	**3.9**	**2.972**	**11.68**	**37.15**	**38.95**
**Quartz**	**(1,-4,5)**	**7.777**	**70.22**	**3.933**	**3.9**	**2.972**	**11.68**	**37.15**	**38.95**
**Quartz**	**(-4,1,-5)**	**7.777**	**70.22**	**3.932**	**3.9**	**2.972**	**11.68**	**37.15**	**38.95**
	Equiv. Refl.: (-4,3,5)								
**Quartz**	**(3,1,5)**	**7.777**	**70.22**	**3.928**	**3.9**	**2.972**	**11.67**	**37.15**	**38.94**
**Quartz**	**(3,-4,-5)**	**7.777**	**70.22**	**3.927**	**3.9**	**2.972**	**11.67**	**37.15**	**38.94**
**Quartz**	**(-1,3,-6)**	**7.888**	**72.63**	**3.877**	**3.7**	**2.585**	**9.6**	**32.31**	**33.71**
**Quartz**	**(-1,-2,6)**	**7.888**	**72.63**	**3.876**	**3.7**	**2.585**	**9.59**	**32.31**	**33.71**
**Quartz**	**(-2,-1,-6)**	**7.888**	**72.63**	**3.874**	**3.7**	**2.585**	**9.59**	**32.31**	**33.71**
**Quartz**	**(-2,3,6)**	**7.888**	**72.63**	**3.871**	**3.7**	**2.585**	**9.58**	**32.31**	**33.7**
**Quartz**	**(3,-1,6)**	**7.888**	**72.63**	**3.869**	**3.7**	**2.585**	**9.58**	**32.31**	**33.7**
	Equiv. Refl.: (3,-2,-6)								
**Sapph**	**(2,4,-2)**	**8.018**	**75.95**	**3.817**	**4.5**	**2.069**	**9.31**	**25.86**	**27.48**
	Equiv. Refl.: (2,-6,2), (4,2,2), (4,-6,-2), (6,-2,2), (6,-4,-2), (-2,6,-2), (-2,-4,2), (-4,6,2), (-4,-2,-2) (-6,2,-2), (-6,4,2)								
**Quartz**	**(1,-3,6)**	**7.888**	**72.63**	**2.972**	**3.7**	**2.585**	**9.6**	**32.31**	**33.71**
**Quartz**	**(1,2,-6)**	**7.888**	**72.63**	**2.971**	**3.7**	**2.585**	**9.59**	**32.31**	**33.71**
**Quartz**	**(2,1,6)**	**7.888**	**72.63**	**2.969**	**3.7**	**2.585**	**9.59**	**32.31**	**33.71**
**Quartz**	**(2,-3,-6)**	**7.888**	**72.63**	**2.967**	**3.7**	**2.585**	**9.58**	**32.31**	**33.7**
**Quartz**	**(-3,1,-6)**	**7.888**	**72.63**	**2.965**	**3.7**	**2.585**	**9.58**	**32.31**	**33.7**
	Equiv. Refl.: (-3,2,6)								
**Quartz**	**(5,0,3)**	**8.057**	**77.11**	**2.395**	**3.4**	**1.891**	**6.39**	**23.64**	**24.49**
	Equiv. Refl.: (-5,0,-3), (-5,5,3)								
**Quartz**	**(5,-5,-3)**	**8.057**	**77.11**	**2.394**	**3.4**	**1.891**	**6.39**	**23.64**	**24.49**
**Quartz**	**(0,5,-3)**	**8.057**	**77.11**	**2.39**	**3.4**	**1.891**	**6.38**	**23.64**	**24.49**
	Equiv. Refl.: (0,-5,3)								
**LiNbO3**	**(1,5,5)**	**8.058**	**77.16**	**2.118**	**5.5**	**1.884**	**10.35**	**23.55**	**25.72**
	Equiv. Refl.: (1,-6,-5), (5,1,-5), (5,-6,5), (6,-1,-5), (6,-5,5), (-1,6,5), (-1,-5,-5), (-5,6,-5), (-5,-1,5) (-6,1,5), (-6,5,-5)								
**Quartz**	**(1,4,4)**	**8.101**	**78.57**	**2.02**	**2.8**	**1.67**	**4.67**	**20.88**	**21.4**
	Equiv. Refl.: (1,-5,-4)								
**Quartz**	**(-5,4,-4)**	**8.101**	**78.57**	**2.016**	**2.8**	**1.67**	**4.66**	**20.88**	**21.39**
**Quartz**	**(-5,1,4)**	**8.101**	**78.57**	**2.014**	**2.8**	**1.67**	**4.66**	**20.88**	**21.39**
**Quartz**	**(4,1,-4)**	**8.101**	**78.57**	**2.013**	**2.8**	**1.67**	**4.66**	**20.88**	**21.39**
	Equiv. Refl.: (4,-5,4)								

Ei =	8.265	keV						ΔEg	ΔEt
Cryst	Refl	EB	ΘB	∫ IR dΘ	Width	Ei cotΘ	ΔE	2m, 50µm	2m, 50µm
	(h,k,l)	[keV]	[°]	[µrad]	[µrad]	[meV/µrad]	[meV]	[meV]	[meV]
**Quartz**	**(-1,5,4)**	**8.101**	**78.57**	**1.508**	**2.8**	**1.67**	**4.67**	**20.88**	**21.4**
	Equiv. Refl.: (-1,-4,-4)								
**Quartz**	**(5,-4,4)**	**8.101**	**78.57**	**1.504**	**2.8**	**1.67**	**4.66**	**20.88**	**21.39**
**Quartz**	**(5,-1,-4)**	**8.101**	**78.57**	**1.502**	**2.8**	**1.67**	**4.66**	**20.88**	**21.39**
	Equiv. Refl.: (-4,5,-4), (-4,-1,4)								
**Quartz**	**(-3,6,2)**	**7.911**	**73.16**	**1.223**	**1.8**	**2.501**	**4.38**	**31.27**	**31.57**
	Equiv. Refl.: (-3,-3,-2)								
**Quartz**	**(-3,6,-2)**	**7.911**	**73.16**	**1.222**	**1.7**	**2.501**	**4.38**	**31.27**	**31.57**
**Quartz**	**(6,-3,-2)**	**7.911**	**73.16**	**1.221**	**1.7**	**2.501**	**4.37**	**31.27**	**31.57**
	Equiv. Refl.: (-3,-3,2)								
**Quartz**	**(6,-3,2)**	**7.911**	**73.16**	**1.22**	**1.7**	**2.501**	**4.37**	**31.27**	**31.57**
**Quartz**	**(3,3,2)**	**7.911**	**73.16**	**0.881**	**1.8**	**2.501**	**4.38**	**31.27**	**31.57**
	Equiv. Refl.: (3,-6,-2)								
**Quartz**	**(3,3,-2)**	**7.911**	**73.16**	**0.88**	**1.7**	**2.501**	**4.38**	**31.27**	**31.57**
	Equiv. Refl.: (3,-6,2)								
**Quartz**	**(-6,3,2)**	**7.911**	**73.16**	**0.879**	**1.7**	**2.501**	**4.37**	**31.27**	**31.57**
	Equiv. Refl.: (-6,3,-2)								
**Quartz**	**(-5,6,-1)**	**8.193**	**82.42**	**0.68**	**1.4**	**1.099**	**1.56**	**13.74**	**13.83**
**Quartz**	**(6,-1,-1)**	**8.193**	**82.42**	**0.679**	**1.4**	**1.099**	**1.56**	**13.74**	**13.83**
	Equiv. Refl.: (6,-5,1), (-1,6,1), (-1,-5,-1), (-5,-1,1)								
**Quartz**	**(6,-2,1)**	**7.795**	**70.57**	**0.58**	**1**	**2.915**	**3.06**	**36.43**	**36.56**
	Equiv. Refl.: (6,-4,-1), (-2,6,-1), (-2,-4,1), (-4,6,1), (-4,-2,-1)								
**LiNbO3**	**(2,3,11)**	**7.806**	**70.81**	**0.533**	**2.2**	**2.876**	**6.42**	**35.95**	**36.52**
	Equiv. Refl.: (2,-5,-11), (3,2,-11), (3,-5,11), (5,-2,-11), (5,-3,11), (-2,5,11), (-2,-3,-11), (-3,5,-11), (-3,-2,11) (-5,2,11), (-5,3,-11)								
**Quartz**	**(2,4,-1)**	**7.795**	**70.57**	**0.461**	**1**	**2.915**	**3.06**	**36.43**	**36.56**
	Equiv. Refl.: (2,-6,1), (4,2,1), (4,-6,-1), (-6,2,-1), (-6,4,1)								
**Quartz**	**(1,5,1)**	**8.193**	**82.42**	**0.264**	**1.4**	**1.099**	**1.56**	**13.74**	**13.83**
	Equiv. Refl.: (1,-6,-1), (5,1,-1), (5,-6,1), (-6,1,1), (-6,5,-1)								
**Sapph**	**(3,3,3)**	**7.947**	**74.06**	**0.106**	**0.6**	**2.36**	**1.35**	**29.51**	**29.54**
	Equiv. Refl.: (3,3,-3), (3,-6,3), (3,-6,-3), (6,-3,3), (6,-3,-3), (-3,6,3), (-3,6,-3), (-3,-3,3), (-3,-3,-3) (-6,3,3), (-6,3,-3)								

Ei =	8.9805	keV						ΔEg	ΔEt
Cryst	Refl	EB	ΘB	∫ IR dΘ	Width	Ei cotΘ	ΔE	2m, 50μm	2m, 50μm
	(h,k,l)	[keV]	[°]	[μrad]	[μrad]	[meV/μrad]	[meV]	[meV]	[meV]
**Ge**	**(3,3,7)**	**8.969**	**87.14**	**80.2**	**81.5**	**0.448**	**36.51**	**5.6**	**36.94**
**Ge**	**(0,0,8)**	**8.766**	**77.46**	**30.3**	**28.2**	**1.998**	**56.34**	**24.97**	**61.62**
**Si**	**(2,4,6)**	**8.542**	**72.02**	**13.1**	**11.5**	**2.915**	**33.48**	**36.44**	**49.48**
**Si**	**(1,3,7)**	**8.768**	**77.5**	**11.8**	**10.8**	**1.991**	**21.56**	**24.89**	**32.93**
	Equiv. Refl.: (3,5,5)								
**LiNbO3**	**(1,5,-10)**	**8.941**	**84.6**	**58.1**	**55.8**	**0.85**	**47.45**	**10.62**	**48.62**
	Equiv. Refl.: (5,-6,-10), (6,-5,-10), (-1,6,-10), (-5,-1,-10), (-6,1,-10)								
**LiNbO3**	**(1,-6,10)**	**8.941**	**84.6**	**56.4**	**55.8**	**0.85**	**47.45**	**10.62**	**48.62**
	Equiv. Refl.: (5,1,10), (6,-1,10), (-1,-5,10), (-5,6,10), (-6,5,10)								
**Quartz**	**(-4,6,4)**	**8.972**	**87.44**	**37.5**	**34.4**	**0.401**	**13.78**	**5.01**	**14.66**
	Equiv. Refl.: (-4,-2,-4)								
**Quartz**	**(6,-2,4)**	**8.972**	**87.44**	**37.4**	**34.4**	**0.401**	**13.77**	**5.01**	**14.65**
	Equiv. Refl.: (6,-4,-4), (-2,6,-4), (-2,-4,4)								
**Quartz**	**(4,-6,-4)**	**8.972**	**87.44**	**36.3**	**34.4**	**0.401**	**13.78**	**5.01**	**14.66**
**Quartz**	**(2,4,-4)**	**8.972**	**87.44**	**36.2**	**34.4**	**0.401**	**13.77**	**5.01**	**14.66**
	Equiv. Refl.: (2,-6,4), (4,2,4), (-6,2,-4), (-6,4,4)								
**Quartz**	**(6,-2,-4)**	**8.972**	**87.44**	**28.5**	**26.9**	**0.401**	**10.79**	**5.01**	**11.89**
	Equiv. Refl.: (6,-4,4), (-2,6,4), (-2,-4,-4), (-4,6,-4), (-4,-2,4)								
**LiNbO3**	**(2,3,14)**	**8.714**	**76**	**28.2**	**25.9**	**2.239**	**57.99**	**27.99**	**64.39**
	Equiv. Refl.: (3,-5,14), (5,-3,14), (-2,5,14), (-3,-2,14), (-5,2,14)								
**Quartz**	**(2,4,4)**	**8.972**	**87.44**	**26.8**	**26.9**	**0.401**	**10.8**	**5.01**	**11.9**
	Equiv. Refl.: (2,-6,-4), (4,2,-4), (4,-6,4), (-6,2,4), (-6,4,-4)								
**LiNbO3**	**(2,-5,-14)**	**8.714**	**76**	**26.3**	**25.9**	**2.239**	**57.99**	**27.99**	**64.39**
	Equiv. Refl.: (3,2,-14), (5,-2,-14), (-2,-3,-14), (-3,5,-14), (-5,3,-14)								
**LiNbO3**	**(1,3,16)**	**8.737**	**76.61**	**25.8**	**24.2**	**2.137**	**51.68**	**26.72**	**58.18**
	Equiv. Refl.: (3,-4,16), (4,-3,16), (-1,4,16), (-3,-1,16), (-4,1,16)								
**LiNbO3**	**(0,-6,6)**	**8.764**	**77.39**	**25.6**	**24.5**	**2.01**	**49.33**	**25.12**	**55.36**
	Equiv. Refl.: (6,0,6), (-6,6,6)								
**LiNbO3**	**(2,-7,0)**	**8.684**	**75.23**	**24.9**	**23.7**	**2.368**	**56.22**	**29.61**	**63.54**
	Equiv. Refl.: (5,2,0), (7,-2,0), (-2,-5,0), (-5,7,0), (-7,5,0)								
**LiNbO3**	**(0,6,-6)**	**8.764**	**77.39**	**24.7**	**24.5**	**2.01**	**49.33**	**25.12**	**55.36**
	Equiv. Refl.: (2,5,0), (5,-7,0), (6,-6,-6), (7,-5,0), (-2,7,0), (-5,-2,0), (-6,0,-6), (-7,2,0)								
**LiNbO3**	**(1,-4,-16)**	**8.737**	**76.61**	**24.2**	**24.2**	**2.137**	**51.68**	**26.72**	**58.18**
	Equiv. Refl.: (3,1,-16), (4,-1,-16), (-1,-3,-16), (-3,4,-16), (-4,3,-16)								
**LiNbO3**	**(2,-6,-10)**	**8.61**	**73.49**	**23.5**	**21.6**	**2.662**	**57.56**	**33.28**	**66.49**
	Equiv. Refl.: (4,2,-10), (6,-2,-10), (-2,-4,-10), (-4,6,-10), (-6,4,-10)								
**LiNbO3**	**(0,-6,-6)**	**8.764**	**77.39**	**22.6**	**22.3**	**2.01**	**44.88**	**25.12**	**51.43**
	Equiv. Refl.: (6,0,-6), (-6,6,-6)								
**LiNbO3**	**(0,6,6)**	**8.764**	**77.39**	**22.3**	**22.3**	**2.01**	**44.88**	**25.12**	**51.43**
	Equiv. Refl.: (6,-6,6), (-6,0,6)								
**LiNbO3**	**(2,4,10)**	**8.61**	**73.49**	**21.8**	**21.6**	**2.662**	**57.56**	**33.28**	**66.49**
	Equiv. Refl.: (3,-7,-2), (4,3,-2), (4,-6,10), (6,-4,10), (7,-3,-2), (-2,6,10), (-3,-4,-2), (-4,7,-2), (-4,-2,10) (-6,2,10), (-7,4,-2)								
**Quartz**	**(7,-3,-1)**	**8.936**	**84.32**	**21.3**	**19.1**	**0.893**	**17.08**	**11.16**	**20.4**
	Equiv. Refl.: (7,-4,1), (-3,7,1), (-3,-4,-1), (-4,7,-1), (-4,-3,1)								
**Quartz**	**(3,4,1)**	**8.936**	**84.32**	**21.1**	**19.1**	**0.893**	**17.08**	**11.16**	**20.4**
	Equiv. Refl.: (3,-7,-1), (4,3,-1), (4,-7,1), (-7,3,1), (-7,4,-1)								
**LiNbO3**	**(3,4,2)**	**8.505**	**71.27**	**20.7**	**20.1**	**3.044**	**61.33**	**38.05**	**72.17**
	Equiv. Refl.: (4,-7,2), (7,-4,2), (-3,7,2), (-4,-3,2), (-7,3,2)								
**Quartz**	**(7,-3,1)**	**8.936**	**84.32**	**20.4**	**18**	**0.893**	**16.1**	**11.16**	**19.59**
	Equiv. Refl.: (7,-4,-1), (-3,7,-1), (-3,-4,1), (-4,7,1), (-4,-3,-1)								

**Ei =**	**8.9805**	**keV**						**ΔEg**	**ΔEt**
**Cryst**	**Refl**	**EB**	**ΘB**	**∫ IR dΘ**	**Width**	**Ei cotΘ**	**ΔE**	**2m, 50mm**	**2m, 50mm**
	**(h,k,l)**	**[keV]**	**[°]**	**[μrad]**	**[μrad]**	**[meV/mrad]**	**[meV]**	**[meV]**	**[meV]**
**Quartz**	**(3,4,-1)**	**8.936**	**84.32**	**19.4**	**18**	**0.893**	**16.1**	**11.16**	**19.59**
	Equiv. Refl.: (3,-7,1), (4,3,1), (4,-7,-1), (-7,3,-1), (-7,4,1)								
**Sapph**	**(0,0,18)**	**8.591**	**73.07**	**18.9**	**15.8**	**2.734**	**43.24**	**34.17**	**55.12**
	Equiv. Refl.: (0,0,-18)								
**LiNbO3**	**(3,-7,4)**	**8.645**	**74.29**	**18.7**	**18.5**	**2.526**	**46.66**	**31.57**	**56.34**
	Equiv. Refl.: (4,3,4), (7,-3,4), (-3,-4,4), (-4,7,4), (-7,4,4)								
**LiNbO3**	**(3,4,-4)**	**8.645**	**74.29**	**18.5**	**18.5**	**2.526**	**46.66**	**31.57**	**56.34**
	Equiv. Refl.: (4,-7,-4), (7,-4,-4), (-3,7,-4), (-4,-3,-4), (-7,3,-4)								
**LiNbO3**	**(1,5,8)**	**8.528**	**71.74**	**18.4**	**17.9**	**2.962**	**52.93**	**37.03**	**64.59**
	Equiv. Refl.: (5,-6,8), (6,-5,8), (-1,6,8), (-5,-1,8), (-6,1,8), (0,5,10), (0,-5,-10), (5,0,-10), (5,-5,10) (-5,0,10), (-5,5,-10)								
**LiNbO3**	**(1,-6,-8)**	**8.528**	**71.74**	**18.3**	**17.9**	**2.962**	**52.93**	**37.03**	**64.59**
	Equiv. Refl.: (5,1,-8), (6,-1,-8), (-1,-5,-8), (-5,6,-8), (-6,5,-8)								
**Quartz**	**(1,2,-7)**	**8.907**	**82.64**	**16.1**	**14.5**	**1.16**	**16.77**	**14.5**	**22.17**
	Equiv. Refl.: (1,-3,7), (2,1,7), (2,-3,-7), (-3,1,-7), (-3,2,7)								
**Quartz**	**(3,-1,7)**	**8.907**	**82.64**	**16**	**14.5**	**1.16**	**16.76**	**14.5**	**22.16**
	Equiv. Refl.: (3,-2,-7), (-1,3,-7), (-1,-2,7), (-2,3,7), (-2,-1,-7)								
**Quartz**	**(6,-1,3)**	**8.812**	**78.88**	**15.8**	**13.5**	**1.766**	**23.9**	**22.07**	**32.53**
	Equiv. Refl.: (6,-5,-3), (-1,6,-3), (-1,-5,3), (-5,6,3), (-5,-1,-3)								
**Quartz**	**(1,5,-3)**	**8.812**	**78.88**	**15.6**	**13.5**	**1.766**	**23.9**	**22.07**	**32.53**
	Equiv. Refl.: (1,-6,3), (5,1,3), (5,-6,-3), (-6,1,-3), (-6,5,3)								
**Quartz**	**(2,3,-5)**	**8.557**	**72.34**	**14.4**	**12**	**2.86**	**34.27**	**35.75**	**49.52**
	Equiv. Refl.: (2,-5,5), (3,2,5), (3,-5,-5), (-5,2,-5), (-5,3,5)								
**Quartz**	**(5,-2,5)**	**8.557**	**72.34**	**14.3**	**12**	**2.86**	**34.28**	**35.75**	**49.53**
	Equiv. Refl.: (5,-3,-5), (-2,5,-5), (-2,-3,5), (-3,5,5), (-3,-2,-5)								
**Quartz**	**(1,2,7)**	**8.907**	**82.64**	**14.1**	**12.6**	**1.16**	**14.59**	**14.5**	**20.57**
	Equiv. Refl.: (1,-3,-7), (2,1,-7), (2,-3,7), (-3,1,7), (-3,2,-7)								
**Sapph**	**(1,5,-4)**	**8.591**	**73.07**	**14**	**12.1**	**2.734**	**33.06**	**34.18**	**47.55**
	Equiv. Refl.: (1,-6,4), (5,1,4), (5,-6,-4), (6,-1,4), (6,-5,-4), (-1,6,-4), (-1,-5,4), (-5,6,4), (-5,-1,-4) (-6,1,-4), (-6,5,4)								
**Quartz**	**(1,4,5)**	**8.802**	**78.55**	**13.8**	**11.7**	**1.819**	**21.28**	**22.74**	**31.14**
	Equiv. Refl.: (1,-5,-5), (4,1,-5), (4,-5,5), (5,-1,-5), (5,-4,5), (-1,5,5), (-1,-4,-5), (-4,5,-5), (-4,-1,5) (-5,1,5), (-5,4,-5)								
**Quartz**	**(3,-2,7)**	**8.907**	**82.64**	**13.4**	**12.6**	**1.16**	**14.58**	**14.5**	**20.56**
	Equiv. Refl.: (-1,3,7), (-1,-2,-7), (-2,3,-7), (-2,-1,7)								
**Quartz**	**(3,-1,-7)**	**8.907**	**82.64**	**13.3**	**12.6**	**1.16**	**14.58**	**14.5**	**20.56**
**Quartz**	**(0,6,-1)**	**8.817**	**79.04**	**13.1**	**11.3**	**1.738**	**19.56**	**21.73**	**29.23**
	Equiv. Refl.: (0,-6,1), (6,0,1), (6,-6,-1), (-6,0,-1), (-6,6,1)								
**Sapph**	**(2,2,15)**	**8.855**	**80.42**	**13**	**12.1**	**1.516**	**18.35**	**18.94**	**26.38**
	Equiv. Refl.: (2,2,-15), (2,-4,15), (2,-4,-15), (4,-2,15), (4,-2,-15), (-2,4,15), (-2,4,-15), (-2,-2,15), (-2,-2,-15) (-4,2,15), (-4,2,-15)								
**Quartz**	**(0,5,4)**	**8.609**	**73.47**	**12.9**	**10.9**	**2.665**	**29.16**	**33.32**	**44.28**
	Equiv. Refl.: (0,-5,-4), (5,0,-4), (5,-5,4), (-5,0,4), (-5,5,-4)								
**Quartz**	**(6,-3,4)**	**8.852**	**80.31**	**10.7**	**9.5**	**1.533**	**14.59**	**19.16**	**24.08**
	Equiv. Refl.: (6,-3,-4), (-3,6,4), (-3,6,-4), (-3,-3,4), (-3,-3,-4)								
**Quartz**	**(3,3,4)**	**8.852**	**80.31**	**10.1**	**9.5**	**1.533**	**14.59**	**19.16**	**24.08**
	Equiv. Refl.: (3,3,-4), (3,-6,4), (3,-6,-4), (-6,3,4), (-6,3,-4)								
**Quartz**	**(6,-1,-3)**	**8.812**	**78.88**	**10**	**8.8**	**1.766**	**15.53**	**22.07**	**26.99**
	Equiv. Refl.: (6,-5,3), (-1,6,3), (-1,-5,-3), (-5,6,-3), (-5,-1,3)								
**Quartz**	**(1,5,3)**	**8.812**	**78.88**	**9.6**	**8.8**	**1.766**	**15.53**	**22.07**	**26.99**
	Equiv. Refl.: (1,-6,-3), (5,1,-3), (5,-6,3), (-6,1,3), (-6,5,-3)								

Ei =	8.9805	keV						ΔEg	ΔEt
Cryst	Refl	EB	ΘB	∫ IR dΘ	Width	Ei cotΘ	ΔE	2m, 50mm	2m, 50mm
	(h,k,l)	[keV]	[°]	[μrad]	[μrad]	[meV/mrad]	[meV]	[meV]	[meV]
**Sapph**	**(1,2,-16)**	**8.612**	**73.52**	**9.3**	**8.4**	**2.656**	**22.24**	**33.2**	**39.96**
	Equiv. Refl.: (1,-3,16), (2,1,16), (2,-3,-16), (3,-1,16), (3,-2,-16), (-1,3,-16), (-1,-2,16), (-2,3,16), (-2,-1,-16) (-3,1,-16), (-3,2,16)								
**Sapph**	**(1,3,-14)**	**8.607**	**73.41**	**9.1**	**8.1**	**2.676**	**21.7**	**33.45**	**39.87**
	Equiv. Refl.: (1,-4,14), (3,1,14), (3,-4,-14), (4,-1,14), (4,-3,-14), (-1,4,-14), (-1,-3,14), (-3,4,14), (-3,-1,-14) (-4,1,-14), (-4,3,14)								
**Quartz**	**(2,2,6)**	**8.534**	**71.87**	**7.4**	**6.4**	**2.941**	**18.85**	**36.76**	**41.32**
	Equiv. Refl.: (2,2,-6), (2,-4,6), (2,-4,-6), (-4,2,6), (-4,2,-6)								
**Quartz**	**(4,-2,6)**	**8.534**	**71.87**	**7.3**	**6.4**	**2.941**	**18.84**	**36.76**	**41.31**
	Equiv. Refl.: (4,-2,-6), (-2,4,6), (-2,4,-6), (-2,-2,6), (-2,-2,-6)								
**Sapph**	**(1,1,18)**	**8.978**	**88.57**	**6.9**	**13.5**	**0.224**	**3.01**	**2.8**	**4.11**
	Equiv. Refl.: (1,1,-18), (1,-2,18), (1,-2,-18), (2,-1,18), (2,-1,-18), (-1,2,18), (-1,2,-18), (-1,-1,18), (-1,-1,-18) (-2,1,18), (-2,1,-18)								
**Sapph**	**(1,4,12)**	**8.963**	**86.41**	**6.4**	**9**	**0.563**	**5.07**	**7.04**	**8.68**
	Equiv. Refl.: (1,4,-12), (1,-5,12), (1,-5,-12), (4,1,12), (4,1,-12), (4,-5,12), (4,-5,-12), (5,-1,12), (5,-1,-12) (5,-4,12), (5,-4,-12), (-1,5,12), (-1,5,-12), (-1,-4,12), (-1,-4,-12), (-4,5,12), (-4,5,-12), (-4,-1,12) (-4,-1,-12), (-5,1,12), (-5,1,-12), (-5,4,12), (-5,4,-12)								
**Sapph**	**(2,4,-8)**	**8.829**	**79.46**	**6.3**	**6.5**	**1.671**	**10.89**	**20.88**	**23.55**
	Equiv. Refl.: (2,-6,8), (4,2,8), (4,-6,-8), (6,-2,8), (6,-4,-8), (-2,6,-8), (-2,-4,8), (-4,6,8), (-4,-2,-8) (-6,2,-8), (-6,4,8)								
**Sapph**	**(1,5,5)**	**8.71**	**75.89**	**6.2**	**6**	**2.257**	**13.57**	**28.22**	**31.31**
	Equiv. Refl.: (1,-6,-5), (5,1,-5), (5,-6,5), (6,-1,-5), (6,-5,5), (-1,6,5), (-1,-5,-5), (-5,6,-5), (-5,-1,5) (-6,1,5), (-6,5,-5)								
**LiNbO3**	**(2,4,-11)**	**8.851**	**80.24**	**5.7**	**9.3**	**1.544**	**14.42**	**19.3**	**24.09**
	Equiv. Refl.: (2,-6,11), (4,2,11), (4,-6,-11), (6,-2,11), (6,-4,-11), (-2,6,-11), (-2,-4,11), (-4,6,11), (-4,-2,-11) (-6,2,-11), (-6,4,11)								
**Quartz**	**(6,-2,3)**	**8.443**	**70.07**	**5.6**	**4.6**	**3.256**	**15**	**40.7**	**43.38**
	Equiv. Refl.: (6,-4,-3), (-2,6,-3), (-2,-4,3), (-3,5,-5), (-3,-2,5), (-4,6,3), (-4,-2,-3)								
**Quartz**	**(4,-1,6)**	**8.658**	**74.6**	**5.5**	**5**	**2.474**	**12.34**	**30.93**	**33.3**
	Equiv. Refl.: (4,-3,-6), (5,-2,-5), (5,-3,5), (-1,4,-6), (-1,-3,6), (-2,5,5), (-2,-3,-5), (-3,4,6), (-3,-1,-6)								
**Quartz**	**(2,3,5)**	**8.557**	**72.34**	**5.4**	**5**	**2.86**	**14.18**	**35.75**	**38.45**
	Equiv. Refl.: (2,-5,-5), (3,2,-5), (3,-5,5), (-5,2,5), (-5,3,-5)								
**Quartz**	**(1,3,-6)**	**8.658**	**74.6**	**5.3**	**5**	**2.474**	**12.35**	**30.93**	**33.3**
	Equiv. Refl.: (1,-4,6), (2,4,3), (2,-6,-3), (3,1,6), (3,-4,-6), (4,2,-3), (4,-6,3), (-4,1,-6), (-4,3,6) (-6,2,3), (-6,4,-3)								
**Quartz**	**(6,-2,-3)**	**8.443**	**70.07**	**5**	**4.6**	**3.256**	**14.89**	**40.7**	**43.34**
	Equiv. Refl.: (6,-4,3), (-2,6,3), (-2,-4,-3), (-4,6,-3), (-4,-2,3)								
**Quartz**	**(4,2,3)**	**8.443**	**70.07**	**4.8**	**4.6**	**3.256**	**15.01**	**40.7**	**43.38**
	Equiv. Refl.: (4,-6,-3), (-6,4,3)								
**Quartz**	**(0,2,-7)**	**8.542**	**72.01**	**4.7**	**4.3**	**2.916**	**12.67**	**36.45**	**38.59**
	Equiv. Refl.: (0,-2,7), (2,0,7), (2,4,-3), (2,-2,-7), (2,-6,3), (-2,0,-7), (-2,2,7), (-6,2,-3)								
**Quartz**	**(0,2,7)**	**8.542**	**72.01**	**4.1**	**3.9**	**2.916**	**11.37**	**36.45**	**38.18**
	Equiv. Refl.: (0,-2,-7), (2,0,-7), (2,-2,7), (-2,0,7), (-2,2,-7)								
**Sapph**	**(2,4,7)**	**8.633**	**74.02**	**4**	**4.1**	**2.572**	**10.45**	**32.15**	**33.81**
	Equiv. Refl.: (2,-6,-7), (4,2,-7), (4,-6,7), (6,-2,-7), (6,-4,7), (-2,6,7), (-2,-4,-7), (-4,6,-7), (-4,-2,7) (-6,2,7), (-6,4,-7), (1,3,6), (1,-4,-6), (3,1,-6), (3,-4,6), (-4,1,6), (-4,3,-6)								
**Quartz**	**(4,-1,-6)**	**8.658**	**74.6**	**3.8**	**3.8**	**2.474**	**9.45**	**30.93**	**32.34**
	Equiv. Refl.: (4,-3,6), (-1,4,6), (-1,-3,-6), (-3,4,-6), (-3,-1,6)								

Ei = Cryst	8.9805 Refl (h,k,l)	keV EB [keV]	ΘB [°]	∫ IR dΘ [µrad]	Width [µrad]	Ei cotΘ [meV/mrad]	ΔE [meV]	ΔEg 2m, 50mm [meV]	ΔEt 2m, 50mm [meV]
**Quartz**	**(7,-3,0)**	**8.862**	**80.7**	**2.5**	**3.2**	**1.47**	**4.75**	**18.38**	**18.98**
	Equiv. Refl.: (7,-4,0), (-3,7,0), (-3,-4,0), (-4,7,0), (-4,-3,0)								
**LiNbO3**	**(3,4,5)**	**8.749**	**76.95**	**2.4**	**5.1**	**2.082**	**10.67**	**26.02**	**28.12**
	Equiv. Refl.: (3,-7,-5), (4,3,-5), (4,-7,5), (7,-3,-5), (7,-4,5), (-3,7,5), (-3,-4,-5), (-4,7,-5), (-4,-3,5) (-7,3,5), (-7,4,-5)								
**Quartz**	**(3,4,0)**	**8.862**	**80.7**	**2.1**	**3.2**	**1.47**	**4.75**	**18.38**	**18.98**
	Equiv. Refl.: (3,-7,0), (4,3,0), (4,-7,0), (-7,3,0), (-7,4,0)								
**Quartz**	**(0,6,1)**	**8.817**	**79.04**	**2**	**2.8**	**1.738**	**4.85**	**21.73**	**22.26**
	Equiv. Refl.: (0,-6,-1), (6,0,-1), (6,-6,1), (-6,0,1), (-6,6,-1)								
**LiNbO3**	**(3,4,-1)**	**8.47**	**70.58**	**1.8**	**3.8**	**3.165**	**11.89**	**39.57**	**41.31**
	Equiv. Refl.: (3,-7,1), (4,3,1), (4,-7,-1), (7,-3,1), (7,-4,-1), (-3,7,-1), (-3,-4,1), (-4,7,1), (-4,-3,-1) (-7,3,-1), (-7,4,1)								
**Quartz**	**(1,4,-5)**	**8.802**	**78.55**	**1.7**	**2.2**	**1.819**	**4.06**	**22.74**	**23.1**
	Equiv. Refl.: (1,-5,5), (4,1,5), (4,-5,-5), (-5,1,-5), (-5,4,5)								
**Quartz**	**(0,6,0)**	**8.742**	**76.76**	**1.6**	**2.3**	**2.112**	**4.91**	**26.4**	**26.86**
	Equiv. Refl.: (0,-6,0), (6,0,0), (6,-6,0), (-6,0,0), (-6,6,0)								
**Quartz**	**(5,-1,5)**	**8.802**	**78.55**	**1.3**	**2.2**	**1.819**	**4.07**	**22.74**	**23.1**
	Equiv. Refl.: (5,-4,-5), (-1,5,-5), (-1,-4,5), (-4,5,5), (-4,-1,-5)								
**Quartz**	**(0,5,-4)**	**8.609**	**73.47**	**1.2**	**1.7**	**2.665**	**4.64**	**33.32**	**33.64**
	Equiv. Refl.: (0,-5,4), (5,0,4), (5,-5,-4), (-5,0,-4), (-5,5,4)								
**LiNbO3**	**(1,2,17)**	**8.445**	**70.12**	**1.1**	**2.8**	**3.247**	**9.06**	**40.59**	**41.59**
	Equiv. Refl.: (1,-3,-17), (2,1,-17), (2,-3,17), (3,-1,-17), (3,-2,17), (-1,3,17), (-1,-2,-17), (-2,3,-17), (-2,-1,17) (-3,1,17), (-3,2,-17)								
**LiNbO3**	**(2,5,3)**	**8.787**	**78.07**	**1**	**3.2**	**1.897**	**6.16**	**23.71**	**24.5**
	Equiv. Refl.: (2,5,-3), (2,-7,3), (2,-7,-3), (5,2,3), (5,2,-3), (5,-7,3), (5,-7,-3), (7,-2,3), (7,-2,-3) (7,-5,3), (7,-5,-3), (-2,7,3), (-2,7,-3), (-2,-5,3), (-2,-5,-3), (-5,7,3), (-5,7,-3), (-5,-2,3) (-5,-2,-3), (-7,2,3), (-7,2,-3), (-7,5,3), (-7,5,-3)								
**Sapph**	**(3,3,9)**	**8.92**	**83.33**	**0.2**	**0.9**	**1.05**	**0.97**	**13.13**	**13.17**
	Equiv. Refl.: (3,3,-9), (3,-6,9), (3,-6,-9), (6,-3,9), (6,-3,-9), (-3,6,9), (-3,6,-9), (-3,-3,9), (-3,-3,-9) (-6,3,9), (-6,3,-9)								

Ei =	8.048	keV						ΔEg	ΔEt
Cryst	Refl (h,k,l)	EB [keV]	ΘB [°]	∫ IR dΘ [µrad]	Width [µrad]	Ei cotΘ [meV/µrad]	ΔE [meV]	2m, 50µm [meV]	2m, 50µm [meV]
Ge	(4,4,4)	7.592	70.62	33.6915	30.92	2.8317	87.56	35.4	94.44
Si	(4,4,4)	7.908	79.3	29.1381	25.94	1.5202	39.44	19	43.78
Ge	(1,1,7)	7.825	76.49	28.8222	28.8	1.9331	55.68	24.16	60.69
	Equiv. Refl.: (1,5,5), (1,7,1), (5,1,5), (5,5,1), (7,1,1)								
LiNbO3	(1,2,-16)	8.045	88.5	278.608	275.3	0.211	58.15	2.64	58.21
	Equiv. Refl.: (-1,3,-16), (-2,-1,-16), (-3,1,-16)								
LiNbO3	(2,-3,-16)	8.045	88.5	278.607	275.3	0.211	58.15	2.64	58.21
	Equiv. Refl.: (3,-2,-16)								
LiNbO3	(1,-3,16)	8.045	88.5	276.559	275.3	0.211	58.15	2.64	58.21
	Equiv. Refl.: (3,-1,16)								
LiNbO3	(2,1,16)	8.045	88.5	276.558	275.3	0.211	58.15	2.64	58.21
	Equiv. Refl.: (-1,-2,16)								
LiNbO3	(-2,3,16)	8.045	88.5	276.557	275.3	0.211	58.15	2.64	58.21
	Equiv. Refl.: (-3,2,16)								
Quartz	(4,2,-2)	8.044	88.13	110.601	97.5	0.263	25.65	3.29	25.86
Quartz	(4,-6,2)	8.044	88.13	110.595	97.5	0.263	25.64	3.29	25.85
Quartz	(-6,2,2)	8.044	88.13	110.591	97.5	0.263	25.64	3.29	25.85
Quartz	(-6,4,-2)	8.044	88.13	110.589	97.5	0.263	25.64	3.29	25.85
Quartz	(2,4,2)	8.044	88.13	110.587	97.5	0.263	25.64	3.29	25.85
	Equiv. Refl.: (2,-6,-2)								
Quartz	(-4,-2,2)	8.044	88.13	108.375	97.5	0.263	25.65	3.29	25.86
Quartz	(-4,6,-2)	8.044	88.13	108.368	97.5	0.263	25.64	3.29	25.85
Quartz	(-2,6,2)	8.044	88.13	108.363	97.5	0.263	25.64	3.29	25.85
	Equiv. Refl.: (-2,-4,-2)								
Quartz	(6,-2,-2)	8.044	88.13	108.362	97.5	0.263	25.64	3.29	25.85
Quartz	(6,-4,2)	8.044	88.13	108.36	97.5	0.263	25.64	3.29	25.85
LiNbO3	(1,3,-14)	8.021	85.26	95.329	90.5	0.667	60.35	8.34	60.92
	Equiv. Refl.: (3,-4,-14), (4,-3,-14), (-1,4,-14), (-3,-1,-14), (-4,1,-14)								
LiNbO3	(1,-4,14)	8.021	85.26	88.177	90.5	0.667	60.35	8.34	60.92
	Equiv. Refl.: (3,1,14), (4,-1,14), (-1,-3,14)								
LiNbO3	(-3,4,14)	8.021	85.26	88.176	90.5	0.667	60.35	8.34	60.92
	Equiv. Refl.: (-4,3,14)								
LiNbO3	(1,-6,4)	7.946	80.86	52.578	50.3	1.295	65.1	16.18	67.08
	Equiv. Refl.: (5,1,4), (6,-1,4), (-1,-5,4), (-5,6,4), (-6,5,4)								
LiNbO3	(1,5,-4)	7.946	80.86	50.474	50.3	1.295	65.1	16.18	67.08
	Equiv. Refl.: (5,-6,-4), (6,-5,-4), (-1,6,-4), (-5,-1,-4), (-6,1,-4)								
LiNbO3	(0,5,-8)	7.819	76.29	44.14	40.2	1.963	78.87	24.53	82.6
	Equiv. Refl.: (5,-5,-8), (-5,0,-8)								
Quartz	(6,-4,-2)	8.044	88.13	40.791	40.9	0.263	10.75	3.29	11.24
Quartz	(6,-2,2)	8.044	88.13	40.788	40.9	0.263	10.75	3.29	11.24
	Equiv. Refl.: (-2,6,-2)								
Quartz	(-2,-4,2)	8.044	88.13	40.786	40.9	0.263	10.75	3.29	11.24
Quartz	(-4,-2,-2)	8.044	88.13	40.757	40.8	0.263	10.74	3.29	11.23
Quartz	(-4,6,2)	8.044	88.13	40.754	40.8	0.263	10.74	3.29	11.23
LiNbO3	(0,-5,8)	7.819	76.29	40.67	40.2	1.963	78.87	24.53	82.6
	Equiv. Refl.: (5,0,8), (-5,5,8)								
Sapph	(3,3,0)	7.817	76.25	38.819	32.3	1.97	63.54	24.63	68.14
	Equiv. Refl.: (3,-6,0), (6,-3,0), (-3,6,0), (-3,-3,0), (-6,3,0)								

Ei =	8.048	keV						ΔEg	ΔEt
Cryst	Refl	EB	ΘB	∫ IR dΘ	Width	Ei cotΘ	ΔE	2m, 50µm	2m, 50µm
	(h,k,l)	[keV]	[°]	[µrad]	[µrad]	[meV/µrad]	[meV]	[meV]	[meV]
Quartz	(-6,4,2)	8.044	88.13	36.002	40.9	0.263	10.75	3.29	11.24
Quartz	(2,-6,2)	8.044	88.13	36	40.9	0.263	10.75	3.29	11.24
Quartz	(-6,2,-2)	8.044	88.13	35.999	40.9	0.263	10.75	3.29	11.24
Quartz	(2,4,-2)	8.044	88.13	35.997	40.9	0.263	10.75	3.29	11.24
Quartz	(4,2,2)	8.044	88.13	35.972	40.8	0.263	10.74	3.29	11.23
Quartz	(4,-6,-2)	8.044	88.13	35.97	40.8	0.263	10.74	3.29	11.23
LiNbO3	(1,5,2)	7.793	75.55	31.15	30.4	2.074	63.15	25.92	68.26
	Equiv. Refl.: (5,-6,2), (6,-5,2), (-1,6,2), (-5,-1,2), (-6,1,2)								
LiNbO3	(1,-6,-2)	7.793	75.55	30.419	30.4	2.074	63.15	25.92	68.26
	Equiv. Refl.: (5,1,-2), (6,-1,-2), (-1,-5,-2), (-5,6,-2), (-6,5,-2)								
LiNbO3	(0,-2,-16)	7.676	72.52	26.275	25	2.535	63.4	31.68	70.87
	Equiv. Refl.: (2,0,-16), (-2,2,-16)								
Sapph	(0,4,-10)	7.681	72.62	26.161	22.1	2.519	55.62	31.48	63.92
	Equiv. Refl.: (0,-4,10), (4,0,10), (4,-4,-10), (-4,0,-10), (-4,4,10)								
LiNbO3	(0,2,16)	7.676	72.52	24.021	25	2.535	63.4	31.68	70.87
	Equiv. Refl.: (2,-2,16), (-2,0,16)								
LiNbO3	(3,3,6)	7.707	73.27	23.381	23.2	2.419	56.17	30.24	63.8
	Equiv. Refl.: (3,-6,6), (6,-3,6), (-3,6,6), (-3,-3,6), (-6,3,6)								
Quartz	(2,3,4)	7.835	76.78	23.355	19.7	1.89	37.24	23.63	44.11
	Equiv. Refl.: (2,-5,-4)								
Quartz	(3,2,-4)	7.835	76.78	23.352	19.7	1.89	37.24	23.63	44.1
Quartz	(3,-5,4)	7.835	76.78	23.351	19.7	1.89	37.24	23.63	44.1
Quartz	(-5,2,4)	7.835	76.78	23.35	19.7	1.89	37.24	23.63	44.1
	Equiv. Refl.: (-5,3,-4)								
Quartz	(0,5,-2)	7.638	71.62	23.131	19.2	2.674	51.29	33.42	61.22
	Equiv. Refl.: (0,-5,2)								
Quartz	(5,0,2)	7.638	71.62	23.129	19.2	2.674	51.29	33.42	61.21
	Equiv. Refl.: (5,-5,-2), (-5,0,-2), (-5,5,2)								
LiNbO3	(2,4,4)	7.572	70.2	23.06	22.1	2.898	64.11	36.22	73.64
	Equiv. Refl.: (4,-6,4), (6,-4,4), (-2,6,4), (-4,-2,4), (-6,2,4)								
Quartz	(-2,-3,-4)	7.835	76.78	22.928	19.7	1.89	37.24	23.63	44.11
Quartz	(-2,5,4)	7.835	76.78	22.927	19.7	1.89	37.24	23.63	44.11
Quartz	(-3,-2,4)	7.835	76.78	22.926	19.7	1.89	37.24	23.63	44.1
Quartz	(-3,5,-4)	7.835	76.78	22.925	19.7	1.89	37.24	23.63	44.1
Sapph	(0,5,4)	7.761	74.64	22.924	19.8	2.21	43.86	27.63	51.83
	Equiv. Refl.: (0,-5,-4), (5,0,-4), (5,-5,4), (-5,0,4), (-5,5,-4), (5,-2,-4), (5,-3,4)								
LiNbO3	(3,3,-6)	7.707	73.27	22.205	23.2	2.419	56.17	30.24	63.8
	Equiv. Refl.: (3,-6,-6), (6,-3,-6), (-3,6,-6), (-3,-3,-6), (-6,3,-6)								
LiNbO3	(4,2,-4)	7.572	70.2	21.257	22.1	2.898	64.11	36.22	73.64
	Equiv. Refl.: (-2,-4,-4), (-4,6,-4), (-6,4,-4)								
LiNbO3	(2,-6,-4)	7.572	70.2	21.256	22.1	2.898	64.11	36.22	73.64
	Equiv. Refl.: (6,-2,-4)								
Sapph	(1,2,14)	7.778	75.11	20.06	17.7	2.14	37.83	26.75	46.33
	Equiv. Refl.: (1,-3,-14), (2,1,-14), (2,-3,14), (3,-1,-14), (3,-2,14), (-1,3,14), (-1,-2,-14), (-2,3,-14), (-2,-1,14) (-3,1,14), (-3,2,-14)								
Quartz	(2,1,-6)	7.888	78.56	17.485	15.3	1.628	24.89	20.35	32.15
Quartz	(1,2,6)	7.888	78.56	17.482	15.3	1.628	24.89	20.35	32.15
Quartz	(1,-3,-6)	7.888	78.56	17.481	15.3	1.628	24.89	20.35	32.15
	Equiv. Refl.: (2,-3,6)								

**Ei =**	**8.048**	**keV**						**ΔEg**	**ΔEt**
**Cryst**	**Refl**	**EB**	**ΘB**	**∫ IR dΘ**	**Width**	**Ei cotΘ**	**ΔE**	**2m, 50µm**	**2m, 50µm**
	**(h,k,l)**	**[keV]**	**[°]**	**[µrad]**	**[µrad]**	**[meV/µrad]**	**[meV]**	**[meV]**	**[meV]**
**Quartz**	**(-3,1,6)**	**7.888**	**78.56**	**17.471**	**15.3**	**1.628**	**24.88**	**20.35**	**32.14**
	Equiv. Refl.: (-3,2,-6)								
**Quartz**	**(-2,-1,6)**	**7.888**	**78.56**	**17.109**	**15.3**	**1.628**	**24.89**	**20.35**	**32.15**
**Quartz**	**(-1,-2,-6)**	**7.888**	**78.56**	**17.107**	**15.3**	**1.628**	**24.89**	**20.35**	**32.15**
**Quartz**	**(-1,3,6)**	**7.888**	**78.56**	**17.105**	**15.3**	**1.628**	**24.89**	**20.35**	**32.15**
	Equiv. Refl.: (-2,3,-6)								
**Quartz**	**(3,-1,-6)**	**7.888**	**78.56**	**17.095**	**15.3**	**1.628**	**24.88**	**20.35**	**32.14**
	Equiv. Refl.: (3,-2,6)								
**Quartz**	**(2,4,0)**	**7.71**	**73.33**	**15.141**	**12.7**	**2.41**	**30.66**	**30.13**	**42.98**
	Equiv. Refl.: (2,-6,0), (4,2,0), (4,-6,0), (-6,2,0), (-6,4,0)								
**Quartz**	**(6,-2,0)**	**7.71**	**73.33**	**14.71**	**12.7**	**2.41**	**30.66**	**30.13**	**42.98**
	Equiv. Refl.: (6,-4,0), (-2,6,0), (-2,-4,0), (-4,6,0), (-4,-2,0)								
**Sapph**	**(1,1,15)**	**7.619**	**71.2**	**12.386**	**11.3**	**2.739**	**30.91**	**34.24**	**46.12**
	Equiv. Refl.: (1,1,-15), (1,-2,15), (1,-2,-15), (2,-1,15), (2,-1,-15), (-1,2,15), (-1,2,-15), (-1,-1,15), (-1,-1,-15) (-2,1,15), (-2,1,-15)								
**Quartz**	**(2,2,5)**	**7.64**	**71.67**	**11.53**	**10.1**	**2.666**	**26.82**	**33.33**	**42.78**
	Equiv. Refl.: (2,-4,-5)								
**Quartz**	**(2,2,-5)**	**7.64**	**71.67**	**11.529**	**10.1**	**2.666**	**26.82**	**33.33**	**42.78**
**LiNbO3**	**(2,4,7)**	**7.996**	**83.48**	**11.527**	**19.8**	**0.92**	**18.17**	**11.5**	**21.5**
	Equiv. Refl.: (2,-6,-7), (4,2,-7), (4,-6,7), (6,-2,-7), (6,-4,7), (-2,6,7), (-2,-4,-7), (-4,6,-7), (-4,-2,7) (-6,2,7), (-6,4,-7), (2,-4,5)								
**Quartz**	**(-4,2,5)**	**7.64**	**71.67**	**11.526**	**10.1**	**2.666**	**26.81**	**33.33**	**42.77**
**Quartz**	**(-4,2,-5)**	**7.64**	**71.67**	**11.525**	**10.1**	**2.666**	**26.81**	**33.33**	**42.77**
**Quartz**	**(-2,4,5)**	**7.64**	**71.67**	**11.259**	**10.1**	**2.666**	**26.82**	**33.33**	**42.78**
	Equiv. Refl.: (-2,-2,-5)								
**Quartz**	**(-2,-2,5)**	**7.64**	**71.67**	**11.258**	**10.1**	**2.666**	**26.82**	**33.33**	**42.78**
**Quartz**	**(-2,4,-5)**	**7.64**	**71.67**	**11.256**	**10.1**	**2.666**	**26.82**	**33.33**	**42.78**
**Quartz**	**(4,-2,5)**	**7.64**	**71.67**	**11.254**	**10.1**	**2.666**	**26.81**	**33.33**	**42.77**
	Equiv. Refl.: (4,-2,-5)								
**Sapph**	**(0,1,-16)**	**7.783**	**75.27**	**10.905**	**10.4**	**2.116**	**22.05**	**26.45**	**34.44**
	Equiv. Refl.: (0,-1,16), (1,0,16), (1,-1,-16), (-1,0,-16), (-1,1,16)								
**Quartz**	**(3,3,1)**	**7.657**	**72.07**	**10.604**	**9.3**	**2.604**	**24.2**	**32.55**	**40.56**
	Equiv. Refl.: (3,-6,-1), (-6,3,1), (-6,3,-1)								
**Quartz**	**(3,3,-1)**	**7.657**	**72.07**	**10.602**	**9.3**	**2.604**	**24.2**	**32.55**	**40.56**
	Equiv. Refl.: (3,-6,1)								
**Sapph**	**(2,4,-2)**	**8.018**	**85.02**	**10.432**	**13**	**0.701**	**9.08**	**8.76**	**12.62**
	Equiv. Refl.: (2,-6,2), (4,2,2), (4,-6,-2), (6,-2,2), (6,-4,-2), (-2,6,-2), (-2,-4,2), (-4,6,2), (-4,-2,-2) (-6,2,-2), (-6,4,2)								
**Quartz**	**(6,-3,0)**	**7.571**	**70.17**	**10.391**	**9.1**	**2.902**	**26.29**	**36.28**	**44.8**
	Equiv. Refl.: (-3,6,0), (-3,-3,0)								
**Quartz**	**(3,3,0)**	**7.571**	**70.17**	**10.329**	**9.1**	**2.902**	**26.29**	**36.28**	**44.8**
	Equiv. Refl.: (3,-6,0), (-6,3,0)								
**Quartz**	**(6,-3,1)**	**7.657**	**72.07**	**10.107**	**9.3**	**2.604**	**24.2**	**32.55**	**40.56**
	Equiv. Refl.: (6,-3,-1), (-3,6,1), (-3,-3,-1)								
**Quartz**	**(-3,6,-1)**	**7.657**	**72.07**	**10.105**	**9.3**	**2.604**	**24.2**	**32.55**	**40.56**
	Equiv. Refl.: (-3,-3,1)								
**Sapph**	**(2,4,1)**	**7.975**	**82.27**	**10.099**	**11.4**	**1.092**	**12.43**	**13.65**	**18.46**
	Equiv. Refl.: (2,-6,-1), (4,2,-1), (4,-6,1), (6,-2,-1), (6,-4,1), (-2,6,1), (-2,-4,-1), (-4,6,-1), (-4,-2,1) (-6,2,1), (-6,4,-1)								
**Quartz**	**(5,-2,4)**	**7.835**	**76.78**	**9.609**	**9.1**	**1.89**	**17.23**	**23.63**	**29.25**
	Equiv. Refl.: (5,-3,-4)								

Ei =	8.048	keV						ΔEg	ΔEt
**Cryst**	**Refl (h,k,l)**	**EB [keV]**	**ΘB [°]**	**∫IR dΘ [µrad]**	**Width [µrad]**	**Ei cotΘ [meV/µrad]**	**ΔE [meV]**	**2m, 50µm [meV]**	**2m, 50µm [meV]**
**Quartz**	**(-2,5,-4)**	**7.835**	**76.78**	**9.599**	**9.1**	**1.89**	**17.22**	**23.63**	**29.24**
	Equiv. Refl.: (-2,-3,4)								
**Quartz**	**(-3,-2,-4)**	**7.835**	**76.78**	**9.596**	**9.1**	**1.89**	**17.21**	**23.63**	**29.23**
**Quartz**	**(-3,5,4)**	**7.835**	**76.78**	**9.595**	**9.1**	**1.89**	**17.21**	**23.63**	**29.23**
**Quartz**	**(-5,3,4)**	**7.835**	**76.78**	**9.446**	**9.1**	**1.89**	**17.23**	**23.63**	**29.25**
**Quartz**	**(-5,2,-4)**	**7.835**	**76.78**	**9.445**	**9.1**	**1.89**	**17.23**	**23.63**	**29.25**
**Quartz**	**(2,3,-4)**	**7.835**	**76.78**	**9.436**	**9.1**	**1.89**	**17.22**	**23.63**	**29.24**
	Equiv. Refl.: (2,-5,4)								
**Quartz**	**(3,2,4)**	**7.835**	**76.78**	**9.433**	**9.1**	**1.89**	**17.21**	**23.63**	**29.23**
**Quartz**	**(3,-5,-4)**	**7.835**	**76.78**	**9.431**	**9.1**	**1.89**	**17.21**	**23.63**	**29.23**
**Quartz**	**(4,2,-1)**	**7.795**	**75.58**	**8.796**	**8.1**	**2.069**	**16.74**	**25.86**	**30.81**
	Equiv. Refl.: (4,-6,1)								
**Quartz**	**(2,-6,-1)**	**7.795**	**75.58**	**8.795**	**8.1**	**2.069**	**16.74**	**25.86**	**30.81**
	Equiv. Refl.: (-6,2,1), (-6,4,-1)								
**Quartz**	**(2,4,1)**	**7.795**	**75.58**	**8.794**	**8.1**	**2.069**	**16.74**	**25.86**	**30.81**
**Quartz**	**(-4,3,-5)**	**7.777**	**75.1**	**8.407**	**7.6**	**2.141**	**16.37**	**26.77**	**31.38**
**Quartz**	**(-4,1,5)**	**7.777**	**75.1**	**8.405**	**7.6**	**2.141**	**16.37**	**26.77**	**31.38**
**Quartz**	**(1,3,5)**	**7.777**	**75.1**	**8.402**	**7.6**	**2.141**	**16.36**	**26.77**	**31.37**
	Equiv. Refl.: (1,-4,-5)								
**Quartz**	**(3,1,-5)**	**7.777**	**75.1**	**8.401**	**7.6**	**2.141**	**16.36**	**26.77**	**31.37**
**Quartz**	**(3,-4,5)**	**7.777**	**75.1**	**8.399**	**7.6**	**2.141**	**16.36**	**26.77**	**31.37**
**Quartz**	**(-4,6,-1)**	**7.795**	**75.58**	**8.011**	**8.1**	**2.069**	**16.74**	**25.86**	**30.81**
	Equiv. Refl.: (-4,-2,1)								
**Quartz**	**(6,-2,-1)**	**7.795**	**75.58**	**8.009**	**8.1**	**2.069**	**16.74**	**25.86**	**30.81**
	Equiv. Refl.: (6,-4,1), (-2,6,1), (-2,-4,-1)								
**Quartz**	**(4,-3,5)**	**7.777**	**75.1**	**7.511**	**7.6**	**2.141**	**16.37**	**26.77**	**31.38**
**Quartz**	**(4,-1,-5)**	**7.777**	**75.1**	**7.509**	**7.6**	**2.141**	**16.37**	**26.77**	**31.38**
**Quartz**	**(-1,4,5)**	**7.777**	**75.1**	**7.507**	**7.6**	**2.141**	**16.36**	**26.77**	**31.37**
	Equiv. Refl.: (-1,-3,-5)								
**Quartz**	**(-3,-1,5)**	**7.777**	**75.1**	**7.506**	**7.6**	**2.141**	**16.36**	**26.77**	**31.37**
**Quartz**	**(-3,4,-5)**	**7.777**	**75.1**	**7.505**	**7.6**	**2.141**	**16.36**	**26.77**	**31.37**
**Quartz**	**(-1,3,-6)**	**7.888**	**78.56**	**5.722**	**5.7**	**1.628**	**9.35**	**20.35**	**22.4**
**Quartz**	**(-1,-2,6)**	**7.888**	**78.56**	**5.721**	**5.7**	**1.628**	**9.35**	**20.35**	**22.4**
**Quartz**	**(-2,-1,-6)**	**7.888**	**78.56**	**5.717**	**5.7**	**1.628**	**9.34**	**20.35**	**22.4**
**Quartz**	**(-2,3,6)**	**7.888**	**78.56**	**5.714**	**5.7**	**1.628**	**9.34**	**20.35**	**22.39**
**Quartz**	**(3,-2,-6)**	**7.888**	**78.56**	**5.711**	**5.7**	**1.628**	**9.34**	**20.35**	**22.39**
**Quartz**	**(3,-1,6)**	**7.888**	**78.56**	**5.71**	**5.7**	**1.628**	**9.34**	**20.35**	**22.39**
**Quartz**	**(-1,4,-5)**	**7.777**	**75.1**	**5.235**	**5.3**	**2.141**	**11.39**	**26.77**	**29.09**
	Equiv. Refl.: (-1,-3,5)								
**Quartz**	**(4,-1,5)**	**7.777**	**75.1**	**5.233**	**5.3**	**2.141**	**11.39**	**26.77**	**29.09**
	Equiv. Refl.: (4,-3,-5)								
**Quartz**	**(-3,-1,-5)**	**7.777**	**75.1**	**5.23**	**5.3**	**2.141**	**11.38**	**26.77**	**29.09**
**Quartz**	**(-3,4,5)**	**7.777**	**75.1**	**5.227**	**5.3**	**2.141**	**11.38**	**26.77**	**29.09**
**LiNbO3**	**(2,4,-5)**	**7.69**	**72.85**	**5.055**	**8.3**	**2.484**	**20.66**	**31.05**	**37.3**
	Equiv. Refl.: (2,-6,5), (4,2,5), (4,-6,-5), (6,-2,5), (6,-4,-5), (-2,6,-5), (-2,-4,5), (-4,6,5), (-4,-2,-5) (-6,2,-5), (-6,4,5)								
**Quartz**	**(1,3,-5)**	**7.777**	**75.1**	**4.994**	**5.3**	**2.141**	**11.39**	**26.77**	**29.09**
**Quartz**	**(1,-4,5)**	**7.777**	**75.1**	**4.993**	**5.3**	**2.141**	**11.39**	**26.77**	**29.09**
**Quartz**	**(-4,1,-5)**	**7.777**	**75.1**	**4.992**	**5.3**	**2.141**	**11.39**	**26.77**	**29.09**
**Quartz**	**(-4,3,5)**	**7.777**	**75.1**	**4.991**	**5.3**	**2.141**	**11.39**	**26.77**	**29.09**

Ei =	8.048	keV						ΔEg	ΔEt
**Cryst**	**Refl**	**EB**	**ΘB**	**∫ IR dΘ**	**Width**	**Ei cotΘ**	**ΔE**	**2m, 50µm**	**2m, 50µm**
	**(h,k,l)**	**[keV]**	**[°]**	**[µrad]**	**[µrad]**	**[meV/µrad]**	**[meV]**	**[meV]**	**[meV]**
**Quartz**	**(3,1,5)**	**7.777**	**75.1**	**4.987**	**5.3**	**2.141**	**11.38**	**26.77**	**29.09**
**Quartz**	**(3,-4,-5)**	**7.777**	**75.1**	**4.985**	**5.3**	**2.141**	**11.38**	**26.77**	**29.09**
**Quartz**	**(1,-3,6)**	**7.888**	**78.56**	**4.682**	**5.7**	**1.628**	**9.35**	**20.35**	**22.4**
**Quartz**	**(1,2,-6)**	**7.888**	**78.56**	**4.68**	**5.7**	**1.628**	**9.35**	**20.35**	**22.4**
**Quartz**	**(2,1,6)**	**7.888**	**78.56**	**4.677**	**5.7**	**1.628**	**9.34**	**20.35**	**22.4**
**Quartz**	**(2,-3,-6)**	**7.888**	**78.56**	**4.673**	**5.7**	**1.628**	**9.34**	**20.35**	**22.39**
**Quartz**	**(-3,1,-6)**	**7.888**	**78.56**	**4.67**	**5.7**	**1.628**	**9.34**	**20.35**	**22.39**
	Equiv. Refl.: (-3,2,6)								
**Sapph**	**(2,2,12)**	**7.744**	**74.19**	**3.421**	**4.4**	**2.279**	**9.97**	**28.48**	**30.18**
	Equiv. Refl.: (2,2,-12), (2,-4,12), (2,-4,-12), (4,-2,12), (4,-2,-12), (-2,4,12), (-2,4,-12), (-2,-2,12), (-2,-2,-12) (-4,2,12), (-4,2,-12)								
**LiNbO3**	**(1,3,13)**	**7.677**	**72.52**	**2.761**	**5.9**	**2.534**	**15.05**	**31.67**	**35.07**
	Equiv. Refl.: (1,-4,-13), (3,1,-13), (3,-4,13), (4,-1,-13), (4,-3,13), (-1,4,13), (-1,-3,-13), (-3,4,-13), (-3,-1,13) (-4,1,13), (-4,3,-13)								
**LiNbO3**	**(1,5,-1)**	**7.755**	**74.49**	**1.981**	**5.1**	**2.234**	**11.45**	**27.92**	**30.18**
	Equiv. Refl.: (1,-6,1), (5,1,1), (5,-6,-1), (6,-1,1), (6,-5,-1), (-1,6,-1), (-1,-5,1), (-5,6,1), (-5,-1,-1) (-6,1,-1), (-6,5,1)								
**Quartz**	**(-3,-3,-2)**	**7.911**	**79.4**	**1.94**	**2.8**	**1.506**	**4.26**	**18.83**	**19.3**
**Quartz**	**(-3,6,2)**	**7.911**	**79.4**	**1.939**	**2.8**	**1.506**	**4.26**	**18.83**	**19.3**
**Quartz**	**(-3,6,-2)**	**7.911**	**79.4**	**1.937**	**2.8**	**1.506**	**4.26**	**18.83**	**19.3**
	Equiv. Refl.: (-3,-3,2)								
**Quartz**	**(6,-3,-2)**	**7.911**	**79.4**	**1.936**	**2.8**	**1.506**	**4.26**	**18.83**	**19.3**
**Quartz**	**(6,-3,2)**	**7.911**	**79.4**	**1.935**	**2.8**	**1.506**	**4.25**	**18.83**	**19.3**
**Sapph**	**(2,3,8)**	**7.588**	**70.54**	**1.381**	**2.3**	**2.844**	**6.55**	**35.54**	**36.14**
	Equiv. Refl.: (2,-5,-8), (3,2,-8), (3,-5,8), (5,-2,-8), (5,-3,8), (-2,5,8), (-2,-3,-8), (-3,5,-8), (-3,-2,8) (-5,2,8), (-5,3,-8)								
**Quartz**	**(3,3,2)**	**7.911**	**79.4**	**1.374**	**2.8**	**1.506**	**4.26**	**18.83**	**19.3**
	Equiv. Refl.: (3,-6,-2)								
**Quartz**	**(3,-6,2)**	**7.911**	**79.4**	**1.373**	**2.8**	**1.506**	**4.26**	**18.83**	**19.3**
**Quartz**	**(3,3,-2)**	**7.911**	**79.4**	**1.372**	**2.8**	**1.506**	**4.26**	**18.83**	**19.3**
**Quartz**	**(-6,3,2)**	**7.911**	**79.4**	**1.371**	**2.8**	**1.506**	**4.26**	**18.83**	**19.3**
**Quartz**	**(-6,3,-2)**	**7.911**	**79.4**	**1.37**	**2.8**	**1.506**	**4.25**	**18.83**	**19.3**
**Quartz**	**(0,5,2)**	**7.638**	**71.62**	**1.209**	**2**	**2.674**	**5.25**	**33.42**	**33.83**
	Equiv. Refl.: (0,-5,-2)								
**Quartz**	**(5,0,-2)**	**7.638**	**71.62**	**1.206**	**2**	**2.674**	**5.24**	**33.42**	**33.83**
	Equiv. Refl.: (-5,0,2)								
**Quartz**	**(5,-5,2)**	**7.638**	**71.62**	**1.205**	**2**	**2.674**	**5.23**	**33.42**	**33.83**
	Equiv. Refl.: (-5,5,-2)								
**Quartz**	**(6,-2,1)**	**7.795**	**75.58**	**0.781**	**1.4**	**2.069**	**2.99**	**25.86**	**26.04**
	Equiv. Refl.: (6,-4,-1), (-2,6,-1), (-2,-4,1), (-4,6,1), (-4,-2,-1)								
**LiNbO3**	**(2,3,11)**	**7.806**	**75.91**	**0.706**	**3.1**	**2.02**	**6.26**	**25.25**	**26.01**
	Equiv. Refl.: (2,-5,-11), (3,2,-11), (3,-5,11), (5,-2,-11), (5,-3,11), (-2,5,11), (-2,-3,-11), (-3,5,-11), (-3,-2,11) (-5,2,11), (-5,3,-11)								
**Quartz**	**(2,4,-1)**	**7.795**	**75.58**	**0.616**	**1.4**	**2.069**	**2.99**	**25.86**	**26.04**
	Equiv. Refl.: (2,-6,1), (4,2,1), (4,-6,-1), (-6,2,-1), (-6,4,1)								
**Sapph**	**(3,3,3)**	**7.947**	**80.92**	**0.179**	**1**	**1.286**	**1.31**	**16.07**	**16.12**
	Equiv. Refl.: (3,3,-3), (3,-6,3), (3,-6,-3), (6,-3,3), (6,-3,-3), (-3,6,3), (-3,6,-3), (-3,-3,3), (-3,-3,-3) (-6,3,3), (-6,3,-3)								

Ei =	8.028	keV						ΔEg	ΔEt
Cryst	Refl	EB	ΘB	∫ IR dΘ	Width	Ei cotΘ	ΔE	2m, 50µm	2m, 50µm
	(h,k,l)	[keV]	[°]	[µrad]	[µrad]	[meV/µrad]	[meV]	[meV]	[meV]
**Ge**	**(4,4,4)**	**7.592**	**71.03**	**34.4718**	**31.66**	**2.7603**	**87.38**	**34.5**	**93.95**
**Si**	**(4,4,4)**	**7.908**	**80.09**	**31.4796**	**28.04**	**1.4029**	**39.34**	**17.54**	**43.07**
**Ge**	**(1,1,7)**	**7.825**	**77.1**	**30.2176**	**30.22**	**1.8384**	**55.57**	**22.98**	**60.13**
	Equiv. Refl.: (1,5,5), (1,7,1), (5,1,5), (5,5,1), (7,1,1)								
**LiNbO3**	**(1,3,-14)**	**8.021**	**87.52**	**182.451**	**173.4**	**0.347**	**60.21**	**4.34**	**60.37**
	Equiv. Refl.: (-1,4,-14), (-3,-1,-14), (-4,1,-14)								
**LiNbO3**	**(3,-4,-14)**	**8.021**	**87.52**	**182.45**	**173.4**	**0.347**	**60.21**	**4.34**	**60.37**
	Equiv. Refl.: (4,-3,-14)								
**LiNbO3**	**(1,-4,14)**	**8.021**	**87.52**	**168.813**	**173.4**	**0.347**	**60.21**	**4.34**	**60.37**
	Equiv. Refl.: (4,-1,14)								
**LiNbO3**	**(3,1,14)**	**8.021**	**87.52**	**168.812**	**173.4**	**0.347**	**60.21**	**4.34**	**60.37**
	Equiv. Refl.: (-1,-3,14), (-3,4,14), (-4,3,14)								
**LiNbO3**	**(1,-6,4)**	**7.946**	**81.8**	**58.635**	**56.1**	**1.157**	**64.95**	**14.47**	**66.54**
	Equiv. Refl.: (5,1,4), (6,-1,4), (-1,-5,4)								
**LiNbO3**	**(-5,6,4)**	**7.946**	**81.8**	**58.634**	**56.1**	**1.157**	**64.95**	**14.47**	**66.54**
	Equiv. Refl.: (-6,5,4)								
**LiNbO3**	**(1,5,-4)**	**7.946**	**81.8**	**56.279**	**56.1**	**1.157**	**64.95**	**14.47**	**66.54**
	Equiv. Refl.: (-1,6,-4), (-5,-1,-4), (-6,1,-4)								
**LiNbO3**	**(5,-6,-4)**	**7.946**	**81.8**	**56.278**	**56.1**	**1.157**	**64.95**	**14.47**	**66.54**
	Equiv. Refl.: (6,-5,-4)								
**LiNbO3**	**(0,5,-8)**	**7.819**	**76.89**	**46.211**	**42.1**	**1.869**	**78.69**	**23.37**	**82.08**
	Equiv. Refl.: (5,-5,-8), (-5,0,-8)								
**LiNbO3**	**(0,-5,8)**	**7.819**	**76.89**	**42.565**	**42.1**	**1.869**	**78.69**	**23.37**	**82.08**
	Equiv. Refl.: (5,0,8), (-5,5,8)								
**Sapph**	**(3,3,0)**	**7.817**	**76.84**	**40.379**	**33.8**	**1.877**	**63.39**	**23.46**	**67.59**
	Equiv. Refl.: (3,-6,0), (6,-3,0), (-3,6,0), (-3,-3,0), (-6,3,0)								
**LiNbO3**	**(1,5,2)**	**7.793**	**76.11**	**32.451**	**31.7**	**1.985**	**63**	**24.81**	**67.71**
	Equiv. Refl.: (5,-6,2), (6,-5,2), (-1,6,2), (-5,-1,2), (-6,1,2)								
**LiNbO3**	**(1,-6,-2)**	**7.793**	**76.11**	**31.687**	**31.7**	**1.985**	**63**	**24.81**	**67.71**
	Equiv. Refl.: (5,1,-2), (6,-1,-2), (-1,-5,-2), (-5,6,-2), (-6,5,-2)								
**LiNbO3**	**(0,-2,-16)**	**7.676**	**72.98**	**27.015**	**25.7**	**2.458**	**63.25**	**30.72**	**70.32**
	Equiv. Refl.: (2,0,-16), (-2,2,-16)								
**Sapph**	**(0,4,-10)**	**7.681**	**73.09**	**26.911**	**22.7**	**2.441**	**55.49**	**30.52**	**63.33**
	Equiv. Refl.: (0,-4,10), (4,0,10), (4,-4,-10), (-4,0,-10), (-4,4,10)								
**LiNbO3**	**(0,2,16)**	**7.676**	**72.98**	**24.689**	**25.7**	**2.458**	**63.25**	**30.72**	**70.32**
	Equiv. Refl.: (2,-2,16), (-2,0,16)								
**Quartz**	**(2,3,4)**	**7.835**	**77.4**	**24.541**	**20.7**	**1.794**	**37.15**	**22.42**	**43.4**
**Quartz**	**(2,-5,-4)**	**7.835**	**77.4**	**24.54**	**20.7**	**1.794**	**37.15**	**22.42**	**43.4**
**Quartz**	**(3,2,-4)**	**7.835**	**77.4**	**24.537**	**20.7**	**1.794**	**37.15**	**22.42**	**43.39**
**Quartz**	**(3,-5,4)**	**7.835**	**77.4**	**24.536**	**20.7**	**1.794**	**37.15**	**22.42**	**43.39**
	Equiv. Refl.: (-5,2,4), (-5,3,-4)								
**LiNbO3**	**(3,3,6)**	**7.707**	**73.75**	**24.1**	**24**	**2.34**	**56.04**	**29.25**	**63.22**
	Equiv. Refl.: (3,-6,6), (6,-3,6), (-3,6,6), (-3,-3,6), (-6,3,6)								
**Quartz**	**(-2,5,4)**	**7.835**	**77.4**	**24.09**	**20.7**	**1.794**	**37.15**	**22.42**	**43.4**
	Equiv. Refl.: (-2,-3,-4)								
**Quartz**	**(-3,-2,4)**	**7.835**	**77.4**	**24.088**	**20.7**	**1.794**	**37.15**	**22.42**	**43.39**
**Quartz**	**(-3,5,-4)**	**7.835**	**77.4**	**24.087**	**20.7**	**1.794**	**37.15**	**22.42**	**43.39**

Ei =<br>Cryst	8.028<br>Refl<br>(h,k,l)	keV<br>EB<br>[keV]	ΘB<br>[°]	∫ IR dΘ<br>[µrad]	Width<br>[µrad]	Ei cotΘ<br>[meV/µrad]	ΔE<br>[meV]	ΔEg<br>2m, 50µm<br>[meV]	ΔEt<br>2m, 50µm<br>[meV]
**Quartz**	**(5,-2,-4)**	**7.835**	**77.4**	**24.086**	**20.7**	**1.794**	**37.15**	**22.42**	**43.39**
	Equiv. Refl.: (5,-3,4)								
**Sapph**	**(0,5,4)**	**7.761**	**75.17**	**23.77**	**20.6**	**2.126**	**43.75**	**26.57**	**51.19**
	Equiv. Refl.: (0,-5,-4), (5,0,-4), (5,-5,4), (-5,0,4), (-5,5,-4)								
**Quartz**	**(0,5,-2)**	**7.638**	**72.06**	**23.724**	**19.7**	**2.599**	**51.17**	**32.49**	**60.61**
	Equiv. Refl.: (0,-5,2)								
**Quartz**	**(5,0,2)**	**7.638**	**72.06**	**23.722**	**19.7**	**2.599**	**51.16**	**32.49**	**60.61**
	Equiv. Refl.: (5,-5,-2), (-5,0,-2), (-5,5,2)								
**LiNbO3**	**(2,4,4)**	**7.572**	**70.6**	**23.563**	**22.6**	**2.828**	**63.96**	**35.34**	**73.08**
	Equiv. Refl.: (4,-6,4), (6,-4,4), (-2,6,4), (-4,-2,4), (-6,2,4)								
**LiNbO3**	**(3,3,-6)**	**7.707**	**73.75**	**22.883**	**24**	**2.34**	**56.04**	**29.25**	**63.22**
	Equiv. Refl.: (3,-6,-6), (6,-3,-6), (-3,6,-6), (-3,-3,-6), (-6,3,-6)								
**LiNbO3**	**(2,-6,-4)**	**7.572**	**70.6**	**21.713**	**22.6**	**2.828**	**63.96**	**35.34**	**73.08**
	Equiv. Refl.: (4,2,-4), (6,-2,-4), (-2,-4,-4), (-4,6,-4), (-6,4,-4)								
**Sapph**	**(1,2,14)**	**7.778**	**75.66**	**20.849**	**18.4**	**2.053**	**37.74**	**25.66**	**45.64**
	Equiv. Refl.: (1,-3,-14), (2,1,-14), (2,-3,14), (3,-1,-14), (3,-2,14), (-1,3,14), (-1,-2,-14), (-2,3,-14), (-2,-1,14) (-3,1,14), (-3,2,-14)								
**Quartz**	**(2,1,-6)**	**7.888**	**79.29**	**18.693**	**16.4**	**1.518**	**24.83**	**18.98**	**31.26**
**Quartz**	**(1,2,6)**	**7.888**	**79.29**	**18.69**	**16.4**	**1.518**	**24.83**	**18.98**	**31.25**
**Quartz**	**(2,-3,6)**	**7.888**	**79.29**	**18.689**	**16.4**	**1.518**	**24.83**	**18.98**	**31.25**
**Quartz**	**(1,-3,-6)**	**7.888**	**79.29**	**18.688**	**16.4**	**1.518**	**24.83**	**18.98**	**31.25**
**Quartz**	**(-3,1,6)**	**7.888**	**79.29**	**18.678**	**16.3**	**1.518**	**24.82**	**18.98**	**31.24**
	Equiv. Refl.: (-3,2,-6)								
**Quartz**	**(-2,-1,6)**	**7.888**	**79.29**	**18.289**	**16.4**	**1.518**	**24.83**	**18.98**	**31.26**
**Quartz**	**(-1,-2,-6)**	**7.888**	**79.29**	**18.287**	**16.4**	**1.518**	**24.83**	**18.98**	**31.25**
**Quartz**	**(-1,3,6)**	**7.888**	**79.29**	**18.285**	**16.4**	**1.518**	**24.83**	**18.98**	**31.25**
	Equiv. Refl.: (-2,3,-6)								
**Quartz**	**(3,-1,-6)**	**7.888**	**79.29**	**18.274**	**16.3**	**1.518**	**24.82**	**18.98**	**31.24**
	Equiv. Refl.: (3,-2,6)								
**Sapph**	**(2,4,-2)**	**8.018**	**87.09**	**17.605**	**22.2**	**0.408**	**9.06**	**5.09**	**10.39**
	Equiv. Refl.: (2,-6,2), (4,2,2), (4,-6,-2), (6,-2,2), (6,-4,-2), (-2,6,-2), (-2,-4,2), (-4,6,2), (-4,-2,-2) (-6,2,-2), (-6,4,2)								
**Quartz**	**(2,4,0)**	**7.71**	**73.81**	**15.186**	**13.1**	**2.331**	**30.59**	**29.13**	**42.24**
	Equiv. Refl.: (2,-6,0), (4,2,0), (4,-6,0), (-6,2,0), (-6,4,0)								
**Quartz**	**(6,-2,0)**	**7.71**	**73.81**	**15.165**	**13.1**	**2.331**	**30.59**	**29.13**	**42.24**
	Equiv. Refl.: (6,-4,0), (-2,6,0), (-2,-4,0), (-4,6,0), (-4,-2,0)								
**LiNbO3**	**(2,4,7)**	**7.996**	**84.88**	**14.658**	**25.2**	**0.72**	**18.12**	**8.99**	**20.23**
	Equiv. Refl.: (2,-6,-7), (4,2,-7), (4,-6,7), (6,-2,-7), (6,-4,7), (-2,6,7), (-2,-4,-7), (-4,6,-7), (-4,-2,7) (-6,2,7), (-6,4,-7)								
**Sapph**	**(1,1,15)**	**7.619**	**71.63**	**12.682**	**11.6**	**2.666**	**30.83**	**33.33**	**45.4**
	Equiv. Refl.: (1,1,-15), (1,-2,15), (1,-2,-15), (2,-1,15), (2,-1,-15), (-1,2,15), (-1,2,-15), (-1,-1,15), (-1,-1,-15) (-2,1,15), (-2,1,-15)								
**Sapph**	**(2,4,1)**	**7.975**	**83.41**	**11.838**	**13.4**	**0.928**	**12.4**	**11.6**	**16.98**
	Equiv. Refl.: (2,-6,-1), (4,2,-1), (4,-6,1), (6,-2,-1), (6,-4,1), (-2,6,1), (-2,-4,-1), (-4,6,-1), (-4,-2,1) (-6,2,1), (-6,4,-1)								
**Quartz**	**(2,2,5)**	**7.64**	**72.11**	**11.823**	**10.3**	**2.592**	**26.76**	**32.4**	**42.02**
	Equiv. Refl.: (2,-4,-5)								
**Quartz**	**(2,2,-5)**	**7.64**	**72.11**	**11.822**	**10.3**	**2.592**	**26.76**	**32.4**	**42.02**
**Quartz**	**(2,-4,5)**	**7.64**	**72.11**	**11.82**	**10.3**	**2.592**	**26.75**	**32.4**	**42.02**
**Quartz**	**(-4,2,5)**	**7.64**	**72.11**	**11.818**	**10.3**	**2.592**	**26.75**	**32.4**	**42.01**
**Quartz**	**(-4,2,-5)**	**7.64**	**72.11**	**11.817**	**10.3**	**2.592**	**26.75**	**32.4**	**42.01**

Ei =	8.028	keV						ΔEg	ΔEt
**Cryst**	**Refl**	**EB**	**ΘB**	**∫ IR dΘ**	**Width**	**Ei cotΘ**	**ΔE**	**2m, 50µm**	**2m, 50µm**
	**(h,k,l)**	**[keV]**	**[°]**	**[µrad]**	**[µrad]**	**[meV/µrad]**	**[meV]**	**[meV]**	**[meV]**
**Quartz**	**(-2,4,5)**	**7.64**	**72.11**	**11.544**	**10.3**	**2.592**	**26.76**	**32.4**	**42.02**
	Equiv. Refl.: (-2,-2,-5)								
**Quartz**	**(-2,-2,5)**	**7.64**	**72.11**	**11.543**	**10.3**	**2.592**	**26.76**	**32.4**	**42.02**
**Quartz**	**(-2,4,-5)**	**7.64**	**72.11**	**11.54**	**10.3**	**2.592**	**26.75**	**32.4**	**42.02**
**Quartz**	**(4,-2,-5)**	**7.64**	**72.11**	**11.539**	**10.3**	**2.592**	**26.75**	**32.4**	**42.01**
**Quartz**	**(4,-2,5)**	**7.64**	**72.11**	**11.538**	**10.3**	**2.592**	**26.75**	**32.4**	**42.01**
**Sapph**	**(0,1,-16)**	**7.783**	**75.82**	**11.339**	**10.8**	**2.028**	**22**	**25.35**	**33.57**
	Equiv. Refl.: (0,-1,16), (1,0,16), (1,-1,-16), (-1,0,-16), (-1,1,16)								
**Quartz**	**(3,3,1)**	**7.657**	**72.52**	**10.886**	**9.5**	**2.529**	**24.14**	**31.61**	**39.78**
	Equiv. Refl.: (3,-6,-1), (-6,3,1), (-6,3,-1)								
**Quartz**	**(3,3,-1)**	**7.657**	**72.52**	**10.884**	**9.5**	**2.529**	**24.14**	**31.61**	**39.77**
	Equiv. Refl.: (3,-6,1)								
**Quartz**	**(6,-3,0)**	**7.571**	**70.57**	**10.616**	**9.3**	**2.832**	**26.22**	**35.4**	**44.06**
	Equiv. Refl.: (-3,6,0), (-3,-3,0)								
**Quartz**	**(3,3,0)**	**7.571**	**70.57**	**10.553**	**9.3**	**2.832**	**26.22**	**35.4**	**44.06**
	Equiv. Refl.: (3,-6,0), (-6,3,0)								
**Quartz**	**(6,-3,1)**	**7.657**	**72.52**	**10.373**	**9.5**	**2.529**	**24.15**	**31.61**	**39.78**
	Equiv. Refl.: (6,-3,-1), (-3,6,1), (-3,-3,-1)								
**Quartz**	**(-3,6,-1)**	**7.657**	**72.52**	**10.372**	**9.5**	**2.529**	**24.14**	**31.61**	**39.77**
	Equiv. Refl.: (-3,-3,1)								
**Quartz**	**(5,-2,4)**	**7.835**	**77.4**	**10.09**	**9.6**	**1.794**	**17.19**	**22.42**	**28.26**
	Equiv. Refl.: (5,-3,-4)								
**Quartz**	**(-2,-3,4)**	**7.835**	**77.4**	**10.081**	**9.6**	**1.794**	**17.18**	**22.42**	**28.25**
**Quartz**	**(-2,5,-4)**	**7.835**	**77.4**	**10.08**	**9.6**	**1.794**	**17.18**	**22.42**	**28.25**
**Quartz**	**(-3,-2,-4)**	**7.835**	**77.4**	**10.077**	**9.6**	**1.794**	**17.17**	**22.42**	**28.24**
**Quartz**	**(-3,5,4)**	**7.835**	**77.4**	**10.076**	**9.6**	**1.794**	**17.17**	**22.42**	**28.24**
**Quartz**	**(-5,3,4)**	**7.835**	**77.4**	**9.919**	**9.6**	**1.794**	**17.19**	**22.42**	**28.26**
**Quartz**	**(-5,2,-4)**	**7.835**	**77.4**	**9.918**	**9.6**	**1.794**	**17.19**	**22.42**	**28.26**
**Quartz**	**(2,3,-4)**	**7.835**	**77.4**	**9.909**	**9.6**	**1.794**	**17.18**	**22.42**	**28.25**
**Quartz**	**(2,-5,4)**	**7.835**	**77.4**	**9.908**	**9.6**	**1.794**	**17.18**	**22.42**	**28.25**
**Quartz**	**(3,2,4)**	**7.835**	**77.4**	**9.905**	**9.6**	**1.794**	**17.17**	**22.42**	**28.24**
**Quartz**	**(3,-5,-4)**	**7.835**	**77.4**	**9.904**	**9.6**	**1.794**	**17.17**	**22.42**	**28.24**
**Quartz**	**(4,2,-1)**	**7.795**	**76.15**	**9.164**	**8.4**	**1.98**	**16.7**	**24.75**	**29.86**
	Equiv. Refl.: (4,-6,1)								
**Quartz**	**(2,-6,-1)**	**7.795**	**76.15**	**9.162**	**8.4**	**1.98**	**16.7**	**24.75**	**29.85**
	Equiv. Refl.: (-6,2,1), (-6,4,-1)								
**Quartz**	**(2,4,1)**	**7.795**	**76.15**	**9.161**	**8.4**	**1.98**	**16.7**	**24.75**	**29.85**
**Quartz**	**(-4,3,-5)**	**7.777**	**75.65**	**8.735**	**8**	**2.054**	**16.33**	**25.68**	**30.43**
**Quartz**	**(-4,1,5)**	**7.777**	**75.65**	**8.733**	**7.9**	**2.054**	**16.33**	**25.68**	**30.43**
**Quartz**	**(1,3,5)**	**7.777**	**75.65**	**8.73**	**7.9**	**2.054**	**16.32**	**25.68**	**30.43**
	Equiv. Refl.: (1,-4,-5)								
**Quartz**	**(3,1,-5)**	**7.777**	**75.65**	**8.729**	**7.9**	**2.054**	**16.32**	**25.68**	**30.43**
**Quartz**	**(3,-4,5)**	**7.777**	**75.65**	**8.727**	**7.9**	**2.054**	**16.32**	**25.68**	**30.43**
**Quartz**	**(-4,6,-1)**	**7.795**	**76.15**	**8.342**	**8.4**	**1.98**	**16.7**	**24.75**	**29.86**
	Equiv. Refl.: (-4,-2,1)								
**Quartz**	**(6,-2,-1)**	**7.795**	**76.15**	**8.34**	**8.4**	**1.98**	**16.7**	**24.75**	**29.85**
	Equiv. Refl.: (6,-4,1), (-2,6,1), (-2,-4,-1)								
**Quartz**	**(4,-3,5)**	**7.777**	**75.65**	**7.8**	**8**	**2.054**	**16.33**	**25.68**	**30.43**
**Quartz**	**(4,-1,-5)**	**7.777**	**75.65**	**7.798**	**7.9**	**2.054**	**16.33**	**25.68**	**30.43**

Ei =	8.028	keV						ΔEg	ΔEt
Cryst	Refl	EB	ΘB	∫ IR dΘ	Width	Ei cotΘ	ΔE	2m, 50µm	2m, 50µm
	(h,k,l)	[keV]	[°]	[µrad]	[µrad]	[meV/µrad]	[meV]	[meV]	[meV]
Quartz	(-1,4,5)	7.777	75.65	7.796	7.9	2.054	16.33	25.68	30.43
	Equiv. Refl.: (-1,-3,-5)								
Quartz	(-3,-1,5)	7.777	75.65	7.795	7.9	2.054	16.32	25.68	30.43
Quartz	(-3,4,-5)	7.777	75.65	7.794	7.9	2.054	16.32	25.68	30.43
Quartz	(-1,3,-6)	7.888	79.29	6.114	6.1	1.518	9.33	18.98	21.15
Quartz	(-1,-2,6)	7.888	79.29	6.112	6.1	1.518	9.33	18.98	21.15
Quartz	(-2,-1,-6)	7.888	79.29	6.108	6.1	1.518	9.32	18.98	21.14
Quartz	(-2,3,6)	7.888	79.29	6.105	6.1	1.518	9.32	18.98	21.14
Quartz	(3,-1,6)	7.888	79.29	6.101	6.1	1.518	9.31	18.98	21.14
	Equiv. Refl.: (3,-2,-6)								
Quartz	(-1,4,-5)	7.777	75.65	5.436	5.5	2.054	11.37	25.68	28.08
	Equiv. Refl.: (-1,-3,5)								
Quartz	(4,-1,5)	7.777	75.65	5.434	5.5	2.054	11.36	25.68	28.08
	Equiv. Refl.: (4,-3,-5)								
Quartz	(-3,-1,-5)	7.777	75.65	5.43	5.5	2.054	11.36	25.68	28.08
Quartz	(-3,4,5)	7.777	75.65	5.428	5.5	2.054	11.35	25.68	28.08
LiNbO3	(2,4,-5)	7.69	73.31	5.192	8.6	2.406	20.61	30.08	36.46
	Equiv. Refl.: (2,-6,5), (4,2,5), (4,-6,-5), (6,-2,5), (6,-4,-5), (-2,6,-5), (-2,-4,5), (-4,6,5), (-4,-2,-5) (-6,2,-5), (-6,4,5)								
Quartz	(1,3,-5)	7.777	75.65	5.184	5.5	2.054	11.37	25.68	28.08
	Equiv. Refl.: (1,-4,5)								
Quartz	(-4,1,-5)	7.777	75.65	5.182	5.5	2.054	11.36	25.68	28.08
	Equiv. Refl.: (-4,3,5)								
Quartz	(3,1,5)	7.777	75.65	5.177	5.5	2.054	11.36	25.68	28.08
Quartz	(3,-4,-5)	7.777	75.65	5.175	5.5	2.054	11.35	25.68	28.08
Quartz	(1,-3,6)	7.888	79.29	4.997	6.1	1.518	9.33	18.98	21.15
Quartz	(1,2,-6)	7.888	79.29	4.996	6.1	1.518	9.33	18.98	21.15
Quartz	(2,1,6)	7.888	79.29	4.992	6.1	1.518	9.32	18.98	21.14
Quartz	(2,-3,-6)	7.888	79.29	4.988	6.1	1.518	9.32	18.98	21.14
Quartz	(-3,1,-6)	7.888	79.29	4.985	6.1	1.518	9.31	18.98	21.14
	Equiv. Refl.: (-3,2,6)								
Sapph	(2,2,12)	7.744	74.7	3.758	4.5	2.196	9.95	27.45	29.19
	Equiv. Refl.: (2,2,-12), (2,-4,12), (2,-4,-12), (4,-2,12), (4,-2,-12), (-2,4,12), (-2,4,-12), (-2,-2,12), (-2,-2,-12) (-4,2,12), (-4,2,-12)								
LiNbO3	(1,3,13)	7.677	72.98	2.833	6.1	2.457	15.02	30.71	34.19
	Equiv. Refl.: (1,-4,-13), (3,1,-13), (3,-4,13), (4,-1,-13), (4,-3,13), (-1,4,13), (-1,-3,-13), (-3,4,-13), (-3,-1,13) (-4,1,13), (-4,3,-13)								
Quartz	(-3,-3,-2)	7.911	80.19	2.096	3.1	1.388	4.25	17.35	17.86
Quartz	(-3,6,2)	7.911	80.19	2.095	3.1	1.388	4.25	17.35	17.86
Quartz	(-3,6,-2)	7.911	80.19	2.093	3.1	1.388	4.25	17.35	17.86
	Equiv. Refl.: (-3,-3,2)								
Quartz	(6,-3,-2)	7.911	80.19	2.092	3.1	1.388	4.24	17.35	17.86
Quartz	(6,-3,2)	7.911	80.19	2.091	3.1	1.388	4.24	17.35	17.86
LiNbO3	(1,5,-1)	7.755	75.01	2.047	5.3	2.15	11.42	26.87	29.2
	Equiv. Refl.: (1,-6,1), (5,1,1), (5,-6,-1), (6,-1,1), (6,-5,-1), (-1,6,-1), (-1,-5,1), (-5,6,1), (-5,-1,-1) (-6,1,-1), (-6,5,1)								
Quartz	(3,3,2)	7.911	80.19	1.482	3.1	1.388	4.25	17.35	17.86
	Equiv. Refl.: (3,-6,-2)								
Quartz	(3,-6,2)	7.911	80.19	1.481	3.1	1.388	4.25	17.35	17.86
Quartz	(3,3,-2)	7.911	80.19	1.48	3.1	1.388	4.25	17.35	17.86
Quartz	(-6,3,2)	7.911	80.19	1.479	3.1	1.388	4.24	17.35	17.86

Ei = Cryst	8.028 Refl (h,k,l)	keV EB [keV]	ΘB [°]	∫ IR dΘ [µrad]	Width [µrad]	Ei cotΘ [meV/µrad]	ΔE [meV]	ΔEg 2m, 50µm [meV]	ΔEt 2m, 50µm [meV]
**Quartz**	**(-6,3,-2)**	**7.911**	**80.19**	**1.478**	**3.1**	**1.388**	**4.24**	**17.35**	**17.86**
**Sapph**	**(2,3,8)**	**7.588**	**70.95**	**1.41**	**2.4**	**2.772**	**6.53**	**34.65**	**35.26**
	Equiv. Refl.: (2,-5,-8), (3,2,-8), (3,-5,8), (5,-2,-8), (5,-3,8), (-2,5,8), (-2,-3,-8), (-3,5,-8), (-3,-2,8) (-5,2,8), (-5,3,-8)								
**Sapph**	**(1,3,-11)**	**7.549**	**70.11**	**1.403**	**2.3**	**2.905**	**6.78**	**36.31**	**36.94**
	Equiv. Refl.: (1,-4,11), (3,1,11), (3,-4,-11), (4,-1,11), (4,-3,-11), (-1,4,-11), (-1,-3,11), (-3,4,11), (-3,-1,-11) (-4,1,-11), (-4,3,11)								
**Quartz**	**(0,5,2)**	**7.638**	**72.06**	**1.238**	**2**	**2.599**	**5.23**	**32.49**	**32.91**
	Equiv. Refl.: (0,-5,-2)								
**Quartz**	**(5,0,-2)**	**7.638**	**72.06**	**1.234**	**2**	**2.599**	**5.22**	**32.49**	**32.91**
	Equiv. Refl.: (5,-5,2), (-5,0,2), (-5,5,-2)								
**Quartz**	**(6,-2,1)**	**7.795**	**76.15**	**0.813**	**1.5**	**1.98**	**2.98**	**24.75**	**24.93**
	Equiv. Refl.: (6,-4,-1), (-2,6,-1), (-2,-4,1), (-4,6,1), (-4,-2,-1)								
**LiNbO3**	**(2,3,11)**	**7.806**	**76.49**	**0.735**	**3.2**	**1.929**	**6.24**	**24.11**	**24.9**
	Equiv. Refl.: (2,-5,-11), (3,2,-11), (3,-5,11), (5,-2,-11), (5,-3,11), (-2,5,11), (-2,-3,-11), (-3,5,-11), (-3,-2,11) (-5,2,11), (-5,3,-11)								
**Quartz**	**(2,4,-1)**	**7.795**	**76.15**	**0.641**	**1.5**	**1.98**	**2.98**	**24.75**	**24.93**
	Equiv. Refl.: (2,-6,1), (4,2,1), (4,-6,-1), (-6,4,1)								
**Quartz**	**(-6,2,-1)**	**7.795**	**76.15**	**0.64**	**1.5**	**1.98**	**2.98**	**24.75**	**24.93**
**Sapph**	**(3,3,3)**	**7.947**	**81.87**	**0.199**	**1.1**	**1.147**	**1.31**	**14.34**	**14.4**
	Equiv. Refl.: (3,3,-3), (3,-6,3), (3,-6,-3), (6,-3,3), (6,-3,-3), (-3,6,3), (-3,6,-3), (-3,-3,3), (-3,-3,-3) (-6,3,3), (-6,3,-3)								

Ei =	8.905	keV						ΔEg	ΔEt
Cryst	Refl	EB	ΘB	∫ IR dΘ	Width	Ei cotΘ	ΔE	2m, 50µm	2m, 50µm
	(h,k,l)	[keV]	[°]	[µrad]	[µrad]	[meV/µrad]	[meV]	[meV]	[meV]
**Ge**	**(0,0,8)**	**8.766**	**79.87**	**37.81**	**35.22**	**1.5907**	**56.03**	**19.88**	**59.45**
	Equiv. Refl.: (0,8,0), (8,0,0)								
**Ge**	**(1,3,7)**	**8.417**	**70.94**	**15.3739**	**14.99**	**3.0765**	**46.13**	**38.46**	**60.06**
	Equiv. Refl.: (1,7,3), (3,1,7), (3,5,5), (3,7,1), (5,3,5), (5,5,3), (7,1,3), (7,3,1)								
**Si**	**(1,3,7)**	**8.768**	**79.92**	**14.6281**	**13.52**	**1.5827**	**21.39**	**19.78**	**29.14**
	Equiv. Refl.: (1,7,3), (3,1,7), (3,5,5), (3,7,1), (5,3,5), (5,5,3), (7,1,3), (7,3,1)								
**Si**	**(2,4,6)**	**8.542**	**73.58**	**14.3539**	**12.66**	**2.6244**	**33.21**	**32.8**	**46.68**
	Equiv. Refl.: (2,6,4), (4,2,6), (4,6,2), (6,2,4), (6,4,2)								
**LiNbO3**	**(2,3,14)**	**8.714**	**78.1**	**33.358**	**30.7**	**1.876**	**57.54**	**23.45**	**62.14**
	Equiv. Refl.: (3,-5,14), (5,-3,14), (-2,5,14), (-3,-2,14), (-5,2,14)								
**LiNbO3**	**(0,-6,6)**	**8.764**	**79.78**	**31.768**	**30.5**	**1.605**	**48.95**	**20.07**	**52.9**
	Equiv. Refl.: (6,0,6), (-6,6,6)								
**LiNbO3**	**(2,-5,-14)**	**8.714**	**78.1**	**31.101**	**30.7**	**1.876**	**57.54**	**23.45**	**62.14**
	Equiv. Refl.: (3,2,-14), (5,-2,-14), (-2,-3,-14), (-3,5,-14), (-5,3,-14)								
**LiNbO3**	**(1,3,16)**	**8.737**	**78.84**	**31.03**	**29.2**	**1.757**	**51.28**	**21.97**	**55.79**
	Equiv. Refl.: (3,-4,16), (4,-3,16), (-1,4,16), (-3,-1,16), (-4,1,16)								
**LiNbO3**	**(0,6,-6)**	**8.764**	**79.78**	**30.562**	**30.5**	**1.605**	**48.95**	**20.07**	**52.9**
	Equiv. Refl.: (6,-6,-6), (-6,0,-6)								
**LiNbO3**	**(3,1,-16)**	**8.737**	**78.84**	**29.091**	**29.2**	**1.757**	**51.28**	**21.97**	**55.79**
	Equiv. Refl.: (-1,-3,-16), (-3,4,-16), (-4,3,-16)								
**LiNbO3**	**(1,-4,-16)**	**8.737**	**78.84**	**29.09**	**29.2**	**1.757**	**51.28**	**21.97**	**55.79**
	Equiv. Refl.: (4,-1,-16)								
**LiNbO3**	**(2,-7,0)**	**8.684**	**77.2**	**28.9**	**27.6**	**2.024**	**55.79**	**25.3**	**61.26**
	Equiv. Refl.: (5,2,0), (7,-2,0), (-2,-5,0), (-5,7,0), (-7,5,0)								
**LiNbO3**	**(2,5,0)**	**8.684**	**77.2**	**28.646**	**27.6**	**2.024**	**55.79**	**25.3**	**61.26**
	Equiv. Refl.: (5,-7,0), (7,-5,0), (-2,7,0), (-5,-2,0), (-7,2,0)								
**LiNbO3**	**(0,-6,-6)**	**8.764**	**79.78**	**27.967**	**27.7**	**1.605**	**44.54**	**20.07**	**48.85**
	Equiv. Refl.: (6,0,-6), (-6,6,-6)								
**LiNbO3**	**(0,6,6)**	**8.764**	**79.78**	**27.684**	**27.7**	**1.605**	**44.54**	**20.07**	**48.85**
	Equiv. Refl.: (6,-6,6), (-6,0,6)								
**LiNbO3**	**(2,-6,-10)**	**8.61**	**75.21**	**26.33**	**24.3**	**2.351**	**57.12**	**29.38**	**64.23**
	Equiv. Refl.: (4,2,-10), (6,-2,-10), (-2,-4,-10), (-4,6,-10), (-6,4,-10)								
**LiNbO3**	**(2,4,10)**	**8.61**	**75.21**	**24.448**	**24.3**	**2.351**	**57.12**	**29.38**	**64.23**
	Equiv. Refl.: (4,-6,10), (6,-4,10), (-2,6,10), (-4,-2,10), (-6,2,10)								
**LiNbO3**	**(3,-7,-2)**	**8.505**	**72.77**	**23.779**	**22**	**2.762**	**60.86**	**34.53**	**69.97**
	Equiv. Refl.: (4,3,-2), (7,-3,-2), (-3,-4,-2), (-4,7,-2), (-7,4,-2)								
**LiNbO3**	**(3,4,2)**	**8.505**	**72.77**	**22.593**	**22**	**2.762**	**60.86**	**34.53**	**69.97**
	Equiv. Refl.: (4,-7,2), (7,-4,2), (-3,7,2), (-4,-3,2), (-7,3,2)								
**Quartz**	**(-5,6,3)**	**8.812**	**81.7**	**21.281**	**18.3**	**1.299**	**23.71**	**16.23**	**28.74**
	Equiv. Refl.: (-5,-1,-3)								
**Quartz**	**(6,-5,-3)**	**8.812**	**81.7**	**21.28**	**18.3**	**1.299**	**23.71**	**16.23**	**28.74**
**Quartz**	**(6,-1,3)**	**8.812**	**81.7**	**21.279**	**18.3**	**1.299**	**23.71**	**16.23**	**28.73**
	Equiv. Refl.: (-1,6,-3), (-1,-5,3)								
**LiNbO3**	**(3,-7,4)**	**8.645**	**76.12**	**21.229**	**21**	**2.2**	**46.31**	**27.5**	**53.86**
	Equiv. Refl.: (4,3,4), (7,-3,4), (-3,-4,4), (-4,7,4), (-7,4,4)								
**LiNbO3**	**(3,4,-4)**	**8.645**	**76.12**	**21.071**	**21**	**2.2**	**46.31**	**27.5**	**53.86**
	Equiv. Refl.: (4,-7,-4), (7,-4,-4), (-3,7,-4), (-4,-3,-4), (-7,3,-4)								

Ei =	8.905	keV						ΔEg	ΔEt
**Cryst**	**Refl**	**EB**	**ΘB**	**∫ IR dΘ**	**Width**	**Ei cotΘ**	**ΔE**	**2m, 50μm**	**2m, 50μm**
	**(h,k,l)**	**[keV]**	**[°]**	**[μrad]**	**[μrad]**	**[meV/μrad]**	**[meV]**	**[meV]**	**[meV]**
**Sapph**	**(0,0,18)**	**8.591**	**74.75**	**21.046**	**17.7**	**2.429**	**42.89**	**30.36**	**52.55**
	Equiv. Refl.: (0,0,-18)								
**Quartz**	**(5,1,3)**	**8.812**	**81.7**	**20.994**	**18.3**	**1.299**	**23.71**	**16.23**	**28.74**
	Equiv. Refl.: (5,-6,-3)								
**Quartz**	**(1,5,-3)**	**8.812**	**81.7**	**20.992**	**18.3**	**1.299**	**23.71**	**16.23**	**28.73**
	Equiv. Refl.: (-6,5,3)								
**Quartz**	**(1,-6,3)**	**8.812**	**81.7**	**20.991**	**18.3**	**1.299**	**23.71**	**16.23**	**28.73**
	Equiv. Refl.: (-6,1,-3)								
**Sapph**	**(2,2,15)**	**8.855**	**83.94**	**20.602**	**19.3**	**0.945**	**18.2**	**11.81**	**21.7**
	Equiv. Refl.: (2,2,-15), (2,-4,15), (2,-4,-15), (4,-2,15), (4,-2,-15), (-2,4,15), (-2,4,-15), (-2,-2,15), (-2,-2,-15) (-4,2,15), (-4,2,-15)								
**LiNbO3**	**(1,5,8)**	**8.528**	**73.28**	**20.155**	**19.6**	**2.675**	**52.52**	**33.44**	**62.26**
	Equiv. Refl.: (5,-6,8), (6,-5,8), (-1,6,8), (-5,-1,8), (-6,1,8)								
**LiNbO3**	**(1,-6,-8)**	**8.528**	**73.28**	**20.094**	**19.6**	**2.675**	**52.52**	**33.44**	**62.26**
	Equiv. Refl.: (5,1,-8), (6,-1,-8), (-1,-5,-8), (-5,6,-8), (-6,5,-8)								
**LiNbO3**	**(0,-4,-14)**	**8.374**	**70.12**	**18.586**	**17.4**	**3.22**	**56.08**	**40.25**	**69.03**
	Equiv. Refl.: (4,0,-14), (-4,4,-14)								
**LiNbO3**	**(1,1,-18)**	**8.402**	**70.65**	**18.558**	**17.4**	**3.128**	**54.57**	**39.1**	**67.13**
	Equiv. Refl.: (1,-2,-18), (2,-1,-18), (-1,2,-18), (-1,-1,-18), (-2,1,-18)								
**Quartz**	**(5,-1,-5)**	**8.802**	**81.27**	**17.609**	**15.4**	**1.368**	**21.11**	**17.1**	**27.17**
	Equiv. Refl.: (5,-4,5)								
**Quartz**	**(-4,5,-5)**	**8.802**	**81.27**	**17.607**	**15.4**	**1.368**	**21.11**	**17.1**	**27.17**
**Quartz**	**(-4,-1,5)**	**8.802**	**81.27**	**17.606**	**15.4**	**1.368**	**21.11**	**17.1**	**27.17**
**Quartz**	**(-1,5,5)**	**8.802**	**81.27**	**17.605**	**15.4**	**1.368**	**21.11**	**17.1**	**27.17**
**Quartz**	**(-1,-4,-5)**	**8.802**	**81.27**	**17.604**	**15.4**	**1.368**	**21.11**	**17.1**	**27.17**
**Quartz**	**(-5,1,5)**	**8.802**	**81.27**	**17.594**	**15.4**	**1.368**	**21.11**	**17.1**	**27.17**
	Equiv. Refl.: (-5,4,-5)								
**Quartz**	**(4,-5,5)**	**8.802**	**81.27**	**17.592**	**15.4**	**1.368**	**21.11**	**17.1**	**27.17**
**Quartz**	**(4,1,-5)**	**8.802**	**81.27**	**17.591**	**15.4**	**1.368**	**21.11**	**17.1**	**27.17**
**Quartz**	**(1,-5,-5)**	**8.802**	**81.27**	**17.59**	**15.4**	**1.368**	**21.11**	**17.1**	**27.17**
**Quartz**	**(1,4,5)**	**8.802**	**81.27**	**17.588**	**15.4**	**1.368**	**21.11**	**17.1**	**27.17**
**Quartz**	**(0,6,-1)**	**8.817**	**81.93**	**17.326**	**15.4**	**1.262**	**19.4**	**15.78**	**25.01**
	Equiv. Refl.: (0,-6,1), (6,0,1), (6,-6,-1), (-6,0,-1), (-6,6,1)								
**LiNbO3**	**(0,4,14)**	**8.374**	**70.12**	**17.304**	**17.4**	**3.22**	**56.08**	**40.25**	**69.03**
	Equiv. Refl.: (4,-4,14), (-4,0,14)								
**LiNbO3**	**(1,1,18)**	**8.402**	**70.65**	**17.248**	**17.4**	**3.128**	**54.57**	**39.1**	**67.13**
	Equiv. Refl.: (1,-2,18), (2,-1,18), (-1,2,18), (-1,-1,18), (-2,1,18)								
**Quartz**	**(-3,6,-4)**	**8.852**	**83.77**	**16.696**	**14.9**	**0.972**	**14.48**	**12.14**	**18.9**
**Quartz**	**(-3,-3,4)**	**8.852**	**83.77**	**16.692**	**14.9**	**0.972**	**14.48**	**12.14**	**18.9**
**Quartz**	**(6,-3,4)**	**8.852**	**83.77**	**16.686**	**14.9**	**0.972**	**14.48**	**12.14**	**18.89**
**Quartz**	**(6,-3,-4)**	**8.852**	**83.77**	**16.685**	**14.9**	**0.972**	**14.47**	**12.14**	**18.89**
**Quartz**	**(-3,6,4)**	**8.852**	**83.77**	**16.683**	**14.9**	**0.972**	**14.47**	**12.14**	**18.89**
	Equiv. Refl.: (-3,-3,-4)								
**Quartz**	**(-5,2,-5)**	**8.557**	**73.93**	**15.941**	**13.3**	**2.565**	**34**	**32.06**	**46.73**
	Equiv. Refl.: (-5,3,5)								
**Quartz**	**(3,-5,-5)**	**8.557**	**73.93**	**15.94**	**13.3**	**2.565**	**34**	**32.06**	**46.73**
**Quartz**	**(2,3,-5)**	**8.557**	**73.93**	**15.939**	**13.3**	**2.565**	**34**	**32.06**	**46.73**
	Equiv. Refl.: (2,-5,5), (3,2,5)								
**Quartz**	**(5,-2,5)**	**8.557**	**73.93**	**15.776**	**13.3**	**2.565**	**34**	**32.06**	**46.73**
	Equiv. Refl.: (5,-3,-5)								

Ei = Cryst	8.905 Refl (h,k,l)	keV EB [keV]	ΘB [°]	∫ IR dΘ [µrad]	Width [µrad]	Ei cotΘ [meV/µrad]	ΔE [meV]	ΔEg 2m, 50µm [meV]	ΔEt 2m, 50µm [meV]
**Quartz**	**(-2,5,-5)**	**8.557**	**73.93**	**15.774**	**13.3**	**2.565**	**34**	**32.06**	**46.73**
	Equiv. Refl.: (-2,-3,5), (-3,5,5)								
**Quartz**	**(-3,-2,-5)**	**8.557**	**73.93**	**15.773**	**13.3**	**2.565**	**34**	**32.06**	**46.73**
**Quartz**	**(3,-6,4)**	**8.852**	**83.77**	**15.717**	**14.9**	**0.972**	**14.48**	**12.14**	**18.9**
**Quartz**	**(3,3,-4)**	**8.852**	**83.77**	**15.713**	**14.9**	**0.972**	**14.48**	**12.14**	**18.9**
**Quartz**	**(-6,3,-4)**	**8.852**	**83.77**	**15.707**	**14.9**	**0.972**	**14.48**	**12.14**	**18.89**
**Quartz**	**(-6,3,4)**	**8.852**	**83.77**	**15.706**	**14.9**	**0.972**	**14.47**	**12.14**	**18.89**
**Quartz**	**(3,3,4)**	**8.852**	**83.77**	**15.703**	**14.9**	**0.972**	**14.47**	**12.14**	**18.89**
	Equiv. Refl.: (3,-6,-4)								
**Sapph**	**(1,5,-4)**	**8.591**	**74.74**	**15.618**	**13.5**	**2.429**	**32.79**	**30.37**	**44.69**
	Equiv. Refl.: (1,-6,4), (5,1,4), (5,-6,-4), (6,-1,4), (6,-5,-4), (-1,6,-4), (-1,-5,4), (-5,6,4), (-5,-1,-4) (-6,1,-4), (-6,5,4)								
**Quartz**	**(5,0,-4)**	**8.609**	**75.19**	**14.491**	**12.3**	**2.354**	**28.93**	**29.42**	**41.26**
	Equiv. Refl.: (5,-5,4), (-5,0,4), (-5,5,-4)								
**Quartz**	**(0,5,4)**	**8.609**	**75.19**	**14.49**	**12.3**	**2.354**	**28.93**	**29.42**	**41.26**
	Equiv. Refl.: (0,-5,-4)								
**Quartz**	**(-1,6,3)**	**8.812**	**81.7**	**13.475**	**11.9**	**1.299**	**15.41**	**16.23**	**22.38**
	Equiv. Refl.: (-1,-5,-3)								
**Quartz**	**(-5,6,-3)**	**8.812**	**81.7**	**13.471**	**11.9**	**1.299**	**15.41**	**16.23**	**22.38**
	Equiv. Refl.: (-5,-1,3)								
**Quartz**	**(6,-1,-3)**	**8.812**	**81.7**	**13.47**	**11.9**	**1.299**	**15.41**	**16.23**	**22.38**
**Quartz**	**(6,-5,3)**	**8.812**	**81.7**	**13.469**	**11.9**	**1.299**	**15.41**	**16.23**	**22.38**
**Quartz**	**(1,5,3)**	**8.812**	**81.7**	**12.95**	**11.9**	**1.299**	**15.41**	**16.23**	**22.38**
	Equiv. Refl.: (1,-6,-3)								
**Quartz**	**(5,-6,3)**	**8.812**	**81.7**	**12.947**	**11.9**	**1.299**	**15.41**	**16.23**	**22.38**
	Equiv. Refl.: (-6,1,3)								
**Quartz**	**(5,1,-3)**	**8.812**	**81.7**	**12.946**	**11.9**	**1.299**	**15.41**	**16.23**	**22.38**
**Quartz**	**(-6,5,-3)**	**8.812**	**81.7**	**12.945**	**11.9**	**1.299**	**15.41**	**16.23**	**22.38**
**Sapph**	**(1,2,-16)**	**8.612**	**75.25**	**10.475**	**9.4**	**2.344**	**22.06**	**29.3**	**36.68**
	Equiv. Refl.: (1,-3,16), (2,1,16), (2,-3,-16), (3,-1,16), (3,-2,-16), (-1,3,-16), (-1,-2,16), (-2,3,16), (-2,-1,-16) (-3,1,-16), (-3,2,16)								
**Sapph**	**(1,3,-14)**	**8.607**	**75.12**	**10.182**	**9.1**	**2.365**	**21.53**	**29.57**	**36.57**
	Equiv. Refl.: (1,-4,14), (3,1,14), (3,-4,-14), (4,-1,14), (4,-3,-14), (-1,4,-14), (-1,-3,14), (-3,4,14), (-3,-1,-14) (-4,1,-14), (-4,3,14)								
**Sapph**	**(2,4,-8)**	**8.829**	**82.51**	**8.908**	**9.2**	**1.171**	**10.8**	**14.64**	**18.19**
	Equiv. Refl.: (2,-6,8), (4,2,8), (4,-6,-8), (6,-2,8), (6,-4,-8), (-2,6,-8), (-2,-4,8), (-4,6,8), (-4,-2,-8) (-6,2,-8), (-6,4,8)								
**LiNbO3**	**(2,4,-11)**	**8.851**	**83.66**	**8.664**	**14.5**	**0.989**	**14.3**	**12.36**	**18.9**
	Equiv. Refl.: (2,-6,11), (4,2,11), (4,-6,-11), (6,-2,11), (6,-4,-11), (-2,6,-11), (-2,-4,11), (-4,6,11), (-4,-2,-11) (-6,2,-11), (-6,4,11)								
**Quartz**	**(2,2,6)**	**8.534**	**73.41**	**8.151**	**7.1**	**2.652**	**18.7**	**33.15**	**38.07**
**Quartz**	**(2,-4,6)**	**8.534**	**73.41**	**8.15**	**7.1**	**2.652**	**18.7**	**33.15**	**38.07**
	Equiv. Refl.: (2,-4,-6)								
**Quartz**	**(2,2,-6)**	**8.534**	**73.41**	**8.149**	**7.1**	**2.652**	**18.7**	**33.15**	**38.07**
**Quartz**	**(-4,2,-6)**	**8.534**	**73.41**	**8.148**	**7**	**2.652**	**18.7**	**33.15**	**38.06**
**Quartz**	**(-4,2,6)**	**8.534**	**73.41**	**8.146**	**7**	**2.652**	**18.69**	**33.15**	**38.06**
**Quartz**	**(-2,4,6)**	**8.534**	**73.41**	**8.032**	**7.1**	**2.652**	**18.7**	**33.15**	**38.07**
	Equiv. Refl.: (-2,-2,-6)								
**Quartz**	**(-2,4,-6)**	**8.534**	**73.41**	**8.031**	**7.1**	**2.652**	**18.7**	**33.15**	**38.07**
	Equiv. Refl.: (-2,-2,6)								
**Quartz**	**(4,-2,6)**	**8.534**	**73.41**	**8.027**	**7**	**2.652**	**18.7**	**33.15**	**38.06**

Ei =	8.905	keV						ΔEg	ΔEt
Cryst	Refl	EB	ΘB	∫ IR dΘ	Width	Ei cotΘ	ΔE	2m, 50µm	2m, 50µm
	(h,k,l)	[keV]	[°]	[µrad]	[µrad]	[meV/µrad]	[meV]	[meV]	[meV]
**Quartz**	**(4,-2,-6)**	**8.534**	**73.41**	**8.026**	**7**	**2.652**	**18.69**	**33.15**	**38.06**
**Sapph**	**(1,5,5)**	**8.71**	**77.97**	**7.276**	**7.1**	**1.897**	**13.46**	**23.71**	**27.27**
	Equiv. Refl.: (1,-6,-5), (5,1,-5), (5,-6,5), (6,-1,-5), (6,-5,5), (-1,6,5), (-1,-5,-5), (-5,6,-5), (-5,-1,5) (-6,1,5), (-6,5,-5)								
**Quartz**	**(-2,1,-7)**	**8.416**	**70.93**	**7.058**	**6.1**	**3.078**	**18.87**	**38.48**	**42.85**
**Quartz**	**(1,-2,-7)**	**8.416**	**70.93**	**7.057**	**6.1**	**3.078**	**18.86**	**38.48**	**42.85**
	Equiv. Refl.: (-2,1,7)								
**Quartz**	**(1,1,7)**	**8.416**	**70.93**	**7.055**	**6.1**	**3.078**	**18.86**	**38.48**	**42.85**
**Quartz**	**(1,-2,7)**	**8.416**	**70.93**	**7.054**	**6.1**	**3.078**	**18.86**	**38.48**	**42.85**
**Quartz**	**(1,1,-7)**	**8.416**	**70.93**	**7.053**	**6.1**	**3.078**	**18.85**	**38.48**	**42.85**
**Quartz**	**(2,-1,7)**	**8.416**	**70.93**	**6.787**	**6.1**	**3.078**	**18.87**	**38.48**	**42.85**
	Equiv. Refl.: (2,-1,-7)								
**Quartz**	**(-1,2,7)**	**8.416**	**70.93**	**6.785**	**6.1**	**3.078**	**18.86**	**38.48**	**42.85**
**Quartz**	**(-1,2,-7)**	**8.416**	**70.93**	**6.783**	**6.1**	**3.078**	**18.86**	**38.48**	**42.85**
	Equiv. Refl.: (-1,-1,-7)								
**Quartz**	**(-1,-1,7)**	**8.416**	**70.93**	**6.782**	**6.1**	**3.078**	**18.85**	**38.48**	**42.85**
**Quartz**	**(-1,-3,6)**	**8.658**	**76.47**	**6.279**	**5.7**	**2.142**	**12.25**	**26.78**	**29.45**
**Quartz**	**(-1,4,-6)**	**8.658**	**76.47**	**6.278**	**5.7**	**2.142**	**12.25**	**26.78**	**29.45**
**Quartz**	**(4,-3,-6)**	**8.658**	**76.47**	**6.276**	**5.7**	**2.142**	**12.25**	**26.78**	**29.45**
**Quartz**	**(4,-1,6)**	**8.658**	**76.47**	**6.274**	**5.7**	**2.142**	**12.24**	**26.78**	**29.45**
	Equiv. Refl.: (-3,4,6), (-3,-1,-6)								
**Quartz**	**(-3,5,-5)**	**8.557**	**73.93**	**6.116**	**5.5**	**2.565**	**14.07**	**32.06**	**35.01**
**Quartz**	**(-3,-2,5)**	**8.557**	**73.93**	**6.115**	**5.5**	**2.565**	**14.07**	**32.06**	**35.01**
**Quartz**	**(-2,5,5)**	**8.557**	**73.93**	**6.113**	**5.5**	**2.565**	**14.06**	**32.06**	**35.01**
**Quartz**	**(-2,-3,-5)**	**8.557**	**73.93**	**6.112**	**5.5**	**2.565**	**14.06**	**32.06**	**35.01**
**Quartz**	**(5,-2,-5)**	**8.557**	**73.93**	**6.108**	**5.5**	**2.565**	**14.05**	**32.06**	**35.01**
	Equiv. Refl.: (5,-3,5)								
**Quartz**	**(1,3,-6)**	**8.658**	**76.47**	**6.056**	**5.7**	**2.142**	**12.25**	**26.78**	**29.45**
**Quartz**	**(1,-4,6)**	**8.658**	**76.47**	**6.055**	**5.7**	**2.142**	**12.25**	**26.78**	**29.45**
**Quartz**	**(-4,3,6)**	**8.658**	**76.47**	**6.054**	**5.7**	**2.142**	**12.25**	**26.78**	**29.45**
**Quartz**	**(3,1,6)**	**8.658**	**76.47**	**6.053**	**5.7**	**2.142**	**12.24**	**26.78**	**29.45**
	Equiv. Refl.: (3,-4,-6)								
**Quartz**	**(-4,1,-6)**	**8.658**	**76.47**	**6.052**	**5.7**	**2.142**	**12.24**	**26.78**	**29.45**
**Quartz**	**(-4,-2,-3)**	**8.443**	**71.46**	**6.012**	**5**	**2.987**	**14.89**	**37.34**	**40.19**
**Quartz**	**(-4,6,3)**	**8.443**	**71.46**	**6.011**	**5**	**2.987**	**14.88**	**37.34**	**40.19**
**Quartz**	**(6,-4,-3)**	**8.443**	**71.46**	**6.009**	**5**	**2.987**	**14.88**	**37.34**	**40.19**
**Quartz**	**(6,-2,3)**	**8.443**	**71.46**	**6.008**	**5**	**2.987**	**14.88**	**37.34**	**40.19**
**Quartz**	**(-2,6,-3)**	**8.443**	**71.46**	**6.007**	**5**	**2.987**	**14.88**	**37.34**	**40.19**
	Equiv. Refl.: (-2,-4,3)								
**Quartz**	**(3,2,-5)**	**8.557**	**73.93**	**5.999**	**5.5**	**2.565**	**14.07**	**32.06**	**35.01**
	Equiv. Refl.: (3,-5,5)								
**Quartz**	**(2,-5,-5)**	**8.557**	**73.93**	**5.996**	**5.5**	**2.565**	**14.06**	**32.06**	**35.01**
**Quartz**	**(2,3,5)**	**8.557**	**73.93**	**5.995**	**5.5**	**2.565**	**14.06**	**32.06**	**35.01**
**Quartz**	**(-5,2,5)**	**8.557**	**73.93**	**5.992**	**5.5**	**2.565**	**14.05**	**32.06**	**35.01**
**Quartz**	**(-5,3,-5)**	**8.557**	**73.93**	**5.991**	**5.5**	**2.565**	**14.05**	**32.06**	**35.01**
**Quartz**	**(2,4,3)**	**8.443**	**71.46**	**5.719**	**4.9**	**2.987**	**14.78**	**37.34**	**40.15**
	Equiv. Refl.: (2,-6,-3)								
**Quartz**	**(-6,4,-3)**	**8.443**	**71.46**	**5.717**	**4.9**	**2.987**	**14.77**	**37.34**	**40.15**
**Quartz**	**(-6,2,3)**	**8.443**	**71.46**	**5.716**	**4.9**	**2.987**	**14.77**	**37.34**	**40.15**

Ei = Cryst	8.905 Refl (h,k,l)	keV EB [keV]	ΘB [°]	∫ IR dΘ [µrad]	Width [µrad]	Ei cotΘ [meV/µrad]	ΔE [meV]	ΔEg 2m, 50µm [meV]	ΔEt 2m, 50µm [meV]
**Quartz**	**(4,2,-3)**	**8.443**	**71.46**	**5.715**	**4.9**	**2.987**	**14.77**	**37.34**	**40.15**
	Equiv. Refl.: (4,-6,3)								
**Quartz**	**(-2,6,3)**	**8.443**	**71.46**	**5.339**	**4.9**	**2.987**	**14.78**	**37.34**	**40.15**
	Equiv. Refl.: (-2,-4,-3)								
**Quartz**	**(6,-4,3)**	**8.443**	**71.46**	**5.338**	**4.9**	**2.987**	**14.77**	**37.34**	**40.15**
**Quartz**	**(6,-2,-3)**	**8.443**	**71.46**	**5.337**	**4.9**	**2.987**	**14.77**	**37.34**	**40.15**
**Quartz**	**(-4,6,-3)**	**8.443**	**71.46**	**5.336**	**4.9**	**2.987**	**14.77**	**37.34**	**40.15**
	Equiv. Refl.: (-4,-2,3)								
**Quartz**	**(2,-2,-7)**	**8.542**	**73.57**	**5.147**	**4.8**	**2.625**	**12.59**	**32.82**	**35.15**
	Equiv. Refl.: (-2,2,7)								
**Quartz**	**(2,0,7)**	**8.542**	**73.57**	**5.145**	**4.8**	**2.625**	**12.59**	**32.82**	**35.15**
	Equiv. Refl.: (-2,0,-7)								
**Quartz**	**(0,2,-7)**	**8.542**	**73.57**	**5.136**	**4.8**	**2.625**	**12.57**	**32.82**	**35.14**
	Equiv. Refl.: (0,-2,7)								
**Quartz**	**(4,2,3)**	**8.443**	**71.46**	**5.113**	**5**	**2.987**	**14.89**	**37.34**	**40.19**
**Quartz**	**(4,-6,-3)**	**8.443**	**71.46**	**5.112**	**5**	**2.987**	**14.88**	**37.34**	**40.19**
**Quartz**	**(-6,4,3)**	**8.443**	**71.46**	**5.111**	**5**	**2.987**	**14.88**	**37.34**	**40.19**
**Quartz**	**(-6,2,-3)**	**8.443**	**71.46**	**5.11**	**5**	**2.987**	**14.88**	**37.34**	**40.19**
**Quartz**	**(2,-6,3)**	**8.443**	**71.46**	**5.109**	**5**	**2.987**	**14.88**	**37.34**	**40.19**
**Quartz**	**(2,4,-3)**	**8.443**	**71.46**	**5.108**	**5**	**2.987**	**14.87**	**37.34**	**40.19**
**Sapph**	**(1,5,-1)**	**8.39**	**70.42**	**5.058**	**4.9**	**3.168**	**15.37**	**39.6**	**42.48**
	Equiv. Refl.: (1,-6,1), (5,1,1), (5,-6,-1), (6,-1,1), (6,-5,-1), (-1,6,-1), (-1,-5,1), (-5,6,1), (-5,-1,-1) (-6,1,-1), (-6,5,1)								
**Quartz**	**(-5,6,-2)**	**8.43**	**71.21**	**4.942**	**4.5**	**3.03**	**13.59**	**37.88**	**40.24**
	Equiv. Refl.: (-5,-1,2)								
**Quartz**	**(-1,-5,-2)**	**8.43**	**71.21**	**4.941**	**4.5**	**3.03**	**13.59**	**37.88**	**40.24**
**Quartz**	**(6,-1,-2)**	**8.43**	**71.21**	**4.94**	**4.5**	**3.03**	**13.58**	**37.88**	**40.24**
	Equiv. Refl.: (6,-5,2), (-1,6,2)								
**Quartz**	**(5,1,-2)**	**8.43**	**71.21**	**4.851**	**4.5**	**3.03**	**13.59**	**37.88**	**40.24**
	Equiv. Refl.: (5,-6,2)								
**Quartz**	**(1,5,2)**	**8.43**	**71.21**	**4.85**	**4.5**	**3.03**	**13.59**	**37.88**	**40.24**
	Equiv. Refl.: (1,-6,-2), (-6,5,-2)								
**Quartz**	**(-6,1,2)**	**8.43**	**71.21**	**4.849**	**4.5**	**3.03**	**13.58**	**37.88**	**40.24**
**Quartz**	**(7,-3,0)**	**8.862**	**84.4**	**4.715**	**5.4**	**0.873**	**4.71**	**10.91**	**11.89**
	Equiv. Refl.: (7,-4,0), (-3,7,0), (-3,-4,0), (-4,7,0), (-4,-3,0)								
**Quartz**	**(3,1,-6)**	**8.658**	**76.47**	**4.603**	**4.4**	**2.142**	**9.38**	**26.78**	**28.38**
**Quartz**	**(3,-4,6)**	**8.658**	**76.47**	**4.602**	**4.4**	**2.142**	**9.38**	**26.78**	**28.38**
**Quartz**	**(-4,1,6)**	**8.658**	**76.47**	**4.598**	**4.4**	**2.142**	**9.38**	**26.78**	**28.37**
	Equiv. Refl.: (-4,3,-6)								
**Quartz**	**(1,-4,-6)**	**8.658**	**76.47**	**4.594**	**4.4**	**2.142**	**9.37**	**26.78**	**28.37**
**Quartz**	**(1,3,6)**	**8.658**	**76.47**	**4.592**	**4.4**	**2.142**	**9.37**	**26.78**	**28.37**
**Quartz**	**(2,0,-7)**	**8.542**	**73.57**	**4.492**	**4.3**	**2.625**	**11.31**	**32.82**	**34.71**
	Equiv. Refl.: (-2,0,7)								
**Quartz**	**(2,-2,7)**	**8.542**	**73.57**	**4.491**	**4.3**	**2.625**	**11.31**	**32.82**	**34.71**
	Equiv. Refl.: (-2,2,-7)								
**Quartz**	**(0,2,7)**	**8.542**	**73.57**	**4.477**	**4.3**	**2.625**	**11.28**	**32.82**	**34.7**
**Quartz**	**(0,-2,-7)**	**8.542**	**73.57**	**4.476**	**4.3**	**2.625**	**11.28**	**32.82**	**34.7**
**Sapph**	**(2,4,7)**	**8.633**	**75.81**	**4.46**	**4.6**	**2.251**	**10.37**	**28.14**	**29.99**
	Equiv. Refl.: (2,-6,-7), (4,2,-7), (4,-6,7), (6,-2,-7), (6,-4,7), (-2,6,7), (-2,-4,-7), (-4,6,-7), (-4,-2,7) (-6,2,7), (-6,4,-7)								
**Quartz**	**(-3,-1,6)**	**8.658**	**76.47**	**4.165**	**4.4**	**2.142**	**9.38**	**26.78**	**28.38**

Ei =	8.905	keV						ΔEg	ΔEt
Cryst	Refl	EB	ΘB	∫ IR dΘ	Width	Ei cotΘ	ΔE	2m, 50µm	2m, 50µm
	(h,k,l)	[keV]	[°]	[µrad]	[µrad]	[meV/µrad]	[meV]	[meV]	[meV]
Quartz	(-3,4,-6)	8.658	76.47	4.164	4.4	2.142	9.38	26.78	28.38
Quartz	(4,-1,-6)	8.658	76.47	4.162	4.4	2.142	9.38	26.78	28.37
	Equiv. Refl.: (4,-3,6)								
Quartz	(-1,4,6)	8.658	76.47	4.16	4.4	2.142	9.37	26.78	28.37
Quartz	(-1,-3,-6)	8.658	76.47	4.158	4.4	2.142	9.37	26.78	28.37
Quartz	(3,4,0)	8.862	84.4	3.95	5.4	0.873	4.71	10.91	11.89
	Equiv. Refl.: (3,-7,0), (4,3,0), (4,-7,0), (-7,3,0), (-7,4,0)								
Quartz	(6,0,-1)	8.817	81.93	2.952	3.8	1.262	4.81	15.78	16.5
	Equiv. Refl.: (6,-6,1), (-6,0,1), (-6,6,-1)								
Quartz	(0,6,1)	8.817	81.93	2.951	3.8	1.262	4.81	15.78	16.5
	Equiv. Refl.: (0,-6,-1)								
LiNbO3	(3,4,5)	8.749	79.24	2.882	6.3	1.692	10.58	21.15	23.65
	Equiv. Refl.: (3,-7,-5), (4,3,-5), (4,-7,5), (7,-3,-5), (7,-4,5), (-3,7,5), (-3,-4,-5), (-4,7,-5), (-4,-3,5) (-7,3,5), (-7,4,-5)								
Quartz	(4,1,5)	8.802	81.27	2.213	3	1.368	4.04	17.1	17.57
	Equiv. Refl.: (4,-5,-5)								
Quartz	(-5,1,-5)	8.802	81.27	2.212	3	1.368	4.04	17.1	17.57
Sapph	(2,3,11)	8.4	70.62	2.21	2.9	3.132	9.04	39.16	40.19
	Equiv. Refl.: (2,-5,-11), (3,2,-11), (3,-5,11), (5,-2,-11), (5,-3,11), (-2,5,11), (-2,-3,-11), (-3,5,-11), (-3,-2,11) (-5,2,11), (-5,3,-11), (-5,4,5)								
Quartz	(1,4,-5)	8.802	81.27	2.204	2.9	1.368	4.03	17.1	17.57
	Equiv. Refl.: (1,-5,5)								
Quartz	(0,6,0)	8.742	79.02	1.987	2.8	1.728	4.87	21.6	22.14
	Equiv. Refl.: (0,-6,0), (6,0,0), (6,-6,0), (-6,0,0), (-6,6,0)								
LiNbO3	(3,4,-1)	8.47	72.01	1.934	4.1	2.891	11.79	36.14	38.01
	Equiv. Refl.: (3,-7,1), (4,3,1), (4,-7,-1), (7,-3,1), (7,-4,-1), (-3,7,-1), (-3,-4,1), (-4,7,1), (-4,-3,-1) (-7,3,-1), (-7,4,1)								
Quartz	(5,-1,5)	8.802	81.27	1.674	3	1.368	4.04	17.1	17.57
	Equiv. Refl.: (-4,5,5), (-4,-1,-5)								
Quartz	(5,-4,-5)	8.802	81.27	1.671	3	1.368	4.04	17.1	17.57
Quartz	(-1,5,-5)	8.802	81.27	1.666	2.9	1.368	4.03	17.1	17.57
	Equiv. Refl.: (-1,-4,5)								
Quartz	(6,-1,2)	8.43	71.21	1.438	1.9	3.03	5.74	37.88	38.31
	Equiv. Refl.: (6,-5,-2), (-5,-1,-2)								
Quartz	(-1,6,-2)	8.43	71.21	1.437	1.9	3.03	5.74	37.88	38.31
	Equiv. Refl.: (-1,-5,2), (-5,6,2)								
Quartz	(5,0,4)	8.609	75.19	1.326	2	2.354	4.61	29.42	29.78
	Equiv. Refl.: (-5,0,-4)								
Quartz	(5,-5,-4)	8.609	75.19	1.325	2	2.354	4.61	29.42	29.78
	Equiv. Refl.: (-5,5,4)								
Quartz	(0,5,-4)	8.609	75.19	1.321	2	2.354	4.6	29.42	29.78
	Equiv. Refl.: (0,-5,4)								
Quartz	(5,1,2)	8.43	71.21	1.312	1.9	3.03	5.74	37.88	38.31
	Equiv. Refl.: (-6,1,-2), (-6,5,2)								
Quartz	(1,5,-2)	8.43	71.21	1.311	1.9	3.03	5.74	37.88	38.31
	Equiv. Refl.: (1,-6,2), (5,-6,-2)								
LiNbO3	(2,5,3)	8.787	80.65	1.251	4.2	1.467	6.11	18.33	19.33
	Equiv. Refl.: (2,5,-3), (2,-7,3), (2,-7,-3), (5,2,3), (5,2,-3), (5,-7,3), (5,-7,-3), (7,-2,3), (7,-2,-3) (7,-5,3), (7,-5,-3), (-2,7,3), (-2,7,-3), (-2,-5,3), (-2,-5,-3), (-5,7,3), (-5,7,-3), (-5,-2,3) (-5,-2,-3), (-7,2,3), (-7,2,-3), (-7,5,3), (-7,5,-3)								
LiNbO3	(1,2,17)	8.445	71.51	1.173	3	2.978	8.99	37.22	38.29
	Equiv. Refl.: (1,-3,-17), (2,1,-17), (2,-3,17), (3,-1,-17), (3,-2,17), (-1,3,17), (-1,-2,-17), (-2,3,-17), (-2,-1,17)								

	(-3,1,17), (-3,2,-17)								
**Sapph**	**(0,5,-8)**	**8.436**	**71.32**	**0.812**	**1.4**	**3.011**	**4.31**	**37.64**	**37.89**
	Equiv. Refl.: (0,-5,8), (5,0,8), (5,-5,-8), (-5,0,-8), (-5,5,8)								
**LiNbO3**	**(2,3,-13)**	**8.398**	**70.58**	**0.426**	**1.7**	**3.14**	**5.42**	**39.25**	**39.62**
	Equiv. Refl.: (2,-5,13), (3,2,13), (3,-5,-13), (5,-2,13), (5,-3,-13), (-2,5,-13), (-2,-3,13), (-3,5,13), (-3,-2,-13) (-5,2,-13), (-5,3,13)								
**Sapph**	**(1,5,2)**	**8.43**	**71.21**	**0.147**	**0.5**	**3.03**	**1.59**	**37.87**	**37.9**
	Equiv. Refl.: (1,-6,-2), (5,1,-2), (5,-6,2), (6,-1,-2), (6,-5,2), (-1,6,2), (-1,-5,-2), (-5,6,-2), (-5,-1,2) (-6,1,2), (-6,5,-2)								

Ei =	9.659	keV						ΔEg	ΔEt
**Cryst**	**Refl**	**EB**	**ΘB**	**∫ IR dΘ**	**Width**	**Ei cotΘ**	**ΔE**	**2m, 50μm**	**2m, 50μm**
	**(h,k,l)**	**[keV]**	**[°]**	**[μrad]**	**[μrad]**	**[meV/μrad]**	**[meV]**	**[meV]**	**[meV]**
**Ge**	**(0,6,6)**	**9.298**	**74.29**	**18.4**	**17.2**	**2.718**	**46.64**	**33.97**	**57.7**
	Equiv. Refl.: (2,2,8)								
**Ge**	**(5,5,5)**	**9.49**	**79.26**	**16.5**	**16.6**	**1.832**	**30.41**	**22.91**	**38.07**
	Equiv. Refl.: (1,5,7)								
**Si**	**(0,0,8)**	**9.132**	**70.98**	**10**	**8.5**	**3.33**	**28.14**	**41.63**	**50.24**
**Si**	**(3,3,7)**	**9.343**	**75.31**	**7.9**	**7.2**	**2.533**	**18.27**	**31.66**	**36.55**
**LiNbO3**	**(4,4,0)**	**9.634**	**85.84**	**57.8**	**56.2**	**0.702**	**39.46**	**8.78**	**40.43**
	Equiv. Refl.: (4,-8,0), (8,-4,0), (-4,8,0), (-4,-4,0), (-8,4,0)								
**LiNbO3**	**(3,4,-10)**	**9.567**	**82.1**	**41**	**37.5**	**1.34**	**50.27**	**16.75**	**52.99**
	Equiv. Refl.: (4,-7,-10), (7,-4,-10), (-3,7,-10), (-4,-3,-10), (-7,3,-10)								
**LiNbO3**	**(3,-7,10)**	**9.567**	**82.1**	**39.4**	**37.5**	**1.34**	**50.27**	**16.75**	**52.99**
	Equiv. Refl.: (4,3,10), (7,-3,10), (-3,-4,10), (-4,7,10), (-7,4,10)								
**Sapph**	**(1,5,-10)**	**9.641**	**86.47**	**30.8**	**28**	**0.596**	**16.68**	**7.45**	**18.27**
	Equiv. Refl.: (1,-6,10), (5,1,10), (5,-6,-10), (6,-1,10), (6,-5,-10), (-1,6,-10), (-1,-5,10), (-5,6,10), (-5,-1,-10) (-6,1,-10), (-6,5,10)								
**LiNbO3**	**(0,-5,14)**	**9.356**	**75.6**	**23.5**	**20.9**	**2.479**	**51.89**	**30.99**	**60.44**
	Equiv. Refl.: (5,0,14), (-5,5,14)								
**Quartz**	**(1,4,-6)**	**9.589**	**83.08**	**22.1**	**18.7**	**1.172**	**21.95**	**14.65**	**26.39**
	Equiv. Refl.: (1,-5,6), (4,1,6), (4,-5,-6), (5,-1,6), (5,-4,-6), (-1,5,-6), (-1,-4,6), (-4,5,6), (-4,-1,-6) (-5,1,-6), (-5,4,6)								
**LiNbO3**	**(0,5,-14)**	**9.356**	**75.6**	**21.2**	**20.9**	**2.479**	**51.89**	**30.99**	**60.44**
	Equiv. Refl.: (5,-5,-14), (-5,0,-14)								
**LiNbO3**	**(2,2,-18)**	**9.38**	**76.2**	**20**	**18.8**	**2.372**	**44.6**	**29.65**	**53.56**
	Equiv. Refl.: (2,3,-16), (2,-4,-18), (3,-5,-16), (4,-2,-18), (5,-3,-16), (-2,4,-18), (-2,5,-16), (-2,-2,-18), (-3,-2,-16) (-4,2,-18), (-5,2,-16)								
**LiNbO3**	**(2,2,18)**	**9.38**	**76.2**	**19.2**	**18.8**	**2.372**	**44.6**	**29.65**	**53.56**
	Equiv. Refl.: (2,-4,18), (4,-2,18), (-2,4,18), (-2,-2,18), (-4,2,18)								
**LiNbO3**	**(1,6,-2)**	**9.162**	**71.54**	**19.1**	**17.5**	**3.225**	**56.39**	**40.31**	**69.32**
	Equiv. Refl.: (6,-7,-2), (7,-6,-2), (-1,7,-2), (-6,-1,-2), (-7,1,-2)								
**LiNbO3**	**(1,-7,2)**	**9.162**	**71.54**	**18.6**	**17.5**	**3.225**	**56.39**	**40.31**	**69.32**
	Equiv. Refl.: (6,1,2), (7,-1,2), (-1,-6,2), (-6,7,2), (-7,6,2)								
**LiNbO3**	**(2,-5,16)**	**9.377**	**76.12**	**18.2**	**18.4**	**2.387**	**43.83**	**29.84**	**53.02**
	Equiv. Refl.: (3,2,16), (5,-2,16), (-2,-3,16), (-3,5,16), (-5,3,16)								
**LiNbO3**	**(0,2,-20)**	**9.366**	**75.85**	**17.5**	**16.6**	**2.435**	**40.43**	**30.44**	**50.61**
	Equiv. Refl.: (2,-2,-20), (-2,0,-20), (4,-1,7), (4,-3,-7), (-1,4,-7), (-1,-3,7), (-3,4,7), (-3,-1,-7)								
**LiNbO3**	**(3,4,8)**	**9.183**	**71.95**	**17.1**	**15.7**	**3.148**	**49.28**	**39.36**	**63.06**
	Equiv. Refl.: (4,-7,8), (7,-4,8), (-3,7,8), (-4,-3,8), (-7,3,8)								
**Quartz**	**(1,3,-7)**	**9.595**	**83.4**	**16.8**	**15**	**1.118**	**16.77**	**13.97**	**21.83**
	Equiv. Refl.: (1,-4,7), (3,1,7), (3,-4,-7), (-4,1,-7), (-4,3,7)								
**LiNbO3**	**(0,-2,20)**	**9.366**	**75.85**	**16.4**	**16.6**	**2.435**	**40.43**	**30.44**	**50.61**
	Equiv. Refl.: (2,0,20), (-2,2,20)								
**LiNbO3**	**(3,-7,-8)**	**9.183**	**71.95**	**15.8**	**15.7**	**3.148**	**49.28**	**39.36**	**63.06**
	Equiv. Refl.: (4,3,-8), (7,-3,-8), (-3,-4,-8), (-4,7,-8), (-7,4,-8)								
**LiNbO3**	**(1,6,4)**	**9.292**	**74.15**	**15.3**	**14.7**	**2.742**	**40.27**	**34.28**	**52.88**
	Equiv. Refl.: (6,-7,4), (7,-6,4), (-1,7,4), (-6,-1,4), (-7,1,4)								
**LiNbO3**	**(2,-7,-6)**	**9.089**	**70.21**	**15.2**	**14.4**	**3.475**	**49.92**	**43.44**	**66.17**
	Equiv. Refl.: (5,2,-6), (7,-2,-6), (-2,-5,-6), (-5,7,-6), (-7,5,-6)								
**LiNbO3**	**(2,5,6)**	**9.089**	**70.21**	**15.1**	**14.4**	**3.475**	**49.92**	**43.44**	**66.17**
	Equiv. Refl.: (5,-7,6), (7,-5,6), (-2,7,6), (-5,-2,6), (-7,2,6)								

Ei =	9.659	keV						ΔEg	ΔEt
**Cryst**	**Refl**	**EB**	**ΘB**	**∫ IR dΘ**	**Width**	**Ei cotΘ**	**ΔE**	**2m, 50mm**	**2m, 50mm**
	**(h,k,l)**	**[keV]**	**[°]**	**[μrad]**	**[μrad]**	**[meV/mrad]**	**[meV]**	**[meV]**	**[meV]**
**LiNbO3**	**(1,6,7)**	**9.64**	**86.44**	**15**	**22.7**	**0.601**	**13.63**	**7.51**	**15.56**
	Equiv. Refl.: (1,-7,-7), (6,1,-7), (6,-7,7), (7,-1,-7), (7,-6,7), (-1,7,7), (-1,-6,-7), (-6,7,-7), (-6,-1,7) (-7,1,7), (-7,6,-7), (7,-1,0), (7,-6,0), (-1,7,0), (-1,-6,0), (-6,7,0), (-6,-1,0)								
**LiNbO3**	**(2,5,-6)**	**9.089**	**70.21**	**14.8**	**14**	**3.475**	**48.54**	**43.44**	**65.14**
	Equiv. Refl.: (5,-7,-6), (7,-5,-6), (-2,7,-6), (-5,-2,-6), (-7,2,-6), (1,6,0), (1,-7,0), (6,1,0), (6,-7,0) (-7,1,0), (-7,6,0)								
**LiNbO3**	**(1,-7,-4)**	**9.292**	**74.15**	**14.6**	**14.7**	**2.742**	**40.27**	**34.28**	**52.88**
	Equiv. Refl.: (6,1,-4), (7,-1,-4), (-1,-6,-4), (-6,7,-4), (-7,6,-4)								
**LiNbO3**	**(2,-7,6)**	**9.089**	**70.21**	**14.5**	**14**	**3.475**	**48.54**	**43.44**	**65.14**
	Equiv. Refl.: (5,2,6), (7,-2,6), (-2,-5,6), (-5,7,6), (-7,5,6)								
**Sapph**	**(2,3,14)**	**9.362**	**75.76**	**13.5**	**11.5**	**2.451**	**28.27**	**30.64**	**41.69**
	Equiv. Refl.: (2,-5,-14), (3,2,-14), (3,-5,14), (5,-2,-14), (5,-3,14), (-2,5,14), (-2,-3,-14), (-3,5,-14), (-3,-2,14) (-5,2,14), (-5,3,-14)								
**Quartz**	**(0,6,3)**	**9.395**	**76.57**	**11.2**	**9.3**	**2.307**	**21.39**	**28.84**	**35.91**
	Equiv. Refl.: (0,-6,-3), (6,0,-3), (6,-6,3), (-6,0,3), (-6,6,-3)								
**Quartz**	**(7,-1,-1)**	**9.623**	**85.03**	**10.8**	**10.3**	**0.84**	**8.61**	**10.5**	**13.58**
	Equiv. Refl.: (7,-6,1), (-1,7,1), (-1,-6,-1), (-6,7,-1), (-6,-1,1)								
**Sapph**	**(2,4,10)**	**9.282**	**73.94**	**10.4**	**8.8**	**2.781**	**24.35**	**34.77**	**42.45**
	Equiv. Refl.: (2,-6,-10), (4,2,-10), (4,-6,10), (6,-2,-10), (6,-4,10), (-2,6,10), (-2,-4,-10), (-4,6,-10), (-4,-2,10) (-6,2,10), (-6,4,-10)								
**Quartz**	**(1,6,1)**	**9.623**	**85.03**	**10.2**	**10.3**	**0.84**	**8.61**	**10.5**	**13.58**
	Equiv. Refl.: (1,-7,-1), (6,1,-1), (6,-7,1), (-7,1,1), (-7,6,-1)								
**Quartz**	**(2,5,1)**	**9.171**	**71.71**	**10.1**	**8**	**3.193**	**25.7**	**39.91**	**47.47**
	Equiv. Refl.: (2,-7,-1), (5,2,-1), (5,-7,1), (-7,2,1), (-7,5,-1)								
**Quartz**	**(7,-2,-1)**	**9.171**	**71.71**	**10**	**8**	**3.193**	**25.7**	**39.91**	**47.47**
	Equiv. Refl.: (7,-5,1), (-2,7,1), (-2,-5,-1), (-5,7,-1), (-5,-2,1)								
**Quartz**	**(7,-3,3)**	**9.507**	**79.82**	**9.8**	**8.5**	**1.734**	**14.8**	**21.67**	**26.24**
	Equiv. Refl.: (7,-4,-3), (-3,7,-3), (-3,-4,3), (-4,7,3), (-4,-3,-3)								
**Quartz**	**(1,3,7)**	**9.595**	**83.4**	**9.6**	**8.7**	**1.118**	**9.74**	**13.97**	**17.03**
	Equiv. Refl.: (1,-4,-7), (3,4,-3), (3,-7,3), (4,3,3), (4,-7,-3), (7,-2,2), (7,-5,-2), (-2,7,-2), (-2,-5,2) (-5,7,2), (-5,-2,-2), (-7,3,-3), (-7,4,3)								
**Quartz**	**(0,1,-8)**	**9.291**	**74.14**	**9.5**	**7.9**	**2.745**	**21.62**	**34.31**	**40.55**
	Equiv. Refl.: (0,2,-8), (0,3,7), (0,-1,8), (0,-2,8), (0,-3,-7), (1,0,8), (1,-1,-8), (2,0,8), (2,-2,-8) (3,0,-7), (3,1,-7), (3,-3,7), (3,-4,7), (-1,0,-8), (-1,1,8), (-2,0,-8), (-2,2,8), (-3,0,7) (-3,3,-7), (-4,1,7), (-4,3,-7)								
**Quartz**	**(6,-2,-5)**	**9.609**	**84.16**	**9.4**	**8.8**	**0.988**	**8.7**	**12.35**	**15.11**
	Equiv. Refl.: (6,-4,5), (-2,6,5), (-2,-4,-5), (-4,6,-5), (-4,-2,5)								
**Quartz**	**(2,5,-2)**	**9.384**	**76.29**	**9.3**	**7.9**	**2.357**	**18.55**	**29.47**	**34.82**
	Equiv. Refl.: (2,-7,2), (5,2,2), (5,-7,-2), (6,-3,5), (6,-3,-5), (-3,6,5), (-3,6,-5), (-3,-3,5), (-3,-3,-5) (-7,2,-2), (-7,5,2)								
**Quartz**	**(3,3,5)**	**9.498**	**79.52**	**9.1**	**8.1**	**1.787**	**14.52**	**22.34**	**26.65**
	Equiv. Refl.: (3,3,-5), (3,-6,5), (3,-6,-5), (6,-2,5), (6,-4,-5), (-2,6,-5), (-2,-4,5), (-4,6,5), (-4,-2,-5) (-6,3,5), (-6,3,-5)								
**Quartz**	**(2,4,-5)**	**9.609**	**84.16**	**8.9**	**8.8**	**0.988**	**8.68**	**12.35**	**15.1**
	Equiv. Refl.: (2,-6,5), (4,2,5), (4,-6,-5), (-6,2,-5), (-6,4,5)								
**Quartz**	**(4,-1,-7)**	**9.595**	**83.4**	**8.8**	**8.7**	**1.118**	**9.73**	**13.97**	**17.03**
	Equiv. Refl.: (4,-3,7), (-1,4,7), (-1,-3,-7), (-3,4,-7), (-3,-1,7)								
**Quartz**	**(2,4,5)**	**9.609**	**84.16**	**8.7**	**8.8**	**0.988**	**8.69**	**12.35**	**15.1**
	Equiv. Refl.: (2,-6,-5), (4,2,-5), (4,-6,5), (-6,2,5), (-6,4,-5)								
**Sapph**	**(2,5,0)**	**9.395**	**76.58**	**7.9**	**7.1**	**2.305**	**16.3**	**28.82**	**33.11**
	Equiv. Refl.: (2,-7,0), (5,2,0), (5,-7,0), (7,-2,0), (7,-5,0), (-2,7,0), (-2,-5,0), (-5,7,0), (-5,-2,0) (-7,2,0), (-7,5,0)								

Ei =	9.659	keV						ΔEg	ΔEt
**Cryst**	**Refl**	**EB**	**ΘB**	**∫ IR dΘ**	**Width**	**Ei cotΘ**	**ΔE**	**2m, 50mm**	**2m, 50mm**
	**(h,k,l)**	**[keV]**	**[°]**	**[μrad]**	**[μrad]**	**[meV/mrad]**	**[meV]**	**[meV]**	**[meV]**
**Quartz**	**(3,4,2)**	**9.155**	**71.4**	**7.6**	**6.2**	**3.25**	**20.1**	**40.63**	**45.33**
	Equiv. Refl.: (3,-7,-2), (4,3,-2), (4,-7,2), (-7,3,2), (-7,4,-2)								
**Sapph**	**(3,4,2)**	**9.201**	**72.28**	**7.2**	**6.4**	**3.086**	**19.71**	**38.58**	**43.32**
	Equiv. Refl.: (3,-7,-2), (4,3,-2), (4,-7,2), (7,-3,-2), (7,-4,2), (-3,7,2), (-3,-4,-2), (-4,7,-2), (-4,-3,2)<br>(-7,3,2), (-7,4,-2), (0,5,-5), (0,-5,5), (5,0,5), (5,-5,-5), (7,-3,-2), (7,-4,2), (-3,7,2)<br>(-3,-4,-2), (-4,7,-2), (-4,-3,2), (-5,0,-5), (-5,5,5)								
**Quartz**	**(2,5,2)**	**9.384**	**76.29**	**6.9**	**6.1**	**2.357**	**14.49**	**29.47**	**32.84**
	Equiv. Refl.: (2,-7,-2), (5,2,-2), (5,-7,2), (7,-2,-2), (7,-5,2), (-2,7,2), (-2,-5,-2), (-5,7,-2), (-5,-2,2)<br>(-7,2,2), (-7,5,-2)								
**Quartz**	**(1,5,4)**	**9.32**	**74.77**	**6.8**	**5.9**	**2.63**	**15.54**	**32.87**	**36.36**
	Equiv. Refl.: (1,-6,-4), (5,1,-4), (5,-6,4), (-6,1,4), (-6,5,-4)								
**Quartz**	**(6,-1,-4)**	**9.32**	**74.77**	**6.7**	**5.9**	**2.63**	**15.55**	**32.87**	**36.36**
	Equiv. Refl.: (6,-5,4), (-1,6,4), (-1,-5,-4), (-5,6,-4), (-5,-1,4)								
**Sapph**	**(2,5,3)**	**9.504**	**79.71**	**6.5**	**6.2**	**1.754**	**10.91**	**21.92**	**24.49**
	Equiv. Refl.: (2,5,-3), (2,-7,3), (2,-7,-3), (5,2,3), (5,2,-3), (5,-7,3), (5,-7,-3), (7,-2,3), (7,-2,-3)<br>(7,-5,3), (7,-5,-3), (-2,7,3), (-2,7,-3), (-2,-5,3), (-2,-5,-3), (-5,7,3), (-5,7,-3), (-5,-2,3)<br>(-5,-2,-3), (-7,2,3), (-7,2,-3), (-7,5,3), (-7,5,-3)								
**Quartz**	**(1,6,-1)**	**9.623**	**85.03**	**5.8**	**6.3**	**0.84**	**5.25**	**10.5**	**11.74**
	Equiv. Refl.: (1,-7,1), (6,1,1), (6,-7,-1), (-7,1,-1), (-7,6,1)								
**Quartz**	**(0,2,8)**	**9.628**	**85.39**	**5.7**	**6.5**	**0.779**	**5.04**	**9.74**	**10.97**
	Equiv. Refl.: (0,-2,-8), (2,0,-8), (2,-2,8), (-2,0,8), (-2,2,-8)								
**Quartz**	**(7,-1,1)**	**9.623**	**85.03**	**5.6**	**6.3**	**0.84**	**5.26**	**10.5**	**11.74**
	Equiv. Refl.: (7,-6,-1), (-1,7,-1), (-1,-6,1), (-6,7,1), (-6,-1,-1)								
**Quartz**	**(1,1,8)**	**9.517**	**80.16**	**5.1**	**4.5**	**1.676**	**7.61**	**20.95**	**22.29**
	Equiv. Refl.: (1,1,-8), (1,-2,8), (1,-2,-8), (-2,1,8), (-2,1,-8)								
**Sapph**	**(1,3,16)**	**9.367**	**75.88**	**4.9**	**4.6**	**2.43**	**11.29**	**30.38**	**32.41**
	Equiv. Refl.: (1,-4,-16), (3,1,-16), (3,-4,16), (4,-1,-16), (4,-3,16), (-1,4,16), (-1,-3,-16), (-3,4,-16), (-3,-1,16)<br>(-4,1,16), (-4,3,-16)								
**Sapph**	**(2,4,-11)**	**9.536**	**80.85**	**4.7**	**5.1**	**1.556**	**7.86**	**19.45**	**20.98**
	Equiv. Refl.: (2,-6,11), (4,2,11), (4,-6,-11), (6,-2,11), (6,-4,-11), (-2,6,-11), (-2,-4,11), (-4,6,11), (-4,-2,-11)<br>(-6,2,-11), (-6,4,11)								
**Quartz**	**(0,3,-7)**	**9.142**	**71.16**	**4.5**	**3.9**	**3.295**	**12.92**	**41.18**	**43.16**
	Equiv. Refl.: (0,-3,7), (3,0,7), (3,-3,-7), (-3,0,-7), (-3,3,7)								
**LiNbO3**	**(1,6,-5)**	**9.388**	**76.4**	**4.4**	**6.4**	**2.337**	**14.89**	**29.21**	**32.78**
	Equiv. Refl.: (1,-7,5), (6,1,5), (6,-7,-5), (7,-1,5), (7,-6,-5), (-1,7,-5), (-1,-6,5), (-6,7,5), (-6,-1,-5)<br>(-7,1,-5), (-7,6,5)								
**Quartz**	**(2,-1,8)**	**9.517**	**80.16**	**4.2**	**4.5**	**1.676**	**7.62**	**20.95**	**22.29**
**Quartz**	**(2,-1,-8)**	**9.517**	**80.16**	**4.1**	**4.5**	**1.676**	**7.62**	**20.95**	**22.29**
	Equiv. Refl.: (-1,2,8), (-1,2,-8), (-1,-1,8), (-1,-1,-8)								
**Quartz**	**(0,6,-3)**	**9.395**	**76.57**	**3.8**	**3.5**	**2.307**	**8.06**	**28.84**	**29.94**
	Equiv. Refl.: (0,-6,3), (3,4,3), (3,-7,-3), (4,3,-3), (4,-7,3), (6,0,3), (6,-6,-3), (-6,0,-3), (-6,6,3)<br>(-7,3,3), (-7,4,-3)								
**Quartz**	**(2,5,-1)**	**9.171**	**71.71**	**3.5**	**3.1**	**3.193**	**9.88**	**39.91**	**41.12**
	Equiv. Refl.: (2,-7,1), (5,2,1), (5,-7,-1), (-7,2,-1), (-7,5,1)								
**Quartz**	**(7,-2,1)**	**9.171**	**71.71**	**3.4**	**3.1**	**3.193**	**9.88**	**39.91**	**41.12**
	Equiv. Refl.: (7,-5,-1), (-2,7,-1), (-2,-5,1), (-5,7,1), (-5,-2,-1)								
**LiNbO3**	**(1,6,1)**	**9.129**	**70.93**	**3.3**	**4.9**	**3.339**	**16.34**	**41.73**	**44.82**
	Equiv. Refl.: (1,-7,-1), (6,1,-1), (6,-7,1), (7,-1,-1), (7,-6,1), (-1,7,1), (-1,-6,-1), (-6,7,-1), (-6,-1,1)<br>(-7,1,1), (-7,6,-1), (3,4,-4), (3,-7,4), (4,3,4), (4,-7,-4), (7,-3,4), (7,-4,-4), (-3,7,-4)<br>(-3,-4,4), (-4,7,4), (-4,-3,-4), (-7,3,-4), (-7,4,4)								
**Quartz**	**(7,-3,-3)**	**9.507**	**79.82**	**3.2**	**3.6**	**1.734**	**6.18**	**21.67**	**22.53**
	Equiv. Refl.: (7,-4,3), (-3,7,3), (-3,-4,-3), (-4,7,-3), (-4,-3,3)								

Ei =	9.659	keV						ΔEg	ΔEt
Cryst	Refl	EB	ΘB	∫ IR dΘ	Width	Ei cotΘ	ΔE	2m, 50mm	2m, 50mm
	(h,k,l)	[keV]	[°]	[μrad]	[μrad]	[meV/mrad]	[meV]	[meV]	[meV]
**LiNbO3**	**(2,4,13)**	**9.377**	**76.13**	**3.1**	**5.3**	**2.386**	**12.76**	**29.82**	**32.44**
	Equiv. Refl.: (2,-6,-13), (4,2,-13), (4,-6,13), (6,-2,-13), (6,-4,13), (-2,6,13), (-2,-4,-13), (-4,6,-13), (-4,-2,13) (-6,2,13), (-6,4,-13)								
**Quartz**	**(4,-2,7)**	**9.484**	**79.07**	**2.9**	**3.1**	**1.866**	**5.79**	**23.32**	**24.03**
	Equiv. Refl.: (4,-2,-7), (-2,4,7), (-2,4,-7), (-2,-2,7), (-2,-2,-7)								
**Quartz**	**(0,1,8)**	**9.291**	**74.14**	**2.7**	**2.7**	**2.745**	**7.47**	**34.31**	**35.12**
	Equiv. Refl.: (0,-1,-8), (1,0,-8), (1,-1,8), (-1,0,8), (-1,1,-8)								
**Quartz**	**(2,2,7)**	**9.484**	**79.07**	**2.6**	**3.1**	**1.866**	**5.79**	**23.32**	**24.03**
	Equiv. Refl.: (2,2,-7), (2,-4,7), (2,-4,-7), (-4,2,7), (-4,2,-7)								
**LiNbO3**	**(1,3,-17)**	**9.106**	**70.52**	**1.5**	**3**	**3.416**	**10.19**	**42.7**	**43.9**
	Equiv. Refl.: (1,-4,17), (3,1,17), (3,-4,-17), (4,-1,17), (4,-3,-17), (-1,4,-17), (-1,-3,17), (-3,4,17), (-3,-1,-17) (-4,1,-17), (-4,3,17), (7,-3,2), (7,-4,-2), (-3,7,-2), (-3,-4,2), (-4,7,2), (-4,-3,-2)								
**Quartz**	**(0,5,5)**	**9.272**	**73.72**	**1.4**	**1.8**	**2.821**	**5.21**	**35.27**	**35.65**
	Equiv. Refl.: (0,-5,-5), (3,4,-2), (3,-7,2), (4,3,2), (4,-7,-2), (5,0,-5), (5,-2,6), (5,-3,-6), (5,-5,5) (-2,5,-6), (-2,-3,6), (-3,5,6), (-3,-2,-6), (-5,0,5), (-5,5,-5), (-7,3,-2), (-7,4,2)								
**LiNbO3**	**(1,4,15)**	**9.252**	**73.3**	**1.3**	**3**	**2.897**	**8.6**	**36.22**	**37.22**
	Equiv. Refl.: (1,4,-15), (1,-5,15), (1,-5,-15), (4,1,15), (4,1,-15), (4,-5,15), (4,-5,-15), (5,-1,15), (5,-1,-15) (5,-4,15), (5,-4,-15), (-1,5,15), (-1,5,-15), (-1,-4,15), (-1,-4,-15), (-4,5,15), (-4,5,-15), (-4,-1,15) (-4,-1,-15), (-5,1,15), (-5,1,-15), (-5,4,15), (-5,4,-15), (0,6,6), (0,6,-6), (0,-6,6), (0,-6,-6) (6,0,6), (6,0,-6), (6,-6,6), (6,-6,-6), (-6,0,6), (-6,0,-6), (-6,6,6), (-6,6,-6), (2,3,-6) (2,-5,6), (3,2,6), (3,-5,-6), (-5,2,-6), (-5,3,6)								
**LiNbO3**	**(2,5,9)**	**9.571**	**82.26**	**1.2**	**3.8**	**1.314**	**4.97**	**16.42**	**17.16**
	Equiv. Refl.: (2,5,-9), (2,-7,9), (2,-7,-9), (5,2,9), (5,2,-9), (5,-7,9), (5,-7,-9), (7,-2,9), (7,-2,-9) (7,-5,9), (7,-5,-9), (-2,7,9), (-2,7,-9), (-2,-5,9), (-2,-5,-9), (-5,7,9), (-5,7,-9), (-5,-2,9) (-5,-2,-9), (-7,2,9), (-7,2,-9), (-7,5,9), (-7,5,-9)								
**Quartz**	**(5,-1,-6)**	**9.589**	**83.08**	**1.1**	**1.7**	**1.172**	**1.95**	**14.65**	**14.78**
	Equiv. Refl.: (5,-4,6), (-1,5,6), (-1,-4,-6), (-4,5,-6), (-4,-1,6)								
**LiNbO3**	**(1,2,-19)**	**9.259**	**73.45**	**1**	**2.5**	**2.871**	**7.16**	**35.89**	**36.6**
	Equiv. Refl.: (1,5,11), (1,-3,19), (1,-6,-11), (2,1,19), (2,-3,-19), (3,-1,19), (3,-2,-19), (5,1,-11), (5,-6,11) (6,-1,-11), (6,-5,11), (-1,3,-19), (-1,6,11), (-1,-2,19), (-1,-5,-11), (-2,3,19), (-2,-1,-19), (-3,1,-19) (-3,2,19), (-5,6,-11), (-5,-1,11), (-6,1,11), (-6,5,-11), (1,5,8), (1,-6,-8), (5,1,-8), (5,-6,8) (6,-1,-8), (6,-5,8), (-1,6,8), (-1,-5,-8), (-5,6,-8), (-5,-1,8), (-6,1,8), (-6,5,-8)								
**Quartz**	**(1,4,6)**	**9.589**	**83.08**	**0.7**	**1.7**	**1.172**	**1.96**	**14.65**	**14.78**
	Equiv. Refl.: (1,-5,-6), (4,1,-6), (4,-5,6), (-5,1,6), (-5,4,-6)								
**Quartz**	**(2,3,6)**	**9.365**	**75.82**	**0.6**	**0.9**	**2.441**	**2.21**	**30.51**	**30.59**
	Equiv. Refl.: (2,-5,-6), (3,2,-6), (3,-5,6), (-5,2,6), (-5,3,-6)								
**Sapph**	**(3,4,5)**	**9.457**	**78.26**	**0.4**	**1**	**2.007**	**1.95**	**25.08**	**25.16**
	Equiv. Refl.: (3,-7,-5), (4,3,-5), (4,-7,5), (7,-3,-5), (7,-4,5), (-3,7,5), (-3,-4,-5), (-4,7,-5), (-4,-3,5) (-7,3,5), (-7,4,-5), (5,-2,-6), (5,-3,6), (-2,5,6), (-2,-3,-6), (-3,5,-6), (-3,-2,6)								
**Sapph**	**(3,4,-1)**	**9.163**	**71.57**	**0.3**	**0.7**	**3.219**	**2.18**	**40.24**	**40.3**
	Equiv. Refl.: (3,-7,1), (4,3,1), (4,-7,-1), (7,-3,1), (7,-4,-1), (-3,7,-1), (-3,-4,1), (-4,7,1), (-4,-3,-1) (-7,3,-1), (-7,4,1), (1,5,-4), (1,-6,4), (5,1,4), (5,-6,-4), (6,-1,4), (6,-5,-4), (7,-2,0) (7,-5,0), (-1,6,-4), (-1,-5,4), (-2,7,0), (-2,-5,0), (-5,6,4), (-5,7,0), (-5,-1,-4), (-5,-2,0) (-6,1,-4), (-6,5,4)								
**Quartz**	**(2,5,0)**	**9.099**	**70.39**	**0.2**	**0.6**	**3.441**	**1.95**	**43.01**	**43.06**
	Equiv. Refl.: (2,-7,0), (5,2,0), (5,-7,0), (-7,2,0), (-7,5,0)								

Ei =	8.639	keV						ΔEg	ΔEt
Cryst	Refl	EB	ΘB	∫ IR dΘ	Width	Ei cotΘ	ΔE	2m, 50µm	2m, 50µm
	(h,k,l)	[keV]	[°]	[µrad]	[µrad]	[meV/µrad]	[meV]	[meV]	[meV]
**Si**	**(2,4,6)**	**8.542**	**81.4**	**27.7966**	**24.69**	**1.3071**	**32.28**	**16.34**	**36.18**
	Equiv. Refl.: (2,6,4), (4,2,6), (4,6,2), (6,2,4), (6,4,2)								
**Ge**	**(2,4,6)**	**8.2**	**71.66**	**26.6719**	**24.54**	**2.8642**	**70.29**	**35.8**	**78.88**
	Equiv. Refl.: (2,6,4), (4,2,6), (4,6,2), (6,2,4), (6,4,2)								
**Ge**	**(1,3,7)**	**8.417**	**76.98**	**22.9302**	**22.61**	**1.9979**	**45.17**	**24.97**	**51.61**
	Equiv. Refl.: (1,7,3), (3,1,7), (3,5,5), (3,7,1), (5,3,5), (5,5,3), (7,1,3), (7,3,1)								
**Si**	**(1,1,7)**	**8.152**	**70.66**	**9.7109**	**8.87**	**3.032**	**26.88**	**37.9**	**46.46**
	Equiv. Refl.: (1,5,5), (1,7,1), (5,1,5), (5,5,1), (7,1,1)								
**LiNbO3**	**(2,-6,-10)**	**8.61**	**85.31**	**84.358**	**78.4**	**0.709**	**55.55**	**8.86**	**56.25**
	Equiv. Refl.: (4,2,-10), (6,-2,-10), (-2,-4,-10), (-4,6,-10), (-6,4,-10)								
**LiNbO3**	**(4,-6,10)**	**8.61**	**85.31**	**78.053**	**78.4**	**0.709**	**55.55**	**8.86**	**56.25**
	Equiv. Refl.: (6,-4,10)								
**LiNbO3**	**(2,4,10)**	**8.61**	**85.31**	**78.052**	**78.4**	**0.709**	**55.55**	**8.86**	**56.25**
	Equiv. Refl.: (-2,6,10), (-4,-2,10), (-6,2,10)								
**Sapph**	**(0,0,18)**	**8.591**	**83.97**	**53.728**	**45.7**	**0.912**	**41.67**	**11.4**	**43.2**
	Equiv. Refl.: (0,0,-18)								
**Quartz**	**(-5,0,4)**	**8.609**	**85.25**	**45.893**	**39.2**	**0.718**	**28.11**	**8.97**	**29.51**
**Quartz**	**(5,0,-4)**	**8.609**	**85.25**	**45.892**	**39.2**	**0.718**	**28.11**	**8.97**	**29.51**
	Equiv. Refl.: (5,-5,4), (-5,5,-4)								
**Quartz**	**(0,5,4)**	**8.609**	**85.25**	**45.888**	**39.2**	**0.718**	**28.11**	**8.97**	**29.51**
	Equiv. Refl.: (0,-5,-4)								
**LiNbO3**	**(3,-7,-2)**	**8.505**	**79.9**	**41.196**	**38.5**	**1.539**	**59.18**	**19.23**	**62.23**
	Equiv. Refl.: (4,3,-2), (7,-3,-2), (-3,-4,-2), (-4,7,-2), (-7,4,-2)								
**Sapph**	**(1,5,-4)**	**8.591**	**83.96**	**40.054**	**34.9**	**0.914**	**31.87**	**11.42**	**33.85**
	Equiv. Refl.: (1,-6,4), (5,1,4), (5,-6,-4), (6,-1,4), (6,-5,-4), (-1,6,-4), (-1,-5,4), (-5,6,4), (-5,-1,-4) (-6,1,-4), (-6,5,4)								
**LiNbO3**	**(3,4,2)**	**8.505**	**79.9**	**39.046**	**38.5**	**1.539**	**59.18**	**19.23**	**62.23**
	Equiv. Refl.: (4,-7,2), (7,-4,2), (-3,7,2), (-4,-3,2), (-7,3,2)								
**LiNbO3**	**(1,5,8)**	**8.528**	**80.83**	**37.225**	**36.6**	**1.395**	**51.09**	**17.44**	**53.98**
	Equiv. Refl.: (5,-6,8), (6,-5,8), (-1,6,8), (-5,-1,8), (-6,1,8)								
**LiNbO3**	**(1,-6,-8)**	**8.528**	**80.83**	**37.107**	**36.6**	**1.395**	**51.09**	**17.44**	**53.98**
	Equiv. Refl.: (5,1,-8), (6,-1,-8), (-1,-5,-8), (-5,6,-8), (-6,5,-8)								
**Sapph**	**(1,2,-16)**	**8.612**	**85.44**	**33.676**	**31.2**	**0.688**	**21.44**	**8.6**	**23.1**
	Equiv. Refl.: (1,-3,16), (2,1,16), (2,-3,-16), (3,-1,16), (3,-2,-16), (-1,3,-16), (-1,-2,16), (-2,3,16), (-2,-1,-16) (-3,1,-16), (-3,2,16)								
**Quartz**	**(-5,2,-5)**	**8.557**	**82.11**	**32.99**	**27.6**	**1.198**	**33.04**	**14.97**	**36.27**
	Equiv. Refl.: (-5,3,5)								
**Quartz**	**(3,-5,-5)**	**8.557**	**82.11**	**32.986**	**27.6**	**1.198**	**33.03**	**14.97**	**36.27**
**Quartz**	**(2,3,-5)**	**8.557**	**82.11**	**32.985**	**27.6**	**1.198**	**33.03**	**14.97**	**36.27**
	Equiv. Refl.: (2,-5,5), (3,2,5)								
**Quartz**	**(5,-2,5)**	**8.557**	**82.11**	**32.629**	**27.6**	**1.198**	**33.04**	**14.97**	**36.27**
	Equiv. Refl.: (5,-3,-5)								
**Quartz**	**(-2,5,-5)**	**8.557**	**82.11**	**32.625**	**27.6**	**1.198**	**33.03**	**14.97**	**36.27**
	Equiv. Refl.: (-2,-3,5), (-3,5,5)								
**Quartz**	**(-3,-2,-5)**	**8.557**	**82.11**	**32.623**	**27.6**	**1.198**	**33.03**	**14.97**	**36.27**
**LiNbO3**	**(0,6,0)**	**8.343**	**74.96**	**30.577**	**28.6**	**2.322**	**66.43**	**29.02**	**72.5**
	Equiv. Refl.: (6,-6,0), (-6,0,0)								

**Ei =**	**8.639**	**keV**						**ΔEg**	**ΔEt**
**Cryst**	**Refl**	**EB**	**ΘB**	**∫ IR dΘ**	**Width**	**Ei cotΘ**	**ΔE**	**2m, 50µm**	**2m, 50µm**
	**(h,k,l)**	**[keV]**	**[°]**	**[µrad]**	**[µrad]**	**[meV/µrad]**	**[meV]**	**[meV]**	**[meV]**
**Sapph**	**(1,3,-14)**	**8.607**	**85.03**	**30.087**	**27.9**	**0.751**	**20.93**	**9.38**	**22.93**
	Equiv. Refl.: (1,-4,14), (3,1,14), (3,-4,-14), (4,-1,14), (4,-3,-14), (-1,4,-14), (-1,-3,14), (-3,4,14), (-3,-1,-14) (-4,1,-14), (-4,3,14)								
**LiNbO3**	**(0,-6,0)**	**8.343**	**74.96**	**29.919**	**28.6**	**2.322**	**66.43**	**29.02**	**72.5**
	Equiv. Refl.: (6,0,0)								
**LiNbO3**	**(-6,6,0)**	**8.343**	**74.96**	**29.918**	**28.6**	**2.322**	**66.43**	**29.02**	**72.5**
**Sapph**	**(2,4,7)**	**8.633**	**87.92**	**28.688**	**32.2**	**0.313**	**10.07**	**3.91**	**10.8**
	Equiv. Refl.: (2,-6,-7), (4,2,-7), (4,-6,7), (6,-2,-7), (6,-4,7), (-2,6,7), (-2,-4,-7), (-4,6,-7), (-4,-2,7) (-6,2,7), (-6,4,-7)								
**LiNbO3**	**(1,1,-18)**	**8.402**	**76.54**	**27.088**	**25.7**	**2.067**	**53.07**	**25.84**	**59.03**
	Equiv. Refl.: (1,-2,-18), (2,-1,-18), (-1,2,-18), (-1,-1,-18), (-2,1,-18)								
**LiNbO3**	**(0,-4,-14)**	**8.374**	**75.78**	**26.375**	**24.9**	**2.189**	**54.55**	**27.37**	**61.02**
	Equiv. Refl.: (4,0,-14), (-4,4,-14)								
**LiNbO3**	**(1,-5,12)**	**8.331**	**74.64**	**25.127**	**23.6**	**2.373**	**56.05**	**29.66**	**63.42**
	Equiv. Refl.: (4,1,12), (5,-1,12), (-1,-4,12), (-4,5,12), (-5,4,12)								
**LiNbO3**	**(1,1,18)**	**8.402**	**76.54**	**25.09**	**25.7**	**2.067**	**53.07**	**25.84**	**59.03**
	Equiv. Refl.: (1,-2,18), (2,-1,18), (-1,2,18), (-1,-1,18), (-2,1,18)								
**LiNbO3**	**(0,4,14)**	**8.374**	**75.78**	**24.474**	**24.9**	**2.189**	**54.55**	**27.37**	**61.02**
	Equiv. Refl.: (4,-4,14), (-4,0,14)								
**LiNbO3**	**(1,4,12)**	**8.331**	**74.64**	**24.307**	**23**	**2.373**	**54.47**	**29.66**	**62.02**
	Equiv. Refl.: (4,-5,12), (5,-4,12), (-1,5,12), (-4,-1,12), (-5,1,12)								
**LiNbO3**	**(1,4,-12)**	**8.331**	**74.64**	**23.267**	**23.6**	**2.373**	**56.05**	**29.66**	**63.42**
	Equiv. Refl.: (4,-5,-12), (5,-4,-12), (-1,5,-12), (-4,-1,-12), (-5,1,-12)								
**LiNbO3**	**(-4,5,-12)**	**8.331**	**74.64**	**22.46**	**23**	**2.373**	**54.47**	**29.66**	**62.02**
	Equiv. Refl.: (-5,4,-12)								
**LiNbO3**	**(1,-5,-12)**	**8.331**	**74.64**	**22.459**	**23**	**2.373**	**54.47**	**29.66**	**62.02**
	Equiv. Refl.: (4,1,-12), (5,-1,-12), (-1,-4,-12)								
**LiNbO3**	**(0,5,10)**	**8.266**	**73.11**	**22.354**	**20.5**	**2.623**	**53.87**	**32.79**	**63.06**
	Equiv. Refl.: (5,-5,10), (-5,0,10)								
**LiNbO3**	**(0,-5,-10)**	**8.266**	**73.11**	**19.228**	**20.5**	**2.623**	**53.87**	**32.79**	**63.06**
	Equiv. Refl.: (5,0,-10), (-5,5,-10)								
**LiNbO3**	**(2,-6,8)**	**8.181**	**71.27**	**19.093**	**18.2**	**2.93**	**53.44**	**36.62**	**64.78**
	Equiv. Refl.: (4,2,8), (6,-2,8), (-2,-4,8), (-4,6,8), (-6,4,8)								
**LiNbO3**	**(2,4,-8)**	**8.181**	**71.27**	**17.64**	**18.2**	**2.93**	**53.44**	**36.62**	**64.78**
	Equiv. Refl.: (4,-6,-8), (6,-4,-8), (-2,6,-8), (-4,-2,-8), (-6,2,-8)								
**Quartz**	**(2,2,6)**	**8.534**	**81.08**	**15.354**	**13.4**	**1.356**	**18.18**	**16.95**	**24.86**
	Equiv. Refl.: (2,-4,-6)								
**Quartz**	**(2,2,-6)**	**8.534**	**81.08**	**15.352**	**13.4**	**1.356**	**18.17**	**16.95**	**24.85**
	Equiv. Refl.: (2,-4,6)								
**Quartz**	**(-4,2,-6)**	**8.534**	**81.08**	**15.348**	**13.4**	**1.356**	**18.17**	**16.95**	**24.85**
**Quartz**	**(-4,2,6)**	**8.534**	**81.08**	**15.346**	**13.4**	**1.356**	**18.17**	**16.95**	**24.85**
**Quartz**	**(-2,-2,-6)**	**8.534**	**81.08**	**15.119**	**13.4**	**1.356**	**18.18**	**16.95**	**24.86**
**Quartz**	**(-2,4,6)**	**8.534**	**81.08**	**15.118**	**13.4**	**1.356**	**18.18**	**16.95**	**24.86**
**Quartz**	**(-2,4,-6)**	**8.534**	**81.08**	**15.117**	**13.4**	**1.356**	**18.18**	**16.95**	**24.85**
	Equiv. Refl.: (-2,-2,6)								
**Quartz**	**(4,-2,6)**	**8.534**	**81.08**	**15.109**	**13.4**	**1.356**	**18.17**	**16.95**	**24.85**
**Quartz**	**(4,-2,-6)**	**8.534**	**81.08**	**15.107**	**13.4**	**1.356**	**18.17**	**16.95**	**24.85**
**Quartz**	**(-3,5,-5)**	**8.557**	**82.11**	**12.075**	**11.4**	**1.198**	**13.66**	**14.97**	**20.27**
**Quartz**	**(-3,-2,5)**	**8.557**	**82.11**	**12.073**	**11.4**	**1.198**	**13.66**	**14.97**	**20.27**
**Quartz**	**(-2,5,5)**	**8.557**	**82.11**	**12.068**	**11.4**	**1.198**	**13.66**	**14.97**	**20.27**
**Quartz**	**(-2,-3,-5)**	**8.557**	**82.11**	**12.067**	**11.4**	**1.198**	**13.66**	**14.97**	**20.27**

Ei =	8.639	keV						ΔEg	ΔEt
**Cryst**	**Refl**	**EB**	**ΘB**	**∫ IR dΘ**	**Width**	**Ei cotΘ**	**ΔE**	**2m, 50µm**	**2m, 50µm**
	**(h,k,l)**	**[keV]**	**[°]**	**[µrad]**	**[µrad]**	**[meV/µrad]**	**[meV]**	**[meV]**	**[meV]**
**Quartz**	**(5,-2,-5)**	**8.557**	**82.11**	**12.06**	**11.4**	**1.198**	**13.65**	**14.97**	**20.26**
	Equiv. Refl.: (5,-3,5)								
**Sapph**	**(2,4,4)**	**8.186**	**71.37**	**12.004**	**10.4**	**2.912**	**30.22**	**36.4**	**47.31**
	Equiv. Refl.: (2,-6,-4), (4,2,-4), (4,-6,4), (6,-2,-4), (6,-4,4), (-2,6,4), (-2,-4,-4), (-4,6,-4), (-4,-2,4) (-6,2,4), (-6,4,-4)								
**Quartz**	**(3,-5,5)**	**8.557**	**82.11**	**11.831**	**11.4**	**1.198**	**13.66**	**14.97**	**20.27**
**Quartz**	**(3,2,-5)**	**8.557**	**82.11**	**11.83**	**11.4**	**1.198**	**13.66**	**14.97**	**20.27**
**Quartz**	**(2,-5,-5)**	**8.557**	**82.11**	**11.825**	**11.4**	**1.198**	**13.66**	**14.97**	**20.27**
**Quartz**	**(2,3,5)**	**8.557**	**82.11**	**11.823**	**11.4**	**1.198**	**13.66**	**14.97**	**20.27**
**Quartz**	**(-5,2,5)**	**8.557**	**82.11**	**11.816**	**11.4**	**1.198**	**13.65**	**14.97**	**20.26**
	Equiv. Refl.: (-5,3,-5)								
**Sapph**	**(0,2,16)**	**8.208**	**71.83**	**11.285**	**9.9**	**2.836**	**28.16**	**35.45**	**45.27**
	Equiv. Refl.: (0,-2,-16), (2,0,-16), (2,-2,16), (-2,0,16), (-2,2,-16)								
**Quartz**	**(1,-6,1)**	**8.193**	**71.51**	**11.227**	**9.5**	**2.89**	**27.38**	**36.12**	**45.33**
**Quartz**	**(1,5,-1)**	**8.193**	**71.51**	**11.226**	**9.5**	**2.89**	**27.38**	**36.12**	**45.33**
	Equiv. Refl.: (-6,1,-1), (-6,5,1)								
**Quartz**	**(5,1,1)**	**8.193**	**71.51**	**11.225**	**9.5**	**2.89**	**27.38**	**36.12**	**45.32**
	Equiv. Refl.: (5,-6,-1)								
**Quartz**	**(-1,6,-1)**	**8.193**	**71.51**	**11.176**	**9.5**	**2.89**	**27.38**	**36.12**	**45.33**
**Quartz**	**(6,-1,1)**	**8.193**	**71.51**	**11.175**	**9.5**	**2.89**	**27.38**	**36.12**	**45.33**
	Equiv. Refl.: (6,-5,-1), (-1,-5,1)								
**Quartz**	**(-5,6,1)**	**8.193**	**71.51**	**11.174**	**9.5**	**2.89**	**27.38**	**36.12**	**45.32**
	Equiv. Refl.: (-5,-1,-1)								
**Quartz**	**(-2,1,-7)**	**8.416**	**76.96**	**10.46**	**9.2**	**2**	**18.33**	**25**	**31**
**Quartz**	**(-2,1,7)**	**8.416**	**76.96**	**10.459**	**9.2**	**2**	**18.33**	**25**	**31**
**Quartz**	**(1,-2,-7)**	**8.416**	**76.96**	**10.458**	**9.2**	**2**	**18.33**	**25**	**31**
**Quartz**	**(1,1,7)**	**8.416**	**76.96**	**10.456**	**9.2**	**2**	**18.32**	**25**	**31**
**Quartz**	**(1,-2,7)**	**8.416**	**76.96**	**10.454**	**9.2**	**2**	**18.32**	**25**	**31**
**Quartz**	**(1,1,-7)**	**8.416**	**76.96**	**10.453**	**9.2**	**2**	**18.32**	**25**	**30.99**
**Quartz**	**(2,-1,7)**	**8.416**	**76.96**	**10.037**	**9.2**	**2**	**18.33**	**25**	**31**
**Quartz**	**(2,-1,-7)**	**8.416**	**76.96**	**10.036**	**9.2**	**2**	**18.33**	**25**	**31**
**Quartz**	**(-1,2,7)**	**8.416**	**76.96**	**10.033**	**9.2**	**2**	**18.33**	**25**	**31**
**Quartz**	**(-1,-1,-7)**	**8.416**	**76.96**	**10.031**	**9.2**	**2**	**18.32**	**25**	**31**
**Quartz**	**(-1,2,-7)**	**8.416**	**76.96**	**10.03**	**9.2**	**2**	**18.32**	**25**	**31**
**Quartz**	**(-1,-1,7)**	**8.416**	**76.96**	**10.029**	**9.2**	**2**	**18.32**	**25**	**30.99**
**Quartz**	**(-2,2,7)**	**8.542**	**81.39**	**9.537**	**9.3**	**1.309**	**12.23**	**16.36**	**20.42**
**Quartz**	**(2,-2,-7)**	**8.542**	**81.39**	**9.536**	**9.3**	**1.309**	**12.23**	**16.36**	**20.42**
**Quartz**	**(-2,0,-7)**	**8.542**	**81.39**	**9.534**	**9.3**	**1.309**	**12.23**	**16.36**	**20.42**
**Quartz**	**(2,0,7)**	**8.542**	**81.39**	**9.533**	**9.3**	**1.309**	**12.23**	**16.36**	**20.42**
**Quartz**	**(0,2,-7)**	**8.542**	**81.39**	**9.518**	**9.3**	**1.309**	**12.21**	**16.36**	**20.41**
	Equiv. Refl.: (0,-2,7)								
**Quartz**	**(-4,-2,-3)**	**8.443**	**77.76**	**8.887**	**7.7**	**1.874**	**14.46**	**23.42**	**27.52**
**Quartz**	**(-4,6,3)**	**8.443**	**77.76**	**8.885**	**7.7**	**1.874**	**14.46**	**23.42**	**27.52**
**Quartz**	**(6,-4,-3)**	**8.443**	**77.76**	**8.882**	**7.7**	**1.874**	**14.45**	**23.42**	**27.52**
**Quartz**	**(6,-2,3)**	**8.443**	**77.76**	**8.881**	**7.7**	**1.874**	**14.45**	**23.42**	**27.52**
**Quartz**	**(-2,6,-3)**	**8.443**	**77.76**	**8.88**	**7.7**	**1.874**	**14.45**	**23.42**	**27.52**
**Quartz**	**(-2,-4,3)**	**8.443**	**77.76**	**8.879**	**7.7**	**1.874**	**14.45**	**23.42**	**27.52**
**Quartz**	**(2,4,3)**	**8.443**	**77.76**	**8.486**	**7.7**	**1.874**	**14.36**	**23.42**	**27.47**
	Equiv. Refl.: (2,-6,-3)								
**Quartz**	**(-6,4,-3)**	**8.443**	**77.76**	**8.484**	**7.7**	**1.874**	**14.36**	**23.42**	**27.47**

Ei = Cryst	8.639 Refl (h,k,l)	keV EB [keV]	ΘB [°]	∫ IR dΘ [μrad]	Width [μrad]	Ei cotΘ [meV/μrad]	ΔE [meV]	ΔEg 2m, 50μm [meV]	ΔEt 2m, 50μm [meV]
**Quartz**	**(-6,2,3)**	**8.443**	**77.76**	**8.482**	**7.7**	**1.874**	**14.35**	**23.42**	**27.47**
**Quartz**	**(4,2,-3)**	**8.443**	**77.76**	**8.481**	**7.7**	**1.874**	**14.35**	**23.42**	**27.47**
	Equiv. Refl.: (4,-6,3)								
**Quartz**	**(2,0,-7)**	**8.542**	**81.39**	**8.328**	**8.4**	**1.309**	**10.99**	**16.36**	**19.7**
	Equiv. Refl.: (-2,0,7)								
**Quartz**	**(2,-2,7)**	**8.542**	**81.39**	**8.326**	**8.4**	**1.309**	**10.98**	**16.36**	**19.7**
	Equiv. Refl.: (-2,2,-7)								
**Quartz**	**(0,2,7)**	**8.542**	**81.39**	**8.3**	**8.4**	**1.309**	**10.96**	**16.36**	**19.69**
	Equiv. Refl.: (0,-2,-7)								
**Quartz**	**(1,0,-7)**	**8.16**	**70.84**	**7.897**	**6.9**	**3.002**	**20.81**	**37.53**	**42.91**
	Equiv. Refl.: (-1,0,7)								
**Quartz**	**(-1,1,-7)**	**8.16**	**70.84**	**7.896**	**6.9**	**3.002**	**20.81**	**37.53**	**42.91**
**Quartz**	**(0,1,7)**	**8.16**	**70.84**	**7.895**	**6.9**	**3.002**	**20.81**	**37.53**	**42.91**
	Equiv. Refl.: (0,-1,-7), (1,-1,7)								
**Quartz**	**(-2,6,3)**	**8.443**	**77.76**	**7.894**	**7.7**	**1.874**	**14.36**	**23.42**	**27.47**
**Quartz**	**(-2,-4,-3)**	**8.443**	**77.76**	**7.893**	**7.7**	**1.874**	**14.36**	**23.42**	**27.47**
**Quartz**	**(6,-4,3)**	**8.443**	**77.76**	**7.891**	**7.7**	**1.874**	**14.36**	**23.42**	**27.47**
**Quartz**	**(6,-2,-3)**	**8.443**	**77.76**	**7.889**	**7.7**	**1.874**	**14.35**	**23.42**	**27.47**
**Quartz**	**(-4,6,-3)**	**8.443**	**77.76**	**7.888**	**7.7**	**1.874**	**14.35**	**23.42**	**27.47**
	Equiv. Refl.: (-4,-2,3)								
**Sapph**	**(1,4,9)**	**8.123**	**70.09**	**7.607**	**7.1**	**3.128**	**22.26**	**39.1**	**44.99**
	Equiv. Refl.: (1,4,-9), (1,-5,9), (1,-5,-9), (4,1,9), (4,1,-9), (4,-5,9), (4,-5,-9), (5,-1,9), (5,-1,-9) (5,-4,9), (5,-4,-9), (-1,5,9), (-1,5,-9), (-1,-4,9), (-1,-4,-9), (-4,5,9), (-4,5,-9), (-4,-1,9) (-4,-1,-9), (-5,1,9), (-5,1,-9), (-5,4,9), (-5,4,-9)								
**Quartz**	**(3,-6,-3)**	**8.316**	**74.28**	**7.507**	**6.8**	**2.431**	**16.53**	**30.39**	**34.59**
	Equiv. Refl.: (-6,3,3)								
**Quartz**	**(3,3,3)**	**8.316**	**74.28**	**7.506**	**6.8**	**2.431**	**16.53**	**30.39**	**34.59**
	Equiv. Refl.: (-6,3,-3)								
**Quartz**	**(3,3,-3)**	**8.316**	**74.28**	**7.505**	**6.8**	**2.431**	**16.53**	**30.39**	**34.59**
	Equiv. Refl.: (3,-6,3)								
**Quartz**	**(4,2,3)**	**8.443**	**77.76**	**7.49**	**7.7**	**1.874**	**14.46**	**23.42**	**27.52**
**Quartz**	**(4,-6,-3)**	**8.443**	**77.76**	**7.488**	**7.7**	**1.874**	**14.46**	**23.42**	**27.52**
**Quartz**	**(-6,4,3)**	**8.443**	**77.76**	**7.486**	**7.7**	**1.874**	**14.45**	**23.42**	**27.52**
**Quartz**	**(-6,2,-3)**	**8.443**	**77.76**	**7.485**	**7.7**	**1.874**	**14.45**	**23.42**	**27.52**
**Quartz**	**(2,-6,3)**	**8.443**	**77.76**	**7.483**	**7.7**	**1.874**	**14.45**	**23.42**	**27.52**
**Quartz**	**(2,4,-3)**	**8.443**	**77.76**	**7.482**	**7.7**	**1.874**	**14.45**	**23.42**	**27.52**
**Quartz**	**(-3,6,3)**	**8.316**	**74.28**	**7.464**	**6.8**	**2.431**	**16.53**	**30.39**	**34.59**
**Quartz**	**(6,-3,3)**	**8.316**	**74.28**	**7.463**	**6.8**	**2.431**	**16.53**	**30.39**	**34.59**
	Equiv. Refl.: (6,-3,-3), (-3,6,-3), (-3,-3,3), (-3,-3,-3)								
**Quartz**	**(-5,6,-2)**	**8.43**	**77.38**	**7.42**	**6.8**	**1.934**	**13.2**	**24.18**	**27.55**
	Equiv. Refl.: (-5,-1,2)								
**Quartz**	**(-1,6,2)**	**8.43**	**77.38**	**7.418**	**6.8**	**1.934**	**13.2**	**24.18**	**27.55**
	Equiv. Refl.: (-1,-5,-2)								
**Quartz**	**(6,-1,-2)**	**8.43**	**77.38**	**7.417**	**6.8**	**1.934**	**13.2**	**24.18**	**27.55**
	Equiv. Refl.: (6,-5,2)								
**Quartz**	**(5,1,-2)**	**8.43**	**77.38**	**7.276**	**6.8**	**1.934**	**13.2**	**24.18**	**27.55**
	Equiv. Refl.: (5,-6,2)								
**Quartz**	**(1,5,2)**	**8.43**	**77.38**	**7.275**	**6.8**	**1.934**	**13.2**	**24.18**	**27.55**
**Quartz**	**(1,-6,-2)**	**8.43**	**77.38**	**7.274**	**6.8**	**1.934**	**13.2**	**24.18**	**27.55**
	Equiv. Refl.: (-6,1,2), (-6,5,-2)								

Ei =	8.639	keV						ΔEg	ΔEt
Cryst	Refl	EB	ΘB	∫ IR dΘ	Width	Ei cotΘ	ΔE	2m, 50µm	2m, 50µm
	(h,k,l)	[keV]	[°]	[µrad]	[µrad]	[meV/µrad]	[meV]	[meV]	[meV]
**Sapph**	**(1,5,-1)**	**8.39**	**76.21**	**7.212**	**7**	**2.121**	**14.93**	**26.51**	**30.43**
	Equiv. Refl.: (1,-6,1), (5,1,1), (5,-6,-1), (6,-1,1), (6,-5,-1), (-1,6,-1), (-1,-5,1), (-5,6,1), (-5,-1,-1) (-6,1,-1), (-6,5,1)								
**Quartz**	**(0,3,6)**	**8.153**	**70.69**	**6.338**	**5.8**	**3.028**	**17.69**	**37.84**	**41.78**
	Equiv. Refl.: (0,-3,-6)								
**Quartz**	**(3,-3,6)**	**8.153**	**70.69**	**6.335**	**5.8**	**3.028**	**17.68**	**37.84**	**41.77**
	Equiv. Refl.: (-3,3,-6)								
**Quartz**	**(3,0,-6)**	**8.153**	**70.69**	**6.334**	**5.8**	**3.028**	**17.68**	**37.84**	**41.77**
	Equiv. Refl.: (-3,0,6)								
**Quartz**	**(0,4,-5)**	**8.177**	**71.17**	**4.776**	**4.5**	**2.946**	**13.32**	**36.83**	**39.16**
	Equiv. Refl.: (0,-4,5)								
**Quartz**	**(4,0,5)**	**8.177**	**71.17**	**4.768**	**4.5**	**2.946**	**13.3**	**36.83**	**39.16**
	Equiv. Refl.: (4,-4,-5), (-4,4,5)								
**Quartz**	**(-4,0,-5)**	**8.177**	**71.17**	**4.767**	**4.5**	**2.946**	**13.3**	**36.83**	**39.16**
**Quartz**	**(5,0,4)**	**8.609**	**85.25**	**4.59**	**6.2**	**0.718**	**4.48**	**8.97**	**10.03**
**Quartz**	**(-5,0,-4)**	**8.609**	**85.25**	**4.589**	**6.2**	**0.718**	**4.48**	**8.97**	**10.03**
**Quartz**	**(5,-5,-4)**	**8.609**	**85.25**	**4.587**	**6.2**	**0.718**	**4.48**	**8.97**	**10.03**
	Equiv. Refl.: (-5,5,4)								
**Quartz**	**(0,5,-4)**	**8.609**	**85.25**	**4.572**	**6.2**	**0.718**	**4.47**	**8.97**	**10.02**
	Equiv. Refl.: (0,-5,4)								
**Sapph**	**(2,4,-5)**	**8.311**	**74.15**	**4.402**	**4.7**	**2.452**	**11.52**	**30.65**	**32.75**
	Equiv. Refl.: (2,-6,5), (4,2,5), (4,-6,-5), (6,-2,5), (6,-4,-5), (-2,6,-5), (-2,-4,5), (-4,6,5), (-4,-2,-5) (-6,2,-5), (-6,4,5)								
**Sapph**	**(2,3,11)**	**8.4**	**76.5**	**3.695**	**4.2**	**2.074**	**8.78**	**25.92**	**27.37**
	Equiv. Refl.: (2,-5,-11), (3,2,-11), (3,-5,11), (5,-2,-11), (5,-3,11), (-2,5,11), (-2,-3,-11), (-3,5,-11), (-3,-2,11) (-5,2,11), (-5,3,-11)								
**LiNbO3**	**(2,2,15)**	**8.258**	**72.92**	**3.528**	**6.3**	**2.654**	**16.71**	**33.18**	**37.15**
	Equiv. Refl.: (2,2,-15), (2,-4,15), (2,-4,-15), (4,-2,15), (4,-2,-15), (-2,4,15), (-2,4,-15), (-2,-2,15), (-2,-2,-15) (-4,2,15), (-4,2,-15)								
**LiNbO3**	**(3,4,-1)**	**8.47**	**78.64**	**3.019**	**6.6**	**1.735**	**11.45**	**21.69**	**24.53**
	Equiv. Refl.: (3,-7,1), (4,3,1), (4,-7,-1), (7,-3,1), (7,-4,-1), (-3,7,-1), (-3,-4,1), (-4,7,1), (-4,-3,-1) (-7,3,-1), (-7,4,1)								
**Quartz**	**(3,-3,-6)**	**8.153**	**70.69**	**2.519**	**2.9**	**3.028**	**8.64**	**37.84**	**38.82**
	Equiv. Refl.: (-3,3,6)								
**Quartz**	**(3,0,6)**	**8.153**	**70.69**	**2.518**	**2.9**	**3.028**	**8.64**	**37.84**	**38.82**
	Equiv. Refl.: (-3,0,-6)								
**Quartz**	**(0,3,-6)**	**8.153**	**70.69**	**2.51**	**2.8**	**3.028**	**8.62**	**37.84**	**38.81**
	Equiv. Refl.: (0,-3,6)								
**Quartz**	**(6,-1,2)**	**8.43**	**77.38**	**2.15**	**2.9**	**1.934**	**5.58**	**24.18**	**24.81**
	Equiv. Refl.: (6,-5,-2), (-5,-1,-2)								
**Quartz**	**(-5,6,2)**	**8.43**	**77.38**	**2.149**	**2.9**	**1.934**	**5.58**	**24.18**	**24.81**
**Quartz**	**(-1,6,-2)**	**8.43**	**77.38**	**2.148**	**2.9**	**1.934**	**5.58**	**24.18**	**24.81**
	Equiv. Refl.: (-1,-5,2)								
**Quartz**	**(0,4,5)**	**8.177**	**71.17**	**1.995**	**2.6**	**2.946**	**7.62**	**36.83**	**37.61**
	Equiv. Refl.: (0,-4,-5)								
**Quartz**	**(-4,4,-5)**	**8.177**	**71.17**	**1.994**	**2.6**	**2.946**	**7.61**	**36.83**	**37.61**
**Quartz**	**(4,0,-5)**	**8.177**	**71.17**	**1.993**	**2.6**	**2.946**	**7.61**	**36.83**	**37.61**
	Equiv. Refl.: (4,-4,5), (-4,0,5)								
**Quartz**	**(5,1,2)**	**8.43**	**77.38**	**1.952**	**2.9**	**1.934**	**5.58**	**24.18**	**24.81**

Ei = Cryst	8.639 Refl (h,k,l)	keV EB [keV]	ΘB [°]	∫ IR dΘ [µrad]	Width [µrad]	Ei cotΘ [meV/µrad]	ΔE [meV]	ΔEg 2m, 50µm [meV]	ΔEt 2m, 50µm [meV]
**Quartz**	**(5,-6,-2)**	**8.43**	**77.38**	**1.951**	**2.9**	**1.934**	**5.58**	**24.18**	**24.81**
	Equiv. Refl.: (-6,1,-2), (-6,5,2)								
**Quartz**	**(1,5,-2)**	**8.43**	**77.38**	**1.95**	**2.9**	**1.934**	**5.58**	**24.18**	**24.81**
	Equiv. Refl.: (1,-6,2)								
**LiNbO3**	**(1,2,17)**	**8.445**	**77.85**	**1.749**	**4.7**	**1.86**	**8.73**	**23.25**	**24.84**
	Equiv. Refl.: (1,-3,-17), (2,1,-17), (2,-3,17), (3,-1,-17), (3,-2,17), (-1,3,17), (-1,-2,-17), (-2,3,-17), (-2,-1,17) (-3,1,17), (-3,2,-17)								
**Quartz**	**(0,1,-7)**	**8.16**	**70.84**	**1.742**	**2.3**	**3.002**	**6.97**	**37.53**	**38.17**
**Quartz**	**(0,-1,7)**	**8.16**	**70.84**	**1.741**	**2.3**	**3.002**	**6.97**	**37.53**	**38.17**
**Quartz**	**(1,-1,-7)**	**8.16**	**70.84**	**1.735**	**2.3**	**3.002**	**6.95**	**37.53**	**38.17**
	Equiv. Refl.: (-1,1,7)								
**Quartz**	**(-1,0,-7)**	**8.16**	**70.84**	**1.734**	**2.3**	**3.002**	**6.94**	**37.53**	**38.17**
**Quartz**	**(1,0,7)**	**8.16**	**70.84**	**1.733**	**2.3**	**3.002**	**6.94**	**37.53**	**38.17**
**LiNbO3**	**(1,5,-7)**	**8.351**	**75.16**	**1.655**	**4.2**	**2.289**	**9.6**	**28.62**	**30.18**
	Equiv. Refl.: (1,-6,7), (5,1,7), (5,-6,-7), (6,-1,7), (6,-5,-7), (-1,6,-7), (-1,-5,7), (-5,6,7), (-5,-1,-7) (-6,1,-7), (-6,5,7)								
**Sapph**	**(0,5,-8)**	**8.436**	**77.55**	**1.211**	**2.2**	**1.908**	**4.2**	**23.85**	**24.21**
	Equiv. Refl.: (0,-5,8), (5,0,8), (5,-5,-8), (-5,0,-8), (-5,5,8)								
**Sapph**	**(1,3,13)**	**8.241**	**72.55**	**1.196**	**2**	**2.715**	**5.36**	**33.94**	**34.36**
	Equiv. Refl.: (1,-4,-13), (3,1,-13), (3,-4,13), (4,-1,-13), (4,-3,13), (-1,4,13), (-1,-3,-13), (-3,4,-13), (-3,-1,13) (-4,1,13), (-4,3,-13)								
**Sapph**	**(3,3,6)**	**8.325**	**74.51**	**0.653**	**1.3**	**2.394**	**3.21**	**29.93**	**30.1**
	Equiv. Refl.: (3,3,-6), (3,-6,6), (3,-6,-6), (6,-3,6), (6,-3,-6), (-3,6,6), (-3,6,-6), (-3,-3,6), (-3,-3,-6) (-6,3,6), (-6,3,-6)								
**LiNbO3**	**(2,3,-13)**	**8.398**	**76.44**	**0.592**	**2.5**	**2.083**	**5.26**	**26.04**	**26.57**
	Equiv. Refl.: (2,-5,13), (3,2,13), (3,-5,-13), (5,-2,13), (5,-3,-13), (-2,5,-13), (-2,-3,13), (-3,5,13), (-3,-2,-13) (-5,2,-13), (-5,3,13)								
**Quartz**	**(6,-1,-1)**	**8.193**	**71.51**	**0.282**	**0.6**	**2.89**	**1.63**	**36.12**	**36.16**
	Equiv. Refl.: (6,-5,1), (-1,6,1), (-1,-5,-1), (-5,6,-1), (-5,-1,1)								
**Sapph**	**(1,5,2)**	**8.43**	**77.39**	**0.214**	**0.8**	**1.933**	**1.55**	**24.17**	**24.22**
	Equiv. Refl.: (1,-6,-2), (5,1,-2), (5,-6,2), (6,-1,-2), (6,-5,2), (-1,6,2), (-1,-5,-2), (-5,6,-2), (-5,-1,2) (-6,1,2), (-6,5,-2)								
**LiNbO3**	**(3,3,9)**	**8.27**	**73.2**	**0.188**	**1.3**	**2.608**	**3.43**	**32.59**	**32.77**
	Equiv. Refl.: (3,3,-9), (3,-6,9), (3,-6,-9), (6,-3,9), (6,-3,-9), (-3,6,9), (-3,6,-9), (-3,-3,9), (-3,-3,-9) (-6,3,9), (-6,3,-9)								
**Quartz**	**(1,5,1)**	**8.193**	**71.51**	**0.118**	**0.6**	**2.89**	**1.63**	**36.12**	**36.16**
	Equiv. Refl.: (1,-6,-1), (5,1,-1), (5,-6,1), (-6,1,1), (-6,5,-1)								

Ei =	8.616	keV						ΔEg	ΔEt
Cryst	Refl (h,k,l)	EB [keV]	ΘB [°]	∫IR dΘ [µrad]	Width [µrad]	Ei cotΘ [meV/µrad]	ΔE [meV]	2m, 50µm [meV]	2m, 50µm [meV]
**Si**	**(2,4,6)**	**8.542**	**82.47**	**31.8187**	**28.29**	**1.1382**	**32.2**	**14.23**	**35.2**
	Equiv. Refl.: (2,6,4), (4,2,6), (4,6,2), (6,2,4), (6,4,2)								
**Ge**	**(2,4,6)**	**8.2**	**72.12**	**27.4205**	**25.24**	**2.7788**	**70.15**	**34.74**	**78.28**
	Equiv. Refl.: (2,6,4), (4,2,6), (4,6,2), (6,2,4), (6,4,2)								
**Ge**	**(1,3,7)**	**8.417**	**77.66**	**23.8416**	**23.91**	**1.8854**	**45.08**	**23.57**	**50.87**
	Equiv. Refl.: (1,7,3), (3,1,7), (3,5,5), (3,7,1), (5,3,5), (5,5,3), (7,1,3), (7,3,1)								
**Si**	**(1,1,7)**	**8.152**	**71.1**	**9.9475**	**9.09**	**2.9497**	**26.81**	**36.87**	**45.59**
	Equiv. Refl.: (1,5,5), (1,7,1), (5,1,5), (5,5,1), (7,1,1)								
**LiNbO3**	**(4,2,-10)**	**8.61**	**87.88**	**186.531**	**173.4**	**0.32**	**55.41**	**3.99**	**55.55**
	Equiv. Refl.: (-2,-4,-10), (-4,6,-10), (-6,4,-10)								
**LiNbO3**	**(2,-6,-10)**	**8.61**	**87.88**	**186.53**	**173.4**	**0.32**	**55.41**	**3.99**	**55.55**
	Equiv. Refl.: (6,-2,-10)								
**LiNbO3**	**(4,-6,10)**	**8.61**	**87.88**	**172.534**	**173.4**	**0.32**	**55.41**	**3.99**	**55.55**
	Equiv. Refl.: (6,-4,10)								
**LiNbO3**	**(2,4,10)**	**8.61**	**87.88**	**172.533**	**173.4**	**0.32**	**55.41**	**3.99**	**55.55**
	Equiv. Refl.: (-2,6,10), (-4,-2,10), (-6,2,10)								
**Quartz**	**(5,0,-4)**	**8.609**	**87.75**	**96.52**	**82.7**	**0.339**	**28.04**	**4.24**	**28.36**
	Equiv. Refl.: (-5,0,4)								
**Quartz**	**(5,-5,4)**	**8.609**	**87.75**	**96.519**	**82.7**	**0.339**	**28.04**	**4.24**	**28.36**
	Equiv. Refl.: (-5,5,-4)								
**Quartz**	**(0,5,4)**	**8.609**	**87.75**	**96.51**	**82.7**	**0.339**	**28.04**	**4.24**	**28.36**
	Equiv. Refl.: (0,-5,-4)								
**Sapph**	**(1,2,-16)**	**8.612**	**88.19**	**84.814**	**78.7**	**0.272**	**21.39**	**3.4**	**21.66**
	Equiv. Refl.: (1,-3,16), (2,1,16), (2,-3,-16), (3,-1,16), (3,-2,-16), (-1,3,-16), (-1,-2,16), (-2,3,16), (-2,-1,-16) (-3,1,-16), (-3,2,16)								
**Sapph**	**(0,0,18)**	**8.591**	**85.66**	**74.649**	**63.5**	**0.655**	**41.56**	**8.18**	**42.36**
	Equiv. Refl.: (0,0,-18)								
**Sapph**	**(1,3,-14)**	**8.607**	**87.32**	**55.769**	**51.7**	**0.404**	**20.87**	**5.05**	**21.48**
	Equiv. Refl.: (1,-4,14), (3,1,14), (3,-4,-14), (4,-1,14), (4,-3,-14), (-1,4,-14), (-1,-3,14), (-3,4,14), (-3,-1,-14) (-4,1,-14), (-4,3,14)								
**Sapph**	**(1,5,-4)**	**8.591**	**85.64**	**55.365**	**48.4**	**0.657**	**31.79**	**8.21**	**32.83**
	Equiv. Refl.: (1,-6,4), (5,1,4), (5,-6,-4), (6,-1,4), (6,-5,-4), (-1,6,-4), (-1,-5,4), (-5,6,4), (-5,-1,-4) (-6,1,-4), (-6,5,4)								
**LiNbO3**	**(4,3,-2)**	**8.505**	**80.8**	**45.278**	**42.3**	**1.396**	**59.04**	**17.44**	**61.56**
	Equiv. Refl.: (-3,-4,-2), (-4,7,-2), (-7,4,-2)								
**LiNbO3**	**(3,-7,-2)**	**8.505**	**80.8**	**45.277**	**42.3**	**1.396**	**59.04**	**17.44**	**61.56**
	Equiv. Refl.: (7,-3,-2)								
**LiNbO3**	**(3,4,2)**	**8.505**	**80.8**	**42.905**	**42.3**	**1.396**	**59.04**	**17.44**	**61.56**
	Equiv. Refl.: (4,-7,2), (7,-4,2), (-3,7,2), (-4,-3,2), (-7,3,2)								
**LiNbO3**	**(1,5,8)**	**8.528**	**81.83**	**41.833**	**41.2**	**1.237**	**50.96**	**15.47**	**53.26**
	Equiv. Refl.: (5,-6,8), (6,-5,8), (-1,6,8), (-5,-1,8), (-6,1,8)								
**LiNbO3**	**(1,-6,-8)**	**8.528**	**81.83**	**41.7**	**41.2**	**1.237**	**50.96**	**15.47**	**53.26**
	Equiv. Refl.: (5,1,-8), (6,-1,-8), (-1,-5,-8), (-5,6,-8), (-6,5,-8)								
**Quartz**	**(-5,2,-5)**	**8.557**	**83.3**	**38.918**	**32.6**	**1.012**	**32.95**	**12.65**	**35.3**
	Equiv. Refl.: (-5,3,5)								
**Quartz**	**(3,-5,-5)**	**8.557**	**83.3**	**38.914**	**32.5**	**1.012**	**32.95**	**12.65**	**35.29**
**Quartz**	**(2,-5,5)**	**8.557**	**83.3**	**38.913**	**32.5**	**1.012**	**32.95**	**12.65**	**35.29**

Ei =	8.616	keV						ΔEg	ΔEt
**Cryst**	**Refl**	**EB**	**ΘB**	**∫IR dΘ**	**Width**	**Ei cotΘ**	**ΔE**	**2m, 50µm**	**2m, 50µm**
	**(h,k,l)**	**[keV]**	**[°]**	**[µrad]**	**[µrad]**	**[meV/µrad]**	**[meV]**	**[meV]**	**[meV]**
**Quartz**	**(2,3,-5)**	**8.557**	**83.3**	**38.912**	**32.5**	**1.012**	**32.95**	**12.65**	**35.29**
	Equiv. Refl.: (3,2,5)								
**Quartz**	**(5,-2,5)**	**8.557**	**83.3**	**38.491**	**32.6**	**1.012**	**32.95**	**12.65**	**35.3**
**Quartz**	**(5,-3,-5)**	**8.557**	**83.3**	**38.49**	**32.6**	**1.012**	**32.95**	**12.65**	**35.3**
**Quartz**	**(-2,5,-5)**	**8.557**	**83.3**	**38.486**	**32.5**	**1.012**	**32.95**	**12.65**	**35.29**
	Equiv. Refl.: (-2,-3,5)								
**Quartz**	**(-3,5,5)**	**8.557**	**83.3**	**38.485**	**32.5**	**1.012**	**32.95**	**12.65**	**35.29**
**Quartz**	**(-3,-2,-5)**	**8.557**	**83.3**	**38.483**	**32.5**	**1.012**	**32.95**	**12.65**	**35.29**
**LiNbO3**	**(0,6,0)**	**8.343**	**75.54**	**31.843**	**29.8**	**2.222**	**66.27**	**27.78**	**71.86**
	Equiv. Refl.: (6,-6,0), (-6,0,0)								
**LiNbO3**	**(0,-6,0)**	**8.343**	**75.54**	**31.155**	**29.8**	**2.222**	**66.27**	**27.78**	**71.86**
	Equiv. Refl.: (6,0,0), (-6,6,0)								
**LiNbO3**	**(1,1,-18)**	**8.402**	**77.2**	**28.51**	**27**	**1.958**	**52.94**	**24.48**	**58.33**
	Equiv. Refl.: (1,-2,-18), (2,-1,-18), (-1,2,-18), (-1,-1,-18), (-2,1,-18)								
**LiNbO3**	**(0,-4,-14)**	**8.374**	**76.4**	**27.607**	**26.1**	**2.085**	**54.41**	**26.06**	**60.33**
	Equiv. Refl.: (4,0,-14), (-4,4,-14)								
**LiNbO3**	**(1,1,18)**	**8.402**	**77.2**	**26.399**	**27**	**1.958**	**52.94**	**24.48**	**58.33**
	Equiv. Refl.: (1,-2,18), (2,-1,18), (-1,2,18), (-1,-1,18), (-2,1,18)								
**LiNbO3**	**(1,-5,12)**	**8.331**	**75.21**	**26.125**	**24.6**	**2.275**	**55.92**	**28.44**	**62.73**
	Equiv. Refl.: (4,1,12), (5,-1,12), (-1,-4,12), (-4,5,12), (-5,4,12)								
**LiNbO3**	**(0,4,14)**	**8.374**	**76.4**	**25.609**	**26.1**	**2.085**	**54.41**	**26.06**	**60.33**
	Equiv. Refl.: (4,-4,14), (-4,0,14)								
**LiNbO3**	**(1,4,12)**	**8.331**	**75.21**	**25.272**	**23.9**	**2.275**	**54.34**	**28.44**	**61.33**
	Equiv. Refl.: (4,-5,12), (5,-4,12), (-1,5,12), (-4,-1,12), (-5,1,12)								
**LiNbO3**	**(1,4,-12)**	**8.331**	**75.21**	**24.184**	**24.6**	**2.275**	**55.92**	**28.44**	**62.73**
	Equiv. Refl.: (4,-5,-12), (5,-4,-12), (-1,5,-12), (-4,-1,-12), (-5,1,-12)								
**LiNbO3**	**(1,-5,-12)**	**8.331**	**75.21**	**23.344**	**23.9**	**2.275**	**54.34**	**28.44**	**61.33**
	Equiv. Refl.: (4,1,-12), (5,-1,-12), (-1,-4,-12), (-4,5,-12), (-5,4,-12)								
**LiNbO3**	**(5,-5,10)**	**8.266**	**73.62**	**23.085**	**21.2**	**2.532**	**53.74**	**31.65**	**62.37**
**LiNbO3**	**(0,5,10)**	**8.266**	**73.62**	**23.084**	**21.2**	**2.532**	**53.74**	**31.65**	**62.37**
	Equiv. Refl.: (-5,0,10)								
**LiNbO3**	**(0,-5,-10)**	**8.266**	**73.62**	**19.844**	**21.2**	**2.532**	**53.74**	**31.65**	**62.37**
	Equiv. Refl.: (5,0,-10), (-5,5,-10)								
**LiNbO3**	**(2,-6,8)**	**8.181**	**71.72**	**19.596**	**18.7**	**2.846**	**53.31**	**35.57**	**64.08**
	Equiv. Refl.: (4,2,8), (6,-2,8), (-2,-4,8)								
**LiNbO3**	**(-4,6,8)**	**8.181**	**71.72**	**19.595**	**18.7**	**2.846**	**53.31**	**35.57**	**64.08**
	Equiv. Refl.: (-6,4,8)								
**LiNbO3**	**(2,4,-8)**	**8.181**	**71.72**	**18.098**	**18.7**	**2.846**	**53.31**	**35.57**	**64.08**
	Equiv. Refl.: (4,-6,-8), (6,-4,-8), (-2,6,-8), (-4,-2,-8), (-6,2,-8)								
**Quartz**	**(2,2,6)**	**8.534**	**82.11**	**16.86**	**15.2**	**1.194**	**18.13**	**14.92**	**23.48**
**Quartz**	**(2,-4,-6)**	**8.534**	**82.11**	**16.859**	**15.2**	**1.194**	**18.13**	**14.92**	**23.48**
**Quartz**	**(2,-4,6)**	**8.534**	**82.11**	**16.858**	**15.2**	**1.194**	**18.13**	**14.92**	**23.48**
**Quartz**	**(2,2,-6)**	**8.534**	**82.11**	**16.857**	**15.2**	**1.194**	**18.13**	**14.92**	**23.48**
**Quartz**	**(-4,2,-6)**	**8.534**	**82.11**	**16.854**	**15.2**	**1.194**	**18.12**	**14.92**	**23.48**
**Quartz**	**(-4,2,6)**	**8.534**	**82.11**	**16.851**	**15.2**	**1.194**	**18.12**	**14.92**	**23.47**
**Quartz**	**(-2,-2,-6)**	**8.534**	**82.11**	**16.6**	**15.2**	**1.194**	**18.13**	**14.92**	**23.48**
**Quartz**	**(-2,4,6)**	**8.534**	**82.11**	**16.599**	**15.2**	**1.194**	**18.13**	**14.92**	**23.48**
**Quartz**	**(-2,4,-6)**	**8.534**	**82.11**	**16.598**	**15.2**	**1.194**	**18.13**	**14.92**	**23.48**
	Equiv. Refl.: (-2,-2,6)								
**Quartz**	**(4,-2,6)**	**8.534**	**82.11**	**16.59**	**15.2**	**1.194**	**18.12**	**14.92**	**23.48**
**Quartz**	**(4,-2,-6)**	**8.534**	**82.11**	**16.587**	**15.2**	**1.194**	**18.12**	**14.92**	**23.47**

**Ei =**	**8.616**	**keV**						**ΔEg**	**ΔEt**
**Cryst**	**Refl**	**EB**	**ΘB**	**∫ IR dΘ**	**Width**	**Ei cotΘ**	**ΔE**	**2m, 50µm**	**2m, 50µm**
	**(h,k,l)**	**[keV]**	**[°]**	**[µrad]**	**[µrad]**	**[meV/µrad]**	**[meV]**	**[meV]**	**[meV]**
**Quartz**	**(-3,5,-5)**	**8.557**	**83.3**	**14.235**	**13.5**	**1.012**	**13.63**	**12.65**	**18.6**
**Quartz**	**(-3,-2,5)**	**8.557**	**83.3**	**14.233**	**13.5**	**1.012**	**13.63**	**12.65**	**18.6**
**Quartz**	**(-2,5,5)**	**8.557**	**83.3**	**14.228**	**13.5**	**1.012**	**13.62**	**12.65**	**18.59**
**Quartz**	**(-2,-3,-5)**	**8.557**	**83.3**	**14.227**	**13.5**	**1.012**	**13.62**	**12.65**	**18.59**
**Quartz**	**(5,-2,-5)**	**8.557**	**83.3**	**14.218**	**13.5**	**1.012**	**13.62**	**12.65**	**18.59**
**Quartz**	**(5,-3,5)**	**8.557**	**83.3**	**14.217**	**13.4**	**1.012**	**13.62**	**12.65**	**18.59**
**Quartz**	**(3,-5,5)**	**8.557**	**83.3**	**13.947**	**13.5**	**1.012**	**13.63**	**12.65**	**18.6**
**Quartz**	**(3,2,-5)**	**8.557**	**83.3**	**13.945**	**13.5**	**1.012**	**13.63**	**12.65**	**18.6**
**Quartz**	**(2,-5,-5)**	**8.557**	**83.3**	**13.939**	**13.5**	**1.012**	**13.62**	**12.65**	**18.59**
**Quartz**	**(2,3,5)**	**8.557**	**83.3**	**13.938**	**13.5**	**1.012**	**13.62**	**12.65**	**18.59**
**Quartz**	**(-5,2,5)**	**8.557**	**83.3**	**13.929**	**13.5**	**1.012**	**13.62**	**12.65**	**18.59**
	Equiv. Refl.: (-5,3,-5)								
**Sapph**	**(2,4,4)**	**8.186**	**71.83**	**12.322**	**10.7**	**2.828**	**30.14**	**35.35**	**46.45**
	Equiv. Refl.: (2,-6,-4), (4,2,-4), (4,-6,4), (6,-2,-4), (6,-4,4), (-2,6,4), (-2,-4,-4), (-4,6,-4), (-4,-2,4) (-6,2,4), (-6,4,-4)								
**Sapph**	**(0,2,16)**	**8.208**	**72.3**	**11.599**	**10.2**	**2.75**	**28.08**	**34.38**	**44.39**
	Equiv. Refl.: (0,-2,-16), (2,0,-16), (2,-2,16), (-2,0,16), (-2,2,-16)								
**Quartz**	**(1,5,-1)**	**8.193**	**71.97**	**11.529**	**9.7**	**2.805**	**27.31**	**35.06**	**44.44**
	Equiv. Refl.: (1,-6,1)								
**Quartz**	**(-6,1,-1)**	**8.193**	**71.97**	**11.528**	**9.7**	**2.805**	**27.31**	**35.06**	**44.44**
	Equiv. Refl.: (-6,5,1)								
**Quartz**	**(5,1,1)**	**8.193**	**71.97**	**11.527**	**9.7**	**2.805**	**27.31**	**35.06**	**44.44**
	Equiv. Refl.: (5,-6,-1)								
**Quartz**	**(6,-1,1)**	**8.193**	**71.97**	**11.476**	**9.7**	**2.805**	**27.31**	**35.06**	**44.44**
	Equiv. Refl.: (-1,6,-1), (-1,-5,1)								
**Quartz**	**(6,-5,-1)**	**8.193**	**71.97**	**11.475**	**9.7**	**2.805**	**27.31**	**35.06**	**44.44**
	Equiv. Refl.: (-5,6,1), (-5,-1,-1)								
**Quartz**	**(-2,1,-7)**	**8.416**	**77.64**	**11.047**	**9.7**	**1.888**	**18.28**	**23.6**	**29.85**
**Quartz**	**(-2,1,7)**	**8.416**	**77.64**	**11.046**	**9.7**	**1.888**	**18.28**	**23.6**	**29.85**
**Quartz**	**(1,-2,-7)**	**8.416**	**77.64**	**11.045**	**9.7**	**1.888**	**18.28**	**23.6**	**29.85**
**Quartz**	**(1,1,7)**	**8.416**	**77.64**	**11.042**	**9.7**	**1.888**	**18.28**	**23.6**	**29.85**
**Quartz**	**(1,-2,7)**	**8.416**	**77.64**	**11.04**	**9.7**	**1.888**	**18.27**	**23.6**	**29.84**
**Quartz**	**(1,1,-7)**	**8.416**	**77.64**	**11.039**	**9.7**	**1.888**	**18.27**	**23.6**	**29.84**
**Quartz**	**(2,-2,-7)**	**8.542**	**82.46**	**10.906**	**10.7**	**1.14**	**12.2**	**14.25**	**18.76**
	Equiv. Refl.: (-2,2,7)								
**Quartz**	**(-2,0,-7)**	**8.542**	**82.46**	**10.903**	**10.7**	**1.14**	**12.19**	**14.25**	**18.76**
**Quartz**	**(2,0,7)**	**8.542**	**82.46**	**10.902**	**10.7**	**1.14**	**12.19**	**14.25**	**18.76**
**Quartz**	**(0,2,-7)**	**8.542**	**82.46**	**10.884**	**10.7**	**1.14**	**12.18**	**14.25**	**18.75**
	Equiv. Refl.: (0,-2,7)								
**Quartz**	**(2,-1,7)**	**8.416**	**77.64**	**10.598**	**9.7**	**1.888**	**18.28**	**23.6**	**29.85**
**Quartz**	**(2,-1,-7)**	**8.416**	**77.64**	**10.596**	**9.7**	**1.888**	**18.28**	**23.6**	**29.85**
**Quartz**	**(-1,2,7)**	**8.416**	**77.64**	**10.594**	**9.7**	**1.888**	**18.28**	**23.6**	**29.85**
**Quartz**	**(-1,-1,-7)**	**8.416**	**77.64**	**10.591**	**9.7**	**1.888**	**18.28**	**23.6**	**29.85**
**Quartz**	**(-1,2,-7)**	**8.416**	**77.64**	**10.59**	**9.7**	**1.888**	**18.27**	**23.6**	**29.84**
**Quartz**	**(-1,-1,7)**	**8.416**	**77.64**	**10.589**	**9.7**	**1.888**	**18.27**	**23.6**	**29.84**
**Sapph**	**(2,3,-10)**	**8.111**	**70.28**	**9.755**	**8.6**	**3.088**	**26.52**	**38.61**	**46.84**
	Equiv. Refl.: (2,-5,10), (3,2,10), (3,-5,-10), (5,-2,10), (5,-3,-10), (-2,5,-10), (-2,-3,10), (-3,5,10), (-3,-2,-10) (-5,2,-10), (-5,3,10)								
**Quartz**	**(2,0,-7)**	**8.542**	**82.46**	**9.523**	**9.6**	**1.14**	**10.96**	**14.25**	**17.98**
	Equiv. Refl.: (-2,0,7)								

Ei =	8.616	keV						ΔEg	ΔEt
Cryst	Refl	EB	ΘB	∫ IR dΘ	Width	Ei cotΘ	ΔE	2m, 50µm	2m, 50µm
	(h,k,l)	[keV]	[°]	[µrad]	[µrad]	[meV/µrad]	[meV]	[meV]	[meV]
**Quartz**	**(2,-2,7)**	**8.542**	**82.46**	**9.521**	**9.6**	**1.14**	**10.96**	**14.25**	**17.98**
	Equiv. Refl.: (-2,2,-7)								
**Quartz**	**(5,0,4)**	**8.609**	**87.75**	**9.5**	**13.2**	**0.339**	**4.47**	**4.24**	**6.16**
**Quartz**	**(-5,0,-4)**	**8.609**	**87.75**	**9.499**	**13.2**	**0.339**	**4.47**	**4.24**	**6.16**
**Quartz**	**(5,-5,-4)**	**8.609**	**87.75**	**9.495**	**13.2**	**0.339**	**4.47**	**4.24**	**6.16**
	Equiv. Refl.: (-5,5,4)								
**Quartz**	**(0,2,7)**	**8.542**	**82.46**	**9.491**	**9.6**	**1.14**	**10.93**	**14.25**	**17.96**
	Equiv. Refl.: (0,-2,-7)								
**Quartz**	**(0,5,-4)**	**8.609**	**87.75**	**9.463**	**13.1**	**0.339**	**4.46**	**4.24**	**6.15**
	Equiv. Refl.: (0,-5,4)								
**Quartz**	**(-4,-2,-3)**	**8.443**	**78.49**	**9.459**	**8.2**	**1.755**	**14.42**	**21.93**	**26.25**
**Quartz**	**(-4,6,3)**	**8.443**	**78.49**	**9.457**	**8.2**	**1.755**	**14.42**	**21.93**	**26.25**
**Quartz**	**(6,-4,-3)**	**8.443**	**78.49**	**9.454**	**8.2**	**1.755**	**14.42**	**21.93**	**26.25**
**Quartz**	**(6,-2,3)**	**8.443**	**78.49**	**9.453**	**8.2**	**1.755**	**14.41**	**21.93**	**26.24**
**Quartz**	**(-2,6,-3)**	**8.443**	**78.49**	**9.452**	**8.2**	**1.755**	**14.41**	**21.93**	**26.24**
**Quartz**	**(-2,-4,3)**	**8.443**	**78.49**	**9.451**	**8.2**	**1.755**	**14.41**	**21.93**	**26.24**
**Quartz**	**(2,-6,-3)**	**8.443**	**78.49**	**9.032**	**8.2**	**1.755**	**14.32**	**21.93**	**26.2**
**Quartz**	**(2,4,3)**	**8.443**	**78.49**	**9.031**	**8.2**	**1.755**	**14.32**	**21.93**	**26.2**
**Quartz**	**(-6,4,-3)**	**8.443**	**78.49**	**9.029**	**8.2**	**1.755**	**14.32**	**21.93**	**26.19**
**Quartz**	**(-6,2,3)**	**8.443**	**78.49**	**9.027**	**8.2**	**1.755**	**14.32**	**21.93**	**26.19**
**Quartz**	**(4,2,-3)**	**8.443**	**78.49**	**9.026**	**8.2**	**1.755**	**14.32**	**21.93**	**26.19**
	Equiv. Refl.: (4,-6,3)								
**Quartz**	**(5,-1,4)**	**8.101**	**70.09**	**8.758**	**7.5**	**3.12**	**23.37**	**39**	**45.47**
	Equiv. Refl.: (5,-4,-4), (-1,-4,4)								
**Quartz**	**(-1,5,-4)**	**8.101**	**70.09**	**8.757**	**7.5**	**3.12**	**23.37**	**39**	**45.46**
	Equiv. Refl.: (-4,5,4), (-4,-1,-4)								
**Quartz**	**(1,4,-4)**	**8.101**	**70.09**	**8.607**	**7.5**	**3.12**	**23.37**	**39**	**45.47**
**Quartz**	**(1,-5,4)**	**8.101**	**70.09**	**8.606**	**7.5**	**3.12**	**23.37**	**39**	**45.46**
	Equiv. Refl.: (4,1,4), (-5,1,-4), (-5,4,4)								
**Quartz**	**(4,-5,-4)**	**8.101**	**70.09**	**8.605**	**7.5**	**3.12**	**23.36**	**39**	**45.46**
**Quartz**	**(-2,6,3)**	**8.443**	**78.49**	**8.398**	**8.2**	**1.755**	**14.32**	**21.93**	**26.2**
	Equiv. Refl.: (-2,-4,-3)								
**Quartz**	**(6,-4,3)**	**8.443**	**78.49**	**8.395**	**8.2**	**1.755**	**14.32**	**21.93**	**26.19**
**Quartz**	**(6,-2,-3)**	**8.443**	**78.49**	**8.393**	**8.2**	**1.755**	**14.32**	**21.93**	**26.19**
**Quartz**	**(-4,6,-3)**	**8.443**	**78.49**	**8.392**	**8.2**	**1.755**	**14.32**	**21.93**	**26.19**
	Equiv. Refl.: (-4,-2,3)								
**Quartz**	**(-1,0,7)**	**8.16**	**71.28**	**8.094**	**7.1**	**2.92**	**20.76**	**36.49**	**41.99**
**Quartz**	**(1,0,-7)**	**8.16**	**71.28**	**8.093**	**7.1**	**2.92**	**20.76**	**36.49**	**41.99**
	Equiv. Refl.: (-1,1,-7)								
**Quartz**	**(0,1,7)**	**8.16**	**71.28**	**8.092**	**7.1**	**2.92**	**20.76**	**36.49**	**41.98**
	Equiv. Refl.: (0,-1,-7), (1,-1,7)								
**Quartz**	**(4,2,3)**	**8.443**	**78.49**	**7.965**	**8.2**	**1.755**	**14.42**	**21.93**	**26.25**
**Quartz**	**(4,-6,-3)**	**8.443**	**78.49**	**7.964**	**8.2**	**1.755**	**14.42**	**21.93**	**26.25**
**Quartz**	**(-6,4,3)**	**8.443**	**78.49**	**7.961**	**8.2**	**1.755**	**14.42**	**21.93**	**26.25**
**Quartz**	**(-6,2,-3)**	**8.443**	**78.49**	**7.96**	**8.2**	**1.755**	**14.41**	**21.93**	**26.24**
**Quartz**	**(2,-6,3)**	**8.443**	**78.49**	**7.959**	**8.2**	**1.755**	**14.41**	**21.93**	**26.24**
**Quartz**	**(2,4,-3)**	**8.443**	**78.49**	**7.958**	**8.2**	**1.755**	**14.41**	**21.93**	**26.24**
**Quartz**	**(-6,3,3)**	**8.316**	**74.84**	**7.789**	**7.1**	**2.335**	**16.49**	**29.19**	**33.52**
**Quartz**	**(3,3,3)**	**8.316**	**74.84**	**7.788**	**7.1**	**2.335**	**16.49**	**29.19**	**33.52**
	Equiv. Refl.: (3,-6,-3), (-6,3,-3)								

Ei =	8.616	keV						ΔEg	ΔEt
Cryst	Refl	EB	ΘB	∫ IR dΘ	Width	Ei cotΘ	ΔE	2m, 50µm	2m, 50µm
	(h,k,l)	[keV]	[°]	[µrad]	[µrad]	[meV/µrad]	[meV]	[meV]	[meV]
**Quartz**	**(3,3,-3)**	**8.316**	**74.84**	**7.787**	**7.1**	**2.335**	**16.49**	**29.19**	**33.52**
	Equiv. Refl.: (3,-6,3)								
**Sapph**	**(1,4,9)**	**8.123**	**70.52**	**7.78**	**7.3**	**3.047**	**22.2**	**38.09**	**44.09**
	Equiv. Refl.: (1,4,-9), (1,-5,9), (1,-5,-9), (4,1,9), (4,1,-9), (4,-5,9), (4,-5,-9), (5,-1,9), (5,-1,-9) (5,-4,9), (5,-4,-9), (-1,5,9), (-1,5,-9), (-1,-4,9), (-1,-4,-9), (-4,5,9), (-4,5,-9), (-4,-1,9) (-4,-1,-9), (-5,1,9), (-5,1,-9), (-5,4,9), (-5,4,-9)								
**Quartz**	**(-3,6,3)**	**8.316**	**74.84**	**7.744**	**7.1**	**2.335**	**16.49**	**29.19**	**33.52**
**Quartz**	**(6,-3,3)**	**8.316**	**74.84**	**7.743**	**7.1**	**2.335**	**16.49**	**29.19**	**33.52**
	Equiv. Refl.: (6,-3,-3), (-3,6,-3), (-3,-3,3), (-3,-3,-3)								
**Quartz**	**(-5,6,-2)**	**8.43**	**78.08**	**7.63**	**7.2**	**1.818**	**13.17**	**22.73**	**26.27**
	Equiv. Refl.: (-5,-1,2)								
**Quartz**	**(6,-5,2)**	**8.43**	**78.08**	**7.628**	**7.2**	**1.818**	**13.17**	**22.73**	**26.27**
	Equiv. Refl.: (-1,6,2), (-1,-5,-2)								
**Quartz**	**(6,-1,-2)**	**8.43**	**78.08**	**7.627**	**7.2**	**1.818**	**13.17**	**22.73**	**26.27**
**Quartz**	**(5,1,-2)**	**8.43**	**78.08**	**7.481**	**7.2**	**1.818**	**13.17**	**22.73**	**26.27**
	Equiv. Refl.: (5,-6,2)								
**Quartz**	**(1,5,2)**	**8.43**	**78.08**	**7.48**	**7.2**	**1.818**	**13.17**	**22.73**	**26.27**
	Equiv. Refl.: (-6,5,-2)								
**Quartz**	**(1,-6,-2)**	**8.43**	**78.08**	**7.479**	**7.2**	**1.818**	**13.17**	**22.73**	**26.27**
	Equiv. Refl.: (-6,1,2)								
**Sapph**	**(1,5,-1)**	**8.39**	**76.84**	**7.292**	**7.4**	**2.014**	**14.89**	**25.17**	**29.25**
	Equiv. Refl.: (1,-6,1), (5,1,1), (5,-6,-1), (6,-1,1), (6,-5,-1), (-1,6,-1), (-1,-5,1), (-5,6,1), (-5,-1,-1) (-6,1,-1), (-6,5,1)								
**Quartz**	**(0,3,6)**	**8.153**	**71.13**	**6.493**	**6**	**2.945**	**17.65**	**36.82**	**40.83**
**Quartz**	**(0,-3,-6)**	**8.153**	**71.13**	**6.492**	**6**	**2.945**	**17.65**	**36.82**	**40.83**
**Quartz**	**(-3,3,-6)**	**8.153**	**71.13**	**6.489**	**6**	**2.945**	**17.64**	**36.82**	**40.82**
**Quartz**	**(3,0,-6)**	**8.153**	**71.13**	**6.488**	**6**	**2.945**	**17.64**	**36.82**	**40.82**
	Equiv. Refl.: (3,-3,6), (-3,0,6)								
**Quartz**	**(0,-4,5)**	**8.177**	**71.62**	**4.897**	**4.6**	**2.862**	**13.28**	**35.78**	**38.17**
**Quartz**	**(0,4,-5)**	**8.177**	**71.62**	**4.896**	**4.6**	**2.862**	**13.28**	**35.78**	**38.17**
**Quartz**	**(4,0,5)**	**8.177**	**71.62**	**4.888**	**4.6**	**2.862**	**13.27**	**35.78**	**38.16**
	Equiv. Refl.: (4,-4,-5), (-4,0,-5), (-4,4,5)								
**Sapph**	**(2,4,-5)**	**8.311**	**74.7**	**4.56**	**4.9**	**2.357**	**11.49**	**29.46**	**31.62**
	Equiv. Refl.: (2,-6,5), (4,2,5), (4,-6,-5), (6,-2,5), (6,-4,-5), (-2,6,-5), (-2,-4,5), (-4,6,5), (-4,-2,-5) (-6,2,-5), (-6,4,5)								
**Quartz**	**(1,5,0)**	**8.112**	**70.31**	**4.111**	**3.7**	**3.083**	**11.52**	**38.54**	**40.23**
	Equiv. Refl.: (1,-6,0), (5,1,0), (5,-6,0), (-6,1,0), (-6,5,0)								
**Sapph**	**(2,3,11)**	**8.4**	**77.16**	**3.881**	**4.5**	**1.964**	**8.76**	**24.56**	**26.07**
	Equiv. Refl.: (2,-5,-11), (3,2,-11), (3,-5,11), (5,-2,-11), (5,-3,11), (-2,5,11), (-2,-3,-11), (-3,5,-11), (-3,-2,11) (-5,2,11), (-5,3,-11)								
**LiNbO3**	**(2,2,15)**	**8.258**	**73.42**	**3.774**	**6.5**	**2.564**	**16.67**	**32.06**	**36.13**
	Equiv. Refl.: (2,2,-15), (2,-4,15), (2,-4,-15), (4,-2,15), (4,-2,-15), (-2,4,15), (-2,4,-15), (-2,-2,15), (-2,-2,-15) (-4,2,15), (-4,2,-15)								
**Quartz**	**(6,-1,0)**	**8.112**	**70.31**	**3.683**	**3.7**	**3.083**	**11.52**	**38.54**	**40.23**
	Equiv. Refl.: (6,-5,0), (-1,6,0), (-1,-5,0), (-5,6,0), (-5,-1,0)								
**LiNbO3**	**(3,4,-1)**	**8.47**	**79.43**	**3.24**	**7.1**	**1.608**	**11.42**	**20.1**	**23.11**
	Equiv. Refl.: (3,-7,1), (4,3,1), (4,-7,-1), (7,-3,1), (7,-4,-1), (-3,7,-1), (-3,-4,1), (-4,7,1), (-4,-3,-1) (-7,3,-1), (-7,4,1)								
**Quartz**	**(3,-3,-6)**	**8.153**	**71.13**	**2.578**	**2.9**	**2.945**	**8.62**	**36.82**	**37.81**
	Equiv. Refl.: (-3,3,6)								

Ei =<br>Cryst	8.616<br>Refl<br>(h,k,l)	keV<br>EB<br>[keV]	ΘB<br>[°]	∫ IR dΘ<br>[µrad]	Width<br>[µrad]	Ei cotΘ<br>[meV/µrad]	ΔE<br>[meV]	ΔEg<br>2m, 50µm<br>[meV]	ΔEt<br>2m, 50µm<br>[meV]
**Quartz**	**(3,0,6)**	**8.153**	**71.13**	**2.577**	**2.9**	**2.945**	**8.62**	**36.82**	**37.81**
	Equiv. Refl.: (-3,0,-6)								
**Quartz**	**(0,3,-6)**	**8.153**	**71.13**	**2.569**	**2.9**	**2.945**	**8.6**	**36.82**	**37.81**
	Equiv. Refl.: (0,-3,6)								
**Quartz**	**(6,-1,2)**	**8.43**	**78.08**	**2.277**	**3.1**	**1.818**	**5.56**	**22.73**	**23.4**
	Equiv. Refl.: (6,-5,-2), (-5,6,2), (-5,-1,-2)								
**Quartz**	**(-1,6,-2)**	**8.43**	**78.08**	**2.276**	**3.1**	**1.818**	**5.56**	**22.73**	**23.4**
	Equiv. Refl.: (-1,-5,2)								
**Quartz**	**(5,1,2)**	**8.43**	**78.08**	**2.066**	**3.1**	**1.818**	**5.57**	**22.73**	**23.4**
	Equiv. Refl.: (5,-6,-2), (-6,1,-2), (-6,5,2)								
**Quartz**	**(1,5,-2)**	**8.43**	**78.08**	**2.065**	**3.1**	**1.818**	**5.56**	**22.73**	**23.4**
	Equiv. Refl.: (1,-6,2)								
**Quartz**	**(0,4,5)**	**8.177**	**71.62**	**2.045**	**2.7**	**2.862**	**7.6**	**35.78**	**36.58**
	Equiv. Refl.: (0,-4,-5)								
**Quartz**	**(4,-4,5)**	**8.177**	**71.62**	**2.043**	**2.7**	**2.862**	**7.59**	**35.78**	**36.58**
	Equiv. Refl.: (-4,4,-5)								
**Quartz**	**(4,0,-5)**	**8.177**	**71.62**	**2.042**	**2.7**	**2.862**	**7.59**	**35.78**	**36.58**
	Equiv. Refl.: (-4,0,5)								
**LiNbO3**	**(1,2,17)**	**8.445**	**78.58**	**1.858**	**5**	**1.741**	**8.71**	**21.76**	**23.44**
	Equiv. Refl.: (1,-3,-17), (2,1,-17), (2,-3,17), (3,-1,-17), (3,-2,17), (-1,3,17), (-1,-2,-17), (-2,3,-17), (-2,-1,17) (-3,1,17), (-3,2,-17)								
**Quartz**	**(0,1,-7)**	**8.16**	**71.28**	**1.783**	**2.4**	**2.92**	**6.95**	**36.49**	**37.15**
	Equiv. Refl.: (0,-1,7)								
**Quartz**	**(1,-1,-7)**	**8.16**	**71.28**	**1.776**	**2.4**	**2.92**	**6.93**	**36.49**	**37.15**
	Equiv. Refl.: (-1,1,7)								
**Quartz**	**(1,0,7)**	**8.16**	**71.28**	**1.775**	**2.4**	**2.92**	**6.92**	**36.49**	**37.15**
	Equiv. Refl.: (-1,0,-7)								
**LiNbO3**	**(1,5,-7)**	**8.351**	**75.75**	**1.72**	**4.4**	**2.189**	**9.58**	**27.36**	**28.99**
	Equiv. Refl.: (1,-6,7), (5,1,7), (5,-6,-7), (6,-1,7), (6,-5,-7), (-1,6,-7), (-1,-5,7), (-5,6,7), (-5,-1,-7) (-6,1,-7), (-6,5,7)								
**Sapph**	**(0,5,-8)**	**8.436**	**78.26**	**1.284**	**2.3**	**1.791**	**4.19**	**22.38**	**22.77**
	Equiv. Refl.: (0,-5,8), (5,0,8), (5,-5,-8), (-5,0,-8), (-5,5,8)								
**Sapph**	**(1,3,13)**	**8.241**	**73.04**	**1.23**	**2**	**2.627**	**5.35**	**32.84**	**33.27**
	Equiv. Refl.: (1,-4,-13), (3,1,-13), (3,-4,13), (4,-1,-13), (4,-3,13), (-1,4,13), (-1,-3,-13), (-3,4,-13), (-3,-1,13) (-4,1,13), (-4,3,-13)								
**Quartz**	**(1,4,4)**	**8.101**	**70.09**	**1.155**	**1.6**	**3.12**	**4.86**	**39**	**39.3**
	Equiv. Refl.: (1,-5,-4)								
**Quartz**	**(-5,4,-4)**	**8.101**	**70.09**	**1.153**	**1.6**	**3.12**	**4.85**	**39**	**39.3**
**Quartz**	**(4,1,-4)**	**8.101**	**70.09**	**1.152**	**1.6**	**3.12**	**4.85**	**39**	**39.3**
	Equiv. Refl.: (4,-5,4), (-5,1,4)								
**Quartz**	**(-1,5,4)**	**8.101**	**70.09**	**0.881**	**1.6**	**3.12**	**4.86**	**39**	**39.3**
	Equiv. Refl.: (-1,-4,-4)								
**Quartz**	**(5,-4,4)**	**8.101**	**70.09**	**0.879**	**1.6**	**3.12**	**4.85**	**39**	**39.3**
**Quartz**	**(-4,5,-4)**	**8.101**	**70.09**	**0.878**	**1.6**	**3.12**	**4.85**	**39**	**39.3**
	Equiv. Refl.: (-4,-1,4)								
**Quartz**	**(5,-1,-4)**	**8.101**	**70.09**	**0.877**	**1.6**	**3.12**	**4.85**	**39**	**39.3**
**Sapph**	**(3,3,6)**	**8.325**	**75.07**	**0.677**	**1.4**	**2.297**	**3.2**	**28.71**	**28.89**
	Equiv. Refl.: (3,3,-6), (3,-6,6), (3,-6,-6), (6,-3,6), (6,-3,-6), (-3,6,6), (-3,6,-6), (-3,-3,6), (-3,-3,-6) (-6,3,6), (-6,3,-6)								
**LiNbO3**	**(2,3,-13)**	**8.398**	**77.09**	**0.62**	**2.7**	**1.975**	**5.25**	**24.69**	**25.24**
	Equiv. Refl.: (2,-5,13), (3,2,13), (3,-5,-13), (5,-2,13), (5,-3,-13), (-2,5,-13), (-2,-3,13), (-3,5,13), (-3,-2,-13) (-5,2,-13), (-5,3,13)								

**Quartz**	**(6,-1,-1)**	**8.193**	**71.97**	**0.289**	**0.6**	**2.805**	**1.63**	**35.06**	**35.1**
	Equiv. Refl.: (6,-5,1), (-1,6,1), (-1,-5,-1), (-5,6,-1), (-5,-1,1)								
**Sapph**	**(1,5,2)**	**8.43**	**78.09**	**0.226**	**0.9**	**1.817**	**1.55**	**22.72**	**22.77**
	Equiv. Refl.: (1,-6,-2), (5,1,-2), (5,-6,2), (6,-1,-2), (6,-5,2), (-1,6,2), (-1,-5,-2), (-5,6,-2), (-5,-1,2) (-6,1,2), (-6,5,-2)								
**LiNbO3**	**(3,3,9)**	**8.27**	**73.72**	**0.193**	**1.4**	**2.516**	**3.42**	**31.46**	**31.64**
	Equiv. Refl.: (3,3,-9), (3,-6,9), (3,-6,-9), (6,-3,9), (6,-3,-9), (-3,6,9), (-3,6,-9), (-3,-3,9), (-3,-3,-9) (-6,3,9), (-6,3,-9)								
**Quartz**	**(1,5,1)**	**8.193**	**71.97**	**0.121**	**0.6**	**2.805**	**1.63**	**35.06**	**35.1**
	Equiv. Refl.: (1,-6,-1), (5,1,-1), (5,-6,1), (-6,1,1), (-6,5,-1)								

Ei =	9.572	keV							
								ΔEg	ΔEt
Cryst	Refl	EB	ΘB	∫ IR dΘ	Width	Ei cotΘ	ΔE	2m, 50µm	2m, 50µm
	(h,k,l)	[keV]	[°]	[µrad]	[µrad]	[meV/µrad]	[meV]	[meV]	[meV]
Ge	(5,5,5)	9.49	82.48	23.7964	24	1.2631	30.31	15.79	34.18
	Equiv. Refl.: (1,7,5), (5,1,7), (1,5,7), (5,7,1), (7,1,5), (7,5,1)								
Ge	(0,6,6)	9.298	76.26	21.2518	19.85	2.3409	46.47	29.26	54.92
	Equiv. Refl.: (2,2,8), (2,8,2), (6,0,6), (6,6,0), (8,2,2)								
Si	(0,0,8)	9.132	72.55	10.9607	9.27	3.0086	27.9	37.61	46.83
	Equiv. Refl.: (0,8,0), (8,0,0)								
Si	(3,3,7)	9.343	77.45	9.2372	8.5	2.1316	18.11	26.65	32.22
	Equiv. Refl.: (3,7,3), (7,3,3)								
LiNbO3	(3,4,-10)	9.567	88.22	182.578	167.4	0.298	49.86	3.72	50
	Equiv. Refl.: (-3,7,-10), (-4,-3,-10), (-7,3,-10)								
LiNbO3	(4,-7,-10)	9.567	88.22	182.577	167.4	0.298	49.86	3.72	50
	Equiv. Refl.: (7,-4,-10)								
LiNbO3	(3,-7,10)	9.567	88.22	175.698	167.4	0.298	49.86	3.72	50
	Equiv. Refl.: (7,-3,10)								
LiNbO3	(4,3,10)	9.567	88.22	175.697	167.4	0.298	49.86	3.72	50
	Equiv. Refl.: (-3,-4,10)								
LiNbO3	(-4,7,10)	9.567	88.22	175.696	167.4	0.298	49.86	3.72	50
	Equiv. Refl.: (-7,4,10)								
Quartz	(7,-1,0)	9.554	86.5	35.369	30.6	0.586	17.94	7.33	19.38
	Equiv. Refl.: (7,-6,0), (-1,7,0), (-1,-6,0), (-6,7,0), (-6,-1,0)								
Quartz	(1,6,0)	9.554	86.5	35.013	30.6	0.586	17.94	7.33	19.38
	Equiv. Refl.: (1,-7,0), (6,1,0), (6,-7,0), (-7,1,0), (-7,6,0)								
LiNbO3	(0,-5,14)	9.356	77.8	27.909	24.9	2.07	51.46	25.87	57.6
	Equiv. Refl.: (5,0,14), (-5,5,14)								
LiNbO3	(0,5,-14)	9.356	77.8	25.108	24.9	2.07	51.46	25.87	57.6
	Equiv. Refl.: (-5,0,-14)								
LiNbO3	(5,-5,-14)	9.356	77.8	25.107	24.9	2.07	51.46	25.87	57.6
LiNbO3	(2,2,-18)	9.38	78.52	24.109	22.7	1.945	44.24	24.31	50.48
	Equiv. Refl.: (2,-4,-18), (4,-2,-18), (-2,4,-18), (-2,-2,-18), (-4,2,-18)								
LiNbO3	(2,3,-16)	9.377	78.41	24.016	22.2	1.962	43.48	24.53	49.92
	Equiv. Refl.: (-2,5,-16), (-3,-2,-16), (-5,2,-16)								
LiNbO3	(3,-5,-16)	9.377	78.41	24.015	22.2	1.962	43.48	24.53	49.92
	Equiv. Refl.: (5,-3,-16)								
LiNbO3	(2,2,18)	9.38	78.52	23.169	22.7	1.945	44.24	24.31	50.48
	Equiv. Refl.: (2,-4,18), (4,-2,18), (-2,4,18), (-2,-2,18), (-4,2,18)								
LiNbO3	(2,-5,16)	9.377	78.41	21.887	22.2	1.962	43.48	24.53	49.92
	Equiv. Refl.: (3,2,16), (5,-2,16), (-2,-3,16), (-3,5,16), (-5,3,16)								
LiNbO3	(0,-3,18)	9.066	71.28	21.836	19.3	3.243	62.56	40.53	74.54
	Equiv. Refl.: (3,0,18), (-3,3,18)								
LiNbO3	(0,3,18)	9.066	71.28	21.61	19.1	3.243	61.89	40.53	73.98
	Equiv. Refl.: (3,-3,18), (-3,0,18)								
LiNbO3	(1,6,-2)	9.162	73.17	21.016	19.3	2.896	55.92	36.2	66.62
	Equiv. Refl.: (6,-7,-2), (7,-6,-2), (-1,7,-2), (-6,-1,-2), (-7,1,-2)								
LiNbO3	(0,2,-20)	9.366	78.09	20.926	19.9	2.019	40.11	25.24	47.39
	Equiv. Refl.: (2,-2,-20), (-2,0,-20)								
LiNbO3	(1,-7,2)	9.162	73.17	20.465	19.3	2.896	55.92	36.2	66.62
	Equiv. Refl.: (6,1,2), (7,-1,2), (-1,-6,2), (-6,7,2), (-7,6,2)								

Ei =	9.572	keV						ΔEg	ΔEt
Cryst	Refl	EB	ΘB	∫ IR dΘ	Width	Ei cotΘ	ΔE	2m, 50μm	2m, 50μm
	(h,k,l)	[keV]	[°]	[μrad]	[μrad]	[meV/μrad]	[meV]	[meV]	[meV]
**LiNbO3**	**(0,3,-18)**	**9.066**	**71.28**	**20.195**	**19.3**	**3.243**	**62.56**	**40.53**	**74.54**
	Equiv. Refl.: (3,-3,-18), (-3,0,-18)								
**LiNbO3**	**(0,-3,-18)**	**9.066**	**71.28**	**19.903**	**19.1**	**3.243**	**61.89**	**40.53**	**73.98**
	Equiv. Refl.: (3,0,-18), (-3,3,-18)								
**LiNbO3**	**(0,-2,20)**	**9.366**	**78.09**	**19.582**	**19.9**	**2.019**	**40.11**	**25.24**	**47.39**
	Equiv. Refl.: (2,0,20), (-2,2,20)								
**LiNbO3**	**(3,4,8)**	**9.183**	**73.62**	**18.97**	**17.4**	**2.814**	**48.88**	**35.17**	**60.21**
	Equiv. Refl.: (4,-7,8), (7,-4,8), (-3,7,8), (-4,-3,8), (-7,3,8)								
**LiNbO3**	**(1,6,4)**	**9.292**	**76.1**	**17.564**	**16.9**	**2.368**	**39.95**	**29.6**	**49.72**
	Equiv. Refl.: (6,-7,4), (7,-6,4), (-1,7,4), (-6,-1,4), (-7,1,4)								
**LiNbO3**	**(3,-7,-8)**	**9.183**	**73.62**	**17.511**	**17.4**	**2.814**	**48.87**	**35.17**	**60.21**
	Equiv. Refl.: (4,3,-8), (7,-3,-8), (-3,-4,-8), (-4,7,-8), (-7,4,-8)								
**LiNbO3**	**(0,-1,-20)**	**9.051**	**71.01**	**16.76**	**15.8**	**3.294**	**52.19**	**41.18**	**66.48**
	Equiv. Refl.: (1,0,-20), (-1,1,-20)								
**LiNbO3**	**(0,1,20)**	**9.051**	**71.01**	**16.738**	**15.8**	**3.294**	**52.19**	**41.18**	**66.48**
	Equiv. Refl.: (1,-1,20), (-1,0,20)								
**LiNbO3**	**(1,-7,-4)**	**9.292**	**76.1**	**16.688**	**16.9**	**2.368**	**39.95**	**29.6**	**49.72**
	Equiv. Refl.: (6,1,-4), (7,-1,-4), (-1,-6,-4), (-6,7,-4), (-7,6,-4)								
**LiNbO3**	**(2,-7,-6)**	**9.089**	**71.71**	**16.468**	**15.7**	**3.163**	**49.51**	**39.54**	**63.36**
	Equiv. Refl.: (5,2,-6), (7,-2,-6), (-2,-5,-6), (-5,7,-6), (-7,5,-6)								
**LiNbO3**	**(2,5,6)**	**9.089**	**71.71**	**16.37**	**15.7**	**3.163**	**49.51**	**39.54**	**63.36**
	Equiv. Refl.: (5,-7,6), (7,-5,6), (-2,7,6), (-5,-2,6), (-7,2,6)								
**LiNbO3**	**(3,3,-12)**	**9**	**70.09**	**16.166**	**14.9**	**3.467**	**51.73**	**43.33**	**67.48**
	Equiv. Refl.: (-3,6,-12), (-3,-3,-12), (-6,3,-12)								
**LiNbO3**	**(3,-6,-12)**	**9**	**70.09**	**16.165**	**14.9**	**3.467**	**51.73**	**43.33**	**67.48**
	Equiv. Refl.: (6,-3,-12)								
**Sapph**	**(2,3,14)**	**9.362**	**77.98**	**16.085**	**13.8**	**2.037**	**28.03**	**25.47**	**37.87**
	Equiv. Refl.: (2,-5,-14), (3,2,-14), (3,-5,14), (5,-2,-14), (5,-3,14), (-2,5,14), (-2,-3,-14), (-3,5,-14), (-3,-2,14) (-5,2,14), (-5,3,-14)								
**LiNbO3**	**(2,5,-6)**	**9.089**	**71.71**	**16.049**	**15.2**	**3.163**	**48.14**	**39.54**	**62.3**
	Equiv. Refl.: (-2,7,-6), (-5,-2,-6), (-7,2,-6)								
**LiNbO3**	**(5,-7,-6)**	**9.089**	**71.71**	**16.048**	**15.2**	**3.163**	**48.14**	**39.54**	**62.3**
	Equiv. Refl.: (7,-5,-6)								
**Sapph**	**(0,6,0)**	**9.027**	**70.56**	**15.747**	**13**	**3.378**	**43.86**	**42.22**	**60.88**
	Equiv. Refl.: (0,-6,0), (6,0,0), (6,-6,0), (-6,0,0), (-6,6,0)								
**LiNbO3**	**(2,-7,6)**	**9.089**	**71.71**	**15.718**	**15.2**	**3.163**	**48.14**	**39.54**	**62.3**
	Equiv. Refl.: (5,2,6), (7,-2,6), (-2,-5,6), (-5,7,6), (-7,5,6)								
**LiNbO3**	**(0,-4,16)**	**9.062**	**71.22**	**15.656**	**14.6**	**3.255**	**47.38**	**40.69**	**62.45**
	Equiv. Refl.: (4,0,16), (-4,4,16)								
**LiNbO3**	**(3,3,12)**	**9**	**70.09**	**15.271**	**14.9**	**3.467**	**51.73**	**43.33**	**67.48**
	Equiv. Refl.: (3,-6,12), (6,-3,12), (-3,-3,12)								
**LiNbO3**	**(-3,6,12)**	**9**	**70.09**	**15.27**	**14.9**	**3.467**	**51.73**	**43.33**	**67.48**
	Equiv. Refl.: (-6,3,12)								
**Quartz**	**(7,-3,3)**	**9.507**	**83.32**	**14.943**	**13.1**	**1.12**	**14.67**	**14.01**	**20.28**
	Equiv. Refl.: (7,-4,-3), (-3,7,-3)								
**Quartz**	**(-3,-4,3)**	**9.507**	**83.32**	**14.941**	**13.1**	**1.12**	**14.67**	**14.01**	**20.28**
**Quartz**	**(-4,7,3)**	**9.507**	**83.32**	**14.934**	**13.1**	**1.12**	**14.66**	**14.01**	**20.28**
**Quartz**	**(-4,-3,-3)**	**9.507**	**83.32**	**14.933**	**13.1**	**1.12**	**14.66**	**14.01**	**20.28**
**Quartz**	**(-7,3,-3)**	**9.507**	**83.32**	**14.75**	**13.1**	**1.12**	**14.67**	**14.01**	**20.28**
	Equiv. Refl.: (-7,4,3)								
**Quartz**	**(3,-7,3)**	**9.507**	**83.32**	**14.749**	**13.1**	**1.12**	**14.67**	**14.01**	**20.28**

Ei =	9.572	keV						ΔEg	ΔEt
Cryst	Refl	EB	ΘB	∫ IR dΘ	Width	Ei cotΘ	ΔE	2m, 50μm	2m, 50μm
	(h,k,l)	[keV]	[°]	[μrad]	[μrad]	[meV/μrad]	[meV]	[meV]	[meV]
**Quartz**	**(3,4,-3)**	**9.507**	**83.32**	**14.748**	**13.1**	**1.12**	**14.67**	**14.01**	**20.28**
**Quartz**	**(4,-7,-3)**	**9.507**	**83.32**	**14.74**	**13.1**	**1.12**	**14.66**	**14.01**	**20.28**
**Quartz**	**(4,3,3)**	**9.507**	**83.32**	**14.739**	**13.1**	**1.12**	**14.66**	**14.01**	**20.28**
**LiNbO3**	**(0,4,-16)**	**9.062**	**71.22**	**14.603**	**14.6**	**3.255**	**47.38**	**40.69**	**62.45**
	Equiv. Refl.: (4,-4,-16), (-4,0,-16)								
**Quartz**	**(-3,6,-5)**	**9.498**	**82.86**	**13.776**	**12**	**1.199**	**14.4**	**14.99**	**20.79**
	Equiv. Refl.: (-3,-3,5)								
**Quartz**	**(6,-3,5)**	**9.498**	**82.86**	**13.768**	**12**	**1.199**	**14.4**	**14.99**	**20.79**
**Quartz**	**(6,-3,-5)**	**9.498**	**82.86**	**13.767**	**12**	**1.199**	**14.4**	**14.99**	**20.78**
**Quartz**	**(-3,-3,-5)**	**9.498**	**82.86**	**13.765**	**12**	**1.199**	**14.39**	**14.99**	**20.78**
**Quartz**	**(-3,6,5)**	**9.498**	**82.86**	**13.762**	**12**	**1.199**	**14.39**	**14.99**	**20.78**
**Quartz**	**(6,-6,3)**	**9.395**	**78.96**	**13.666**	**11.4**	**1.868**	**21.22**	**23.35**	**31.55**
	Equiv. Refl.: (-6,6,-3)								
**Quartz**	**(6,0,-3)**	**9.395**	**78.96**	**13.665**	**11.4**	**1.868**	**21.22**	**23.35**	**31.55**
	Equiv. Refl.: (-6,0,3)								
**Quartz**	**(0,6,3)**	**9.395**	**78.96**	**13.658**	**11.4**	**1.868**	**21.21**	**23.35**	**31.54**
	Equiv. Refl.: (0,-6,-3)								
**Quartz**	**(3,3,-5)**	**9.498**	**82.86**	**13.382**	**12**	**1.199**	**14.4**	**14.99**	**20.79**
	Equiv. Refl.: (3,-6,5)								
**Quartz**	**(-6,3,-5)**	**9.498**	**82.86**	**13.374**	**12**	**1.199**	**14.4**	**14.99**	**20.79**
**Quartz**	**(-6,3,5)**	**9.498**	**82.86**	**13.373**	**12**	**1.199**	**14.4**	**14.99**	**20.78**
**Quartz**	**(3,3,5)**	**9.498**	**82.86**	**13.372**	**12**	**1.199**	**14.39**	**14.99**	**20.78**
**Quartz**	**(3,-6,-5)**	**9.498**	**82.86**	**13.369**	**12**	**1.199**	**14.39**	**14.99**	**20.78**
**Sapph**	**(2,4,10)**	**9.282**	**75.86**	**11.88**	**10**	**2.412**	**24.14**	**30.15**	**38.63**
	Equiv. Refl.: (2,-6,-10), (4,2,-10), (4,-6,10), (6,-2,-10), (6,-4,10), (-2,6,10), (-2,-4,-10), (-4,6,-10), (-4,-2,10) (-6,2,10), (-6,4,-10)								
**Quartz**	**(7,-2,2)**	**9.384**	**78.61**	**11.609**	**9.5**	**1.928**	**18.39**	**24.1**	**30.31**
	Equiv. Refl.: (7,-5,-2)								
**Quartz**	**(-2,7,-2)**	**9.384**	**78.61**	**11.607**	**9.5**	**1.928**	**18.39**	**24.1**	**30.31**
	Equiv. Refl.: (-2,-5,2)								
**Quartz**	**(-5,7,2)**	**9.384**	**78.61**	**11.606**	**9.5**	**1.928**	**18.39**	**24.1**	**30.31**
	Equiv. Refl.: (-5,-2,-2)								
**Quartz**	**(-7,2,-2)**	**9.384**	**78.61**	**11.275**	**9.5**	**1.928**	**18.39**	**24.1**	**30.31**
	Equiv. Refl.: (-7,5,2)								
**Quartz**	**(2,-7,2)**	**9.384**	**78.61**	**11.274**	**9.5**	**1.928**	**18.39**	**24.1**	**30.31**
**Quartz**	**(2,5,-2)**	**9.384**	**78.61**	**11.273**	**9.5**	**1.928**	**18.39**	**24.1**	**30.31**
**Quartz**	**(5,2,2)**	**9.384**	**78.61**	**11.272**	**9.5**	**1.928**	**18.39**	**24.1**	**30.31**
	Equiv. Refl.: (5,-7,-2)								
**Quartz**	**(-1,1,8)**	**9.291**	**76.09**	**10.931**	**9**	**2.371**	**21.44**	**29.64**	**36.59**
**Quartz**	**(1,-1,-8)**	**9.291**	**76.09**	**10.93**	**9**	**2.371**	**21.44**	**29.64**	**36.59**
	Equiv. Refl.: (-1,0,-8)								
**Quartz**	**(1,0,8)**	**9.291**	**76.09**	**10.929**	**9**	**2.371**	**21.44**	**29.64**	**36.59**
**Quartz**	**(0,1,-8)**	**9.291**	**76.09**	**10.924**	**9**	**2.371**	**21.43**	**29.64**	**36.58**
	Equiv. Refl.: (0,-1,8)								
**Quartz**	**(2,5,1)**	**9.171**	**73.35**	**10.613**	**8.9**	**2.862**	**25.48**	**35.77**	**43.92**
	Equiv. Refl.: (2,-7,-1), (5,2,-1), (5,-7,1), (-7,2,1), (-7,5,-1)								
**Quartz**	**(7,-2,-1)**	**9.171**	**73.35**	**10.525**	**8.9**	**2.862**	**25.48**	**35.77**	**43.92**
	Equiv. Refl.: (7,-5,1), (-5,7,-1), (-5,-2,1)								
**Quartz**	**(-2,7,1)**	**9.171**	**73.35**	**10.524**	**8.9**	**2.862**	**25.48**	**35.77**	**43.92**
	Equiv. Refl.: (-2,-5,-1)								

Ei =	9.572	keV						ΔEg	ΔEt
Cryst	Refl	EB	ΘB	∫ IR dΘ	Width	Ei cotΘ	ΔE	2m, 50µm	2m, 50µm
	(h,k,l)	[keV]	[°]	[µrad]	[µrad]	[meV/µrad]	[meV]	[meV]	[meV]
Quartz	(3,0,-7)	9.142	72.76	10.409	8.4	2.971	24.96	37.14	44.75
Quartz	(0,3,7)	9.142	72.76	10.408	8.4	2.971	24.96	37.14	44.75
	Equiv. Refl.: (0,-3,-7), (3,-3,7), (-3,0,7)								
Quartz	(-3,3,-7)	9.142	72.76	10.407	8.4	2.971	24.96	37.14	44.75
LiNbO3	(2,5,9)	9.571	89.13	10.302	33.9	0.145	4.93	1.82	5.25
	Equiv. Refl.: (2,5,-9), (2,-7,9), (2,-7,-9), (5,2,9), (5,2,-9), (5,-7,9), (5,-7,-9), (7,-2,9), (7,-2,-9) (7,-5,9), (7,-5,-9), (-2,7,9), (-2,7,-9), (-2,-5,9), (-2,-5,-9), (-5,7,9), (-5,7,-9), (-5,-2,9) (-5,-2,-9), (-7,2,9), (-7,2,-9), (-7,5,9), (-7,5,-9)								
Sapph	(2,5,0)	9.395	78.97	9.659	8.7	1.866	16.16	23.33	28.38
	Equiv. Refl.: (2,-7,0), (5,2,0), (5,-7,0), (7,-2,0), (7,-5,0), (-2,7,0), (-2,-5,0), (-5,7,0), (-5,-2,0) (-7,2,0), (-7,5,0)								
Sapph	(2,5,3)	9.504	83.15	9.349	9.4	1.151	10.81	14.38	17.99
	Equiv. Refl.: (2,5,-3), (2,-7,3), (2,-7,-3), (5,2,3), (5,2,-3), (5,-7,3), (5,-7,-3), (7,-2,3), (7,-2,-3) (7,-5,3), (7,-5,-3), (-2,7,3), (-2,7,-3), (-2,-5,3), (-2,-5,-3), (-5,7,3), (-5,7,-3), (-5,-2,3) (-5,-2,-3), (-7,2,3), (-7,2,-3), (-7,5,3), (-7,5,-3)								
Sapph	(2,4,-11)	9.536	85.03	8.731	9.4	0.832	7.79	10.4	12.99
	Equiv. Refl.: (2,-6,11), (4,2,11), (4,-6,-11), (6,-2,11), (6,-4,-11), (-2,6,-11), (-2,-4,11), (-4,6,11), (-4,-2,-11) (-6,2,-11), (-6,4,11)								
Quartz	(7,-2,-2)	9.384	78.61	8.361	7.5	1.928	14.37	24.1	28.06
	Equiv. Refl.: (7,-5,2)								
Quartz	(-2,7,2)	9.384	78.61	8.36	7.5	1.928	14.37	24.1	28.06
	Equiv. Refl.: (-2,-5,-2)								
Quartz	(-5,7,-2)	9.384	78.61	8.359	7.5	1.928	14.37	24.1	28.05
	Equiv. Refl.: (-5,-2,2)								
Quartz	(-7,2,2)	9.384	78.61	8.343	7.5	1.928	14.37	24.1	28.06
Quartz	(2,-7,-2)	9.384	78.61	8.342	7.5	1.928	14.37	24.1	28.06
	Equiv. Refl.: (-7,5,-2)								
Quartz	(2,5,2)	9.384	78.61	8.341	7.5	1.928	14.37	24.1	28.05
	Equiv. Refl.: (5,2,-2), (5,-7,2)								
Quartz	(3,4,2)	9.155	73.02	8.332	6.8	2.923	19.93	36.54	41.62
	Equiv. Refl.: (3,-7,-2), (-7,3,2), (-7,4,-2)								
Quartz	(4,3,-2)	9.155	73.02	8.33	6.8	2.923	19.92	36.54	41.62
	Equiv. Refl.: (4,-7,2)								
Quartz	(-2,1,-8)	9.517	83.85	8.211	7.3	1.032	7.56	12.9	14.95
Quartz	(-2,1,8)	9.517	83.85	8.207	7.3	1.032	7.55	12.9	14.95
Quartz	(1,-2,-8)	9.517	83.85	8.196	7.3	1.032	7.54	12.9	14.95
Quartz	(1,1,8)	9.517	83.85	8.193	7.3	1.032	7.54	12.9	14.94
Quartz	(1,1,-8)	9.517	83.85	8.182	7.3	1.032	7.53	12.9	14.94
Quartz	(1,-2,8)	9.517	83.85	8.177	7.3	1.032	7.53	12.9	14.94
Quartz	(5,0,5)	9.272	75.61	8.164	6.9	2.457	16.93	30.71	35.06
	Equiv. Refl.: (5,-5,-5), (-5,0,-5), (-5,5,5)								
Quartz	(0,5,-5)	9.272	75.61	8.163	6.9	2.457	16.93	30.71	35.06
	Equiv. Refl.: (0,-5,5)								
Sapph	(3,4,2)	9.201	73.99	7.961	7.1	2.747	19.54	34.33	39.5
	Equiv. Refl.: (3,-7,-2), (4,3,-2), (4,-7,2), (7,-3,-2), (7,-4,2), (-3,7,2), (-3,-4,-2), (-4,7,-2), (-4,-3,2) (-7,3,2), (-7,4,-2)								
Quartz	(7,-3,-2)	9.155	73.02	7.938	6.8	2.923	19.93	36.54	41.62
	Equiv. Refl.: (7,-4,2), (-3,7,2), (-3,-4,-2)								
Quartz	(-4,7,-2)	9.155	73.02	7.936	6.8	2.923	19.92	36.54	41.62
	Equiv. Refl.: (-4,-3,2)								

Ei = Cryst	9.572 Refl (h,k,l)	keV EB [keV]	ΘB [°]	∫ IR dΘ [µrad]	Width [µrad]	Ei cotΘ [meV/µrad]	ΔE [meV]	ΔEg 2m, 50µm [meV]	ΔEt 2m, 50µm [meV]
**Quartz**	**(-6,1,4)**	**9.32**	**76.82**	**7.849**	**6.9**	**2.242**	**15.41**	**28.03**	**31.99**
	Equiv. Refl.: (-6,5,-4)								
**Quartz**	**(1,5,4)**	**9.32**	**76.82**	**7.846**	**6.9**	**2.242**	**15.41**	**28.03**	**31.98**
	Equiv. Refl.: (1,-6,-4)								
**Quartz**	**(5,1,-4)**	**9.32**	**76.82**	**7.84**	**6.9**	**2.242**	**15.4**	**28.03**	**31.98**
	Equiv. Refl.: (5,-6,4)								
**Quartz**	**(6,-5,4)**	**9.32**	**76.82**	**7.742**	**6.9**	**2.242**	**15.42**	**28.03**	**31.99**
**Quartz**	**(6,-1,-4)**	**9.32**	**76.82**	**7.741**	**6.9**	**2.242**	**15.41**	**28.03**	**31.99**
**Quartz**	**(-1,6,4)**	**9.32**	**76.82**	**7.739**	**6.9**	**2.242**	**15.41**	**28.03**	**31.98**
	Equiv. Refl.: (-1,-5,-4)								
**Quartz**	**(-5,6,-4)**	**9.32**	**76.82**	**7.733**	**6.9**	**2.242**	**15.4**	**28.03**	**31.98**
	Equiv. Refl.: (-5,-1,4)								
**Quartz**	**(4,0,-6)**	**9.018**	**70.42**	**7.629**	**6.3**	**3.406**	**21.61**	**42.57**	**47.74**
	Equiv. Refl.: (4,-4,6), (-4,0,6), (-4,4,-6)								
**Quartz**	**(0,4,6)**	**9.018**	**70.42**	**7.626**	**6.3**	**3.406**	**21.6**	**42.57**	**47.74**
	Equiv. Refl.: (0,-4,-6)								
**Quartz**	**(2,-1,8)**	**9.517**	**83.85**	**6.634**	**7.3**	**1.032**	**7.56**	**12.9**	**14.95**
**Quartz**	**(2,-1,-8)**	**9.517**	**83.85**	**6.631**	**7.3**	**1.032**	**7.55**	**12.9**	**14.95**
**Quartz**	**(-1,2,8)**	**9.517**	**83.85**	**6.621**	**7.3**	**1.032**	**7.54**	**12.9**	**14.95**
**Quartz**	**(-1,-1,-8)**	**9.517**	**83.85**	**6.618**	**7.3**	**1.032**	**7.54**	**12.9**	**14.94**
**Quartz**	**(-1,-1,8)**	**9.517**	**83.85**	**6.608**	**7.3**	**1.032**	**7.53**	**12.9**	**14.94**
**Quartz**	**(-1,2,-8)**	**9.517**	**83.85**	**6.603**	**7.3**	**1.032**	**7.53**	**12.9**	**14.94**
**Quartz**	**(6,0,2)**	**9.038**	**70.77**	**6.217**	**5.4**	**3.339**	**18.02**	**41.74**	**45.46**
	Equiv. Refl.: (-6,0,-2)								
**Quartz**	**(6,-6,-2)**	**9.038**	**70.77**	**6.216**	**5.4**	**3.339**	**18.02**	**41.74**	**45.46**
	Equiv. Refl.: (-6,6,2)								
**Quartz**	**(0,6,-2)**	**9.038**	**70.77**	**6.214**	**5.4**	**3.339**	**18.02**	**41.74**	**45.46**
	Equiv. Refl.: (0,-6,2)								
**Sapph**	**(1,3,16)**	**9.367**	**78.12**	**5.779**	**5.6**	**2.013**	**11.2**	**25.17**	**27.55**
	Equiv. Refl.: (1,-4,-16), (3,1,-16), (3,-4,16), (4,-1,-16), (4,-3,16), (-1,4,16), (-1,-3,-16), (-3,4,-16), (-3,-1,16) (-4,1,16), (-4,3,-16)								
**Quartz**	**(-7,3,3)**	**9.507**	**83.32**	**5.581**	**5.5**	**1.12**	**6.12**	**14.01**	**15.29**
	Equiv. Refl.: (-7,4,-3)								
**Quartz**	**(4,3,-3)**	**9.507**	**83.32**	**5.576**	**5.5**	**1.12**	**6.12**	**14.01**	**15.28**
**Quartz**	**(4,-7,3)**	**9.507**	**83.32**	**5.575**	**5.5**	**1.12**	**6.12**	**14.01**	**15.28**
**Quartz**	**(3,-7,-3)**	**9.507**	**83.32**	**5.574**	**5.5**	**1.12**	**6.12**	**14.01**	**15.28**
**Quartz**	**(3,4,3)**	**9.507**	**83.32**	**5.573**	**5.5**	**1.12**	**6.12**	**14.01**	**15.28**
**LiNbO3**	**(1,6,-5)**	**9.388**	**78.75**	**5.318**	**7.8**	**1.903**	**14.76**	**23.79**	**28**
	Equiv. Refl.: (1,-7,5), (6,1,5), (6,-7,-5), (7,-1,5), (7,-6,-5), (-1,7,-5), (-1,-6,5), (-6,7,5), (-6,-1,-5) (-7,1,-5), (-7,6,5)								
**Quartz**	**(0,3,-7)**	**9.142**	**72.76**	**4.882**	**4.3**	**2.971**	**12.81**	**37.14**	**39.28**
	Equiv. Refl.: (0,-3,7)								
**Quartz**	**(3,0,7)**	**9.142**	**72.76**	**4.881**	**4.3**	**2.971**	**12.8**	**37.14**	**39.28**
	Equiv. Refl.: (-3,0,-7)								
**Quartz**	**(3,-3,-7)**	**9.142**	**72.76**	**4.879**	**4.3**	**2.971**	**12.8**	**37.14**	**39.28**
	Equiv. Refl.: (-3,3,7)								
**Quartz**	**(7,-4,3)**	**9.507**	**83.32**	**4.732**	**5.5**	**1.12**	**6.12**	**14.01**	**15.29**
**Quartz**	**(7,-3,-3)**	**9.507**	**83.32**	**4.731**	**5.5**	**1.12**	**6.12**	**14.01**	**15.29**
**Quartz**	**(-4,-3,3)**	**9.507**	**83.32**	**4.727**	**5.5**	**1.12**	**6.12**	**14.01**	**15.28**
**Quartz**	**(-4,7,-3)**	**9.507**	**83.32**	**4.726**	**5.5**	**1.12**	**6.12**	**14.01**	**15.28**

Ei =	9.572	keV						ΔEg	ΔEt
Cryst	Refl	EB	ΘB	∫ IR dΘ	Width	Ei cotΘ	ΔE	2m, 50μm	2m, 50μm
	(h,k,l)	[keV]	[°]	[μrad]	[μrad]	[meV/μrad]	[meV]	[meV]	[meV]
Quartz	(-3,7,3)	9.507	83.32	4.725	5.5	1.12	6.12	14.01	15.28
Quartz	(-3,-4,-3)	9.507	83.32	4.724	5.5	1.12	6.12	14.01	15.28
Quartz	(4,-2,7)	9.484	82.21	4.432	4.4	1.309	5.74	16.37	17.34
	Equiv. Refl.: (4,-2,-7)								
Quartz	(-2,-2,-7)	9.484	82.21	4.43	4.4	1.309	5.74	16.37	17.34
Quartz	(-2,4,7)	9.484	82.21	4.426	4.4	1.309	5.73	16.37	17.34
Quartz	(-2,4,-7)	9.484	82.21	4.424	4.4	1.309	5.73	16.37	17.34
	Equiv. Refl.: (-2,-2,7)								
Quartz	(6,0,3)	9.395	78.96	4.389	4.3	1.868	8	23.35	24.68
	Equiv. Refl.: (-6,0,-3)								
Quartz	(6,-6,-3)	9.395	78.96	4.388	4.3	1.868	8	23.35	24.68
	Equiv. Refl.: (-6,6,3)								
Quartz	(0,6,-3)	9.395	78.96	4.385	4.3	1.868	8	23.35	24.68
	Equiv. Refl.: (0,-6,3)								
Quartz	(0,4,-6)	9.018	70.42	4.137	3.7	3.406	12.45	42.57	44.35
	Equiv. Refl.: (0,-4,6), (4,0,6), (-4,0,-6), (-4,4,6)								
Quartz	(4,-4,-6)	9.018	70.42	4.136	3.7	3.406	12.44	42.57	44.35
Sapph	(1,5,-7)	9.018	70.41	3.935	3.7	3.406	12.75	42.58	44.45
	Equiv. Refl.: (1,-6,7), (5,1,7), (5,-6,-7), (6,-1,7), (6,-5,-7), (-1,6,-7), (-1,-5,7), (-5,6,7), (-5,-1,-7) (-6,1,-7), (-6,5,7)								
Quartz	(-4,2,-7)	9.484	82.21	3.905	4.4	1.309	5.74	16.37	17.34
Quartz	(-4,2,7)	9.484	82.21	3.904	4.4	1.309	5.74	16.37	17.34
Quartz	(2,2,7)	9.484	82.21	3.902	4.4	1.309	5.74	16.37	17.34
Quartz	(2,-4,-7)	9.484	82.21	3.898	4.4	1.309	5.73	16.37	17.34
Sapph	(3,4,-4)	9.348	77.58	3.896	4.2	2.108	8.88	26.35	27.8
	Equiv. Refl.: (3,-7,4), (4,3,4), (4,-7,-4), (7,-3,4), (7,-4,-4), (-3,7,-4), (-3,-4,4), (-4,7,4), (-4,-3,-4) (-7,3,-4), (-7,4,4), (2,2,-7), (2,-4,7)								
LiNbO3	(2,4,13)	9.377	78.42	3.881	6.4	1.961	12.65	24.52	27.59
	Equiv. Refl.: (2,-6,-13), (4,2,-13), (4,-6,13), (6,-2,-13), (6,-4,13), (-2,6,13), (-2,-4,-13), (-4,6,-13), (-4,-2,13) (-6,2,13), (-6,4,-13)								
Quartz	(-5,7,1)	9.171	73.35	3.754	3.4	2.862	9.79	35.77	37.09
	Equiv. Refl.: (-5,-2,-1)								
Quartz	(7,-2,1)	9.171	73.35	3.753	3.4	2.862	9.79	35.77	37.09
	Equiv. Refl.: (7,-5,-1), (-2,7,-1), (-2,-5,1)								
Quartz	(5,2,1)	9.171	73.35	3.688	3.4	2.862	9.79	35.77	37.09
	Equiv. Refl.: (5,-7,-1)								
Quartz	(2,5,-1)	9.171	73.35	3.687	3.4	2.862	9.79	35.77	37.09
	Equiv. Refl.: (2,-7,1), (-7,2,-1), (-7,5,1)								
LiNbO3	(1,6,1)	9.129	72.5	3.598	5.4	3.018	16.2	37.72	41.06
	Equiv. Refl.: (1,-7,-1), (6,1,-1), (6,-7,1), (7,-1,-1), (7,-6,1), (-1,7,1), (-1,-6,-1), (-6,7,-1), (-6,-1,1) (-7,1,1), (-7,6,-1)								
Quartz	(0,1,8)	9.291	76.09	3.256	3.1	2.371	7.41	29.64	30.55
	Equiv. Refl.: (0,-1,-8)								
Quartz	(-1,1,-8)	9.291	76.09	3.253	3.1	2.371	7.4	29.64	30.55
Quartz	(1,-1,8)	9.291	76.09	3.252	3.1	2.371	7.4	29.64	30.55
Quartz	(-1,0,8)	9.291	76.09	3.251	3.1	2.371	7.4	29.64	30.55
Quartz	(1,0,-8)	9.291	76.09	3.25	3.1	2.371	7.4	29.64	30.55
Sapph	(0,6,6)	9.47	81.62	1.743	2.8	1.409	3.88	17.62	18.04
	Equiv. Refl.: (0,6,-6), (0,-6,6), (0,-6,-6), (6,0,6), (6,0,-6), (6,-6,6), (6,-6,-6), (-6,0,6), (-6,0,-6) (-6,6,6), (-6,6,-6)								

Ei =	9.572	keV						ΔEg	ΔEt
Cryst	Refl	EB	ΘB	∫ IR dΘ	Width	Ei cotΘ	ΔE	2m, 50µm	2m, 50µm
	(h,k,l)	[keV]	[°]	[µrad]	[µrad]	[meV/µrad]	[meV]	[meV]	[meV]
**Sapph**	**(2,3,-13)**	**9.028**	**70.59**	**1.718**	**2.2**	**3.373**	**7.51**	**42.16**	**42.83**
	Equiv. Refl.: (2,-5,13), (3,2,13), (3,-5,-13), (5,-2,13), (5,-3,-13), (-2,5,-13), (-2,-3,13), (-3,5,13), (-3,-2,-13) (-5,2,-13), (-5,3,13)								
**Quartz**	**(7,-3,2)**	**9.155**	**73.02**	**1.683**	**2**	**2.923**	**5.97**	**36.54**	**37.03**
	Equiv. Refl.: (7,-4,-2), (-3,7,-2), (-3,-4,2), (-4,7,2), (-4,-3,-2)								
**Quartz**	**(5,-2,6)**	**9.365**	**78.05**	**1.679**	**2.2**	**2.025**	**4.43**	**25.32**	**25.7**
	Equiv. Refl.: (5,-3,-6), (-3,-2,-6)								
**Quartz**	**(-3,5,6)**	**9.365**	**78.05**	**1.678**	**2.2**	**2.025**	**4.42**	**25.32**	**25.7**
**Quartz**	**(-2,5,-6)**	**9.365**	**78.05**	**1.675**	**2.2**	**2.025**	**4.42**	**25.32**	**25.7**
	Equiv. Refl.: (-2,-3,6)								
**Quartz**	**(0,5,5)**	**9.272**	**75.61**	**1.604**	**2.1**	**2.457**	**5.17**	**30.71**	**31.14**
	Equiv. Refl.: (0,-5,-5)								
**Quartz**	**(5,0,-5)**	**9.272**	**75.61**	**1.597**	**2.1**	**2.457**	**5.15**	**30.71**	**31.14**
	Equiv. Refl.: (5,-5,5), (-5,0,5), (-5,5,-5)								
**LiNbO3**	**(1,3,-17)**	**9.106**	**72.05**	**1.594**	**3.3**	**3.1**	**10.1**	**38.75**	**40.05**
	Equiv. Refl.: (1,-4,17), (3,1,17), (3,-4,-17), (4,-1,17), (4,-3,-17), (-1,4,-17), (-1,-3,17), (-3,4,17), (-3,-1,-17) (-4,1,-17), (-4,3,17)								
**Quartz**	**(4,-7,-2)**	**9.155**	**73.02**	**1.549**	**2**	**2.923**	**5.97**	**36.54**	**37.03**
	Equiv. Refl.: (-7,3,-2), (-7,4,2)								
**Quartz**	**(3,4,-2)**	**9.155**	**73.02**	**1.548**	**2**	**2.923**	**5.97**	**36.54**	**37.03**
	Equiv. Refl.: (3,-7,2), (4,3,2)								
**Quartz**	**(-5,2,-6)**	**9.365**	**78.05**	**1.517**	**2.2**	**2.025**	**4.43**	**25.32**	**25.7**
**Quartz**	**(3,2,6)**	**9.365**	**78.05**	**1.516**	**2.2**	**2.025**	**4.43**	**25.32**	**25.7**
	Equiv. Refl.: (-5,3,6)								
**Quartz**	**(3,-5,-6)**	**9.365**	**78.05**	**1.514**	**2.2**	**2.025**	**4.42**	**25.32**	**25.7**
**Quartz**	**(2,3,-6)**	**9.365**	**78.05**	**1.512**	**2.2**	**2.025**	**4.42**	**25.32**	**25.7**
	Equiv. Refl.: (2,-5,6)								
**LiNbO3**	**(1,4,15)**	**9.252**	**75.14**	**1.496**	**3.4**	**2.54**	**8.53**	**31.75**	**32.88**
	Equiv. Refl.: (1,4,-15), (1,-5,15), (1,-5,-15), (4,1,15), (4,1,-15), (4,-5,15), (4,-5,-15), (5,-1,15), (5,-1,-15) (5,-4,15), (5,-4,-15), (-1,5,15), (-1,5,-15), (-1,-4,15), (-1,-4,-15), (-4,5,15), (-4,5,-15), (-4,-1,15) (-4,-1,-15), (-5,1,15), (-5,1,-15), (-5,4,15), (-5,4,-15)								
**LiNbO3**	**(3,4,-7)**	**9.019**	**70.42**	**1.469**	**3**	**3.404**	**10.26**	**42.55**	**43.77**
	Equiv. Refl.: (3,-7,7), (4,3,7), (4,-7,-7), (7,-3,7), (7,-4,-7), (-3,7,-7), (-3,-4,7), (-4,7,7), (-4,-3,-7) (-7,3,-7), (-7,4,7)								
**Sapph**	**(1,2,17)**	**9.038**	**70.77**	**1.338**	**1.8**	**3.34**	**6.16**	**41.75**	**42.2**
	Equiv. Refl.: (1,-3,-17), (2,1,-17), (2,-3,17), (3,-1,-17), (3,-2,17), (-1,3,17), (-1,-2,-17), (-2,3,-17), (-2,-1,17) (-3,1,17), (-3,2,-17)								
**Quartz**	**(0,6,2)**	**9.038**	**70.77**	**1.165**	**1.5**	**3.339**	**5.15**	**41.74**	**42.05**
	Equiv. Refl.: (0,-6,-2), (6,0,-2), (-6,0,2), (-6,6,-2)								
**Quartz**	**(6,-6,2)**	**9.038**	**70.77**	**1.164**	**1.5**	**3.339**	**5.15**	**41.74**	**42.05**
**Sapph**	**(1,5,8)**	**9.206**	**74.09**	**1.118**	**1.7**	**2.728**	**4.62**	**34.1**	**34.41**
	Equiv. Refl.: (1,-6,-8), (5,1,-8), (5,-6,8), (6,-1,-8), (6,-5,8), (-1,6,8), (-1,-5,-8), (-5,6,-8), (-5,-1,8) (-6,1,8), (-6,5,-8)								
**LiNbO3**	**(1,5,11)**	**9.172**	**73.39**	**1.116**	**2.7**	**2.856**	**7.76**	**35.7**	**36.53**
	Equiv. Refl.: (1,-6,-11), (5,1,-11), (5,-6,11), (6,-1,-11), (6,-5,11), (-1,6,11), (-1,-5,-11), (-5,6,-11), (-5,-1,11) (-6,1,11), (-6,5,-11)								
**LiNbO3**	**(1,2,-19)**	**9.259**	**75.3**	**1.101**	**2.8**	**2.511**	**7.1**	**31.39**	**32.18**
	Equiv. Refl.: (1,-3,19), (2,1,19), (2,-3,-19), (3,-1,19), (3,-2,-19), (-1,3,-19), (-1,-2,19), (-2,3,19), (-2,-1,-19) (-3,1,-19), (-3,2,19)								
**Quartz**	**(2,-5,-6)**	**9.365**	**78.05**	**0.672**	**1.1**	**2.025**	**2.2**	**25.32**	**25.41**

Ei =	9.572	keV						ΔEg	ΔEt
**Cryst**	**Refl**	**EB**	**ΘB**	**∫ IR dΘ**	**Width**	**Ei cotΘ**	**ΔE**	**2m, 50µm**	**2m, 50µm**
	**(h,k,l)**	**[keV]**	**[°]**	**[µrad]**	**[µrad]**	**[meV/µrad]**	**[meV]**	**[meV]**	**[meV]**
**Quartz**	**(2,3,6)**	**9.365**	**78.05**	**0.67**	**1.1**	**2.025**	**2.19**	**25.32**	**25.41**
**Quartz**	**(-5,2,6)**	**9.365**	**78.05**	**0.669**	**1.1**	**2.025**	**2.19**	**25.32**	**25.41**
	Equiv. Refl.: (-5,3,-6)								
**Quartz**	**(3,2,-6)**	**9.365**	**78.05**	**0.665**	**1.1**	**2.025**	**2.18**	**25.32**	**25.41**
**Quartz**	**(3,-5,6)**	**9.365**	**78.05**	**0.664**	**1.1**	**2.025**	**2.18**	**25.32**	**25.41**
**Sapph**	**(3,4,5)**	**9.457**	**81.11**	**0.523**	**1.3**	**1.497**	**1.93**	**18.71**	**18.81**
	Equiv. Refl.: (3,-7,-5), (4,3,-5), (4,-7,5), (7,-3,-5), (7,-4,5), (-3,7,5), (-3,-4,-5), (-4,7,-5), (-4,-3,5) (-7,3,5), (-7,4,-5)								
**Quartz**	**(-2,5,6)**	**9.365**	**78.05**	**0.485**	**1.1**	**2.025**	**2.2**	**25.32**	**25.41**
**Quartz**	**(-2,-3,-6)**	**9.365**	**78.05**	**0.483**	**1.1**	**2.025**	**2.19**	**25.32**	**25.41**
**Quartz**	**(5,-2,-6)**	**9.365**	**78.05**	**0.482**	**1.1**	**2.025**	**2.19**	**25.32**	**25.41**
	Equiv. Refl.: (5,-3,6)								
**Quartz**	**(-3,5,-6)**	**9.365**	**78.05**	**0.478**	**1.1**	**2.025**	**2.18**	**25.32**	**25.41**
	Equiv. Refl.: (-3,-2,6)								
**Quartz**	**(-1,-5,4)**	**9.32**	**76.82**	**0.339**	**0.7**	**2.242**	**1.63**	**28.03**	**28.07**
**Quartz**	**(6,-5,-4)**	**9.32**	**76.82**	**0.338**	**0.7**	**2.242**	**1.63**	**28.03**	**28.07**
	Equiv. Refl.: (-1,6,-4)								
**Quartz**	**(6,-1,4)**	**9.32**	**76.82**	**0.337**	**0.7**	**2.242**	**1.63**	**28.03**	**28.07**
**Quartz**	**(-5,6,4)**	**9.32**	**76.82**	**0.335**	**0.7**	**2.242**	**1.62**	**28.03**	**28.07**
	Equiv. Refl.: (-5,-1,-4)								
**Quartz**	**(1,5,-4)**	**9.32**	**76.82**	**0.324**	**0.7**	**2.242**	**1.63**	**28.03**	**28.07**
	Equiv. Refl.: (1,-6,4)								
**Quartz**	**(-6,5,4)**	**9.32**	**76.82**	**0.323**	**0.7**	**2.242**	**1.63**	**28.03**	**28.07**
**Quartz**	**(7,-2,0)**	**9.099**	**71.91**	**0.322**	**0.6**	**3.127**	**1.94**	**39.08**	**39.13**
	Equiv. Refl.: (7,-5,0), (-2,7,0), (-2,-5,0), (-5,7,0), (-5,-2,0), (-6,1,-4)								
**Quartz**	**(5,1,4)**	**9.32**	**76.82**	**0.32**	**0.7**	**2.242**	**1.62**	**28.03**	**28.07**
	Equiv. Refl.: (5,-6,-4)								
**Sapph**	**(3,4,-1)**	**9.163**	**73.2**	**0.315**	**0.7**	**2.89**	**2.16**	**36.12**	**36.19**
	Equiv. Refl.: (3,-7,1), (4,3,1), (4,-7,-1), (7,-3,1), (7,-4,-1), (-3,7,-1), (-3,-4,1), (-4,7,1), (-4,-3,-1) (-7,3,-1), (-7,4,1)								
**Quartz**	**(2,5,0)**	**9.099**	**71.91**	**0.262**	**0.6**	**3.127**	**1.94**	**39.08**	**39.13**
	Equiv. Refl.: (2,-7,0), (5,2,0), (5,-7,0), (-7,2,0), (-7,5,0)								

Ei =	10.367	keV						ΔEg	ΔEt
**Cryst**	**Refl**	**EB**	**ΘB**	**∫IR dΘ**	**Width**	**Ei cotΘ**	**ΔE**	**2m, 50μm**	**2m, 50μm**
	**(h,k,l)**	**[keV]**	**[°]**	**[μrad]**	**[μrad]**	**[meV/μrad]**	**[meV]**	**[meV]**	**[meV]**
**Ge**	**(4,6,6)**	**10.279**	**82.54**	**23.7**	**22.8**	**1.357**	**30.9**	**16.96**	**35.25**
**Si**	**(0,4,8)**	**10.209**	**80**	**12.5**	**10.7**	**1.829**	**19.61**	**22.86**	**30.12**
**Ge**	**(0,4,8)**	**9.801**	**70.98**	**11.5**	**10.6**	**3.574**	**37.74**	**44.67**	**58.48**
**Ge**	**(1,1,9)**	**9.983**	**74.36**	**8.5**	**8.5**	**2.903**	**24.72**	**36.29**	**43.9**
	Equiv. Refl.: (3,5,7)								
**Si**	**(5,5,5)**	**9.885**	**72.46**	**5.5**	**4.8**	**3.276**	**15.74**	**40.95**	**43.87**
	Equiv. Refl.: (1,5,7)								
**Quartz**	**(0,0,9)**	**10.323**	**84.73**	**33.9**	**27.7**	**0.956**	**26.47**	**11.95**	**29.05**
	Equiv. Refl.: (0,0,-9)								
**Sapph**	**(3,4,-10)**	**10.321**	**84.6**	**32.9**	**27.5**	**0.98**	**26.94**	**12.25**	**29.6**
	Equiv. Refl.: (3,-7,10), (4,3,10), (4,-7,-10), (7,-3,10), (7,-4,-10), (-3,7,-10), (-3,-4,10), (-4,7,10), (-4,-3,-10) (-7,3,-10), (-7,4,10)								
**Sapph**	**(1,1,21)**	**10.356**	**87.4**	**30.7**	**28.1**	**0.472**	**13.27**	**5.89**	**14.52**
	Equiv. Refl.: (1,1,-21), (1,-2,21), (1,-2,-21), (2,-1,21), (2,-1,-21), (-1,2,21), (-1,2,-21), (-1,-1,21), (-1,-1,-21) (-2,1,21), (-2,1,-21)								
**LiNbO3**	**(2,4,16)**	**10.263**	**81.88**	**29.2**	**26.9**	**1.48**	**39.8**	**18.5**	**43.89**
	Equiv. Refl.: (4,-6,16), (6,-4,16), (-2,6,16), (-4,-2,16), (-6,2,16)								
**LiNbO3**	**(1,-4,20)**	**10.253**	**81.49**	**29.1**	**26.9**	**1.551**	**41.8**	**19.39**	**46.08**
	Equiv. Refl.: (3,1,20), (4,-1,20), (-1,-3,20), (-3,4,20), (-4,3,20)								
**LiNbO3**	**(1,3,-20)**	**10.253**	**81.49**	**28.7**	**26.9**	**1.551**	**41.8**	**19.39**	**46.08**
	Equiv. Refl.: (3,-4,-20), (4,-3,-20), (-1,4,-20), (-3,-1,-20), (-4,1,-20)								
**LiNbO3**	**(2,-6,-16)**	**10.263**	**81.88**	**28.1**	**26.9**	**1.48**	**39.8**	**18.5**	**43.89**
	Equiv. Refl.: (4,2,-16), (6,-2,-16), (-2,-4,-16), (-4,6,-16), (-6,4,-16)								
**LiNbO3**	**(1,-5,-18)**	**10.266**	**82**	**27.3**	**25.5**	**1.457**	**37.19**	**18.22**	**41.41**
	Equiv. Refl.: (4,1,-18), (5,-1,-18), (-1,-4,-18), (-4,5,-18), (-5,4,-18)								
**LiNbO3**	**(0,2,22)**	**10.223**	**80.45**	**27.1**	**24.6**	**1.744**	**42.96**	**21.8**	**48.18**
	Equiv. Refl.: (2,-2,22), (-2,0,22)								
**LiNbO3**	**(1,4,-18)**	**10.266**	**82**	**26.6**	**24.9**	**1.457**	**36.34**	**18.22**	**40.65**
	Equiv. Refl.: (4,-5,-18), (5,-4,-18), (-1,5,-18), (-4,-1,-18), (-5,1,-18)								
**LiNbO3**	**(1,4,18)**	**10.266**	**82**	**26.4**	**25.5**	**1.457**	**37.19**	**18.22**	**41.41**
	Equiv. Refl.: (4,-5,18), (5,-4,18), (-1,5,18), (-4,-1,18), (-5,1,18)								
**LiNbO3**	**(0,-2,-22)**	**10.223**	**80.45**	**26**	**24.6**	**1.744**	**42.96**	**21.8**	**48.18**
	Equiv. Refl.: (2,0,-22), (-2,2,-22)								
**Sapph**	**(1,2,20)**	**10.342**	**86.05**	**25.8**	**22.8**	**0.715**	**16.29**	**8.94**	**18.58**
	Equiv. Refl.: (1,-3,-20), (2,1,-20), (2,-3,20), (3,-1,-20), (3,-2,20), (-1,3,20), (-1,-2,-20), (-2,3,-20), (-2,-1,20) (-3,1,20), (-3,2,-20)								
**LiNbO3**	**(1,-5,18)**	**10.266**	**82**	**25.7**	**24.9**	**1.457**	**36.34**	**18.22**	**40.65**
	Equiv. Refl.: (4,1,18), (5,-1,18), (-1,-4,18), (-4,5,18), (-5,4,18)								
**Quartz**	**(4,4,2)**	**10.352**	**86.89**	**25**	**22**	**0.564**	**12.4**	**7.05**	**14.27**
	Equiv. Refl.: (4,4,-2), (4,-8,2), (4,-8,-2), (-8,4,2), (-8,4,-2)								
**Quartz**	**(8,-4,2)**	**10.352**	**86.89**	**24.7**	**22**	**0.564**	**12.4**	**7.05**	**14.27**
	Equiv. Refl.: (8,-4,-2), (-4,8,2), (-4,8,-2), (-4,-4,2), (-4,-4,-2)								
**LiNbO3**	**(2,6,-4)**	**10.185**	**79.26**	**24.4**	**22.5**	**1.967**	**44.31**	**24.59**	**50.67**
	Equiv. Refl.: (6,-8,-4), (8,-6,-4), (-2,8,-4), (-6,-2,-4), (-8,2,-4)								
**LiNbO3**	**(2,-8,4)**	**10.185**	**79.26**	**24.3**	**22.5**	**1.967**	**44.31**	**24.59**	**50.67**
	Equiv. Refl.: (6,2,4), (8,-2,4), (-2,-6,4), (-6,8,4), (-8,6,4)								
**LiNbO3**	**(1,-7,-10)**	**10.156**	**78.41**	**23**	**20.7**	**2.126**	**44.01**	**26.58**	**51.42**
	Equiv. Refl.: (6,1,-10), (7,-1,-10), (-1,-6,-10), (-6,7,-10), (-7,6,-10)								
**LiNbO3**	**(2,-7,12)**	**10.208**	**79.95**	**22.1**	**20.9**	**1.838**	**38.35**	**22.97**	**44.71**
	Equiv. Refl.: (5,2,12), (7,-2,12), (-2,-5,12), (-5,7,12), (-7,5,12)								

Ei =	10.367	keV						ΔEg	ΔEt
**Cryst**	**Refl**	**EB**	**ΘB**	**∫ IR dΘ**	**Width**	**Ei cotΘ**	**ΔE**	**2m, 50μm**	**2m, 50μm**
	**(h,k,l)**	**[keV]**	**[°]**	**[μrad]**	**[μrad]**	**[meV/μrad]**	**[meV]**	**[meV]**	**[meV]**
**LiNbO3**	**(2,5,-12)**	**10.208**	**79.95**	**22**	**20.9**	**1.838**	**38.35**	**22.97**	**44.71**
	Equiv. Refl.: (5,-7,-12), (7,-5,-12), (-2,7,-12), (-5,-2,-12), (-7,2,-12)								
**LiNbO3**	**(1,6,10)**	**10.156**	**78.41**	**21.7**	**20.7**	**2.126**	**44.01**	**26.58**	**51.42**
	Equiv. Refl.: (6,-7,10), (7,-6,10), (-1,7,10), (-6,-1,10), (-7,1,10)								
**LiNbO3**	**(2,5,12)**	**10.208**	**79.95**	**21**	**20**	**1.838**	**36.76**	**22.97**	**43.35**
	Equiv. Refl.: (5,-7,12), (7,-5,12), (-2,7,12), (-5,-2,12), (-7,2,12)								
**LiNbO3**	**(2,-7,-12)**	**10.208**	**79.95**	**20.9**	**20**	**1.838**	**36.76**	**22.97**	**43.35**
	Equiv. Refl.: (5,2,-12), (7,-2,-12), (-2,-5,-12), (-5,7,-12), (-7,5,-12)								
**LiNbO3**	**(1,6,-11)**	**10.36**	**87.93**	**20.7**	**30.7**	**0.375**	**11.5**	**4.68**	**12.42**
	Equiv. Refl.: (1,-7,11), (6,1,11), (6,-7,-11), (7,-1,11), (7,-6,-11), (-1,7,-11), (-1,-6,11), (-6,7,11), (-6,-1,-11) (-7,1,-11), (-7,6,11)								
**LiNbO3**	**(3,-8,-4)**	**9.896**	**72.67**	**19.1**	**17**	**3.235**	**54.99**	**40.44**	**68.26**
	Equiv. Refl.: (5,3,-4), (8,-3,-4), (-3,-5,-4), (-5,8,-4), (-8,5,-4)								
**LiNbO3**	**(3,5,4)**	**9.896**	**72.67**	**18.6**	**17**	**3.235**	**54.99**	**40.44**	**68.26**
	Equiv. Refl.: (5,-8,4), (8,-5,4), (-3,8,4), (-5,-3,4), (-8,3,4)								
**Quartz**	**(4,2,6)**	**10.335**	**85.46**	**17.5**	**15**	**0.823**	**12.34**	**10.28**	**16.06**
	Equiv. Refl.: (4,-6,-6), (-6,2,-6), (-6,4,6)								
**Quartz**	**(2,4,-6)**	**10.335**	**85.46**	**17.4**	**15**	**0.823**	**12.33**	**10.28**	**16.06**
	Equiv. Refl.: (2,-6,6)								
**LiNbO3**	**(4,4,-6)**	**10**	**74.71**	**17**	**15.6**	**2.833**	**44.12**	**35.42**	**56.58**
	Equiv. Refl.: (4,-8,-6), (8,-4,-6), (-4,8,-6), (-4,-4,-6), (-8,4,-6)								
**Quartz**	**(6,-2,6)**	**10.335**	**85.46**	**16.8**	**15**	**0.823**	**12.34**	**10.28**	**16.06**
	Equiv. Refl.: (6,-4,-6), (-2,6,-6), (-2,-4,6), (-4,6,6), (-4,-2,-6)								
**LiNbO3**	**(4,4,6)**	**10**	**74.71**	**16.4**	**15.6**	**2.833**	**44.12**	**35.42**	**56.58**
	Equiv. Refl.: (4,-8,6), (8,-4,6), (-4,8,6), (-4,-4,6), (-8,4,6)								
**LiNbO3**	**(1,-6,-14)**	**9.956**	**73.82**	**16.3**	**15.1**	**3.008**	**45.34**	**37.6**	**58.9**
	Equiv. Refl.: (5,1,-14), (6,-1,-14), (-1,-5,-14), (-5,6,-14), (-6,5,-14)								
**LiNbO3**	**(1,5,14)**	**9.956**	**73.82**	**16.2**	**15.1**	**3.008**	**45.34**	**37.6**	**58.9**
	Equiv. Refl.: (5,-6,14), (6,-5,14), (-1,6,14), (-5,-1,14), (-6,1,14)								
**LiNbO3**	**(0,-6,-12)**	**9.92**	**73.11**	**15.1**	**14**	**3.148**	**43.99**	**39.35**	**59.02**
	Equiv. Refl.: (6,0,-12), (-6,6,-12)								
**LiNbO3**	**(0,6,12)**	**9.92**	**73.11**	**14.8**	**14**	**3.148**	**43.99**	**39.35**	**59.02**
	Equiv. Refl.: (6,-6,12), (-6,0,12)								
**LiNbO3**	**(0,-5,-16)**	**9.976**	**74.22**	**14.7**	**13.1**	**2.929**	**38.49**	**36.62**	**53.13**
	Equiv. Refl.: (5,0,-16), (-5,5,-16)								
**LiNbO3**	**(0,1,-22)**	**9.936**	**73.41**	**13.8**	**13**	**3.088**	**40.22**	**38.6**	**55.74**
	Equiv. Refl.: (1,-1,-22), (2,6,2), (6,-8,2), (8,-6,2), (-1,0,-22), (-2,8,2), (-6,-2,2), (-8,2,2)								
**LiNbO3**	**(0,-1,22)**	**9.936**	**73.41**	**13.7**	**13**	**3.088**	**40.22**	**38.6**	**55.74**
	Equiv. Refl.: (1,0,22), (-1,1,22), (6,-2,-6), (6,-4,6), (-2,6,6), (-2,-4,-6), (-4,6,-6), (-4,-2,6)								
**LiNbO3**	**(2,-8,-2)**	**10.067**	**76.18**	**13.6**	**13.3**	**2.551**	**34.04**	**31.88**	**46.64**
	Equiv. Refl.: (6,2,-2), (8,-2,-2), (-2,-6,-2), (-6,8,-2), (-8,6,-2)								
**LiNbO3**	**(0,6,-12)**	**9.92**	**73.11**	**13.3**	**12.4**	**3.148**	**39.12**	**39.35**	**55.49**
	Equiv. Refl.: (6,-6,-12), (-6,0,-12)								
**LiNbO3**	**(0,-7,-2)**	**9.774**	**70.53**	**13**	**11.9**	**3.664**	**43.67**	**45.8**	**63.28**
	Equiv. Refl.: (7,0,-2), (-7,7,-2)								
**LiNbO3**	**(0,7,-4)**	**9.896**	**72.67**	**12.9**	**12.1**	**3.235**	**39.27**	**40.44**	**56.37**
	Equiv. Refl.: (7,-7,-4), (-7,0,-4)								
**LiNbO3**	**(0,5,16)**	**9.976**	**74.22**	**12.8**	**13.1**	**2.929**	**38.49**	**36.62**	**53.13**
	Equiv. Refl.: (0,-6,12), (5,-5,16), (6,0,12), (-5,0,16), (-6,6,12)								
**Quartz**	**(0,3,-8)**	**10.164**	**78.64**	**12.7**	**10.6**	**2.082**	**22.03**	**26.03**	**34.1**
	Equiv. Refl.: (0,-3,8), (3,0,8), (3,-3,-8), (-3,0,-8), (-3,3,8)								

Ei =	10.367	keV						ΔEg	ΔEt
**Cryst**	**Refl (h,k,l)**	**EB [keV]**	**ΘB [°]**	**∫ IR dΘ [μrad]**	**Width [μrad]**	**Ei cotΘ [meV/μrad]**	**ΔE [meV]**	**2m, 50μm [meV]**	**2m, 50μm [meV]**
**LiNbO3**	**(0,-7,4)**	**9.896**	**72.67**	**12.6**	**12.1**	**3.235**	**39.27**	**40.44**	**56.37**
	Equiv. Refl.: (7,0,4), (-7,7,4)								
**LiNbO3**	**(0,7,2)**	**9.774**	**70.53**	**12.4**	**11.9**	**3.664**	**43.67**	**45.8**	**63.28**
	Equiv. Refl.: (7,-7,2), (-7,0,2), (0,7,1), (0,-7,-1), (7,0,-1), (7,-7,1), (-7,0,1), (-7,7,-1)								
**LiNbO3**	**(1,-7,8)**	**9.795**	**70.87**	**12.3**	**11.2**	**3.595**	**40.37**	**44.94**	**60.41**
	Equiv. Refl.: (6,1,8), (7,-1,8), (-1,-6,8), (-6,7,8), (-7,6,8)								
**Quartz**	**(2,4,6)**	**10.335**	**85.46**	**12**	**11.7**	**0.823**	**9.63**	**10.28**	**14.09**
	Equiv. Refl.: (2,-6,-6), (4,2,-6), (4,-6,6), (-6,2,6), (-6,4,-6)								
**LiNbO3**	**(1,6,-8)**	**9.795**	**70.87**	**11.5**	**11.2**	**3.595**	**40.37**	**44.94**	**60.41**
	Equiv. Refl.: (6,-7,-8), (7,-6,-8), (-1,7,-8), (-6,-1,-8), (-7,1,-8)								
**Sapph**	**(0,5,-14)**	**10.061**	**76.05**	**11.3**	**9.3**	**2.574**	**23.88**	**32.18**	**40.07**
	Equiv. Refl.: (0,-5,14), (5,0,14), (5,-5,-14), (-5,0,-14), (-5,5,14)								
**Sapph**	**(2,5,9)**	**10.331**	**85.19**	**9.9**	**9.9**	**0.872**	**8.65**	**10.9**	**13.91**
	Equiv. Refl.: (2,5,-9), (2,-7,9), (2,-7,-9), (5,2,9), (5,2,-9), (5,-7,9), (5,-7,-9), (7,-2,9), (7,-2,-9) (7,-5,9), (7,-5,-9), (-2,7,9), (-2,7,-9), (-2,-5,9), (-2,-5,-9), (-5,7,9), (-5,7,-9), (-5,-2,9) (-5,-2,-9), (-7,2,9), (-7,2,-9), (-7,5,9), (-7,5,-9)								
**Quartz**	**(0,7,0)**	**10.199**	**79.67**	**8.7**	**7.5**	**1.89**	**14.21**	**23.62**	**27.57**
	Equiv. Refl.: (0,-7,0), (7,0,0), (7,-7,0), (-7,0,0), (-7,7,0)								
**Sapph**	**(2,3,-16)**	**10.066**	**76.16**	**8.4**	**7.2**	**2.555**	**18.34**	**31.94**	**36.83**
	Equiv. Refl.: (2,-5,16), (3,2,16), (3,-5,-16), (5,-2,16), (5,-3,-16), (-2,5,-16), (-2,-3,16), (-3,5,16), (-3,-2,-16) (-5,2,-16), (-5,3,16)								
**Sapph**	**(2,5,6)**	**9.822**	**71.34**	**8.2**	**6.8**	**3.502**	**23.79**	**43.77**	**49.82**
	Equiv. Refl.: (2,5,-6), (2,-7,6), (2,-7,-6), (5,2,6), (5,2,-6), (5,-7,6), (5,-7,-6), (7,-2,6), (7,-2,-6) (7,-5,6), (7,-5,-6), (-2,7,6), (-2,7,-6), (-2,-5,6), (-2,-5,-6), (-5,7,6), (-5,7,-6), (-5,-2,6) (-5,-2,-6), (-7,2,6), (-7,2,-6), (-7,5,6), (-7,5,-6)								
**LiNbO3**	**(3,-8,2)**	**9.774**	**70.53**	**8**	**7.9**	**3.664**	**28.93**	**45.8**	**54.17**
	Equiv. Refl.: (5,3,2), (8,-3,2), (-3,-5,2), (-5,8,2), (-8,5,2)								
**LiNbO3**	**(3,5,-2)**	**9.774**	**70.53**	**7.7**	**7.9**	**3.664**	**28.93**	**45.8**	**54.17**
	Equiv. Refl.: (5,-8,-2), (8,-5,-2), (-3,8,-2), (-5,-3,-2), (-8,3,-2)								
**Quartz**	**(3,3,6)**	**10.231**	**80.72**	**7.2**	**6.3**	**1.694**	**10.76**	**21.18**	**23.75**
	Equiv. Refl.: (3,3,-6), (3,-6,6), (3,-6,-6), (6,-3,6), (6,-3,-6), (-3,6,6), (-3,6,-6), (-3,-3,6), (-3,-3,-6) (-6,3,6), (-6,3,-6)								
**Quartz**	**(7,-1,-2)**	**9.826**	**71.4**	**7**	**5.6**	**3.488**	**19.68**	**43.61**	**47.84**
	Equiv. Refl.: (7,-6,2), (-1,7,2), (-1,-6,-2), (-6,7,-2), (-6,-1,2)								
**Sapph**	**(0,2,-20)**	**10.009**	**74.89**	**6.9**	**6**	**2.798**	**16.66**	**34.98**	**38.74**
	Equiv. Refl.: (0,-2,20), (2,0,20), (2,-2,-20), (3,4,8), (3,-7,-8), (4,3,-8), (4,-7,8), (7,-3,-8), (7,-4,8) (-2,0,-20), (-2,2,20), (-3,7,8), (-3,-4,-8), (-4,7,-8), (-4,-3,8), (-7,3,8), (-7,4,-8)								
**Quartz**	**(1,6,2)**	**9.826**	**71.4**	**6.7**	**5.6**	**3.488**	**19.68**	**43.61**	**47.84**
	Equiv. Refl.: (1,-7,-2), (6,1,-2), (6,-7,2), (-7,1,2), (-7,6,-2)								
**Quartz**	**(8,-3,0)**	**10.199**	**79.67**	**6.2**	**5.2**	**1.89**	**9.89**	**23.62**	**25.61**
	Equiv. Refl.: (8,-4,0), (8,-5,0), (-3,8,0), (-3,-5,0), (-4,8,0), (-4,-4,0), (-5,8,0), (-5,-3,0)								
**Quartz**	**(3,5,0)**	**10.199**	**79.67**	**5.9**	**5.2**	**1.89**	**9.89**	**23.62**	**25.61**
	Equiv. Refl.: (3,-8,0), (5,3,0), (5,-8,0), (-8,3,0), (-8,5,0)								
**Quartz**	**(0,4,-7)**	**9.921**	**73.14**	**5.6**	**4.7**	**3.142**	**14.92**	**39.28**	**42.02**
	Equiv. Refl.: (0,-4,7), (1,2,-8), (1,-3,8), (2,1,8), (2,-3,-8), (3,-1,8), (3,-2,-8), (4,0,7), (4,4,0) (4,-4,-7), (4,-8,0), (-1,3,-8), (-1,-2,8), (-2,3,8), (-2,-1,-8), (-3,1,-8), (-3,2,8), (-4,0,-7) (-4,4,7), (-8,4,0)								
**Sapph**	**(1,6,4)**	**10.048**	**75.75**	**5.3**	**4.7**	**2.632**	**12.35**	**32.9**	**35.14**
	Equiv. Refl.: (1,-7,-4), (6,1,-4), (6,-7,4), (7,-1,-4), (7,-6,4), (-1,7,4), (-1,-6,-4), (-6,7,-4), (-6,-1,4) (-7,1,4), (-7,6,-4)								

Ei =	10.367	keV						ΔEg	ΔEt
**Cryst**	**Refl (h,k,l)**	**EB [keV]**	**ΘB [°]**	**∫ IR dΘ [μrad]**	**Width [μrad]**	**Ei cotΘ [meV/μrad]**	**ΔE [meV]**	**2m, 50μm [meV]**	**2m, 50μm [meV]**
**Quartz**	**(7,-3,4)**	**9.98**	**74.29**	**5.1**	**4.2**	**2.916**	**12.33**	**36.45**	**38.48**
	Equiv. Refl.: (7,-4,-4), (-3,7,-4), (-3,-4,4), (-4,7,4), (-4,-3,-4)								
**Quartz**	**(0,6,-4)**	**9.873**	**72.24**	**5**	**4.1**	**3.321**	**13.77**	**41.51**	**43.74**
	Equiv. Refl.: (0,-6,4), (3,4,-4), (3,-7,4), (4,3,4), (4,-7,-4), (6,0,4), (6,-6,-4), (-6,0,-4), (-6,6,4) (-7,3,-4), (-7,4,4)								
**Quartz**	**(1,5,-5)**	**9.935**	**73.4**	**4.9**	**4.1**	**3.091**	**12.67**	**38.64**	**40.67**
	Equiv. Refl.: (1,-6,5), (5,1,5), (5,-6,-5), (-6,1,-5), (-6,5,5)								
**Quartz**	**(6,-1,5)**	**9.935**	**73.4**	**4.8**	**4.1**	**3.091**	**12.67**	**38.64**	**40.67**
	Equiv. Refl.: (6,-5,-5), (-1,6,-5), (-1,-5,5), (-5,6,5), (-5,-1,-5)								
**Sapph**	**(1,4,15)**	**9.939**	**73.48**	**4.7**	**4.2**	**3.075**	**12.93**	**38.43**	**40.55**
	Equiv. Refl.: (1,4,-15), (1,-5,15), (1,-5,-15), (4,1,15), (4,1,-15), (4,-5,15), (4,-5,-15), (5,-1,15), (5,-1,-15) (5,-4,15), (5,-4,-15), (-1,5,15), (-1,5,-15), (-1,-4,15), (-1,-4,-15), (-4,5,15), (-4,5,-15), (-4,-1,15) (-4,-1,-15), (-5,1,15), (-5,1,-15), (-5,4,15), (-5,4,-15)								
**Quartz**	**(1,2,8)**	**9.953**	**73.75**	**4.6**	**3.8**	**3.021**	**11.51**	**37.77**	**39.48**
	Equiv. Refl.: (1,-3,-8), (2,1,-8), (2,-3,8), (-3,1,8), (-3,2,-8)								
**Quartz**	**(3,-1,-8)**	**9.953**	**73.75**	**4.4**	**3.8**	**3.021**	**11.51**	**37.77**	**39.48**
	Equiv. Refl.: (3,-2,8), (-1,3,8), (-1,-2,-8), (-2,3,-8), (-2,-1,8)								
**Quartz**	**(1,6,-3)**	**10.155**	**78.39**	**3.9**	**3.4**	**2.13**	**7.21**	**26.62**	**27.58**
	Equiv. Refl.: (1,-7,3), (6,1,3), (6,-7,-3), (7,-2,4), (7,-5,-4), (-2,7,-4), (-2,-5,4), (-5,7,4), (-5,-2,-4) (-7,1,-3), (-7,6,3)								
**Quartz**	**(2,5,-4)**	**10.19**	**79.4**	**3.8**	**3.7**	**1.94**	**7.09**	**24.24**	**25.26**
	Equiv. Refl.: (2,-7,4), (5,2,4), (5,-7,-4), (-7,2,-4), (-7,5,4)								
**Sapph**	**(1,6,-2)**	**9.911**	**72.95**	**3.7**	**3.4**	**3.18**	**10.91**	**39.75**	**41.22**
	Equiv. Refl.: (1,-7,2), (6,1,2), (6,-7,-2), (7,-1,2), (7,-6,-2), (-1,7,-2), (-1,-6,2), (-6,7,2), (-6,-1,-2) (-7,1,-2), (-7,6,2), (7,-1,-3), (7,-6,3), (-1,7,3), (-1,-6,-3), (-6,7,-3), (-6,-1,3)								
**Quartz**	**(7,-1,3)**	**10.155**	**78.39**	**3.5**	**3.4**	**2.13**	**7.21**	**26.62**	**27.58**
	Equiv. Refl.: (7,-6,-3), (-1,7,-3), (-1,-6,3), (-6,7,3), (-6,-1,-3)								
**Sapph**	**(1,5,11)**	**9.886**	**72.47**	**3.3**	**3**	**3.274**	**9.91**	**40.93**	**42.11**
	Equiv. Refl.: (1,-6,-11), (5,1,-11), (5,-6,11), (6,-1,-11), (6,-5,11), (-1,6,11), (-1,-5,-11), (-5,6,-11), (-5,-1,11) (-6,1,11), (-6,5,-11), (-7,1,3), (-7,6,-3)								
**Quartz**	**(1,6,3)**	**10.155**	**78.39**	**3.2**	**3.2**	**2.13**	**6.73**	**26.62**	**27.46**
	Equiv. Refl.: (1,-7,-3), (6,1,-3), (6,-7,3)								
**Quartz**	**(2,5,4)**	**10.19**	**79.4**	**3.1**	**3.1**	**1.94**	**5.95**	**24.24**	**24.96**
	Equiv. Refl.: (2,-7,-4), (5,2,-4), (5,-7,4), (-7,2,4), (-7,5,-4)								
**Quartz**	**(7,-2,-4)**	**10.19**	**79.4**	**2.9**	**3.1**	**1.94**	**5.95**	**24.24**	**24.96**
	Equiv. Refl.: (7,-5,4), (-2,7,4), (-2,-5,-4), (-5,7,-4), (-5,-2,4)								
**Quartz**	**(0,7,-1)**	**10.263**	**81.89**	**2.7**	**3**	**1.478**	**4.45**	**18.48**	**19.01**
	Equiv. Refl.: (0,-7,1), (7,0,1), (7,-7,-1), (-7,0,-1), (-7,7,1)								
**Sapph**	**(2,4,13)**	**10.093**	**76.8**	**2.6**	**2.8**	**2.431**	**6.86**	**30.39**	**31.16**
	Equiv. Refl.: (2,-6,-13), (4,2,-13), (4,-6,13), (6,-2,-13), (6,-4,13), (-2,6,13), (-2,-4,-13), (-4,6,-13), (-4,-2,13) (-6,2,13), (-6,4,-13)								
**LiNbO3**	**(3,4,-13)**	**10.263**	**81.88**	**2.3**	**4.9**	**1.479**	**7.21**	**18.48**	**19.84**
	Equiv. Refl.: (3,-7,13), (4,3,13), (4,-7,-13), (7,-3,13), (7,-4,-13), (-3,7,-13), (-3,-4,13), (-4,7,13), (-4,-3,-13) (-7,3,-13), (-7,4,13), (5,-2,-7), (5,-3,7), (-2,5,7), (-2,-3,-7), (-3,5,-7), (-3,-2,7)								
**Quartz**	**(2,3,7)**	**10.237**	**80.92**	**2.1**	**2.7**	**1.656**	**4.51**	**20.7**	**21.19**
	Equiv. Refl.: (2,-5,-7), (3,2,-7), (3,-5,7), (-5,2,7), (-5,3,-7)								
**Quartz**	**(3,4,4)**	**9.98**	**74.29**	**2**	**2.3**	**2.916**	**6.56**	**36.45**	**37.04**
	Equiv. Refl.: (3,-7,-4), (4,3,-4), (4,-7,4), (-7,3,4), (-7,4,-4)								
**Quartz**	**(7,-3,-4)**	**9.98**	**74.29**	**1.8**	**2.2**	**2.916**	**6.56**	**36.45**	**37.04**
	Equiv. Refl.: (7,-4,4), (8,-4,1), (8,-4,-1), (-3,7,4), (-3,-4,-4), (-4,7,-4), (-4,8,1), (-4,8,-1), (-4,-3,4) (-4,-4,1), (-4,-4,-1)								

**Ei =**	**10.367**	**keV**						**ΔEg**	**ΔEt**
**Cryst**	**Refl**	**EB**	**ΘB**	**∫IR dΘ**	**Width**	**Ei cotΘ**	**ΔE**	**2m, 50µm**	**2m, 50µm**
	**(h,k,l)**	**[keV]**	**[°]**	**[µrad]**	**[µrad]**	**[meV/µrad]**	**[meV]**	**[meV]**	**[meV]**
**Quartz**	**(1,6,-2)**	**9.826**	**71.4**	**1.7**	**1.9**	**3.488**	**6.78**	**43.61**	**44.13**
	Equiv. Refl.: (1,-7,2), (6,1,2), (6,-7,-2), (-7,1,-2), (-7,6,2)								
**Quartz**	**(7,-1,2)**	**9.826**	**71.4**	**1.6**	**1.9**	**3.488**	**6.78**	**43.61**	**44.13**
	Equiv. Refl.: (7,-6,-2), (-1,7,-2), (-1,-6,2), (-6,7,2), (-6,-1,-2)								
**Quartz**	**(4,4,1)**	**10.159**	**78.51**	**1.5**	**2.1**	**2.107**	**4.36**	**26.34**	**26.7**
	Equiv. Refl.: (4,4,-1), (4,-8,1), (4,-8,-1), (-8,4,1), (-8,4,-1)								
**Sapph**	**(1,6,-5)**	**10.15**	**78.25**	**1.3**	**1.8**	**2.157**	**3.96**	**26.96**	**27.25**
	Equiv. Refl.: (1,-7,5), (6,1,5), (6,-7,-5), (7,-1,5), (7,-6,-5), (-1,7,-5), (-1,-6,5), (-6,7,5), (-6,-1,-5)<br>(-7,1,-5), (-7,6,5), (2,3,-7), (2,-5,7), (3,2,7), (3,-5,-7), (-5,2,-7), (-5,3,7)								
**LiNbO3**	**(1,3,19)**	**9.865**	**72.1**	**1.2**	**2.5**	**3.348**	**8.36**	**41.85**	**42.68**
	Equiv. Refl.: (1,-4,-19), (3,1,-19), (3,4,11), (3,-4,19), (3,-7,-11), (4,3,-11), (4,-1,-19), (4,-3,19), (4,-7,11)<br>(7,-3,-11), (7,-4,11), (-1,4,19), (-1,-3,-19), (-3,4,-19), (-3,7,11), (-3,-1,19), (-3,-4,-11), (-4,1,19)<br>(-4,3,-19), (-4,7,-11), (-4,-3,11), (-7,3,11), (-7,4,-11)								
**Sapph**	**(1,2,-19)**	**9.904**	**72.8**	**1.1**	**1.5**	**3.208**	**4.79**	**40.1**	**40.39**
	Equiv. Refl.: (1,-3,19), (2,1,19), (2,-3,-19), (3,-1,19), (3,-2,-19), (-1,3,-19), (-1,-2,19), (-2,3,19), (-2,-1,-19)<br>(-3,1,-19), (-3,2,19), (5,-2,7), (5,-3,-7), (-2,5,-7), (-2,-3,7), (-3,5,7), (-3,-2,-7)								
**Quartz**	**(0,5,6)**	**10.022**	**75.17**	**1**	**1.4**	**2.745**	**3.72**	**34.31**	**34.51**
	Equiv. Refl.: (0,-5,-6), (3,5,1), (3,-8,-1), (5,0,-6), (5,3,-1), (5,-5,6), (5,-8,1), (-5,0,6), (-5,5,-6)<br>(-8,3,1), (-8,5,-1)								
**LiNbO3**	**(2,6,5)**	**10.273**	**82.29**	**0.9**	**2.9**	**1.404**	**4.09**	**17.55**	**18.02**
	Equiv. Refl.: (2,-8,-5), (6,2,-5), (6,-8,5), (8,-2,-5), (8,-6,5), (-2,8,5), (-2,-6,-5), (-6,8,-5), (-6,-2,5)<br>(-8,2,5), (-8,6,-5), (1,6,1), (1,-7,-1), (6,1,-1), (6,-7,1), (7,-1,-1), (7,-6,1), (-1,7,1)<br>(-1,-6,-1), (-6,7,-1), (-6,-1,1), (-7,1,1), (-7,6,-1), (8,-3,-1), (8,-5,1), (-3,8,1), (-3,-5,-1)<br>(-5,8,-1), (-5,-3,1)								
**Sapph**	**(1,3,-17)**	**9.76**	**70.3**	**0.6**	**0.9**	**3.712**	**3.5**	**46.41**	**46.54**
	Equiv. Refl.: (1,-4,17), (2,2,18), (2,2,-18), (2,-4,18), (2,-4,-18), (3,1,17), (3,-4,-17), (4,-1,17), (4,-2,18)<br>(4,-2,-18), (4,-3,-17), (-1,4,-17), (-1,-3,17), (-2,4,18), (-2,4,-18), (-2,-2,18), (-2,-2,-18), (-3,4,17)<br>(-3,-1,-17), (-4,1,-17), (-4,2,18), (-4,2,-18), (-4,3,17)								
**LiNbO3**	**(2,6,-1)**	**10.037**	**75.5**	**0.5**	**1.6**	**2.681**	**4.42**	**33.51**	**33.8**
	Equiv. Refl.: (2,-8,1), (6,2,1), (6,-8,-1), (8,-2,1), (8,-6,-1), (-2,8,-1), (-2,-6,1), (-6,8,1), (-6,-2,-1)<br>(-8,2,-1), (-8,6,1), (8,-3,1), (8,-5,-1), (-3,8,-1), (-3,-5,1), (-5,8,1), (-5,-3,-1)								
**LiNbO3**	**(3,5,7)**	**10.224**	**80.48**	**0.4**	**1.7**	**1.738**	**3.01**	**21.72**	**21.93**
	Equiv. Refl.: (3,-8,-7), (5,3,-7), (5,-8,7), (8,-3,-7), (8,-5,7), (-3,8,7), (-3,-5,-7), (-5,8,-7), (-5,-3,7)<br>(-8,3,7), (-8,5,-7), (0,6,4), (0,-6,-4), (3,5,-1), (3,-8,1), (5,3,1), (5,-8,-1), (6,0,-4)<br>(6,-6,4), (-6,0,4), (-6,6,-4), (-8,3,-1), (-8,5,1)								
**LiNbO3**	**(3,5,-5)**	**9.987**	**74.44**	**0.3**	**1.1**	**2.887**	**3.26**	**36.09**	**36.24**
	Equiv. Refl.: (3,-8,5), (5,3,5), (5,-8,-5), (8,-3,5), (8,-5,-5), (-3,8,-5), (-3,-5,5), (-5,8,5), (-5,-3,-5)<br>(-8,3,-5), (-8,5,5)								
**LiNbO3**	**(3,5,1)**	**9.744**	**70.03**	**0.2**	**0.9**	**3.767**	**3.53**	**47.09**	**47.22**
	Equiv. Refl.: (3,-8,-1), (5,3,-1), (5,-8,1), (8,-3,-1), (8,-5,1), (-3,8,1), (-3,-5,-1), (-5,8,-1), (-5,-3,1)<br>(-8,3,1), (-8,5,-1), (3,4,-7), (3,-7,7), (4,3,7), (4,-7,-7), (7,-3,7), (7,-4,-7), (-3,7,-7)<br>(-3,-4,7), (-4,7,7), (-4,-3,-7), (-7,3,-7), (-7,4,7), (0,5,-6), (0,-5,6), (5,0,6), (5,-5,-6)<br>(-5,0,-6), (-5,5,6)								
**LiNbO3**	**(3,3,15)**	**9.859**	**71.99**	**0.1**	**0.7**	**3.371**	**2.28**	**42.14**	**42.2**
	Equiv. Refl.: (3,3,-15), (3,-6,15), (3,-6,-15), (6,-3,15), (6,-3,-15), (-3,6,15), (-3,6,-15), (-3,-3,15), (-3,-3,-15)<br>(-6,3,15), (-6,3,-15)								

Ei =	11.103	keV						ΔEg	ΔEt
Cryst	Refl	EB	ΘB	∫ IR dΘ	Width	E cotΘ	ΔE	2m	2m
	(h,k,l)	[keV]	[°]	[μrad]	[μrad]	[keV]	[meV]	[meV]	[meV]
Si	(4,6,6)	10.708	74.67	6.639	5.7	3.045	17.27	38.06	41.79
Si	(1,3,9)	10.889	78.72	5.759	5.2	2.214	11.4	27.67	29.93
Ge	(4,4,8)	10.736	75.24	1.845	2.8	2.926	8.31	36.58	37.51
Ge	(1,7,7)	10.903	79.11	1.583	2.8	2.137	6.04	26.71	27.39
	Equiv. Refl.: (3,3,9), (5,5,7)								
Ge	(1,3,9)	10.453	70.3	0.711	1.4	3.976	5.49	49.7	50
LiNbO3	(3,-7,16)	11.078	86.18	43.9	40.6	0.741	30.07	9.27	31.46
	Equiv. Refl.: (4,3,16), (7,-3,16), (-3,-4,16), (-4,7,16), (-7,4,16)								
LiNbO3	(3,4,-16)	11.078	86.18	41.4	40.6	0.741	30.07	9.27	31.46
	Equiv. Refl.: (4,-7,-16), (7,-4,-16), (-3,7,-16), (-4,-3,-16), (-7,3,-16)								
LiNbO3	(1,6,-14)	11.06	84.98	36.8	33.4	0.976	32.62	12.19	34.83
	Equiv. Refl.: (6,-7,-14), (7,-6,-14), (-1,7,-14), (-6,-1,-14), (-7,1,-14)								
LiNbO3	(1,-7,14)	11.06	84.98	34.4	33.4	0.976	32.62	12.19	34.83
	Equiv. Refl.: (6,1,14), (7,-1,14), (-1,-6,14), (-6,7,14), (-7,6,14)								
LiNbO3	(3,6,0)	11.037	83.73	28.7	26.6	1.219	32.49	15.24	35.89
	Equiv. Refl.: (6,-9,0), (9,-6,0), (-3,9,0), (-6,-3,0), (-9,3,0)								
LiNbO3	(3,-9,0)	11.037	83.73	28	26.6	1.219	32.49	15.24	35.89
	Equiv. Refl.: (6,3,0), (9,-3,0), (-3,-6,0), (-6,9,0), (-9,6,0)								
LiNbO3	(1,1,24)	10.999	82.16	27.8	25.4	1.529	38.79	19.12	43.25
	Equiv. Refl.: (1,-2,24), (2,-1,24), (-1,2,24), (-1,-1,24), (-2,1,24)								
LiNbO3	(1,1,-24)	10.999	82.16	27.6	25.4	1.529	38.79	19.12	43.25
	Equiv. Refl.: (1,-2,-24), (2,-1,-24), (-1,2,-24), (-1,-1,-24), (-2,1,-24)								
LiNbO3	(1,-4,-22)	11.042	83.98	26.7	25.2	1.172	29.5	14.65	32.94
	Equiv. Refl.: (3,1,-22), (4,-1,-22), (-1,-3,-22), (-3,4,-22), (-4,3,-22)								
LiNbO3	(4,4,12)	11.027	83.31	26.1	24.1	1.303	31.4	16.29	35.37
	Equiv. Refl.: (4,-8,12), (8,-4,12), (-4,8,12), (-4,-4,12), (-8,4,12)								
LiNbO3	(1,3,22)	11.042	83.98	26	25.2	1.172	29.5	14.65	32.94
	Equiv. Refl.: (3,-4,22), (4,-3,22), (-1,4,22), (-3,-1,22), (-4,1,22)								
LiNbO3	(4,4,-12)	11.027	83.31	24.8	24.1	1.303	31.4	16.29	35.37
	Equiv. Refl.: (4,-8,-12), (8,-4,-12), (-4,8,-12), (-4,-4,-12), (-8,4,-12)								
LiNbO3	(4,-9,4)	11.006	82.44	23.5	21.5	1.474	31.68	18.43	36.65
	Equiv. Refl.: (5,4,4), (9,-4,4), (-4,-5,4), (-5,9,4), (-9,5,4)								
LiNbO3	(4,5,-4)	11.006	82.44	21.9	21.5	1.474	31.68	18.43	36.65
	Equiv. Refl.: (5,-9,-4), (9,-5,-4), (-4,9,-4), (-5,-4,-4), (-9,4,-4)								
LiNbO3	(2,-8,10)	10.979	81.43	20.3	18.9	1.674	31.59	20.93	37.89
	Equiv. Refl.: (6,2,10), (8,-2,10), (-2,-6,10), (-6,8,10), (-8,6,10)								
LiNbO3	(2,6,-10)	10.979	81.43	19.6	18.9	1.674	31.59	20.93	37.89
	Equiv. Refl.: (6,-8,-10), (8,-6,-10), (-2,8,-10), (-6,-2,-10), (-8,2,-10)								
LiNbO3	(4,5,2)	10.897	78.94	19.1	17.4	2.17	37.86	27.13	46.57
	Equiv. Refl.: (5,-9,2), (9,-5,2), (-4,9,2), (-5,-4,2), (-9,4,2)								
LiNbO3	(4,-9,-2)	10.897	78.94	18.8	17.4	2.17	37.86	27.13	46.57
	Equiv. Refl.: (5,4,-2), (9,-4,-2), (-4,-5,-2), (-5,9,-2), (-9,5,-2)								
Quartz	(4,4,4)	11.088	87.03	18	15.9	0.576	9.19	7.21	11.68
	Equiv. Refl.: (4,4,-4), (4,-8,4), (4,-8,-4), (8,-4,4), (8,-4,-4), (-4,8,-4), (-4,-4,4), (-8,4,4), (-8,4,-4)								
Quartz	(-4,8,4)	11.088	87.03	17.9	16	0.576	9.19	7.21	11.68
	Equiv. Refl.: (-4,-4,-4)								
LiNbO3	(3,3,18)	10.816	76.95	16.7	15	2.573	38.68	32.17	50.31
	Equiv. Refl.: (3,-6,18), (6,-3,18), (-3,6,18), (-3,-3,18), (-6,3,18)								

Ei =	11.103	keV						ΔEg	ΔEt
Cryst	Refl	EB	ΘB	∫ IR dΘ	Width	E cotΘ	ΔE	2m	2m
	(h,k,l)	[keV]	[°]	[μrad]	[μrad]	[keV]	[meV]	[meV]	[meV]
**LiNbO3**	**(3,3,-18)**	**10.816**	**76.95**	**16**	**15**	**2.573**	**38.68**	**32.17**	**50.31**
	Equiv. Refl.: (3,-6,-18), (6,-3,-18), (-3,6,-18), (-3,-3,-18), (-6,3,-18)								
**LiNbO3**	**(1,-8,-6)**	**10.835**	**77.4**	**15.6**	**14.5**	**2.483**	**35.92**	**31.04**	**47.47**
	Equiv. Refl.: (7,1,-6), (8,-1,-6), (-1,-7,-6), (-7,8,-6), (-8,7,-6)								
**LiNbO3**	**(1,7,6)**	**10.835**	**77.4**	**15.5**	**14.5**	**2.483**	**35.92**	**31.04**	**47.47**
	Equiv. Refl.: (7,-8,6), (8,-7,6), (-1,8,6), (-7,-1,6), (-8,1,6)								
**Sapph**	**(2,6,-4)**	**11.015**	**82.79**	**15.3**	**13**	**1.404**	**18.29**	**17.55**	**25.35**
	Equiv. Refl.: (2,-8,4), (6,2,4), (6,-8,-4), (8,-2,4), (8,-6,-4), (-2,8,-4), (-2,-6,4), (-6,8,4), (-6,-2,-4) (-8,2,-4), (-8,6,4)								
**LiNbO3**	**(1,7,-6)**	**10.835**	**77.4**	**14.8**	**13.6**	**2.483**	**33.89**	**31.04**	**45.95**
	Equiv. Refl.: (7,-8,-6), (8,-7,-6), (-1,8,-6), (-7,-1,-6), (-8,1,-6)								
**LiNbO3**	**(0,7,-10)**	**10.711**	**74.74**	**14.6**	**13.3**	**3.029**	**40.22**	**37.87**	**55.24**
	Equiv. Refl.: (7,-7,-10), (-7,0,-10)								
**LiNbO3**	**(0,-7,10)**	**10.711**	**74.74**	**14.4**	**13.3**	**3.029**	**40.22**	**37.87**	**55.24**
	Equiv. Refl.: (7,0,10), (-7,7,10)								
**LiNbO3**	**(1,-8,6)**	**10.835**	**77.4**	**14.3**	**13.6**	**2.483**	**33.89**	**31.04**	**45.95**
	Equiv. Refl.: (7,1,6), (8,-1,6), (-1,-7,6), (-7,8,6), (-8,7,6)								
**LiNbO3**	**(2,-8,-8)**	**10.646**	**73.5**	**12.8**	**11.7**	**3.288**	**38.43**	**41.1**	**56.27**
	Equiv. Refl.: (6,2,-8), (8,-2,-8), (-2,-6,-8), (-6,8,-8), (-8,6,-8)								
**Sapph**	**(1,3,-20)**	**10.979**	**81.44**	**12.7**	**10.8**	**1.671**	**18.1**	**20.89**	**27.64**
	Equiv. Refl.: (1,-4,20), (3,1,20), (3,-4,-20), (4,-1,20), (4,-3,-20), (-1,4,-20), (-1,-3,20), (-3,4,20), (-3,-1,-20) (-4,1,-20), (-4,3,20)								
**LiNbO3**	**(0,4,20)**	**10.532**	**71.54**	**12.5**	**11.4**	**3.705**	**42.19**	**46.32**	**62.66**
	Equiv. Refl.: (2,6,8), (4,-4,20), (6,-8,8), (8,-6,8), (-2,8,8), (-4,0,20), (-6,-2,8), (-8,2,8), (1,6,10) (1,-7,-10), (6,1,-10), (6,-7,10), (7,-1,-10), (7,-6,10), (-1,7,10), (-1,-6,-10), (-6,7,-10), (-6,-1,10) (-7,1,10), (-7,6,-10)								
**LiNbO3**	**(0,-4,-20)**	**10.532**	**71.54**	**12.4**	**11.4**	**3.705**	**42.19**	**46.32**	**62.66**
	Equiv. Refl.: (4,0,-20), (-4,4,-20)								
**LiNbO3**	**(2,-5,-20)**	**10.804**	**76.67**	**12.2**	**11.1**	**2.631**	**29.24**	**32.89**	**44**
	Equiv. Refl.: (3,2,-20), (3,5,10), (5,-2,-20), (5,-8,10), (8,-5,10), (-2,-3,-20), (-3,5,-20), (-3,8,10), (-5,3,-20) (-5,-3,10), (-8,3,10)								
**Quartz**	**(2,6,-3)**	**11.056**	**84.71**	**11.4**	**9.9**	**1.029**	**10.18**	**12.86**	**16.4**
	Equiv. Refl.: (2,-8,3), (6,2,3), (6,-8,-3), (8,-2,3), (8,-6,-3), (-2,8,-3), (-2,-6,3), (-8,2,-3), (-8,6,3)								
**LiNbO3**	**(2,3,20)**	**10.804**	**76.67**	**11.3**	**11.1**	**2.631**	**29.24**	**32.89**	**44**
	Equiv. Refl.: (3,-5,20), (3,-8,-10), (5,3,-10), (5,-3,20), (8,-3,-10), (-2,5,20), (-3,-2,20), (-3,-5,-10), (-5,2,20) (-5,8,-10), (-8,5,-10), (-6,8,3), (-6,-2,-3)								
**LiNbO3**	**(1,5,-16)**	**10.542**	**71.7**	**11.2**	**10.4**	**3.671**	**38.13**	**45.89**	**59.66**
	Equiv. Refl.: (5,-6,-16), (6,-5,-16), (-1,6,-16), (-5,-1,-16), (-6,1,-16)								
**LiNbO3**	**(1,-6,16)**	**10.542**	**71.7**	**11.1**	**10.4**	**3.671**	**38.13**	**45.89**	**59.66**
	Equiv. Refl.: (5,1,16), (6,-1,16), (-1,-5,16), (-5,6,16), (-6,5,16)								
**LiNbO3**	**(1,-3,22)**	**10.503**	**71.08**	**10.9**	**9.9**	**3.805**	**37.49**	**47.57**	**60.56**
	Equiv. Refl.: (1,-8,0), (2,1,22), (3,-1,22), (7,1,0), (8,-1,0), (-1,-2,22), (-1,-7,0), (-2,3,22), (-3,2,22) (-7,8,0), (-8,7,0)								
**LiNbO3**	**(1,2,-22)**	**10.503**	**71.08**	**10.7**	**9.9**	**3.805**	**37.49**	**47.57**	**60.56**
	Equiv. Refl.: (1,7,0), (2,-3,-22), (3,-2,-22), (7,-8,0), (8,-7,0), (-1,3,-22), (-1,8,0), (-2,-1,-22), (-3,1,-22) (-7,-1,0), (-8,1,0)								
**LiNbO3**	**(3,-7,-14)**	**10.523**	**71.4**	**10.6**	**9.5**	**3.737**	**35.54**	**46.71**	**58.7**
	Equiv. Refl.: (4,3,-14), (7,-3,-14), (-3,-4,-14), (-4,7,-14), (-7,4,-14)								
**LiNbO3**	**(3,4,14)**	**10.523**	**71.4**	**10.1**	**9.5**	**3.737**	**35.54**	**46.71**	**58.7**
	Equiv. Refl.: (4,-7,14), (7,-4,14), (-3,7,14), (-4,-3,14), (-7,3,14)								

Ei =	11.103	keV						ΔEg	ΔEt
Cryst	Refl	EB	ΘB	∫ IR dΘ	Width	E cotΘ	ΔE	2m	2m
	(h,k,l)	[keV]	[°]	[μrad]	[μrad]	[keV]	[meV]	[meV]	[meV]
**Quartz**	**(0,5,7)**	**10.841**	**77.54**	**9.6**	**7.6**	**2.454**	**18.56**	**30.67**	**35.85**
	Equiv. Refl.: (0,-5,-7), (5,0,-7), (5,-5,7), (8,-1,-1), (8,-7,1), (-1,8,1), (-1,-7,-1), (-5,0,7), (-5,5,-7) (-7,8,-1), (-7,-1,1)								
**Quartz**	**(1,7,1)**	**11.06**	**84.93**	**9.4**	**8.5**	**0.984**	**8.41**	**12.3**	**14.9**
	Equiv. Refl.: (1,-8,-1), (7,1,-1), (7,-8,1), (-8,1,1), (-8,7,-1)								
**Quartz**	**(0,4,8)**	**10.87**	**78.25**	**9.2**	**7.5**	**2.309**	**17.25**	**28.86**	**33.62**
	Equiv. Refl.: (0,-4,-8), (4,0,-8), (4,-4,8), (8,-1,0), (8,-7,0), (-1,8,0), (-1,-7,0), (-4,0,8), (-4,4,-8) (-7,8,0), (-7,-1,0)								
**Sapph**	**(2,4,16)**	**11.031**	**83.49**	**9.1**	**8.3**	**1.268**	**10.48**	**15.84**	**19**
	Equiv. Refl.: (2,-6,-16), (4,2,-16), (4,-6,16), (6,-2,-16), (6,-4,16), (-2,6,16), (-2,-4,-16), (-4,6,-16), (-4,-2,16) (-6,2,16), (-6,4,-16)								
**Quartz**	**(1,7,0)**	**11**	**82.19**	**9**	**7.6**	**1.523**	**11.65**	**19.04**	**22.32**
	Equiv. Refl.: (1,-8,0), (7,1,0), (7,-8,0), (-8,1,0), (-8,7,0)								
**Quartz**	**(3,5,-3)**	**10.764**	**75.8**	**8.7**	**6.8**	**2.809**	**19.2**	**35.12**	**40.03**
	Equiv. Refl.: (3,-8,3), (5,3,3), (5,-8,-3), (8,-3,3), (8,-5,-3), (-3,8,-3), (-3,-5,3), (-5,8,3), (-5,-3,-3) (-8,3,-3), (-8,5,3)								
**LiNbO3**	**(0,0,-24)**	**10.732**	**75.15**	**8.3**	**7.8**	**2.944**	**23.01**	**36.79**	**43.4**
**Quartz**	**(8,-2,-3)**	**11.056**	**84.71**	**8.1**	**7.2**	**1.029**	**7.36**	**12.86**	**14.82**
	Equiv. Refl.: (8,-6,3), (-2,8,3), (-2,-6,-3), (-6,8,-3), (-6,-2,3)								
**Sapph**	**(1,5,14)**	**10.715**	**74.81**	**7.9**	**6.5**	**3.015**	**19.46**	**37.68**	**42.41**
	Equiv. Refl.: (1,-6,-14), (5,1,-14), (5,-6,14), (6,-1,-14), (6,-5,14), (-1,6,14), (-1,-5,-14), (-5,6,-14), (-5,-1,14) (-6,1,14), (-6,5,-14)								
**Sapph**	**(3,5,4)**	**10.703**	**74.57**	**7.8**	**6.4**	**3.065**	**19.68**	**38.32**	**43.08**
	Equiv. Refl.: (3,-8,-4), (5,3,-4), (5,-8,4), (8,-3,-4), (8,-5,4), (-3,8,4), (-3,-5,-4), (-5,8,-4), (-5,-3,4) (-8,3,4), (-8,5,-4)								
**Quartz**	**(8,-1,1)**	**11.06**	**84.93**	**7.6**	**6.9**	**0.984**	**6.76**	**12.3**	**14.04**
	Equiv. Refl.: (8,-7,-1), (-1,8,-1), (-1,-7,1), (-7,8,1), (-7,-1,-1)								
**Quartz**	**(2,6,3)**	**11.056**	**84.71**	**7.4**	**7.2**	**1.029**	**7.37**	**12.86**	**14.82**
	Equiv. Refl.: (2,-8,-3), (6,2,-3), (6,-8,3), (-8,2,3), (-8,6,-3)								
**LiNbO3**	**(0,0,24)**	**10.732**	**75.15**	**7.2**	**7.8**	**2.944**	**23.01**	**36.79**	**43.4**
**Sapph**	**(4,4,6)**	**10.809**	**76.79**	**7**	**6**	**2.606**	**15.59**	**32.58**	**36.12**
	Equiv. Refl.: (4,4,-6), (4,-8,6), (4,-8,-6), (8,-4,6), (8,-4,-6), (-4,8,6), (-4,8,-6), (-4,-4,6), (-4,-4,-6) (-8,4,6), (-8,4,-6), (1,7,-1), (1,-8,1), (7,1,1), (7,-8,-1), (-8,1,-1), (-8,7,1)								
**Sapph**	**(0,5,16)**	**10.719**	**74.89**	**6.3**	**5.3**	**2.998**	**16.02**	**37.47**	**40.75**
	Equiv. Refl.: (0,-5,-16), (5,0,-16), (5,-5,16), (-5,0,16), (-5,5,-16)								
**Sapph**	**(0,7,2)**	**10.574**	**72.24**	**5.5**	**4.7**	**3.555**	**16.72**	**44.44**	**47.48**
	Equiv. Refl.: (0,-7,-2), (7,0,-2), (7,-7,2), (-7,0,2), (-7,7,-2)								
**Quartz**	**(1,5,6)**	**10.638**	**73.36**	**5.3**	**4.4**	**3.318**	**14.48**	**41.47**	**43.93**
	Equiv. Refl.: (1,-6,-6), (5,1,-6), (5,-6,6), (6,-1,-6), (6,-5,6), (-1,6,6), (-1,-5,-6), (-5,6,-6), (-5,-1,6) (-6,1,6), (-6,5,-6)								
**Quartz**	**(0,2,-9)**	**10.727**	**75.04**	**5.1**	**4.1**	**2.967**	**12.19**	**37.09**	**39.04**
	Equiv. Refl.: (0,-2,9), (2,0,9), (2,-2,-9), (8,-2,-2), (8,-6,2), (-2,0,-9), (-2,2,9), (-2,8,2), (-2,-6,-2) (-6,8,-2), (-6,-2,2)								
**Quartz**	**(2,6,2)**	**10.754**	**75.6**	**5**	**4.1**	**2.852**	**11.63**	**35.64**	**37.49**
	Equiv. Refl.: (2,-8,-2), (6,2,-2), (6,-8,2), (-8,2,2), (-8,6,-2)								
**Quartz**	**(2,6,1)**	**10.569**	**72.16**	**4.9**	**3.9**	**3.574**	**13.97**	**44.68**	**46.81**
	Equiv. Refl.: (2,-8,-1), (6,2,-1), (6,-8,1), (8,-2,-1), (8,-6,1), (-2,8,1), (-2,-6,-1), (-6,8,-1), (-6,-2,1) (-8,2,1), (-8,6,-1)								

Ei =	11.103	keV						ΔEg	ΔEt
**Cryst**	**Refl**	**EB**	**ΘB**	**∫ IR dΘ**	**Width**	**E cotΘ**	**ΔE**	**2m**	**2m**
	**(h,k,l)**	**[keV]**	**[°]**	**[μrad]**	**[μrad]**	**[keV]**	**[meV]**	**[meV]**	**[meV]**
**Sapph**	**(0,6,12)**	**10.69**	**74.33**	**4.8**	**4.1**	**3.115**	**12.79**	**38.94**	**40.98**
	Equiv. Refl.: (0,6,-12), (0,-6,12), (0,-6,-12), (6,0,12), (6,0,-12), (6,-6,12), (6,-6,-12), (-6,0,12), (-6,0,-12) (-6,6,12), (-6,6,-12)								
**Quartz**	**(0,6,5)**	**10.455**	**70.33**	**4.6**	**3.7**	**3.968**	**14.54**	**49.6**	**51.69**
	Equiv. Refl.: (0,-6,-5), (6,0,-5), (6,-6,5), (-6,0,5), (-6,6,-5)								
**LiNbO3**	**(4,5,5)**	**11.088**	**87**	**4.3**	**9.4**	**0.581**	**5.47**	**7.26**	**9.09**
	Equiv. Refl.: (4,-9,-5), (5,4,-5), (5,-9,5), (9,-4,-5), (9,-5,5), (-4,9,5), (-4,-5,-5), (-5,9,-5), (-5,-4,5) (-9,4,5), (-9,5,-5)								
**Quartz**	**(1,6,-4)**	**10.599**	**72.66**	**4**	**3.2**	**3.466**	**11.04**	**43.32**	**44.71**
	Equiv. Refl.: (1,-7,4), (4,4,3), (4,4,-3), (4,-8,3), (4,-8,-3), (6,1,4), (6,-7,-4), (-7,1,-4), (-7,6,4) (-8,4,3), (-8,4,-3)								
**Sapph**	**(2,6,-1)**	**10.859**	**77.97**	**3.9**	**3.6**	**2.367**	**8.57**	**29.58**	**30.8**
	Equiv. Refl.: (2,-8,1), (6,2,1), (6,-8,-1), (8,-2,1), (8,-6,-1), (-2,8,-1), (-2,-6,1), (-6,8,1), (-6,-2,-1) (-8,2,-1), (-8,6,1)								
**Quartz**	**(8,-4,3)**	**10.665**	**73.85**	**3.8**	**3.3**	**3.216**	**10.53**	**40.2**	**41.55**
	Equiv. Refl.: (8,-4,-3), (-4,8,3), (-4,8,-3), (-4,-4,3), (-4,-4,-3)								
**Sapph**	**(1,6,-8)**	**10.578**	**72.32**	**3.7**	**3.3**	**3.54**	**11.51**	**44.25**	**45.72**
	Equiv. Refl.: (1,-7,8), (3,5,7), (3,-8,-7), (5,3,-7), (5,-8,7), (6,1,8), (6,-7,-8), (7,-1,8), (7,-6,-8) (8,-3,-7), (8,-5,7), (-1,7,-8), (-1,-6,8), (-3,8,7), (-3,-5,-7), (-5,8,-7), (-5,-3,7), (-6,7,8) (-6,-1,-8), (-7,1,-8), (-7,6,8), (-8,3,7), (-8,5,-7), (7,-1,4), (7,-6,-4), (-1,7,-4), (-1,-6,4) (-6,7,4), (-6,-1,-4)								
**Quartz**	**(1,3,-8)**	**10.573**	**72.23**	**3.4**	**2.8**	**3.558**	**9.93**	**44.47**	**45.57**
	Equiv. Refl.: (1,-4,8), (3,1,8), (3,5,-2), (3,-4,-8), (3,-8,2), (4,-1,8), (4,-3,-8), (5,3,2), (5,-8,-2) (8,-3,2), (8,-5,-2), (-1,4,-8), (-1,-3,8), (-3,4,8), (-3,8,-2), (-3,-1,-8), (-3,-5,2), (-4,1,-8) (-4,3,8), (-5,8,2), (-5,-3,-2), (-8,3,-2), (-8,5,2)								
**Quartz**	**(7,-3,-5)**	**10.556**	**71.95**	**3.3**	**3**	**3.619**	**10.8**	**45.24**	**46.51**
	Equiv. Refl.: (7,-4,5), (-3,7,5), (-3,-4,-5), (-4,7,-5), (-4,-3,5)								
**Quartz**	**(3,4,5)**	**10.556**	**71.95**	**3.2**	**3**	**3.619**	**10.8**	**45.24**	**46.51**
	Equiv. Refl.: (3,5,3), (3,-7,-5), (3,-8,-3), (4,3,-5), (4,-7,5), (5,3,-3), (5,-8,3), (-7,3,5), (-7,4,-5) (-8,3,3), (-8,5,-3)								
**Quartz**	**(8,-3,-3)**	**10.764**	**75.8**	**3**	**2.9**	**2.809**	**8.13**	**35.12**	**36.05**
	Equiv. Refl.: (8,-5,3), (-3,8,3), (-3,-5,-3), (-5,8,-3), (-5,-3,3)								
**Quartz**	**(1,2,-9)**	**11.019**	**82.96**	**2.9**	**2.9**	**1.37**	**3.99**	**17.13**	**17.59**
	Equiv. Refl.: (1,-3,9), (2,1,9), (2,-3,-9), (-3,1,-9), (-3,2,9)								
**LiNbO3**	**(1,6,13)**	**10.814**	**76.89**	**2.7**	**4.1**	**2.586**	**10.69**	**32.33**	**34.05**
	Equiv. Refl.: (1,-7,-13), (6,1,-13), (6,-7,13), (7,-1,-13), (7,-6,13), (-1,7,13), (-1,-6,-13), (-6,7,-13), (-6,-1,13) (-7,1,13), (-7,6,-13), (8,-2,1), (8,-6,-1), (-2,8,-1), (-2,-6,1), (-6,8,1), (-6,-2,-1)								
**Quartz**	**(2,6,-1)**	**10.569**	**72.16**	**2.6**	**2.5**	**3.574**	**8.99**	**44.68**	**45.57**
	Equiv. Refl.: (2,-8,1), (6,2,1), (6,-8,-1), (-8,2,-1), (-8,6,1)								
**Sapph**	**(4,4,3)**	**10.521**	**71.36**	**2.4**	**2.4**	**3.744**	**8.85**	**46.81**	**47.63**
	Equiv. Refl.: (4,4,-3), (4,-8,3), (4,-8,-3), (8,-4,3), (8,-4,-3), (-4,8,3), (-4,8,-3), (-4,-4,3), (-4,-4,-3) (-8,4,3), (-8,4,-3), (1,1,9), (1,1,-9), (1,5,-6), (1,-2,9), (1,-2,-9), (1,-6,6), (2,5,-5) (2,-7,5), (5,1,6), (5,2,5), (5,-6,-6), (5,-7,-5), (6,-1,6), (6,-5,-6), (-1,6,-6), (-1,-5,6) (-2,1,9), (-2,1,-9), (-5,6,6), (-5,-1,-6), (-6,1,-6), (-6,5,6), (-7,2,-5), (-7,5,5)								
**Quartz**	**(3,-1,9)**	**11.019**	**82.96**	**2.2**	**2.9**	**1.37**	**3.98**	**17.13**	**17.58**
	Equiv. Refl.: (3,-2,-9), (-1,3,-9), (-1,-2,9), (-2,3,9), (-2,-1,-9)								
**Quartz**	**(1,4,7)**	**10.443**	**70.14**	**1.9**	**2.1**	**4.011**	**8.39**	**50.14**	**50.83**
	Equiv. Refl.: (1,-5,-7), (2,-1,9), (2,-1,-9), (4,1,-7), (4,-5,7), (5,-1,-7), (5,-4,7), (7,-2,5), (7,-5,-5) (-1,2,9), (-1,2,-9), (-1,5,7), (-1,-1,9), (-1,-1,-9), (-1,-4,-7), (-2,7,-5), (-2,-5,5), (-4,5,-7) (-4,-1,7), (-5,1,7), (-5,4,-7), (-5,7,5), (-5,-2,-5)								

Ei =	11.103	keV						ΔEg	ΔEt
Cryst	Refl	EB	ΘB	∫ IR dΘ	Width	E cotΘ	ΔE	2m	2m
	(h,k,l)	[keV]	[°]	[μrad]	[μrad]	[keV]	[meV]	[meV]	[meV]
**Quartz**	**(0,2,9)**	**10.727**	**75.04**	**1.8**	**2**	**2.967**	**6.05**	**37.09**	**37.58**
	Equiv. Refl.: (0,-2,-9), (2,0,-9), (2,-2,9), (3,5,2), (3,-8,-2), (5,3,-2), (5,-8,2), (8,-3,-2), (8,-5,2) (-2,0,9), (-2,2,-9), (-3,8,2), (-3,-5,-2), (-5,8,-2), (-5,-3,2), (-8,3,2), (-8,5,-2)								
**LiNbO3**	**(2,4,-17)**	**10.58**	**72.34**	**1.7**	**2.8**	**3.536**	**9.8**	**44.19**	**45.27**
	Equiv. Refl.: (2,-6,17), (4,2,17), (4,-6,-17), (6,-2,17), (6,-4,-17), (-2,6,-17), (-2,-4,17), (-4,6,17), (-4,-2,-17) (-6,2,-17), (-6,4,17), (2,6,-2), (2,-8,2), (6,2,2), (6,-8,-2), (-8,2,-2), (-8,6,2)								
**Quartz**	**(0,7,-2)**	**10.454**	**70.31**	**1.6**	**1.8**	**3.974**	**6.96**	**49.67**	**50.16**
	Equiv. Refl.: (0,-7,2), (7,0,2), (7,-7,-2), (-7,0,-2), (-7,7,2)								
**LiNbO3**	**(2,2,21)**	**10.554**	**71.91**	**1.5**	**2.6**	**3.628**	**9.44**	**45.34**	**46.32**
	Equiv. Refl.: (2,2,-21), (2,-4,21), (2,-4,-21), (4,-2,21), (4,-2,-21), (-2,4,21), (-2,4,-21), (-2,-2,21), (-2,-2,-21) (-4,2,21), (-4,2,-21), (2,6,0), (2,-8,0), (5,-1,7), (5,-4,-7), (6,2,0), (6,-8,0), (8,-2,2) (8,-6,-2), (-1,5,-7), (-1,-4,7), (-2,8,-2), (-2,-6,2), (-4,5,7), (-4,-1,-7), (-6,8,2), (-6,-2,-2) (-8,2,0), (-8,6,0)								
**Sapph**	**(1,4,18)**	**11.015**	**82.8**	**1.4**	**2.1**	**1.403**	**2.95**	**17.54**	**17.79**
	Equiv. Refl.: (1,4,-18), (1,-5,18), (1,-5,-18), (3,5,-5), (3,-8,5), (4,1,18), (4,1,-18), (4,-5,18), (4,-5,-18) (5,3,5), (5,-1,18), (5,-1,-18), (5,-4,18), (5,-4,-18), (5,-8,-5), (8,-3,5), (8,-5,-5), (-1,5,18) (-1,5,-18), (-1,-4,18), (-1,-4,-18), (-3,8,-5), (-3,-5,5), (-4,5,18), (-4,5,-18), (-4,-1,18), (-4,-1,-18) (-5,1,18), (-5,1,-18), (-5,4,18), (-5,4,-18), (-5,8,5), (-5,-3,-5), (-8,3,-5), (-8,5,5), (1,2,9) (1,3,8), (1,4,-7), (1,-3,-9), (1,-4,-8), (1,-5,7), (2,1,-9), (2,-3,9), (3,1,-8), (3,-4,8) (4,1,7), (4,-1,-8), (4,-3,8), (4,-5,-7), (-1,4,8), (-1,-3,-8), (-3,1,9), (-3,2,-9), (-3,4,-8) (-3,-1,8), (-4,1,8), (-4,3,-8), (-5,1,-7), (-5,4,7)								
**Quartz**	**(0,7,3)**	**10.764**	**75.8**	**1.3**	**1.6**	**2.809**	**4.4**	**35.12**	**35.39**
	Equiv. Refl.: (0,-7,-3), (3,-1,-9), (3,-2,9), (7,0,-3), (7,-7,3), (8,-2,0), (8,-6,0), (-1,3,9), (-1,-2,-9) (-2,3,-9), (-2,8,0), (-2,-1,9), (-2,-6,0), (-6,8,0), (-6,-2,0), (-7,0,3), (-7,7,-3)								
**LiNbO3**	**(4,5,-1)**	**10.869**	**78.22**	**1.2**	**2.5**	**2.315**	**5.83**	**28.94**	**29.52**
	Equiv. Refl.: (4,-9,1), (5,4,1), (5,-9,-1), (9,-4,1), (9,-5,-1), (-4,9,-1), (-4,-5,1), (-5,9,1), (-5,-4,-1) (-9,4,-1), (-9,5,1), (2,5,12), (2,5,-12), (2,-7,12), (2,-7,-12), (5,2,12), (5,2,-12), (5,-7,12) (5,-7,-12), (7,-2,12), (7,-2,-12), (7,-5,12), (7,-5,-12), (-2,7,12), (-2,7,-12), (-2,-5,12), (-2,-5,-12) (-5,7,12), (-5,7,-12), (-5,-2,12), (-5,-2,-12), (-7,2,12), (-7,2,-12), (-7,5,12), (-7,5,-12), (0,7,-3) (0,-7,3), (3,4,-5), (3,-7,5), (4,3,5), (4,-2,8), (4,-2,-8), (4,-7,-5), (7,0,3), (7,-7,-3) (-2,4,8), (-2,4,-8), (-2,-2,8), (-2,-2,-8), (-7,0,-3), (-7,3,-5), (-7,4,5), (-7,7,3)								
**Sapph**	**(3,5,1)**	**10.542**	**71.7**	**1.1**	**1.4**	**3.671**	**5.23**	**45.89**	**46.18**
	Equiv. Refl.: (3,-8,-1), (5,3,-1), (5,-8,1), (8,-3,-1), (8,-5,1), (-3,8,1), (-3,-5,-1), (-5,8,-1), (-5,-3,1) (-8,3,1), (-8,5,-1), (2,2,8), (2,2,-8), (2,-4,8), (2,-4,-8), (-4,2,8), (-4,2,-8)								
**Quartz**	**(1,6,4)**	**10.599**	**72.66**	**1**	**1.2**	**3.466**	**4.22**	**43.32**	**43.53**
	Equiv. Refl.: (1,-7,-4), (6,1,-4), (6,-7,4), (7,-1,-4), (7,-3,5), (7,-4,-5), (7,-6,4), (-1,7,4), (-1,-6,-4) (-3,7,-5), (-3,-4,5), (-4,7,5), (-4,-3,-5), (-6,7,-4), (-6,-1,4), (-7,1,4), (-7,6,-4)								
**LiNbO3**	**(1,2,23)**	**10.923**	**79.68**	**0.9**	**2.3**	**2.023**	**4.75**	**25.28**	**25.73**
	Equiv. Refl.: (1,5,17), (1,-3,-23), (1,-6,-17), (2,1,-23), (2,-3,23), (3,-1,-23), (3,-2,23), (5,1,-17), (5,-6,17) (6,-1,-17), (6,-5,17), (-1,3,23), (-1,6,17), (-1,-2,-23), (-1,-5,-17), (-2,3,-23), (-2,-1,23), (-3,1,23) (-3,2,-23), (-5,6,-17), (-5,-1,17), (-6,1,17), (-6,5,-17), (0,6,-5), (0,-6,5), (6,0,5), (6,-6,-5) (-6,0,-5), (-6,6,5)								
**Quartz**	**(3,3,-7)**	**11.036**	**83.68**	**0.8**	**1.1**	**1.23**	**1.39**	**15.37**	**15.43**
**LiNbO3**	**(2,5,15)**	**10.973**	**81.21**	**0.7**	**2.1**	**1.717**	**3.63**	**21.46**	**21.77**
	Equiv. Refl.: (2,5,-15), (2,-7,15), (2,-7,-15), (5,2,15), (5,2,-15), (5,-7,15), (5,-7,-15), (7,-2,15), (7,-2,-15) (7,-5,15), (7,-5,-15), (-2,7,15), (-2,7,-15), (-2,-5,15), (-2,-5,-15), (-5,7,15), (-5,7,-15), (-5,-2,15) (-5,-2,-15), (-7,2,15), (-7,2,-15), (-7,5,15), (-7,5,-15), (0,2,22), (0,7,-4), (0,-2,-22), (0,-7,4) (2,0,-22), (2,-2,22), (7,0,4), (7,-7,-4), (-2,0,22), (-2,2,-22), (-7,0,-4), (-7,7,4), (0,4,-8) (0,5,-7), (0,-4,8), (0,-5,7), (3,3,7), (3,-6,7), (3,-6,-7), (4,0,8), (4,-4,-8), (5,0,7) (5,-5,-7), (7,-2,-5), (7,-5,5), (-2,7,5), (-2,-5,-5), (-4,0,-8), (-4,4,8), (-5,0,-7), (-5,5,7) (-5,7,-5), (-5,-2,5), (-6,3,7), (-6,3,-7)								

Ei = Cryst	11.103 Refl (h,k,l)	keV EB [keV]	ΘB [°]	∫ IR dΘ [µrad]	Width [µrad]	E cotΘ [keV]	ΔE [meV]	ΔEg 2m [meV]	ΔEt 2m [meV]
**Quartz**	**(2,5,5)**	**10.756**	**75.63**	**0.6**	**0.9**	**2.845**	**2.55**	**35.56**	**35.65**
	Equiv. Refl.: (2,-7,-5), (5,2,-5), (5,-7,5), (-7,2,5), (-7,5,-5)								
**Sapph**	**(1,3,19)**	**10.567**	**72.12**	**0.5**	**0.8**	**3.581**	**2.81**	**44.76**	**44.85**
	Equiv. Refl.: (1,-4,-19), (3,1,-19), (3,4,-13), (3,-4,19), (3,-7,13), (4,3,13), (4,-1,-19), (4,-3,19), (4,-7,-13) (7,-3,13), (7,-4,-13), (-1,4,19), (-1,-3,-19), (-3,4,-19), (-3,7,-13), (-3,-1,19), (-3,-4,13), (-4,1,19) (-4,3,-19), (-4,7,13), (-4,-3,-13), (-7,3,-13), (-7,4,13), (6,-3,7), (6,-3,-7), (-3,6,7), (-3,6,-7) (-3,-3,7), (-3,-3,-7)								
**LiNbO3**	**(2,6,-7)**	**10.504**	**71.1**	**0.4**	**1.1**	**3.802**	**4.06**	**47.53**	**47.7**
	Equiv. Refl.: (2,-8,7), (6,2,7), (6,-8,-7), (8,-2,7), (8,-6,-7), (-2,8,-7), (-2,-6,7), (-6,8,7), (-6,-2,-7) (-8,2,-7), (-8,6,7), (0,7,2), (0,-7,-2), (7,0,-2), (7,-7,2), (-7,0,2), (-7,7,-2)								
**LiNbO3**	**(3,5,-11)**	**10.906**	**79.18**	**0.3**	**1.2**	**2.121**	**2.61**	**26.52**	**26.64**
	Equiv. Refl.: (3,-8,11), (5,3,11), (5,-8,-11), (8,-3,11), (8,-5,-11), (-3,8,-11), (-3,-5,11), (-5,8,11), (-5,-3,-11) (-8,3,-11), (-8,5,11), (0,1,-22), (0,-1,22), (1,0,22), (1,-1,-22), (-1,0,-22), (-1,1,22)								
**LiNbO3**	**(1,7,3)**	**10.583**	**72.4**	**0.2**	**0.9**	**3.522**	**3.19**	**44.02**	**44.14**
	Equiv. Refl.: (1,7,-3), (1,-8,3), (1,-8,-3), (2,3,-19), (2,-5,19), (3,2,19), (3,-5,-19), (5,-2,19), (5,-3,-19) (7,1,3), (7,1,-3), (7,-8,3), (7,-8,-3), (8,-1,3), (8,-1,-3), (8,-7,3), (8,-7,-3), (-1,8,3) (-1,8,-3), (-1,-7,3), (-1,-7,-3), (-2,5,-19), (-2,-3,19), (-3,5,19), (-3,-2,-19), (-5,2,-19), (-5,3,19) (-7,8,3), (-7,8,-3), (-7,-1,3), (-7,-1,-3), (-8,1,3), (-8,1,-3), (-8,7,3), (-8,7,-3), (2,6,2) (2,-8,-2), (3,4,11), (3,-7,-11), (4,3,-11), (4,-7,11), (6,2,-2), (6,-8,2), (7,-3,-11), (7,-4,11) (8,-2,-2), (8,-6,2), (-2,8,2), (-2,-6,-2), (-3,7,11), (-3,-4,-11), (-4,7,-11), (-4,-3,11), (-6,8,-2) (-6,-2,2), (-7,3,11), (-7,4,-11), (-8,2,2), (-8,6,-2)								
**LiNbO3**	**(4,4,9)**	**10.44**	**70.11**	**0**	**0.2**	**4.018**	**0.94**	**50.22**	**50.23**
	Equiv. Refl.: (4,4,-9), (4,-8,9), (4,-8,-9), (8,-4,9), (8,-4,-9), (-4,8,9), (-4,8,-9), (-4,-4,9), (-4,-4,-9) (-8,4,9), (-8,4,-9), (3,3,15), (3,3,-15), (3,-6,15), (3,-6,-15), (6,-3,15), (6,-3,-15), (-3,6,15) (-3,6,-15), (-3,-3,15), (-3,-3,-15), (-6,3,15), (-6,3,-15)								

Ei =	11.867	keV							
Cryst	Refl (h,k,l)	EB [keV]	ΘB [°]	∫IR dΘ [μrad]	Width [μrad]	E cotΘ [keV]	ΔE [meV]	ΔEg 2m [meV]	ΔEt 2m [meV]
**Si**	**(1,5,9)**	**11.807**	**84.25**	**7.712**	**7.2**	**1.196**	**8.64**	**14.95**	**17.27**
	Equiv. Refl.: (3,7,7)								
**Si**	**(0,2,10)**	**11.64**	**78.79**	**6.388**	**5.5**	**2.352**	**12.98**	**29.4**	**32.14**
	Equiv. Refl.: (2,6,8)								
**Si**	**(4,4,8)**	**11.184**	**70.46**	**4.464**	**3.6**	**4.211**	**15.36**	**52.63**	**54.83**
**Si**	**(1,7,7)**	**11.357**	**73.15**	**3.368**	**2.8**	**3.595**	**10.19**	**44.94**	**46.08**
	Equiv. Refl.: (3,3,9), (5,5,7)								
**Ge**	**(3,5,9)**	**11.751**	**81.98**	**3.178**	**9**	**1.672**	**15.08**	**20.9**	**25.78**
**Ge**	**(0,2,10)**	**11.175**	**70.33**	**3.089**	**6.3**	**4.241**	**26.52**	**53.01**	**59.28**
	Equiv. Refl.: (2,6,8)								
**Ge**	**(1,5,9)**	**11.335**	**72.78**	**1.781**	**4.8**	**3.679**	**17.64**	**45.99**	**49.25**
	Equiv. Refl.: (3,7,7)								
**LiNbO3**	**(2,5,18)**	**11.84**	**86.16**	**39.5**	**36.5**	**0.797**	**29.06**	**9.96**	**30.72**
	Equiv. Refl.: (5,-7,18), (7,-5,18), (-5,-2,18)								
**LiNbO3**	**(-2,7,18)**	**11.84**	**86.16**	**39.4**	**36.5**	**0.797**	**29.06**	**9.96**	**30.72**
	Equiv. Refl.: (-7,2,18)								
**LiNbO3**	**(2,-7,-18)**	**11.84**	**86.16**	**39.2**	**36.5**	**0.797**	**29.06**	**9.96**	**30.72**
	Equiv. Refl.: (5,2,-18), (7,-2,-18), (-2,-5,-18), (-5,7,-18), (-7,5,-18)								
**LiNbO3**	**(2,-7,18)**	**11.84**	**86.16**	**38.1**	**35.3**	**0.797**	**28.1**	**9.96**	**29.81**
	Equiv. Refl.: (5,2,18), (7,-2,18), (-2,-5,18), (-5,7,18), (-7,5,18)								
**LiNbO3**	**(2,5,-18)**	**11.84**	**86.16**	**37.6**	**35.3**	**0.797**	**28.1**	**9.96**	**29.81**
	Equiv. Refl.: (5,-7,-18), (7,-5,-18), (-2,7,-18), (-5,-2,-18), (-7,2,-18)								
**LiNbO3**	**(2,6,14)**	**11.821**	**84.95**	**30.9**	**28.1**	**1.049**	**29.45**	**13.12**	**32.24**
	Equiv. Refl.: (6,-8,14), (8,-6,14), (-2,8,14), (-6,-2,14), (-8,2,14)								
**LiNbO3**	**(2,-8,-14)**	**11.821**	**84.95**	**29.7**	**28.1**	**1.049**	**29.45**	**13.12**	**32.24**
	Equiv. Refl.: (6,2,-14), (8,-2,-14), (-2,-6,-14), (-6,8,-14), (-8,6,-14)								
**LiNbO3**	**(1,5,20)**	**11.829**	**85.41**	**29.2**	**27.5**	**0.953**	**26.17**	**11.91**	**28.75**
	Equiv. Refl.: (5,-6,20), (6,-5,20), (-1,6,20), (-5,-1,20), (-6,1,20)								
**LiNbO3**	**(1,-6,-20)**	**11.829**	**85.41**	**29**	**27.5**	**0.953**	**26.17**	**11.91**	**28.75**
	Equiv. Refl.: (5,1,-20), (6,-1,-20), (-1,-5,-20), (-5,6,-20), (-6,5,-20)								
**LiNbO3**	**(1,-8,12)**	**11.79**	**83.47**	**22.9**	**21.1**	**1.359**	**28.63**	**16.99**	**33.29**
	Equiv. Refl.: (7,1,12), (8,-1,12), (-1,-7,12), (-7,8,12), (-8,7,12)								
**LiNbO3**	**(1,7,-12)**	**11.79**	**83.47**	**22.4**	**21.1**	**1.359**	**28.63**	**16.99**	**33.29**
	Equiv. Refl.: (7,-8,-12), (8,-7,-12), (-1,8,-12), (-7,-1,-12), (-8,1,-12)								
**LiNbO3**	**(2,2,24)**	**11.764**	**82.43**	**22.1**	**20.2**	**1.577**	**31.83**	**19.71**	**37.44**
	Equiv. Refl.: (2,-4,24), (4,-2,24), (-2,4,24), (-2,-2,24), (-4,2,24)								
**LiNbO3**	**(2,2,-24)**	**11.764**	**82.43**	**22**	**20.2**	**1.577**	**31.83**	**19.71**	**37.44**
	Equiv. Refl.: (2,-4,-24), (4,-2,-24), (-2,4,-24), (-2,-2,-24), (-4,2,-24)								
**LiNbO3**	**(1,7,12)**	**11.79**	**83.47**	**20.3**	**19.1**	**1.359**	**25.91**	**16.99**	**30.99**
	Equiv. Refl.: (7,-8,12), (8,-7,12), (-1,8,12), (-7,-1,12), (-8,1,12)								
**LiNbO3**	**(1,-8,-12)**	**11.79**	**83.47**	**19.9**	**19.1**	**1.359**	**25.91**	**16.99**	**30.99**
	Equiv. Refl.: (7,1,-12), (8,-1,-12), (-1,-7,-12), (-7,8,-12), (-8,7,-12)								
**LiNbO3**	**(0,8,-8)**	**11.685**	**79.95**	**19.5**	**17**	**2.103**	**35.84**	**26.28**	**44.45**
	Equiv. Refl.: (8,-8,-8), (-8,0,-8)								
**LiNbO3**	**(0,-8,8)**	**11.685**	**79.95**	**18.3**	**17**	**2.103**	**35.84**	**26.28**	**44.45**
	Equiv. Refl.: (8,0,8), (-8,8,8)								
**LiNbO3**	**(4,5,-10)**	**11.745**	**81.77**	**18.1**	**16.3**	**1.717**	**28.07**	**21.46**	**35.34**
	Equiv. Refl.: (5,-9,-10), (9,-5,-10), (-4,9,-10), (-5,-4,-10), (-9,4,-10)								

Ei =	11.867	keV						ΔEg	ΔEt
Cryst	Refl	EB	ΘB	∫ IR dΘ	Width	E cotΘ	ΔE	2m	2m
	(h,k,l)	[keV]	[°]	[μrad]	[μrad]	[keV]	[meV]	[meV]	[meV]
LiNbO3	(4,-9,10)	11.745	81.77	16.9	16.3	1.717	28.07	21.46	35.34
	Equiv. Refl.: (5,4,10), (9,-4,10), (-4,-5,10), (-5,9,10), (-9,5,10)								
Quartz	(4,0,9)	11.855	87.39	15.5	13.5	0.542	7.32	6.77	9.98
	Equiv. Refl.: (4,-4,-9), (-4,0,-9), (-4,4,9)								
Quartz	(0,4,-9)	11.855	87.39	15.4	13.5	0.542	7.31	6.77	9.97
	Equiv. Refl.: (0,-4,9)								
Sapph	(2,6,-10)	11.852	87.12	14.8	13.4	0.596	7.97	7.45	10.91
	Equiv. Refl.: (2,-8,10), (6,2,10), (6,-8,-10), (8,-2,10), (8,-6,-10), (-2,8,-10), (-2,-6,10), (-6,8,10), (-6,-2,-10) (-8,2,-10), (-8,6,10)								
LiNbO3	(0,1,26)	11.709	80.65	14.4	13.6	1.953	26.48	24.42	36.02
	Equiv. Refl.: (1,-1,26), (-1,0,26)								
LiNbO3	(0,-1,-26)	11.709	80.65	14.2	13.6	1.953	26.48	24.42	36.02
	Equiv. Refl.: (0,-6,18), (1,0,-26), (6,0,18), (-1,1,-26), (-6,6,18)								
LiNbO3	(0,6,-18)	11.593	77.66	14	12.9	2.596	33.44	32.45	46.59
	Equiv. Refl.: (2,-5,22), (3,2,22), (5,-2,22), (6,-6,-18), (-2,-3,22), (-3,5,22), (-5,3,22), (-6,0,-18)								
LiNbO3	(3,-8,14)	11.573	77.22	13.6	12	2.692	32.25	33.65	46.61
	Equiv. Refl.: (5,3,14), (8,-3,14), (-3,-5,14), (-5,8,14), (-8,5,14)								
LiNbO3	(2,3,-22)	11.555	76.83	13.5	12.5	2.776	34.69	34.7	49.07
	Equiv. Refl.: (3,-5,-22), (5,-3,-22), (-2,5,-22), (-3,-2,-22), (-5,2,-22)								
LiNbO3	(0,6,18)	11.593	77.66	13	11.8	2.596	30.66	32.45	44.64
	Equiv. Refl.: (6,-6,18), (-6,0,18)								
LiNbO3	(2,-6,20)	11.581	77.4	12.9	11.8	2.653	31.22	33.16	45.54
	Equiv. Refl.: (4,2,20), (6,-2,20), (-2,-4,20), (-4,6,20), (-6,4,20)								
LiNbO3	(0,-8,-4)	11.267	71.7	12.7	11.1	3.925	43.7	49.06	65.7
	Equiv. Refl.: (1,6,16), (6,-7,16), (7,-6,16), (8,0,-4), (-1,7,16), (-6,-1,16), (-7,1,16), (-8,8,-4)								
LiNbO3	(2,4,-20)	11.581	77.4	12.6	11.8	2.653	31.22	33.16	45.54
	Equiv. Refl.: (4,-6,-20), (6,-4,-20), (-2,6,-20), (-4,-2,-20), (-6,2,-20), (1,1,24), (1,1,-24), (1,-2,24), (1,-2,-24) (2,-1,24), (2,-1,-24), (-1,2,24), (-1,2,-24), (-1,-1,24), (-1,-1,-24), (-2,1,24), (-2,1,-24)								
LiNbO3	(0,-6,-18)	11.593	77.66	12.5	11.8	2.596	30.66	32.45	44.64
	Equiv. Refl.: (3,5,-14), (5,-8,-14), (6,0,-18), (8,-5,-14), (-3,8,-14), (-5,-3,-14), (-6,6,-18), (-8,3,-14)								
LiNbO3	(0,8,4)	11.267	71.7	12.4	11.1	3.925	43.7	49.06	65.7
	Equiv. Refl.: (8,-8,4), (-8,0,4)								
LiNbO3	(1,-7,-16)	11.59	77.6	12	11.5	2.61	30	32.62	44.32
	Equiv. Refl.: (6,1,-16), (7,-1,-16), (-1,-6,-16), (-6,7,-16), (-7,6,-16)								
LiNbO3	(2,7,-2)	11.417	74.17	11.7	10.6	3.366	35.84	42.07	55.27
	Equiv. Refl.: (2,-9,2), (7,2,2), (7,-9,-2), (9,-2,2), (9,-7,-2), (-2,9,-2), (-2,-7,2), (-7,9,2), (-7,-2,-2) (-9,2,-2), (-9,7,2)								
LiNbO3	(0,-7,-14)	11.573	77.22	11.2	10.2	2.692	27.47	33.65	43.44
	Equiv. Refl.: (7,0,-14), (-7,7,-14)								
LiNbO3	(0,7,14)	11.573	77.22	10.9	10.2	2.692	27.47	33.65	43.44
	Equiv. Refl.: (7,-7,14), (-7,0,14)								
Quartz	(0,4,9)	11.855	87.39	10.6	9.8	0.542	5.33	6.77	8.62
	Equiv. Refl.: (0,-4,-9), (4,0,-9), (4,-4,9), (-4,0,9), (-4,4,-9)								
Sapph	(0,7,-10)	11.562	76.98	10.5	8.2	2.743	22.57	34.29	41.05
	Equiv. Refl.: (0,-7,10), (7,0,10), (7,-7,-10), (-7,0,-10), (-7,7,10)								
LiNbO3	(2,7,4)	11.521	76.14	10.3	9.3	2.929	27.1	36.61	45.55
	Equiv. Refl.: (3,6,-6), (3,-9,6), (6,3,6), (6,-9,-6), (7,-9,4), (9,-3,6), (9,-6,-6), (9,-7,4), (-2,9,4) (-3,9,-6), (-3,-6,6), (-6,9,6), (-6,-3,-6), (-7,-2,4), (-9,2,4), (-9,3,-6), (-9,6,6)								
LiNbO3	(2,-9,-4)	11.521	76.14	9.7	9.3	2.929	27.1	36.61	45.55
	Equiv. Refl.: (7,2,-4), (9,-2,-4), (-2,-7,-4), (-7,9,-4), (-9,7,-4)								

Ei =	11.867	keV						ΔEg	ΔEt
**Cryst**	**Refl**	**EB**	**ΘB**	**∫IR dΘ**	**Width**	**E cotΘ**	**ΔE**	**2m**	**2m**
	**(h,k,l)**	**[keV]**	**[°]**	**[µrad]**	**[µrad]**	**[keV]**	**[meV]**	**[meV]**	**[meV]**
**LiNbO3**	**(3,-9,-6)**	**11.358**	**73.16**	**9.6**	**8.6**	**3.592**	**30.9**	**44.9**	**54.51**
	Equiv. Refl.: (6,3,-6), (9,-3,-6), (-3,-6,-6), (-6,9,-6), (-9,6,-6)								
**LiNbO3**	**(3,6,6)**	**11.358**	**73.16**	**9.3**	**8.6**	**3.592**	**30.9**	**44.9**	**54.51**
	Equiv. Refl.: (6,-9,6), (9,-6,6), (-3,9,6), (-6,-3,6), (-9,3,6)								
**Quartz**	**(0,7,-5)**	**11.701**	**80.4**	**9.2**	**7.2**	**2.007**	**14.54**	**25.09**	**29**
	Equiv. Refl.: (0,-7,5), (7,0,5), (7,-7,-5), (-7,0,-5), (-7,7,5)								
**LiNbO3**	**(0,4,-22)**	**11.301**	**72.24**	**8.8**	**7.9**	**3.801**	**29.98**	**47.52**	**56.18**
	Equiv. Refl.: (4,-4,-22), (-4,0,-22)								
**LiNbO3**	**(4,5,8)**	**11.434**	**74.48**	**8.5**	**7.9**	**3.296**	**25.94**	**41.2**	**48.69**
	Equiv. Refl.: (5,-9,8), (9,-5,8), (-4,9,8), (-5,-4,8), (-9,4,8)								
**LiNbO3**	**(0,-4,22)**	**11.301**	**72.24**	**8.4**	**7.9**	**3.801**	**29.98**	**47.52**	**56.18**
	Equiv. Refl.: (4,0,22), (-4,4,22)								
**LiNbO3**	**(4,-9,-8)**	**11.434**	**74.48**	**8.2**	**7.9**	**3.296**	**25.94**	**41.2**	**48.69**
	Equiv. Refl.: (5,4,-8), (9,-4,-8), (-4,-5,-8), (-5,9,-8), (-9,5,-8)								
**LiNbO3**	**(0,-3,-24)**	**11.514**	**76**	**8.1**	**7.5**	**2.959**	**22.13**	**36.99**	**43.1**
	Equiv. Refl.: (3,0,-24), (-3,3,-24)								
**LiNbO3**	**(0,3,-24)**	**11.514**	**76**	**7.9**	**7.4**	**2.959**	**21.87**	**36.99**	**42.97**
	Equiv. Refl.: (3,-3,-24), (-3,0,-24), (3,3,18), (3,3,-18), (3,-6,18), (3,-6,-18), (6,-3,18), (6,-3,-18), (-3,6,18) (-3,6,-18), (-3,-3,18), (-3,-3,-18), (-6,3,18), (-6,3,-18), (3,6,-2), (3,-9,2), (6,3,2), (6,-9,-2) (9,-3,2), (9,-6,-2), (-3,9,-2), (-3,-6,2), (-6,9,2), (-6,-3,-2), (-9,3,-2), (-9,6,2)								
**LiNbO3**	**(0,5,-20)**	**11.328**	**72.66**	**7.7**	**6.8**	**3.704**	**25.15**	**46.3**	**52.69**
	Equiv. Refl.: (5,-5,-20), (-5,0,-20)								
**Sapph**	**(1,7,6)**	**11.714**	**80.78**	**7.6**	**6.5**	**1.927**	**12.49**	**24.09**	**27.13**
	Equiv. Refl.: (1,7,-6), (1,-8,6), (1,-8,-6), (7,1,6), (7,1,-6), (7,-8,6), (7,-8,-6), (8,-1,6), (8,-1,-6) (8,-7,6), (8,-7,-6), (-1,8,6), (-1,8,-6), (-1,-7,6), (-1,-7,-6), (-7,8,6), (-7,8,-6), (-7,-1,6) (-7,-1,-6), (-8,1,6), (-8,1,-6), (-8,7,6), (-8,7,-6)								
**LiNbO3**	**(0,3,24)**	**11.514**	**76**	**7.4**	**7.5**	**2.959**	**22.13**	**36.99**	**43.1**
	Equiv. Refl.: (3,-3,24), (-3,0,24)								
**LiNbO3**	**(0,-3,24)**	**11.514**	**76**	**7.3**	**7.4**	**2.959**	**21.87**	**36.99**	**42.97**
	Equiv. Refl.: (3,0,24), (-3,3,24)								
**Quartz**	**(3,6,2)**	**11.79**	**83.46**	**7.2**	**6.2**	**1.36**	**8.37**	**17**	**18.95**
	Equiv. Refl.: (3,-9,-2), (6,3,-2), (6,-9,2), (-9,3,2), (-9,6,-2)								
**Quartz**	**(9,-3,-2)**	**11.79**	**83.46**	**7.1**	**6.2**	**1.36**	**8.37**	**17**	**18.95**
	Equiv. Refl.: (9,-6,2), (-3,9,2), (-3,-6,-2), (-6,9,-2), (-6,-3,2)								
**LiNbO3**	**(0,-5,20)**	**11.328**	**72.66**	**6.7**	**6.8**	**3.704**	**25.15**	**46.3**	**52.69**
	Equiv. Refl.: (5,0,20), (-5,5,20)								
**Sapph**	**(0,4,20)**	**11.284**	**71.97**	**6.5**	**5.2**	**3.863**	**20.18**	**48.28**	**52.33**
	Equiv. Refl.: (0,-4,-20), (4,0,-20), (4,-4,20), (-4,0,20), (-4,4,-20)								
**Quartz**	**(0,8,-1)**	**11.712**	**80.74**	**6.2**	**5.2**	**1.936**	**10.02**	**24.2**	**26.19**
	Equiv. Refl.: (0,-8,1), (8,0,1), (8,-8,-1), (-8,0,-1), (-8,8,1)								
**Quartz**	**(0,2,-10)**	**11.835**	**85.76**	**5.7**	**5.3**	**0.88**	**4.69**	**10.99**	**11.95**
	Equiv. Refl.: (0,-2,10), (2,0,10), (2,-2,-10), (-2,0,-10), (-2,2,10)								
**Sapph**	**(2,5,15)**	**11.812**	**84.48**	**5.6**	**5.3**	**1.146**	**6.09**	**14.32**	**15.57**
	Equiv. Refl.: (2,5,-15), (2,-7,15), (2,-7,-15), (5,2,15), (5,2,-15), (5,-7,15), (5,-7,-15), (7,-2,15), (7,-2,-15) (7,-5,15), (7,-5,-15), (-2,7,15), (-2,7,-15), (-2,-5,15), (-2,-5,-15), (-5,7,15), (-5,7,-15), (-5,-2,15) (-5,-2,-15), (-7,2,15), (-7,2,-15), (-7,5,15), (-7,5,-15), (7,-1,6), (7,-6,-6), (-1,7,-6), (-1,-6,6) (-6,7,6), (-6,-1,-6)								
**LiNbO3**	**(0,-8,2)**	**11.16**	**70.12**	**5.3**	**4.8**	**4.291**	**20.56**	**53.64**	**57.45**
	Equiv. Refl.: (8,0,2), (-8,8,2), (1,3,9), (1,-4,-9), (3,1,-9), (3,-4,9), (4,-1,-9), (4,-3,9), (-1,4,9) (-1,-3,-9), (-3,4,-9), (-3,-1,9), (-4,1,9), (-4,3,-9)								

Ei =	11.867	keV						ΔEg	ΔEt
Cryst	Refl	EB	ΘB	∫ IR dΘ	Width	E cotΘ	ΔE	2m	2m
	(h,k,l)	[keV]	[°]	[µrad]	[µrad]	[keV]	[meV]	[meV]	[meV]
**Quartz**	**(0,1,10)**	**11.562**	**76.99**	**5.2**	**4.1**	**2.742**	**11.2**	**34.28**	**36.06**
	Equiv. Refl.: (0,-1,-10), (1,0,-10), (1,6,6), (1,6,-6), (1,-1,10), (1,-7,6), (1,-7,-6), (6,1,6), (6,1,-6)<br>(6,-7,6), (6,-7,-6), (-1,0,10), (-1,1,-10), (-7,1,6), (-7,1,-6), (-7,6,6), (-7,6,-6)								
**Quartz**	**(0,1,-10)**	**11.562**	**76.99**	**5.1**	**4.2**	**2.742**	**11.54**	**34.28**	**36.17**
	Equiv. Refl.: (0,-1,10), (1,0,10), (1,-1,-10), (4,-1,9), (4,-3,-9), (-1,0,-10), (-1,1,10), (-1,4,-9), (-1,-3,9)<br>(-3,4,9), (-3,-1,-9)								
**LiNbO3**	**(0,8,-2)**	**11.16**	**70.12**	**5**	**4.8**	**4.291**	**20.56**	**53.64**	**57.45**
	Equiv. Refl.: (8,-8,-2), (-8,0,-2), (0,7,8), (0,-7,-8), (7,0,-8), (7,-7,8), (-7,0,8), (-7,7,-8), (1,3,-9)<br>(1,-4,9), (3,1,9), (3,-4,-9), (-4,1,-9), (-4,3,9)								
**Quartz**	**(0,7,4)**	**11.183**	**70.46**	**4.7**	**3.6**	**4.212**	**15.09**	**52.65**	**54.77**
	Equiv. Refl.: (0,-7,-4), (7,0,-4), (7,-1,-6), (7,-6,6), (7,-7,4), (-1,7,6), (-1,-6,-6), (-7,0,4), (-7,7,-4)								
**Sapph**	**(4,5,-1)**	**11.76**	**82.29**	**4.6**	**4.2**	**1.608**	**6.8**	**20.09**	**21.21**
	Equiv. Refl.: (4,-9,1), (5,4,1), (5,-9,-1), (9,-4,1), (9,-5,-1), (-4,9,-1), (-4,-5,1), (-5,9,1), (-5,-4,-1)<br>(-9,4,-1), (-9,5,1), (4,5,-2), (4,-9,2), (5,4,2), (5,-9,-2), (-6,7,-6), (-6,-1,6), (-9,4,-2)<br>(-9,5,2)								
**LiNbO3**	**(2,7,7)**	**11.804**	**84.1**	**4.5**	**6.5**	**1.225**	**8**	**15.32**	**17.28**
	Equiv. Refl.: (2,-9,-7), (7,2,-7), (7,-9,7), (9,-2,-7), (9,-7,7), (-2,9,7), (-2,-7,-7), (-7,9,-7), (-7,-2,7)<br>(-9,2,7), (-9,7,-7), (9,-4,2), (9,-5,-2), (-4,9,-2), (-4,-5,2), (-5,9,2), (-5,-4,-2)								
**Sapph**	**(1,5,-16)**	**11.335**	**72.78**	**4.4**	**3.6**	**3.679**	**13.21**	**45.98**	**47.84**
	Equiv. Refl.: (1,-6,16), (5,1,16), (5,-6,-16), (6,-1,16), (6,-5,-16), (-1,6,-16), (-1,-5,16), (-5,6,16), (-5,-1,-16)<br>(-6,1,-16), (-6,5,16), (4,-2,9), (4,-2,-9), (-2,4,9), (-2,4,-9), (-2,-2,9), (-2,-2,-9)								
**Sapph**	**(2,3,20)**	**11.581**	**77.4**	**4.3**	**3.6**	**2.652**	**9.6**	**33.16**	**34.52**
	Equiv. Refl.: (2,-5,-20), (3,2,-20), (3,-5,20), (5,-2,-20), (5,-3,20), (-2,5,20), (-2,-3,-20), (-3,5,-20), (-3,-2,20)<br>(-5,2,20), (-5,3,-20), (9,-4,-1), (9,-5,1), (-4,9,1), (-4,-5,-1), (-5,9,-1), (-5,-4,1)								
**Quartz**	**(2,2,9)**	**11.491**	**75.54**	**4.2**	**3.4**	**3.061**	**10.46**	**38.26**	**39.66**
	Equiv. Refl.: (2,2,-9), (2,-4,9), (2,-4,-9), (4,5,1), (4,-9,-1), (5,4,-1), (5,-9,1), (-4,2,9), (-4,2,-9)<br>(-9,4,1), (-9,5,-1)								
**Sapph**	**(1,7,0)**	**11.358**	**73.16**	**3.9**	**3.3**	**3.592**	**11.7**	**44.9**	**46.4**
	Equiv. Refl.: (1,-8,0), (7,1,0), (7,-8,0), (8,-1,0), (8,-7,0), (-1,8,0), (-1,-7,0), (-7,8,0), (-7,-1,0)<br>(-8,1,0), (-8,7,0)								
**Sapph**	**(3,5,10)**	**11.562**	**76.98**	**3.6**	**3.4**	**2.743**	**9.28**	**34.29**	**35.53**
	Equiv. Refl.: (3,-8,-10), (5,3,-10), (5,-8,10), (8,-3,-10), (8,-5,10), (-3,8,10), (-3,-5,-10), (-5,8,-10), (-5,-3,10)<br>(-8,3,10), (-8,5,-10), (1,5,-7), (1,-6,7), (5,1,7), (5,-6,-7), (6,-1,7), (6,-5,-7), (-1,6,-7)<br>(-1,-5,7), (-5,6,7), (-5,-1,-7), (-6,1,-7), (-6,5,7)								
**Quartz**	**(0,8,0)**	**11.656**	**79.18**	**3.5**	**3**	**2.269**	**6.86**	**28.36**	**29.18**
	Equiv. Refl.: (0,-8,0), (8,0,0), (8,-8,0), (-8,0,0), (-8,8,0)								
**Quartz**	**(3,6,0)**	**11.564**	**77.03**	**3.4**	**2.8**	**2.732**	**7.61**	**34.15**	**34.99**
	Equiv. Refl.: (3,-9,0), (6,3,0), (6,-9,0), (-9,3,0), (-9,6,0)								
**Quartz**	**(2,6,-4)**	**11.465**	**75.04**	**3.3**	**3**	**3.172**	**9.43**	**39.65**	**40.75**
	Equiv. Refl.: (2,-8,4), (6,2,4), (6,-8,-4), (7,-3,-6), (7,-4,6), (8,-1,-3), (8,-7,3), (9,-3,0), (9,-6,0)<br>(-1,8,3), (-1,-7,-3), (-3,7,6), (-3,9,0), (-3,-4,-6), (-3,-6,0), (-4,7,-6), (-4,-3,6), (-6,9,0)<br>(-6,-3,0), (-7,8,-3), (-7,-1,3), (-8,2,-4), (-8,6,4)								
**Sapph**	**(2,2,21)**	**11.297**	**72.17**	**3.2**	**2.6**	**3.817**	**10.02**	**47.71**	**48.75**
	Equiv. Refl.: (2,2,-21), (2,-4,21), (2,-4,-21), (4,-2,21), (4,-2,-21), (-2,4,21), (-2,4,-21), (-2,-2,21), (-2,-2,-21)<br>(-4,2,21), (-4,2,-21), (1,7,3), (1,-8,-3), (3,4,6), (3,-7,-6), (4,3,-6), (4,4,5), (4,4,-5)<br>(4,-7,6), (4,-8,5), (4,-8,-5), (7,1,-3), (7,-8,3), (8,-2,4), (8,-6,-4), (-2,8,-4), (-2,-6,4)<br>(-6,8,4), (-6,-2,-4), (-7,3,6), (-7,4,-6), (-8,1,3), (-8,1,-3), (-8,4,5), (-8,4,-5), (-8,7,-3)								

Ei =	11.867	keV						ΔEg	ΔEt
**Cryst**	**Refl**	**EB**	**ΘB**	**∫ IR dΘ**	**Width**	**E cotΘ**	**ΔE**	**2m**	**2m**
	**(h,k,l)**	**[keV]**	**[°]**	**[µrad]**	**[µrad]**	**[keV]**	**[meV]**	**[meV]**	**[meV]**
**Sapph**	**(1,5,17)**	**11.662**	**79.33**	**3.1**	**2.8**	**2.236**	**6.24**	**27.95**	**28.64**
	Equiv. Refl.: (1,-6,-17), (5,1,-17), (5,-6,17), (6,-1,-17), (6,-5,17), (-1,6,17), (-1,-5,-17), (-5,6,-17), (-5,-1,17) (-6,1,17), (-6,5,-17), (1,7,-3), (1,-8,3), (7,1,3), (7,-8,-3), (8,-4,5), (8,-4,-5), (-4,8,5) (-4,8,-5), (-4,-4,5), (-4,-4,-5), (-8,7,3)								
**Quartz**	**(1,4,8)**	**11.348**	**72.99**	**3**	**2.7**	**3.629**	**9.65**	**45.37**	**46.38**
	Equiv. Refl.: (1,-5,-8), (4,1,-8), (4,-5,8), (-5,1,8), (-5,4,-8)								
**Quartz**	**(3,6,-1)**	**11.621**	**78.32**	**2.9**	**2.3**	**2.454**	**5.67**	**30.67**	**31.19**
	Equiv. Refl.: (3,-9,1), (5,-1,-8), (5,-4,8), (6,3,1), (6,-9,-1), (8,-1,3), (8,-7,-3), (-1,5,8), (-1,8,-3) (-1,-4,-8), (-1,-7,3), (-4,5,-8), (-4,-1,8), (-7,8,3), (-7,-1,-3), (-9,3,-1), (-9,6,1)								
**Quartz**	**(4,5,-1)**	**11.437**	**74.53**	**2.8**	**2.2**	**3.284**	**7.36**	**41.05**	**41.71**
	Equiv. Refl.: (4,-9,1), (5,4,1), (5,-9,-1), (-9,4,-1), (-9,5,1)								
**Quartz**	**(3,5,5)**	**11.701**	**80.4**	**2.6**	**2.5**	**2.007**	**4.96**	**25.09**	**25.58**
	Equiv. Refl.: (3,-8,-5), (5,3,-5), (5,-8,5), (-8,3,5), (-8,5,-5)								
**Quartz**	**(8,-3,-5)**	**11.701**	**80.4**	**2.5**	**2.5**	**2.007**	**4.96**	**25.09**	**25.58**
	Equiv. Refl.: (8,-5,5), (9,-4,1), (9,-5,-1), (-3,8,5), (-3,-5,-5), (-4,9,-1), (-4,-5,1), (-5,8,-5), (-5,9,1) (-5,-3,5), (-5,-4,-1)								
**Quartz**	**(2,3,-8)**	**11.16**	**70.12**	**2.4**	**2.1**	**4.292**	**8.99**	**53.65**	**54.4**
	Equiv. Refl.: (2,-5,8), (3,2,8), (3,-5,-8), (5,-2,8), (5,-3,-8), (-2,5,-8), (-2,-3,8), (-3,5,8), (-3,-2,-8) (-5,2,-8), (-5,3,8)								
**LiNbO3**	**(2,7,-5)**	**11.599**	**77.8**	**2.1**	**3.3**	**2.565**	**8.45**	**32.06**	**33.16**
	Equiv. Refl.: (2,-9,5), (7,2,5), (7,-9,-5), (9,-2,5), (9,-7,-5), (-2,9,-5), (-2,-7,5), (-7,9,5), (-7,-2,-5) (-9,2,-5), (-9,7,5)								
**Sapph**	**(2,6,-7)**	**11.351**	**73.05**	**1.9**	**2.2**	**3.617**	**7.8**	**45.22**	**45.88**
	Equiv. Refl.: (2,-8,7), (3,5,-11), (3,-8,11), (5,3,11), (5,-8,-11), (6,2,7), (6,-8,-7), (8,-2,7), (8,-3,11) (8,-5,-11), (8,-6,-7), (-2,8,-7), (-2,-6,7), (-3,8,-11), (-3,-5,11), (-5,8,11), (-5,-3,-11), (-6,8,7) (-6,-2,-7), (-8,2,-7), (-8,3,-11), (-8,5,11), (-8,6,7), (9,-3,1), (9,-6,-1), (-3,9,-1), (-3,-6,1) (-6,9,1), (-6,-3,-1)								
**LiNbO3**	**(2,7,1)**	**11.39**	**73.71**	**1.7**	**2.6**	**3.469**	**8.96**	**43.36**	**44.27**
	Equiv. Refl.: (2,-9,-1), (7,2,-1), (7,-9,1), (9,-2,-1), (9,-7,1), (-2,9,1), (-2,-7,-1), (-7,9,-1), (-7,-2,1) (-9,2,1), (-9,7,-1), (1,3,22), (1,-4,-22), (3,1,-22), (3,-4,22), (4,4,9), (4,4,-9), (4,-1,-22) (4,-3,22), (4,-8,9), (4,-8,-9), (8,-4,9), (8,-4,-9), (-1,4,22), (-1,-3,-22), (-3,4,-22), (-3,-1,22) (-4,1,22), (-4,3,-22), (-4,8,9), (-4,8,-9), (-4,-4,9), (-4,-4,-9), (-8,4,9), (-8,4,-9), (0,3,-9) (0,7,5), (0,-3,9), (0,-7,-5), (3,0,9), (3,5,-4), (3,-3,-9), (3,-8,4), (5,3,4), (5,-8,-4) (7,0,-5), (7,-7,5), (8,-3,4), (8,-3,5), (8,-5,-4), (8,-5,-5), (-3,0,-9), (-3,3,9), (-3,8,-4) (-3,8,-5), (-3,-5,4), (-3,-5,5), (-5,8,4), (-5,8,5), (-5,-3,-4), (-5,-3,-5), (-7,0,5), (-7,7,-5) (-8,3,-4), (-8,5,4)								
**Sapph**	**(1,7,3)**	**11.448**	**74.73**	**1.6**	**1.8**	**3.24**	**5.92**	**40.5**	**40.93**
	Equiv. Refl.: (1,7,-3), (1,-8,3), (1,-8,-3), (7,1,3), (7,1,-3), (7,-8,3), (7,-8,-3), (8,-1,3), (8,-1,-3) (8,-7,3), (8,-7,-3), (-1,8,3), (-1,8,-3), (-1,-7,3), (-1,-7,-3), (-7,8,3), (-7,8,-3), (-7,-1,3) (-7,-1,-3), (-8,1,3), (-8,1,-3), (-8,7,3), (-8,7,-3), (3,5,-5), (3,-8,5), (5,3,5), (5,-8,-5) (-8,3,-5), (-8,5,5)								
**Sapph**	**(3,4,14)**	**11.331**	**72.71**	**1.5**	**1.7**	**3.693**	**6.24**	**46.16**	**46.58**
	Equiv. Refl.: (3,-7,-14), (4,3,-14), (4,-7,14), (7,-3,-14), (7,-4,14), (-3,7,14), (-3,-4,-14), (-4,7,-14), (-4,-3,14) (-7,3,14), (-7,4,-14)								
**Quartz**	**(1,4,-8)**	**11.348**	**72.99**	**1.4**	**1.6**	**3.629**	**5.71**	**45.37**	**45.72**
	Equiv. Refl.: (1,-5,8), (2,5,6), (2,-7,-6), (4,1,8), (4,5,2), (4,-5,-8), (4,-9,-2), (5,2,-6), (5,4,-2) (5,-1,8), (5,-4,-8), (5,-7,6), (5,-9,2), (7,-2,-6), (7,-5,6), (9,-4,-2), (9,-5,2), (-1,5,-8) (-1,-4,8), (-2,7,6), (-2,-5,-6), (-4,5,8), (-4,9,2), (-4,-1,-8), (-4,-5,-2), (-5,1,-8), (-5,4,8) (-5,7,-6), (-5,9,-2), (-5,-2,6), (-5,-4,2), (-7,2,6), (-7,5,-6), (-9,4,2), (-9,5,-2)								
**LiNbO3**	**(2,4,19)**	**11.239**	**71.28**	**1.3**	**2.1**	**4.021**	**8.59**	**50.26**	**50.99**
	Equiv. Refl.: (2,-6,-19), (4,2,-19), (4,-6,19), (6,-2,-19), (6,-4,19), (-2,6,19), (-2,-4,-19), (-4,6,-19), (-4,-2,19) (-6,2,19), (-6,4,-19)								

Ei =	11.867	keV						ΔEg	ΔEt
**Cryst**	**Refl**	**EB**	**ΘB**	**∫ IR dΘ**	**Width**	**E cotΘ**	**ΔE**	**2m**	**2m**
	**(h,k,l)**	**[keV]**	**[°]**	**[μrad]**	**[μrad]**	**[keV]**	**[meV]**	**[meV]**	**[meV]**
**Sapph**	**(2,4,-17)**	**11.367**	**73.31**	**1.2**	**1.4**	**3.558**	**5.1**	**44.48**	**44.77**
	Equiv. Refl.: (2,-6,17), (4,2,17), (4,-6,-17), (6,-2,17), (6,-4,-17), (-2,6,-17), (-2,-4,17), (-4,6,17), (-4,-2,-17) (-6,2,-17), (-6,4,17), (0,8,1), (0,-8,-1), (8,0,-1), (8,-8,1), (-8,0,1), (-8,8,-1)								
**LiNbO3**	**(1,2,-25)**	**11.769**	**82.64**	**1.1**	**2.6**	**1.533**	**3.99**	**19.17**	**19.58**
	Equiv. Refl.: (1,-3,25), (2,1,25), (2,-3,-25), (3,-1,25), (3,-2,-25), (-1,3,-25), (-1,-2,25), (-2,3,25), (-2,-1,-25) (-3,1,-25), (-3,2,25), (0,2,10), (0,5,-8), (0,-2,-10), (0,-5,8), (1,1,10), (1,1,-10), (1,-2,10) (1,-2,-10), (2,0,-10), (2,-2,10), (5,0,8), (5,-5,-8), (-2,0,10), (-2,1,10), (-2,1,-10), (-2,2,-10) (-5,0,-8), (-5,5,8)								
**Sapph**	**(1,2,23)**	**11.677**	**79.73**	**1**	**1.4**	**2.149**	**3.02**	**26.87**	**27.04**
	Equiv. Refl.: (1,-3,-23), (2,1,-23), (2,-3,23), (3,-1,-23), (3,-2,23), (-1,3,23), (-1,-2,-23), (-2,3,-23), (-2,-1,23) (-3,1,23), (-3,2,-23)								
**LiNbO3**	**(1,3,-23)**	**11.442**	**74.62**	**0.9**	**1.8**	**3.265**	**5.88**	**40.81**	**41.23**
	Equiv. Refl.: (1,-4,23), (3,1,23), (3,4,17), (3,-4,-23), (3,-7,-17), (4,3,-17), (4,-1,23), (4,-3,-23), (4,-7,17) (7,-3,-17), (7,-4,17), (-1,4,-23), (-1,-3,23), (-3,4,23), (-3,7,17), (-3,-1,-23), (-3,-4,-17), (-4,1,-23) (-4,3,23), (-4,7,-17), (-4,-3,17), (-7,3,17), (-7,4,-17), (2,-1,10), (2,-1,-10), (3,4,-6), (3,-7,6) (4,3,6), (4,-7,-6), (-1,2,10), (-1,2,-10), (-1,-1,10), (-1,-1,-10), (-7,3,-6), (-7,4,6)								
**Sapph**	**(1,6,13)**	**11.654**	**79.13**	**0.8**	**1.2**	**2.278**	**2.74**	**28.48**	**28.61**
	Equiv. Refl.: (1,-7,-13), (2,3,-19), (2,-5,19), (3,2,19), (3,-5,-19), (5,-2,19), (5,-3,-19), (6,1,-13), (6,-7,13) (7,-1,-13), (7,-6,13), (-1,7,13), (-1,-6,-13), (-2,5,-19), (-2,-3,19), (-3,5,19), (-3,-2,-19), (-5,2,-19) (-5,3,19), (-6,7,-13), (-6,-1,13), (-7,1,13), (-7,6,-13), (8,-1,2), (8,-7,-2), (-1,8,-2), (-1,-7,2) (-7,8,2), (-7,-1,-2)								
**LiNbO3**	**(1,4,21)**	**11.348**	**73**	**0.7**	**1.5**	**3.628**	**5.37**	**45.35**	**45.67**
	Equiv. Refl.: (1,4,-21), (1,5,-19), (1,-5,21), (1,-5,-21), (1,-6,19), (3,6,9), (3,6,-9), (3,-9,9), (3,-9,-9) (4,1,21), (4,1,-21), (4,5,-7), (4,-5,21), (4,-5,-21), (4,-9,7), (5,1,19), (5,4,7), (5,-1,21) (5,-1,-21), (5,-4,21), (5,-4,-21), (5,-6,-19), (5,-9,-7), (6,3,9), (6,3,-9), (6,-1,19), (6,-5,-19) (6,-9,9), (6,-9,-9), (9,-3,9), (9,-3,-9), (9,-4,7), (9,-5,-7), (9,-6,9), (9,-6,-9), (-1,5,21) (-1,5,-21), (-1,6,-19), (-1,-4,21), (-1,-4,-21), (-1,-5,19), (-3,9,9), (-3,9,-9), (-3,-6,9), (-3,-6,-9) (-4,5,21), (-4,5,-21), (-4,9,-7), (-4,-1,21), (-4,-1,-21), (-4,-5,7), (-5,1,21), (-5,1,-21), (-5,4,21) (-5,4,-21), (-5,6,19), (-5,9,7), (-5,-1,-19), (-5,-4,-7), (-6,1,-19), (-6,5,19), (-6,9,9), (-6,9,-9) (-6,-3,9), (-6,-3,-9), (-9,3,9), (-9,3,-9), (-9,4,-7), (-9,5,7), (-9,6,9), (-9,6,-9), (1,7,-2) (1,-8,2), (7,1,2), (7,-3,6), (7,-4,-6), (7,-8,-2), (-3,7,-6), (-3,-4,6), (-4,7,6), (-4,-3,-6) (-8,1,-2), (-8,7,2)								
**Quartz**	**(0,7,-4)**	**11.183**	**70.46**	**0.6**	**0.8**	**4.212**	**3.25**	**52.65**	**52.75**
	Equiv. Refl.: (0,-7,4), (7,0,4), (7,-7,-4), (8,-3,-4), (8,-5,4), (-3,8,4), (-3,-5,-4), (-5,8,-4), (-5,-3,4) (-7,0,-4), (-7,7,4)								
**Sapph**	**(1,6,-11)**	**11.175**	**70.34**	**0.5**	**0.8**	**4.24**	**3.19**	**53**	**53.09**
	Equiv. Refl.: (1,-7,11), (6,1,11), (6,-7,-11), (7,-1,11), (7,-6,-11), (-1,7,-11), (-1,-6,11), (-6,7,11), (-6,-1,-11) (-7,1,-11), (-7,6,11), (3,5,4), (3,-8,-4), (5,3,-4), (5,-8,4), (8,-2,-4), (8,-6,4), (-2,8,4) (-2,-6,-4), (-6,8,-4), (-6,-2,4), (-8,3,4), (-8,5,-4)								
**LiNbO3**	**(2,6,-13)**	**11.59**	**77.6**	**0.4**	**1.2**	**2.609**	**3.15**	**32.61**	**32.76**
	Equiv. Refl.: (2,-8,13), (6,2,13), (6,-8,-13), (8,-2,13), (8,-6,-13), (-2,8,-13), (-2,-6,13), (-6,8,13), (-6,-2,-13) (-8,2,-13), (-8,6,13), (2,5,-6), (2,6,4), (2,-7,6), (2,-8,-4), (5,2,6), (5,-7,-6), (6,2,-4) (6,-1,-7), (6,-5,7), (6,-8,4), (7,-2,6), (7,-5,-6), (-1,6,7), (-1,-5,-7), (-2,7,-6), (-2,-5,6) (-5,6,-7), (-5,7,6), (-5,-1,7), (-5,-2,-6), (-7,2,-6), (-7,5,6), (-8,2,4), (-8,6,-4)								
**LiNbO3**	**(2,6,11)**	**11.169**	**70.24**	**0.3**	**0.8**	**4.262**	**3.55**	**53.28**	**53.39**
	Equiv. Refl.: (2,-8,-11), (3,3,21), (3,3,-21), (3,-6,21), (3,-6,-21), (6,2,-11), (6,-3,21), (6,-3,-21), (6,-8,11) (8,-2,-11), (8,-6,11), (-2,8,11), (-2,-6,-11), (-3,6,21), (-3,6,-21), (-3,-3,21), (-3,-3,-21), (-6,3,21) (-6,3,-21), (-6,8,-11), (-6,-2,11), (-8,2,11), (-8,6,-11), (0,3,9), (0,-3,-9), (1,5,7), (1,7,2) (1,-6,-7), (1,-8,-2), (2,3,8), (2,-5,-8), (3,0,-9), (3,2,-8), (3,-3,9), (3,-5,8), (4,5,0) (4,-9,0), (5,1,-7), (5,4,0), (5,-2,-8), (5,-3,8), (5,-6,7), (5,-9,0), (7,1,-2), (7,-8,2) (8,-1,-2), (8,-7,2), (9,-4,0), (9,-5,0), (-1,8,2), (-1,-7,-2), (-2,5,8), (-2,-3,-8), (-3,0,9) (-3,3,-9), (-3,5,-8), (-3,-2,8), (-4,9,0), (-4,-5,0), (-5,2,8), (-5,3,-8), (-5,9,0), (-5,-4,0)								

	(-6,1,7), (-6,5,-7), (-7,8,-2), (-7,-1,2), (-8,1,2), (-8,7,-2), (-9,4,0), (-9,5,0)								
**LiNbO3**	**(1,7,9)**	**11.243**	**71.34**	**0.2**	**0.7**	**4.008**	**2.8**	**50.1**	**50.18**
	Equiv. Refl.: (1,7,-9), (1,-8,9), (1,-8,-9), (3,5,13), (3,-8,-13), (5,3,-13), (5,-8,13), (7,1,9), (7,1,-9) (7,-8,9), (7,-8,-9), (8,-1,9), (8,-1,-9), (8,-3,-13), (8,-5,13), (8,-7,9), (8,-7,-9), (-1,8,9) (-1,8,-9), (-1,-7,9), (-1,-7,-9), (-3,8,13), (-3,-5,-13), (-5,8,-13), (-5,-3,13), (-7,8,9), (-7,8,-9) (-7,-1,9), (-7,-1,-9), (-8,1,9), (-8,1,-9), (-8,3,13), (-8,5,-13), (-8,7,9), (-8,7,-9), (0,0,24) (0,0,-24), (3,5,-8), (3,-8,8), (5,3,8), (5,-8,-8), (8,-3,8), (8,-5,-8), (-3,8,-8), (-3,-5,8) (-5,8,8), (-5,-3,-8), (-8,3,-8), (-8,5,8)								
**Sapph**	**(1,2,-22)**	**11.229**	**71.13**	**0.1**	**0.2**	**4.055**	**0.89**	**50.69**	**50.7**
	Equiv. Refl.: (1,-3,22), (2,1,22), (2,6,8), (2,-3,-22), (2,-8,-8), (3,-1,22), (3,-2,-22), (4,5,2), (4,-9,-2) (5,4,-2), (5,-9,2), (6,2,-8), (6,-8,8), (8,-2,-8), (8,-6,8), (9,-4,-2), (9,-5,2), (-1,3,-22) (-1,-2,22), (-2,3,22), (-2,8,8), (-2,-1,-22), (-2,-6,-8), (-3,1,-22), (-3,2,22), (-4,9,2), (-4,-5,-2) (-5,9,-2), (-5,-4,2), (-6,8,-8), (-6,-2,8), (-8,2,8), (-8,6,-8), (-9,4,2), (-9,5,-2)								
**LiNbO3**	**(4,4,15)**	**11.739**	**81.57**	**0**	**0.4**	**1.759**	**0.68**	**21.99**	**22**
	Equiv. Refl.: (4,4,-15), (4,-8,15), (4,-8,-15), (8,-4,15), (8,-4,-15), (-4,8,15), (-4,8,-15), (-4,-4,15), (-4,-4,-15) (-8,4,15), (-8,4,-15)								

Ei =	12.658	keV						ΔEg	ΔEt
Cryst	Refl (h,k,l)	EB [keV]	ΘB [°]	∫ IR dΘ [µrad]	Width [µrad]	Ei cotΘ [meV/µrad]	ΔE [meV]	2m, 50µm [meV]	2m, 50µm [meV]
**Si**	**(2,4,10)**	**12.504**	**81.05**	**5.823**	**5.1**	**1.993**	**10.13**	**24.92**	**26.9**
**Ge**	**(0,8,8)**	**12.397**	**78.35**	**3.668**	**7.8**	**2.609**	**20.34**	**32.62**	**38.44**
**Ge**	**(1,3,11)**	**12.542**	**82.23**	**2.783**	**7.9**	**1.727**	**13.69**	**21.59**	**25.56**
	Equiv. Refl.: (1,7,9), (5,5,9)								
**Si**	**(3,5,9)**	**12.241**	**75.25**	**2.769**	**2.4**	**3.334**	**7.86**	**41.67**	**42.4**
**Ge**	**(2,4,10)**	**12.004**	**71.5**	**2.685**	**5.5**	**4.236**	**23.34**	**52.95**	**57.87**
**Ge**	**(1,1,11)**	**12.153**	**73.76**	**1.567**	**4.2**	**3.688**	**15.66**	**46.1**	**48.68**
	Equiv. Refl.: (5,7,7)								
**LiNbO3**	**(4,-10,8)**	**12.639**	**86.85**	**37.7**	**34.2**	**0.697**	**23.81**	**8.71**	**25.36**
	Equiv. Refl.: (6,4,8), (10,-4,8), (-4,-6,8), (-6,10,8), (-10,6,8)								
**LiNbO3**	**(4,6,-8)**	**12.639**	**86.85**	**35.7**	**34.2**	**0.697**	**23.81**	**8.71**	**25.36**
	Equiv. Refl.: (6,-10,-8), (10,-6,-8), (-4,10,-8), (-6,-4,-8), (-10,4,-8)								
**LiNbO3**	**(0,1,-28)**	**12.598**	**84.42**	**22.9**	**21**	**1.238**	**26.05**	**15.47**	**30.29**
	Equiv. Refl.: (0,-1,28), (1,0,28), (1,-1,-28), (-1,0,-28), (-1,1,28)								
**Quartz**	**(5,5,0)**	**12.618**	**85.44**	**19.4**	**15.7**	**1.01**	**15.86**	**12.63**	**20.28**
	Equiv. Refl.: (5,-10,0), (-10,5,0)								
**Quartz**	**(10,-5,0)**	**12.618**	**85.44**	**19.3**	**15.7**	**1.01**	**15.86**	**12.63**	**20.28**
	Equiv. Refl.: (-5,10,0), (-5,-5,0)								
**LiNbO3**	**(4,-9,-14)**	**12.535**	**82.02**	**17.4**	**15.6**	**1.775**	**27.62**	**22.19**	**35.43**
	Equiv. Refl.: (5,4,-14), (9,-4,-14), (-4,-5,-14), (-5,9,-14), (-9,5,-14)								
**LiNbO3**	**(4,5,14)**	**12.535**	**82.02**	**16.7**	**15.6**	**1.775**	**27.62**	**22.19**	**35.43**
	Equiv. Refl.: (5,-9,14), (9,-5,14), (-4,9,14), (-5,-4,14), (-9,4,14)								
**LiNbO3**	**(4,4,-18)**	**12.554**	**82.64**	**15.7**	**14.4**	**1.635**	**23.59**	**20.44**	**31.21**
	Equiv. Refl.: (4,-8,-18), (8,-4,-18), (-4,8,-18), (-4,-4,-18), (-8,4,-18)								
**LiNbO3**	**(4,4,18)**	**12.554**	**82.64**	**15.2**	**14.4**	**1.635**	**23.59**	**20.44**	**31.21**
	Equiv. Refl.: (4,-8,18), (8,-4,18), (-4,8,18), (-4,-4,18), (-8,4,18)								
**LiNbO3**	**(1,4,-24)**	**12.481**	**80.42**	**14.3**	**13**	**2.137**	**27.86**	**26.72**	**38.6**
	Equiv. Refl.: (1,-5,24), (4,1,24), (4,-5,-24), (5,-1,24), (5,-4,-24), (-1,5,-24), (-1,-4,24), (-4,5,24), (-4,-1,-24) (-5,1,-24), (-5,4,24)								
**LiNbO3**	**(1,4,24)**	**12.481**	**80.42**	**14.2**	**12.9**	**2.137**	**27.66**	**26.72**	**38.46**
	Equiv. Refl.: (4,-5,24), (5,-4,24), (-1,5,24), (-4,-1,24), (-5,1,24)								
**LiNbO3**	**(1,-5,-24)**	**12.481**	**80.42**	**14.1**	**12.9**	**2.137**	**27.66**	**26.72**	**38.46**
	Equiv. Refl.: (4,1,-24), (5,-1,-24), (-1,-4,-24), (-4,5,-24), (-5,4,-24)								
**LiNbO3**	**(1,-6,22)**	**12.519**	**81.5**	**13.7**	**12.6**	**1.893**	**23.9**	**23.66**	**33.63**
	Equiv. Refl.: (5,1,22), (6,-1,22), (-1,-5,22), (-5,6,22), (-6,5,22)								
**LiNbO3**	**(0,9,0)**	**12.514**	**81.36**	**13.3**	**12.1**	**1.923**	**23.31**	**24.04**	**33.48**
	Equiv. Refl.: (1,5,-22), (5,-6,-22), (6,-5,-22), (9,-9,0), (-1,6,-22), (-5,-1,-22), (-6,1,-22), (-9,0,0)								
**LiNbO3**	**(3,7,-4)**	**12.488**	**80.59**	**12.7**	**11.7**	**2.098**	**24.56**	**26.22**	**35.93**
	Equiv. Refl.: (7,-10,-4), (10,-7,-4), (-3,10,-4), (-7,-3,-4), (-10,3,-4)								
**LiNbO3**	**(0,-9,0)**	**12.514**	**81.36**	**12.6**	**12.1**	**1.923**	**23.31**	**24.04**	**33.48**
	Equiv. Refl.: (9,0,0), (-9,9,0), (2,2,24), (2,2,-24), (2,-4,24), (2,-4,-24), (4,-2,24), (4,-2,-24), (-2,4,24) (-2,4,-24), (-2,-2,24), (-2,-2,-24), (-4,2,24), (-4,2,-24)								
**LiNbO3**	**(3,-10,4)**	**12.488**	**80.59**	**12.5**	**11.7**	**2.098**	**24.56**	**26.22**	**35.93**
	Equiv. Refl.: (7,3,4), (10,-3,4), (-3,-7,4), (-7,10,4), (-10,7,4)								
**LiNbO3**	**(3,4,20)**	**12.31**	**76.52**	**10.6**	**9.7**	**3.033**	**29.27**	**37.91**	**47.9**
	Equiv. Refl.: (3,-7,-20), (4,3,-20), (4,-7,20), (7,-3,-20), (7,-4,20), (-3,7,20), (-3,-4,-20), (-4,7,-20), (-4,-3,20) (-7,3,20), (-7,4,-20)								

Ei =	12.658	keV						ΔEg	ΔEt
Cryst	Refl	EB	ΘB	∫ IR dΘ	Width	Ei cotΘ	ΔE	2m, 50µm	2m, 50µm
	(h,k,l)	[keV]	[°]	[µrad]	[µrad]	[meV/µrad]	[meV]	[meV]	[meV]
**LiNbO3**	**(3,-10,-2)**	**12.391**	**78.22**	**10.1**	**9.1**	**2.641**	**23.92**	**33.01**	**40.76**
	Equiv. Refl.: (7,3,-2), (10,-3,-2), (-3,-7,-2), (-7,10,-2), (-10,7,-2)								
**LiNbO3**	**(0,5,22)**	**12.047**	**72.12**	**10**	**8.7**	**4.084**	**35.63**	**51.05**	**62.25**
	Equiv. Refl.: (5,-5,22), (-5,0,22)								
**LiNbO3**	**(1,8,8)**	**12.407**	**78.58**	**9.7**	**8.9**	**2.558**	**22.84**	**31.97**	**39.29**
	Equiv. Refl.: (3,7,2), (7,-10,2), (8,-9,8), (9,-8,8), (10,-7,2), (-1,9,8), (-3,10,2), (-7,-3,2), (-8,-1,8) (-9,1,8), (-10,3,2)								
**LiNbO3**	**(0,-5,-22)**	**12.047**	**72.12**	**9.6**	**8.7**	**4.084**	**35.63**	**51.05**	**62.25**
	Equiv. Refl.: (1,-9,-8), (5,0,-22), (8,1,-8), (9,-1,-8), (-1,-8,-8), (-5,5,-22), (-8,9,-8), (-9,8,-8)								
**LiNbO3**	**(2,-9,-10)**	**12.229**	**75.03**	**9.5**	**8.2**	**3.384**	**27.9**	**42.3**	**50.67**
	Equiv. Refl.: (3,6,12), (6,-9,12), (7,2,-10), (9,-2,-10), (9,-6,12), (-2,-7,-10), (-3,9,12), (-6,-3,12), (-7,9,-10) (-9,3,12), (-9,7,-10)								
**LiNbO3**	**(2,6,-16)**	**12.318**	**76.69**	**9.3**	**8.2**	**2.995**	**24.66**	**37.44**	**44.83**
	Equiv. Refl.: (3,-9,-12), (6,3,-12), (6,-8,-16), (8,-6,-16), (9,-3,-12), (-2,8,-16), (-3,-6,-12), (-6,9,-12), (-6,-2,-16) (-8,2,-16), (-9,6,-12), (4,4,15), (4,4,-15), (4,-8,15), (4,-8,-15), (8,-4,15), (8,-4,-15), (-4,8,15) (-4,8,-15), (-4,-4,15), (-4,-4,-15), (-8,4,15), (-8,4,-15)								
**LiNbO3**	**(5,5,0)**	**12.042**	**72.05**	**9.2**	**8.2**	**4.1**	**33.75**	**51.25**	**61.37**
	Equiv. Refl.: (5,-10,0), (10,-5,0), (-5,10,0), (-5,-5,0), (-10,5,0)								
**LiNbO3**	**(2,7,10)**	**12.229**	**75.03**	**8.9**	**8.2**	**3.384**	**27.9**	**42.3**	**50.67**
	Equiv. Refl.: (4,6,-2), (6,-10,-2), (7,-9,10), (9,-7,10), (10,-6,-2), (-2,9,10), (-4,10,-2), (-6,-4,-2), (-7,-2,10) (-9,2,10), (-10,4,-2)								
**LiNbO3**	**(2,-8,16)**	**12.318**	**76.69**	**8.8**	**8.2**	**2.995**	**24.66**	**37.44**	**44.83**
	Equiv. Refl.: (6,2,16), (8,-2,16), (-2,-6,16), (-6,8,16), (-8,6,16)								
**LiNbO3**	**(4,-10,2)**	**12.155**	**73.79**	**8.6**	**7.8**	**3.679**	**28.63**	**45.99**	**54.17**
	Equiv. Refl.: (6,4,2), (10,-4,2), (-4,-6,2), (-6,10,2), (-10,6,2), (4,-1,-10), (4,-3,10), (-1,4,10), (-1,-3,-10) (-3,4,-10), (-3,-1,10)								
**LiNbO3**	**(5,5,6)**	**12.337**	**77.07**	**8.3**	**7.5**	**2.905**	**21.89**	**36.31**	**42.4**
	Equiv. Refl.: (5,-10,6), (10,-5,6), (-5,10,6), (-5,-5,6), (-10,5,6)								
**LiNbO3**	**(0,2,-26)**	**11.955**	**70.81**	**8.2**	**7.2**	**4.406**	**31.79**	**55.07**	**63.59**
	Equiv. Refl.: (0,-2,26), (1,2,26), (1,-3,-26), (2,0,26), (2,1,-26), (2,-2,-26), (2,-3,26), (3,-1,-26), (3,-2,26) (3,-9,12), (6,3,12), (9,-3,12), (-1,3,26), (-1,-2,-26), (-2,0,-26), (-2,2,26), (-2,3,-26), (-2,-1,26) (-3,1,26), (-3,2,-26), (-3,-6,12), (-6,9,12), (-9,6,12)								
**Quartz**	**(1,3,10)**	**12.616**	**85.33**	**8.1**	**7.2**	**1.035**	**7.4**	**12.93**	**14.9**
	Equiv. Refl.: (1,-4,-10), (3,-4,10)								
**LiNbO3**	**(3,6,-12)**	**12.272**	**75.81**	**8**	**7.4**	**3.199**	**23.82**	**39.99**	**46.55**
	Equiv. Refl.: (6,-9,-12), (9,-6,-12), (-3,9,-12), (-6,-3,-12), (-9,3,-12), (3,1,-10), (-4,1,10), (-4,3,-10)								
**LiNbO3**	**(5,5,-6)**	**12.337**	**77.07**	**7.9**	**7.5**	**2.905**	**21.89**	**36.31**	**42.4**
	Equiv. Refl.: (5,-10,-6), (10,-5,-6), (-5,10,-6), (-5,-5,-6), (-10,5,-6)								
**LiNbO3**	**(2,4,22)**	**12.285**	**76.06**	**7.8**	**7.2**	**3.143**	**22.67**	**39.29**	**45.36**
	Equiv. Refl.: (2,-6,-22), (4,2,-22), (4,6,4), (4,-6,22), (4,-10,-4), (6,4,-4), (6,-2,-22), (6,-4,22), (6,-10,4) (10,-4,-4), (10,-6,4), (-2,6,22), (-2,-4,-22), (-4,6,-22), (-4,10,4), (-4,-2,22), (-4,-6,-4), (-6,2,22) (-6,4,-22), (-6,10,-4), (-6,-4,4), (-10,4,4), (-10,6,-4)								
**LiNbO3**	**(1,-9,4)**	**12.014**	**71.65**	**7.7**	**6.7**	**4.199**	**28.25**	**52.49**	**59.61**
	Equiv. Refl.: (8,1,4), (9,-1,4), (-1,-8,4), (-8,9,4), (-9,8,4), (0,8,-4), (0,-8,4), (8,0,4), (8,-8,-4) (-8,0,-4), (-8,8,4)								
**LiNbO3**	**(2,4,-23)**	**12.646**	**87.5**	**7.6**	**11.4**	**0.553**	**6.3**	**6.92**	**9.36**
	Equiv. Refl.: (2,-6,23), (4,2,23), (4,-6,-23), (6,-2,23), (6,-4,-23), (-2,6,-23), (-2,-4,23), (-4,6,23), (-4,-2,-23) (-6,2,-23), (-6,4,23)								
**LiNbO3**	**(1,8,2)**	**11.914**	**70.26**	**7.5**	**6.6**	**4.543**	**29.92**	**56.79**	**64.19**
	Equiv. Refl.: (8,-9,2), (9,-8,2), (-1,9,2), (-8,-1,2), (-9,1,2), (3,5,-14), (3,-8,14), (5,3,14), (5,-8,-14) (8,-3,14), (8,-5,-14), (-3,8,-14), (-3,-5,14), (-5,8,14), (-5,-3,-14), (-8,3,-14), (-8,5,14)								

Ei =	12.658	keV						ΔEg	ΔEt
**Cryst**	**Refl**	**EB**	**ΘB**	**∫ IR dΘ**	**Width**	**Ei cotΘ**	**ΔE**	**2m, 50μm**	**2m, 50μm**
	**(h,k,l)**	**[keV]**	**[°]**	**[μrad]**	**[μrad]**	**[meV/μrad]**	**[meV]**	**[meV]**	**[meV]**
**LiNbO3**	**(1,8,-4)**	**12.014**	**71.65**	**7.4**	**6.7**	**4.199**	**28.25**	**52.49**	**59.61**
	Equiv. Refl.: (1,-9,-2), (3,-8,-16), (5,3,-16), (8,1,-2), (8,-3,-16), (8,-9,-4), (9,-1,-2), (9,-8,-4), (-1,9,-4) (-1,-8,-2), (-3,-5,-16), (-5,8,-16), (-8,5,-16), (-8,9,-2), (-8,-1,-4), (-9,1,-4), (-9,8,-2)								
**LiNbO3**	**(0,8,10)**	**11.989**	**71.29**	**7.1**	**6.1**	**4.287**	**26.08**	**53.59**	**59.6**
	Equiv. Refl.: (8,-8,10), (-8,0,10)								
**LiNbO3**	**(0,-7,16)**	**12.08**	**72.62**	**6.8**	**6.2**	**3.962**	**24.37**	**49.52**	**55.19**
	Equiv. Refl.: (7,0,16), (-7,7,16)								
**Sapph**	**(0,6,18)**	**12.461**	**79.89**	**6.7**	**5.6**	**2.257**	**12.58**	**28.21**	**30.89**
	Equiv. Refl.: (0,6,-18), (0,-6,18), (0,-6,-18), (6,0,18), (6,0,-18), (6,-6,18), (6,-6,-18), (-6,0,18), (-6,0,-18) (-6,6,18), (-6,6,-18)								
**LiNbO3**	**(0,7,-16)**	**12.08**	**72.62**	**6.6**	**6.2**	**3.962**	**24.37**	**49.52**	**55.19**
	Equiv. Refl.: (3,5,16), (5,-8,16), (7,-7,-16), (8,-5,16), (-3,8,16), (-5,-3,16), (-7,0,-16), (-8,3,16)								
**LiNbO3**	**(0,-8,-10)**	**11.989**	**71.29**	**6.3**	**6.1**	**4.287**	**26.08**	**53.59**	**59.6**
	Equiv. Refl.: (8,0,-10), (-8,8,-10)								
**LiNbO3**	**(2,-9,8)**	**11.931**	**70.48**	**6.1**	**5.5**	**4.487**	**24.59**	**56.09**	**61.24**
	Equiv. Refl.: (7,2,8), (9,-2,8), (-2,-7,8), (-7,9,8), (-9,7,8), (7,-1,7), (7,-6,-7), (-1,7,-7), (-1,-6,7) (-6,7,7), (-6,-1,-7)								
**Quartz**	**(1,6,-7)**	**12.48**	**80.38**	**5.9**	**4.9**	**2.146**	**10.5**	**26.83**	**28.81**
	Equiv. Refl.: (1,-7,7), (3,5,6), (3,-8,-6), (5,3,-6), (5,-8,6), (6,1,7), (6,-7,-7), (8,-3,-6), (8,-5,6) (-3,8,6), (-3,-5,-6), (-5,8,-6), (-5,-3,6), (-7,1,-7), (-7,6,7), (-8,3,6), (-8,5,-6)								
**LiNbO3**	**(2,7,-8)**	**11.931**	**70.48**	**5.7**	**5.5**	**4.487**	**24.59**	**56.09**	**61.24**
	Equiv. Refl.: (7,-9,-8), (9,-7,-8), (-2,9,-8), (-7,-2,-8), (-9,2,-8), (0,8,4), (0,-8,-4), (2,6,6), (2,-8,-6) (6,2,-6), (6,-8,6), (8,0,-4), (8,-8,4), (-8,0,4), (-8,2,6), (-8,6,-6), (-8,8,-4)								
**Quartz**	**(8,-2,-6)**	**12.56**	**82.86**	**5.5**	**4.8**	**1.586**	**7.61**	**19.82**	**21.23**
	Equiv. Refl.: (8,-6,6), (-2,8,6), (-2,-6,-6), (-6,8,-6), (-6,-2,6)								
**Sapph**	**(2,4,-20)**	**12.43**	**79.1**	**5.3**	**4.3**	**2.438**	**10.39**	**30.47**	**32.2**
	Equiv. Refl.: (2,-6,20), (4,2,20), (4,-6,-20), (6,-2,20), (6,-4,-20), (-2,6,-20), (-2,-4,20), (-4,6,20), (-4,-2,-20) (-6,2,-20), (-6,4,20), (9,-1,1), (9,-8,-1), (-1,9,-1), (-1,-8,1), (-8,9,1), (-8,-1,-1)								
**Sapph**	**(3,6,0)**	**11.941**	**70.62**	**5.2**	**4.1**	**4.452**	**18.38**	**55.65**	**58.61**
	Equiv. Refl.: (3,-9,0), (6,3,0), (6,-9,0), (9,-3,0), (9,-6,0), (-3,9,0), (-3,-6,0), (-6,9,0), (-6,-3,0) (-9,3,0), (-9,6,0), (1,8,-1), (1,-9,1), (8,1,1), (8,-9,-1), (-9,1,-1), (-9,8,1)								
**Sapph**	**(2,7,4)**	**12.461**	**79.89**	**5.1**	**4.2**	**2.258**	**9.56**	**28.22**	**29.8**
	Equiv. Refl.: (2,-9,-4), (7,2,-4), (7,-9,4), (9,-2,-4), (9,-7,4), (-2,9,4), (-2,-7,-4), (-7,9,-4), (-7,-2,4) (-9,2,4), (-9,7,-4), (3,6,-4), (3,-9,4), (6,3,4), (6,-9,-4), (9,-3,4), (9,-6,-4), (-3,9,-4) (-3,-6,4), (-6,9,4), (-6,-3,-4), (-9,3,-4), (-9,6,4)								
**Sapph**	**(0,8,4)**	**12.186**	**74.3**	**4.7**	**3.7**	**3.558**	**13.24**	**44.47**	**46.4**
	Equiv. Refl.: (0,-8,-4), (8,0,-4), (8,-8,4), (-8,0,4), (-8,8,-4)								
**Quartz**	**(1,5,8)**	**12.248**	**75.37**	**4.6**	**3.6**	**3.303**	**11.79**	**41.29**	**42.94**
	Equiv. Refl.: (1,-6,-8), (5,1,-8), (5,-6,8), (6,-1,-8), (6,-5,8), (9,-1,0), (9,-8,0), (-1,6,8), (-1,9,0) (-1,-5,-8), (-1,-8,0), (-5,6,-8), (-5,-1,8), (-6,1,8), (-6,5,-8), (-8,9,0), (-8,-1,0)								
**Quartz**	**(1,8,0)**	**12.448**	**79.56**	**4.5**	**3.7**	**2.332**	**8.54**	**29.15**	**30.37**
	Equiv. Refl.: (1,-9,0), (2,2,10), (2,2,-10), (2,-4,10), (2,-4,-10), (4,-2,10), (4,-2,-10), (8,1,0), (8,-9,0) (-2,4,10), (-2,4,-10), (-2,-2,10), (-2,-2,-10), (-4,2,10), (-4,2,-10), (-9,1,0), (-9,8,0)								
**Quartz**	**(2,7,3)**	**12.412**	**78.7**	**4.4**	**3.5**	**2.53**	**8.91**	**31.63**	**32.86**
	Equiv. Refl.: (2,-9,-3), (7,2,-3), (7,-2,-7), (7,-5,7), (7,-9,3), (9,-2,-3), (9,-7,3), (-2,7,7), (-2,9,3) (-2,-5,-7), (-2,-7,-3), (-5,7,-7), (-5,-2,7), (-7,9,-3), (-7,-2,3), (-9,2,3), (-9,7,-3)								
**Quartz**	**(2,5,7)**	**12.135**	**73.47**	**4.3**	**3.3**	**3.756**	**12.3**	**46.96**	**48.54**
	Equiv. Refl.: (2,-7,-7), (5,2,-7), (5,-7,7), (-7,2,7), (-7,5,-7)								
**Quartz**	**(0,3,10)**	**12.275**	**75.87**	**4.1**	**3.2**	**3.188**	**10.19**	**39.84**	**41.13**
	Equiv. Refl.: (0,-3,-10), (3,0,-10), (3,-3,10), (-3,0,10), (-3,3,-10)								

**Ei =**	**12.658**	**keV**						**ΔEg**	**ΔEt**
**Cryst**	**Refl**	**EB**	**ΘB**	**∫ IR dΘ**	**Width**	**Ei cotΘ**	**ΔE**	**2m, 50µm**	**2m, 50µm**
	**(h,k,l)**	**[keV]**	**[°]**	**[µrad]**	**[µrad]**	**[meV/µrad]**	**[meV]**	**[meV]**	**[meV]**
**Sapph**	**(4,5,-4)**	**11.904**	**70.12**	**3.9**	**3.1**	**4.576**	**13.98**	**57.2**	**58.89**
	Equiv. Refl.: (4,-9,4), (5,4,4), (5,-9,-4), (9,-4,4), (9,-5,-4), (-4,9,-4), (-4,-5,4), (-5,9,4), (-5,-4,-4)<br>(-9,4,-4), (-9,5,4), (1,7,5), (1,-8,-5), (4,5,-4), (4,-9,4), (5,4,4), (5,-9,-4), (7,1,-5)<br>(7,-8,5), (9,-4,4), (9,-5,-4), (-4,9,-4), (-4,-5,4), (-5,9,4), (-5,-4,-4), (-8,1,5), (-8,7,-5)<br>(-9,4,-4), (-9,5,4)								
**Quartz**	**(1,7,-5)**	**12.405**	**78.53**	**3.8**	**3**	**2.568**	**7.81**	**32.1**	**33.04**
	Equiv. Refl.: (1,-8,5), (3,6,4), (3,-9,-4), (6,3,-4), (6,-9,4), (7,1,5), (7,-8,-5), (8,-1,-5), (8,-7,5)<br>(9,-3,-4), (9,-6,4), (-1,8,5), (-1,-7,-5), (-3,9,4), (-3,-6,-4), (-6,9,-4), (-6,-3,4), (-7,8,-5)<br>(-7,-1,5), (-8,1,-5), (-8,7,5), (-9,3,4), (-9,6,-4)								
**Quartz**	**(8,-1,5)**	**12.405**	**78.53**	**3.7**	**3**	**2.568**	**7.81**	**32.1**	**33.04**
	Equiv. Refl.: (8,-7,-5), (-1,8,-5), (-1,-7,5), (-7,8,5), (-7,-1,-5)								
**Sapph**	**(2,6,-13)**	**12.498**	**80.87**	**3.3**	**2.9**	**2.035**	**5.88**	**25.43**	**26.1**
	Equiv. Refl.: (2,-8,13), (6,2,13), (6,-8,-13), (8,-2,13), (8,-6,-13), (-2,8,-13), (-2,-6,13), (-6,8,13), (-6,-2,-13)<br>(-8,2,-13), (-8,6,13), (1,3,-10), (1,-4,10), (3,1,10), (3,-4,-10), (8,-2,6), (8,-6,-6), (-2,8,-6)<br>(-2,-6,6), (-4,1,-10), (-4,3,10), (-6,8,6), (-6,-2,-6)								
**Quartz**	**(2,3,9)**	**12.12**	**73.24**	**2.8**	**2.2**	**3.812**	**8.3**	**47.65**	**48.37**
	Equiv. Refl.: (2,-5,-9), (3,2,-9), (3,4,-7), (3,-5,9), (3,-7,7), (4,3,7), (4,-7,-7), (5,-2,-9), (5,-3,9)<br>(-2,5,9), (-2,-3,-9), (-3,5,-9), (-3,-2,9), (-5,2,9), (-5,3,-9), (-7,3,-7), (-7,4,7)								
**Quartz**	**(2,7,1)**	**11.981**	**71.18**	**2.7**	**2.3**	**4.315**	**9.87**	**53.94**	**54.83**
	Equiv. Refl.: (2,-9,-1), (7,2,-1), (7,-3,7), (7,-4,-7), (7,-9,1), (-3,7,-7), (-3,-4,7), (-4,7,7), (-4,-3,-7)<br>(-9,2,1), (-9,7,-1)								
**Quartz**	**(2,6,-6)**	**12.56**	**82.86**	**2.6**	**2.7**	**1.586**	**4.25**	**19.82**	**20.27**
	Equiv. Refl.: (2,-8,6), (6,2,6), (6,-8,-6), (9,-2,2), (9,-7,-2), (-2,9,-2), (-2,-7,2), (-7,9,2), (-7,-2,-2)<br>(-8,2,-6), (-8,6,6)								
**Sapph**	**(0,1,26)**	**12.5**	**80.95**	**2.5**	**2.5**	**2.016**	**4.97**	**25.2**	**25.69**
	Equiv. Refl.: (0,-1,-26), (1,0,-26), (1,-1,26), (-1,0,26), (-1,1,-26), (2,7,-2), (2,-9,2), (7,2,2), (7,-9,-2)<br>(9,-2,-1), (9,-7,1), (-2,9,1), (-2,-7,-1), (-7,9,-1), (-7,-2,1), (-9,2,-2), (-9,7,2)								
**Sapph**	**(0,5,-20)**	**12.153**	**73.77**	**2.4**	**2.2**	**3.685**	**8.15**	**46.07**	**46.78**
	Equiv. Refl.: (0,-5,20), (5,0,20), (5,-5,-20), (-5,0,-20), (-5,5,20)								
**LiNbO3**	**(1,6,19)**	**12.463**	**79.93**	**2.3**	**3.4**	**2.248**	**7.73**	**28.1**	**29.14**
	Equiv. Refl.: (1,-7,-19), (6,1,-19), (6,-7,19), (7,-1,-19), (7,-6,19), (-1,7,19), (-1,-6,-19), (-6,7,-19), (-6,-1,19)<br>(-7,1,19), (-7,6,-19), (1,7,-4), (1,-8,4), (2,3,-9), (2,-5,9), (3,2,9), (3,-5,-9), (5,-2,9)<br>(5,-3,-9), (7,1,4), (7,-8,-4), (-2,5,-9), (-2,-3,9), (-3,5,9), (-3,-2,-9), (-5,2,-9), (-5,3,9)<br>(-8,1,-4), (-8,7,4)								
**Quartz**	**(4,-1,10)**	**12.616**	**85.33**	**2.1**	**2.9**	**1.035**	**2.96**	**12.93**	**13.27**
	Equiv. Refl.: (4,-3,-10), (-1,4,-10), (-1,-3,10), (-3,4,10), (-3,-1,-10)								
**LiNbO3**	**(4,6,7)**	**12.52**	**81.52**	**2**	**3.3**	**1.887**	**6.24**	**23.59**	**24.4**
	Equiv. Refl.: (4,-10,-7), (6,4,-7), (6,-10,7), (10,-4,-7), (10,-6,7), (-4,10,7), (-4,-6,-7), (-6,10,-7), (-6,-4,7)<br>(-10,4,7), (-10,6,-7)								
**Sapph**	**(1,4,21)**	**12.165**	**73.96**	**1.9**	**2.1**	**3.639**	**7.59**	**45.49**	**46.12**
	Equiv. Refl.: (1,4,-21), (1,-5,21), (1,-5,-21), (4,1,21), (4,1,-21), (4,-5,21), (4,-5,-21), (5,-1,21), (5,-1,-21)<br>(5,-4,21), (5,-4,-21), (-1,5,21), (-1,5,-21), (-1,-4,21), (-1,-4,-21), (-4,5,21), (-4,5,-21), (-4,-1,21)<br>(-4,-1,-21), (-5,1,21), (-5,1,-21), (-5,4,21), (-5,4,-21), (8,-1,4), (8,-7,-4), (-1,8,-4), (-1,-7,4)<br>(-7,8,4), (-7,-1,-4)								

Ei =	12.658	keV						ΔEg	ΔEt
**Cryst**	**Refl**	**EB**	**ΘB**	**∫ IR dΘ**	**Width**	**Ei cotΘ**	**ΔE**	**2m, 50µm**	**2m, 50µm**
	**(h,k,l)**	**[keV]**	**[°]**	**[µrad]**	**[µrad]**	**[meV/µrad]**	**[meV]**	**[meV]**	**[meV]**
**LiNbO3**	**(1,1,27)**	**12.312**	**76.57**	**1.8**	**2.7**	**3.024**	**8.2**	**37.8**	**38.67**
	Equiv. Refl.: (1,1,-27), (1,-2,27), (1,-2,-27), (2,7,-11), (2,-1,27), (2,-1,-27), (2,-9,11), (7,2,11), (7,-9,-11) (9,-2,11), (9,-7,-11), (-1,2,27), (-1,2,-27), (-1,-1,27), (-1,-1,-27), (-2,1,27), (-2,1,-27), (-2,9,-11) (-2,-7,11), (-7,9,11), (-7,-2,-11), (-9,2,-11), (-9,7,11), (1,6,16), (1,6,-14), (1,-7,14), (1,-7,-16) (6,1,14), (6,1,-16), (6,-7,16), (6,-7,-14), (7,-1,14), (7,-1,-16), (7,-6,16), (7,-6,-14), (-1,7,16) (-1,7,-14), (-1,-6,14), (-1,-6,-16), (-6,7,14), (-6,7,-16), (-6,-1,16), (-6,-1,-14), (-7,1,16), (-7,1,-14) (-7,6,14), (-7,6,-16), (2,6,5), (2,-8,-5), (3,-1,-10), (3,-2,10), (6,2,-5), (6,-8,5), (-1,3,10) (-1,-2,-10), (-2,3,-10), (-2,-1,10), (-8,2,5), (-8,6,-5)								
**Sapph**	**(1,5,-19)**	**12.345**	**77.23**	**1.7**	**1.9**	**2.868**	**5.42**	**35.85**	**36.26**
	Equiv. Refl.: (1,-6,19), (2,7,-5), (2,-9,5), (4,5,-7), (4,-9,7), (5,1,19), (5,4,7), (5,-6,-19), (5,-9,-7) (6,-1,19), (6,-5,-19), (7,2,5), (7,-9,-5), (9,-2,5), (9,-4,7), (9,-5,-7), (9,-7,-5), (-1,6,-19) (-1,-5,19), (-2,9,-5), (-2,-7,5), (-4,9,-7), (-4,-5,7), (-5,6,19), (-5,9,7), (-5,-1,-19), (-5,-4,-7) (-6,1,-19), (-6,5,19), (-7,9,5), (-7,-2,-5), (-9,2,-5), (-9,4,-7), (-9,5,7), (-9,7,5), (1,2,10) (1,2,-10), (1,-3,10), (1,-3,-10), (2,1,10), (2,1,-10), (2,-3,10), (2,-3,-10), (3,-1,10), (3,-2,-10) (8,-2,-5), (8,-6,5), (-1,3,-10), (-1,-2,10), (-2,3,10), (-2,8,5), (-2,-1,-10), (-2,-6,-5), (-3,1,10) (-3,1,-10), (-3,2,10), (-3,2,-10), (-6,8,-5), (-6,-2,5)								
**Quartz**	**(1,6,7)**	**12.48**	**80.38**	**1.6**	**1.7**	**2.146**	**3.72**	**26.83**	**27.08**
	Equiv. Refl.: (1,-7,-7), (2,7,-1), (2,-9,1), (4,5,4), (4,-9,-4), (5,4,-4), (5,-9,4), (6,1,-7), (6,-7,7) (7,2,1), (7,-9,-1), (9,-2,1), (9,-3,3), (9,-4,-4), (9,-5,4), (9,-6,-3), (9,-7,-1), (-2,9,-1) (-2,-7,1), (-3,9,-3), (-3,-6,3), (-4,9,4), (-4,-5,-4), (-5,9,-4), (-5,-4,4), (-6,9,3), (-6,-3,-3) (-7,1,7), (-7,6,-7), (-7,9,1), (-7,-2,-1), (-9,2,-1), (-9,4,4), (-9,5,-4), (-9,7,1)								
**Sapph**	**(2,6,11)**	**12.052**	**72.2**	**1.5**	**1.6**	**4.064**	**6.7**	**50.79**	**51.23**
	Equiv. Refl.: (2,-8,-11), (6,2,-11), (6,-8,11), (8,-2,-11), (8,-6,11), (-2,8,11), (-2,-6,-11), (-6,8,-11), (-6,-2,11) (-8,2,11), (-8,6,-11), (2,5,-7), (2,-7,7), (3,3,8), (3,3,-8), (3,6,-3), (3,-6,8), (3,-6,-8) (3,-9,3), (4,4,6), (4,4,-6), (4,-8,6), (4,-8,-6), (5,2,7), (5,-7,-7), (6,3,3), (6,-9,-3) (7,-1,-7), (7,-6,7), (8,-4,6), (8,-4,-6), (-1,7,7), (-1,-6,-7), (-4,8,6), (-4,8,-6), (-4,-4,6) (-4,-4,-6), (-6,3,8), (-6,3,-8), (-6,7,-7), (-6,-1,7), (-7,2,-7), (-7,5,7), (-8,4,6), (-8,4,-6) (-9,3,-3), (-9,6,3)								
**LiNbO3**	**(4,6,-5)**	**12.326**	**76.86**	**1.4**	**2.2**	**2.955**	**6.55**	**36.94**	**37.52**
	Equiv. Refl.: (4,-10,5), (6,4,5), (6,-10,-5), (10,-4,5), (10,-6,-5), (-4,10,-5), (-4,-6,5), (-6,10,5), (-6,-4,-5) (-10,4,-5), (-10,6,5), (4,5,5), (4,-9,-5), (5,4,-5), (5,-9,5), (9,-4,-5), (9,-5,5), (-4,9,5) (-4,-5,-5), (-5,9,-5), (-5,-4,5), (-9,4,5), (-9,5,-5), (1,7,4), (1,-8,-4), (6,-3,8), (6,-3,-8) (7,1,-4), (7,-2,7), (7,-3,-7), (7,-4,7), (7,-5,-7), (7,-8,4), (8,-1,-4), (8,-7,4), (9,-3,-3) (9,-6,3), (-1,8,4), (-1,-7,-4), (-2,7,-7), (-2,-5,7), (-3,6,8), (-3,6,-8), (-3,7,7), (-3,9,3) (-3,-3,8), (-3,-3,-8), (-3,-4,-7), (-3,-6,-3), (-4,7,-7), (-4,-3,7), (-5,7,7), (-5,-2,-7), (-6,9,-3) (-6,-3,3), (-7,8,-4), (-7,-1,4), (-8,1,4), (-8,7,-4)								
**Sapph**	**(1,2,-25)**	**12.579**	**83.58**	**1.3**	**1.7**	**1.424**	**2.47**	**17.8**	**17.97**
	Equiv. Refl.: (1,-3,25), (2,1,25), (2,-3,-25), (3,-1,25), (3,-2,-25), (-1,3,-25), (-1,-2,25), (-2,3,25), (-2,-1,-25) (-3,1,-25), (-3,2,25), (2,4,-8), (2,-6,8), (3,4,7), (3,6,3), (3,-7,-7), (3,-9,-3), (4,3,-7) (4,-7,7), (6,3,-3), (6,-9,3), (-6,2,-8), (-6,4,8), (-7,3,7), (-7,4,-7), (-9,3,3), (-9,6,-3)								
**Sapph**	**(1,7,9)**	**12.143**	**73.6**	**1.2**	**1.4**	**3.724**	**5.1**	**46.55**	**46.83**
	Equiv. Refl.: (1,7,-9), (1,-8,9), (1,-8,-9), (7,1,9), (7,1,-9), (7,-8,9), (7,-8,-9), (8,-1,9), (8,-1,-9) (8,-7,9), (8,-7,-9), (-1,8,9), (-1,8,-9), (-1,-7,9), (-1,-7,-9), (-7,8,9), (-7,8,-9), (-7,-1,9) (-7,-1,-9), (-8,1,9), (-8,1,-9), (-8,7,9), (-8,7,-9), (1,4,9), (1,-5,-9), (3,5,-6), (3,-8,6) (4,1,-9), (4,2,8), (4,-5,9), (4,-6,-8), (5,3,6), (5,-8,-6), (6,-2,8), (6,-4,-8), (-2,6,-8) (-2,-4,8), (-4,6,8), (-4,-2,-8), (-5,1,9), (-5,4,-9), (-8,3,-6), (-8,5,6)								
**LiNbO3**	**(4,6,1)**	**12.13**	**73.4**	**1.1**	**1.8**	**3.774**	**6.89**	**47.18**	**47.68**
	Equiv. Refl.: (4,-10,-1), (6,4,-1), (6,-10,1), (10,-4,-1), (10,-6,1), (-4,10,1), (-4,-6,-1), (-6,10,-1), (-6,-4,1) (-10,4,1), (-10,6,-1), (0,4,-22), (0,-4,22), (2,7,1), (2,-9,-1), (4,0,22), (4,-4,-22), (7,2,-1) (7,-9,1), (9,-2,-1), (9,-7,1), (-2,9,1), (-2,-7,-1), (-4,0,-22), (-4,4,22), (-7,9,-1), (-7,-2,1) (-9,2,1), (-9,7,-1), (5,-1,-9), (5,-4,9), (8,-3,6), (8,-5,-6), (-1,5,9), (-1,-4,-9), (-4,5,-9) (-4,-1,9), (-5,8,6), (-5,-3,-6)								

Ei =	12.658	keV						ΔEg	ΔEt
**Cryst**	**Refl**	**EB**	**ΘB**	**∫ IR dΘ**	**Width**	**Ei cotΘ**	**ΔE**	**2m, 50µm**	**2m, 50µm**
	**(h,k,l)**	**[keV]**	**[°]**	**[µrad]**	**[µrad]**	**[meV/µrad]**	**[meV]**	**[meV]**	**[meV]**
**Sapph**	**(0,8,-8)**	**12.627**	**85.96**	**1**	**1.7**	**0.894**	**1.48**	**11.18**	**11.27**
	Equiv. Refl.: (0,-8,8), (3,6,6), (3,6,-6), (3,-9,6), (3,-9,-6), (6,3,6), (6,3,-6), (6,-9,6), (6,-9,-6)<br>(8,0,8), (8,-8,-8), (9,-3,6), (9,-3,-6), (9,-6,6), (9,-6,-6), (-3,9,6), (-3,9,-6), (-3,-6,6)<br>(-3,-6,-6), (-6,9,6), (-6,9,-6), (-6,-3,6), (-6,-3,-6), (-8,0,-8), (-8,8,8), (-9,3,6), (-9,3,-6)<br>(-9,6,6), (-9,6,-6), (-3,8,-6), (-3,-5,6)								
**LiNbO3**	**(3,7,5)**	**12.56**	**82.85**	**0.9**	**2.2**	**1.588**	**3.52**	**19.85**	**20.16**
	Equiv. Refl.: (3,-10,-5), (7,3,-5), (7,-10,5), (10,-3,-5), (10,-7,5), (-3,10,5), (-3,-7,-5), (-7,10,-5), (-7,-3,5)<br>(-10,3,5), (-10,7,-5), (0,7,14), (0,-7,-14), (2,4,19), (2,-6,-19), (4,2,-19), (4,-6,19), (6,-2,-19)<br>(6,-4,19), (7,0,-14), (7,-7,14), (-2,6,19), (-2,-4,-19), (-4,6,-19), (-4,-2,19), (-6,2,19), (-6,4,-19)<br>(-7,0,14), (-7,7,-14), (2,7,2), (2,-9,-2), (7,2,-2), (7,-9,2), (9,-2,-2), (9,-7,2), (-2,9,2)<br>(-2,-7,-2), (-7,9,-2), (-7,-2,2), (-9,2,2), (-9,7,-2)								
**LiNbO3**	**(1,3,25)**	**12.252**	**75.45**	**0.8**	**1.5**	**3.285**	**5.04**	**41.06**	**41.37**
	Equiv. Refl.: (1,-4,-25), (3,1,-25), (3,-4,25), (4,-1,-25), (4,-3,25), (-1,4,25), (-1,-3,-25), (-3,4,-25), (-3,-1,25)<br>(-4,1,25), (-4,3,-25), (2,3,-22), (2,7,-2), (2,-5,22), (2,-9,2), (3,2,22), (3,5,13), (3,-5,-22)<br>(3,-8,-13), (5,3,-13), (5,-2,22), (5,-3,-22), (5,-8,13), (7,2,2), (7,-9,-2), (8,-3,-13), (8,-5,13)<br>(9,-2,2), (9,-7,-2), (-2,5,-22), (-2,9,-2), (-2,-3,22), (-2,-7,2), (-3,5,22), (-3,8,13), (-3,-2,-22)<br>(-3,-5,-13), (-5,2,-22), (-5,3,22), (-5,8,-13), (-5,-3,13), (-7,9,2), (-7,-2,-2), (-8,3,13), (-8,5,-13)<br>(-9,2,-2), (-9,7,2), (0,5,9), (0,8,-3), (0,-5,-9), (0,-8,3), (5,0,-9), (5,-5,9), (8,0,3)<br>(8,-8,-3), (-5,0,9), (-5,5,-9), (-8,0,-3), (-8,8,3)								
**LiNbO3**	**(3,4,-19)**	**11.989**	**71.28**	**0.7**	**1.2**	**4.289**	**5.3**	**53.61**	**53.87**
	Equiv. Refl.: (3,-7,19), (4,3,19), (4,5,-13), (4,-7,-19), (4,-9,13), (5,4,13), (5,-9,-13), (7,-3,19), (7,-4,-19)<br>(9,-4,13), (9,-5,-13), (-3,7,-19), (-3,-4,19), (-4,7,19), (-4,9,-13), (-4,-3,-19), (-4,-5,13), (-5,9,13)<br>(-5,-4,-13), (-7,3,-19), (-7,4,19), (-9,4,-13), (-9,5,13), (1,8,1), (1,-9,-1), (8,1,-1), (8,-9,1)<br>(-9,1,1), (-9,8,-1)								
**LiNbO3**	**(2,6,17)**	**12.583**	**83.76**	**0.6**	**1.9**	**1.385**	**2.59**	**17.31**	**17.5**
	Equiv. Refl.: (2,-8,-17), (3,7,-1), (3,-10,1), (6,2,-17), (6,-8,17), (7,3,1), (7,-10,-1), (8,-2,-17), (8,-6,17)<br>(10,-3,1), (10,-7,-1), (-2,8,17), (-2,-6,-17), (-3,10,-1), (-3,-7,1), (-6,8,-17), (-6,-2,17), (-7,10,1)<br>(-7,-3,-1), (-8,2,17), (-8,6,-17), (-10,3,-1), (-10,7,1), (0,3,-10), (0,-3,10), (3,0,10), (3,-3,-10)<br>(8,-2,5), (8,-6,-5), (-2,8,-5), (-2,-6,5), (-3,0,-10), (-3,3,10), (-6,8,5), (-6,-2,-5)								
**LiNbO3**	**(4,5,11)**	**11.922**	**70.37**	**0.5**	**1.1**	**4.516**	**4.93**	**56.44**	**56.66**
	Equiv. Refl.: (4,-9,-11), (5,4,-11), (5,-9,11), (9,-4,-11), (9,-5,11), (-4,9,11), (-4,-5,-11), (-5,9,-11), (-5,-4,11)<br>(-9,4,11), (-9,5,-11), (4,5,8), (4,-9,-8), (5,4,-8), (5,-9,8), (9,-4,-8), (9,-5,8), (-4,9,8)<br>(-4,-5,-8), (-5,9,-8), (-5,-4,8), (-9,4,8), (-9,5,-8), (1,4,-9), (1,-5,9), (2,4,8), (2,6,-5)<br>(2,-6,-8), (2,-8,5), (4,1,9), (4,2,-8), (4,-5,-9), (4,-6,8), (5,-1,9), (5,-4,-9), (6,2,5)<br>(6,-8,-5), (9,-1,-1), (9,-8,1), (-1,5,-9), (-1,9,1), (-1,-4,9), (-1,-8,-1), (-4,5,9), (-4,-1,-9)<br>(-5,1,-9), (-5,4,9), (-5,6,8), (-5,-1,-8), (-6,2,8), (-6,4,-8), (-8,2,-5), (-8,6,5), (-8,9,-1)<br>(-8,-1,1)								
**Sapph**	**(1,3,-23)**	**12.245**	**75.32**	**0.4**	**0.6**	**3.316**	**1.88**	**41.46**	**41.5**
	Equiv. Refl.: (1,-4,23), (3,1,23), (3,-4,-23), (4,-1,23), (4,-3,-23), (-1,4,-23), (-1,-3,23), (-3,4,23), (-3,-1,-23)<br>(-4,1,-23), (-4,3,23), (1,5,-8), (1,-6,8), (5,1,8), (5,-6,-8), (6,-1,8), (6,-2,-8), (6,-4,8)<br>(6,-5,-8), (9,-2,3), (9,-7,-3), (-1,6,-8), (-1,-5,8), (-2,6,8), (-2,9,-3), (-2,-4,-8), (-2,-7,3)<br>(-4,6,-8), (-4,-2,8), (-6,1,-8), (-6,5,8), (-7,9,3), (-7,-2,-3)								
**LiNbO3**	**(1,7,15)**	**12.458**	**79.8**	**0.3**	**0.9**	**2.278**	**2.15**	**28.47**	**28.55**
	Equiv. Refl.: (1,7,-15), (1,-8,15), (1,-8,-15), (7,1,15), (7,1,-15), (7,-8,15), (7,-8,-15), (8,-1,15), (8,-1,-15)<br>(8,-7,15), (8,-7,-15), (-1,8,15), (-1,8,-15), (-1,-7,15), (-1,-7,-15), (-7,8,15), (-7,8,-15), (-7,-1,15)<br>(-7,-1,-15), (-8,1,15), (-8,1,-15), (-8,7,15), (-8,7,-15), (0,3,24), (0,3,-24), (0,-3,24), (0,-3,-24)<br>(3,0,24), (3,0,-24), (3,4,-16), (3,-3,24), (3,-3,-24), (3,-7,16), (4,3,16), (4,-7,-16), (7,-3,16)<br>(7,-4,-16), (-3,0,24), (-3,0,-24), (-3,3,24), (-3,3,-24), (-3,7,-16), (-3,-4,16), (-4,7,16), (-4,-3,-16)<br>(-7,3,-16), (-7,4,16), (2,7,-3), (2,-9,3), (7,2,3), (7,-9,-3), (9,-2,0), (9,-7,0), (-2,9,0)<br>(-2,-7,0), (-7,9,0), (-7,-2,0), (-9,2,-3), (-9,7,3)								
**LiNbO3**	**(2,3,23)**	**11.938**	**70.58**	**0.2**	**0.5**	**4.462**	**2.4**	**55.77**	**55.82**
	Equiv. Refl.: (2,-5,-23), (3,2,-23), (3,5,-17), (3,-5,23), (3,-8,17), (5,3,17), (5,-2,-23), (5,-3,23), (5,-8,-17)								

	(8,-3,17), (8,-5,-17), (-2,5,23), (-2,-3,-23), (-3,5,-23), (-3,8,-17), (-3,-2,23), (-3,-5,17), (-5,2,23) (-5,3,-23), (-5,8,17), (-5,-3,-17), (-8,3,-17), (-8,5,17), (3,6,3), (3,6,-3), (3,-9,3), (3,-9,-3) (6,3,3), (6,3,-3), (6,-9,3), (6,-9,-3), (9,-3,3), (9,-3,-3), (9,-6,3), (9,-6,-3), (-3,9,3) (-3,9,-3), (-3,-6,3), (-3,-6,-3), (-6,9,3), (-6,9,-3), (-6,-3,3), (-6,-3,-3), (-9,3,3), (-9,3,-3) (-9,6,3), (-9,6,-3), (2,7,0), (2,-9,0), (7,2,0), (7,-9,0), (-9,2,0), (-9,7,0)							
**LiNbO3**	**(1,8,5)**	**12.089**	**72.75**	**0.1**	**0.5**	**3.929**	**1.89**	**49.12**	**49.15**
	Equiv. Refl.: (1,8,-7), (1,-9,7), (1,-9,-5), (8,1,7), (8,1,-5), (8,-9,5), (8,-9,-7), (9,-1,7), (9,-1,-5) (9,-8,5), (9,-8,-7), (-1,9,5), (-1,9,-7), (-1,-8,7), (-1,-8,-5), (-8,9,7), (-8,9,-5), (-8,-1,5) (-8,-1,-7), (-9,1,5), (-9,1,-7), (-9,8,7), (-9,8,-5), (3,4,17), (3,-7,-17), (4,3,-17), (4,-7,17) (7,-3,-17), (7,-4,17), (-3,7,17), (-3,-4,-17), (-4,7,-17), (-4,-3,17), (-7,3,17), (-7,4,-17)							
**LiNbO3**	**(5,5,3)**	**12.116**	**73.18**	**0**	**0.1**	**3.826**	**0.55**	**47.83**	**47.83**
	Equiv. Refl.: (5,5,-3), (5,-10,3), (5,-10,-3), (10,-5,3), (10,-5,-3), (-5,10,3), (-5,10,-3), (-5,-5,3), (-5,-5,-3) (-10,5,3), (-10,5,-3)							

Ei =	13.474	keV						ΔEg	ΔEt
**Cryst**	**Refl**	**EB**	**ΘB**	**∫IR dΘ**	**Width**	**Ei cotΘ**	**ΔE**	**2m, 50µm**	**2m, 50µm**
	**(h,k,l)**	**[keV]**	**[°]**	**[µrad]**	**[µrad]**	**[meV/µrad]**	**[meV]**	**[meV]**	**[meV]**
**Si**	**(3,3,11)**	**13.457**	**87.16**	**8.5763**	**8.18**	**0.6688**	**5.47**	**8.36**	**9.99**
	Equiv. Refl.: (3,7,9), (3,9,7), (3,11,3), (7,3,9), (7,9,3), (9,3,7), (9,7,3), (11,3,3)								
**Si**	**(0,6,10)**	**13.311**	**81.09**	**4.5518**	**3.85**	**2.1123**	**8.13**	**26.4**	**27.63**
	Equiv. Refl.: (0,10,6), (6,0,10), (6,6,8), (6,8,6), (6,10,0), (8,6,6), (10,0,6), (10,6,0)								
**Si**	**(0,8,8)**	**12.914**	**73.42**	**2.9342**	**2.32**	**4.0109**	**9.31**	**50.14**	**50.99**
	Equiv. Refl.: (8,0,8), (8,8,0)								
**Ge**	**(0,0,12)**	**13.149**	**77.4**	**2.7615**	**5.95**	**3.0125**	**17.93**	**37.66**	**41.71**
	Equiv. Refl.: (0,12,0), (4,8,8), (8,4,8), (8,8,4), (12,0,0)								
**Ge**	**(0,6,10)**	**12.779**	**71.52**	**2.1614**	**4.5**	**4.5043**	**20.26**	**56.3**	**59.84**
	Equiv. Refl.: (0,10,6), (6,0,10), (6,6,8), (6,8,6), (6,10,0), (8,6,6), (10,0,6), (10,6,0)								
**Ge**	**(1,5,11)**	**13.286**	**80.41**	**1.8516**	**5.33**	**2.277**	**12.13**	**28.46**	**30.94**
	Equiv. Refl.: (1,11,5), (5,1,11), (5,11,1), (7,7,7), (11,1,5), (11,5,1)								
**Si**	**(1,3,11)**	**13.064**	**75.84**	**1.6618**	**1.84**	**3.4002**	**6.25**	**42.5**	**42.96**
	Equiv. Refl.: (1,7,9), (1,9,7), (1,11,3), (3,1,11), (3,11,1), (5,5,9), (5,9,5), (7,1,9), (7,9,1) (9,1,7), (9,5,5), (9,7,1), (11,1,3), (11,3,1)								
**Ge**	**(3,3,11)**	**12.919**	**73.5**	**1.2477**	**3.43**	**3.9915**	**13.67**	**49.89**	**51.73**
	Equiv. Refl.: (3,7,9), (3,9,7), (3,11,3), (7,3,9), (7,9,3), (9,3,7), (9,7,3), (11,3,3)								
**LiNbO3**	**(7,-9,16)**	**13.444**	**86.16**	**27.497**	**24.6**	**0.905**	**22.26**	**11.31**	**24.97**
	Equiv. Refl.: (9,-7,16)								
**LiNbO3**	**(2,7,16)**	**13.444**	**86.16**	**27.496**	**24.6**	**0.905**	**22.26**	**11.31**	**24.97**
	Equiv. Refl.: (-2,9,16), (-7,-2,16), (-9,2,16)								
**LiNbO3**	**(2,-9,-16)**	**13.444**	**86.16**	**26.275**	**24.6**	**0.905**	**22.26**	**11.31**	**24.97**
	Equiv. Refl.: (7,2,-16), (9,-2,-16), (-2,-7,-16), (-7,9,-16), (-9,7,-16)								
**LiNbO3**	**(0,0,-30)**	**13.415**	**84.65**	**25.384**	**22.2**	**1.262**	**28**	**15.77**	**32.14**
**LiNbO3**	**(0,0,30)**	**13.415**	**84.65**	**24.694**	**22.2**	**1.262**	**28**	**15.77**	**32.14**
**LiNbO3**	**(1,-9,-14)**	**13.429**	**85.31**	**22.575**	**20.4**	**1.105**	**22.56**	**13.81**	**26.46**
	Equiv. Refl.: (8,1,-14), (9,-1,-14), (-1,-8,-14), (-8,9,-14), (-9,8,-14)								
**LiNbO3**	**(1,8,14)**	**13.429**	**85.31**	**22.135**	**20.4**	**1.105**	**22.56**	**13.81**	**26.46**
	Equiv. Refl.: (8,-9,14), (9,-8,14), (-1,9,14), (-8,-1,14), (-9,1,14)								
**LiNbO3**	**(-6,8,-20)**	**13.436**	**85.7**	**19.493**	**18**	**1.014**	**18.25**	**12.67**	**22.22**
	Equiv. Refl.: (-8,6,-20)								
**LiNbO3**	**(2,-8,-20)**	**13.436**	**85.7**	**19.492**	**18**	**1.014**	**18.25**	**12.67**	**22.22**
	Equiv. Refl.: (6,2,-20), (8,-2,-20), (-2,-6,-20)								
**LiNbO3**	**(2,6,20)**	**13.436**	**85.7**	**18.604**	**18**	**1.014**	**18.25**	**12.67**	**22.22**
	Equiv. Refl.: (6,-8,20), (8,-6,20), (-2,8,20), (-6,-2,20), (-8,2,20)								
**LiNbO3**	**(5,6,-4)**	**13.384**	**83.39**	**16.859**	**15.1**	**1.561**	**23.58**	**19.51**	**30.61**
	Equiv. Refl.: (6,-11,-4), (11,-6,-4), (-5,11,-4), (-6,-5,-4), (-11,5,-4)								
**LiNbO3**	**(5,-11,4)**	**13.384**	**83.39**	**16.399**	**15.1**	**1.561**	**23.58**	**19.51**	**30.61**
	Equiv. Refl.: (6,5,4), (11,-5,4), (-5,-6,4), (-6,11,4), (-11,6,4)								
**LiNbO3**	**(4,-11,0)**	**13.409**	**84.39**	**15.476**	**14.4**	**1.324**	**19.07**	**16.56**	**25.25**
	Equiv. Refl.: (7,4,0), (11,-4,0), (-4,-7,0), (-7,11,0), (-11,7,0)								
**LiNbO3**	**(4,7,0)**	**13.409**	**84.39**	**15.196**	**14.4**	**1.324**	**19.07**	**16.56**	**25.25**
	Equiv. Refl.: (-7,-4,0)								
**LiNbO3**	**(7,-11,0)**	**13.409**	**84.39**	**15.195**	**14.4**	**1.324**	**19.07**	**16.56**	**25.25**
	Equiv. Refl.: (11,-7,0), (-4,11,0), (-11,4,0)								
**LiNbO3**	**(1,-7,-22)**	**13.414**	**84.57**	**15**	**13.9**	**1.281**	**17.87**	**16.01**	**23.99**
	Equiv. Refl.: (6,1,-22), (7,-1,-22), (-1,-6,-22), (-6,7,-22), (-7,6,-22)								

Ei =	13.474	keV						ΔEg	ΔEt
Cryst	Refl	EB	ΘB	∫ IR dΘ	Width	Ei cotΘ	ΔE	2m, 50µm	2m, 50µm
	(h,k,l)	[keV]	[°]	[µrad]	[µrad]	[meV/µrad]	[meV]	[meV]	[meV]
LiNbO3	(1,6,22)	13.414	84.57	14.387	13.9	1.281	17.87	16.01	23.99
	Equiv. Refl.: (6,-7,22), (7,-6,22), (-6,-1,22)								
LiNbO3	(-1,7,22)	13.414	84.57	14.386	13.9	1.281	17.87	16.01	23.99
	Equiv. Refl.: (-7,1,22)								
LiNbO3	(1,9,4)	13.384	83.39	12.35	11.5	1.561	18	19.51	26.55
	Equiv. Refl.: (9,-10,4), (10,-9,4), (-1,10,4), (-9,-1,4), (-10,1,4)								
LiNbO3	(1,-10,-4)	13.384	83.39	12.209	11.5	1.561	18	19.51	26.55
	Equiv. Refl.: (9,1,-4), (10,-1,-4), (-1,-9,-4), (-9,10,-4), (-10,9,-4)								
LiNbO3	(1,9,-2)	13.295	80.64	11.934	10.7	2.221	23.85	27.77	36.6
	Equiv. Refl.: (9,-10,-2), (10,-9,-2), (-1,10,-2), (-9,-1,-2), (-10,1,-2)								
LiNbO3	(1,-10,2)	13.295	80.64	11.699	10.7	2.221	23.85	27.77	36.6
	Equiv. Refl.: (9,1,2), (10,-1,2), (-1,-9,2), (-9,10,2), (-10,9,2)								
LiNbO3	(0,-7,-20)	13.218	78.82	10.159	8.9	2.663	23.73	33.28	40.88
	Equiv. Refl.: (7,0,-20), (-7,7,-20)								
LiNbO3	(0,7,20)	13.218	78.82	9.976	8.9	2.663	23.73	33.28	40.88
	Equiv. Refl.: (7,-7,20), (-7,0,20)								
Quartz	(7,3,-3)	13.399	83.97	9.801	7.7	1.424	10.9	17.8	20.87
	Equiv. Refl.: (7,-10,3), (-10,3,3), (-10,7,-3)								
Quartz	(3,7,3)	13.399	83.97	9.799	7.7	1.424	10.9	17.8	20.87
	Equiv. Refl.: (3,-10,-3)								
LiNbO3	(1,-8,-18)	13.229	79.05	9.767	8.7	2.607	22.6	32.59	39.66
	Equiv. Refl.: (7,1,-18), (8,-1,-18), (-1,-7,-18), (-7,8,-18), (-8,7,-18)								
Quartz	(10,-3,-3)	13.399	83.97	9.757	7.7	1.424	10.9	17.8	20.87
	Equiv. Refl.: (10,-7,3), (-7,10,-3), (-7,-3,3)								
Quartz	(-3,10,3)	13.399	83.97	9.755	7.7	1.424	10.9	17.8	20.87
	Equiv. Refl.: (-3,-7,-3)								
LiNbO3	(1,7,18)	13.229	79.05	9.649	8.7	2.607	22.6	32.59	39.66
	Equiv. Refl.: (7,-8,18), (8,-7,18), (-1,8,18), (-7,-1,18), (-8,1,18)								
LiNbO3	(2,3,26)	13.112	76.68	9.404	8.2	3.19	26.13	39.87	47.67
	Equiv. Refl.: (3,-5,26), (5,-3,26), (-2,5,26), (-3,-2,26), (-5,2,26)								
LiNbO3	(2,-5,-26)	13.112	76.68	9.363	8.2	3.19	26.13	39.87	47.67
	Equiv. Refl.: (3,2,-26), (5,-2,-26), (-2,-3,-26), (-3,5,-26), (-5,3,-26)								
LiNbO3	(5,5,-12)	13.183	78.08	8.995	7.8	2.844	22.31	35.55	41.97
	Equiv. Refl.: (-5,10,-12), (-5,-5,-12), (-10,5,-12)								
LiNbO3	(5,-10,-12)	13.183	78.08	8.994	7.8	2.844	22.31	35.55	41.97
	Equiv. Refl.: (10,-5,-12)								
LiNbO3	(-8,8,-16)	13.226	78.99	8.987	7.7	2.62	20.06	32.75	38.41
LiNbO3	(0,-8,-16)	13.226	78.99	8.986	7.7	2.62	20.06	32.75	38.41
	Equiv. Refl.: (8,0,-16)								
LiNbO3	(3,-10,10)	13.143	77.27	8.928	7.9	3.043	23.99	38.03	44.97
	Equiv. Refl.: (7,3,10), (10,-3,10), (-3,-7,10), (-7,10,10), (-10,7,10)								
LiNbO3	(3,7,-10)	13.143	77.27	8.876	7.9	3.043	23.99	38.03	44.97
	Equiv. Refl.: (7,-10,-10), (10,-7,-10), (-3,10,-10), (-7,-3,-10), (-10,3,-10)								
LiNbO3	(1,-8,18)	13.229	79.05	8.779	8	2.607	20.73	32.59	38.62
	Equiv. Refl.: (7,1,18), (8,-1,18), (-1,-7,18), (-7,8,18), (-8,7,18)								
Sapph	(0,1,-28)	13.449	86.48	8.764	7.8	0.829	6.46	10.36	12.21
	Equiv. Refl.: (0,-1,28), (1,0,28), (1,-1,-28), (-1,0,-28), (-1,1,28)								
LiNbO3	(1,7,-18)	13.229	79.05	8.74	8	2.607	20.73	32.59	38.62
	Equiv. Refl.: (7,-8,-18), (8,-7,-18), (-1,8,-18), (-7,-1,-18), (-8,1,-18)								

Ei =	13.474	keV						ΔEg	ΔEt
Cryst	Refl	EB	ΘB	∫ IR dΘ	Width	Ei cotΘ	ΔE	2m, 50µm	2m, 50µm
	(h,k,l)	[keV]	[°]	[µrad]	[µrad]	[meV/µrad]	[meV]	[meV]	[meV]
**Sapph**	**(1,4,24)**	**13.37**	**82.86**	**8.581**	**6.9**	**1.687**	**11.72**	**21.09**	**24.13**
	Equiv. Refl.: (1,4,-24), (1,-5,24), (1,-5,-24), (4,1,24), (4,1,-24), (4,-5,24), (4,-5,-24), (5,-1,24), (5,-1,-24) (5,-4,24), (5,-4,-24), (-1,5,24), (-1,5,-24), (-1,-4,24), (-1,-4,-24), (-4,5,24), (-4,5,-24), (-4,-1,24) (-4,-1,-24), (-5,1,24), (-5,1,-24), (-5,4,24), (-5,4,-24)								
**LiNbO3**	**(5,5,12)**	**13.183**	**78.08**	**8.51**	**7.8**	**2.844**	**22.31**	**35.55**	**41.97**
	Equiv. Refl.: (5,-10,12), (10,-5,12), (-5,10,12), (-5,-5,12), (-10,5,12)								
**Quartz**	**(10,-2,1)**	**13.403**	**84.1**	**8.343**	**6.6**	**1.392**	**9.21**	**17.4**	**19.69**
	Equiv. Refl.: (10,-8,-1), (-2,10,-1), (-2,-8,1)								
**Quartz**	**(-8,10,1)**	**13.403**	**84.1**	**8.342**	**6.6**	**1.392**	**9.21**	**17.4**	**19.69**
	Equiv. Refl.: (-8,-2,-1)								
**LiNbO3**	**(5,-11,-2)**	**13.295**	**80.64**	**8.257**	**7.7**	**2.221**	**17.12**	**27.77**	**32.62**
	Equiv. Refl.: (6,5,-2), (11,-5,-2), (-5,-6,-2), (-6,11,-2), (-11,6,-2)								
**Quartz**	**(2,8,-1)**	**13.403**	**84.1**	**8.224**	**6.6**	**1.392**	**9.21**	**17.4**	**19.69**
	Equiv. Refl.: (2,-10,1)								
**Quartz**	**(8,2,1)**	**13.403**	**84.1**	**8.223**	**6.6**	**1.392**	**9.21**	**17.4**	**19.69**
	Equiv. Refl.: (8,-10,-1), (-10,2,-1), (-10,8,1)								
**LiNbO3**	**(5,6,2)**	**13.295**	**80.64**	**8.114**	**7.7**	**2.221**	**17.12**	**27.77**	**32.62**
	Equiv. Refl.: (6,-11,2), (11,-6,2), (-5,11,2), (-6,-5,2), (-11,5,2)								
**LiNbO3**	**(4,-9,16)**	**13.005**	**74.84**	**8.015**	**7**	**3.651**	**25.43**	**45.63**	**52.24**
	Equiv. Refl.: (5,4,16), (9,-4,16), (-4,-5,16), (-5,9,16), (-9,5,16)								
**LiNbO3**	**(4,5,-16)**	**13.005**	**74.84**	**7.821**	**7**	**3.651**	**25.43**	**45.63**	**52.24**
	Equiv. Refl.: (5,-9,-16), (9,-5,-16), (-4,9,-16), (-5,-4,-16), (-9,4,-16)								
**LiNbO3**	**(0,8,16)**	**13.226**	**78.99**	**7.791**	**7.7**	**2.62**	**20.06**	**32.75**	**38.41**
	Equiv. Refl.: (8,-8,16), (-8,0,16)								
**LiNbO3**	**(2,7,-14)**	**12.99**	**74.59**	**7.769**	**6.7**	**3.713**	**24.81**	**46.41**	**52.63**
	Equiv. Refl.: (7,-9,-14), (9,-7,-14), (-2,9,-14), (-7,-2,-14), (-9,2,-14), (3,7,2), (3,-10,-2), (7,3,-2), (7,-10,2) (10,-3,-2), (10,-7,2), (-3,10,2), (-3,-7,-2), (-7,10,-2), (-7,-3,2), (-10,3,2), (-10,7,-2)								
**Quartz**	**(3,6,6)**	**13.457**	**87.15**	**7.586**	**6.8**	**0.67**	**4.58**	**8.38**	**9.55**
**Quartz**	**(3,-9,-6)**	**13.457**	**87.15**	**7.584**	**6.8**	**0.67**	**4.58**	**8.38**	**9.55**
**Quartz**	**(-9,6,-6)**	**13.457**	**87.15**	**7.581**	**6.8**	**0.67**	**4.58**	**8.38**	**9.55**
**Quartz**	**(-9,3,6)**	**13.457**	**87.15**	**7.58**	**6.8**	**0.67**	**4.58**	**8.38**	**9.55**
**Quartz**	**(6,3,-6)**	**13.457**	**87.15**	**7.579**	**6.8**	**0.67**	**4.58**	**8.38**	**9.55**
**Quartz**	**(6,-9,6)**	**13.457**	**87.15**	**7.578**	**6.8**	**0.67**	**4.57**	**8.38**	**9.55**
**Quartz**	**(-3,-6,-6)**	**13.457**	**87.15**	**7.575**	**6.8**	**0.67**	**4.58**	**8.38**	**9.55**
**Quartz**	**(-3,9,6)**	**13.457**	**87.15**	**7.574**	**6.8**	**0.67**	**4.58**	**8.38**	**9.55**
**Quartz**	**(9,-6,6)**	**13.457**	**87.15**	**7.571**	**6.8**	**0.67**	**4.58**	**8.38**	**9.55**
**Quartz**	**(9,-3,-6)**	**13.457**	**87.15**	**7.57**	**6.8**	**0.67**	**4.58**	**8.38**	**9.55**
**Quartz**	**(-6,-3,6)**	**13.457**	**87.15**	**7.568**	**6.8**	**0.67**	**4.58**	**8.38**	**9.55**
**Quartz**	**(-6,9,-6)**	**13.457**	**87.15**	**7.567**	**6.8**	**0.67**	**4.57**	**8.38**	**9.55**
**LiNbO3**	**(4,-10,-10)**	**12.921**	**73.52**	**7.564**	**6.6**	**3.986**	**26.12**	**49.82**	**56.26**
	Equiv. Refl.: (6,4,-10), (10,-4,-10), (-4,-6,-10), (-6,10,-10), (-10,6,-10)								
**LiNbO3**	**(1,2,-28)**	**13.05**	**75.59**	**7.48**	**6.7**	**3.462**	**23.03**	**43.27**	**49.02**
	Equiv. Refl.: (3,5,-20), (5,-8,-20), (8,-5,-20), (-1,3,-28), (-2,-1,-28), (-3,1,-28), (-3,8,-20), (-5,-3,-20), (-8,3,-20)								
**LiNbO3**	**(2,-3,-28)**	**13.05**	**75.59**	**7.479**	**6.7**	**3.462**	**23.03**	**43.27**	**49.02**
	Equiv. Refl.: (3,-2,-28)								
**LiNbO3**	**(1,-3,28)**	**13.05**	**75.59**	**7.396**	**6.7**	**3.462**	**23.03**	**43.27**	**49.02**
	Equiv. Refl.: (2,1,28), (3,-1,28), (-1,-2,28), (-2,3,28), (-3,2,28)								
**LiNbO3**	**(4,6,10)**	**12.921**	**73.52**	**7.37**	**6.6**	**3.986**	**26.12**	**49.82**	**56.26**
	Equiv. Refl.: (6,-10,10), (10,-6,10), (-4,10,10), (-6,-4,10), (-10,4,10)								
**LiNbO3**	**(2,-9,14)**	**12.99**	**74.59**	**7.356**	**6.7**	**3.713**	**24.81**	**46.41**	**52.63**
	Equiv. Refl.: (7,2,14), (9,-2,14), (-2,-7,14), (-7,9,14), (-9,7,14)								

Ei =	13.474	keV						ΔEg	ΔEt
**Cryst**	**Refl (h,k,l)**	**EB [keV]**	**ΘB [°]**	**∫ IR dΘ [µrad]**	**Width [µrad]**	**Ei cotΘ [meV/µrad]**	**ΔE [meV]**	**2m, 50µm [meV]**	**2m, 50µm [meV]**
**LiNbO3**	**(0,-9,6)**	**12.799**	**71.78**	**7.191**	**6.2**	**4.434**	**27.71**	**55.43**	**61.97**
	Equiv. Refl.: (9,0,6), (-9,9,6)								
**LiNbO3**	**(0,9,-6)**	**12.799**	**71.78**	**7.151**	**6.2**	**4.434**	**27.71**	**55.43**	**61.97**
	Equiv. Refl.: (9,-9,-6), (-9,0,-6)								
**LiNbO3**	**(2,-10,0)**	**12.744**	**71.05**	**7.137**	**6.2**	**4.625**	**28.48**	**57.82**	**64.45**
	Equiv. Refl.: (8,2,0), (10,-2,0), (-2,-8,0), (-8,10,0), (-10,8,0)								
**LiNbO3**	**(0,-8,14)**	**12.765**	**71.32**	**7.065**	**5.9**	**4.554**	**26.9**	**56.93**	**62.96**
	Equiv. Refl.: (8,0,14), (-8,8,14)								
**LiNbO3**	**(2,8,0)**	**12.744**	**71.05**	**7.041**	**6.2**	**4.625**	**28.48**	**57.82**	**64.45**
	Equiv. Refl.: (8,-10,0), (10,-8,0), (-2,10,0), (-8,-2,0), (-10,2,0)								
**LiNbO3**	**(1,-7,20)**	**12.772**	**71.42**	**6.986**	**6**	**4.528**	**27.35**	**56.6**	**62.86**
	Equiv. Refl.: (6,1,20), (7,-1,20), (-1,-6,20), (-6,7,20), (-7,6,20)								
**LiNbO3**	**(3,7,8)**	**12.866**	**72.73**	**6.954**	**6.1**	**4.19**	**25.46**	**52.37**	**58.23**
	Equiv. Refl.: (7,-10,8), (10,-7,8), (-3,10,8), (-7,-3,8), (-10,3,8)								
**LiNbO3**	**(-1,7,-20)**	**12.772**	**71.42**	**6.833**	**6**	**4.528**	**27.35**	**56.6**	**62.86**
	Equiv. Refl.: (-7,1,-20)								
**LiNbO3**	**(1,6,-20)**	**12.772**	**71.42**	**6.832**	**6**	**4.528**	**27.35**	**56.6**	**62.86**
	Equiv. Refl.: (6,-7,-20), (7,-6,-20), (-6,-1,-20)								
**LiNbO3**	**(3,-10,-8)**	**12.866**	**72.73**	**6.812**	**6.1**	**4.19**	**25.46**	**52.37**	**58.23**
	Equiv. Refl.: (7,3,-8), (10,-3,-8), (-3,-7,-8), (-7,10,-8), (-10,7,-8)								
**LiNbO3**	**(2,8,6)**	**13.023**	**75.14**	**6.584**	**5.9**	**3.575**	**21**	**44.69**	**49.38**
	Equiv. Refl.: (8,-10,6), (10,-8,6), (-2,10,6), (-8,-2,6), (-10,2,6)								
**LiNbO3**	**(3,-8,20)**	**13.218**	**78.82**	**6.563**	**6.6**	**2.663**	**17.55**	**33.28**	**37.62**
	Equiv. Refl.: (5,3,20), (8,-3,20), (-3,-5,20), (-5,8,20), (-8,5,20)								
**LiNbO3**	**(3,4,-22)**	**12.974**	**74.34**	**6.493**	**5.8**	**3.777**	**21.77**	**47.21**	**51.99**
	Equiv. Refl.: (4,-7,-22), (7,-4,-22), (-3,7,-22), (-4,-3,-22), (-7,3,-22)								
**Sapph**	**(3,4,20)**	**13.224**	**78.94**	**6.473**	**5.2**	**2.634**	**13.61**	**32.93**	**35.63**
	Equiv. Refl.: (3,-7,-20), (4,3,-20), (4,-7,20), (7,-3,-20), (7,-4,20), (-3,7,20), (-3,-4,-20), (-4,7,-20), (-4,-3,20) (-7,3,20), (-7,4,-20)								
**LiNbO3**	**(0,8,-14)**	**12.765**	**71.32**	**6.417**	**5.9**	**4.554**	**26.9**	**56.93**	**62.96**
	Equiv. Refl.: (8,-8,-14), (-8,0,-14)								
**LiNbO3**	**(2,-10,-6)**	**13.023**	**75.14**	**6.317**	**5.9**	**3.575**	**21**	**44.69**	**49.38**
	Equiv. Refl.: (8,2,-6), (10,-2,-6), (-2,-8,-6), (-8,10,-6), (-10,8,-6)								
**LiNbO3**	**(3,-7,22)**	**12.974**	**74.34**	**6.232**	**5.8**	**3.777**	**21.77**	**47.21**	**51.99**
	Equiv. Refl.: (4,3,22), (7,-3,22), (-3,-4,22), (-4,7,22), (-7,4,22)								
**LiNbO3**	**(0,-9,-6)**	**12.799**	**71.78**	**6.153**	**5.3**	**4.434**	**23.65**	**55.43**	**60.26**
	Equiv. Refl.: (9,0,-6), (-9,9,-6)								
**Sapph**	**(5,5,6)**	**13.34**	**81.9**	**6.04**	**5**	**1.917**	**9.67**	**23.96**	**25.84**
	Equiv. Refl.: (5,5,-6), (5,-10,6), (5,-10,-6), (10,-5,6), (10,-5,-6), (-5,10,6), (-5,10,-6), (-5,-5,6), (-5,-5,-6) (-10,5,6), (-10,5,-6)								
**LiNbO3**	**(2,-10,6)**	**13.023**	**75.14**	**5.818**	**5.4**	**3.575**	**19.2**	**44.69**	**48.64**
	Equiv. Refl.: (8,2,6), (10,-2,6), (-2,-8,6), (-8,10,6), (-10,8,6)								
**LiNbO3**	**(2,8,-6)**	**13.023**	**75.14**	**5.766**	**5.4**	**3.575**	**19.2**	**44.69**	**48.64**
	Equiv. Refl.: (8,-10,-6), (10,-8,-6), (-2,10,-6), (-8,-2,-6), (-10,2,-6)								
**LiNbO3**	**(0,9,6)**	**12.799**	**71.78**	**5.752**	**5.3**	**4.434**	**23.65**	**55.43**	**60.26**
	Equiv. Refl.: (9,-9,6), (-9,0,6)								
**LiNbO3**	**(0,4,26)**	**12.888**	**73.05**	**5.677**	**4.8**	**4.107**	**19.77**	**51.34**	**55.02**
	Equiv. Refl.: (4,-4,26), (-4,0,26)								
**LiNbO3**	**(1,8,-10)**	**12.694**	**70.41**	**5.67**	**5**	**4.795**	**23.95**	**59.94**	**64.55**
	Equiv. Refl.: (8,-9,-10), (9,-8,-10), (-1,9,-10), (-8,-1,-10), (-9,1,-10)								

Ei =	13.474	keV						ΔEg	ΔEt
Cryst	Refl	EB	ΘB	∫ IR dΘ	Width	Ei cotΘ	ΔE	2m, 50µm	2m, 50µm
	(h,k,l)	[keV]	[°]	[µrad]	[µrad]	[meV/µrad]	[meV]	[meV]	[meV]
**LiNbO3**	**(3,3,-24)**	**12.938**	**73.78**	**5.571**	**5.1**	**3.92**	**19.8**	**48.99**	**52.85**
	Equiv. Refl.: (3,-6,-24), (6,-3,-24), (-3,6,-24), (-3,-3,-24), (-6,3,-24)								
**LiNbO3**	**(1,-9,10)**	**12.694**	**70.41**	**5.464**	**5**	**4.795**	**23.95**	**59.94**	**64.55**
	Equiv. Refl.: (8,1,10), (9,-1,10), (-1,-8,10), (-8,9,10), (-9,8,10)								
**LiNbO3**	**(0,-4,-26)**	**12.888**	**73.05**	**5.454**	**4.8**	**4.107**	**19.77**	**51.34**	**55.02**
	Equiv. Refl.: (4,0,-26), (-4,4,-26)								
**LiNbO3**	**(3,3,24)**	**12.938**	**73.78**	**5.365**	**5.1**	**3.92**	**19.8**	**48.99**	**52.85**
	Equiv. Refl.: (3,-6,24), (6,-3,24), (-3,6,24), (-3,-3,24), (-6,3,24)								
**LiNbO3**	**(0,-2,-28)**	**12.826**	**72.16**	**5.321**	**4.9**	**4.337**	**21.07**	**54.21**	**58.16**
	Equiv. Refl.: (2,0,-28), (-2,2,-28)								
**LiNbO3**	**(0,2,28)**	**12.826**	**72.16**	**5.29**	**4.9**	**4.337**	**21.07**	**54.21**	**58.16**
	Equiv. Refl.: (2,-2,28), (-2,0,28)								
**LiNbO3**	**(1,9,-5)**	**13.452**	**86.69**	**5.236**	**7.4**	**0.779**	**5.78**	**9.74**	**11.33**
	Equiv. Refl.: (1,-10,5), (9,1,5), (9,-10,-5), (10,-1,5), (10,-9,-5), (-1,10,-5), (-1,-9,5), (-9,10,5), (-9,-1,-5) (-10,1,-5), (-10,9,5)								
**LiNbO3**	**(1,-4,26)**	**12.661**	**70**	**5.018**	**4.3**	**4.904**	**20.97**	**61.3**	**64.79**
	Equiv. Refl.: (3,1,26), (4,-1,26), (-1,-3,26), (-3,4,26), (-4,3,26)								
**Quartz**	**(-3,9,-6)**	**13.457**	**87.15**	**5.012**	**4.5**	**0.67**	**3.04**	**8.38**	**8.91**
**Quartz**	**(9,-3,6)**	**13.457**	**87.15**	**5.009**	**4.5**	**0.67**	**3.04**	**8.38**	**8.91**
	Equiv. Refl.: (9,-6,-6), (-3,-6,6)								
**Quartz**	**(-6,9,6)**	**13.457**	**87.15**	**5.007**	**4.5**	**0.67**	**3.03**	**8.38**	**8.91**
	Equiv. Refl.: (-6,-3,-6)								
**LiNbO3**	**(1,3,-26)**	**12.661**	**70**	**4.925**	**4.3**	**4.904**	**20.97**	**61.3**	**64.79**
	Equiv. Refl.: (3,-4,-26), (4,-3,-26), (-1,4,-26), (-3,-1,-26), (-4,1,-26)								
**Sapph**	**(2,6,-16)**	**13.267**	**79.94**	**4.898**	**3.9**	**2.39**	**9.39**	**29.88**	**31.32**
	Equiv. Refl.: (2,-8,16), (6,2,16), (6,-8,-16), (8,-2,16), (8,-6,-16), (-2,8,-16), (-2,-6,16), (-6,8,16), (-6,-2,-16) (-8,2,-16), (-8,6,16)								
**Quartz**	**(10,-3,3)**	**13.399**	**83.97**	**4.794**	**3.8**	**1.424**	**5.39**	**17.8**	**18.6**
	Equiv. Refl.: (10,-7,-3), (-7,10,3), (-7,-3,-3)								
**Quartz**	**(-3,10,-3)**	**13.399**	**83.97**	**4.792**	**3.8**	**1.424**	**5.38**	**17.8**	**18.6**
	Equiv. Refl.: (-3,-7,3)								
**Quartz**	**(4,1,10)**	**13.272**	**80.06**	**4.689**	**3.6**	**2.36**	**8.5**	**29.5**	**30.7**
	Equiv. Refl.: (4,-5,-10)								
**Quartz**	**(1,4,-10)**	**13.272**	**80.06**	**4.686**	**3.6**	**2.36**	**8.5**	**29.5**	**30.7**
**Quartz**	**(1,-5,10)**	**13.272**	**80.06**	**4.685**	**3.6**	**2.36**	**8.5**	**29.5**	**30.7**
**Quartz**	**(-5,1,-10)**	**13.272**	**80.06**	**4.681**	**3.6**	**2.36**	**8.49**	**29.5**	**30.7**
	Equiv. Refl.: (-5,4,10)								
**Quartz**	**(-4,5,10)**	**13.272**	**80.06**	**4.585**	**3.6**	**2.36**	**8.5**	**29.5**	**30.7**
	Equiv. Refl.: (-4,-1,-10)								
**Quartz**	**(-1,-4,10)**	**13.272**	**80.06**	**4.582**	**3.6**	**2.36**	**8.5**	**29.5**	**30.7**
**Quartz**	**(-1,5,-10)**	**13.272**	**80.06**	**4.581**	**3.6**	**2.36**	**8.5**	**29.5**	**30.7**
**Quartz**	**(5,-1,10)**	**13.272**	**80.06**	**4.577**	**3.6**	**2.36**	**8.49**	**29.5**	**30.7**
	Equiv. Refl.: (5,-4,-10)								
**Quartz**	**(3,-9,6)**	**13.457**	**87.15**	**4.475**	**4.5**	**0.67**	**3.04**	**8.38**	**8.91**
**Quartz**	**(3,6,-6)**	**13.457**	**87.15**	**4.472**	**4.5**	**0.67**	**3.04**	**8.38**	**8.91**
**Quartz**	**(-9,3,-6)**	**13.457**	**87.15**	**4.471**	**4.5**	**0.67**	**3.04**	**8.38**	**8.91**
	Equiv. Refl.: (-9,6,6)								
**Quartz**	**(6,3,6)**	**13.457**	**87.15**	**4.469**	**4.5**	**0.67**	**3.03**	**8.38**	**8.91**
	Equiv. Refl.: (6,-9,-6)								
**Quartz**	**(3,-3,11)**	**13.353**	**82.31**	**4.321**	**3.5**	**1.819**	**6.42**	**22.74**	**23.63**
	Equiv. Refl.: (-3,3,-11)								

Ei = Cryst	13.474 Refl (h,k,l)	keV EB [keV]	ΘB [°]	∫ IR dΘ [μrad]	Width [μrad]	Ei cotΘ [meV/μrad]	ΔE [meV]	ΔEg 2m, 50μm [meV]	ΔEt 2m, 50μm [meV]
**Quartz**	**(3,0,-11)**	**13.353**	**82.31**	**4.32**	**3.5**	**1.819**	**6.42**	**22.74**	**23.63**
	Equiv. Refl.: (-3,0,11)								
**Quartz**	**(0,3,11)**	**13.353**	**82.31**	**4.309**	**3.5**	**1.819**	**6.4**	**22.74**	**23.62**
	Equiv. Refl.: (0,-3,-11)								
**Quartz**	**(7,3,3)**	**13.399**	**83.97**	**4.299**	**3.8**	**1.424**	**5.39**	**17.8**	**18.6**
	Equiv. Refl.: (-10,7,3)								
**Quartz**	**(7,-10,-3)**	**13.399**	**83.97**	**4.298**	**3.8**	**1.424**	**5.39**	**17.8**	**18.6**
	Equiv. Refl.: (-10,3,-3)								
**Quartz**	**(3,7,-3)**	**13.399**	**83.97**	**4.297**	**3.8**	**1.424**	**5.38**	**17.8**	**18.6**
	Equiv. Refl.: (3,-10,3)								
**Quartz**	**(8,0,-5)**	**12.99**	**74.6**	**4.29**	**3.2**	**3.711**	**11.87**	**46.38**	**47.88**
	Equiv. Refl.: (8,-8,5), (-8,0,5), (-8,8,-5)								
**Quartz**	**(0,8,5)**	**12.99**	**74.6**	**4.288**	**3.2**	**3.711**	**11.87**	**46.38**	**47.88**
	Equiv. Refl.: (0,-8,-5)								
**Sapph**	**(1,8,-4)**	**12.995**	**74.67**	**4.235**	**3.3**	**3.693**	**12.13**	**46.16**	**47.73**
	Equiv. Refl.: (1,-9,4), (8,1,4), (8,-9,-4), (9,-1,4), (9,-8,-4), (-1,9,-4), (-1,-8,4), (-8,9,4), (-8,-1,-4) (-9,1,-4), (-9,8,4)								
**Quartz**	**(2,5,-8)**	**12.923**	**73.55**	**4.202**	**3.1**	**3.978**	**12.37**	**49.73**	**51.24**
	Equiv. Refl.: (2,-7,8)								
**Quartz**	**(-7,2,-8)**	**12.923**	**73.55**	**4.201**	**3.1**	**3.978**	**12.37**	**49.73**	**51.24**
	Equiv. Refl.: (-7,5,8)								
**Quartz**	**(5,2,8)**	**12.923**	**73.55**	**4.2**	**3.1**	**3.978**	**12.36**	**49.73**	**51.24**
	Equiv. Refl.: (5,-7,-8)								
**Quartz**	**(-2,7,-8)**	**12.923**	**73.55**	**4.198**	**3.1**	**3.978**	**12.37**	**49.73**	**51.24**
	Equiv. Refl.: (-2,-5,8)								
**Quartz**	**(7,-2,8)**	**12.923**	**73.55**	**4.197**	**3.1**	**3.978**	**12.37**	**49.73**	**51.24**
	Equiv. Refl.: (7,-5,-8)								
**Quartz**	**(-5,7,8)**	**12.923**	**73.55**	**4.196**	**3.1**	**3.978**	**12.36**	**49.73**	**51.24**
	Equiv. Refl.: (-5,-2,-8)								
**Quartz**	**(5,-2,-10)**	**13.111**	**76.67**	**4.126**	**3.1**	**3.192**	**9.94**	**39.91**	**41.12**
	Equiv. Refl.: (-3,5,-10), (-3,-2,10)								
**Quartz**	**(5,-3,10)**	**13.111**	**76.67**	**4.125**	**3.1**	**3.192**	**9.94**	**39.91**	**41.12**
	Equiv. Refl.: (-2,5,10), (-2,-3,-10)								
**Sapph**	**(2,7,10)**	**13.207**	**78.57**	**4.102**	**3.3**	**2.724**	**8.98**	**34.05**	**35.21**
	Equiv. Refl.: (2,-9,-10), (7,2,-10), (7,-9,10), (9,-2,-10), (9,-7,10), (-2,9,10), (-2,-7,-10), (-7,9,-10), (-7,-2,10) (-9,2,10), (-9,7,-10)								
**Quartz**	**(3,2,-10)**	**13.111**	**76.67**	**4.082**	**3.1**	**3.192**	**9.94**	**39.91**	**41.12**
	Equiv. Refl.: (3,-5,10)								
**Quartz**	**(2,3,10)**	**13.111**	**76.67**	**4.081**	**3.1**	**3.192**	**9.93**	**39.91**	**41.12**
	Equiv. Refl.: (-5,2,10), (-5,3,-10)								
**Quartz**	**(2,-5,-10)**	**13.111**	**76.67**	**4.08**	**3.1**	**3.192**	**9.93**	**39.91**	**41.12**
**Sapph**	**(2,6,14)**	**12.741**	**71.02**	**3.806**	**2.9**	**4.635**	**13.54**	**57.93**	**59.49**
	Equiv. Refl.: (2,-8,-14), (6,2,-14), (6,-8,14), (8,-2,-14), (8,-6,14), (-2,8,14), (-2,-6,-14), (-6,8,-14), (-6,-2,14) (-8,2,14), (-8,6,-14)								
**LiNbO3**	**(4,6,13)**	**13.444**	**86.17**	**3.803**	**5.9**	**0.903**	**5.33**	**11.29**	**12.48**
	Equiv. Refl.: (4,-10,-13), (6,4,-13), (6,-10,13), (10,-4,-13), (10,-6,13), (-4,10,13), (-4,-6,-13), (-6,10,-13), (-6,-4,13) (-10,4,13), (-10,6,-13)								

Ei =	13.474	keV						ΔEg	ΔEt
Cryst	Refl	EB	ΘB	∫ IR dΘ	Width	Ei cotΘ	ΔE	2m, 50μm	2m, 50μm
	(h,k,l)	[keV]	[°]	[μrad]	[μrad]	[meV/μrad]	[meV]	[meV]	[meV]
**Sapph**	**(1,7,15)**	**13.426**	**85.17**	**3.572**	**3.3**	**1.138**	**3.79**	**14.22**	**14.72**
	Equiv. Refl.: (1,7,-15), (1,-8,15), (1,-8,-15), (7,1,15), (7,1,-15), (7,-8,15), (7,-8,-15), (8,-1,15), (8,-1,-15) (8,-7,15), (8,-7,-15), (-1,8,15), (-1,8,-15), (-1,-7,15), (-1,-7,-15), (-7,8,15), (-7,8,-15), (-7,-1,15) (-7,-1,-15), (-8,1,15), (-8,1,-15), (-8,7,15), (-8,7,-15)								
**Quartz**	**(-3,3,11)**	**13.353**	**82.31**	**3.365**	**3**	**1.819**	**5.54**	**22.74**	**23.4**
**Quartz**	**(0,3,-11)**	**13.353**	**82.31**	**3.364**	**3**	**1.819**	**5.54**	**22.74**	**23.4**
	Equiv. Refl.: (0,-3,11), (3,-3,-11), (-3,0,-11)								
**Quartz**	**(3,0,11)**	**13.353**	**82.31**	**3.363**	**3**	**1.819**	**5.54**	**22.74**	**23.4**
**Sapph**	**(3,5,16)**	**13.008**	**74.89**	**3.326**	**2.7**	**3.637**	**9.71**	**45.46**	**46.49**
	Equiv. Refl.: (3,-8,-16), (5,3,-16), (5,-8,16), (8,-3,-16), (8,-5,16), (-3,8,16), (-3,-5,-16), (-5,8,-16), (-5,-3,16) (-8,3,16), (-8,5,-16)								
**Sapph**	**(1,8,-7)**	**13.281**	**80.29**	**3.293**	**2.7**	**2.306**	**6.24**	**28.83**	**29.49**
	Equiv. Refl.: (1,-9,7), (8,1,7), (8,-9,-7), (9,-1,7), (9,-8,-7), (-1,9,-7), (-1,-8,7), (-8,9,7), (-8,-1,-7) (-9,1,-7), (-9,8,7)								
**Quartz**	**(9,-4,-5)**	**12.743**	**71.04**	**3.291**	**2.5**	**4.629**	**11.58**	**57.86**	**59.01**
	Equiv. Refl.: (9,-5,5)								
**Quartz**	**(-4,9,5)**	**12.743**	**71.04**	**3.29**	**2.5**	**4.629**	**11.58**	**57.86**	**59.01**
	Equiv. Refl.: (-4,-5,-5), (-5,9,-5), (-5,-4,5)								
**Quartz**	**(-6,4,-9)**	**12.884**	**72.99**	**3.289**	**2.5**	**4.123**	**10.25**	**51.53**	**52.54**
**Quartz**	**(2,4,9)**	**12.884**	**72.99**	**3.288**	**2.5**	**4.123**	**10.25**	**51.53**	**52.54**
	Equiv. Refl.: (2,-6,-9), (-6,2,9)								
**Quartz**	**(4,2,-9)**	**12.884**	**72.99**	**3.287**	**2.5**	**4.123**	**10.24**	**51.53**	**52.54**
	Equiv. Refl.: (4,-6,9)								
**Quartz**	**(-9,4,5)**	**12.743**	**71.04**	**3.28**	**2.5**	**4.629**	**11.58**	**57.86**	**59.01**
	Equiv. Refl.: (-9,5,-5)								
**Quartz**	**(4,5,5)**	**12.743**	**71.04**	**3.279**	**2.5**	**4.629**	**11.58**	**57.86**	**59.01**
	Equiv. Refl.: (4,-9,-5), (5,4,-5), (5,-9,5)								
**Quartz**	**(9,-2,5)**	**13.233**	**79.15**	**3.238**	**2.8**	**2.582**	**7.33**	**32.27**	**33.09**
	Equiv. Refl.: (9,-7,-5)								
**Quartz**	**(-2,9,-5)**	**13.233**	**79.15**	**3.236**	**2.8**	**2.582**	**7.33**	**32.27**	**33.09**
	Equiv. Refl.: (-7,9,5), (-7,-2,-5)								
**Quartz**	**(-2,-7,5)**	**13.233**	**79.15**	**3.235**	**2.8**	**2.582**	**7.33**	**32.27**	**33.09**
**Quartz**	**(-9,2,-5)**	**13.233**	**79.15**	**3.199**	**2.8**	**2.582**	**7.33**	**32.27**	**33.09**
	Equiv. Refl.: (-9,7,5)								
**Quartz**	**(2,-9,5)**	**13.233**	**79.15**	**3.197**	**2.8**	**2.582**	**7.33**	**32.27**	**33.09**
	Equiv. Refl.: (7,2,5), (7,-9,-5)								
**Quartz**	**(2,7,-5)**	**13.233**	**79.15**	**3.196**	**2.8**	**2.582**	**7.33**	**32.27**	**33.09**
**Quartz**	**(6,-4,9)**	**12.884**	**72.99**	**3.189**	**2.5**	**4.123**	**10.25**	**51.53**	**52.54**
**Quartz**	**(6,-2,-9)**	**12.884**	**72.99**	**3.188**	**2.5**	**4.123**	**10.25**	**51.53**	**52.54**
**Quartz**	**(-2,6,9)**	**12.884**	**72.99**	**3.187**	**2.5**	**4.123**	**10.25**	**51.53**	**52.54**
	Equiv. Refl.: (-2,-4,-9), (-4,6,-9), (-4,-2,9)								
**Quartz**	**(4,6,-1)**	**12.753**	**71.18**	**3.15**	**2.4**	**4.593**	**10.86**	**57.41**	**58.43**
	Equiv. Refl.: (4,-10,1), (6,4,1), (6,-10,-1), (-10,4,-1), (-10,6,1)								
**Quartz**	**(10,-4,1)**	**12.753**	**71.18**	**3.054**	**2.4**	**4.593**	**10.87**	**57.41**	**58.43**
	Equiv. Refl.: (10,-6,-1), (-4,10,-1), (-4,-6,1), (-6,10,1), (-6,-4,-1)								
**Sapph**	**(4,6,-2)**	**13.15**	**77.41**	**3.039**	**2.4**	**3.01**	**7.3**	**37.62**	**38.32**
	Equiv. Refl.: (4,-10,2), (6,4,2), (6,-10,-2), (10,-4,2), (10,-6,-2), (-4,10,-2), (-4,-6,2), (-6,10,2), (-6,-4,-2) (-10,4,-2), (-10,6,2)								
**Sapph**	**(1,1,27)**	**13.148**	**77.36**	**3.013**	**2.4**	**3.021**	**7.28**	**37.76**	**38.45**
	Equiv. Refl.: (1,1,-27), (1,-2,27), (1,-2,-27), (2,-1,27), (2,-1,-27), (-1,2,27), (-1,2,-27), (-1,-1,27), (-1,-1,-27) (-2,1,27), (-2,1,-27)								
**Quartz**	**(-1,2,-11)**	**12.867**	**72.74**	**2.986**	**2.2**	**4.187**	**9.22**	**52.34**	**53.14**

Equiv. Refl.: (-1,-1,11), (-1,-1,-11)

Ei =	13.474	keV						ΔEg	ΔEt
Cryst	Refl	EB	ΘB	∫ IR dΘ	Width	Ei cotΘ	ΔE	2m, 50µm	2m, 50µm
	(h,k,l)	[keV]	[°]	[µrad]	[µrad]	[meV/µrad]	[meV]	[meV]	[meV]
Quartz	(9,0,2)	13.312	81.11	2.985	2.7	2.108	5.72	26.35	26.97
	Equiv. Refl.: (9,-9,-2), (-1,2,11), (-9,0,-2), (-9,9,2)								
Quartz	(2,-1,11)	12.867	72.74	2.984	2.2	4.187	9.22	52.34	53.14
	Equiv. Refl.: (2,-1,-11)								
Quartz	(0,9,-2)	13.312	81.11	2.983	2.7	2.108	5.72	26.35	26.97
	Equiv. Refl.: (0,-9,2)								
Quartz	(1,1,11)	12.867	72.74	2.959	2.2	4.187	9.22	52.34	53.14
	Equiv. Refl.: (1,1,-11)								
Quartz	(1,-2,11)	12.867	72.74	2.958	2.2	4.187	9.22	52.34	53.14
	Equiv. Refl.: (1,-2,-11)								
Quartz	(-2,1,11)	12.867	72.74	2.956	2.2	4.187	9.22	52.34	53.14
	Equiv. Refl.: (-2,1,-11)								
Quartz	(3,-1,-11)	13.193	78.28	2.949	2.6	2.796	7.27	34.95	35.7
	Equiv. Refl.: (3,-2,11), (-1,3,11), (-2,3,-11)								
Quartz	(-1,-2,-11)	13.193	78.28	2.948	2.6	2.796	7.26	34.95	35.7
	Equiv. Refl.: (-2,-1,11)								
Quartz	(-3,1,11)	13.193	78.28	2.899	2.6	2.796	7.27	34.95	35.7
Quartz	(1,2,11)	13.193	78.28	2.898	2.6	2.796	7.26	34.95	35.7
	Equiv. Refl.: (1,-3,-11), (2,-3,11), (-3,2,-11)								
Quartz	(2,1,-11)	13.193	78.28	2.897	2.6	2.796	7.26	34.95	35.7
Quartz	(-6,8,7)	13.223	78.93	2.886	2.6	2.637	6.74	32.96	33.64
Quartz	(-2,8,-7)	13.223	78.93	2.885	2.6	2.637	6.74	32.96	33.64
	Equiv. Refl.: (-2,-6,7), (-6,-2,-7)								
Quartz	(8,-2,7)	13.223	78.93	2.883	2.6	2.637	6.73	32.96	33.64
	Equiv. Refl.: (8,-6,-7)								
Quartz	(2,6,-7)	13.223	78.93	2.797	2.6	2.637	6.74	32.96	33.64
	Equiv. Refl.: (2,-8,7), (6,2,7), (6,-8,-7)								
Quartz	(-8,2,-7)	13.223	78.93	2.795	2.6	2.637	6.73	32.96	33.64
	Equiv. Refl.: (-8,6,7)								
Quartz	(-3,9,5)	12.908	73.34	2.657	2	4.032	7.91	50.4	51.02
	Equiv. Refl.: (-3,-6,-5), (-6,9,-5), (-6,-3,5)								
Quartz	(9,-3,-5)	12.908	73.34	2.655	2	4.032	7.9	50.4	51.01
	Equiv. Refl.: (9,-6,5)								
Quartz	(8,1,4)	13.267	79.95	2.439	2.1	2.389	5.06	29.86	30.29
	Equiv. Refl.: (8,-9,-4)								
Quartz	(1,8,-4)	13.267	79.95	2.438	2.1	2.389	5.06	29.86	30.29
	Equiv. Refl.: (-9,1,-4)								
Quartz	(1,-9,4)	13.267	79.95	2.437	2.1	2.389	5.06	29.86	30.29
	Equiv. Refl.: (-9,8,4)								
Quartz	(0,2,11)	12.949	73.96	2.381	2	3.874	7.91	48.43	49.07
	Equiv. Refl.: (0,-2,-11)								
Quartz	(2,0,-11)	12.949	73.96	2.376	2	3.874	7.9	48.43	49.07
	Equiv. Refl.: (2,-2,11), (-2,0,11), (-2,2,-11)								
Quartz	(-1,9,4)	13.267	79.95	2.356	2.1	2.389	4.96	29.86	30.27
	Equiv. Refl.: (-1,-8,-4)								
Quartz	(9,-1,-4)	13.267	79.95	2.355	2.1	2.389	4.96	29.86	30.27
	Equiv. Refl.: (9,-8,4), (-8,9,-4), (-8,-1,4)								
Quartz	(8,0,5)	12.99	74.6	2.336	2	3.711	7.47	46.38	46.98
	Equiv. Refl.: (8,-8,-5), (-8,0,-5), (-8,8,5)								

Ei =	13.474	keV						ΔEg	ΔEt
Cryst	Refl	EB	ΘB	∫ IR dΘ	Width	Ei cotΘ	ΔE	2m, 50μm	2m, 50μm
	(h,k,l)	[keV]	[°]	[μrad]	[μrad]	[meV/μrad]	[meV]	[meV]	[meV]
**Quartz**	**(0,8,-5)**	**12.99**	**74.6**	**2.335**	**2**	**3.711**	**7.47**	**46.38**	**46.98**
	Equiv. Refl.: (0,-8,5)								
**Quartz**	**(2,4,-9)**	**12.884**	**72.99**	**2.318**	**2**	**4.123**	**8.04**	**51.53**	**52.16**
	Equiv. Refl.: (2,-6,9)								
**Quartz**	**(4,2,9)**	**12.884**	**72.99**	**2.315**	**1.9**	**4.123**	**8.04**	**51.53**	**52.16**
	Equiv. Refl.: (4,-6,-9), (-6,2,-9), (-6,4,9)								
**Quartz**	**(-4,7,8)**	**12.757**	**71.23**	**2.312**	**1.9**	**4.58**	**8.92**	**57.25**	**57.94**
**Quartz**	**(7,-3,8)**	**12.757**	**71.23**	**2.311**	**1.9**	**4.58**	**8.92**	**57.25**	**57.94**
	Equiv. Refl.: (7,-4,-8), (-2,6,-9), (-2,-4,9), (-3,7,-8), (-3,-4,8), (-4,-3,-8)								
**Quartz**	**(6,-2,9)**	**12.884**	**72.99**	**2.309**	**1.9**	**4.123**	**8.04**	**51.53**	**52.16**
	Equiv. Refl.: (-4,-2,-9)								
**Quartz**	**(6,-4,-9)**	**12.884**	**72.99**	**2.308**	**1.9**	**4.123**	**8.04**	**51.53**	**52.16**
	Equiv. Refl.: (-4,6,9)								
**Quartz**	**(-10,3,-2)**	**13.152**	**77.44**	**2.295**	**2**	**3.002**	**6.03**	**37.52**	**38**
	Equiv. Refl.: (-10,7,2)								
**Quartz**	**(3,7,-2)**	**13.152**	**77.44**	**2.294**	**2**	**3.002**	**6.03**	**37.52**	**38**
	Equiv. Refl.: (3,-10,2), (7,3,2), (7,-10,-2)								
**Quartz**	**(-8,1,-6)**	**12.975**	**74.37**	**2.289**	**2**	**3.771**	**7.47**	**47.13**	**47.72**
	Equiv. Refl.: (-8,7,6)								
**Quartz**	**(7,1,6)**	**12.975**	**74.37**	**2.288**	**2**	**3.771**	**7.47**	**47.13**	**47.72**
	Equiv. Refl.: (7,-8,-6)								
**Quartz**	**(1,7,-6)**	**12.975**	**74.37**	**2.287**	**2**	**3.771**	**7.46**	**47.13**	**47.72**
	Equiv. Refl.: (1,-8,6)								
**Quartz**	**(4,-7,-8)**	**12.757**	**71.23**	**2.286**	**1.9**	**4.58**	**8.92**	**57.25**	**57.94**
**Quartz**	**(3,4,-8)**	**12.757**	**71.23**	**2.285**	**1.9**	**4.58**	**8.92**	**57.25**	**57.94**
	Equiv. Refl.: (3,-7,8), (4,3,8), (-7,3,-8), (-7,4,8)								
**Quartz**	**(-8,3,7)**	**12.98**	**74.44**	**2.284**	**2**	**3.752**	**7.38**	**46.9**	**47.48**
	Equiv. Refl.: (-8,5,-7)								
**Quartz**	**(3,5,7)**	**12.98**	**74.44**	**2.283**	**2**	**3.752**	**7.38**	**46.9**	**47.48**
	Equiv. Refl.: (3,-8,-7)								
**Quartz**	**(5,3,-7)**	**12.98**	**74.44**	**2.281**	**2**	**3.752**	**7.37**	**46.9**	**47.47**
	Equiv. Refl.: (5,-8,7)								
**Quartz**	**(8,-1,6)**	**12.975**	**74.37**	**2.269**	**2**	**3.771**	**7.47**	**47.13**	**47.72**
	Equiv. Refl.: (8,-3,-7), (8,-5,7), (8,-7,-6), (-7,-1,-6)								
**Quartz**	**(-3,-5,-7)**	**12.98**	**74.44**	**2.268**	**2**	**3.752**	**7.38**	**46.9**	**47.48**
	Equiv. Refl.: (-7,8,6)								
**Quartz**	**(-1,8,-6)**	**12.975**	**74.37**	**2.267**	**2**	**3.771**	**7.46**	**47.13**	**47.72**
	Equiv. Refl.: (-1,-7,6), (-3,8,7)								
**Quartz**	**(-5,8,-7)**	**12.98**	**74.44**	**2.265**	**2**	**3.752**	**7.37**	**46.9**	**47.47**
	Equiv. Refl.: (-5,-3,7)								
**LiNbO3**	**(2,4,25)**	**13.383**	**83.35**	**2.253**	**3.6**	**1.57**	**5.63**	**19.63**	**20.42**
	Equiv. Refl.: (2,-6,-25), (4,2,-25), (4,-6,25), (6,-2,-25), (6,-4,25), (-2,6,25), (-2,-4,-25), (-4,6,-25), (-4,-2,25) (-6,2,25), (-6,4,-25)								
**Quartz**	**(2,8,0)**	**13.353**	**82.33**	**1.985**	**2.2**	**1.814**	**3.94**	**22.68**	**23.02**
	Equiv. Refl.: (2,-10,0), (8,2,0), (8,-10,0), (-10,2,0), (-10,8,0)								
**Sapph**	**(4,5,-13)**	**13.288**	**80.46**	**1.981**	**2.2**	**2.265**	**4.97**	**28.31**	**28.75**
	Equiv. Refl.: (4,-9,13), (5,4,13), (5,-9,-13), (9,-4,13), (9,-5,-13), (-4,9,-13), (-4,-5,13), (-5,9,13), (-5,-4,-13) (-9,4,-13), (-9,5,13)								
**Quartz**	**(10,-2,0)**	**13.353**	**82.33**	**1.965**	**2.2**	**1.814**	**3.94**	**22.68**	**23.02**
	Equiv. Refl.: (10,-8,0), (-2,10,0), (-2,-8,0), (-8,10,0), (-8,-2,0)								

Ei =	13.474	keV						ΔEg	ΔEt
**Cryst**	**Refl**	**EB**	**ΘB**	**∫ IR dΘ**	**Width**	**Ei cotΘ**	**ΔE**	**2m, 50μm**	**2m, 50μm**
	**(h,k,l)**	**[keV]**	**[°]**	**[μrad]**	**[μrad]**	**[meV/μrad]**	**[meV]**	**[meV]**	**[meV]**
**Sapph**	**(3,6,12)**	**13.244**	**79.39**	**1.954**	**2.1**	**2.525**	**5.4**	**31.56**	**32.02**
	Equiv. Refl.: (3,6,-12), (3,-9,12), (3,-9,-12), (6,3,12), (6,3,-12), (6,-9,12), (6,-9,-12), (9,-3,12), (9,-3,-12) (9,-6,12), (9,-6,-12), (-3,9,12), (-3,9,-12), (-3,-6,12), (-3,-6,-12), (-6,9,12), (-6,9,-12), (-6,-3,12) (-6,-3,-12), (-9,3,12), (-9,3,-12), (-9,6,12), (-9,6,-12)								
**Quartz**	**(-8,9,4)**	**13.267**	**79.95**	**1.931**	**2.1**	**2.389**	**5.06**	**29.86**	**30.29**
	Equiv. Refl.: (-8,-1,-4)								
**Quartz**	**(9,-1,4)**	**13.267**	**79.95**	**1.929**	**2.1**	**2.389**	**5.06**	**29.86**	**30.29**
	Equiv. Refl.: (9,-8,-4), (-1,9,-4), (-1,-8,4)								
**Quartz**	**(1,8,4)**	**13.267**	**79.95**	**1.921**	**2.1**	**2.389**	**4.96**	**29.86**	**30.27**
	Equiv. Refl.: (1,-9,-4), (8,1,-4), (8,-9,4), (-9,1,4), (-9,8,-4)								
**Quartz**	**(10,-3,2)**	**13.152**	**77.44**	**1.901**	**2**	**3.002**	**6.03**	**37.52**	**38**
	Equiv. Refl.: (10,-7,-2), (-3,10,-2), (-3,-7,2), (-7,10,2)								
**Quartz**	**(-7,-3,-2)**	**13.152**	**77.44**	**1.9**	**2**	**3.002**	**6.03**	**37.52**	**38**
**Quartz**	**(9,0,-1)**	**13.163**	**77.66**	**1.891**	**2**	**2.946**	**5.85**	**36.83**	**37.29**
	Equiv. Refl.: (9,-9,1), (-9,9,-1)								
**Quartz**	**(0,9,1)**	**13.163**	**77.66**	**1.89**	**2**	**2.946**	**5.84**	**36.83**	**37.29**
	Equiv. Refl.: (0,-9,-1), (-9,0,1)								
**Quartz**	**(3,6,5)**	**12.908**	**73.34**	**1.869**	**2**	**4.032**	**7.91**	**50.4**	**51.02**
	Equiv. Refl.: (3,-9,-5), (6,3,-5), (6,-9,5)								
**Quartz**	**(-9,3,5)**	**12.908**	**73.34**	**1.868**	**2**	**4.032**	**7.9**	**50.4**	**51.01**
	Equiv. Refl.: (-9,6,-5)								
**Quartz**	**(-2,9,4)**	**12.778**	**71.5**	**1.863**	**1.9**	**4.507**	**8.43**	**56.34**	**56.97**
	Equiv. Refl.: (-2,-7,-4), (-7,9,-4), (-7,-2,4)								
**Quartz**	**(9,-2,-4)**	**12.778**	**71.5**	**1.862**	**1.9**	**4.507**	**8.43**	**56.34**	**56.97**
	Equiv. Refl.: (9,-7,4)								
**Sapph**	**(1,8,5)**	**13.073**	**75.99**	**1.836**	**2**	**3.361**	**6.6**	**42.01**	**42.53**
	Equiv. Refl.: (1,-9,-5), (8,1,-5), (8,-9,5), (9,-1,-5), (9,-8,5), (-1,9,5), (-1,-8,-5), (-8,9,-5), (-8,-1,5) (-9,1,5), (-9,8,-5)								
**Quartz**	**(2,7,4)**	**12.778**	**71.5**	**1.802**	**1.9**	**4.507**	**8.43**	**56.34**	**56.97**
	Equiv. Refl.: (2,-9,-4), (7,2,-4), (7,-9,4)								
**Quartz**	**(-9,2,4)**	**12.778**	**71.5**	**1.801**	**1.9**	**4.507**	**8.43**	**56.34**	**56.97**
	Equiv. Refl.: (-9,7,-4)								
**Sapph**	**(5,5,0)**	**13.029**	**75.23**	**1.782**	**1.9**	**3.553**	**6.75**	**44.41**	**44.92**
	Equiv. Refl.: (5,-10,0), (10,-5,0), (-5,10,0), (-5,-5,0), (-10,5,0)								
**Sapph**	**(0,8,10)**	**12.947**	**73.93**	**1.758**	**1.9**	**3.882**	**7.23**	**48.53**	**49.07**
	Equiv. Refl.: (0,-8,-10), (8,0,-10), (8,-8,10), (-8,0,10), (-8,8,-10)								
**Quartz**	**(1,7,6)**	**12.975**	**74.37**	**1.718**	**1.7**	**3.771**	**6.5**	**47.13**	**47.58**
	Equiv. Refl.: (1,-8,-6), (-8,1,6), (-8,7,-6)								
**Quartz**	**(7,1,-6)**	**12.975**	**74.37**	**1.717**	**1.7**	**3.771**	**6.5**	**47.13**	**47.58**
	Equiv. Refl.: (7,-8,6)								
**Quartz**	**(4,-4,-10)**	**12.866**	**72.72**	**1.676**	**1.7**	**4.192**	**7.25**	**52.39**	**52.89**
**Quartz**	**(0,4,-10)**	**12.866**	**72.72**	**1.675**	**1.7**	**4.192**	**7.25**	**52.39**	**52.89**
	Equiv. Refl.: (0,-4,10), (4,0,10), (-4,0,-10), (-4,4,10)								
**Quartz**	**(3,7,0)**	**12.95**	**73.97**	**1.673**	**1.7**	**3.872**	**6.55**	**48.4**	**48.84**
	Equiv. Refl.: (3,-10,0), (6,-3,9), (6,-3,-9), (7,3,0), (7,-10,0), (-3,6,9), (-3,6,-9), (-3,-3,9), (-3,-3,-9) (-10,3,0), (-10,7,0)								
**Quartz**	**(3,3,9)**	**12.802**	**71.82**	**1.666**	**1.7**	**4.424**	**7.6**	**55.3**	**55.82**
	Equiv. Refl.: (3,3,-9), (3,-6,9), (3,-6,-9), (-6,3,9), (-6,3,-9)								
**LiNbO3**	**(1,9,1)**	**13.272**	**80.06**	**1.642**	**2.6**	**2.36**	**6.02**	**29.5**	**30.11**
	Equiv. Refl.: (1,-10,-1), (9,1,-1), (9,-10,1), (10,-1,-1), (10,-9,1), (-1,10,1), (-1,-9,-1), (-9,10,-1), (-9,-1,1) (-10,1,1), (-10,9,-1)								

Ei = Cryst	13.474 Refl (h,k,l)	keV EB [keV]	ΘB [°]	∫ IR dΘ [μrad]	Width [μrad]	Ei cotΘ [meV/μrad]	ΔE [meV]	ΔEg 2m, 50μm [meV]	ΔEt 2m, 50μm [meV]
**Sapph**	**(2,7,-11)**	**13.387**	**83.48**	**1.641**	**2**	**1.541**	**3.07**	**19.26**	**19.51**
	Equiv. Refl.: (2,-9,11), (7,2,11), (7,-9,-11), (9,-2,11), (9,-7,-11), (-2,9,-11), (-2,-7,11), (-7,9,11), (-7,-2,-11) (-9,2,-11), (-9,7,11)								
**Sapph**	**(4,5,-10)**	**12.682**	**70.26**	**1.619**	**1.7**	**4.835**	**8.22**	**60.43**	**60.99**
	Equiv. Refl.: (4,-9,10), (5,4,10), (5,-9,-10), (9,-4,10), (9,-5,-10), (-4,9,-10), (-4,-5,10), (-5,9,10), (-5,-4,-10) (-9,4,-10), (-9,5,10), (5,5,4), (5,-10,-4)								
**Quartz**	**(5,5,-4)**	**13.426**	**85.17**	**1.614**	**2**	**1.139**	**2.24**	**14.23**	**14.41**
	Equiv. Refl.: (5,-10,4), (-10,5,4)								
**Quartz**	**(-10,5,-4)**	**13.426**	**85.17**	**1.613**	**2**	**1.139**	**2.24**	**14.23**	**14.41**
**Quartz**	**(-5,10,4)**	**13.426**	**85.17**	**1.605**	**2**	**1.139**	**2.25**	**14.23**	**14.41**
**Quartz**	**(-5,-5,-4)**	**13.426**	**85.17**	**1.604**	**2**	**1.139**	**2.25**	**14.23**	**14.41**
**Quartz**	**(-5,-5,4)**	**13.426**	**85.17**	**1.6**	**2**	**1.139**	**2.24**	**14.23**	**14.41**
**Quartz**	**(10,-5,4)**	**13.426**	**85.17**	**1.599**	**2**	**1.139**	**2.24**	**14.23**	**14.41**
	Equiv. Refl.: (10,-5,-4), (-5,10,-4)								
**Sapph**	**(1,2,26)**	**13.032**	**75.29**	**1.598**	**1.7**	**3.538**	**6.11**	**44.22**	**44.64**
	Equiv. Refl.: (1,-3,-26), (2,1,-26), (2,-3,26), (3,-1,-26), (3,-2,26), (-1,3,26), (-1,-2,-26), (-2,3,-26), (-2,-1,26) (-3,1,26), (-3,2,-26)								
**Quartz**	**(8,-1,-6)**	**12.975**	**74.37**	**1.597**	**1.7**	**3.771**	**6.5**	**47.13**	**47.58**
	Equiv. Refl.: (8,-7,6), (-1,8,6), (-1,-7,-6)								
**Quartz**	**(-7,8,-6)**	**12.975**	**74.37**	**1.596**	**1.7**	**3.771**	**6.5**	**47.13**	**47.58**
	Equiv. Refl.: (-7,-1,6)								
**Quartz**	**(10,-3,0)**	**12.95**	**73.97**	**1.578**	**1.7**	**3.872**	**6.55**	**48.4**	**48.84**
	Equiv. Refl.: (10,-7,0), (-3,10,0), (-3,-7,0), (-7,10,0), (-7,-3,0)								
**Quartz**	**(-3,7,8)**	**12.757**	**71.23**	**1.574**	**1.6**	**4.58**	**7.4**	**57.25**	**57.73**
**Quartz**	**(-3,-4,-8)**	**12.757**	**71.23**	**1.573**	**1.6**	**4.58**	**7.4**	**57.25**	**57.73**
	Equiv. Refl.: (-4,7,-8)								
**Quartz**	**(7,-3,-8)**	**12.757**	**71.23**	**1.572**	**1.6**	**4.58**	**7.39**	**57.25**	**57.72**
	Equiv. Refl.: (7,-4,8), (-4,-3,8)								
**Quartz**	**(6,-8,7)**	**13.223**	**78.93**	**1.57**	**1.6**	**2.637**	**4.34**	**32.96**	**33.24**
**Quartz**	**(6,2,-7)**	**13.223**	**78.93**	**1.569**	**1.6**	**2.637**	**4.34**	**32.96**	**33.24**
	Equiv. Refl.: (-8,2,7), (-8,6,-7)								
**Quartz**	**(2,-8,-7)**	**13.223**	**78.93**	**1.568**	**1.6**	**2.637**	**4.34**	**32.96**	**33.24**
**Quartz**	**(2,6,7)**	**13.223**	**78.93**	**1.567**	**1.6**	**2.637**	**4.34**	**32.96**	**33.24**
**Sapph**	**(0,2,-26)**	**12.769**	**71.39**	**1.562**	**1.7**	**4.538**	**7.51**	**56.73**	**57.22**
	Equiv. Refl.: (0,-2,26), (2,0,26), (2,-2,-26), (-2,0,-26), (-2,2,26)								
**Sapph**	**(1,8,-1)**	**12.863**	**72.67**	**1.561**	**1.7**	**4.203**	**7**	**52.54**	**53.01**
	Equiv. Refl.: (1,-9,1), (8,1,1), (8,-9,-1), (9,-1,1), (9,-8,-1), (-1,9,-1), (-1,-8,1), (-8,9,1), (-8,-1,-1) (-9,1,-1), (-9,8,1)								
**Quartz**	**(3,4,8)**	**12.757**	**71.23**	**1.557**	**1.6**	**4.58**	**7.4**	**57.25**	**57.73**
	Equiv. Refl.: (3,-7,-8)								
**Quartz**	**(4,3,-8)**	**12.757**	**71.23**	**1.556**	**1.6**	**4.58**	**7.4**	**57.25**	**57.72**
	Equiv. Refl.: (4,-7,8), (-7,3,8), (-7,4,-8)								
**Sapph**	**(1,5,20)**	**12.7**	**70.48**	**1.548**	**1.6**	**4.776**	**7.8**	**59.7**	**60.21**
	Equiv. Refl.: (1,-6,-20), (5,1,-20), (5,-6,20), (6,-1,-20), (6,-5,20), (-1,6,20), (-1,-5,-20), (-5,6,-20), (-5,-1,20) (-6,1,20), (-6,5,-20)								
**Quartz**	**(4,-8,-7)**	**12.898**	**73.19**	**1.511**	**1.6**	**4.071**	**6.41**	**50.89**	**51.29**
	Equiv. Refl.: (-8,4,7), (-8,4,-7)								
**Quartz**	**(4,4,7)**	**12.898**	**73.19**	**1.51**	**1.6**	**4.071**	**6.41**	**50.89**	**51.29**
	Equiv. Refl.: (4,4,-7), (4,-8,7)								

Ei =	13.474	keV						ΔEg	ΔEt
Cryst	Refl	EB	ΘB	∫ IR dΘ	Width	Ei cotΘ	ΔE	2m, 50µm	2m, 50µm
	(h,k,l)	[keV]	[°]	[µrad]	[µrad]	[meV/µrad]	[meV]	[meV]	[meV]
**LiNbO3**	**(5,6,5)**	**13.452**	**86.69**	**1.506**	**3.6**	**0.779**	**2.81**	**9.74**	**10.14**
	Equiv. Refl.: (5,-11,-5), (6,5,-5), (6,-11,5), (11,-5,-5), (11,-6,5), (-5,11,5), (-5,-6,-5), (-6,11,-5), (-6,-5,5) (-11,5,5), (-11,6,-5), (8,-4,7), (8,-4,-7), (-4,8,7)								
**Quartz**	**(-4,8,-7)**	**12.898**	**73.19**	**1.505**	**1.6**	**4.071**	**6.41**	**50.89**	**51.29**
	Equiv. Refl.: (-4,-4,7), (-4,-4,-7)								
**Quartz**	**(10,-4,-2)**	**12.907**	**73.32**	**1.483**	**1.5**	**4.037**	**6.02**	**50.46**	**50.82**
	Equiv. Refl.: (10,-6,2), (-4,10,2), (-4,-6,-2), (-6,10,-2), (-6,-4,2)								
**Quartz**	**(8,-2,-7)**	**13.223**	**78.93**	**1.463**	**1.6**	**2.637**	**4.34**	**32.96**	**33.24**
	Equiv. Refl.: (8,-6,7), (-6,8,-7), (-6,-2,7)								
**Quartz**	**(-2,8,7)**	**13.223**	**78.93**	**1.461**	**1.6**	**2.637**	**4.34**	**32.96**	**33.24**
**Quartz**	**(-2,-6,-7)**	**13.223**	**78.93**	**1.46**	**1.6**	**2.637**	**4.34**	**32.96**	**33.24**
**Quartz**	**(-1,7,8)**	**13.247**	**79.47**	**1.445**	**1.5**	**2.505**	**3.83**	**31.31**	**31.55**
	Equiv. Refl.: (-1,-6,-8)								
**Quartz**	**(-6,7,-8)**	**13.247**	**79.47**	**1.444**	**1.5**	**2.505**	**3.82**	**31.31**	**31.55**
**Quartz**	**(-6,-1,8)**	**13.247**	**79.47**	**1.443**	**1.5**	**2.505**	**3.82**	**31.31**	**31.55**
**Quartz**	**(7,-1,-8)**	**13.247**	**79.47**	**1.442**	**1.5**	**2.505**	**3.82**	**31.31**	**31.55**
	Equiv. Refl.: (7,-6,8)								
**Sapph**	**(1,5,-22)**	**13.432**	**85.48**	**1.42**	**1.9**	**1.066**	**2.06**	**13.32**	**13.48**
	Equiv. Refl.: (1,-6,22), (5,1,22), (5,-6,-22), (6,-1,22), (6,-5,-22), (-1,6,-22), (-1,-5,22), (-5,6,22), (-5,-1,-22) (-6,1,-22), (-6,5,22)								
**Quartz**	**(7,-1,8)**	**13.247**	**79.47**	**1.411**	**1.5**	**2.505**	**3.84**	**31.31**	**31.55**
	Equiv. Refl.: (7,-6,-8), (-1,7,-8), (-1,-6,8), (-6,7,8), (-6,-1,-8)								
**Quartz**	**(4,5,-6)**	**13.299**	**80.75**	**1.409**	**1.6**	**2.195**	**3.41**	**27.44**	**27.65**
	Equiv. Refl.: (4,-9,6), (-9,4,-6), (-9,5,6)								
**Quartz**	**(5,4,6)**	**13.299**	**80.75**	**1.407**	**1.6**	**2.195**	**3.41**	**27.44**	**27.65**
	Equiv. Refl.: (5,-9,-6)								
**Quartz**	**(-6,1,-9)**	**13.129**	**77.01**	**1.391**	**1.4**	**3.108**	**4.49**	**38.85**	**39.11**
	Equiv. Refl.: (-6,5,9)								
**Quartz**	**(5,-6,-9)**	**13.129**	**77.01**	**1.39**	**1.4**	**3.108**	**4.49**	**38.85**	**39.11**
**Quartz**	**(1,5,-9)**	**13.129**	**77.01**	**1.389**	**1.4**	**3.108**	**4.49**	**38.85**	**39.11**
	Equiv. Refl.: (5,1,9)								
**Quartz**	**(1,-6,9)**	**13.129**	**77.01**	**1.388**	**1.4**	**3.108**	**4.49**	**38.85**	**39.11**
**Quartz**	**(6,1,8)**	**13.247**	**79.47**	**1.366**	**1.5**	**2.505**	**3.85**	**31.31**	**31.55**
	Equiv. Refl.: (6,-7,-8), (-7,1,-8), (-7,6,8)								
**Quartz**	**(0,6,8)**	**12.674**	**70.15**	**1.365**	**1.4**	**4.863**	**6.91**	**60.79**	**61.18**
	Equiv. Refl.: (0,-6,-8), (1,6,-8), (1,-7,8), (6,0,-8), (6,-6,8), (-6,0,8), (-6,6,-8)								
**Quartz**	**(4,6,2)**	**12.907**	**73.32**	**1.358**	**1.5**	**4.037**	**6.02**	**50.46**	**50.82**
	Equiv. Refl.: (4,-10,-2), (6,4,-2), (6,-10,2), (-10,4,2), (-10,6,-2)								
**Quartz**	**(-4,9,-6)**	**13.299**	**80.75**	**1.351**	**1.6**	**2.195**	**3.41**	**27.44**	**27.65**
	Equiv. Refl.: (-4,-5,6)								
**Quartz**	**(9,-4,6)**	**13.299**	**80.75**	**1.35**	**1.6**	**2.195**	**3.41**	**27.44**	**27.65**
	Equiv. Refl.: (9,-5,-6)								
**Quartz**	**(-5,9,6)**	**13.299**	**80.75**	**1.348**	**1.6**	**2.195**	**3.41**	**27.44**	**27.65**
	Equiv. Refl.: (-5,-4,-6)								
**Quartz**	**(1,6,8)**	**13.247**	**79.47**	**1.319**	**1.5**	**2.505**	**3.83**	**31.31**	**31.55**
	Equiv. Refl.: (1,-7,-8), (6,-7,8)								
**Quartz**	**(6,1,-8)**	**13.247**	**79.47**	**1.318**	**1.5**	**2.505**	**3.82**	**31.31**	**31.55**
**Quartz**	**(-7,1,8)**	**13.247**	**79.47**	**1.317**	**1.5**	**2.505**	**3.82**	**31.31**	**31.55**
	Equiv. Refl.: (-7,6,-8)								
**Quartz**	**(10,-4,3)**	**13.16**	**77.6**	**1.296**	**1.4**	**2.963**	**4.12**	**37.04**	**37.27**
	Equiv. Refl.: (10,-6,-3), (-4,10,-3), (-4,-6,3)								

**Ei =**	**13.474**	**keV**						**ΔEg**	**ΔEt**
**Cryst**	**Refl**	**EB**	**ΘB**	**∫ IR dΘ**	**Width**	**Ei cotΘ**	**ΔE**	**2m, 50µm**	**2m, 50µm**
	**(h,k,l)**	**[keV]**	**[°]**	**[µrad]**	**[µrad]**	**[meV/µrad]**	**[meV]**	**[meV]**	**[meV]**
**Quartz**	**(-6,10,3)**	**13.16**	**77.6**	**1.295**	**1.4**	**2.963**	**4.12**	**37.04**	**37.27**
	Equiv. Refl.: (-6,-4,-3)								
**Quartz**	**(10,-4,-1)**	**12.753**	**71.18**	**1.288**	**1.3**	**4.593**	**6.16**	**57.41**	**57.74**
	Equiv. Refl.: (10,-6,1), (-4,10,1), (-4,-6,-1), (-6,10,-1), (-6,-4,1)								
**Quartz**	**(6,-1,9)**	**13.129**	**77.01**	**1.28**	**1.4**	**3.108**	**4.49**	**38.85**	**39.11**
	Equiv. Refl.: (6,-5,-9)								
**Quartz**	**(-1,-5,9)**	**13.129**	**77.01**	**1.279**	**1.4**	**3.108**	**4.49**	**38.85**	**39.11**
	Equiv. Refl.: (-5,6,9), (-5,-1,-9)								
**Quartz**	**(-1,6,-9)**	**13.129**	**77.01**	**1.278**	**1.4**	**3.108**	**4.49**	**38.85**	**39.11**
**Quartz**	**(4,6,1)**	**12.753**	**71.18**	**1.263**	**1.3**	**4.593**	**6.16**	**57.41**	**57.74**
	Equiv. Refl.: (4,-10,-1), (6,4,-1), (6,-10,1), (-10,4,1), (-10,6,-1)								
**Quartz**	**(4,6,-3)**	**13.16**	**77.6**	**1.239**	**1.4**	**2.963**	**4.12**	**37.04**	**37.27**
	Equiv. Refl.: (4,-10,3), (-10,4,-3), (-10,6,3)								
**Quartz**	**(6,4,3)**	**13.16**	**77.6**	**1.238**	**1.4**	**2.963**	**4.12**	**37.04**	**37.27**
	Equiv. Refl.: (6,-10,-3)								
**Quartz**	**(9,0,1)**	**13.163**	**77.66**	**1.221**	**1.3**	**2.946**	**3.97**	**36.83**	**37.04**
	Equiv. Refl.: (9,-9,-1), (-9,0,-1), (-9,9,1)								
**Quartz**	**(0,9,-1)**	**13.163**	**77.66**	**1.22**	**1.3**	**2.946**	**3.97**	**36.83**	**37.04**
	Equiv. Refl.: (0,-9,1)								
**Sapph**	**(4,5,11)**	**12.87**	**72.77**	**1.213**	**1.3**	**4.178**	**5.57**	**52.22**	**52.52**
	Equiv. Refl.: (4,-9,-11), (5,4,-11), (5,-9,11), (9,-4,-11), (9,-5,11), (-4,9,11), (-4,-5,-11), (-5,9,-11), (-5,-4,11) (-9,4,11), (-9,5,-11)								
**Quartz**	**(6,3,5)**	**12.908**	**73.34**	**1.181**	**1.3**	**4.032**	**5.08**	**50.4**	**50.65**
	Equiv. Refl.: (6,-9,-5), (-9,6,5)								
**Quartz**	**(3,6,-5)**	**12.908**	**73.34**	**1.18**	**1.3**	**4.032**	**5.08**	**50.4**	**50.65**
	Equiv. Refl.: (3,-9,5), (-9,3,-5)								
**Quartz**	**(9,-6,-5)**	**12.908**	**73.34**	**1.172**	**1.3**	**4.032**	**5.08**	**50.4**	**50.65**
	Equiv. Refl.: (-6,9,5), (-6,-3,-5)								
**Quartz**	**(9,-3,5)**	**12.908**	**73.34**	**1.171**	**1.3**	**4.032**	**5.08**	**50.4**	**50.65**
	Equiv. Refl.: (-3,9,-5), (-3,-6,5)								
**Quartz**	**(9,-4,5)**	**12.743**	**71.04**	**1.107**	**1.2**	**4.629**	**5.43**	**57.86**	**58.12**
	Equiv. Refl.: (9,-5,-5), (-5,9,5), (-5,-4,-5)								
**Sapph**	**(4,6,4)**	**13.253**	**79.62**	**1.106**	**1.3**	**2.468**	**3.27**	**30.85**	**31.03**
	Equiv. Refl.: (4,-10,-4), (6,4,-4), (6,-10,4), (10,-4,-4), (10,-6,4), (-4,10,4), (-4,-6,-4), (-6,10,-4), (-6,-4,4) (-10,4,4), (-10,6,-4), (-4,9,-5), (-4,-5,5)								
**Quartz**	**(4,-9,5)**	**12.743**	**71.04**	**1.093**	**1.2**	**4.629**	**5.42**	**57.86**	**58.12**
	Equiv. Refl.: (5,4,5), (5,-9,-5), (-9,4,-5), (-9,5,5)								
**Quartz**	**(4,5,-5)**	**12.743**	**71.04**	**1.092**	**1.2**	**4.629**	**5.42**	**57.86**	**58.12**
**Quartz**	**(1,8,3)**	**12.915**	**73.44**	**1.091**	**1.1**	**4.006**	**4.51**	**50.07**	**50.27**
	Equiv. Refl.: (1,-9,-3), (-9,1,3), (-9,8,-3)								
**Quartz**	**(8,1,-3)**	**12.915**	**73.44**	**1.09**	**1.1**	**4.006**	**4.51**	**50.07**	**50.27**
	Equiv. Refl.: (8,-9,3)								
**LiNbO3**	**(4,6,-11)**	**13.082**	**76.15**	**1.086**	**1.7**	**3.323**	**5.79**	**41.54**	**41.94**
	Equiv. Refl.: (4,-10,11), (6,4,11), (6,-10,-11), (10,-4,11), (10,-6,-11), (-4,10,-11), (-4,-6,11), (-6,10,11), (-6,-4,-11) (-10,4,-11), (-10,6,11)								
**LiNbO3**	**(2,7,13)**	**12.78**	**71.53**	**1.046**	**1.6**	**4.499**	**7.06**	**56.24**	**56.68**
	Equiv. Refl.: (2,-9,-13), (7,2,-13), (7,-9,13), (9,-2,-13), (9,-7,13), (-2,9,13), (-2,-7,-13), (-7,9,-13), (-7,-2,13) (-9,2,13), (-9,7,-13)								

Ei =	13.474	keV						ΔEg	ΔEt
**Cryst**	**Refl**	**EB**	**ΘB**	**∫ IR dΘ**	**Width**	**Ei cotΘ**	**ΔE**	**2m, 50μm**	**2m, 50μm**
	**(h,k,l)**	**[keV]**	**[°]**	**[μrad]**	**[μrad]**	**[meV/μrad]**	**[meV]**	**[meV]**	**[meV]**
**LiNbO3**	**(3,4,23)**	**13.316**	**81.22**	**1.029**	**2**	**2.081**	**4.07**	**26.01**	**26.32**
	Equiv. Refl.: (3,-7,-23), (4,3,-23), (4,-7,23), (7,-3,-23), (7,-4,23), (-3,7,23), (-3,-4,-23), (-4,7,-23), (-4,-3,23) (-7,3,23), (-7,4,-23)								
**LiNbO3**	**(2,2,27)**	**12.999**	**74.74**	**0.998**	**1.6**	**3.675**	**5.88**	**45.94**	**46.31**
	Equiv. Refl.: (2,2,-27), (2,-4,27), (2,-4,-27), (4,-2,27), (4,-2,-27), (-2,4,27), (-2,4,-27), (-2,-2,27), (-2,-2,-27) (-4,2,27), (-4,2,-27)								
**Quartz**	**(-4,9,6)**	**13.299**	**80.75**	**0.991**	**1.1**	**2.195**	**2.5**	**27.44**	**27.55**
	Equiv. Refl.: (-4,-5,-6)								
**Quartz**	**(9,-4,-6)**	**13.299**	**80.75**	**0.988**	**1.1**	**2.195**	**2.49**	**27.44**	**27.55**
**Sapph**	**(3,5,-17)**	**13.294**	**80.63**	**0.987**	**1.2**	**2.223**	**2.75**	**27.79**	**27.93**
	Equiv. Refl.: (3,-8,17), (5,3,17), (5,-8,-17), (8,-3,17), (8,-5,-17), (-3,8,-17), (-3,-5,17), (-5,8,17), (-5,-3,-17) (-8,3,-17), (-8,5,17), (9,-5,6), (-5,9,-6)								
**Quartz**	**(-5,-4,6)**	**13.299**	**80.75**	**0.986**	**1.1**	**2.195**	**2.49**	**27.44**	**27.55**
**Quartz**	**(9,-8,3)**	**12.915**	**73.44**	**0.983**	**1.1**	**4.006**	**4.51**	**50.07**	**50.27**
	Equiv. Refl.: (-1,9,3), (-1,-8,-3)								
**Quartz**	**(9,-1,-3)**	**12.915**	**73.44**	**0.982**	**1.1**	**4.006**	**4.51**	**50.07**	**50.27**
	Equiv. Refl.: (-8,9,-3), (-8,-1,3)								
**Quartz**	**(-8,9,3)**	**12.915**	**73.44**	**0.962**	**1**	**4.006**	**4.2**	**50.07**	**50.25**
	Equiv. Refl.: (-8,-1,-3)								
**Quartz**	**(9,-1,3)**	**12.915**	**73.44**	**0.961**	**1**	**4.006**	**4.2**	**50.07**	**50.25**
	Equiv. Refl.: (9,-8,-3), (-1,9,-3), (-1,-8,3)								
**Quartz**	**(8,1,3)**	**12.915**	**73.44**	**0.947**	**1**	**4.006**	**4.2**	**50.07**	**50.25**
	Equiv. Refl.: (8,-9,-3)								
**Sapph**	**(1,6,19)**	**13.4**	**83.99**	**0.946**	**1.3**	**1.418**	**1.88**	**17.73**	**17.83**
	Equiv. Refl.: (1,-7,-19), (6,1,-19), (6,-7,19), (7,-1,-19), (7,-6,19), (-1,7,19), (-1,-6,-19), (-6,7,-19), (-6,-1,19) (-7,1,19), (-7,6,-19), (1,8,-3), (1,-9,3), (-9,1,-3), (-9,8,3)								
**Quartz**	**(4,-9,-6)**	**13.299**	**80.75**	**0.913**	**1.1**	**2.195**	**2.5**	**27.44**	**27.55**
**Quartz**	**(4,5,6)**	**13.299**	**80.75**	**0.912**	**1.1**	**2.195**	**2.5**	**27.44**	**27.55**
**Quartz**	**(-9,4,6)**	**13.299**	**80.75**	**0.909**	**1.1**	**2.195**	**2.49**	**27.44**	**27.55**
	Equiv. Refl.: (-9,5,-6)								
**Quartz**	**(5,-9,6)**	**13.299**	**80.75**	**0.908**	**1.1**	**2.195**	**2.49**	**27.44**	**27.55**
**Quartz**	**(5,4,-6)**	**13.299**	**80.75**	**0.907**	**1.1**	**2.195**	**2.49**	**27.44**	**27.55**
**Quartz**	**(0,6,-8)**	**12.674**	**70.15**	**0.899**	**1**	**4.863**	**4.75**	**60.79**	**60.98**
	Equiv. Refl.: (0,-6,8), (6,0,8), (6,-6,-8), (-6,0,-8), (-6,6,8)								
**Quartz**	**(2,1,11)**	**13.193**	**78.28**	**0.898**	**1**	**2.796**	**2.74**	**34.95**	**35.06**
	Equiv. Refl.: (2,-3,-11)								
**Quartz**	**(-3,1,-11)**	**13.193**	**78.28**	**0.896**	**1**	**2.796**	**2.74**	**34.95**	**35.06**
**Quartz**	**(1,2,-11)**	**13.193**	**78.28**	**0.895**	**1**	**2.796**	**2.73**	**34.95**	**35.06**
	Equiv. Refl.: (1,-3,11)								
**Quartz**	**(-3,2,11)**	**13.193**	**78.28**	**0.894**	**1**	**2.796**	**2.73**	**34.95**	**35.06**
**Quartz**	**(10,-5,3)**	**13.079**	**76.09**	**0.873**	**1**	**3.338**	**3.29**	**41.72**	**41.85**
	Equiv. Refl.: (10,-5,-3), (-5,10,3), (-5,10,-3), (-5,-5,3), (-5,-5,-3)								
**Quartz**	**(5,5,3)**	**13.079**	**76.09**	**0.864**	**1**	**3.338**	**3.29**	**41.72**	**41.85**
	Equiv. Refl.: (5,-10,-3)								
**Quartz**	**(5,5,-3)**	**13.079**	**76.09**	**0.863**	**1**	**3.338**	**3.29**	**41.72**	**41.85**
	Equiv. Refl.: (5,-10,3), (-10,5,3), (-10,5,-3)								
**Quartz**	**(1,5,9)**	**13.129**	**77.01**	**0.856**	**0.9**	**3.108**	**2.83**	**38.85**	**38.96**
	Equiv. Refl.: (1,-6,-9)								
**Quartz**	**(5,1,-9)**	**13.129**	**77.01**	**0.855**	**0.9**	**3.108**	**2.82**	**38.85**	**38.96**
**Quartz**	**(5,-6,9)**	**13.129**	**77.01**	**0.854**	**0.9**	**3.108**	**2.82**	**38.85**	**38.96**
**Quartz**	**(-6,1,9)**	**13.129**	**77.01**	**0.852**	**0.9**	**3.108**	**2.82**	**38.85**	**38.96**
	Equiv. Refl.: (-6,5,-9)								

Ei =	13.474	keV						ΔEg	ΔEt
Cryst	Refl (h,k,l)	EB [keV]	ΘB [°]	∫ IR dΘ [µrad]	Width [µrad]	Ei cotΘ [meV/µrad]	ΔE [meV]	2m, 50µm [meV]	2m, 50µm [meV]
**Quartz**	**(10,-3,-2)**	**13.152**	**77.44**	**0.839**	**1**	**3.002**	**2.9**	**37.52**	**37.63**
	Equiv. Refl.: (10,-7,2)								
**Quartz**	**(-3,10,2)**	**13.152**	**77.44**	**0.838**	**1**	**3.002**	**2.89**	**37.52**	**37.63**
	Equiv. Refl.: (-3,-7,-2), (-7,10,-2), (-7,-3,2)								
**Quartz**	**(-10,3,2)**	**13.152**	**77.44**	**0.818**	**1**	**3.002**	**2.9**	**37.52**	**37.63**
	Equiv. Refl.: (-10,7,-2)								
**Sapph**	**(2,7,-8)**	**12.893**	**73.11**	**0.817**	**1**	**4.092**	**3.91**	**51.15**	**51.3**
	Equiv. Refl.: (2,-9,8), (7,2,8), (7,-9,-8), (9,-2,8), (9,-7,-8), (-2,9,-8), (-2,-7,8), (-7,9,8), (-7,-2,-8) (-9,2,-8), (-9,7,8), (0,7,7), (0,-7,-7), (7,3,-2)								
**Quartz**	**(3,7,2)**	**13.152**	**77.44**	**0.816**	**1**	**3.002**	**2.9**	**37.52**	**37.63**
	Equiv. Refl.: (3,-10,-2), (7,-10,2)								
**Quartz**	**(7,0,-7)**	**12.98**	**74.44**	**0.815**	**0.9**	**3.752**	**3.46**	**46.9**	**47.03**
	Equiv. Refl.: (7,-7,7), (-7,0,7), (-7,7,-7)								
**Quartz**	**(0,1,-11)**	**12.701**	**70.5**	**0.804**	**0.9**	**4.772**	**4.23**	**59.65**	**59.8**
	Equiv. Refl.: (0,-1,11)								
**Quartz**	**(1,0,11)**	**12.701**	**70.5**	**0.803**	**0.9**	**4.772**	**4.22**	**59.65**	**59.8**
	Equiv. Refl.: (1,-1,-11)								
**Quartz**	**(-1,0,-11)**	**12.701**	**70.5**	**0.802**	**0.9**	**4.772**	**4.22**	**59.65**	**59.8**
	Equiv. Refl.: (-1,1,11)								
**Quartz**	**(9,-2,-5)**	**13.233**	**79.15**	**0.781**	**0.9**	**2.582**	**2.31**	**32.27**	**32.35**
	Equiv. Refl.: (9,-7,5), (-7,9,-5), (-7,-2,5)								
**Quartz**	**(-2,9,5)**	**13.233**	**79.15**	**0.78**	**0.9**	**2.582**	**2.3**	**32.27**	**32.35**
	Equiv. Refl.: (-2,-7,-5)								
**Quartz**	**(2,5,8)**	**12.923**	**73.55**	**0.774**	**0.9**	**3.978**	**3.43**	**49.73**	**49.85**
**Quartz**	**(2,-7,-8)**	**12.923**	**73.55**	**0.773**	**0.9**	**3.978**	**3.43**	**49.73**	**49.85**
	Equiv. Refl.: (-7,2,8), (-7,5,-8)								
**Quartz**	**(5,2,-8)**	**12.923**	**73.55**	**0.772**	**0.9**	**3.978**	**3.42**	**49.73**	**49.85**
	Equiv. Refl.: (5,-7,8)								
**Quartz**	**(-2,3,11)**	**13.193**	**78.28**	**0.771**	**1**	**2.796**	**2.74**	**34.95**	**35.06**
	Equiv. Refl.: (-2,-1,-11)								
**Quartz**	**(3,-1,11)**	**13.193**	**78.28**	**0.769**	**1**	**2.796**	**2.74**	**34.95**	**35.06**
	Equiv. Refl.: (8,-3,7)								
**Quartz**	**(3,-2,-11)**	**13.193**	**78.28**	**0.768**	**1**	**2.796**	**2.73**	**34.95**	**35.06**
	Equiv. Refl.: (8,-5,-7), (-1,3,-11), (-1,-2,11), (-3,8,-7), (-3,-5,7)								
**LiNbO3**	**(4,5,17)**	**13.256**	**79.69**	**0.766**	**1.5**	**2.452**	**3.77**	**30.64**	**30.87**
	Equiv. Refl.: (4,-9,-17), (5,4,-17), (5,-9,17), (9,-4,-17), (9,-5,17), (-4,9,17), (-4,-5,-17), (-5,9,-17), (-5,-4,17) (-9,4,17), (-9,5,-17), (-5,8,7), (-5,-3,-7)								
**Quartz**	**(3,5,-7)**	**12.98**	**74.44**	**0.75**	**0.9**	**3.752**	**3.26**	**46.9**	**47.01**
	Equiv. Refl.: (3,-8,7), (-8,3,-7), (-8,5,7)								
**Quartz**	**(5,3,7)**	**12.98**	**74.44**	**0.747**	**0.9**	**3.752**	**3.25**	**46.9**	**47.01**
	Equiv. Refl.: (5,-8,-7)								
**Quartz**	**(7,-5,8)**	**12.923**	**73.55**	**0.744**	**0.9**	**3.978**	**3.43**	**49.73**	**49.85**
	Equiv. Refl.: (-2,7,8), (-2,-5,-8)								
**Quartz**	**(7,-2,-8)**	**12.923**	**73.55**	**0.743**	**0.9**	**3.978**	**3.43**	**49.73**	**49.85**
**Quartz**	**(-5,7,-8)**	**12.923**	**73.55**	**0.742**	**0.9**	**3.978**	**3.42**	**49.73**	**49.85**
	Equiv. Refl.: (-5,-2,8)								
**Quartz**	**(10,-4,0)**	**12.702**	**70.51**	**0.733**	**0.8**	**4.77**	**3.9**	**59.62**	**59.75**
	Equiv. Refl.: (10,-6,0), (-4,10,0), (-4,-6,0), (-6,10,0), (-6,-4,0)								
**Quartz**	**(4,6,0)**	**12.702**	**70.51**	**0.73**	**0.8**	**4.77**	**3.9**	**59.62**	**59.75**
	Equiv. Refl.: (4,-10,0), (6,4,0), (6,-10,0), (-10,4,0), (-10,6,0)								

Ei =	13.474	keV						ΔEg	ΔEt
Cryst	Refl	EB	ΘB	∫ IR dΘ	Width	Ei cotΘ	ΔE	2m, 50µm	2m, 50µm
	(h,k,l)	[keV]	[°]	[µrad]	[µrad]	[meV/µrad]	[meV]	[meV]	[meV]
**Quartz**	**(10,-3,-1)**	**13.001**	**74.77**	**0.713**	**0.8**	**3.669**	**3**	**45.86**	**45.96**
	Equiv. Refl.: (10,-7,1), (-3,10,1), (-3,-7,-1), (-7,10,-1), (-7,-3,1)								
**Quartz**	**(-1,6,9)**	**13.129**	**77.01**	**0.706**	**0.9**	**3.108**	**2.83**	**38.85**	**38.96**
**Quartz**	**(-1,-5,-9)**	**13.129**	**77.01**	**0.705**	**0.9**	**3.108**	**2.83**	**38.85**	**38.96**
**Quartz**	**(-5,6,-9)**	**13.129**	**77.01**	**0.704**	**0.9**	**3.108**	**2.82**	**38.85**	**38.96**
	Equiv. Refl.: (-5,-1,9)								
**Quartz**	**(6,-1,-9)**	**13.129**	**77.01**	**0.702**	**0.9**	**3.108**	**2.82**	**38.85**	**38.96**
	Equiv. Refl.: (6,-5,9)								
**Quartz**	**(3,7,1)**	**13.001**	**74.77**	**0.692**	**0.8**	**3.669**	**3**	**45.86**	**45.96**
	Equiv. Refl.: (3,-10,-1), (7,3,-1), (7,-10,1), (-10,3,1), (-10,7,-1)								
**Quartz**	**(0,4,10)**	**12.866**	**72.72**	**0.69**	**0.8**	**4.192**	**3.31**	**52.39**	**52.5**
	Equiv. Refl.: (0,-4,-10)								
**Quartz**	**(4,0,-10)**	**12.866**	**72.72**	**0.687**	**0.8**	**4.192**	**3.3**	**52.39**	**52.5**
	Equiv. Refl.: (4,-4,10), (-4,0,10), (-4,4,-10)								
**Quartz**	**(7,2,-5)**	**13.233**	**79.15**	**0.683**	**0.9**	**2.582**	**2.31**	**32.27**	**32.35**
	Equiv. Refl.: (7,-9,5), (-9,7,-5)								
**Quartz**	**(2,7,5)**	**13.233**	**79.15**	**0.682**	**0.9**	**2.582**	**2.31**	**32.27**	**32.35**
	Equiv. Refl.: (-9,2,5)								
**Quartz**	**(2,-9,-5)**	**13.233**	**79.15**	**0.681**	**0.9**	**2.582**	**2.3**	**32.27**	**32.35**
**Quartz**	**(-10,6,-3)**	**13.16**	**77.6**	**0.671**	**0.8**	**2.963**	**2.26**	**37.04**	**37.11**
**Quartz**	**(4,6,3)**	**13.16**	**77.6**	**0.67**	**0.8**	**2.963**	**2.26**	**37.04**	**37.11**
	Equiv. Refl.: (4,-10,-3), (6,4,-3), (6,-10,3), (-10,4,3)								
**Sapph**	**(2,7,7)**	**12.759**	**71.26**	**0.668**	**0.8**	**4.572**	**3.64**	**57.15**	**57.27**
	Equiv. Refl.: (2,-9,-7), (7,2,-7), (7,-9,7), (9,-2,-7), (9,-7,7), (-2,9,7), (-2,-7,-7), (-7,9,-7), (-7,-2,7) (-9,2,7), (-9,7,-7)								
**Quartz**	**(1,4,10)**	**13.272**	**80.06**	**0.657**	**0.8**	**2.36**	**1.95**	**29.5**	**29.57**
**Quartz**	**(1,-5,-10)**	**13.272**	**80.06**	**0.656**	**0.8**	**2.36**	**1.95**	**29.5**	**29.57**
	Equiv. Refl.: (4,1,-10), (4,-5,10)								
**Quartz**	**(-5,4,-10)**	**13.272**	**80.06**	**0.651**	**0.8**	**2.36**	**1.94**	**29.5**	**29.57**
**Quartz**	**(-5,1,10)**	**13.272**	**80.06**	**0.65**	**0.8**	**2.36**	**1.94**	**29.5**	**29.57**
**LiNbO3**	**(3,7,11)**	**13.302**	**80.83**	**0.639**	**1.4**	**2.175**	**3.13**	**27.18**	**27.37**
	Equiv. Refl.: (3,-10,-11), (7,3,-11), (7,-10,11), (10,-3,-11), (10,-7,11), (-3,10,11), (-3,-7,-11), (-7,10,-11), (-7,-3,11) (-10,3,11), (-10,7,-11)								
**Quartz**	**(-1,5,10)**	**13.272**	**80.06**	**0.637**	**0.8**	**2.36**	**1.95**	**29.5**	**29.57**
	Equiv. Refl.: (-1,-4,-10)								
**Quartz**	**(-4,5,-10)**	**13.272**	**80.06**	**0.636**	**0.8**	**2.36**	**1.95**	**29.5**	**29.57**
	Equiv. Refl.: (-4,-1,10)								
**Quartz**	**(5,-1,-10)**	**13.272**	**80.06**	**0.631**	**0.8**	**2.36**	**1.94**	**29.5**	**29.57**
	Equiv. Refl.: (5,-4,10)								
**Quartz**	**(10,-5,1)**	**12.67**	**70.11**	**0.628**	**0.7**	**4.876**	**3.49**	**60.95**	**61.05**
	Equiv. Refl.: (10,-5,-1), (-5,10,1), (-5,10,-1), (-5,-5,1), (-5,-5,-1)								
**Quartz**	**(5,5,1)**	**12.67**	**70.11**	**0.626**	**0.7**	**4.876**	**3.49**	**60.95**	**61.05**
	Equiv. Refl.: (5,-10,1), (5,-10,-1), (-10,5,-1)								
**Quartz**	**(5,5,-1)**	**12.67**	**70.11**	**0.625**	**0.7**	**4.876**	**3.49**	**60.95**	**61.05**
	Equiv. Refl.: (-10,5,1)								
**Quartz**	**(-2,9,-4)**	**12.778**	**71.5**	**0.596**	**0.7**	**4.507**	**3**	**56.34**	**56.42**
	Equiv. Refl.: (-2,-7,4)								
**Quartz**	**(9,-2,4)**	**12.778**	**71.5**	**0.595**	**0.7**	**4.507**	**3**	**56.34**	**56.42**
	Equiv. Refl.: (9,-7,-4), (-7,9,4), (-7,-2,-4)								

Ei =	13.474	keV						ΔEg	ΔEt
Cryst	Refl	EB	ΘB	∫ IR dΘ	Width	Ei cotΘ	ΔE	2m, 50µm	2m, 50µm
	(h,k,l)	[keV]	[°]	[µrad]	[µrad]	[meV/µrad]	[meV]	[meV]	[meV]
Quartz	(10,-2,-1)	13.403	84.1	0.581	0.8	1.392	1.13	17.4	17.44
	Equiv. Refl.: (10,-8,1), (-8,10,-1), (-8,-2,1)								
Quartz	(-2,10,1)	13.403	84.1	0.58	0.8	1.392	1.13	17.4	17.44
	Equiv. Refl.: (-2,-8,-1)								
Quartz	(10,-4,-3)	13.16	77.6	0.569	0.8	2.963	2.26	37.04	37.11
	Equiv. Refl.: (10,-6,3), (-4,10,3), (-4,-6,-3)								
Quartz	(-6,10,-3)	13.16	77.6	0.568	0.8	2.963	2.26	37.04	37.11
	Equiv. Refl.: (-6,-4,3)								
Sapph	(0,5,22)	12.917	73.46	0.565	0.7	4.001	2.85	50.01	50.09
	Equiv. Refl.: (0,-5,-22), (5,0,-22), (5,-5,22), (-5,0,22), (-5,5,-22)								
LiNbO3	(2,8,9)	13.364	82.69	0.543	1.4	1.729	2.49	21.61	21.76
	Equiv. Refl.: (2,8,-9), (2,-10,9), (2,-10,-9), (8,2,9), (8,2,-9), (8,-10,9), (8,-10,-9), (10,-2,9), (10,-2,-9) (10,-8,9), (10,-8,-9), (-2,10,9), (-2,10,-9), (-2,-8,9), (-2,-8,-9), (-8,10,9), (-8,10,-9), (-8,-2,9) (-8,-2,-9), (-10,2,9), (-10,2,-9), (-10,8,9), (-10,8,-9)								
Quartz	(2,7,-4)	12.778	71.5	0.541	0.7	4.507	3	56.34	56.42
	Equiv. Refl.: (2,-9,4), (7,2,4), (7,-9,-4)								
Quartz	(-9,2,-4)	12.778	71.5	0.54	0.7	4.507	3	56.34	56.42
	Equiv. Refl.: (-9,7,4)								
Sapph	(2,3,23)	12.787	71.63	0.536	0.7	4.475	3	55.94	56.02
	Equiv. Refl.: (2,-5,-23), (3,2,-23), (3,-5,23), (5,-2,-23), (5,-3,23), (-2,5,23), (-2,-3,-23), (-3,5,-23), (-3,-2,23) (-5,2,23), (-5,3,-23)								
Sapph	(5,5,3)	13.107	76.6	0.526	0.7	3.21	2.26	40.13	40.19
	Equiv. Refl.: (5,5,-3), (5,-10,3), (5,-10,-3), (10,-5,3), (10,-5,-3), (-5,10,3), (-5,10,-3), (-5,-5,3), (-5,-5,-3) (-10,5,3), (-10,5,-3)								
LiNbO3	(5,6,-1)	13.272	80.06	0.524	1.2	2.36	2.93	29.5	29.65
	Equiv. Refl.: (5,-11,1), (6,5,1), (6,-11,-1), (11,-5,1), (11,-6,-1), (-5,11,-1), (-5,-6,1), (-6,11,1), (-6,-5,-1) (-11,5,-1), (-11,6,1)								
Quartz	(10,-3,1)	13.001	74.77	0.508	0.6	3.669	2.24	45.86	45.91
	Equiv. Refl.: (10,-7,-1), (-3,10,-1), (-3,-7,1), (-7,10,1), (-7,-3,-1)								
Quartz	(3,7,-1)	13.001	74.77	0.471	0.6	3.669	2.23	45.86	45.91
	Equiv. Refl.: (3,-10,1), (7,3,1), (7,-10,-1), (8,2,-1), (8,-10,1), (-10,2,1), (-10,3,-1), (-10,7,1), (-10,8,-1)								
Quartz	(2,8,1)	13.403	84.1	0.47	0.8	1.392	1.13	17.4	17.44
	Equiv. Refl.: (2,-10,-1)								
LiNbO3	(1,5,23)	12.873	72.83	0.405	0.9	4.164	3.61	52.05	52.18
	Equiv. Refl.: (1,-6,-23), (5,1,-23), (5,-6,23), (6,-1,-23), (6,-5,23), (-1,6,23), (-1,-5,-23), (-5,6,-23), (-5,-1,23) (-6,1,23), (-6,5,-23)								
Quartz	(0,2,-11)	12.949	73.96	0.379	0.5	3.874	1.92	48.43	48.47
	Equiv. Refl.: (0,-2,11)								
Quartz	(2,0,11)	12.949	73.96	0.375	0.5	3.874	1.9	48.43	48.47
	Equiv. Refl.: (2,-2,-11), (-2,0,-11), (-2,2,11)								
Sapph	(1,6,-17)	12.773	71.44	0.362	0.5	4.524	2.24	56.55	56.59
	Equiv. Refl.: (1,-7,17), (6,1,17), (6,-7,-17), (7,-1,17), (7,-6,-17), (-1,7,-17), (-1,-6,17), (-6,7,17), (-6,-1,-17) (-7,1,-17), (-7,6,17)								
LiNbO3	(3,7,-7)	12.749	71.12	0.355	0.8	4.608	3.57	57.59	57.71
	Equiv. Refl.: (3,-10,7), (7,3,7), (7,-10,-7), (10,-3,7), (10,-7,-7), (-3,10,-7), (-3,-7,7), (-7,10,7), (-7,-3,-7) (-10,3,-7), (-10,7,7)								
Sapph	(2,5,18)	12.731	70.88	0.322	0.4	4.67	2.1	58.38	58.41
	Equiv. Refl.: (2,5,-18), (2,-7,18), (2,-7,-18), (5,2,18), (5,2,-18), (5,-7,18), (5,-7,-18), (7,-2,18), (7,-2,-18) (7,-5,18), (7,-5,-18), (-2,7,18), (-2,7,-18), (-2,-5,18), (-2,-5,-18), (-5,7,18), (-5,7,-18), (-5,-2,18) (-5,-2,-18), (-7,2,18), (-7,2,-18), (-7,5,18), (-7,5,-18)								
Sapph	(1,3,25)	13.107	76.6	0.317	0.5	3.209	1.57	40.12	40.15
	Equiv. Refl.: (1,-4,-25), (3,1,-25), (3,-4,25), (4,-1,-25), (4,-3,25), (-1,4,25), (-1,-3,-25), (-3,4,-25), (-3,-1,25)								

	(-4,1,25), (-4,3,-25)								
**Quartz**	**(-2,5,-10)**	**13.111**	**76.67**	**0.315**	**0.4**	**3.192**	**1.39**	**39.91**	**39.93**
	Equiv. Refl.: (-2,-3,10), (-3,5,10), (-3,-2,-10)								
**Quartz**	**(5,-3,-10)**	**13.111**	**76.67**	**0.312**	**0.4**	**3.192**	**1.38**	**39.91**	**39.93**
**Quartz**	**(5,-2,10)**	**13.111**	**76.67**	**0.311**	**0.4**	**3.192**	**1.38**	**39.91**	**39.93**
**Quartz**	**(0,7,-7)**	**12.98**	**74.44**	**0.291**	**0.4**	**3.752**	**1.53**	**46.9**	**46.92**
	Equiv. Refl.: (0,-7,7), (2,3,-10), (2,-5,10), (3,2,10), (3,-5,-10), (7,0,7), (7,-7,-7), (-7,0,-7), (-7,7,7)								
**Quartz**	**(-5,3,10)**	**13.111**	**76.67**	**0.288**	**0.4**	**3.192**	**1.38**	**39.91**	**39.93**
**LiNbO3**	**(3,6,15)**	**12.915**	**73.44**	**0.287**	**0.7**	**4.007**	**2.88**	**50.08**	**50.16**
	Equiv. Refl.: (3,6,-15), (3,-9,15), (3,-9,-15), (6,3,15), (6,3,-15), (6,-9,15), (6,-9,-15), (9,-3,15), (9,-3,-15) (9,-6,15), (9,-6,-15), (-3,9,15), (-3,9,-15), (-3,-6,15), (-3,-6,-15), (-6,9,15), (-6,9,-15), (-6,-3,15) (-6,-3,-15), (-9,3,15), (-9,3,-15), (-9,6,15), (-9,6,-15), (-5,2,-10)								
**LiNbO3**	**(2,6,-19)**	**13.143**	**77.27**	**0.282**	**0.8**	**3.045**	**2.41**	**38.06**	**38.14**
	Equiv. Refl.: (2,-8,19), (6,2,19), (6,-8,-19), (8,-2,19), (8,-6,-19), (-2,8,-19), (-2,-6,19), (-6,8,19), (-6,-2,-19) (-8,2,-19), (-8,6,19)								
**LiNbO3**	**(2,8,3)**	**12.814**	**72**	**0.253**	**0.6**	**4.378**	**2.84**	**54.73**	**54.8**
	Equiv. Refl.: (2,8,-3), (2,-10,3), (2,-10,-3), (8,2,3), (8,2,-3), (8,-10,3), (8,-10,-3), (10,-2,3), (10,-2,-3) (10,-8,3), (10,-8,-3), (-2,10,3), (-2,10,-3), (-2,-8,3), (-2,-8,-3), (-8,10,3), (-8,10,-3), (-8,-2,3) (-8,-2,-3), (-10,2,3), (-10,2,-3), (-10,8,3), (-10,8,-3)								
**LiNbO3**	**(2,5,21)**	**12.79**	**71.67**	**0.226**	**0.6**	**4.464**	**2.7**	**55.8**	**55.87**
	Equiv. Refl.: (2,5,-21), (2,-7,21), (2,-7,-21), (5,2,21), (5,2,-21), (5,-7,21), (5,-7,-21), (7,-2,21), (7,-2,-21) (7,-5,21), (7,-5,-21), (-2,7,21), (-2,7,-21), (-2,-5,21), (-2,-5,-21), (-5,7,21), (-5,7,-21), (-5,-2,21) (-5,-2,-21), (-7,2,21), (-7,2,-21), (-7,5,21), (-7,5,-21)								
**Sapph**	**(4,6,-5)**	**13.331**	**81.63**	**0.139**	**0.3**	**1.982**	**0.68**	**24.77**	**24.78**
	Equiv. Refl.: (4,-10,5), (6,4,5), (6,-10,-5), (10,-4,5), (10,-6,-5), (-4,10,-5), (-4,-6,5), (-6,10,5), (-6,-4,-5) (-10,4,-5), (-10,6,5)								
**LiNbO3**	**(1,8,-13)**	**13.226**	**79**	**0.137**	**0.6**	**2.62**	**1.52**	**32.74**	**32.78**
	Equiv. Refl.: (1,-9,13), (8,1,13), (8,-9,-13), (9,-1,13), (9,-8,-13), (-1,9,-13), (-1,-8,13), (-8,9,13), (-8,-1,-13) (-9,1,-13), (-9,8,13)								
**LiNbO3**	**(2,3,-25)**	**12.717**	**70.7**	**0.132**	**0.4**	**4.719**	**2.09**	**58.98**	**59.02**
	Equiv. Refl.: (2,-5,25), (3,2,25), (3,-5,-25), (5,-2,25), (5,-3,-25), (-2,5,-25), (-2,-3,25), (-3,5,25), (-3,-2,-25) (-5,2,-25), (-5,3,25)								
**LiNbO3**	**(3,5,19)**	**12.92**	**73.51**	**0.126**	**0.5**	**3.988**	**1.84**	**49.85**	**49.88**
	Equiv. Refl.: (3,-8,-19), (5,3,-19), (5,-8,19), (8,-3,-19), (8,-5,19), (-3,8,19), (-3,-5,-19), (-5,8,-19), (-5,-3,19) (-8,3,19), (-8,5,-19)								
**Sapph**	**(3,6,9)**	**12.69**	**70.36**	**0.122**	**0.2**	**4.809**	**1.12**	**60.11**	**60.12**
	Equiv. Refl.: (3,6,-9), (3,-9,9), (3,-9,-9), (6,3,9), (6,3,-9), (6,-9,9), (6,-9,-9), (9,-3,9), (9,-3,-9) (9,-6,9), (9,-6,-9), (-3,9,9), (-3,9,-9), (-3,-6,9), (-3,-6,-9), (-6,9,9), (-6,9,-9), (-6,-3,9) (-6,-3,-9), (-9,3,9), (-9,3,-9), (-9,6,9), (-9,6,-9)								
**Sapph**	**(1,8,2)**	**12.889**	**73.06**	**0.121**	**0.2**	**4.105**	**0.99**	**51.31**	**51.32**
	Equiv. Refl.: (1,-9,-2), (8,1,-2), (8,-9,2), (9,-1,-2), (9,-8,2), (-1,9,2), (-1,-8,-2), (-8,9,-2), (-8,-1,2) (-9,1,2), (-9,8,-2)								
**Sapph**	**(3,4,-19)**	**12.883**	**72.97**	**0.1**	**0.2**	**4.127**	**0.89**	**51.58**	**51.59**
	Equiv. Refl.: (3,-7,19), (4,3,19), (4,-7,-19), (7,-3,19), (7,-4,-19), (-3,7,-19), (-3,-4,19), (-4,7,19), (-4,-3,-19) (-7,3,-19), (-7,4,19)								
**LiNbO3**	**(1,8,11)**	**12.858**	**72.61**	**0.096**	**0.4**	**4.219**	**1.66**	**52.74**	**52.76**
	Equiv. Refl.: (1,-9,-11), (8,1,-11), (8,-9,11), (9,-1,-11), (9,-8,11), (-1,9,11), (-1,-8,-11), (-8,9,-11), (-8,-1,11) (-9,1,11), (-9,8,-11)								
**Sapph**	**(4,6,1)**	**13.124**	**76.91**	**0.094**	**0.2**	**3.133**	**0.72**	**39.16**	**39.17**
	Equiv. Refl.: (4,-10,-1), (6,4,-1), (6,-10,1), (10,-4,-1), (10,-6,1), (-4,10,1), (-4,-6,-1), (-6,10,-1), (-6,-4,1) (-10,4,1), (-10,6,-1)								
**LiNbO3**	**(4,4,21)**	**13.453**	**86.82**	**0.046**	**0.7**	**0.748**	**0.51**	**9.35**	**9.36**
	Equiv. Refl.: (4,4,-21), (4,-8,21), (4,-8,-21), (8,-4,21), (8,-4,-21), (-4,8,21), (-4,8,-21), (-4,-4,21), (-4,-4,-21) (-8,4,21), (-8,4,-21)								
**Sapph**	**(1,7,12)**	**12.721**	**70.75**	**0.045**	**0.1**	**4.706**	**0.61**	**58.82**	**58.83**
	Equiv. Refl.: (1,7,-12), (1,-8,12), (1,-8,-12), (7,1,12), (7,1,-12), (7,-8,12), (7,-8,-12), (8,-1,12), (8,-1,-12)								

	(8,-7,12), (8,-7,-12), (-1,8,12), (-1,8,-12), (-1,-7,12), (-1,-7,-12), (-7,8,12), (-7,8,-12), (-7,-1,12) (-7,-1,-12), (-8,1,12), (-8,1,-12), (-8,7,12), (-8,7,-12)								
**LiNbO3**	**(5,5,9)**	**12.697**	**70.44**	**0.006**	**0.1**	**4.786**	**0.5**	**59.83**	**59.83**
	Equiv. Refl.: (5,5,-9), (5,-10,9), (5,-10,-9), (10,-5,9), (10,-5,-9), (-5,10,9), (-5,10,-9), (-5,-5,9), (-5,-5,-9) (-10,5,9), (-10,5,-9)								

Ei =	22.117	keV						ΔEg	ΔEt
Cryst	Refl	EB	ΘB	∫ IR dΘ	Width	Ei cotΘ	ΔE	2m, 50µm	2m, 50µm
	(h,k,l)	[keV]	[°]	[µrad]	[µrad]	[meV/µrad]	[meV]	[meV]	[meV]
**Si**	**(1,3,19)**	**21.986**	**83.76**	**0.2513**	**0.31**	**2.42**	**0.75**	**30.25**	**30.26**
	Equiv. Refl.: (1,9,17), (5,11,15), (9,11,13)								
**Ge**	**(0,0,20)**	**21.916**	**82.26**	**0.2247**	**0.87**	**3.0053**	**2.6**	**37.57**	**37.66**
	Equiv. Refl.: (0,12,16)								
**Si**	**(0,6,18)**	**21.657**	**78.3**	**0.2232**	**0.25**	**4.5806**	**1.16**	**57.26**	**57.27**
	Equiv. Refl.: (2,10,16), (8,10,14)								
**Si**	**(8,12,12)**	**21.415**	**75.53**	**0.1921**	**0.22**	**5.7076**	**1.24**	**71.34**	**71.36**
**Si**	**(2,4,18)**	**21.171**	**73.18**	**0.176**	**0.2**	**6.6865**	**1.32**	**83.58**	**83.59**
	Equiv. Refl.: (2,12,14), (10,10,12)								
**Ge**	**(0,14,14)**	**21.695**	**78.79**	**0.1713**	**0.63**	**4.3817**	**2.77**	**54.77**	**54.84**
	Equiv. Refl.: (2,8,18), (6,10,16)								
**Si**	**(4,8,16)**	**20.923**	**71.09**	**0.1668**	**0.19**	**7.5777**	**1.41**	**94.72**	**94.73**
**Si**	**(1,1,19)**	**21.747**	**79.51**	**0.1596**	**0.2**	**4.0946**	**0.8**	**51.18**	**51.19**
	Equiv. Refl.: (5,7,17), (5,13,13), (11,11,11)								
**Ge**	**(8,8,16)**	**21.473**	**76.14**	**0.1529**	**0.54**	**5.458**	**2.96**	**68.23**	**68.29**
**Ge**	**(4,6,18)**	**21.248**	**73.89**	**0.1451**	**0.49**	**6.39**	**3.16**	**79.87**	**79.94**
	Equiv. Refl.: (6,12,14)								
**Ge**	**(0,6,18)**	**20.791**	**70.06**	**0.1426**	**0.45**	**8.0242**	**3.6**	**100.3**	**100.37**
	Equiv. Refl.: (2,10,16), (8,10,14)								
**Ge**	**(3,13,15)**	**21.998**	**84.04**	**0.1385**	**0.78**	**2.3073**	**1.79**	**28.84**	**28.9**
**Si**	**(3,11,15)**	**21.506**	**76.51**	**0.1324**	**0.16**	**5.3073**	**0.85**	**66.34**	**66.35**
	Equiv. Refl.: (7,9,15)								
**Si**	**(1,11,15)**	**21.263**	**74.02**	**0.1194**	**0.14**	**6.332**	**0.91**	**79.15**	**79.16**
	Equiv. Refl.: (3,7,17), (3,13,13)								
**Si**	**(1,7,17)**	**21.016**	**71.85**	**0.1123**	**0.13**	**7.2515**	**0.97**	**90.64**	**90.65**
	Equiv. Refl.: (1,13,13), (5,5,17), (7,11,13)								
**Ge**	**(1,13,15)**	**21.778**	**79.96**	**0.0912**	**0.49**	**3.9164**	**1.91**	**48.96**	**48.99**
	Equiv. Refl.: (3,5,19), (5,9,17), (7,11,15)								
**Ge**	**(1,5,19)**	**21.557**	**77.07**	**0.0787**	**0.4**	**5.0762**	**2.04**	**63.45**	**63.48**
	Equiv. Refl.: (7,7,17), (7,13,13), (9,9,15)								
**Ge**	**(3,3,19)**	**21.333**	**74.69**	**0.0737**	**0.36**	**6.0528**	**2.18**	**75.66**	**75.69**
	Equiv. Refl.: (3,9,17)								
**Ge**	**(1,1,19)**	**20.877**	**70.73**	**0.0721**	**0.32**	**7.7338**	**2.49**	**96.67**	**96.7**
	Equiv. Refl.: (5,7,17), (5,13,13), (11,11,11)								
**Ge**	**(1,3,19)**	**21.106**	**72.61**	**0.072**	**0.34**	**6.9264**	**2.33**	**86.58**	**86.61**
	Equiv. Refl.: (1,9,17), (5,11,15), (9,11,13)								

Ei =	19.279	keV						ΔEg	ΔEt
Cryst	Refl	EB	ΘB	∫ IR dΘ	Width	Ei cotΘ	ΔE	2m, 50µm	2m, 50µm
	(h,k,l)	[keV]	[°]	[µrad]	[µrad]	[meV/µrad]	[meV]	[meV]	[meV]
**Si**	**(6,10,12)**	**19.1**	**82.19**	**0.6857**	**0.76**	**2.6454**	**2.01**	**33.07**	**33.13**
	Equiv. Refl.: (6,12,10), (10,6,12), (10,12,6), (12,6,10), (12,10,6)								
**Si**	**(3,7,15)**	**19.202**	**84.88**	**0.6751**	**0.8**	**1.7275**	**1.38**	**21.59**	**21.64**
	Equiv. Refl.: (3,15,7), (7,3,15), (7,15,3), (9,9,11), (9,11,9), (11,9,9), (15,3,7), (15,7,3)								
**Ge**	**(4,12,12)**	**19.106**	**82.31**	**0.6619**	**1.94**	**2.6035**	**5.04**	**32.54**	**32.93**
	Equiv. Refl.: (12,4,12), (12,12,4)								
**Ge**	**(1,9,15)**	**19.2**	**84.8**	**0.4816**	**1.98**	**1.755**	**3.47**	**21.94**	**22.21**
	Equiv. Refl.: (1,15,9), (3,3,17), (3,17,3), (9,1,15), (9,15,1), (15,1,9), (15,9,1), (17,3,3)								
**Ge**	**(0,10,14)**	**18.853**	**77.93**	**0.4659**	**1.31**	**4.1239**	**5.42**	**51.55**	**51.83**
	Equiv. Refl.: (0,14,10), (2,6,16), (2,16,6), (6,2,16), (6,8,14), (6,14,8), (6,16,2), (8,6,14), (8,14,6) (10,0,14), (10,14,0), (14,0,10), (14,6,8), (14,8,6), (14,10,0), (16,2,6), (16,6,2)								
**Si**	**(0,4,16)**	**18.825**	**77.54**	**0.462**	**0.51**	**4.2586**	**2.17**	**53.23**	**53.28**
	Equiv. Refl.: (0,16,4), (4,0,16), (4,16,0), (8,8,12), (8,12,8), (12,8,8), (16,0,4), (16,4,0)								
**Ge**	**(0,12,12)**	**18.596**	**74.7**	**0.4064**	**1.1**	**5.2729**	**5.82**	**65.91**	**66.17**
	Equiv. Refl.: (4,4,16), (4,16,4), (12,0,12), (12,12,0), (16,4,4)								
**Si**	**(2,2,16)**	**18.546**	**74.15**	**0.3906**	**0.43**	**5.4725**	**2.34**	**68.41**	**68.45**
	Equiv. Refl.: (2,8,14), (2,14,8), (2,16,2), (8,2,14), (8,10,10), (8,14,2), (10,8,10), (10,10,8), (14,2,8) (14,8,2), (16,2,2)								
**Ge**	**(6,10,12)**	**18.336**	**72**	**0.3817**	**1**	**6.2624**	**6.27**	**78.28**	**78.53**
	Equiv. Refl.: (6,12,10), (10,6,12), (10,12,6), (12,6,10), (12,10,6)								
**Si**	**(0,0,16)**	**18.263**	**71.32**	**0.3568**	**0.39**	**6.5192**	**2.53**	**81.49**	**81.53**
	Equiv. Refl.: (0,16,0), (16,0,0)								
**Si**	**(1,7,15)**	**18.929**	**79.06**	**0.3402**	**0.4**	**3.726**	**1.49**	**46.58**	**46.6**
	Equiv. Refl.: (1,15,7), (5,5,15), (5,9,13), (5,13,9), (5,15,5), (7,1,15), (7,15,1), (9,5,13), (9,13,5) (13,5,9), (13,9,5), (15,1,7), (15,5,5), (15,7,1)								
**Si**	**(5,11,11)**	**18.651**	**75.34**	**0.2736**	**0.32**	**5.0434**	**1.61**	**63.04**	**63.06**
	Equiv. Refl.: (7,7,13), (7,13,7), (11,5,11), (11,11,5), (13,7,7)								
**Ge**	**(1,3,17)**	**18.948**	**79.36**	**0.262**	**1.03**	**3.6204**	**3.73**	**45.25**	**45.41**
	Equiv. Refl.: (1,17,3), (3,1,17), (3,11,13), (3,13,11), (3,17,1), (5,7,15), (5,15,7), (7,5,15), (7,9,13) (7,13,9), (7,15,5), (9,7,13), (9,13,7), (11,3,13), (11,13,3), (13,3,11), (13,7,9), (13,9,7) (13,11,3), (15,5,7), (15,7,5), (17,1,3), (17,3,1)								
**Si**	**(3,5,15)**	**18.37**	**72.33**	**0.245**	**0.28**	**6.1402**	**1.73**	**76.75**	**76.77**
	Equiv. Refl.: (3,9,13), (3,13,9), (3,15,5), (5,3,15), (5,15,3), (9,3,13), (9,13,3), (13,3,9), (13,9,3) (15,3,5), (15,5,3)								
**Ge**	**(1,1,17)**	**18.693**	**75.83**	**0.2188**	**0.82**	**4.8668**	**4.01**	**60.83**	**60.97**
	Equiv. Refl.: (1,11,13), (1,13,11), (1,17,1), (7,11,11), (11,1,13), (11,7,11), (11,11,7), (11,13,1), (13,1,11) (13,11,1), (17,1,1)								
**Ge**	**(3,7,15)**	**18.434**	**72.97**	**0.2025**	**0.73**	**5.9044**	**4.31**	**73.8**	**73.93**
	Equiv. Refl.: (3,15,7), (7,3,15), (7,15,3), (9,9,11), (9,11,9), (11,9,9), (15,3,7), (15,7,3)								
**Ge**	**(1,7,15)**	**18.171**	**70.48**	**0.1965**	**0.68**	**6.8329**	**4.65**	**85.41**	**85.54**
	Equiv. Refl.: (1,15,7), (5,5,15), (5,9,13), (5,13,9), (5,15,5), (7,1,15), (7,15,1), (9,5,13), (9,13,5) (13,5,9), (13,9,5), (15,1,7), (15,5,5), (15,7,1)								

Ei =	19.15	keV						ΔEg	ΔEt
Cryst	Refl	EB	ΘB	∫ IR dΘ	Width	Ei cotΘ	ΔE	2m, 50µm	2m, 50µm
	(h,k,l)	[keV]	[°]	[µrad]	[µrad]	[meV/µrad]	[meV]	[meV]	[meV]
**Si**	**(6,10,12)**	**19.1**	**85.86**	**1.2977**	**1.44**	**1.3863**	**2**	**17.33**	**17.44**
	Equiv. Refl.: (6,12,10), (10,6,12), (10,12,6), (12,6,10), (12,10,6)								
**Ge**	**(4,12,12)**	**19.106**	**86.1**	**1.2974**	**3.83**	**1.3067**	**5**	**16.33**	**17.08**
	Equiv. Refl.: (12,4,12), (12,12,4)								
**Ge**	**(0,10,14)**	**18.853**	**79.89**	**0.5536**	**1.57**	**3.4154**	**5.38**	**42.69**	**43.03**
	Equiv. Refl.: (0,14,10), (2,6,16), (2,16,6), (6,2,16), (6,8,14), (6,14,8), (6,16,2), (8,6,14), (8,14,6) (10,0,14), (10,14,0), (14,0,10), (14,6,8), (14,8,6), (14,10,0), (16,2,6), (16,6,2)								
**Si**	**(0,4,16)**	**18.825**	**79.43**	**0.5463**	**0.6**	**3.5727**	**2.15**	**44.66**	**44.71**
	Equiv. Refl.: (0,16,4), (4,0,16), (4,16,0), (8,8,12), (8,12,8), (12,8,8), (16,0,4), (16,4,0)								
**Ge**	**(0,12,12)**	**18.596**	**76.18**	**0.4478**	**1.23**	**4.7091**	**5.78**	**58.86**	**59.15**
	Equiv. Refl.: (4,4,16), (4,16,4), (12,0,12), (12,12,0), (16,4,4)								
**Si**	**(2,2,16)**	**18.546**	**75.57**	**0.4305**	**0.47**	**4.9258**	**2.32**	**61.57**	**61.62**
	Equiv. Refl.: (2,8,14), (2,14,8), (2,16,2), (8,2,14), (8,10,10), (8,14,2), (10,8,10), (10,10,8), (14,2,8) (14,8,2), (16,2,2)								
**Si**	**(1,7,15)**	**18.929**	**81.28**	**0.4278**	**0.5**	**2.9367**	**1.48**	**36.71**	**36.74**
	Equiv. Refl.: (1,15,7), (5,5,15), (5,9,13), (5,13,9), (5,15,5), (7,1,15), (7,15,1), (9,5,13), (9,13,5) (13,5,9), (13,9,5), (15,1,7), (15,5,5), (15,7,1)								
**Ge**	**(6,10,12)**	**18.336**	**73.23**	**0.4078**	**1.08**	**5.7695**	**6.22**	**72.12**	**72.39**
	Equiv. Refl.: (6,12,10), (10,6,12), (10,12,6), (12,6,10), (12,10,6)								
**Ge**	**(0,4,16)**	**18.072**	**70.68**	**0.3912**	**1**	**6.7121**	**6.71**	**83.9**	**84.17**
	Equiv. Refl.: (0,16,4), (4,0,16), (4,16,0), (8,8,12), (8,12,8), (12,8,8), (16,0,4), (16,4,0)								
**Si**	**(0,0,16)**	**18.263**	**72.49**	**0.382**	**0.42**	**6.04**	**2.51**	**75.5**	**75.54**
	Equiv. Refl.: (0,16,0), (16,0,0)								
**Ge**	**(1,3,17)**	**18.948**	**81.67**	**0.3321**	**1.32**	**2.8051**	**3.7**	**35.06**	**35.26**
	Equiv. Refl.: (1,17,3), (3,1,17), (3,11,13), (3,13,11), (3,17,1), (5,7,15), (5,15,7), (7,5,15), (7,9,13) (7,13,9), (7,15,5), (9,7,13), (9,13,7), (11,3,13), (11,13,3), (13,3,11), (13,7,9), (13,9,7) (13,11,3), (15,5,7), (15,7,5), (17,1,3), (17,3,1)								
**Si**	**(5,11,11)**	**18.651**	**76.9**	**0.3069**	**0.36**	**4.4577**	**1.6**	**55.72**	**55.74**
	Equiv. Refl.: (7,7,13), (7,13,7), (11,5,11), (11,11,5), (13,7,7)								
**Si**	**(3,5,15)**	**18.37**	**73.59**	**0.2644**	**0.31**	**5.6402**	**1.72**	**70.5**	**70.52**
	Equiv. Refl.: (3,9,13), (3,13,9), (3,15,5), (5,3,15), (5,15,3), (9,3,13), (9,13,3), (13,3,9), (13,9,3) (15,3,5), (15,5,3)								
**Ge**	**(1,1,17)**	**18.693**	**77.45**	**0.2454**	**0.93**	**4.2622**	**3.98**	**53.28**	**53.43**
	Equiv. Refl.: (1,11,13), (1,13,11), (1,17,1), (7,11,11), (11,1,13), (11,7,11), (11,11,7), (11,13,1), (13,1,11) (13,11,1), (17,1,1)								
**Si**	**(1,5,15)**	**18.084**	**70.79**	**0.2441**	**0.28**	**6.6719**	**1.86**	**83.4**	**83.42**
	Equiv. Refl.: (1,9,13), (1,13,9), (1,15,5), (3,11,11), (5,1,15), (5,15,1), (7,9,11), (7,11,9), (9,1,13) (9,7,11), (9,11,7), (9,13,1), (11,3,11), (11,7,9), (11,9,7), (11,11,3), (13,1,9), (13,9,1) (15,1,5), (15,5,1)								
**Ge**	**(3,7,15)**	**18.434**	**74.28**	**0.218**	**0.79**	**5.3895**	**4.28**	**67.37**	**67.5**
	Equiv. Refl.: (3,15,7), (7,3,15), (7,15,3), (9,9,11), (9,11,9), (11,9,9), (15,3,7), (15,7,3)								
**Ge**	**(1,7,15)**	**18.171**	**71.6**	**0.2072**	**0.72**	**6.3687**	**4.61**	**79.61**	**79.74**
	Equiv. Refl.: (1,15,7), (5,5,15), (5,9,13), (5,13,9), (5,15,5), (7,1,15), (7,15,1), (9,5,13), (9,13,5) (13,5,9), (13,9,5), (15,1,7), (15,5,5), (15,7,1)								

Ei =	21.657	keV						ΔEg	ΔEt
Cryst	Refl	EB	ΘB	∫ IR dΘ	Width	Ei cotΘ	ΔE	2m, 50µm	2m, 50µm
	(h,k,l)	[keV]	[°]	[µrad]	[µrad]	[meV/µrad]	[meV]	[meV]	[meV]
**Si**	**(8,12,12)**	**21.415**	**81.43**	**0.3272**	**0.37**	**3.2623**	**1.21**	**40.78**	**40.8**
	Equiv. Refl.: (12,8,12), (12,12,8)								
**Ge**	**(8,8,16)**	**21.473**	**82.52**	**0.278**	**1.02**	**2.8427**	**2.89**	**35.53**	**35.65**
	Equiv. Refl.: (8,16,8), (16,8,8)								
**Si**	**(3,11,15)**	**21.506**	**83.24**	**0.2657**	**0.33**	**2.5669**	**0.84**	**32.09**	**32.1**
	Equiv. Refl.: (3,15,11), (7,9,15), (7,15,9), (9,7,15), (9,15,7), (11,3,15), (11,15,3), (15,3,11), (15,7,9) (15,9,7), (15,11,3)								
**Si**	**(2,4,18)**	**21.171**	**77.83**	**0.2454**	**0.28**	**4.6688**	**1.29**	**58.36**	**58.37**
	Equiv. Refl.: (2,12,14), (2,14,12), (2,18,4), (4,2,18), (4,18,2), (10,10,12), (10,12,10), (12,2,14), (12,10,10) (12,14,2), (14,2,12), (14,12,2), (18,2,4), (18,4,2)								
**Si**	**(4,8,16)**	**20.923**	**75.04**	**0.2128**	**0.24**	**5.7865**	**1.38**	**72.33**	**72.34**
	Equiv. Refl.: (4,16,8), (8,4,16), (8,16,4), (16,4,8), (16,8,4)								
**Ge**	**(4,6,18)**	**21.248**	**78.85**	**0.2058**	**0.72**	**4.2699**	**3.09**	**53.37**	**53.46**
	Equiv. Refl.: (4,18,6), (6,4,18), (6,12,14), (6,14,12), (6,18,4), (12,6,14), (12,14,6), (14,6,12), (14,12,6) (18,4,6), (18,6,4)								
**Si**	**(0,2,18)**	**20.672**	**72.66**	**0.1957**	**0.22**	**6.7631**	**1.47**	**84.54**	**84.55**
	Equiv. Refl.: (0,18,2), (2,0,18), (2,18,0), (6,6,16), (6,16,6), (16,6,6), (18,0,2), (18,2,0)								
**Si**	**(0,8,16)**	**20.419**	**70.53**	**0.1861**	**0.21**	**7.6557**	**1.58**	**95.7**	**95.71**
	Equiv. Refl.: (0,16,8), (8,0,16), (8,16,0), (16,0,8), (16,8,0)								
**Ge**	**(1,5,19)**	**21.557**	**84.48**	**0.1797**	**0.95**	**2.0934**	**2**	**26.17**	**26.24**
	Equiv. Refl.: (1,19,5), (5,1,19), (5,19,1), (7,7,17), (7,13,13), (7,17,7), (9,9,15), (9,15,9), (13,7,13) (13,13,7), (15,9,9), (17,7,7), (19,1,5), (19,5,1)								
**Si**	**(1,11,15)**	**21.263**	**79.05**	**0.1752**	**0.21**	**4.1898**	**0.89**	**52.37**	**52.38**
	Equiv. Refl.: (1,15,11), (3,7,17), (3,13,13), (3,17,7), (7,3,17), (7,17,3), (11,1,15), (11,15,1), (13,3,13) (13,13,3), (15,1,11), (15,11,1), (17,3,7), (17,7,3)								
**Ge**	**(0,6,18)**	**20.791**	**73.74**	**0.172**	**0.56**	**6.3157**	**3.53**	**78.95**	**79.02**
	Equiv. Refl.: (0,18,6), (2,10,16), (2,16,10), (6,0,18), (6,18,0), (8,10,14), (8,14,10), (10,2,16), (10,8,14) (10,14,8), (10,16,2), (14,8,10), (14,10,8), (16,2,10), (16,10,2), (18,0,6), (18,6,0)								
**Ge**	**(8,12,12)**	**20.559**	**71.67**	**0.1683**	**0.53**	**7.1732**	**3.77**	**89.66**	**89.74**
	Equiv. Refl.: (12,8,12), (12,12,8)								
**Si**	**(1,7,17)**	**21.016**	**76.03**	**0.1467**	**0.18**	**5.3886**	**0.95**	**67.36**	**67.36**
	Equiv. Refl.: (1,13,13), (1,17,7), (5,5,17), (5,17,5), (7,1,17), (7,11,13), (7,13,11), (7,17,1), (11,7,13) (11,13,7), (13,1,13), (13,7,11), (13,11,7), (13,13,1), (17,1,7), (17,5,5), (17,7,1)								
**Si**	**(5,9,15)**	**20.767**	**73.51**	**0.1329**	**0.16**	**6.4089**	**1.02**	**80.11**	**80.12**
	Equiv. Refl.: (5,15,9), (9,5,15), (9,9,13), (9,13,9), (9,15,5), (13,9,9), (15,5,9), (15,9,5)								
**Si**	**(3,5,17)**	**20.514**	**71.3**	**0.1254**	**0.15**	**7.3286**	**1.09**	**91.61**	**91.61**
	Equiv. Refl.: (3,17,5), (5,3,17), (5,17,3), (7,7,15), (7,15,7), (9,11,11), (11,9,11), (11,11,9), (15,7,7) (17,3,5), (17,5,3)								
**Ge**	**(3,3,19)**	**21.333**	**80.07**	**0.111**	**0.56**	**3.7915**	**2.13**	**47.39**	**47.44**
	Equiv. Refl.: (3,9,17), (3,17,9), (3,19,3), (9,3,17), (9,17,3), (17,3,9), (17,9,3), (19,3,3)								
**Ge**	**(1,3,19)**	**21.106**	**77.05**	**0.0945**	**0.46**	**4.9799**	**2.28**	**62.25**	**62.29**
	Equiv. Refl.: (1,9,17), (1,17,9), (1,19,3), (3,1,19), (3,19,1), (5,11,15), (5,15,11), (9,1,17), (9,11,13) (9,13,11), (9,17,1), (11,5,15), (11,9,13), (11,13,9), (11,15,5), (13,9,11), (13,11,9), (15,5,11) (15,11,5), (17,1,9), (17,9,1), (19,1,3), (19,3,1)								
**Ge**	**(1,1,19)**	**20.877**	**74.58**	**0.0881**	**0.41**	**5.9735**	**2.43**	**74.67**	**74.71**
	Equiv. Refl.: (1,19,1), (5,7,17), (5,13,13), (5,17,7), (7,5,17), (7,17,5), (11,11,11), (13,5,13), (13,13,5) (17,5,7), (17,7,5), (19,1,1)								

Ei =	21.657	keV						ΔEg	ΔEt
Cryst	Refl	EB	ΘB	∫ IR dΘ	Width	Ei cotΘ	ΔE	2m, 50µm	2m, 50µm
	(h,k,l)	[keV]	[°]	[µrad]	[µrad]	[meV/µrad]	[meV]	[meV]	[meV]
**Ge**	**(3,11,15)**	**20.646**	**72.42**	**0.0858**	**0.38**	**6.8597**	**2.6**	**85.75**	**85.79**
	Equiv. Refl.: (3,15,11), (7,9,15), (7,15,9), (9,7,15), (9,15,7), (11,3,15), (11,15,3), (15,3,11), (15,7,9) (15,9,7), (15,11,3)								
**Ge**	**(1,11,15)**	**20.412**	**70.48**	**0.0857**	**0.36**	**7.6781**	**2.78**	**95.98**	**96.02**
	Equiv. Refl.: (1,15,11), (3,7,17), (3,13,13), (3,17,7), (7,3,17), (7,17,3), (11,1,15), (11,15,1), (13,3,13) (13,13,3), (15,1,11), (15,11,1), (17,3,7), (17,7,3)								

Ei = Cryst	11.271 Refl (h,k,l)	keV EB [keV]	ΘB [°]	∫ IR dΘ [µrad]	Width [µrad]	Ei cotΘ [meV/µrad]	ΔE [meV]	ΔEg 2m, 50µm [meV]	ΔEt 2m, 50µm [meV]
**Si**	**(4,4,8)**	**11.184**	**82.87**	**12.0217**	**10.38**	**1.41**	**14.64**	**17.62**	**22.91**
	Equiv. Refl.: (4,8,4), (8,4,4)								
**Ge**	**(0,2,10)**	**11.175**	**82.51**	**5.6818**	**12.62**	**1.4821**	**18.71**	**18.53**	**26.33**
	Equiv. Refl.: (0,10,2), (2,0,10), (2,6,8), (2,8,6), (2,10,0), (6,2,8), (6,8,2), (8,2,6), (8,6,2) (10,0,2), (10,2,0)								
**Si**	**(4,6,6)**	**10.708**	**71.81**	**5.5575**	**4.73**	**3.7037**	**17.51**	**46.3**	**49.5**
	Equiv. Refl.: (6,4,6), (6,6,4)								
**Si**	**(1,3,9)**	**10.889**	**75.03**	**4.319**	**3.84**	**3.0129**	**11.56**	**37.66**	**39.4**
	Equiv. Refl.: (1,9,3), (3,1,9), (3,9,1), (9,1,3), (9,3,1)								
**Ge**	**(4,4,8)**	**10.736**	**72.28**	**3.1476**	**6.27**	**3.6008**	**22.59**	**45.01**	**50.36**
	Equiv. Refl.: (4,8,4), (8,4,4)								
**Ge**	**(1,7,7)**	**10.903**	**75.32**	**1.7274**	**5.03**	**2.9535**	**14.86**	**36.92**	**39.8**
	Equiv. Refl.: (3,3,9), (3,9,3), (5,5,7), (5,7,5), (7,1,7), (7,5,5), (7,7,1), (9,3,3)								
**LiNbO3**	**(0,-8,-4)**	**11.267**	**88.43**	**153.332**	**135.5**	**0.308**	**41.74**	**3.85**	**41.92**
	Equiv. Refl.: (8,0,-4), (-8,8,-4)								
**LiNbO3**	**(0,8,4)**	**11.267**	**88.43**	**149.007**	**135.5**	**0.308**	**41.74**	**3.85**	**41.92**
	Equiv. Refl.: (8,-8,4), (-8,0,4)								
**LiNbO3**	**(1,1,24)**	**10.999**	**77.39**	**17.171**	**15.6**	**2.521**	**39.31**	**31.52**	**50.39**
	Equiv. Refl.: (1,-2,24), (2,-1,24), (-1,2,24), (-1,-1,24), (-2,1,24)								
**LiNbO3**	**(1,1,-24)**	**10.999**	**77.39**	**17.058**	**15.6**	**2.521**	**39.31**	**31.52**	**50.39**
	Equiv. Refl.: (1,-2,-24), (2,-1,-24), (-1,2,-24), (-1,-1,-24), (-2,1,-24)								
**LiNbO3**	**(1,6,-14)**	**11.06**	**78.91**	**16.522**	**15**	**2.21**	**33.05**	**27.63**	**43.08**
	Equiv. Refl.: (6,-7,-14), (7,-6,-14), (-1,7,-14), (-6,-1,-14), (-7,1,-14)								
**LiNbO3**	**(3,-7,16)**	**11.078**	**79.39**	**15.671**	**14.4**	**2.111**	**30.46**	**26.39**	**40.3**
	Equiv. Refl.: (4,3,16), (7,-3,16), (-3,-4,16), (-4,7,16), (-7,4,16)								
**LiNbO3**	**(1,-7,14)**	**11.06**	**78.91**	**15.47**	**15**	**2.21**	**33.05**	**27.63**	**43.08**
	Equiv. Refl.: (6,1,14), (7,-1,14), (-1,-6,14), (-6,7,14), (-7,6,14)								
**LiNbO3**	**(3,6,0)**	**11.037**	**78.3**	**15.223**	**14.1**	**2.335**	**32.92**	**29.19**	**43.99**
	Equiv. Refl.: (6,-9,0), (9,-6,0), (-3,9,0), (-6,-3,0), (-9,3,0)								
**LiNbO3**	**(3,-9,0)**	**11.037**	**78.3**	**14.892**	**14.1**	**2.335**	**32.92**	**29.19**	**43.99**
	Equiv. Refl.: (6,3,0), (9,-3,0), (-3,-6,0), (-6,9,0), (-9,6,0)								
**LiNbO3**	**(3,4,-16)**	**11.078**	**79.39**	**14.786**	**14.4**	**2.111**	**30.46**	**26.39**	**40.3**
	Equiv. Refl.: (4,-7,-16), (7,-4,-16), (-3,7,-16), (-4,-3,-16), (-7,3,-16)								
**Sapph**	**(0,7,8)**	**11.202**	**83.65**	**14.76**	**12.5**	**1.255**	**15.66**	**15.68**	**22.16**
	Equiv. Refl.: (0,-7,-8), (7,0,-8), (7,-7,8), (-7,0,8), (-7,7,-8)								
**LiNbO3**	**(4,4,12)**	**11.027**	**78.06**	**14.535**	**13.4**	**2.383**	**31.81**	**29.78**	**43.58**
	Equiv. Refl.: (4,-8,12), (8,-4,12), (-4,8,12), (-4,-4,12), (-8,4,12)								
**LiNbO3**	**(4,-9,4)**	**11.006**	**77.56**	**14.186**	**12.9**	**2.486**	**32.1**	**31.08**	**44.68**
	Equiv. Refl.: (5,4,4), (9,-4,4), (-4,-5,4)								
**LiNbO3**	**(-5,9,4)**	**11.006**	**77.56**	**14.185**	**12.9**	**2.486**	**32.1**	**31.08**	**44.68**
	Equiv. Refl.: (-9,5,4)								
**LiNbO3**	**(4,5,2)**	**10.897**	**75.2**	**14.156**	**12.9**	**2.979**	**38.36**	**37.24**	**53.46**
	Equiv. Refl.: (5,-9,2), (9,-5,2), (-4,9,2), (-5,-4,2), (-9,4,2)								
**LiNbO3**	**(4,-9,-2)**	**10.897**	**75.2**	**13.931**	**12.9**	**2.979**	**38.36**	**37.24**	**53.46**
	Equiv. Refl.: (5,4,-2), (9,-4,-2), (-4,-5,-2), (-5,9,-2), (-9,5,-2)								
**LiNbO3**	**(4,4,-12)**	**11.027**	**78.06**	**13.816**	**13.4**	**2.383**	**31.81**	**29.78**	**43.58**
	Equiv. Refl.: (4,-8,-12), (8,-4,-12), (-4,8,-12), (-4,-4,-12), (-8,4,-12)								

Ei =	11.271	keV						ΔEg	ΔEt
Cryst	Refl	EB	ΘB	∫ IR dΘ	Width	Ei cotΘ	ΔE	2m, 50µm	2m, 50µm
	(h,k,l)	[keV]	[°]	[µrad]	[µrad]	[meV/µrad]	[meV]	[meV]	[meV]
LiNbO3	(1,-4,-22)	11.042	78.42	13.785	12.9	2.309	29.88	28.86	41.55
	Equiv. Refl.: (3,1,-22), (4,-1,-22), (-1,-3,-22), (-3,4,-22), (-4,3,-22)								
LiNbO3	(1,3,22)	11.042	78.42	13.41	12.9	2.309	29.88	28.86	41.55
	Equiv. Refl.: (3,-4,22), (4,-3,22), (-1,4,22), (-3,-1,22), (-4,1,22)								
LiNbO3	(3,3,18)	10.816	73.67	13.248	11.9	3.302	39.2	41.28	56.92
	Equiv. Refl.: (3,-6,18), (6,-3,18), (-3,6,18), (-3,-3,18), (-6,3,18)								
LiNbO3	(4,5,-4)	11.006	77.56	13.244	12.9	2.486	32.1	31.08	44.68
	Equiv. Refl.: (5,-9,-4), (9,-5,-4), (-4,9,-4), (-5,-4,-4), (-9,4,-4)								
LiNbO3	(2,-8,10)	10.979	76.93	13.175	12.2	2.617	32	32.71	45.76
	Equiv. Refl.: (6,2,10), (8,-2,10), (-2,-6,10), (-6,8,10), (-8,6,10)								
LiNbO3	(2,6,-10)	10.979	76.93	12.776	12.2	2.617	32	32.71	45.76
	Equiv. Refl.: (6,-8,-10), (8,-6,-10), (-2,8,-10), (-6,-2,-10), (-8,2,-10)								
LiNbO3	(3,3,-18)	10.816	73.67	12.66	11.9	3.302	39.2	41.28	56.92
	Equiv. Refl.: (3,-6,-18), (6,-3,-18), (-3,6,-18), (-3,-3,-18), (-6,3,-18)								
LiNbO3	(0,-8,2)	11.16	81.94	12.53	12.4	1.595	19.74	19.94	28.05
	Equiv. Refl.: (8,0,2), (-8,8,2)								
LiNbO3	(1,-8,-6)	10.835	74.02	12.211	11.3	3.228	36.4	40.35	54.34
	Equiv. Refl.: (7,1,-6), (8,-1,-6), (-1,-7,-6), (-7,8,-6), (-8,7,-6)								
LiNbO3	(0,7,-10)	10.711	71.87	12.152	11	3.69	40.75	46.13	61.55
	Equiv. Refl.: (-7,0,-10)								
LiNbO3	(7,-7,-10)	10.711	71.87	12.151	11	3.69	40.75	46.13	61.55
LiNbO3	(1,7,6)	10.835	74.02	12.135	11.3	3.228	36.4	40.35	54.34
	Equiv. Refl.: (7,-8,6), (8,-7,6), (-1,8,6), (-7,-1,6), (-8,1,6)								
Quartz	(7,-7,4)	11.183	82.85	12.066	10.2	1.414	14.39	17.67	22.79
Quartz	(7,0,-4)	11.183	82.85	12.065	10.2	1.414	14.39	17.67	22.79
	Equiv. Refl.: (-7,0,4), (-7,7,-4)								
Quartz	(0,7,4)	11.183	82.85	12.059	10.2	1.414	14.38	17.67	22.78
	Equiv. Refl.: (0,-7,-4)								
LiNbO3	(0,-7,10)	10.711	71.87	12.028	11	3.69	40.75	46.13	61.55
	Equiv. Refl.: (7,0,10), (-7,7,10)								
LiNbO3	(0,8,-2)	11.16	81.94	11.746	12.4	1.595	19.74	19.94	28.05
	Equiv. Refl.: (8,-8,-2), (-8,0,-2)								
LiNbO3	(1,7,-6)	10.835	74.02	11.569	10.6	3.228	34.34	40.35	52.98
	Equiv. Refl.: (7,-8,-6), (8,-7,-6), (-1,8,-6), (-7,-1,-6), (-8,1,-6)								
LiNbO3	(1,-8,6)	10.835	74.02	11.442	10.6	3.228	34.34	40.35	52.98
	Equiv. Refl.: (7,1,6), (8,-1,6), (-1,-7,6), (-7,8,6), (-8,7,6)								
Quartz	(-4,-3,6)	11.221	84.59	11.282	9.6	1.067	10.29	13.34	16.84
Quartz	(-4,7,-6)	11.221	84.59	11.281	9.6	1.067	10.28	13.34	16.84
Quartz	(-3,-4,-6)	11.221	84.59	11.275	9.6	1.067	10.28	13.34	16.84
Quartz	(7,-3,-6)	11.221	84.59	11.272	9.6	1.067	10.28	13.34	16.84
	Equiv. Refl.: (7,-4,6), (-3,7,6)								
Quartz	(4,3,-6)	11.221	84.59	11.062	9.6	1.067	10.29	13.34	16.84
Quartz	(4,-7,6)	11.221	84.59	11.06	9.6	1.067	10.28	13.34	16.84
Quartz	(3,4,6)	11.221	84.59	11.054	9.6	1.067	10.28	13.34	16.84
Quartz	(3,-7,-6)	11.221	84.59	11.051	9.6	1.067	10.28	13.34	16.84
Quartz	(-7,3,6)	11.221	84.59	11.05	9.6	1.067	10.28	13.34	16.84
	Equiv. Refl.: (-7,4,-6)								
LiNbO3	(2,-8,-8)	10.646	70.83	10.906	9.9	3.918	38.95	48.98	62.58
	Equiv. Refl.: (6,2,-8), (8,-2,-8), (-2,-6,-8), (-6,8,-8), (-8,6,-8)								
LiNbO3	(2,6,8)	10.646	70.83	10.662	9.9	3.918	38.95	48.98	62.58
	Equiv. Refl.: (6,-8,8), (8,-6,8), (-2,8,8), (-6,-2,8), (-8,2,8)								

Ei =	11.271	keV						ΔEg	ΔEt
**Cryst**	**Refl**	**EB**	**ΘB**	**∫ IR dΘ**	**Width**	**Ei cotΘ**	**ΔE**	**2m, 50µm**	**2m, 50µm**
	**(h,k,l)**	**[keV]**	**[°]**	**[µrad]**	**[µrad]**	**[meV/µrad]**	**[meV]**	**[meV]**	**[meV]**
**LiNbO3**	**(3,5,10)**	**10.711**	**71.87**	**10.438**	**9.1**	**3.69**	**33.76**	**46.13**	**57.16**
	Equiv. Refl.: (5,-8,10), (8,-5,10), (-3,8,10), (-5,-3,10), (-8,3,10)								
**LiNbO3**	**(2,-5,-20)**	**10.804**	**73.45**	**9.725**	**8.8**	**3.35**	**29.61**	**41.88**	**51.29**
	Equiv. Refl.: (3,2,-20), (5,-2,-20), (-2,-3,-20), (-3,5,-20), (-5,3,-20)								
**LiNbO3**	**(3,-8,-10)**	**10.711**	**71.87**	**9.43**	**9.1**	**3.69**	**33.76**	**46.13**	**57.16**
	Equiv. Refl.: (5,3,-10), (8,-3,-10), (-3,-5,-10), (-5,8,-10), (-8,5,-10)								
**Sapph**	**(2,6,-4)**	**11.015**	**77.77**	**9.239**	**7.6**	**2.443**	**18.55**	**30.54**	**35.73**
	Equiv. Refl.: (2,-8,4), (6,2,4), (6,-8,-4), (8,-2,4), (8,-6,-4), (-2,8,-4), (-2,-6,4), (-6,8,4), (-6,-2,-4) (-8,2,-4), (-8,6,4)								
**LiNbO3**	**(2,3,20)**	**10.804**	**73.45**	**9.026**	**8.8**	**3.35**	**29.61**	**41.88**	**51.29**
	Equiv. Refl.: (3,-5,20), (5,-3,20), (-2,5,20), (-3,-2,20), (-5,2,20)								
**Quartz**	**(0,6,-6)**	**11.126**	**80.79**	**8.988**	**7.4**	**1.827**	**13.56**	**22.83**	**26.56**
	Equiv. Refl.: (0,-6,6)								
**Quartz**	**(6,0,6)**	**11.126**	**80.79**	**8.978**	**7.4**	**1.827**	**13.55**	**22.83**	**26.55**
	Equiv. Refl.: (-6,0,-6)								
**Quartz**	**(6,-6,-6)**	**11.126**	**80.79**	**8.977**	**7.4**	**1.827**	**13.55**	**22.83**	**26.55**
	Equiv. Refl.: (-6,6,6)								
**Sapph**	**(1,6,10)**	**10.959**	**76.49**	**8.837**	**7.2**	**2.708**	**19.46**	**33.85**	**39.05**
	Equiv. Refl.: (1,-7,-10), (6,1,-10), (6,-7,10), (7,-1,-10), (7,-6,10), (-1,7,10), (-1,-6,-10), (-6,7,-10), (-6,-1,10) (-7,1,10), (-7,6,-10)								
**Sapph**	**(1,3,-20)**	**10.979**	**76.94**	**8.562**	**7**	**2.615**	**18.36**	**32.69**	**37.49**
	Equiv. Refl.: (1,-4,20), (3,1,20), (3,-4,-20), (4,-1,20), (4,-3,-20), (-1,4,-20), (-1,-3,20), (-3,4,20), (-3,-1,-20) (-4,1,-20), (-4,3,20)								
**Quartz**	**(5,0,-7)**	**10.841**	**74.13**	**7.512**	**5.9**	**3.204**	**18.83**	**40.05**	**44.26**
	Equiv. Refl.: (5,-5,7), (-5,0,7), (-5,5,-7)								
**Quartz**	**(0,5,7)**	**10.841**	**74.13**	**7.51**	**5.9**	**3.204**	**18.83**	**40.05**	**44.26**
	Equiv. Refl.: (0,-5,-7)								
**Quartz**	**(2,4,-7)**	**11.131**	**80.97**	**7.086**	**6**	**1.791**	**10.72**	**22.39**	**24.83**
	Equiv. Refl.: (2,-6,7)								
**Quartz**	**(4,-6,-7)**	**11.131**	**80.97**	**7.083**	**6**	**1.791**	**10.72**	**22.39**	**24.82**
	Equiv. Refl.: (-6,2,-7), (-6,4,7)								
**Quartz**	**(4,2,7)**	**11.131**	**80.97**	**7.081**	**6**	**1.791**	**10.71**	**22.39**	**24.82**
	Equiv. Refl.: (-3,8,-3), (-3,-5,3)								
**Quartz**	**(8,-3,3)**	**10.764**	**72.75**	**7.08**	**5.6**	**3.501**	**19.48**	**43.76**	**47.9**
	Equiv. Refl.: (8,-5,-3), (-5,8,3), (-5,-3,-3)								
**Quartz**	**(3,5,-3)**	**10.764**	**72.75**	**7.064**	**5.6**	**3.501**	**19.48**	**43.76**	**47.9**
**Quartz**	**(3,-8,3)**	**10.764**	**72.75**	**7.063**	**5.6**	**3.501**	**19.48**	**43.76**	**47.9**
	Equiv. Refl.: (5,3,3), (5,-8,-3), (-8,3,-3), (-8,5,3)								
**Quartz**	**(0,4,8)**	**10.87**	**74.68**	**7.01**	**5.7**	**3.088**	**17.5**	**38.6**	**42.38**
	Equiv. Refl.: (0,-4,-8)								
**Quartz**	**(4,0,-8)**	**10.87**	**74.68**	**7.007**	**5.7**	**3.088**	**17.49**	**38.6**	**42.38**
	Equiv. Refl.: (-4,0,8)								
**Quartz**	**(4,-4,8)**	**10.87**	**74.68**	**7.006**	**5.7**	**3.088**	**17.49**	**38.6**	**42.38**
	Equiv. Refl.: (-4,4,-8)								
**Quartz**	**(-2,6,-7)**	**11.131**	**80.97**	**6.912**	**6**	**1.791**	**10.72**	**22.39**	**24.83**
	Equiv. Refl.: (-2,-4,7)								
**Quartz**	**(6,-4,-7)**	**11.131**	**80.97**	**6.911**	**6**	**1.791**	**10.72**	**22.39**	**24.83**
**Quartz**	**(6,-2,7)**	**11.131**	**80.97**	**6.91**	**6**	**1.791**	**10.72**	**22.39**	**24.82**
	Equiv. Refl.: (-4,6,7)								
**Quartz**	**(-4,-2,-7)**	**11.131**	**80.97**	**6.908**	**6**	**1.791**	**10.71**	**22.39**	**24.82**

Ei =	11.271	keV						ΔEg	ΔEt
Cryst	Refl	EB	ΘB	∫ IR dΘ	Width	Ei cotΘ	ΔE	2m, 50μm	2m, 50μm
	(h,k,l)	[keV]	[°]	[μrad]	[μrad]	[meV/μrad]	[meV]	[meV]	[meV]
LiNbO3	(0,0,-24)	10.732	72.21	6.891	6.4	3.616	23.3	45.2	50.85
Sapph	(1,5,14)	10.715	71.93	6.552	5.4	3.678	19.74	45.97	50.03
	Equiv. Refl.: (1,-6,-14), (5,1,-14), (5,-6,14), (6,-1,-14), (6,-5,14), (-1,6,14), (-1,-5,-14), (-5,6,-14), (-5,-1,14) (-6,1,14), (-6,5,-14)								
Sapph	(3,5,4)	10.703	71.73	6.538	5.4	3.722	19.96	46.52	50.62
	Equiv. Refl.: (3,-8,-4), (5,3,-4), (5,-8,4), (8,-3,-4), (8,-5,4), (-3,8,4), (-3,-5,-4), (-5,8,-4), (-5,-3,4) (-8,3,4), (-8,5,-4)								
Quartz	(3,0,9)	11.21	84.06	6.506	6	1.174	7.07	14.67	16.28
	Equiv. Refl.: (-3,0,-9)								
Quartz	(3,-3,-9)	11.21	84.06	6.505	6	1.174	7.07	14.67	16.28
	Equiv. Refl.: (-3,3,9)								
Quartz	(0,3,-9)	11.21	84.06	6.486	6	1.174	7.05	14.67	16.28
	Equiv. Refl.: (0,-3,9)								
LiNbO3	(0,0,24)	10.732	72.21	6.372	6.4	3.616	23.3	45.2	50.85
Quartz	(-3,-2,-8)	11.16	81.94	6.341	5.4	1.597	8.57	19.96	21.73
Quartz	(-3,5,8)	11.16	81.94	6.34	5.4	1.597	8.57	19.96	21.73
Quartz	(5,-3,-8)	11.16	81.94	6.337	5.4	1.597	8.57	19.96	21.72
	Equiv. Refl.: (-2,5,-8)								
Quartz	(5,-2,8)	11.16	81.94	6.336	5.4	1.597	8.57	19.96	21.72
	Equiv. Refl.: (-2,-3,8)								
LiNbO3	(2,4,19)	11.239	85.71	6.233	9.7	0.845	8.17	10.57	13.36
	Equiv. Refl.: (2,-6,-19), (4,2,-19), (4,-6,19), (6,-2,-19), (6,-4,19), (-2,6,19), (-2,-4,-19), (-4,6,-19), (-4,-2,19) (-6,2,19), (-6,4,-19)								
Quartz	(1,-7,-5)	11.143	81.37	6.116	5.1	1.712	8.79	21.39	23.13
Quartz	(1,6,5)	11.143	81.37	6.115	5.1	1.712	8.79	21.39	23.13
Quartz	(-7,6,-5)	11.143	81.37	6.113	5.1	1.712	8.79	21.39	23.13
Quartz	(6,1,-5)	11.143	81.37	6.112	5.1	1.712	8.79	21.39	23.13
	Equiv. Refl.: (-7,1,5)								
Quartz	(6,-7,5)	11.143	81.37	6.111	5.1	1.712	8.79	21.39	23.13
Quartz	(2,-5,8)	11.16	81.94	5.983	5.4	1.597	8.57	19.96	21.72
	Equiv. Refl.: (3,2,8), (3,-5,-8)								
Quartz	(-5,3,8)	11.16	81.94	5.982	5.4	1.597	8.57	19.96	21.72
Quartz	(2,3,-8)	11.16	81.94	5.981	5.4	1.597	8.57	19.96	21.72
	Equiv. Refl.: (-5,2,-8)								
Quartz	(3,-8,4)	11.183	82.85	5.868	5.1	1.414	7.27	17.67	19.11
	Equiv. Refl.: (-8,3,-4)								
Quartz	(3,5,-4)	11.183	82.85	5.867	5.1	1.414	7.27	17.67	19.11
	Equiv. Refl.: (5,3,4), (5,-8,-4)								
Quartz	(-8,5,4)	11.183	82.85	5.866	5.1	1.414	7.27	17.67	19.11
Quartz	(-3,8,-4)	11.183	82.85	5.789	5.1	1.414	7.27	17.67	19.11
Quartz	(8,-3,4)	11.183	82.85	5.788	5.1	1.414	7.27	17.67	19.11
	Equiv. Refl.: (-3,-5,4), (-5,8,4), (-5,-3,-4)								
Quartz	(8,-5,-4)	11.183	82.85	5.787	5.1	1.414	7.27	17.67	19.11
Quartz	(-1,7,5)	11.143	81.37	5.735	5.1	1.712	8.79	21.39	23.13
Quartz	(-1,-6,-5)	11.143	81.37	5.733	5.1	1.712	8.79	21.39	23.13
Quartz	(7,-6,5)	11.143	81.37	5.732	5.1	1.712	8.79	21.39	23.13
Quartz	(7,-1,-5)	11.143	81.37	5.731	5.1	1.712	8.79	21.39	23.13
	Equiv. Refl.: (-6,-1,5)								
Quartz	(-6,7,-5)	11.143	81.37	5.73	5.1	1.712	8.79	21.39	23.13

Ei =	11.271	keV						ΔEg	ΔEt
Cryst	Refl	EB	ΘB	∫ IR dΘ	Width	Ei cotΘ	ΔE	2m, 50μm	2m, 50μm
	(h,k,l)	[keV]	[°]	[μrad]	[μrad]	[meV/μrad]	[meV]	[meV]	[meV]
**Quartz**	**(8,-1,0)**	**11**	**77.41**	**5.656**	**4.7**	**2.517**	**11.81**	**31.47**	**33.61**
	Equiv. Refl.: (8,-7,0), (-1,8,0), (-1,-7,0), (-7,8,0), (-7,-1,0)								
**Sapph**	**(4,4,6)**	**10.809**	**73.54**	**5.61**	**4.7**	**3.33**	**15.81**	**41.62**	**44.52**
	Equiv. Refl.: (4,4,-6), (4,-8,6), (4,-8,-6), (8,-4,6), (8,-4,-6), (-4,8,6), (-4,8,-6), (-4,-4,6), (-4,-4,-6) (-8,4,6), (-8,4,-6)								
**Quartz**	**(2,6,-3)**	**11.056**	**78.78**	**5.606**	**4.6**	**2.236**	**10.33**	**27.94**	**29.79**
	Equiv. Refl.: (2,-8,3)								
**Quartz**	**(-8,2,-3)**	**11.056**	**78.78**	**5.604**	**4.6**	**2.236**	**10.32**	**27.94**	**29.79**
	Equiv. Refl.: (-8,6,3)								
**Quartz**	**(6,2,3)**	**11.056**	**78.78**	**5.602**	**4.6**	**2.236**	**10.32**	**27.94**	**29.79**
	Equiv. Refl.: (6,-8,-3)								
**Quartz**	**(-2,8,-3)**	**11.056**	**78.78**	**5.573**	**4.6**	**2.236**	**10.33**	**27.94**	**29.79**
	Equiv. Refl.: (-2,-6,3)								
**Quartz**	**(8,-2,3)**	**11.056**	**78.78**	**5.571**	**4.6**	**2.236**	**10.32**	**27.94**	**29.79**
	Equiv. Refl.: (8,-6,-3)								
**Quartz**	**(-6,8,3)**	**11.056**	**78.78**	**5.569**	**4.6**	**2.236**	**10.32**	**27.94**	**29.79**
	Equiv. Refl.: (-6,-2,-3)								
**Quartz**	**(1,7,0)**	**11**	**77.41**	**5.55**	**4.7**	**2.517**	**11.81**	**31.47**	**33.61**
	Equiv. Refl.: (1,-8,0), (7,1,0), (7,-8,0), (-8,1,0), (-8,7,0)								
**Sapph**	**(0,5,16)**	**10.719**	**72**	**5.482**	**4.4**	**3.663**	**16.25**	**45.79**	**48.58**
	Equiv. Refl.: (0,-5,-16), (5,0,-16), (5,-5,16), (-5,0,16), (-5,5,-16)								
**Quartz**	**(4,4,-4)**	**11.088**	**79.66**	**5.47**	**4.5**	**2.056**	**9.33**	**25.7**	**27.34**
	Equiv. Refl.: (4,-8,4)								
**Quartz**	**(-8,4,4)**	**11.088**	**79.66**	**5.469**	**4.5**	**2.056**	**9.33**	**25.7**	**27.34**
**Quartz**	**(-8,4,-4)**	**11.088**	**79.66**	**5.468**	**4.5**	**2.056**	**9.33**	**25.7**	**27.34**
**Quartz**	**(4,4,4)**	**11.088**	**79.66**	**5.466**	**4.5**	**2.056**	**9.32**	**25.7**	**27.34**
	Equiv. Refl.: (4,-8,-4)								
**Quartz**	**(8,-4,4)**	**11.088**	**79.66**	**5.351**	**4.5**	**2.056**	**9.33**	**25.7**	**27.34**
	Equiv. Refl.: (8,-4,-4), (-4,8,-4), (-4,-4,4)								
**Quartz**	**(-4,8,4)**	**11.088**	**79.66**	**5.348**	**4.5**	**2.056**	**9.33**	**25.7**	**27.34**
**Quartz**	**(-4,-4,-4)**	**11.088**	**79.66**	**5.347**	**4.5**	**2.056**	**9.32**	**25.7**	**27.34**
**Sapph**	**(2,4,16)**	**11.031**	**78.16**	**5.196**	**4.5**	**2.362**	**10.63**	**29.53**	**31.38**
	Equiv. Refl.: (2,-6,-16), (4,2,-16), (4,-6,16), (6,-2,-16), (6,-4,16), (-2,6,16), (-2,-4,-16), (-4,6,-16), (-4,-2,16) (-6,2,16), (-6,4,-16)								
**Quartz**	**(6,-1,-6)**	**10.638**	**70.71**	**4.744**	**3.7**	**3.945**	**14.69**	**49.31**	**51.45**
**Quartz**	**(6,-5,6)**	**10.638**	**70.71**	**4.743**	**3.7**	**3.945**	**14.68**	**49.31**	**51.45**
	Equiv. Refl.: (-1,6,6), (-1,-5,-6), (-5,6,-6), (-5,-1,6)								
**Quartz**	**(-6,1,6)**	**10.638**	**70.71**	**4.727**	**3.7**	**3.945**	**14.69**	**49.31**	**51.45**
**Quartz**	**(1,-6,-6)**	**10.638**	**70.71**	**4.726**	**3.7**	**3.945**	**14.68**	**49.31**	**51.45**
	Equiv. Refl.: (-6,5,-6)								
**Quartz**	**(1,5,6)**	**10.638**	**70.71**	**4.725**	**3.7**	**3.945**	**14.68**	**49.31**	**51.45**
	Equiv. Refl.: (5,1,-6), (5,-6,6)								
**Quartz**	**(8,-1,-1)**	**11.06**	**78.89**	**4.532**	**3.9**	**2.214**	**8.53**	**27.68**	**28.96**
	Equiv. Refl.: (8,-7,1)								
**Quartz**	**(-1,8,1)**	**11.06**	**78.89**	**4.531**	**3.9**	**2.214**	**8.53**	**27.68**	**28.96**
	Equiv. Refl.: (-1,-7,-1), (-7,8,-1), (-7,-1,1)								
**Sapph**	**(2,6,5)**	**11.108**	**80.24**	**4.482**	**4.1**	**1.938**	**8.02**	**24.23**	**25.52**
	Equiv. Refl.: (2,-8,-5), (6,2,-5), (6,-8,5), (8,-2,-5), (8,-6,5), (-2,8,5), (-2,-6,-5), (-6,8,-5), (-6,-2,5) (-8,2,5), (-8,6,-5)								
**Quartz**	**(1,7,1)**	**11.06**	**78.89**	**4.454**	**3.9**	**2.214**	**8.53**	**27.68**	**28.96**
	Equiv. Refl.: (-8,1,1), (-8,7,-1)								

Ei =	11.271	keV						ΔEg	ΔEt
**Cryst**	**Refl**	**EB**	**ΘB**	**∫ IR dΘ**	**Width**	**Ei cotΘ**	**ΔE**	**2m, 50μm**	**2m, 50μm**
	**(h,k,l)**	**[keV]**	**[°]**	**[μrad]**	**[μrad]**	**[meV/μrad]**	**[meV]**	**[meV]**	**[meV]**
**Quartz**	**(1,-8,-1)**	**11.06**	**78.89**	**4.453**	**3.9**	**2.214**	**8.53**	**27.68**	**28.96**
	Equiv. Refl.: (7,1,-1), (7,-8,1)								
**Quartz**	**(0,2,-9)**	**10.727**	**72.12**	**4.264**	**3.4**	**3.636**	**12.37**	**45.45**	**47.11**
	Equiv. Refl.: (0,-2,9)								
**Quartz**	**(-2,0,-9)**	**10.727**	**72.12**	**4.263**	**3.4**	**3.636**	**12.36**	**45.45**	**47.11**
**Quartz**	**(2,0,9)**	**10.727**	**72.12**	**4.262**	**3.4**	**3.636**	**12.36**	**45.45**	**47.11**
	Equiv. Refl.: (2,-2,-9), (-2,2,9)								
**Quartz**	**(8,-2,-2)**	**10.754**	**72.58**	**4.156**	**3.3**	**3.537**	**11.8**	**44.21**	**45.76**
	Equiv. Refl.: (8,-6,2)								
**Quartz**	**(-2,8,2)**	**10.754**	**72.58**	**4.155**	**3.3**	**3.537**	**11.8**	**44.21**	**45.76**
	Equiv. Refl.: (-2,-6,-2), (-6,8,-2), (-6,-2,2)								
**Quartz**	**(2,6,2)**	**10.754**	**72.58**	**4.087**	**3.3**	**3.537**	**11.8**	**44.21**	**45.76**
	Equiv. Refl.: (2,-8,-2), (6,2,-2), (-8,2,2), (-8,6,-2)								
**Quartz**	**(6,-8,2)**	**10.754**	**72.58**	**4.086**	**3.3**	**3.537**	**11.8**	**44.21**	**45.76**
**Sapph**	**(0,6,12)**	**10.69**	**71.53**	**4.079**	**3.4**	**3.765**	**12.98**	**47.06**	**48.82**
	Equiv. Refl.: (0,6,-12), (0,-6,12), (0,-6,-12), (6,0,12), (6,0,-12), (6,-6,12), (6,-6,-12), (-6,0,12), (-6,0,-12) (-6,6,12), (-6,6,-12)								
**Quartz**	**(-2,8,3)**	**11.056**	**78.78**	**3.972**	**3.3**	**2.236**	**7.47**	**27.94**	**28.93**
	Equiv. Refl.: (-2,-6,-3)								
**Quartz**	**(-6,8,-3)**	**11.056**	**78.78**	**3.971**	**3.3**	**2.236**	**7.47**	**27.94**	**28.93**
	Equiv. Refl.: (-6,-2,3)								
**Quartz**	**(8,-2,-3)**	**11.056**	**78.78**	**3.97**	**3.3**	**2.236**	**7.47**	**27.94**	**28.93**
	Equiv. Refl.: (8,-6,3)								
**Quartz**	**(8,-1,2)**	**11.237**	**85.53**	**3.957**	**4**	**0.882**	**3.53**	**11.02**	**11.57**
	Equiv. Refl.: (8,-7,-2), (-1,8,-2), (-1,-7,2)								
**Quartz**	**(-7,-1,-2)**	**11.237**	**85.53**	**3.956**	**4**	**0.882**	**3.53**	**11.02**	**11.57**
**Quartz**	**(-7,8,2)**	**11.237**	**85.53**	**3.955**	**4**	**0.882**	**3.53**	**11.02**	**11.57**
**Quartz**	**(3,4,-6)**	**11.221**	**84.59**	**3.727**	**3.5**	**1.067**	**3.72**	**13.34**	**13.85**
**Quartz**	**(4,-7,-6)**	**11.221**	**84.59**	**3.725**	**3.5**	**1.067**	**3.72**	**13.34**	**13.84**
**Quartz**	**(3,-7,6)**	**11.221**	**84.59**	**3.724**	**3.5**	**1.067**	**3.72**	**13.34**	**13.84**
	Equiv. Refl.: (4,3,6)								
**Quartz**	**(-7,3,-6)**	**11.221**	**84.59**	**3.717**	**3.5**	**1.067**	**3.71**	**13.34**	**13.84**
	Equiv. Refl.: (-7,4,6)								
**Quartz**	**(1,7,-2)**	**11.237**	**85.53**	**3.709**	**4**	**0.882**	**3.53**	**11.02**	**11.57**
	Equiv. Refl.: (1,-8,2)								
**Quartz**	**(7,1,2)**	**11.237**	**85.53**	**3.708**	**4**	**0.882**	**3.53**	**11.02**	**11.57**
	Equiv. Refl.: (-8,1,-2), (-8,7,2)								
**Quartz**	**(7,-8,-2)**	**11.237**	**85.53**	**3.707**	**4**	**0.882**	**3.53**	**11.02**	**11.57**
**Quartz**	**(2,6,3)**	**11.056**	**78.78**	**3.614**	**3.3**	**2.236**	**7.47**	**27.94**	**28.93**
**Quartz**	**(2,-8,-3)**	**11.056**	**78.78**	**3.613**	**3.3**	**2.236**	**7.47**	**27.94**	**28.93**
	Equiv. Refl.: (6,-8,3)								
**Quartz**	**(6,2,-3)**	**11.056**	**78.78**	**3.612**	**3.3**	**2.236**	**7.47**	**27.94**	**28.93**
	Equiv. Refl.: (-8,2,3)								
**Quartz**	**(-8,6,-3)**	**11.056**	**78.78**	**3.611**	**3.3**	**2.236**	**7.47**	**27.94**	**28.93**
**Quartz**	**(-1,8,-1)**	**11.06**	**78.89**	**3.581**	**3.1**	**2.214**	**6.86**	**27.68**	**28.51**
	Equiv. Refl.: (-1,-7,1), (-7,8,1), (-7,-1,-1)								
**Quartz**	**(8,-1,1)**	**11.06**	**78.89**	**3.58**	**3.1**	**2.214**	**6.86**	**27.68**	**28.51**
	Equiv. Refl.: (8,-7,-1)								
**Quartz**	**(1,7,-1)**	**11.06**	**78.89**	**3.495**	**3.1**	**2.214**	**6.86**	**27.68**	**28.51**
	Equiv. Refl.: (1,-8,1)								

Ei =	11.271	keV						ΔEg	ΔEt
Cryst	Refl	EB	ΘB	∫ IR dΘ	Width	Ei cotΘ	ΔE	2m, 50µm	2m, 50µm
	(h,k,l)	[keV]	[°]	[µrad]	[µrad]	[meV/µrad]	[meV]	[meV]	[meV]
**Quartz**	**(7,1,1)**	**11.06**	**78.89**	**3.494**	**3.1**	**2.214**	**6.86**	**27.68**	**28.51**
	Equiv. Refl.: (7,-8,-1), (-8,7,1)								
**Quartz**	**(-8,1,-1)**	**11.06**	**78.89**	**3.493**	**3.1**	**2.214**	**6.86**	**27.68**	**28.51**
**Quartz**	**(6,1,4)**	**10.599**	**70.11**	**3.44**	**2.7**	**4.078**	**11.2**	**50.97**	**52.19**
	Equiv. Refl.: (6,-7,-4)								
**Quartz**	**(1,6,-4)**	**10.599**	**70.11**	**3.439**	**2.7**	**4.078**	**11.2**	**50.97**	**52.19**
**Quartz**	**(1,-7,4)**	**10.599**	**70.11**	**3.438**	**2.7**	**4.078**	**11.2**	**50.97**	**52.19**
	Equiv. Refl.: (-7,1,-4), (-7,6,4)								
**Quartz**	**(4,4,3)**	**10.665**	**71.12**	**3.427**	**2.8**	**3.854**	**10.68**	**48.18**	**49.35**
	Equiv. Refl.: (4,-8,-3)								
**Quartz**	**(4,4,-3)**	**10.665**	**71.12**	**3.426**	**2.8**	**3.854**	**10.68**	**48.18**	**49.35**
	Equiv. Refl.: (4,-8,3), (-8,4,3), (-8,4,-3)								
**Quartz**	**(-4,8,3)**	**10.665**	**71.12**	**3.232**	**2.8**	**3.854**	**10.68**	**48.18**	**49.35**
	Equiv. Refl.: (-4,-4,-3)								
**Quartz**	**(8,-4,3)**	**10.665**	**71.12**	**3.231**	**2.8**	**3.854**	**10.68**	**48.18**	**49.35**
	Equiv. Refl.: (8,-4,-3), (-4,8,-3), (-4,-4,3)								
**Quartz**	**(-1,-6,4)**	**10.599**	**70.11**	**3.217**	**2.7**	**4.078**	**11.2**	**50.97**	**52.19**
	Equiv. Refl.: (-6,7,4), (-6,-1,-4)								
**Quartz**	**(7,-1,4)**	**10.599**	**70.11**	**3.216**	**2.7**	**4.078**	**11.2**	**50.97**	**52.19**
	Equiv. Refl.: (7,-6,-4), (-1,7,-4)								
**Sapph**	**(2,6,-1)**	**10.859**	**74.46**	**3.176**	**2.8**	**3.134**	**8.7**	**39.17**	**40.13**
	Equiv. Refl.: (2,-8,1), (6,2,1), (6,-8,-1), (8,-2,1), (8,-6,-1), (-2,8,-1), (-2,-6,1), (-6,8,1), (-6,-2,-1) (-8,2,-1), (-8,6,1)								
**Quartz**	**(-3,-4,6)**	**11.221**	**84.59**	**2.84**	**3.5**	**1.067**	**3.72**	**13.34**	**13.85**
**Quartz**	**(-4,7,6)**	**11.221**	**84.59**	**2.839**	**3.5**	**1.067**	**3.72**	**13.34**	**13.84**
**Quartz**	**(-3,7,-6)**	**11.221**	**84.59**	**2.838**	**3.5**	**1.067**	**3.72**	**13.34**	**13.84**
	Equiv. Refl.: (-4,-3,-6)								
**Quartz**	**(7,-3,6)**	**11.221**	**84.59**	**2.832**	**3.5**	**1.067**	**3.71**	**13.34**	**13.84**
**Quartz**	**(7,-4,-6)**	**11.221**	**84.59**	**2.831**	**3.5**	**1.067**	**3.71**	**13.34**	**13.84**
**Quartz**	**(3,5,3)**	**10.764**	**72.75**	**2.602**	**2.4**	**3.501**	**8.25**	**43.76**	**44.53**
	Equiv. Refl.: (3,-8,-3)								
**Quartz**	**(5,3,-3)**	**10.764**	**72.75**	**2.601**	**2.4**	**3.501**	**8.25**	**43.76**	**44.53**
	Equiv. Refl.: (5,-8,3), (-8,3,3), (-8,5,-3)								
**Quartz**	**(-3,8,3)**	**10.764**	**72.75**	**2.419**	**2.4**	**3.501**	**8.25**	**43.76**	**44.53**
**Quartz**	**(8,-3,-3)**	**10.764**	**72.75**	**2.418**	**2.4**	**3.501**	**8.25**	**43.76**	**44.53**
	Equiv. Refl.: (8,-5,3), (-3,-5,-3), (-5,8,-3), (-5,-3,3)								
**Sapph**	**(2,3,-19)**	**11.191**	**83.17**	**2.311**	**3**	**1.349**	**4.06**	**16.87**	**17.35**
	Equiv. Refl.: (2,-5,19), (3,2,19), (3,-5,-19), (5,-2,19), (5,-3,-19), (-2,5,-19), (-2,-3,19), (-3,5,19), (-3,-2,-19) (-5,2,-19), (-5,3,19)								
**LiNbO3**	**(1,6,13)**	**10.814**	**73.62**	**2.143**	**3.3**	**3.313**	**10.85**	**41.41**	**42.81**
	Equiv. Refl.: (1,-7,-13), (6,1,-13), (6,-7,13), (7,-1,-13), (7,-6,13), (-1,7,13), (-1,-6,-13), (-6,7,-13), (-6,-1,13) (-7,1,13), (-7,6,-13)								
**Quartz**	**(-6,7,5)**	**11.143**	**81.37**	**2.02**	**2.4**	**1.712**	**4.1**	**21.39**	**21.78**
**Quartz**	**(-6,-1,-5)**	**11.143**	**81.37**	**2.019**	**2.4**	**1.712**	**4.1**	**21.39**	**21.78**
**Quartz**	**(7,-6,-5)**	**11.143**	**81.37**	**2.018**	**2.4**	**1.712**	**4.09**	**21.39**	**21.78**
	Equiv. Refl.: (-1,7,-5), (-1,-6,5)								
**Quartz**	**(7,-1,5)**	**11.143**	**81.37**	**2.017**	**2.4**	**1.712**	**4.09**	**21.39**	**21.78**
**Quartz**	**(6,-7,-5)**	**11.143**	**81.37**	**1.941**	**2.4**	**1.712**	**4.1**	**21.39**	**21.78**
**Quartz**	**(1,6,-5)**	**11.143**	**81.37**	**1.94**	**2.4**	**1.712**	**4.09**	**21.39**	**21.78**
	Equiv. Refl.: (6,1,5)								

Ei =	11.271	keV						ΔEg	ΔEt
Cryst	Refl	EB	ΘB	∫ IR dΘ	Width	Ei cotΘ	ΔE	2m, 50µm	2m, 50µm
	(h,k,l)	[keV]	[°]	[µrad]	[µrad]	[meV/µrad]	[meV]	[meV]	[meV]
**Quartz**	**(1,-7,5)**	**11.143**	**81.37**	**1.939**	**2.4**	**1.712**	**4.09**	**21.39**	**21.78**
**Quartz**	**(-7,6,5)**	**11.143**	**81.37**	**1.938**	**2.4**	**1.712**	**4.09**	**21.39**	**21.78**
**Quartz**	**(-7,1,-5)**	**11.143**	**81.37**	**1.937**	**2.4**	**1.712**	**4.09**	**21.39**	**21.78**
**Quartz**	**(-4,6,-7)**	**11.131**	**80.97**	**1.853**	**2.2**	**1.791**	**3.91**	**22.39**	**22.73**
**Quartz**	**(-4,-2,7)**	**11.131**	**80.97**	**1.852**	**2.2**	**1.791**	**3.91**	**22.39**	**22.73**
**Quartz**	**(6,-2,-7)**	**11.131**	**80.97**	**1.851**	**2.2**	**1.791**	**3.91**	**22.39**	**22.73**
**Quartz**	**(6,-4,7)**	**11.131**	**80.97**	**1.85**	**2.2**	**1.791**	**3.91**	**22.39**	**22.73**
**Quartz**	**(-2,6,7)**	**11.131**	**80.97**	**1.848**	**2.2**	**1.791**	**3.91**	**22.39**	**22.73**
	Equiv. Refl.: (-2,-4,-7)								
**Quartz**	**(1,1,-9)**	**10.627**	**70.54**	**1.8**	**1.9**	**3.982**	**7.53**	**49.78**	**50.35**
**Quartz**	**(1,-2,9)**	**10.627**	**70.54**	**1.799**	**1.9**	**3.982**	**7.53**	**49.78**	**50.35**
**Quartz**	**(-2,1,9)**	**10.627**	**70.54**	**1.795**	**1.9**	**3.982**	**7.52**	**49.78**	**50.34**
	Equiv. Refl.: (-2,1,-9)								
**Quartz**	**(1,1,9)**	**10.627**	**70.54**	**1.794**	**1.9**	**3.982**	**7.51**	**49.78**	**50.34**
	Equiv. Refl.: (1,-2,-9)								
**Quartz**	**(6,-5,-6)**	**10.638**	**70.71**	**1.792**	**1.9**	**3.945**	**7.63**	**49.31**	**49.9**
**Quartz**	**(6,-1,6)**	**10.638**	**70.71**	**1.791**	**1.9**	**3.945**	**7.63**	**49.31**	**49.9**
	Equiv. Refl.: (-1,6,-6), (-1,-5,6), (-5,6,6), (-5,-1,-6)								
**Quartz**	**(5,1,6)**	**10.638**	**70.71**	**1.743**	**1.9**	**3.945**	**7.63**	**49.31**	**49.9**
	Equiv. Refl.: (5,-6,-6), (-6,1,-6), (-6,5,6)								
**Quartz**	**(1,5,-6)**	**10.638**	**70.71**	**1.742**	**1.9**	**3.945**	**7.62**	**49.31**	**49.9**
	Equiv. Refl.: (1,-6,6)								
**Quartz**	**(4,2,-7)**	**11.131**	**80.97**	**1.724**	**2.2**	**1.791**	**3.91**	**22.39**	**22.73**
	Equiv. Refl.: (4,-6,7)								
**Quartz**	**(-6,2,7)**	**11.131**	**80.97**	**1.723**	**2.2**	**1.791**	**3.91**	**22.39**	**22.73**
**Quartz**	**(5,2,5)**	**10.756**	**72.6**	**1.722**	**1.8**	**3.531**	**6.5**	**44.14**	**44.62**
	Equiv. Refl.: (5,-7,-5), (-6,4,-7)								
**Quartz**	**(-7,2,-5)**	**10.756**	**72.6**	**1.721**	**1.8**	**3.531**	**6.5**	**44.14**	**44.62**
	Equiv. Refl.: (-7,5,5)								
**Quartz**	**(2,4,7)**	**11.131**	**80.97**	**1.72**	**2.2**	**1.791**	**3.91**	**22.39**	**22.73**
	Equiv. Refl.: (2,5,-5), (2,-6,-7), (2,-7,5)								
**Quartz**	**(0,7,-4)**	**11.183**	**82.85**	**1.681**	**2.2**	**1.414**	**3.09**	**17.67**	**17.94**
	Equiv. Refl.: (0,-7,4)								
**Quartz**	**(7,0,4)**	**11.183**	**82.85**	**1.675**	**2.2**	**1.414**	**3.09**	**17.67**	**17.94**
	Equiv. Refl.: (7,-7,-4), (-7,0,-4), (-7,7,4)								
**Quartz**	**(-1,2,-9)**	**10.627**	**70.54**	**1.651**	**1.9**	**3.982**	**7.53**	**49.78**	**50.35**
	Equiv. Refl.: (-1,-1,9)								
**Quartz**	**(2,-1,9)**	**10.627**	**70.54**	**1.647**	**1.9**	**3.982**	**7.52**	**49.78**	**50.34**
**Quartz**	**(2,-1,-9)**	**10.627**	**70.54**	**1.646**	**1.9**	**3.982**	**7.52**	**49.78**	**50.34**
	Equiv. Refl.: (-1,2,9)								
**Quartz**	**(-1,-1,-9)**	**10.627**	**70.54**	**1.645**	**1.9**	**3.982**	**7.51**	**49.78**	**50.34**
**Quartz**	**(-3,8,4)**	**11.183**	**82.85**	**1.62**	**2**	**1.414**	**2.86**	**17.67**	**17.9**
**Quartz**	**(8,-3,-4)**	**11.183**	**82.85**	**1.619**	**2**	**1.414**	**2.86**	**17.67**	**17.9**
	Equiv. Refl.: (8,-5,4), (-3,-5,-4), (-5,8,-4)								
**Quartz**	**(-5,-3,4)**	**11.183**	**82.85**	**1.618**	**2**	**1.414**	**2.86**	**17.67**	**17.9**
**Quartz**	**(-5,7,5)**	**10.756**	**72.6**	**1.581**	**1.8**	**3.531**	**6.51**	**44.14**	**44.62**
	Equiv. Refl.: (-5,-2,-5)								
**Quartz**	**(7,-2,5)**	**10.756**	**72.6**	**1.579**	**1.8**	**3.531**	**6.5**	**44.14**	**44.62**
	Equiv. Refl.: (7,-5,-5)								

Ei =	11.271	keV						ΔEg	ΔEt
Cryst	Refl	EB	ΘB	∫ IR dΘ	Width	Ei cotΘ	ΔE	2m, 50µm	2m, 50µm
	(h,k,l)	[keV]	[°]	[µrad]	[µrad]	[meV/µrad]	[meV]	[meV]	[meV]
**Quartz**	**(-2,7,-5)**	**10.756**	**72.6**	**1.578**	**1.8**	**3.531**	**6.5**	**44.14**	**44.62**
	Equiv. Refl.: (-2,-5,5)								
**Sapph**	**(3,5,7)**	**11.048**	**78.59**	**1.544**	**2**	**2.275**	**4.48**	**28.43**	**28.79**
	Equiv. Refl.: (3,-8,-7), (5,3,-7), (5,-8,7), (8,-3,-7), (8,-5,7), (-3,8,7), (-3,-5,-7), (-5,8,-7), (-5,-3,7) (-8,3,7), (-8,5,-7)								
**Quartz**	**(0,2,9)**	**10.727**	**72.12**	**1.496**	**1.7**	**3.636**	**6.14**	**45.45**	**45.87**
	Equiv. Refl.: (0,-2,-9)								
**Quartz**	**(2,0,-9)**	**10.727**	**72.12**	**1.495**	**1.7**	**3.636**	**6.13**	**45.45**	**45.87**
	Equiv. Refl.: (2,-2,9), (-2,0,9), (-2,2,-9)								
**Quartz**	**(1,2,-9)**	**11.019**	**77.87**	**1.481**	**1.7**	**2.422**	**4.05**	**30.28**	**30.55**
	Equiv. Refl.: (2,-3,-9)								
**Quartz**	**(1,-3,9)**	**11.019**	**77.87**	**1.48**	**1.7**	**2.422**	**4.05**	**30.28**	**30.55**
**Quartz**	**(2,1,9)**	**11.019**	**77.87**	**1.479**	**1.7**	**2.422**	**4.04**	**30.28**	**30.55**
**Quartz**	**(-3,1,-9)**	**11.019**	**77.87**	**1.476**	**1.7**	**2.422**	**4.04**	**30.28**	**30.55**
	Equiv. Refl.: (-3,2,9)								
**Quartz**	**(3,5,4)**	**11.183**	**82.85**	**1.424**	**2**	**1.414**	**2.86**	**17.67**	**17.9**
	Equiv. Refl.: (3,-8,-4), (-8,3,4)								
**Quartz**	**(5,-8,4)**	**11.183**	**82.85**	**1.423**	**2**	**1.414**	**2.86**	**17.67**	**17.9**
	Equiv. Refl.: (-8,5,-4)								
**Sapph**	**(1,6,-11)**	**11.175**	**82.53**	**1.422**	**2.1**	**1.479**	**3.03**	**18.48**	**18.73**
	Equiv. Refl.: (1,-7,11), (6,1,11), (6,-7,-11), (7,-1,11), (7,-6,-11), (-1,7,-11), (-1,-6,11), (-6,7,11), (-6,-1,-11) (-7,1,-11), (-7,6,11), (5,3,-4)								
**Quartz**	**(2,6,-2)**	**10.754**	**72.58**	**1.379**	**1.5**	**3.537**	**5.29**	**44.21**	**44.52**
	Equiv. Refl.: (2,-8,2), (6,2,2), (6,-8,-2), (-8,2,-2), (-8,6,2)								
**Quartz**	**(-1,-2,9)**	**11.019**	**77.87**	**1.267**	**1.7**	**2.422**	**4.05**	**30.28**	**30.55**
**Quartz**	**(-1,3,-9)**	**11.019**	**77.87**	**1.266**	**1.7**	**2.422**	**4.05**	**30.28**	**30.55**
	Equiv. Refl.: (-2,3,9)								
**Quartz**	**(-2,-1,-9)**	**11.019**	**77.87**	**1.265**	**1.7**	**2.422**	**4.04**	**30.28**	**30.55**
**Quartz**	**(3,-1,9)**	**11.019**	**77.87**	**1.263**	**1.7**	**2.422**	**4.04**	**30.28**	**30.55**
	Equiv. Refl.: (3,-2,-9)								
**LiNbO3**	**(4,5,5)**	**11.088**	**79.66**	**1.253**	**2.7**	**2.057**	**5.55**	**25.71**	**26.31**
	Equiv. Refl.: (4,-9,-5), (5,4,-5), (5,-9,5), (9,-4,-5), (9,-5,5), (-4,9,5), (-4,-5,-5), (-5,9,-5), (-5,-4,5) (-9,4,5), (-9,5,-5)								
**Quartz**	**(8,-2,2)**	**10.754**	**72.58**	**1.201**	**1.5**	**3.537**	**5.29**	**44.21**	**44.52**
	Equiv. Refl.: (8,-6,-2), (-2,8,-2), (-2,-6,2)								
**Quartz**	**(-6,8,2)**	**10.754**	**72.58**	**1.2**	**1.5**	**3.537**	**5.29**	**44.21**	**44.52**
	Equiv. Refl.: (-6,-2,-2)								
**Quartz**	**(1,7,2)**	**11.237**	**85.53**	**1.185**	**1.9**	**0.882**	**1.68**	**11.02**	**11.15**
**Quartz**	**(1,-8,-2)**	**11.237**	**85.53**	**1.184**	**1.9**	**0.882**	**1.67**	**11.02**	**11.15**
**Quartz**	**(-8,1,2)**	**11.237**	**85.53**	**1.181**	**1.9**	**0.882**	**1.67**	**11.02**	**11.15**
	Equiv. Refl.: (-8,7,-2)								
**Quartz**	**(7,1,-2)**	**11.237**	**85.53**	**1.18**	**1.9**	**0.882**	**1.67**	**11.02**	**11.15**
	Equiv. Refl.: (7,-8,2)								
**Sapph**	**(3,5,-5)**	**10.798**	**73.34**	**1.142**	**1.4**	**3.373**	**4.86**	**42.16**	**42.44**
	Equiv. Refl.: (3,-8,5), (5,3,5), (5,-8,-5), (8,-3,5), (8,-5,-5), (-3,8,-5), (-3,-5,5), (-5,8,5), (-5,-3,-5) (-8,3,-5), (-8,5,5)								
**Quartz**	**(-1,8,2)**	**11.237**	**85.53**	**1.137**	**1.9**	**0.882**	**1.67**	**11.02**	**11.15**
	Equiv. Refl.: (-1,-7,-2)								
**Quartz**	**(8,-1,-2)**	**11.237**	**85.53**	**1.133**	**1.9**	**0.882**	**1.67**	**11.02**	**11.15**
	Equiv. Refl.: (8,-7,2), (-7,8,-2), (-7,-1,2)								

**Ei =**	**11.271**	**keV**						**ΔEg**	**ΔEt**
**Cryst**	**Refl**	**EB**	**ΘB**	**∫ IR dΘ**	**Width**	**Ei cotΘ**	**ΔE**	**2m, 50µm**	**2m, 50µm**
	**(h,k,l)**	**[keV]**	**[°]**	**[µrad]**	**[µrad]**	**[meV/µrad]**	**[meV]**	**[meV]**	**[meV]**
**Quartz**	**(0,7,3)**	**10.764**	**72.75**	**1.057**	**1.3**	**3.501**	**4.46**	**43.76**	**43.99**
	Equiv. Refl.: (0,-7,-3), (7,0,-3), (7,-7,3), (-7,0,3), (-7,7,-3)								
**Quartz**	**(0,3,9)**	**11.21**	**84.06**	**1.055**	**1.6**	**1.174**	**1.92**	**14.67**	**14.79**
	Equiv. Refl.: (0,-3,-9)								
**Quartz**	**(3,-3,9)**	**11.21**	**84.06**	**1.05**	**1.6**	**1.174**	**1.91**	**14.67**	**14.79**
**Quartz**	**(3,0,-9)**	**11.21**	**84.06**	**1.049**	**1.6**	**1.174**	**1.91**	**14.67**	**14.79**
	Equiv. Refl.: (-3,3,-9)								
**Quartz**	**(-3,0,9)**	**11.21**	**84.06**	**1.048**	**1.6**	**1.174**	**1.91**	**14.67**	**14.79**
**Quartz**	**(0,6,6)**	**11.126**	**80.79**	**1.011**	**1.4**	**1.827**	**2.55**	**22.83**	**22.97**
	Equiv. Refl.: (0,-6,-6)								
**Quartz**	**(6,0,-6)**	**11.126**	**80.79**	**1.005**	**1.4**	**1.827**	**2.54**	**22.83**	**22.97**
	Equiv. Refl.: (6,-6,6), (-6,0,6), (-6,6,-6)								
**Quartz**	**(0,7,-3)**	**10.764**	**72.75**	**0.969**	**1.2**	**3.501**	**4.16**	**43.76**	**43.96**
	Equiv. Refl.: (0,-7,3), (7,0,3), (7,-7,-3), (-7,0,-3), (-7,7,3)								
**LiNbO3**	**(4,5,-1)**	**10.869**	**74.66**	**0.904**	**1.9**	**3.093**	**5.92**	**38.66**	**39.11**
	Equiv. Refl.: (4,-9,1), (5,4,1), (5,-9,-1), (9,-4,1), (9,-5,-1), (-4,9,-1), (-4,-5,1), (-5,9,1), (-5,-4,-1) (-9,4,-1), (-9,5,1)								
**LiNbO3**	**(1,7,9)**	**11.243**	**85.96**	**0.869**	**3.3**	**0.796**	**2.66**	**9.96**	**10.3**
	Equiv. Refl.: (1,7,-9), (1,-8,9), (1,-8,-9), (7,1,9), (7,1,-9), (7,-8,9), (7,-8,-9), (8,-1,9), (8,-1,-9) (8,-7,9), (8,-7,-9), (-1,8,9), (-1,8,-9), (-1,-7,9), (-1,-7,-9), (-7,8,9), (-7,8,-9), (-7,-1,9) (-7,-1,-9), (-8,1,9), (-8,1,-9), (-8,7,9), (-8,7,-9)								
**Quartz**	**(-7,1,4)**	**10.599**	**70.11**	**0.864**	**1**	**4.078**	**4.28**	**50.97**	**51.15**
**Quartz**	**(6,1,-4)**	**10.599**	**70.11**	**0.863**	**1**	**4.078**	**4.28**	**50.97**	**51.15**
	Equiv. Refl.: (6,-7,4), (-7,6,-4)								
**Quartz**	**(1,6,4)**	**10.599**	**70.11**	**0.862**	**1**	**4.078**	**4.27**	**50.97**	**51.15**
	Equiv. Refl.: (1,-7,-4)								
**Quartz**	**(7,-1,-4)**	**10.599**	**70.11**	**0.849**	**1**	**4.078**	**4.28**	**50.97**	**51.15**
	Equiv. Refl.: (7,-6,4), (-6,7,-4), (-6,-1,4)								
**Quartz**	**(-1,7,4)**	**10.599**	**70.11**	**0.848**	**1**	**4.078**	**4.27**	**50.97**	**51.15**
	Equiv. Refl.: (-1,-6,-4)								
**Sapph**	**(1,4,18)**	**11.015**	**77.77**	**0.839**	**1.2**	**2.442**	**2.99**	**30.53**	**30.67**
	Equiv. Refl.: (1,4,-18), (1,-5,18), (1,-5,-18), (4,1,18), (4,1,-18), (4,-5,18), (4,-5,-18), (5,-1,18), (5,-1,-18) (5,-4,18), (5,-4,-18), (-1,5,18), (-1,5,-18), (-1,-4,18), (-1,-4,-18), (-4,5,18), (-4,5,-18), (-4,-1,18) (-4,-1,-18), (-5,1,18), (-5,1,-18), (-5,4,18), (-5,4,-18)								
**Quartz**	**(3,2,-8)**	**11.16**	**81.94**	**0.806**	**1.2**	**1.597**	**1.89**	**19.96**	**20.05**
**Quartz**	**(3,-5,8)**	**11.16**	**81.94**	**0.805**	**1.2**	**1.597**	**1.89**	**19.96**	**20.05**
**Quartz**	**(2,3,8)**	**11.16**	**81.94**	**0.804**	**1.2**	**1.597**	**1.89**	**19.96**	**20.05**
**Quartz**	**(2,-5,-8)**	**11.16**	**81.94**	**0.803**	**1.2**	**1.597**	**1.89**	**19.96**	**20.05**
**Quartz**	**(-5,3,-8)**	**11.16**	**81.94**	**0.796**	**1.2**	**1.597**	**1.88**	**19.96**	**20.05**
**Quartz**	**(-5,2,8)**	**11.16**	**81.94**	**0.795**	**1.2**	**1.597**	**1.87**	**19.96**	**20.05**
**Quartz**	**(1,-3,-9)**	**11.019**	**77.87**	**0.792**	**1.1**	**2.422**	**2.62**	**30.28**	**30.39**
**Quartz**	**(1,2,9)**	**11.019**	**77.87**	**0.791**	**1.1**	**2.422**	**2.62**	**30.28**	**30.39**
**Quartz**	**(-3,1,9)**	**11.019**	**77.87**	**0.79**	**1.1**	**2.422**	**2.61**	**30.28**	**30.39**
	Equiv. Refl.: (-3,2,-9)								
**Quartz**	**(2,1,-9)**	**11.019**	**77.87**	**0.788**	**1.1**	**2.422**	**2.61**	**30.28**	**30.39**
	Equiv. Refl.: (2,-3,9)								
**Quartz**	**(-1,3,9)**	**11.019**	**77.87**	**0.766**	**1.1**	**2.422**	**2.62**	**30.28**	**30.39**
**Quartz**	**(-1,-2,-9)**	**11.019**	**77.87**	**0.765**	**1.1**	**2.422**	**2.62**	**30.28**	**30.39**
**Quartz**	**(3,-1,-9)**	**11.019**	**77.87**	**0.764**	**1.1**	**2.422**	**2.61**	**30.28**	**30.39**
	Equiv. Refl.: (3,-2,9)								
**Quartz**	**(-2,3,-9)**	**11.019**	**77.87**	**0.763**	**1.1**	**2.422**	**2.61**	**30.28**	**30.39**

Ei =	11.271	keV						ΔEg	ΔEt
Cryst	Refl	EB	ΘB	∫ IR dΘ	Width	Ei cotΘ	ΔE	2m, 50µm	2m, 50µm
	(h,k,l)	[keV]	[°]	[µrad]	[µrad]	[meV/µrad]	[meV]	[meV]	[meV]
**Quartz**	**(-2,-1,9)**	**11.019**	**77.87**	**0.762**	**1.1**	**2.422**	**2.61**	**30.28**	**30.39**
**Sapph**	**(2,5,12)**	**11.003**	**77.49**	**0.752**	**1.1**	**2.501**	**2.83**	**31.27**	**31.4**
	Equiv. Refl.: (2,5,-12), (2,-7,12), (2,-7,-12), (5,2,12), (5,2,-12), (5,-7,12), (5,-7,-12), (7,-2,12), (7,-2,-12) (7,-5,12), (7,-5,-12), (-2,7,12), (-2,7,-12), (-2,-5,12), (-2,-5,-12), (-5,7,12), (-5,7,-12), (-5,-2,12) (-5,-2,-12), (-7,2,12), (-7,2,-12), (-7,5,12), (-7,5,-12)								
**LiNbO3**	**(3,6,3)**	**11.118**	**80.55**	**0.74**	**2.1**	**1.877**	**3.89**	**23.46**	**23.78**
	Equiv. Refl.: (3,6,-3), (3,-9,3), (3,-9,-3), (6,3,3), (6,3,-3), (6,-9,3), (6,-9,-3), (9,-3,3), (9,-3,-3) (9,-6,3), (9,-6,-3), (-3,9,3), (-3,9,-3), (-3,-6,3), (-3,-6,-3), (-6,9,3), (-6,9,-3), (-6,-3,3) (-6,-3,-3), (-9,3,3), (-9,3,-3), (-9,6,3), (-9,6,-3)								
**LiNbO3**	**(1,5,17)**	**10.85**	**74.3**	**0.711**	**1.6**	**3.169**	**5.22**	**39.61**	**39.96**
	Equiv. Refl.: (1,-6,-17), (5,1,-17), (5,-6,17), (6,-1,-17), (6,-5,17), (-1,6,17), (-1,-5,-17), (-5,6,-17), (-5,-1,17) (-6,1,17), (-6,5,-17)								
**Quartz**	**(-3,5,-8)**	**11.16**	**81.94**	**0.707**	**1.2**	**1.597**	**1.89**	**19.96**	**20.05**
	Equiv. Refl.: (-3,-2,8)								
**LiNbO3**	**(2,6,11)**	**11.169**	**82.27**	**0.706**	**2.2**	**1.53**	**3.38**	**19.13**	**19.43**
	Equiv. Refl.: (2,-8,-11), (6,2,-11), (6,-8,11), (8,-2,-11), (8,-6,11), (-2,8,11), (-2,-6,-11), (-6,8,-11), (-6,-2,11) (-8,2,11), (-8,6,-11), (-2,-3,-8)								
**Quartz**	**(-2,5,8)**	**11.16**	**81.94**	**0.705**	**1.2**	**1.597**	**1.89**	**19.96**	**20.05**
**Quartz**	**(5,-2,-8)**	**11.16**	**81.94**	**0.697**	**1.2**	**1.597**	**1.87**	**19.96**	**20.05**
	Equiv. Refl.: (5,-3,8)								
**LiNbO3**	**(1,2,23)**	**10.923**	**75.73**	**0.691**	**1.7**	**2.867**	**4.82**	**35.83**	**36.16**
	Equiv. Refl.: (1,-3,-23), (2,1,-23), (2,-3,23), (3,-1,-23), (3,-2,23), (-1,3,23), (-1,-2,-23), (-2,3,-23), (-2,-1,23) (-3,1,23), (-3,2,-23)								
**Sapph**	**(0,7,-4)**	**10.703**	**71.73**	**0.615**	**0.9**	**3.722**	**3.28**	**46.52**	**46.64**
	Equiv. Refl.: (0,-7,4), (7,0,4), (7,-7,-4), (-7,0,-4), (-7,7,4)								
**Sapph**	**(3,5,-8)**	**11.202**	**83.65**	**0.584**	**1.2**	**1.255**	**1.49**	**15.68**	**15.76**
	Equiv. Refl.: (3,-8,8), (5,3,8), (5,-8,-8), (8,-3,8), (8,-5,-8), (-3,8,-8), (-3,-5,8), (-5,8,8), (-5,-3,-8) (-8,3,-8), (-8,5,8)								
**Quartz**	**(5,0,7)**	**10.841**	**74.13**	**0.56**	**0.8**	**3.204**	**2.55**	**40.05**	**40.13**
	Equiv. Refl.: (5,-5,-7), (-5,0,-7), (-5,5,7)								
**Quartz**	**(0,5,-7)**	**10.841**	**74.13**	**0.554**	**0.8**	**3.204**	**2.53**	**40.05**	**40.13**
	Equiv. Refl.: (0,-5,7)								
**Quartz**	**(4,0,8)**	**10.87**	**74.68**	**0.547**	**0.8**	**3.088**	**2.43**	**38.6**	**38.67**
	Equiv. Refl.: (4,-4,-8), (-4,0,-8), (-4,4,8)								
**Quartz**	**(-2,7,5)**	**10.756**	**72.6**	**0.545**	**0.7**	**3.531**	**2.59**	**44.14**	**44.22**
	Equiv. Refl.: (-2,-5,-5)								
**Quartz**	**(7,-2,-5)**	**10.756**	**72.6**	**0.543**	**0.7**	**3.531**	**2.58**	**44.14**	**44.21**
	Equiv. Refl.: (7,-5,5), (-5,7,-5), (-5,-2,5)								
**Quartz**	**(0,4,-8)**	**10.87**	**74.68**	**0.54**	**0.8**	**3.088**	**2.41**	**38.6**	**38.67**
	Equiv. Refl.: (0,-4,8)								
**Quartz**	**(2,5,5)**	**10.756**	**72.6**	**0.487**	**0.7**	**3.531**	**2.59**	**44.14**	**44.22**
	Equiv. Refl.: (2,-7,-5)								
**Quartz**	**(5,2,-5)**	**10.756**	**72.6**	**0.485**	**0.7**	**3.531**	**2.58**	**44.14**	**44.21**
	Equiv. Refl.: (5,-7,5), (-7,2,5), (-7,5,-5)								
**Sapph**	**(0,2,22)**	**10.923**	**75.73**	**0.477**	**0.8**	**2.868**	**2.29**	**35.85**	**35.92**
	Equiv. Refl.: (0,-2,-22), (2,0,-22), (2,-2,22), (-2,0,22), (-2,2,-22)								
**LiNbO3**	**(2,5,15)**	**10.973**	**76.79**	**0.465**	**1.4**	**2.647**	**3.68**	**33.08**	**33.29**
	Equiv. Refl.: (2,5,-15), (2,-7,15), (2,-7,-15), (5,2,15), (5,2,-15), (5,-7,15), (5,-7,-15), (7,-2,15), (7,-2,-15) (7,-5,15), (7,-5,-15), (-2,7,15), (-2,7,-15), (-2,-5,15), (-2,-5,-15), (-5,7,15), (-5,7,-15), (-5,-2,15) (-5,-2,-15), (-7,2,15), (-7,2,-15), (-7,5,15), (-7,5,-15)								
**Quartz**	**(3,3,-7)**	**11.036**	**78.27**	**0.406**	**0.6**	**2.341**	**1.41**	**29.26**	**29.29**
**Quartz**	**(3,-6,7)**	**11.036**	**78.27**	**0.404**	**0.6**	**2.341**	**1.41**	**29.26**	**29.29**

**Quartz**	**(-6,3,7)**	**11.036**	**78.27**	**0.403**	**0.6**	**2.341**	**1.41**	**29.26**	**29.29**
	Equiv. Refl.: (-6,3,-7)								
**Quartz**	**(3,3,7)**	**11.036**	**78.27**	**0.401**	**0.6**	**2.341**	**1.4**	**29.26**	**29.29**
	Equiv. Refl.: (3,-6,-7)								
**Sapph**	**(1,2,-22)**	**11.229**	**85.08**	**0.275**	**0.9**	**0.97**	**0.85**	**12.12**	**12.15**
	Equiv. Refl.: (1,-3,22), (2,1,22), (2,-3,-22), (3,-1,22), (3,-2,-22), (-1,3,-22), (-1,-2,22), (-2,3,22), (-2,-1,-22) (-3,1,-22), (-3,2,22)								
**Quartz**	**(-3,-3,7)**	**11.036**	**78.27**	**0.266**	**0.6**	**2.341**	**1.41**	**29.26**	**29.29**
**Quartz**	**(6,-3,7)**	**11.036**	**78.27**	**0.265**	**0.6**	**2.341**	**1.41**	**29.26**	**29.29**
	Equiv. Refl.: (6,-3,-7), (-3,6,-7)								
**Quartz**	**(-3,6,7)**	**11.036**	**78.27**	**0.262**	**0.6**	**2.341**	**1.4**	**29.26**	**29.29**
	Equiv. Refl.: (-3,-3,-7)								
**Sapph**	**(3,4,-13)**	**11.056**	**78.8**	**0.247**	**0.6**	**2.232**	**1.29**	**27.9**	**27.93**
	Equiv. Refl.: (3,-7,13), (4,3,13), (4,-7,-13), (7,-3,13), (7,-4,-13), (-3,7,-13), (-3,-4,13), (-4,7,13), (-4,-3,-13) (-7,3,-13), (-7,4,13)								
**Sapph**	**(0,1,-22)**	**10.608**	**70.24**	**0.223**	**0.4**	**4.048**	**1.79**	**50.6**	**50.63**
	Equiv. Refl.: (0,-1,22), (1,0,22), (1,-1,-22), (-1,0,-22), (-1,1,22)								
**LiNbO3**	**(3,5,-11)**	**10.906**	**75.37**	**0.22**	**0.9**	**2.941**	**2.65**	**36.77**	**36.86**
	Equiv. Refl.: (3,-8,11), (5,3,11), (5,-8,-11), (8,-3,11), (8,-5,-11), (-3,8,-11), (-3,-5,11), (-5,8,11), (-5,-3,-11) (-8,3,-11), (-8,5,11)								
**Sapph**	**(2,6,2)**	**10.89**	**75.07**	**0.126**	**0.3**	**3.006**	**1.05**	**37.57**	**37.58**
	Equiv. Refl.: (2,-8,-2), (6,2,-2), (6,-8,2), (8,-2,-2), (8,-6,2), (-2,8,2), (-2,-6,-2), (-6,8,-2), (-6,-2,2) (-8,2,2), (-8,6,-2)								
**Sapph**	**(3,3,15)**	**10.6**	**70.13**	**0.035**	**0.2**	**4.073**	**0.65**	**50.91**	**50.91**
	Equiv. Refl.: (3,3,-15), (3,-6,15), (3,-6,-15), (6,-3,15), (6,-3,-15), (-3,6,15), (-3,6,-15), (-3,-3,15), (-3,-3,-15) (-6,3,15), (-6,3,-15)								

Ei =	10.739	keV						ΔEg	ΔEt
Cryst	Refl	EB	ΘB	∫ IR dΘ	Width	Ei cotΘ	ΔE	2m, 50µm	2m, 50µm
	(h,k,l)	[keV]	[°]	[µrad]	[µrad]	[meV/µrad]	[meV]	[meV]	[meV]
**Ge**	**(4,4,8)**	**10.736**	**88.74**	**103.256**	**102.1**	**0.2361**	**24.11**	**2.95**	**24.29**
	Equiv. Refl.: (4,8,4), (8,4,4)								
**Si**	**(4,6,6)**	**10.708**	**85.62**	**22.8931**	**20.37**	**0.8217**	**16.74**	**10.27**	**19.64**
	Equiv. Refl.: (6,4,6), (6,6,4)								
**Ge**	**(4,6,6)**	**10.279**	**73.18**	**9.5623**	**9**	**3.2473**	**29.23**	**40.59**	**50.02**
	Equiv. Refl.: (6,4,6), (6,6,4)								
**Ge**	**(1,3,9)**	**10.453**	**76.75**	**7.3024**	**7.59**	**2.5289**	**19.2**	**31.61**	**36.99**
	Equiv. Refl.: (1,9,3), (3,1,9), (3,9,1), (9,1,3), (9,3,1)								
**Si**	**(0,4,8)**	**10.209**	**71.93**	**6.8166**	**5.78**	**3.5036**	**20.27**	**43.8**	**48.26**
	Equiv. Refl.: (0,8,4), (4,0,8), (4,8,0), (8,0,4), (8,4,0)								
**Si**	**(1,1,9)**	**10.399**	**75.55**	**5.4206**	**4.81**	**2.7683**	**13.32**	**34.6**	**37.08**
	Equiv. Refl.: (1,9,1), (3,5,7), (3,7,5), (5,3,7), (5,7,3), (7,3,5), (7,5,3), (9,1,1)								
**LiNbO3**	**(0,0,-24)**	**10.732**	**87.97**	**61.175**	**58.7**	**0.381**	**22.38**	**4.77**	**22.88**
**LiNbO3**	**(0,7,-10)**	**10.711**	**85.9**	**55.12**	**50.7**	**0.77**	**39.04**	**9.63**	**40.21**
	Equiv. Refl.: (-7,0,-10)								
**LiNbO3**	**(7,-7,-10)**	**10.711**	**85.9**	**55.119**	**50.7**	**0.77**	**39.04**	**9.63**	**40.21**
**LiNbO3**	**(0,-7,10)**	**10.711**	**85.9**	**54.516**	**50.7**	**0.77**	**39.04**	**9.63**	**40.21**
	Equiv. Refl.: (7,0,10)								
**LiNbO3**	**(-7,7,10)**	**10.711**	**85.9**	**54.515**	**50.7**	**0.77**	**39.04**	**9.63**	**40.21**
**LiNbO3**	**(0,0,24)**	**10.732**	**87.97**	**52.932**	**58.7**	**0.381**	**22.38**	**4.77**	**22.88**
**LiNbO3**	**(3,5,10)**	**10.711**	**85.9**	**46.377**	**42**	**0.77**	**32.36**	**9.63**	**33.76**
	Equiv. Refl.: (5,-8,10), (8,-5,10), (-3,8,10), (-5,-3,10), (-8,3,10)								
**LiNbO3**	**(3,-8,-10)**	**10.711**	**85.9**	**41.592**	**42**	**0.77**	**32.36**	**9.63**	**33.76**
	Equiv. Refl.: (5,3,-10), (8,-3,-10), (-3,-5,-10), (-5,8,-10), (-8,5,-10)								
**Sapph**	**(1,5,14)**	**10.715**	**86.17**	**30.637**	**26.2**	**0.719**	**18.86**	**8.98**	**20.89**
	Equiv. Refl.: (1,-6,-14), (5,1,-14), (5,-6,14), (6,-1,-14), (6,-5,14), (-1,6,14), (-1,-5,-14), (-5,6,-14), (-5,-1,14) (-6,1,14), (-6,5,-14)								
**LiNbO3**	**(2,-8,-8)**	**10.646**	**82.45**	**28.447**	**26.2**	**1.422**	**37.32**	**17.78**	**41.34**
	Equiv. Refl.: (6,2,-8), (8,-2,-8), (-2,-6,-8), (-6,8,-8), (-8,6,-8)								
**LiNbO3**	**(2,6,8)**	**10.646**	**82.45**	**27.764**	**26.2**	**1.422**	**37.32**	**17.78**	**41.34**
	Equiv. Refl.: (6,-8,8), (8,-6,8), (-2,8,8), (-6,-2,8), (-8,2,8)								
**Sapph**	**(0,5,16)**	**10.719**	**86.52**	**27.034**	**23.8**	**0.653**	**15.52**	**8.17**	**17.54**
	Equiv. Refl.: (0,-5,-16), (5,0,-16), (5,-5,16), (-5,0,16), (-5,5,-16)								
**Quartz**	**(0,2,-9)**	**10.727**	**87.24**	**26.085**	**22.8**	**0.517**	**11.82**	**6.47**	**13.47**
	Equiv. Refl.: (0,-2,9)								
**Quartz**	**(-2,0,-9)**	**10.727**	**87.24**	**26.076**	**22.8**	**0.517**	**11.82**	**6.47**	**13.47**
**Quartz**	**(-2,2,9)**	**10.727**	**87.24**	**26.073**	**22.8**	**0.517**	**11.81**	**6.47**	**13.47**
**Quartz**	**(2,0,9)**	**10.727**	**87.24**	**26.071**	**22.8**	**0.517**	**11.82**	**6.47**	**13.47**
**Quartz**	**(2,-2,-9)**	**10.727**	**87.24**	**26.069**	**22.8**	**0.517**	**11.81**	**6.47**	**13.47**
**Sapph**	**(3,5,4)**	**10.703**	**85.28**	**25.133**	**21.5**	**0.886**	**19.07**	**11.08**	**22.06**
	Equiv. Refl.: (3,-8,-4), (5,3,-4), (5,-8,4), (8,-3,-4), (8,-5,4), (-3,8,4), (-3,-5,-4), (-5,8,-4), (-5,-3,4) (-8,3,4), (-8,5,-4)								
**LiNbO3**	**(4,-4,20)**	**10.532**	**78.73**	**20.79**	**19.1**	**2.14**	**40.96**	**26.75**	**48.92**
**LiNbO3**	**(0,4,20)**	**10.532**	**78.73**	**20.789**	**19.1**	**2.14**	**40.96**	**26.75**	**48.92**
	Equiv. Refl.: (-4,0,20)								
**LiNbO3**	**(-4,4,-20)**	**10.532**	**78.73**	**20.705**	**19.1**	**2.14**	**40.96**	**26.75**	**48.92**
**LiNbO3**	**(0,-4,-20)**	**10.532**	**78.73**	**20.704**	**19.1**	**2.14**	**40.96**	**26.75**	**48.92**
	Equiv. Refl.: (4,0,-20)								

Ei =	10.739	keV						ΔEg	ΔEt
**Cryst**	**Refl**	**EB**	**ΘB**	**∫ IR dΘ**	**Width**	**Ei cotΘ**	**ΔE**	**2m, 50µm**	**2m, 50µm**
	**(h,k,l)**	**[keV]**	**[°]**	**[µrad]**	**[µrad]**	**[meV/µrad]**	**[meV]**	**[meV]**	**[meV]**
**LiNbO3**	**(-1,6,-16)**	**10.542**	**79**	**18.995**	**17.7**	**2.087**	**37.02**	**26.09**	**45.29**
	Equiv. Refl.: (-6,1,-16)								
**LiNbO3**	**(1,5,-16)**	**10.542**	**79**	**18.994**	**17.7**	**2.087**	**37.02**	**26.09**	**45.29**
	Equiv. Refl.: (5,-6,-16), (6,-5,-16), (-5,-1,-16)								
**LiNbO3**	**(1,-6,16)**	**10.542**	**79**	**18.839**	**17.7**	**2.087**	**37.02**	**26.09**	**45.29**
	Equiv. Refl.: (5,1,16), (6,-1,16), (-1,-5,16), (-5,6,16), (-6,5,16)								
**LiNbO3**	**(3,5,-8)**	**10.37**	**74.94**	**17.783**	**15.8**	**2.89**	**45.66**	**36.13**	**58.22**
	Equiv. Refl.: (5,-8,-8), (8,-5,-8), (-3,8,-8), (-5,-3,-8), (-8,3,-8)								
**LiNbO3**	**(1,-3,22)**	**10.503**	**77.97**	**17.067**	**15.9**	**2.288**	**36.41**	**28.6**	**46.3**
	Equiv. Refl.: (2,1,22), (3,-1,22), (-1,-2,22)								
**LiNbO3**	**(-2,3,22)**	**10.503**	**77.97**	**17.066**	**15.9**	**2.288**	**36.41**	**28.6**	**46.3**
	Equiv. Refl.: (-3,2,22)								
**LiNbO3**	**(4,3,-14)**	**10.523**	**78.49**	**17.055**	**15.8**	**2.188**	**34.52**	**27.34**	**44.04**
	Equiv. Refl.: (-3,-4,-14), (-4,7,-14), (-7,4,-14)								
**LiNbO3**	**(3,-7,-14)**	**10.523**	**78.49**	**17.054**	**15.8**	**2.188**	**34.52**	**27.34**	**44.04**
	Equiv. Refl.: (7,-3,-14)								
**LiNbO3**	**(1,-8,0)**	**10.498**	**77.84**	**16.978**	**15.8**	**2.315**	**36.64**	**28.93**	**46.69**
	Equiv. Refl.: (7,1,0), (8,-1,0), (-1,-7,0), (-7,8,0), (-8,7,0)								
**LiNbO3**	**(3,-8,8)**	**10.37**	**74.94**	**16.866**	**15.8**	**2.89**	**45.66**	**36.13**	**58.22**
	Equiv. Refl.: (5,3,8), (8,-3,8), (-3,-5,8), (-5,8,8), (-8,5,8)								
**LiNbO3**	**(1,2,-22)**	**10.503**	**77.97**	**16.722**	**15.9**	**2.288**	**36.41**	**28.6**	**46.3**
	Equiv. Refl.: (2,-3,-22), (3,-2,-22), (-1,3,-22), (-2,-1,-22), (-3,1,-22)								
**LiNbO3**	**(1,7,0)**	**10.498**	**77.84**	**16.668**	**15.8**	**2.315**	**36.64**	**28.93**	**46.69**
	Equiv. Refl.: (7,-8,0), (8,-7,0), (-1,8,0), (-7,-1,0), (-8,1,0)								
**LiNbO3**	**(3,4,14)**	**10.523**	**78.49**	**16.158**	**15.8**	**2.188**	**34.52**	**27.34**	**44.04**
	Equiv. Refl.: (4,-7,14), (7,-4,14), (-3,7,14), (-4,-3,14), (-7,3,14)								
**LiNbO3**	**(0,7,8)**	**10.37**	**74.94**	**15.345**	**13.9**	**2.89**	**40.3**	**36.13**	**54.13**
	Equiv. Refl.: (7,-7,8), (-7,0,8)								
**LiNbO3**	**(0,-7,-8)**	**10.37**	**74.94**	**14.649**	**13.9**	**2.89**	**40.3**	**36.13**	**54.13**
	Equiv. Refl.: (7,0,-8), (-7,7,-8)								
**LiNbO3**	**(0,2,22)**	**10.223**	**72.17**	**14.218**	**12.8**	**3.453**	**44.34**	**43.17**	**61.88**
	Equiv. Refl.: (2,-2,22), (-2,0,22)								
**LiNbO3**	**(1,-4,20)**	**10.253**	**72.69**	**14.062**	**12.9**	**3.346**	**43.13**	**41.82**	**60.08**
	Equiv. Refl.: (3,1,20), (4,-1,20), (-1,-3,20), (-3,4,20), (-4,3,20)								
**LiNbO3**	**(2,6,-4)**	**10.185**	**71.52**	**13.93**	**12.7**	**3.589**	**45.73**	**44.86**	**64.06**
	Equiv. Refl.: (6,-8,-4), (8,-6,-4), (-2,8,-4), (-6,-2,-4), (-8,2,-4)								
**LiNbO3**	**(1,3,-20)**	**10.253**	**72.69**	**13.869**	**12.9**	**3.346**	**43.13**	**41.82**	**60.08**
	Equiv. Refl.: (3,-4,-20), (4,-3,-20), (-1,4,-20), (-3,-1,-20), (-4,1,-20)								
**LiNbO3**	**(2,-8,4)**	**10.185**	**71.52**	**13.867**	**12.7**	**3.589**	**45.73**	**44.86**	**64.06**
	Equiv. Refl.: (6,2,4), (8,-2,4), (-2,-6,4), (-6,8,4), (-8,6,4)								
**LiNbO3**	**(1,-7,-10)**	**10.156**	**71.03**	**13.764**	**12.3**	**3.692**	**45.43**	**46.15**	**64.76**
	Equiv. Refl.: (6,1,-10), (7,-1,-10), (-1,-6,-10), (-6,7,-10), (-7,6,-10)								
**LiNbO3**	**(0,-2,-22)**	**10.223**	**72.17**	**13.694**	**12.8**	**3.453**	**44.34**	**43.17**	**61.88**
	Equiv. Refl.: (2,0,-22), (-2,2,-22)								
**LiNbO3**	**(2,4,16)**	**10.263**	**72.88**	**13.596**	**12.4**	**3.309**	**41.07**	**41.36**	**58.29**
	Equiv. Refl.: (4,-6,16), (6,-4,16), (-4,-2,16)								
**LiNbO3**	**(-2,6,16)**	**10.263**	**72.88**	**13.595**	**12.4**	**3.309**	**41.07**	**41.36**	**58.29**
	Equiv. Refl.: (-6,2,16)								

Ei =	10.739	keV						ΔEg	ΔEt
Cryst	Refl (h,k,l)	EB [keV]	ΘB [°]	∫ IR dΘ [µrad]	Width [µrad]	Ei cotΘ [meV/µrad]	ΔE [meV]	2m, 50µm [meV]	2m, 50µm [meV]
Sapph	(0,6,12)	10.69	84.54	13.387	12.1	1.026	12.39	12.83	17.83
	Equiv. Refl.: (0,6,-12), (0,-6,12), (0,-6,-12), (6,0,12), (6,0,-12), (6,-6,12), (6,-6,-12), (-6,0,12), (-6,0,-12) (-6,6,12), (-6,6,-12)								
LiNbO3	(2,-6,-16)	10.263	72.88	13.097	12.4	3.309	41.07	41.36	58.29
	Equiv. Refl.: (4,2,-16), (6,-2,-16), (-2,-4,-16), (-4,6,-16), (-6,4,-16)								
LiNbO3	(1,6,10)	10.156	71.03	13.026	12.3	3.692	45.43	46.15	64.76
	Equiv. Refl.: (6,-7,10), (7,-6,10), (-1,7,10), (-6,-1,10), (-7,1,10)								
LiNbO3	(1,-5,-18)	10.266	72.93	12.583	11.6	3.297	38.37	41.21	56.31
	Equiv. Refl.: (4,1,-18), (5,-1,-18), (-1,-4,-18), (-4,5,-18), (-5,4,-18)								
LiNbO3	(1,4,-18)	10.266	72.93	12.225	11.4	3.297	37.49	41.21	55.72
	Equiv. Refl.: (4,-5,-18), (5,-4,-18), (-1,5,-18), (-4,-1,-18), (-5,1,-18)								
LiNbO3	(1,4,18)	10.266	72.93	12.154	11.6	3.297	38.37	41.21	56.31
	Equiv. Refl.: (4,-5,18), (5,-4,18), (-1,5,18), (-4,-1,18), (-5,1,18)								
LiNbO3	(2,-7,12)	10.208	71.9	12.059	11.3	3.509	39.57	43.86	59.07
	Equiv. Refl.: (5,2,12), (7,-2,12), (-2,-5,12), (-5,7,12), (-7,5,12)								
LiNbO3	(2,5,-12)	10.208	71.9	11.992	11.3	3.509	39.57	43.86	59.07
	Equiv. Refl.: (5,-7,-12), (7,-5,-12), (-2,7,-12), (-5,-2,-12), (-7,2,-12)								
LiNbO3	(1,-5,18)	10.266	72.93	11.849	11.4	3.297	37.49	41.21	55.72
	Equiv. Refl.: (4,1,18), (5,-1,18), (-1,-4,18), (-4,5,18), (-5,4,18), (0,2,9), (0,-2,-9)								
Quartz	(2,-2,9)	10.727	87.24	11.845	11.4	0.517	5.87	6.47	8.74
Quartz	(2,0,-9)	10.727	87.24	11.844	11.3	0.517	5.87	6.47	8.74
Quartz	(-2,2,-9)	10.727	87.24	11.843	11.4	0.517	5.87	6.47	8.74
Quartz	(-2,0,9)	10.727	87.24	11.84	11.3	0.517	5.87	6.47	8.74
LiNbO3	(2,5,12)	10.208	71.9	11.71	10.8	3.509	37.92	43.86	57.98
	Equiv. Refl.: (5,-7,12), (7,-5,12), (-5,-2,12)								
LiNbO3	(-2,7,12)	10.208	71.9	11.709	10.8	3.509	37.92	43.86	57.98
	Equiv. Refl.: (-7,2,12)								
LiNbO3	(5,2,-12)	10.208	71.9	11.673	10.8	3.509	37.92	43.86	57.98
	Equiv. Refl.: (-2,-5,-12), (-5,7,-12), (-7,5,-12)								
LiNbO3	(2,-7,-12)	10.208	71.9	11.672	10.8	3.509	37.92	43.86	57.98
	Equiv. Refl.: (7,-2,-12)								
Quartz	(6,-1,-6)	10.638	82.14	11.382	9.5	1.482	14.03	18.53	23.24
Quartz	(6,-5,6)	10.638	82.14	11.381	9.5	1.482	14.03	18.53	23.24
	Equiv. Refl.: (-1,6,6)								
Quartz	(-1,-5,-6)	10.638	82.14	11.379	9.5	1.482	14.03	18.53	23.24
	Equiv. Refl.: (-5,6,-6), (-5,-1,6)								
Quartz	(-6,1,6)	10.638	82.14	11.337	9.5	1.482	14.03	18.53	23.24
Quartz	(1,-6,-6)	10.638	82.14	11.336	9.5	1.482	14.03	18.53	23.24
	Equiv. Refl.: (-6,5,-6)								
Quartz	(1,5,6)	10.638	82.14	11.335	9.5	1.482	14.03	18.53	23.24
Quartz	(5,1,-6)	10.638	82.14	11.334	9.5	1.482	14.03	18.53	23.24
	Equiv. Refl.: (5,-6,6)								
Quartz	(0,0,9)	10.323	74	10.942	8.9	3.079	27.38	38.48	47.23
	Equiv. Refl.: (0,0,-9)								
Sapph	(3,4,-10)	10.321	73.96	10.877	9	3.087	27.86	38.59	47.6
	Equiv. Refl.: (3,-7,10), (4,3,10), (4,-7,-10), (7,-3,10), (7,-4,-10), (-3,7,-10), (-3,-4,10), (-4,7,10), (-4,-3,-10) (-7,3,-10), (-7,4,10)								
Sapph	(0,7,2)	10.574	79.95	9.846	8.5	1.904	16.19	23.8	28.78
	Equiv. Refl.: (0,-7,-2), (7,0,-2), (7,-7,2), (-7,0,2), (-7,7,-2)								
Quartz	(4,4,3)	10.665	83.26	9.371	8	1.27	10.2	15.87	18.87
	Equiv. Refl.: (4,-8,-3)								
Quartz	(-8,4,-3)	10.665	83.26	9.37	8	1.27	10.2	15.87	18.87

Ei =	10.739	keV						ΔEg	ΔEt
Cryst	Refl	EB	ΘB	∫ IR dΘ	Width	Ei cotΘ	ΔE	2m, 50µm	2m, 50µm
	(h,k,l)	[keV]	[°]	[µrad]	[µrad]	[meV/µrad]	[meV]	[meV]	[meV]
**Quartz**	**(4,4,-3)**	**10.665**	**83.26**	**9.369**	**8**	**1.27**	**10.2**	**15.87**	**18.87**
	Equiv. Refl.: (4,-8,3)								
**Quartz**	**(-8,4,3)**	**10.665**	**83.26**	**9.368**	**8**	**1.27**	**10.2**	**15.87**	**18.87**
**Quartz**	**(-4,8,3)**	**10.665**	**83.26**	**8.79**	**8**	**1.27**	**10.2**	**15.87**	**18.87**
	Equiv. Refl.: (-4,-4,-3)								
**Quartz**	**(8,-4,3)**	**10.665**	**83.26**	**8.789**	**8**	**1.27**	**10.2**	**15.87**	**18.87**
**Quartz**	**(-4,8,-3)**	**10.665**	**83.26**	**8.788**	**8**	**1.27**	**10.2**	**15.87**	**18.87**
	Equiv. Refl.: (-4,-4,3)								
**Quartz**	**(8,-4,-3)**	**10.665**	**83.26**	**8.787**	**8**	**1.27**	**10.2**	**15.87**	**18.87**
**Quartz**	**(8,-2,-1)**	**10.569**	**79.79**	**8.288**	**7**	**1.934**	**13.53**	**24.18**	**27.71**
	Equiv. Refl.: (8,-6,1), (-2,8,1), (-2,-6,-1), (-6,8,-1), (-6,-2,1)								
**Quartz**	**(2,6,1)**	**10.569**	**79.79**	**8.194**	**7**	**1.934**	**13.53**	**24.18**	**27.71**
	Equiv. Refl.: (2,-8,-1), (6,2,-1), (6,-8,1), (-8,2,1), (-8,6,-1)								
**Quartz**	**(0,3,-8)**	**10.164**	**71.17**	**7.91**	**6.2**	**3.663**	**22.78**	**45.79**	**51.14**
	Equiv. Refl.: (0,-3,8), (3,0,8), (3,-3,-8), (-3,0,-8), (-3,3,8)								
**Sapph**	**(2,4,-14)**	**10.393**	**75.42**	**7.277**	**6.2**	**2.792**	**17.21**	**34.91**	**38.92**
	Equiv. Refl.: (2,-6,14), (4,2,14), (4,-6,-14), (6,-2,14), (6,-4,-14), (-2,6,-14), (-2,-4,14), (-4,6,14), (-4,-2,-14) (-6,2,-14), (-6,4,14)								
**Quartz**	**(6,1,4)**	**10.599**	**80.73**	**7.228**	**6.1**	**1.754**	**10.7**	**21.92**	**24.39**
	Equiv. Refl.: (6,-7,-4)								
**Quartz**	**(1,6,-4)**	**10.599**	**80.73**	**7.226**	**6.1**	**1.754**	**10.7**	**21.92**	**24.39**
**Quartz**	**(1,-7,4)**	**10.599**	**80.73**	**7.225**	**6.1**	**1.754**	**10.7**	**21.92**	**24.39**
	Equiv. Refl.: (-7,1,-4), (-7,6,4)								
**Quartz**	**(-6,7,4)**	**10.599**	**80.73**	**6.72**	**6.1**	**1.754**	**10.7**	**21.92**	**24.39**
**Quartz**	**(-6,-1,-4)**	**10.599**	**80.73**	**6.719**	**6.1**	**1.754**	**10.7**	**21.92**	**24.39**
**Quartz**	**(-1,-6,4)**	**10.599**	**80.73**	**6.718**	**6.1**	**1.754**	**10.7**	**21.92**	**24.39**
**Quartz**	**(7,-1,4)**	**10.599**	**80.73**	**6.717**	**6.1**	**1.754**	**10.7**	**21.92**	**24.39**
	Equiv. Refl.: (-1,7,-4)								
**Quartz**	**(7,-6,-4)**	**10.599**	**80.73**	**6.716**	**6.1**	**1.754**	**10.69**	**21.92**	**24.39**
**Quartz**	**(6,0,-5)**	**10.455**	**76.8**	**6.677**	**5.6**	**2.519**	**14.09**	**31.49**	**34.49**
	Equiv. Refl.: (-6,0,5)								
**Quartz**	**(0,6,5)**	**10.455**	**76.8**	**6.676**	**5.6**	**2.519**	**14.09**	**31.49**	**34.49**
	Equiv. Refl.: (0,-6,-5), (6,-6,5), (-6,6,-5)								
**Sapph**	**(1,2,20)**	**10.342**	**74.38**	**6.607**	**5.6**	**3.002**	**16.84**	**37.53**	**41.13**
	Equiv. Refl.: (1,-3,-20), (2,1,-20), (2,-3,20), (3,-1,-20), (3,-2,20), (-1,3,20), (-1,-2,-20), (-2,3,-20), (-2,-1,20) (-3,1,20), (-3,2,-20)								
**Sapph**	**(4,4,0)**	**10.423**	**76.06**	**6.493**	**5.6**	**2.665**	**14.9**	**33.31**	**36.49**
	Equiv. Refl.: (4,-8,0), (8,-4,0), (-4,8,0), (-4,-4,0), (-8,4,0)								
**Sapph**	**(1,6,-8)**	**10.578**	**80.08**	**6.448**	**5.9**	**1.879**	**11.15**	**23.49**	**26**
	Equiv. Refl.: (1,-7,8), (6,1,8), (6,-7,-8), (7,-1,8), (7,-6,-8), (-1,7,-8), (-1,-6,8), (-6,7,8), (-6,-1,-8) (-7,1,-8), (-7,6,8)								
**Quartz**	**(7,-3,-5)**	**10.556**	**79.42**	**6.167**	**5.2**	**2.007**	**10.47**	**25.08**	**27.18**
	Equiv. Refl.: (7,-4,5), (-4,7,-5)								
**Quartz**	**(-3,7,5)**	**10.556**	**79.42**	**6.165**	**5.2**	**2.007**	**10.47**	**25.08**	**27.18**
	Equiv. Refl.: (-3,-4,-5), (-4,-3,5)								
**Quartz**	**(4,-1,8)**	**10.573**	**79.93**	**6.054**	**5**	**1.908**	**9.63**	**23.85**	**25.72**
	Equiv. Refl.: (4,-3,-8)								
**Quartz**	**(-3,-1,-8)**	**10.573**	**79.93**	**6.051**	**5**	**1.908**	**9.63**	**23.85**	**25.72**
**Quartz**	**(-3,4,8)**	**10.573**	**79.93**	**6.048**	**5**	**1.908**	**9.62**	**23.85**	**25.71**

Ei =	10.739	keV						ΔEg	ΔEt
Cryst	Refl	EB	ΘB	∫ IR dΘ	Width	Ei cotΘ	ΔE	2m, 50µm	2m, 50µm
	(h,k,l)	[keV]	[°]	[µrad]	[µrad]	[meV/µrad]	[meV]	[meV]	[meV]
**Quartz**	**(-1,4,-8)**	**10.573**	**79.93**	**6.047**	**5**	**1.908**	**9.62**	**23.85**	**25.71**
	Equiv. Refl.: (-1,-3,8)								
**Quartz**	**(0,7,1)**	**10.263**	**72.88**	**6.045**	**4.9**	**3.308**	**16.28**	**41.35**	**44.44**
	Equiv. Refl.: (0,-7,-1), (7,0,-1), (7,-7,1), (-7,0,1), (-7,7,-1)								
**Quartz**	**(4,-7,5)**	**10.556**	**79.42**	**5.831**	**5.2**	**2.007**	**10.47**	**25.08**	**27.18**
	Equiv. Refl.: (-7,3,5)								
**Quartz**	**(4,3,-5)**	**10.556**	**79.42**	**5.83**	**5.2**	**2.007**	**10.47**	**25.08**	**27.18**
	Equiv. Refl.: (-7,4,-5)								
**Quartz**	**(3,4,5)**	**10.556**	**79.42**	**5.828**	**5.2**	**2.007**	**10.47**	**25.08**	**27.18**
	Equiv. Refl.: (3,-7,-5)								
**Quartz**	**(-4,1,-8)**	**10.573**	**79.93**	**5.688**	**5**	**1.908**	**9.63**	**23.85**	**25.72**
	Equiv. Refl.: (-4,3,8)								
**Quartz**	**(3,1,8)**	**10.573**	**79.93**	**5.686**	**5**	**1.908**	**9.63**	**23.85**	**25.72**
**Quartz**	**(3,-4,-8)**	**10.573**	**79.93**	**5.683**	**5**	**1.908**	**9.62**	**23.85**	**25.71**
**Quartz**	**(1,3,-8)**	**10.573**	**79.93**	**5.682**	**5**	**1.908**	**9.62**	**23.85**	**25.71**
	Equiv. Refl.: (1,-4,8)								
**Quartz**	**(6,-5,-6)**	**10.638**	**82.14**	**5.522**	**4.9**	**1.482**	**7.3**	**18.53**	**19.91**
**Quartz**	**(6,-1,6)**	**10.638**	**82.14**	**5.521**	**4.9**	**1.482**	**7.29**	**18.53**	**19.91**
**Quartz**	**(-5,6,6)**	**10.638**	**82.14**	**5.52**	**4.9**	**1.482**	**7.29**	**18.53**	**19.91**
	Equiv. Refl.: (-5,-1,-6)								
**Quartz**	**(-1,-5,6)**	**10.638**	**82.14**	**5.518**	**4.9**	**1.482**	**7.29**	**18.53**	**19.91**
**Quartz**	**(-1,6,-6)**	**10.638**	**82.14**	**5.517**	**4.9**	**1.482**	**7.29**	**18.53**	**19.91**
**Sapph**	**(1,1,21)**	**10.356**	**74.66**	**5.459**	**4.7**	**2.946**	**13.73**	**36.83**	**39.31**
	Equiv. Refl.: (1,1,-21), (1,-2,21), (1,-2,-21), (2,-1,21), (2,-1,-21), (-1,2,21), (-1,2,-21), (-1,-1,21), (-1,-1,-21) (-2,1,21), (-2,1,-21)								
**Quartz**	**(-6,5,6)**	**10.638**	**82.14**	**5.358**	**4.9**	**1.482**	**7.3**	**18.53**	**19.91**
**Quartz**	**(5,1,6)**	**10.638**	**82.14**	**5.357**	**4.9**	**1.482**	**7.29**	**18.53**	**19.91**
	Equiv. Refl.: (5,-6,-6), (-6,1,-6)								
**Quartz**	**(1,5,-6)**	**10.638**	**82.14**	**5.355**	**4.9**	**1.482**	**7.29**	**18.53**	**19.91**
**Quartz**	**(1,-6,6)**	**10.638**	**82.14**	**5.354**	**4.9**	**1.482**	**7.29**	**18.53**	**19.91**
**Quartz**	**(4,4,2)**	**10.352**	**74.57**	**5.312**	**4.3**	**2.965**	**12.82**	**37.06**	**39.22**
	Equiv. Refl.: (4,4,-2), (4,-8,2), (4,-8,-2)								
**Quartz**	**(-8,4,2)**	**10.352**	**74.57**	**5.311**	**4.3**	**2.965**	**12.82**	**37.06**	**39.22**
	Equiv. Refl.: (-8,4,-2)								
**Quartz**	**(4,2,6)**	**10.335**	**74.22**	**5.28**	**4.2**	**3.034**	**12.76**	**37.92**	**40.01**
	Equiv. Refl.: (4,-6,-6)								
**Quartz**	**(-6,2,-6)**	**10.335**	**74.22**	**5.278**	**4.2**	**3.034**	**12.76**	**37.92**	**40.01**
**Quartz**	**(-6,4,6)**	**10.335**	**74.22**	**5.276**	**4.2**	**3.034**	**12.75**	**37.92**	**40.01**
**Quartz**	**(2,4,-6)**	**10.335**	**74.22**	**5.275**	**4.2**	**3.034**	**12.75**	**37.92**	**40.01**
	Equiv. Refl.: (2,-6,6)								
**Quartz**	**(1,1,-9)**	**10.627**	**81.72**	**5.264**	**4.6**	**1.562**	**7.2**	**19.53**	**20.81**
**Quartz**	**(1,-2,9)**	**10.627**	**81.72**	**5.262**	**4.6**	**1.562**	**7.19**	**19.53**	**20.81**
**Quartz**	**(8,-2,1)**	**10.569**	**79.79**	**5.257**	**4.5**	**1.934**	**8.72**	**24.18**	**25.7**
	Equiv. Refl.: (8,-6,-1)								
**Quartz**	**(-2,8,-1)**	**10.569**	**79.79**	**5.256**	**4.5**	**1.934**	**8.72**	**24.18**	**25.7**
	Equiv. Refl.: (-2,-6,1), (-6,8,1), (-6,-2,-1)								
**Quartz**	**(-2,1,-9)**	**10.627**	**81.72**	**5.249**	**4.6**	**1.562**	**7.18**	**19.53**	**20.81**
	Equiv. Refl.: (-4,-4,2)								
**Quartz**	**(-4,8,2)**	**10.352**	**74.57**	**5.248**	**4.3**	**2.965**	**12.82**	**37.06**	**39.22**
	Equiv. Refl.: (-4,8,-2), (-4,-4,-2)								

Ei =	10.739	keV						ΔEg	ΔEt
Cryst	Refl	EB	ΘB	∫ IR dΘ	Width	Ei cotΘ	ΔE	2m, 50µm	2m, 50µm
	(h,k,l)	[keV]	[°]	[µrad]	[µrad]	[meV/µrad]	[meV]	[meV]	[meV]
**Quartz**	**(8,-4,2)**	**10.352**	**74.57**	**5.247**	**4.3**	**2.965**	**12.82**	**37.06**	**39.22**
	Equiv. Refl.: (8,-4,-2), (-2,1,9)								
**Quartz**	**(1,-2,-9)**	**10.627**	**81.72**	**5.245**	**4.6**	**1.562**	**7.17**	**19.53**	**20.8**
**Quartz**	**(1,1,9)**	**10.627**	**81.72**	**5.244**	**4.6**	**1.562**	**7.17**	**19.53**	**20.8**
**Quartz**	**(5,3,2)**	**10.454**	**76.76**	**5.153**	**4.3**	**2.526**	**10.95**	**31.57**	**33.42**
	Equiv. Refl.: (5,-8,-2)								
**Quartz**	**(3,5,-2)**	**10.454**	**76.76**	**5.151**	**4.3**	**2.526**	**10.95**	**31.57**	**33.42**
	Equiv. Refl.: (3,-8,2), (-8,3,-2), (-8,5,2)								
**Quartz**	**(0,7,0)**	**10.199**	**71.75**	**5.132**	**4.2**	**3.541**	**14.7**	**44.26**	**46.64**
	Equiv. Refl.: (0,-7,0), (7,0,0), (7,-7,0), (-7,0,0), (-7,7,0)								
**Quartz**	**(-5,8,2)**	**10.454**	**76.76**	**5.128**	**4.3**	**2.526**	**10.95**	**31.57**	**33.42**
	Equiv. Refl.: (-5,-3,-2)								
**Quartz**	**(8,-3,2)**	**10.454**	**76.76**	**5.126**	**4.3**	**2.526**	**10.95**	**31.57**	**33.42**
	Equiv. Refl.: (8,-5,-2), (-3,8,-2), (-3,-5,2)								
**Quartz**	**(2,6,-1)**	**10.569**	**79.79**	**5.103**	**4.5**	**1.934**	**8.72**	**24.18**	**25.7**
	Equiv. Refl.: (2,-8,1), (6,2,1), (6,-8,-1), (-8,2,-1), (-8,6,1)								
**Quartz**	**(-4,6,6)**	**10.335**	**74.22**	**4.98**	**4.2**	**3.034**	**12.76**	**37.92**	**40.01**
	Equiv. Refl.: (-4,-2,-6)								
**Quartz**	**(6,-2,6)**	**10.335**	**74.22**	**4.978**	**4.2**	**3.034**	**12.76**	**37.92**	**40.01**
**Quartz**	**(6,-4,-6)**	**10.335**	**74.22**	**4.976**	**4.2**	**3.034**	**12.75**	**37.92**	**40.01**
**Quartz**	**(-2,6,-6)**	**10.335**	**74.22**	**4.975**	**4.2**	**3.034**	**12.75**	**37.92**	**40.01**
	Equiv. Refl.: (-2,-4,6)								
**Quartz**	**(-1,-1,9)**	**10.627**	**81.72**	**4.791**	**4.6**	**1.562**	**7.2**	**19.53**	**20.81**
**Quartz**	**(-1,2,-9)**	**10.627**	**81.72**	**4.79**	**4.6**	**1.562**	**7.19**	**19.53**	**20.81**
**Quartz**	**(2,-1,9)**	**10.627**	**81.72**	**4.777**	**4.6**	**1.562**	**7.18**	**19.53**	**20.81**
**Quartz**	**(2,-1,-9)**	**10.627**	**81.72**	**4.775**	**4.6**	**1.562**	**7.18**	**19.53**	**20.8**
**Quartz**	**(-1,2,9)**	**10.627**	**81.72**	**4.774**	**4.6**	**1.562**	**7.17**	**19.53**	**20.8**
**Quartz**	**(-1,-1,-9)**	**10.627**	**81.72**	**4.772**	**4.6**	**1.562**	**7.17**	**19.53**	**20.8**
**Quartz**	**(8,-4,0)**	**10.094**	**70.05**	**4.201**	**3.3**	**3.899**	**13**	**48.74**	**50.44**
	Equiv. Refl.: (-4,8,0), (-4,-4,0)								
**Sapph**	**(4,4,3)**	**10.521**	**78.43**	**4.125**	**3.9**	**2.198**	**8.57**	**27.48**	**28.78**
	Equiv. Refl.: (4,4,-3), (4,-8,3), (4,-8,-3), (8,-4,3), (8,-4,-3), (-4,8,3), (-4,8,-3), (-4,-4,3), (-4,-4,-3) (-8,4,3), (-8,4,-3)								
**Quartz**	**(-2,-4,-6)**	**10.335**	**74.22**	**4.049**	**3.3**	**3.034**	**9.96**	**37.92**	**39.21**
**Quartz**	**(6,-2,-6)**	**10.335**	**74.22**	**4.048**	**3.3**	**3.034**	**9.96**	**37.92**	**39.21**
	Equiv. Refl.: (-2,6,6)								
**Quartz**	**(6,-4,6)**	**10.335**	**74.22**	**4.047**	**3.3**	**3.034**	**9.95**	**37.92**	**39.21**
**Quartz**	**(-4,6,-6)**	**10.335**	**74.22**	**4.045**	**3.3**	**3.034**	**9.95**	**37.92**	**39.21**
**Quartz**	**(-4,-2,6)**	**10.335**	**74.22**	**4.044**	**3.3**	**3.034**	**9.95**	**37.92**	**39.21**
**Quartz**	**(4,4,0)**	**10.094**	**70.05**	**3.859**	**3.3**	**3.899**	**13**	**48.74**	**50.44**
	Equiv. Refl.: (4,-8,0), (-8,4,0)								
**Quartz**	**(2,4,6)**	**10.335**	**74.22**	**3.579**	**3.3**	**3.034**	**9.96**	**37.92**	**39.21**
	Equiv. Refl.: (-6,2,6)								
**Quartz**	**(2,-6,-6)**	**10.335**	**74.22**	**3.578**	**3.3**	**3.034**	**9.96**	**37.92**	**39.21**
	Equiv. Refl.: (-6,4,-6)								
**Quartz**	**(4,-6,6)**	**10.335**	**74.22**	**3.576**	**3.3**	**3.034**	**9.95**	**37.92**	**39.21**
**Quartz**	**(4,2,-6)**	**10.335**	**74.22**	**3.575**	**3.3**	**3.034**	**9.95**	**37.92**	**39.21**
**Quartz**	**(5,-1,-7)**	**10.443**	**76.51**	**3.566**	**3.2**	**2.577**	**8.15**	**32.21**	**33.23**
**Quartz**	**(5,-4,7)**	**10.443**	**76.51**	**3.565**	**3.2**	**2.577**	**8.14**	**32.21**	**33.23**
**Quartz**	**(-5,8,-2)**	**10.454**	**76.76**	**3.562**	**3.1**	**2.526**	**7.73**	**31.57**	**32.51**
	Equiv. Refl.: (-5,-3,2)								

Ei =	10.739	keV						ΔEg	ΔEt
Cryst	Refl	EB	ΘB	∫ IR dΘ	Width	Ei cotΘ	ΔE	2m, 50µm	2m, 50µm
	(h,k,l)	[keV]	[°]	[µrad]	[µrad]	[meV/µrad]	[meV]	[meV]	[meV]
**Quartz**	**(8,-3,-2)**	**10.454**	**76.76**	**3.559**	**3.1**	**2.526**	**7.73**	**31.57**	**32.51**
	Equiv. Refl.: (8,-5,2), (-3,-5,-2)								
**Quartz**	**(-3,8,2)**	**10.454**	**76.76**	**3.558**	**3.1**	**2.526**	**7.73**	**31.57**	**32.51**
**Quartz**	**(0,1,9)**	**10.425**	**76.12**	**3.556**	**3.2**	**2.653**	**8.41**	**33.17**	**34.22**
	Equiv. Refl.: (0,-1,-9), (-4,5,-7), (-4,-1,7)								
**Quartz**	**(-1,-4,-7)**	**10.443**	**76.51**	**3.555**	**3.2**	**2.577**	**8.12**	**32.21**	**33.22**
**Quartz**	**(-1,5,7)**	**10.443**	**76.51**	**3.554**	**3.2**	**2.577**	**8.12**	**32.21**	**33.22**
**Quartz**	**(1,0,-9)**	**10.425**	**76.12**	**3.544**	**3.2**	**2.653**	**8.39**	**33.17**	**34.21**
	Equiv. Refl.: (1,-1,9), (-1,0,9), (-1,1,-9)								
**Quartz**	**(5,3,-2)**	**10.454**	**76.76**	**3.537**	**3.1**	**2.526**	**7.73**	**31.57**	**32.51**
	Equiv. Refl.: (5,-8,2)								
**Quartz**	**(3,5,2)**	**10.454**	**76.76**	**3.534**	**3.1**	**2.526**	**7.73**	**31.57**	**32.51**
	Equiv. Refl.: (3,-8,-2), (-8,3,2), (-8,5,-2)								
**Quartz**	**(3,-6,-6)**	**10.231**	**72.31**	**3.514**	**3.2**	**3.425**	**11.12**	**42.81**	**44.23**
**Quartz**	**(3,3,6)**	**10.231**	**72.31**	**3.513**	**3.2**	**3.425**	**11.12**	**42.81**	**44.23**
	Equiv. Refl.: (3,3,-6), (3,-6,6), (-6,3,6)								
**Quartz**	**(-6,3,-6)**	**10.231**	**72.31**	**3.512**	**3.2**	**3.425**	**11.12**	**42.81**	**44.23**
**Quartz**	**(6,-3,-6)**	**10.231**	**72.31**	**3.508**	**3.2**	**3.425**	**11.12**	**42.81**	**44.23**
	Equiv. Refl.: (-3,6,6)								
**Quartz**	**(6,-3,6)**	**10.231**	**72.31**	**3.507**	**3.2**	**3.425**	**11.12**	**42.81**	**44.23**
	Equiv. Refl.: (-3,6,-6), (-3,-3,6), (-3,-3,-6)								
**Sapph**	**(1,5,-13)**	**10.424**	**76.09**	**3.487**	**3.2**	**2.66**	**8.47**	**33.25**	**34.31**
	Equiv. Refl.: (1,-6,13), (5,1,13), (5,-6,-13), (6,-1,13), (6,-5,-13), (-1,6,-13), (-1,-5,13), (-5,6,13), (-5,-1,-13) (-6,1,-13), (-6,5,13)								
**Quartz**	**(-5,1,7)**	**10.443**	**76.51**	**3.477**	**3.2**	**2.577**	**8.15**	**32.21**	**33.23**
**Quartz**	**(-5,4,-7)**	**10.443**	**76.51**	**3.476**	**3.2**	**2.577**	**8.14**	**32.21**	**33.23**
**Quartz**	**(4,1,-7)**	**10.443**	**76.51**	**3.468**	**3.2**	**2.577**	**8.13**	**32.21**	**33.22**
**Quartz**	**(4,-5,7)**	**10.443**	**76.51**	**3.467**	**3.2**	**2.577**	**8.13**	**32.21**	**33.22**
**Quartz**	**(1,4,7)**	**10.443**	**76.51**	**3.466**	**3.2**	**2.577**	**8.12**	**32.21**	**33.22**
**Quartz**	**(1,-5,-7)**	**10.443**	**76.51**	**3.465**	**3.2**	**2.577**	**8.12**	**32.21**	**33.22**
**Sapph**	**(2,5,9)**	**10.331**	**74.15**	**3.25**	**2.9**	**3.05**	**8.95**	**38.12**	**39.16**
	Equiv. Refl.: (2,5,-9), (2,-7,9), (2,-7,-9), (5,2,9), (5,2,-9), (5,-7,9), (5,-7,-9), (7,-2,9), (7,-2,-9) (7,-5,9), (7,-5,-9), (-2,7,9), (-2,7,-9), (-2,-5,9), (-2,-5,-9), (-5,7,9), (-5,7,-9), (-5,-2,9) (-5,-2,-9), (-7,2,9), (-7,2,-9), (-7,5,9), (-7,5,-9)								
**Quartz**	**(8,-3,0)**	**10.199**	**71.75**	**3.173**	**2.9**	**3.541**	**10.22**	**44.26**	**45.43**
	Equiv. Refl.: (8,-5,0), (-3,8,0), (-3,-5,0), (-5,8,0), (-5,-3,0)								
**Quartz**	**(3,5,0)**	**10.199**	**71.75**	**3.045**	**2.9**	**3.541**	**10.22**	**44.26**	**45.43**
	Equiv. Refl.: (3,-8,0), (5,3,0), (5,-8,0), (-8,3,0), (-8,5,0)								
**LiNbO3**	**(2,4,-17)**	**10.58**	**80.11**	**2.996**	**5.1**	**1.871**	**9.49**	**23.39**	**25.24**
	Equiv. Refl.: (2,-6,17), (4,2,17), (4,-6,-17), (6,-2,17), (6,-4,-17), (-2,6,-17), (-2,-4,17), (-4,6,17), (-4,-2,-17) (-6,2,-17), (-6,4,17)								
**Quartz**	**(4,-3,8)**	**10.573**	**79.93**	**2.866**	**2.9**	**1.908**	**5.62**	**23.85**	**24.5**
**Quartz**	**(4,-1,-8)**	**10.573**	**79.93**	**2.865**	**2.9**	**1.908**	**5.62**	**23.85**	**24.5**
	Equiv. Refl.: (-1,-3,-8)								
**Quartz**	**(-1,4,8)**	**10.573**	**79.93**	**2.863**	**2.9**	**1.908**	**5.62**	**23.85**	**24.5**
**Quartz**	**(-3,4,-8)**	**10.573**	**79.93**	**2.86**	**2.9**	**1.908**	**5.61**	**23.85**	**24.5**
	Equiv. Refl.: (-3,-1,8)								
**Quartz**	**(1,3,8)**	**10.573**	**79.93**	**2.791**	**2.9**	**1.908**	**5.62**	**23.85**	**24.5**
	Equiv. Refl.: (-4,3,-8)								

Ei =	10.739	keV						ΔEg	ΔEt
Cryst	Refl	EB	ΘB	∫ IR dΘ	Width	Ei cotΘ	ΔE	2m, 50µm	2m, 50µm
	(h,k,l)	[keV]	[°]	[µrad]	[µrad]	[meV/µrad]	[meV]	[meV]	[meV]
**Quartz**	**(1,-4,-8)**	**10.573**	**79.93**	**2.79**	**2.9**	**1.908**	**5.62**	**23.85**	**24.5**
	Equiv. Refl.: (-4,1,8)								
**Quartz**	**(3,1,-8)**	**10.573**	**79.93**	**2.786**	**2.9**	**1.908**	**5.62**	**23.85**	**24.5**
	Equiv. Refl.: (3,-4,8)								
**Quartz**	**(-7,0,-2)**	**10.454**	**76.76**	**2.742**	**2.7**	**2.526**	**6.76**	**31.57**	**32.29**
**Quartz**	**(0,7,-2)**	**10.454**	**76.76**	**2.741**	**2.7**	**2.526**	**6.76**	**31.57**	**32.29**
	Equiv. Refl.: (0,-7,2), (7,0,2), (7,-7,-2), (-7,7,2)								
**Quartz**	**(2,6,0)**	**10.506**	**78.06**	**2.673**	**2.5**	**2.272**	**5.73**	**28.4**	**28.97**
	Equiv. Refl.: (2,-8,0), (6,2,0), (6,-8,0), (-8,2,0), (-8,6,0)								
**LiNbO3**	**(1,6,-11)**	**10.36**	**74.74**	**2.624**	**4.1**	**2.93**	**11.9**	**36.63**	**38.51**
	Equiv. Refl.: (1,-7,11), (6,1,11), (6,-7,-11), (7,-1,11), (7,-6,-11), (-1,7,-11), (-1,-6,11), (-6,7,11), (-6,-1,-11) (-7,1,-11), (-7,6,11)								
**LiNbO3**	**(2,2,21)**	**10.554**	**79.35**	**2.615**	**4.5**	**2.02**	**9.14**	**25.24**	**26.85**
	Equiv. Refl.: (2,2,-21), (2,-4,21), (2,-4,-21), (4,-2,21), (4,-2,-21), (-2,4,21), (-2,4,-21), (-2,-2,21), (-2,-2,-21) (-4,2,21), (-4,2,-21)								
**Quartz**	**(5,-1,7)**	**10.443**	**76.51**	**2.458**	**2.4**	**2.577**	**6.23**	**32.21**	**32.81**
	Equiv. Refl.: (5,-4,-7)								
**Quartz**	**(-4,-1,-7)**	**10.443**	**76.51**	**2.457**	**2.4**	**2.577**	**6.23**	**32.21**	**32.81**
**Quartz**	**(-4,5,7)**	**10.443**	**76.51**	**2.456**	**2.4**	**2.577**	**6.23**	**32.21**	**32.81**
**Quartz**	**(-1,-4,7)**	**10.443**	**76.51**	**2.455**	**2.4**	**2.577**	**6.22**	**32.21**	**32.81**
**Quartz**	**(-1,5,-7)**	**10.443**	**76.51**	**2.454**	**2.4**	**2.577**	**6.22**	**32.21**	**32.81**
**Sapph**	**(0,7,-4)**	**10.703**	**85.28**	**2.402**	**3.6**	**0.886**	**3.15**	**11.08**	**11.52**
	Equiv. Refl.: (0,-7,4), (7,0,4), (7,-7,-4), (-7,0,-4), (-7,7,4)								
**Quartz**	**(3,4,-5)**	**10.556**	**79.42**	**2.047**	**2.3**	**2.007**	**4.67**	**25.08**	**25.51**
	Equiv. Refl.: (3,-7,5), (-7,3,-5)								
**Quartz**	**(-7,4,5)**	**10.556**	**79.42**	**2.046**	**2.3**	**2.007**	**4.66**	**25.08**	**25.51**
**Quartz**	**(4,3,5)**	**10.556**	**79.42**	**2.045**	**2.3**	**2.007**	**4.66**	**25.08**	**25.51**
	Equiv. Refl.: (4,-7,-5), (-5,1,-7), (-5,4,7)								
**Quartz**	**(4,1,7)**	**10.443**	**76.51**	**2.043**	**2.4**	**2.577**	**6.23**	**32.21**	**32.81**
	Equiv. Refl.: (4,-5,-7)								
**Quartz**	**(1,4,-7)**	**10.443**	**76.51**	**2.042**	**2.4**	**2.577**	**6.22**	**32.21**	**32.81**
	Equiv. Refl.: (1,-5,7)								
**Quartz**	**(8,-2,0)**	**10.506**	**78.06**	**2.012**	**2.5**	**2.272**	**5.73**	**28.4**	**28.97**
	Equiv. Refl.: (8,-6,0), (-2,8,0), (-2,-6,0), (-6,8,0), (-6,-2,0)								
**Quartz**	**(6,1,3)**	**10.155**	**71.02**	**1.942**	**2**	**3.694**	**7.46**	**46.18**	**46.78**
	Equiv. Refl.: (6,-7,-3)								
**Quartz**	**(1,6,-3)**	**10.155**	**71.02**	**1.941**	**2**	**3.694**	**7.46**	**46.18**	**46.78**
	Equiv. Refl.: (1,-7,3)								
**Quartz**	**(-7,1,-3)**	**10.155**	**71.02**	**1.94**	**2**	**3.694**	**7.46**	**46.18**	**46.78**
	Equiv. Refl.: (-7,6,3)								
**Quartz**	**(-2,4,-8)**	**10.473**	**77.21**	**1.905**	**2.2**	**2.438**	**5.25**	**30.47**	**30.92**
	Equiv. Refl.: (-2,-2,8)								
**Quartz**	**(4,-2,-8)**	**10.473**	**77.21**	**1.903**	**2.2**	**2.438**	**5.25**	**30.47**	**30.92**
**Quartz**	**(4,-2,8)**	**10.473**	**77.21**	**1.902**	**2.2**	**2.438**	**5.25**	**30.47**	**30.92**
**Quartz**	**(-2,4,8)**	**10.473**	**77.21**	**1.898**	**2.1**	**2.438**	**5.24**	**30.47**	**30.92**
	Equiv. Refl.: (-2,-2,-8)								
**Sapph**	**(3,5,1)**	**10.542**	**79**	**1.889**	**2.4**	**2.087**	**5.06**	**26.09**	**26.58**
	Equiv. Refl.: (3,-8,-1), (5,3,-1), (5,-8,1), (8,-3,-1), (8,-5,1), (-3,8,1), (-3,-5,-1), (-5,8,-1), (-5,-3,1) (-8,3,1), (-8,5,-1)								
**Quartz**	**(-7,1,4)**	**10.599**	**80.73**	**1.876**	**2.3**	**1.754**	**4.09**	**21.92**	**22.3**
	Equiv. Refl.: (-7,6,-4)								

Ei = Cryst	10.739 Refl (h,k,l)	keV EB [keV]	ΘB [°]	∫ IR dΘ [µrad]	Width [µrad]	Ei cotΘ [meV/µrad]	ΔE [meV]	ΔEg 2m, 50µm [meV]	ΔEt 2m, 50µm [meV]
**Quartz**	**(6,1,-4)**	**10.599**	**80.73**	**1.874**	**2.3**	**1.754**	**4.09**	**21.92**	**22.3**
	Equiv. Refl.: (6,-7,4)								
**Quartz**	**(1,6,4)**	**10.599**	**80.73**	**1.873**	**2.3**	**1.754**	**4.09**	**21.92**	**22.3**
	Equiv. Refl.: (1,-7,-4)								
**Quartz**	**(-5,7,4)**	**10.19**	**71.6**	**1.85**	**2.1**	**3.572**	**7.34**	**44.65**	**45.25**
	Equiv. Refl.: (-5,-2,-4)								
**Quartz**	**(7,-2,4)**	**10.19**	**71.6**	**1.849**	**2.1**	**3.572**	**7.34**	**44.65**	**45.24**
	Equiv. Refl.: (7,-5,-4)								
**Quartz**	**(-2,7,-4)**	**10.19**	**71.6**	**1.848**	**2.1**	**3.572**	**7.34**	**44.65**	**45.24**
	Equiv. Refl.: (-2,-5,4)								
**Quartz**	**(7,-1,-4)**	**10.599**	**80.73**	**1.843**	**2.3**	**1.754**	**4.09**	**21.92**	**22.3**
**Quartz**	**(7,-6,4)**	**10.599**	**80.73**	**1.842**	**2.3**	**1.754**	**4.09**	**21.92**	**22.3**
**Quartz**	**(-6,7,-4)**	**10.599**	**80.73**	**1.841**	**2.3**	**1.754**	**4.09**	**21.92**	**22.3**
	Equiv. Refl.: (-6,-1,4)								
**Quartz**	**(-1,7,4)**	**10.599**	**80.73**	**1.84**	**2.3**	**1.754**	**4.09**	**21.92**	**22.3**
	Equiv. Refl.: (-1,-6,-4)								
**Quartz**	**(5,2,4)**	**10.19**	**71.6**	**1.803**	**2.1**	**3.572**	**7.34**	**44.65**	**45.25**
	Equiv. Refl.: (5,-7,-4)								
**Quartz**	**(-7,2,-4)**	**10.19**	**71.6**	**1.802**	**2.1**	**3.572**	**7.34**	**44.65**	**45.24**
	Equiv. Refl.: (-7,5,4)								
**Quartz**	**(2,5,-4)**	**10.19**	**71.6**	**1.801**	**2.1**	**3.572**	**7.34**	**44.65**	**45.24**
	Equiv. Refl.: (2,-7,4)								
**Quartz**	**(7,-3,5)**	**10.556**	**79.42**	**1.781**	**2.3**	**2.007**	**4.67**	**25.08**	**25.51**
	Equiv. Refl.: (-3,7,-5), (-3,-4,5)								
**Quartz**	**(7,-4,-5)**	**10.556**	**79.42**	**1.78**	**2.3**	**2.007**	**4.66**	**25.08**	**25.51**
**Quartz**	**(-4,7,5)**	**10.556**	**79.42**	**1.779**	**2.3**	**2.007**	**4.66**	**25.08**	**25.51**
	Equiv. Refl.: (-4,-3,-5)								
**Quartz**	**(7,-1,-3)**	**10.155**	**71.02**	**1.756**	**1.9**	**3.694**	**6.96**	**46.18**	**46.7**
	Equiv. Refl.: (7,-6,3), (-1,-6,-3)								
**Quartz**	**(-1,7,3)**	**10.155**	**71.02**	**1.755**	**1.9**	**3.694**	**6.96**	**46.18**	**46.7**
	Equiv. Refl.: (-6,7,-3), (-6,-1,3)								
**Quartz**	**(2,-4,8)**	**10.473**	**77.21**	**1.726**	**2.2**	**2.438**	**5.25**	**30.47**	**30.92**
**Quartz**	**(2,2,-8)**	**10.473**	**77.21**	**1.725**	**2.2**	**2.438**	**5.25**	**30.47**	**30.92**
**Quartz**	**(-4,2,8)**	**10.473**	**77.21**	**1.724**	**2.2**	**2.438**	**5.25**	**30.47**	**30.92**
**Quartz**	**(-4,2,-8)**	**10.473**	**77.21**	**1.723**	**2.2**	**2.438**	**5.25**	**30.47**	**30.92**
**Quartz**	**(2,2,8)**	**10.473**	**77.21**	**1.719**	**2.1**	**2.438**	**5.24**	**30.47**	**30.92**
	Equiv. Refl.: (2,-4,-8)								
**Quartz**	**(-6,7,3)**	**10.155**	**71.02**	**1.665**	**2**	**3.694**	**7.46**	**46.18**	**46.78**
	Equiv. Refl.: (-6,-1,-3)								
**Quartz**	**(7,-1,3)**	**10.155**	**71.02**	**1.664**	**2**	**3.694**	**7.46**	**46.18**	**46.78**
	Equiv. Refl.: (-1,7,-3), (-1,-6,3)								
**Quartz**	**(7,-6,-3)**	**10.155**	**71.02**	**1.663**	**2**	**3.694**	**7.46**	**46.18**	**46.78**
**Quartz**	**(-7,1,3)**	**10.155**	**71.02**	**1.564**	**1.9**	**3.694**	**6.96**	**46.18**	**46.7**
	Equiv. Refl.: (-7,6,-3)								
**Quartz**	**(1,6,3)**	**10.155**	**71.02**	**1.563**	**1.9**	**3.694**	**6.96**	**46.18**	**46.7**
	Equiv. Refl.: (1,-7,-3), (6,1,-3), (6,-7,3)								
**Quartz**	**(2,5,4)**	**10.19**	**71.6**	**1.533**	**1.7**	**3.572**	**6.14**	**44.65**	**45.07**
	Equiv. Refl.: (2,-7,-4)								
**Quartz**	**(-7,2,4)**	**10.19**	**71.6**	**1.532**	**1.7**	**3.572**	**6.14**	**44.65**	**45.07**
	Equiv. Refl.: (-7,5,-4)								

Ei = Cryst	10.739 Refl (h,k,l)	keV EB [keV]	ΘB [°]	∫ IR dΘ [μrad]	Width [μrad]	Ei cotΘ [meV/μrad]	ΔE [meV]	ΔEg 2m, 50μm [meV]	ΔEt 2m, 50μm [meV]
**Quartz**	**(5,2,-4)**	**10.19**	**71.6**	**1.531**	**1.7**	**3.572**	**6.13**	**44.65**	**45.06**
	Equiv. Refl.: (5,-7,4)								
**Sapph**	**(2,4,13)**	**10.093**	**70.03**	**1.514**	**1.8**	**3.903**	**7.1**	**48.79**	**49.3**
	Equiv. Refl.: (2,-6,-13), (4,2,-13), (4,-6,13), (6,-2,-13), (6,-4,13), (-2,6,13), (-2,-4,-13), (-4,6,-13), (-4,-2,13) (-6,2,13), (-6,4,-13)								
**Sapph**	**(2,3,17)**	**10.433**	**76.28**	**1.468**	**1.9**	**2.622**	**4.99**	**32.77**	**33.15**
	Equiv. Refl.: (2,-5,-17), (3,2,-17), (3,-5,17), (5,-2,-17), (5,-3,17), (-2,5,17), (-2,-3,-17), (-3,5,-17), (-3,-2,17) (-5,2,17), (-5,3,-17)								
**Quartz**	**(-2,7,4)**	**10.19**	**71.6**	**1.434**	**1.7**	**3.572**	**6.14**	**44.65**	**45.07**
	Equiv. Refl.: (-2,-5,-4)								
**Quartz**	**(7,-2,-4)**	**10.19**	**71.6**	**1.433**	**1.7**	**3.572**	**6.14**	**44.65**	**45.07**
	Equiv. Refl.: (7,-5,4)								
**Quartz**	**(-5,7,-4)**	**10.19**	**71.6**	**1.432**	**1.7**	**3.572**	**6.13**	**44.65**	**45.06**
	Equiv. Refl.: (-5,-2,4)								
**Quartz**	**(6,0,5)**	**10.455**	**76.8**	**1.303**	**1.6**	**2.519**	**4.14**	**31.49**	**31.76**
	Equiv. Refl.: (6,-6,-5), (-6,0,-5), (-6,6,5)								
**Quartz**	**(0,6,-5)**	**10.455**	**76.8**	**1.299**	**1.6**	**2.519**	**4.13**	**31.49**	**31.76**
	Equiv. Refl.: (0,-6,5)								
**Quartz**	**(5,-2,-7)**	**10.237**	**72.42**	**1.163**	**1.4**	**3.403**	**4.65**	**42.54**	**42.8**
	Equiv. Refl.: (-2,5,7), (-2,-3,-7)								
**Quartz**	**(5,-3,7)**	**10.237**	**72.42**	**1.162**	**1.4**	**3.403**	**4.65**	**42.54**	**42.8**
**Quartz**	**(-3,5,-7)**	**10.237**	**72.42**	**1.161**	**1.4**	**3.403**	**4.64**	**42.54**	**42.79**
	Equiv. Refl.: (-3,-2,7)								
**Quartz**	**(0,7,-1)**	**10.263**	**72.88**	**1.125**	**1.4**	**3.308**	**4.61**	**41.35**	**41.6**
	Equiv. Refl.: (0,-7,1), (7,0,1), (7,-7,-1), (-7,0,-1), (-7,7,1)								
**LiNbO3**	**(3,4,-13)**	**10.263**	**72.88**	**1.122**	**2.3**	**3.308**	**7.46**	**41.35**	**42.02**
	Equiv. Refl.: (3,-7,13), (4,3,13), (4,-7,-13), (7,-3,13), (7,-4,-13), (-3,7,-13), (-3,-4,13), (-4,7,13), (-4,-3,-13) (-7,3,-13), (-7,4,13)								
**Quartz**	**(8,-4,1)**	**10.159**	**71.09**	**1.085**	**1.2**	**3.679**	**4.51**	**45.99**	**46.21**
	Equiv. Refl.: (8,-4,-1), (-4,8,1), (-4,8,-1), (-4,-4,1), (-4,-4,-1)								
**Quartz**	**(2,3,7)**	**10.237**	**72.42**	**1.059**	**1.4**	**3.403**	**4.65**	**42.54**	**42.8**
	Equiv. Refl.: (2,-5,-7), (-5,2,7), (-5,3,-7)								
**Quartz**	**(3,2,-7)**	**10.237**	**72.42**	**1.058**	**1.4**	**3.403**	**4.64**	**42.54**	**42.79**
	Equiv. Refl.: (3,-5,7)								
**Sapph**	**(1,6,7)**	**10.416**	**75.9**	**0.965**	**1.4**	**2.697**	**3.73**	**33.71**	**33.92**
	Equiv. Refl.: (1,-7,-7), (6,1,-7), (6,-7,7), (7,-1,-7), (7,-6,7), (-1,7,7), (-1,-6,-7), (-6,7,-7), (-6,-1,7) (-7,1,7), (-7,6,-7)								
**Quartz**	**(1,0,9)**	**10.425**	**76.12**	**0.956**	**1.3**	**2.653**	**3.4**	**33.17**	**33.34**
	Equiv. Refl.: (1,-1,-9), (-1,0,-9), (-1,1,9)								
**Quartz**	**(0,1,-9)**	**10.425**	**76.12**	**0.953**	**1.3**	**2.653**	**3.39**	**33.17**	**33.34**
	Equiv. Refl.: (0,-1,9)								
**Quartz**	**(4,4,1)**	**10.159**	**71.09**	**0.884**	**1.2**	**3.679**	**4.51**	**45.99**	**46.21**
	Equiv. Refl.: (4,4,-1), (4,-8,1), (4,-8,-1), (-8,4,1), (-8,4,-1)								
**Sapph**	**(1,3,19)**	**10.567**	**79.73**	**0.861**	**1.4**	**1.946**	**2.72**	**24.32**	**24.47**
	Equiv. Refl.: (1,-4,-19), (3,1,-19), (3,-4,19), (4,-1,-19), (4,-3,19), (-1,4,19), (-1,-3,-19), (-3,4,-19), (-3,-1,19) (-4,1,19), (-4,3,-19)								
**Sapph**	**(1,6,-5)**	**10.15**	**70.93**	**0.781**	**1.1**	**3.712**	**4.09**	**46.4**	**46.58**
	Equiv. Refl.: (1,-7,5), (6,1,5), (6,-7,-5), (7,-1,5), (7,-6,-5), (-1,7,-5), (-1,-6,5), (-6,7,5), (-6,-1,-5) (-7,1,-5), (-7,6,5)								
**Quartz**	**(3,-5,-7)**	**10.237**	**72.42**	**0.692**	**0.9**	**3.403**	**3.07**	**42.54**	**42.65**

Ei =	10.739	keV						ΔEg	ΔEt
Cryst	Refl	EB	ΘB	∫ IR dΘ	Width	Ei cotΘ	ΔE	2m, 50μm	2m, 50μm
	(h,k,l)	[keV]	[°]	[μrad]	[μrad]	[meV/μrad]	[meV]	[meV]	[meV]
**Quartz**	**(3,2,7)**	**10.237**	**72.42**	**0.691**	**0.9**	**3.403**	**3.07**	**42.54**	**42.65**
	Equiv. Refl.: (-5,2,-7)								
**Quartz**	**(2,-5,7)**	**10.237**	**72.42**	**0.69**	**0.9**	**3.403**	**3.07**	**42.54**	**42.65**
	Equiv. Refl.: (-5,3,7)								
**Quartz**	**(2,3,-7)**	**10.237**	**72.42**	**0.689**	**0.9**	**3.403**	**3.06**	**42.54**	**42.65**
**Quartz**	**(0,7,2)**	**10.454**	**76.76**	**0.653**	**1**	**2.526**	**2.48**	**31.57**	**31.67**
	Equiv. Refl.: (0,-7,-2)								
**Quartz**	**(7,0,-2)**	**10.454**	**76.76**	**0.652**	**1**	**2.526**	**2.48**	**31.57**	**31.67**
	Equiv. Refl.: (7,-7,2), (-7,0,2), (-7,7,-2)								
**Quartz**	**(-3,5,7)**	**10.237**	**72.42**	**0.594**	**0.9**	**3.403**	**3.07**	**42.54**	**42.65**
	Equiv. Refl.: (-3,-2,-7)								
**Quartz**	**(5,-2,7)**	**10.237**	**72.42**	**0.593**	**0.9**	**3.403**	**3.07**	**42.54**	**42.65**
	Equiv. Refl.: (5,-3,-7)								
**Quartz**	**(-2,5,-7)**	**10.237**	**72.42**	**0.592**	**0.9**	**3.403**	**3.07**	**42.54**	**42.65**
	Equiv. Refl.: (-2,-3,7)								
**LiNbO3**	**(2,6,-7)**	**10.504**	**78**	**0.547**	**1.7**	**2.283**	**3.94**	**28.54**	**28.81**
	Equiv. Refl.: (2,-8,7), (6,2,7), (6,-8,-7), (8,-2,7), (8,-6,-7), (-2,8,-7), (-2,-6,7), (-6,8,7), (-6,-2,-7) (-8,2,-7), (-8,6,7)								
**Quartz**	**(5,3,-1)**	**10.263**	**72.88**	**0.496**	**0.7**	**3.308**	**2.38**	**41.35**	**41.41**
	Equiv. Refl.: (5,-8,1)								
**Quartz**	**(3,5,1)**	**10.263**	**72.88**	**0.495**	**0.7**	**3.308**	**2.38**	**41.35**	**41.41**
	Equiv. Refl.: (3,-8,-1), (-8,3,1), (-8,5,-1)								
**Sapph**	**(0,1,-22)**	**10.608**	**81.03**	**0.477**	**1**	**1.695**	**1.71**	**21.19**	**21.26**
	Equiv. Refl.: (0,-1,22), (1,0,22), (1,-1,-22), (-1,0,-22), (-1,1,22)								
**LiNbO3**	**(1,7,3)**	**10.583**	**80.23**	**0.43**	**1.7**	**1.849**	**3.09**	**23.11**	**23.31**
	Equiv. Refl.: (1,7,-3), (1,-8,3), (1,-8,-3), (7,1,3), (7,1,-3), (7,-8,3), (7,-8,-3), (8,-1,3), (8,-1,-3) (8,-7,3), (8,-7,-3), (-1,8,3), (-1,8,-3), (-1,-7,3), (-1,-7,-3), (-7,8,3), (-7,8,-3), (-7,-1,3) (-7,-1,-3), (-8,1,3), (-8,1,-3), (-8,7,3), (-8,7,-3)								
**Quartz**	**(8,-3,-1)**	**10.263**	**72.88**	**0.429**	**0.7**	**3.308**	**2.38**	**41.35**	**41.41**
	Equiv. Refl.: (8,-5,1), (-3,8,1), (-3,-5,-1), (-5,8,-1), (-5,-3,1)								
**LiNbO3**	**(2,6,5)**	**10.273**	**73.06**	**0.421**	**1.3**	**3.27**	**4.23**	**40.88**	**41.1**
	Equiv. Refl.: (2,-8,-5), (6,2,-5), (6,-8,5), (8,-2,-5), (8,-6,5), (-2,8,5), (-2,-6,-5), (-6,8,-5), (-6,-2,5) (-8,2,5), (-8,6,-5)								
**LiNbO3**	**(2,3,-19)**	**10.437**	**76.37**	**0.311**	**1.2**	**2.604**	**3.16**	**32.54**	**32.7**
	Equiv. Refl.: (2,-5,19), (3,2,19), (3,-5,-19), (5,-2,19), (5,-3,-19), (-2,5,-19), (-2,-3,19), (-3,5,19), (-3,-2,-19) (-5,2,-19), (-5,3,19)								
**Sapph**	**(3,4,11)**	**10.55**	**79.24**	**0.301**	**0.7**	**2.041**	**1.46**	**25.51**	**25.55**
	Equiv. Refl.: (3,-7,-11), (4,3,-11), (4,-7,11), (7,-3,-11), (7,-4,11), (-3,7,11), (-3,-4,-11), (-4,7,-11), (-4,-3,11) (-7,3,11), (-7,4,-11)								
**Quartz**	**(8,-3,1)**	**10.263**	**72.88**	**0.259**	**0.5**	**3.308**	**1.51**	**41.35**	**41.37**
	Equiv. Refl.: (8,-5,-1), (-3,8,-1), (-3,-5,1), (-5,8,1), (-5,-3,-1)								
**LiNbO3**	**(3,5,7)**	**10.224**	**72.19**	**0.22**	**0.9**	**3.45**	**3.12**	**43.12**	**43.24**
	Equiv. Refl.: (3,-8,-7), (5,3,-7), (5,-8,7), (8,-3,-7), (8,-5,7), (-3,8,7), (-3,-5,-7), (-5,8,-7), (-5,-3,7) (-8,3,7), (-8,5,-7)								
**Quartz**	**(3,5,-1)**	**10.263**	**72.88**	**0.205**	**0.5**	**3.308**	**1.51**	**41.35**	**41.37**
	Equiv. Refl.: (3,-8,1), (5,3,1), (5,-8,-1), (-8,3,-1), (-8,5,1)								
**Sapph**	**(3,3,15)**	**10.6**	**80.78**	**0.071**	**0.4**	**1.743**	**0.62**	**21.79**	**21.8**
	Equiv. Refl.: (3,3,-15), (3,-6,15), (3,-6,-15), (6,-3,15), (6,-3,-15), (-3,6,15), (-3,6,-15), (-3,-3,15), (-3,-3,-15) (-6,3,15), (-6,3,-15)								
**LiNbO3**	**(4,4,9)**	**10.44**	**76.46**	**0.021**	**0.4**	**2.586**	**0.91**	**32.33**	**32.34**
	Equiv. Refl.: (4,4,-9), (4,-8,9), (4,-8,-9), (8,-4,9), (8,-4,-9), (-4,8,9), (-4,8,-9), (-4,-4,9), (-4,-4,-9) (-8,4,9), (-8,4,-9)								

Ei =	9.561	keV						ΔEg	ΔEt
Cryst	Refl	EB	ΘB	∫ IR dΘ	Width	Ei cotΘ	ΔE	2m, 50µm	2m, 50µm
	(h,k,l)	[keV]	[°]	[µrad]	[µrad]	[meV/µrad]	[meV]	[meV]	[meV]
**Ge**	**(5,5,5)**	**9.49**	**83**	**25.5818**	**25.81**	**1.174**	**30.3**	**14.67**	**33.66**
	Equiv. Refl.: (1,7,5), (5,1,7), (1,5,7), (5,7,1), (7,1,5), (7,5,1)								
**Ge**	**(0,6,6)**	**9.298**	**76.53**	**21.7066**	**20.28**	**2.2901**	**46.45**	**28.63**	**54.56**
	Equiv. Refl.: (2,2,8), (2,8,2), (6,0,6), (6,6,0), (8,2,2)								
**Si**	**(0,0,8)**	**9.132**	**72.76**	**11.101**	**9.4**	**2.9665**	**27.87**	**37.08**	**46.39**
	Equiv. Refl.: (0,8,0), (8,0,0)								
**Si**	**(3,3,7)**	**9.343**	**77.74**	**9.4669**	**8.71**	**2.0768**	**18.09**	**25.96**	**31.64**
	Equiv. Refl.: (3,7,3), (7,3,3)								
**Quartz**	**(7,-1,0)**	**9.554**	**87.82**	**56.733**	**49.3**	**0.364**	**17.92**	**4.54**	**18.49**
	Equiv. Refl.: (7,-6,0), (-1,7,0), (-1,-6,0), (-6,7,0), (-6,-1,0)								
**Quartz**	**(1,6,0)**	**9.554**	**87.82**	**56.159**	**49.3**	**0.364**	**17.92**	**4.54**	**18.49**
	Equiv. Refl.: (1,-7,0), (6,1,0), (6,-7,0), (-7,1,0), (-7,6,0)								
**LiNbO3**	**(0,-5,14)**	**9.356**	**78.11**	**28.652**	**25.5**	**2.014**	**51.41**	**25.17**	**57.24**
	Equiv. Refl.: (5,0,14)								
**LiNbO3**	**(-5,5,14)**	**9.356**	**78.11**	**28.651**	**25.5**	**2.014**	**51.41**	**25.17**	**57.24**
**LiNbO3**	**(0,5,-14)**	**9.356**	**78.11**	**25.771**	**25.5**	**2.014**	**51.41**	**25.17**	**57.24**
	Equiv. Refl.: (-5,0,-14)								
**LiNbO3**	**(5,-5,-14)**	**9.356**	**78.11**	**25.77**	**25.5**	**2.014**	**51.41**	**25.17**	**57.24**
**LiNbO3**	**(2,2,-18)**	**9.38**	**78.84**	**24.834**	**23.4**	**1.885**	**44.19**	**23.57**	**50.08**
	Equiv. Refl.: (2,-4,-18), (4,-2,-18), (-2,4,-18), (-2,-2,-18), (-4,2,-18)								
**LiNbO3**	**(2,3,-16)**	**9.377**	**78.74**	**24.726**	**22.8**	**1.904**	**43.43**	**23.79**	**49.53**
	Equiv. Refl.: (3,-5,-16), (5,-3,-16), (-2,5,-16), (-3,-2,-16), (-5,2,-16)								
**LiNbO3**	**(2,-4,18)**	**9.38**	**78.84**	**23.865**	**23.4**	**1.885**	**44.19**	**23.57**	**50.08**
	Equiv. Refl.: (4,-2,18)								
**LiNbO3**	**(2,2,18)**	**9.38**	**78.84**	**23.864**	**23.4**	**1.885**	**44.19**	**23.57**	**50.08**
	Equiv. Refl.: (-2,4,18), (-2,-2,18), (-4,2,18)								
**LiNbO3**	**(2,-5,16)**	**9.377**	**78.74**	**22.531**	**22.8**	**1.904**	**43.43**	**23.79**	**49.53**
	Equiv. Refl.: (3,2,16), (5,-2,16), (-2,-3,16), (-3,5,16), (-5,3,16)								
**LiNbO3**	**(0,-3,18)**	**9.066**	**71.48**	**22.081**	**19.5**	**3.203**	**62.49**	**40.03**	**74.21**
	Equiv. Refl.: (3,0,18), (-3,3,18)								
**LiNbO3**	**(3,-3,18)**	**9.066**	**71.48**	**21.853**	**19.3**	**3.203**	**61.82**	**40.03**	**73.65**
**LiNbO3**	**(0,3,18)**	**9.066**	**71.48**	**21.852**	**19.3**	**3.203**	**61.82**	**40.03**	**73.65**
	Equiv. Refl.: (-3,0,18)								
**LiNbO3**	**(0,2,-20)**	**9.366**	**78.41**	**21.51**	**20.4**	**1.962**	**40.07**	**24.52**	**46.97**
	Equiv. Refl.: (2,-2,-20), (-2,0,-20)								
**LiNbO3**	**(1,6,-2)**	**9.162**	**73.38**	**21.306**	**19.6**	**2.853**	**55.87**	**35.66**	**66.28**
	Equiv. Refl.: (6,-7,-2), (7,-6,-2), (-1,7,-2), (-6,-1,-2), (-7,1,-2)								
**LiNbO3**	**(1,-7,2)**	**9.162**	**73.38**	**20.746**	**19.6**	**2.853**	**55.87**	**35.66**	**66.28**
	Equiv. Refl.: (6,1,2), (7,-1,2), (-1,-6,2), (-6,7,2), (-7,6,2)								
**LiNbO3**	**(0,3,-18)**	**9.066**	**71.48**	**20.419**	**19.5**	**3.203**	**62.49**	**40.03**	**74.21**
	Equiv. Refl.: (3,-3,-18), (-3,0,-18)								
**LiNbO3**	**(0,-2,20)**	**9.366**	**78.41**	**20.126**	**20.4**	**1.962**	**40.07**	**24.52**	**46.97**
	Equiv. Refl.: (2,0,20), (-2,2,20)								
**LiNbO3**	**(0,-3,-18)**	**9.066**	**71.48**	**20.123**	**19.3**	**3.203**	**61.82**	**40.03**	**73.65**
	Equiv. Refl.: (3,0,-18), (-3,3,-18)								
**LiNbO3**	**(3,4,8)**	**9.183**	**73.84**	**19.247**	**17.6**	**2.77**	**48.82**	**34.62**	**59.85**
	Equiv. Refl.: (4,-7,8), (7,-4,8), (-3,7,8), (-4,-3,8), (-7,3,8)								

Ei =	9.561	keV						ΔEg	ΔEt
**Cryst**	**Refl**	**EB**	**ΘB**	**∫ IR dΘ**	**Width**	**Ei cotΘ**	**ΔE**	**2m, 50µm**	**2m, 50µm**
	**(h,k,l)**	**[keV]**	**[°]**	**[µrad]**	**[µrad]**	**[meV/µrad]**	**[meV]**	**[meV]**	**[meV]**
**LiNbO3**	**(1,6,4)**	**9.292**	**76.37**	**17.921**	**17.2**	**2.318**	**39.91**	**28.97**	**49.32**
	Equiv. Refl.: (6,-7,4), (7,-6,4), (-1,7,4), (-6,-1,4), (-7,1,4)								
**LiNbO3**	**(3,-7,-8)**	**9.183**	**73.84**	**17.764**	**17.6**	**2.77**	**48.82**	**34.62**	**59.85**
	Equiv. Refl.: (4,3,-8), (7,-3,-8), (-3,-4,-8), (-4,7,-8), (-7,4,-8)								
**LiNbO3**	**(1,-7,-4)**	**9.292**	**76.37**	**17.025**	**17.2**	**2.318**	**39.91**	**28.97**	**49.32**
	Equiv. Refl.: (6,1,-4), (7,-1,-4), (-1,-6,-4), (-6,7,-4), (-7,6,-4)								
**LiNbO3**	**(0,-1,-20)**	**9.051**	**71.2**	**16.941**	**16**	**3.255**	**52.14**	**40.68**	**66.13**
	Equiv. Refl.: (1,0,-20), (-1,1,-20)								
**LiNbO3**	**(0,1,20)**	**9.051**	**71.2**	**16.919**	**16**	**3.255**	**52.14**	**40.68**	**66.13**
	Equiv. Refl.: (1,-1,20), (-1,0,20)								
**LiNbO3**	**(2,-7,-6)**	**9.089**	**71.91**	**16.66**	**15.8**	**3.122**	**49.46**	**39.03**	**63.01**
	Equiv. Refl.: (5,2,-6), (7,-2,-6), (-2,-5,-6), (-5,7,-6), (-7,5,-6)								
**LiNbO3**	**(2,5,6)**	**9.089**	**71.91**	**16.56**	**15.8**	**3.122**	**49.46**	**39.03**	**63.01**
	Equiv. Refl.: (5,-7,6), (7,-5,6), (-2,7,6), (-5,-2,6), (-7,2,6)								
**Sapph**	**(2,3,14)**	**9.362**	**78.3**	**16.525**	**14.1**	**1.98**	**28**	**24.75**	**37.37**
	Equiv. Refl.: (2,-5,-14), (3,2,-14), (3,-5,14), (5,-2,-14), (5,-3,14), (-2,5,14), (-2,-3,-14), (-3,5,-14), (-3,-2,14) (-5,2,14), (-5,3,-14)								
**Quartz**	**(7,-3,3)**	**9.507**	**83.91**	**16.397**	**14.4**	**1.02**	**14.66**	**12.75**	**19.42**
	Equiv. Refl.: (7,-4,-3)								
**Quartz**	**(-3,7,-3)**	**9.507**	**83.91**	**16.396**	**14.4**	**1.02**	**14.66**	**12.75**	**19.42**
**Quartz**	**(-3,-4,3)**	**9.507**	**83.91**	**16.395**	**14.4**	**1.02**	**14.65**	**12.75**	**19.42**
**Quartz**	**(-4,7,3)**	**9.507**	**83.91**	**16.387**	**14.4**	**1.02**	**14.65**	**12.75**	**19.42**
**Quartz**	**(-4,-3,-3)**	**9.507**	**83.91**	**16.386**	**14.4**	**1.02**	**14.65**	**12.75**	**19.42**
**LiNbO3**	**(3,3,-12)**	**9**	**70.27**	**16.325**	**15.1**	**3.428**	**51.68**	**42.85**	**67.13**
	Equiv. Refl.: (3,-6,-12), (6,-3,-12), (-3,6,-12), (-3,-3,-12), (-6,3,-12)								
**LiNbO3**	**(2,5,-6)**	**9.089**	**71.91**	**16.236**	**15.4**	**3.122**	**48.09**	**39.03**	**61.94**
	Equiv. Refl.: (-2,7,-6), (-5,-2,-6), (-7,2,-6)								
**LiNbO3**	**(5,-7,-6)**	**9.089**	**71.91**	**16.235**	**15.4**	**3.122**	**48.09**	**39.03**	**61.94**
	Equiv. Refl.: (7,-5,-6)								
**Quartz**	**(3,-7,3)**	**9.507**	**83.91**	**16.184**	**14.4**	**1.02**	**14.66**	**12.75**	**19.42**
	Equiv. Refl.: (-7,3,-3), (-7,4,3)								
**Quartz**	**(3,4,-3)**	**9.507**	**83.91**	**16.182**	**14.4**	**1.02**	**14.65**	**12.75**	**19.42**
**Quartz**	**(4,-7,-3)**	**9.507**	**83.91**	**16.174**	**14.4**	**1.02**	**14.65**	**12.75**	**19.42**
**Quartz**	**(4,3,3)**	**9.507**	**83.91**	**16.173**	**14.4**	**1.02**	**14.65**	**12.75**	**19.42**
**Sapph**	**(0,6,0)**	**9.027**	**70.75**	**15.911**	**13.1**	**3.339**	**43.81**	**41.73**	**60.5**
	Equiv. Refl.: (0,-6,0), (6,0,0), (6,-6,0), (-6,0,0), (-6,6,0)								
**LiNbO3**	**(2,-7,6)**	**9.089**	**71.91**	**15.901**	**15.4**	**3.122**	**48.09**	**39.03**	**61.94**
	Equiv. Refl.: (5,2,6), (7,-2,6), (-2,-5,6), (-5,7,6), (-7,5,6)								
**LiNbO3**	**(0,-4,16)**	**9.062**	**71.41**	**15.83**	**14.7**	**3.215**	**47.33**	**40.19**	**62.09**
	Equiv. Refl.: (4,0,16), (-4,4,16)								
**LiNbO3**	**(3,3,12)**	**9**	**70.27**	**15.42**	**15.1**	**3.428**	**51.68**	**42.85**	**67.13**
	Equiv. Refl.: (3,-6,12), (6,-3,12), (-3,6,12), (-3,-3,12), (-6,3,12)								
**Quartz**	**(-3,6,-5)**	**9.498**	**83.41**	**14.926**	**13**	**1.105**	**14.39**	**13.82**	**19.95**
	Equiv. Refl.: (-3,-3,5)								
**Quartz**	**(6,-3,5)**	**9.498**	**83.41**	**14.917**	**13**	**1.105**	**14.38**	**13.82**	**19.94**
**Quartz**	**(6,-3,-5)**	**9.498**	**83.41**	**14.916**	**13**	**1.105**	**14.38**	**13.82**	**19.94**
**Quartz**	**(-3,-3,-5)**	**9.498**	**83.41**	**14.914**	**13**	**1.105**	**14.38**	**13.82**	**19.94**
**Quartz**	**(-3,6,5)**	**9.498**	**83.41**	**14.911**	**13**	**1.105**	**14.38**	**13.82**	**19.94**
**LiNbO3**	**(0,4,-16)**	**9.062**	**71.41**	**14.763**	**14.7**	**3.215**	**47.33**	**40.19**	**62.09**
	Equiv. Refl.: (4,-4,-16), (-4,0,-16)								

Ei =	9.561	keV						ΔEg	ΔEt
**Cryst**	**Refl**	**EB**	**ΘB**	**∫ IR dΘ**	**Width**	**Ei cotΘ**	**ΔE**	**2m, 50µm**	**2m, 50µm**
	**(h,k,l)**	**[keV]**	**[°]**	**[µrad]**	**[µrad]**	**[meV/µrad]**	**[meV]**	**[meV]**	**[meV]**
**Quartz**	**(3,3,-5)**	**9.498**	**83.41**	**14.498**	**13**	**1.105**	**14.39**	**13.82**	**19.95**
	Equiv. Refl.: (3,-6,5)								
**Quartz**	**(-6,3,-5)**	**9.498**	**83.41**	**14.489**	**13**	**1.105**	**14.38**	**13.82**	**19.94**
**Quartz**	**(-6,3,5)**	**9.498**	**83.41**	**14.488**	**13**	**1.105**	**14.38**	**13.82**	**19.94**
**Quartz**	**(3,3,5)**	**9.498**	**83.41**	**14.487**	**13**	**1.105**	**14.38**	**13.82**	**19.94**
**Quartz**	**(3,-6,-5)**	**9.498**	**83.41**	**14.483**	**13**	**1.105**	**14.38**	**13.82**	**19.94**
**Quartz**	**(-6,6,-3)**	**9.395**	**79.3**	**14.112**	**11.7**	**1.806**	**21.2**	**22.58**	**30.97**
**Quartz**	**(6,0,-3)**	**9.395**	**79.3**	**14.111**	**11.7**	**1.806**	**21.2**	**22.58**	**30.97**
	Equiv. Refl.: (6,-6,3), (-6,0,3)								
**Quartz**	**(0,6,3)**	**9.395**	**79.3**	**14.104**	**11.7**	**1.806**	**21.19**	**22.58**	**30.96**
	Equiv. Refl.: (0,-6,-3)								
**Sapph**	**(2,4,10)**	**9.282**	**76.12**	**12.113**	**10.2**	**2.362**	**24.12**	**29.53**	**38.13**
	Equiv. Refl.: (2,-6,-10), (4,2,-10), (4,-6,10), (6,-2,-10), (6,-4,10), (-2,6,10), (-2,-4,-10), (-4,6,-10), (-4,-2,10) (-6,2,10), (-6,4,-10)								
**Quartz**	**(7,-2,2)**	**9.384**	**78.95**	**11.965**	**9.8**	**1.868**	**18.37**	**23.35**	**29.71**
	Equiv. Refl.: (7,-5,-2)								
**Quartz**	**(-2,7,-2)**	**9.384**	**78.95**	**11.963**	**9.8**	**1.868**	**18.37**	**23.35**	**29.71**
**Quartz**	**(-2,-5,2)**	**9.384**	**78.95**	**11.962**	**9.8**	**1.868**	**18.37**	**23.35**	**29.71**
	Equiv. Refl.: (-5,7,2), (-5,-2,-2)								
**Quartz**	**(-7,2,-2)**	**9.384**	**78.95**	**11.62**	**9.8**	**1.868**	**18.37**	**23.35**	**29.71**
	Equiv. Refl.: (-7,5,2)								
**Quartz**	**(2,5,-2)**	**9.384**	**78.95**	**11.618**	**9.8**	**1.868**	**18.37**	**23.35**	**29.71**
	Equiv. Refl.: (2,-7,2)								
**Quartz**	**(5,2,2)**	**9.384**	**78.95**	**11.617**	**9.8**	**1.868**	**18.37**	**23.35**	**29.71**
	Equiv. Refl.: (5,-7,-2)								
**Quartz**	**(1,-1,-8)**	**9.291**	**76.35**	**11.152**	**9.2**	**2.321**	**21.42**	**29.02**	**36.07**
	Equiv. Refl.: (-1,1,8)								
**Quartz**	**(-1,0,-8)**	**9.291**	**76.35**	**11.151**	**9.2**	**2.321**	**21.42**	**29.02**	**36.06**
**Quartz**	**(1,0,8)**	**9.291**	**76.35**	**11.15**	**9.2**	**2.321**	**21.42**	**29.02**	**36.06**
**Quartz**	**(0,1,-8)**	**9.291**	**76.35**	**11.145**	**9.2**	**2.321**	**21.41**	**29.02**	**36.06**
	Equiv. Refl.: (0,-1,8)								
**Quartz**	**(2,5,1)**	**9.171**	**73.58**	**10.763**	**9**	**2.818**	**25.45**	**35.23**	**43.46**
	Equiv. Refl.: (2,-7,-1), (5,2,-1), (5,-7,1), (-7,2,1), (-7,5,-1)								
**Quartz**	**(7,-2,-1)**	**9.171**	**73.58**	**10.673**	**9**	**2.818**	**25.45**	**35.23**	**43.46**
	Equiv. Refl.: (7,-5,1), (-2,7,1), (-2,-5,-1), (-5,7,-1), (-5,-2,1)								
**Quartz**	**(3,0,-7)**	**9.142**	**72.97**	**10.546**	**8.5**	**2.929**	**24.93**	**36.61**	**44.29**
**Quartz**	**(0,3,7)**	**9.142**	**72.97**	**10.545**	**8.5**	**2.929**	**24.93**	**36.61**	**44.29**
	Equiv. Refl.: (0,-3,-7), (3,-3,7), (-3,0,7)								
**Quartz**	**(-3,3,-7)**	**9.142**	**72.97**	**10.544**	**8.5**	**2.929**	**24.93**	**36.61**	**44.29**
**Sapph**	**(2,5,3)**	**9.504**	**83.72**	**10.203**	**10.3**	**1.053**	**10.8**	**13.16**	**17.02**
	Equiv. Refl.: (2,5,-3), (2,-7,3), (2,-7,-3), (5,2,3), (5,2,-3), (5,-7,3), (5,-7,-3), (7,-2,3), (7,-2,-3) (7,-5,3), (7,-5,-3), (-2,7,3), (-2,7,-3), (-2,-5,3), (-2,-5,-3), (-5,7,3), (-5,7,-3), (-5,-2,3) (-5,-2,-3), (-7,2,3), (-7,2,-3), (-7,5,3), (-7,5,-3)								
**Sapph**	**(2,4,-11)**	**9.536**	**85.86**	**10.177**	**11.2**	**0.692**	**7.78**	**8.65**	**11.63**
	Equiv. Refl.: (2,-6,11), (4,2,11), (4,-6,-11), (6,-2,11), (6,-4,-11), (-2,6,-11), (-2,-4,11), (-4,6,11), (-4,-2,-11) (-6,2,-11), (-6,4,11)								
**Sapph**	**(2,5,0)**	**9.395**	**79.31**	**9.974**	**8.9**	**1.805**	**16.15**	**22.56**	**27.74**
	Equiv. Refl.: (2,-7,0), (5,2,0), (5,-7,0), (7,-2,0), (7,-5,0), (-2,7,0), (-2,-5,0), (-5,7,0), (-5,-2,0) (-7,2,0), (-7,5,0)								
**Quartz**	**(-2,1,-8)**	**9.517**	**84.49**	**8.913**	**8.2**	**0.922**	**7.55**	**11.53**	**13.78**
**Quartz**	**(-2,1,8)**	**9.517**	**84.49**	**8.909**	**8.2**	**0.922**	**7.54**	**11.53**	**13.78**

Ei =	9.561	keV						ΔEg	ΔEt
Cryst	Refl	EB	ΘB	∫ IR dΘ	Width	Ei cotΘ	ΔE	2m, 50µm	2m, 50µm
	(h,k,l)	[keV]	[°]	[µrad]	[µrad]	[meV/µrad]	[meV]	[meV]	[meV]
Quartz	(1,-2,-8)	9.517	84.49	8.897	8.2	0.922	7.54	11.53	13.77
Quartz	(1,1,8)	9.517	84.49	8.894	8.2	0.922	7.53	11.53	13.77
Quartz	(1,1,-8)	9.517	84.49	8.882	8.2	0.922	7.52	11.53	13.77
Quartz	(1,-2,8)	9.517	84.49	8.877	8.2	0.922	7.52	11.53	13.76
Quartz	(7,-2,-2)	9.384	78.95	8.616	7.7	1.868	14.36	23.35	27.41
	Equiv. Refl.: (7,-5,2)								
Quartz	(-2,7,2)	9.384	78.95	8.615	7.7	1.868	14.35	23.35	27.41
Quartz	(-2,-5,-2)	9.384	78.95	8.614	7.7	1.868	14.35	23.35	27.41
	Equiv. Refl.: (-5,7,-2), (-5,-2,2)								
Quartz	(-7,2,2)	9.384	78.95	8.597	7.7	1.868	14.36	23.35	27.41
	Equiv. Refl.: (-7,5,-2)								
Quartz	(2,-7,-2)	9.384	78.95	8.596	7.7	1.868	14.35	23.35	27.41
Quartz	(2,5,2)	9.384	78.95	8.595	7.7	1.868	14.35	23.35	27.41
	Equiv. Refl.: (5,2,-2), (5,-7,2)								
Quartz	(3,4,2)	9.155	73.23	8.445	6.9	2.88	19.91	36	41.14
	Equiv. Refl.: (3,-7,-2), (-7,3,2), (-7,4,-2)								
Quartz	(4,3,-2)	9.155	73.23	8.443	6.9	2.88	19.9	36	41.14
	Equiv. Refl.: (4,-7,2)								
Quartz	(0,5,-5)	9.272	75.87	8.318	7	2.408	16.91	30.1	34.52
	Equiv. Refl.: (5,0,5), (5,-5,-5), (-5,0,-5), (-5,5,5)								
Quartz	(0,-5,5)	9.272	75.87	8.317	7	2.408	16.91	30.1	34.52
Sapph	(3,4,2)	9.201	74.22	8.081	7.2	2.702	19.52	33.77	39.01
	Equiv. Refl.: (3,-7,-2), (4,3,-2), (4,-7,2), (7,-3,-2), (7,-4,2), (-3,7,2), (-3,-4,-2), (-4,7,-2), (-4,-3,2) (-7,3,2), (-7,4,-2)								
Quartz	(7,-3,-2)	9.155	73.23	8.045	6.9	2.88	19.91	36	41.14
	Equiv. Refl.: (7,-4,2), (-3,7,2)								
Quartz	(-3,-4,-2)	9.155	73.23	8.044	6.9	2.88	19.91	36	41.14
Quartz	(-4,7,-2)	9.155	73.23	8.043	6.9	2.88	19.9	36	41.14
	Equiv. Refl.: (-4,-3,2)								
Quartz	(-6,1,4)	9.32	77.1	8.026	7	2.19	15.4	27.37	31.4
	Equiv. Refl.: (-6,5,-4)								
Quartz	(1,5,4)	9.32	77.1	8.023	7	2.19	15.39	27.37	31.4
	Equiv. Refl.: (1,-6,-4)								
Quartz	(5,1,-4)	9.32	77.1	8.017	7	2.19	15.38	27.37	31.4
	Equiv. Refl.: (5,-6,4)								
Quartz	(6,-1,-4)	9.32	77.1	7.916	7	2.19	15.4	27.37	31.4
	Equiv. Refl.: (6,-5,4)								
Quartz	(-1,6,4)	9.32	77.1	7.913	7	2.19	15.39	27.37	31.4
	Equiv. Refl.: (-1,-5,-4)								
Quartz	(-5,-1,4)	9.32	77.1	7.907	7	2.19	15.38	27.37	31.4
Quartz	(-5,6,-4)	9.32	77.1	7.906	7	2.19	15.38	27.37	31.4
Quartz	(4,-4,6)	9.018	70.6	7.707	6.4	3.367	21.59	42.08	47.3
	Equiv. Refl.: (-4,4,-6)								
Quartz	(4,0,-6)	9.018	70.6	7.706	6.4	3.367	21.58	42.08	47.3
	Equiv. Refl.: (-4,0,6)								
Quartz	(0,4,6)	9.018	70.6	7.703	6.4	3.367	21.58	42.08	47.29
	Equiv. Refl.: (0,-4,-6)								
Quartz	(2,-1,8)	9.517	84.49	7.41	8.2	0.922	7.55	11.53	13.78
Quartz	(2,-1,-8)	9.517	84.49	7.407	8.2	0.922	7.54	11.53	13.78
Quartz	(-1,2,8)	9.517	84.49	7.396	8.2	0.922	7.54	11.53	13.77

Ei =	9.561	keV							
Cryst	Refl (h,k,l)	EB [keV]	ΘB [°]	∫ IR dΘ [μrad]	Width [μrad]	Ei cotΘ [meV/μrad]	ΔE [meV]	ΔEg 2m, 50μm [meV]	ΔEt 2m, 50μm [meV]
**Quartz**	**(-1,-1,-8)**	**9.517**	**84.49**	**7.392**	**8.2**	**0.922**	**7.53**	**11.53**	**13.77**
**Quartz**	**(-1,-1,8)**	**9.517**	**84.49**	**7.381**	**8.2**	**0.922**	**7.52**	**11.53**	**13.77**
**Quartz**	**(-1,2,-8)**	**9.517**	**84.49**	**7.375**	**8.2**	**0.922**	**7.52**	**11.53**	**13.76**
**Quartz**	**(6,0,2)**	**9.038**	**70.96**	**6.281**	**5.5**	**3.3**	**18**	**41.25**	**45**
	Equiv. Refl.: (6,-6,-2), (-6,0,-2), (-6,6,2)								
**Quartz**	**(0,6,-2)**	**9.038**	**70.96**	**6.279**	**5.5**	**3.3**	**18**	**41.25**	**45**
	Equiv. Refl.: (0,-6,2)								
**Quartz**	**(-7,3,3)**	**9.507**	**83.91**	**6.122**	**6**	**1.02**	**6.12**	**12.75**	**14.14**
	Equiv. Refl.: (-7,4,-3)								
**Quartz**	**(4,3,-3)**	**9.507**	**83.91**	**6.117**	**6**	**1.02**	**6.11**	**12.75**	**14.14**
**Quartz**	**(4,-7,3)**	**9.507**	**83.91**	**6.115**	**6**	**1.02**	**6.11**	**12.75**	**14.13**
**Quartz**	**(3,-7,-3)**	**9.507**	**83.91**	**6.114**	**6**	**1.02**	**6.11**	**12.75**	**14.13**
**Quartz**	**(3,4,3)**	**9.507**	**83.91**	**6.113**	**6**	**1.02**	**6.11**	**12.75**	**14.13**
**Sapph**	**(1,3,16)**	**9.367**	**78.44**	**5.939**	**5.7**	**1.956**	**11.19**	**24.45**	**26.89**
	Equiv. Refl.: (1,-4,-16), (3,1,-16), (3,-4,16), (4,-1,-16), (4,-3,16), (-1,4,16), (-1,-3,-16), (-3,4,-16), (-3,-1,16) (-4,1,16), (-4,3,-16)								
**LiNbO3**	**(1,6,-5)**	**9.388**	**79.09**	**5.48**	**8**	**1.843**	**14.74**	**23.04**	**27.35**
	Equiv. Refl.: (1,-7,5), (6,1,5), (6,-7,-5), (7,-1,5), (7,-6,-5), (-1,7,-5), (-1,-6,5), (-6,7,5), (-6,-1,-5) (-7,1,-5), (-7,6,5)								
**Quartz**	**(7,-4,3)**	**9.507**	**83.91**	**5.189**	**6**	**1.02**	**6.12**	**12.75**	**14.14**
**Quartz**	**(7,-3,-3)**	**9.507**	**83.91**	**5.188**	**6**	**1.02**	**6.12**	**12.75**	**14.14**
**Quartz**	**(-4,-3,3)**	**9.507**	**83.91**	**5.183**	**6**	**1.02**	**6.11**	**12.75**	**14.14**
**Quartz**	**(-4,7,-3)**	**9.507**	**83.91**	**5.182**	**6**	**1.02**	**6.11**	**12.75**	**14.13**
**Quartz**	**(-3,7,3)**	**9.507**	**83.91**	**5.181**	**6**	**1.02**	**6.11**	**12.75**	**14.13**
**Quartz**	**(-3,-4,-3)**	**9.507**	**83.91**	**5.18**	**6**	**1.02**	**6.11**	**12.75**	**14.13**
**Quartz**	**(0,3,-7)**	**9.142**	**72.97**	**4.945**	**4.4**	**2.929**	**12.79**	**36.61**	**38.78**
	Equiv. Refl.: (0,-3,7)								
**Quartz**	**(3,0,7)**	**9.142**	**72.97**	**4.944**	**4.4**	**2.929**	**12.79**	**36.61**	**38.78**
**Quartz**	**(-3,0,-7)**	**9.142**	**72.97**	**4.943**	**4.4**	**2.929**	**12.79**	**36.61**	**38.78**
**Quartz**	**(3,-3,-7)**	**9.142**	**72.97**	**4.942**	**4.4**	**2.929**	**12.79**	**36.61**	**38.78**
	Equiv. Refl.: (-3,3,7)								
**Sapph**	**(0,4,14)**	**8.992**	**70.14**	**4.684**	**4.3**	**3.453**	**14.7**	**43.17**	**45.6**
	Equiv. Refl.: (0,-4,-14), (4,0,-14), (4,-4,14), (-4,0,14), (-4,4,-14)								
**Quartz**	**(4,-2,7)**	**9.484**	**82.71**	**4.579**	**4.7**	**1.223**	**5.73**	**15.29**	**16.33**
**Quartz**	**(4,-2,-7)**	**9.484**	**82.71**	**4.578**	**4.7**	**1.223**	**5.73**	**15.29**	**16.33**
**Quartz**	**(-2,-2,-7)**	**9.484**	**82.71**	**4.577**	**4.7**	**1.223**	**5.73**	**15.29**	**16.33**
**Quartz**	**(-2,4,7)**	**9.484**	**82.71**	**4.573**	**4.7**	**1.223**	**5.73**	**15.29**	**16.33**
**Quartz**	**(-2,4,-7)**	**9.484**	**82.71**	**4.571**	**4.7**	**1.223**	**5.73**	**15.29**	**16.33**
	Equiv. Refl.: (-2,-2,7)								
**Quartz**	**(6,0,3)**	**9.395**	**79.3**	**4.531**	**4.4**	**1.806**	**7.99**	**22.58**	**23.95**
	Equiv. Refl.: (-6,0,-3)								
**Quartz**	**(6,-6,-3)**	**9.395**	**79.3**	**4.53**	**4.4**	**1.806**	**7.99**	**22.58**	**23.95**
	Equiv. Refl.: (-6,6,3)								
**Quartz**	**(0,6,-3)**	**9.395**	**79.3**	**4.527**	**4.4**	**1.806**	**7.99**	**22.58**	**23.95**
	Equiv. Refl.: (0,-6,3)								
**Quartz**	**(0,4,-6)**	**9.018**	**70.6**	**4.179**	**3.7**	**3.367**	**12.43**	**42.08**	**43.88**
	Equiv. Refl.: (0,-4,6), (-4,0,-6)								
**Quartz**	**(4,0,6)**	**9.018**	**70.6**	**4.178**	**3.7**	**3.367**	**12.43**	**42.08**	**43.88**
	Equiv. Refl.: (4,-4,-6), (-4,4,6)								
**Quartz**	**(-4,2,-7)**	**9.484**	**82.71**	**4.17**	**4.7**	**1.223**	**5.73**	**15.29**	**16.33**

Ei = Cryst	9.561 Refl (h,k,l)	keV EB [keV]	ΘB [°]	∫ IR dΘ [µrad]	Width [µrad]	Ei cotΘ [meV/µrad]	ΔE [meV]	ΔEg 2m, 50µm [meV]	ΔEt 2m, 50µm [meV]
**Quartz**	**(-4,2,7)**	**9.484**	**82.71**	**4.169**	**4.7**	**1.223**	**5.73**	**15.29**	**16.33**
**Quartz**	**(2,2,7)**	**9.484**	**82.71**	**4.167**	**4.7**	**1.223**	**5.73**	**15.29**	**16.33**
**Quartz**	**(2,-4,-7)**	**9.484**	**82.71**	**4.163**	**4.7**	**1.223**	**5.73**	**15.29**	**16.33**
**Quartz**	**(2,2,-7)**	**9.484**	**82.71**	**4.161**	**4.7**	**1.223**	**5.73**	**15.29**	**16.33**
	Equiv. Refl.: (2,-4,7)								
**Sapph**	**(3,4,-4)**	**9.348**	**77.89**	**3.995**	**4.3**	**2.052**	**8.87**	**25.65**	**27.14**
	Equiv. Refl.: (3,-7,4), (4,3,4), (4,-7,-4), (7,-3,4), (7,-4,-4), (-3,7,-4), (-3,-4,4), (-4,7,4), (-4,-3,-4) (-7,3,-4), (-7,4,4)								
**LiNbO3**	**(2,4,13)**	**9.377**	**78.75**	**3.992**	**6.6**	**1.902**	**12.63**	**23.78**	**26.93**
	Equiv. Refl.: (2,-6,-13), (4,2,-13), (4,-6,13), (6,-2,-13), (6,-4,13), (-2,6,13), (-2,-4,-13), (-4,6,-13), (-4,-2,13) (-6,2,13), (-6,4,-13)								
**Sapph**	**(1,5,-7)**	**9.018**	**70.6**	**3.973**	**3.8**	**3.367**	**12.74**	**42.09**	**43.98**
	Equiv. Refl.: (1,-6,7), (5,1,7), (5,-6,-7), (6,-1,7), (6,-5,-7), (-1,6,-7), (-1,-5,7), (-5,6,7), (-5,-1,-7) (-6,1,-7), (-6,5,7)								
**Quartz**	**(-5,7,1)**	**9.171**	**73.58**	**3.805**	**3.5**	**2.818**	**9.78**	**35.23**	**36.56**
	Equiv. Refl.: (-5,-2,-1)								
**Quartz**	**(7,-2,1)**	**9.171**	**73.58**	**3.804**	**3.5**	**2.818**	**9.78**	**35.23**	**36.56**
	Equiv. Refl.: (7,-5,-1), (-2,7,-1), (-2,-5,1)								
**Quartz**	**(2,5,-1)**	**9.171**	**73.58**	**3.738**	**3.5**	**2.818**	**9.78**	**35.23**	**36.56**
	Equiv. Refl.: (2,-7,1), (5,2,1), (5,-7,-1), (-7,2,-1)								
**Quartz**	**(-7,5,1)**	**9.171**	**73.58**	**3.737**	**3.5**	**2.818**	**9.78**	**35.23**	**36.56**
**LiNbO3**	**(1,6,1)**	**9.129**	**72.71**	**3.64**	**5.4**	**2.976**	**16.18**	**37.2**	**40.57**
	Equiv. Refl.: (1,-7,-1), (6,1,-1), (6,-7,1), (7,-1,-1), (7,-6,1), (-1,7,1), (-1,-6,-1), (-6,7,-1), (-6,-1,1) (-7,1,1), (-7,6,-1)								
**Quartz**	**(0,1,8)**	**9.291**	**76.35**	**3.32**	**3.2**	**2.321**	**7.4**	**29.02**	**29.94**
	Equiv. Refl.: (0,-1,-8)								
**Quartz**	**(1,-1,8)**	**9.291**	**76.35**	**3.317**	**3.2**	**2.321**	**7.39**	**29.02**	**29.94**
	Equiv. Refl.: (-1,1,-8)								
**Quartz**	**(1,0,-8)**	**9.291**	**76.35**	**3.315**	**3.2**	**2.321**	**7.39**	**29.02**	**29.94**
	Equiv. Refl.: (-1,0,8)								
**Sapph**	**(0,6,6)**	**9.47**	**82.08**	**1.844**	**2.9**	**1.329**	**3.87**	**16.62**	**17.06**
	Equiv. Refl.: (0,6,-6), (0,-6,6), (0,-6,-6), (6,0,6), (6,0,-6), (6,-6,6), (6,-6,-6), (-6,0,6), (-6,0,-6) (-6,6,6), (-6,6,-6)								
**Sapph**	**(2,3,-13)**	**9.028**	**70.78**	**1.735**	**2.2**	**3.334**	**7.5**	**41.68**	**42.35**
	Equiv. Refl.: (2,-5,13), (3,2,13), (3,-5,-13), (5,-2,13), (5,-3,-13), (-2,5,-13), (-2,-3,13), (-3,5,13), (-3,-2,-13) (-5,2,-13), (-5,3,13)								
**Quartz**	**(5,-2,6)**	**9.365**	**78.37**	**1.725**	**2.2**	**1.968**	**4.42**	**24.6**	**25**
	Equiv. Refl.: (5,-3,-6), (-3,-2,-6)								
**Quartz**	**(-3,5,6)**	**9.365**	**78.37**	**1.724**	**2.2**	**1.968**	**4.42**	**24.6**	**25**
**Quartz**	**(-2,-3,6)**	**9.365**	**78.37**	**1.721**	**2.2**	**1.968**	**4.41**	**24.6**	**24.99**
**Quartz**	**(-2,5,-6)**	**9.365**	**78.37**	**1.72**	**2.2**	**1.968**	**4.41**	**24.6**	**24.99**
**Quartz**	**(7,-4,-2)**	**9.155**	**73.23**	**1.706**	**2.1**	**2.88**	**5.97**	**36**	**36.5**
**Quartz**	**(7,-3,2)**	**9.155**	**73.23**	**1.705**	**2.1**	**2.88**	**5.97**	**36**	**36.5**
	Equiv. Refl.: (-3,7,-2), (-3,-4,2), (-4,7,2), (-4,-3,-2)								
**Quartz**	**(0,5,5)**	**9.272**	**75.87**	**1.633**	**2.1**	**2.408**	**5.16**	**30.1**	**30.54**
	Equiv. Refl.: (0,-5,-5)								
**Quartz**	**(5,-5,5)**	**9.272**	**75.87**	**1.627**	**2.1**	**2.408**	**5.15**	**30.1**	**30.53**
	Equiv. Refl.: (-5,5,-5)								
**Quartz**	**(5,0,-5)**	**9.272**	**75.87**	**1.626**	**2.1**	**2.408**	**5.15**	**30.1**	**30.53**
	Equiv. Refl.: (-5,0,5)								

Ei =	9.561	keV						ΔEg	ΔEt
**Cryst**	**Refl**	**EB**	**ΘB**	**∫ IR dΘ**	**Width**	**Ei cotΘ**	**ΔE**	**2m, 50µm**	**2m, 50µm**
	**(h,k,l)**	**[keV]**	**[°]**	**[µrad]**	**[µrad]**	**[meV/µrad]**	**[meV]**	**[meV]**	**[meV]**
**LiNbO3**	**(1,3,-17)**	**9.106**	**72.26**	**1.611**	**3.3**	**3.059**	**10.09**	**38.23**	**39.54**
	Equiv. Refl.: (1,-4,17), (3,1,17), (3,-4,-17), (4,-1,17), (4,-3,-17), (-1,4,-17), (-1,-3,17), (-3,4,17), (-3,-1,-17) (-4,1,-17), (-4,3,17)								
**Quartz**	**(3,4,-2)**	**9.155**	**73.23**	**1.569**	**2.1**	**2.88**	**5.97**	**36**	**36.5**
	Equiv. Refl.: (3,-7,2), (4,3,2), (4,-7,-2), (-7,3,-2), (-7,4,2)								
**Quartz**	**(-5,2,-6)**	**9.365**	**78.37**	**1.558**	**2.2**	**1.968**	**4.42**	**24.6**	**25**
**Quartz**	**(3,2,6)**	**9.365**	**78.37**	**1.557**	**2.2**	**1.968**	**4.42**	**24.6**	**25**
	Equiv. Refl.: (-5,3,6)								
**Quartz**	**(3,-5,-6)**	**9.365**	**78.37**	**1.555**	**2.2**	**1.968**	**4.42**	**24.6**	**25**
**Quartz**	**(2,3,-6)**	**9.365**	**78.37**	**1.553**	**2.2**	**1.968**	**4.41**	**24.6**	**24.99**
	Equiv. Refl.: (2,-5,6)								
**LiNbO3**	**(1,4,15)**	**9.252**	**75.39**	**1.521**	**3.4**	**2.493**	**8.52**	**31.16**	**32.3**
	Equiv. Refl.: (1,4,-15), (1,-5,15), (1,-5,-15), (4,1,15), (4,1,-15), (4,-5,15), (4,-5,-15), (5,-1,15), (5,-1,-15) (5,-4,15), (5,-4,-15), (-1,5,15), (-1,5,-15), (-1,-4,15), (-1,-4,-15), (-4,5,15), (-4,5,-15), (-4,-1,15) (-4,-1,-15), (-5,1,15), (-5,1,-15), (-5,4,15), (-5,4,-15)								
**LiNbO3**	**(3,4,-7)**	**9.019**	**70.61**	**1.482**	**3**	**3.365**	**10.24**	**42.07**	**43.29**
	Equiv. Refl.: (3,-7,7), (4,3,7), (4,-7,-7), (7,-3,7), (7,-4,-7), (-3,7,-7), (-3,-4,7), (-4,7,7), (-4,-3,-7) (-7,3,-7), (-7,4,7)								
**Sapph**	**(1,2,17)**	**9.038**	**70.96**	**1.351**	**1.9**	**3.301**	**6.16**	**41.26**	**41.71**
	Equiv. Refl.: (1,-3,-17), (2,1,-17), (2,-3,17), (3,-1,-17), (3,-2,17), (-1,3,17), (-1,-2,-17), (-2,3,-17), (-2,-1,17) (-3,1,17), (-3,2,-17)								
**Quartz**	**(0,6,2)**	**9.038**	**70.96**	**1.177**	**1.6**	**3.3**	**5.15**	**41.25**	**41.57**
	Equiv. Refl.: (0,-6,-2), (-6,0,2), (-6,6,-2)								
**Quartz**	**(6,0,-2)**	**9.038**	**70.96**	**1.176**	**1.6**	**3.3**	**5.15**	**41.25**	**41.57**
	Equiv. Refl.: (6,-6,2)								
**Sapph**	**(1,5,8)**	**9.206**	**74.33**	**1.134**	**1.7**	**2.683**	**4.62**	**33.53**	**33.85**
	Equiv. Refl.: (1,-6,-8), (5,1,-8), (5,-6,8), (6,-1,-8), (6,-5,8), (-1,6,8), (-1,-5,-8), (-5,6,-8), (-5,-1,8) (-6,1,8), (-6,5,-8)								
**LiNbO3**	**(1,5,11)**	**9.172**	**73.61**	**1.13**	**2.8**	**2.812**	**7.75**	**35.15**	**36**
	Equiv. Refl.: (1,-6,-11), (5,1,-11), (5,-6,11), (6,-1,-11), (6,-5,11), (-1,6,11), (-1,-5,-11), (-5,6,-11), (-5,-1,11) (-6,1,11), (-6,5,-11)								
**LiNbO3**	**(1,2,-19)**	**9.259**	**75.55**	**1.119**	**2.9**	**2.463**	**7.09**	**30.79**	**31.6**
	Equiv. Refl.: (1,-3,19), (2,1,19), (2,-3,-19), (3,-1,19), (3,-2,-19), (-1,3,-19), (-1,-2,19), (-2,3,19), (-2,-1,-19) (-3,1,-19), (-3,2,19)								
**Quartz**	**(2,-5,-6)**	**9.365**	**78.37**	**0.69**	**1.1**	**1.968**	**2.19**	**24.6**	**24.7**
**Quartz**	**(2,3,6)**	**9.365**	**78.37**	**0.688**	**1.1**	**1.968**	**2.19**	**24.6**	**24.7**
**Quartz**	**(-5,2,6)**	**9.365**	**78.37**	**0.687**	**1.1**	**1.968**	**2.19**	**24.6**	**24.7**
	Equiv. Refl.: (-5,3,-6)								
**Quartz**	**(3,2,-6)**	**9.365**	**78.37**	**0.682**	**1.1**	**1.968**	**2.18**	**24.6**	**24.7**
	Equiv. Refl.: (3,-5,6)								
**Sapph**	**(3,4,5)**	**9.457**	**81.55**	**0.549**	**1.4**	**1.421**	**1.93**	**17.77**	**17.87**
	Equiv. Refl.: (3,-7,-5), (4,3,-5), (4,-7,5), (7,-3,-5), (7,-4,5), (-3,7,5), (-3,-4,-5), (-4,7,-5), (-4,-3,5) (-7,3,5), (-7,4,-5)								
**Quartz**	**(-2,5,6)**	**9.365**	**78.37**	**0.497**	**1.1**	**1.968**	**2.19**	**24.6**	**24.7**
**Quartz**	**(-2,-3,-6)**	**9.365**	**78.37**	**0.496**	**1.1**	**1.968**	**2.19**	**24.6**	**24.7**
**Quartz**	**(5,-2,-6)**	**9.365**	**78.37**	**0.495**	**1.1**	**1.968**	**2.19**	**24.6**	**24.7**
	Equiv. Refl.: (5,-3,6)								
**Quartz**	**(-3,5,-6)**	**9.365**	**78.37**	**0.491**	**1.1**	**1.968**	**2.18**	**24.6**	**24.7**
	Equiv. Refl.: (-3,-2,6)								
**Quartz**	**(-1,6,-4)**	**9.32**	**77.1**	**0.346**	**0.7**	**2.19**	**1.63**	**27.37**	**27.42**
	Equiv. Refl.: (-1,-5,4)								
**Quartz**	**(6,-5,-4)**	**9.32**	**77.1**	**0.345**	**0.7**	**2.19**	**1.63**	**27.37**	**27.42**

**Quartz**	**(6,-1,4)**	**9.32**	**77.1**	**0.344**	**0.7**	**2.19**	**1.62**	**27.37**	**27.42**
**Quartz**	**(-5,6,4)**	**9.32**	**77.1**	**0.342**	**0.7**	**2.19**	**1.62**	**27.37**	**27.42**
	Equiv. Refl.: (-5,-1,-4)								
**Quartz**	**(1,5,-4)**	**9.32**	**77.1**	**0.331**	**0.7**	**2.19**	**1.63**	**27.37**	**27.42**
	Equiv. Refl.: (1,-6,4)								
**Quartz**	**(-6,1,-4)**	**9.32**	**77.1**	**0.329**	**0.7**	**2.19**	**1.62**	**27.37**	**27.42**
	Equiv. Refl.: (-6,5,4)								
**Quartz**	**(5,1,4)**	**9.32**	**77.1**	**0.326**	**0.7**	**2.19**	**1.62**	**27.37**	**27.42**
	Equiv. Refl.: (5,-6,-4)								
**Quartz**	**(7,-2,0)**	**9.099**	**72.11**	**0.325**	**0.6**	**3.086**	**1.93**	**38.57**	**38.62**
	Equiv. Refl.: (7,-5,0), (-2,7,0), (-2,-5,0), (-5,7,0), (-5,-2,0)								
**Sapph**	**(3,4,-1)**	**9.163**	**73.42**	**0.319**	**0.8**	**2.847**	**2.16**	**35.58**	**35.65**
	Equiv. Refl.: (3,-7,1), (4,3,1), (4,-7,-1), (7,-3,1), (7,-4,-1), (-3,7,-1), (-3,-4,1), (-4,7,1), (-4,-3,-1) (-7,3,-1), (-7,4,1)								
**Quartz**	**(2,5,0)**	**9.099**	**72.11**	**0.265**	**0.6**	**3.086**	**1.93**	**38.57**	**38.62**
	Equiv. Refl.: (2,-7,0), (5,2,0), (5,-7,0), (-7,2,0), (-7,5,0)								

Ei = Cryst	11.682 Refl (h,k,l)	keV EB [keV]	ΘB [°]	∫ IR dΘ [µrad]	Width [µrad]	Ei cotΘ [meV/µrad]	ΔE [meV]	ΔEg 2m, 50µm [meV]	ΔEt 2m, 50µm [meV]
**Si**	**(0,2,10)**	**11.64**	**85.17**	**14.931**	**13**	**0.987**	**12.8**	**12.34**	**17.78**
	Equiv. Refl.: (2,6,8)								
**Si**	**(4,4,8)**	**11.184**	**73.21**	**5.229**	**4.3**	**3.525**	**15.14**	**44.07**	**46.6**
**Si**	**(1,7,7)**	**11.357**	**76.46**	**3.993**	**3.6**	**2.814**	**10.04**	**35.17**	**36.57**
	Equiv. Refl.: (3,3,9), (5,5,7)								
**Ge**	**(0,2,10)**	**11.175**	**73.05**	**3.417**	**7**	**3.559**	**24.91**	**44.49**	**50.99**
	Equiv. Refl.: (2,6,8)								
**Ge**	**(1,5,9)**	**11.335**	**76**	**2.052**	**5.7**	**2.913**	**16.55**	**36.42**	**40**
	Equiv. Refl.: (3,7,7)								
**LiNbO3**	**(0,-6,18)**	**11.593**	**82.92**	**25**	**22.7**	**1.452**	**32.98**	**18.15**	**37.65**
	Equiv. Refl.: (6,0,18), (-6,6,18)								
**LiNbO3**	**(0,6,-18)**	**11.593**	**82.92**	**24.5**	**22.7**	**1.452**	**32.98**	**18.15**	**37.65**
	Equiv. Refl.: (6,-6,-18), (-6,0,-18)								
**LiNbO3**	**(0,6,18)**	**11.593**	**82.92**	**22.9**	**20.8**	**1.452**	**30.24**	**18.15**	**35.27**
	Equiv. Refl.: (6,-6,18), (-6,0,18)								
**LiNbO3**	**(3,-8,14)**	**11.573**	**82.16**	**22.4**	**19.8**	**1.608**	**31.81**	**20.09**	**37.63**
	Equiv. Refl.: (5,3,14), (8,-3,14), (-3,-5,14), (-5,8,14), (-8,5,14)								
**LiNbO3**	**(1,6,16)**	**11.59**	**82.81**	**22.2**	**20.1**	**1.474**	**29.6**	**18.43**	**34.86**
	Equiv. Refl.: (6,-7,16), (7,-6,16), (-1,7,16), (-6,-1,16), (-7,1,16)								
**LiNbO3**	**(2,-5,22)**	**11.555**	**81.55**	**22**	**19.7**	**1.736**	**34.22**	**21.7**	**40.52**
	Equiv. Refl.: (3,2,22), (5,-2,22), (-2,-3,22), (-3,5,22), (-5,3,22)								
**LiNbO3**	**(0,-6,-18)**	**11.593**	**82.92**	**21.9**	**20.8**	**1.452**	**30.24**	**18.15**	**35.27**
	Equiv. Refl.: (6,0,-18), (-6,6,-18)								
**LiNbO3**	**(2,-6,20)**	**11.581**	**82.47**	**21.8**	**19.9**	**1.545**	**30.8**	**19.31**	**36.35**
	Equiv. Refl.: (4,2,20), (6,-2,20), (-2,-4,20), (-4,6,20), (-6,4,20)								
**LiNbO3**	**(2,4,-20)**	**11.581**	**82.47**	**21.2**	**19.9**	**1.545**	**30.8**	**19.31**	**36.35**
	Equiv. Refl.: (4,-6,-20), (6,-4,-20), (-2,6,-20), (-4,-2,-20), (-6,2,-20)								
**LiNbO3**	**(2,3,-22)**	**11.555**	**81.55**	**21.1**	**19.7**	**1.736**	**34.22**	**21.7**	**40.52**
	Equiv. Refl.: (3,-5,-22), (5,-3,-22), (-2,5,-22), (-3,-2,-22), (-5,2,-22)								
**LiNbO3**	**(1,-7,-16)**	**11.59**	**82.81**	**20.9**	**20.1**	**1.474**	**29.6**	**18.43**	**34.86**
	Equiv. Refl.: (6,1,-16), (7,-1,-16), (-1,-6,-16), (-6,7,-16), (-7,6,-16)								
**LiNbO3**	**(3,5,-14)**	**11.573**	**82.16**	**20.5**	**19.8**	**1.608**	**31.81**	**20.09**	**37.63**
	Equiv. Refl.: (5,-8,-14), (8,-5,-14), (-3,8,-14), (-5,-3,-14), (-8,3,-14)								
**LiNbO3**	**(0,-7,-14)**	**11.573**	**82.16**	**18**	**16.9**	**1.608**	**27.11**	**20.09**	**33.74**
	Equiv. Refl.: (7,0,-14), (-7,7,-14)								
**LiNbO3**	**(0,7,14)**	**11.573**	**82.16**	**17.6**	**16.9**	**1.608**	**27.11**	**20.09**	**33.74**
	Equiv. Refl.: (7,-7,14), (-7,0,14)								
**Sapph**	**(0,7,-10)**	**11.562**	**81.78**	**16**	**13.2**	**1.687**	**22.24**	**21.09**	**30.65**
	Equiv. Refl.: (0,-7,10), (7,0,10), (7,-7,-10), (-7,0,-10), (-7,7,10)								
**LiNbO3**	**(0,-8,-4)**	**11.267**	**74.68**	**15.3**	**13.5**	**3.201**	**43.09**	**40.01**	**58.8**
	Equiv. Refl.: (2,7,-2), (2,-9,2), (7,2,2), (7,-9,-2), (8,0,-4), (9,-2,2), (9,-7,-2), (-2,9,-2), (-2,-7,2) (-7,9,2), (-7,-2,-2), (-8,8,-4), (-9,2,-2), (-9,7,2)								
**Sapph**	**(3,3,18)**	**11.615**	**83.88**	**15**	**12.7**	**1.253**	**15.94**	**15.66**	**22.34**
	Equiv. Refl.: (3,3,-18), (3,-6,18), (3,-6,-18), (6,-3,18), (6,-3,-18), (-3,6,18), (-3,6,-18), (-3,-3,18), (-3,-3,-18) (-6,3,18), (-6,3,-18)								
**LiNbO3**	**(0,8,4)**	**11.267**	**74.68**	**14.9**	**13.5**	**3.201**	**43.09**	**40.01**	**58.8**
	Equiv. Refl.: (8,-8,4), (-8,0,4)								

Ei =	11.682	keV						ΔEg	ΔEt
**Cryst**	**Refl**	**EB**	**ΘB**	**∫ IR dΘ**	**Width**	**Ei cotΘ**	**ΔE**	**2m, 50µm**	**2m, 50µm**
	**(h,k,l)**	**[keV]**	**[°]**	**[µrad]**	**[µrad]**	**[meV/µrad]**	**[meV]**	**[meV]**	**[meV]**
**LiNbO3**	**(2,7,4)**	**11.521**	**80.49**	**14.8**	**13.7**	**1.958**	**26.74**	**24.47**	**36.25**
	Equiv. Refl.: (7,-9,4), (9,-7,4), (-2,9,4), (-7,-2,4), (-9,2,4)								
**LiNbO3**	**(2,-9,-4)**	**11.521**	**80.49**	**13.9**	**13.7**	**1.958**	**26.74**	**24.47**	**36.25**
	Equiv. Refl.: (7,2,-4), (9,-2,-4), (-2,-7,-4), (-7,9,-4), (-9,7,-4)								
**LiNbO3**	**(3,6,-6)**	**11.358**	**76.48**	**12.6**	**11.7**	**2.81**	**32.76**	**35.12**	**48.03**
	Equiv. Refl.: (3,-9,6), (6,3,6), (6,-9,-6), (9,-3,6), (9,-6,-6), (-3,9,-6), (-3,-6,6), (-6,9,6), (-6,-3,-6) (-9,3,-6), (-9,6,6)								
**LiNbO3**	**(3,-9,-6)**	**11.358**	**76.48**	**11.8**	**10.9**	**2.81**	**30.49**	**35.12**	**46.51**
	Equiv. Refl.: (6,3,-6), (9,-3,-6), (-3,-6,-6), (-6,9,-6), (-9,6,-6)								
**LiNbO3**	**(0,-3,-24)**	**11.514**	**80.28**	**11.7**	**10.9**	**2**	**21.85**	**25**	**33.2**
	Equiv. Refl.: (3,0,-24), (-3,3,-24)								
**LiNbO3**	**(0,3,-24)**	**11.514**	**80.28**	**11.5**	**10.8**	**2**	**21.59**	**25**	**33.04**
	Equiv. Refl.: (3,-3,-24), (-3,0,-24)								
**LiNbO3**	**(3,6,6)**	**11.358**	**76.48**	**11.4**	**10.9**	**2.81**	**30.49**	**35.12**	**46.51**
	Equiv. Refl.: (6,-9,6), (9,-6,6), (-3,9,6), (-6,-3,6), (-9,3,6)								
**LiNbO3**	**(4,5,8)**	**11.434**	**78.18**	**11.3**	**10.5**	**2.446**	**25.61**	**30.57**	**39.88**
	Equiv. Refl.: (5,-9,8), (9,-5,8), (-4,9,8), (-5,-4,8), (-9,4,8)								
**LiNbO3**	**(4,-9,-8)**	**11.434**	**78.18**	**10.9**	**10.5**	**2.446**	**25.61**	**30.57**	**39.88**
	Equiv. Refl.: (5,4,-8), (9,-4,-8), (-4,-5,-8), (-5,9,-8), (-9,5,-8)								
**LiNbO3**	**(1,1,24)**	**10.999**	**70.31**	**10.8**	**9.7**	**4.18**	**40.57**	**52.25**	**66.15**
	Equiv. Refl.: (1,-2,24), (2,-1,24), (-1,2,24), (-1,-1,24), (-2,1,24)								
**LiNbO3**	**(0,3,24)**	**11.514**	**80.28**	**10.7**	**10.9**	**2**	**21.85**	**25**	**33.2**
	Equiv. Refl.: (0,4,-22), (1,1,-24), (1,-2,-24), (2,-1,-24), (3,-3,24), (4,-4,-22), (-1,2,-24), (-1,-1,-24), (-2,1,-24) (-3,0,24), (-4,0,-22)								
**LiNbO3**	**(0,-3,24)**	**11.514**	**80.28**	**10.6**	**10.8**	**2**	**21.59**	**25**	**33.04**
	Equiv. Refl.: (3,0,24), (-3,3,24)								
**LiNbO3**	**(0,-4,22)**	**11.301**	**75.33**	**10.3**	**9.7**	**3.058**	**29.58**	**38.22**	**48.33**
	Equiv. Refl.: (4,0,22), (-4,4,22)								
**LiNbO3**	**(1,6,-14)**	**11.06**	**71.22**	**9.8**	**8.6**	**3.971**	**34.1**	**49.64**	**60.22**
	Equiv. Refl.: (6,-7,-14), (7,-6,-14), (-1,7,-14), (-6,-1,-14), (-7,1,-14)								
**LiNbO3**	**(0,5,-20)**	**11.328**	**75.86**	**9.5**	**8.4**	**2.943**	**24.82**	**36.79**	**44.38**
	Equiv. Refl.: (5,-5,-20), (-5,0,-20), (0,8,0), (0,-8,0), (8,0,0), (8,-8,0), (-8,0,0), (-8,8,0)								
**LiNbO3**	**(3,6,0)**	**11.037**	**70.87**	**9.4**	**8.4**	**4.053**	**33.95**	**50.66**	**60.99**
	Equiv. Refl.: (6,-9,0), (9,-6,0), (-3,9,0), (-6,-3,0), (-9,3,0)								
**LiNbO3**	**(1,-7,14)**	**11.06**	**71.22**	**9.2**	**8.6**	**3.971**	**34.1**	**49.64**	**60.22**
	Equiv. Refl.: (3,-9,0), (6,1,14), (6,3,0), (7,-1,14), (9,-3,0), (-1,-6,14), (-3,-6,0), (-6,7,14), (-6,9,0) (-7,6,14), (-9,6,0)								
**Quartz**	**(4,-1,-9)**	**11.583**	**82.53**	**9.1**	**7.5**	**1.531**	**11.45**	**19.14**	**22.3**
**LiNbO3**	**(3,-7,16)**	**11.078**	**71.5**	**9**	**8**	**3.909**	**31.41**	**48.86**	**58.08**
	Equiv. Refl.: (4,3,16), (4,4,12), (4,-8,12), (4,-9,4), (5,4,4), (7,-3,16), (8,-4,12), (9,-4,4), (-3,-4,16) (-4,7,16), (-4,8,12), (-4,-4,12), (-4,-5,4), (-5,9,4), (-7,4,16), (-8,4,12), (-9,5,4), (1,5,17) (1,-6,-17), (5,1,-17), (5,-6,17), (6,-1,-17), (6,-5,17), (-1,6,17), (-1,-5,-17), (-5,6,-17), (-5,-1,17) (-6,1,17), (-6,5,-17), (4,-3,9), (-1,4,9), (-1,-3,-9), (-3,4,-9), (-3,-1,9)								
**Quartz**	**(1,3,9)**	**11.583**	**82.53**	**8.9**	**7.5**	**1.531**	**11.44**	**19.14**	**22.3**
	Equiv. Refl.: (1,-4,-9), (3,1,-9), (3,-4,9), (-4,1,9), (-4,3,-9)								
**LiNbO3**	**(2,-8,10)**	**10.979**	**70.02**	**8.7**	**7.8**	**4.247**	**33.01**	**53.09**	**62.51**
	Equiv. Refl.: (6,2,10), (8,-2,10), (-2,-6,10), (-6,8,10), (-8,6,10)								
**LiNbO3**	**(4,4,-12)**	**11.027**	**70.73**	**8.6**	**8**	**4.085**	**32.81**	**51.06**	**60.69**
	Equiv. Refl.: (4,-8,-12), (8,-4,-12), (-4,8,-12), (-4,-4,-12), (-8,4,-12)								

Ei =	11.682	keV						ΔEg	ΔEt
**Cryst**	**Refl**	**EB**	**ΘB**	**∫ IR dΘ**	**Width**	**Ei cotΘ**	**ΔE**	**2m, 50μm**	**2m, 50μm**
	**(h,k,l)**	**[keV]**	**[°]**	**[μrad]**	**[μrad]**	**[meV/μrad]**	**[meV]**	**[meV]**	**[meV]**
**LiNbO3**	**(3,4,-16)**	**11.078**	**71.5**	**8.5**	**8**	**3.909**	**31.41**	**48.86**	**58.08**
	Equiv. Refl.: (4,5,-4), (4,-7,-16), (5,-9,-4), (7,-4,-16), (9,-5,-4), (-3,7,-16), (-4,9,-4), (-4,-3,-16), (-5,-4,-4) (-7,3,-16), (-9,4,-4)								
**LiNbO3**	**(1,-4,-22)**	**11.042**	**70.94**	**8.4**	**7.6**	**4.036**	**30.81**	**50.45**	**59.11**
	Equiv. Refl.: (2,6,-10), (3,1,-22), (4,-1,-22), (6,-8,-10), (8,-6,-10), (-1,-3,-22), (-2,8,-10), (-3,4,-22), (-4,3,-22) (-6,-2,-10), (-8,2,-10)								
**Quartz**	**(4,5,-2)**	**11.608**	**83.56**	**8.3**	**7**	**1.318**	**9.18**	**16.47**	**18.86**
	Equiv. Refl.: (4,-9,2), (5,4,2), (5,-9,-2), (-9,4,-2), (-9,5,2)								
**LiNbO3**	**(0,-5,20)**	**11.328**	**75.86**	**8.2**	**8.4**	**2.943**	**24.82**	**36.79**	**44.38**
	Equiv. Refl.: (1,3,22), (3,-4,22), (4,-3,22), (5,0,20), (-1,4,22), (-3,-1,22), (-4,1,22), (-5,5,20), (4,-1,9) (4,-3,-9), (-1,4,-9), (-1,-3,9), (-3,4,9), (-3,-1,-9)								
**Quartz**	**(0,1,-10)**	**11.562**	**81.79**	**8.1**	**6.7**	**1.685**	**11.37**	**21.06**	**23.94**
	Equiv. Refl.: (0,-1,10), (1,0,10), (1,3,-9), (1,-1,-10), (1,-4,9), (3,1,9), (3,-4,-9), (9,-4,2), (9,-5,-2) (-1,0,-10), (-1,1,10), (-4,1,-9), (-4,3,9), (-4,9,-2), (-4,-5,2), (-5,9,2), (-5,-4,-2)								
**Sapph**	**(0,4,20)**	**11.284**	**75.01**	**7.9**	**6.4**	**3.129**	**19.89**	**39.11**	**43.88**
	Equiv. Refl.: (0,-4,-20), (4,0,-20), (4,-4,20), (-4,0,20), (-4,4,-20), (0,1,10), (0,-1,-10), (1,0,-10), (1,-1,10) (-1,0,10), (-1,1,-10)								
**Sapph**	**(1,2,23)**	**11.677**	**88.33**	**7**	**8.7**	**0.341**	**2.98**	**4.26**	**5.2**
	Equiv. Refl.: (1,-3,-23), (2,1,-23), (2,-3,23), (3,-1,-23), (3,-2,23), (-1,3,23), (-1,-2,-23), (-2,3,-23), (-2,-1,23) (-3,1,23), (-3,2,-23)								
**Sapph**	**(2,3,20)**	**11.581**	**82.47**	**6.9**	**6.1**	**1.544**	**9.46**	**19.3**	**21.5**
	Equiv. Refl.: (2,-5,-20), (3,2,-20), (3,-5,20), (5,-2,-20), (5,-3,20), (-2,5,20), (-2,-3,-20), (-3,5,-20), (-3,-2,20) (-5,2,20), (-5,3,-20)								
**Sapph**	**(3,5,10)**	**11.562**	**81.78**	**6.3**	**5.4**	**1.687**	**9.15**	**21.09**	**22.99**
	Equiv. Refl.: (3,-8,-10), (5,3,-10), (5,-8,10), (8,-3,-10), (8,-5,10), (-3,8,10), (-3,-5,-10), (-5,8,-10), (-5,-3,10) (-8,3,10), (-8,5,-10)								
**LiNbO3**	**(0,-8,2)**	**11.16**	**72.8**	**6.1**	**5.6**	**3.615**	**20.31**	**45.19**	**49.54**
	Equiv. Refl.: (8,0,2), (-8,8,2)								
**Quartz**	**(4,-2,9)**	**11.491**	**79.62**	**5.9**	**4.8**	**2.139**	**10.31**	**26.74**	**28.66**
	Equiv. Refl.: (4,-2,-9), (-2,4,9), (-2,4,-9), (-2,-2,9), (-2,-2,-9)								
**LiNbO3**	**(0,8,-2)**	**11.16**	**72.8**	**5.8**	**5.6**	**3.615**	**20.31**	**45.19**	**49.54**
	Equiv. Refl.: (8,-8,-2), (-8,0,-2)								
**Sapph**	**(2,6,-4)**	**11.015**	**70.55**	**5.7**	**4.7**	**4.126**	**19.19**	**51.57**	**55.02**
	Equiv. Refl.: (2,-8,4), (6,2,4), (6,-8,-4), (8,-2,4), (8,-6,-4), (-2,8,-4), (-2,-6,4), (-6,8,4), (-6,-2,-4) (-8,2,-4), (-8,6,4), (4,4,5), (4,4,-5), (4,-8,5), (4,-8,-5), (-8,4,5), (-8,4,-5)								
**Sapph**	**(0,7,8)**	**11.202**	**73.52**	**5.6**	**4.7**	**3.457**	**16.2**	**43.21**	**46.15**
	Equiv. Refl.: (0,-7,-8), (7,0,-8), (7,-7,8), (-7,0,8), (-7,7,-8), (2,2,9), (2,2,-9), (2,-4,9), (2,-4,-9) (8,-4,5), (8,-4,-5), (-4,2,9), (-4,2,-9), (-4,8,5), (-4,8,-5), (-4,-4,5), (-4,-4,-5)								
**Sapph**	**(1,3,-20)**	**10.979**	**70.03**	**5.5**	**4.5**	**4.246**	**18.99**	**53.07**	**56.37**
	Equiv. Refl.: (1,5,-16), (1,-4,20), (1,-6,16), (3,1,20), (3,-4,-20), (4,-1,20), (4,-3,-20), (5,1,16), (5,-6,-16) (6,-1,16), (6,-5,-16), (-1,4,-20), (-1,6,-16), (-1,-3,20), (-1,-5,16), (-3,4,20), (-3,-1,-20), (-4,1,-20) (-4,3,20), (-5,6,16), (-5,-1,-16), (-6,1,-16), (-6,5,16), (3,6,0), (3,6,-1), (3,-9,0), (3,-9,1) (6,3,0), (6,3,1), (6,-9,0), (6,-9,-1), (8,-1,-3), (8,-7,3), (9,-4,-1), (9,-5,1), (-1,8,3) (-1,-7,-3), (-4,9,1), (-4,-5,-1), (-5,9,-1), (-5,-4,1), (-7,8,-3), (-7,-1,3), (-9,3,0), (-9,3,-1) (-9,6,0), (-9,6,1)								
**Quartz**	**(0,7,4)**	**11.183**	**73.2**	**5.3**	**4.2**	**3.527**	**14.87**	**44.09**	**46.53**
	Equiv. Refl.: (0,-7,-4), (1,7,3), (1,-8,-3), (4,5,1), (4,-9,-1), (5,4,-1), (5,-9,1), (7,0,-4), (7,1,-3) (7,-7,4), (7,-8,3), (-7,0,4), (-7,7,-4), (-8,1,3), (-8,7,-3), (-9,4,1), (-9,5,-1)								
**Quartz**	**(1,7,-3)**	**11.526**	**80.62**	**5.2**	**4.2**	**1.931**	**8.03**	**24.13**	**25.43**
	Equiv. Refl.: (1,-8,3), (7,1,3), (7,-8,-3), (-8,1,-3), (-8,7,3)								
**Quartz**	**(2,6,-4)**	**11.465**	**78.93**	**5.1**	**4.1**	**2.286**	**9.29**	**28.57**	**30.05**
	Equiv. Refl.: (2,-8,4), (6,2,4), (6,-8,-4), (-8,2,-4), (-8,6,4)								

Ei =	11.682	keV						ΔEg	ΔEt
Cryst	Refl	EB	ΘB	∫ IR dΘ	Width	Ei cotΘ	ΔE	2m, 50µm	2m, 50µm
	(h,k,l)	[keV]	[°]	[µrad]	[µrad]	[meV/µrad]	[meV]	[meV]	[meV]
**Quartz**	**(9,-3,0)**	**11.564**	**81.87**	**5**	**4.5**	**1.67**	**7.5**	**20.87**	**22.18**
	Equiv. Refl.: (9,-6,0), (-3,9,0), (-3,-6,0), (-6,9,0), (-6,-3,0)								
**Sapph**	**(1,7,0)**	**11.358**	**76.48**	**4.9**	**4.1**	**2.81**	**11.53**	**35.12**	**36.97**
	Equiv. Refl.: (1,-8,0), (7,1,0), (7,-8,0), (8,-1,0), (8,-7,0), (-1,8,0), (-1,-7,0), (-7,8,0), (-7,-1,0) (-8,1,0), (-8,7,0), (8,-2,4), (8,-6,-4), (-2,8,-4), (-2,-6,4), (-6,8,4), (-6,-2,-4)								
**Quartz**	**(0,6,-6)**	**11.126**	**72.25**	**4.8**	**3.8**	**3.74**	**14.03**	**46.74**	**48.81**
	Equiv. Refl.: (0,-6,6), (6,0,6), (6,-6,-6), (-6,0,-6), (-6,6,6)								
**Quartz**	**(6,-1,7)**	**11.414**	**77.7**	**4.7**	**3.8**	**2.548**	**9.69**	**31.84**	**33.28**
	Equiv. Refl.: (6,-5,-7), (8,-1,3), (8,-7,-3), (9,-3,1), (9,-6,-1), (-1,6,-7), (-1,8,-3), (-1,-5,7), (-1,-7,3) (-3,9,-1), (-3,-6,1), (-6,9,1), (-6,-3,-1), (-7,8,3), (-7,-1,-3)								
**Quartz**	**(1,5,-7)**	**11.414**	**77.7**	**4.6**	**3.8**	**2.548**	**9.69**	**31.84**	**33.28**
	Equiv. Refl.: (1,-6,7), (5,1,7), (5,-6,-7), (-5,6,7), (-5,-1,-7), (-6,1,-7), (-6,5,7)								
**Quartz**	**(1,4,8)**	**11.348**	**76.27**	**4.1**	**3.3**	**2.854**	**9.51**	**35.68**	**36.93**
	Equiv. Refl.: (1,-5,-8), (4,1,-8), (4,-5,8), (-5,1,8), (-5,4,-8)								
**Quartz**	**(5,-1,-8)**	**11.348**	**76.27**	**4**	**3.3**	**2.854**	**9.51**	**35.68**	**36.93**
	Equiv. Refl.: (5,-4,8), (-1,5,8), (-1,-4,-8), (-4,5,-8), (-4,-1,8)								
**LiNbO3**	**(2,7,-5)**	**11.599**	**83.17**	**3.9**	**6**	**1.399**	**8.33**	**17.48**	**19.36**
	Equiv. Refl.: (2,-9,5), (7,2,5), (7,-9,-5), (9,-2,5), (9,-7,-5), (-2,9,-5), (-2,-7,5), (-7,9,5), (-7,-2,-5) (-9,2,-5), (-9,7,5), (7,-3,-6), (7,-4,6), (-3,7,6), (-3,-4,-6), (-4,7,-6), (-4,-3,6)								
**Quartz**	**(3,4,6)**	**11.221**	**73.85**	**3.8**	**3.1**	**3.383**	**10.64**	**42.29**	**43.61**
	Equiv. Refl.: (3,-7,-6), (4,3,-6), (4,-7,6), (-7,3,6), (-7,4,-6)								
**Sapph**	**(2,2,21)**	**11.297**	**75.25**	**3.7**	**3.2**	**3.076**	**9.87**	**38.45**	**39.69**
	Equiv. Refl.: (2,2,-21), (2,-4,21), (2,-4,-21), (4,-2,21), (4,-2,-21), (-2,4,21), (-2,4,-21), (-2,-2,21), (-2,-2,-21) (-4,2,21), (-4,2,-21), (2,4,-7), (2,-6,7), (4,2,7), (4,-6,-7), (-6,2,-7), (-6,4,7)								
**Quartz**	**(6,-2,7)**	**11.131**	**72.34**	**3.6**	**3**	**3.72**	**11.09**	**46.5**	**47.8**
	Equiv. Refl.: (6,-4,-7), (-2,6,-7), (-2,-4,7), (-4,6,7), (-4,-2,-7)								
**Quartz**	**(4,5,-1)**	**11.437**	**78.25**	**3.5**	**3**	**2.43**	**7.25**	**30.38**	**31.23**
	Equiv. Refl.: (4,-9,1), (5,4,1), (5,-9,-1), (-9,4,-1), (-9,5,1)								
**Quartz**	**(9,-4,1)**	**11.437**	**78.25**	**3.4**	**3**	**2.43**	**7.25**	**30.38**	**31.23**
	Equiv. Refl.: (9,-5,-1), (-4,9,-1), (-4,-5,1), (-5,9,1), (-5,-4,-1)								
**Sapph**	**(2,4,16)**	**11.031**	**70.79**	**3.3**	**2.7**	**4.071**	**10.99**	**50.89**	**52.06**
	Equiv. Refl.: (2,-6,-16), (4,2,-16), (4,-6,16), (6,-2,-16), (6,-4,16), (-2,6,16), (-2,-4,-16), (-4,6,-16), (-4,-2,16) (-6,2,16), (-6,4,-16), (1,6,5), (1,7,0), (1,-7,-5), (1,-8,0), (2,6,-3), (2,-8,3), (4,5,2) (4,-9,-2), (5,4,-2), (5,-9,2), (6,1,-5), (6,2,3), (6,-7,5), (6,-8,-3), (7,1,0), (7,-8,0) (8,-1,0), (8,-2,3), (8,-6,-3), (8,-7,0), (9,-4,-2), (9,-5,2), (-1,8,0), (-1,-7,0), (-2,8,-3) (-2,-6,3), (-4,9,2), (-4,-5,-2), (-5,9,-2), (-5,-4,2), (-6,8,3), (-6,-2,-3), (-7,1,5), (-7,6,-5) (-7,8,0), (-7,-1,0), (-8,1,0), (-8,2,-3), (-8,6,3), (-8,7,0), (-9,4,2), (-9,5,-2)								
**Quartz**	**(4,4,4)**	**11.088**	**71.65**	**3.2**	**2.5**	**3.874**	**9.64**	**48.43**	**49.38**
	Equiv. Refl.: (4,4,-4), (4,-8,4), (4,-8,-4), (-8,4,4), (-8,4,-4)								
**Sapph**	**(2,6,-7)**	**11.351**	**76.34**	**3.1**	**2.7**	**2.84**	**7.68**	**35.5**	**36.32**
	Equiv. Refl.: (2,-8,7), (6,2,7), (6,-8,-7), (8,-2,7), (8,-6,-7), (-2,8,-7), (-2,-6,7), (-6,8,7), (-6,-2,-7) (-8,2,-7), (-8,6,7), (8,-4,4), (8,-4,-4), (-4,8,4), (-4,8,-4), (-4,-4,4), (-4,-4,-4)								
**Quartz**	**(7,-1,-5)**	**11.143**	**72.53**	**3**	**2.5**	**3.676**	**9.09**	**45.95**	**46.84**
	Equiv. Refl.: (7,-6,5), (-1,7,5), (-1,-6,-5), (-6,7,-5), (-6,-1,5)								
**Quartz**	**(5,-2,8)**	**11.16**	**72.8**	**2.8**	**2.4**	**3.616**	**8.86**	**45.2**	**46.06**
	Equiv. Refl.: (5,-3,-8), (8,-1,-1), (8,-7,1), (-1,8,1), (-1,-7,-1), (-2,5,-8), (-2,-3,8), (-3,5,8), (-3,-2,-8) (-7,8,-1), (-7,-1,1)								
**Quartz**	**(2,3,-8)**	**11.16**	**72.8**	**2.6**	**2.4**	**3.616**	**8.86**	**45.2**	**46.06**
	Equiv. Refl.: (2,-5,8), (3,2,8), (3,-5,-8), (-5,2,-8), (-5,3,8)								

Ei =	11.682	keV							
**Cryst**	**Refl** (h,k,l)	**EB** [keV]	**ΘB** [°]	**∫ IR dΘ** [µrad]	**Width** [µrad]	**Ei cotΘ** [meV/µrad]	**ΔE** [meV]	**ΔEg** 2m, 50µm [meV]	**ΔEt** 2m, 50µm [meV]
**Quartz**	**(1,7,1)**	**11.06**	**71.21**	**2.5**	**2.2**	**3.974**	**8.83**	**49.67**	**50.45**
	Equiv. Refl.: (1,-8,-1), (7,1,-1), (7,-8,1), (-8,1,1), (-8,7,-1)								
**Sapph**	**(1,7,3)**	**11.448**	**78.51**	**2.4**	**2.5**	**2.374**	**5.83**	**29.67**	**30.24**
	Equiv. Refl.: (1,7,-3), (1,-8,3), (1,-8,-3), (7,1,3), (7,1,-3), (7,-8,3), (7,-8,-3), (8,-1,3), (8,-1,-3) (8,-7,3), (8,-7,-3), (-1,8,3), (-1,8,-3), (-1,-7,3), (-1,-7,-3), (-7,8,3), (-7,8,-3), (-7,-1,3) (-7,-1,-3), (-8,1,3), (-8,1,-3), (-8,7,3), (-8,7,-3), (3,5,-4), (3,-8,4), (5,3,4), (5,-8,-4) (-8,3,-4)								
**Sapph**	**(1,6,13)**	**11.654**	**86.05**	**2.3**	**3.3**	**0.807**	**2.7**	**10.09**	**10.45**
	Equiv. Refl.: (1,-7,-13), (4,4,9), (4,4,-9), (4,-8,9), (4,-8,-9), (6,1,-13), (6,-7,13), (7,-1,-13), (7,-6,13) (8,-4,9), (8,-4,-9), (-1,7,13), (-1,-6,-13), (-4,8,9), (-4,8,-9), (-4,-4,9), (-4,-4,-9), (-6,7,-13) (-6,-1,13), (-7,1,13), (-7,6,-13), (-8,4,9), (-8,4,-9), (0,3,-9), (0,-3,9), (3,0,9), (3,-3,-9) (8,-3,4), (8,-5,-4), (-3,0,-9), (-3,3,9), (-3,8,-4), (-3,-5,4), (-5,8,4), (-5,-3,-4), (-8,5,4)								
**LiNbO3**	**(2,7,1)**	**11.39**	**77.17**	**2.1**	**3.3**	**2.66**	**8.82**	**33.25**	**34.4**
	Equiv. Refl.: (2,-9,-1), (7,2,-1), (7,-9,1), (9,-2,-1), (9,-7,1), (-2,9,1), (-2,-7,-1), (-7,9,-1), (-7,-2,1) (-9,2,1), (-9,7,-1)								
**Sapph**	**(2,6,5)**	**11.108**	**71.96**	**2**	**2.2**	**3.804**	**8.3**	**47.55**	**48.27**
	Equiv. Refl.: (2,-8,-5), (6,2,-5), (6,-8,5), (8,-2,-5), (8,-6,5), (-2,8,5), (-2,-6,-5), (-6,8,-5), (-6,-2,5) (-8,2,5), (-8,6,-5)								
**Quartz**	**(8,-2,-3)**	**11.056**	**71.15**	**1.9**	**1.9**	**3.988**	**7.73**	**49.85**	**50.44**
	Equiv. Refl.: (8,-6,3), (-2,8,3), (-2,-6,-3), (-6,8,-3), (-6,-2,3)								
**Sapph**	**(3,4,14)**	**11.331**	**75.92**	**1.8**	**2.1**	**2.93**	**6.15**	**36.63**	**37.14**
	Equiv. Refl.: (3,-7,-14), (4,3,-14), (4,-7,14), (7,-3,-14), (7,-4,14), (-3,7,14), (-3,-4,-14), (-4,7,-14), (-4,-3,14) (-7,3,14), (-7,4,-14), (2,5,6), (2,-7,-6), (5,2,-6), (5,-1,8), (5,-4,-8), (5,-7,6), (7,-2,-6) (7,-5,6), (-1,5,-8), (-1,-4,8), (-2,7,6), (-2,-5,-6), (-4,5,8), (-4,-1,-8), (-5,7,-6), (-5,-2,6) (-7,2,6), (-7,5,-6)								
**Quartz**	**(1,4,-8)**	**11.348**	**76.27**	**1.7**	**2**	**2.854**	**5.63**	**35.68**	**36.12**
	Equiv. Refl.: (1,-5,8), (2,6,3), (2,-8,-3), (4,1,8), (4,-5,-8), (6,2,-3), (6,-8,3), (8,-1,1), (8,-7,-1) (-1,8,-1), (-1,-7,1), (-5,1,-8), (-5,4,8), (-7,8,1), (-7,-1,-1), (-8,2,3), (-8,6,-3)								
**LiNbO3**	**(2,4,19)**	**11.239**	**74.18**	**1.6**	**2.6**	**3.31**	**8.46**	**41.38**	**42.24**
	Equiv. Refl.: (2,-6,-19), (4,2,-19), (4,-6,19), (6,-2,-19), (6,-4,19), (-2,6,19), (-2,-4,-19), (-4,6,-19), (-4,-2,19) (-6,2,19), (-6,4,-19), (1,7,-1), (1,-8,1), (7,1,1), (7,-8,-1), (-8,1,-1), (-8,7,1)								
**Sapph**	**(2,4,-17)**	**11.367**	**76.66**	**1.5**	**1.8**	**2.769**	**5.02**	**34.62**	**34.98**
	Equiv. Refl.: (2,-6,17), (4,2,17), (4,-6,-17), (6,-2,17), (6,-4,-17), (-2,6,-17), (-2,-4,17), (-4,6,17), (-4,-2,-17) (-6,2,-17), (-6,4,17)								
**LiNbO3**	**(1,3,-23)**	**11.442**	**78.36**	**1.2**	**2.4**	**2.406**	**5.79**	**30.07**	**30.62**
	Equiv. Refl.: (1,-4,23), (3,1,23), (3,-4,-23), (4,-1,23), (4,-3,-23), (-1,4,-23), (-1,-3,23), (-3,4,23), (-3,-1,-23) (-4,1,-23), (-4,3,23)								
**LiNbO3**	**(3,4,17)**	**11.372**	**76.78**	**1.1**	**2.1**	**2.745**	**5.8**	**34.31**	**34.8**
	Equiv. Refl.: (3,-7,-17), (4,3,-17), (4,-7,17), (7,-3,-17), (7,-4,17), (-3,7,17), (-3,-4,-17), (-4,7,-17), (-4,-3,17) (-7,3,17), (-7,4,-17)								
**Quartz**	**(3,4,-6)**	**11.221**	**73.85**	**1**	**1.1**	**3.383**	**3.85**	**42.29**	**42.47**
	Equiv. Refl.: (3,-7,6), (4,3,6), (4,-7,-6), (6,1,5), (6,-7,-5), (7,-1,5), (7,-6,-5), (-1,7,-5), (-1,-6,5) (-6,7,5), (-6,-1,-5), (-7,3,-6), (-7,4,6)								

**Ei =**	**11.682**	**keV**						**ΔEg**	**ΔEt**
**Cryst**	**Refl**	**EB**	**ΘB**	**∫ IR dΘ**	**Width**	**Ei cotΘ**	**ΔE**	**2m, 50μm**	**2m, 50μm**
	**(h,k,l)**	**[keV]**	**[°]**	**[μrad]**	**[μrad]**	**[meV/μrad]**	**[meV]**	**[meV]**	**[meV]**
**LiNbO3**	**(1,4,21)**	**11.348**	**76.28**	**0.9**	**1.9**	**2.853**	**5.29**	**35.66**	**36.05**
	Equiv. Refl.: (1,4,-21), (1,5,-19), (1,-5,21), (1,-5,-21), (1,-6,19), (4,1,21), (4,1,-21), (4,-5,21), (4,-5,-21) (5,1,19), (5,-1,21), (5,-1,-21), (5,-4,21), (5,-4,-21), (5,-6,-19), (6,-1,19), (6,-5,-19), (-1,5,21) (-1,5,-21), (-1,6,-19), (-1,-4,21), (-1,-4,-21), (-1,-5,19), (-4,5,21), (-4,5,-21), (-4,-1,21), (-4,-1,-21) (-5,1,21), (-5,1,-21), (-5,4,21), (-5,4,-21), (-5,6,19), (-5,-1,-19), (-6,1,-19), (-6,5,19), (2,3,-19) (2,-5,19), (3,2,19), (3,5,7), (3,-5,-19), (3,-8,-7), (5,3,-7), (5,-2,19), (5,-3,-19), (5,-8,7) (8,-3,-7), (8,-5,7), (-2,5,-19), (-2,-3,19), (-3,5,19), (-3,8,7), (-3,-2,-19), (-3,-5,-7), (-5,2,-19) (-5,3,19), (-5,8,-7), (-5,-3,7), (-8,3,7), (-8,5,-7), (1,2,-9), (1,6,-5), (1,7,-2), (1,-3,9) (1,-7,5), (1,-8,2), (2,1,9), (2,4,7), (2,-3,-9), (2,-6,-7), (4,2,-7), (4,-6,7), (6,-2,-7) (6,-4,7), (7,1,2), (7,-3,6), (7,-4,-6), (7,-8,-2), (8,-1,2), (8,-7,-2), (-1,8,-2), (-1,-7,2) (-2,6,7), (-2,-4,-7), (-3,1,-9), (-3,2,9), (-3,7,-6), (-3,-4,6), (-4,6,-7), (-4,7,6), (-4,-2,7) (-4,-3,-6), (-6,2,7), (-6,4,-7), (-7,1,-5), (-7,6,5), (-7,8,2), (-7,-1,-2), (-8,1,-2), (-8,7,2)								
**LiNbO3**	**(4,5,-7)**	**11.302**	**75.35**	**0.8**	**1.8**	**3.054**	**5.4**	**38.17**	**38.55**
	Equiv. Refl.: (4,-9,7), (5,4,7), (5,-9,-7), (9,-4,7), (9,-5,-7), (-4,9,-7), (-4,-5,7), (-5,9,7), (-5,-4,-7) (-9,4,-7), (-9,5,7), (3,-1,9), (3,-2,-9), (-1,3,-9), (-1,-2,9), (-2,3,9), (-2,-1,-9)								
**LiNbO3**	**(2,6,-13)**	**11.59**	**82.81**	**0.7**	**2.1**	**1.473**	**3.11**	**18.41**	**18.67**
	Equiv. Refl.: (2,-8,13), (4,5,5), (4,-9,-5), (5,4,-5), (5,-9,5), (6,2,13), (6,-8,-13), (8,-2,13), (8,-6,-13) (9,-4,-5), (9,-5,5), (-2,8,-13), (-2,-6,13), (-4,9,5), (-4,-5,-5), (-5,9,-5), (-5,-4,5), (-6,8,13) (-6,-2,-13), (-8,2,-13), (-8,6,13), (-9,4,5), (-9,5,-5), (0,7,-4), (0,-7,4), (7,0,4), (7,-7,-4) (8,-2,-4), (8,-3,-4), (8,-5,4), (8,-6,4), (-2,8,4), (-2,-6,-4), (-3,8,4), (-3,-5,-4), (-5,8,-4) (-5,-3,4), (-6,8,-4), (-6,-2,4), (-7,0,-4), (-7,7,4)								
**Sapph**	**(1,6,-11)**	**11.175**	**73.06**	**0.6**	**0.9**	**3.558**	**3.14**	**44.47**	**44.58**
	Equiv. Refl.: (1,-7,11), (6,1,11), (6,-7,-11), (7,-1,11), (7,-6,-11), (-1,7,-11), (-1,-6,11), (-6,7,11), (-6,-1,-11) (-7,1,-11), (-7,6,11), (3,5,4), (3,-8,-4), (5,3,-4), (5,-8,4), (7,-2,6), (7,-5,-6), (-2,7,-6) (-2,-5,6), (-5,7,6), (-5,-2,-6), (-8,3,4), (-8,5,-4)								
**Sapph**	**(1,4,18)**	**11.015**	**70.55**	**0.5**	**0.7**	**4.125**	**3.08**	**51.57**	**51.66**
	Equiv. Refl.: (1,4,-18), (1,-5,18), (1,-5,-18), (2,5,12), (2,5,-12), (2,-7,12), (2,-7,-12), (4,1,18), (4,1,-18) (4,-5,18), (4,-5,-18), (5,2,12), (5,2,-12), (5,-1,18), (5,-1,-18), (5,-4,18), (5,-4,-18), (5,-7,12) (5,-7,-12), (7,-2,12), (7,-2,-12), (7,-5,12), (7,-5,-12), (-1,5,18), (-1,5,-18), (-1,-4,18), (-1,-4,-18) (-2,7,12), (-2,7,-12), (-2,-5,12), (-2,-5,-12), (-4,5,18), (-4,5,-18), (-4,-1,18), (-4,-1,-18), (-5,1,18) (-5,1,-18), (-5,4,18), (-5,4,-18), (-5,7,12), (-5,7,-12), (-5,-2,12), (-5,-2,-12), (-7,2,12), (-7,2,-12) (-7,5,12), (-7,5,-12), (0,6,6), (0,-6,-6), (1,2,9), (1,-3,-9), (2,1,-9), (2,5,-6), (2,6,4) (2,-3,9), (2,-7,6), (2,-8,-4), (3,-1,-9), (3,-2,9), (5,2,6), (5,-7,-6), (6,0,-6), (6,2,-4) (6,-1,-7), (6,-5,7), (6,-6,6), (6,-8,4), (-1,3,9), (-1,6,7), (-1,-2,-9), (-1,-5,-7), (-2,3,-9) (-2,-1,9), (-3,1,9), (-3,2,-9), (-5,6,-7), (-5,-1,7), (-6,0,6), (-6,6,-6), (-7,2,-6), (-7,5,6) (-8,2,4), (-8,6,-4)								
**LiNbO3**	**(3,6,3)**	**11.118**	**72.12**	**0.4**	**1.1**	**3.768**	**4.03**	**47.1**	**47.28**
	Equiv. Refl.: (3,6,-3), (3,-9,3), (3,-9,-3), (6,3,3), (6,3,-3), (6,-9,3), (6,-9,-3), (9,-3,3), (9,-3,-3) (9,-6,3), (9,-6,-3), (-3,9,3), (-3,9,-3), (-3,-6,3), (-3,-6,-3), (-6,9,3), (-6,9,-3), (-6,-3,3) (-6,-3,-3), (-9,3,3), (-9,3,-3), (-9,6,3), (-9,6,-3), (0,3,9), (0,-3,-9), (1,5,7), (1,-6,-7) (2,3,8), (2,-5,-8), (3,0,-9), (3,2,-8), (3,-3,9), (3,-5,8), (4,5,0), (4,-9,0), (5,1,-7) (5,4,0), (5,-6,7), (5,-9,0), (9,-4,0), (9,-5,0), (-3,0,9), (-3,3,-9), (-4,9,0), (-4,-5,0) (-5,2,8), (-5,3,-8), (-5,9,0), (-5,-4,0), (-6,1,7), (-6,5,-7), (-9,4,0), (-9,5,0)								
**LiNbO3**	**(2,6,11)**	**11.169**	**72.95**	**0.3**	**1**	**3.583**	**3.5**	**44.79**	**44.92**
	Equiv. Refl.: (2,-8,-11), (6,2,-11), (6,-8,11), (8,-2,-11), (8,-6,11), (-2,8,11), (-2,-6,-11), (-6,8,-11), (-6,-2,11) (-8,2,11), (-8,6,-11), (1,7,2), (1,-8,-2), (5,-2,-8), (5,-3,8), (7,1,-2), (7,-8,2), (8,-1,-2) (8,-7,2), (-1,8,2), (-1,-7,-2), (-2,5,8), (-2,-3,-8), (-3,5,-8), (-3,-2,8), (-7,8,-2), (-7,-1,2) (-8,1,2), (-8,7,-2)								
**LiNbO3**	**(1,7,9)**	**11.243**	**74.24**	**0.2**	**0.8**	**3.296**	**2.75**	**41.2**	**41.3**
	Equiv. Refl.: (1,7,-9), (1,-8,9), (1,-8,-9), (3,5,13), (3,-8,-13), (5,3,-13), (5,-8,13), (7,1,9), (7,1,-9) (7,-8,9), (7,-8,-9), (8,-1,9), (8,-1,-9), (8,-3,-13), (8,-5,13), (8,-7,9), (8,-7,-9), (-1,8,9) (-1,8,-9), (-1,-7,9), (-1,-7,-9), (-3,8,13), (-3,-5,-13), (-5,8,-13), (-5,-3,13), (-7,8,9), (-7,8,-9) (-7,-1,9), (-7,-1,-9), (-8,1,9), (-8,1,-9), (-8,3,13), (-8,5,-13), (-8,7,9), (-8,7,-9), (0,0,24) (0,0,-24), (3,4,-13), (3,5,-8), (3,-7,13), (3,-8,8), (4,3,13), (4,-7,-13), (5,3,8), (5,-8,-8) (7,-3,13), (7,-4,-13), (8,-3,8), (8,-5,-8), (-3,7,-13), (-3,8,-8), (-3,-4,13), (-3,-5,8), (-4,7,13)								

	(-4,-3,-13), (-5,8,8), (-5,-3,-8), (-7,3,-13), (-7,4,13), (-8,3,-8), (-8,5,8), (3,3,7), (3,3,-7) (3,-6,7), (3,-6,-7), (6,-3,7), (6,-3,-7), (-3,6,7), (-3,6,-7), (-3,-3,7), (-3,-3,-7), (-6,3,7) (-6,3,-7)								
**Sapph**	**(1,2,-22)**	**11.229**	**74**	**0.1**	**0.3**	**3.35**	**0.88**	**41.87**	**41.88**
	Equiv. Refl.: (1,-3,22), (2,1,22), (2,6,8), (2,-3,-22), (2,-8,-8), (3,-1,22), (3,-2,-22), (6,2,-8), (6,-8,8) (8,-2,-8), (8,-6,8), (-1,3,-22), (-1,-2,22), (-2,3,22), (-2,8,8), (-2,-1,-22), (-2,-6,-8), (-3,1,-22) (-3,2,22), (-6,8,-8), (-6,-2,8), (-8,2,8), (-8,6,-8)								

Ei =	11.136	keV						ΔEg	ΔEt
**Cryst**	**Refl**	**EB**	**ΘB**	**∫ IR dΘ**	**Width**	**Ei cotΘ**	**ΔE**	**2m, 50μm**	**2m, 50μm**
	**(h,k,l)**	**[keV]**	**[°]**	**[μrad]**	**[μrad]**	**[meV/μrad]**	**[meV]**	**[meV]**	**[meV]**
**Si**	**(4,6,6)**	**10.708**	**74.06**	**6.377**	**5.4**	**3.181**	**17.32**	**39.76**	**43.37**
**Si**	**(1,3,9)**	**10.889**	**77.9**	**5.363**	**4.8**	**2.387**	**11.43**	**29.84**	**31.95**
**Ge**	**(4,4,8)**	**10.736**	**74.6**	**0.649**	**0.8**	**3.066**	**2.34**	**38.33**	**38.4**
**Ge**	**(1,7,7)**	**10.903**	**78.26**	**0.348**	**0.9**	**2.315**	**2**	**28.94**	**29.01**
	Equiv. Refl.: (3,3,9), (5,5,7)								
**Quartz**	**(2,4,-7)**	**11.131**	**88.33**	**37.7**	**32.7**	**0.324**	**10.6**	**4.05**	**11.35**
	Equiv. Refl.: Equiv. Refl.: (2,-6,7), (4,2,7), (4,-6,-7), (-6,2,-7), (-6,4,7)								
**Quartz**	**(2,4,-7)**	**11.131**	**88.33**	**37.7**	**32.7**	**0.324**	**10.6**	**4.05**	**11.35**
	Equiv. Refl.: (2,-6,7), (4,2,7), (4,-6,-7), (-6,2,-7), (-6,4,7)								
**Quartz**	**(6,-2,7)**	**11.131**	**88.33**	**36.8**	**32.7**	**0.324**	**10.6**	**4.05**	**11.34**
	Equiv. Refl.: (6,-4,-7), (-2,6,-7), (-2,-4,7), (-4,6,7), (-4,-2,-7)								
**Quartz**	**(0,6,-6)**	**11.126**	**87.55**	**33.1**	**28.2**	**0.476**	**13.41**	**5.95**	**14.67**
	Equiv. Refl.: (0,-6,6)								
**Quartz**	**(6,0,6)**	**11.126**	**87.55**	**33**	**28.2**	**0.476**	**13.4**	**5.95**	**14.66**
	Equiv. Refl.: (6,-6,-6), (-6,0,-6), (-6,6,6)								
**LiNbO3**	**(3,-7,16)**	**11.078**	**84.17**	**28.7**	**26.5**	**1.138**	**30.14**	**14.22**	**33.33**
	Equiv. Refl.: (4,3,16), (7,-3,16), (-3,-4,16), (-4,7,16), (-7,4,16)								
**LiNbO3**	**(1,6,-14)**	**11.06**	**83.32**	**27.6**	**25.1**	**1.304**	**32.71**	**16.3**	**36.55**
	Equiv. Refl.: (6,-7,-14), (7,-6,-14), (-1,7,-14), (-6,-1,-14), (-7,1,-14)								
**LiNbO3**	**(3,4,-16)**	**11.078**	**84.17**	**27**	**26.5**	**1.138**	**30.14**	**14.22**	**33.33**
	Equiv. Refl.: (4,-7,-16), (7,-4,-16), (-3,7,-16), (-4,-3,-16), (-7,3,-16)								
**LiNbO3**	**(1,-7,14)**	**11.06**	**83.32**	**25.8**	**25.1**	**1.304**	**32.71**	**16.3**	**36.55**
	Equiv. Refl.: (6,1,14), (7,-1,14), (-1,-6,14), (-6,7,14), (-7,6,14)								
**LiNbO3**	**(1,1,24)**	**10.999**	**81.01**	**24.2**	**22.1**	**1.762**	**38.9**	**22.03**	**44.7**
	Equiv. Refl.: (1,-2,24), (2,-1,24), (-1,2,24), (-1,-1,24), (-2,1,24)								
**LiNbO3**	**(1,1,-24)**	**10.999**	**81.01**	**24.1**	**22.1**	**1.762**	**38.9**	**22.03**	**44.7**
	Equiv. Refl.: (1,-2,-24), (2,-1,-24), (-1,2,-24), (-1,-1,-24), (-2,1,-24)								
**LiNbO3**	**(3,6,0)**	**11.037**	**82.34**	**23.4**	**21.8**	**1.498**	**32.57**	**18.72**	**37.57**
	Equiv. Refl.: (6,-9,0), (9,-6,0), (-3,9,0), (-6,-3,0), (-9,3,0)								
**LiNbO3**	**(3,-9,0)**	**11.037**	**82.34**	**22.9**	**21.8**	**1.498**	**32.57**	**18.72**	**37.57**
	Equiv. Refl.: (6,3,0), (9,-3,0), (-3,-6,0), (-6,9,0), (-9,6,0)								
**LiNbO3**	**(4,4,12)**	**11.027**	**81.99**	**21.8**	**20.1**	**1.568**	**31.48**	**19.59**	**37.08**
	Equiv. Refl.: (4,-8,12), (8,-4,12), (-4,8,12), (-4,-4,12), (-8,4,12)								
**LiNbO3**	**(1,-4,-22)**	**11.042**	**82.54**	**21.5**	**20.3**	**1.459**	**29.58**	**18.23**	**34.74**
	Equiv. Refl.: (3,1,-22), (4,-1,-22), (-1,-3,-22), (-3,4,-22), (-4,3,-22)								
**LiNbO3**	**(1,3,22)**	**11.042**	**82.54**	**20.9**	**20.3**	**1.459**	**29.58**	**18.23**	**34.74**
	Equiv. Refl.: (3,-4,22), (4,-3,22), (-1,4,22), (-3,-1,22), (-4,1,22)								
**LiNbO3**	**(4,4,-12)**	**11.027**	**81.99**	**20.7**	**20.1**	**1.568**	**31.48**	**19.59**	**37.08**
	Equiv. Refl.: (4,-8,-12), (8,-4,-12), (-4,8,-12), (-4,-4,-12), (-8,4,-12)								
**LiNbO3**	**(4,-9,4)**	**11.006**	**81.25**	**20.3**	**18.5**	**1.714**	**31.76**	**21.43**	**38.31**
	Equiv. Refl.: (5,4,4), (9,-4,4), (-4,-5,4), (-5,9,4), (-9,5,4)								
**LiNbO3**	**(4,5,-4)**	**11.006**	**81.25**	**18.9**	**18.5**	**1.714**	**31.76**	**21.43**	**38.31**
	Equiv. Refl.: (5,-9,-4), (9,-5,-4), (-4,9,-4), (-5,-4,-4), (-9,4,-4)								
**LiNbO3**	**(2,-8,10)**	**10.979**	**80.36**	**18**	**16.8**	**1.891**	**31.67**	**23.63**	**39.52**
	Equiv. Refl.: (6,2,10), (8,-2,10), (-2,-6,10), (-6,8,10), (-8,6,10)								
**LiNbO3**	**(4,5,2)**	**10.897**	**78.1**	**17.7**	**16.2**	**2.346**	**37.96**	**29.32**	**47.97**
	Equiv. Refl.: (5,-9,2), (9,-5,2), (-4,9,2), (-5,-4,2), (-9,4,2)								

Ei =	11.136	keV						ΔEg	ΔEt
Cryst	Refl	EB	ΘB	∫ IR dΘ	Width	Ei cotΘ	ΔE	2m, 50μm	2m, 50μm
	(h,k,l)	[keV]	[°]	[μrad]	[μrad]	[meV/μrad]	[meV]	[meV]	[meV]
**LiNbO3**	**(2,6,-10)**	**10.979**	**80.36**	**17.4**	**16.8**	**1.891**	**31.67**	**23.63**	**39.52**
	Equiv. Refl.: (4,-9,-2), (5,4,-2), (6,-8,-10), (8,-6,-10), (9,-4,-2), (-2,8,-10), (-4,-5,-2), (-5,9,-2), (-6,-2,-10) (-8,2,-10), (-9,5,-2)								
**LiNbO3**	**(3,3,18)**	**10.816**	**76.24**	**15.8**	**14.2**	**2.727**	**38.78**	**34.09**	**51.64**
	Equiv. Refl.: (3,-6,18), (6,-3,18), (-3,6,18), (-3,-3,18), (-6,3,18)								
**LiNbO3**	**(3,3,-18)**	**10.816**	**76.24**	**15.1**	**14.2**	**2.727**	**38.78**	**34.09**	**51.64**
	Equiv. Refl.: (3,-6,-18), (6,-3,-18), (-3,6,-18), (-3,-3,-18), (-6,3,-18)								
**LiNbO3**	**(1,-8,-6)**	**10.835**	**76.66**	**14.7**	**13.6**	**2.641**	**36.02**	**33.02**	**48.86**
	Equiv. Refl.: (7,1,-6), (8,-1,-6), (-1,-7,-6), (-7,8,-6), (-8,7,-6)								
**LiNbO3**	**(1,7,6)**	**10.835**	**76.66**	**14.6**	**13.6**	**2.641**	**36.02**	**33.02**	**48.86**
	Equiv. Refl.: (7,-8,6), (8,-7,6), (-1,8,6), (-7,-1,6), (-8,1,6)								
**LiNbO3**	**(0,7,-10)**	**10.711**	**74.13**	**14**	**12.7**	**3.166**	**40.32**	**39.58**	**56.5**
	Equiv. Refl.: (7,-7,-10), (-7,0,-10)								
**LiNbO3**	**(1,7,-6)**	**10.835**	**76.66**	**13.9**	**12.9**	**2.641**	**33.98**	**33.02**	**47.38**
	Equiv. Refl.: (7,-8,-6), (8,-7,-6), (-1,8,-6), (-7,-1,-6), (-8,1,-6)								
**LiNbO3**	**(0,-7,10)**	**10.711**	**74.13**	**13.8**	**12.7**	**3.166**	**40.32**	**39.58**	**56.5**
	Equiv. Refl.: (7,0,10), (-7,7,10)								
**LiNbO3**	**(1,-8,6)**	**10.835**	**76.66**	**13.5**	**12.9**	**2.641**	**33.98**	**33.02**	**47.38**
	Equiv. Refl.: (7,1,6), (8,-1,6), (-1,-7,6), (-7,8,6), (-8,7,6)								
**Sapph**	**(2,6,-4)**	**11.015**	**81.55**	**13**	**11.1**	**1.653**	**18.34**	**20.67**	**27.63**
	Equiv. Refl.: (2,-8,4), (6,2,4), (6,-8,-4), (8,-2,4), (8,-6,-4), (-2,8,-4), (-2,-6,4), (-6,8,4), (-6,-2,-4) (-8,2,-4), (-8,6,4)								
**LiNbO3**	**(2,-8,-8)**	**10.646**	**72.94**	**12.3**	**11.3**	**3.417**	**38.54**	**42.72**	**57.53**
	Equiv. Refl.: (6,2,-8), (8,-2,-8), (-2,-6,-8), (-6,8,-8), (-8,6,-8)								
**LiNbO3**	**(0,4,20)**	**10.532**	**71.04**	**12.1**	**11.1**	**3.825**	**42.31**	**47.82**	**63.84**
	Equiv. Refl.: (0,-4,-20), (2,6,8), (4,0,-20), (4,-4,20), (6,-8,8), (8,-6,8), (-2,8,8), (-4,0,20), (-4,4,-20) (-6,-2,8), (-8,2,8)								
**LiNbO3**	**(3,5,10)**	**10.711**	**74.13**	**11.7**	**10.6**	**3.166**	**33.4**	**39.58**	**51.79**
	Equiv. Refl.: (5,-8,10), (8,-5,10), (-3,8,10), (-5,-3,10), (-8,3,10), (1,3,-20), (1,6,10), (1,-4,20), (1,-7,-10) (3,1,20), (3,-4,-20), (4,-1,20), (4,-3,-20), (6,1,-10), (6,-7,10), (7,-1,-10), (7,-6,10), (-1,4,-20) (-1,7,10), (-1,-3,20), (-1,-6,-10), (-3,4,20), (-3,-1,-20), (-4,1,-20), (-4,3,20), (-6,7,-10), (-6,-1,10) (-7,1,10), (-7,6,-10)								
**LiNbO3**	**(2,-5,-20)**	**10.804**	**75.97**	**11.5**	**10.5**	**2.783**	**29.31**	**34.78**	**45.49**
	Equiv. Refl.: (3,2,-20), (5,-2,-20), (-2,-3,-20), (-3,5,-20), (-5,3,-20), (-4,6,-7)								
**Quartz**	**(6,-2,-7)**	**11.131**	**88.33**	**11.4**	**11.9**	**0.324**	**3.87**	**4.05**	**5.6**
	Equiv. Refl.: (6,-4,7), (-2,6,7), (-2,-4,-7), (-4,-2,7)								
**LiNbO3**	**(1,5,-16)**	**10.542**	**71.2**	**11.2**	**10.1**	**3.791**	**38.23**	**47.39**	**60.89**
	Equiv. Refl.: (5,-6,-16), (6,-5,-16), (-1,6,-16), (-5,-1,-16), (-6,1,-16)								
**LiNbO3**	**(1,-6,16)**	**10.542**	**71.2**	**11.1**	**10.1**	**3.791**	**38.23**	**47.39**	**60.89**
	Equiv. Refl.: (5,1,16), (6,-1,16), (-1,-5,16), (-5,6,16), (-6,5,16)								
**LiNbO3**	**(3,-8,-10)**	**10.711**	**74.13**	**10.8**	**10.6**	**3.166**	**33.4**	**39.58**	**51.79**
	Equiv. Refl.: (5,3,-10), (8,-3,-10), (-3,-5,-10), (-5,8,-10), (-8,5,-10)								
**LiNbO3**	**(2,3,20)**	**10.804**	**75.97**	**10.7**	**10.5**	**2.783**	**29.31**	**34.78**	**45.49**
	Equiv. Refl.: (3,-5,20), (5,-3,20), (-2,5,20), (-3,-2,20), (-5,2,20)								
**LiNbO3**	**(1,-3,22)**	**10.503**	**70.59**	**10.6**	**9.6**	**3.923**	**37.59**	**49.04**	**61.79**
	Equiv. Refl.: (1,-8,0), (2,1,22), (3,-1,22), (7,1,0), (8,-1,0), (-1,-2,22), (-1,-7,0), (-2,3,22), (-3,2,22) (-7,8,0), (-8,7,0), (2,4,7), (2,-6,-7), (4,2,-7), (4,-6,7), (-6,2,7), (-6,4,-7)								
**LiNbO3**	**(1,7,0)**	**10.498**	**70.51**	**10.5**	**9.6**	**3.941**	**37.83**	**49.26**	**62.11**
	Equiv. Refl.: (7,-8,0), (8,-7,0), (-1,8,0), (-7,-1,0), (-8,1,0)								
**LiNbO3**	**(1,2,-22)**	**10.503**	**70.59**	**10.4**	**9.6**	**3.923**	**37.59**	**49.04**	**61.79**
	Equiv. Refl.: (2,-3,-22), (3,-2,-22), (-1,3,-22), (-2,-1,-22), (-3,1,-22)								

Ei =	11.136	keV						ΔEg	ΔEt
**Cryst**	**Refl**	**EB**	**ΘB**	**∫ IR dΘ**	**Width**	**Ei cotΘ**	**ΔE**	**2m, 50µm**	**2m, 50µm**
	**(h,k,l)**	**[keV]**	**[°]**	**[µrad]**	**[µrad]**	**[meV/µrad]**	**[meV]**	**[meV]**	**[meV]**
**LiNbO3**	**(3,-7,-14)**	**10.523**	**70.9**	**10.3**	**9.2**	**3.856**	**35.63**	**48.2**	**59.94**
	Equiv. Refl.: (4,3,-14), (7,-3,-14), (-3,-4,-14), (-4,7,-14), (-7,4,-14), (2,6,5), (2,-8,-5), (6,2,-5), (6,-8,5) (8,-2,-5), (8,-6,5), (-2,8,5), (-2,-6,-5), (-6,8,-5), (-6,-2,5), (-8,2,5), (-8,6,-5), (4,4,-4) (4,-8,4)								
**Quartz**	**(4,4,4)**	**11.088**	**84.68**	**10.2**	**8.9**	**1.037**	**9.22**	**12.96**	**15.9**
	Equiv. Refl.: (4,-8,-4), (-8,4,4), (-8,4,-4)								
**Quartz**	**(8,-4,4)**	**11.088**	**84.68**	**10**	**8.9**	**1.037**	**9.22**	**12.96**	**15.9**
	Equiv. Refl.: (8,-4,-4), (-4,8,4), (-4,8,-4), (-4,-4,4), (-4,-4,-4)								
**LiNbO3**	**(3,4,14)**	**10.523**	**70.9**	**9.8**	**9.2**	**3.856**	**35.63**	**48.2**	**59.94**
	Equiv. Refl.: (4,-7,14), (7,-4,14), (-3,7,14), (-4,-3,14), (-7,3,14)								
**Quartz**	**(0,5,7)**	**10.841**	**76.79**	**9.1**	**7.1**	**2.613**	**18.62**	**32.67**	**37.6**
	Equiv. Refl.: (0,-5,-7), (5,0,-7), (5,-5,7), (-5,0,7), (-5,5,-7)								
**Quartz**	**(2,6,-3)**	**11.056**	**83.11**	**8.8**	**7.6**	**1.345**	**10.21**	**16.82**	**19.67**
	Equiv. Refl.: (2,-8,3), (6,2,3), (6,-8,-3), (-8,2,-3), (-8,6,3)								
**Quartz**	**(8,-2,3)**	**11.056**	**83.11**	**8.7**	**7.6**	**1.345**	**10.21**	**16.82**	**19.67**
	Equiv. Refl.: (8,-6,-3), (-2,8,-3), (-2,-6,3), (-6,8,3), (-6,-2,-3)								
**Quartz**	**(0,4,8)**	**10.87**	**77.46**	**8.6**	**7**	**2.476**	**17.3**	**30.96**	**35.46**
	Equiv. Refl.: (0,-4,-8), (4,0,-8), (4,-4,8), (-4,0,8), (-4,4,-8)								
**Quartz**	**(3,5,-3)**	**10.764**	**75.14**	**8.3**	**6.5**	**2.954**	**19.26**	**36.92**	**41.64**
	Equiv. Refl.: (3,-8,3), (5,3,3), (5,-8,-3), (8,-3,3), (8,-5,-3), (-3,8,-3), (-3,-5,3), (-5,8,3), (-5,-3,-3) (-8,3,-3), (-8,5,3)								
**LiNbO3**	**(0,0,-24)**	**10.732**	**74.52**	**8**	**7.5**	**3.083**	**23.06**	**38.54**	**44.92**
	Equiv. Refl.: (8,-1,0), (8,-7,0), (-1,8,0), (-1,-7,0), (-7,8,0), (-7,-1,0)								
**Quartz**	**(1,7,0)**	**11**	**81.04**	**7.8**	**6.6**	**1.757**	**11.68**	**21.96**	**24.87**
	Equiv. Refl.: (1,-8,0), (7,1,0), (7,-8,0), (-8,1,0), (-8,7,0)								
**Sapph**	**(2,4,16)**	**11.031**	**82.14**	**7.6**	**6.8**	**1.538**	**10.51**	**19.22**	**21.9**
	Equiv. Refl.: (2,-6,-16), (4,2,-16), (4,-6,16), (6,-2,-16), (6,-4,16), (-2,6,16), (-2,-4,-16), (-4,6,-16), (-4,-2,16) (-6,2,16), (-6,4,-16)								
**Sapph**	**(1,5,14)**	**10.715**	**74.2**	**7.5**	**6.2**	**3.152**	**19.52**	**39.4**	**43.97**
	Equiv. Refl.: (1,-6,-14), (3,5,4), (3,-8,-4), (5,1,-14), (5,3,-4), (5,-6,14), (5,-8,4), (6,-1,-14), (6,-5,14) (8,-3,-4), (8,-5,4), (-1,6,14), (-1,-5,-14), (-3,8,4), (-3,-5,-4), (-5,6,-14), (-5,8,-4), (-5,-1,14) (-5,-3,4), (-6,1,14), (-6,5,-14), (-8,3,4), (-8,5,-4)								
**Quartz**	**(8,-1,-1)**	**11.06**	**83.29**	**7.2**	**6.4**	**1.311**	**8.43**	**16.39**	**18.43**
	Equiv. Refl.: (8,-7,1), (-1,8,1), (-1,-7,-1), (-7,8,-1), (-7,-1,1)								
**Quartz**	**(1,7,1)**	**11.06**	**83.29**	**7.1**	**6.4**	**1.311**	**8.43**	**16.39**	**18.43**
	Equiv. Refl.: (1,-8,-1), (7,1,-1), (7,-8,1), (-8,1,1), (-8,7,-1)								
**LiNbO3**	**(0,0,24)**	**10.732**	**74.52**	**6.9**	**7.5**	**3.083**	**23.06**	**38.54**	**44.92**
**Sapph**	**(4,4,6)**	**10.809**	**76.08**	**6.7**	**5.7**	**2.759**	**15.63**	**34.49**	**37.87**
	Equiv. Refl.: (4,4,-6), (4,-8,6), (4,-8,-6), (8,-4,6), (8,-4,-6), (-4,8,6), (-4,8,-6), (-4,-4,6), (-4,-4,-6) (-8,4,6), (-8,4,-6)								
**Quartz**	**(8,-2,-3)**	**11.056**	**83.11**	**6.5**	**5.5**	**1.345**	**7.38**	**16.82**	**18.37**
	Equiv. Refl.: (8,-6,3), (-2,8,3), (-2,-6,-3), (-6,8,-3), (-6,-2,3)								
**Sapph**	**(0,5,16)**	**10.719**	**74.27**	**6**	**5.1**	**3.136**	**16.06**	**39.2**	**42.36**
	Equiv. Refl.: (0,-5,-16), (5,0,-16), (5,-5,16), (-5,0,16), (-5,5,-16), (8,-1,1), (8,-7,-1), (-1,8,-1), (-1,-7,1) (-7,8,1), (-7,-1,-1)								
**Quartz**	**(2,6,3)**	**11.056**	**83.11**	**5.9**	**5.5**	**1.345**	**7.39**	**16.82**	**18.37**
	Equiv. Refl.: (2,-8,-3), (6,2,-3), (6,-8,3), (-8,2,3), (-8,6,-3)								
**Quartz**	**(1,7,-1)**	**11.06**	**83.29**	**5.5**	**5.2**	**1.311**	**6.78**	**16.39**	**17.73**
	Equiv. Refl.: (1,-8,1), (7,1,1), (7,-8,-1), (-8,1,-1), (-8,7,1)								

Ei =	11.136	keV						ΔEg	ΔEt
**Cryst**	**Refl**	**EB**	**ΘB**	**∫ IR dΘ**	**Width**	**Ei cotΘ**	**ΔE**	**2m, 50μm**	**2m, 50μm**
	**(h,k,l)**	**[keV]**	**[°]**	**[μrad]**	**[μrad]**	**[meV/μrad]**	**[meV]**	**[meV]**	**[meV]**
**Sapph**	**(0,7,2)**	**10.574**	**71.72**	**5.3**	**4.6**	**3.678**	**16.76**	**45.98**	**48.94**
	Equiv. Refl.: (0,-7,-2), (7,0,-2), (7,-7,2), (-7,0,2), (-7,7,-2)								
**Quartz**	**(1,5,6)**	**10.638**	**72.8**	**5.1**	**4.2**	**3.446**	**14.52**	**43.08**	**45.46**
	Equiv. Refl.: (1,-6,-6), (5,1,-6), (5,-6,6), (6,-1,-6), (6,-5,6), (-1,6,6), (-1,-5,-6), (-5,6,-6), (-5,-1,6) (-6,1,6), (-6,5,-6)								
**Quartz**	**(0,2,-9)**	**10.727**	**74.41**	**4.9**	**3.9**	**3.106**	**12.23**	**38.83**	**40.71**
	Equiv. Refl.: (0,-2,9), (2,0,9), (2,-2,-9), (-2,0,-9), (-2,2,9)								
**Quartz**	**(2,6,2)**	**10.754**	**74.95**	**4.8**	**3.9**	**2.994**	**11.67**	**37.43**	**39.21**
	Equiv. Refl.: (2,-8,-2), (6,2,-2), (6,-8,2), (8,-2,-1), (8,-2,-2), (8,-6,1), (8,-6,2), (-2,8,1), (-2,8,2) (-2,-6,-1), (-2,-6,-2), (-6,8,-1), (-6,8,-2), (-6,-2,1), (-6,-2,2), (-8,2,2), (-8,6,-2)								
**Sapph**	**(0,6,12)**	**10.69**	**73.73**	**4.7**	**3.9**	**3.249**	**12.83**	**40.61**	**42.59**
	Equiv. Refl.: (0,6,-12), (0,-6,12), (0,-6,-12), (6,0,12), (6,0,-12), (6,-6,12), (6,-6,-12), (-6,0,12), (-6,0,-12) (-6,6,12), (-6,6,-12), (2,6,1), (2,-8,-1), (6,2,-1), (6,-8,1), (-8,2,1), (-8,6,-1)								
**Quartz**	**(0,6,6)**	**11.126**	**87.55**	**4.2**	**5.3**	**0.476**	**2.53**	**5.95**	**6.46**
	Equiv. Refl.: (0,-6,-6), (6,-6,6), (-6,6,-6)								
**Quartz**	**(6,0,-6)**	**11.126**	**87.55**	**4.1**	**5.3**	**0.476**	**2.52**	**5.95**	**6.46**
	Equiv. Refl.: (-6,0,6)								
**Quartz**	**(4,4,3)**	**10.665**	**73.27**	**3.9**	**3.2**	**3.347**	**10.56**	**41.84**	**43.15**
	Equiv. Refl.: (4,4,-3), (4,-8,3), (4,-8,-3), (-8,4,3), (-8,4,-3)								
**Quartz**	**(1,6,-4)**	**10.599**	**72.13**	**3.8**	**3.1**	**3.591**	**11.07**	**44.88**	**46.23**
	Equiv. Refl.: (1,-7,4), (6,1,4), (6,-7,-4), (-7,1,-4), (-7,6,4)								
**Sapph**	**(2,6,-1)**	**10.859**	**77.19**	**3.7**	**3.4**	**2.531**	**8.59**	**31.64**	**32.79**
	Equiv. Refl.: (2,-8,1), (6,2,1), (6,-8,-1), (8,-2,1), (8,-6,-1), (-2,8,-1), (-2,-6,1), (-6,8,1), (-6,-2,-1) (-8,2,-1), (-8,6,1), (8,-4,3), (8,-4,-3), (-4,8,3), (-4,8,-3), (-4,-4,3), (-4,-4,-3)								
**Sapph**	**(1,6,-8)**	**10.578**	**71.79**	**3.6**	**3.2**	**3.663**	**11.54**	**45.79**	**47.22**
	Equiv. Refl.: (1,-7,8), (6,1,8), (6,-7,-8), (7,-1,8), (7,-6,-8), (-1,7,-8), (-1,-6,8), (-6,7,8), (-6,-1,-8) (-7,1,-8), (-7,6,8), (7,-1,4), (7,-6,-4), (-1,7,-4), (-1,-6,4), (-6,7,4), (-6,-1,-4)								
**Quartz**	**(1,3,-8)**	**10.573**	**71.71**	**3.3**	**2.7**	**3.681**	**9.96**	**46.01**	**47.07**
	Equiv. Refl.: (1,-4,8), (3,1,8), (3,-4,-8), (4,-1,8), (4,-3,-8), (7,-3,-5), (7,-4,5), (-1,4,-8), (-1,-3,8) (-3,4,8), (-3,7,5), (-3,-1,-8), (-3,-4,-5), (-4,1,-8), (-4,3,8), (-4,7,-5), (-4,-3,5)								
**Quartz**	**(3,4,5)**	**10.556**	**71.43**	**3.1**	**2.9**	**3.741**	**10.83**	**46.76**	**48**
	Equiv. Refl.: (3,-7,-5), (4,3,-5), (4,-7,5), (-7,3,5), (-7,4,-5)								
**Quartz**	**(3,5,3)**	**10.764**	**75.14**	**3**	**2.8**	**2.954**	**8.16**	**36.92**	**37.81**
	Equiv. Refl.: (3,-8,-3), (5,3,-3), (5,-8,3), (-8,3,3), (-8,5,-3)								
**Quartz**	**(8,-3,-3)**	**10.764**	**75.14**	**2.8**	**2.8**	**2.954**	**8.16**	**36.92**	**37.81**
	Equiv. Refl.: (8,-5,3), (-3,8,3), (-3,-5,-3), (-5,8,-3), (-5,-3,3)								
**Sapph**	**(3,5,7)**	**11.048**	**82.8**	**2.7**	**3.1**	**1.407**	**4.43**	**17.58**	**18.13**
	Equiv. Refl.: (3,-8,-7), (5,3,-7), (5,-8,7), (8,-3,-7), (8,-5,7), (-3,8,7), (-3,-5,-7), (-5,8,-7), (-5,-3,7) (-8,3,7), (-8,5,-7)								
**Quartz**	**(2,6,-1)**	**10.569**	**71.64**	**2.6**	**2.4**	**3.697**	**9.02**	**46.21**	**47.08**
	Equiv. Refl.: (2,-8,1), (6,2,1), (6,-8,-1), (8,-2,1), (8,-6,-1), (-2,8,-1), (-2,-6,1), (-6,8,1), (-6,-2,-1) (-8,2,-1), (-8,6,1)								
**LiNbO3**	**(1,6,13)**	**10.814**	**76.18**	**2.5**	**3.9**	**2.74**	**10.72**	**34.25**	**35.88**
	Equiv. Refl.: (1,-7,-13), (6,1,-13), (6,-7,13), (7,-1,-13), (7,-6,13), (-1,7,13), (-1,-6,-13), (-6,7,-13), (-6,-1,13) (-7,1,13), (-7,6,-13)								
**LiNbO3**	**(4,5,5)**	**11.088**	**84.67**	**2.4**	**5.3**	**1.039**	**5.49**	**12.99**	**14.1**
	Equiv. Refl.: (4,-9,-5), (5,4,-5), (5,-9,5), (9,-4,-5), (9,-5,5), (-4,9,5), (-4,-5,-5), (-5,9,-5), (-5,-4,5) (-9,4,5), (-9,5,-5), (1,1,9), (1,1,-9), (1,-2,9), (1,-2,-9), (6,-1,6), (6,-5,-6), (-1,6,-6) (-1,-5,6), (-2,1,9), (-2,1,-9), (-5,6,6), (-5,-1,-6)								

Ei =	11.136	keV						ΔEg	ΔEt
Cryst	Refl	EB	ΘB	∫ IR dΘ	Width	Ei cotΘ	ΔE	2m, 50μm	2m, 50μm
	(h,k,l)	[keV]	[°]	[μrad]	[μrad]	[meV/μrad]	[meV]	[meV]	[meV]
**Sapph**	**(4,4,3)**	**10.521**	**70.87**	**2.3**	**2.3**	**3.863**	**8.87**	**48.29**	**49.1**
	Equiv. Refl.: (4,4,-3), (4,-8,3), (4,-8,-3), (8,-4,3), (8,-4,-3), (-4,8,3), (-4,8,-3), (-4,-4,3), (-4,-4,-3)<br>(-8,4,3), (-8,4,-3)								
**Quartz**	**(1,2,-9)**	**11.019**	**81.7**	**2.2**	**2.5**	**1.624**	**4**	**20.3**	**20.69**
	Equiv. Refl.: (1,-3,9), (2,1,9), (2,-3,-9), (-3,1,-9), (-3,2,9)								
**LiNbO3**	**(3,6,3)**	**11.118**	**86.73**	**2.1**	**6**	**0.636**	**3.84**	**7.95**	**8.83**
	Equiv. Refl.: (3,6,-3), (3,-9,3), (3,-9,-3), (6,3,3), (6,3,-3), (6,-9,3), (6,-9,-3), (9,-3,3), (9,-3,-3)<br>(9,-6,3), (9,-6,-3), (-3,9,3), (-3,9,-3), (-3,-6,3), (-3,-6,-3), (-6,9,3), (-6,9,-3), (-6,-3,3)<br>(-6,-3,-3), (-9,3,3), (-9,3,-3), (-9,6,3), (-9,6,-3)								
**Quartz**	**(1,5,-6)**	**10.638**	**72.8**	**2**	**2.2**	**3.446**	**7.54**	**43.08**	**43.74**
	Equiv. Refl.: (1,-6,6), (2,5,-5), (2,-7,5), (5,1,6), (5,2,5), (5,-6,-6), (5,-7,-5), (-6,1,-6), (-6,5,6)<br>(-7,2,-5), (-7,5,5)								
**Quartz**	**(2,-1,9)**	**10.627**	**72.61**	**1.9**	**2.1**	**3.487**	**7.43**	**43.59**	**44.22**
	Equiv. Refl.: (2,-1,-9), (-1,2,-9), (-1,3,-9), (-1,-1,9), (-1,-2,9), (-2,3,9), (-2,-1,-9)								
**Quartz**	**(3,-1,9)**	**11.019**	**81.7**	**1.8**	**2.5**	**1.624**	**3.99**	**20.3**	**20.69**
	Equiv. Refl.: (3,-2,-9), (7,-2,5), (7,-5,-5), (-1,2,9), (-1,-1,-9), (-2,7,-5), (-2,-5,5), (-5,7,5), (-5,-2,-5)								
**Quartz**	**(0,2,9)**	**10.727**	**74.41**	**1.7**	**2**	**3.106**	**6.07**	**38.83**	**39.3**
	Equiv. Refl.: (0,-2,-9), (2,0,-9), (2,-2,9), (-2,0,9), (-2,2,-9)								
**LiNbO3**	**(2,4,-17)**	**10.58**	**71.81**	**1.6**	**2.7**	**3.659**	**9.83**	**45.74**	**46.78**
	Equiv. Refl.: (2,-6,17), (4,2,17), (4,-6,-17), (6,-2,17), (6,-4,-17), (-2,6,-17), (-2,-4,17), (-4,6,17), (-4,-2,-17)<br>(-6,2,-17), (-6,4,17), (2,6,-2), (2,-8,2), (6,2,2), (6,-8,-2), (-8,2,-2), (-8,6,2)								
**LiNbO3**	**(2,2,21)**	**10.554**	**71.39**	**1.5**	**2.5**	**3.749**	**9.47**	**46.86**	**47.81**
	Equiv. Refl.: (2,2,-21), (2,-4,21), (2,-4,-21), (4,-2,21), (4,-2,-21), (-2,4,21), (-2,4,-21), (-2,-2,21), (-2,-2,-21)<br>(-4,2,21), (-4,2,-21)								
**Quartz**	**(1,3,8)**	**10.573**	**71.71**	**1.4**	**1.6**	**3.681**	**5.81**	**46.01**	**46.37**
	Equiv. Refl.: (1,-4,-8), (2,6,0), (2,-8,0), (3,1,-8), (3,-4,8), (4,-1,-8), (4,-3,8), (6,2,0), (6,-8,0)<br>(8,-2,2), (8,-6,-2), (-1,4,8), (-1,-3,-8), (-2,8,-2), (-2,-6,2), (-3,4,-8), (-3,-1,8), (-4,1,8)<br>(-4,3,-8), (-6,8,2), (-6,-2,-2), (-8,2,0), (-8,6,0)								
**Sapph**	**(3,5,-5)**	**10.798**	**75.85**	**1.3**	**1.7**	**2.808**	**4.81**	**35.1**	**35.43**
	Equiv. Refl.: (3,-8,5), (5,3,5), (5,-8,-5), (8,-3,5), (8,-5,-5), (-3,8,-5), (-3,-5,5), (-5,8,5), (-5,-3,-5)<br>(-8,3,-5), (-8,5,5)								
**Sapph**	**(1,4,18)**	**11.015**	**81.56**	**1.2**	**1.8**	**1.653**	**2.96**	**20.66**	**20.87**
	Equiv. Refl.: (1,4,-18), (1,-5,18), (1,-5,-18), (4,1,18), (4,1,-18), (4,-5,18), (4,-5,-18), (5,-1,18), (5,-1,-18)<br>(5,-4,18), (5,-4,-18), (-1,5,18), (-1,5,-18), (-1,-4,18), (-1,-4,-18), (-4,5,18), (-4,5,-18), (-4,-1,18)<br>(-4,-1,-18), (-5,1,18), (-5,1,-18), (-5,4,18), (-5,4,-18), (0,7,3), (0,-7,-3), (1,2,9), (1,-3,-9)<br>(2,1,-9), (2,-3,9), (3,4,-5), (3,-7,5), (4,3,5), (4,-2,8), (4,-2,-8), (4,-7,-5), (7,0,-3)<br>(7,-7,3), (8,-2,0), (8,-6,0), (-2,4,8), (-2,4,-8), (-2,8,0), (-2,-2,8), (-2,-2,-8), (-2,-6,0)<br>(-3,1,9), (-3,2,-9), (-6,8,0), (-6,-2,0), (-7,0,3), (-7,3,-5), (-7,4,5), (-7,7,-3)								
**LiNbO3**	**(4,5,-1)**	**10.869**	**77.43**	**1.1**	**2.4**	**2.482**	**5.85**	**31.03**	**31.57**
	Equiv. Refl.: (4,-9,1), (5,4,1), (5,-9,-1), (9,-4,1), (9,-5,-1), (-4,9,-1), (-4,-5,1), (-5,9,1), (-5,-4,-1)<br>(-9,4,-1), (-9,5,1), (2,5,12), (2,5,-12), (2,-7,12), (2,-7,-12), (3,5,1), (3,-8,-1), (5,2,12)<br>(5,2,-12), (5,3,-1), (5,-7,12), (5,-7,-12), (5,-8,1), (7,-2,12), (7,-2,-12), (7,-5,12), (7,-5,-12)<br>(8,-3,-1), (8,-5,1), (-2,7,12), (-2,7,-12), (-2,-5,12), (-2,-5,-12), (-3,8,1), (-3,-5,-1), (-5,7,12)<br>(-5,7,-12), (-5,8,-1), (-5,-2,12), (-5,-2,-12), (-5,-3,1), (-7,2,12), (-7,2,-12), (-7,5,12), (-7,5,-12)<br>(-8,3,1), (-8,5,-1), (0,7,-3), (0,-7,3), (2,2,8), (2,2,-8), (2,-4,8), (2,-4,-8), (3,-1,-9)<br>(3,-2,9), (7,0,3), (7,-7,-3), (-1,3,9), (-1,-2,-9), (-2,3,-9), (-2,-1,9), (-4,2,8), (-4,2,-8)<br>(-7,0,-3), (-7,7,3)								
**Quartz**	**(1,6,4)**	**10.599**	**72.13**	**1**	**1.2**	**3.591**	**4.23**	**44.88**	**45.08**
	Equiv. Refl.: (1,-7,-4), (6,1,-4), (6,-7,4), (7,-3,5), (7,-4,-5), (-3,7,-5), (-3,-4,5), (-4,7,5), (-4,-3,-5)<br>(-7,1,4), (-7,6,-4)								

Ei =	11.136	keV						ΔEg	ΔEt
Cryst	Refl	EB	ΘB	∫ IR dΘ	Width	Ei cotΘ	ΔE	2m, 50µm	2m, 50µm
	(h,k,l)	[keV]	[°]	[µrad]	[µrad]	[meV/µrad]	[meV]	[meV]	[meV]
**LiNbO3**	**(1,2,23)**	**10.923**	**78.78**	**0.9**	**2.2**	**2.209**	**4.76**	**27.61**	**28.02**
	Equiv. Refl.: (1,5,17), (1,-3,-23), (1,-6,-17), (2,1,-23), (2,-3,23), (3,-1,-23), (3,-2,23), (5,1,-17), (5,-6,17)<br>(6,-1,-17), (6,-5,17), (-1,3,23), (-1,6,17), (-1,-2,-23), (-1,-5,-17), (-2,3,-23), (-2,-1,23), (-3,1,23)<br>(-3,2,-23), (-5,6,-17), (-5,-1,17), (-6,1,17), (-6,5,-17), (7,-1,-4), (7,-6,4), (-1,7,4), (-1,-6,-4)<br>(-6,7,-4), (-6,-1,4)								
**Sapph**	**(0,7,-4)**	**10.703**	**73.96**	**0.7**	**1**	**3.201**	**3.24**	**40.01**	**40.14**
	Equiv. Refl.: (0,-7,4), (7,0,4), (7,-7,-4), (-7,0,-4), (-7,7,4), (0,4,-8), (0,5,-7), (0,-4,8), (0,-5,7)<br>(4,0,8), (4,-4,-8), (5,0,7), (5,-5,-7), (-4,0,-8), (-4,4,8), (-5,0,-7), (-5,5,7)								
**LiNbO3**	**(2,5,15)**	**10.973**	**80.17**	**0.6**	**1.9**	**1.929**	**3.64**	**24.11**	**24.39**
	Equiv. Refl.: (2,5,-15), (2,-7,15), (2,-7,-15), (5,2,15), (5,2,-15), (5,-7,15), (5,-7,-15), (7,-2,15), (7,-2,-15)<br>(7,-5,15), (7,-5,-15), (-2,7,15), (-2,7,-15), (-2,-5,15), (-2,-5,-15), (-5,7,15), (-5,7,-15), (-5,-2,15)<br>(-5,-2,-15), (-7,2,15), (-7,2,-15), (-7,5,15), (-7,5,-15), (0,2,22), (0,-2,-22), (2,0,-22), (2,-2,22)<br>(-2,0,22), (-2,2,-22), (2,5,5), (2,-7,-5), (3,3,7), (3,3,-7), (3,-6,7), (3,-6,-7), (5,2,-5)<br>(5,-7,5), (7,-2,-5), (7,-5,5), (-2,7,5), (-2,-5,-5), (-5,7,-5), (-5,-2,5), (-6,3,7), (-6,3,-7)<br>(-7,2,5), (-7,5,-5)								
**Sapph**	**(1,3,19)**	**10.567**	**71.6**	**0.5**	**0.8**	**3.703**	**2.82**	**46.29**	**46.38**
	Equiv. Refl.: (1,-4,-19), (3,1,-19), (3,-4,19), (4,-1,-19), (4,-3,19), (-1,4,19), (-1,-3,-19), (-3,4,-19), (-3,-1,19)<br>(-4,1,19), (-4,3,-19)								
**Sapph**	**(3,4,-13)**	**11.056**	**83.14**	**0.4**	**1**	**1.34**	**1.27**	**16.75**	**16.8**
	Equiv. Refl.: (3,-7,13), (4,3,13), (4,-7,-13), (7,-3,13), (7,-4,-13), (-3,7,-13), (-3,-4,13), (-4,7,13), (-4,-3,-13)<br>(-7,3,-13), (-7,4,13), (6,-3,7), (6,-3,-7), (-3,6,7), (-3,6,-7), (-3,-3,7), (-3,-3,-7)								
**LiNbO3**	**(2,6,-7)**	**10.504**	**70.61**	**0.3**	**1**	**3.92**	**4.08**	**49**	**49.17**
	Equiv. Refl.: (2,-8,7), (3,5,-11), (3,-8,11), (5,3,11), (5,-8,-11), (6,2,7), (6,-8,-7), (8,-2,7), (8,-3,11)<br>(8,-5,-11), (8,-6,-7), (-2,8,-7), (-2,-6,7), (-3,8,-11), (-3,-5,11), (-5,8,11), (-5,-3,-11), (-6,8,7)<br>(-6,-2,-7), (-8,2,-7), (-8,3,-11), (-8,5,11), (-8,6,7)								
**LiNbO3**	**(1,7,3)**	**10.583**	**71.87**	**0.2**	**0.9**	**3.646**	**3.2**	**45.57**	**45.68**
	Equiv. Refl.: (1,7,-3), (1,-8,3), (1,-8,-3), (7,1,3), (7,1,-3), (7,-8,3), (7,-8,-3), (8,-1,3), (8,-1,-3)<br>(8,-7,3), (8,-7,-3), (-1,8,3), (-1,8,-3), (-1,-7,3), (-1,-7,-3), (-7,8,3), (-7,8,-3), (-7,-1,3)<br>(-7,-1,-3), (-8,1,3), (-8,1,-3), (-8,7,3), (-8,7,-3), (0,1,-22), (0,-1,22), (1,0,22), (1,-1,-22)<br>(2,6,2), (2,-8,-2), (3,4,11), (3,-7,-11), (4,3,-11), (4,-7,11), (6,2,-2), (6,-8,2), (7,-3,-11)<br>(7,-4,11), (8,-2,-2), (8,-6,2), (-1,0,-22), (-1,1,22), (-2,8,2), (-2,-6,-2), (-3,7,11), (-3,-4,-11)<br>(-4,7,-11), (-4,-3,11), (-6,8,-2), (-6,-2,2), (-7,3,11), (-7,4,-11), (-8,2,2), (-8,6,-2)								
**Sapph**	**(3,3,15)**	**10.6**	**72.16**	**0**	**0.2**	**3.585**	**0.64**	**44.81**	**44.82**
	Equiv. Refl.: (3,3,-15), (3,-6,15), (3,-6,-15), (6,-3,15), (6,-3,-15), (-3,6,15), (-3,6,-15), (-3,-3,15), (-3,-3,-15)<br>(-6,3,15), (-6,3,-15)								

Ei =	9.881	keV						ΔEg	ΔEt
**Cryst**	**Refl**	**EB**	**ΘB**	**∫IR dΘ**	**Width**	**Ei cotΘ**	**ΔE**	**2m, 50μm**	**2m, 50μm**
	**(h,k,l)**	**[keV]**	**[°]**	**[μrad]**	**[μrad]**	**[meV/μrad]**	**[meV]**	**[meV]**	**[meV]**
**Ge**	**(0,4,8)**	**9.801**	**82.7**	**31.714**	**30**	**1.265**	**38**	**15.82**	**41.16**
**Ge**	**(0,6,6)**	**9.298**	**70.22**	**14.687**	**13.2**	**3.554**	**46.96**	**44.42**	**64.64**
	Equiv. Refl.: (2,2,8)								
**Si**	**(0,6,6)**	**9.685**	**78.58**	**13.517**	**11.5**	**1.995**	**23.02**	**24.94**	**33.94**
	Equiv. Refl.: (2,2,8)								
**Ge**	**(5,5,5)**	**9.49**	**73.82**	**10.687**	**10.7**	**2.867**	**30.61**	**35.83**	**47.13**
	Equiv. Refl.: (1,5,7)								
**Si**	**(3,3,7)**	**9.343**	**71.01**	**6.251**	**5.5**	**3.401**	**18.66**	**42.51**	**46.43**
**Quartz**	**(0,6,-4)**	**9.873**	**87.66**	**36.4**	**32.6**	**0.404**	**13.17**	**5.05**	**14.1**
	Equiv. Refl.: (0,-6,4), (6,0,4), (6,-6,-4), (-6,0,-4), (-6,6,4)								
**LiNbO3**	**(1,-7,8)**	**9.795**	**82.42**	**31.9**	**29.4**	**1.314**	**38.68**	**16.43**	**42.02**
	Equiv. Refl.: (6,1,8), (7,-1,8), (-1,-6,8), (-6,7,8), (-7,6,8)								
**LiNbO3**	**(0,-7,-2)**	**9.774**	**81.58**	**30.8**	**28.6**	**1.463**	**41.83**	**18.29**	**45.66**
	Equiv. Refl.: (7,0,-2), (-7,7,-2)								
**LiNbO3**	**(0,7,2)**	**9.774**	**81.58**	**29.2**	**28.6**	**1.463**	**41.83**	**18.29**	**45.66**
	Equiv. Refl.: (7,-7,2), (-7,0,2)								
**LiNbO3**	**(1,6,-8)**	**9.795**	**82.42**	**29**	**29.4**	**1.314**	**38.68**	**16.43**	**42.02**
	Equiv. Refl.: (6,-7,-8), (7,-6,-8), (-1,7,-8), (-6,-1,-8), (-7,1,-8)								
**Sapph**	**(2,5,6)**	**9.822**	**83.73**	**24.2**	**20.9**	**1.086**	**22.73**	**13.57**	**26.48**
	Equiv. Refl.: (2,5,-6), (2,-7,6), (2,-7,-6), (5,2,6), (5,2,-6), (5,-7,6), (5,-7,-6), (7,-2,6), (7,-2,-6) (7,-5,6), (7,-5,-6), (-2,7,6), (-2,7,-6), (-2,-5,6), (-2,-5,-6), (-5,7,6), (-5,7,-6), (-5,-2,6) (-5,-2,-6), (-7,2,6), (-7,2,-6), (-7,5,6), (-7,5,-6)								
**LiNbO3**	**(2,4,-14)**	**9.661**	**77.88**	**24**	**21.9**	**2.122**	**46.55**	**26.52**	**53.58**
	Equiv. Refl.: (4,-6,-14), (6,-4,-14), (-2,6,-14), (-4,-2,-14), (-6,2,-14)								
**LiNbO3**	**(2,-6,14)**	**9.661**	**77.88**	**22.7**	**21.9**	**2.122**	**46.55**	**26.52**	**53.58**
	Equiv. Refl.: (4,2,14), (6,-2,14), (-2,-4,14), (-4,6,14), (-6,4,14)								
**LiNbO3**	**(3,4,-10)**	**9.567**	**75.53**	**22.1**	**20.1**	**2.551**	**51.32**	**31.88**	**60.42**
	Equiv. Refl.: (4,-7,-10), (7,-4,-10), (-3,7,-10), (-4,-3,-10), (-7,3,-10)								
**LiNbO3**	**(3,-7,10)**	**9.567**	**75.53**	**21.3**	**20.1**	**2.551**	**51.32**	**31.88**	**60.42**
	Equiv. Refl.: (4,3,10), (7,-3,10), (-3,-4,10), (-4,7,10), (-7,4,10), (7,-1,-2), (7,-6,2), (-1,7,2), (-1,-6,-2) (-6,7,-2), (-6,-1,2)								
**LiNbO3**	**(1,-3,-20)**	**9.671**	**78.16**	**21.1**	**20.1**	**2.072**	**41.63**	**25.9**	**49.03**
	Equiv. Refl.: (2,1,-20), (3,-1,-20), (-1,-2,-20), (-2,3,-20), (-3,2,-20)								
**LiNbO3**	**(1,2,20)**	**9.671**	**78.16**	**20.9**	**20.1**	**2.072**	**41.63**	**25.9**	**49.03**
	Equiv. Refl.: (2,-3,20), (3,-2,20), (-1,3,20), (-2,-1,20), (-3,1,20)								
**Quartz**	**(1,6,2)**	**9.826**	**83.93**	**20.5**	**17.9**	**1.05**	**18.8**	**13.13**	**22.93**
	Equiv. Refl.: (1,-7,-2), (6,1,-2), (6,-7,2), (-7,1,2), (-7,6,-2)								
**LiNbO3**	**(3,-8,2)**	**9.774**	**81.58**	**18.7**	**19**	**1.463**	**27.76**	**18.29**	**33.25**
	Equiv. Refl.: (5,3,2), (8,-3,2), (-3,-5,2), (-5,8,2), (-8,5,2)								
**LiNbO3**	**(4,4,0)**	**9.634**	**77.15**	**18.5**	**17.9**	**2.254**	**40.27**	**28.17**	**49.15**
	Equiv. Refl.: (4,-8,0), (8,-4,0), (-4,8,0), (-4,-4,0), (-8,4,0)								
**Sapph**	**(0,3,18)**	**9.705**	**79.16**	**18.3**	**15.4**	**1.893**	**29.11**	**23.66**	**37.51**
	Equiv. Refl.: (0,3,-18), (0,-3,18), (0,-3,-18), (3,0,18), (3,0,-18), (3,-3,18), (3,-3,-18), (-3,0,18), (-3,0,-18) (-3,3,18), (-3,3,-18)								
**LiNbO3**	**(3,5,-2)**	**9.774**	**81.58**	**17.9**	**19**	**1.463**	**27.76**	**18.29**	**33.25**
	Equiv. Refl.: (5,-8,-2), (8,-5,-2), (-3,8,-2), (-5,-3,-2), (-8,3,-2)								
**LiNbO3**	**(0,-5,14)**	**9.356**	**71.23**	**17.8**	**15.8**	**3.357**	**52.98**	**41.96**	**67.58**
	Equiv. Refl.: (5,0,14), (-5,5,14)								

Ei =	9.881	keV						ΔEg	ΔEt
Cryst	Refl	EB	ΘB	∫ IR dΘ	Width	Ei cotΘ	ΔE	2m, 50µm	2m, 50µm
	(h,k,l)	[keV]	[°]	[µrad]	[µrad]	[meV/µrad]	[meV]	[meV]	[meV]
**LiNbO3**	**(0,5,-14)**	**9.356**	**71.23**	**16.1**	**15.8**	**3.357**	**52.98**	**41.96**	**67.58**
	Equiv. Refl.: (5,-5,-14), (-5,0,-14)								
**LiNbO3**	**(2,2,-18)**	**9.38**	**71.68**	**14.9**	**13.9**	**3.271**	**45.52**	**40.89**	**61.19**
	Equiv. Refl.: (2,3,-16), (2,-4,-18), (3,-5,-16), (4,-2,-18), (5,-3,-16), (-2,4,-18), (-2,5,-16), (-2,-2,-18), (-3,-2,-16) (-4,2,-18), (-5,2,-16)								
**LiNbO3**	**(2,2,18)**	**9.38**	**71.68**	**14.3**	**13.9**	**3.271**	**45.52**	**40.89**	**61.19**
	Equiv. Refl.: (2,-4,18), (4,-2,18), (-2,4,18), (-2,-2,18), (-4,2,18)								
**LiNbO3**	**(2,-5,16)**	**9.377**	**71.62**	**13.6**	**13.6**	**3.283**	**44.74**	**41.04**	**60.71**
	Equiv. Refl.: (3,2,16), (5,-2,16), (-2,-3,16), (-3,5,16), (-5,3,16)								
**LiNbO3**	**(0,2,-20)**	**9.366**	**71.42**	**13.4**	**12.4**	**3.322**	**41.26**	**41.52**	**58.54**
	Equiv. Refl.: (2,-2,-20), (-2,0,-20)								
**Sapph**	**(0,1,20)**	**9.664**	**77.96**	**13.3**	**11.1**	**2.107**	**23.41**	**26.34**	**35.24**
	Equiv. Refl.: (0,-1,-20), (1,0,-20), (1,-1,20), (-1,0,20), (-1,1,-20)								
**LiNbO3**	**(0,-2,20)**	**9.366**	**71.42**	**12.6**	**12.4**	**3.322**	**41.26**	**41.52**	**58.54**
	Equiv. Refl.: (2,0,20), (-2,2,20)								
**LiNbO3**	**(1,6,4)**	**9.292**	**70.11**	**12.3**	**11.5**	**3.574**	**41.1**	**44.68**	**60.7**
	Equiv. Refl.: (6,-7,4), (7,-6,4), (-1,7,4), (-6,-1,4), (-7,1,4)								
**LiNbO3**	**(1,-7,-4)**	**9.292**	**70.11**	**11.7**	**11.5**	**3.574**	**41.1**	**44.68**	**60.7**
	Equiv. Refl.: (6,1,-4), (7,-1,-4), (-1,-6,-4), (-6,7,-4), (-7,6,-4)								
**Quartz**	**(1,4,-6)**	**9.589**	**76.03**	**11.1**	**9.1**	**2.458**	**22.43**	**30.73**	**38.05**
	Equiv. Refl.: (1,-5,6), (4,1,6), (4,-5,-6), (5,-1,6), (5,-4,-6), (-1,5,-6), (-1,-4,6), (-4,5,6), (-4,-1,-6) (-5,1,-6), (-5,4,6)								
**Sapph**	**(1,6,1)**	**9.877**	**88.31**	**10.6**	**14.3**	**0.292**	**4.16**	**3.65**	**5.54**
	Equiv. Refl.: (1,-7,-1), (2,3,14), (2,-5,-14), (3,2,-14), (3,-5,14), (5,-2,-14), (5,-3,14), (6,1,-1), (6,-7,1) (7,-1,-1), (7,-6,1), (-1,7,1), (-1,-6,-1), (-2,5,14), (-2,-3,-14), (-3,5,-14), (-3,-2,14), (-5,2,14) (-5,3,-14), (-6,7,-1), (-6,-1,1), (-7,1,1), (-7,6,-1)								
**Sapph**	**(3,3,12)**	**9.691**	**78.74**	**10.1**	**9**	**1.967**	**17.71**	**24.59**	**30.3**
	Equiv. Refl.: (3,3,-12), (3,-6,12), (3,-6,-12), (6,-3,12), (6,-3,-12), (-3,6,12), (-3,6,-12), (-3,-3,12), (-3,-3,-12) (-6,3,12), (-6,3,-12)								
**Sapph**	**(1,5,-10)**	**9.641**	**77.34**	**8.8**	**7.7**	**2.22**	**17.04**	**27.75**	**32.56**
	Equiv. Refl.: (1,-6,10), (5,1,10), (5,-6,-10), (6,-1,10), (6,-5,-10), (-1,6,-10), (-1,-5,10), (-5,6,10), (-5,-1,-10) (-6,1,-10), (-6,5,10)								
**Quartz**	**(4,-1,7)**	**9.595**	**76.18**	**8.5**	**7**	**2.431**	**17.13**	**30.38**	**34.88**
	Equiv. Refl.: (4,-3,-7), (7,-1,0), (7,-6,0), (-1,4,-7), (-1,7,0), (-1,-3,7), (-1,-6,0), (-3,4,7), (-3,-1,-7) (-6,7,0), (-6,-1,0)								
**Quartz**	**(1,6,0)**	**9.554**	**75.22**	**8.4**	**7.1**	**2.607**	**18.49**	**32.59**	**37.47**
	Equiv. Refl.: (1,-7,0), (6,1,0), (6,-7,0), (-7,1,0), (-7,6,0)								
**Quartz**	**(0,6,3)**	**9.395**	**71.95**	**8.2**	**6.8**	**3.22**	**21.86**	**40.25**	**45.8**
	Equiv. Refl.: (0,-6,-3), (6,0,-3), (6,-6,3), (-6,0,3), (-6,6,-3)								
**Quartz**	**(1,3,-7)**	**9.595**	**76.18**	**8.1**	**7.1**	**2.431**	**17.14**	**30.38**	**34.88**
	Equiv. Refl.: (1,-4,7), (3,1,7), (3,-4,-7), (-4,1,-7), (-4,3,7)								
**Quartz**	**(0,1,-8)**	**9.291**	**70.1**	**7.5**	**6.2**	**3.577**	**22.09**	**44.71**	**49.87**
	Equiv. Refl.: (0,-1,8), (1,0,8), (1,-1,-8), (-1,0,-8), (-1,1,8)								
**LiNbO3**	**(1,3,19)**	**9.865**	**86.76**	**7.1**	**14.3**	**0.559**	**7.98**	**6.98**	**10.61**
	Equiv. Refl.: (1,-4,-19), (3,1,-19), (3,-4,19), (4,-1,-19), (4,-3,19), (-1,4,19), (-1,-3,-19), (-3,4,-19), (-3,-1,19) (-4,1,19), (-4,3,-19), (7,-2,2), (7,-5,-2), (-2,7,-2), (-2,-5,2), (-5,7,2), (-5,-2,-2)								
**Quartz**	**(2,5,-2)**	**9.384**	**71.74**	**6.9**	**5.8**	**3.26**	**18.95**	**40.74**	**44.93**
	Equiv. Refl.: (2,-7,2), (5,2,2), (5,-7,-2), (-7,2,-2), (-7,5,2)								
**Quartz**	**(1,6,-2)**	**9.826**	**83.93**	**6.4**	**6.2**	**1.05**	**6.48**	**13.13**	**14.64**
	Equiv. Refl.: (1,-7,2), (6,1,2), (6,-7,-2), (-7,1,-2), (-7,6,2)								

Ei =	9.881	keV						ΔEg	ΔEt
Cryst	Refl	EB	ΘB	∫ IR dΘ	Width	Ei cotΘ	ΔE	2m, 50µm	2m, 50µm
	(h,k,l)	[keV]	[°]	[µrad]	[µrad]	[meV/µrad]	[meV]	[meV]	[meV]
**Quartz**	**(3,4,-3)**	**9.507**	**74.19**	**6.2**	**5.4**	**2.798**	**15.12**	**34.98**	**38.11**
	Equiv. Refl.: (3,-7,3), (7,-3,3), (7,-4,-3), (-3,7,-3), (-3,-4,3), (-4,7,3), (-4,-3,-3), (-7,3,-3), (-7,4,3)								
**Quartz**	**(4,3,3)**	**9.507**	**74.19**	**6.1**	**5.4**	**2.798**	**15.12**	**34.98**	**38.11**
	Equiv. Refl.: (4,-7,-3), (6,-3,5), (6,-3,-5), (-3,6,5), (-3,6,-5), (-3,-3,5), (-3,-3,-5)								
**Sapph**	**(2,5,0)**	**9.395**	**71.96**	**6**	**5.2**	**3.219**	**16.65**	**40.23**	**43.54**
	Equiv. Refl.: (2,-7,0), (5,2,0), (5,-7,0), (7,-2,0), (7,-5,0), (-2,7,0), (-2,-5,0), (-5,7,0), (-5,-2,0) (-7,2,0), (-7,5,0)								
**Quartz**	**(3,3,5)**	**9.498**	**73.99**	**5.9**	**5.2**	**2.835**	**14.84**	**35.44**	**38.42**
	Equiv. Refl.: (3,3,-5), (3,-6,5), (3,-6,-5), (-6,3,5), (-6,3,-5)								
**Quartz**	**(7,-1,2)**	**9.826**	**83.93**	**5.8**	**6.2**	**1.05**	**6.48**	**13.13**	**14.64**
	Equiv. Refl.: (7,-6,-2), (-1,7,-2), (-1,-6,2), (-6,7,2), (-6,-1,-2)								
**Quartz**	**(1,5,4)**	**9.32**	**70.6**	**5.5**	**4.6**	**3.48**	**15.89**	**43.51**	**46.32**
	Equiv. Refl.: (1,-6,-4), (5,1,-4), (5,-6,4), (-6,1,4), (-6,5,-4)								
**Quartz**	**(2,5,2)**	**9.384**	**71.74**	**5.4**	**4.5**	**3.26**	**14.81**	**40.74**	**43.35**
	Equiv. Refl.: (2,-7,-2), (5,2,-2), (5,-7,2), (6,-1,-4), (6,-5,4), (7,-2,-2), (7,-5,2), (-1,6,4), (-1,-5,-4) (-2,7,2), (-2,-5,-2), (-5,6,-4), (-5,7,-2), (-5,-1,4), (-5,-2,2), (-7,2,2), (-7,5,-2)								
**LiNbO3**	**(1,1,21)**	**9.695**	**78.85**	**5**	**7.2**	**1.947**	**13.98**	**24.33**	**28.06**
	Equiv. Refl.: (1,1,-21), (1,-2,21), (1,-2,-21), (2,-1,21), (2,-1,-21), (-1,2,21), (-1,2,-21), (-1,-1,21), (-1,-1,-21) (-2,1,21), (-2,1,-21)								
**Quartz**	**(1,3,7)**	**9.595**	**76.18**	**4.7**	**4.1**	**2.431**	**9.95**	**30.38**	**31.97**
	Equiv. Refl.: (1,-4,-7), (3,1,-7), (3,-4,7), (-4,1,7), (-4,3,-7)								
**Quartz**	**(2,5,-3)**	**9.728**	**79.9**	**4.5**	**4.3**	**1.76**	**7.52**	**22.01**	**23.26**
	Equiv. Refl.: (2,-7,3), (5,2,3), (5,-7,-3), (-7,2,-3), (-7,5,3)								
**Quartz**	**(4,-1,-7)**	**9.595**	**76.18**	**4.3**	**4.1**	**2.431**	**9.94**	**30.38**	**31.97**
	Equiv. Refl.: (4,-3,7), (7,-2,3), (7,-5,-3), (-1,4,7), (-1,-3,-7), (-2,7,-3), (-2,-5,3), (-3,4,-7), (-3,-1,7) (-5,7,3), (-5,-2,-3)								
**LiNbO3**	**(1,6,7)**	**9.64**	**77.33**	**4.2**	**6.3**	**2.221**	**13.93**	**27.77**	**31.07**
	Equiv. Refl.: (1,-7,-7), (6,1,-7), (6,-7,7), (7,-1,-7), (7,-6,7), (-1,7,7), (-1,-6,-7), (-6,7,-7), (-6,-1,7) (-7,1,7), (-7,6,-7), (2,5,3), (2,5,-3), (2,-7,3), (2,-7,-3), (5,2,3), (5,2,-3), (5,-7,3) (5,-7,-3), (7,-2,3), (7,-2,-3), (7,-5,3), (7,-5,-3), (-2,7,3), (-2,7,-3), (-2,-5,3), (-2,-5,-3) (-5,7,3), (-5,7,-3), (-5,-2,3), (-5,-2,-3), (-7,2,3), (-7,2,-3), (-7,5,3), (-7,5,-3), (6,-2,-5) (6,-4,5), (7,-1,-1), (7,-6,1), (-1,7,1), (-1,-6,-1), (-2,6,5), (-2,-4,-5), (-4,6,-5), (-4,-2,5) (-6,7,-1), (-6,-1,1)								
**Quartz**	**(6,-2,5)**	**9.609**	**76.52**	**4.1**	**3.7**	**2.368**	**8.88**	**29.6**	**30.91**
	Equiv. Refl.: (6,-4,-5), (-2,6,-5), (-2,-4,5), (-4,6,5), (-4,-2,-5)								
**Quartz**	**(1,6,1)**	**9.623**	**76.87**	**4**	**3.8**	**2.305**	**8.8**	**28.81**	**30.12**
	Equiv. Refl.: (1,-7,-1), (2,4,-5), (2,-6,5), (4,2,5), (4,-6,-5), (6,1,-1), (6,-7,1), (-6,2,-5), (-6,4,5) (-7,1,1), (-7,6,-1)								
**Quartz**	**(2,4,5)**	**9.609**	**76.52**	**3.9**	**3.7**	**2.368**	**8.88**	**29.6**	**30.91**
	Equiv. Refl.: (2,-6,-5), (4,2,-5), (4,-6,5), (-6,2,5), (-6,4,-5)								
**Sapph**	**(1,3,16)**	**9.367**	**71.44**	**3.7**	**3.5**	**3.318**	**11.53**	**41.47**	**43.05**
	Equiv. Refl.: (1,-4,-16), (3,1,-16), (3,-4,16), (4,-1,-16), (4,-3,16), (-1,4,16), (-1,-3,-16), (-3,4,-16), (-3,-1,16) (-4,1,16), (-4,3,-16)								
**Quartz**	**(0,2,-8)**	**9.628**	**77**	**3.6**	**3.4**	**2.281**	**7.68**	**28.52**	**29.53**
	Equiv. Refl.: (0,-2,8), (2,0,8), (2,-2,-8), (-2,0,-8), (-2,2,8)								
**Quartz**	**(1,1,8)**	**9.517**	**74.4**	**3.4**	**2.8**	**2.76**	**7.77**	**34.49**	**35.36**
	Equiv. Refl.: (1,-2,-8), (-2,1,8), (-2,1,-8)								
**Quartz**	**(1,1,-8)**	**9.517**	**74.4**	**3.3**	**2.8**	**2.76**	**7.76**	**34.49**	**35.36**
	Equiv. Refl.: (1,-2,8)								

Ei =	9.881	keV						ΔEg	ΔEt
**Cryst**	**Refl**	**EB**	**ΘB**	**∫ IR dΘ**	**Width**	**Ei cotΘ**	**ΔE**	**2m, 50µm**	**2m, 50µm**
	**(h,k,l)**	**[keV]**	**[°]**	**[µrad]**	**[µrad]**	**[meV/µrad]**	**[meV]**	**[meV]**	**[meV]**
**LiNbO3**	**(1,6,-5)**	**9.388**	**71.83**	**3.2**	**4.7**	**3.243**	**15.22**	**40.54**	**43.3**
	Equiv. Refl.: (1,-7,5), (6,1,5), (6,-7,-5), (7,-1,5), (7,-6,-5), (-1,7,-5), (-1,-6,5), (-6,7,5), (-6,-1,-5) (-7,1,-5), (-7,6,5), (0,4,-16), (0,-4,16), (4,0,16), (4,-4,-16), (-4,0,-16), (-4,4,16)								
**Quartz**	**(0,6,4)**	**9.873**	**87.66**	**3**	**5.3**	**0.404**	**2.15**	**5.05**	**5.48**
	Equiv. Refl.: (0,-6,-4), (6,0,-4), (6,-6,4), (-6,0,4), (-6,6,-4)								
**Quartz**	**(0,6,-3)**	**9.395**	**71.95**	**2.8**	**2.6**	**3.22**	**8.24**	**40.25**	**41.08**
	Equiv. Refl.: (0,-6,3), (6,0,3), (6,-6,-3), (-6,0,-3), (-6,6,3)								
**LiNbO3**	**(3,4,11)**	**9.784**	**81.98**	**2.7**	**5.8**	**1.392**	**8.06**	**17.4**	**19.18**
	Equiv. Refl.: (3,-7,-11), (4,3,-11), (4,-7,11), (7,-3,-11), (7,-4,11), (-3,7,11), (-3,-4,-11), (-4,7,-11), (-4,-3,11) (-7,3,11), (-7,4,-11), (2,4,-11), (2,-6,11), (4,2,11), (4,-6,-11), (6,-2,11), (6,-4,-11), (-2,6,-11) (-2,-4,11), (-4,6,11), (-4,-2,-11), (-6,2,-11), (-6,4,11)								
**Sapph**	**(3,4,-4)**	**9.348**	**71.1**	**2.5**	**2.7**	**3.384**	**9.13**	**42.3**	**43.27**
	Equiv. Refl.: (3,-7,4), (4,3,4), (4,-7,-4), (7,-3,4), (7,-4,-4), (-3,7,-4), (-3,-4,4), (-4,7,4), (-4,-3,-4) (-7,3,-4), (-7,4,4), (2,-1,8), (2,-1,-8), (-1,2,8), (-1,2,-8), (-1,-1,8), (-1,-1,-8)								
**LiNbO3**	**(2,4,13)**	**9.377**	**71.62**	**2.4**	**4**	**3.282**	**13.04**	**41.03**	**43.05**
	Equiv. Refl.: (2,-6,-13), (4,2,-13), (4,-6,13), (6,-2,-13), (6,-4,13), (-2,6,13), (-2,-4,-13), (-4,6,-13), (-4,-2,13) (-6,2,13), (-6,4,-13)								
**Quartz**	**(3,4,3)**	**9.507**	**74.19**	**2**	**2.3**	**2.798**	**6.3**	**34.98**	**35.54**
	Equiv. Refl.: (3,-7,-3), (4,3,-3), (4,-7,3), (-7,3,3), (-7,4,-3)								
**Quartz**	**(1,6,-1)**	**9.623**	**76.87**	**1.9**	**2.3**	**2.305**	**5.36**	**28.81**	**29.3**
	Equiv. Refl.: (1,-7,1), (6,1,1), (6,-7,-1), (-7,1,-1), (-7,6,1)								
**Quartz**	**(0,1,8)**	**9.291**	**70.1**	**1.8**	**2.1**	**3.577**	**7.63**	**44.71**	**45.35**
	Equiv. Refl.: (0,2,8), (0,-1,-8), (0,-2,-8), (1,0,-8), (1,-1,8), (2,0,-8), (2,-2,8), (4,-2,7), (4,-2,-7) (7,-1,1), (7,-6,-1), (-1,0,8), (-1,1,-8), (-1,-7,-1), (-1,-6,1), (-2,0,8), (-2,2,-8), (-2,4,7) (-2,4,-7), (-2,-2,7), (-2,-2,-7), (-6,7,1), (-6,-1,-1)								
**Quartz**	**(7,-3,-3)**	**9.507**	**74.19**	**1.7**	**2.3**	**2.798**	**6.31**	**34.98**	**35.54**
	Equiv. Refl.: (7,-4,3), (-3,7,3), (-3,-4,-3), (-4,7,-3), (-4,-3,3)								
**Quartz**	**(2,2,7)**	**9.484**	**73.7**	**1.6**	**2**	**2.89**	**5.92**	**36.12**	**36.61**
	Equiv. Refl.: (2,2,-7), (2,-4,7), (2,-4,-7), (-4,2,7), (-4,2,-7)								
**LiNbO3**	**(1,5,-13)**	**9.682**	**78.47**	**1.3**	**3.3**	**2.016**	**6.68**	**25.2**	**26.07**
	Equiv. Refl.: (1,-6,13), (5,1,13), (5,-6,-13), (6,-1,13), (6,-5,-13), (-1,6,-13), (-1,-5,13), (-5,6,13), (-5,-1,-13) (-6,1,-13), (-6,5,13), (1,3,-17), (1,-4,17), (3,1,17), (3,-4,-17), (4,-1,17), (4,-3,-17), (-1,4,-17) (-1,-3,17), (-3,4,17), (-3,-1,-17), (-4,1,-17), (-4,3,17)								
**Quartz**	**(5,-2,6)**	**9.365**	**71.4**	**1.1**	**1.4**	**3.326**	**4.56**	**41.58**	**41.83**
	Equiv. Refl.: (5,-3,-6), (-2,5,-6), (-2,-3,6), (-3,5,6), (-3,-2,-6)								
**Quartz**	**(2,3,-6)**	**9.365**	**71.4**	**1**	**1.4**	**3.326**	**4.55**	**41.58**	**41.83**
	Equiv. Refl.: (2,-5,6), (3,2,6), (3,-5,-6), (-5,2,-6), (-5,3,6)								
**Sapph**	**(0,6,6)**	**9.47**	**73.41**	**0.9**	**1.4**	**2.943**	**3.98**	**36.79**	**37**
	Equiv. Refl.: (0,6,-6), (0,-6,6), (0,-6,-6), (6,0,6), (6,0,-6), (6,-6,6), (6,-6,-6), (-6,0,6), (-6,0,-6) (-6,6,6), (-6,6,-6)								
**Quartz**	**(-2,7,3)**	**9.728**	**79.9**	**0.8**	**1.2**	**1.76**	**2.16**	**22.01**	**22.11**
	Equiv. Refl.: (-2,-5,-3)								
**Quartz**	**(7,-2,-3)**	**9.728**	**79.9**	**0.7**	**1.2**	**1.76**	**2.16**	**22.01**	**22.11**
	Equiv. Refl.: (7,-5,3), (-5,7,-3), (-5,-2,3)								
**LiNbO3**	**(2,5,9)**	**9.571**	**75.61**	**0.6**	**2**	**2.536**	**5.08**	**31.7**	**32.1**
	Equiv. Refl.: (2,5,-9), (2,-7,9), (2,-7,-9), (5,2,9), (5,2,-9), (5,-7,9), (5,-7,-9), (7,-2,9), (7,-2,-9) (7,-5,9), (7,-5,-9), (-2,7,9), (-2,7,-9), (-2,-5,9), (-2,-5,-9), (-5,7,9), (-5,7,-9), (-5,-2,9) (-5,-2,-9), (-7,2,9), (-7,2,-9), (-7,5,9), (-7,5,-9), (2,5,3), (2,-7,-3), (5,2,-3), (5,-7,3) (-7,2,3), (-7,5,-3)								

Ei =	9.881	keV						ΔEg	ΔEt
**Cryst**	**Refl**	**EB**	**ΘB**	**∫ IR dΘ**	**Width**	**Ei cotΘ**	**ΔE**	**2m, 50μm**	**2m, 50μm**
	**(h,k,l)**	**[keV]**	**[°]**	**[μrad]**	**[μrad]**	**[meV/μrad]**	**[meV]**	**[meV]**	**[meV]**
**LiNbO3**	**(2,3,17)**	**9.722**	**79.72**	**0.5**	**2.1**	**1.792**	**3.68**	**22.4**	**22.7**
	Equiv. Refl.: (2,-5,-17), (3,2,-17), (3,3,15), (3,3,-15), (3,5,1), (3,-5,17), (3,-6,15), (3,-6,-15), (3,-8,-1) (5,3,-1), (5,-2,-17), (5,-3,17), (5,-8,1), (6,-3,15), (6,-3,-15), (8,-3,-1), (8,-5,1), (-2,5,17) (-2,-3,-17), (-3,5,-17), (-3,6,15), (-3,6,-15), (-3,8,1), (-3,-2,17), (-3,-3,15), (-3,-3,-15), (-3,-5,-1) (-5,2,17), (-5,3,-17), (-5,8,-1), (-5,-3,1), (-6,3,15), (-6,3,-15), (-8,3,1), (-8,5,-1), (5,-1,-6) (5,-4,6), (-1,5,6), (-1,-4,-6), (-4,5,-6), (-4,-1,6)								
**Sapph**	**(3,4,-7)**	**9.742**	**80.37**	**0.4**	**1.1**	**1.676**	**1.79**	**20.95**	**21.03**
	Equiv. Refl.: (3,-7,7), (4,3,7), (4,-7,-7), (7,-3,7), (7,-4,-7), (-3,7,-7), (-3,-4,7), (-4,7,7), (-4,-3,-7) (-7,3,-7), (-7,4,7), (2,3,6), (2,-5,-6), (3,2,-6), (3,-5,6), (-5,2,6), (-5,3,-6)								
**Sapph**	**(3,4,5)**	**9.457**	**73.16**	**0.3**	**0.7**	**2.991**	**1.99**	**37.39**	**37.45**
	Equiv. Refl.: (3,-7,-5), (4,3,-5), (4,-7,5), (7,-3,-5), (7,-4,5), (-3,7,5), (-3,-4,-5), (-4,7,-5), (-4,-3,5) (-7,3,5), (-7,4,-5), (1,4,6), (1,-5,-6), (4,1,-6), (4,-5,6), (5,-2,-6), (5,-3,6), (-2,5,6) (-2,-3,-6), (-3,5,-6), (-3,-2,6), (-5,1,6), (-5,4,-6)								
**Quartz**	**(1,5,-4)**	**9.32**	**70.6**	**0.2**	**0.5**	**3.48**	**1.69**	**43.51**	**43.54**
	Equiv. Refl.: (1,-6,4), (5,1,4), (5,-6,-4), (6,-1,4), (6,-5,-4), (-1,6,-4), (-1,-5,4), (-5,6,4), (-5,-1,-4) (-6,1,-4), (-6,5,4)								
**LiNbO3**	**(4,4,3)**	**9.727**	**79.86**	**0**	**0.6**	**1.768**	**1.06**	**22.1**	**22.12**
	Equiv. Refl.: (4,4,-3), (4,-8,3), (4,-8,-3), (8,-4,3), (8,-4,-3), (-4,8,3), (-4,8,-3), (-4,-4,3), (-4,-4,-3) (-8,4,3), (-8,4,-3)								

Ei = Cryst	12.1 Refl (h,k,l)	keV EB [keV]	ΘB [°]	∫ IR dΘ [μrad]	Width [μrad]	Ei cotΘ [meV/μrad]	ΔE [meV]	ΔEg 2m, 50μm [meV]	ΔEt 2m, 50μm [meV]
**Ge**	**(2,4,10)**	**12.004**	**82.77**	**6.3409**	**13.52**	**1.536**	**20.76**	**19.2**	**28.28**
	Equiv. Refl.: (2,10,4), (4,2,10), (4,10,2), (10,2,4), (10,4,2)								
**Si**	**(0,2,10)**	**11.64**	**74.16**	**4.6661**	**3.85**	**3.4332**	**13.22**	**42.92**	**44.91**
	Equiv. Refl.: (0,10,2), (2,0,10), (2,6,8), (2,8,6), (2,10,0), (6,2,8), (6,8,2), (8,2,6), (8,6,2) (10,0,2), (10,2,0)								
**Si**	**(1,5,9)**	**11.807**	**77.37**	**3.6135**	**3.25**	**2.7114**	**8.8**	**33.89**	**35.02**
	Equiv. Refl.: (1,9,5), (3,7,7), (5,1,9), (5,9,1), (7,3,7), (7,7,3), (9,1,5), (9,5,1)								
**Ge**	**(3,5,9)**	**11.751**	**76.2**	**1.9701**	**5.42**	**2.9711**	**16.09**	**37.14**	**40.48**
	Equiv. Refl.: (3,9,5), (5,3,9), (5,9,3), (9,3,5), (9,5,3)								
**LiNbO3**	**(3,-8,-16)**	**12.08**	**86.72**	**39.385**	**35.1**	**0.693**	**24.35**	**8.67**	**25.84**
	Equiv. Refl.: (5,3,-16), (8,-3,-16), (-3,-5,-16), (-5,8,-16), (-8,5,-16)								
**LiNbO3**	**(0,-7,16)**	**12.08**	**86.72**	**36.15**	**33.9**	**0.693**	**23.49**	**8.67**	**25.03**
	Equiv. Refl.: (7,0,16)								
**LiNbO3**	**(-7,7,16)**	**12.08**	**86.72**	**36.149**	**33.9**	**0.693**	**23.49**	**8.67**	**25.03**
**LiNbO3**	**(0,7,-16)**	**12.08**	**86.72**	**34.969**	**33.9**	**0.693**	**23.49**	**8.67**	**25.03**
	Equiv. Refl.: (-7,0,-16)								
**LiNbO3**	**(7,-7,-16)**	**12.08**	**86.72**	**34.968**	**33.9**	**0.693**	**23.49**	**8.67**	**25.03**
**LiNbO3**	**(3,5,16)**	**12.08**	**86.72**	**34.774**	**35.1**	**0.693**	**24.35**	**8.67**	**25.84**
	Equiv. Refl.: (5,-8,16), (8,-5,16), (-5,-3,16)								
**LiNbO3**	**(-3,8,16)**	**12.08**	**86.72**	**34.773**	**35.1**	**0.693**	**24.35**	**8.67**	**25.84**
	Equiv. Refl.: (-8,3,16)								
**LiNbO3**	**(0,5,22)**	**12.047**	**84.61**	**34.223**	**30**	**1.141**	**34.26**	**14.26**	**37.11**
	Equiv. Refl.: (5,-5,22), (-5,0,22)								
**LiNbO3**	**(0,-5,-22)**	**12.047**	**84.61**	**32.668**	**30**	**1.141**	**34.26**	**14.26**	**37.11**
	Equiv. Refl.: (5,0,-22), (-5,5,-22)								
**LiNbO3**	**(5,5,0)**	**12.042**	**84.39**	**30.164**	**27.3**	**1.189**	**32.47**	**14.87**	**35.71**
	Equiv. Refl.: (5,-10,0), (10,-5,0), (-5,10,0), (-5,-5,0), (-10,5,0)								
**LiNbO3**	**(1,-9,4)**	**12.014**	**83.17**	**20.581**	**18.8**	**1.448**	**27.2**	**18.11**	**32.68**
	Equiv. Refl.: (8,1,4), (9,-1,4), (-1,-8,4), (-8,9,4), (-9,8,4)								
**LiNbO3**	**(1,8,-4)**	**12.014**	**83.17**	**19.773**	**18.8**	**1.448**	**27.2**	**18.11**	**32.68**
	Equiv. Refl.: (8,-9,-4), (9,-8,-4), (-1,9,-4), (-8,-1,-4), (-9,1,-4)								
**LiNbO3**	**(0,-2,26)**	**11.955**	**81.11**	**17.712**	**16.2**	**1.893**	**30.59**	**23.66**	**38.67**
	Equiv. Refl.: (2,0,26), (-2,2,26)								
**LiNbO3**	**(0,2,-26)**	**11.955**	**81.11**	**17.604**	**16.2**	**1.893**	**30.59**	**23.66**	**38.67**
	Equiv. Refl.: (2,-2,-26), (-2,0,-26)								
**LiNbO3**	**(0,8,10)**	**11.989**	**82.24**	**17.144**	**15.2**	**1.65**	**25.12**	**20.62**	**32.5**
	Equiv. Refl.: (8,-8,10), (-8,0,10)								
**LiNbO3**	**(0,-8,-10)**	**11.989**	**82.24**	**15.093**	**15.2**	**1.65**	**25.12**	**20.62**	**32.5**
	Equiv. Refl.: (8,0,-10), (-8,8,-10)								
**LiNbO3**	**(1,8,2)**	**11.914**	**79.94**	**14.622**	**13.4**	**2.147**	**28.81**	**26.83**	**39.37**
	Equiv. Refl.: (8,-9,2), (9,-8,2), (-1,9,2), (-8,-1,2), (-9,1,2)								
**LiNbO3**	**(1,-9,-2)**	**11.914**	**79.94**	**14.404**	**13.4**	**2.147**	**28.81**	**26.83**	**39.37**
	Equiv. Refl.: (8,1,-2), (9,-1,-2), (-1,-8,-2), (-8,9,-2), (-9,8,-2)								
**LiNbO3**	**(0,8,-8)**	**11.685**	**74.95**	**12.843**	**11.2**	**3.253**	**36.46**	**40.67**	**54.62**
	Equiv. Refl.: (-8,0,-8)								
**LiNbO3**	**(8,-8,-8)**	**11.685**	**74.95**	**12.842**	**11.2**	**3.253**	**36.46**	**40.67**	**54.62**
**LiNbO3**	**(2,5,18)**	**11.84**	**78.11**	**12.601**	**11.6**	**2.548**	**29.55**	**31.85**	**43.44**
	Equiv. Refl.: (5,-7,18), (7,-5,18), (-2,7,18), (-5,-2,18), (-7,2,18)								

Ei =	12.1	keV						ΔEg	ΔEt
**Cryst**	**Refl**	**EB**	**ΘB**	**∫ IR dΘ**	**Width**	**Ei cotΘ**	**ΔE**	**2m, 50µm**	**2m, 50µm**
	**(h,k,l)**	**[keV]**	**[°]**	**[µrad]**	**[µrad]**	**[meV/µrad]**	**[meV]**	**[meV]**	**[meV]**
**LiNbO3**	**(2,-9,8)**	**11.931**	**80.4**	**12.539**	**11.6**	**2.046**	**23.7**	**25.58**	**34.87**
	Equiv. Refl.: (7,2,8), (9,-2,8), (-2,-7,8), (-7,9,8), (-9,7,8)								
**LiNbO3**	**(2,-7,-18)**	**11.84**	**78.11**	**12.529**	**11.6**	**2.548**	**29.55**	**31.85**	**43.44**
	Equiv. Refl.: (5,2,-18), (7,-2,-18), (-2,-5,-18), (-5,7,-18), (-7,5,-18)								
**LiNbO3**	**(2,6,14)**	**11.821**	**77.67**	**12.506**	**11.3**	**2.645**	**29.94**	**33.06**	**44.61**
	Equiv. Refl.: (6,-8,14), (8,-6,14), (-2,8,14), (-6,-2,14), (-8,2,14)								
**LiNbO3**	**(2,2,24)**	**11.764**	**76.46**	**12.194**	**11.1**	**2.914**	**32.37**	**36.43**	**48.73**
	Equiv. Refl.: (2,-4,24), (4,-2,24), (-2,4,24), (-2,-2,24), (-4,2,24)								
**LiNbO3**	**(2,2,-24)**	**11.764**	**76.46**	**12.167**	**11.1**	**2.914**	**32.37**	**36.43**	**48.73**
	Equiv. Refl.: (2,-4,-24), (4,-2,-24), (-2,4,-24), (-2,-2,-24), (-4,2,-24)								
**LiNbO3**	**(2,-7,18)**	**11.84**	**78.11**	**12.163**	**11.2**	**2.548**	**28.57**	**31.85**	**42.78**
	Equiv. Refl.: (5,2,18), (7,-2,18), (-2,-5,18), (-5,7,18), (-7,5,18)								
**LiNbO3**	**(0,-8,8)**	**11.685**	**74.95**	**12.117**	**11.2**	**3.253**	**36.46**	**40.67**	**54.62**
	Equiv. Refl.: (8,0,8), (-8,8,8)								
**LiNbO3**	**(-6,8,-14)**	**11.821**	**77.67**	**12.065**	**11.3**	**2.645**	**29.94**	**33.06**	**44.61**
	Equiv. Refl.: (-8,6,-14)								
**LiNbO3**	**(2,-8,-14)**	**11.821**	**77.67**	**12.064**	**11.3**	**2.645**	**29.94**	**33.06**	**44.61**
	Equiv. Refl.: (6,2,-14), (8,-2,-14), (-2,-6,-14)								
**LiNbO3**	**(2,5,-18)**	**11.84**	**78.11**	**12.002**	**11.2**	**2.548**	**28.57**	**31.85**	**42.78**
	Equiv. Refl.: (5,-7,-18), (7,-5,-18), (-2,7,-18), (-5,-2,-18), (-7,2,-18)								
**LiNbO3**	**(2,7,-8)**	**11.931**	**80.4**	**11.941**	**11.6**	**2.046**	**23.7**	**25.58**	**34.87**
	Equiv. Refl.: (7,-9,-8), (9,-7,-8), (-2,9,-8), (-7,-2,-8), (-9,2,-8)								
**LiNbO3**	**(1,-8,12)**	**11.79**	**77**	**11.369**	**10.4**	**2.793**	**29.11**	**34.92**	**45.46**
	Equiv. Refl.: (7,1,12), (8,-1,12), (-1,-7,12)								
**LiNbO3**	**(-7,8,12)**	**11.79**	**77**	**11.368**	**10.4**	**2.793**	**29.11**	**34.92**	**45.46**
	Equiv. Refl.: (-8,7,12)								
**LiNbO3**	**(1,5,20)**	**11.829**	**77.85**	**11.154**	**10.2**	**2.605**	**26.6**	**32.56**	**42.05**
	Equiv. Refl.: (5,-6,20), (6,-5,20), (-1,6,20), (-5,-1,20), (-6,1,20)								
**LiNbO3**	**(1,7,-12)**	**11.79**	**77**	**11.133**	**10.4**	**2.793**	**29.11**	**34.92**	**45.46**
	Equiv. Refl.: (-1,8,-12), (-7,-1,-12), (-8,1,-12)								
**LiNbO3**	**(7,-8,-12)**	**11.79**	**77**	**11.132**	**10.4**	**2.793**	**29.11**	**34.92**	**45.46**
	Equiv. Refl.: (8,-7,-12)								
**Sapph**	**(3,6,0)**	**11.941**	**80.7**	**11.108**	**8.9**	**1.981**	**17.61**	**24.77**	**30.39**
	Equiv. Refl.: (3,-9,0), (6,3,0), (6,-9,0), (9,-3,0), (9,-6,0), (-3,9,0), (-3,-6,0), (-6,9,0), (-6,-3,0) (-9,3,0), (-9,6,0)								
**LiNbO3**	**(1,-6,-20)**	**11.829**	**77.85**	**11.092**	**10.2**	**2.605**	**26.6**	**32.56**	**42.05**
	Equiv. Refl.: (5,1,-20), (6,-1,-20), (-1,-5,-20), (-5,6,-20), (-6,5,-20)								
**LiNbO3**	**(4,5,-10)**	**11.745**	**76.08**	**10.836**	**9.5**	**2.999**	**28.54**	**37.49**	**47.11**
	Equiv. Refl.: (5,-9,-10), (9,-5,-10), (-4,9,-10), (-5,-4,-10), (-9,4,-10)								
**LiNbO3**	**(2,-5,22)**	**11.555**	**72.74**	**10.576**	**9.4**	**3.76**	**35.28**	**47**	**58.77**
	Equiv. Refl.: (3,2,22), (5,-2,22), (-2,-3,22), (-3,5,22), (-5,3,22)								
**LiNbO3**	**(0,-6,18)**	**11.593**	**73.35**	**10.444**	**9.4**	**3.618**	**34**	**45.23**	**56.58**
	Equiv. Refl.: (6,0,18), (-6,6,18)								
**LiNbO3**	**(1,7,12)**	**11.79**	**77**	**10.334**	**9.4**	**2.793**	**26.34**	**34.92**	**43.74**
	Equiv. Refl.: (7,-8,12), (8,-7,12), (-1,8,12), (-7,-1,12), (-8,1,12)								
**LiNbO3**	**(0,6,-18)**	**11.593**	**73.35**	**10.231**	**9.4**	**3.618**	**34**	**45.23**	**56.58**
	Equiv. Refl.: (6,-6,-18), (-6,0,-18)								
**LiNbO3**	**(2,3,-22)**	**11.555**	**72.74**	**10.162**	**9.4**	**3.76**	**35.28**	**47**	**58.77**
	Equiv. Refl.: (3,-5,-22), (5,-3,-22), (-2,5,-22), (-3,-2,-22), (-5,2,-22)								

Ei =	12.1	keV						ΔEg	ΔEt
Cryst	Refl	EB	ΘB	∫ IR dΘ	Width	Ei cotΘ	ΔE	2m, 50µm	2m, 50µm
	(h,k,l)	[keV]	[°]	[µrad]	[µrad]	[meV/µrad]	[meV]	[meV]	[meV]
**LiNbO3**	**(1,-8,-12)**	**11.79**	**77**	**10.141**	**9.4**	**2.793**	**26.34**	**34.92**	**43.74**
	Equiv. Refl.: (7,1,-12), (8,-1,-12), (-1,-7,-12), (-7,8,-12), (-8,7,-12)								
**LiNbO3**	**(4,-9,10)**	**11.745**	**76.08**	**10.127**	**9.5**	**2.999**	**28.54**	**37.49**	**47.11**
	Equiv. Refl.: (5,4,10), (9,-4,10), (-4,-5,10), (-5,9,10), (-9,5,10)								
**LiNbO3**	**(3,-8,14)**	**11.573**	**73.03**	**10.124**	**8.9**	**3.693**	**32.8**	**46.16**	**56.63**
	Equiv. Refl.: (5,3,14), (8,-3,14), (-3,-5,14), (-5,8,14), (-8,5,14)								
**LiNbO3**	**(2,-6,20)**	**11.581**	**73.16**	**9.83**	**8.7**	**3.662**	**31.75**	**45.78**	**55.71**
	Equiv. Refl.: (4,2,20), (6,-2,20), (-2,-4,20), (-4,6,20), (-6,4,20)								
**LiNbO3**	**(0,6,18)**	**11.593**	**73.35**	**9.809**	**8.6**	**3.618**	**31.17**	**45.23**	**54.93**
	Equiv. Refl.: (6,-6,18), (-6,0,18)								
**LiNbO3**	**(2,7,-2)**	**11.417**	**70.65**	**9.785**	**8.6**	**4.248**	**36.45**	**53.11**	**64.41**
	Equiv. Refl.: (-2,9,-2), (-7,-2,-2), (-9,2,-2)								
**LiNbO3**	**(7,-9,-2)**	**11.417**	**70.65**	**9.784**	**8.6**	**4.248**	**36.45**	**53.11**	**64.41**
	Equiv. Refl.: (9,-7,-2)								
**LiNbO3**	**(2,-9,2)**	**11.417**	**70.65**	**9.773**	**8.6**	**4.248**	**36.45**	**53.11**	**64.41**
	Equiv. Refl.: (7,2,2), (9,-2,2), (-2,-7,2), (-7,9,2), (-9,7,2)								
**LiNbO3**	**(1,6,16)**	**11.59**	**73.31**	**9.597**	**8.4**	**3.629**	**30.5**	**45.36**	**54.66**
	Equiv. Refl.: (6,-7,16), (7,-6,16), (-1,7,16), (-6,-1,16), (-7,1,16)								
**LiNbO3**	**(3,5,-14)**	**11.573**	**73.03**	**9.555**	**8.9**	**3.693**	**32.8**	**46.16**	**56.63**
	Equiv. Refl.: (5,-8,-14), (8,-5,-14), (-3,8,-14), (-5,-3,-14), (-8,3,-14)								
**LiNbO3**	**(2,4,-20)**	**11.581**	**73.16**	**9.547**	**8.7**	**3.662**	**31.75**	**45.78**	**55.71**
	Equiv. Refl.: (4,-6,-20), (6,-4,-20), (-2,6,-20), (-4,-2,-20), (-6,2,-20)								
**LiNbO3**	**(0,-6,-18)**	**11.593**	**73.35**	**9.391**	**8.6**	**3.618**	**31.17**	**45.23**	**54.93**
	Equiv. Refl.: (6,0,-18), (-6,6,-18)								
**LiNbO3**	**(0,1,26)**	**11.709**	**75.4**	**9.356**	**8.5**	**3.151**	**26.91**	**39.39**	**47.71**
	Equiv. Refl.: (1,-1,26), (-1,0,26)								
**LiNbO3**	**(-1,1,-26)**	**11.709**	**75.4**	**9.217**	**8.5**	**3.151**	**26.91**	**39.39**	**47.71**
**LiNbO3**	**(0,-1,-26)**	**11.709**	**75.4**	**9.216**	**8.5**	**3.151**	**26.91**	**39.39**	**47.71**
	Equiv. Refl.: (1,0,-26)								
**LiNbO3**	**(1,-7,-16)**	**11.59**	**73.31**	**9.06**	**8.4**	**3.629**	**30.5**	**45.36**	**54.66**
	Equiv. Refl.: (6,1,-16), (7,-1,-16), (-1,-6,-16), (-6,7,-16), (-7,6,-16)								
**LiNbO3**	**(0,-7,-14)**	**11.573**	**73.03**	**8.325**	**7.6**	**3.693**	**27.92**	**46.16**	**53.95**
	Equiv. Refl.: (7,0,-14), (-7,7,-14)								
**LiNbO3**	**(0,7,14)**	**11.573**	**73.03**	**8.159**	**7.6**	**3.693**	**27.92**	**46.16**	**53.95**
	Equiv. Refl.: (7,-7,14), (-7,0,14)								
**LiNbO3**	**(2,7,4)**	**11.521**	**72.21**	**7.946**	**7.1**	**3.883**	**27.55**	**48.54**	**55.81**
	Equiv. Refl.: (7,-9,4), (9,-7,4), (-2,9,4), (-7,-2,4), (-9,2,4)								
**Sapph**	**(0,7,-10)**	**11.562**	**72.85**	**7.864**	**6.2**	**3.734**	**22.99**	**46.67**	**52.03**
	Equiv. Refl.: (0,-7,10), (7,0,10), (7,-7,-10), (-7,0,-10), (-7,7,10)								
**Quartz**	**(-3,9,-3)**	**12.066**	**85.68**	**7.813**	**6.9**	**0.915**	**6.32**	**11.44**	**13.07**
	Equiv. Refl.: (-6,9,3), (-6,-3,-3)								
**Quartz**	**(-3,-6,3)**	**12.066**	**85.68**	**7.812**	**6.9**	**0.915**	**6.32**	**11.44**	**13.07**
**Quartz**	**(9,-3,3)**	**12.066**	**85.68**	**7.81**	**6.9**	**0.915**	**6.32**	**11.44**	**13.07**
	Equiv. Refl.: (9,-6,-3)								
**Sapph**	**(1,1,24)**	**11.748**	**76.14**	**7.602**	**6.1**	**2.986**	**18.19**	**37.32**	**41.52**
	Equiv. Refl.: (1,1,-24), (1,-2,24), (1,-2,-24), (2,-1,24), (2,-1,-24), (-1,2,24), (-1,2,-24), (-1,-1,24), (-1,-1,-24) (-2,1,24), (-2,1,-24)								
**Quartz**	**(6,-9,-3)**	**12.066**	**85.68**	**7.507**	**6.9**	**0.915**	**6.32**	**11.44**	**13.07**
**Quartz**	**(3,-9,3)**	**12.066**	**85.68**	**7.506**	**6.9**	**0.915**	**6.32**	**11.44**	**13.07**
	Equiv. Refl.: (6,3,3)								
**Quartz**	**(3,6,-3)**	**12.066**	**85.68**	**7.505**	**6.9**	**0.915**	**6.32**	**11.44**	**13.07**

**Ei =**	**12.1**	**keV**						**ΔEg**	**ΔEt**
**Cryst**	**Refl**	**EB**	**ΘB**	**∫ IR dΘ**	**Width**	**Ei cotΘ**	**ΔE**	**2m, 50µm**	**2m, 50µm**
	**(h,k,l)**	**[keV]**	**[°]**	**[µrad]**	**[µrad]**	**[meV/µrad]**	**[meV]**	**[meV]**	**[meV]**
**Quartz**	**(-9,3,-3)**	**12.066**	**85.68**	**7.504**	**6.9**	**0.915**	**6.32**	**11.44**	**13.07**
	Equiv. Refl.: (-9,6,3)								
**LiNbO3**	**(2,-9,-4)**	**11.521**	**72.21**	**7.453**	**7.1**	**3.883**	**27.55**	**48.54**	**55.81**
	Equiv. Refl.: (7,2,-4), (9,-2,-4), (-2,-7,-4), (-7,9,-4), (-9,7,-4)								
**Sapph**	**(4,5,-4)**	**11.904**	**79.67**	**7.296**	**6.1**	**2.205**	**13.4**	**27.56**	**30.65**
	Equiv. Refl.: (4,-9,4), (5,4,4), (5,-9,-4), (9,-4,4), (9,-5,-4), (-4,9,-4), (-4,-5,4), (-5,9,4), (-5,-4,-4) (-9,4,-4), (-9,5,4)								
**Quartz**	**(9,-3,-3)**	**12.066**	**85.68**	**7.08**	**6.2**	**0.915**	**5.71**	**11.44**	**12.78**
	Equiv. Refl.: (9,-6,3)								
**Quartz**	**(-6,9,-3)**	**12.066**	**85.68**	**7.079**	**6.2**	**0.915**	**5.71**	**11.44**	**12.78**
	Equiv. Refl.: (-6,-3,3)								
**Quartz**	**(-3,-6,-3)**	**12.066**	**85.68**	**7.078**	**6.2**	**0.915**	**5.71**	**11.44**	**12.78**
**Quartz**	**(-3,9,3)**	**12.066**	**85.68**	**7.076**	**6.2**	**0.915**	**5.71**	**11.44**	**12.78**
**LiNbO3**	**(4,5,8)**	**11.434**	**70.9**	**6.874**	**6.3**	**4.189**	**26.37**	**52.37**	**58.63**
	Equiv. Refl.: (5,-9,8), (9,-5,8), (-4,9,8), (-5,-4,8), (-9,4,8)								
**Quartz**	**(2,7,1)**	**11.981**	**81.96**	**6.831**	**5.5**	**1.71**	**9.46**	**21.37**	**23.37**
	Equiv. Refl.: (2,-9,-1)								
**Quartz**	**(7,2,-1)**	**11.981**	**81.96**	**6.83**	**5.5**	**1.71**	**9.46**	**21.37**	**23.37**
	Equiv. Refl.: (7,-9,1), (-9,2,1), (-9,7,-1)								
**Quartz**	**(4,3,7)**	**11.959**	**81.24**	**6.739**	**5.5**	**1.865**	**10.18**	**23.32**	**25.44**
	Equiv. Refl.: (4,-7,-7)								
**Quartz**	**(-7,3,-7)**	**11.959**	**81.24**	**6.734**	**5.5**	**1.865**	**10.17**	**23.32**	**25.44**
**Quartz**	**(3,4,-7)**	**11.959**	**81.24**	**6.733**	**5.5**	**1.865**	**10.17**	**23.32**	**25.44**
	Equiv. Refl.: (-7,4,7)								
**Quartz**	**(3,-7,7)**	**11.959**	**81.24**	**6.732**	**5.5**	**1.865**	**10.17**	**23.32**	**25.44**
**LiNbO3**	**(4,-9,-8)**	**11.434**	**70.9**	**6.622**	**6.3**	**4.189**	**26.37**	**52.37**	**58.63**
	Equiv. Refl.: (5,4,-8), (9,-4,-8), (-4,-5,-8), (-5,9,-8), (-9,5,-8)								
**Sapph**	**(2,6,11)**	**12.052**	**84.9**	**6.46**	**5.9**	**1.079**	**6.41**	**13.49**	**14.93**
	Equiv. Refl.: (2,-8,-11), (6,2,-11), (6,-8,11), (8,-2,-11), (8,-6,11), (-2,8,11), (-2,-6,-11), (-6,8,-11), (-6,-2,11) (-8,2,11), (-8,6,-11)								
**Quartz**	**(-4,7,7)**	**11.959**	**81.24**	**6.452**	**5.5**	**1.865**	**10.18**	**23.32**	**25.44**
	Equiv. Refl.: (-4,-3,-7)								
**Quartz**	**(7,-3,7)**	**11.959**	**81.24**	**6.447**	**5.5**	**1.865**	**10.17**	**23.32**	**25.44**
**Quartz**	**(7,-4,-7)**	**11.959**	**81.24**	**6.446**	**5.5**	**1.865**	**10.17**	**23.32**	**25.44**
	Equiv. Refl.: (-3,-4,7)								
**Quartz**	**(-3,7,-7)**	**11.959**	**81.24**	**6.445**	**5.5**	**1.865**	**10.17**	**23.32**	**25.44**
**Quartz**	**(-9,3,3)**	**12.066**	**85.68**	**6.439**	**6.2**	**0.915**	**5.71**	**11.44**	**12.78**
	Equiv. Refl.: (-9,6,-3)								
**Quartz**	**(3,6,3)**	**12.066**	**85.68**	**6.437**	**6.2**	**0.915**	**5.71**	**11.44**	**12.78**
	Equiv. Refl.: (6,3,-3), (6,-9,3)								
**Quartz**	**(3,-9,-3)**	**12.066**	**85.68**	**6.435**	**6.2**	**0.915**	**5.71**	**11.44**	**12.78**
**Quartz**	**(-2,9,1)**	**11.981**	**81.96**	**6.417**	**5.5**	**1.71**	**9.46**	**21.37**	**23.37**
	Equiv. Refl.: (-2,-7,-1)								
**Quartz**	**(9,-2,-1)**	**11.981**	**81.96**	**6.416**	**5.5**	**1.71**	**9.46**	**21.37**	**23.37**
	Equiv. Refl.: (9,-7,1), (-7,9,-1), (-7,-2,1)								
**LiNbO3**	**(0,-3,-24)**	**11.514**	**72.1**	**6.246**	**5.8**	**3.908**	**22.48**	**48.85**	**53.77**
	Equiv. Refl.: (3,0,-24), (-3,3,-24)								
**LiNbO3**	**(0,3,-24)**	**11.514**	**72.1**	**6.132**	**5.7**	**3.908**	**22.22**	**48.85**	**53.66**
	Equiv. Refl.: (-3,0,-24)								
**LiNbO3**	**(3,-3,-24)**	**11.514**	**72.1**	**6.131**	**5.7**	**3.908**	**22.22**	**48.85**	**53.66**

**Ei =**	**12.1**	**keV**						**ΔEg**	**ΔEt**
**Cryst**	**Refl**	**EB**	**ΘB**	**∫ IR dΘ**	**Width**	**Ei cotΘ**	**ΔE**	**2m, 50µm**	**2m, 50µm**
	**(h,k,l)**	**[keV]**	**[°]**	**[µrad]**	**[µrad]**	**[meV/µrad]**	**[meV]**	**[meV]**	**[meV]**
**LiNbO3**	**(0,3,24)**	**11.514**	**72.1**	**6.079**	**5.8**	**3.908**	**22.48**	**48.85**	**53.77**
	Equiv. Refl.: (3,-3,24), (-3,0,24)								
**LiNbO3**	**(0,-3,24)**	**11.514**	**72.1**	**6.024**	**5.7**	**3.908**	**22.22**	**48.85**	**53.66**
	Equiv. Refl.: (3,0,24), (-3,3,24)								
**Quartz**	**(0,7,-5)**	**11.701**	**75.24**	**5.907**	**4.6**	**3.188**	**14.81**	**39.84**	**42.51**
	Equiv. Refl.: (0,-7,5)								
**Quartz**	**(7,0,5)**	**11.701**	**75.24**	**5.903**	**4.6**	**3.188**	**14.8**	**39.84**	**42.5**
	Equiv. Refl.: (7,-7,-5), (-7,0,-5), (-7,7,5)								
**Sapph**	**(3,3,18)**	**11.615**	**73.73**	**5.674**	**4.7**	**3.531**	**16.48**	**44.14**	**47.12**
	Equiv. Refl.: (3,3,-18), (3,-6,18), (3,-6,-18), (6,-3,18), (6,-3,-18), (-3,6,18), (-3,6,-18), (-3,-3,18), (-3,-3,-18) (-6,3,18), (-6,3,-18)								
**Quartz**	**(2,6,5)**	**11.97**	**81.59**	**5.349**	**4.3**	**1.789**	**7.75**	**22.36**	**23.67**
	Equiv. Refl.: (2,-8,-5)								
**Quartz**	**(-8,2,5)**	**11.97**	**81.59**	**5.347**	**4.3**	**1.789**	**7.75**	**22.36**	**23.67**
	Equiv. Refl.: (-8,6,-5)								
**Quartz**	**(6,2,-5)**	**11.97**	**81.59**	**5.346**	**4.3**	**1.789**	**7.75**	**22.36**	**23.67**
**Quartz**	**(6,-8,5)**	**11.97**	**81.59**	**5.345**	**4.3**	**1.789**	**7.75**	**22.36**	**23.67**
**Quartz**	**(0,6,7)**	**11.87**	**78.8**	**5.276**	**4.2**	**2.396**	**10.13**	**29.94**	**31.61**
	Equiv. Refl.: (0,-6,-7)								
**Quartz**	**(6,0,-7)**	**11.87**	**78.8**	**5.268**	**4.2**	**2.396**	**10.12**	**29.94**	**31.61**
	Equiv. Refl.: (6,-6,7), (-6,0,7), (-6,6,-7)								
**Quartz**	**(-2,8,5)**	**11.97**	**81.59**	**5.1**	**4.3**	**1.789**	**7.75**	**22.36**	**23.67**
	Equiv. Refl.: (-2,-6,-5)								
**Quartz**	**(8,-2,-5)**	**11.97**	**81.59**	**5.098**	**4.3**	**1.789**	**7.75**	**22.36**	**23.67**
	Equiv. Refl.: (8,-6,5), (-6,-2,5)								
**Quartz**	**(-6,8,-5)**	**11.97**	**81.59**	**5.097**	**4.3**	**1.789**	**7.75**	**22.36**	**23.67**
**Sapph**	**(1,7,6)**	**11.714**	**75.48**	**5.027**	**4.1**	**3.134**	**12.72**	**39.17**	**41.18**
	Equiv. Refl.: (1,7,-6), (1,-8,6), (1,-8,-6), (7,1,6), (7,1,-6), (7,-8,6), (7,-8,-6), (8,-1,6), (8,-1,-6) (8,-7,6), (8,-7,-6), (-1,8,6), (-1,8,-6), (-1,-7,6), (-1,-7,-6), (-7,8,6), (-7,8,-6), (-7,-1,6) (-7,-1,-6), (-8,1,6), (-8,1,-6), (-8,7,6), (-8,7,-6)								
**Quartz**	**(-8,1,-4)**	**11.918**	**80.06**	**5.019**	**4**	**2.12**	**8.57**	**26.5**	**27.85**
**Quartz**	**(1,7,-4)**	**11.918**	**80.06**	**5.018**	**4**	**2.12**	**8.57**	**26.5**	**27.85**
	Equiv. Refl.: (1,-8,4), (7,-8,-4), (-8,7,4)								
**Quartz**	**(7,1,4)**	**11.918**	**80.06**	**5.017**	**4**	**2.12**	**8.57**	**26.5**	**27.85**
**Quartz**	**(2,7,-1)**	**11.981**	**81.96**	**4.923**	**4.2**	**1.71**	**7.11**	**21.37**	**22.52**
	Equiv. Refl.: (2,-9,1), (7,2,1), (7,-9,-1)								
**Quartz**	**(-9,2,-1)**	**11.981**	**81.96**	**4.922**	**4.2**	**1.71**	**7.11**	**21.37**	**22.52**
	Equiv. Refl.: (-9,7,1)								
**Quartz**	**(-2,9,-1)**	**11.981**	**81.96**	**4.87**	**4.2**	**1.71**	**7.11**	**21.37**	**22.52**
	Equiv. Refl.: (-2,-7,1), (-7,9,1), (-7,-2,-1)								
**Quartz**	**(9,-2,1)**	**11.981**	**81.96**	**4.869**	**4.2**	**1.71**	**7.11**	**21.37**	**22.52**
	Equiv. Refl.: (9,-7,-1)								
**Quartz**	**(8,-1,4)**	**11.918**	**80.06**	**4.82**	**4**	**2.12**	**8.57**	**26.5**	**27.85**
	Equiv. Refl.: (-1,8,-4), (-1,-7,4)								
**Quartz**	**(8,-7,-4)**	**11.918**	**80.06**	**4.819**	**4**	**2.12**	**8.57**	**26.5**	**27.85**
	Equiv. Refl.: (-7,8,4), (-7,-1,-4)								
**Sapph**	**(1,6,-14)**	**11.915**	**79.97**	**4.523**	**3.9**	**2.14**	**8.36**	**26.74**	**28.02**
	Equiv. Refl.: (1,-7,14), (6,1,14), (6,-7,-14), (7,-1,14), (7,-6,-14), (-1,7,-14), (-1,-6,14), (-6,7,14), (-6,-1,-14) (-7,1,-14), (-7,6,14)								

**Ei =**	**12.1**	**keV**						**ΔEg**	**ΔEt**
**Cryst**	**Refl**	**EB**	**ΘB**	**∫ IR dΘ**	**Width**	**Ei cotΘ**	**ΔE**	**2m, 50µm**	**2m, 50µm**
	**(h,k,l)**	**[keV]**	**[°]**	**[µrad]**	**[µrad]**	**[meV/µrad]**	**[meV]**	**[meV]**	**[meV]**
**Sapph**	**(2,4,19)**	**12.067**	**85.76**	**4.502**	**4.7**	**0.897**	**4.2**	**11.21**	**11.97**
	Equiv. Refl.: (2,-6,-19), (4,2,-19), (4,-6,19), (6,-2,-19), (6,-4,19), (-2,6,19), (-2,-4,-19), (-4,6,-19), (-4,-2,19) (-6,2,19), (-6,4,-19)								
**Sapph**	**(4,5,5)**	**11.99**	**82.26**	**4.272**	**3.9**	**1.644**	**6.46**	**20.55**	**21.55**
	Equiv. Refl.: (4,-9,-5), (5,4,-5), (5,-9,5), (9,-4,-5), (9,-5,5), (-4,9,5), (-4,-5,-5), (-5,9,-5), (-5,-4,5) (-9,4,5), (-9,5,-5)								
**Quartz**	**(4,-1,-9)**	**11.583**	**73.19**	**4.149**	**3.2**	**3.656**	**11.84**	**45.7**	**47.2**
**Quartz**	**(4,-3,9)**	**11.583**	**73.19**	**4.147**	**3.2**	**3.656**	**11.84**	**45.7**	**47.2**
	Equiv. Refl.: (9,-3,2), (9,-6,-2), (-1,4,9), (-3,9,-2), (-3,-6,2), (-6,9,2), (-6,-3,-2)								
**Quartz**	**(-1,-3,-9)**	**11.583**	**73.19**	**4.146**	**3.2**	**3.656**	**11.83**	**45.7**	**47.2**
	Equiv. Refl.: (-3,4,-9), (-3,-1,9)								
**Quartz**	**(3,6,-2)**	**11.79**	**77**	**4.117**	**3.4**	**2.794**	**9.37**	**34.92**	**36.16**
	Equiv. Refl.: (6,-9,-2), (-9,6,2)								
**Quartz**	**(3,-9,2)**	**11.79**	**77**	**4.116**	**3.4**	**2.794**	**9.37**	**34.92**	**36.16**
	Equiv. Refl.: (6,3,2), (-9,3,-2)								
**Quartz**	**(0,8,-1)**	**11.712**	**75.45**	**4.097**	**3.2**	**3.139**	**10.2**	**39.24**	**40.55**
	Equiv. Refl.: (0,-8,1), (8,0,1), (8,-8,-1), (-8,0,-1), (-8,8,1)								
**Quartz**	**(-4,1,9)**	**11.583**	**73.19**	**4.086**	**3.2**	**3.656**	**11.84**	**45.7**	**47.2**
**Quartz**	**(-4,3,-9)**	**11.583**	**73.19**	**4.085**	**3.2**	**3.656**	**11.84**	**45.7**	**47.2**
**Quartz**	**(1,3,9)**	**11.583**	**73.19**	**4.084**	**3.2**	**3.656**	**11.83**	**45.7**	**47.2**
	Equiv. Refl.: (1,-4,-9), (3,1,-9), (3,-4,9)								
**Quartz**	**(0,1,-10)**	**11.562**	**72.86**	**3.995**	**3.1**	**3.733**	**11.76**	**46.66**	**48.12**
	Equiv. Refl.: (0,-1,10)								
**Quartz**	**(1,0,10)**	**11.562**	**72.86**	**3.993**	**3.1**	**3.733**	**11.75**	**46.66**	**48.12**
	Equiv. Refl.: (-1,0,-10)								
**Quartz**	**(1,-1,-10)**	**11.562**	**72.86**	**3.992**	**3.1**	**3.733**	**11.75**	**46.66**	**48.11**
	Equiv. Refl.: (-1,1,10)								
**Quartz**	**(0,1,10)**	**11.562**	**72.86**	**3.882**	**3.1**	**3.733**	**11.41**	**46.66**	**48.03**
	Equiv. Refl.: (0,-1,-10)								
**Quartz**	**(1,0,-10)**	**11.562**	**72.86**	**3.88**	**3.1**	**3.733**	**11.4**	**46.66**	**48.03**
**Quartz**	**(1,-1,10)**	**11.562**	**72.86**	**3.879**	**3.1**	**3.733**	**11.4**	**46.66**	**48.03**
	Equiv. Refl.: (-1,0,10)								
**Quartz**	**(-1,1,-10)**	**11.562**	**72.86**	**3.878**	**3.1**	**3.733**	**11.4**	**46.66**	**48.03**
**Quartz**	**(-3,4,9)**	**11.583**	**73.19**	**3.748**	**3**	**3.656**	**10.87**	**45.7**	**46.97**
	Equiv. Refl.: (-3,-1,-9)								
**Quartz**	**(4,-3,-9)**	**11.583**	**73.19**	**3.747**	**3**	**3.656**	**10.87**	**45.7**	**46.97**
	Equiv. Refl.: (-1,-3,9)								
**Quartz**	**(4,-1,9)**	**11.583**	**73.19**	**3.746**	**3**	**3.656**	**10.87**	**45.7**	**46.97**
	Equiv. Refl.: (-1,4,-9)								
**Quartz**	**(3,1,9)**	**11.583**	**73.19**	**3.724**	**3**	**3.656**	**10.87**	**45.7**	**46.97**
	Equiv. Refl.: (3,-4,-9)								
**Quartz**	**(6,3,-2)**	**11.79**	**77**	**3.723**	**3.1**	**2.794**	**8.53**	**34.92**	**35.95**
	Equiv. Refl.: (6,-9,2), (-9,3,2), (-9,6,-2)								
**Quartz**	**(1,3,-9)**	**11.583**	**73.19**	**3.722**	**3**	**3.656**	**10.87**	**45.7**	**46.97**
	Equiv. Refl.: (1,-4,9), (3,-9,-2), (-4,3,9)								
**Quartz**	**(3,6,2)**	**11.79**	**77**	**3.721**	**3.1**	**2.794**	**8.53**	**34.92**	**35.95**
	Equiv. Refl.: (-3,7,7), (-3,-4,-7), (-4,1,-9)								
**Quartz**	**(-4,7,-7)**	**11.959**	**81.24**	**3.72**	**3.2**	**1.865**	**6**	**23.32**	**24.08**
	Equiv. Refl.: (-4,-3,7)								
**Quartz**	**(7,-3,-7)**	**11.959**	**81.24**	**3.719**	**3.2**	**1.865**	**6**	**23.32**	**24.08**
**Quartz**	**(7,-4,7)**	**11.959**	**81.24**	**3.718**	**3.2**	**1.865**	**6**	**23.32**	**24.08**

Ei =	12.1	keV						ΔEg	ΔEt
**Cryst**	**Refl**	**EB**	**ΘB**	**∫ IR dΘ**	**Width**	**Ei cotΘ**	**ΔE**	**2m, 50µm**	**2m, 50µm**
	**(h,k,l)**	**[keV]**	**[°]**	**[µrad]**	**[µrad]**	**[meV/µrad]**	**[meV]**	**[meV]**	**[meV]**
**Quartz**	**(3,-6,-8)**	**11.896**	**79.47**	**3.717**	**3.1**	**2.25**	**6.93**	**28.12**	**28.96**
**Quartz**	**(3,3,8)**	**11.896**	**79.47**	**3.716**	**3.1**	**2.25**	**6.93**	**28.12**	**28.96**
**Quartz**	**(2,4,-8)**	**11.985**	**82.09**	**3.714**	**3.2**	**1.68**	**5.39**	**21**	**21.68**
	Equiv. Refl.: (-6,3,-8)								
**Quartz**	**(-6,3,8)**	**11.896**	**79.47**	**3.713**	**3.1**	**2.25**	**6.93**	**28.12**	**28.96**
**Quartz**	**(9,-3,-2)**	**11.79**	**77**	**3.712**	**3.1**	**2.794**	**8.53**	**34.92**	**35.95**
	Equiv. Refl.: (9,-6,2), (-6,2,-8), (-6,4,8), (-6,9,-2), (-6,-3,2)								
**Quartz**	**(2,-6,8)**	**11.985**	**82.09**	**3.711**	**3.2**	**1.68**	**5.39**	**21**	**21.68**
	Equiv. Refl.: (3,3,-8), (3,-6,8), (-3,9,2), (-3,-6,-2)								
**Quartz**	**(4,2,8)**	**11.985**	**82.09**	**3.708**	**3.2**	**1.68**	**5.38**	**21**	**21.68**
	Equiv. Refl.: (4,-6,-8)								
**Quartz**	**(3,4,7)**	**11.959**	**81.24**	**3.591**	**3.2**	**1.865**	**6**	**23.32**	**24.08**
	Equiv. Refl.: (3,-7,-7)								
**Quartz**	**(4,3,-7)**	**11.959**	**81.24**	**3.59**	**3.2**	**1.865**	**6**	**23.32**	**24.08**
**Quartz**	**(4,-7,7)**	**11.959**	**81.24**	**3.589**	**3.2**	**1.865**	**6**	**23.32**	**24.08**
	Equiv. Refl.: (-2,-4,8), (-7,3,7)								
**Quartz**	**(6,-2,8)**	**11.985**	**82.09**	**3.587**	**3.2**	**1.68**	**5.39**	**21**	**21.68**
	Equiv. Refl.: (6,-4,-8), (-2,6,-8), (-7,4,-7)								
**Quartz**	**(-4,6,8)**	**11.985**	**82.09**	**3.583**	**3.2**	**1.68**	**5.38**	**21**	**21.68**
	Equiv. Refl.: (-4,-2,-8)								
**Quartz**	**(7,1,-4)**	**11.918**	**80.06**	**3.548**	**3**	**2.12**	**6.41**	**26.5**	**27.26**
	Equiv. Refl.: (7,-8,4)								
**Quartz**	**(1,7,4)**	**11.918**	**80.06**	**3.546**	**3**	**2.12**	**6.4**	**26.5**	**27.26**
	Equiv. Refl.: (1,-8,-4)								
**Quartz**	**(-8,1,4)**	**11.918**	**80.06**	**3.545**	**3**	**2.12**	**6.4**	**26.5**	**27.26**
	Equiv. Refl.: (-8,7,-4)								
**Quartz**	**(-3,6,8)**	**11.896**	**79.47**	**3.485**	**3.1**	**2.25**	**6.93**	**28.12**	**28.96**
	Equiv. Refl.: (-3,-3,-8)								
**Quartz**	**(6,-3,8)**	**11.896**	**79.47**	**3.483**	**3.1**	**2.25**	**6.93**	**28.12**	**28.96**
**Quartz**	**(6,-3,-8)**	**11.896**	**79.47**	**3.482**	**3.1**	**2.25**	**6.93**	**28.12**	**28.96**
**Quartz**	**(-3,-3,8)**	**11.896**	**79.47**	**3.48**	**3.1**	**2.25**	**6.92**	**28.12**	**28.96**
**Quartz**	**(-3,6,-8)**	**11.896**	**79.47**	**3.479**	**3.1**	**2.25**	**6.92**	**28.12**	**28.96**
**Sapph**	**(2,6,-10)**	**11.852**	**78.38**	**3.441**	**3.3**	**2.488**	**8.11**	**31.1**	**32.14**
	Equiv. Refl.: (2,-8,10), (6,2,10), (6,-8,-10), (8,-2,10), (8,-6,-10), (-2,8,-10), (-2,-6,10), (-6,8,10), (-6,-2,-10) (-8,2,-10), (-8,6,10)								
**Quartz**	**(-7,-1,4)**	**11.918**	**80.06**	**3.407**	**3**	**2.12**	**6.41**	**26.5**	**27.26**
**Quartz**	**(-7,8,-4)**	**11.918**	**80.06**	**3.406**	**3**	**2.12**	**6.41**	**26.5**	**27.26**
**Quartz**	**(-1,8,4)**	**11.918**	**80.06**	**3.405**	**3**	**2.12**	**6.4**	**26.5**	**27.26**
	Equiv. Refl.: (-1,-7,-4)								
**Quartz**	**(8,-1,-4)**	**11.918**	**80.06**	**3.404**	**3**	**2.12**	**6.4**	**26.5**	**27.26**
	Equiv. Refl.: (8,-7,4)								
**Quartz**	**(4,0,9)**	**11.855**	**78.44**	**3.273**	**3**	**2.474**	**7.46**	**30.93**	**31.82**
	Equiv. Refl.: (4,-4,-9), (-4,0,-9)								
**Quartz**	**(-4,4,9)**	**11.855**	**78.44**	**3.272**	**3**	**2.474**	**7.46**	**30.93**	**31.82**
**Quartz**	**(0,4,-9)**	**11.855**	**78.44**	**3.268**	**3**	**2.474**	**7.45**	**30.93**	**31.81**
	Equiv. Refl.: (0,-4,9)								
**Quartz**	**(-1,7,-6)**	**11.775**	**76.68**	**3.157**	**2.5**	**2.864**	**7.05**	**35.8**	**36.48**
	Equiv. Refl.: (-6,7,6), (-6,-1,-6)								
**Quartz**	**(-1,-6,6)**	**11.775**	**76.68**	**3.156**	**2.5**	**2.864**	**7.04**	**35.8**	**36.48**

**Ei =**	**12.1**	**keV**						**ΔEg**	**ΔEt**
**Cryst**	**Refl**	**EB**	**ΘB**	**∫ IR dΘ**	**Width**	**Ei cotΘ**	**ΔE**	**2m, 50µm**	**2m, 50µm**
	**(h,k,l)**	**[keV]**	**[°]**	**[µrad]**	**[µrad]**	**[meV/µrad]**	**[meV]**	**[meV]**	**[meV]**
**Quartz**	**(7,-1,6)**	**11.775**	**76.68**	**3.153**	**2.5**	**2.864**	**7.04**	**35.8**	**36.48**
	Equiv. Refl.: (7,-6,-6)								
**Quartz**	**(-5,9,-1)**	**11.437**	**70.95**	**3.093**	**2.7**	**4.179**	**11.3**	**52.24**	**53.45**
	Equiv. Refl.: (-5,-4,1)								
**Quartz**	**(9,-4,-1)**	**11.437**	**70.95**	**3.092**	**2.7**	**4.179**	**11.3**	**52.24**	**53.45**
	Equiv. Refl.: (9,-5,1), (-4,9,1), (-4,-5,-1)								
**Quartz**	**(6,-1,7)**	**11.414**	**70.61**	**3.065**	**2.4**	**4.258**	**10.01**	**53.23**	**54.16**
	Equiv. Refl.: (6,-5,-7), (-1,6,-7), (-1,-5,7)								
**Quartz**	**(-5,6,7)**	**11.414**	**70.61**	**3.062**	**2.3**	**4.258**	**10**	**53.23**	**54.16**
	Equiv. Refl.: (-5,-1,-7)								
**Quartz**	**(4,-2,9)**	**11.491**	**71.74**	**3.061**	**2.7**	**3.992**	**10.66**	**49.89**	**51.02**
	Equiv. Refl.: (4,-2,-9)								
**Quartz**	**(-2,4,-9)**	**11.491**	**71.74**	**3.06**	**2.7**	**3.992**	**10.65**	**49.89**	**51.02**
	Equiv. Refl.: (-2,-2,9)								
**Quartz**	**(-2,4,9)**	**11.491**	**71.74**	**3.059**	**2.7**	**3.992**	**10.65**	**49.89**	**51.02**
	Equiv. Refl.: (-2,-2,-9)								
**Quartz**	**(1,-6,7)**	**11.414**	**70.61**	**3.049**	**2.4**	**4.258**	**10.01**	**53.23**	**54.16**
**Quartz**	**(1,5,-7)**	**11.414**	**70.61**	**3.048**	**2.4**	**4.258**	**10.01**	**53.23**	**54.16**
	Equiv. Refl.: (-6,1,-7), (-6,5,7)								
**Quartz**	**(5,1,7)**	**11.414**	**70.61**	**3.045**	**2.3**	**4.258**	**10**	**53.23**	**54.16**
	Equiv. Refl.: (5,-6,-7)								
**Quartz**	**(4,5,1)**	**11.437**	**70.95**	**3.024**	**2.7**	**4.179**	**11.3**	**52.24**	**53.45**
	Equiv. Refl.: (4,-9,-1), (5,4,-1), (5,-9,1), (-9,4,1), (-9,5,-1)								
**Quartz**	**(5,4,2)**	**11.608**	**73.61**	**3.013**	**2.7**	**3.559**	**9.49**	**44.48**	**45.49**
	Equiv. Refl.: (5,-9,-2)								
**Quartz**	**(4,5,-2)**	**11.608**	**73.61**	**3.012**	**2.7**	**3.559**	**9.49**	**44.48**	**45.49**
	Equiv. Refl.: (4,-9,2), (-9,4,-2), (-9,5,2)								
**Quartz**	**(-4,2,9)**	**11.491**	**71.74**	**2.948**	**2.7**	**3.992**	**10.66**	**49.89**	**51.02**
	Equiv. Refl.: (-4,2,-9)								
**Quartz**	**(2,2,-9)**	**11.491**	**71.74**	**2.947**	**2.7**	**3.992**	**10.65**	**49.89**	**51.02**
	Equiv. Refl.: (2,-4,9)								
**Quartz**	**(2,2,9)**	**11.491**	**71.74**	**2.946**	**2.7**	**3.992**	**10.65**	**49.89**	**51.02**
**Quartz**	**(2,-4,-9)**	**11.491**	**71.74**	**2.945**	**2.7**	**3.992**	**10.65**	**49.89**	**51.02**
**Quartz**	**(-5,9,2)**	**11.608**	**73.61**	**2.927**	**2.7**	**3.559**	**9.49**	**44.48**	**45.49**
	Equiv. Refl.: (-5,-4,-2)								
**Quartz**	**(9,-4,2)**	**11.608**	**73.61**	**2.926**	**2.7**	**3.559**	**9.49**	**44.48**	**45.49**
	Equiv. Refl.: (9,-5,-2), (-4,9,-2), (-4,-5,2)								
**Quartz**	**(6,1,6)**	**11.775**	**76.68**	**2.922**	**2.5**	**2.864**	**7.05**	**35.8**	**36.48**
	Equiv. Refl.: (6,-7,-6)								
**Quartz**	**(1,-7,6)**	**11.775**	**76.68**	**2.921**	**2.5**	**2.864**	**7.05**	**35.8**	**36.48**
**Quartz**	**(1,6,-6)**	**11.775**	**76.68**	**2.92**	**2.5**	**2.864**	**7.04**	**35.8**	**36.48**
**Quartz**	**(-7,1,-6)**	**11.775**	**76.68**	**2.918**	**2.5**	**2.864**	**7.04**	**35.8**	**36.48**
	Equiv. Refl.: (-7,6,6)								
**Quartz**	**(1,6,6)**	**11.775**	**76.68**	**2.909**	**2.3**	**2.864**	**6.52**	**35.8**	**36.39**
	Equiv. Refl.: (1,-7,-6), (-7,1,6), (-7,6,-6)								
**Quartz**	**(6,1,-6)**	**11.775**	**76.68**	**2.905**	**2.3**	**2.864**	**6.52**	**35.8**	**36.38**
	Equiv. Refl.: (6,-7,6)								
**Sapph**	**(2,3,20)**	**11.581**	**73.16**	**2.872**	**2.7**	**3.662**	**9.78**	**45.77**	**46.81**
	Equiv. Refl.: (2,-5,-20), (3,2,-20), (3,-5,20), (5,-2,-20), (5,-3,20), (-2,5,20), (-2,-3,-20), (-3,5,-20), (-3,-2,20) (-5,2,20), (-5,3,-20)								

Ei =	12.1	keV						ΔEg	ΔEt
Cryst	Refl	EB	ΘB	∫ IR dΘ	Width	Ei cotΘ	ΔE	2m, 50μm	2m, 50μm
	(h,k,l)	[keV]	[°]	[μrad]	[μrad]	[meV/μrad]	[meV]	[meV]	[meV]
Quartz	(1,7,-3)	11.526	72.28	2.828	2.1	3.867	8.3	48.34	49.05
	Equiv. Refl.: (7,1,3), (7,-8,-3), (-8,1,-3), (-8,7,3)								
Quartz	(1,-8,3)	11.526	72.28	2.827	2.1	3.867	8.3	48.34	49.05
Sapph	(3,5,10)	11.562	72.85	2.713	2.5	3.734	9.45	46.67	47.62
	Equiv. Refl.: (3,-8,-10), (5,3,-10), (5,-8,10), (8,-3,-10), (8,-5,10), (-3,8,10), (-3,-5,-10), (-5,8,-10), (-5,-3,10) (-8,3,10), (-8,5,-10)								
Quartz	(6,2,4)	11.465	71.35	2.652	2.4	4.084	9.61	51.05	51.95
	Equiv. Refl.: (6,-8,-4)								
Quartz	(-8,2,-4)	11.465	71.35	2.651	2.4	4.084	9.61	51.05	51.95
	Equiv. Refl.: (-8,6,4)								
Quartz	(2,6,-4)	11.465	71.35	2.65	2.4	4.084	9.6	51.05	51.95
	Equiv. Refl.: (2,-8,4)								
Quartz	(8,-1,-3)	11.526	72.28	2.57	2.3	3.867	8.87	48.34	49.15
	Equiv. Refl.: (-1,8,3), (-1,-7,-3), (-7,8,-3), (-7,-1,3)								
Quartz	(8,-7,3)	11.526	72.28	2.569	2.3	3.867	8.87	48.34	49.15
Quartz	(8,-2,4)	11.465	71.35	2.552	2.4	4.084	9.61	51.05	51.95
	Equiv. Refl.: (8,-6,-4), (-6,8,4), (-6,-2,-4)								
Quartz	(-2,8,-4)	11.465	71.35	2.551	2.4	4.084	9.6	51.05	51.95
	Equiv. Refl.: (-2,-6,4)								
Quartz	(1,7,3)	11.526	72.28	2.479	2.3	3.867	8.87	48.34	49.15
	Equiv. Refl.: (1,-8,-3), (7,1,-3), (7,-8,3)								
Quartz	(-8,1,3)	11.526	72.28	2.478	2.3	3.867	8.87	48.34	49.15
	Equiv. Refl.: (-8,7,-3)								
Sapph	(4,5,-1)	11.76	76.38	2.431	2.4	2.932	6.93	36.66	37.3
	Equiv. Refl.: (4,-9,1), (5,4,1), (5,-9,-1), (9,-4,1), (9,-5,-1), (-4,9,-1), (-4,-5,1), (-5,9,1), (-5,-4,-1) (-9,4,-1), (-9,5,1)								
Quartz	(3,6,0)	11.564	72.89	2.412	2.1	3.725	7.75	46.56	47.2
	Equiv. Refl.: (3,-9,0), (6,3,0), (6,-9,0), (-9,3,0), (-9,6,0)								
Quartz	(7,-1,-6)	11.775	76.68	2.382	2.3	2.864	6.52	35.8	36.39
	Equiv. Refl.: (7,-6,6), (-1,7,6), (-1,-6,-6)								
Quartz	(-6,-1,6)	11.775	76.68	2.38	2.3	2.864	6.52	35.8	36.38
Quartz	(-6,7,-6)	11.775	76.68	2.379	2.3	2.864	6.51	35.8	36.38
LiNbO3	(1,6,-17)	11.871	78.84	2.375	3.6	2.386	8.61	29.83	31.05
	Equiv. Refl.: (1,-7,17), (6,1,17), (6,-7,-17), (7,-1,17), (7,-6,-17), (-1,7,-17), (-1,-6,17), (-6,7,17), (-6,-1,-17) (-7,1,-17), (-7,6,17)								
Quartz	(8,-1,3)	11.526	72.28	2.371	2.1	3.867	8.3	48.34	49.05
	Equiv. Refl.: (-1,-7,3), (-7,8,3)								
Quartz	(8,-7,-3)	11.526	72.28	2.37	2.1	3.867	8.3	48.34	49.05
	Equiv. Refl.: (-1,8,-3), (-7,-1,-3)								
Sapph	(2,5,15)	11.812	77.48	2.323	2.3	2.688	6.21	33.6	34.17
	Equiv. Refl.: (2,5,-15), (2,-7,15), (2,-7,-15), (5,2,15), (5,2,-15), (5,-7,15), (5,-7,-15), (7,-2,15), (7,-2,-15) (7,-5,15), (7,-5,-15), (-2,7,15), (-2,7,-15), (-2,-5,15), (-2,-5,-15), (-5,7,15), (-5,7,-15), (-5,-2,15) (-5,-2,-15), (-7,2,15), (-7,2,-15), (-7,5,15), (-7,5,-15)								
Quartz	(0,-4,-9)	11.855	78.44	1.99	2.2	2.474	5.43	30.93	31.4
Quartz	(0,4,9)	11.855	78.44	1.989	2.2	2.474	5.43	30.93	31.4
Quartz	(-4,4,-9)	11.855	78.44	1.985	2.2	2.474	5.42	30.93	31.4
Quartz	(4,0,-9)	11.855	78.44	1.984	2.2	2.474	5.41	30.93	31.4
	Equiv. Refl.: (4,-4,9), (-4,0,9)								
Quartz	(0,8,0)	11.656	74.43	1.934	2.1	3.372	6.99	42.15	42.72
	Equiv. Refl.: (0,-8,0), (8,0,0), (8,-8,0), (-8,0,0), (-8,8,0)								

Ei =	12.1	keV						ΔEg	ΔEt
**Cryst**	**Refl**	**EB**	**ΘB**	**∫ IR dΘ**	**Width**	**Ei cotΘ**	**ΔE**	**2m, 50μm**	**2m, 50μm**
	**(h,k,l)**	**[keV]**	**[°]**	**[μrad]**	**[μrad]**	**[meV/μrad]**	**[meV]**	**[meV]**	**[meV]**
**LiNbO3**	**(2,7,7)**	**11.804**	**77.31**	**1.909**	**3**	**2.726**	**8.15**	**34.07**	**35.03**
	Equiv. Refl.: (2,-9,-7), (7,2,-7), (7,-9,7), (9,-2,-7), (9,-7,7), (-2,9,7), (-2,-7,-7), (-7,9,-7), (-7,-2,7) (-9,2,7), (-9,7,-7)								
**Quartz**	**(9,-3,0)**	**11.564**	**72.89**	**1.888**	**2.1**	**3.725**	**7.75**	**46.56**	**47.2**
	Equiv. Refl.: (9,-6,0), (-3,9,0), (-3,-6,0), (-6,9,0), (-6,-3,0)								
**Quartz**	**(4,5,3)**	**11.888**	**79.27**	**1.847**	**2.1**	**2.294**	**4.71**	**28.67**	**29.05**
	Equiv. Refl.: (4,-9,-3), (5,4,-3), (5,-9,3), (-9,4,3), (-9,5,-3)								
**Quartz**	**(9,-4,-3)**	**11.888**	**79.27**	**1.793**	**2.1**	**2.294**	**4.71**	**28.67**	**29.05**
	Equiv. Refl.: (9,-5,3), (-5,9,-3), (-5,-4,3)								
**Quartz**	**(-4,9,3)**	**11.888**	**79.27**	**1.792**	**2.1**	**2.294**	**4.71**	**28.67**	**29.05**
	Equiv. Refl.: (-4,-5,-3)								
**Quartz**	**(4,4,-5)**	**11.61**	**73.63**	**1.755**	**1.9**	**3.553**	**6.67**	**44.42**	**44.91**
**Quartz**	**(4,-8,5)**	**11.61**	**73.63**	**1.754**	**1.9**	**3.553**	**6.67**	**44.42**	**44.91**
	Equiv. Refl.: (-8,4,5), (-8,4,-5)								
**Quartz**	**(4,4,5)**	**11.61**	**73.63**	**1.753**	**1.9**	**3.553**	**6.66**	**44.42**	**44.91**
	Equiv. Refl.: (4,-8,-5)								
**Quartz**	**(-4,-4,5)**	**11.61**	**73.63**	**1.726**	**1.9**	**3.553**	**6.67**	**44.42**	**44.91**
**Quartz**	**(8,-4,5)**	**11.61**	**73.63**	**1.725**	**1.9**	**3.553**	**6.66**	**44.42**	**44.91**
	Equiv. Refl.: (8,-4,-5), (-4,8,5), (-4,8,-5), (-4,-4,-5)								
**Quartz**	**(4,5,-1)**	**11.437**	**70.95**	**1.689**	**1.8**	**4.179**	**7.49**	**52.24**	**52.77**
	Equiv. Refl.: (5,4,1), (5,-9,-1)								
**Quartz**	**(4,-9,1)**	**11.437**	**70.95**	**1.688**	**1.8**	**4.179**	**7.49**	**52.24**	**52.77**
	Equiv. Refl.: (-9,4,-1), (-9,5,1)								
**Sapph**	**(1,5,17)**	**11.662**	**74.53**	**1.678**	**1.9**	**3.348**	**6.35**	**41.85**	**42.33**
	Equiv. Refl.: (1,-6,-17), (5,1,-17), (5,-6,17), (6,-1,-17), (6,-5,17), (-1,6,17), (-1,-5,-17), (-5,6,-17), (-5,-1,17) (-6,1,17), (-6,5,-17)								
**Quartz**	**(9,-4,1)**	**11.437**	**70.95**	**1.673**	**1.8**	**4.179**	**7.49**	**52.24**	**52.77**
	Equiv. Refl.: (9,-5,-1), (-4,9,-1), (-4,-5,1), (-5,9,1), (-5,-4,-1)								
**Quartz**	**(2,0,10)**	**11.835**	**77.98**	**1.651**	**1.9**	**2.577**	**4.79**	**32.21**	**32.57**
	Equiv. Refl.: (-2,0,-10)								
**Quartz**	**(2,-2,-10)**	**11.835**	**77.98**	**1.65**	**1.9**	**2.577**	**4.79**	**32.21**	**32.57**
	Equiv. Refl.: (-2,2,10)								
**Quartz**	**(0,2,-10)**	**11.835**	**77.98**	**1.645**	**1.9**	**2.577**	**4.78**	**32.21**	**32.57**
	Equiv. Refl.: (0,-2,10)								
**Quartz**	**(3,6,-1)**	**11.621**	**73.83**	**1.606**	**1.6**	**3.509**	**5.78**	**43.86**	**44.24**
	Equiv. Refl.: (3,-9,1), (6,3,1), (6,-9,-1), (-9,3,-1), (-9,6,1)								
**LiNbO3**	**(2,7,-5)**	**11.599**	**73.46**	**1.545**	**2.4**	**3.594**	**8.61**	**44.92**	**45.74**
	Equiv. Refl.: (2,-9,5), (7,2,5), (7,-9,-5), (9,-2,5), (9,-7,-5), (-2,9,-5), (-2,-7,5), (-7,9,5), (-7,-2,-5) (-9,2,-5), (-9,7,5)								
**LiNbO3**	**(3,4,-19)**	**11.989**	**82.22**	**1.544**	**3.1**	**1.654**	**5.08**	**20.67**	**21.28**
	Equiv. Refl.: (3,-7,19), (4,3,19), (4,-7,-19), (7,-3,19), (7,-4,-19), (-3,7,-19), (-3,-4,19), (-4,7,19), (-4,-3,-19) (-7,3,-19), (-7,4,19)								
**Quartz**	**(8,-6,-5)**	**11.97**	**81.59**	**1.446**	**1.6**	**1.789**	**2.93**	**22.36**	**22.56**
	Equiv. Refl.: (-2,8,-5)								
**Quartz**	**(8,-2,5)**	**11.97**	**81.59**	**1.445**	**1.6**	**1.789**	**2.93**	**22.36**	**22.56**
	Equiv. Refl.: (-2,-6,5)								
**Quartz**	**(-6,8,5)**	**11.97**	**81.59**	**1.444**	**1.6**	**1.789**	**2.92**	**22.36**	**22.56**
	Equiv. Refl.: (-6,-2,-5)								
**Quartz**	**(3,5,5)**	**11.701**	**75.24**	**1.429**	**1.6**	**3.188**	**5.05**	**39.84**	**40.16**
	Equiv. Refl.: (3,-8,-5), (-8,3,5), (-8,5,-5)								

Ei =	12.1	keV						ΔEg	ΔEt
Cryst	Refl	EB	ΘB	∫ IR dΘ	Width	Ei cotΘ	ΔE	2m, 50µm	2m, 50µm
	(h,k,l)	[keV]	[°]	[µrad]	[µrad]	[meV/µrad]	[meV]	[meV]	[meV]
**Quartz**	**(5,3,-5)**	**11.701**	**75.24**	**1.428**	**1.6**	**3.188**	**5.05**	**39.84**	**40.16**
	Equiv. Refl.: (5,-8,5)								
**Quartz**	**(-5,-4,-3)**	**11.888**	**79.27**	**1.419**	**1.6**	**2.294**	**3.74**	**28.67**	**28.91**
**Quartz**	**(-5,9,3)**	**11.888**	**79.27**	**1.418**	**1.6**	**2.294**	**3.73**	**28.67**	**28.91**
**Quartz**	**(9,-4,3)**	**11.888**	**79.27**	**1.417**	**1.6**	**2.294**	**3.73**	**28.67**	**28.91**
	Equiv. Refl.: (9,-5,-3)								
**Quartz**	**(-4,9,-3)**	**11.888**	**79.27**	**1.415**	**1.6**	**2.294**	**3.73**	**28.67**	**28.91**
	Equiv. Refl.: (-4,-5,3)								
**Quartz**	**(8,-5,5)**	**11.701**	**75.24**	**1.398**	**1.6**	**3.188**	**5.05**	**39.84**	**40.16**
	Equiv. Refl.: (-3,-5,-5)								
**Quartz**	**(8,-3,-5)**	**11.701**	**75.24**	**1.397**	**1.6**	**3.188**	**5.05**	**39.84**	**40.16**
	Equiv. Refl.: (-3,8,5)								
**Quartz**	**(9,-3,1)**	**11.621**	**73.83**	**1.396**	**1.6**	**3.509**	**5.78**	**43.86**	**44.24**
	Equiv. Refl.: (9,-6,-1), (-5,8,-5), (-5,-3,5), (-6,9,1), (-6,-3,-1)								
**Quartz**	**(-3,9,-1)**	**11.621**	**73.83**	**1.395**	**1.6**	**3.509**	**5.78**	**43.86**	**44.24**
	Equiv. Refl.: (-3,-6,1)								
**LiNbO3**	**(2,7,1)**	**11.39**	**70.28**	**1.37**	**2.1**	**4.337**	**9.13**	**54.21**	**54.98**
	Equiv. Refl.: (2,-9,-1), (7,2,-1), (7,-9,1), (9,-2,-1), (9,-7,1), (-2,9,1), (-2,-7,-1), (-7,9,-1), (-7,-2,1) (-9,2,1), (-9,7,-1)								
**Quartz**	**(5,4,3)**	**11.888**	**79.27**	**1.343**	**1.6**	**2.294**	**3.74**	**28.67**	**28.91**
	Equiv. Refl.: (5,-9,-3)								
**Quartz**	**(-9,4,-3)**	**11.888**	**79.27**	**1.341**	**1.6**	**2.294**	**3.73**	**28.67**	**28.91**
	Equiv. Refl.: (-9,5,3)								
**Quartz**	**(4,5,-3)**	**11.888**	**79.27**	**1.34**	**1.6**	**2.294**	**3.73**	**28.67**	**28.91**
	Equiv. Refl.: (4,-9,3)								
**Quartz**	**(0,8,2)**	**11.879**	**79.04**	**1.286**	**1.5**	**2.342**	**3.58**	**29.28**	**29.49**
	Equiv. Refl.: (0,-8,-2), (8,0,-2), (8,-8,2), (-8,0,2)								
**Quartz**	**(-8,8,-2)**	**11.879**	**79.04**	**1.285**	**1.5**	**2.342**	**3.57**	**29.28**	**29.49**
**Sapph**	**(1,7,3)**	**11.448**	**71.11**	**1.267**	**1.5**	**4.141**	**6.03**	**51.77**	**52.12**
	Equiv. Refl.: (1,7,-3), (1,-8,3), (1,-8,-3), (7,1,3), (7,1,-3), (7,-8,3), (7,-8,-3), (8,-1,3), (8,-1,-3) (8,-7,3), (8,-7,-3), (-1,8,3), (-1,8,-3), (-1,-7,3), (-1,-7,-3), (-7,8,3), (-7,8,-3), (-7,-1,3) (-7,-1,-3), (-8,1,3), (-8,1,-3), (-8,7,3), (-8,7,-3)								
**Quartz**	**(2,-8,5)**	**11.97**	**81.59**	**1.19**	**1.6**	**1.789**	**2.93**	**22.36**	**22.56**
	Equiv. Refl.: (-8,2,-5), (-8,6,5)								
**Quartz**	**(2,6,-5)**	**11.97**	**81.59**	**1.189**	**1.6**	**1.789**	**2.93**	**22.36**	**22.56**
**Quartz**	**(6,2,5)**	**11.97**	**81.59**	**1.188**	**1.6**	**1.789**	**2.92**	**22.36**	**22.56**
	Equiv. Refl.: (6,-8,-5)								
**Quartz**	**(-6,2,8)**	**11.985**	**82.09**	**1.177**	**1.5**	**1.68**	**2.46**	**21**	**21.15**
	Equiv. Refl.: (-6,4,-8)								
**Quartz**	**(4,2,-8)**	**11.985**	**82.09**	**1.176**	**1.5**	**1.68**	**2.46**	**21**	**21.15**
**Quartz**	**(2,4,8)**	**11.985**	**82.09**	**1.175**	**1.5**	**1.68**	**2.45**	**21**	**21.15**
	Equiv. Refl.: (2,-6,-8), (4,-6,8)								
**Quartz**	**(5,2,-6)**	**11.408**	**70.54**	**1.158**	**1.3**	**4.276**	**5.46**	**53.46**	**53.73**
	Equiv. Refl.: (5,-7,6)								
**Quartz**	**(2,-7,-6)**	**11.408**	**70.54**	**1.157**	**1.3**	**4.276**	**5.46**	**53.46**	**53.73**
	Equiv. Refl.: (-7,2,6), (-7,5,-6)								
**Quartz**	**(2,5,6)**	**11.408**	**70.54**	**1.156**	**1.3**	**4.276**	**5.45**	**53.46**	**53.73**
**Quartz**	**(-5,7,-6)**	**11.408**	**70.54**	**1.13**	**1.3**	**4.276**	**5.46**	**53.46**	**53.73**
	Equiv. Refl.: (-5,-2,6)								
**Quartz**	**(7,-2,-6)**	**11.408**	**70.54**	**1.129**	**1.3**	**4.276**	**5.46**	**53.46**	**53.73**
	Equiv. Refl.: (7,-5,6), (-2,7,6), (-2,-5,-6)								

Ei =	12.1	keV						ΔEg	ΔEt
**Cryst**	**Refl**	**EB**	**ΘB**	**∫ IR dΘ**	**Width**	**Ei cotΘ**	**ΔE**	**2m, 50µm**	**2m, 50µm**
	**(h,k,l)**	**[keV]**	**[°]**	**[µrad]**	**[µrad]**	**[meV/µrad]**	**[meV]**	**[meV]**	**[meV]**
**Quartz**	**(7,0,-5)**	**11.701**	**75.24**	**1.11**	**1.3**	**3.188**	**4.12**	**39.84**	**40.06**
	Equiv. Refl.: (7,-7,5), (-7,0,5), (-7,7,-5)								
**Quartz**	**(0,7,5)**	**11.701**	**75.24**	**1.109**	**1.3**	**3.188**	**4.11**	**39.84**	**40.06**
	Equiv. Refl.: (0,-7,-5)								
**Quartz**	**(-5,8,5)**	**11.701**	**75.24**	**1.072**	**1.2**	**3.188**	**3.91**	**39.84**	**40.04**
	Equiv. Refl.: (-5,-3,-5)								
**LiNbO3**	**(4,5,11)**	**11.922**	**80.16**	**1.071**	**2.2**	**2.098**	**4.72**	**26.23**	**26.65**
	Equiv. Refl.: (4,-9,-11), (5,4,-11), (5,-9,11), (9,-4,-11), (9,-5,11), (-4,9,11), (-4,-5,-11), (-5,9,-11), (-5,-4,11) (-9,4,11), (-9,5,-11), (-3,8,-5), (-3,-5,5)								
**Quartz**	**(8,-3,5)**	**11.701**	**75.24**	**1.07**	**1.2**	**3.188**	**3.91**	**39.84**	**40.04**
	Equiv. Refl.: (8,-5,-5)								
**Quartz**	**(6,-2,-8)**	**11.985**	**82.09**	**1.065**	**1.5**	**1.68**	**2.46**	**21**	**21.15**
	Equiv. Refl.: (6,-4,8), (-4,-2,8)								
**Quartz**	**(-2,6,8)**	**11.985**	**82.09**	**1.064**	**1.5**	**1.68**	**2.45**	**21**	**21.15**
	Equiv. Refl.: (-2,-4,-8), (-4,6,-8)								
**Quartz**	**(4,5,2)**	**11.608**	**73.61**	**1.052**	**1.2**	**3.559**	**4.31**	**44.48**	**44.69**
	Equiv. Refl.: (5,4,-2), (5,-9,2), (-9,4,2), (-9,5,-2)								
**Quartz**	**(4,-9,-2)**	**11.608**	**73.61**	**1.051**	**1.2**	**3.559**	**4.31**	**44.48**	**44.69**
**Sapph**	**(3,5,-11)**	**11.767**	**76.53**	**1.048**	**1.3**	**2.898**	**3.83**	**36.23**	**36.43**
	Equiv. Refl.: (3,-8,11), (5,3,11), (5,-8,-11), (8,-3,11), (8,-5,-11), (-3,8,-11), (-3,-5,11), (-5,8,11), (-5,-3,-11) (-8,3,-11), (-8,5,11)								
**Quartz**	**(9,-4,-2)**	**11.608**	**73.61**	**1.042**	**1.2**	**3.559**	**4.31**	**44.48**	**44.69**
	Equiv. Refl.: (9,-5,2), (-5,9,-2), (-5,-4,2)								
**Quartz**	**(-4,9,2)**	**11.608**	**73.61**	**1.041**	**1.2**	**3.559**	**4.31**	**44.48**	**44.69**
	Equiv. Refl.: (-4,-5,-2)								
**Quartz**	**(5,3,5)**	**11.701**	**75.24**	**1.016**	**1.2**	**3.188**	**3.92**	**39.84**	**40.04**
	Equiv. Refl.: (5,-8,-5)								
**Quartz**	**(3,5,-5)**	**11.701**	**75.24**	**1.015**	**1.2**	**3.188**	**3.91**	**39.84**	**40.04**
	Equiv. Refl.: (3,-8,5)								
**Quartz**	**(-8,3,-5)**	**11.701**	**75.24**	**1.014**	**1.2**	**3.188**	**3.91**	**39.84**	**40.04**
	Equiv. Refl.: (-8,5,5)								
**Quartz**	**(6,0,7)**	**11.87**	**78.8**	**0.9**	**1.1**	**2.396**	**2.74**	**29.94**	**30.07**
	Equiv. Refl.: (6,-6,-7), (-6,6,7)								
**Quartz**	**(0,6,-7)**	**11.87**	**78.8**	**0.899**	**1.1**	**2.396**	**2.74**	**29.94**	**30.07**
	Equiv. Refl.: (0,-6,7), (-6,0,-7)								
**LiNbO3**	**(1,3,-23)**	**11.442**	**71.02**	**0.757**	**1.4**	**4.162**	**5.99**	**52.03**	**52.37**
	Equiv. Refl.: (1,-4,23), (3,1,23), (3,-4,-23), (4,-1,23), (4,-3,-23), (-1,4,-23), (-1,-3,23), (-3,4,23), (-3,-1,-23) (-4,1,-23), (-4,3,23)								
**LiNbO3**	**(1,8,5)**	**12.089**	**87.55**	**0.755**	**3.5**	**0.519**	**1.81**	**6.48**	**6.73**
	Equiv. Refl.: (1,-9,-5), (8,1,-5), (8,-9,5), (9,-1,-5), (9,-8,5), (-1,9,5), (-1,-8,-5), (-8,9,-5), (-8,-1,5) (-9,1,5), (-9,8,-5)								
**Quartz**	**(0,8,1)**	**11.712**	**75.45**	**0.733**	**0.9**	**3.139**	**2.91**	**39.24**	**39.35**
	Equiv. Refl.: (0,-8,-1)								
**Quartz**	**(8,0,-1)**	**11.712**	**75.45**	**0.732**	**0.9**	**3.139**	**2.91**	**39.24**	**39.35**
	Equiv. Refl.: (8,-8,1), (-8,0,1), (-8,8,-1)								
**Sapph**	**(1,3,22)**	**11.819**	**77.62**	**0.731**	**1**	**2.656**	**2.72**	**33.2**	**33.31**
	Equiv. Refl.: (1,-4,-22), (3,1,-22), (3,-4,22), (4,-1,-22), (4,-3,22), (-1,4,22), (-1,-3,-22), (-3,4,-22), (-3,-1,22) (-4,1,22), (-4,3,-22)								

Ei =	12.1	keV						ΔEg	ΔEt
Cryst	Refl	EB	ΘB	∫ IR dΘ	Width	Ei cotΘ	ΔE	2m, 50µm	2m, 50µm
	(h,k,l)	[keV]	[°]	[µrad]	[µrad]	[meV/µrad]	[meV]	[meV]	[meV]
**LiNbO3**	**(3,4,17)**	**11.372**	**70.03**	**0.713**	**1.4**	**4.397**	**6**	**54.97**	**55.3**
	Equiv. Refl.: (3,-7,-17), (4,3,-17), (4,-7,17), (7,-3,-17), (7,-4,17), (-3,7,17), (-3,-4,-17), (-4,7,-17), (-4,-3,17) (-7,3,17), (-7,4,-17)								
**Sapph**	**(1,2,23)**	**11.677**	**74.81**	**0.693**	**0.9**	**3.286**	**3.08**	**41.08**	**41.19**
	Equiv. Refl.: (1,-3,-23), (2,1,-23), (2,-3,23), (3,-1,-23), (3,-2,23), (-1,3,23), (-1,-2,-23), (-2,3,-23), (-2,-1,23) (-3,1,23), (-3,2,-23)								
**Quartz**	**(5,0,8)**	**11.716**	**75.53**	**0.682**	**0.9**	**3.122**	**2.72**	**39.02**	**39.12**
	Equiv. Refl.: (5,-5,-8), (-5,0,-8), (-5,5,8)								
**Quartz**	**(0,5,-8)**	**11.716**	**75.53**	**0.68**	**0.9**	**3.122**	**2.71**	**39.02**	**39.12**
	Equiv. Refl.: (0,-5,8)								
**Sapph**	**(3,4,-16)**	**11.919**	**80.07**	**0.667**	**1**	**2.117**	**2.14**	**26.47**	**26.55**
	Equiv. Refl.: (3,-7,16), (4,3,16), (4,-7,-16), (7,-3,16), (7,-4,-16), (-3,7,-16), (-3,-4,16), (-4,7,16), (-4,-3,-16) (-7,3,-16), (-7,4,16)								
**Quartz**	**(1,1,-10)**	**11.745**	**76.08**	**0.656**	**0.8**	**3**	**2.33**	**37.49**	**37.57**
	Equiv. Refl.: (1,-2,10), (1,-2,-10)								
**Quartz**	**(1,1,10)**	**11.745**	**76.08**	**0.655**	**0.8**	**3**	**2.33**	**37.49**	**37.57**
**Quartz**	**(-2,1,10)**	**11.745**	**76.08**	**0.653**	**0.8**	**3**	**2.32**	**37.49**	**37.57**
	Equiv. Refl.: (-2,1,-10)								
**Quartz**	**(9,-2,0)**	**11.926**	**80.27**	**0.6**	**0.8**	**2.075**	**1.65**	**25.94**	**25.99**
	Equiv. Refl.: (9,-7,0), (-2,9,0), (-2,-7,0), (-7,9,0), (-7,-2,0)								
**LiNbO3**	**(1,2,-25)**	**11.769**	**76.57**	**0.594**	**1.4**	**2.889**	**4.07**	**36.11**	**36.34**
	Equiv. Refl.: (1,-3,25), (2,1,25), (2,-3,-25), (3,-1,25), (3,-2,-25), (-1,3,-25), (-1,-2,25), (-2,3,25), (-2,-1,-25) (-3,1,-25), (-3,2,25)								
**Sapph**	**(1,6,13)**	**11.654**	**74.4**	**0.59**	**0.8**	**3.379**	**2.8**	**42.23**	**42.33**
	Equiv. Refl.: (1,-7,-13), (6,1,-13), (6,-7,13), (7,-1,-13), (7,-6,13), (-1,7,13), (-1,-6,-13), (-6,7,-13), (-6,-1,13) (-7,1,13), (-7,6,-13)								
**LiNbO3**	**(1,5,-19)**	**11.495**	**71.8**	**0.523**	**1.2**	**3.979**	**4.64**	**49.73**	**49.95**
	Equiv. Refl.: (1,-6,19), (5,1,19), (5,-6,-19), (6,-1,19), (6,-5,-19), (-1,6,-19), (-1,-5,19), (-5,6,19), (-5,-1,-19) (-6,1,-19), (-6,5,19)								
**Quartz**	**(-1,2,10)**	**11.745**	**76.08**	**0.51**	**0.8**	**3**	**2.33**	**37.49**	**37.57**
	Equiv. Refl.: (-1,2,-10), (-1,-1,10), (-1,-1,-10)								
**Quartz**	**(2,-1,10)**	**11.745**	**76.08**	**0.508**	**0.8**	**3**	**2.33**	**37.49**	**37.57**
**Quartz**	**(2,-1,-10)**	**11.745**	**76.08**	**0.507**	**0.8**	**3**	**2.32**	**37.49**	**37.57**
**Quartz**	**(0,8,-2)**	**11.879**	**79.04**	**0.478**	**0.7**	**2.342**	**1.69**	**29.28**	**29.33**
	Equiv. Refl.: (0,-8,2), (8,0,2), (8,-8,-2), (-8,0,-2), (-8,8,2)								
**Quartz**	**(2,7,0)**	**11.926**	**80.27**	**0.456**	**0.8**	**2.075**	**1.65**	**25.94**	**25.99**
	Equiv. Refl.: (2,-9,0), (7,2,0), (7,-9,0), (-9,2,0), (-9,7,0)								
**Sapph**	**(3,6,3)**	**12.027**	**83.68**	**0.441**	**0.9**	**1.34**	**1.22**	**16.75**	**16.79**
	Equiv. Refl.: (3,6,-3), (3,-9,3), (3,-9,-3), (6,3,3), (6,3,-3), (6,-9,3), (6,-9,-3), (9,-3,3), (9,-3,-3) (9,-6,3), (9,-6,-3), (-3,9,3), (-3,9,-3), (-3,-6,3), (-3,-6,-3), (-6,9,3), (-6,9,-3), (-6,-3,3) (-6,-3,-3), (-9,3,3), (-9,3,-3), (-9,6,3), (-9,6,-3)								
**LiNbO3**	**(3,6,9)**	**11.748**	**76.14**	**0.438**	**1.2**	**2.986**	**3.49**	**37.33**	**37.49**
	Equiv. Refl.: (3,6,-9), (3,-9,9), (3,-9,-9), (6,3,9), (6,3,-9), (6,-9,9), (6,-9,-9), (9,-3,9), (9,-3,-9) (9,-6,9), (9,-6,-9), (-3,9,9), (-3,9,-9), (-3,-6,9), (-3,-6,-9), (-6,9,9), (-6,9,-9), (-6,-3,9) (-6,-3,-9), (-9,3,9), (-9,3,-9), (-9,6,9), (-9,6,-9)								
**Quartz**	**(0,2,10)**	**11.835**	**77.98**	**0.406**	**0.6**	**2.577**	**1.62**	**32.21**	**32.26**
	Equiv. Refl.: (0,-2,-10)								
**Quartz**	**(8,-2,-4)**	**11.465**	**71.35**	**0.405**	**0.5**	**4.084**	**2.09**	**51.05**	**51.09**
	Equiv. Refl.: (8,-6,4)								
**Quartz**	**(-2,-6,-4)**	**11.465**	**71.35**	**0.404**	**0.5**	**4.084**	**2.08**	**51.05**	**51.09**
	Equiv. Refl.: (-6,8,-4), (-6,-2,4)								
**Quartz**	**(-2,8,4)**	**11.465**	**71.35**	**0.403**	**0.5**	**4.084**	**2.08**	**51.05**	**51.09**

Ei =	12.1	keV						ΔEg	ΔEt
**Cryst**	**Refl**	**EB**	**ΘB**	**∫ IR dΘ**	**Width**	**Ei cotΘ**	**ΔE**	**2m, 50μm**	**2m, 50μm**
	**(h,k,l)**	**[keV]**	**[°]**	**[μrad]**	**[μrad]**	**[meV/μrad]**	**[meV]**	**[meV]**	**[meV]**
**Quartz**	**(-2,0,10)**	**11.835**	**77.98**	**0.4**	**0.6**	**2.577**	**1.6**	**32.21**	**32.25**
**Quartz**	**(2,0,-10)**	**11.835**	**77.98**	**0.399**	**0.6**	**2.577**	**1.6**	**32.21**	**32.25**
	Equiv. Refl.: (2,-2,10), (-2,2,-10)								
**Quartz**	**(7,-2,6)**	**11.408**	**70.54**	**0.368**	**0.5**	**4.276**	**2.1**	**53.46**	**53.5**
	Equiv. Refl.: (7,-5,-6)								
**Quartz**	**(-2,7,-6)**	**11.408**	**70.54**	**0.367**	**0.5**	**4.276**	**2.1**	**53.46**	**53.5**
	Equiv. Refl.: (-2,-5,6), (-5,7,6), (-5,-2,-6)								
**Quartz**	**(-1,6,7)**	**11.414**	**70.61**	**0.334**	**0.4**	**4.258**	**1.85**	**53.23**	**53.26**
	Equiv. Refl.: (-1,-5,-7), (-5,6,-7)								
**Quartz**	**(-5,-1,7)**	**11.414**	**70.61**	**0.333**	**0.4**	**4.258**	**1.85**	**53.23**	**53.26**
**Quartz**	**(6,-1,-7)**	**11.414**	**70.61**	**0.332**	**0.4**	**4.258**	**1.85**	**53.23**	**53.26**
	Equiv. Refl.: (6,-5,7)								
**Quartz**	**(-7,2,-6)**	**11.408**	**70.54**	**0.32**	**0.5**	**4.276**	**2.1**	**53.46**	**53.5**
	Equiv. Refl.: (-7,5,6)								
**Quartz**	**(2,5,-6)**	**11.408**	**70.54**	**0.319**	**0.5**	**4.276**	**2.1**	**53.46**	**53.5**
	Equiv. Refl.: (2,-7,6), (5,2,6), (5,-7,-6)								
**Quartz**	**(6,2,-4)**	**11.465**	**71.35**	**0.316**	**0.5**	**4.084**	**2.09**	**51.05**	**51.09**
	Equiv. Refl.: (6,-8,4), (-8,2,4), (-8,6,-4)								
**Quartz**	**(2,6,4)**	**11.465**	**71.35**	**0.315**	**0.5**	**4.084**	**2.08**	**51.05**	**51.09**
**Quartz**	**(2,-8,-4)**	**11.465**	**71.35**	**0.314**	**0.5**	**4.084**	**2.08**	**51.05**	**51.09**
**LiNbO3**	**(2,3,23)**	**11.938**	**80.62**	**0.308**	**1.1**	**2**	**2.3**	**24.99**	**25.1**
	Equiv. Refl.: (2,-5,-23), (3,2,-23), (3,-5,23), (5,-2,-23), (5,-3,23), (-2,5,23), (-2,-3,-23), (-3,5,-23), (-3,-2,23) (-5,2,23), (-5,3,-23)								
**LiNbO3**	**(2,6,-13)**	**11.59**	**73.31**	**0.303**	**0.9**	**3.628**	**3.21**	**45.35**	**45.46**
	Equiv. Refl.: (2,-8,13), (6,2,13), (6,-8,-13), (8,-2,13), (8,-6,-13), (-2,8,-13), (-2,-6,13), (-6,8,13), (-6,-2,-13) (-8,2,-13), (-8,6,13)								
**Quartz**	**(4,5,0)**	**11.379**	**70.13**	**0.268**	**0.4**	**4.374**	**1.77**	**54.67**	**54.7**
	Equiv. Refl.: (4,-9,0), (5,4,0), (5,-9,0), (-9,4,0), (-9,5,0)								
**Quartz**	**(9,-4,0)**	**11.379**	**70.13**	**0.26**	**0.4**	**4.374**	**1.77**	**54.67**	**54.7**
	Equiv. Refl.: (9,-5,0), (-4,9,0), (-4,-5,0), (-5,9,0), (-5,-4,0)								
**Quartz**	**(1,5,7)**	**11.414**	**70.61**	**0.25**	**0.4**	**4.258**	**1.85**	**53.23**	**53.26**
	Equiv. Refl.: (1,-6,-7)								
**Quartz**	**(5,1,-7)**	**11.414**	**70.61**	**0.249**	**0.4**	**4.258**	**1.85**	**53.23**	**53.26**
	Equiv. Refl.: (5,-6,7)								
**Quartz**	**(-6,1,7)**	**11.414**	**70.61**	**0.248**	**0.4**	**4.258**	**1.85**	**53.23**	**53.26**
	Equiv. Refl.: (-6,5,-7)								
**LiNbO3**	**(1,8,-1)**	**11.889**	**79.28**	**0.184**	**0.8**	**2.291**	**1.91**	**28.64**	**28.7**
	Equiv. Refl.: (1,-9,1), (8,1,1), (8,-9,-1), (9,-1,1), (9,-8,-1), (-1,9,-1), (-1,-8,1), (-8,9,1), (-8,-1,-1) (-9,1,-1), (-9,8,1)								
**Sapph**	**(0,0,24)**	**11.455**	**71.21**	**0.121**	**0.3**	**4.117**	**1.05**	**51.47**	**51.48**
	Equiv. Refl.: (0,0,-24)								
**LiNbO3**	**(3,3,21)**	**11.849**	**78.3**	**0.096**	**0.6**	**2.506**	**1.46**	**31.32**	**31.36**
	Equiv. Refl.: (3,3,-21), (3,-6,21), (3,-6,-21), (6,-3,21), (6,-3,-21), (-3,6,21), (-3,6,-21), (-3,-3,21), (-3,-3,-21) (-6,3,21), (-6,3,-21)								
**Sapph**	**(2,6,8)**	**11.501**	**71.89**	**0.083**	**0.2**	**3.956**	**0.88**	**49.45**	**49.46**
	Equiv. Refl.: (2,-8,-8), (6,2,-8), (6,-8,8), (8,-2,-8), (8,-6,8), (-2,8,8), (-2,-6,-8), (-6,8,-8), (-6,-2,8) (-8,2,8), (-8,6,-8)								
**Sapph**	**(4,5,2)**	**11.789**	**76.97**	**0.07**	**0.2**	**2.799**	**0.66**	**34.99**	**35**
	Equiv. Refl.: (4,-9,-2), (5,4,-2), (5,-9,2), (9,-4,-2), (9,-5,2), (-4,9,2), (-4,-5,-2), (-5,9,-2), (-5,-4,2) (-9,4,2), (-9,5,-2)								
**Sapph**	**(4,4,12)**	**11.893**	**79.38**	**0.056**	**0.2**	**2.268**	**0.52**	**28.35**	**28.35**
	Equiv. Refl.: (4,4,-12), (4,-8,12), (4,-8,-12), (8,-4,12), (8,-4,-12), (-4,8,12), (-4,8,-12), (-4,-4,12), (-4,-4,-12)								

	(-8,4,12), (-8,4,-12)								
**LiNbO3**	**(4,4,15)**	**11.739**	**75.96**	**0.015**	**0.2**	**3.025**	**0.7**	**37.81**	**37.82**
	Equiv. Refl.: (4,4,-15), (4,-8,15), (4,-8,-15), (8,-4,15), (8,-4,-15), (-4,8,15), (-4,8,-15), (-4,-4,15), (-4,-4,-15) (-8,4,15), (-8,4,-15)								

Ei =	11.544	keV						ΔEg	ΔEt
Cryst	Refl	EB	ΘB	∫ IR dΘ	Width	Ei cotΘ	ΔE	2m, 50µm	2m, 50µm
	(h,k,l)	[keV]	[°]	[µrad]	[µrad]	[meV/µrad]	[meV]	[meV]	[meV]
**Si**	**(4,4,8)**	**11.184**	**75.65**	**5.9088**	**5.07**	**2.9532**	**14.97**	**36.92**	**39.84**
	Equiv. Refl.: (4,8,4), (8,4,4)								
**Si**	**(1,7,7)**	**11.357**	**79.68**	**5.2578**	**4.72**	**2.1022**	**9.93**	**26.28**	**28.09**
	Equiv. Refl.: (3,3,9), (3,9,3), (5,5,7), (5,7,5), (7,1,7), (7,5,5), (7,7,1), (9,3,3)								
**Ge**	**(0,2,10)**	**11.175**	**75.47**	**3.7974**	**7.83**	**2.9918**	**23.42**	**37.4**	**44.13**
	Equiv. Refl.: (0,10,2), (2,0,10), (2,6,8), (2,8,6), (2,10,0), (6,2,8), (6,8,2), (8,2,6), (8,6,2) (10,0,2), (10,2,0)								
**Si**	**(1,3,9)**	**10.889**	**70.6**	**3.3066**	**2.91**	**4.0649**	**11.82**	**50.81**	**52.17**
	Equiv. Refl.: (1,9,3), (3,1,9), (3,9,1), (9,1,3), (9,3,1)								
**Ge**	**(1,5,9)**	**11.335**	**79.08**	**2.4546**	**6.98**	**2.2279**	**15.55**	**27.85**	**31.89**
	Equiv. Refl.: (1,9,5), (3,7,7), (5,1,9), (5,9,1), (7,3,7), (7,7,3), (9,1,5), (9,5,1)								
**Ge**	**(1,7,7)**	**10.903**	**70.81**	**1.7105**	**4.6**	**4.0167**	**18.46**	**50.21**	**53.49**
	Equiv. Refl.: (3,3,9), (3,9,3), (5,5,7), (5,7,5), (7,1,7), (7,5,5), (7,7,1), (9,3,3)								
**LiNbO3**	**(2,7,4)**	**11.521**	**86.41**	**39.571**	**36.5**	**0.725**	**26.47**	**9.06**	**27.98**
	Equiv. Refl.: (7,-9,4), (9,-7,4), (-2,9,4), (-7,-2,4), (-9,2,4)								
**LiNbO3**	**(2,-9,-4)**	**11.521**	**86.41**	**36.953**	**36.5**	**0.725**	**26.47**	**9.06**	**27.98**
	Equiv. Refl.: (7,2,-4), (9,-2,-4), (-2,-7,-4), (-7,9,-4), (-9,7,-4)								
**LiNbO3**	**(0,-3,-24)**	**11.514**	**85.9**	**27.568**	**26.1**	**0.828**	**21.64**	**10.35**	**23.98**
	Equiv. Refl.: (3,0,-24), (-3,3,-24)								
**LiNbO3**	**(0,3,-24)**	**11.514**	**85.9**	**27.065**	**25.8**	**0.828**	**21.38**	**10.35**	**23.76**
	Equiv. Refl.: (3,-3,-24), (-3,0,-24)								
**LiNbO3**	**(0,3,24)**	**11.514**	**85.9**	**25.065**	**26.1**	**0.828**	**21.64**	**10.35**	**23.98**
	Equiv. Refl.: (3,-3,24), (-3,0,24)								
**LiNbO3**	**(0,-3,24)**	**11.514**	**85.9**	**24.837**	**25.8**	**0.828**	**21.38**	**10.35**	**23.76**
	Equiv. Refl.: (3,0,24)								
**LiNbO3**	**(-3,3,24)**	**11.514**	**85.9**	**24.836**	**25.8**	**0.828**	**21.38**	**10.35**	**23.76**
**LiNbO3**	**(2,7,-2)**	**11.417**	**81.48**	**22.154**	**20.2**	**1.729**	**34.99**	**21.61**	**41.12**
	Equiv. Refl.: (7,-9,-2), (9,-7,-2), (-2,9,-2), (-7,-2,-2), (-9,2,-2)								
**LiNbO3**	**(2,-9,2)**	**11.417**	**81.48**	**22.127**	**20.2**	**1.729**	**34.99**	**21.61**	**41.12**
	Equiv. Refl.: (7,2,2), (9,-2,2), (-2,-7,2), (-7,9,2), (-9,7,2)								
**LiNbO3**	**(0,-8,-4)**	**11.267**	**77.42**	**18.808**	**16.5**	**2.576**	**42.64**	**32.21**	**53.44**
	Equiv. Refl.: (8,0,-4), (-8,8,-4)								
**LiNbO3**	**(0,8,4)**	**11.267**	**77.42**	**18.296**	**16.5**	**2.576**	**42.64**	**32.21**	**53.44**
	Equiv. Refl.: (8,-8,4), (-8,0,4)								
**LiNbO3**	**(3,6,-6)**	**11.358**	**79.7**	**16.708**	**15.5**	**2.097**	**32.43**	**26.21**	**41.7**
	Equiv. Refl.: (-3,9,-6), (-6,-3,-6), (-9,3,-6)								
**LiNbO3**	**(6,-9,-6)**	**11.358**	**79.7**	**16.707**	**15.5**	**2.097**	**32.43**	**26.21**	**41.7**
	Equiv. Refl.: (9,-6,-6)								
**LiNbO3**	**(4,5,8)**	**11.434**	**82.09**	**16.699**	**15.8**	**1.604**	**25.35**	**20.05**	**32.33**
	Equiv. Refl.: (5,-9,8), (9,-5,8), (-4,9,8), (-5,-4,8), (-9,4,8)								
**LiNbO3**	**(3,-9,6)**	**11.358**	**79.7**	**16.662**	**15.5**	**2.097**	**32.43**	**26.21**	**41.7**
	Equiv. Refl.: (6,3,6), (9,-3,6), (-3,-6,6), (-6,9,6), (-9,6,6)								
**LiNbO3**	**(4,-9,-8)**	**11.434**	**82.09**	**16.046**	**15.8**	**1.604**	**25.35**	**20.05**	**32.32**
	Equiv. Refl.: (5,4,-8), (9,-4,-8), (-4,-5,-8), (-5,9,-8), (-9,5,-8)								
**LiNbO3**	**(3,-9,-6)**	**11.358**	**79.7**	**15.634**	**14.4**	**2.097**	**30.18**	**26.21**	**39.97**
	Equiv. Refl.: (6,3,-6), (9,-3,-6), (-3,-6,-6), (-6,9,-6), (-9,6,-6)								
**Quartz**	**(-1,8,3)**	**11.526**	**86.77**	**15.357**	**13**	**0.651**	**8.49**	**8.14**	**11.76**
	Equiv. Refl.: (-1,-7,-3)								

Ei =	11.544	keV						ΔEg	ΔEt
**Cryst**	**Refl**	**EB**	**ΘB**	**∫ IR dΘ**	**Width**	**Ei cotΘ**	**ΔE**	**2m, 50µm**	**2m, 50µm**
	**(h,k,l)**	**[keV]**	**[°]**	**[µrad]**	**[µrad]**	**[meV/µrad]**	**[meV]**	**[meV]**	**[meV]**
**Quartz**	**(-7,8,-3)**	**11.526**	**86.77**	**15.356**	**13**	**0.651**	**8.49**	**8.14**	**11.76**
**Quartz**	**(-7,-1,3)**	**11.526**	**86.77**	**15.355**	**13**	**0.651**	**8.49**	**8.14**	**11.76**
**Quartz**	**(8,-1,-3)**	**11.526**	**86.77**	**15.354**	**13**	**0.651**	**8.49**	**8.14**	**11.76**
**Quartz**	**(8,-7,3)**	**11.526**	**86.77**	**15.353**	**13**	**0.651**	**8.49**	**8.14**	**11.76**
**LiNbO3**	**(3,6,6)**	**11.358**	**79.7**	**15.056**	**14.4**	**2.097**	**30.18**	**26.21**	**39.97**
	Equiv. Refl.: (6,-9,6), (9,-6,6), (-3,9,6), (-6,-3,6), (-9,3,6)								
**Quartz**	**(1,7,3)**	**11.526**	**86.77**	**14.764**	**13**	**0.651**	**8.49**	**8.14**	**11.76**
	Equiv. Refl.: (1,-8,-3), (7,1,-3), (7,-8,3)								
**Quartz**	**(-8,1,3)**	**11.526**	**86.77**	**14.761**	**13**	**0.651**	**8.49**	**8.14**	**11.76**
**Quartz**	**(-8,7,-3)**	**11.526**	**86.77**	**14.76**	**13**	**0.651**	**8.49**	**8.14**	**11.76**
**Quartz**	**(-8,1,-3)**	**11.526**	**86.77**	**14.478**	**12.2**	**0.651**	**7.94**	**8.14**	**11.38**
	Equiv. Refl.: (-8,7,3)								
**Quartz**	**(1,7,-3)**	**11.526**	**86.77**	**14.477**	**12.2**	**0.651**	**7.94**	**8.14**	**11.37**
	Equiv. Refl.: (7,1,3), (7,-8,-3)								
**Quartz**	**(1,-8,3)**	**11.526**	**86.77**	**14.475**	**12.2**	**0.651**	**7.94**	**8.14**	**11.37**
**Quartz**	**(8,-1,3)**	**11.526**	**86.77**	**13.287**	**12.2**	**0.651**	**7.94**	**8.14**	**11.38**
	Equiv. Refl.: (8,-7,-3), (-1,-7,3), (-7,8,3)								
**Quartz**	**(-7,-1,-3)**	**11.526**	**86.77**	**13.286**	**12.2**	**0.651**	**7.94**	**8.14**	**11.37**
**Quartz**	**(-1,8,-3)**	**11.526**	**86.77**	**13.285**	**12.2**	**0.651**	**7.94**	**8.14**	**11.37**
**LiNbO3**	**(0,4,-22)**	**11.301**	**78.23**	**13.155**	**12.2**	**2.405**	**29.28**	**30.07**	**41.97**
	Equiv. Refl.: (4,-4,-22), (-4,0,-22)								
**LiNbO3**	**(0,-4,22)**	**11.301**	**78.23**	**12.625**	**12.2**	**2.405**	**29.28**	**30.07**	**41.97**
	Equiv. Refl.: (4,0,22), (-4,4,22)								
**LiNbO3**	**(0,5,-20)**	**11.328**	**78.9**	**12.137**	**10.8**	**2.265**	**24.58**	**28.31**	**37.49**
	Equiv. Refl.: (5,-5,-20), (-5,0,-20)								
**LiNbO3**	**(1,1,24)**	**10.999**	**72.33**	**12.09**	**10.9**	**3.678**	**40.15**	**45.98**	**61.04**
	Equiv. Refl.: (1,-2,24), (2,-1,24), (-1,-1,24)								
**LiNbO3**	**(-1,2,24)**	**10.999**	**72.33**	**12.089**	**10.9**	**3.678**	**40.15**	**45.98**	**61.04**
	Equiv. Refl.: (-2,1,24)								
**LiNbO3**	**(1,1,-24)**	**10.999**	**72.33**	**12.013**	**10.9**	**3.678**	**40.15**	**45.98**	**61.04**
	Equiv. Refl.: (1,-2,-24), (2,-1,-24), (-1,2,-24), (-1,-1,-24), (-2,1,-24)								
**Quartz**	**(4,-2,9)**	**11.491**	**84.5**	**11.139**	**9.2**	**1.111**	**10.2**	**13.88**	**17.23**
	Equiv. Refl.: (4,-2,-9)								
**Quartz**	**(-2,4,-9)**	**11.491**	**84.5**	**11.135**	**9.2**	**1.111**	**10.19**	**13.88**	**17.22**
**Quartz**	**(-2,-2,9)**	**11.491**	**84.5**	**11.133**	**9.2**	**1.111**	**10.19**	**13.88**	**17.22**
**Quartz**	**(-2,-2,-9)**	**11.491**	**84.5**	**11.131**	**9.2**	**1.111**	**10.19**	**13.88**	**17.22**
**Quartz**	**(-2,4,9)**	**11.491**	**84.5**	**11.129**	**9.2**	**1.111**	**10.19**	**13.88**	**17.22**
**LiNbO3**	**(1,6,-14)**	**11.06**	**73.36**	**10.861**	**9.8**	**3.451**	**33.75**	**43.14**	**54.77**
	Equiv. Refl.: (6,-7,-14), (7,-6,-14), (-1,7,-14), (-6,-1,-14), (-7,1,-14)								
**LiNbO3**	**(4,5,2)**	**10.897**	**70.72**	**10.727**	**9.7**	**4.037**	**39.18**	**50.47**	**63.89**
	Equiv. Refl.: (5,-9,2), (9,-5,2), (-4,9,2), (-5,-4,2), (-9,4,2)								
**Quartz**	**(-4,2,-9)**	**11.491**	**84.5**	**10.691**	**9.2**	**1.111**	**10.2**	**13.88**	**17.23**
**Quartz**	**(-4,2,9)**	**11.491**	**84.5**	**10.69**	**9.2**	**1.111**	**10.2**	**13.88**	**17.23**
**Quartz**	**(2,-4,9)**	**11.491**	**84.5**	**10.688**	**9.2**	**1.111**	**10.19**	**13.88**	**17.22**
**Quartz**	**(2,2,-9)**	**11.491**	**84.5**	**10.686**	**9.2**	**1.111**	**10.19**	**13.88**	**17.22**
**Quartz**	**(2,2,9)**	**11.491**	**84.5**	**10.683**	**9.2**	**1.111**	**10.19**	**13.88**	**17.22**
**Quartz**	**(2,-4,-9)**	**11.491**	**84.5**	**10.681**	**9.2**	**1.111**	**10.19**	**13.88**	**17.22**
**LiNbO3**	**(3,6,0)**	**11.037**	**72.95**	**10.568**	**9.5**	**3.54**	**33.61**	**44.25**	**55.57**
	Equiv. Refl.: (6,-9,0), (9,-6,0), (-3,9,0), (-6,-3,0), (-9,3,0)								

Ei =	11.544	keV						ΔEg	ΔEt
Cryst	Refl	EB	ΘB	∫ IR dΘ	Width	Ei cotΘ	ΔE	2m, 50µm	2m, 50µm
	(h,k,l)	[keV]	[°]	[µrad]	[µrad]	[meV/µrad]	[meV]	[meV]	[meV]
LiNbO3	(4,-9,-2)	10.897	70.72	10.563	9.7	4.037	39.18	50.47	63.89
	Equiv. Refl.: (5,4,-2), (9,-4,-2), (-4,-5,-2), (-5,9,-2), (-9,5,-2)								
LiNbO3	(0,-5,20)	11.328	78.9	10.522	10.8	2.265	24.58	28.31	37.49
	Equiv. Refl.: (5,0,20), (-5,5,20)								
LiNbO3	(1,-7,14)	11.06	73.36	10.45	9.8	3.451	33.75	43.14	54.77
	Equiv. Refl.: (6,1,14), (7,-1,14), (-1,-6,14), (-6,7,14), (-7,6,14)								
LiNbO3	(3,-9,0)	11.037	72.95	10.346	9.5	3.54	33.61	44.25	55.57
	Equiv. Refl.: (6,3,0), (9,-3,0), (-3,-6,0), (-6,9,0), (-9,6,0)								
LiNbO3	(3,-7,16)	11.078	73.67	10.271	9.2	3.382	31.09	42.28	52.48
	Equiv. Refl.: (4,3,16), (7,-3,16), (-3,-4,16), (-4,7,16), (-7,4,16)								
LiNbO3	(4,4,12)	11.027	72.79	10.182	9.1	3.575	32.47	44.69	55.24
	Equiv. Refl.: (4,-8,12), (8,-4,12), (-4,8,12), (-4,-4,12), (-8,4,12)								
LiNbO3	(4,-9,4)	11.006	72.45	10.152	9	3.652	32.77	45.65	56.19
	Equiv. Refl.: (5,4,4), (9,-4,4), (-4,-5,4), (-5,9,4), (-9,5,4)								
Sapph	(0,4,20)	11.284	77.82	9.803	7.9	2.491	19.67	31.14	36.83
	Equiv. Refl.: (0,-4,-20), (4,0,-20), (4,-4,20), (-4,0,20), (-4,4,-20)								
LiNbO3	(3,4,-16)	11.078	73.67	9.71	9.2	3.382	31.09	42.28	52.48
	Equiv. Refl.: (4,-7,-16), (7,-4,-16), (-3,7,-16), (-4,-3,-16), (-7,3,-16)								
LiNbO3	(4,4,-12)	11.027	72.79	9.695	9.1	3.575	32.47	44.69	55.24
	Equiv. Refl.: (-4,8,-12), (-4,-4,-12), (-8,4,-12)								
LiNbO3	(4,-8,-12)	11.027	72.79	9.694	9.1	3.575	32.47	44.69	55.24
	Equiv. Refl.: (8,-4,-12)								
LiNbO3	(2,-8,10)	10.979	72	9.664	8.7	3.751	32.67	46.89	57.15
	Equiv. Refl.: (6,2,10), (8,-2,10), (-2,-6,10), (-6,8,10), (-8,6,10)								
LiNbO3	(4,5,-4)	11.006	72.45	9.501	9	3.652	32.77	45.65	56.19
	Equiv. Refl.: (5,-9,-4), (9,-5,-4), (-4,9,-4), (-5,-4,-4), (-9,4,-4)								
LiNbO3	(1,-4,-22)	11.042	73.04	9.487	8.7	3.521	30.5	44.02	53.55
	Equiv. Refl.: (3,1,-22), (4,-1,-22), (-1,-3,-22), (-3,4,-22), (-4,3,-22)								
LiNbO3	(2,6,-10)	10.979	72	9.38	8.7	3.751	32.67	46.89	57.15
	Equiv. Refl.: (6,-8,-10), (8,-6,-10), (-2,8,-10), (-6,-2,-10), (-8,2,-10)								
LiNbO3	(1,3,22)	11.042	73.04	9.238	8.7	3.521	30.5	44.02	53.55
	Equiv. Refl.: (3,-4,22), (4,-3,22), (-1,4,22), (-3,-1,22), (-4,1,22)								
Quartz	(-5,9,-1)	11.437	82.2	8.28	6.8	1.582	10.81	19.78	22.54
	Equiv. Refl.: (-5,-4,1)								
Quartz	(-4,9,1)	11.437	82.2	8.279	6.8	1.582	10.81	19.78	22.54
	Equiv. Refl.: (-4,-5,-1)								
Quartz	(9,-4,-1)	11.437	82.2	8.278	6.8	1.582	10.81	19.78	22.54
	Equiv. Refl.: (9,-5,1)								
Quartz	(5,4,-1)	11.437	82.2	8.082	6.8	1.582	10.81	19.78	22.54
	Equiv. Refl.: (5,-9,1)								
Quartz	(4,5,1)	11.437	82.2	8.081	6.8	1.582	10.81	19.78	22.54
	Equiv. Refl.: (4,-9,-1)								
Quartz	(-9,4,1)	11.437	82.2	8.08	6.8	1.582	10.81	19.78	22.54
	Equiv. Refl.: (-9,5,-1)								
Quartz	(6,-8,-4)	11.465	83.28	8.036	6.8	1.361	9.19	17.02	19.34
Quartz	(6,2,4)	11.465	83.28	8.034	6.8	1.361	9.19	17.02	19.34
	Equiv. Refl.: (-8,2,-4), (-8,6,4)								
Quartz	(2,6,-4)	11.465	83.28	8.031	6.8	1.361	9.19	17.02	19.34
	Equiv. Refl.: (2,-8,4)								
Quartz	(-6,8,4)	11.465	83.28	7.708	6.8	1.361	9.19	17.02	19.34

Ei =	11.544	keV						ΔEg	ΔEt
Cryst	Refl	EB	ΘB	∫ IR dΘ	Width	Ei cotΘ	ΔE	2m, 50µm	2m, 50µm
	(h,k,l)	[keV]	[°]	[µrad]	[µrad]	[meV/µrad]	[meV]	[meV]	[meV]
**Quartz**	**(8,-2,4)**	**11.465**	**83.28**	**7.706**	**6.8**	**1.361**	**9.19**	**17.02**	**19.34**
	Equiv. Refl.: (8,-6,-4), (-6,-2,-4)								
**Quartz**	**(-2,8,-4)**	**11.465**	**83.28**	**7.704**	**6.8**	**1.361**	**9.19**	**17.02**	**19.34**
	Equiv. Refl.: (-2,-6,4)								
**LiNbO3**	**(0,-8,2)**	**11.16**	**75.18**	**6.723**	**6.6**	**3.055**	**20.12**	**38.19**	**43.16**
	Equiv. Refl.: (8,0,2), (-8,8,2)								
**Sapph**	**(1,5,-16)**	**11.335**	**79.08**	**6.719**	**5.8**	**2.227**	**12.87**	**27.84**	**30.67**
	Equiv. Refl.: (1,-6,16), (5,1,16), (5,-6,-16), (6,-1,16), (6,-5,-16), (-1,6,-16), (-1,-5,16), (-5,6,16), (-5,-1,-16) (-6,1,-16), (-6,5,16)								
**Sapph**	**(0,7,8)**	**11.202**	**76.01**	**6.632**	**5.6**	**2.875**	**16.02**	**35.94**	**39.35**
	Equiv. Refl.: (0,-7,-8), (7,0,-8), (7,-7,8), (-7,0,8), (-7,7,-8)								
**Sapph**	**(1,6,10)**	**10.959**	**71.68**	**6.438**	**5.2**	**3.822**	**19.91**	**47.77**	**51.75**
	Equiv. Refl.: (1,-7,-10), (6,1,-10), (6,-7,10), (7,-1,-10), (7,-6,10), (-1,7,10), (-1,-6,-10), (-6,7,-10), (-6,-1,10) (-7,1,10), (-7,6,-10)								
**Sapph**	**(2,6,-4)**	**11.015**	**72.59**	**6.413**	**5.2**	**3.62**	**18.97**	**45.24**	**49.06**
	Equiv. Refl.: (2,-8,4), (6,2,4), (6,-8,-4), (8,-2,4), (8,-6,-4), (-2,8,-4), (-2,-6,4), (-6,8,4), (-6,-2,-4) (-8,2,-4), (-8,6,4)								
**Quartz**	**(-1,6,-7)**	**11.414**	**81.39**	**6.388**	**5.5**	**1.749**	**9.58**	**21.86**	**23.87**
**Quartz**	**(6,-1,7)**	**11.414**	**81.39**	**6.387**	**5.5**	**1.749**	**9.58**	**21.86**	**23.87**
**Quartz**	**(6,-5,-7)**	**11.414**	**81.39**	**6.386**	**5.5**	**1.749**	**9.58**	**21.86**	**23.87**
	Equiv. Refl.: (-1,-5,7)								
**Quartz**	**(-5,6,7)**	**11.414**	**81.39**	**6.38**	**5.5**	**1.749**	**9.57**	**21.86**	**23.86**
	Equiv. Refl.: (-5,-1,-7)								
**Quartz**	**(1,-6,7)**	**11.414**	**81.39**	**6.35**	**5.5**	**1.749**	**9.58**	**21.86**	**23.87**
**Quartz**	**(1,5,-7)**	**11.414**	**81.39**	**6.349**	**5.5**	**1.749**	**9.58**	**21.86**	**23.87**
	Equiv. Refl.: (-6,1,-7)								
**Quartz**	**(-6,5,7)**	**11.414**	**81.39**	**6.348**	**5.5**	**1.749**	**9.58**	**21.86**	**23.87**
**Quartz**	**(5,1,7)**	**11.414**	**81.39**	**6.342**	**5.5**	**1.749**	**9.57**	**21.86**	**23.86**
	Equiv. Refl.: (5,-6,-7)								
**LiNbO3**	**(0,8,-2)**	**11.16**	**75.18**	**6.315**	**6.6**	**3.055**	**20.12**	**38.19**	**43.16**
	Equiv. Refl.: (-8,0,-2)								
**LiNbO3**	**(8,-8,-2)**	**11.16**	**75.18**	**6.314**	**6.6**	**3.055**	**20.12**	**38.19**	**43.16**
**Sapph**	**(1,7,0)**	**11.358**	**79.7**	**6.284**	**5.4**	**2.097**	**11.41**	**26.21**	**28.59**
	Equiv. Refl.: (1,-8,0), (7,1,0), (7,-8,0), (8,-1,0), (8,-7,0), (-1,8,0), (-1,-7,0), (-7,8,0), (-7,-1,0) (-8,1,0), (-8,7,0)								
**Quartz**	**(7,0,-4)**	**11.183**	**75.64**	**6.227**	**5**	**2.955**	**14.71**	**36.94**	**39.76**
	Equiv. Refl.: (7,-7,4)								
**Quartz**	**(-7,0,4)**	**11.183**	**75.64**	**6.226**	**5**	**2.955**	**14.71**	**36.94**	**39.76**
	Equiv. Refl.: (-7,7,-4)								
**Quartz**	**(0,7,4)**	**11.183**	**75.64**	**6.223**	**5**	**2.955**	**14.71**	**36.94**	**39.76**
	Equiv. Refl.: (0,-7,-4)								
**Sapph**	**(1,3,-20)**	**10.979**	**72.01**	**6.14**	**5**	**3.75**	**18.78**	**46.87**	**50.49**
	Equiv. Refl.: (1,-4,20), (3,1,20), (3,-4,-20), (4,-1,20), (4,-3,-20), (-1,4,-20), (-1,-3,20), (-3,4,20), (-3,-1,-20) (-4,1,-20), (-4,3,20)								
**Quartz**	**(-5,1,8)**	**11.348**	**79.43**	**5.397**	**4.4**	**2.154**	**9.41**	**26.92**	**28.52**
	Equiv. Refl.: (-5,4,-8)								
**Quartz**	**(1,-5,-8)**	**11.348**	**79.43**	**5.396**	**4.4**	**2.154**	**9.41**	**26.92**	**28.52**
**Quartz**	**(1,4,8)**	**11.348**	**79.43**	**5.395**	**4.4**	**2.154**	**9.4**	**26.92**	**28.52**
**Quartz**	**(0,4,8)**	**10.87**	**70.33**	**5.394**	**4.3**	**4.126**	**17.91**	**51.58**	**54.6**
	Equiv. Refl.: (0,-4,-8)								

Ei =	11.544	keV						ΔEg	ΔEt
**Cryst**	**Refl**	**EB**	**ΘB**	**∫ IR dΘ**	**Width**	**Ei cotΘ**	**ΔE**	**2m, 50µm**	**2m, 50µm**
	**(h,k,l)**	**[keV]**	**[°]**	**[µrad]**	**[µrad]**	**[meV/µrad]**	**[meV]**	**[meV]**	**[meV]**
**Quartz**	**(4,0,-8)**	**10.87**	**70.33**	**5.392**	**4.3**	**4.126**	**17.9**	**51.58**	**54.6**
	Equiv. Refl.: (-4,0,8)								
**Quartz**	**(4,-4,8)**	**10.87**	**70.33**	**5.391**	**4.3**	**4.126**	**17.9**	**51.58**	**54.6**
	Equiv. Refl.: (-4,4,-8)								
**Quartz**	**(4,1,-8)**	**11.348**	**79.43**	**5.39**	**4.4**	**2.154**	**9.4**	**26.92**	**28.52**
	Equiv. Refl.: (4,-5,8)								
**Quartz**	**(0,6,-6)**	**11.126**	**74.53**	**5.287**	**4.3**	**3.195**	**13.88**	**39.93**	**42.27**
	Equiv. Refl.: (0,-6,6)								
**Quartz**	**(6,0,6)**	**11.126**	**74.53**	**5.281**	**4.3**	**3.195**	**13.86**	**39.93**	**42.27**
	Equiv. Refl.: (-6,0,-6)								
**Quartz**	**(6,-6,-6)**	**11.126**	**74.53**	**5.28**	**4.3**	**3.195**	**13.86**	**39.93**	**42.27**
	Equiv. Refl.: (-6,6,6)								
**Quartz**	**(4,5,-1)**	**11.437**	**82.2**	**5.225**	**4.5**	**1.582**	**7.17**	**19.78**	**21.04**
	Equiv. Refl.: (5,4,1), (5,-9,-1)								
**Quartz**	**(4,-9,1)**	**11.437**	**82.2**	**5.224**	**4.5**	**1.582**	**7.17**	**19.78**	**21.04**
	Equiv. Refl.: (-9,4,-1), (-9,5,1)								
**Quartz**	**(5,-4,8)**	**11.348**	**79.43**	**5.222**	**4.4**	**2.154**	**9.41**	**26.92**	**28.52**
**Quartz**	**(5,-1,-8)**	**11.348**	**79.43**	**5.221**	**4.4**	**2.154**	**9.41**	**26.92**	**28.52**
**Quartz**	**(-1,5,8)**	**11.348**	**79.43**	**5.22**	**4.4**	**2.154**	**9.41**	**26.92**	**28.52**
**Quartz**	**(-1,-4,-8)**	**11.348**	**79.43**	**5.219**	**4.4**	**2.154**	**9.4**	**26.92**	**28.52**
**Quartz**	**(-4,5,-8)**	**11.348**	**79.43**	**5.215**	**4.4**	**2.154**	**9.4**	**26.92**	**28.52**
	Equiv. Refl.: (-4,-1,8)								
**Quartz**	**(9,-4,1)**	**11.437**	**82.2**	**5.171**	**4.5**	**1.582**	**7.17**	**19.78**	**21.03**
	Equiv. Refl.: (9,-5,-1), (-4,9,-1), (-4,-5,1), (-5,9,1), (-5,-4,-1)								
**Quartz**	**(-4,-3,6)**	**11.221**	**76.41**	**4.659**	**3.8**	**2.79**	**10.52**	**34.88**	**36.43**
**Quartz**	**(-4,7,-6)**	**11.221**	**76.41**	**4.658**	**3.8**	**2.79**	**10.52**	**34.88**	**36.43**
**Quartz**	**(-3,7,6)**	**11.221**	**76.41**	**4.655**	**3.8**	**2.79**	**10.52**	**34.88**	**36.43**
	Equiv. Refl.: (-3,-4,-6)								
**Quartz**	**(7,-3,-6)**	**11.221**	**76.41**	**4.654**	**3.8**	**2.79**	**10.51**	**34.88**	**36.43**
	Equiv. Refl.: (7,-4,6)								
**Sapph**	**(2,2,21)**	**11.297**	**78.13**	**4.62**	**4**	**2.427**	**9.76**	**30.34**	**31.87**
	Equiv. Refl.: (2,2,-21), (2,-4,21), (2,-4,-21), (4,-2,21), (4,-2,-21), (-2,4,21), (-2,4,-21), (-2,-2,21), (-2,-2,-21) (-4,2,21), (-4,2,-21)								
**Quartz**	**(4,3,-6)**	**11.221**	**76.41**	**4.571**	**3.8**	**2.79**	**10.52**	**34.88**	**36.43**
	Equiv. Refl.: (4,-7,6)								
**Quartz**	**(3,4,6)**	**11.221**	**76.41**	**4.568**	**3.8**	**2.79**	**10.52**	**34.88**	**36.43**
**Quartz**	**(3,-7,-6)**	**11.221**	**76.41**	**4.567**	**3.8**	**2.79**	**10.52**	**34.88**	**36.43**
	Equiv. Refl.: (-7,3,6), (-7,4,-6)								
**Quartz**	**(2,4,-7)**	**11.131**	**74.63**	**4.31**	**3.5**	**3.173**	**10.97**	**39.66**	**41.15**
	Equiv. Refl.: (2,-6,7)								
**Quartz**	**(4,-6,-7)**	**11.131**	**74.63**	**4.309**	**3.5**	**3.173**	**10.96**	**39.66**	**41.14**
	Equiv. Refl.: (-6,2,-7), (-6,4,7)								
**Quartz**	**(4,2,7)**	**11.131**	**74.63**	**4.308**	**3.5**	**3.173**	**10.96**	**39.66**	**41.14**
**Quartz**	**(6,-4,-7)**	**11.131**	**74.63**	**4.209**	**3.5**	**3.173**	**10.96**	**39.66**	**41.14**
	Equiv. Refl.: (-2,6,-7), (-2,-4,7)								
**Quartz**	**(6,-2,7)**	**11.131**	**74.63**	**4.208**	**3.5**	**3.173**	**10.96**	**39.66**	**41.14**
	Equiv. Refl.: (-4,6,7)								
**Quartz**	**(-4,-2,-7)**	**11.131**	**74.63**	**4.207**	**3.5**	**3.173**	**10.96**	**39.66**	**41.14**
**Quartz**	**(8,-1,0)**	**11**	**72.34**	**4.165**	**3.3**	**3.675**	**12.08**	**45.94**	**47.5**
	Equiv. Refl.: (8,-7,0), (-1,8,0), (-1,-7,0), (-7,8,0), (-7,-1,0)								

Ei =	11.544	keV						ΔEg	ΔEt
Cryst	Refl	EB	ΘB	∫ IR dΘ	Width	Ei cotΘ	ΔE	2m, 50µm	2m, 50µm
	(h,k,l)	[keV]	[°]	[µrad]	[µrad]	[meV/µrad]	[meV]	[meV]	[meV]
**Quartz**	**(1,7,0)**	**11**	**72.34**	**4.09**	**3.3**	**3.675**	**12.08**	**45.94**	**47.5**
	Equiv. Refl.: (1,-8,0), (7,1,0), (7,-8,0), (-8,1,0), (-8,7,0)								
**Sapph**	**(1,7,3)**	**11.448**	**82.61**	**4.051**	**3.8**	**1.498**	**5.76**	**18.72**	**19.59**
	Equiv. Refl.: (1,7,-3), (1,-8,3), (1,-8,-3), (7,1,3), (7,1,-3), (7,-8,3), (7,-8,-3), (8,-1,3), (8,-1,-3) (8,-7,3), (8,-7,-3), (-1,8,3), (-1,8,-3), (-1,-7,3), (-1,-7,-3), (-7,8,3), (-7,8,-3), (-7,-1,3) (-7,-1,-3), (-8,1,3), (-8,1,-3), (-8,7,3), (-8,7,-3)								
**Sapph**	**(2,6,-7)**	**11.351**	**79.52**	**3.864**	**3.6**	**2.136**	**7.59**	**26.7**	**27.76**
	Equiv. Refl.: (2,-8,7), (6,2,7), (6,-8,-7), (8,-2,7), (8,-6,-7), (-2,8,-7), (-2,-6,7), (-6,8,7), (-6,-2,-7) (-8,2,-7), (-8,6,7)								
**Quartz**	**(2,6,-3)**	**11.056**	**73.27**	**3.725**	**3**	**3.469**	**10.56**	**43.36**	**44.63**
	Equiv. Refl.: (2,-8,3)								
**Quartz**	**(-8,2,-3)**	**11.056**	**73.27**	**3.724**	**3**	**3.469**	**10.56**	**43.36**	**44.63**
**Quartz**	**(-8,6,3)**	**11.056**	**73.27**	**3.723**	**3**	**3.469**	**10.56**	**43.36**	**44.63**
**Quartz**	**(6,2,3)**	**11.056**	**73.27**	**3.722**	**3**	**3.469**	**10.56**	**43.36**	**44.63**
	Equiv. Refl.: (6,-8,-3)								
**Quartz**	**(-2,8,-3)**	**11.056**	**73.27**	**3.704**	**3**	**3.469**	**10.56**	**43.36**	**44.63**
	Equiv. Refl.: (-2,-6,3)								
**Quartz**	**(8,-2,3)**	**11.056**	**73.27**	**3.703**	**3**	**3.469**	**10.56**	**43.36**	**44.63**
**Quartz**	**(8,-6,-3)**	**11.056**	**73.27**	**3.702**	**3**	**3.469**	**10.56**	**43.36**	**44.63**
**Quartz**	**(-6,8,3)**	**11.056**	**73.27**	**3.701**	**3**	**3.469**	**10.56**	**43.36**	**44.63**
	Equiv. Refl.: (-6,-2,-3)								
**Quartz**	**(1,-7,-5)**	**11.143**	**74.86**	**3.602**	**2.9**	**3.124**	**9**	**39.05**	**40.07**
**Quartz**	**(1,6,5)**	**11.143**	**74.86**	**3.601**	**2.9**	**3.124**	**8.99**	**39.05**	**40.07**
**Quartz**	**(-7,6,-5)**	**11.143**	**74.86**	**3.6**	**2.9**	**3.124**	**8.99**	**39.05**	**40.07**
**Quartz**	**(6,1,-5)**	**11.143**	**74.86**	**3.599**	**2.9**	**3.124**	**8.99**	**39.05**	**40.07**
	Equiv. Refl.: (6,-7,5), (-7,1,5)								
**Sapph**	**(2,4,16)**	**11.031**	**72.86**	**3.559**	**3.1**	**3.56**	**10.87**	**44.5**	**45.81**
	Equiv. Refl.: (2,-6,-16), (4,2,-16), (4,-6,16), (6,-2,-16), (6,-4,16), (-2,6,16), (-2,-4,-16), (-4,6,-16), (-4,-2,16) (-6,2,16), (-6,4,-16)								
**Quartz**	**(4,-8,4)**	**11.088**	**73.84**	**3.467**	**2.9**	**3.344**	**9.54**	**41.8**	**42.88**
**Quartz**	**(4,4,-4)**	**11.088**	**73.84**	**3.466**	**2.9**	**3.344**	**9.54**	**41.8**	**42.88**
	Equiv. Refl.: (-8,4,4), (-8,4,-4)								
**Quartz**	**(4,4,4)**	**11.088**	**73.84**	**3.464**	**2.9**	**3.344**	**9.54**	**41.8**	**42.88**
	Equiv. Refl.: (4,-8,-4)								
**Quartz**	**(-4,8,-4)**	**11.088**	**73.84**	**3.395**	**2.9**	**3.344**	**9.54**	**41.8**	**42.88**
**Quartz**	**(8,-4,4)**	**11.088**	**73.84**	**3.394**	**2.9**	**3.344**	**9.54**	**41.8**	**42.88**
	Equiv. Refl.: (8,-4,-4), (-4,-4,4)								
**Quartz**	**(-4,8,4)**	**11.088**	**73.84**	**3.393**	**2.9**	**3.344**	**9.54**	**41.8**	**42.88**
**Quartz**	**(-4,-4,-4)**	**11.088**	**73.84**	**3.392**	**2.9**	**3.344**	**9.54**	**41.8**	**42.88**
**Quartz**	**(5,2,-6)**	**11.408**	**81.21**	**3.293**	**2.9**	**1.785**	**5.22**	**22.31**	**22.91**
	Equiv. Refl.: (5,-7,6)								
**Quartz**	**(-7,2,6)**	**11.408**	**81.21**	**3.291**	**2.9**	**1.785**	**5.22**	**22.31**	**22.91**
**Quartz**	**(-7,5,-6)**	**11.408**	**81.21**	**3.29**	**2.9**	**1.785**	**5.22**	**22.31**	**22.91**
**Quartz**	**(2,-7,-6)**	**11.408**	**81.21**	**3.289**	**2.9**	**1.785**	**5.22**	**22.31**	**22.91**
**Quartz**	**(2,5,6)**	**11.408**	**81.21**	**3.288**	**2.9**	**1.785**	**5.21**	**22.31**	**22.91**
**Quartz**	**(-1,7,5)**	**11.143**	**74.86**	**3.25**	**2.9**	**3.124**	**9**	**39.05**	**40.07**
	Equiv. Refl.: (-1,-6,-5)								
**Quartz**	**(7,-6,5)**	**11.143**	**74.86**	**3.249**	**2.9**	**3.124**	**8.99**	**39.05**	**40.07**
**Quartz**	**(7,-1,-5)**	**11.143**	**74.86**	**3.248**	**2.9**	**3.124**	**8.99**	**39.05**	**40.07**
	Equiv. Refl.: (-6,7,-5), (-6,-1,5)								

Ei =	11.544	keV						ΔEg	ΔEt
Cryst	Refl	EB	ΘB	∫ IR dΘ	Width	Ei cotΘ	ΔE	2m, 50µm	2m, 50µm
	(h,k,l)	[keV]	[°]	[µrad]	[µrad]	[meV/µrad]	[meV]	[meV]	[meV]
Quartz	(-3,5,8)	11.16	75.17	3.221	2.9	3.056	8.76	38.2	39.2
	Equiv. Refl.: (-3,-2,-8)								
Quartz	(5,-3,-8)	11.16	75.17	3.22	2.9	3.056	8.76	38.2	39.2
	Equiv. Refl.: (-2,5,-8)								
Quartz	(5,-2,8)	11.16	75.17	3.219	2.9	3.056	8.76	38.2	39.19
	Equiv. Refl.: (-2,-3,8)								
Quartz	(-5,-2,6)	11.408	81.21	3.207	2.9	1.785	5.22	22.31	22.91
Quartz	(-5,7,-6)	11.408	81.21	3.206	2.9	1.785	5.22	22.31	22.91
Quartz	(7,-2,-6)	11.408	81.21	3.204	2.9	1.785	5.22	22.31	22.91
	Equiv. Refl.: (7,-5,6)								
Quartz	(-2,7,6)	11.408	81.21	3.202	2.9	1.785	5.22	22.31	22.91
	Equiv. Refl.: (-2,-5,-6)								
Quartz	(8,-1,-1)	11.06	73.34	3.163	2.5	3.454	8.73	43.17	44.04
	Equiv. Refl.: (8,-7,1), (-1,8,1), (-1,-7,-1), (-7,8,-1), (-7,-1,1)								
Sapph	(4,4,9)	11.273	77.57	3.143	2.8	2.544	7.17	31.81	32.6
	Equiv. Refl.: (4,4,-9), (4,-8,9), (4,-8,-9), (8,-4,9), (8,-4,-9), (-4,8,9), (-4,8,-9), (-4,-4,9), (-4,-4,-9) (-8,4,9), (-8,4,-9)								
Quartz	(1,7,1)	11.06	73.34	3.111	2.5	3.454	8.73	43.17	44.04
	Equiv. Refl.: (1,-8,-1), (7,1,-1), (7,-8,1), (-8,1,1), (-8,7,-1)								
Quartz	(3,5,-4)	11.183	75.64	3.057	2.5	2.955	7.44	36.94	37.68
	Equiv. Refl.: (3,-8,4), (5,3,4), (5,-8,-4), (-8,3,-4)								
Quartz	(-8,5,4)	11.183	75.64	3.056	2.5	2.955	7.44	36.94	37.68
Quartz	(3,0,9)	11.21	76.19	3.054	2.5	2.837	7.23	35.46	36.19
	Equiv. Refl.: (3,-3,-9), (-3,0,-9), (-3,3,9)								
Quartz	(2,-5,8)	11.16	75.17	3.047	2.9	3.056	8.76	38.2	39.2
	Equiv. Refl.: (3,2,8), (3,-5,-8)								
Quartz	(2,3,-8)	11.16	75.17	3.046	2.9	3.056	8.76	38.2	39.19
	Equiv. Refl.: (-5,2,-8), (-5,3,8)								
Quartz	(0,-3,9)	11.21	76.19	3.045	2.5	2.837	7.21	35.46	36.19
Quartz	(0,3,-9)	11.21	76.19	3.044	2.5	2.837	7.21	35.46	36.19
Quartz	(8,-3,4)	11.183	75.64	3.017	2.5	2.955	7.44	36.94	37.68
	Equiv. Refl.: (-3,8,-4), (-3,-5,4), (-5,8,4), (-5,-3,-4)								
Quartz	(8,-5,-4)	11.183	75.64	3.016	2.5	2.955	7.44	36.94	37.68
Quartz	(-1,5,-8)	11.348	79.43	2.957	2.6	2.154	5.57	26.92	27.49
	Equiv. Refl.: (-1,-4,8)								
Quartz	(5,-1,8)	11.348	79.43	2.956	2.6	2.154	5.56	26.92	27.49
	Equiv. Refl.: (5,-4,-8)								
Quartz	(-4,5,8)	11.348	79.43	2.955	2.6	2.154	5.56	26.92	27.49
	Equiv. Refl.: (-4,-1,-8)								
Quartz	(1,4,-8)	11.348	79.43	2.887	2.6	2.154	5.56	26.92	27.49
	Equiv. Refl.: (1,-5,8)								
Quartz	(-5,1,-8)	11.348	79.43	2.886	2.6	2.154	5.56	26.92	27.49
	Equiv. Refl.: (-5,4,8)								
Quartz	(4,1,8)	11.348	79.43	2.885	2.6	2.154	5.56	26.92	27.49
	Equiv. Refl.: (4,-5,-8)								
LiNbO3	(2,7,1)	11.39	80.64	2.874	4.6	1.902	8.72	23.78	25.33
	Equiv. Refl.: (2,-9,-1), (7,2,-1), (7,-9,1), (9,-2,-1), (9,-7,1), (-2,9,1), (-2,-7,-1), (-7,9,-1), (-7,-2,1) (-9,2,1), (-9,7,-1)								
Sapph	(3,4,14)	11.331	78.98	2.63	2.7	2.249	6.09	28.11	28.76
	Equiv. Refl.: (3,-7,-14), (4,3,-14), (4,-7,14), (7,-3,-14), (7,-4,14), (-3,7,14), (-3,-4,-14), (-4,7,-14), (-4,-3,14) (-7,3,14), (-7,4,-14)								

Ei =	11.544	keV						ΔEg	ΔEt
Cryst	Refl	EB	ΘB	∫ IR dΘ	Width	Ei cotΘ	ΔE	2m, 50µm	2m, 50µm
	(h,k,l)	[keV]	[°]	[µrad]	[µrad]	[meV/µrad]	[meV]	[meV]	[meV]
Sapph	(2,6,5)	11.108	74.2	2.589	2.5	3.266	8.2	40.83	41.65
	Equiv. Refl.: (2,-8,-5), (6,2,-5), (6,-8,5), (8,-2,-5), (8,-6,5), (-2,8,5), (-2,-6,-5), (-6,8,-5), (-6,-2,5) (-8,2,5), (-8,6,-5)								
Quartz	(-2,8,3)	11.056	73.27	2.509	2.2	3.469	7.65	43.36	44.03
	Equiv. Refl.: (-2,-6,-3), (-6,8,-3)								
Quartz	(8,-2,-3)	11.056	73.27	2.508	2.2	3.469	7.64	43.36	44.03
	Equiv. Refl.: (8,-6,3), (-6,-2,3)								
Sapph	(2,4,-17)	11.367	79.95	1.999	2.4	2.045	4.97	25.57	26.04
	Equiv. Refl.: (2,-6,17), (4,2,17), (4,-6,-17), (6,-2,17), (6,-4,-17), (-2,6,-17), (-2,-4,17), (-4,6,17), (-4,-2,-17) (-6,2,-17), (-6,4,17)								
Quartz	(2,6,3)	11.056	73.27	1.951	2.2	3.469	7.65	43.36	44.03
	Equiv. Refl.: (2,-8,-3), (6,-8,3)								
Quartz	(6,2,-3)	11.056	73.27	1.95	2.2	3.469	7.64	43.36	44.03
	Equiv. Refl.: (-8,2,3), (-8,6,-3)								
Sapph	(2,6,-1)	10.859	70.16	1.935	2.1	4.164	8.9	52.06	52.81
	Equiv. Refl.: (2,-8,1), (6,2,1), (6,-8,-1), (8,-2,1), (8,-6,-1), (-2,8,-1), (-2,-6,1), (-6,8,1), (-6,-2,-1) (-8,2,-1), (-8,6,1)								
Quartz	(8,-1,1)	11.06	73.34	1.93	2	3.454	7.02	43.17	43.74
	Equiv. Refl.: (8,-7,-1), (-1,8,-1), (-1,-7,1), (-7,8,1), (-7,-1,-1)								
LiNbO3	(2,4,19)	11.239	76.81	1.875	3.1	2.706	8.37	33.82	34.84
	Equiv. Refl.: (2,-6,-19), (4,2,-19), (4,-6,19), (6,-2,-19), (6,-4,19), (-2,6,19), (-2,-4,-19), (-4,6,-19), (-4,-2,19) (-6,2,19), (-6,4,-19)								
LiNbO3	(1,3,-23)	11.442	82.37	1.851	3.7	1.545	5.72	19.32	20.15
	Equiv. Refl.: (1,-4,23), (3,1,23), (3,-4,-23), (4,-1,23), (4,-3,-23), (-1,4,-23), (-1,-3,23), (-3,4,23), (-3,-1,-23) (-4,1,-23), (-4,3,23)								
Quartz	(1,7,-1)	11.06	73.34	1.794	2	3.454	7.02	43.17	43.74
	Equiv. Refl.: (1,-8,1), (7,1,1), (7,-8,-1)								
Quartz	(-8,1,-1)	11.06	73.34	1.793	2	3.454	7.02	43.17	43.74
	Equiv. Refl.: (-8,7,1)								
LiNbO3	(1,5,-19)	11.495	84.7	1.749	4.1	1.072	4.43	13.4	14.11
	Equiv. Refl.: (1,-6,19), (5,1,19), (5,-6,-19), (6,-1,19), (6,-5,-19), (-1,6,-19), (-1,-5,19), (-5,6,19), (-5,-1,-19) (-6,1,-19), (-6,5,19)								
LiNbO3	(3,4,17)	11.372	80.1	1.414	2.8	2.014	5.74	25.17	25.82
	Equiv. Refl.: (3,-7,-17), (4,3,-17), (4,-7,17), (7,-3,-17), (7,-4,17), (-3,7,17), (-3,-4,-17), (-4,7,-17), (-4,-3,17) (-7,3,17), (-7,4,-17)								
Quartz	(3,4,-6)	11.221	76.41	1.225	1.4	2.79	3.81	34.88	35.09
Quartz	(3,-7,6)	11.221	76.41	1.224	1.4	2.79	3.81	34.88	35.09
	Equiv. Refl.: (4,3,6), (4,-7,-6)								
Quartz	(-7,3,-6)	11.221	76.41	1.222	1.4	2.79	3.8	34.88	35.09
	Equiv. Refl.: (-7,4,6)								
Quartz	(-6,7,5)	11.143	74.86	1.143	1.3	3.124	4.19	39.05	39.27
	Equiv. Refl.: (-6,-1,-5)								
Quartz	(7,-6,-5)	11.143	74.86	1.142	1.3	3.124	4.18	39.05	39.27
	Equiv. Refl.: (-1,7,-5), (-1,-6,5)								
Quartz	(7,-1,5)	11.143	74.86	1.141	1.3	3.124	4.18	39.05	39.27
LiNbO3	(1,4,21)	11.348	79.44	1.132	2.4	2.152	5.23	26.9	27.4
	Equiv. Refl.: (1,4,-21), (1,-5,21), (1,-5,-21), (4,1,21), (4,1,-21), (4,-5,21), (4,-5,-21), (5,-1,21), (5,-1,-21) (5,-4,21), (5,-4,-21), (-1,5,21), (-1,5,-21), (-1,-4,21), (-1,-4,-21), (-4,5,21), (-4,5,-21), (-4,-1,21) (-4,-1,-21), (-5,1,21), (-5,1,-21), (-5,4,21), (-5,4,-21)								

Ei =	11.544	keV						ΔEg	ΔEt
**Cryst**	**Refl**	**EB**	**ΘB**	**∫ IR dΘ**	**Width**	**Ei cotΘ**	**ΔE**	**2m, 50µm**	**2m, 50µm**
	**(h,k,l)**	**[keV]**	**[°]**	**[µrad]**	**[µrad]**	**[meV/µrad]**	**[meV]**	**[meV]**	**[meV]**
**Quartz**	**(8,-2,-4)**	**11.465**	**83.28**	**1.127**	**1.5**	**1.361**	**1.99**	**17.02**	**17.13**
	Equiv. Refl.: (8,-6,4), (-6,8,-4)								
**Quartz**	**(-6,-2,4)**	**11.465**	**83.28**	**1.126**	**1.5**	**1.361**	**1.99**	**17.02**	**17.13**
**Quartz**	**(-2,-6,-4)**	**11.465**	**83.28**	**1.124**	**1.5**	**1.361**	**1.98**	**17.02**	**17.13**
**Quartz**	**(-2,8,4)**	**11.465**	**83.28**	**1.123**	**1.5**	**1.361**	**1.98**	**17.02**	**17.13**
**Quartz**	**(8,-1,2)**	**11.237**	**76.75**	**1.112**	**1.3**	**2.718**	**3.62**	**33.98**	**34.17**
	Equiv. Refl.: (8,-7,-2), (-1,8,-2), (-1,-7,2), (-7,-1,-2)								
**Quartz**	**(-7,8,2)**	**11.237**	**76.75**	**1.111**	**1.3**	**2.718**	**3.62**	**33.98**	**34.17**
**Sapph**	**(2,3,-19)**	**11.191**	**75.8**	**1.108**	**1.4**	**2.922**	**4.16**	**36.52**	**36.76**
	Equiv. Refl.: (2,-5,19), (3,2,19), (3,-5,-19), (5,-2,19), (5,-3,-19), (-2,5,-19), (-2,-3,19), (-3,5,19), (-3,-2,-19) (-5,2,-19), (-5,3,19)								
**Quartz**	**(6,1,5)**	**11.143**	**74.86**	**1.1**	**1.3**	**3.124**	**4.19**	**39.05**	**39.27**
	Equiv. Refl.: (6,-7,-5)								
**Quartz**	**(1,6,-5)**	**11.143**	**74.86**	**1.099**	**1.3**	**3.124**	**4.19**	**39.05**	**39.27**
	Equiv. Refl.: (1,-7,5), (-7,6,5)								
**Quartz**	**(-7,1,-5)**	**11.143**	**74.86**	**1.098**	**1.3**	**3.124**	**4.18**	**39.05**	**39.27**
**Quartz**	**(-4,6,-7)**	**11.131**	**74.63**	**1.08**	**1.3**	**3.173**	**4**	**39.66**	**39.86**
	Equiv. Refl.: (-4,-2,7)								
**Quartz**	**(6,-2,-7)**	**11.131**	**74.63**	**1.079**	**1.3**	**3.173**	**4**	**39.66**	**39.86**
**Quartz**	**(6,-4,7)**	**11.131**	**74.63**	**1.078**	**1.3**	**3.173**	**4**	**39.66**	**39.86**
**Quartz**	**(-2,6,7)**	**11.131**	**74.63**	**1.077**	**1.3**	**3.173**	**3.99**	**39.66**	**39.86**
	Equiv. Refl.: (-2,-4,-7)								
**LiNbO3**	**(4,5,-7)**	**11.302**	**78.25**	**1.046**	**2.2**	**2.401**	**5.34**	**30.01**	**30.48**
	Equiv. Refl.: (4,-9,7), (5,4,7), (5,-9,-7), (9,-4,7), (9,-5,-7), (-4,9,-7), (-4,-5,7), (-5,9,7), (-5,-4,-7) (-9,4,-7), (-9,5,7)								
**Quartz**	**(1,7,-2)**	**11.237**	**76.75**	**1.045**	**1.3**	**2.718**	**3.62**	**33.98**	**34.17**
	Equiv. Refl.: (1,-8,2), (7,1,2), (7,-8,-2), (-8,1,-2), (-8,7,2)								
**Sapph**	**(3,5,7)**	**11.048**	**73.15**	**1.042**	**1.3**	**3.497**	**4.58**	**43.71**	**43.95**
	Equiv. Refl.: (3,-8,-7), (5,3,-7), (5,-8,7), (8,-3,-7), (8,-5,7), (-3,8,7), (-3,-5,-7), (-5,8,-7), (-5,-3,7) (-8,3,7), (-8,5,-7)								
**Quartz**	**(1,2,-9)**	**11.019**	**72.66**	**1.026**	**1.1**	**3.604**	**4.14**	**45.05**	**45.24**
	Equiv. Refl.: (1,-3,9), (2,-3,-9)								
**Quartz**	**(2,1,9)**	**11.019**	**72.66**	**1.025**	**1.1**	**3.604**	**4.13**	**45.05**	**45.24**
**Quartz**	**(-3,1,-9)**	**11.019**	**72.66**	**1.023**	**1.1**	**3.604**	**4.13**	**45.05**	**45.24**
	Equiv. Refl.: (-3,2,9)								
**Quartz**	**(-3,-4,6)**	**11.221**	**76.41**	**1.022**	**1.4**	**2.79**	**3.81**	**34.88**	**35.09**
**Quartz**	**(-3,7,-6)**	**11.221**	**76.41**	**1.021**	**1.4**	**2.79**	**3.81**	**34.88**	**35.09**
	Equiv. Refl.: (-4,7,6), (-4,-3,-6)								
**Quartz**	**(7,-3,6)**	**11.221**	**76.41**	**1.019**	**1.4**	**2.79**	**3.8**	**34.88**	**35.09**
	Equiv. Refl.: (7,-4,-6)								
**Quartz**	**(4,2,-7)**	**11.131**	**74.63**	**1.008**	**1.3**	**3.173**	**4**	**39.66**	**39.86**
	Equiv. Refl.: (4,-6,7)								
**Quartz**	**(-6,2,7)**	**11.131**	**74.63**	**1.007**	**1.3**	**3.173**	**4**	**39.66**	**39.86**
	Equiv. Refl.: (-6,4,-7)								
**Quartz**	**(2,4,7)**	**11.131**	**74.63**	**1.006**	**1.3**	**3.173**	**3.99**	**39.66**	**39.86**
	Equiv. Refl.: (2,-6,-7)								
**Quartz**	**(-1,3,-9)**	**11.019**	**72.66**	**0.884**	**1.1**	**3.604**	**4.14**	**45.05**	**45.24**
	Equiv. Refl.: (-1,-2,9)								
**Quartz**	**(-2,3,9)**	**11.019**	**72.66**	**0.883**	**1.1**	**3.604**	**4.14**	**45.05**	**45.24**
**Quartz**	**(-2,-1,-9)**	**11.019**	**72.66**	**0.882**	**1.1**	**3.604**	**4.13**	**45.05**	**45.24**

Ei =	11.544	keV						ΔEg	ΔEt
Cryst	Refl (h,k,l)	EB [keV]	ΘB [°]	∫ IR dΘ [µrad]	Width [µrad]	Ei cotΘ [meV/µrad]	ΔE [meV]	2m, 50µm [meV]	2m, 50µm [meV]
Quartz	(3,-1,9)	11.019	72.66	0.881	1.1	3.604	4.13	45.05	45.24
	Equiv. Refl.: (3,-2,-9)								
Quartz	(-8,2,4)	11.465	83.28	0.859	1.5	1.361	1.99	17.02	17.13
	Equiv. Refl.: (-8,6,-4)								
Quartz	(6,2,-4)	11.465	83.28	0.858	1.5	1.361	1.99	17.02	17.13
	Equiv. Refl.: (6,-8,4)								
Quartz	(2,6,4)	11.465	83.28	0.855	1.5	1.361	1.98	17.02	17.13
Quartz	(2,-8,-4)	11.465	83.28	0.854	1.5	1.361	1.98	17.02	17.13
Quartz	(0,7,-4)	11.183	75.64	0.834	1.1	2.955	3.16	36.94	37.07
	Equiv. Refl.: (0,-7,4)								
Quartz	(7,0,4)	11.183	75.64	0.831	1.1	2.955	3.16	36.94	37.07
	Equiv. Refl.: (7,-7,-4), (-7,0,-4), (-7,7,4)								
Quartz	(7,-2,6)	11.408	81.21	0.817	1.1	1.785	2.01	22.31	22.4
	Equiv. Refl.: (7,-5,-6)								
Quartz	(-5,-2,-6)	11.408	81.21	0.815	1.1	1.785	2	22.31	22.4
Quartz	(-2,7,-6)	11.408	81.21	0.814	1.1	1.785	2	22.31	22.4
	Equiv. Refl.: (-2,-5,6), (-5,7,6)								
LiNbO3	(4,5,5)	11.088	73.84	0.811	1.7	3.345	5.68	41.82	42.2
	Equiv. Refl.: (4,-9,-5), (5,4,-5), (5,-9,5), (9,-4,-5), (9,-5,5), (-4,9,5), (-4,-5,-5), (-5,9,-5), (-5,-4,5) (-9,4,5), (-9,5,-5)								
Quartz	(8,-3,-4)	11.183	75.64	0.803	1	2.955	2.93	36.94	37.06
	Equiv. Refl.: (8,-5,4), (-3,8,4), (-3,-5,-4), (-5,8,-4)								
Quartz	(-5,-3,4)	11.183	75.64	0.802	1	2.955	2.93	36.94	37.06
Quartz	(-1,6,7)	11.414	81.39	0.755	1	1.749	1.77	21.86	21.93
	Equiv. Refl.: (-1,-5,-7), (-5,6,-7)								
Quartz	(-5,-1,7)	11.414	81.39	0.753	1	1.749	1.77	21.86	21.93
Quartz	(6,-1,-7)	11.414	81.39	0.752	1	1.749	1.76	21.86	21.93
	Equiv. Refl.: (6,-5,7)								
Sapph	(1,6,-11)	11.175	75.48	0.733	1	2.99	3.1	37.38	37.5
	Equiv. Refl.: (1,-7,11), (6,1,11), (6,-7,-11), (7,-1,11), (7,-6,-11), (-1,7,-11), (-1,-6,11), (-6,7,11), (-6,-1,-11) (-7,1,-11), (-7,6,11)								
LiNbO3	(4,5,-1)	10.869	70.31	0.712	1.5	4.13	6.06	51.63	51.98
	Equiv. Refl.: (4,-9,1), (5,4,1), (5,-9,-1), (9,-4,1), (9,-5,-1), (-4,9,-1), (-4,-5,1), (-5,9,1), (-5,-4,-1) (-9,4,-1), (-9,5,1)								
Quartz	(3,5,4)	11.183	75.64	0.71	1	2.955	2.93	36.94	37.06
	Equiv. Refl.: (3,-8,-4), (5,-8,4), (-8,3,4), (-8,5,-4)								
Quartz	(5,3,-4)	11.183	75.64	0.709	1	2.955	2.93	36.94	37.06
Quartz	(-7,2,-6)	11.408	81.21	0.702	1.1	1.785	2.01	22.31	22.4
Quartz	(-7,5,6)	11.408	81.21	0.701	1.1	1.785	2.01	22.31	22.4
Quartz	(2,5,-6)	11.408	81.21	0.699	1.1	1.785	2	22.31	22.4
	Equiv. Refl.: (2,-7,6), (5,2,6)								
Quartz	(5,-7,-6)	11.408	81.21	0.698	1.1	1.785	2	22.31	22.4
Quartz	(0,6,6)	11.126	74.53	0.599	0.8	3.195	2.61	39.93	40.02
	Equiv. Refl.: (0,-6,-6)								
Quartz	(6,0,-6)	11.126	74.53	0.596	0.8	3.195	2.6	39.93	40.02
	Equiv. Refl.: (6,-6,6), (-6,6,-6)								
Quartz	(-6,0,6)	11.126	74.53	0.595	0.8	3.195	2.6	39.93	40.02
Sapph	(1,4,18)	11.015	72.59	0.588	0.8	3.619	3.05	45.24	45.34
	Equiv. Refl.: (1,4,-18), (1,-5,18), (1,-5,-18), (4,1,18), (4,1,-18), (4,-5,18), (4,-5,-18), (5,-1,18), (5,-1,-18) (5,-4,18), (5,-4,-18), (-1,5,18), (-1,5,-18), (-1,-4,18), (-1,-4,-18), (-4,5,18), (-4,5,-18), (-4,-1,18) (-4,-1,-18), (-5,1,18), (-5,1,-18), (-5,4,18), (-5,4,-18)								

Ei =	11.544	keV						ΔEg	ΔEt
Cryst	Refl	EB	ΘB	∫ IR dΘ	Width	Ei cotΘ	ΔE	2m, 50μm	2m, 50μm
	(h,k,l)	[keV]	[°]	[μrad]	[μrad]	[meV/μrad]	[meV]	[meV]	[meV]
**LiNbO3**	**(1,5,17)**	**10.85**	**70.04**	**0.566**	**1.3**	**4.194**	**5.34**	**52.42**	**52.69**
	Equiv. Refl.: (1,-6,-17), (5,1,-17), (5,-6,17), (6,-1,-17), (6,-5,17), (-1,6,17), (-1,-5,-17), (-5,6,-17), (-5,-1,17)<br>(-6,1,17), (-6,5,-17)								
**Quartz**	**(1,2,9)**	**11.019**	**72.66**	**0.554**	**0.7**	**3.604**	**2.68**	**45.05**	**45.13**
	Equiv. Refl.: (1,-3,-9)								
**Quartz**	**(-3,1,9)**	**11.019**	**72.66**	**0.553**	**0.7**	**3.604**	**2.68**	**45.05**	**45.13**
	Equiv. Refl.: (-3,2,-9)								
**Quartz**	**(2,-3,9)**	**11.019**	**72.66**	**0.552**	**0.7**	**3.604**	**2.68**	**45.05**	**45.13**
**Quartz**	**(2,1,-9)**	**11.019**	**72.66**	**0.551**	**0.7**	**3.604**	**2.67**	**45.05**	**45.13**
**Quartz**	**(1,5,7)**	**11.414**	**81.39**	**0.55**	**1**	**1.749**	**1.77**	**21.86**	**21.93**
	Equiv. Refl.: (1,-6,-7), (4,5,0), (4,-9,0), (5,4,0), (5,-6,7), (5,-9,0), (-9,4,0), (-9,5,0)								
**Quartz**	**(5,1,-7)**	**11.414**	**81.39**	**0.548**	**1**	**1.749**	**1.77**	**21.86**	**21.93**
**Quartz**	**(-6,1,7)**	**11.414**	**81.39**	**0.547**	**1**	**1.749**	**1.76**	**21.86**	**21.93**
	Equiv. Refl.: (-6,5,-7)								
**Quartz**	**(-1,3,9)**	**11.019**	**72.66**	**0.537**	**0.7**	**3.604**	**2.68**	**45.05**	**45.13**
	Equiv. Refl.: (-1,-2,-9)								
**Sapph**	**(2,5,12)**	**11.003**	**72.39**	**0.536**	**0.8**	**3.663**	**2.9**	**45.79**	**45.88**
	Equiv. Refl.: (2,5,-12), (2,-7,12), (2,-7,-12), (5,2,12), (5,2,-12), (5,-7,12), (5,-7,-12), (7,-2,12), (7,-2,-12)<br>(7,-5,12), (7,-5,-12), (-2,7,12), (-2,7,-12), (-2,-5,12), (-2,-5,-12), (-5,7,12), (-5,7,-12), (-5,-2,12)<br>(-5,-2,-12), (-7,2,12), (-7,2,-12), (-7,5,12), (-7,5,-12), (3,-2,9)								
**Quartz**	**(3,-1,-9)**	**11.019**	**72.66**	**0.535**	**0.7**	**3.604**	**2.68**	**45.05**	**45.13**
	Equiv. Refl.: (-2,3,-9), (-2,-1,9)								
**LiNbO3**	**(1,2,23)**	**10.923**	**71.12**	**0.53**	**1.3**	**3.947**	**4.93**	**49.34**	**49.58**
	Equiv. Refl.: (1,-3,-23), (2,1,-23), (2,-3,23), (3,-1,-23), (3,-2,23), (-1,3,23), (-1,-2,-23), (-2,3,-23), (-2,-1,23)<br>(-3,1,23), (-3,2,-23), (9,-4,0), (9,-5,0), (-4,9,0), (-4,-5,0), (-5,9,0), (-5,-4,0)								
**LiNbO3**	**(3,6,3)**	**11.118**	**74.38**	**0.456**	**1.2**	**3.227**	**3.98**	**40.33**	**40.53**
	Equiv. Refl.: (3,6,-3), (3,-9,3), (3,-9,-3), (6,3,3), (6,3,-3), (6,-9,3), (6,-9,-3), (9,-3,3), (9,-3,-3)<br>(9,-6,3), (9,-6,-3), (-3,9,3), (-3,9,-3), (-3,-6,3), (-3,-6,-3), (-6,9,3), (-6,9,-3), (-6,-3,3)<br>(-6,-3,-3), (-9,3,3), (-9,3,-3), (-9,6,3), (-9,6,-3)								
**Quartz**	**(0,3,9)**	**11.21**	**76.19**	**0.455**	**0.7**	**2.837**	**1.96**	**35.46**	**35.52**
	Equiv. Refl.: (0,-3,-9)								
**Quartz**	**(3,-3,9)**	**11.21**	**76.19**	**0.453**	**0.7**	**2.837**	**1.96**	**35.46**	**35.52**
**Quartz**	**(3,0,-9)**	**11.21**	**76.19**	**0.452**	**0.7**	**2.837**	**1.95**	**35.46**	**35.52**
	Equiv. Refl.: (-3,0,9), (-3,3,-9)								
**Quartz**	**(3,2,-8)**	**11.16**	**75.17**	**0.439**	**0.6**	**3.056**	**1.94**	**38.2**	**38.25**
	Equiv. Refl.: (3,-5,8)								
**Quartz**	**(2,3,8)**	**11.16**	**75.17**	**0.438**	**0.6**	**3.056**	**1.94**	**38.2**	**38.25**
	Equiv. Refl.: (2,-5,-8)								
**Quartz**	**(-5,2,8)**	**11.16**	**75.17**	**0.434**	**0.6**	**3.056**	**1.92**	**38.2**	**38.25**
	Equiv. Refl.: (-5,3,-8)								
**Quartz**	**(-4,0,-8)**	**10.87**	**70.33**	**0.426**	**0.6**	**4.126**	**2.49**	**51.58**	**51.64**
	Equiv. Refl.: (-4,4,8)								
**Quartz**	**(4,0,8)**	**10.87**	**70.33**	**0.425**	**0.6**	**4.126**	**2.49**	**51.58**	**51.64**
	Equiv. Refl.: (4,-4,-8)								
**Quartz**	**(0,4,-8)**	**10.87**	**70.33**	**0.42**	**0.6**	**4.126**	**2.46**	**51.58**	**51.64**
	Equiv. Refl.: (0,-4,8)								
**Quartz**	**(1,7,2)**	**11.237**	**76.75**	**0.4**	**0.6**	**2.718**	**1.71**	**33.98**	**34.02**
	Equiv. Refl.: (1,-8,-2)								
**Quartz**	**(7,1,-2)**	**11.237**	**76.75**	**0.399**	**0.6**	**2.718**	**1.71**	**33.98**	**34.02**
	Equiv. Refl.: (7,-8,2), (-8,1,2), (-8,7,-2)								

Ei =	11.544	keV						ΔEg	ΔEt
Cryst	Refl	EB	ΘB	∫ IR dΘ	Width	Ei cotΘ	ΔE	2m, 50µm	2m, 50µm
	(h,k,l)	[keV]	[°]	[µrad]	[µrad]	[meV/µrad]	[meV]	[meV]	[meV]
**Quartz**	**(-3,5,-8)**	**11.16**	**75.17**	**0.388**	**0.6**	**3.056**	**1.94**	**38.2**	**38.25**
	Equiv. Refl.: (-3,-2,8)								
**Quartz**	**(-2,5,8)**	**11.16**	**75.17**	**0.387**	**0.6**	**3.056**	**1.94**	**38.2**	**38.25**
	Equiv. Refl.: (-2,-3,-8)								
**Quartz**	**(-1,8,2)**	**11.237**	**76.75**	**0.385**	**0.6**	**2.718**	**1.71**	**33.98**	**34.02**
	Equiv. Refl.: (-1,-7,-2)								
**Quartz**	**(5,-3,8)**	**11.16**	**75.17**	**0.383**	**0.6**	**3.056**	**1.92**	**38.2**	**38.25**
	Equiv. Refl.: (8,-1,-2), (8,-7,2), (-7,8,-2), (-7,-1,2)								
**Quartz**	**(5,-2,-8)**	**11.16**	**75.17**	**0.382**	**0.6**	**3.056**	**1.92**	**38.2**	**38.25**
**LiNbO3**	**(2,6,11)**	**11.169**	**75.35**	**0.38**	**1.1**	**3.018**	**3.46**	**37.73**	**37.89**
	Equiv. Refl.: (2,-8,-11), (6,2,-11), (6,-8,11), (8,-2,-11), (8,-6,11), (-2,8,11), (-2,-6,-11), (-6,8,-11), (-6,-2,11) (-8,2,11), (-8,6,-11)								
**Sapph**	**(0,2,22)**	**10.923**	**71.12**	**0.363**	**0.6**	**3.948**	**2.34**	**49.35**	**49.4**
	Equiv. Refl.: (0,-2,-22), (2,0,-22), (2,-2,22), (-2,0,22), (-2,2,-22)								
**LiNbO3**	**(2,5,15)**	**10.973**	**71.9**	**0.346**	**1**	**3.774**	**3.77**	**47.17**	**47.32**
	Equiv. Refl.: (2,5,-15), (2,-7,15), (2,-7,-15), (5,2,15), (5,2,-15), (5,-7,15), (5,-7,-15), (7,-2,15), (7,-2,-15) (7,-5,15), (7,-5,-15), (-2,7,15), (-2,7,-15), (-2,-5,15), (-2,-5,-15), (-5,7,15), (-5,7,-15), (-5,-2,15) (-5,-2,-15), (-7,2,15), (-7,2,-15), (-7,5,15), (-7,5,-15)								
**Sapph**	**(0,0,24)**	**11.455**	**82.88**	**0.329**	**0.7**	**1.442**	**1.03**	**18.02**	**18.05**
	Equiv. Refl.: (0,0,-24)								
**Sapph**	**(2,6,8)**	**11.501**	**85.05**	**0.299**	**0.8**	**1.001**	**0.85**	**12.51**	**12.54**
	Equiv. Refl.: (2,-8,-8), (6,2,-8), (6,-8,8), (8,-2,-8), (8,-6,8), (-2,8,8), (-2,-6,-8), (-6,8,-8), (-6,-2,8) (-8,2,8), (-8,6,-8)								
**Quartz**	**(3,3,-7)**	**11.036**	**72.93**	**0.28**	**0.4**	**3.545**	**1.45**	**44.31**	**44.33**
**Quartz**	**(3,-6,7)**	**11.036**	**72.93**	**0.279**	**0.4**	**3.545**	**1.45**	**44.31**	**44.33**
**Quartz**	**(-6,3,7)**	**11.036**	**72.93**	**0.278**	**0.4**	**3.545**	**1.44**	**44.31**	**44.33**
	Equiv. Refl.: (-6,3,-7)								
**Quartz**	**(3,3,7)**	**11.036**	**72.93**	**0.277**	**0.4**	**3.545**	**1.44**	**44.31**	**44.33**
	Equiv. Refl.: (3,-6,-7)								
**LiNbO3**	**(1,7,9)**	**11.243**	**76.89**	**0.274**	**1**	**2.689**	**2.72**	**33.62**	**33.73**
	Equiv. Refl.: (1,7,-9), (1,-8,9), (1,-8,-9), (7,1,9), (7,1,-9), (7,-8,9), (7,-8,-9), (8,-1,9), (8,-1,-9) (8,-7,9), (8,-7,-9), (-1,8,9), (-1,8,-9), (-1,-7,9), (-1,-7,-9), (-7,8,9), (-7,8,-9), (-7,-1,9) (-7,-1,-9), (-8,1,9), (-8,1,-9), (-8,7,9), (-8,7,-9)								
**Sapph**	**(3,5,-8)**	**11.202**	**76.01**	**0.267**	**0.5**	**2.875**	**1.52**	**35.94**	**35.97**
	Equiv. Refl.: (3,-8,8), (5,3,8), (5,-8,-8), (8,-3,8), (8,-5,-8), (-3,8,-8), (-3,-5,8), (-5,8,8), (-5,-3,-8) (-8,3,-8), (-8,5,8)								
**LiNbO3**	**(3,5,13)**	**11.337**	**79.14**	**0.262**	**1.1**	**2.215**	**2.39**	**27.68**	**27.78**
	Equiv. Refl.: (3,-8,-13), (5,3,-13), (5,-8,13), (8,-3,-13), (8,-5,13), (-3,8,13), (-3,-5,-13), (-5,8,-13), (-5,-3,13) (-8,3,13), (-8,5,-13)								
**Quartz**	**(-3,6,-7)**	**11.036**	**72.93**	**0.187**	**0.4**	**3.545**	**1.45**	**44.31**	**44.33**
	Equiv. Refl.: (-3,-3,7)								
**Quartz**	**(6,-3,7)**	**11.036**	**72.93**	**0.186**	**0.4**	**3.545**	**1.44**	**44.31**	**44.33**
	Equiv. Refl.: (6,-3,-7)								
**Quartz**	**(-3,6,7)**	**11.036**	**72.93**	**0.185**	**0.4**	**3.545**	**1.44**	**44.31**	**44.33**
	Equiv. Refl.: (-3,-3,-7)								
**LiNbO3**	**(3,5,-11)**	**10.906**	**70.86**	**0.172**	**0.7**	**4.007**	**2.71**	**50.09**	**50.16**
	Equiv. Refl.: (3,-8,11), (5,3,11), (5,-8,-11), (8,-3,11), (8,-5,-11), (-3,8,-11), (-3,-5,11), (-5,8,11), (-5,-3,-11) (-8,3,-11), (-8,5,11)								
**Sapph**	**(3,4,-13)**	**11.056**	**73.29**	**0.168**	**0.4**	**3.467**	**1.32**	**43.33**	**43.35**
	Equiv. Refl.: (3,-7,13), (4,3,13), (4,-7,-13), (7,-3,13), (7,-4,-13), (-3,7,-13), (-3,-4,13), (-4,7,13), (-4,-3,-13) (-7,3,-13), (-7,4,13)								
**Sapph**	**(1,2,-22)**	**11.229**	**76.6**	**0.103**	**0.3**	**2.751**	**0.87**	**34.39**	**34.4**

	Equiv. Refl.: (1,-3,22), (2,1,22), (2,-3,-22), (3,-1,22), (3,-2,-22), (-1,3,-22), (-1,-2,22), (-2,3,22), (-2,-1,-22) (-3,1,-22), (-3,2,22)								
**Sapph**	**(2,6,2)**	**10.89**	**70.63**	**0.1**	**0.3**	**4.059**	**1.08**	**50.74**	**50.75**
	Equiv. Refl.: (2,-8,-2), (6,2,-2), (6,-8,2), (8,-2,-2), (8,-6,2), (-2,8,2), (-2,-6,-2), (-6,8,-2), (-6,-2,2) (-8,2,2), (-8,6,-2)								

Ei =	10.207	keV						ΔEg	ΔEt
Cryst	Refl	EB	ΘB	∫ IR dΘ	Width	Ei cotΘ	ΔE	2m, 50µm	2m, 50µm
	(h,k,l)	[keV]	[°]	[µrad]	[µrad]	[meV/µrad]	[meV]	[meV]	[meV]
**Ge**	**(0,4,8)**	**9.801**	**73.78**	**13.9732**	**12.8**	**2.9684**	**37.98**	**37.1**	**53.1**
	Equiv. Refl.: (0,8,4), (4,0,8), (4,8,0), (8,0,4), (8,4,0)								
**Ge**	**(1,1,9)**	**9.983**	**77.98**	**11.2086**	**11.45**	**2.1742**	**24.89**	**27.18**	**36.85**
	Equiv. Refl.: (1,9,1), (3,5,7), (3,7,5), (5,3,7), (5,7,3), (7,3,5), (7,5,3), (9,1,1)								
**Si**	**(0,6,6)**	**9.685**	**71.61**	**8.2618**	**6.99**	**3.3944**	**23.73**	**42.43**	**48.62**
	Equiv. Refl.: (2,2,8), (2,8,2), (6,0,6), (6,6,0), (8,2,2)								
**Si**	**(5,5,5)**	**9.885**	**75.57**	**6.6632**	**5.91**	**2.6255**	**15.51**	**32.82**	**36.3**
	Equiv. Refl.: (1,7,5), (5,1,7), (1,5,7), (5,7,1), (7,1,5), (7,5,1)								
**LiNbO3**	**(2,6,-4)**	**10.185**	**86.26**	**70.633**	**65.5**	**0.668**	**43.69**	**8.34**	**44.48**
	Equiv. Refl.: (-2,8,-4), (-6,-2,-4), (-8,2,-4)								
**LiNbO3**	**(6,-8,-4)**	**10.185**	**86.26**	**70.632**	**65.5**	**0.668**	**43.69**	**8.34**	**44.48**
	Equiv. Refl.: (8,-6,-4)								
**LiNbO3**	**(2,-8,4)**	**10.185**	**86.26**	**70.289**	**65.5**	**0.668**	**43.69**	**8.34**	**44.48**
	Equiv. Refl.: (6,2,4), (8,-2,4), (-2,-6,4), (-6,8,4), (-8,6,4)								
**LiNbO3**	**(1,-7,-10)**	**10.156**	**84.25**	**46.673**	**42.2**	**1.029**	**43.4**	**12.86**	**45.27**
	Equiv. Refl.: (6,1,-10), (7,-1,-10), (-1,-6,-10), (-6,7,-10), (-7,6,-10)								
**LiNbO3**	**(1,6,10)**	**10.156**	**84.25**	**43.974**	**42.2**	**1.029**	**43.4**	**12.86**	**45.27**
	Equiv. Refl.: (6,-7,10), (7,-6,10), (-1,7,10), (-6,-1,10), (-7,1,10)								
**Quartz**	**(0,7,0)**	**10.199**	**87.72**	**39.027**	**34.4**	**0.407**	**14**	**5.09**	**14.89**
	Equiv. Refl.: (0,-7,0), (7,0,0), (7,-7,0), (-7,0,0), (-7,7,0)								
**Quartz**	**(3,-3,-8)**	**10.164**	**84.74**	**27.696**	**23.1**	**0.94**	**21.7**	**11.75**	**24.68**
	Equiv. Refl.: (-3,3,8)								
**Quartz**	**(0,3,-8)**	**10.164**	**84.74**	**27.695**	**23.1**	**0.94**	**21.7**	**11.75**	**24.68**
	Equiv. Refl.: (0,-3,8), (3,0,8)								
**Quartz**	**(-3,0,-8)**	**10.164**	**84.74**	**27.694**	**23.1**	**0.94**	**21.7**	**11.75**	**24.68**
**Quartz**	**(8,-3,0)**	**10.199**	**87.72**	**26.579**	**24**	**0.407**	**9.75**	**5.09**	**11**
	Equiv. Refl.: (8,-5,0), (-3,8,0), (-3,-5,0), (-5,8,0), (-5,-3,0)								
**Quartz**	**(3,5,0)**	**10.199**	**87.72**	**25.408**	**24**	**0.407**	**9.75**	**5.09**	**11**
	Equiv. Refl.: (3,-8,0), (5,3,0), (5,-8,0), (-8,3,0), (-8,5,0)								
**LiNbO3**	**(3,-8,-4)**	**9.896**	**75.83**	**23.518**	**21**	**2.577**	**54.22**	**32.22**	**63.07**
	Equiv. Refl.: (5,3,-4), (8,-3,-4), (-3,-5,-4), (-5,8,-4), (-8,5,-4)								
**LiNbO3**	**(3,5,4)**	**9.896**	**75.83**	**22.897**	**21**	**2.577**	**54.22**	**32.22**	**63.07**
	Equiv. Refl.: (5,-8,4), (8,-5,4), (-3,8,4), (-5,-3,4), (-8,3,4)								
**LiNbO3**	**(4,4,-6)**	**10**	**78.45**	**22.707**	**20.9**	**2.086**	**43.51**	**26.08**	**50.73**
	Equiv. Refl.: (4,-8,-6), (8,-4,-6), (-4,8,-6), (-4,-4,-6), (-8,4,-6)								
**LiNbO3**	**(4,4,6)**	**10**	**78.45**	**21.891**	**20.9**	**2.086**	**43.51**	**26.08**	**50.73**
	Equiv. Refl.: (4,-8,6), (8,-4,6), (-4,8,6), (-4,-4,6), (-8,4,6)								
**LiNbO3**	**(1,-6,-14)**	**9.956**	**77.28**	**20.856**	**19.4**	**2.304**	**44.71**	**28.8**	**53.18**
	Equiv. Refl.: (5,1,-14), (6,-1,-14), (-1,-5,-14), (-5,6,-14), (-6,5,-14)								
**LiNbO3**	**(1,5,14)**	**9.956**	**77.28**	**20.757**	**19.4**	**2.304**	**44.71**	**28.8**	**53.18**
	Equiv. Refl.: (5,-6,14), (6,-5,14), (-1,6,14), (-5,-1,14), (-6,1,14)								
**LiNbO3**	**(2,6,2)**	**10.067**	**80.49**	**20.24**	**19.6**	**1.71**	**33.58**	**21.37**	**39.8**
	Equiv. Refl.: (6,-8,2), (8,-6,2), (-2,8,2), (-6,-2,2), (-8,2,2)								
**LiNbO3**	**(2,-8,-2)**	**10.067**	**80.49**	**19.936**	**19.6**	**1.71**	**33.58**	**21.37**	**39.8**
	Equiv. Refl.: (6,2,-2), (8,-2,-2), (-2,-6,-2), (-6,8,-2), (-8,6,-2)								
**LiNbO3**	**(0,-5,-16)**	**9.976**	**77.8**	**19.215**	**17.2**	**2.207**	**37.96**	**27.59**	**46.93**
	Equiv. Refl.: (5,0,-16), (-5,5,-16)								

Ei =	10.207	keV						ΔEg	ΔEt
Cryst	Refl	EB	ΘB	∫ IR dΘ	Width	Ei cotΘ	ΔE	2m, 50μm	2m, 50μm
	(h,k,l)	[keV]	[°]	[μrad]	[μrad]	[meV/μrad]	[meV]	[meV]	[meV]
LiNbO3	(0,-6,-12)	9.92	76.37	18.914	17.5	2.474	43.38	30.93	53.28
	Equiv. Refl.: (6,0,-12), (-6,6,-12)								
LiNbO3	(0,6,12)	9.92	76.37	18.459	17.5	2.474	43.38	30.93	53.28
	Equiv. Refl.: (6,-6,12), (-6,0,12)								
LiNbO3	(0,1,-22)	9.936	76.76	17.365	16.5	2.402	39.67	30.02	49.75
	Equiv. Refl.: (1,-1,-22), (-1,0,-22)								
LiNbO3	(0,-1,22)	9.936	76.76	17.314	16.5	2.402	39.67	30.02	49.75
	Equiv. Refl.: (1,0,22), (-1,1,22)								
LiNbO3	(0,5,16)	9.976	77.8	16.649	17.2	2.207	37.96	27.59	46.93
	Equiv. Refl.: (5,-5,16), (-5,0,16)								
LiNbO3	(0,6,-12)	9.92	76.37	16.602	15.6	2.474	38.58	30.93	49.45
	Equiv. Refl.: (6,-6,-12), (-6,0,-12)								
LiNbO3	(0,-6,12)	9.92	76.37	15.957	15.6	2.474	38.58	30.93	49.45
	Equiv. Refl.: (6,0,12), (-6,6,12)								
LiNbO3	(0,7,-4)	9.896	75.83	15.84	15	2.577	38.73	32.22	50.38
	Equiv. Refl.: (7,-7,-4), (-7,0,-4), (0,5,-14), (0,-5,14), (5,0,14), (5,-5,-14), (-5,0,-14), (-5,5,14)								
LiNbO3	(0,-7,4)	9.896	75.83	15.529	15	2.577	38.73	32.22	50.38
	Equiv. Refl.: (7,0,4), (-7,7,4)								
LiNbO3	(0,-7,-2)	9.774	73.26	15.236	14	3.07	43.07	38.38	57.68
	Equiv. Refl.: (7,0,-2), (-7,7,-2)								
LiNbO3	(2,4,-14)	9.661	71.17	15.153	13.8	3.481	47.93	43.51	64.73
	Equiv. Refl.: (4,-6,-14), (6,-4,-14), (-2,6,-14), (-4,-2,-14), (-6,2,-14)								
LiNbO3	(1,-7,8)	9.795	73.66	14.521	13.3	2.993	39.81	37.41	54.63
	Equiv. Refl.: (6,1,8), (7,-1,8), (-1,-6,8), (-6,7,8), (-7,6,8)								
LiNbO3	(0,7,2)	9.774	73.26	14.478	14	3.07	43.07	38.38	57.68
	Equiv. Refl.: (7,-7,2), (-7,0,2)								
LiNbO3	(2,-6,14)	9.661	71.17	14.425	13.8	3.481	47.93	43.51	64.73
	Equiv. Refl.: (4,2,14), (6,-2,14), (-2,-4,14), (-4,6,14), (-6,4,14)								
LiNbO3	(1,6,-8)	9.795	73.66	13.28	13.3	2.993	39.81	37.41	54.63
	Equiv. Refl.: (6,-7,-8), (7,-6,-8), (-1,7,-8), (-6,-1,-8), (-7,1,-8)								
LiNbO3	(1,-3,-20)	9.671	71.34	13.159	12.4	3.446	42.86	43.08	60.77
	Equiv. Refl.: (2,1,-20), (3,-1,-20), (-1,-2,-20), (-2,3,-20), (-3,2,-20)								
LiNbO3	(1,2,20)	9.671	71.34	13.06	12.4	3.446	42.86	43.08	60.77
	Equiv. Refl.: (2,-3,20), (3,-2,20), (-2,-1,20)								
LiNbO3	(-1,3,20)	9.671	71.34	13.059	12.4	3.446	42.86	43.08	60.77
	Equiv. Refl.: (-3,1,20)								
LiNbO3	(4,4,0)	9.634	70.7	12.377	11.6	3.574	41.45	44.67	60.94
	Equiv. Refl.: (4,-8,0), (8,-4,0), (-4,8,0), (-4,-4,0), (-8,4,0)								
Sapph	(2,3,-16)	10.066	80.46	12.217	10.5	1.715	18.07	21.44	28.04
	Equiv. Refl.: (2,-5,16), (3,2,16), (3,-5,-16), (5,-2,16), (5,-3,-16), (-2,5,-16), (-2,-3,16), (-3,5,16), (-3,-2,-16) (-5,2,-16), (-5,3,16)								
Quartz	(-5,7,4)	10.19	86.71	12.196	11.9	0.587	6.99	7.33	10.13
	Equiv. Refl.: (-5,-2,-4)								
Quartz	(7,-5,-4)	10.19	86.71	12.19	11.9	0.587	6.99	7.33	10.13
Quartz	(7,-2,4)	10.19	86.71	12.187	11.9	0.587	6.99	7.33	10.13
Quartz	(-2,-5,4)	10.19	86.71	12.184	11.9	0.587	6.99	7.33	10.13
Quartz	(-2,7,-4)	10.19	86.71	12.182	11.9	0.587	6.99	7.33	10.13
Quartz	(5,2,4)	10.19	86.71	11.855	11.9	0.587	6.99	7.33	10.13
	Equiv. Refl.: (5,-7,-4)								
Quartz	(-7,5,4)	10.19	86.71	11.849	11.9	0.587	6.99	7.33	10.13
Quartz	(-7,2,-4)	10.19	86.71	11.846	11.9	0.587	6.99	7.33	10.13

Ei =	10.207	keV						ΔEg	ΔEt
Cryst	Refl	EB	ΘB	∫ IR dΘ	Width	Ei cotΘ	ΔE	2m, 50µm	2m, 50µm
	(h,k,l)	[keV]	[°]	[µrad]	[µrad]	[meV/µrad]	[meV]	[meV]	[meV]
**Quartz**	**(2,5,-4)**	**10.19**	**86.71**	**11.844**	**11.9**	**0.587**	**6.99**	**7.33**	**10.13**
**Quartz**	**(2,-7,4)**	**10.19**	**86.71**	**11.842**	**11.9**	**0.587**	**6.99**	**7.33**	**10.13**
**Sapph**	**(0,3,18)**	**9.705**	**71.95**	**11.082**	**9**	**3.327**	**30.03**	**41.58**	**51.29**
	Equiv. Refl.: (0,3,-18), (0,-3,18), (0,-3,-18), (3,0,18), (3,0,-18), (3,-3,18), (3,-3,-18), (-3,0,18), (-3,0,-18) (-3,3,18), (-3,3,-18)								
**Quartz**	**(2,5,4)**	**10.19**	**86.71**	**10.417**	**10**	**0.587**	**5.87**	**7.33**	**9.39**
	Equiv. Refl.: (2,-7,-4)								
**Quartz**	**(-7,5,-4)**	**10.19**	**86.71**	**10.411**	**10**	**0.587**	**5.86**	**7.33**	**9.39**
**Quartz**	**(-7,2,4)**	**10.19**	**86.71**	**10.409**	**10**	**0.587**	**5.86**	**7.33**	**9.39**
**Quartz**	**(5,2,-4)**	**10.19**	**86.71**	**10.404**	**10**	**0.587**	**5.86**	**7.33**	**9.39**
**Quartz**	**(5,-7,4)**	**10.19**	**86.71**	**10.402**	**10**	**0.587**	**5.86**	**7.33**	**9.39**
**Sapph**	**(2,5,6)**	**9.822**	**74.21**	**9.745**	**8.1**	**2.886**	**23.44**	**36.08**	**43.03**
	Equiv. Refl.: (2,5,-6), (2,-7,6), (2,-7,-6), (5,2,6), (5,2,-6), (5,-7,6), (5,-7,-6), (7,-2,6), (7,-2,-6) (7,-5,6), (7,-5,-6), (-2,7,6), (-2,7,-6), (-2,-5,6), (-2,-5,-6), (-5,7,6), (-5,7,-6), (-5,-2,6) (-5,-2,-6), (-7,2,6), (-7,2,-6), (-7,5,6), (-7,5,-6)								
**Quartz**	**(-2,-5,-4)**	**10.19**	**86.71**	**9.685**	**10**	**0.587**	**5.87**	**7.33**	**9.39**
**Quartz**	**(-2,7,4)**	**10.19**	**86.71**	**9.684**	**10**	**0.587**	**5.87**	**7.33**	**9.39**
**Quartz**	**(7,-5,4)**	**10.19**	**86.71**	**9.681**	**10**	**0.587**	**5.86**	**7.33**	**9.39**
**Quartz**	**(7,-2,-4)**	**10.19**	**86.71**	**9.68**	**10**	**0.587**	**5.86**	**7.33**	**9.39**
**Quartz**	**(-5,-2,4)**	**10.19**	**86.71**	**9.671**	**10**	**0.587**	**5.86**	**7.33**	**9.39**
**Quartz**	**(-5,7,-4)**	**10.19**	**86.71**	**9.67**	**10**	**0.587**	**5.86**	**7.33**	**9.39**
**Quartz**	**(8,-4,0)**	**10.094**	**81.48**	**9.599**	**8.1**	**1.53**	**12.38**	**19.12**	**22.78**
	Equiv. Refl.: (-4,8,0), (-4,-4,0)								
**LiNbO3**	**(3,-8,2)**	**9.774**	**73.26**	**9.381**	**9.3**	**3.07**	**28.55**	**38.38**	**47.83**
	Equiv. Refl.: (5,3,2), (8,-3,2), (-3,-5,2), (-5,8,2), (-8,5,2)								
**Sapph**	**(0,2,-20)**	**10.009**	**78.69**	**9.275**	**8**	**2.041**	**16.42**	**25.52**	**30.34**
	Equiv. Refl.: (0,-2,20), (2,0,20), (2,-2,-20), (-2,0,-20), (-2,2,20)								
**LiNbO3**	**(3,5,-2)**	**9.774**	**73.26**	**8.982**	**9.3**	**3.07**	**28.55**	**38.38**	**47.83**
	Equiv. Refl.: (5,-8,-2), (8,-5,-2), (-3,8,-2), (-5,-3,-2), (-8,3,-2)								
**Quartz**	**(4,4,0)**	**10.094**	**81.48**	**8.744**	**8.1**	**1.53**	**12.38**	**19.12**	**22.78**
	Equiv. Refl.: (4,-8,0), (-8,4,0)								
**Sapph**	**(3,4,8)**	**9.916**	**76.28**	**8.602**	**7.5**	**2.492**	**18.59**	**31.15**	**36.28**
	Equiv. Refl.: (3,-7,-8), (4,3,-8), (4,-7,8), (7,-3,-8), (7,-4,8), (-3,7,8), (-3,-4,-8), (-4,7,-8), (-4,-3,8) (-7,3,8), (-7,4,-8)								
**Sapph**	**(0,1,20)**	**9.664**	**71.22**	**8.404**	**7**	**3.471**	**24.14**	**43.38**	**49.65**
	Equiv. Refl.: (0,-1,-20), (1,0,-20), (1,-1,20), (-1,0,20), (-1,1,-20)								
**Quartz**	**(-6,7,-2)**	**9.826**	**74.29**	**8.342**	**6.8**	**2.871**	**19.4**	**35.89**	**40.8**
**Quartz**	**(7,-1,-2)**	**9.826**	**74.29**	**8.341**	**6.8**	**2.871**	**19.39**	**35.89**	**40.8**
	Equiv. Refl.: (-1,7,2), (-1,-6,-2), (-6,-1,2)								
**Quartz**	**(7,-6,2)**	**9.826**	**74.29**	**8.34**	**6.8**	**2.871**	**19.39**	**35.89**	**40.8**
**Quartz**	**(6,1,-2)**	**9.826**	**74.29**	**8.044**	**6.8**	**2.871**	**19.39**	**35.89**	**40.8**
	Equiv. Refl.: (6,-7,2)								
**Quartz**	**(1,6,2)**	**9.826**	**74.29**	**8.043**	**6.8**	**2.871**	**19.39**	**35.89**	**40.8**
	Equiv. Refl.: (1,-7,-2), (-7,1,2), (-7,6,-2)								
**Quartz**	**(6,-7,-3)**	**10.155**	**84.21**	**7.59**	**6.9**	**1.035**	**7.11**	**12.94**	**14.77**
**Quartz**	**(6,1,3)**	**10.155**	**84.21**	**7.588**	**6.9**	**1.035**	**7.11**	**12.94**	**14.77**
**Quartz**	**(1,6,-3)**	**10.155**	**84.21**	**7.584**	**6.9**	**1.035**	**7.11**	**12.94**	**14.77**
**Quartz**	**(1,-7,3)**	**10.155**	**84.21**	**7.583**	**6.9**	**1.035**	**7.11**	**12.94**	**14.77**
**Quartz**	**(-7,1,-3)**	**10.155**	**84.21**	**7.581**	**6.9**	**1.035**	**7.11**	**12.94**	**14.77**
**Quartz**	**(-7,6,3)**	**10.155**	**84.21**	**7.58**	**6.9**	**1.035**	**7.11**	**12.94**	**14.77**

Ei =	10.207	keV						ΔEg	ΔEt
Cryst	Refl	EB	ΘB	∫ IR dΘ	Width	Ei cotΘ	ΔE	2m, 50µm	2m, 50µm
	(h,k,l)	[keV]	[°]	[µrad]	[µrad]	[meV/µrad]	[meV]	[meV]	[meV]
**Sapph**	**(1,6,4)**	**10.048**	**79.88**	**7.263**	**6.7**	**1.822**	**12.17**	**22.77**	**25.82**
	Equiv. Refl.: (1,-7,-4), (6,1,-4), (6,-7,4), (7,-1,-4), (7,-6,4), (-1,7,4), (-1,-6,-4), (-6,7,-4), (-6,-1,4) (-7,1,4), (-7,6,-4)								
**Quartz**	**(1,-3,8)**	**9.953**	**77.19**	**7.145**	**6.2**	**2.321**	**14.29**	**29.01**	**32.34**
**Quartz**	**(1,2,-8)**	**9.953**	**77.19**	**7.143**	**6.2**	**2.321**	**14.29**	**29.01**	**32.34**
**Quartz**	**(-3,1,-8)**	**9.953**	**77.19**	**7.137**	**6.2**	**2.321**	**14.28**	**29.01**	**32.33**
**Quartz**	**(-3,2,8)**	**9.953**	**77.19**	**7.135**	**6.2**	**2.321**	**14.27**	**29.01**	**32.33**
**Quartz**	**(2,1,8)**	**9.953**	**77.19**	**7.133**	**6.1**	**2.321**	**14.27**	**29.01**	**32.33**
	Equiv. Refl.: (2,-3,-8)								
**Quartz**	**(-1,3,-8)**	**9.953**	**77.19**	**7.12**	**6.2**	**2.321**	**14.29**	**29.01**	**32.34**
**Quartz**	**(-1,-2,8)**	**9.953**	**77.19**	**7.119**	**6.2**	**2.321**	**14.29**	**29.01**	**32.34**
**Quartz**	**(3,-1,8)**	**9.953**	**77.19**	**7.112**	**6.2**	**2.321**	**14.28**	**29.01**	**32.33**
**Quartz**	**(3,-2,-8)**	**9.953**	**77.19**	**7.111**	**6.2**	**2.321**	**14.27**	**29.01**	**32.33**
**Quartz**	**(-2,3,8)**	**9.953**	**77.19**	**7.109**	**6.1**	**2.321**	**14.27**	**29.01**	**32.33**
	Equiv. Refl.: (-2,-1,-8)								
**Quartz**	**(7,-1,-3)**	**10.155**	**84.21**	**7.038**	**6.4**	**1.035**	**6.64**	**12.94**	**14.54**
**Quartz**	**(7,-6,3)**	**10.155**	**84.21**	**7.037**	**6.4**	**1.035**	**6.63**	**12.94**	**14.54**
	Equiv. Refl.: (-1,-6,-3)								
**Quartz**	**(-1,7,3)**	**10.155**	**84.21**	**7.035**	**6.4**	**1.035**	**6.63**	**12.94**	**14.54**
**Quartz**	**(-6,7,-3)**	**10.155**	**84.21**	**7.033**	**6.4**	**1.035**	**6.63**	**12.94**	**14.54**
	Equiv. Refl.: (-6,-1,3)								
**Quartz**	**(4,-4,-7)**	**9.921**	**76.41**	**6.944**	**6**	**2.467**	**14.71**	**30.84**	**34.17**
	Equiv. Refl.: (-4,4,7)								
**Quartz**	**(4,0,7)**	**9.921**	**76.41**	**6.943**	**6**	**2.467**	**14.71**	**30.84**	**34.17**
	Equiv. Refl.: (-4,0,-7)								
**Quartz**	**(0,4,-7)**	**9.921**	**76.41**	**6.941**	**6**	**2.467**	**14.7**	**30.84**	**34.17**
	Equiv. Refl.: (0,-4,7)								
**Quartz**	**(-6,7,3)**	**10.155**	**84.21**	**6.619**	**6.9**	**1.035**	**7.11**	**12.94**	**14.77**
**Quartz**	**(-6,-1,-3)**	**10.155**	**84.21**	**6.617**	**6.9**	**1.035**	**7.11**	**12.94**	**14.77**
**Quartz**	**(-1,-6,3)**	**10.155**	**84.21**	**6.613**	**6.9**	**1.035**	**7.11**	**12.94**	**14.77**
**Quartz**	**(-1,7,-3)**	**10.155**	**84.21**	**6.612**	**6.9**	**1.035**	**7.11**	**12.94**	**14.77**
**Quartz**	**(7,-1,3)**	**10.155**	**84.21**	**6.611**	**6.9**	**1.035**	**7.11**	**12.94**	**14.77**
**Quartz**	**(7,-6,-3)**	**10.155**	**84.21**	**6.61**	**6.9**	**1.035**	**7.11**	**12.94**	**14.77**
**Quartz**	**(-3,-4,4)**	**9.98**	**77.89**	**6.458**	**5.5**	**2.191**	**12.16**	**27.39**	**29.96**
**Quartz**	**(-3,7,-4)**	**9.98**	**77.89**	**6.457**	**5.5**	**2.191**	**12.16**	**27.39**	**29.96**
**Quartz**	**(-4,7,4)**	**9.98**	**77.89**	**6.455**	**5.5**	**2.191**	**12.15**	**27.39**	**29.96**
	Equiv. Refl.: (-4,-3,-4)								
**Quartz**	**(7,-3,4)**	**9.98**	**77.89**	**6.454**	**5.5**	**2.191**	**12.15**	**27.39**	**29.96**
	Equiv. Refl.: (7,-4,-4)								
**Quartz**	**(3,4,-4)**	**9.98**	**77.89**	**6.3**	**5.5**	**2.191**	**12.16**	**27.39**	**29.96**
	Equiv. Refl.: (3,-7,4)								
**Quartz**	**(4,3,4)**	**9.98**	**77.89**	**6.297**	**5.5**	**2.191**	**12.15**	**27.39**	**29.96**
	Equiv. Refl.: (-7,3,-4), (-7,4,4)								
**Quartz**	**(4,-7,-4)**	**9.98**	**77.89**	**6.296**	**5.5**	**2.191**	**12.15**	**27.39**	**29.96**
**Quartz**	**(-7,1,3)**	**10.155**	**84.21**	**6.199**	**6.4**	**1.035**	**6.64**	**12.94**	**14.54**
	Equiv. Refl.: (-7,6,-3)								
**Quartz**	**(1,6,3)**	**10.155**	**84.21**	**6.198**	**6.4**	**1.035**	**6.63**	**12.94**	**14.54**
**Quartz**	**(1,-7,-3)**	**10.155**	**84.21**	**6.196**	**6.4**	**1.035**	**6.63**	**12.94**	**14.54**
**Quartz**	**(6,1,-3)**	**10.155**	**84.21**	**6.195**	**6.4**	**1.035**	**6.63**	**12.94**	**14.54**
	Equiv. Refl.: (6,-7,3)								

Ei =	10.207	keV						ΔEg	ΔEt
**Cryst**	**Refl**	**EB**	**ΘB**	**∫ IR dΘ**	**Width**	**Ei cotΘ**	**ΔE**	**2m, 50µm**	**2m, 50µm**
	**(h,k,l)**	**[keV]**	**[°]**	**[µrad]**	**[µrad]**	**[meV/µrad]**	**[meV]**	**[meV]**	**[meV]**
**Sapph**	**(3,3,12)**	**9.691**	**71.7**	**6.154**	**5.4**	**3.375**	**18.27**	**42.19**	**45.98**
	Equiv. Refl.: (3,3,-12), (3,-6,12), (3,-6,-12), (6,-3,12), (6,-3,-12), (-3,6,12), (-3,6,-12), (-3,-3,12), (-3,-3,-12) (-6,3,12), (-6,3,-12)								
**Sapph**	**(1,4,15)**	**9.939**	**76.84**	**5.982**	**5.3**	**2.386**	**12.73**	**29.82**	**32.43**
	Equiv. Refl.: (1,4,-15), (1,-5,15), (1,-5,-15), (4,1,15), (4,1,-15), (4,-5,15), (4,-5,-15), (5,-1,15), (5,-1,-15) (5,-4,15), (5,-4,-15), (-1,5,15), (-1,5,-15), (-1,-4,15), (-1,-4,-15), (-4,5,15), (-4,5,-15), (-4,-1,15) (-4,-1,-15), (-5,1,15), (-5,1,-15), (-5,4,15), (-5,4,-15)								
**Quartz**	**(5,1,5)**	**9.935**	**76.74**	**5.911**	**5.2**	**2.406**	**12.49**	**30.08**	**32.57**
	Equiv. Refl.: (5,-6,-5)								
**Quartz**	**(1,5,-5)**	**9.935**	**76.74**	**5.906**	**5.2**	**2.406**	**12.48**	**30.08**	**32.56**
	Equiv. Refl.: (1,-6,5)								
**Quartz**	**(-6,1,-5)**	**9.935**	**76.74**	**5.904**	**5.2**	**2.406**	**12.48**	**30.08**	**32.56**
	Equiv. Refl.: (-6,5,5)								
**Quartz**	**(6,-6,-4)**	**9.873**	**75.3**	**5.869**	**5.1**	**2.678**	**13.57**	**33.48**	**36.13**
	Equiv. Refl.: (-6,6,4)								
**Quartz**	**(0,6,-4)**	**9.873**	**75.3**	**5.868**	**5.1**	**2.678**	**13.57**	**33.48**	**36.13**
	Equiv. Refl.: (0,-6,4), (6,0,4), (-6,0,-4)								
**Quartz**	**(1,-3,-8)**	**9.953**	**77.19**	**5.867**	**4.9**	**2.321**	**11.34**	**29.01**	**31.15**
	Equiv. Refl.: (2,1,-8), (2,-3,8)								
**Quartz**	**(1,2,8)**	**9.953**	**77.19**	**5.865**	**4.9**	**2.321**	**11.34**	**29.01**	**31.15**
**Quartz**	**(-3,1,8)**	**9.953**	**77.19**	**5.864**	**4.9**	**2.321**	**11.34**	**29.01**	**31.15**
	Equiv. Refl.: (-3,2,-8)								
**Quartz**	**(-5,6,5)**	**9.935**	**76.74**	**5.819**	**5.2**	**2.406**	**12.49**	**30.08**	**32.57**
	Equiv. Refl.: (-5,-1,-5)								
**Quartz**	**(-1,6,-5)**	**9.935**	**76.74**	**5.815**	**5.2**	**2.406**	**12.48**	**30.08**	**32.56**
	Equiv. Refl.: (-1,-5,5)								
**Quartz**	**(6,-1,5)**	**9.935**	**76.74**	**5.812**	**5.2**	**2.406**	**12.48**	**30.08**	**32.56**
	Equiv. Refl.: (6,-5,-5)								
**Quartz**	**(-1,4,-7)**	**9.595**	**70.06**	**5.783**	**4.8**	**3.703**	**17.68**	**46.29**	**49.55**
**Quartz**	**(-1,-3,7)**	**9.595**	**70.06**	**5.782**	**4.8**	**3.703**	**17.68**	**46.29**	**49.55**
**Quartz**	**(-3,4,7)**	**9.595**	**70.06**	**5.781**	**4.8**	**3.703**	**17.68**	**46.29**	**49.55**
	Equiv. Refl.: (-3,-1,-7)								
**Quartz**	**(4,-1,7)**	**9.595**	**70.06**	**5.777**	**4.8**	**3.703**	**17.67**	**46.29**	**49.55**
	Equiv. Refl.: (4,-3,-7)								
**Sapph**	**(1,5,-10)**	**9.641**	**70.82**	**5.704**	**4.9**	**3.55**	**17.56**	**44.37**	**47.72**
	Equiv. Refl.: (1,-6,10), (5,1,10), (5,-6,-10), (6,-1,10), (6,-5,-10), (-1,6,-10), (-1,-5,10), (-5,6,10), (-5,-1,-10) (-6,1,-10), (-6,5,10)								
**Quartz**	**(-2,3,-8)**	**9.953**	**77.19**	**5.598**	**4.9**	**2.321**	**11.35**	**29.01**	**31.15**
	Equiv. Refl.: (-2,-1,8)								
**Quartz**	**(-1,3,8)**	**9.953**	**77.19**	**5.597**	**4.9**	**2.321**	**11.34**	**29.01**	**31.15**
**Quartz**	**(3,-1,-8)**	**9.953**	**77.19**	**5.596**	**4.9**	**2.321**	**11.34**	**29.01**	**31.15**
	Equiv. Refl.: (3,-2,8)								
**Quartz**	**(-1,-2,-8)**	**9.953**	**77.19**	**5.595**	**4.9**	**2.321**	**11.34**	**29.01**	**31.15**
**Quartz**	**(1,-4,7)**	**9.595**	**70.06**	**5.57**	**4.8**	**3.703**	**17.68**	**46.29**	**49.55**
**Quartz**	**(1,3,-7)**	**9.595**	**70.06**	**5.569**	**4.8**	**3.703**	**17.68**	**46.29**	**49.55**
**Quartz**	**(3,1,7)**	**9.595**	**70.06**	**5.568**	**4.8**	**3.703**	**17.68**	**46.29**	**49.55**
	Equiv. Refl.: (3,-4,-7)								
**Quartz**	**(-4,1,-7)**	**9.595**	**70.06**	**5.565**	**4.8**	**3.703**	**17.67**	**46.29**	**49.55**
	Equiv. Refl.: (-4,3,7)								

Ei =	10.207	keV						ΔEg	ΔEt
**Cryst**	**Refl**	**EB**	**ΘB**	**∫ IR dΘ**	**Width**	**Ei cotΘ**	**ΔE**	**2m, 50µm**	**2m, 50µm**
	**(h,k,l)**	**[keV]**	**[°]**	**[µrad]**	**[µrad]**	**[meV/µrad]**	**[meV]**	**[meV]**	**[meV]**
**Sapph**	**(1,6,-2)**	**9.911**	**76.17**	**4.589**	**4.3**	**2.512**	**10.75**	**31.4**	**33.19**
	Equiv. Refl.: (1,-7,2), (6,1,2), (6,-7,-2), (7,-1,2), (7,-6,-2), (-1,7,-2), (-1,-6,2), (-6,7,2), (-6,-1,-2) (-7,1,-2), (-7,6,2)								
**Quartz**	**(-4,-4,-1)**	**10.159**	**84.46**	**4.512**	**4.3**	**0.991**	**4.3**	**12.38**	**13.11**
**Quartz**	**(-4,8,1)**	**10.159**	**84.46**	**4.511**	**4.3**	**0.991**	**4.3**	**12.38**	**13.11**
	Equiv. Refl.: (-4,8,-1), (-4,-4,1)								
**Quartz**	**(8,-4,1)**	**10.159**	**84.46**	**4.509**	**4.3**	**0.991**	**4.3**	**12.38**	**13.11**
	Equiv. Refl.: (8,-4,-1)								
**Sapph**	**(2,4,13)**	**10.093**	**81.43**	**4.29**	**4.4**	**1.538**	**6.76**	**19.22**	**20.37**
	Equiv. Refl.: (2,-6,-13), (4,2,-13), (4,-6,13), (6,-2,-13), (6,-4,13), (-2,6,13), (-2,-4,-13), (-4,6,-13), (-4,-2,13) (-6,2,13), (-6,4,-13)								
**Sapph**	**(1,5,11)**	**9.886**	**75.59**	**3.888**	**3.7**	**2.624**	**9.76**	**32.79**	**34.22**
	Equiv. Refl.: (1,-6,-11), (5,1,-11), (5,-6,11), (6,-1,-11), (6,-5,11), (-1,6,11), (-1,-5,-11), (-5,6,-11), (-5,-1,11) (-6,1,11), (-6,5,-11)								
**Quartz**	**(4,4,1)**	**10.159**	**84.46**	**3.606**	**4.3**	**0.991**	**4.3**	**12.38**	**13.11**
	Equiv. Refl.: (4,4,-1), (4,-8,1), (4,-8,-1)								
**Quartz**	**(-8,4,1)**	**10.159**	**84.46**	**3.604**	**4.3**	**0.991**	**4.3**	**12.38**	**13.11**
	Equiv. Refl.: (-8,4,-1)								
**Quartz**	**(1,3,7)**	**9.595**	**70.06**	**3.39**	**2.8**	**3.703**	**10.26**	**46.29**	**47.42**
	Equiv. Refl.: (1,-4,-7)								
**Quartz**	**(-4,3,-7)**	**9.595**	**70.06**	**3.388**	**2.8**	**3.703**	**10.26**	**46.29**	**47.41**
**Quartz**	**(-4,1,7)**	**9.595**	**70.06**	**3.387**	**2.8**	**3.703**	**10.26**	**46.29**	**47.41**
**Quartz**	**(3,1,-7)**	**9.595**	**70.06**	**3.384**	**2.8**	**3.703**	**10.25**	**46.29**	**47.41**
	Equiv. Refl.: (3,-4,7)								
**Quartz**	**(3,4,4)**	**9.98**	**77.89**	**3.251**	**3**	**2.191**	**6.46**	**27.39**	**28.14**
	Equiv. Refl.: (3,-7,-4), (4,3,-4), (4,-7,4), (-7,4,-4)								
**Quartz**	**(-7,3,4)**	**9.98**	**77.89**	**3.249**	**2.9**	**2.191**	**6.46**	**27.39**	**28.14**
**Quartz**	**(-1,4,7)**	**9.595**	**70.06**	**3.14**	**2.8**	**3.703**	**10.26**	**46.29**	**47.42**
**Quartz**	**(-1,-3,-7)**	**9.595**	**70.06**	**3.139**	**2.8**	**3.703**	**10.26**	**46.29**	**47.42**
**Quartz**	**(4,-3,7)**	**9.595**	**70.06**	**3.138**	**2.8**	**3.703**	**10.26**	**46.29**	**47.41**
**Quartz**	**(4,-1,-7)**	**9.595**	**70.06**	**3.137**	**2.8**	**3.703**	**10.26**	**46.29**	**47.41**
**Quartz**	**(-3,-1,7)**	**9.595**	**70.06**	**3.134**	**2.8**	**3.703**	**10.25**	**46.29**	**47.41**
**Quartz**	**(-3,4,-7)**	**9.595**	**70.06**	**3.133**	**2.8**	**3.703**	**10.25**	**46.29**	**47.41**
**Quartz**	**(6,-2,-5)**	**9.609**	**70.29**	**2.983**	**2.5**	**3.657**	**9.16**	**45.71**	**46.62**
**Quartz**	**(6,-4,5)**	**9.609**	**70.29**	**2.982**	**2.5**	**3.657**	**9.16**	**45.71**	**46.62**
**Quartz**	**(-2,6,5)**	**9.609**	**70.29**	**2.979**	**2.5**	**3.657**	**9.15**	**45.71**	**46.62**
	Equiv. Refl.: (-2,-4,-5), (-4,6,-5), (-4,-2,5)								
**Quartz**	**(7,-1,-1)**	**9.623**	**70.52**	**2.941**	**2.5**	**3.611**	**9.08**	**45.13**	**46.04**
**Quartz**	**(7,-6,1)**	**9.623**	**70.52**	**2.94**	**2.5**	**3.611**	**9.08**	**45.13**	**46.04**
	Equiv. Refl.: (-1,7,1), (-1,-6,-1), (-6,7,-1), (-6,-1,1)								
**Quartz**	**(6,-2,5)**	**9.609**	**70.29**	**2.899**	**2.5**	**3.657**	**9.16**	**45.71**	**46.62**
	Equiv. Refl.: (6,-4,-5), (-4,6,5), (-4,-2,-5)								
**Quartz**	**(-2,-4,5)**	**9.609**	**70.29**	**2.896**	**2.5**	**3.657**	**9.15**	**45.71**	**46.62**
**Quartz**	**(-2,6,-5)**	**9.609**	**70.29**	**2.895**	**2.5**	**3.657**	**9.15**	**45.71**	**46.62**
**Quartz**	**(-6,4,5)**	**9.609**	**70.29**	**2.842**	**2.5**	**3.657**	**9.16**	**45.71**	**46.62**
**Quartz**	**(4,2,5)**	**9.609**	**70.29**	**2.841**	**2.5**	**3.657**	**9.16**	**45.71**	**46.62**
	Equiv. Refl.: (4,-6,-5), (-6,2,-5)								
**Quartz**	**(2,4,-5)**	**9.609**	**70.29**	**2.839**	**2.5**	**3.657**	**9.15**	**45.71**	**46.62**
**Quartz**	**(2,-6,5)**	**9.609**	**70.29**	**2.838**	**2.5**	**3.657**	**9.15**	**45.71**	**46.62**

Ei = Cryst	10.207 Refl (h,k,l)	keV EB [keV]	ΘB [°]	∫ IR dΘ [μrad]	Width [μrad]	Ei cotΘ [meV/μrad]	ΔE [meV]	ΔEg 2m, 50μm [meV]	ΔEt 2m, 50μm [meV]
**LiNbO3**	**(1,1,21)**	**9.695**	**71.77**	**2.795**	**4.3**	**3.362**	**14.42**	**42.03**	**44.43**
	Equiv. Refl.: (1,1,-21), (1,-2,21), (1,-2,-21), (2,-1,21), (2,-1,-21), (-1,2,21), (-1,2,-21), (-1,-1,21), (-1,-1,-21) (-2,1,21), (-2,1,-21)								
**Quartz**	**(-6,2,5)**	**9.609**	**70.29**	**2.784**	**2.5**	**3.657**	**9.16**	**45.71**	**46.62**
**Quartz**	**(-6,4,-5)**	**9.609**	**70.29**	**2.783**	**2.5**	**3.657**	**9.16**	**45.71**	**46.62**
**Quartz**	**(2,4,5)**	**9.609**	**70.29**	**2.781**	**2.5**	**3.657**	**9.15**	**45.71**	**46.62**
	Equiv. Refl.: (2,-6,-5), (4,2,-5), (4,-6,5)								
**Quartz**	**(-3,7,4)**	**9.98**	**77.89**	**2.694**	**3**	**2.191**	**6.46**	**27.39**	**28.14**
	Equiv. Refl.: (-3,-4,-4), (-4,7,-4)								
**Quartz**	**(7,-4,4)**	**9.98**	**77.89**	**2.693**	**2.9**	**2.191**	**6.46**	**27.39**	**28.14**
	Equiv. Refl.: (-4,-3,4)								
**Quartz**	**(7,-3,-4)**	**9.98**	**77.89**	**2.692**	**2.9**	**2.191**	**6.46**	**27.39**	**28.14**
**LiNbO3**	**(1,6,7)**	**9.64**	**70.82**	**2.621**	**4**	**3.551**	**14.38**	**44.38**	**46.65**
	Equiv. Refl.: (1,-7,-7), (6,1,-7), (6,-7,7), (7,-1,-7), (7,-6,7), (-1,7,7), (-1,-6,-7), (-6,7,-7), (-6,-1,7) (-7,1,7), (-7,6,-7)								
**Quartz**	**(1,6,1)**	**9.623**	**70.52**	**2.569**	**2.5**	**3.611**	**9.08**	**45.13**	**46.04**
	Equiv. Refl.: (1,-7,-1), (6,1,-1), (6,-7,1), (-7,1,1), (-7,6,-1)								
**Sapph**	**(1,6,-5)**	**10.15**	**83.93**	**2.444**	**3.6**	**1.086**	**3.9**	**13.58**	**14.13**
	Equiv. Refl.: (1,-7,5), (6,1,5), (6,-7,-5), (7,-1,5), (7,-6,-5), (-1,7,-5), (-1,-6,5), (-6,7,5), (-6,-1,-5) (-7,1,-5), (-7,6,5)								
**Quartz**	**(2,5,-3)**	**9.728**	**72.37**	**2.427**	**2.4**	**3.243**	**7.76**	**40.54**	**41.27**
	Equiv. Refl.: (2,-7,3), (5,2,3), (5,-7,-3), (-7,5,3)								
**Quartz**	**(-7,2,-3)**	**9.728**	**72.37**	**2.426**	**2.4**	**3.243**	**7.76**	**40.54**	**41.27**
**Quartz**	**(-2,-5,3)**	**9.728**	**72.37**	**2.365**	**2.4**	**3.243**	**7.76**	**40.54**	**41.27**
	Equiv. Refl.: (-5,7,3)								
**Quartz**	**(7,-2,3)**	**9.728**	**72.37**	**2.364**	**2.4**	**3.243**	**7.76**	**40.54**	**41.27**
	Equiv. Refl.: (7,-5,-3), (-2,7,-3), (-5,-2,-3)								
**Quartz**	**(1,6,-2)**	**9.826**	**74.29**	**2.073**	**2.3**	**2.871**	**6.69**	**35.89**	**36.51**
	Equiv. Refl.: (1,-7,2)								
**Quartz**	**(-7,1,-2)**	**9.826**	**74.29**	**2.072**	**2.3**	**2.871**	**6.68**	**35.89**	**36.51**
	Equiv. Refl.: (-7,6,2)								
**Quartz**	**(6,-7,-2)**	**9.826**	**74.29**	**2.071**	**2.3**	**2.871**	**6.68**	**35.89**	**36.51**
**Quartz**	**(6,1,2)**	**9.826**	**74.29**	**2.07**	**2.3**	**2.871**	**6.68**	**35.89**	**36.51**
**Quartz**	**(2,-2,-8)**	**9.628**	**70.6**	**1.932**	**2.2**	**3.594**	**7.93**	**44.92**	**45.62**
	Equiv. Refl.: (-2,2,8)								
**Quartz**	**(2,0,8)**	**9.628**	**70.6**	**1.931**	**2.2**	**3.594**	**7.93**	**44.92**	**45.62**
	Equiv. Refl.: (-2,0,-8)								
**Quartz**	**(0,2,-8)**	**9.628**	**70.6**	**1.93**	**2.2**	**3.594**	**7.93**	**44.92**	**45.61**
	Equiv. Refl.: (0,-2,8)								
**Quartz**	**(7,-1,2)**	**9.826**	**74.29**	**1.904**	**2.3**	**2.871**	**6.68**	**35.89**	**36.51**
	Equiv. Refl.: (-1,7,-2), (-1,-6,2)								
**Quartz**	**(7,-6,-2)**	**9.826**	**74.29**	**1.903**	**2.3**	**2.871**	**6.68**	**35.89**	**36.51**
**Quartz**	**(-6,7,2)**	**9.826**	**74.29**	**1.902**	**2.3**	**2.871**	**6.68**	**35.89**	**36.51**
	Equiv. Refl.: (-6,-1,-2)								
**Sapph**	**(0,4,-16)**	**9.723**	**72.28**	**1.518**	**1.9**	**3.261**	**6.32**	**40.77**	**41.25**
	Equiv. Refl.: (0,-4,16), (4,0,16), (4,-4,-16), (-4,0,-16), (-4,4,16)								
**LiNbO3**	**(1,3,19)**	**9.865**	**75.13**	**1.492**	**3**	**2.71**	**8.24**	**33.87**	**34.86**
	Equiv. Refl.: (1,-4,-19), (3,1,-19), (3,-4,19), (4,-1,-19), (4,-3,19), (-1,4,19), (-1,-3,-19), (-3,4,-19), (-3,-1,19) (-4,1,19), (-4,3,-19)								
**Quartz**	**(0,5,6)**	**10.022**	**79.06**	**1.36**	**1.9**	**1.972**	**3.67**	**24.65**	**24.92**
	Equiv. Refl.: (0,-5,-6)								

Ei =	10.207	keV						ΔEg	ΔEt
**Cryst**	**Refl**	**EB**	**ΘB**	**∫ IR dΘ**	**Width**	**Ei cotΘ**	**ΔE**	**2m, 50µm**	**2m, 50µm**
	**(h,k,l)**	**[keV]**	**[°]**	**[µrad]**	**[µrad]**	**[meV/µrad]**	**[meV]**	**[meV]**	**[meV]**
**Quartz**	**(5,0,-6)**	**10.022**	**79.06**	**1.355**	**1.9**	**1.972**	**3.66**	**24.65**	**24.92**
	Equiv. Refl.: (5,-5,6), (-5,5,-6)								
**Quartz**	**(-5,0,6)**	**10.022**	**79.06**	**1.354**	**1.9**	**1.972**	**3.66**	**24.65**	**24.92**
**LiNbO3**	**(3,4,11)**	**9.784**	**73.45**	**1.341**	**2.7**	**3.032**	**8.32**	**37.9**	**38.81**
	Equiv. Refl.: (3,-7,-11), (4,3,-11), (4,-7,11), (7,-3,-11), (7,-4,11), (-3,7,11), (-3,-4,-11), (-4,7,-11), (-4,-3,11) (-7,3,11), (-7,4,-11)								
**Sapph**	**(1,2,-19)**	**9.904**	**76**	**1.335**	**1.9**	**2.546**	**4.72**	**31.82**	**32.17**
	Equiv. Refl.: (1,-3,19), (2,1,19), (2,-3,-19), (3,-1,19), (3,-2,-19), (-1,3,-19), (-1,-2,19), (-2,3,19), (-2,-1,-19) (-3,1,-19), (-3,2,19)								
**Quartz**	**(1,6,-1)**	**9.623**	**70.52**	**1.263**	**1.5**	**3.611**	**5.52**	**45.13**	**45.47**
	Equiv. Refl.: (6,1,1), (6,-7,-1), (-7,1,-1), (-7,6,1)								
**Quartz**	**(1,-7,1)**	**9.623**	**70.52**	**1.262**	**1.5**	**3.611**	**5.52**	**45.13**	**45.47**
**Quartz**	**(7,-1,1)**	**9.623**	**70.52**	**1.218**	**1.5**	**3.611**	**5.52**	**45.13**	**45.47**
	Equiv. Refl.: (7,-6,-1)								
**Quartz**	**(-1,7,-1)**	**9.623**	**70.52**	**1.217**	**1.5**	**3.611**	**5.52**	**45.13**	**45.47**
	Equiv. Refl.: (-1,-6,1), (-6,7,1), (-6,-1,-1)								
**Quartz**	**(0,2,8)**	**9.628**	**70.6**	**1.187**	**1.5**	**3.594**	**5.33**	**44.92**	**45.24**
	Equiv. Refl.: (0,-2,-8)								
**Quartz**	**(2,-2,8)**	**9.628**	**70.6**	**1.186**	**1.5**	**3.594**	**5.32**	**44.92**	**45.23**
**Quartz**	**(2,0,-8)**	**9.628**	**70.6**	**1.185**	**1.5**	**3.594**	**5.32**	**44.92**	**45.23**
	Equiv. Refl.: (-2,0,8), (-2,2,-8)								
**Sapph**	**(1,6,1)**	**9.877**	**75.38**	**1.12**	**1.6**	**2.662**	**4.3**	**33.27**	**33.55**
	Equiv. Refl.: (1,-7,-1), (6,1,-1), (6,-7,1), (7,-1,-1), (7,-6,1), (-1,7,1), (-1,-6,-1), (-6,7,-1), (-6,-1,1) (-7,1,1), (-7,6,-1)								
**Sapph**	**(2,2,18)**	**10.048**	**79.88**	**0.889**	**1.5**	**1.821**	**2.77**	**22.76**	**22.93**
	Equiv. Refl.: (2,2,-18), (2,-4,18), (2,-4,-18), (4,-2,18), (4,-2,-18), (-2,4,18), (-2,4,-18), (-2,-2,18), (-2,-2,-18) (-4,2,18), (-4,2,-18)								
**LiNbO3**	**(1,5,-13)**	**9.682**	**71.53**	**0.856**	**2**	**3.408**	**6.9**	**42.6**	**43.16**
	Equiv. Refl.: (1,-6,13), (5,1,13), (5,-6,-13), (6,-1,13), (6,-5,-13), (-1,6,-13), (-1,-5,13), (-5,6,13), (-5,-1,-13) (-6,1,-13), (-6,5,13)								
**LiNbO3**	**(2,6,-1)**	**10.037**	**79.53**	**0.713**	**2.3**	**1.887**	**4.35**	**23.59**	**23.99**
	Equiv. Refl.: (2,-8,1), (6,2,1), (6,-8,-1), (8,-2,1), (8,-6,-1), (-2,8,-1), (-2,-6,1), (-6,8,1), (-6,-2,-1) (-8,2,-1), (-8,6,1)								
**Sapph**	**(1,3,-17)**	**9.76**	**72.98**	**0.688**	**1.1**	**3.124**	**3.45**	**39.05**	**39.2**
	Equiv. Refl.: (1,-4,17), (3,1,17), (3,-4,-17), (4,-1,17), (4,-3,-17), (-1,4,-17), (-1,-3,17), (-3,4,17), (-3,-1,-17) (-4,1,-17), (-4,3,17)								
**Quartz**	**(0,6,4)**	**9.873**	**75.3**	**0.486**	**0.8**	**2.678**	**2.22**	**33.48**	**33.55**
	Equiv. Refl.: (0,-6,-4)								
**Quartz**	**(6,0,-4)**	**9.873**	**75.3**	**0.484**	**0.8**	**2.678**	**2.22**	**33.48**	**33.55**
	Equiv. Refl.: (6,-6,4), (-6,0,4), (-6,6,-4)								
**Quartz**	**(-2,7,3)**	**9.728**	**72.37**	**0.432**	**0.7**	**3.243**	**2.23**	**40.54**	**40.6**
	Equiv. Refl.: (-2,-5,-3)								
**Quartz**	**(7,-2,-3)**	**9.728**	**72.37**	**0.431**	**0.7**	**3.243**	**2.23**	**40.54**	**40.6**
	Equiv. Refl.: (7,-5,3), (-5,7,-3), (-5,-2,3)								
**Quartz**	**(2,5,3)**	**9.728**	**72.37**	**0.361**	**0.7**	**3.243**	**2.23**	**40.54**	**40.6**
	Equiv. Refl.: (2,-7,-3)								
**Quartz**	**(5,-7,3)**	**9.728**	**72.37**	**0.36**	**0.7**	**3.243**	**2.23**	**40.54**	**40.6**
	Equiv. Refl.: (-7,2,3), (-7,5,-3)								
**Quartz**	**(5,2,-3)**	**9.728**	**72.37**	**0.359**	**0.7**	**3.243**	**2.22**	**40.54**	**40.6**
**LiNbO3**	**(3,5,-5)**	**9.987**	**78.08**	**0.342**	**1.5**	**2.155**	**3.21**	**26.93**	**27.12**
	Equiv. Refl.: (3,-8,5), (5,3,5), (5,-8,-5), (8,-3,5), (8,-5,-5), (-3,8,-5), (-3,-5,5), (-5,8,5), (-5,-3,-5) (-8,3,-5), (-8,5,5)								

**LiNbO3**	**(2,3,17)**	**9.722**	**72.27**	**0.299**	**1.2**	**3.262**	**3.8**	**40.78**	**40.96**
	Equiv. Refl.: (2,-5,-17), (3,2,-17), (3,-5,17), (5,-2,-17), (5,-3,17), (-2,5,17), (-2,-3,-17), (-3,5,-17), (-3,-2,17) (-5,2,17), (-5,3,-17)								
**Quartz**	**(5,-5,-6)**	**10.022**	**79.06**	**0.283**	**0.7**	**1.972**	**1.31**	**24.65**	**24.69**
	Equiv. Refl.: (-5,5,6)								
**Quartz**	**(5,0,6)**	**10.022**	**79.06**	**0.282**	**0.7**	**1.972**	**1.31**	**24.65**	**24.69**
	Equiv. Refl.: (-5,0,-6)								
**Quartz**	**(0,5,-6)**	**10.022**	**79.06**	**0.279**	**0.7**	**1.972**	**1.3**	**24.65**	**24.69**
	Equiv. Refl.: (0,-5,6)								
**LiNbO3**	**(3,5,1)**	**9.744**	**72.67**	**0.259**	**1.1**	**3.185**	**3.48**	**39.81**	**39.96**
	Equiv. Refl.: (3,-8,-1), (5,3,-1), (5,-8,1), (8,-3,-1), (8,-5,1), (-3,8,1), (-3,-5,-1), (-5,8,-1), (-5,-3,1) (-8,3,1), (-8,5,-1)								
**Sapph**	**(3,4,-7)**	**9.742**	**72.64**	**0.249**	**0.6**	**3.192**	**1.85**	**39.89**	**39.94**
	Equiv. Refl.: (3,-7,7), (4,3,7), (4,-7,-7), (7,-3,7), (7,-4,-7), (-3,7,-7), (-3,-4,7), (-4,7,7), (-4,-3,-7) (-7,3,-7), (-7,4,7)								
**LiNbO3**	**(3,3,15)**	**9.859**	**74.99**	**0.125**	**0.8**	**2.737**	**2.24**	**34.21**	**34.28**
	Equiv. Refl.: (3,3,-15), (3,-6,15), (3,-6,-15), (6,-3,15), (6,-3,-15), (-3,6,15), (-3,6,-15), (-3,-3,15), (-3,-3,-15) (-6,3,15), (-6,3,-15)								
**LiNbO3**	**(4,4,3)**	**9.727**	**72.35**	**0.02**	**0.3**	**3.248**	**1.09**	**40.59**	**40.61**
	Equiv. Refl.: (4,4,-3), (4,-8,3), (4,-8,-3), (8,-4,3), (8,-4,-3), (-4,8,3), (-4,8,-3), (-4,-4,3), (-4,-4,-3) (-8,4,3), (-8,4,-3)								

Ei =	12.527	keV						ΔEg	ΔEt
Cryst	Refl	EB	ΘB	∫IR dΘ	Width	Ei cotΘ	ΔE	2m, 50µm	2m, 50µm
	(h,k,l)	[keV]	[°]	[µrad]	[µrad]	[meV/µrad]	[meV]	[meV]	[meV]
**Si**	**(2,4,10)**	**12.504**	**86.52**	**15.044**	**13.2**	**0.762**	**10.03**	**9.52**	**13.83**
**Ge**	**(0,8,8)**	**12.397**	**81.75**	**5.094**	**10.9**	**1.817**	**19.85**	**22.71**	**30.16**
**Si**	**(3,5,9)**	**12.241**	**77.73**	**3.338**	**2.9**	**2.726**	**7.78**	**34.07**	**34.95**
**Ge**	**(2,4,10)**	**12.004**	**73.38**	**2.942**	**6.1**	**3.739**	**22.79**	**46.74**	**52**
**Si**	**(1,5,9)**	**11.807**	**70.48**	**1.878**	**2**	**4.44**	**9.09**	**55.5**	**56.24**
	Equiv. Refl.: (3,7,7)								
**Ge**	**(1,1,11)**	**12.153**	**75.96**	**1.775**	**4.9**	**3.133**	**15.29**	**39.16**	**42.03**
	Equiv. Refl.: (5,7,7)								
**Quartz**	**(0,8,-4)**	**12.526**	**89.43**	**106.6**	**89.8**	**0.125**	**11.21**	**1.56**	**11.31**
	Equiv. Refl.: (0,-8,4), (8,0,4), (8,-8,-4), (-8,0,-4), (-8,8,4)								
**Quartz**	**(0,8,4)**	**12.526**	**89.43**	**81.1**	**70.2**	**0.125**	**8.77**	**1.56**	**8.9**
	Equiv. Refl.: (0,-8,-4), (8,0,-4), (8,-8,4), (-8,0,4), (-8,8,-4)								
**LiNbO3**	**(1,-6,22)**	**12.519**	**87.93**	**56.8**	**52.4**	**0.453**	**23.7**	**5.66**	**24.36**
	Equiv. Refl.: (5,1,22), (6,-1,22), (-1,-5,22), (-5,6,22), (-6,5,22)								
**LiNbO3**	**(1,5,-22)**	**12.519**	**87.93**	**55.1**	**52.4**	**0.453**	**23.7**	**5.66**	**24.36**
	Equiv. Refl.: (5,-6,-22), (6,-5,-22), (-1,6,-22), (-5,-1,-22), (-6,1,-22)								
**LiNbO3**	**(0,9,0)**	**12.514**	**87.43**	**45**	**41.1**	**0.563**	**23.11**	**7.03**	**24.16**
	Equiv. Refl.: (9,-9,0), (-9,0,0)								
**LiNbO3**	**(0,-9,0)**	**12.514**	**87.43**	**42.5**	**41.1**	**0.563**	**23.11**	**7.03**	**24.16**
	Equiv. Refl.: (9,0,0), (-9,9,0)								
**LiNbO3**	**(1,4,-24)**	**12.481**	**85.11**	**28.2**	**25.7**	**1.073**	**27.61**	**13.41**	**30.7**
	Equiv. Refl.: (1,-5,24), (4,1,24), (4,-5,-24), (5,-1,24), (5,-4,-24), (-1,5,-24), (-1,-4,24), (-4,5,24), (-4,-1,-24) (-5,1,-24), (-5,4,24)								
**LiNbO3**	**(1,4,24)**	**12.481**	**85.11**	**28**	**25.6**	**1.073**	**27.42**	**13.41**	**30.52**
	Equiv. Refl.: (4,-5,24), (5,-4,24), (-1,5,24), (-4,-1,24), (-5,1,24)								
**LiNbO3**	**(1,-5,-24)**	**12.481**	**85.11**	**27.9**	**25.6**	**1.073**	**27.42**	**13.41**	**30.52**
	Equiv. Refl.: (4,1,-24), (5,-1,-24), (-1,-4,-24), (-4,5,-24), (-5,4,-24)								
**LiNbO3**	**(3,7,-4)**	**12.488**	**85.46**	**26.6**	**24.5**	**0.995**	**24.35**	**12.43**	**27.34**
	Equiv. Refl.: (7,-10,-4), (10,-7,-4), (-3,10,-4), (-7,-3,-4), (-10,3,-4)								
**LiNbO3**	**(3,-10,4)**	**12.488**	**85.46**	**26**	**24.5**	**0.995**	**24.35**	**12.43**	**27.34**
	Equiv. Refl.: (7,3,4), (10,-3,4), (-3,-7,4), (-7,10,4), (-10,7,4)								
**LiNbO3**	**(1,8,8)**	**12.407**	**82.07**	**13.8**	**13**	**1.745**	**22.65**	**21.81**	**31.44**
	Equiv. Refl.: (3,-10,-2), (7,3,-2), (8,-9,8), (9,-8,8), (10,-3,-2), (-1,9,8), (-3,-7,-2), (-7,10,-2), (-8,-1,8) (-9,1,8), (-10,7,-2)								
**LiNbO3**	**(1,-9,-8)**	**12.407**	**82.07**	**13.6**	**13**	**1.745**	**22.65**	**21.81**	**31.44**
	Equiv. Refl.: (8,1,-8), (9,-1,-8), (-1,-8,-8), (-8,9,-8), (-9,8,-8)								
**LiNbO3**	**(3,4,20)**	**12.31**	**79.31**	**13.5**	**12.3**	**2.365**	**29.01**	**29.56**	**41.42**
	Equiv. Refl.: (3,-7,-20), (4,3,-20), (4,-7,20), (7,-3,-20), (7,-4,20), (-3,7,20), (-3,-4,-20), (-4,7,-20), (-4,-3,20) (-7,3,20), (-7,4,-20)								
**LiNbO3**	**(3,7,2)**	**12.391**	**81.56**	**13.3**	**12.8**	**1.859**	**23.71**	**23.24**	**33.2**
	Equiv. Refl.: (7,-10,2), (10,-7,2), (-3,10,2), (-7,-3,2), (-10,3,2), (3,5,-14), (3,-8,14), (5,3,14), (5,-8,-14) (8,-3,14), (8,-5,-14), (-3,8,-14), (-3,-5,14), (-5,8,14), (-5,-3,-14), (-8,3,-14), (-8,5,14)								
**Quartz**	**(9,-1,1)**	**12.501**	**86.32**	**13**	**10.7**	**0.805**	**8.6**	**10.06**	**13.24**
	Equiv. Refl.: (9,-8,-1), (-1,9,-1), (-1,-8,1), (-8,9,1), (-8,-1,-1)								
**Quartz**	**(1,8,-1)**	**12.501**	**86.32**	**12.3**	**10.7**	**0.805**	**8.6**	**10.06**	**13.24**
	Equiv. Refl.: (1,-9,1), (8,1,1), (8,-9,-1), (-9,1,-1), (-9,8,1)								
**Quartz**	**(7,-1,7)**	**12.48**	**85.03**	**11.9**	**9.5**	**1.089**	**10.4**	**13.62**	**17.14**
	Equiv. Refl.: (7,-6,-7), (-1,7,-7), (-1,-6,7), (-6,7,7), (-6,-1,-7)								

Ei =	12.527	keV							
**Cryst**	**Refl (h,k,l)**	**EB [keV]**	**ΘB [°]**	**∫ IR dΘ [µrad]**	**Width [µrad]**	**Ei cotΘ [meV/µrad]**	**ΔE [meV]**	**ΔEg 2m, 50µm [meV]**	**ΔEt 2m, 50µm [meV]**
**LiNbO3**	**(2,6,-16)**	**12.318**	**79.52**	**11.6**	**10.5**	**2.318**	**24.45**	**28.97**	**37.91**
	Equiv. Refl.: (6,-8,-16), (8,-6,-16), (-2,8,-16), (-6,-2,-16), (-8,2,-16), (0,6,18), (0,6,-18), (0,-6,18), (0,-6,-18) (6,0,18), (6,0,-18), (6,-6,18), (6,-6,-18), (-6,0,18), (-6,0,-18), (-6,6,18), (-6,6,-18)								
**LiNbO3**	**(3,6,12)**	**12.272**	**78.42**	**11.4**	**10.4**	**2.567**	**26.74**	**32.08**	**41.76**
	Equiv. Refl.: (6,-9,12), (9,-6,12), (-3,9,12), (-6,-3,12), (-9,3,12), (1,6,-7), (1,-7,7), (6,1,7), (6,-7,-7) (-7,1,-7), (-7,6,7)								
**LiNbO3**	**(0,5,22)**	**12.047**	**74.08**	**11.3**	**9.9**	**3.573**	**35.31**	**44.66**	**56.93**
	Equiv. Refl.: (2,-8,16), (5,-5,22), (6,2,16), (8,-2,16), (-2,-6,16), (-5,0,22), (-6,8,16), (-8,6,16)								
**LiNbO3**	**(2,-9,-10)**	**12.229**	**77.47**	**11.2**	**9.9**	**2.784**	**27.66**	**34.8**	**44.46**
	Equiv. Refl.: (3,-9,-12), (6,3,-12), (7,2,-10), (9,-2,-10), (9,-3,-12), (-2,-7,-10), (-3,-6,-12), (-6,9,-12), (-7,9,-10) (-9,6,-12), (-9,7,-10)								
**LiNbO3**	**(0,-5,-22)**	**12.047**	**74.08**	**10.8**	**9.9**	**3.573**	**35.31**	**44.66**	**56.93**
	Equiv. Refl.: (5,0,-22), (5,5,6), (5,-10,6), (10,-5,6), (-5,5,-22), (-5,10,6), (-5,-5,6), (-10,5,6)								
**LiNbO3**	**(2,7,10)**	**12.229**	**77.47**	**10.4**	**9.9**	**2.784**	**27.66**	**34.8**	**44.46**
	Equiv. Refl.: (5,5,0), (5,-10,0), (7,-9,10), (9,-7,10), (10,-5,0), (-2,9,10), (-5,10,0), (-5,-5,0), (-7,-2,10) (-9,2,10), (-10,5,0)								
**LiNbO3**	**(4,6,-2)**	**12.155**	**76**	**10.3**	**9.1**	**3.123**	**28.38**	**39.04**	**48.27**
	Equiv. Refl.: (5,5,-6), (5,-10,-6), (6,-10,-2), (10,-5,-6), (10,-6,-2), (-4,10,-2), (-5,10,-6), (-5,-5,-6), (-6,-4,-2) (-10,4,-2), (-10,5,-6)								
**LiNbO3**	**(3,-9,12)**	**12.272**	**78.42**	**10.1**	**9.2**	**2.567**	**23.62**	**32.08**	**39.84**
	Equiv. Refl.: (6,3,12), (9,-3,12), (-3,-6,12), (-6,9,12), (-9,6,12)								
**LiNbO3**	**(4,-10,2)**	**12.155**	**76**	**10**	**9.1**	**3.123**	**28.38**	**39.04**	**48.27**
	Equiv. Refl.: (6,4,2), (10,-4,2), (-4,-6,2), (-6,10,2), (-10,6,2)								
**LiNbO3**	**(3,6,-12)**	**12.272**	**78.42**	**9.9**	**9.2**	**2.567**	**23.62**	**32.08**	**39.84**
	Equiv. Refl.: (6,-9,-12), (9,-6,-12), (-3,9,-12), (-6,-3,-12), (-9,3,-12)								
**LiNbO3**	**(1,2,26)**	**12.195**	**76.77**	**9.7**	**8.8**	**2.944**	**25.98**	**36.8**	**45.05**
	Equiv. Refl.: (1,-3,-26), (2,1,-26), (2,4,22), (2,-3,26), (2,-6,-22), (3,-1,-26), (3,-2,26), (4,2,-22), (4,-6,22) (6,-2,-22), (6,-4,22), (-1,3,26), (-1,-2,-26), (-2,3,-26), (-2,6,22), (-2,-1,26), (-2,-4,-22), (-3,1,26) (-3,2,-26), (-4,6,-22), (-4,-2,22), (-6,2,22), (-6,4,-22)								
**LiNbO3**	**(4,6,4)**	**12.253**	**78**	**9.5**	**8.8**	**2.663**	**23.32**	**33.28**	**40.64**
	Equiv. Refl.: (4,-10,-4), (6,4,-4), (6,-10,4), (10,-4,-4), (10,-6,4), (-4,10,4), (-4,-6,-4), (-6,10,-4), (-6,-4,4) (-10,4,4), (-10,6,-4)								
**LiNbO3**	**(4,6,7)**	**12.52**	**88.03**	**9.3**	**14.4**	**0.43**	**6.18**	**5.38**	**8.19**
	Equiv. Refl.: (4,-10,-7), (6,4,-7), (6,-10,7), (10,-4,-7), (10,-6,7), (-4,10,7), (-4,-6,-7), (-6,10,-7), (-6,-4,7) (-10,4,7), (-10,6,-7)								
**LiNbO3**	**(0,2,-26)**	**11.955**	**72.61**	**9.1**	**8**	**3.923**	**31.51**	**49.04**	**58.29**
	Equiv. Refl.: (0,-2,26), (2,0,26), (2,-2,-26), (-2,0,-26), (-2,2,26)								
**LiNbO3**	**(1,-9,4)**	**12.014**	**73.55**	**8.6**	**7.6**	**3.699**	**28.01**	**46.23**	**54.06**
	Equiv. Refl.: (8,1,4), (9,-1,4), (-1,-8,4), (-8,9,4), (-9,8,4)								
**Sapph**	**(2,7,4)**	**12.461**	**84.13**	**8.5**	**7.3**	**1.288**	**9.46**	**16.1**	**18.67**
	Equiv. Refl.: (2,-9,-4), (7,2,-4), (7,-9,4), (9,-2,-4), (9,-7,4), (-2,9,4), (-2,-7,-4), (-7,9,-4), (-7,-2,4) (-9,2,4), (-9,7,-4)								
**LiNbO3**	**(3,-8,-16)**	**12.08**	**74.65**	**8.4**	**7.3**	**3.438**	**25.06**	**42.98**	**49.75**
	Equiv. Refl.: (5,3,-16), (8,-3,-16), (-3,-5,-16), (-5,8,-16), (-8,5,-16)								
**LiNbO3**	**(1,8,-4)**	**12.014**	**73.55**	**8.3**	**7.6**	**3.699**	**28.01**	**46.23**	**54.06**
	Equiv. Refl.: (8,-9,-4), (9,-8,-4), (-1,9,-4), (-8,-1,-4), (-9,1,-4)								
**LiNbO3**	**(1,8,2)**	**11.914**	**72**	**8.2**	**7.3**	**4.07**	**29.66**	**50.88**	**58.89**
	Equiv. Refl.: (8,-9,2), (9,-8,2), (-1,9,2), (-8,-1,2), (-9,1,2)								

Ei =	12.527	keV						ΔEg	ΔEt
**Cryst**	**Refl**	**EB**	**ΘB**	**∫ IR dΘ**	**Width**	**Ei cotΘ**	**ΔE**	**2m, 50µm**	**2m, 50µm**
	**(h,k,l)**	**[keV]**	**[°]**	**[µrad]**	**[µrad]**	**[meV/µrad]**	**[meV]**	**[meV]**	**[meV]**
**LiNbO3**	**(1,-9,-2)**	**11.914**	**72**	**8.1**	**7.3**	**4.07**	**29.66**	**50.88**	**58.89**
	Equiv. Refl.: (8,1,-2), (9,-1,-2), (-1,-8,-2), (-8,9,-2), (-9,8,-2), (9,-3,4), (9,-6,-4), (-3,9,-4), (-3,-6,4) (-6,9,4), (-6,-3,-4)								
**LiNbO3**	**(2,6,14)**	**11.821**	**70.67**	**8**	**7**	**4.394**	**30.84**	**54.93**	**62.99**
	Equiv. Refl.: (6,-8,14), (8,-6,14), (-2,8,14), (-6,-2,14), (-8,2,14)								
**LiNbO3**	**(0,8,10)**	**11.989**	**73.15**	**7.9**	**6.8**	**3.794**	**25.86**	**47.43**	**54.02**
	Equiv. Refl.: (2,5,18), (2,-7,-18), (5,2,-18), (5,-7,18), (7,-2,-18), (7,-5,18), (8,-8,10), (-2,7,18), (-2,-5,-18) (-5,7,-18), (-5,-2,18), (-7,2,18), (-7,5,-18), (-8,0,10)								
**LiNbO3**	**(2,-8,-14)**	**11.821**	**70.67**	**7.8**	**7**	**4.394**	**30.84**	**54.93**	**62.99**
	Equiv. Refl.: (6,2,-14), (8,-2,-14), (-2,-6,-14), (-6,8,-14), (-8,6,-14)								
**LiNbO3**	**(0,-7,16)**	**12.08**	**74.65**	**7.7**	**7**	**3.438**	**24.16**	**42.98**	**49.31**
	Equiv. Refl.: (7,0,16), (-7,7,16), (2,4,-20), (2,-6,20), (4,2,20), (4,-6,-20), (6,-2,20), (6,-4,-20), (-2,6,-20) (-2,-4,20), (-4,6,20), (-4,-2,-20), (-6,2,-20), (-6,4,20), (3,6,-4), (3,-9,4), (6,3,4), (6,-9,-4) (-9,3,-4), (-9,6,4)								
**LiNbO3**	**(2,-7,18)**	**11.84**	**70.94**	**7.6**	**6.8**	**4.328**	**29.41**	**54.1**	**61.57**
	Equiv. Refl.: (5,2,18), (7,-2,18), (-2,-5,18), (-5,7,18), (-7,5,18)								
**LiNbO3**	**(0,7,-16)**	**12.08**	**74.65**	**7.5**	**7**	**3.438**	**24.16**	**42.98**	**49.31**
	Equiv. Refl.: (1,-8,12), (2,5,-18), (3,5,16), (5,-7,-18), (5,-8,16), (7,1,12), (7,-5,-18), (7,-7,-16), (8,-1,12) (8,-5,16), (-1,-7,12), (-2,7,-18), (-3,8,16), (-5,-2,-18), (-5,-3,16), (-7,0,-16), (-7,2,-18), (-7,8,12) (-8,3,16), (-8,7,12), (8,-3,-6), (8,-5,6), (-3,8,6), (-3,-5,-6), (-5,8,-6), (-5,-3,6)								
**LiNbO3**	**(1,7,-12)**	**11.79**	**70.25**	**7.4**	**6.7**	**4.498**	**29.97**	**56.23**	**63.72**
	Equiv. Refl.: (7,-8,-12), (8,-7,-12), (-1,8,-12), (-7,-1,-12), (-8,1,-12), (3,5,6), (3,-8,-6), (5,3,-6), (5,-8,6) (-8,3,6), (-8,5,-6)								
**Quartz**	**(9,-1,0)**	**12.448**	**83.58**	**7.2**	**6**	**1.409**	**8.45**	**17.61**	**19.54**
	Equiv. Refl.: (9,-8,0), (-1,9,0), (-1,-8,0), (-8,9,0), (-8,-1,0)								
**Sapph**	**(2,6,-13)**	**12.498**	**86.07**	**7.1**	**6.8**	**0.86**	**5.82**	**10.75**	**12.22**
	Equiv. Refl.: (2,-8,13), (6,2,13), (6,-8,-13), (8,-2,13), (8,-6,-13), (-2,8,-13), (-2,-6,13), (-6,8,13), (-6,-2,-13) (-8,2,-13), (-8,6,13)								
**LiNbO3**	**(0,-8,-10)**	**11.989**	**73.15**	**7**	**6.8**	**3.794**	**25.86**	**47.43**	**54.02**
	Equiv. Refl.: (8,0,-10), (-8,8,-10), (1,8,0), (1,-9,0), (8,1,0), (8,-9,0), (-9,1,0), (-9,8,0)								
**LiNbO3**	**(1,5,20)**	**11.829**	**70.78**	**6.9**	**6.3**	**4.367**	**27.38**	**54.58**	**61.07**
	Equiv. Refl.: (1,-6,-20), (5,1,-20), (5,-6,20), (6,-1,-20), (6,-5,20), (-1,6,20), (-1,-5,-20), (-5,6,-20), (-5,-1,20) (-6,1,20), (-6,5,-20)								
**LiNbO3**	**(2,-9,8)**	**11.931**	**72.25**	**6.8**	**6.1**	**4.01**	**24.39**	**50.13**	**55.74**
	Equiv. Refl.: (7,2,8), (9,-2,8), (-2,-7,8), (-7,9,8), (-9,7,8)								
**LiNbO3**	**(1,7,12)**	**11.79**	**70.25**	**6.7**	**6**	**4.498**	**27.11**	**56.23**	**62.42**
	Equiv. Refl.: (7,-8,12), (8,-7,12), (-1,8,12), (-7,-1,12), (-8,1,12)								
**LiNbO3**	**(1,-8,-12)**	**11.79**	**70.25**	**6.6**	**6**	**4.498**	**27.11**	**56.23**	**62.42**
	Equiv. Refl.: (7,1,-12), (8,-1,-12), (-1,-7,-12), (-7,8,-12), (-8,7,-12)								
**LiNbO3**	**(2,7,-8)**	**11.931**	**72.25**	**6.4**	**6.1**	**4.01**	**24.39**	**50.13**	**55.74**
	Equiv. Refl.: (7,-9,-8), (9,-7,-8), (-2,9,-8), (-7,-2,-8), (-9,2,-8)								
**Sapph**	**(0,1,26)**	**12.5**	**86.27**	**6.3**	**6**	**0.817**	**4.93**	**10.21**	**11.34**
	Equiv. Refl.: (0,-1,-26), (1,0,-26), (1,-1,26), (-1,0,26), (-1,1,-26)								
**Quartz**	**(2,7,3)**	**12.412**	**82.25**	**6.2**	**5.2**	**1.706**	**8.82**	**21.32**	**23.07**
	Equiv. Refl.: (2,-9,-3), (7,2,-3), (7,-9,3), (-9,2,3), (-9,7,-3)								
**Quartz**	**(9,-2,-3)**	**12.412**	**82.25**	**6.1**	**5.2**	**1.706**	**8.82**	**21.32**	**23.07**
	Equiv. Refl.: (9,-7,3), (-2,9,3), (-2,-7,-3), (-7,9,-3), (-7,-2,3)								
**Sapph**	**(3,6,0)**	**11.941**	**72.41**	**5.8**	**4.6**	**3.973**	**18.2**	**49.66**	**52.89**
	Equiv. Refl.: (3,-9,0), (6,3,0), (6,-9,0), (9,-3,0), (9,-6,0), (-3,9,0), (-3,-6,0), (-6,9,0), (-6,-3,0) (-9,3,0), (-9,6,0), (3,6,4), (3,-9,-4), (6,3,-4), (6,-9,4), (-3,9,4), (-3,-6,-4), (-9,3,4) (-9,6,-4)								

Ei =	12.527	keV						ΔEg	ΔEt
**Cryst**	**Refl**	**EB**	**ΘB**	**∫ IR dΘ**	**Width**	**Ei cotΘ**	**ΔE**	**2m, 50µm**	**2m, 50µm**
	**(h,k,l)**	**[keV]**	**[°]**	**[µrad]**	**[µrad]**	**[meV/µrad]**	**[meV]**	**[meV]**	**[meV]**
**Quartz**	**(9,-3,-4)**	**12.441**	**83.3**	**5.7**	**5**	**1.472**	**7.31**	**18.4**	**19.8**
	Equiv. Refl.: (9,-6,4), (-6,9,-4), (-6,-3,4)								
**Quartz**	**(8,-1,-5)**	**12.405**	**82.01**	**5.5**	**4.5**	**1.759**	**7.98**	**21.99**	**23.39**
	Equiv. Refl.: (8,-7,5), (-1,8,5), (-1,-7,-5), (-7,8,-5), (-7,-1,5)								
**Quartz**	**(1,7,5)**	**12.405**	**82.01**	**5.4**	**4.5**	**1.759**	**7.98**	**21.99**	**23.39**
	Equiv. Refl.: (1,7,-5), (1,-8,5), (1,-8,-5), (5,1,-8), (5,-6,8), (7,1,5), (7,1,-5), (7,-8,5), (7,-8,-5) (-8,1,5), (-8,1,-5), (-8,7,5), (-8,7,-5)								
**Sapph**	**(0,8,4)**	**12.186**	**76.6**	**5.3**	**4.4**	**2.985**	**13.11**	**37.31**	**39.55**
	Equiv. Refl.: (0,-8,-4), (8,0,-4), (8,-8,4), (-8,0,4), (-8,8,-4), (1,5,8), (1,-6,-8), (6,-1,-8), (6,-5,8) (8,-1,5), (8,-7,-5), (-1,6,8), (-1,8,-5), (-1,-5,-8), (-1,-7,5), (-5,6,-8), (-5,-1,8), (-6,1,8) (-6,5,-8), (-7,8,5), (-7,-1,-5)								
**Quartz**	**(0,3,10)**	**12.275**	**78.48**	**4.9**	**4**	**2.552**	**10.09**	**31.91**	**33.46**
	Equiv. Refl.: (0,-3,-10), (3,0,-10), (3,-3,10), (-3,0,10), (-3,3,-10)								
**Quartz**	**(4,5,-4)**	**12.27**	**78.36**	**4.8**	**3.8**	**2.58**	**9.84**	**32.25**	**33.71**
	Equiv. Refl.: (4,-9,4), (5,4,4), (5,-9,-4), (7,-2,-7), (7,-5,7), (9,-4,4), (9,-5,-4), (-2,7,7), (-2,-5,-7) (-4,9,-4), (-4,-5,4), (-5,7,-7), (-5,9,4), (-5,-2,7), (-5,-4,-4), (-9,4,-4), (-9,5,4)								
**Quartz**	**(2,5,7)**	**12.135**	**75.63**	**4.7**	**3.8**	**3.21**	**12.18**	**40.13**	**41.93**
	Equiv. Refl.: (2,-7,-7), (5,2,-7), (5,-7,7), (-7,2,7), (-7,5,-7)								
**LiNbO3**	**(1,6,19)**	**12.463**	**84.21**	**4.4**	**6**	**1.271**	**7.65**	**15.89**	**17.63**
	Equiv. Refl.: (1,-7,-19), (6,1,-19), (6,-7,19), (7,-1,-19), (7,-6,19), (-1,7,19), (-1,-6,-19), (-6,7,-19), (-6,-1,19) (-7,1,19), (-7,6,-19)								
**Sapph**	**(4,5,-4)**	**11.904**	**71.85**	**4.3**	**3.4**	**4.106**	**13.85**	**51.32**	**53.15**
	Equiv. Refl.: (4,-9,4), (5,4,4), (5,-9,-4), (9,-4,4), (9,-5,-4), (-4,9,-4), (-4,-5,4), (-5,9,4), (-5,-4,-4) (-9,4,-4), (-9,5,4)								
**Sapph**	**(1,6,16)**	**12.476**	**84.81**	**4.1**	**4**	**1.139**	**4.57**	**14.23**	**14.95**
	Equiv. Refl.: (1,-7,-16), (6,1,-16), (6,-7,16), (7,-1,-16), (7,-6,16), (-1,7,16), (-1,-6,-16), (-6,7,-16), (-6,-1,16) (-7,1,16), (-7,6,-16)								
**Quartz**	**(1,6,7)**	**12.48**	**85.03**	**3.8**	**3.4**	**1.089**	**3.68**	**13.62**	**14.11**
	Equiv. Refl.: (1,-7,-7), (6,1,-7), (6,-7,7), (-7,1,7), (-7,6,-7)								
**Quartz**	**(7,-1,-7)**	**12.48**	**85.03**	**3.6**	**3.4**	**1.089**	**3.68**	**13.62**	**14.11**
	Equiv. Refl.: (7,-6,7), (-1,7,7), (-1,-6,-7), (-6,7,-7), (-6,-1,7)								
**Quartz**	**(2,3,9)**	**12.12**	**75.36**	**3.2**	**2.5**	**3.272**	**8.22**	**40.91**	**41.72**
	Equiv. Refl.: (2,-5,-9), (3,2,-9), (3,-5,9), (5,-2,-9), (5,-3,9), (-2,5,9), (-2,-3,-9), (-3,5,-9), (-3,-2,9) (-5,2,9), (-5,3,-9)								
**Quartz**	**(0,6,7)**	**11.87**	**71.36**	**3.1**	**2.5**	**4.227**	**10.48**	**52.83**	**53.86**
	Equiv. Refl.: (0,-6,-7), (3,4,-7), (3,-7,7), (4,3,7), (4,-7,-7), (6,0,-7), (6,-6,7), (-6,0,7), (-6,6,-7) (-7,3,-7), (-7,4,7)								
**Quartz**	**(2,3,-9)**	**12.12**	**75.36**	**3**	**2.3**	**3.272**	**7.67**	**40.91**	**41.62**
	Equiv. Refl.: (2,7,1), (2,-5,9), (2,-9,-1), (3,2,9), (3,-5,-9), (5,-2,9), (5,-3,-9), (7,2,-1), (7,-3,7) (7,-4,-7), (7,-9,1), (9,-2,2), (9,-7,-2), (-2,5,-9), (-2,9,-2), (-2,-3,9), (-2,-7,2), (-3,5,9) (-3,7,-7), (-3,-2,-9), (-3,-4,7), (-4,7,7), (-4,-3,-7), (-5,2,-9), (-5,3,9), (-7,9,2), (-7,-2,-2) (-9,2,1), (-9,7,-1)								
**Sapph**	**(1,4,21)**	**12.165**	**76.2**	**2.9**	**2.4**	**3.078**	**7.52**	**38.47**	**39.2**
	Equiv. Refl.: (1,4,-21), (1,-5,21), (1,-5,-21), (4,1,21), (4,1,-21), (4,-5,21), (4,-5,-21), (5,-1,21), (5,-1,-21) (5,-4,21), (5,-4,-21), (-1,5,21), (-1,5,-21), (-1,-4,21), (-1,-4,-21), (-4,5,21), (-4,5,-21), (-4,-1,21) (-4,-1,-21), (-5,1,21), (-5,1,-21), (-5,4,21), (-5,4,-21), (1,7,-4), (1,-8,4), (2,7,-2), (2,-9,2) (7,1,4), (7,2,2), (7,-8,-4), (7,-9,-2), (-8,1,-4), (-8,7,4), (-9,2,-2), (-9,7,2)								
**Quartz**	**(3,6,-2)**	**11.79**	**70.25**	**2.8**	**2.2**	**4.499**	**9.68**	**56.23**	**57.06**
	Equiv. Refl.: (3,-9,2), (6,3,2), (6,-9,-2), (8,-1,4), (8,-7,-4), (9,-2,-1), (9,-3,2), (9,-6,-2), (9,-7,1) (-1,8,-4), (-1,-7,4), (-2,9,1), (-2,-7,-1), (-3,9,-2), (-3,-6,2), (-6,9,2), (-6,-3,-2), (-7,8,4) (-7,9,-1), (-7,-1,-4), (-7,-2,1), (-9,3,-2), (-9,6,2)								

Ei =	12.527	keV						ΔEg	ΔEt
**Cryst**	**Refl**	**EB**	**ΘB**	**∫ IR dΘ**	**Width**	**Ei cotΘ**	**ΔE**	**2m, 50µm**	**2m, 50µm**
	**(h,k,l)**	**[keV]**	**[°]**	**[µrad]**	**[µrad]**	**[meV/µrad]**	**[meV]**	**[meV]**	**[meV]**
**Sapph**	**(0,5,-20)**	**12.153**	**75.97**	**2.7**	**2.6**	**3.13**	**8.07**	**39.12**	**39.95**
	Equiv. Refl.: (0,-5,20), (5,0,20), (5,-5,-20), (-5,0,-20), (-5,5,20)								
**LiNbO3**	**(2,7,-11)**	**12.399**	**81.81**	**2.6**	**4**	**1.804**	**7.22**	**22.55**	**23.68**
	Equiv. Refl.: (2,-9,11), (7,2,11), (7,-9,-11), (9,-2,11), (9,-7,-11), (-2,9,-11), (-2,-7,11), (-7,9,11), (-7,-2,-11) (-9,2,-11), (-9,7,11)								
**Sapph**	**(1,5,-19)**	**12.345**	**80.22**	**2.5**	**2.5**	**2.159**	**5.36**	**26.98**	**27.51**
	Equiv. Refl.: (1,-6,19), (5,1,19), (5,-6,-19), (6,-1,19), (6,-5,-19), (-1,6,-19), (-1,-5,19), (-5,6,19), (-5,-1,-19) (-6,1,-19), (-6,5,19)								
**Sapph**	**(1,6,-14)**	**11.915**	**72.02**	**2.4**	**2.1**	**4.066**	**8.63**	**50.82**	**51.55**
	Equiv. Refl.: (1,-7,14), (6,1,14), (6,-7,-14), (7,-1,14), (7,-6,-14), (-1,7,-14), (-1,-6,14), (-6,7,14), (-6,-1,-14) (-7,1,-14), (-7,6,14), (1,2,10), (1,2,-10), (1,-3,10), (2,1,10), (2,1,-10), (2,-3,10), (2,-3,-10) (3,-1,-10), (3,-2,10), (5,4,-4), (5,-9,4), (8,-2,-5), (8,-6,5), (9,-4,-4), (9,-5,4), (-1,3,10) (-1,-2,-10), (-2,3,-10), (-2,8,5), (-2,-1,10), (-2,-6,-5), (-3,1,10), (-3,1,-10), (-3,2,10), (-3,2,-10) (-4,9,4), (-4,-5,-4), (-5,9,-4), (-5,-4,4), (-6,8,-5), (-6,-2,5), (-9,4,4)								
**LiNbO3**	**(1,1,27)**	**12.312**	**79.36**	**2.3**	**3.5**	**2.353**	**8.12**	**29.42**	**30.52**
	Equiv. Refl.: (1,1,-27), (1,-2,27), (1,-2,-27), (2,-1,27), (2,-1,-27), (-1,2,27), (-1,2,-27), (-1,-1,27), (-1,-1,-27) (-2,1,27), (-2,1,-27), (1,-3,-10), (2,6,5), (2,-8,-5), (4,5,4), (4,-9,-4), (6,2,-5), (6,-8,5) (-8,2,5), (-8,6,-5), (-9,5,-4)								
**Sapph**	**(4,5,-7)**	**12.216**	**77.2**	**2**	**2.2**	**2.846**	**6.25**	**35.58**	**36.12**
	Equiv. Refl.: (4,-9,7), (5,4,7), (5,-9,-7), (9,-4,7), (9,-5,-7), (-4,9,-7), (-4,-5,7), (-5,9,7), (-5,-4,-7) (-9,4,-7), (-9,5,7)								
**Quartz**	**(3,6,2)**	**11.79**	**70.25**	**1.9**	**2**	**4.499**	**8.81**	**56.23**	**56.92**
	Equiv. Refl.: (3,-1,10), (3,-2,-10), (3,-9,-2), (6,3,-2), (6,-9,2), (9,-3,-2), (9,-6,2), (-1,3,-10), (-1,-2,10) (-2,3,10), (-2,-1,-10), (-3,9,2), (-3,-6,-2), (-6,9,-2), (-6,-3,2), (-9,3,2), (-9,6,-2)								
**Sapph**	**(2,6,-10)**	**11.852**	**71.11**	**1.8**	**2**	**4.287**	**8.37**	**53.59**	**54.24**
	Equiv. Refl.: (2,-8,10), (6,2,10), (6,-8,-10), (8,-2,10), (8,-6,-10), (-2,8,-10), (-2,-6,10), (-6,8,10), (-6,-2,-10) (-8,2,-10), (-8,6,10), (2,7,-1), (2,-9,1), (4,4,6), (4,4,-6), (4,-8,6), (4,-8,-6), (7,2,1) (7,-9,-1), (9,-2,1), (9,-3,3), (9,-6,-3), (9,-7,-1), (-2,9,-1), (-2,-7,1), (-3,9,-3), (-3,-6,3) (-6,9,3), (-6,-3,-3), (-7,9,1), (-7,-2,-1), (-8,4,6), (-8,4,-6), (-9,2,-1), (-9,7,1)								
**LiNbO3**	**(4,6,-5)**	**12.326**	**79.73**	**1.7**	**2.9**	**2.269**	**6.48**	**28.36**	**29.09**
	Equiv. Refl.: (4,-10,5), (6,4,5), (6,-10,-5), (10,-4,5), (10,-6,-5), (-4,10,-5), (-4,-6,5), (-6,10,5), (-6,-4,-5) (-10,4,-5), (-10,6,5), (2,6,11), (2,-8,-11), (6,2,-11), (6,-8,11), (8,-2,-11), (8,-6,11), (-2,8,11) (-2,-6,-11), (-6,8,-11), (-6,-2,11), (-8,2,11), (-8,6,-11), (0,4,-9), (0,-4,9), (2,5,-7), (2,-7,7) (3,3,8), (3,3,-8), (3,6,-3), (3,-6,8), (3,-6,-8), (3,-9,3), (4,0,9), (4,-4,-9), (5,2,7) (5,-7,-7), (6,3,3), (6,-9,-3), (7,-2,7), (7,-5,-7), (8,-4,6), (8,-4,-6), (-2,7,-7), (-2,-5,7) (-4,0,-9), (-4,4,9), (-4,8,6), (-4,8,-6), (-4,-4,6), (-4,-4,-6), (-5,7,7), (-5,-2,-7), (-6,3,8) (-6,3,-8), (-7,2,-7), (-7,5,7), (-9,3,-3), (-9,6,3)								
**Sapph**	**(0,7,14)**	**12.472**	**84.63**	**1.6**	**2.2**	**1.178**	**2.58**	**14.72**	**14.95**
	Equiv. Refl.: (0,-7,-14), (4,5,5), (4,-9,-5), (5,4,-5), (5,-9,5), (7,0,-14), (7,-7,14), (9,-4,-5), (9,-5,5) (-4,9,5), (-4,-5,-5), (-5,9,-5), (-5,-4,5), (-7,0,14), (-7,7,-14), (-9,4,5), (-9,5,-5), (1,7,4) (1,8,1), (1,-8,-4), (1,-9,-1), (6,-3,8), (6,-3,-8), (7,1,-4), (7,-1,6), (7,-6,-6), (7,-8,4) (8,1,-1), (8,-9,1), (9,-3,-3), (9,-6,3), (-1,7,-6), (-1,-6,6), (-3,6,8), (-3,6,-8), (-3,9,3) (-3,-3,8), (-3,-3,-8), (-3,-6,-3), (-6,7,6), (-6,9,-3), (-6,-1,-6), (-6,-3,3), (-8,1,4), (-8,7,-4) (-9,1,1), (-9,8,-1)								
**Quartz**	**(1,6,6)**	**11.775**	**70.04**	**1.5**	**1.5**	**4.549**	**6.74**	**56.86**	**57.26**
	Equiv. Refl.: (1,6,-6), (1,-7,6), (1,-7,-6), (3,4,7), (3,5,-6), (3,6,3), (3,-7,-7), (3,-8,6), (3,-9,-3) (4,3,-7), (4,-7,7), (5,3,6), (5,-8,-6), (6,1,6), (6,1,-6), (6,3,-3), (6,-7,6), (6,-7,-6) (6,-9,3), (7,-3,-7), (7,-4,7), (8,-1,-4), (8,-7,4), (-1,8,4), (-1,-7,-4), (-3,7,7), (-3,-4,-7) (-4,7,-7), (-4,-3,7), (-7,1,6), (-7,1,-6), (-7,3,7), (-7,4,-7), (-7,6,6), (-7,6,-6), (-7,8,-4) (-7,-1,4), (-8,3,-6), (-8,5,6), (-9,3,3), (-9,6,-3)								

Ei =	12.527	keV						ΔEg	ΔEt
Cryst	Refl	EB	ΘB	∫ IR dΘ	Width	Ei cotΘ	ΔE	2m, 50µm	2m, 50µm
	(h,k,l)	[keV]	[°]	[µrad]	[µrad]	[meV/µrad]	[meV]	[meV]	[meV]
**LiNbO3**	**(1,6,-17)**	**11.871**	**71.38**	**1.4**	**2.1**	**4.22**	**8.91**	**52.76**	**53.5**
	Equiv. Refl.: (1,-7,17), (6,1,17), (6,-7,-17), (7,-1,17), (7,-6,-17), (-1,7,-17), (-1,-6,17), (-6,7,17), (-6,-1,-17) (-7,1,-17), (-7,6,17), (1,7,9), (1,7,-9), (1,-8,9), (1,-8,-9), (2,7,1), (2,-9,-1), (7,1,9) (7,1,-9), (7,2,-1), (7,-8,9), (7,-8,-9), (7,-9,1), (8,-1,9), (8,-1,-9), (8,-7,9), (8,-7,-9) (9,-2,-1), (9,-7,1), (-1,8,9), (-1,8,-9), (-1,-7,9), (-1,-7,-9), (-2,9,1), (-2,-7,-1), (-7,8,9) (-7,8,-9), (-7,9,-1), (-7,-1,9), (-7,-1,-9), (-7,-2,1), (-8,1,9), (-8,1,-9), (-8,7,9), (-8,7,-9) (-9,2,1), (-9,7,-1), (1,4,9), (1,-5,-9), (2,4,-8), (2,-6,8), (4,1,-9), (4,2,8), (4,-5,9) (4,-6,-8), (5,-1,-9), (5,-4,9), (-1,5,9), (-1,-4,-9), (-4,5,-9), (-4,-1,9), (-5,1,9), (-5,4,-9) (-6,2,-8), (-6,4,8)								
**LiNbO3**	**(4,6,1)**	**12.13**	**75.54**	**1.3**	**2.1**	**3.23**	**6.82**	**40.38**	**40.95**
	Equiv. Refl.: (4,-10,-1), (6,4,-1), (6,-10,1), (10,-4,-1), (10,-6,1), (-4,10,1), (-4,-6,-1), (-6,10,-1), (-6,-4,1) (-10,4,1), (-10,6,-1), (2,5,15), (2,5,-15), (2,-7,15), (2,-7,-15), (5,2,15), (5,2,-15), (5,-7,15) (5,-7,-15), (7,-2,15), (7,-2,-15), (7,-5,15), (7,-5,-15), (-2,7,15), (-2,7,-15), (-2,-5,15), (-2,-5,-15) (-5,7,15), (-5,7,-15), (-5,-2,15), (-5,-2,-15), (-7,2,15), (-7,2,-15), (-7,5,15), (-7,5,-15), (6,-2,8) (6,-4,-8), (7,-1,-6), (7,-6,6), (8,-3,6), (8,-5,-6), (-1,7,6), (-1,-6,-6), (-2,6,-8), (-2,-4,8) (-3,8,-6), (-3,-5,6), (-4,6,8), (-4,-2,-8), (-5,8,6), (-5,-3,-6), (-6,7,-6), (-6,-1,6)								
**LiNbO3**	**(2,7,7)**	**11.804**	**70.44**	**1.2**	**1.9**	**4.45**	**8.43**	**55.63**	**56.26**
	Equiv. Refl.: (2,-9,-7), (7,2,-7), (7,-9,7), (9,-2,-7), (9,-7,7), (-2,9,7), (-2,-7,-7), (-7,9,-7), (-7,-2,7) (-9,2,7), (-9,7,-7), (0,4,-22), (0,-4,22), (3,6,6), (3,6,-6), (3,-9,6), (3,-9,-6), (4,0,22) (4,-4,-22), (6,3,6), (6,3,-6), (6,-9,6), (6,-9,-6), (9,-3,6), (9,-3,-6), (9,-6,6), (9,-6,-6) (-3,9,6), (-3,9,-6), (-3,-6,6), (-3,-6,-6), (-4,0,-22), (-4,4,22), (-6,9,6), (-6,9,-6), (-6,-3,6) (-6,-3,-6), (-9,3,6), (-9,3,-6), (-9,6,6), (-9,6,-6), (0,4,9), (0,-4,-9), (4,0,-9), (4,-4,9) (-4,0,9), (-4,4,-9)								
**Sapph**	**(2,3,-22)**	**12.38**	**81.21**	**1.1**	**1.4**	**1.937**	**2.78**	**24.21**	**24.37**
	Equiv. Refl.: (2,-5,22), (3,2,22), (3,-5,-22), (5,-2,22), (5,-3,-22), (-2,5,-22), (-2,-3,22), (-3,5,22), (-3,-2,-22) (-5,2,-22), (-5,3,22), (2,7,2), (2,-9,-2), (4,5,3), (4,-9,-3), (5,4,-3), (5,-9,3), (7,2,-2) (7,-9,2), (9,-1,-1), (9,-2,-2), (9,-7,2), (9,-8,1), (-1,9,1), (-1,-8,-1), (-2,9,2), (-2,-7,-2) (-7,9,-2), (-7,-2,2), (-8,9,-1), (-8,-1,1), (-9,2,2), (-9,4,3), (-9,5,-3), (-9,7,-2)								
**LiNbO3**	**(1,3,25)**	**12.252**	**77.97**	**1**	**1.9**	**2.668**	**4.99**	**33.36**	**33.73**
	Equiv. Refl.: (1,-4,-25), (3,1,-25), (3,-4,25), (4,-1,-25), (4,-3,25), (-1,4,25), (-1,-3,-25), (-3,4,-25), (-3,-1,25) (-4,1,25), (-4,3,-25), (2,4,19), (2,7,-2), (2,-6,-19), (2,-9,2), (3,5,13), (3,-8,-13), (4,2,-19) (4,-6,19), (5,3,-13), (5,-8,13), (6,-2,-19), (6,-4,19), (7,2,2), (7,-9,-2), (8,-3,-13), (8,-5,13) (9,-2,2), (9,-7,-2), (-2,6,19), (-2,9,-2), (-2,-4,-19), (-2,-7,2), (-3,8,13), (-3,-5,-13), (-4,6,-19) (-4,-2,19), (-5,8,-13), (-5,-3,13), (-6,2,19), (-6,4,-19), (-7,9,2), (-7,-2,-2), (-8,3,13), (-8,5,-13) (-9,2,-2), (-9,7,2), (0,2,-10), (0,-2,10), (2,0,10), (2,-2,-10), (9,-4,-3), (9,-5,3), (-2,0,-10) (-2,2,10), (-4,9,3), (-4,-5,-3), (-5,9,-3), (-5,-4,3)								
**LiNbO3**	**(4,5,-13)**	**12.318**	**79.52**	**0.9**	**1.9**	**2.317**	**4.4**	**28.96**	**29.29**
	Equiv. Refl.: (4,-9,13), (5,4,13), (5,-9,-13), (9,-4,13), (9,-5,-13), (-4,9,-13), (-4,-5,13), (-5,9,13), (-5,-4,-13) (-9,4,-13), (-9,5,13), (0,8,-3), (0,-8,3), (8,0,3), (8,-8,-3), (-8,0,-3), (-8,8,3)								
**LiNbO3**	**(3,7,-1)**	**12.367**	**80.83**	**0.8**	**1.8**	**2.021**	**3.66**	**25.27**	**25.53**
	Equiv. Refl.: (3,-10,1), (7,3,1), (7,-10,-1), (10,-3,1), (10,-7,-1), (-3,10,-1), (-3,-7,1), (-7,10,1), (-7,-3,-1) (-10,3,-1), (-10,7,1), (0,8,2), (0,-8,-2), (4,5,-3), (4,-9,3), (5,4,3), (5,-9,-3), (8,0,-2) (8,-8,2), (9,-4,-3), (9,-5,3), (-4,9,-3), (-4,-5,3), (-5,9,3), (-5,-4,-3), (-8,0,2), (-8,8,-2) (-9,4,-3), (-9,5,3)								
**LiNbO3**	**(3,4,-19)**	**11.989**	**73.14**	**0.7**	**1.4**	**3.796**	**5.25**	**47.45**	**47.74**
	Equiv. Refl.: (3,-7,19), (4,3,19), (4,-7,-19), (7,-3,19), (7,-4,-19), (-3,7,-19), (-3,-4,19), (-4,7,19), (-4,-3,-19) (-7,3,-19), (-7,4,19), (0,3,-10), (0,-3,10), (3,0,10), (3,-3,-10), (8,-2,5), (8,-6,-5), (-2,8,-5) (-2,-6,5), (-3,0,-10), (-3,3,10), (-6,8,5), (-6,-2,-5)								

Ei =	12.527	keV						ΔEg	ΔEt
Cryst	Refl	EB	ΘB	∫ IR dΘ	Width	Ei cotΘ	ΔE	2m, 50µm	2m, 50µm
	(h,k,l)	[keV]	[°]	[µrad]	[µrad]	[meV/µrad]	[meV]	[meV]	[meV]
**LiNbO3**	**(4,5,11)**	**11.922**	**72.12**	**0.6**	**1.2**	**4.041**	**4.88**	**50.51**	**50.74**
	Equiv. Refl.: (4,-9,-11), (5,4,-11), (5,-9,11), (9,-4,-11), (9,-5,11), (-4,9,11), (-4,-5,-11), (-5,9,-11), (-5,-4,11)<br>(-9,4,11), (-9,5,-11), (4,5,8), (4,-9,-8), (5,4,-8), (5,-9,8), (9,-4,-8), (9,-5,8), (-4,9,8)<br>(-4,-5,-8), (-5,9,-8), (-5,-4,8), (-9,4,8), (-9,5,-8), (1,4,-9), (1,-5,9), (2,6,-5), (2,-8,5)<br>(4,1,9), (4,-5,-9), (5,-1,9), (5,-4,-9), (6,2,5), (6,-8,-5), (9,-2,3), (9,-7,-3), (-1,5,-9)<br>(-1,-4,9), (-2,9,-3), (-2,-7,3), (-4,5,9), (-4,-1,-9), (-5,1,-9), (-5,4,9), (-7,9,3), (-7,-2,-3)<br>(-8,2,-5), (-8,6,5)								
**Sapph**	**(1,3,22)**	**11.819**	**70.64**	**0.5**	**0.6**	**4.402**	**2.81**	**55.02**	**55.09**
	Equiv. Refl.: (1,-4,-22), (3,1,-22), (3,-4,22), (4,-1,-22), (4,-3,22), (-1,4,22), (-1,-3,-22), (-3,4,-22), (-3,-1,22)<br>(-4,1,22), (-4,3,-22), (0,6,-7), (0,-6,7), (1,5,-8), (1,-6,8), (2,4,8), (2,7,-3), (2,-6,-8)<br>(2,-9,3), (4,2,-8), (4,-6,8), (5,1,8), (5,-6,-8), (6,0,7), (6,-1,8), (6,-2,-8), (6,-4,8)<br>(6,-5,-8), (6,-6,-7), (7,2,3), (7,-9,-3), (-1,6,-8), (-1,-5,8), (-2,6,8), (-2,-4,-8), (-4,6,-8)<br>(-4,-2,8), (-5,6,8), (-5,-1,-8), (-6,0,-7), (-6,1,-8), (-6,2,8), (-6,4,-8), (-6,5,8), (-6,6,7)<br>(-9,2,-3), (-9,7,3)								
**LiNbO3**	**(1,7,15)**	**12.458**	**83.98**	**0.4**	**1.6**	**1.321**	**2.13**	**16.51**	**16.65**
	Equiv. Refl.: (1,7,-15), (1,-8,15), (1,-8,-15), (7,1,15), (7,1,-15), (7,-8,15), (7,-8,-15), (8,-1,15), (8,-1,-15)<br>(8,-7,15), (8,-7,-15), (-1,8,15), (-1,8,-15), (-1,-7,15), (-1,-7,-15), (-7,8,15), (-7,8,-15), (-7,-1,15)<br>(-7,-1,-15), (-8,1,15), (-8,1,-15), (-8,7,15), (-8,7,-15), (0,3,24), (0,3,-24), (0,-3,24), (0,-3,-24)<br>(1,3,-23), (1,-4,23), (3,0,24), (3,0,-24), (3,1,23), (3,4,-16), (3,-3,24), (3,-3,-24), (3,-4,-23)<br>(3,-7,16), (4,3,16), (4,-1,23), (4,-3,-23), (4,-7,-16), (7,-3,16), (7,-4,-16), (-1,4,-23), (-1,-3,23)<br>(-3,0,24), (-3,0,-24), (-3,3,24), (-3,3,-24), (-3,4,23), (-3,7,-16), (-3,-1,-23), (-3,-4,16), (-4,1,-23)<br>(-4,3,23), (-4,7,16), (-4,-3,-16), (-7,3,-16), (-7,4,16)								
**Quartz**	**(0,2,10)**	**11.835**	**70.86**	**0.3**	**0.4**	**4.348**	**1.67**	**54.34**	**54.37**
	Equiv. Refl.: (0,8,-2), (0,-2,-10), (0,-8,2), (2,0,-10), (2,7,0), (2,-2,10), (2,-9,0), (7,2,0), (7,-9,0)<br>(8,0,2), (8,-8,-2), (9,-2,0), (9,-7,0), (-2,0,10), (-2,2,-10), (-2,9,0), (-2,-7,0), (-7,9,0)<br>(-7,-2,0), (-8,0,-2), (-8,8,2), (-9,2,0), (-9,7,0)								
**LiNbO3**	**(1,8,-7)**	**12.286**	**78.74**	**0.2**	**0.7**	**2.494**	**1.78**	**31.18**	**31.23**
	Equiv. Refl.: (1,-9,7), (2,3,23), (2,-5,-23), (3,2,-23), (3,5,-17), (3,-5,23), (3,-8,17), (5,3,17), (5,-2,-23)<br>(5,-3,23), (5,-8,-17), (8,1,7), (8,-3,17), (8,-5,-17), (8,-9,-7), (9,-1,7), (9,-8,-7), (-1,9,-7)<br>(-1,-8,7), (-2,5,23), (-2,-3,-23), (-3,5,-23), (-3,8,-17), (-3,-2,23), (-3,-5,17), (-5,2,23), (-5,3,-23)<br>(-5,8,17), (-5,-3,-17), (-8,3,-17), (-8,5,17), (-8,9,7), (-8,-1,-7), (-9,1,-7), (-9,8,7), (3,4,17)<br>(3,6,3), (3,6,-3), (3,-7,-17), (3,-9,3), (3,-9,-3), (4,3,-17), (4,-7,17), (6,3,3), (6,3,-3)<br>(6,-9,3), (6,-9,-3), (7,-3,-17), (7,-4,17), (9,-3,3), (9,-3,-3), (9,-6,3), (9,-6,-3), (-3,7,17)<br>(-3,9,3), (-3,9,-3), (-3,-4,-17), (-3,-6,3), (-3,-6,-3), (-4,7,-17), (-4,-3,17), (-6,9,3), (-6,9,-3)<br>(-6,-3,3), (-6,-3,-3), (-7,3,17), (-7,4,-17), (-9,3,3), (-9,3,-3), (-9,6,3), (-9,6,-3)								
**LiNbO3**	**(1,8,5)**	**12.089**	**74.8**	**0.1**	**0.5**	**3.403**	**1.87**	**42.54**	**42.58**
	Equiv. Refl.: (1,8,-1), (1,-9,1), (1,-9,-5), (3,3,21), (3,3,-21), (3,-6,21), (3,-6,-21), (6,-3,21), (6,-3,-21)<br>(8,1,1), (8,1,-5), (8,-9,5), (8,-9,-1), (9,-1,1), (9,-1,-5), (9,-8,5), (9,-8,-1), (-1,9,5)<br>(-1,9,-1), (-1,-8,1), (-1,-8,-5), (-3,6,21), (-3,6,-21), (-3,-3,21), (-3,-3,-21), (-6,3,21), (-6,3,-21)<br>(-8,9,1), (-8,9,-5), (-8,-1,5), (-8,-1,-1), (-9,1,5), (-9,1,-1), (-9,8,1), (-9,8,-5)								
**LiNbO3**	**(5,5,3)**	**12.116**	**75.29**	**0**	**0.2**	**3.288**	**0.54**	**41.11**	**41.11**
	Equiv. Refl.: (5,5,-3), (5,-10,3), (5,-10,-3), (10,-5,3), (10,-5,-3), (-5,10,3), (-5,10,-3), (-5,-5,3), (-5,-5,-3)<br>(-10,5,3), (-10,5,-3), (4,4,12), (4,4,-12), (4,5,2), (4,-8,12), (4,-8,-12), (4,-9,-2), (5,4,-2)<br>(5,-9,2), (8,-4,12), (8,-4,-12), (9,-4,-2), (9,-5,2), (-4,8,12), (-4,8,-12), (-4,9,2), (-4,-4,12)<br>(-4,-4,-12), (-4,-5,-2), (-5,9,-2), (-5,-4,2), (-8,4,12), (-8,4,-12), (-9,4,2), (-9,5,-2)								

Ei =	11.959	keV						ΔEg	ΔEt
Cryst	Refl	EB	ΘB	∫ IR dΘ	Width	Ei cotΘ	ΔE	2m, 50µm	2m, 50µm
	(h,k,l)	[keV]	[°]	[µrad]	[µrad]	[meV/µrad]	[meV]	[meV]	[meV]
Si	(0,2,10)	11.64	76.75	5.604	4.6	2.817	13.08	35.21	37.56
	Equiv. Refl.: (2,6,8)								
Si	(1,5,9)	11.807	80.86	5.011	4.5	1.924	8.7	24.05	25.57
	Equiv. Refl.: (3,7,7)								
Si	(1,7,7)	11.357	71.75	3.103	2.6	3.944	10.26	49.3	50.36
	Equiv. Refl.: (3,3,9), (5,5,7)								
Ge	(3,5,9)	11.751	79.3	2.448	6.9	2.26	15.5	28.26	32.23
Ge	(1,5,9)	11.335	71.41	1.693	4.5	4.023	18.11	50.29	53.45
	Equiv. Refl.: (3,7,7)								
Quartz	(4,3,7)	11.959	89.6	143.9	121.4	0.083	10.07	1.04	10.12
	Equiv. Refl.: (4,-7,-7)								
Quartz	(3,4,-7)	11.959	89.6	143.8	121.3	0.083	10.06	1.04	10.11
	Equiv. Refl.: (3,-7,7), (-7,3,-7), (-7,4,7)								
Quartz	(-4,7,7)	11.959	89.6	137.7	121.4	0.083	10.07	1.04	10.12
	Equiv. Refl.: (-4,-3,-7)								
Quartz	(7,-3,7)	11.959	89.6	137.6	121.3	0.083	10.06	1.04	10.12
	Equiv. Refl.: (-3,-4,7)								
Quartz	(7,-4,-7)	11.959	89.6	137.5	121.3	0.083	10.06	1.04	10.11
	Equiv. Refl.: (-3,7,-7)								
LiNbO3	(0,-2,26)	11.955	88.44	101.5	92.9	0.326	30.28	4.08	30.56
	Equiv. Refl.: (2,0,26), (-2,2,26)								
LiNbO3	(0,2,-26)	11.955	88.44	100.9	92.9	0.326	30.28	4.08	30.56
	Equiv. Refl.: (2,-2,-26), (-2,0,-26)								
Quartz	(7,-3,-7)	11.959	89.6	78	71.6	0.083	5.93	1.04	6.02
	Equiv. Refl.: (-3,7,7), (-3,-4,-7), (-4,7,-7), (-4,-3,7)								
Quartz	(7,-4,7)	11.959	89.6	77.9	71.5	0.083	5.93	1.04	6.02
Quartz	(3,4,7)	11.959	89.6	75.2	71.6	0.083	5.94	1.04	6.03
	Equiv. Refl.: (3,-7,-7), (4,3,-7), (4,-7,7), (-7,3,7)								
Quartz	(-7,4,-7)	11.959	89.6	75.1	71.5	0.083	5.93	1.04	6.02
Sapph	(3,6,0)	11.941	86.85	31.7	26.5	0.658	17.42	8.22	19.26
	Equiv. Refl.: (3,-9,0), (6,3,0), (6,-9,0), (9,-3,0), (9,-6,0), (-3,9,0), (-3,-6,0), (-6,9,0), (-6,-3,0) (-9,3,0), (-9,6,0)								
LiNbO3	(2,-9,8)	11.931	86.05	30.7	28.4	0.826	23.47	10.32	25.64
	Equiv. Refl.: (7,2,8), (9,-2,8), (-2,-7,8), (-7,9,8), (-9,7,8)								
LiNbO3	(1,8,2)	11.914	85.03	29.8	27.4	1.041	28.52	13.01	31.35
	Equiv. Refl.: (8,-9,2), (9,-8,2), (-1,9,2), (-8,-1,2), (-9,1,2)								
LiNbO3	(1,-9,-2)	11.914	85.03	29.3	27.4	1.041	28.52	13.01	31.35
	Equiv. Refl.: (8,1,-2), (9,-1,-2), (-1,-8,-2), (-8,9,-2), (-9,8,-2)								
LiNbO3	(2,7,-8)	11.931	86.05	28.6	28.4	0.826	23.47	10.32	25.64
	Equiv. Refl.: (7,-9,-8), (9,-7,-8), (-2,9,-8), (-7,-2,-8), (-9,2,-8)								
LiNbO3	(2,5,18)	11.84	81.92	18.7	17.2	1.697	29.25	21.21	36.14
	Equiv. Refl.: (5,-7,18), (7,-5,18), (-2,7,18), (-5,-2,18), (-7,2,18)								
LiNbO3	(2,-7,-18)	11.84	81.92	18.6	17.2	1.697	29.25	21.21	36.14
	Equiv. Refl.: (5,2,-18), (7,-2,-18), (-2,-5,-18), (-5,7,-18), (-7,5,-18)								
LiNbO3	(2,-7,18)	11.84	81.92	18	16.7	1.697	28.28	21.21	35.36
	Equiv. Refl.: (5,2,18), (7,-2,18), (-2,-5,18), (-5,7,18), (-7,5,18)								

Ei = Cryst	11.959 Refl (h,k,l)	keV EB [keV]	ΘB [°]	∫ IR dΘ [µrad]	Width [µrad]	Ei cotΘ [meV/µrad]	ΔE [meV]	ΔEg 2m, 50µm [meV]	ΔEt 2m, 50µm [meV]
**LiNbO3**	**(2,5,-18)**	**11.84**	**81.92**	**17.8**	**16.7**	**1.697**	**28.28**	**21.21**	**35.36**
	Equiv. Refl.: (2,6,14), (5,-7,-18), (6,-8,14), (7,-5,-18), (8,-6,14), (-2,7,-18), (-2,8,14), (-5,-2,-18), (-6,-2,14) (-7,2,-18), (-8,2,14)								
**LiNbO3**	**(2,-8,-14)**	**11.821**	**81.28**	**17.2**	**16.2**	**1.833**	**29.64**	**22.92**	**37.47**
	Equiv. Refl.: (6,2,-14), (8,-2,-14), (-2,-6,-14), (-6,8,-14), (-8,6,-14)								
**LiNbO3**	**(2,2,24)**	**11.764**	**79.63**	**16**	**14.6**	**2.189**	**32.04**	**27.36**	**42.13**
	Equiv. Refl.: (2,2,-24), (2,-4,24), (2,-4,-24), (4,-2,24), (4,-2,-24), (-2,4,24), (-2,4,-24), (-2,-2,24), (-2,-2,-24) (-4,2,24), (-4,2,-24)								
**LiNbO3**	**(0,8,-8)**	**11.685**	**77.71**	**15.8**	**13.9**	**2.605**	**36.09**	**32.56**	**48.61**
	Equiv. Refl.: (1,5,20), (5,-6,20), (6,-5,20), (8,-8,-8), (-1,6,20), (-5,-1,20), (-6,1,20), (-8,0,-8)								
**LiNbO3**	**(1,-6,-20)**	**11.829**	**81.54**	**15.7**	**14.8**	**1.778**	**26.34**	**22.23**	**34.47**
	Equiv. Refl.: (5,1,-20), (6,-1,-20), (-1,-5,-20), (-5,6,-20), (-6,5,-20)								
**LiNbO3**	**(1,-8,12)**	**11.79**	**80.35**	**15.4**	**14.2**	**2.033**	**28.82**	**25.41**	**38.42**
	Equiv. Refl.: (7,1,12), (8,-1,12), (-1,-7,12), (-7,8,12), (-8,7,12)								
**LiNbO3**	**(1,7,-12)**	**11.79**	**80.35**	**15.1**	**14.2**	**2.033**	**28.82**	**25.41**	**38.42**
	Equiv. Refl.: (7,-8,-12), (8,-7,-12), (-1,8,-12), (-7,-1,-12), (-8,1,-12)								
**LiNbO3**	**(0,-8,8)**	**11.685**	**77.71**	**14.9**	**13.9**	**2.605**	**36.09**	**32.56**	**48.61**
	Equiv. Refl.: (8,0,8), (-8,8,8)								
**Sapph**	**(4,5,-4)**	**11.904**	**84.5**	**13.8**	**11.5**	**1.151**	**13.26**	**14.39**	**19.56**
	Equiv. Refl.: (4,-9,4), (5,4,4), (5,-9,-4), (9,-4,4), (9,-5,-4), (-4,9,-4), (-4,-5,4), (-5,9,4), (-5,-4,-4) (-9,4,-4), (-9,5,4)								
**LiNbO3**	**(1,7,12)**	**11.79**	**80.35**	**13.7**	**12.8**	**2.033**	**26.08**	**25.41**	**36.41**
	Equiv. Refl.: (7,-8,12), (8,-7,12), (-1,8,12), (-7,-1,12), (-8,1,12)								
**LiNbO3**	**(4,5,-10)**	**11.745**	**79.14**	**13.6**	**12.3**	**2.295**	**28.26**	**28.69**	**40.27**
	Equiv. Refl.: (5,-9,-10), (9,-5,-10), (-4,9,-10), (-5,-4,-10), (-9,4,-10)								
**LiNbO3**	**(1,-8,-12)**	**11.79**	**80.35**	**13.5**	**12.8**	**2.033**	**26.08**	**25.41**	**36.41**
	Equiv. Refl.: (7,1,-12), (8,-1,-12), (-1,-7,-12), (-7,8,-12), (-8,7,-12)								
**LiNbO3**	**(4,-9,10)**	**11.745**	**79.14**	**12.7**	**12.3**	**2.295**	**28.26**	**28.69**	**40.27**
	Equiv. Refl.: (5,4,10), (9,-4,10), (-4,-5,10), (-5,9,10), (-9,5,10)								
**LiNbO3**	**(0,-6,18)**	**11.593**	**75.79**	**12.3**	**11.1**	**3.029**	**33.66**	**37.87**	**50.67**
	Equiv. Refl.: (2,-5,22), (3,2,22), (5,-2,22), (6,0,18), (-2,-3,22), (-3,5,22), (-5,3,22), (-6,6,18)								
**LiNbO3**	**(0,6,-18)**	**11.593**	**75.79**	**12.1**	**11.1**	**3.029**	**33.66**	**37.87**	**50.67**
	Equiv. Refl.: (6,-6,-18), (-6,0,-18)								
**LiNbO3**	**(3,-8,14)**	**11.573**	**75.4**	**11.9**	**10.4**	**3.115**	**32.46**	**38.93**	**50.69**
	Equiv. Refl.: (5,3,14), (8,-3,14), (-3,-5,14), (-5,8,14), (-8,5,14)								
**LiNbO3**	**(0,-8,-4)**	**11.267**	**70.41**	**11.8**	**10.3**	**4.256**	**44**	**53.2**	**69.04**
	Equiv. Refl.: (2,3,-22), (3,-5,-22), (5,-3,-22), (8,0,-4), (-2,-5,-22), (-3,-2,-22), (-5,2,-22), (-8,8,-4)								
**LiNbO3**	**(0,1,26)**	**11.709**	**78.27**	**11.7**	**10.7**	**2.482**	**26.65**	**31.03**	**40.9**
	Equiv. Refl.: (1,-1,26), (-1,0,26)								
**LiNbO3**	**(0,8,4)**	**11.267**	**70.41**	**11.5**	**10.3**	**4.256**	**44**	**53.2**	**69.04**
	Equiv. Refl.: (0,-1,-26), (1,0,-26), (8,-8,4), (-1,1,-26), (-8,0,4)								
**LiNbO3**	**(0,6,18)**	**11.593**	**75.79**	**11.3**	**10.2**	**3.029**	**30.86**	**37.87**	**48.85**
	Equiv. Refl.: (6,-6,18), (-6,0,18)								
**LiNbO3**	**(2,-6,20)**	**11.581**	**75.56**	**11.2**	**10.2**	**3.08**	**31.43**	**38.5**	**49.7**
	Equiv. Refl.: (4,2,20), (6,-2,20), (-2,-4,20), (-4,6,20), (-6,4,20)								
**LiNbO3**	**(1,6,16)**	**11.59**	**75.73**	**11**	**9.9**	**3.041**	**30.2**	**38.02**	**48.55**
	Equiv. Refl.: (6,-7,16), (7,-6,16), (-1,7,16), (-6,-1,16), (-7,1,16)								
**LiNbO3**	**(2,4,-20)**	**11.581**	**75.56**	**10.9**	**10.2**	**3.08**	**31.43**	**38.5**	**49.7**
	Equiv. Refl.: (3,5,-14), (4,-6,-20), (5,-8,-14), (6,-4,-20), (8,-5,-14), (-2,6,-20), (-3,8,-14), (-4,-2,-20), (-5,-3,-14) (-6,2,-20), (-8,3,-14)								
**LiNbO3**	**(0,-6,-18)**	**11.593**	**75.79**	**10.8**	**10.2**	**3.029**	**30.86**	**37.87**	**48.85**
	Equiv. Refl.: (6,0,-18), (-6,6,-18)								

Ei =	11.959	keV						ΔEg	ΔEt
**Cryst**	**Refl**	**EB**	**ΘB**	**∫ IR dΘ**	**Width**	**Ei cotΘ**	**ΔE**	**2m, 50µm**	**2m, 50µm**
	**(h,k,l)**	**[keV]**	**[°]**	**[µrad]**	**[µrad]**	**[meV/µrad]**	**[meV]**	**[meV]**	**[meV]**
**LiNbO3**	**(1,-7,-16)**	**11.59**	**75.73**	**10.7**	**9.9**	**3.041**	**30.2**	**38.02**	**48.55**
	Equiv. Refl.: (2,7,-2), (2,-9,2), (6,1,-16), (7,2,2), (7,-1,-16), (7,-9,-2), (9,-2,2), (9,-7,-2), (-1,-6,-16) (-2,9,-2), (-2,-7,2), (-6,7,-16), (-7,6,-16), (-7,9,2), (-7,-2,-2), (-9,2,-2), (-9,7,2)								
**Quartz**	**(1,7,-4)**	**11.918**	**85.28**	**10.1**	**8.6**	**0.987**	**8.48**	**12.34**	**14.97**
	Equiv. Refl.: (1,-8,4), (7,1,4), (7,-8,-4), (-8,1,-4), (-8,7,4)								
**Sapph**	**(1,1,24)**	**11.748**	**79.21**	**9.8**	**7.9**	**2.279**	**17.99**	**28.48**	**33.69**
	Equiv. Refl.: (1,1,-24), (1,-2,24), (1,-2,-24), (2,-1,24), (2,-1,-24), (-1,2,24), (-1,2,-24), (-1,-1,24), (-1,-1,-24) (-2,1,24), (-2,1,-24)								
**LiNbO3**	**(0,-7,-14)**	**11.573**	**75.4**	**9.7**	**8.9**	**3.115**	**27.65**	**38.93**	**47.75**
	Equiv. Refl.: (7,0,-14), (-7,7,-14), (8,-1,4), (8,-7,-4), (-1,8,-4), (-1,-7,4), (-7,8,4), (-7,-1,-4)								
**LiNbO3**	**(0,7,14)**	**11.573**	**75.4**	**9.5**	**8.9**	**3.115**	**27.65**	**38.93**	**47.75**
	Equiv. Refl.: (3,6,-6), (3,-9,6), (6,3,6), (6,-9,-6), (7,-7,14), (9,-3,6), (9,-6,-6), (-3,9,-6), (-3,-6,6) (-6,9,6), (-6,-3,-6), (-7,0,14), (-9,3,-6), (-9,6,6)								
**LiNbO3**	**(2,7,4)**	**11.521**	**74.45**	**9.2**	**8.2**	**3.328**	**27.28**	**41.59**	**49.74**
	Equiv. Refl.: (7,-9,4), (9,-7,4), (-2,9,4), (-7,-2,4), (-9,2,4), (0,7,-10), (0,-7,10), (7,0,10), (7,-7,-10) (-7,0,-10), (-7,7,10)								
**Sapph**	**(1,6,-14)**	**11.915**	**85.09**	**9**	**8.1**	**1.027**	**8.27**	**12.83**	**15.27**
	Equiv. Refl.: (1,-7,14), (6,1,14), (6,-7,-14), (7,-1,14), (7,-6,-14), (-1,7,-14), (-1,-6,14), (-6,7,14), (-6,-1,-14) (-7,1,-14), (-7,6,14)								
**LiNbO3**	**(3,-9,-6)**	**11.358**	**71.76**	**8.9**	**7.9**	**3.941**	**31.11**	**49.26**	**58.26**
	Equiv. Refl.: (6,3,-6), (9,-3,-6), (-3,-6,-6), (-6,9,-6), (-9,6,-6)								
**LiNbO3**	**(2,-9,-4)**	**11.521**	**74.45**	**8.6**	**8.2**	**3.328**	**27.28**	**41.59**	**49.74**
	Equiv. Refl.: (3,6,6), (6,-9,6), (7,2,-4), (9,-2,-4), (9,-6,6), (-2,-7,-4), (-3,9,6), (-6,-3,6), (-7,9,-4) (-9,3,6), (-9,7,-4)								
**LiNbO3**	**(0,4,-22)**	**11.301**	**70.91**	**8.1**	**7.3**	**4.139**	**30.17**	**51.74**	**59.89**
	Equiv. Refl.: (4,-4,-22), (-4,0,-22), (0,6,7), (0,-6,-7), (6,0,-7), (6,-6,7), (-6,0,7), (-6,6,-7)								
**LiNbO3**	**(0,-4,22)**	**11.301**	**70.91**	**7.8**	**7.3**	**4.139**	**30.17**	**51.74**	**59.89**
	Equiv. Refl.: (4,0,22), (4,5,8), (5,-9,8), (9,-5,8), (-4,4,22), (-4,9,8), (-5,-4,8), (-9,4,8)								
**LiNbO3**	**(4,-9,-8)**	**11.434**	**72.96**	**7.5**	**7.1**	**3.665**	**26.11**	**45.81**	**52.73**
	Equiv. Refl.: (5,4,-8), (9,-4,-8), (-4,-5,-8), (-5,9,-8), (-9,5,-8)								
**Quartz**	**(0,7,-5)**	**11.701**	**78.07**	**7.4**	**5.8**	**2.526**	**14.65**	**31.58**	**34.81**
	Equiv. Refl.: (0,-7,5), (7,0,5), (7,-7,-5), (-7,0,-5), (-7,7,5)								
**LiNbO3**	**(0,-3,-24)**	**11.514**	**74.33**	**7.2**	**6.6**	**3.355**	**22.27**	**41.94**	**47.48**
	Equiv. Refl.: (3,0,-24), (-3,3,-24), (1,7,4), (1,-8,-4), (7,1,-4), (7,-8,4), (-8,1,4), (-8,7,-4)								
**LiNbO3**	**(0,5,-20)**	**11.328**	**71.3**	**7.1**	**6.3**	**4.047**	**25.31**	**50.59**	**56.57**
	Equiv. Refl.: (5,-5,-20), (-5,0,-20)								
**LiNbO3**	**(0,3,-24)**	**11.514**	**74.33**	**7**	**6.6**	**3.355**	**22.01**	**41.94**	**47.36**
	Equiv. Refl.: (3,-3,-24), (-3,0,-24)								
**Quartz**	**(8,-1,-4)**	**11.918**	**85.28**	**6.9**	**6.4**	**0.987**	**6.33**	**12.34**	**13.87**
	Equiv. Refl.: (8,-7,4), (-1,8,4), (-1,-7,-4), (-7,8,-4), (-7,1,4)								
**Sapph**	**(3,3,18)**	**11.615**	**76.23**	**6.7**	**5.6**	**2.93**	**16.3**	**36.63**	**40.09**
	Equiv. Refl.: (3,3,-18), (3,-6,18), (3,-6,-18), (6,-3,18), (6,-3,-18), (-3,6,18), (-3,6,-18), (-3,-3,18), (-3,-3,-18) (-6,3,18), (-6,3,-18)								
**LiNbO3**	**(0,3,24)**	**11.514**	**74.33**	**6.6**	**6.6**	**3.355**	**22.27**	**41.94**	**47.48**
	Equiv. Refl.: (3,-3,24), (-3,0,24)								
**LiNbO3**	**(0,-3,24)**	**11.514**	**74.33**	**6.5**	**6.6**	**3.355**	**22.01**	**41.94**	**47.36**
	Equiv. Refl.: (3,0,24), (-3,3,24)								
**Quartz**	**(3,3,8)**	**11.896**	**84.12**	**6.4**	**5.6**	**1.231**	**6.85**	**15.39**	**16.85**
	Equiv. Refl.: (3,3,-8), (3,-6,8), (3,-6,-8), (-6,3,8), (-6,3,-8)								

Ei =	11.959	keV						ΔEg	ΔEt
**Cryst**	**Refl**	**EB**	**ΘB**	**∫ IR dΘ**	**Width**	**Ei cotΘ**	**ΔE**	**2m, 50µm**	**2m, 50µm**
	**(h,k,l)**	**[keV]**	**[°]**	**[µrad]**	**[µrad]**	**[meV/µrad]**	**[meV]**	**[meV]**	**[meV]**
**LiNbO3**	**(0,-5,20)**	**11.328**	**71.3**	**6.2**	**6.3**	**4.047**	**25.31**	**50.59**	**56.57**
	Equiv. Refl.: (5,0,20), (-5,5,20)								
**Sapph**	**(0,4,20)**	**11.284**	**70.66**	**6.1**	**4.8**	**4.197**	**20.33**	**52.46**	**56.26**
	Equiv. Refl.: (0,-4,-20), (4,0,-20), (4,-4,20), (-4,0,20), (-4,4,-20)								
**Sapph**	**(1,7,6)**	**11.714**	**78.37**	**6**	**5.1**	**2.461**	**12.58**	**30.76**	**33.23**
	Equiv. Refl.: (1,7,-6), (1,-8,6), (1,-8,-6), (7,1,6), (7,1,-6), (7,-8,6), (7,-8,-6), (8,-1,6), (8,-1,-6) (8,-7,6), (8,-7,-6), (-1,8,6), (-1,8,-6), (-1,-7,6), (-1,-7,-6), (-7,8,6), (-7,8,-6), (-7,-1,6) (-7,-1,-6), (-8,1,6), (-8,1,-6), (-8,7,6), (-8,7,-6), (6,-3,8), (6,-3,-8), (-3,6,8), (-3,6,-8) (-3,-3,8), (-3,-3,-8)								
**Sapph**	**(2,6,-10)**	**11.852**	**82.33**	**5.7**	**5**	**1.61**	**8.02**	**20.13**	**21.67**
	Equiv. Refl.: (2,-8,10), (6,2,10), (6,-8,-10), (8,-2,10), (8,-6,-10), (-2,8,-10), (-2,-6,10), (-6,8,10), (-6,-2,-10) (-8,2,-10), (-8,6,10)								
**Quartz**	**(3,6,-2)**	**11.79**	**80.35**	**5.6**	**4.6**	**2.033**	**9.26**	**25.42**	**27.05**
	Equiv. Refl.: (3,-9,2), (6,3,2), (6,-9,-2), (-9,3,-2), (-9,6,2)								
**Quartz**	**(0,4,-9)**	**11.855**	**82.43**	**5.5**	**4.6**	**1.59**	**7.37**	**19.88**	**21.2**
	Equiv. Refl.: (0,-4,9), (4,0,9), (4,-4,-9), (-4,0,-9), (-4,4,9)								
**Quartz**	**(9,-3,2)**	**11.79**	**80.35**	**5.4**	**4.6**	**2.033**	**9.26**	**25.42**	**27.05**
	Equiv. Refl.: (9,-6,-2), (-3,9,-2), (-3,-6,2), (-6,9,2), (-6,-3,-2)								
**Quartz**	**(0,8,-1)**	**11.712**	**78.34**	**5.1**	**4.1**	**2.468**	**10.09**	**30.85**	**32.46**
	Equiv. Refl.: (0,-8,1), (8,0,1), (8,-8,-1), (-8,0,-1), (-8,8,1)								
**Quartz**	**(3,6,2)**	**11.79**	**80.35**	**5**	**4.1**	**2.033**	**8.43**	**25.42**	**26.78**
	Equiv. Refl.: (3,-9,-2), (6,3,-2), (6,-9,2), (9,-3,-2), (9,-6,2), (-3,9,2), (-3,-6,-2), (-6,9,-2), (-6,-3,2) (-9,3,2), (-9,6,-2)								
**Quartz**	**(4,-1,-9)**	**11.583**	**75.59**	**4.9**	**3.8**	**3.072**	**11.71**	**38.4**	**40.15**
	Equiv. Refl.: (4,-3,9), (-1,4,9), (-1,-3,-9), (-3,4,-9), (-3,-1,9)								
**Quartz**	**(1,3,9)**	**11.583**	**75.59**	**4.8**	**3.8**	**3.072**	**11.7**	**38.4**	**40.14**
	Equiv. Refl.: (1,-4,-9), (3,1,-9), (3,-4,9), (-4,1,9), (-4,3,-9)								
**Quartz**	**(0,1,-10)**	**11.562**	**75.2**	**4.7**	**3.7**	**3.159**	**11.63**	**39.49**	**41.17**
	Equiv. Refl.: (0,-1,10), (1,0,10), (-1,0,-10)								
**Quartz**	**(1,-1,-10)**	**11.562**	**75.2**	**4.6**	**3.7**	**3.159**	**11.62**	**39.49**	**41.16**
	Equiv. Refl.: (-1,1,10)								
**Quartz**	**(0,1,10)**	**11.562**	**75.2**	**4.5**	**3.6**	**3.159**	**11.28**	**39.49**	**41.07**
	Equiv. Refl.: (0,-1,-10), (1,0,-10), (1,-1,10), (-1,0,10), (-1,1,-10)								
**Quartz**	**(1,3,-9)**	**11.583**	**75.59**	**4.4**	**3.5**	**3.072**	**10.75**	**38.4**	**39.88**
	Equiv. Refl.: (1,-4,9), (3,1,9), (3,-4,-9), (4,-1,9), (4,-3,-9), (-1,4,-9), (-1,-3,9), (-3,4,9), (-3,-1,-9) (-4,1,-9), (-4,3,9)								
**LiNbO3**	**(1,6,-17)**	**11.871**	**83.06**	**4.2**	**5.8**	**1.456**	**8.52**	**18.2**	**20.09**
	Equiv. Refl.: (1,-7,17), (6,1,17), (6,-7,-17), (7,-1,17), (7,-6,-17), (-1,7,-17), (-1,-6,17), (-6,7,17), (-6,-1,-17) (-7,1,-17), (-7,6,17)								
**Sapph**	**(1,5,-16)**	**11.335**	**71.41**	**4.1**	**3.3**	**4.023**	**13.31**	**50.28**	**52.01**
	Equiv. Refl.: (1,-6,16), (5,1,16), (5,-6,-16), (6,-1,16), (6,-5,-16), (-1,6,-16), (-1,-5,16), (-5,6,16), (-5,-1,-16) (-6,1,-16), (-6,5,16)								
**Quartz**	**(4,5,-2)**	**11.608**	**76.09**	**4**	**3.2**	**2.962**	**9.39**	**37.02**	**38.19**
	Equiv. Refl.: (4,-9,2), (5,4,2), (5,-9,-2), (7,-1,6), (7,-6,-6), (-1,7,-6), (-1,-6,6), (-6,7,6), (-6,-1,-6) (-9,4,-2), (-9,5,2)								
**Quartz**	**(4,-2,9)**	**11.491**	**73.92**	**3.9**	**3.1**	**3.448**	**10.54**	**43.1**	**44.37**
	Equiv. Refl.: (4,-2,-9), (9,-4,-1), (9,-5,1), (-2,4,9), (-2,4,-9), (-2,-2,9), (-2,-2,-9), (-4,9,1), (-4,-5,-1) (-5,9,-1), (-5,-4,1)								
**Quartz**	**(4,5,3)**	**11.888**	**83.77**	**3.8**	**3.6**	**1.306**	**4.66**	**16.32**	**16.98**
	Equiv. Refl.: (4,-9,-3), (5,4,-3), (5,-9,3), (-9,4,3), (-9,5,-3)								

Ei =	11.959	keV						ΔEg	ΔEt
**Cryst**	**Refl**	**EB**	**ΘB**	**∫ IR dΘ**	**Width**	**Ei cotΘ**	**ΔE**	**2m, 50µm**	**2m, 50µm**
	**(h,k,l)**	**[keV]**	**[°]**	**[µrad]**	**[µrad]**	**[meV/µrad]**	**[meV]**	**[meV]**	**[meV]**
**Quartz**	**(1,6,6)**	**11.775**	**79.93**	**3.7**	**3**	**2.124**	**6.45**	**26.55**	**27.32**
	Equiv. Refl.: (1,6,-6), (1,-7,6), (1,-7,-6), (6,1,6), (6,1,-6), (6,-7,6), (6,-7,-6), (9,-4,-3), (9,-5,3) (-4,9,3), (-4,-5,-3), (-5,9,-3), (-5,-4,3), (-7,1,6), (-7,1,-6), (-7,6,6), (-7,6,-6)								
**Sapph**	**(2,5,15)**	**11.812**	**81.01**	**3.6**	**3.2**	**1.892**	**6.14**	**23.65**	**24.44**
	Equiv. Refl.: (2,5,-15), (2,-7,15), (2,-7,-15), (5,2,15), (5,2,-15), (5,-7,15), (5,-7,-15), (7,-2,15), (7,-2,-15) (7,-5,15), (7,-5,-15), (-2,7,15), (-2,7,-15), (-2,-5,15), (-2,-5,-15), (-5,7,15), (-5,7,-15), (-5,-2,15) (-5,-2,-15), (-7,2,15), (-7,2,-15), (-7,5,15), (-7,5,-15)								
**Sapph**	**(4,5,-1)**	**11.76**	**79.52**	**3.5**	**3.1**	**2.212**	**6.85**	**27.65**	**28.48**
	Equiv. Refl.: (4,-9,1), (5,4,1), (5,-9,-1), (9,-4,1), (9,-5,-1), (-4,9,-1), (-4,-5,1), (-5,9,1), (-5,-4,-1) (-9,4,-1), (-9,5,1), (0,4,9), (0,-4,-9), (4,0,-9), (4,-4,9), (9,-4,2), (9,-5,-2), (-4,0,9) (-4,4,-9), (-4,9,-2), (-4,-5,2), (-5,9,2), (-5,-4,-2)								
**Sapph**	**(2,3,20)**	**11.581**	**75.56**	**3.4**	**3.1**	**3.079**	**9.67**	**38.49**	**39.69**
	Equiv. Refl.: (2,-5,-20), (3,2,-20), (3,-5,20), (5,-2,-20), (5,-3,20), (-2,5,20), (-2,-3,-20), (-3,5,-20), (-3,-2,20) (-5,2,20), (-5,3,-20), (2,2,9), (2,2,-9), (2,-4,9), (2,-4,-9), (4,5,1), (4,-9,-1), (5,4,-1) (5,-9,1), (-4,2,9), (-4,2,-9), (-9,4,1), (-9,5,-1)								
**Sapph**	**(1,7,0)**	**11.358**	**71.76**	**3.3**	**3**	**3.941**	**11.79**	**49.26**	**50.65**
	Equiv. Refl.: (1,-8,0), (7,1,0), (7,-8,0), (8,-1,0), (8,-7,0), (-1,8,0), (-1,-7,0), (-7,8,0), (-7,-1,0) (-8,1,0), (-8,7,0), (6,-1,7), (6,-5,-7), (7,-1,-6), (7,-6,6), (-1,6,-7), (-1,7,6), (-1,-5,7) (-1,-6,-6), (-5,6,7), (-5,-1,-7), (-6,7,-6), (-6,-1,6)								
**Sapph**	**(3,5,10)**	**11.562**	**75.2**	**3.2**	**3**	**3.16**	**9.35**	**39.51**	**40.6**
	Equiv. Refl.: (3,-8,-10), (5,3,-10), (5,-8,10), (8,-3,-10), (8,-5,10), (-3,8,10), (-3,-5,-10), (-5,8,-10), (-5,-3,10) (-8,3,10), (-8,5,-10), (1,5,-7), (1,-6,7), (5,1,7), (5,-6,-7), (-6,1,-7), (-6,5,7)								
**Quartz**	**(0,8,0)**	**11.656**	**77.07**	**3.1**	**2.5**	**2.745**	**6.91**	**34.31**	**35**
	Equiv. Refl.: (0,-8,0), (3,6,0), (3,-9,0), (6,3,0), (6,-9,0), (8,0,0), (8,-8,0), (-8,0,0), (-8,8,0) (-9,3,0), (-9,6,0)								
**Sapph**	**(2,2,21)**	**11.297**	**70.85**	**3**	**2.4**	**4.154**	**10.1**	**51.92**	**52.89**
	Equiv. Refl.: (2,2,-21), (2,-4,21), (2,-4,-21), (4,-2,21), (4,-2,-21), (-2,4,21), (-2,4,-21), (-2,-2,21), (-2,-2,-21) (-4,2,21), (-4,2,-21), (0,2,-10), (0,-2,10), (2,0,10), (2,6,-4), (2,-2,-10), (2,-8,4), (6,2,4) (6,-8,-4), (8,-1,-3), (8,-7,3), (-1,8,3), (-1,-7,-3), (-2,0,-10), (-2,2,10), (-5,9,3), (-5,-4,-3) (-7,8,-3), (-7,-1,3), (-8,2,-4), (-8,6,4)								
**Quartz**	**(1,7,3)**	**11.526**	**74.53**	**2.9**	**2.7**	**3.31**	**8.77**	**41.38**	**42.3**
	Equiv. Refl.: (1,-8,-3), (7,1,-3), (7,-8,3), (8,-2,4), (8,-6,-4), (9,-3,0), (9,-6,0), (-2,8,-4), (-2,-6,4) (-3,9,0), (-3,-6,0), (-6,8,4), (-6,9,0), (-6,-2,-4), (-6,-3,0), (-8,1,3), (-8,7,-3)								
**Quartz**	**(1,7,-3)**	**11.526**	**74.53**	**2.8**	**2.5**	**3.31**	**8.21**	**41.38**	**42.18**
	Equiv. Refl.: (1,-8,3), (7,1,3), (7,-8,-3), (9,-4,3), (9,-5,-3), (-4,9,-3), (-4,-5,3), (-8,1,-3), (-8,7,3)								
**Quartz**	**(1,4,8)**	**11.348**	**71.61**	**2.7**	**2.4**	**3.976**	**9.72**	**49.7**	**50.65**
	Equiv. Refl.: (1,-5,-8), (4,1,-8), (4,4,5), (4,4,-5), (4,-5,8), (4,-8,5), (4,-8,-5), (5,-1,-8), (5,-4,8) (-1,5,8), (-1,-4,-8), (-4,5,-8), (-4,-1,8), (-5,1,8), (-5,4,-8), (-8,4,5), (-8,4,-5)								
**LiNbO3**	**(2,7,7)**	**11.804**	**80.77**	**2.6**	**4.1**	**1.943**	**8.06**	**24.29**	**25.59**
	Equiv. Refl.: (2,-9,-7), (7,2,-7), (7,-9,7), (9,-2,-7), (9,-7,7), (-2,9,7), (-2,-7,-7), (-7,9,-7), (-7,-2,7) (-9,2,7), (-9,7,-7), (4,5,-3), (4,-9,3), (5,4,3), (5,-9,-3), (8,-1,3), (8,-7,-3), (-1,8,-3) (-1,-7,3), (-7,8,3), (-7,-1,-3), (-9,4,-3), (-9,5,3)								
**Quartz**	**(8,-4,5)**	**11.61**	**76.12**	**2.4**	**2.2**	**2.955**	**6.59**	**36.94**	**37.53**
	Equiv. Refl.: (8,-4,-5), (-4,8,5), (-4,8,-5), (-4,-4,5), (-4,-4,-5)								
**LiNbO3**	**(4,5,11)**	**11.922**	**85.5**	**2.3**	**5**	**0.941**	**4.66**	**11.77**	**12.66**
	Equiv. Refl.: (4,-9,-11), (5,4,-11), (5,-9,11), (9,-4,-11), (9,-5,11), (-4,9,11), (-4,-5,-11), (-5,9,-11), (-5,-4,11) (-9,4,11), (-9,5,-11)								
**Quartz**	**(0,8,2)**	**11.879**	**83.39**	**2.1**	**2.6**	**1.386**	**3.54**	**17.32**	**17.68**
	Equiv. Refl.: (0,-8,-2), (8,0,-2), (8,-8,2), (-8,0,2), (-8,8,-2)								

Ei =	11.959	keV						ΔEg	ΔEt
**Cryst**	**Refl**	**EB**	**ΘB**	**∫ IR dΘ**	**Width**	**Ei cotΘ**	**ΔE**	**2m, 50µm**	**2m, 50µm**
	**(h,k,l)**	**[keV]**	**[°]**	**[µrad]**	**[µrad]**	**[meV/µrad]**	**[meV]**	**[meV]**	**[meV]**
**Sapph**	**(1,5,17)**	**11.662**	**77.2**	**2**	**2.3**	**2.717**	**6.28**	**33.96**	**34.54**
	Equiv. Refl.: (1,-6,-17), (5,1,-17), (5,-6,17), (6,-1,-17), (6,-5,17), (-1,6,17), (-1,-5,-17), (-5,6,-17), (-5,-1,17)<br>(-6,1,17), (-6,5,-17)								
**Quartz**	**(3,6,-1)**	**11.621**	**76.35**	**1.9**	**2**	**2.904**	**5.71**	**36.3**	**36.75**
	Equiv. Refl.: (3,-9,1), (4,5,-1), (4,-9,1), (5,4,1), (5,-9,-1), (6,3,1), (6,-9,-1), (9,-4,1), (9,-5,-1)<br>(-4,9,-1), (-4,-5,1), (-5,9,1), (-5,-4,-1), (-9,3,-1), (-9,4,-1), (-9,5,1), (-9,6,1)								
**LiNbO3**	**(2,7,-5)**	**11.599**	**75.91**	**1.8**	**2.8**	**3.002**	**8.52**	**37.52**	**38.48**
	Equiv. Refl.: (2,-9,5), (7,2,5), (7,-9,-5), (9,-2,5), (9,-7,-5), (-2,9,-5), (-2,-7,5), (-7,9,5), (-7,-2,-5)<br>(-9,2,-5), (-9,7,5), (2,6,-7), (2,-8,7), (6,2,7), (6,-8,-7), (8,-2,7), (8,-6,-7), (-2,8,-7)<br>(-2,-6,7), (-6,8,7), (-6,-2,-7), (-8,2,-7), (-8,6,7), (3,5,5), (3,-8,-5), (5,3,-5), (5,-8,5)<br>(-8,3,5), (-8,5,-5)								
**Quartz**	**(8,-3,-5)**	**11.701**	**78.07**	**1.7**	**2**	**2.526**	**4.99**	**31.58**	**31.97**
	Equiv. Refl.: (8,-5,5), (9,-3,1), (9,-6,-1), (-3,8,5), (-3,9,-1), (-3,-5,-5), (-3,-6,1), (-5,8,-5), (-5,-3,5)<br>(-6,9,1), (-6,-3,-1)								
**Sapph**	**(4,4,9)**	**11.273**	**70.51**	**1.6**	**1.8**	**4.234**	**7.42**	**52.92**	**53.44**
	Equiv. Refl.: (4,4,-9), (4,-8,9), (4,-8,-9), (8,-4,9), (8,-4,-9), (-4,8,9), (-4,8,-9), (-4,-4,9), (-4,-4,-9)<br>(-8,4,9), (-8,4,-9)								
**LiNbO3**	**(2,7,1)**	**11.39**	**72.26**	**1.5**	**2.4**	**3.826**	**9.03**	**47.82**	**48.66**
	Equiv. Refl.: (2,-9,-1), (7,2,-1), (7,-9,1), (9,-2,-1), (9,-7,1), (-2,9,1), (-2,-7,-1), (-7,9,-1), (-7,-2,1)<br>(-9,2,1), (-9,7,-1)								
**Sapph**	**(1,7,3)**	**11.448**	**73.19**	**1.4**	**1.7**	**3.613**	**5.96**	**45.16**	**45.55**
	Equiv. Refl.: (1,7,-3), (1,-8,3), (1,-8,-3), (3,4,14), (3,4,-16), (3,5,-11), (3,-7,16), (3,-7,-14), (3,-8,11)<br>(4,3,16), (4,3,-14), (4,-7,14), (4,-7,-16), (5,3,11), (5,-8,-11), (7,1,3), (7,1,-3), (7,-3,16)<br>(7,-3,-14), (7,-4,14), (7,-4,-16), (7,-8,3), (7,-8,-3), (8,-1,3), (8,-1,-3), (8,-3,11), (8,-5,-11)<br>(8,-7,3), (8,-7,-3), (-1,8,3), (-1,8,-3), (-1,-7,3), (-1,-7,-3), (-3,7,14), (-3,7,-16), (-3,8,-11)<br>(-3,-4,16), (-3,-4,-14), (-3,-5,11), (-4,7,16), (-4,7,-14), (-4,-3,14), (-4,-3,-16), (-5,8,11), (-5,-3,-11)<br>(-7,3,14), (-7,3,-16), (-7,4,16), (-7,4,-14), (-7,8,3), (-7,8,-3), (-7,-1,3), (-7,-1,-3), (-8,1,3)<br>(-8,1,-3), (-8,3,-11), (-8,5,11), (-8,7,3), (-8,7,-3), (0,6,-7), (0,7,5), (0,-6,7), (0,-7,-5)<br>(6,0,7), (6,-6,-7), (7,0,-5), (7,-7,5), (9,-2,0), (9,-7,0), (-2,9,0), (-2,-7,0), (-6,0,-7)<br>(-6,6,7), (-7,0,5), (-7,7,-5), (-7,9,0), (-7,-2,0)								
**Quartz**	**(1,4,-8)**	**11.348**	**71.61**	**1.3**	**1.4**	**3.976**	**5.75**	**49.7**	**50.04**
	Equiv. Refl.: (1,-5,8), (2,5,6), (2,-7,-6), (3,5,-5), (3,-8,5), (4,1,8), (4,-5,-8), (5,2,-6), (5,3,5)<br>(5,-1,8), (5,-4,-8), (5,-7,6), (5,-8,-5), (7,-2,-6), (7,-5,6), (8,-3,5), (8,-5,-5), (-1,5,-8)<br>(-1,-4,8), (-2,7,6), (-2,-5,-6), (-3,8,-5), (-3,-5,5), (-4,5,8), (-4,-1,-8), (-5,1,-8), (-5,4,8)<br>(-5,7,-6), (-5,8,5), (-5,-2,6), (-5,-3,-5), (-7,2,6), (-7,5,-6), (-8,3,-5), (-8,5,5)								
**LiNbO3**	**(2,4,19)**	**11.239**	**70.02**	**1.2**	**2**	**4.347**	**8.66**	**54.34**	**55.03**
	Equiv. Refl.: (2,-6,-19), (4,2,-19), (4,-6,19), (6,-2,-19), (6,-4,19), (-2,6,19), (-2,-4,-19), (-4,6,-19), (-4,-2,19)<br>(-6,2,19), (-6,4,-19), (4,5,2), (4,-9,-2), (5,4,-2), (5,-9,2), (9,-4,-2), (9,-5,2), (-4,9,2)<br>(-4,-5,-2), (-5,9,-2), (-5,4,2), (-9,4,2), (-9,5,-2)								
**Sapph**	**(2,4,-17)**	**11.367**	**71.9**	**1.1**	**1.3**	**3.91**	**5.14**	**48.87**	**49.14**
	Equiv. Refl.: (2,-6,17), (4,2,17), (4,-6,-17), (6,-2,17), (6,-4,-17), (-2,6,-17), (-2,-4,17), (-4,6,17), (-4,-2,-17)<br>(-6,2,-17), (-6,4,17)								
**Sapph**	**(1,3,22)**	**11.819**	**81.21**	**1**	**1.5**	**1.848**	**2.69**	**23.1**	**23.26**
	Equiv. Refl.: (1,-4,-22), (3,1,-22), (3,-4,22), (4,-1,-22), (4,-3,22), (-1,4,22), (-1,-3,-22), (-3,4,-22), (-3,-1,22)<br>(-4,1,22), (-4,3,-22), (2,7,0), (2,-9,0), (7,2,0), (7,-9,0), (-9,2,0), (-9,7,0)								
**Quartz**	**(0,5,-8)**	**11.716**	**78.44**	**0.9**	**1.1**	**2.447**	**2.68**	**30.58**	**30.7**
	Equiv. Refl.: (0,8,1), (0,-5,8), (0,-8,-1), (5,0,8), (5,-5,-8), (8,0,-1), (8,-8,1), (-5,0,-8), (-5,5,8)<br>(-8,0,1), (-8,8,-1)								

Ei =	11.959	keV						ΔEg	ΔEt
Cryst	Refl	EB	ΘB	∫ IR dΘ	Width	Ei cotΘ	ΔE	2m, 50µm	2m, 50µm
	(h,k,l)	[keV]	[°]	[µrad]	[µrad]	[meV/µrad]	[meV]	[meV]	[meV]
**LiNbO3**	**(1,2,-25)**	**11.769**	**79.78**	**0.8**	**1.9**	**2.157**	**4.02**	**26.96**	**27.25**
	Equiv. Refl.: (1,3,-23), (1,-3,25), (1,-4,23), (2,1,25), (2,3,23), (2,-3,-25), (2,-5,-23), (3,1,23), (3,2,-23) (3,4,17), (3,-1,25), (3,-2,-25), (3,-4,-23), (3,-5,23), (3,-7,-17), (4,3,-17), (4,-1,23), (4,-3,-23) (4,-7,17), (5,-2,-23), (5,-3,23), (7,-3,-17), (7,-4,17), (-1,3,-25), (-1,4,-23), (-1,-2,25), (-1,-3,23) (-2,3,25), (-2,5,23), (-2,-1,-25), (-2,-3,-23), (-3,1,-25), (-3,2,25), (-3,4,23), (-3,5,-23), (-3,7,17) (-3,-1,-23), (-3,-2,23), (-3,-4,-17), (-4,1,-23), (-4,3,23), (-4,7,-17), (-4,-3,17), (-5,2,23), (-5,3,-23) (-7,3,17), (-7,4,-17), (1,2,23), (1,-3,-23), (2,1,-23), (2,-3,23), (3,-1,-23), (3,-2,23), (-1,3,23) (-1,-2,-23), (-2,3,-23), (-2,-1,23), (-3,1,23), (-3,2,-23), (0,8,-2), (0,-8,2), (1,1,10), (1,1,-10) (1,-2,10), (1,-2,-10), (8,0,2), (8,-8,-2), (-2,1,10), (-2,1,-10), (-8,0,-2), (-8,8,2)								
**LiNbO3**	**(1,4,21)**	**11.348**	**71.61**	**0.7**	**1.4**	**3.975**	**5.41**	**49.69**	**49.98**
	Equiv. Refl.: (1,4,-21), (1,-5,21), (1,-5,-21), (4,1,21), (4,1,-21), (4,5,-7), (4,-5,21), (4,-5,-21), (4,-9,7) (5,4,7), (5,-1,21), (5,-1,-21), (5,-4,21), (5,-4,-21), (5,-9,-7), (9,-4,7), (9,-5,-7), (-1,5,21) (-1,5,-21), (-1,-4,21), (-1,-4,-21), (-4,5,21), (-4,5,-21), (-4,9,-7), (-4,-1,21), (-4,-1,-21), (-4,-5,7) (-5,1,21), (-5,1,-21), (-5,4,21), (-5,4,-21), (-5,9,7), (-5,-4,-7), (-9,4,-7), (-9,5,7), (1,6,13) (1,-7,-13), (6,1,-13), (6,-7,13), (7,-1,-13), (7,-6,13), (-1,7,13), (-1,-6,-13), (-6,7,-13), (-6,-1,13) (-7,1,13), (-7,6,-13), (2,-1,10), (-1,2,10), (-1,2,-10), (-1,-1,10), (-1,-1,-10)								
**LiNbO3**	**(1,5,-19)**	**11.495**	**73.98**	**0.6**	**1.3**	**3.434**	**4.59**	**42.92**	**43.17**
	Equiv. Refl.: (1,-6,19), (3,6,9), (3,6,-9), (3,-9,9), (3,-9,-9), (5,1,19), (5,-6,-19), (6,3,9), (6,3,-9) (6,-1,19), (6,-5,-19), (6,-9,9), (6,-9,-9), (9,-3,9), (9,-3,-9), (9,-6,9), (9,-6,-9), (-1,6,-19) (-1,-5,19), (-3,9,9), (-3,9,-9), (-3,-6,9), (-3,-6,-9), (-5,6,19), (-5,-1,-19), (-6,1,-19), (-6,5,19) (-6,9,9), (-6,9,-9), (-6,-3,9), (-6,-3,-9), (-9,3,9), (-9,3,-9), (-9,6,9), (-9,6,-9), (0,2,10) (0,-2,-10), (2,0,-10), (2,-1,-10), (2,-2,10), (-2,0,10), (-2,2,-10)								
**Quartz**	**(8,-2,-4)**	**11.465**	**73.47**	**0.5**	**0.6**	**3.55**	**2.06**	**44.37**	**44.42**
	Equiv. Refl.: (8,-6,4), (-2,8,4), (-2,-6,-4), (-6,8,-4), (-6,-2,4)								
**LiNbO3**	**(2,6,-13)**	**11.59**	**75.73**	**0.4**	**1**	**3.041**	**3.18**	**38.01**	**38.14**
	Equiv. Refl.: (2,-8,13), (6,2,13), (6,-8,-13), (8,-2,13), (8,-6,-13), (-2,8,-13), (-2,-6,13), (-6,8,13), (-6,-2,-13) (-8,2,-13), (-8,6,13), (2,5,-6), (2,6,4), (2,-7,6), (2,-8,-4), (5,2,6), (5,-7,-6), (6,2,-4) (6,-1,-7), (6,-5,7), (6,-8,4), (7,-2,6), (7,-5,-6), (-1,6,7), (-1,-5,-7), (-2,7,-6), (-2,-5,6) (-5,6,-7), (-5,7,6), (-5,-1,7), (-5,-2,-6), (-7,2,-6), (-7,5,6), (-8,2,4), (-8,6,-4)								
**LiNbO3**	**(1,8,-1)**	**11.889**	**83.79**	**0.3**	**1.4**	**1.302**	**1.88**	**16.28**	**16.38**
	Equiv. Refl.: (1,-9,1), (8,1,1), (8,-9,-1), (9,-1,1), (9,-8,-1), (-1,9,-1), (-1,-8,1), (-8,9,1), (-8,-1,-1) (-9,1,-1), (-9,8,1), (1,5,7), (1,-6,-7), (4,5,0), (4,-9,0), (5,1,-7), (5,4,0), (5,-6,7) (5,-9,0), (9,-4,0), (9,-5,0), (-4,9,0), (-4,-5,0), (-5,9,0), (-5,-4,0), (-6,1,7), (-6,5,-7) (-9,4,0), (-9,5,0)								
**LiNbO3**	**(1,7,9)**	**11.243**	**70.07**	**0.2**	**0.6**	**4.336**	**2.82**	**54.19**	**54.27**
	Equiv. Refl.: (1,7,-9), (1,-8,9), (1,-8,-9), (3,5,13), (3,-8,-13), (5,3,-13), (5,-8,13), (7,1,9), (7,1,-9) (7,-8,9), (7,-8,-9), (8,-1,9), (8,-1,-9), (8,-3,-13), (8,-5,13), (8,-7,9), (8,-7,-9), (-1,8,9) (-1,8,-9), (-1,-7,9), (-1,-7,-9), (-3,8,13), (-3,-5,-13), (-5,8,-13), (-5,-3,13), (-7,8,9), (-7,8,-9) (-7,-1,9), (-7,-1,-9), (-8,1,9), (-8,1,-9), (-8,3,13), (-8,5,-13), (-8,7,9), (-8,7,-9)								
**LiNbO3**	**(3,3,21)**	**11.849**	**82.21**	**0.1**	**0.9**	**1.637**	**1.44**	**20.46**	**20.51**
	Equiv. Refl.: (3,3,-21), (3,-6,21), (3,-6,-21), (6,-3,21), (6,-3,-21), (-3,6,21), (-3,6,-21), (-3,-3,21), (-3,-3,-21) (-6,3,21), (-6,3,-21), (0,0,24), (0,0,-24), (2,6,8), (2,-8,-8), (4,4,12), (4,4,-12), (4,5,2) (4,-8,12), (4,-8,-12), (4,-9,-2), (5,4,-2), (5,-9,2), (6,2,-8), (6,-8,8), (8,-2,-8), (8,-4,12) (8,-4,-12), (8,-6,8), (9,-4,-2), (9,-5,2), (-2,8,8), (-2,-6,-8), (-4,8,12), (-4,8,-12), (-4,9,2) (-4,-4,12), (-4,-4,-12), (-4,-5,-2), (-5,9,-2), (-5,-4,2), (-6,8,-8), (-6,-2,8), (-8,2,8), (-8,4,12) (-8,4,-12), (-8,6,-8), (-9,4,2), (-9,5,-2)								
**LiNbO3**	**(4,4,15)**	**11.739**	**78.99**	**0**	**0.3**	**2.327**	**0.69**	**29.09**	**29.1**
	Equiv. Refl.: (4,4,-15), (4,-8,15), (4,-8,-15), (8,-4,15), (8,-4,-15), (-4,8,15), (-4,8,-15), (-4,-4,15), (-4,-4,-15) (-8,4,15), (-8,4,-15)								

Ei =	10.535	keV						ΔEg	ΔEt
Cryst	Refl	EB	ΘB	∫ IR dΘ	Width	Ei cotΘ	ΔE	2m, 50µm	2m, 50µm
	(h,k,l)	[keV]	[°]	[µrad]	[µrad]	[meV/µrad]	[meV]	[meV]	[meV]
Ge	(1,3,9)	10.453	82.85	14.386	15.1	1.322	20	16.52	25.94
Ge	(4,6,6)	10.279	77.35	13.725	12.9	2.364	30.4	29.55	42.4
Si	(0,4,8)	10.209	75.72	8.702	7.4	2.682	19.91	33.52	38.99
Si	(1,1,9)	10.399	80.79	8.269	7.7	1.709	13.08	21.36	25.05
	Equiv. Refl.: (3,5,7)								
Ge	(1,1,9)	9.983	71.37	6.872	6.9	3.551	24.35	44.39	50.63
	Equiv. Refl.: (3,5,7)								
LiNbO3	(0,4,20)	10.532	88.62	171.9	158.9	0.253	40.27	3.17	40.39
	Equiv. Refl.: (4,-4,20), (-4,0,20)								
LiNbO3	(0,-4,-20)	10.532	88.62	171.2	158.9	0.253	40.27	3.17	40.39
	Equiv. Refl.: (4,0,-20), (-4,4,-20)								
LiNbO3	(3,-7,-14)	10.523	87.25	72.2	67.2	0.505	33.94	6.32	34.52
	Equiv. Refl.: (4,3,-14), (7,-3,-14), (-3,-4,-14), (-4,7,-14), (-7,4,-14)								
LiNbO3	(3,4,14)	10.523	87.25	68.3	67.2	0.505	33.94	6.32	34.52
	Equiv. Refl.: (4,-7,14), (7,-4,14), (-3,7,14), (-4,-3,14), (-7,3,14)								
LiNbO3	(1,-3,22)	10.503	85.55	46.6	43.7	0.82	35.8	10.25	37.23
	Equiv. Refl.: (2,1,22), (3,-1,22), (-1,-2,22), (-2,3,22), (-3,2,22)								
LiNbO3	(1,2,-22)	10.503	85.55	45.6	43.7	0.82	35.8	10.25	37.23
	Equiv. Refl.: (2,-3,-22), (3,-2,-22), (-1,3,-22), (-2,-1,-22), (-3,1,-22)								
LiNbO3	(1,-8,0)	10.498	85.19	43.4	40.7	0.886	36.03	11.07	37.69
	Equiv. Refl.: (7,1,0), (8,-1,0), (-1,-7,0), (-7,8,0), (-8,7,0)								
LiNbO3	(1,7,0)	10.498	85.19	42.6	40.7	0.886	36.03	11.07	37.69
	Equiv. Refl.: (7,-8,0), (8,-7,0), (-1,8,0), (-7,-1,0), (-8,1,0)								
LiNbO3	(3,5,-8)	10.37	79.85	26.7	23.8	1.887	44.88	23.58	50.7
	Equiv. Refl.: (5,-8,-8), (8,-5,-8), (-3,8,-8), (-5,-3,-8), (-8,3,-8)								
LiNbO3	(3,-8,8)	10.37	79.85	25.3	23.8	1.887	44.88	23.58	50.7
	Equiv. Refl.: (5,3,8), (8,-3,8), (-3,-5,8), (-5,8,8), (-8,5,8)								
LiNbO3	(0,7,8)	10.37	79.85	23	21	1.887	39.62	23.58	46.11
	Equiv. Refl.: (7,-7,8), (-7,0,8)								
LiNbO3	(0,-7,-8)	10.37	79.85	21.9	21	1.887	39.62	23.58	46.11
	Equiv. Refl.: (7,0,-8), (-7,7,-8)								
LiNbO3	(1,-4,20)	10.253	76.71	18.5	17	2.488	42.4	31.1	52.59
	Equiv. Refl.: (3,1,20), (4,-1,20), (-1,-3,20), (-3,4,20), (-4,3,20)								
LiNbO3	(0,2,22)	10.223	76.03	18.3	16.6	2.621	43.59	32.76	54.53
	Equiv. Refl.: (2,-2,22), (-2,0,22)								
LiNbO3	(1,3,-20)	10.253	76.71	18.2	17	2.488	42.4	31.1	52.59
	Equiv. Refl.: (3,-4,-20), (4,-3,-20), (-1,4,-20), (-3,-1,-20), (-4,1,-20)								
LiNbO3	(2,4,16)	10.263	76.95	18	16.5	2.442	40.38	30.52	50.61
	Equiv. Refl.: (4,-6,16), (6,-4,16), (-2,6,16), (-4,-2,16), (-6,2,16)								
LiNbO3	(0,-2,-22)	10.223	76.03	17.6	16.6	2.621	43.59	32.76	54.53
	Equiv. Refl.: (2,0,-22), (2,6,-4), (6,-8,-4), (8,-6,-4), (-2,2,-22), (-2,8,-4), (-6,-2,-4), (-8,2,-4)								
LiNbO3	(2,-8,4)	10.185	75.19	17.5	16.1	2.784	44.95	34.81	56.85
	Equiv. Refl.: (6,2,4), (8,-2,4), (-2,-6,4), (-6,8,4), (-8,6,4)								
LiNbO3	(2,-6,-16)	10.263	76.95	17.3	16.5	2.442	40.38	30.52	50.61
	Equiv. Refl.: (4,2,-16), (6,-2,-16), (-2,-4,-16), (-4,6,-16), (-6,4,-16)								
LiNbO3	(1,-7,-10)	10.156	74.58	17.1	15.4	2.907	44.65	36.33	57.57
	Equiv. Refl.: (6,1,-10), (7,-1,-10), (-1,-6,-10), (-6,7,-10), (-7,6,-10)								

Ei =	10.535	keV						ΔEg	ΔEt
**Cryst**	**Refl**	**EB**	**ΘB**	**∫ IR dΘ**	**Width**	**Ei cotΘ**	**ΔE**	**2m, 50µm**	**2m, 50µm**
	**(h,k,l)**	**[keV]**	**[°]**	**[µrad]**	**[µrad]**	**[meV/µrad]**	**[meV]**	**[meV]**	**[meV]**
**LiNbO3**	**(1,-5,-18)**	**10.266**	**77.03**	**16.7**	**15.5**	**2.427**	**37.72**	**30.34**	**48.41**
	Equiv. Refl.: (4,1,-18), (5,-1,-18), (-1,-4,-18), (-4,5,-18), (-5,4,-18)								
**LiNbO3**	**(1,4,-18)**	**10.266**	**77.03**	**16.2**	**15.2**	**2.427**	**36.86**	**30.34**	**47.74**
	Equiv. Refl.: (1,6,10), (4,-5,-18), (5,-4,-18), (6,-7,10), (7,-6,10), (-1,5,-18), (-1,7,10), (-4,-1,-18), (-5,1,-18) (-6,-1,10), (-7,1,10)								
**LiNbO3**	**(1,4,18)**	**10.266**	**77.03**	**16.1**	**15.5**	**2.427**	**37.72**	**30.34**	**48.41**
	Equiv. Refl.: (4,-5,18), (5,-4,18), (-1,5,18), (-4,-1,18), (-5,1,18)								
**LiNbO3**	**(1,-5,18)**	**10.266**	**77.03**	**15.7**	**15.2**	**2.427**	**36.86**	**30.34**	**47.74**
	Equiv. Refl.: (4,1,18), (5,-1,18), (-1,-4,18), (-4,5,18), (-5,4,18)								
**Sapph**	**(4,4,3)**	**10.521**	**87.03**	**15.5**	**15.4**	**0.547**	**8.41**	**6.84**	**10.84**
	Equiv. Refl.: (4,4,-3), (4,-8,3), (4,-8,-3), (8,-4,3), (8,-4,-3), (-4,8,3), (-4,8,-3), (-4,-4,3), (-4,-4,-3) (-8,4,3), (-8,4,-3)								
**LiNbO3**	**(2,-7,12)**	**10.208**	**75.68**	**15.4**	**14.5**	**2.689**	**38.9**	**33.61**	**51.41**
	Equiv. Refl.: (5,2,12), (7,-2,12), (-2,-5,12), (-5,7,12), (-7,5,12), (0,0,9), (0,0,-9)								
**LiNbO3**	**(2,5,-12)**	**10.208**	**75.68**	**15.3**	**14.5**	**2.689**	**38.9**	**33.61**	**51.41**
	Equiv. Refl.: (5,-7,-12), (7,-5,-12), (-2,7,-12), (-5,-2,-12), (-7,2,-12)								
**Sapph**	**(3,4,-10)**	**10.321**	**78.43**	**15.2**	**12.7**	**2.157**	**27.36**	**26.96**	**38.41**
	Equiv. Refl.: (3,-7,10), (4,3,10), (4,-7,-10), (7,-3,10), (7,-4,-10), (-3,7,-10), (-3,-4,10), (-4,7,10), (-4,-3,-10) (-7,3,-10), (-7,4,10)								
**LiNbO3**	**(2,5,12)**	**10.208**	**75.68**	**14.6**	**13.9**	**2.689**	**37.28**	**33.61**	**50.19**
	Equiv. Refl.: (2,-7,-12), (5,2,-12), (5,-7,12), (7,-2,-12), (7,-5,12), (-2,7,12), (-2,-5,-12), (-5,7,-12), (-5,-2,12) (-7,2,12), (-7,5,-12)								
**LiNbO3**	**(4,4,-6)**	**10**	**71.67**	**14.1**	**12.8**	**3.491**	**44.76**	**43.64**	**62.51**
	Equiv. Refl.: (4,-8,-6), (8,-4,-6), (-4,8,-6), (-4,-4,-6), (-8,4,-6)								
**LiNbO3**	**(1,-6,-14)**	**9.956**	**70.92**	**13.7**	**12.6**	**3.643**	**45.99**	**45.54**	**64.73**
	Equiv. Refl.: (5,1,-14), (6,-1,-14), (-1,-5,-14), (-5,6,-14), (-6,5,-14)								
**LiNbO3**	**(1,5,14)**	**9.956**	**70.92**	**13.6**	**12.6**	**3.643**	**45.99**	**45.54**	**64.73**
	Equiv. Refl.: (4,4,6), (4,-8,6), (5,-6,14), (6,-5,14), (8,-4,6), (-1,6,14), (-4,8,6), (-4,-4,6), (-5,-1,14) (-6,1,14), (-8,4,6)								
**LiNbO3**	**(0,-6,-12)**	**9.92**	**70.32**	**12.9**	**11.8**	**3.768**	**44.62**	**47.1**	**64.88**
	Equiv. Refl.: (6,0,-12), (-6,6,-12)								
**LiNbO3**	**(0,6,12)**	**9.92**	**70.32**	**12.6**	**11.8**	**3.768**	**44.62**	**47.1**	**64.88**
	Equiv. Refl.: (6,-6,12), (-6,0,12), (0,6,5), (0,-6,-5), (6,0,-5), (6,-6,5), (-6,0,5), (-6,6,-5)								
**LiNbO3**	**(0,-5,-16)**	**9.976**	**71.26**	**12.3**	**10.9**	**3.575**	**39.05**	**44.68**	**59.34**
	Equiv. Refl.: (5,0,-16), (-5,5,-16)								
**LiNbO3**	**(0,1,-22)**	**9.936**	**70.58**	**11.9**	**11**	**3.714**	**40.8**	**46.42**	**61.8**
	Equiv. Refl.: (0,-1,22), (1,0,22), (1,-1,-22), (-1,0,-22), (-1,1,22)								
**LiNbO3**	**(0,6,-12)**	**9.92**	**70.32**	**11.5**	**10.5**	**3.768**	**39.68**	**47.1**	**61.58**
	Equiv. Refl.: (6,-6,-12), (-6,0,-12)								
**Sapph**	**(2,4,-14)**	**10.393**	**80.59**	**11.4**	**9.7**	**1.745**	**16.9**	**21.81**	**27.6**
	Equiv. Refl.: (2,-6,14), (4,2,14), (4,-6,-14), (6,-2,14), (6,-4,-14), (-2,6,-14), (-2,-4,14), (-4,6,14), (-4,-2,-14) (-6,2,-14), (-6,4,14)								
**LiNbO3**	**(2,6,2)**	**10.067**	**72.85**	**11.2**	**10.6**	**3.25**	**34.52**	**40.63**	**53.31**
	Equiv. Refl.: (6,-8,2), (8,-6,2), (-2,8,2), (-6,-2,2), (-8,2,2)								
**LiNbO3**	**(0,-6,12)**	**9.92**	**70.32**	**11.1**	**10.5**	**3.768**	**39.68**	**47.1**	**61.58**
	Equiv. Refl.: (2,-8,-2), (6,0,12), (6,2,-2), (8,-2,-2), (-2,-6,-2), (-6,6,12), (-6,8,-2), (-8,6,-2)								
**LiNbO3**	**(0,5,16)**	**9.976**	**71.26**	**10.9**	**10.9**	**3.575**	**39.05**	**44.68**	**59.34**
	Equiv. Refl.: (5,-5,16), (-5,0,16), (4,4,0), (4,-8,0), (8,-4,0), (-4,8,0), (-4,-4,0), (-8,4,0)								
**Quartz**	**(0,3,-8)**	**10.164**	**74.75**	**9.9**	**7.8**	**2.872**	**22.36**	**35.9**	**42.3**
	Equiv. Refl.: (0,-3,8), (3,0,8), (3,-3,-8), (-3,0,-8), (-3,3,8)								

**Ei =**	**10.535**	**keV**						**ΔEg**	**ΔEt**
**Cryst**	**Refl**	**EB**	**ΘB**	**∫ IR dΘ**	**Width**	**Ei cotΘ**	**ΔE**	**2m, 50µm**	**2m, 50µm**
	**(h,k,l)**	**[keV]**	**[°]**	**[µrad]**	**[µrad]**	**[meV/µrad]**	**[meV]**	**[meV]**	**[meV]**
**Sapph**	**(1,2,20)**	**10.342**	**79.03**	**9.5**	**8.1**	**2.042**	**16.54**	**25.53**	**30.42**
	Equiv. Refl.: (1,-3,-20), (2,1,-20), (2,-3,20), (3,-1,-20), (3,-2,20), (-1,3,20), (-1,-2,-20), (-2,3,-20), (-2,-1,20) (-3,1,20), (-3,2,-20)								
**Quartz**	**(3,5,-2)**	**10.454**	**82.88**	**9.2**	**8.2**	**1.316**	**10.75**	**16.45**	**19.65**
	Equiv. Refl.: (3,-8,2), (5,3,2), (5,-8,-2), (8,-3,2), (8,-5,-2), (-3,8,-2), (-3,-5,2), (-5,8,2), (-5,-3,-2) (-8,3,-2), (-8,5,2)								
**Sapph**	**(0,5,-14)**	**10.061**	**72.76**	**9.1**	**7.4**	**3.27**	**24.24**	**40.88**	**47.52**
	Equiv. Refl.: (0,-5,14), (5,0,14), (5,-5,-14), (-5,0,-14), (-5,5,14)								
**Quartz**	**(0,7,1)**	**10.263**	**76.96**	**8**	**6.6**	**2.441**	**15.99**	**30.51**	**34.44**
	Equiv. Refl.: (0,-7,-1), (7,0,-1), (7,-7,1), (-7,0,1), (-7,7,-1)								
**Quartz**	**(2,6,0)**	**10.506**	**85.78**	**7.9**	**7.2**	**0.777**	**5.63**	**9.71**	**11.22**
	Equiv. Refl.: (2,-8,0), (6,2,0), (6,-8,0), (-8,2,0), (-8,6,0)								
**Sapph**	**(1,1,21)**	**10.356**	**79.43**	**7.6**	**6.9**	**1.966**	**13.48**	**24.57**	**28.02**
	Equiv. Refl.: (1,1,-21), (1,-2,21), (1,-2,-21), (2,-1,21), (2,-1,-21), (-1,2,21), (-1,2,-21), (-1,-1,21), (-1,-1,-21) (-2,1,21), (-2,1,-21)								
**Quartz**	**(4,4,2)**	**10.352**	**79.3**	**7.4**	**6.3**	**1.991**	**12.59**	**24.89**	**27.9**
	Equiv. Refl.: (4,4,-2), (4,-8,2), (4,-8,-2), (-8,4,2), (-8,4,-2)								
**Quartz**	**(8,-4,2)**	**10.352**	**79.3**	**7.3**	**6.3**	**1.991**	**12.59**	**24.89**	**27.9**
	Equiv. Refl.: (8,-4,-2), (-4,8,2), (-4,8,-2), (-4,-4,2), (-4,-4,-2)								
**Quartz**	**(2,4,-6)**	**10.335**	**78.8**	**7.2**	**6**	**2.085**	**12.52**	**26.07**	**28.92**
	Equiv. Refl.: (2,-6,6), (4,2,6), (4,-6,-6), (-6,2,-6), (-6,4,6)								
**Quartz**	**(6,-2,6)**	**10.335**	**78.8**	**6.8**	**6**	**2.085**	**12.53**	**26.07**	**28.92**
	Equiv. Refl.: (6,-4,-6), (8,-2,0), (8,-6,0), (-2,6,-6), (-2,8,0), (-2,-4,6), (-2,-6,0), (-4,6,6), (-4,-2,-6) (-6,8,0), (-6,-2,0)								
**Sapph**	**(2,3,-16)**	**10.066**	**72.84**	**6.7**	**5.7**	**3.254**	**18.62**	**40.67**	**44.73**
	Equiv. Refl.: (2,-5,16), (3,2,16), (3,-5,-16), (5,-2,16), (5,-3,-16), (-2,5,-16), (-2,-3,16), (-3,5,16), (-3,-2,-16) (-5,2,-16), (-5,3,16)								
**Quartz**	**(5,-1,-7)**	**10.443**	**82.4**	**6.4**	**5.7**	**1.405**	**8**	**17.57**	**19.3**
	Equiv. Refl.: (5,-4,7), (-4,5,-7), (-4,-1,7)								
**Quartz**	**(3,5,2)**	**10.454**	**82.88**	**6.3**	**5.8**	**1.316**	**7.59**	**16.45**	**18.12**
	Equiv. Refl.: (3,-8,-2), (5,3,-2), (5,-8,2), (8,-3,-2), (8,-5,2), (-1,5,7), (-1,-4,-7), (-3,8,2), (-3,-5,-2) (-5,8,-2), (-5,-3,2), (-8,3,2), (-8,5,-2)								
**Quartz**	**(0,7,0)**	**10.199**	**75.49**	**6.2**	**5.3**	**2.727**	**14.43**	**34.09**	**37.01**
	Equiv. Refl.: (0,-7,0), (1,4,7), (1,-5,-7), (4,1,-7), (4,-5,7), (7,0,0), (7,-7,0), (-5,1,7), (-5,4,-7) (-7,0,0), (-7,7,0)								
**Quartz**	**(0,1,9)**	**10.425**	**81.73**	**6**	**5.4**	**1.531**	**8.26**	**19.14**	**20.85**
	Equiv. Refl.: (0,-1,-9), (1,0,-9), (1,-1,9), (-1,0,9), (-1,1,-9)								
**Sapph**	**(3,4,8)**	**9.916**	**70.26**	**5.9**	**5.1**	**3.781**	**19.17**	**47.26**	**51**
	Equiv. Refl.: (3,-7,-8), (4,3,-8), (4,-7,8), (7,-3,-8), (7,-4,8), (-3,7,8), (-3,-4,-8), (-4,7,-8), (-4,-3,8) (-7,3,8), (-7,4,-8)								
**Quartz**	**(6,-2,-6)**	**10.335**	**78.8**	**5.8**	**4.7**	**2.085**	**9.78**	**26.07**	**27.84**
	Equiv. Refl.: (6,-4,6), (-2,6,6), (-2,-4,-6)								
**Sapph**	**(0,2,-20)**	**10.009**	**71.81**	**5.7**	**4.9**	**3.461**	**16.91**	**43.26**	**46.45**
	Equiv. Refl.: (0,-2,20), (2,0,20), (2,-2,-20), (-2,0,-20), (-2,2,20), (-4,6,-6), (-4,-2,6)								
**Sapph**	**(1,5,-13)**	**10.424**	**81.68**	**5.6**	**5.4**	**1.541**	**8.31**	**19.26**	**20.98**
	Equiv. Refl.: (1,-6,13), (5,1,13), (5,-6,-13), (6,-1,13), (6,-5,-13), (-1,6,-13), (-1,-5,13), (-5,6,13), (-5,-1,-13) (-6,1,-13), (-6,5,13)								
**Quartz**	**(0,7,-2)**	**10.454**	**82.88**	**5.5**	**5**	**1.316**	**6.64**	**16.45**	**17.74**
	Equiv. Refl.: (0,-7,2), (7,0,2), (7,-7,-2), (-7,0,-2), (-7,7,2)								
**Quartz**	**(2,4,6)**	**10.335**	**78.8**	**5.1**	**4.7**	**2.085**	**9.78**	**26.07**	**27.84**
	Equiv. Refl.: (2,-6,-6), (4,2,-6), (4,-6,6), (8,-4,0), (-4,8,0), (-4,-4,0), (-6,2,6), (-6,4,-6)								

Ei =	10.535	keV						ΔEg	ΔEt
**Cryst**	**Refl**	**EB**	**ΘB**	**∫ IR dΘ**	**Width**	**Ei cotΘ**	**ΔE**	**2m, 50µm**	**2m, 50µm**
	**(h,k,l)**	**[keV]**	**[°]**	**[µrad]**	**[µrad]**	**[meV/µrad]**	**[meV]**	**[meV]**	**[meV]**
**Quartz**	**(0,4,-7)**	**9.921**	**70.35**	**5**	**4**	**3.762**	**15.15**	**47.03**	**49.41**
	Equiv. Refl.: (0,-4,7), (3,3,6), (3,3,-6), (3,-6,6), (3,-6,-6), (4,0,7), (4,-4,-7), (6,-3,6), (6,-3,-6) (-3,6,6), (-3,6,-6), (-3,-3,6), (-3,-3,-6), (-4,0,-7), (-4,4,7), (-6,3,6), (-6,3,-6)								
**Quartz**	**(1,2,-8)**	**9.953**	**70.87**	**4.9**	**4**	**3.655**	**14.73**	**45.69**	**48**
	Equiv. Refl.: (1,-3,8), (2,1,8), (2,-3,-8), (3,-1,8), (3,-2,-8), (-1,3,-8), (-1,-2,8), (-2,3,8), (-2,-1,-8) (-3,1,-8), (-3,2,8)								
**Quartz**	**(4,4,0)**	**10.094**	**73.37**	**4.7**	**4.1**	**3.147**	**12.76**	**39.34**	**41.35**
	Equiv. Refl.: (4,-8,0), (5,-1,7), (5,-4,-7), (-1,5,-7), (-1,-4,7), (-4,5,7), (-4,-1,-7), (-8,4,0)								
**Quartz**	**(4,-2,8)**	**10.473**	**83.76**	**4.6**	**4.5**	**1.152**	**5.15**	**14.4**	**15.29**
	Equiv. Refl.: (4,-2,-8), (-2,4,8), (-2,4,-8), (-2,-2,8), (-2,-2,-8)								
**Sapph**	**(2,5,9)**	**10.331**	**78.69**	**4.4**	**4.2**	**2.106**	**8.79**	**26.33**	**27.76**
	Equiv. Refl.: (2,5,-9), (2,-7,9), (2,-7,-9), (5,2,9), (5,2,-9), (5,-7,9), (5,-7,-9), (7,-2,9), (7,-2,-9) (7,-5,9), (7,-5,-9), (-2,7,9), (-2,7,-9), (-2,-5,9), (-2,-5,-9), (-5,7,9), (-5,7,-9), (-5,-2,9) (-5,-2,-9), (-7,2,9), (-7,2,-9), (-7,5,9), (-7,5,-9), (1,4,-7), (1,-5,7), (4,1,7), (4,-5,-7) (8,-3,0), (8,-5,0), (-3,8,0), (-3,-5,0), (-5,1,-7), (-5,4,7), (-5,8,0), (-5,-3,0)								
**Sapph**	**(1,6,4)**	**10.048**	**72.51**	**4.3**	**3.8**	**3.319**	**12.53**	**41.49**	**43.34**
	Equiv. Refl.: (1,-7,-4), (6,1,-4), (6,-7,4), (7,-1,-4), (7,-6,4), (-1,7,4), (-1,-6,-4), (-6,7,-4), (-6,-1,4) (-7,1,4), (-7,6,-4), (7,-3,4), (7,-4,-4), (-3,7,-4), (-3,-4,4), (-4,7,4), (-4,-3,-4)								
**Quartz**	**(1,5,-5)**	**9.935**	**70.56**	**4.2**	**3.5**	**3.717**	**12.87**	**46.47**	**48.21**
	Equiv. Refl.: (1,-6,5), (2,2,-8), (2,-4,8), (3,4,-4), (3,5,0), (3,-7,4), (3,-8,0), (4,3,4), (4,-7,-4) (5,1,5), (5,3,0), (5,-6,-5), (5,-8,0), (-4,2,8), (-4,2,-8), (-6,1,-5), (-6,5,5), (-7,3,-4) (-7,4,4), (-8,3,0), (-8,5,0)								
**Quartz**	**(2,2,8)**	**10.473**	**83.76**	**4.1**	**4.5**	**1.152**	**5.14**	**14.4**	**15.29**
	Equiv. Refl.: (2,-4,-8), (6,-1,5), (6,-5,-5), (-1,6,-5), (-1,-5,5), (-5,6,5), (-5,-1,-5)								
**LiNbO3**	**(1,6,-11)**	**10.36**	**79.55**	**4**	**6**	**1.943**	**11.69**	**24.29**	**26.95**
	Equiv. Refl.: (1,-7,11), (6,1,11), (6,-7,-11), (7,-1,11), (7,-6,-11), (-1,7,-11), (-1,-6,11), (-6,7,11), (-6,-1,-11) (-7,1,-11), (-7,6,11), (1,4,15), (1,4,-15), (1,-5,15), (1,-5,-15), (4,1,15), (4,1,-15), (4,-5,15) (4,-5,-15), (5,-1,15), (5,-1,-15), (5,-4,15), (5,-4,-15), (-1,5,15), (-1,5,-15), (-1,-4,15), (-1,-4,-15) (-4,5,15), (-4,5,-15), (-4,-1,15), (-4,-1,-15), (-5,1,15), (-5,1,-15), (-5,4,15), (-5,4,-15)								
**Quartz**	**(1,2,8)**	**9.953**	**70.87**	**3.5**	**3.2**	**3.655**	**11.68**	**45.69**	**47.16**
	Equiv. Refl.: (1,-3,-8), (2,1,-8), (2,-3,8), (-3,1,8), (-3,2,-8)								
**Quartz**	**(3,-1,-8)**	**9.953**	**70.87**	**3.4**	**3.2**	**3.655**	**11.68**	**45.69**	**47.16**
	Equiv. Refl.: (3,-2,8), (-1,3,8), (-1,-2,-8), (-2,3,-8), (-2,-1,8)								
**Sapph**	**(1,6,-2)**	**9.911**	**70.18**	**3.3**	**2.9**	**3.796**	**11.08**	**47.45**	**48.73**
	Equiv. Refl.: (1,-7,2), (6,1,2), (6,-7,-2), (7,-1,2), (7,-6,-2), (-1,7,-2), (-1,-6,2), (-6,7,2), (-6,-1,-2) (-7,1,-2), (-7,6,2)								
**Quartz**	**(1,6,-3)**	**10.155**	**74.56**	**3.1**	**2.5**	**2.909**	**7.32**	**36.37**	**37.1**
	Equiv. Refl.: (1,-7,3), (6,1,3), (6,-7,-3), (-7,1,-3), (-7,6,3)								
**Quartz**	**(7,-2,4)**	**10.19**	**75.3**	**2.9**	**2.6**	**2.764**	**7.2**	**34.55**	**35.29**
	Equiv. Refl.: (7,-5,-4), (-2,7,-4), (-2,-5,4), (-5,7,4), (-5,-2,-4)								
**Sapph**	**(2,3,17)**	**10.433**	**82**	**2.7**	**3.3**	**1.48**	**4.9**	**18.5**	**19.14**
	Equiv. Refl.: (2,-5,-17), (3,2,-17), (3,-5,17), (5,-2,-17), (5,-3,17), (-2,5,17), (-2,-3,-17), (-3,5,-17), (-3,-2,17) (-5,2,17), (-5,3,-17), (0,6,-5), (0,-6,5), (6,0,5), (6,-6,-5), (-6,0,-5), (-6,6,5)								
**Quartz**	**(2,5,-4)**	**10.19**	**75.3**	**2.6**	**2.6**	**2.764**	**7.2**	**34.55**	**35.29**
	Equiv. Refl.: (2,-7,4), (5,2,4), (5,-7,-4), (7,-1,-3), (7,-6,3), (-1,7,3), (-1,-6,-3), (-6,7,-3), (-6,-1,3) (-7,2,-4), (-7,5,4)								
**Quartz**	**(7,-1,3)**	**10.155**	**74.56**	**2.4**	**2.5**	**2.909**	**7.32**	**36.37**	**37.1**
	Equiv. Refl.: (7,-6,-3), (-1,7,-3), (-1,-6,3), (-6,7,3), (-6,-1,-3)								

Ei =	10.535	keV						ΔEg	ΔEt
Cryst	Refl	EB	ΘB	∫ IR dΘ	Width	Ei cotΘ	ΔE	2m, 50µm	2m, 50µm
	(h,k,l)	[keV]	[°]	[µrad]	[µrad]	[meV/µrad]	[meV]	[meV]	[meV]
**Quartz**	**(1,6,3)**	**10.155**	**74.56**	**1.9**	**2.3**	**2.909**	**6.83**	**36.37**	**37**
	Equiv. Refl.: (1,-7,-3), (2,5,4), (2,-7,-4), (5,2,-4), (5,-7,4), (6,1,-3), (6,-7,3), (-7,1,3), (-7,2,4) (-7,5,-4), (-7,6,-3)								
**Sapph**	**(2,4,13)**	**10.093**	**73.35**	**1.8**	**2.2**	**3.151**	**6.97**	**39.39**	**40**
	Equiv. Refl.: (2,-6,-13), (4,2,-13), (4,-6,13), (6,-2,-13), (6,-4,13), (-2,6,13), (-2,-4,-13), (-4,6,-13), (-4,-2,13) (-6,2,13), (-6,4,-13), (7,-2,-4), (7,-5,4), (-2,7,4), (-2,-5,-4), (-5,7,-4), (-5,-2,4)								
**Quartz**	**(3,4,4)**	**9.98**	**71.31**	**1.7**	**1.9**	**3.563**	**6.67**	**44.54**	**45.03**
	Equiv. Refl.: (3,-7,-4), (4,3,-4), (4,-7,4), (-7,3,4), (-7,4,-4)								
**Sapph**	**(1,6,7)**	**10.416**	**81.36**	**1.6**	**2.3**	**1.6**	**3.66**	**20**	**20.33**
	Equiv. Refl.: (1,-7,-7), (6,1,-7), (6,-7,7), (7,-1,-7), (7,-6,7), (-1,7,7), (-1,-6,-7), (-6,7,-7), (-6,-1,7) (-7,1,7), (-7,6,-7), (0,1,-9), (0,-1,9), (1,0,9), (1,-1,-9), (-1,0,-9), (-1,1,9)								
**LiNbO3**	**(2,6,-7)**	**10.504**	**85.62**	**1.5**	**4.8**	**0.807**	**3.86**	**10.09**	**10.81**
	Equiv. Refl.: (2,-8,7), (3,4,-13), (3,-7,13), (4,3,13), (4,-7,-13), (6,2,7), (6,-8,-7), (7,-3,13), (7,-4,-13) (8,-2,7), (8,-6,-7), (-2,8,-7), (-2,-6,7), (-3,7,-13), (-3,-4,13), (-4,7,13), (-4,-3,-13), (-6,8,7) (-6,-2,-7), (-7,3,-13), (-7,4,13), (-8,2,-7), (-8,6,7), (0,7,-1), (0,-7,1), (5,-2,-7), (5,-3,7) (7,0,1), (7,-3,-4), (7,-4,4), (7,-7,-1), (-2,5,7), (-2,-3,-7), (-3,5,-7), (-3,7,4), (-3,-2,7) (-3,-4,-4), (-4,7,-4), (-4,-3,4), (-7,0,-1), (-7,7,1)								
**Quartz**	**(2,3,7)**	**10.237**	**76.34**	**1.4**	**1.8**	**2.56**	**4.57**	**31.99**	**32.32**
	Equiv. Refl.: (2,-5,-7), (3,2,-7), (3,-5,7), (-5,2,7), (-5,3,-7)								
**Quartz**	**(8,-4,1)**	**10.159**	**74.65**	**1.3**	**1.5**	**2.892**	**4.43**	**36.15**	**36.42**
	Equiv. Refl.: (8,-4,-1), (-4,8,1), (-4,8,-1), (-4,-4,1), (-4,-4,-1)								
**Quartz**	**(0,7,2)**	**10.454**	**82.88**	**1.2**	**1.9**	**1.316**	**2.44**	**16.45**	**16.63**
	Equiv. Refl.: (0,-7,-2), (7,0,-2), (7,-7,2), (-7,0,2), (-7,7,-2)								
**Quartz**	**(4,4,1)**	**10.159**	**74.65**	**1.1**	**1.5**	**2.892**	**4.43**	**36.15**	**36.42**
	Equiv. Refl.: (4,4,-1), (4,-8,1), (4,-8,-1), (-8,4,1), (-8,4,-1)								
**Sapph**	**(1,6,-5)**	**10.15**	**74.46**	**1**	**1.4**	**2.93**	**4.02**	**36.63**	**36.85**
	Equiv. Refl.: (1,-7,5), (6,1,5), (6,-7,-5), (7,-1,5), (7,-6,-5), (-1,7,-5), (-1,-6,5), (-6,7,5), (-6,-1,-5) (-7,1,-5), (-7,6,5)								
**Sapph**	**(1,2,-19)**	**9.904**	**70.06**	**0.9**	**1.3**	**3.821**	**4.87**	**47.77**	**48.01**
	Equiv. Refl.: (1,-3,19), (2,1,19), (2,-3,-19), (3,-1,19), (3,-2,-19), (-1,3,-19), (-1,-2,19), (-2,3,19), (-2,-1,-19) (-3,1,-19), (-3,2,19), (2,3,-7), (2,-5,7), (3,2,7), (3,-5,-7), (-5,2,-7), (-5,3,7)								
**Quartz**	**(0,5,6)**	**10.022**	**72.04**	**0.8**	**1.1**	**3.415**	**3.78**	**42.69**	**42.85**
	Equiv. Refl.: (0,-5,-6), (5,0,-6), (5,-2,7), (5,-3,-7), (5,-5,6), (-2,5,-7), (-2,-3,7), (-3,5,7), (-3,-2,-7) (-5,0,6), (-5,5,-6)								
**Quartz**	**(3,5,1)**	**10.263**	**76.96**	**0.7**	**1**	**2.441**	**2.34**	**30.51**	**30.6**
	Equiv. Refl.: (3,-8,-1), (5,3,-1), (5,-8,1)								
**Quartz**	**(8,-3,-1)**	**10.263**	**76.96**	**0.6**	**1**	**2.441**	**2.34**	**30.51**	**30.6**
	Equiv. Refl.: (8,-5,1), (-3,8,1), (-3,-5,-1), (-5,8,-1), (-5,-3,1), (-8,3,1), (-8,5,-1)								
**LiNbO3**	**(2,3,-19)**	**10.437**	**82.16**	**0.5**	**2.1**	**1.45**	**3.1**	**18.12**	**18.38**
	Equiv. Refl.: (2,6,5), (2,-5,19), (2,-8,-5), (3,2,19), (3,-5,-19), (5,-2,19), (5,-3,-19), (6,2,-5), (6,-8,5) (8,-2,-5), (8,-6,5), (-2,5,-19), (-2,8,5), (-2,-3,19), (-2,-6,-5), (-3,5,19), (-3,-2,-19), (-5,2,-19) (-5,3,19), (-6,8,-5), (-6,-2,5), (-8,2,5), (-8,6,-5), (2,2,18), (2,2,-18), (2,-4,18), (2,-4,-18) (4,-2,18), (4,-2,-18), (-2,4,18), (-2,4,-18), (-2,-2,18), (-2,-2,-18), (-4,2,18), (-4,2,-18)								
**LiNbO3**	**(2,6,-1)**	**10.037**	**72.31**	**0.4**	**1.3**	**3.36**	**4.49**	**42**	**42.24**
	Equiv. Refl.: (2,-8,1), (6,2,1), (6,-8,-1), (8,-2,1), (8,-6,-1), (-2,8,-1), (-2,-6,1), (-6,8,1), (-6,-2,-1) (-8,2,-1), (-8,6,1)								
**LiNbO3**	**(3,5,7)**	**10.224**	**76.05**	**0.3**	**1.2**	**2.617**	**3.06**	**32.71**	**32.85**
	Equiv. Refl.: (3,-8,-7), (5,3,-7), (5,-8,7), (8,-3,-7), (8,-5,7), (-3,8,7), (-3,-5,-7), (-5,8,-7), (-5,-3,7) (-8,3,7), (-8,5,-7), (3,5,-1), (3,-8,1), (5,3,1), (5,-8,-1), (8,-3,1), (8,-5,-1), (-3,8,-1) (-3,-5,1), (-5,8,1), (-5,-3,-1), (-8,3,-1), (-8,5,1)								
**LiNbO3**	**(3,5,-5)**	**9.987**	**71.44**	**0.2**	**0.9**	**3.538**	**3.31**	**44.22**	**44.35**
	Equiv. Refl.: (3,-8,5), (5,3,5), (5,-8,-5), (8,-3,5), (8,-5,-5), (-3,8,-5), (-3,-5,5), (-5,8,5), (-5,-3,-5)								

	(-8,3,-5), (-8,5,5), (0,5,-6), (0,-5,6), (5,0,6), (5,-5,-6), (-5,0,-6), (-5,5,6)								
**LiNbO3**	**(4,4,9)**	**10.44**	**82.32**	**0**	**0.6**	**1.421**	**0.89**	**17.76**	**17.78**
	Equiv. Refl.: (4,4,-9), (4,-8,9), (4,-8,-9), (8,-4,9), (8,-4,-9), (-4,8,9), (-4,8,-9), (-4,-4,9), (-4,-4,-9) (-8,4,9), (-8,4,-9)								

Ei =	12.968	keV						ΔEg	ΔEt
Cryst	Refl	EB	ΘB	∫ IR dΘ	Width	Ei cotΘ	ΔE	2m, 50µm	2m, 50µm
	(h,k,l)	[keV]	[°]	[µrad]	[µrad]	[meV/µrad]	[meV]	[meV]	[meV]
**Si**	**(0,8,8)**	**12.914**	**84.77**	**8.616**	**7.6**	**1.187**	**8.98**	**14.84**	**17.35**
**Ge**	**(0,6,10)**	**12.779**	**80.2**	**3.901**	**8.4**	**2.239**	**18.82**	**27.99**	**33.73**
	Equiv. Refl.: (6,6,8)								
**Ge**	**(3,3,11)**	**12.919**	**85.02**	**3.889**	**11.2**	**1.13**	**12.7**	**14.12**	**18.99**
	Equiv. Refl.: (3,7,9)								
**Si**	**(2,4,10)**	**12.504**	**74.63**	**3.154**	**2.9**	**3.566**	**10.36**	**44.57**	**45.76**
**Si**	**(1,1,11)**	**12.659**	**77.47**	**2.804**	**2.4**	**2.882**	**6.94**	**36.02**	**36.68**
	Equiv. Refl.: (5,7,7)								
**Ge**	**(0,8,8)**	**12.397**	**72.94**	**2.588**	**5.4**	**3.98**	**21.43**	**49.75**	**54.17**
**Si**	**(3,5,9)**	**12.241**	**70.72**	**1.621**	**1.8**	**4.537**	**8.04**	**56.71**	**57.27**
**Ge**	**(1,3,11)**	**12.542**	**75.27**	**1.536**	**4.2**	**3.409**	**14.42**	**42.62**	**44.99**
	Equiv. Refl.: (1,7,9), (5,5,9)								
**LiNbO3**	**(4,-10,-10)**	**12.921**	**85.09**	**25.271**	**22.7**	**1.113**	**25.31**	**13.91**	**28.89**
	Equiv. Refl.: (6,4,-10), (10,-4,-10), (-4,-6,-10), (-6,10,-10), (-10,6,-10)								
**LiNbO3**	**(4,6,10)**	**12.921**	**85.09**	**24.59**	**22.7**	**1.113**	**25.31**	**13.91**	**28.89**
	Equiv. Refl.: (6,-10,10), (10,-6,10), (-6,-4,10)								
**LiNbO3**	**(-4,10,10)**	**12.921**	**85.09**	**24.589**	**22.7**	**1.113**	**25.31**	**13.91**	**28.89**
	Equiv. Refl.: (-10,4,10)								
**LiNbO3**	**(3,3,-24)**	**12.938**	**86.08**	**23.143**	**21.7**	**0.888**	**19.23**	**11.1**	**22.2**
	Equiv. Refl.: (3,-6,-24), (6,-3,-24), (-3,6,-24), (-3,-3,-24), (-6,3,-24)								
**LiNbO3**	**(3,-6,24)**	**12.938**	**86.08**	**22.244**	**21.7**	**0.888**	**19.23**	**11.1**	**22.2**
	Equiv. Refl.: (6,-3,24)								
**LiNbO3**	**(3,3,24)**	**12.938**	**86.08**	**22.243**	**21.7**	**0.888**	**19.23**	**11.1**	**22.2**
	Equiv. Refl.: (-3,6,24), (-3,-3,24), (-6,3,24)								
**LiNbO3**	**(3,7,8)**	**12.866**	**82.82**	**16.65**	**15.1**	**1.634**	**24.68**	**20.42**	**32.04**
	Equiv. Refl.: (7,-10,8), (10,-7,8), (-3,10,8), (-7,-3,8), (-10,3,8)								
**LiNbO3**	**(3,-10,-8)**	**12.866**	**82.82**	**16.293**	**15.1**	**1.634**	**24.68**	**20.42**	**32.04**
	Equiv. Refl.: (7,3,-8), (10,-3,-8), (-3,-7,-8), (-7,10,-8), (-10,7,-8)								
**LiNbO3**	**(0,4,26)**	**12.888**	**83.65**	**14.213**	**13.3**	**1.443**	**19.2**	**18.03**	**26.34**
	Equiv. Refl.: (4,-4,26), (-4,0,26)								
**LiNbO3**	**(0,-9,6)**	**12.799**	**80.73**	**14.037**	**12.7**	**2.116**	**26.85**	**26.45**	**37.69**
	Equiv. Refl.: (9,0,6), (-9,9,6)								
**LiNbO3**	**(0,9,-6)**	**12.799**	**80.73**	**13.954**	**12.7**	**2.116**	**26.85**	**26.45**	**37.69**
	Equiv. Refl.: (9,-9,-6), (-9,0,-6)								
**LiNbO3**	**(0,-4,-26)**	**12.888**	**83.65**	**13.628**	**13.3**	**1.443**	**19.2**	**18.03**	**26.34**
	Equiv. Refl.: (4,0,-26), (-4,4,-26)								
**Quartz**	**(2,-7,8)**	**12.923**	**85.2**	**13.285**	**11**	**1.089**	**11.93**	**13.61**	**18.1**
**Quartz**	**(2,5,-8)**	**12.923**	**85.2**	**13.284**	**11**	**1.089**	**11.93**	**13.61**	**18.1**
**Quartz**	**(-7,2,-8)**	**12.923**	**85.2**	**13.281**	**11**	**1.089**	**11.93**	**13.61**	**18.1**
	Equiv. Refl.: (-7,5,8)								
**Quartz**	**(5,2,8)**	**12.923**	**85.2**	**13.277**	**10.9**	**1.089**	**11.92**	**13.61**	**18.1**
	Equiv. Refl.: (5,-7,-8)								
**Quartz**	**(-2,7,-8)**	**12.923**	**85.2**	**13.273**	**11**	**1.089**	**11.93**	**13.61**	**18.1**
**Quartz**	**(-2,-5,8)**	**12.923**	**85.2**	**13.272**	**11**	**1.089**	**11.93**	**13.61**	**18.1**
**Quartz**	**(7,-2,8)**	**12.923**	**85.2**	**13.269**	**11**	**1.089**	**11.93**	**13.61**	**18.1**
**Quartz**	**(7,-5,-8)**	**12.923**	**85.2**	**13.268**	**11**	**1.089**	**11.93**	**13.61**	**18.1**
**Quartz**	**(-5,7,8)**	**12.923**	**85.2**	**13.265**	**10.9**	**1.089**	**11.92**	**13.61**	**18.1**
	Equiv. Refl.: (-5,-2,-8)								

Ei =	12.968	keV						ΔEg	ΔEt
Cryst	Refl	EB	ΘB	∫ IR dΘ	Width	Ei cotΘ	ΔE	2m, 50μm	2m, 50μm
	(h,k,l)	[keV]	[°]	[μrad]	[μrad]	[meV/μrad]	[meV]	[meV]	[meV]
**LiNbO3**	**(1,-7,20)**	**12.772**	**80.03**	**12.932**	**11.6**	**2.28**	**26.5**	**28.5**	**38.92**
	Equiv. Refl.: (6,1,20), (7,-1,20), (-1,-6,20), (-6,7,20), (-7,6,20)								
**LiNbO3**	**(0,-8,14)**	**12.765**	**79.84**	**12.917**	**11.2**	**2.324**	**26.06**	**29.05**	**39.03**
	Equiv. Refl.: (8,0,14), (-8,8,14)								
**Quartz**	**(0,2,11)**	**12.949**	**86.92**	**12.893**	**10.9**	**0.697**	**7.63**	**8.71**	**11.58**
	Equiv. Refl.: (0,-2,-11)								
**Quartz**	**(2,0,-11)**	**12.949**	**86.92**	**12.87**	**10.9**	**0.697**	**7.62**	**8.71**	**11.57**
	Equiv. Refl.: (-2,0,11)								
**Quartz**	**(2,-2,11)**	**12.949**	**86.92**	**12.867**	**10.9**	**0.697**	**7.61**	**8.71**	**11.57**
	Equiv. Refl.: (-2,2,-11)								
**LiNbO3**	**(1,6,-20)**	**12.772**	**80.03**	**12.633**	**11.6**	**2.28**	**26.5**	**28.5**	**38.92**
	Equiv. Refl.: (6,-7,-20), (7,-6,-20), (-1,7,-20), (-6,-1,-20), (-7,1,-20)								
**LiNbO3**	**(2,-10,0)**	**12.744**	**79.34**	**12.584**	**11.3**	**2.442**	**27.6**	**30.52**	**41.15**
	Equiv. Refl.: (8,2,0), (10,-2,0), (-2,-8,0), (-8,10,0), (-10,8,0)								
**LiNbO3**	**(2,8,0)**	**12.744**	**79.34**	**12.406**	**11.3**	**2.442**	**27.6**	**30.52**	**41.15**
	Equiv. Refl.: (8,-10,0), (10,-8,0), (-2,10,0), (-8,-2,0), (-10,2,0)								
**LiNbO3**	**(0,-9,-6)**	**12.799**	**80.73**	**12.081**	**10.8**	**2.116**	**22.94**	**26.45**	**35.01**
	Equiv. Refl.: (9,0,-6), (-9,9,-6)								
**LiNbO3**	**(0,8,-14)**	**12.765**	**79.84**	**11.673**	**11.2**	**2.324**	**26.06**	**29.05**	**39.03**
	Equiv. Refl.: (8,-8,-14), (-8,0,-14)								
**LiNbO3**	**(0,-2,-28)**	**12.826**	**81.52**	**11.442**	**10.6**	**1.935**	**20.45**	**24.18**	**31.67**
	Equiv. Refl.: (2,0,-28), (-2,2,-28)								
**LiNbO3**	**(2,-2,28)**	**12.826**	**81.52**	**11.372**	**10.6**	**1.935**	**20.45**	**24.18**	**31.67**
**LiNbO3**	**(0,2,28)**	**12.826**	**81.52**	**11.371**	**10.6**	**1.935**	**20.45**	**24.18**	**31.67**
	Equiv. Refl.: (-2,0,28)								
**LiNbO3**	**(0,9,6)**	**12.799**	**80.73**	**11.253**	**10.8**	**2.116**	**22.94**	**26.45**	**35.01**
	Equiv. Refl.: (9,-9,6), (-9,0,6)								
**Quartz**	**(3,7,0)**	**12.95**	**86.98**	**11.163**	**9.2**	**0.684**	**6.32**	**8.56**	**10.64**
	Equiv. Refl.: (3,-10,0), (7,3,0), (7,-10,0), (-10,3,0), (-10,7,0)								
**Sapph**	**(0,8,10)**	**12.947**	**86.76**	**10.753**	**9.5**	**0.735**	**6.99**	**9.19**	**11.54**
	Equiv. Refl.: (0,-8,-10), (8,0,-10), (8,-8,10), (-8,0,10), (-8,8,-10)								
**Quartz**	**(10,-3,0)**	**12.95**	**86.98**	**10.482**	**9.2**	**0.684**	**6.32**	**8.56**	**10.64**
	Equiv. Refl.: (10,-7,0), (-3,10,0), (-3,-7,0), (-7,10,0), (-7,-3,0)								
**LiNbO3**	**(1,8,-10)**	**12.694**	**78.2**	**9.635**	**8.6**	**2.709**	**23.23**	**33.86**	**41.06**
	Equiv. Refl.: (8,-9,-10), (9,-8,-10), (-1,9,-10), (-8,-1,-10), (-9,1,-10)								
**LiNbO3**	**(4,-9,-14)**	**12.535**	**75.16**	**9.503**	**8.2**	**3.437**	**28.18**	**42.96**	**51.38**
	Equiv. Refl.: (5,4,-14), (9,-4,-14), (-4,-5,-14), (-5,9,-14), (-9,5,-14)								
**LiNbO3**	**(0,1,-28)**	**12.598**	**76.28**	**9.466**	**8.4**	**3.166**	**26.57**	**39.58**	**47.67**
	Equiv. Refl.: (1,-1,-28), (-1,0,-28)								
**LiNbO3**	**(0,-1,28)**	**12.598**	**76.28**	**9.447**	**8.4**	**3.166**	**26.57**	**39.58**	**47.67**
	Equiv. Refl.: (1,0,28), (-1,1,28)								
**LiNbO3**	**(4,-10,8)**	**12.639**	**77.06**	**9.289**	**8.2**	**2.979**	**24.29**	**37.23**	**44.46**
	Equiv. Refl.: (6,4,8), (10,-4,8), (-4,-6,8), (-6,10,8), (-10,6,8)								
**LiNbO3**	**(1,-9,10)**	**12.694**	**78.2**	**9.266**	**8.6**	**2.709**	**23.23**	**33.86**	**41.06**
	Equiv. Refl.: (8,1,10), (9,-1,10), (-1,-8,10), (-8,9,10), (-9,8,10)								
**LiNbO3**	**(5,-9,14)**	**12.535**	**75.16**	**9.143**	**8.2**	**3.437**	**28.18**	**42.96**	**51.38**
	Equiv. Refl.: (9,-5,14)								
**LiNbO3**	**(4,5,14)**	**12.535**	**75.16**	**9.142**	**8.2**	**3.437**	**28.18**	**42.96**	**51.38**
	Equiv. Refl.: (-4,9,14), (-5,-4,14), (-9,4,14)								

Ei =	12.968	keV						ΔEg	ΔEt
Cryst	Refl	EB	ΘB	∫ IR dΘ	Width	Ei cotΘ	ΔE	2m, 50μm	2m, 50μm
	(h,k,l)	[keV]	[°]	[μrad]	[μrad]	[meV/μrad]	[meV]	[meV]	[meV]
**LiNbO3**	**(1,4,-24)**	**12.481**	**74.25**	**8.839**	**7.8**	**3.657**	**28.42**	**45.71**	**53.82**
	Equiv. Refl.: (4,-5,-24), (5,-4,-24), (-1,5,-24), (-4,-1,-24), (-5,1,-24)								
**LiNbO3**	**(1,-5,24)**	**12.481**	**74.25**	**8.832**	**7.8**	**3.657**	**28.42**	**45.71**	**53.82**
	Equiv. Refl.: (4,1,24), (5,-1,24), (-1,-4,24), (-4,5,24), (-5,4,24)								
**LiNbO3**	**(4,6,-8)**	**12.639**	**77.06**	**8.807**	**8.2**	**2.979**	**24.29**	**37.23**	**44.46**
	Equiv. Refl.: (6,-10,-8), (10,-6,-8), (-4,10,-8), (-6,-4,-8), (-10,4,-8)								
**LiNbO3**	**(1,4,24)**	**12.481**	**74.25**	**8.787**	**7.7**	**3.657**	**28.23**	**45.71**	**53.72**
	Equiv. Refl.: (4,-5,24), (5,-4,24), (-1,5,24), (-4,-1,24), (-5,1,24)								
**LiNbO3**	**(1,-5,-24)**	**12.481**	**74.25**	**8.739**	**7.7**	**3.657**	**28.23**	**45.71**	**53.72**
	Equiv. Refl.: (4,1,-24), (5,-1,-24), (-1,-4,-24), (-4,5,-24), (-5,4,-24)								
**Quartz**	**(-6,4,-9)**	**12.884**	**83.49**	**8.342**	**6.7**	**1.48**	**9.88**	**18.5**	**20.98**
**Quartz**	**(-6,2,9)**	**12.884**	**83.49**	**8.34**	**6.7**	**1.48**	**9.88**	**18.5**	**20.98**
**Quartz**	**(2,4,9)**	**12.884**	**83.49**	**8.338**	**6.7**	**1.48**	**9.88**	**18.5**	**20.97**
	Equiv. Refl.: (2,-6,-9), (4,-6,9)								
**Quartz**	**(4,2,-9)**	**12.884**	**83.49**	**8.337**	**6.7**	**1.48**	**9.88**	**18.5**	**20.97**
**Quartz**	**(6,-4,9)**	**12.884**	**83.49**	**8.069**	**6.7**	**1.48**	**9.88**	**18.5**	**20.98**
**Quartz**	**(6,-2,-9)**	**12.884**	**83.49**	**8.066**	**6.7**	**1.48**	**9.88**	**18.5**	**20.98**
**Quartz**	**(-2,6,9)**	**12.884**	**83.49**	**8.065**	**6.7**	**1.48**	**9.88**	**18.5**	**20.97**
	Equiv. Refl.: (-2,-4,-9), (-4,6,-9), (-4,-2,9)								
**LiNbO3**	**(4,4,-18)**	**12.554**	**75.48**	**8.022**	**7.2**	**3.359**	**24.06**	**41.99**	**48.39**
	Equiv. Refl.: (4,-8,-18), (8,-4,-18), (-4,8,-18), (-4,-4,-18), (-8,4,-18)								
**LiNbO3**	**(3,4,20)**	**12.31**	**71.66**	**7.935**	**6.9**	**4.298**	**29.87**	**53.72**	**61.47**
	Equiv. Refl.: (4,-7,20), (7,-4,20), (-3,7,20), (-4,-3,20), (-7,3,20)								
**LiNbO3**	**(3,-7,-20)**	**12.31**	**71.66**	**7.924**	**6.9**	**4.298**	**29.87**	**53.72**	**61.47**
	Equiv. Refl.: (4,3,-20), (7,-3,-20), (-3,-4,-20), (-4,7,-20), (-7,4,-20)								
**LiNbO3**	**(1,-6,22)**	**12.519**	**74.88**	**7.804**	**7**	**3.505**	**24.37**	**43.81**	**50.13**
	Equiv. Refl.: (5,1,22), (6,-1,22), (-1,-5,22), (-5,6,22), (-6,5,22)								
**LiNbO3**	**(4,4,18)**	**12.554**	**75.48**	**7.802**	**7.2**	**3.359**	**24.06**	**41.99**	**48.39**
	Equiv. Refl.: (4,-8,18), (8,-4,18), (-4,8,18), (-4,-4,18), (-8,4,18)								
**LiNbO3**	**(3,7,-4)**	**12.488**	**74.36**	**7.744**	**6.9**	**3.631**	**25.05**	**45.39**	**51.84**
	Equiv. Refl.: (7,-10,-4), (10,-7,-4), (-3,10,-4), (-7,-3,-4), (-10,3,-4)								
**LiNbO3**	**(1,-4,26)**	**12.661**	**77.52**	**7.678**	**7.1**	**2.871**	**20.36**	**35.88**	**41.26**
	Equiv. Refl.: (3,1,26), (4,-1,26), (-1,-3,26), (-3,4,26), (-4,3,26)								
**LiNbO3**	**(0,9,0)**	**12.514**	**74.8**	**7.628**	**6.7**	**3.523**	**23.77**	**44.04**	**50.04**
	Equiv. Refl.: (9,-9,0), (-9,0,0)								
**LiNbO3**	**(3,-10,4)**	**12.488**	**74.36**	**7.585**	**6.9**	**3.631**	**25.05**	**45.39**	**51.84**
	Equiv. Refl.: (7,3,4), (10,-3,4), (-3,-7,4), (-7,10,4), (-10,7,4)								
**LiNbO3**	**(1,5,-22)**	**12.519**	**74.88**	**7.583**	**7**	**3.505**	**24.37**	**43.81**	**50.13**
	Equiv. Refl.: (5,-6,-22), (6,-5,-22), (-1,6,-22), (-5,-1,-22), (-6,1,-22)								
**LiNbO3**	**(1,3,-26)**	**12.661**	**77.52**	**7.528**	**7.1**	**2.871**	**20.36**	**35.88**	**41.26**
	Equiv. Refl.: (3,-4,-26), (4,-3,-26), (-1,4,-26), (-3,-1,-26), (-4,1,-26)								
**Quartz**	**(-6,9,-5)**	**12.908**	**84.51**	**7.45**	**6.1**	**1.247**	**7.63**	**15.59**	**17.35**
**Quartz**	**(-3,9,5)**	**12.908**	**84.51**	**7.449**	**6.1**	**1.247**	**7.63**	**15.59**	**17.35**
	Equiv. Refl.: (-3,-6,-5)								
**Quartz**	**(-6,-3,5)**	**12.908**	**84.51**	**7.448**	**6.1**	**1.247**	**7.63**	**15.59**	**17.35**
**Quartz**	**(9,-3,-5)**	**12.908**	**84.51**	**7.443**	**6.1**	**1.247**	**7.62**	**15.59**	**17.35**
	Equiv. Refl.: (9,-6,5)								
**LiNbO3**	**(2,-9,-10)**	**12.229**	**70.56**	**7.243**	**6.2**	**4.577**	**28.47**	**57.22**	**63.91**
	Equiv. Refl.: (7,2,-10), (9,-2,-10), (-2,-7,-10), (-7,9,-10), (-9,7,-10)								
**LiNbO3**	**(0,-9,0)**	**12.514**	**74.8**	**7.227**	**6.7**	**3.523**	**23.77**	**44.04**	**50.04**
	Equiv. Refl.: (9,0,0), (-9,9,0)								

Ei =	12.968	keV						ΔEg	ΔEt
Cryst	Refl	EB	ΘB	∫ IR dΘ	Width	Ei cotΘ	ΔE	2m, 50µm	2m, 50µm
	(h,k,l)	[keV]	[°]	[µrad]	[µrad]	[meV/µrad]	[meV]	[meV]	[meV]
**Quartz**	**(6,-9,5)**	**12.908**	**84.51**	**7.084**	**6.1**	**1.247**	**7.63**	**15.59**	**17.35**
**Quartz**	**(3,6,5)**	**12.908**	**84.51**	**7.083**	**6.1**	**1.247**	**7.63**	**15.59**	**17.35**
	Equiv. Refl.: (3,-9,-5)								
**Quartz**	**(6,3,-5)**	**12.908**	**84.51**	**7.082**	**6.1**	**1.247**	**7.63**	**15.59**	**17.35**
**Quartz**	**(-9,3,5)**	**12.908**	**84.51**	**7.077**	**6.1**	**1.247**	**7.62**	**15.59**	**17.35**
	Equiv. Refl.: (-9,6,-5)								
**LiNbO3**	**(3,6,12)**	**12.272**	**71.14**	**7.046**	**6.2**	**4.429**	**27.51**	**55.36**	**61.82**
	Equiv. Refl.: (6,-9,12), (9,-6,12), (-3,9,12), (-6,-3,12), (-9,3,12)								
**Quartz**	**(5,5,0)**	**12.618**	**76.66**	**6.966**	**5.3**	**3.076**	**16.23**	**38.45**	**41.74**
	Equiv. Refl.: (5,-10,0), (-10,5,0)								
**Quartz**	**(10,-5,0)**	**12.618**	**76.66**	**6.96**	**5.3**	**3.076**	**16.23**	**38.45**	**41.74**
	Equiv. Refl.: (-5,10,0), (-5,-5,0)								
**LiNbO3**	**(3,-9,-12)**	**12.272**	**71.14**	**6.91**	**6.2**	**4.429**	**27.51**	**55.36**	**61.82**
	Equiv. Refl.: (6,3,-12), (9,-3,-12), (-3,-6,-12), (-6,9,-12), (-9,6,-12)								
**LiNbO3**	**(3,-10,-2)**	**12.391**	**72.85**	**6.836**	**6.1**	**4.002**	**24.39**	**50.03**	**55.66**
	Equiv. Refl.: (7,3,-2), (10,-3,-2), (-3,-7,-2), (-7,10,-2), (-10,7,-2)								
**LiNbO3**	**(2,7,10)**	**12.229**	**70.56**	**6.8**	**6.2**	**4.577**	**28.47**	**57.22**	**63.91**
	Equiv. Refl.: (7,-9,10), (9,-7,10), (-2,9,10), (-7,-2,10), (-9,2,10)								
**LiNbO3**	**(2,6,-16)**	**12.318**	**71.78**	**6.671**	**5.9**	**4.268**	**25.15**	**53.35**	**58.98**
	Equiv. Refl.: (6,-8,-16), (8,-6,-16), (-2,8,-16), (-6,-2,-16), (-8,2,-16)								
**Quartz**	**(-1,-1,11)**	**12.867**	**82.85**	**6.66**	**5.5**	**1.627**	**8.9**	**20.34**	**22.2**
**Quartz**	**(-1,2,-11)**	**12.867**	**82.85**	**6.659**	**5.5**	**1.627**	**8.89**	**20.34**	**22.2**
	Equiv. Refl.: (-1,-1,-11)								
**Quartz**	**(-1,2,11)**	**12.867**	**82.85**	**6.657**	**5.5**	**1.627**	**8.89**	**20.34**	**22.2**
**Quartz**	**(2,-1,11)**	**12.867**	**82.85**	**6.654**	**5.5**	**1.627**	**8.89**	**20.34**	**22.2**
	Equiv. Refl.: (2,-1,-11)								
**LiNbO3**	**(3,7,2)**	**12.391**	**72.85**	**6.597**	**6.1**	**4.002**	**24.39**	**50.03**	**55.66**
	Equiv. Refl.: (7,-10,2), (10,-7,2), (-3,10,2), (-7,-3,2), (-10,3,2)								
**Quartz**	**(1,1,-11)**	**12.867**	**82.85**	**6.594**	**5.5**	**1.627**	**8.9**	**20.34**	**22.2**
**Quartz**	**(1,1,11)**	**12.867**	**82.85**	**6.593**	**5.5**	**1.627**	**8.89**	**20.34**	**22.2**
	Equiv. Refl.: (1,-2,11)								
**Quartz**	**(1,-2,-11)**	**12.867**	**82.85**	**6.591**	**5.5**	**1.627**	**8.89**	**20.34**	**22.2**
**Quartz**	**(-2,1,11)**	**12.867**	**82.85**	**6.588**	**5.5**	**1.627**	**8.89**	**20.34**	**22.2**
	Equiv. Refl.: (-2,1,-11)								
**Sapph**	**(2,6,14)**	**12.741**	**79.27**	**6.536**	**5.3**	**2.457**	**13.06**	**30.71**	**33.38**
	Equiv. Refl.: (2,-8,-14), (6,2,-14), (6,-8,14), (8,-2,-14), (8,-6,14), (-2,8,14), (-2,-6,-14), (-6,8,-14), (-6,-2,14) (-8,2,14), (-8,6,-14)								
**LiNbO3**	**(1,8,8)**	**12.407**	**73.09**	**6.497**	**5.9**	**3.943**	**23.29**	**49.28**	**54.51**
	Equiv. Refl.: (8,-9,8), (9,-8,8), (-1,9,8), (-8,-1,8), (-9,1,8)								
**LiNbO3**	**(1,-9,-8)**	**12.407**	**73.09**	**6.414**	**5.9**	**3.943**	**23.29**	**49.28**	**54.51**
	Equiv. Refl.: (8,1,-8), (9,-1,-8), (-1,-8,-8), (-8,9,-8), (-9,8,-8)								
**LiNbO3**	**(2,-8,16)**	**12.318**	**71.78**	**6.365**	**5.9**	**4.268**	**25.15**	**53.35**	**58.98**
	Equiv. Refl.: (8,-2,16)								
**LiNbO3**	**(6,2,16)**	**12.318**	**71.78**	**6.364**	**5.9**	**4.268**	**25.15**	**53.35**	**58.98**
	Equiv. Refl.: (-2,-6,16), (-6,8,16), (-8,6,16)								
**LiNbO3**	**(1,2,26)**	**12.195**	**70.11**	**6.358**	**5.7**	**4.691**	**26.73**	**58.63**	**64.44**
	Equiv. Refl.: (2,-3,26), (3,-2,26), (-1,3,26), (-2,-1,26), (-3,1,26)								
**LiNbO3**	**(1,-3,-26)**	**12.195**	**70.11**	**6.347**	**5.7**	**4.691**	**26.73**	**58.63**	**64.44**
	Equiv. Refl.: (2,1,-26), (3,-1,-26), (-1,-2,-26), (-2,3,-26), (-3,2,-26)								

Ei =	12.968	keV						ΔEg	ΔEt
Cryst	Refl	EB	ΘB	∫ IR dΘ	Width	Ei cotΘ	ΔE	2m, 50μm	2m, 50μm
	(h,k,l)	[keV]	[°]	[μrad]	[μrad]	[meV/μrad]	[meV]	[meV]	[meV]
Quartz	(2,4,-9)	12.884	83.49	6.265	5.2	1.48	7.76	18.5	20.06
	Equiv. Refl.: (2,-6,9)								
Quartz	(4,2,9)	12.884	83.49	6.258	5.2	1.48	7.75	18.5	20.06
	Equiv. Refl.: (-6,2,-9)								
Quartz	(4,-6,-9)	12.884	83.49	6.257	5.2	1.48	7.75	18.5	20.06
	Equiv. Refl.: (-6,4,9)								
Quartz	(-2,6,-9)	12.884	83.49	6.246	5.2	1.48	7.76	18.5	20.06
	Equiv. Refl.: (-2,-4,9)								
Quartz	(6,-2,9)	12.884	83.49	6.239	5.2	1.48	7.75	18.5	20.06
	Equiv. Refl.: (-4,-2,-9)								
Quartz	(6,-4,-9)	12.884	83.49	6.238	5.2	1.48	7.75	18.5	20.06
	Equiv. Refl.: (-4,6,9)								
LiNbO3	(3,-9,12)	12.272	71.14	6.068	5.5	4.429	24.29	55.36	60.46
	Equiv. Refl.: (6,3,12), (9,-3,12), (-3,-6,12), (-6,9,12), (-9,6,12)								
LiNbO3	(3,6,-12)	12.272	71.14	5.966	5.5	4.429	24.29	55.36	60.46
	Equiv. Refl.: (6,-9,-12), (9,-6,-12), (-3,9,-12), (-6,-3,-12), (-9,3,-12)								
LiNbO3	(5,5,6)	12.337	72.06	5.865	5.3	4.199	22.32	52.49	57.04
	Equiv. Refl.: (5,-10,6), (10,-5,6), (-5,10,6), (-5,-5,6), (-10,5,6)								
LiNbO3	(4,6,4)	12.253	70.89	5.849	5.3	4.493	23.98	56.17	61.07
	Equiv. Refl.: (6,-10,4), (10,-6,4), (-4,10,4), (-6,-4,4), (-10,4,4)								
LiNbO3	(4,-10,-4)	12.253	70.89	5.824	5.3	4.493	23.98	56.17	61.07
	Equiv. Refl.: (6,4,-4), (10,-4,-4), (-4,-6,-4), (-6,10,-4), (-10,6,-4)								
LiNbO3	(2,-6,-22)	12.285	71.32	5.739	5.3	4.384	23.11	54.8	59.47
	Equiv. Refl.: (4,2,-22), (6,-2,-22), (-2,-4,-22), (-4,6,-22), (-6,4,-22)								
Quartz	(10,-4,-2)	12.907	84.45	5.73	4.6	1.26	5.81	15.75	16.79
	Equiv. Refl.: (-4,10,2), (-4,-6,-2)								
Quartz	(10,-6,2)	12.907	84.45	5.729	4.6	1.26	5.81	15.75	16.79
	Equiv. Refl.: (-6,10,-2), (-6,-4,2)								
LiNbO3	(2,4,22)	12.285	71.32	5.72	5.3	4.384	23.11	54.8	59.47
	Equiv. Refl.: (4,-6,22), (6,-4,22), (-2,6,22), (-4,-2,22), (-6,2,22)								
Sapph	(2,2,24)	12.585	76.04	5.71	4.6	3.225	14.78	40.31	42.93
	Equiv. Refl.: (2,2,-24), (2,-4,24), (2,-4,-24), (4,-2,24), (4,-2,-24), (-2,4,24), (-2,4,-24), (-2,-2,24), (-2,-2,-24) (-4,2,24), (-4,2,-24)								
Quartz	(9,-4,-5)	12.743	79.31	5.65	4.6	2.448	11.16	30.6	32.57
Quartz	(9,-5,5)	12.743	79.31	5.649	4.6	2.448	11.16	30.6	32.57
Quartz	(-4,9,5)	12.743	79.31	5.648	4.6	2.448	11.16	30.6	32.57
	Equiv. Refl.: (-4,-5,-5), (-5,9,-5), (-5,-4,5)								
Quartz	(-9,4,5)	12.743	79.31	5.63	4.6	2.448	11.16	30.6	32.57
Quartz	(-9,5,-5)	12.743	79.31	5.629	4.6	2.448	11.16	30.6	32.57
Quartz	(4,5,5)	12.743	79.31	5.628	4.6	2.448	11.16	30.6	32.57
	Equiv. Refl.: (4,-9,-5), (5,4,-5), (5,-9,5)								
LiNbO3	(5,5,-6)	12.337	72.06	5.625	5.3	4.199	22.32	52.49	57.04
	Equiv. Refl.: (5,-10,-6), (10,-5,-6), (-5,10,-6), (-5,-5,-6), (-10,5,-6)								
Quartz	(6,-10,-1)	12.753	79.56	5.518	4.4	2.389	10.48	29.86	31.65
	Equiv. Refl.: (-10,4,-1), (-10,6,1)								
Quartz	(4,6,-1)	12.753	79.56	5.517	4.4	2.389	10.48	29.86	31.65
	Equiv. Refl.: (4,-10,1), (6,4,1)								
Quartz	(-8,4,7)	12.898	84.05	5.5	4.6	1.352	6.19	16.9	17.99
	Equiv. Refl.: (-8,4,-7)								
Quartz	(4,-8,-7)	12.898	84.05	5.499	4.6	1.352	6.19	16.9	17.99
Quartz	(4,4,7)	12.898	84.05	5.498	4.6	1.352	6.18	16.9	17.99

Ei =	12.968	keV						ΔEg	ΔEt
**Cryst**	**Refl**	**EB**	**ΘB**	**∫ IR dΘ**	**Width**	**Ei cotΘ**	**ΔE**	**2m, 50µm**	**2m, 50µm**
	**(h,k,l)**	**[keV]**	**[°]**	**[µrad]**	**[µrad]**	**[meV/µrad]**	**[meV]**	**[meV]**	**[meV]**
**Quartz**	**(4,-8,7)**	**12.898**	**84.05**	**5.497**	**4.6**	**1.352**	**6.18**	**16.9**	**17.99**
**Quartz**	**(4,4,-7)**	**12.898**	**84.05**	**5.496**	**4.6**	**1.352**	**6.18**	**16.9**	**17.99**
**Quartz**	**(8,-4,-7)**	**12.898**	**84.05**	**5.48**	**4.6**	**1.352**	**6.19**	**16.9**	**17.99**
**Quartz**	**(8,-4,7)**	**12.898**	**84.05**	**5.479**	**4.6**	**1.352**	**6.19**	**16.9**	**17.99**
	Equiv. Refl.: (-4,8,7)								
**Quartz**	**(-4,-4,-7)**	**12.898**	**84.05**	**5.478**	**4.6**	**1.352**	**6.18**	**16.9**	**17.99**
**Quartz**	**(-4,8,-7)**	**12.898**	**84.05**	**5.477**	**4.6**	**1.352**	**6.18**	**16.9**	**17.99**
	Equiv. Refl.: (-4,-4,7)								
**Quartz**	**(10,-4,1)**	**12.753**	**79.56**	**5.338**	**4.4**	**2.389**	**10.48**	**29.86**	**31.65**
	Equiv. Refl.: (10,-6,-1), (-6,10,1), (-6,-4,-1)								
**Quartz**	**(-4,10,-1)**	**12.753**	**79.56**	**5.337**	**4.4**	**2.389**	**10.48**	**29.86**	**31.65**
	Equiv. Refl.: (-4,-6,1)								
**Quartz**	**(4,-4,-10)**	**12.866**	**82.8**	**5.221**	**4.3**	**1.637**	**7**	**20.47**	**21.63**
	Equiv. Refl.: (-4,4,10)								
**Quartz**	**(4,0,10)**	**12.866**	**82.8**	**5.219**	**4.3**	**1.637**	**6.99**	**20.47**	**21.63**
	Equiv. Refl.: (-4,0,-10)								
**Quartz**	**(0,4,-10)**	**12.866**	**82.8**	**5.218**	**4.3**	**1.637**	**6.99**	**20.47**	**21.63**
	Equiv. Refl.: (0,-4,10)								
**Quartz**	**(4,6,2)**	**12.907**	**84.45**	**5.213**	**4.6**	**1.26**	**5.81**	**15.75**	**16.79**
	Equiv. Refl.: (4,-10,-2), (-10,4,2), (-10,6,-2)								
**Quartz**	**(6,4,-2)**	**12.907**	**84.45**	**5.212**	**4.6**	**1.26**	**5.81**	**15.75**	**16.79**
	Equiv. Refl.: (6,-10,2)								
**Sapph**	**(3,5,-14)**	**12.472**	**74.1**	**4.792**	**3.7**	**3.693**	**13.7**	**46.17**	**48.16**
	Equiv. Refl.: (3,-8,14), (5,3,14), (5,-8,-14), (8,-3,14), (8,-5,-14), (-3,8,-14), (-3,-5,14), (-5,8,14), (-5,-3,-14) (-8,3,-14), (-8,5,14)								
**Sapph**	**(1,8,-1)**	**12.863**	**82.69**	**4.668**	**4.1**	**1.663**	**6.74**	**20.79**	**21.86**
	Equiv. Refl.: (1,-9,1), (8,1,1), (8,-9,-1), (9,-1,1), (9,-8,-1), (-1,9,-1), (-1,-8,1), (-8,9,1), (-8,-1,-1) (-9,1,-1), (-9,8,1)								
**Quartz**	**(-2,-7,-4)**	**12.778**	**80.18**	**4.659**	**3.6**	**2.244**	**8.14**	**28.05**	**29.21**
	Equiv. Refl.: (-7,9,-4), (-7,-2,4)								
**Quartz**	**(-2,9,4)**	**12.778**	**80.18**	**4.658**	**3.6**	**2.244**	**8.14**	**28.05**	**29.21**
**Quartz**	**(9,-2,-4)**	**12.778**	**80.18**	**4.655**	**3.6**	**2.244**	**8.13**	**28.05**	**29.21**
	Equiv. Refl.: (9,-7,4)								
**Quartz**	**(7,-4,-8)**	**12.757**	**79.65**	**4.591**	**3.6**	**2.367**	**8.6**	**29.59**	**30.82**
	Equiv. Refl.: (-4,7,8), (-4,-3,-8)								
**Quartz**	**(7,-3,8)**	**12.757**	**79.65**	**4.59**	**3.6**	**2.367**	**8.6**	**29.59**	**30.82**
	Equiv. Refl.: (-3,7,-8), (-3,-4,8)								
**Quartz**	**(-5,8,-6)**	**12.304**	**71.58**	**4.54**	**3.4**	**4.319**	**14.48**	**53.98**	**55.89**
	Equiv. Refl.: (-5,-3,6)								
**Quartz**	**(8,-3,-6)**	**12.304**	**71.58**	**4.539**	**3.4**	**4.319**	**14.48**	**53.98**	**55.89**
	Equiv. Refl.: (8,-5,6), (-3,8,6), (-3,-5,-6)								
**Quartz**	**(4,-7,-8)**	**12.757**	**79.65**	**4.536**	**3.6**	**2.367**	**8.6**	**29.59**	**30.82**
**Quartz**	**(4,3,8)**	**12.757**	**79.65**	**4.535**	**3.6**	**2.367**	**8.6**	**29.59**	**30.82**
	Equiv. Refl.: (-7,4,8)								
**Quartz**	**(3,4,-8)**	**12.757**	**79.65**	**4.534**	**3.6**	**2.367**	**8.6**	**29.59**	**30.82**
	Equiv. Refl.: (3,-7,8), (-7,3,-8)								
**Quartz**	**(6,-9,-5)**	**12.908**	**84.51**	**4.519**	**3.9**	**1.247**	**4.9**	**15.59**	**16.34**
**Quartz**	**(6,3,5)**	**12.908**	**84.51**	**4.518**	**3.9**	**1.247**	**4.9**	**15.59**	**16.34**
**Quartz**	**(-9,6,5)**	**12.908**	**84.51**	**4.517**	**3.9**	**1.247**	**4.9**	**15.59**	**16.34**
**Quartz**	**(-9,3,-5)**	**12.908**	**84.51**	**4.516**	**3.9**	**1.247**	**4.9**	**15.59**	**16.34**

Ei = Cryst	12.968 Refl (h,k,l)	keV EB [keV]	ΘB [°]	∫ IR dΘ [µrad]	Width [µrad]	Ei cotΘ [meV/µrad]	ΔE [meV]	ΔEg 2m, 50µm [meV]	ΔEt 2m, 50µm [meV]
**Quartz**	**(3,6,-5)**	**12.908**	**84.51**	**4.515**	**3.9**	**1.247**	**4.9**	**15.59**	**16.34**
	Equiv. Refl.: (3,-9,5)								
**Quartz**	**(5,3,-6)**	**12.304**	**71.58**	**4.508**	**3.4**	**4.319**	**14.48**	**53.98**	**55.89**
	Equiv. Refl.: (5,-8,6)								
**Quartz**	**(3,5,6)**	**12.304**	**71.58**	**4.507**	**3.4**	**4.319**	**14.47**	**53.98**	**55.89**
	Equiv. Refl.: (3,-8,-6), (-8,3,6), (-8,5,-6)								
**Quartz**	**(2,7,4)**	**12.778**	**80.18**	**4.494**	**3.6**	**2.244**	**8.14**	**28.05**	**29.21**
	Equiv. Refl.: (7,2,-4), (7,-9,4)								
**Quartz**	**(2,-9,-4)**	**12.778**	**80.18**	**4.493**	**3.6**	**2.244**	**8.14**	**28.05**	**29.21**
**Quartz**	**(-9,2,4)**	**12.778**	**80.18**	**4.491**	**3.6**	**2.244**	**8.13**	**28.05**	**29.21**
	Equiv. Refl.: (-9,7,-4)								
**Quartz**	**(-6,9,5)**	**12.908**	**84.51**	**4.482**	**3.9**	**1.247**	**4.9**	**15.59**	**16.34**
**Quartz**	**(-6,-3,-5)**	**12.908**	**84.51**	**4.481**	**3.9**	**1.247**	**4.9**	**15.59**	**16.34**
**Quartz**	**(9,-6,-5)**	**12.908**	**84.51**	**4.48**	**3.9**	**1.247**	**4.9**	**15.59**	**16.34**
**Quartz**	**(9,-3,5)**	**12.908**	**84.51**	**4.479**	**3.9**	**1.247**	**4.9**	**15.59**	**16.34**
	Equiv. Refl.: (-3,-6,5)								
**Quartz**	**(-3,9,-5)**	**12.908**	**84.51**	**4.478**	**3.9**	**1.247**	**4.9**	**15.59**	**16.34**
**Quartz**	**(0,8,-4)**	**12.526**	**75**	**4.398**	**3.3**	**3.474**	**11.58**	**43.42**	**44.94**
	Equiv. Refl.: (0,-8,4)								
**Quartz**	**(8,0,4)**	**12.526**	**75**	**4.396**	**3.3**	**3.474**	**11.57**	**43.42**	**44.94**
	Equiv. Refl.: (8,-8,-4), (-8,0,-4), (-8,8,4)								
**Sapph**	**(0,6,18)**	**12.461**	**73.93**	**4.386**	**3.4**	**3.735**	**12.87**	**46.69**	**48.43**
	Equiv. Refl.: (0,6,-18), (0,-6,18), (0,-6,-18), (6,0,18), (6,0,-18), (6,-6,18), (6,-6,-18), (-6,0,18), (-6,0,-18) (-6,6,18), (-6,6,-18)								
**Quartz**	**(-9,8,-3)**	**12.915**	**84.83**	**4.376**	**3.7**	**1.172**	**4.35**	**14.65**	**15.29**
**Quartz**	**(1,8,3)**	**12.915**	**84.83**	**4.375**	**3.7**	**1.172**	**4.35**	**14.65**	**15.29**
	Equiv. Refl.: (1,-9,-3), (-9,1,3)								
**Quartz**	**(8,1,-3)**	**12.915**	**84.83**	**4.373**	**3.7**	**1.172**	**4.35**	**14.65**	**15.29**
**Quartz**	**(8,-9,3)**	**12.915**	**84.83**	**4.372**	**3.7**	**1.172**	**4.35**	**14.65**	**15.29**
**Quartz**	**(6,-3,-9)**	**12.802**	**80.81**	**4.343**	**3.5**	**2.097**	**7.34**	**26.21**	**27.22**
	Equiv. Refl.: (-3,6,9), (-3,6,-9), (-3,-3,9), (-3,-3,-9)								
**Quartz**	**(6,-3,9)**	**12.802**	**80.81**	**4.342**	**3.5**	**2.097**	**7.33**	**26.21**	**27.22**
**Quartz**	**(-6,3,9)**	**12.802**	**80.81**	**4.324**	**3.5**	**2.097**	**7.34**	**26.21**	**27.22**
**Quartz**	**(3,3,9)**	**12.802**	**80.81**	**4.323**	**3.5**	**2.097**	**7.33**	**26.21**	**27.22**
	Equiv. Refl.: (3,3,-9), (3,-6,9), (3,-6,-9), (-6,3,-9)								
**Quartz**	**(-1,9,3)**	**12.915**	**84.83**	**3.912**	**3.7**	**1.172**	**4.35**	**14.65**	**15.29**
	Equiv. Refl.: (-1,-8,-3)								
**Quartz**	**(9,-1,-3)**	**12.915**	**84.83**	**3.911**	**3.7**	**1.172**	**4.35**	**14.65**	**15.29**
	Equiv. Refl.: (9,-8,3)								
**Quartz**	**(-8,9,-3)**	**12.915**	**84.83**	**3.909**	**3.7**	**1.172**	**4.35**	**14.65**	**15.29**
	Equiv. Refl.: (-8,-1,3)								
**Quartz**	**(7,-6,-7)**	**12.48**	**74.23**	**3.874**	**2.9**	**3.662**	**10.75**	**45.78**	**47.02**
**Quartz**	**(7,-1,7)**	**12.48**	**74.23**	**3.873**	**2.9**	**3.662**	**10.75**	**45.78**	**47.02**
**Quartz**	**(-6,-1,-7)**	**12.48**	**74.23**	**3.872**	**2.9**	**3.662**	**10.75**	**45.78**	**47.02**
**Quartz**	**(-1,7,-7)**	**12.48**	**74.23**	**3.871**	**2.9**	**3.662**	**10.75**	**45.78**	**47.02**
	Equiv. Refl.: (-1,-6,7), (-6,7,7)								
**Quartz**	**(-8,9,3)**	**12.915**	**84.83**	**3.834**	**3.5**	**1.172**	**4.06**	**14.65**	**15.2**
	Equiv. Refl.: (-8,-1,-3)								
**Quartz**	**(-1,9,-3)**	**12.915**	**84.83**	**3.833**	**3.5**	**1.172**	**4.05**	**14.65**	**15.2**
**Quartz**	**(-1,-8,3)**	**12.915**	**84.83**	**3.832**	**3.5**	**1.172**	**4.05**	**14.65**	**15.2**

Ei =	12.968	keV						ΔEg	ΔEt
Cryst	Refl	EB	ΘB	∫IR dΘ	Width	Ei cotΘ	ΔE	2m, 50µm	2m, 50µm
	(h,k,l)	[keV]	[°]	[µrad]	[µrad]	[meV/µrad]	[meV]	[meV]	[meV]
**Quartz**	**(9,-1,3)**	**12.915**	**84.83**	**3.831**	**3.5**	**1.172**	**4.05**	**14.65**	**15.2**
	Equiv. Refl.: (9,-8,-3)								
**Quartz**	**(8,1,3)**	**12.915**	**84.83**	**3.77**	**3.5**	**1.172**	**4.06**	**14.65**	**15.2**
	Equiv. Refl.: (8,-9,-3)								
**Quartz**	**(1,-9,3)**	**12.915**	**84.83**	**3.769**	**3.5**	**1.172**	**4.05**	**14.65**	**15.2**
**Quartz**	**(1,8,-3)**	**12.915**	**84.83**	**3.768**	**3.5**	**1.172**	**4.05**	**14.65**	**15.2**
	Equiv. Refl.: (-9,1,-3), (-9,8,3)								
**Quartz**	**(9,-8,-2)**	**12.658**	**77.45**	**3.728**	**3**	**2.887**	**8.55**	**36.09**	**37.09**
	Equiv. Refl.: (-1,9,-2), (-1,-8,2), (-8,9,2), (-8,-1,-2)								
**Quartz**	**(9,-1,2)**	**12.658**	**77.45**	**3.727**	**3**	**2.887**	**8.55**	**36.09**	**37.09**
**Quartz**	**(-7,1,-7)**	**12.48**	**74.23**	**3.715**	**2.9**	**3.662**	**10.75**	**45.78**	**47.02**
	Equiv. Refl.: (-7,6,7)								
**Quartz**	**(6,1,7)**	**12.48**	**74.23**	**3.714**	**2.9**	**3.662**	**10.75**	**45.78**	**47.02**
**Quartz**	**(1,6,-7)**	**12.48**	**74.23**	**3.713**	**2.9**	**3.662**	**10.75**	**45.78**	**47.02**
	Equiv. Refl.: (1,8,-2), (1,-7,7), (1,-9,2), (6,-7,-7), (8,1,2), (8,-9,-2)								
**Quartz**	**(-9,1,-2)**	**12.658**	**77.45**	**3.712**	**3**	**2.887**	**8.55**	**36.09**	**37.09**
	Equiv. Refl.: (-9,8,2)								
**Sapph**	**(0,2,-26)**	**12.769**	**79.95**	**3.675**	**3.2**	**2.298**	**7.24**	**28.72**	**29.62**
	Equiv. Refl.: (0,-2,26), (2,0,26), (2,-2,-26), (-2,0,-26), (-2,2,26)								
**Sapph**	**(4,5,11)**	**12.87**	**82.93**	**3.655**	**3.3**	**1.607**	**5.37**	**20.09**	**20.8**
	Equiv. Refl.: (4,-9,-11), (5,4,-11), (5,-9,11), (9,-4,-11), (9,-5,11), (-4,9,11), (-4,-5,-11), (-5,9,-11), (-5,-4,11) (-9,4,11), (-9,5,-11)								
**Quartz**	**(5,1,-8)**	**12.248**	**70.82**	**3.495**	**2.7**	**4.512**	**12.08**	**56.4**	**57.68**
	Equiv. Refl.: (5,-6,8)								
**Quartz**	**(-6,5,-8)**	**12.248**	**70.82**	**3.493**	**2.7**	**4.512**	**12.07**	**56.4**	**57.68**
**Quartz**	**(-6,1,8)**	**12.248**	**70.82**	**3.492**	**2.7**	**4.512**	**12.07**	**56.4**	**57.68**
**Quartz**	**(1,5,8)**	**12.248**	**70.82**	**3.491**	**2.7**	**4.512**	**12.06**	**56.4**	**57.68**
	Equiv. Refl.: (1,-6,-8)								
**Quartz**	**(-5,6,-8)**	**12.248**	**70.82**	**3.489**	**2.7**	**4.512**	**12.08**	**56.4**	**57.68**
	Equiv. Refl.: (-5,-1,8)								
**Quartz**	**(6,-5,8)**	**12.248**	**70.82**	**3.487**	**2.7**	**4.512**	**12.07**	**56.4**	**57.68**
**Quartz**	**(6,-1,-8)**	**12.248**	**70.82**	**3.486**	**2.7**	**4.512**	**12.07**	**56.4**	**57.68**
**Quartz**	**(-1,-5,-8)**	**12.248**	**70.82**	**3.485**	**2.7**	**4.512**	**12.06**	**56.4**	**57.68**
**Quartz**	**(-1,6,8)**	**12.248**	**70.82**	**3.484**	**2.7**	**4.512**	**12.06**	**56.4**	**57.68**
**Quartz**	**(9,-3,4)**	**12.441**	**73.62**	**3.403**	**2.6**	**3.813**	**9.93**	**47.66**	**48.69**
	Equiv. Refl.: (9,-6,-4)								
**Quartz**	**(-3,9,-4)**	**12.441**	**73.62**	**3.401**	**2.6**	**3.813**	**9.93**	**47.66**	**48.68**
	Equiv. Refl.: (-3,-6,4), (-6,9,4), (-6,-3,-4)								
**Quartz**	**(-3,7,8)**	**12.757**	**79.65**	**3.378**	**3**	**2.367**	**7.14**	**29.59**	**30.44**
**Quartz**	**(-3,-4,-8)**	**12.757**	**79.65**	**3.377**	**3**	**2.367**	**7.14**	**29.59**	**30.44**
**Quartz**	**(-4,7,-8)**	**12.757**	**79.65**	**3.376**	**3**	**2.367**	**7.14**	**29.59**	**30.44**
**Quartz**	**(-4,-3,8)**	**12.757**	**79.65**	**3.375**	**3**	**2.367**	**7.14**	**29.59**	**30.44**
**Quartz**	**(7,-3,-8)**	**12.757**	**79.65**	**3.374**	**3**	**2.367**	**7.14**	**29.59**	**30.44**
	Equiv. Refl.: (7,-4,8)								
**Quartz**	**(2,5,8)**	**12.923**	**85.2**	**3.343**	**3**	**1.089**	**3.3**	**13.61**	**14.01**
	Equiv. Refl.: (2,-7,-8)								
**Quartz**	**(-7,5,-8)**	**12.923**	**85.2**	**3.342**	**3**	**1.089**	**3.3**	**13.61**	**14.01**
**Quartz**	**(3,4,8)**	**12.757**	**79.65**	**3.34**	**3**	**2.367**	**7.14**	**29.59**	**30.44**
	Equiv. Refl.: (3,-7,-8), (-7,2,8)								
**Quartz**	**(4,-7,8)**	**12.757**	**79.65**	**3.338**	**3**	**2.367**	**7.14**	**29.59**	**30.44**

Ei =	12.968	keV						ΔEg	ΔEt
Cryst	Refl	EB	ΘB	∫ IR dΘ	Width	Ei cotΘ	ΔE	2m, 50µm	2m, 50µm
	(h,k,l)	[keV]	[°]	[µrad]	[µrad]	[meV/µrad]	[meV]	[meV]	[meV]
**Quartz**	**(4,3,-8)**	**12.757**	**79.65**	**3.337**	**3**	**2.367**	**7.14**	**29.59**	**30.44**
	Equiv. Refl.: (-7,3,8), (-7,4,-8)								
**Quartz**	**(5,2,-8)**	**12.923**	**85.2**	**3.335**	**3**	**1.089**	**3.3**	**13.61**	**14.01**
	Equiv. Refl.: (5,-7,8)								
**Quartz**	**(0,8,4)**	**12.526**	**75**	**3.283**	**2.6**	**3.474**	**9.06**	**43.42**	**44.36**
	Equiv. Refl.: (0,-8,-4)								
**Quartz**	**(8,-8,4)**	**12.526**	**75**	**3.282**	**2.6**	**3.474**	**9.06**	**43.42**	**44.36**
**Quartz**	**(8,0,-4)**	**12.526**	**75**	**3.281**	**2.6**	**3.474**	**9.06**	**43.42**	**44.36**
	Equiv. Refl.: (-8,0,4), (-8,8,-4)								
**Quartz**	**(-9,3,-4)**	**12.441**	**73.62**	**3.258**	**2.6**	**3.813**	**9.93**	**47.66**	**48.69**
	Equiv. Refl.: (-9,6,4)								
**Quartz**	**(3,6,-4)**	**12.441**	**73.62**	**3.257**	**2.6**	**3.813**	**9.93**	**47.66**	**48.68**
	Equiv. Refl.: (3,-9,4)								
**Quartz**	**(6,3,4)**	**12.441**	**73.62**	**3.256**	**2.6**	**3.813**	**9.93**	**47.66**	**48.68**
	Equiv. Refl.: (6,-9,-4)								
**Quartz**	**(9,-1,1)**	**12.501**	**74.58**	**3.21**	**2.5**	**3.577**	**8.89**	**44.71**	**45.58**
	Equiv. Refl.: (9,-8,-1), (-1,9,-1), (-1,-8,1), (-8,9,1), (-8,-1,-1)								
**Sapph**	**(2,7,4)**	**12.461**	**73.93**	**3.208**	**2.6**	**3.735**	**9.77**	**46.69**	**47.7**
	Equiv. Refl.: (2,-9,-4), (7,2,-4), (7,-9,4), (9,-2,-4), (9,-7,4), (-2,9,4), (-2,-7,-4), (-7,9,-4), (-7,-2,4) (-9,2,4), (-9,7,-4)								
**Quartz**	**(-2,-5,-8)**	**12.923**	**85.2**	**3.206**	**3**	**1.089**	**3.3**	**13.61**	**14.01**
**Quartz**	**(7,-5,8)**	**12.923**	**85.2**	**3.205**	**3**	**1.089**	**3.3**	**13.61**	**14.01**
	Equiv. Refl.: (-2,7,8)								
**Quartz**	**(7,-2,-8)**	**12.923**	**85.2**	**3.203**	**3**	**1.089**	**3.3**	**13.61**	**14.01**
**Quartz**	**(-5,7,-8)**	**12.923**	**85.2**	**3.198**	**3**	**1.089**	**3.3**	**13.61**	**14.01**
	Equiv. Refl.: (-5,-2,8)								
**Quartz**	**(10,-4,-1)**	**12.753**	**79.56**	**3.138**	**2.5**	**2.389**	**5.94**	**29.86**	**30.45**
	Equiv. Refl.: (10,-6,1), (-4,10,1), (-4,-6,-1), (-6,10,-1), (-6,-4,1)								
**Quartz**	**(-1,4,10)**	**12.616**	**76.62**	**3.107**	**2.5**	**3.085**	**7.58**	**38.56**	**39.3**
	Equiv. Refl.: (-1,-3,-10), (-3,4,-10)								
**Quartz**	**(-3,-1,10)**	**12.616**	**76.62**	**3.105**	**2.5**	**3.085**	**7.57**	**38.56**	**39.3**
**Sapph**	**(4,5,-10)**	**12.682**	**77.95**	**3.102**	**2.9**	**2.768**	**7.93**	**34.6**	**35.5**
	Equiv. Refl.: (4,-9,10), (5,4,10), (5,-9,-10), (9,-4,10), (9,-5,-10), (-4,9,-10), (-4,-5,10), (-5,9,10), (-5,-4,-10) (-9,4,-10), (-9,5,10), (2,7,3), (2,-9,-3), (4,-1,-10), (4,-3,10), (7,2,-3), (7,-9,3)								
**Quartz**	**(-9,2,3)**	**12.412**	**73.17**	**3.101**	**2.3**	**3.923**	**9.11**	**49.04**	**49.88**
	Equiv. Refl.: (-9,7,-3)								
**Quartz**	**(1,3,10)**	**12.616**	**76.62**	**3.088**	**2.5**	**3.085**	**7.58**	**38.56**	**39.3**
**Quartz**	**(1,-4,-10)**	**12.616**	**76.62**	**3.087**	**2.5**	**3.085**	**7.58**	**38.56**	**39.3**
	Equiv. Refl.: (3,-4,10)								
**Quartz**	**(3,1,-10)**	**12.616**	**76.62**	**3.085**	**2.5**	**3.085**	**7.57**	**38.56**	**39.3**
	Equiv. Refl.: (9,-1,0), (9,-8,0), (-1,9,0), (-1,-8,0), (-4,-5,4), (-5,9,4), (-5,-4,-4), (-8,9,0), (-8,-1,0)								
**Quartz**	**(9,-4,4)**	**12.27**	**71.11**	**3.084**	**2.3**	**4.437**	**10.17**	**55.47**	**56.39**
	Equiv. Refl.: (9,-5,-4), (-4,9,-4)								
**Quartz**	**(-4,1,10)**	**12.616**	**76.62**	**3.082**	**2.5**	**3.085**	**7.57**	**38.56**	**39.3**
	Equiv. Refl.: (-4,3,-10)								
**Quartz**	**(6,2,-6)**	**12.56**	**75.59**	**3.078**	**2.3**	**3.333**	**7.79**	**41.66**	**42.38**
	Equiv. Refl.: (6,-8,6), (-8,6,-6)								
**Quartz**	**(-8,2,6)**	**12.56**	**75.59**	**3.077**	**2.3**	**3.333**	**7.79**	**41.66**	**42.38**
**Quartz**	**(2,6,6)**	**12.56**	**75.59**	**3.075**	**2.3**	**3.333**	**7.79**	**41.66**	**42.38**
	Equiv. Refl.: (2,-8,-6)								

Ei =	12.968	keV						ΔEg	ΔEt
Cryst	Refl	EB	ΘB	∫IR dΘ	Width	Ei cotΘ	ΔE	2m, 50µm	2m, 50µm
	(h,k,l)	[keV]	[°]	[µrad]	[µrad]	[meV/µrad]	[meV]	[meV]	[meV]
**Quartz**	**(4,6,1)**	**12.753**	**79.56**	**3.074**	**2.5**	**2.389**	**5.94**	**29.86**	**30.45**
	Equiv. Refl.: (4,-10,-1), (6,4,-1), (6,-10,1), (-10,4,1), (-10,6,-1)								
**Sapph**	**(2,4,-20)**	**12.43**	**73.43**	**3.071**	**2.8**	**3.858**	**10.63**	**48.23**	**49.38**
	Equiv. Refl.: (2,-6,20), (4,2,20), (4,-6,-20), (6,-2,20), (6,-4,-20), (-2,6,-20), (-2,-4,20), (-4,6,20), (-4,-2,-20) (-6,2,-20), (-6,4,20)								
**Quartz**	**(4,5,-4)**	**12.27**	**71.11**	**3.067**	**2.3**	**4.437**	**10.17**	**55.47**	**56.39**
	Equiv. Refl.: (4,-9,4), (5,4,4), (5,-9,-4)								
**Quartz**	**(-9,4,-4)**	**12.27**	**71.11**	**3.066**	**2.3**	**4.437**	**10.17**	**55.47**	**56.39**
	Equiv. Refl.: (-9,5,4)								
**Quartz**	**(-2,9,3)**	**12.412**	**73.17**	**3.056**	**2.3**	**3.923**	**9.12**	**49.04**	**49.88**
	Equiv. Refl.: (-2,-7,-3), (-7,9,-3), (-7,-2,3)								
**Quartz**	**(9,-2,-3)**	**12.412**	**73.17**	**3.055**	**2.3**	**3.923**	**9.11**	**49.04**	**49.88**
	Equiv. Refl.: (9,-7,3)								
**Quartz**	**(-9,1,-1)**	**12.501**	**74.58**	**3.033**	**2.5**	**3.577**	**8.89**	**44.71**	**45.58**
	Equiv. Refl.: (-9,8,1)								
**Quartz**	**(1,8,-1)**	**12.501**	**74.58**	**3.032**	**2.5**	**3.577**	**8.89**	**44.71**	**45.58**
	Equiv. Refl.: (1,-9,1), (8,1,1), (8,-9,-1)								
**Sapph**	**(1,5,20)**	**12.7**	**78.33**	**3.018**	**2.8**	**2.679**	**7.53**	**33.49**	**34.33**
	Equiv. Refl.: (1,-6,-20), (5,1,-20), (5,-6,20), (6,-1,-20), (6,-5,20), (-1,6,20), (-1,-5,-20), (-5,6,-20), (-5,-1,20) (-6,1,20), (-6,5,-20)								
**Quartz**	**(1,8,0)**	**12.448**	**73.73**	**3.013**	**2.3**	**3.785**	**8.73**	**47.32**	**48.12**
	Equiv. Refl.: (1,-9,0), (8,1,0), (8,-9,0), (-9,1,0), (-9,8,0)								
**Quartz**	**(-6,8,-6)**	**12.56**	**75.59**	**3.005**	**2.3**	**3.333**	**7.79**	**41.66**	**42.38**
**Quartz**	**(8,-2,-6)**	**12.56**	**75.59**	**3.004**	**2.3**	**3.333**	**7.79**	**41.66**	**42.38**
	Equiv. Refl.: (8,-6,6), (-6,-2,6)								
**Quartz**	**(-2,8,6)**	**12.56**	**75.59**	**3.001**	**2.3**	**3.333**	**7.79**	**41.66**	**42.38**
	Equiv. Refl.: (-2,-6,-6)								
**Quartz**	**(7,1,-5)**	**12.405**	**73.06**	**2.77**	**2.1**	**3.95**	**8.25**	**49.37**	**50.06**
	Equiv. Refl.: (7,-8,5)								
**Quartz**	**(-8,7,-5)**	**12.405**	**73.06**	**2.769**	**2.1**	**3.95**	**8.25**	**49.37**	**50.06**
**Quartz**	**(1,7,5)**	**12.405**	**73.06**	**2.768**	**2.1**	**3.95**	**8.25**	**49.37**	**50.06**
	Equiv. Refl.: (1,-8,-5), (-8,1,5)								
**Quartz**	**(0,3,10)**	**12.275**	**71.18**	**2.722**	**2.4**	**4.419**	**10.42**	**55.24**	**56.22**
	Equiv. Refl.: (0,-3,-10), (3,0,-10), (3,-3,10), (-3,0,10), (-3,3,-10)								
**Sapph**	**(2,7,-8)**	**12.893**	**83.82**	**2.588**	**2.7**	**1.404**	**3.77**	**17.56**	**17.96**
	Equiv. Refl.: (2,-9,8), (7,2,8), (7,-9,-8), (9,-2,8), (9,-7,-8), (-2,9,-8), (-2,-7,8), (-7,9,8), (-7,-2,-8) (-9,2,-8), (-9,7,8)								
**Quartz**	**(0,6,8)**	**12.674**	**77.77**	**2.587**	**2.4**	**2.811**	**6.66**	**35.14**	**35.76**
	Equiv. Refl.: (0,-6,-8), (6,0,-8), (6,-6,8), (-6,0,8)								
**Quartz**	**(-6,6,-8)**	**12.674**	**77.77**	**2.586**	**2.4**	**2.811**	**6.66**	**35.14**	**35.76**
**Quartz**	**(5,4,5)**	**12.743**	**79.31**	**2.398**	**2.1**	**2.448**	**5.24**	**30.6**	**31.04**
	Equiv. Refl.: (5,-9,-5), (-9,4,-5), (-9,5,5)								
**Quartz**	**(4,5,-5)**	**12.743**	**79.31**	**2.397**	**2.1**	**2.448**	**5.24**	**30.6**	**31.04**
	Equiv. Refl.: (4,-9,5)								
**Quartz**	**(-7,8,-5)**	**12.405**	**73.06**	**2.396**	**2.1**	**3.95**	**8.25**	**49.37**	**50.06**
	Equiv. Refl.: (-7,-1,5)								
**Quartz**	**(8,-1,-5)**	**12.405**	**73.06**	**2.395**	**2.1**	**3.95**	**8.25**	**49.37**	**50.06**
	Equiv. Refl.: (8,-7,5)								
**Quartz**	**(-1,8,5)**	**12.405**	**73.06**	**2.394**	**2.1**	**3.95**	**8.25**	**49.37**	**50.06**
	Equiv. Refl.: (-1,-7,-5)								

Ei =	12.968	keV						ΔEg	ΔEt
**Cryst**	**Refl** (h,k,l)	**EB** [keV]	**ΘB** [°]	**∫ IR dΘ** [µrad]	**Width** [µrad]	**Ei cotΘ** [meV/µrad]	**ΔE** [meV]	**2m, 50µm** [meV]	**2m, 50µm** [meV]
**Quartz**	**(-8,1,-5)**	**12.405**	**73.06**	**2.361**	**2**	**3.95**	**7.99**	**49.37**	**50.02**
	Equiv. Refl.: (-8,7,5)								
**Quartz**	**(1,7,-5)**	**12.405**	**73.06**	**2.36**	**2**	**3.95**	**7.99**	**49.37**	**50.02**
	Equiv. Refl.: (1,-8,5)								
**Quartz**	**(7,1,5)**	**12.405**	**73.06**	**2.359**	**2**	**3.95**	**7.99**	**49.37**	**50.02**
	Equiv. Refl.: (7,-8,-5)								
**Quartz**	**(8,-1,5)**	**12.405**	**73.06**	**2.313**	**2**	**3.95**	**7.99**	**49.37**	**50.02**
	Equiv. Refl.: (8,-7,-5)								
**Quartz**	**(-1,8,-5)**	**12.405**	**73.06**	**2.312**	**2**	**3.95**	**7.99**	**49.37**	**50.02**
	Equiv. Refl.: (-1,-7,5)								
**Quartz**	**(-7,8,5)**	**12.405**	**73.06**	**2.311**	**2**	**3.95**	**7.99**	**49.37**	**50.02**
	Equiv. Refl.: (-7,-1,-5)								
**Quartz**	**(4,-2,10)**	**12.532**	**75.09**	**2.303**	**2**	**3.452**	**6.97**	**43.16**	**43.71**
	Equiv. Refl.: (4,-2,-10)								
**Quartz**	**(-2,4,-10)**	**12.532**	**75.09**	**2.301**	**2**	**3.452**	**6.97**	**43.16**	**43.71**
	Equiv. Refl.: (-2,-2,10)								
**Quartz**	**(-2,4,10)**	**12.532**	**75.09**	**2.299**	**2**	**3.452**	**6.96**	**43.16**	**43.71**
	Equiv. Refl.: (-2,-2,-10), (-4,2,10)								
**Quartz**	**(9,-4,5)**	**12.743**	**79.31**	**2.298**	**2.1**	**2.448**	**5.24**	**30.6**	**31.04**
	Equiv. Refl.: (9,-5,-5), (-4,2,-10), (-4,9,-5), (-5,9,5), (-5,-4,-5)								
**Quartz**	**(-4,-5,5)**	**12.743**	**79.31**	**2.297**	**2.1**	**2.448**	**5.24**	**30.6**	**31.04**
**Quartz**	**(2,2,-10)**	**12.532**	**75.09**	**2.296**	**2**	**3.452**	**6.97**	**43.16**	**43.71**
	Equiv. Refl.: (2,-4,10)								
**Quartz**	**(2,-4,-10)**	**12.532**	**75.09**	**2.295**	**2**	**3.452**	**6.96**	**43.16**	**43.71**
**Quartz**	**(2,2,10)**	**12.532**	**75.09**	**2.294**	**2**	**3.452**	**6.96**	**43.16**	**43.71**
	Equiv. Refl.: (3,6,4), (3,-9,-4), (6,3,-4), (-9,3,4), (-9,6,-4)								
**Quartz**	**(6,-9,4)**	**12.441**	**73.62**	**2.293**	**2**	**3.813**	**7.55**	**47.66**	**48.26**
	Equiv. Refl.: (9,-3,-4), (9,-6,4), (-3,9,4), (-3,-6,-4), (-6,9,-4), (-6,-3,4)								
**Quartz**	**(0,2,-11)**	**12.949**	**86.92**	**1.987**	**2.7**	**0.697**	**1.85**	**8.71**	**8.9**
	Equiv. Refl.: (0,-2,11)								
**LiNbO3**	**(2,7,13)**	**12.78**	**80.24**	**1.974**	**3**	**2.231**	**6.8**	**27.88**	**28.7**
	Equiv. Refl.: (2,-9,-13), (7,2,-13), (7,-9,13), (9,-2,-13), (9,-7,13), (-2,9,13), (-2,-7,-13), (-7,9,-13), (-7,-2,13) (-9,2,13), (-9,7,-13)								
**Quartz**	**(-2,2,11)**	**12.949**	**86.92**	**1.965**	**2.6**	**0.697**	**1.83**	**8.71**	**8.9**
**Quartz**	**(2,-2,-11)**	**12.949**	**86.92**	**1.964**	**2.6**	**0.697**	**1.83**	**8.71**	**8.9**
**Quartz**	**(2,0,11)**	**12.949**	**86.92**	**1.963**	**2.6**	**0.697**	**1.83**	**8.71**	**8.9**
	Equiv. Refl.: (-2,0,-11)								
**Quartz**	**(8,1,-2)**	**12.658**	**77.45**	**1.923**	**2**	**2.887**	**5.63**	**36.09**	**36.53**
**Quartz**	**(1,8,2)**	**12.658**	**77.45**	**1.922**	**2**	**2.887**	**5.63**	**36.09**	**36.53**
	Equiv. Refl.: (1,-9,-2), (8,-9,2), (-9,1,2), (-9,8,-2)								
**Sapph**	**(0,5,22)**	**12.917**	**84.9**	**1.842**	**2.4**	**1.157**	**2.74**	**14.46**	**14.72**
	Equiv. Refl.: (0,-5,-22), (5,0,-22), (5,-5,22), (-5,0,22), (-5,5,-22)								
**Quartz**	**(-1,9,2)**	**12.658**	**77.45**	**1.735**	**2**	**2.887**	**5.63**	**36.09**	**36.53**
	Equiv. Refl.: (-1,-8,-2), (-8,9,-2), (-8,-1,2)								
**Quartz**	**(9,-1,-2)**	**12.658**	**77.45**	**1.734**	**2**	**2.887**	**5.63**	**36.09**	**36.52**
	Equiv. Refl.: (9,-8,2)								
**Quartz**	**(0,4,10)**	**12.866**	**82.8**	**1.678**	**2**	**1.637**	**3.19**	**20.47**	**20.72**
	Equiv. Refl.: (0,-4,-10)								
**Quartz**	**(4,0,-10)**	**12.866**	**82.8**	**1.672**	**1.9**	**1.637**	**3.18**	**20.47**	**20.71**
	Equiv. Refl.: (4,-4,10), (-4,0,10), (-4,4,-10)								

Ei =	12.968	keV						ΔEg	ΔEt
**Cryst**	**Refl**	**EB**	**ΘB**	**∫ IR dΘ**	**Width**	**Ei cotΘ**	**ΔE**	**2m, 50µm**	**2m, 50µm**
	**(h,k,l)**	**[keV]**	**[°]**	**[µrad]**	**[µrad]**	**[meV/µrad]**	**[meV]**	**[meV]**	**[meV]**
**Sapph**	**(4,4,15)**	**12.645**	**77.18**	**1.61**	**1.8**	**2.95**	**5.32**	**36.88**	**37.26**
	Equiv. Refl.: (4,4,-15), (4,-8,15), (4,-8,-15), (8,-4,15), (8,-4,-15), (-4,8,15), (-4,8,-15), (-4,-4,15), (-4,-4,-15) (-8,4,15), (-8,4,-15)								
**Sapph**	**(2,6,-13)**	**12.498**	**74.52**	**1.521**	**1.7**	**3.591**	**6.02**	**44.89**	**45.29**
	Equiv. Refl.: (2,-8,13), (6,2,13), (6,-8,-13), (8,-2,13), (8,-6,-13), (-2,8,-13), (-2,-6,13), (-6,8,13), (-6,-2,-13) (-8,2,-13), (-8,6,13)								
**Quartz**	**(0,6,-8)**	**12.674**	**77.77**	**1.485**	**1.6**	**2.811**	**4.58**	**35.14**	**35.43**
	Equiv. Refl.: (0,-6,8), (6,0,8), (6,-6,-8), (-6,0,-8)								
**Quartz**	**(-6,6,8)**	**12.674**	**77.77**	**1.484**	**1.6**	**2.811**	**4.58**	**35.14**	**35.43**
**LiNbO3**	**(1,6,19)**	**12.463**	**73.96**	**1.435**	**2.1**	**3.729**	**7.91**	**46.61**	**47.28**
	Equiv. Refl.: (1,-7,-19), (6,1,-19), (6,-7,19), (7,-1,-19), (7,-6,19), (-1,7,19), (-1,-6,-19), (-6,7,-19), (-6,-1,19) (-7,1,19), (-7,6,-19)								
**LiNbO3**	**(2,4,-23)**	**12.646**	**77.2**	**1.373**	**2.2**	**2.945**	**6.45**	**36.82**	**37.38**
	Equiv. Refl.: (2,-6,23), (4,2,23), (4,-6,-23), (6,-2,23), (6,-4,-23), (-2,6,-23), (-2,-4,23), (-4,6,23), (-4,-2,-23) (-6,2,-23), (-6,4,23)								
**Quartz**	**(0,1,-11)**	**12.701**	**78.35**	**1.37**	**1.5**	**2.673**	**4.09**	**33.41**	**33.66**
	Equiv. Refl.: (0,-1,11)								
**Quartz**	**(1,-1,-11)**	**12.701**	**78.35**	**1.369**	**1.5**	**2.673**	**4.08**	**33.41**	**33.66**
**Quartz**	**(1,0,11)**	**12.701**	**78.35**	**1.368**	**1.5**	**2.673**	**4.08**	**33.41**	**33.66**
	Equiv. Refl.: (-1,1,11)								
**Quartz**	**(-1,0,-11)**	**12.701**	**78.35**	**1.367**	**1.5**	**2.673**	**4.08**	**33.41**	**33.66**
**LiNbO3**	**(1,1,27)**	**12.312**	**71.69**	**1.343**	**2**	**4.291**	**8.4**	**53.63**	**54.29**
	Equiv. Refl.: (1,1,-27), (1,-2,27), (1,-2,-27), (2,-1,27), (2,-1,-27), (-1,2,27), (-1,2,-27), (-1,-1,27), (-1,-1,-27) (-2,1,27), (-2,1,-27)								
**Sapph**	**(4,5,-7)**	**12.216**	**70.39**	**1.274**	**1.4**	**4.621**	**6.47**	**57.76**	**58.12**
	Equiv. Refl.: (4,-9,7), (5,4,7), (5,-9,-7), (9,-4,7), (9,-5,-7), (-4,9,-7), (-4,-5,7), (-5,9,7), (-5,-4,-7) (-9,4,-7), (-9,5,7)								
**Sapph**	**(0,1,26)**	**12.5**	**74.57**	**1.255**	**1.4**	**3.58**	**5.09**	**44.75**	**45.04**
	Equiv. Refl.: (0,-1,-26), (1,0,-26), (1,-1,26), (-1,0,26), (-1,1,-26)								
**Quartz**	**(8,-6,-6)**	**12.56**	**75.59**	**1.254**	**1.3**	**3.333**	**4.35**	**41.66**	**41.89**
	Equiv. Refl.: (-6,8,6), (-6,-2,-6)								
**Quartz**	**(8,-2,6)**	**12.56**	**75.59**	**1.253**	**1.3**	**3.333**	**4.35**	**41.66**	**41.89**
	Equiv. Refl.: (-2,8,-6), (-2,-6,6)								
**Quartz**	**(10,-4,0)**	**12.702**	**78.37**	**1.248**	**1.4**	**2.669**	**3.76**	**33.37**	**33.58**
	Equiv. Refl.: (10,-6,0), (-4,10,0), (-4,-6,0), (-6,10,0), (-6,-4,0)								
**Quartz**	**(4,6,0)**	**12.702**	**78.37**	**1.242**	**1.4**	**2.669**	**3.76**	**33.37**	**33.58**
	Equiv. Refl.: (4,-10,0), (6,4,0), (6,-10,0), (-10,4,0), (-10,6,0)								
**LiNbO3**	**(2,7,-11)**	**12.399**	**72.97**	**1.236**	**1.9**	**3.973**	**7.47**	**49.66**	**50.22**
	Equiv. Refl.: (2,-9,11), (7,2,11), (7,-9,-11), (9,-2,11), (9,-7,-11), (-2,9,-11), (-2,-7,11), (-7,9,11), (-7,-2,-11) (-9,2,-11), (-9,7,11)								
**Sapph**	**(2,7,7)**	**12.759**	**79.71**	**1.228**	**1.5**	**2.354**	**3.51**	**29.43**	**29.64**
	Equiv. Refl.: (2,-9,-7), (7,2,-7), (7,-9,7), (9,-2,-7), (9,-7,7), (-2,9,7), (-2,-7,-7), (-7,9,-7), (-7,-2,7) (-9,2,7), (-9,7,-7)								
**Quartz**	**(4,5,4)**	**12.27**	**71.11**	**1.21**	**1.3**	**4.437**	**5.72**	**55.47**	**55.76**
	Equiv. Refl.: (4,-9,-4), (-9,4,4), (-9,5,-4)								
**Quartz**	**(5,4,-4)**	**12.27**	**71.11**	**1.209**	**1.3**	**4.437**	**5.72**	**55.47**	**55.76**
	Equiv. Refl.: (5,-9,4)								
**Quartz**	**(-4,-5,-4)**	**12.27**	**71.11**	**1.192**	**1.3**	**4.437**	**5.72**	**55.47**	**55.76**
**Quartz**	**(9,-4,-4)**	**12.27**	**71.11**	**1.191**	**1.3**	**4.437**	**5.72**	**55.47**	**55.76**
	Equiv. Refl.: (9,-5,4), (-4,9,4), (-5,9,-4), (-5,-4,4)								

Ei = Cryst	12.968 Refl (h,k,l)	keV EB [keV]	ΘB [°]	∫ IR dΘ [μrad]	Width [μrad]	Ei cotΘ [meV/μrad]	ΔE [meV]	ΔEg 2m, 50μm [meV]	ΔEt 2m, 50μm [meV]
**Sapph**	**(1,5,-19)**	**12.345**	**72.17**	**1.185**	**1.3**	**4.171**	**5.54**	**52.14**	**52.44**
	Equiv. Refl.: (1,-6,19), (5,1,19), (5,-6,-19), (6,-1,19), (6,-5,-19), (-1,6,-19), (-1,-5,19), (-5,6,19), (-5,-1,-19) (-6,1,-19), (-6,5,19)								
**Quartz**	**(-8,4,6)**	**12.217**	**70.41**	**1.16**	**1.2**	**4.616**	**5.66**	**57.7**	**57.98**
	Equiv. Refl.: (-8,4,-6)								
**Quartz**	**(4,4,6)**	**12.217**	**70.41**	**1.159**	**1.2**	**4.616**	**5.65**	**57.7**	**57.98**
	Equiv. Refl.: (4,4,-6), (4,-8,6), (4,-8,-6)								
**Quartz**	**(-2,9,-4)**	**12.778**	**80.18**	**1.137**	**1.3**	**2.244**	**2.89**	**28.05**	**28.2**
**Quartz**	**(-2,-7,4)**	**12.778**	**80.18**	**1.136**	**1.3**	**2.244**	**2.89**	**28.05**	**28.2**
	Equiv. Refl.: (-7,9,4), (-7,-2,-4)								
**Quartz**	**(9,-2,4)**	**12.778**	**80.18**	**1.135**	**1.3**	**2.244**	**2.88**	**28.05**	**28.2**
	Equiv. Refl.: (9,-7,-4)								
**LiNbO3**	**(4,6,7)**	**12.52**	**74.89**	**1.126**	**1.8**	**3.502**	**6.39**	**43.77**	**44.23**
	Equiv. Refl.: (4,-10,-7), (6,4,-7), (6,-10,7), (10,-4,-7), (10,-6,7), (-4,10,7), (-4,-6,-7), (-6,10,-7), (-6,-4,7) (-10,4,7), (-10,6,-7)								
**Sapph**	**(1,6,16)**	**12.476**	**74.16**	**1.117**	**1.3**	**3.679**	**4.72**	**45.99**	**46.23**
	Equiv. Refl.: (1,-7,-16), (6,1,-16), (6,-7,16), (7,-1,-16), (7,-6,16), (-1,7,16), (-1,-6,-16), (-6,7,-16), (-6,-1,16) (-7,1,16), (-7,6,-16)								
**Quartz**	**(8,-4,6)**	**12.217**	**70.41**	**1.116**	**1.2**	**4.616**	**5.66**	**57.7**	**57.98**
**Quartz**	**(8,-4,-6)**	**12.217**	**70.41**	**1.115**	**1.2**	**4.616**	**5.66**	**57.7**	**57.98**
	Equiv. Refl.: (-4,8,6), (-4,8,-6), (-4,-4,6), (-4,-4,-6)								
**Quartz**	**(6,2,6)**	**12.56**	**75.59**	**1.107**	**1.3**	**3.333**	**4.36**	**41.66**	**41.89**
**Quartz**	**(6,-8,-6)**	**12.56**	**75.59**	**1.106**	**1.3**	**3.333**	**4.36**	**41.66**	**41.89**
	Equiv. Refl.: (-8,6,6)								
**Quartz**	**(2,6,-6)**	**12.56**	**75.59**	**1.105**	**1.3**	**3.333**	**4.35**	**41.66**	**41.89**
	Equiv. Refl.: (2,-8,6), (-8,2,-6)								
**Sapph**	**(2,3,23)**	**12.787**	**80.42**	**1.034**	**1.3**	**2.188**	**2.89**	**27.36**	**27.51**
	Equiv. Refl.: (2,-5,-23), (3,2,-23), (3,-5,23), (5,-2,-23), (5,-3,23), (-2,5,23), (-2,-3,-23), (-3,5,-23), (-3,-2,23) (-5,2,23), (-5,3,-23)								
**Quartz**	**(10,-5,1)**	**12.67**	**77.69**	**1.028**	**1.2**	**2.83**	**3.36**	**35.37**	**35.53**
	Equiv. Refl.: (-5,10,1), (-5,10,-1), (-5,-5,1), (-5,-5,-1)								
**Quartz**	**(10,-5,-1)**	**12.67**	**77.69**	**1.027**	**1.2**	**2.83**	**3.36**	**35.37**	**35.53**
**Quartz**	**(2,-9,4)**	**12.778**	**80.18**	**1.025**	**1.3**	**2.244**	**2.89**	**28.05**	**28.2**
**Quartz**	**(2,7,-4)**	**12.778**	**80.18**	**1.024**	**1.3**	**2.244**	**2.89**	**28.05**	**28.2**
	Equiv. Refl.: (5,5,1), (5,5,-1), (5,-10,1), (5,-10,-1), (7,2,4), (7,-9,-4), (-10,5,1), (-10,5,-1)								
**Quartz**	**(-9,2,-4)**	**12.778**	**80.18**	**1.023**	**1.3**	**2.244**	**2.88**	**28.05**	**28.2**
	Equiv. Refl.: (-9,7,4)								
**LiNbO3**	**(1,5,23)**	**12.873**	**83.07**	**0.985**	**2.2**	**1.577**	**3.48**	**19.71**	**20.01**
	Equiv. Refl.: (1,-6,-23), (4,6,-5), (4,-10,5), (5,1,-23), (5,-6,23), (6,4,5), (6,-1,-23), (6,-5,23), (6,-10,-5) (10,-4,5), (10,-6,-5), (-1,6,23), (-1,-5,-23), (-4,10,-5), (-4,-6,5), (-5,6,-23), (-5,-1,23), (-6,1,23) (-6,5,-23), (-6,10,5), (-6,-4,-5), (-10,4,-5), (-10,6,5)								
**Quartz**	**(6,1,-7)**	**12.48**	**74.23**	**0.943**	**1**	**3.662**	**3.81**	**45.78**	**45.94**
**Quartz**	**(6,-7,7)**	**12.48**	**74.23**	**0.942**	**1**	**3.662**	**3.81**	**45.78**	**45.94**
**Quartz**	**(1,-7,-7)**	**12.48**	**74.23**	**0.941**	**1**	**3.662**	**3.81**	**45.78**	**45.94**
**Quartz**	**(1,6,7)**	**12.48**	**74.23**	**0.94**	**1**	**3.662**	**3.8**	**45.78**	**45.94**
**Quartz**	**(-7,1,7)**	**12.48**	**74.23**	**0.939**	**1**	**3.662**	**3.8**	**45.78**	**45.94**
	Equiv. Refl.: (-7,6,-7)								
**Quartz**	**(3,1,10)**	**12.616**	**76.62**	**0.925**	**1**	**3.085**	**3.04**	**38.56**	**38.68**
	Equiv. Refl.: (3,-4,-10)								

Ei =	12.968	keV						ΔEg	ΔEt
Cryst	Refl	EB	ΘB	∫ IR dΘ	Width	Ei cotΘ	ΔE	2m, 50µm	2m, 50µm
	(h,k,l)	[keV]	[°]	[µrad]	[µrad]	[meV/µrad]	[meV]	[meV]	[meV]
**Quartz**	**(1,3,-10)**	**12.616**	**76.62**	**0.922**	**1**	**3.085**	**3.03**	**38.56**	**38.68**
	Equiv. Refl.: (1,-4,10), (-4,1,-10)								
**Quartz**	**(-4,3,10)**	**12.616**	**76.62**	**0.921**	**1**	**3.085**	**3.03**	**38.56**	**38.68**
**Sapph**	**(2,7,-5)**	**12.543**	**75.3**	**0.909**	**1.1**	**3.403**	**3.73**	**42.54**	**42.7**
	Equiv. Refl.: (2,-9,5), (7,2,5), (7,-9,-5), (9,-2,5), (9,-7,-5), (-2,9,-5), (-2,-7,5), (-7,9,5), (-7,-2,-5) (-9,2,-5), (-9,7,5)								
**LiNbO3**	**(3,6,15)**	**12.915**	**84.82**	**0.896**	**2.4**	**1.175**	**2.78**	**14.68**	**14.94**
	Equiv. Refl.: (3,6,-15), (3,-9,15), (3,-9,-15), (6,3,15), (6,3,-15), (6,-9,15), (6,-9,-15), (9,-3,15), (9,-3,-15) (9,-6,15), (9,-6,-15), (-3,9,15), (-3,9,-15), (-3,-6,15), (-3,-6,-15), (-6,9,15), (-6,9,-15), (-6,-3,15) (-6,-3,-15), (-9,3,15), (-9,3,-15), (-9,6,15), (-9,6,-15)								
**Quartz**	**(-6,7,-7)**	**12.48**	**74.23**	**0.889**	**1**	**3.662**	**3.81**	**45.78**	**45.94**
	Equiv. Refl.: (-6,-1,7)								
**Quartz**	**(-1,7,7)**	**12.48**	**74.23**	**0.887**	**1**	**3.662**	**3.81**	**45.78**	**45.94**
	Equiv. Refl.: (-1,-6,-7)								
**Quartz**	**(7,-1,-7)**	**12.48**	**74.23**	**0.886**	**1**	**3.662**	**3.8**	**45.78**	**45.94**
	Equiv. Refl.: (7,-6,7)								
**Quartz**	**(5,3,6)**	**12.304**	**71.58**	**0.883**	**0.9**	**4.319**	**4.02**	**53.98**	**54.13**
	Equiv. Refl.: (5,-8,-6), (-8,3,-6), (-8,5,6)								
**Quartz**	**(3,5,-6)**	**12.304**	**71.58**	**0.882**	**0.9**	**4.319**	**4.02**	**53.98**	**54.13**
**Quartz**	**(3,-8,6)**	**12.304**	**71.58**	**0.881**	**0.9**	**4.319**	**4.02**	**53.98**	**54.13**
**Quartz**	**(1,4,9)**	**12.294**	**71.45**	**0.846**	**0.9**	**4.352**	**4.1**	**54.4**	**54.55**
	Equiv. Refl.: (1,-5,-9)								
**Quartz**	**(4,1,-9)**	**12.294**	**71.45**	**0.845**	**0.9**	**4.352**	**4.09**	**54.4**	**54.55**
	Equiv. Refl.: (4,-5,9), (-5,1,9), (-5,4,-9)								
**Quartz**	**(-1,-4,-9)**	**12.294**	**71.45**	**0.823**	**0.9**	**4.352**	**4.1**	**54.4**	**54.55**
**Quartz**	**(5,-1,-9)**	**12.294**	**71.45**	**0.822**	**0.9**	**4.352**	**4.09**	**54.4**	**54.55**
	Equiv. Refl.: (5,-4,9), (-1,5,9), (-4,-1,9)								
**Quartz**	**(-4,5,-9)**	**12.294**	**71.45**	**0.821**	**0.9**	**4.352**	**4.09**	**54.4**	**54.55**
**Sapph**	**(2,7,1)**	**12.323**	**71.86**	**0.78**	**0.9**	**4.249**	**3.97**	**53.11**	**53.26**
	Equiv. Refl.: (2,-9,-1), (7,2,-1), (7,-9,1), (9,-2,-1), (9,-7,1), (-2,9,1), (-2,-7,-1), (-7,9,-1), (-7,-2,1) (-9,2,1), (-9,7,-1)								
**Quartz**	**(8,-3,6)**	**12.304**	**71.58**	**0.769**	**0.9**	**4.319**	**4.02**	**53.98**	**54.13**
	Equiv. Refl.: (8,-5,-6), (-5,8,6), (-5,-3,-6)								
**Quartz**	**(-3,8,-6)**	**12.304**	**71.58**	**0.768**	**0.9**	**4.319**	**4.02**	**53.98**	**54.13**
	Equiv. Refl.: (-3,-5,6)								
**Quartz**	**(-3,4,10)**	**12.616**	**76.62**	**0.748**	**1**	**3.085**	**3.04**	**38.56**	**38.68**
**Quartz**	**(-3,-1,-10)**	**12.616**	**76.62**	**0.747**	**1**	**3.085**	**3.04**	**38.56**	**38.68**
**Quartz**	**(4,-1,10)**	**12.616**	**76.62**	**0.745**	**1**	**3.085**	**3.03**	**38.56**	**38.68**
	Equiv. Refl.: (-1,-3,10)								
**Quartz**	**(4,-3,-10)**	**12.616**	**76.62**	**0.744**	**1**	**3.085**	**3.03**	**38.56**	**38.68**
	Equiv. Refl.: (-1,4,-10)								
**Sapph**	**(3,6,6)**	**12.28**	**71.25**	**0.736**	**0.9**	**4.403**	**3.9**	**55.04**	**55.17**
	Equiv. Refl.: (3,6,-6), (3,-9,6), (3,-9,-6), (6,3,6), (6,3,-6), (6,-9,6), (6,-9,-6), (9,-3,6), (9,-3,-6) (9,-6,6), (9,-6,-6), (-3,9,6), (-3,9,-6), (-3,-6,6), (-3,-6,-6), (-6,9,6), (-6,9,-6), (-6,-3,6) (-6,-3,-6), (-9,3,6), (-9,3,-6), (-9,6,6), (-9,6,-6)								
**Sapph**	**(1,6,-17)**	**12.773**	**80.06**	**0.675**	**0.9**	**2.273**	**2.15**	**28.41**	**28.49**
	Equiv. Refl.: (1,-7,17), (6,1,17), (6,-7,-17), (7,-1,17), (7,-6,-17), (-1,7,-17), (-1,-6,17), (-6,7,17), (-6,-1,-17) (-7,1,-17), (-7,6,17)								
**Sapph**	**(3,5,13)**	**12.223**	**70.48**	**0.63**	**0.8**	**4.596**	**3.57**	**57.45**	**57.57**
	Equiv. Refl.: (3,-8,-13), (5,3,-13), (5,-8,13), (8,-3,-13), (8,-5,13), (-3,8,13), (-3,-5,-13), (-5,8,-13), (-5,-3,13) (-8,3,13), (-8,5,-13)								

Ei =	12.968	keV						ΔEg	ΔEt
**Cryst**	**Refl**	**EB**	**ΘB**	**∫ IR dΘ**	**Width**	**Ei cotΘ**	**ΔE**	**2m, 50µm**	**2m, 50µm**
	**(h,k,l)**	**[keV]**	**[°]**	**[µrad]**	**[µrad]**	**[meV/µrad]**	**[meV]**	**[meV]**	**[meV]**
**LiNbO3**	**(3,7,-7)**	**12.749**	**79.46**	**0.622**	**1.4**	**2.413**	**3.44**	**30.16**	**30.36**
	Equiv. Refl.: (3,-10,7), (7,3,7), (7,-10,-7), (10,-3,7), (10,-7,-7), (-3,10,-7), (-3,-7,7), (-7,10,7), (-7,-3,-7) (-10,3,-7), (-10,7,7)								
**LiNbO3**	**(1,3,25)**	**12.252**	**70.87**	**0.618**	**1.1**	**4.497**	**5.16**	**56.22**	**56.45**
	Equiv. Refl.: (1,-4,-25), (3,1,-25), (3,-4,25), (4,-1,-25), (4,-3,25), (-1,4,25), (-1,-3,-25), (-3,4,-25), (-3,-1,25) (-4,1,25), (-4,3,-25)								
**Sapph**	**(1,2,-25)**	**12.579**	**75.92**	**0.578**	**0.8**	**3.251**	**2.53**	**40.64**	**40.72**
	Equiv. Refl.: (1,-3,25), (2,1,25), (2,-3,-25), (3,-1,25), (3,-2,-25), (-1,3,-25), (-1,-2,25), (-2,3,25), (-2,-1,-25) (-3,1,-25), (-3,2,25)								
**Sapph**	**(2,5,18)**	**12.731**	**79.03**	**0.564**	**0.8**	**2.514**	**2.03**	**31.43**	**31.49**
	Equiv. Refl.: (2,5,-18), (2,-7,18), (2,-7,-18), (5,2,18), (5,2,-18), (5,-7,18), (5,-7,-18), (7,-2,18), (7,-2,-18) (7,-5,18), (7,-5,-18), (-2,7,18), (-2,7,-18), (-2,-5,18), (-2,-5,-18), (-5,7,18), (-5,7,-18), (-5,-2,18) (-5,-2,-18), (-7,2,18), (-7,2,-18), (-7,5,18), (-7,5,-18)								
**Sapph**	**(2,3,-22)**	**12.38**	**72.68**	**0.545**	**0.7**	**4.045**	**2.87**	**50.56**	**50.64**
	Equiv. Refl.: (2,7,-2), (2,-5,22), (2,-9,2), (3,2,22), (3,-5,-22), (5,-2,22), (5,-3,-22), (7,2,2), (7,-9,-2) (9,-2,2), (9,-7,-2), (-2,5,-22), (-2,9,-2), (-2,-3,22), (-2,-7,2), (-3,5,22), (-3,-2,-22), (-5,2,-22) (-5,3,22), (-7,9,2), (-7,-2,-2), (-9,2,-2), (-9,7,2)								
**Sapph**	**(0,7,14)**	**12.472**	**74.1**	**0.544**	**0.7**	**3.693**	**2.65**	**46.17**	**46.24**
	Equiv. Refl.: (0,-7,-14), (7,0,-14), (7,-7,14), (-7,0,14), (-7,7,-14)								
**LiNbO3**	**(4,5,-13)**	**12.318**	**71.78**	**0.536**	**1.1**	**4.268**	**4.55**	**53.35**	**53.54**
	Equiv. Refl.: (4,-9,13), (5,4,13), (5,-9,-13), (9,-4,13), (9,-5,-13), (-4,9,-13), (-4,-5,13), (-5,9,13), (-5,-4,-13) (-9,4,-13), (-9,5,13)								
**LiNbO3**	**(2,8,3)**	**12.814**	**81.17**	**0.502**	**1.4**	**2.014**	**2.73**	**25.17**	**25.32**
	Equiv. Refl.: (2,8,-3), (2,-10,3), (2,-10,-3), (8,2,3), (8,2,-3), (8,-10,3), (8,-10,-3), (10,-2,3), (10,-2,-3) (10,-8,3), (10,-8,-3), (-2,10,3), (-2,10,-3), (-2,-8,3), (-2,-8,-3), (-8,10,3), (-8,10,-3), (-8,2,3) (-8,-2,-3), (-10,2,3), (-10,2,-3), (-10,8,3), (-10,8,-3)								
**LiNbO3**	**(3,7,5)**	**12.56**	**75.58**	**0.477**	**1.1**	**3.334**	**3.6**	**41.68**	**41.83**
	Equiv. Refl.: (3,-10,-5), (7,3,-5), (7,-10,5), (10,-3,-5), (10,-7,5), (-3,10,5), (-3,-7,-5), (-7,10,-5), (-7,-3,5) (-10,3,5), (-10,7,-5)								
**Quartz**	**(0,3,-10)**	**12.275**	**71.18**	**0.432**	**0.5**	**4.419**	**2.42**	**55.24**	**55.3**
	Equiv. Refl.: (0,-3,10)								
**Quartz**	**(3,0,10)**	**12.275**	**71.18**	**0.428**	**0.5**	**4.419**	**2.41**	**55.24**	**55.29**
	Equiv. Refl.: (3,-3,-10), (-3,0,-10), (-3,3,10)								
**LiNbO3**	**(2,5,21)**	**12.79**	**80.5**	**0.423**	**1.2**	**2.17**	**2.6**	**27.12**	**27.24**
	Equiv. Refl.: (2,5,-21), (2,-7,21), (2,-7,-21), (5,2,21), (5,2,-21), (5,-7,21), (5,-7,-21), (7,-2,21), (7,-2,-21) (7,-5,21), (7,-5,-21), (-2,7,21), (-2,7,-21), (-2,-5,21), (-2,-5,-21), (-5,7,21), (-5,7,-21), (-5,-2,21) (-5,-2,-21), (-7,2,21), (-7,2,-21), (-7,5,21), (-7,5,-21)								
**LiNbO3**	**(3,7,-1)**	**12.367**	**72.49**	**0.413**	**0.9**	**4.092**	**3.78**	**51.15**	**51.29**
	Equiv. Refl.: (3,-10,1), (7,3,1), (7,-10,-1), (10,-3,1), (10,-7,-1), (-3,10,-1), (-3,-7,1), (-7,10,1), (-7,-3,-1) (-10,3,-1), (-10,7,1)								
**LiNbO3**	**(3,5,19)**	**12.92**	**85.07**	**0.406**	**1.6**	**1.118**	**1.77**	**13.98**	**14.09**
	Equiv. Refl.: (3,-8,-19), (5,3,-19), (5,-8,19), (8,-3,-19), (8,-5,19), (-3,8,19), (-3,-5,-19), (-5,8,-19), (-5,-3,19) (-8,3,19), (-8,5,-19)								
**Quartz**	**(1,8,1)**	**12.501**	**74.58**	**0.385**	**0.5**	**3.577**	**1.65**	**44.71**	**44.74**
	Equiv. Refl.: (1,-9,-1), (8,1,-1), (8,-9,1), (-9,1,1), (-9,8,-1)								
**Quartz**	**(1,4,-9)**	**12.294**	**71.45**	**0.352**	**0.5**	**4.352**	**2.02**	**54.4**	**54.44**
	Equiv. Refl.: (-5,1,-9), (-5,4,9)								
**Quartz**	**(1,-5,9)**	**12.294**	**71.45**	**0.351**	**0.5**	**4.352**	**2.02**	**54.4**	**54.44**
**Quartz**	**(4,1,9)**	**12.294**	**71.45**	**0.35**	**0.5**	**4.352**	**2.02**	**54.4**	**54.44**
	Equiv. Refl.: (4,-5,-9)								

Ei = Cryst	12.968 Refl (h,k,l)	keV EB [keV]	ΘB [°]	∫IR dΘ [µrad]	Width [µrad]	Ei cotΘ [meV/µrad]	ΔE [meV]	ΔEg 2m, 50µm [meV]	ΔEt 2m, 50µm [meV]	Equiv. Refl.
**Quartz**	**(-5,6,8)**	**12.248**	**70.82**	**0.344**	**0.4**	**4.512**	**1.95**	**56.4**	**56.43**	(-5,-1,-8)
**Quartz**	**(5,-1,9)**	**12.294**	**71.45**	**0.343**	**0.5**	**4.352**	**2.02**	**54.4**	**54.44**	(5,-4,-9), (-1,-4,9)
**Quartz**	**(-1,5,-9)**	**12.294**	**71.45**	**0.342**	**0.5**	**4.352**	**2.02**	**54.4**	**54.44**	(-1,6,-8), (-1,-5,8)
**Quartz**	**(6,-1,8)**	**12.248**	**70.82**	**0.341**	**0.4**	**4.512**	**1.94**	**56.4**	**56.43**	(6,-5,-8), (-4,5,9), (-4,-1,-9)
**Sapph**	**(4,5,8)**	**12.355**	**72.31**	**0.34**	**0.5**	**4.136**	**2.06**	**51.7**	**51.75**	(4,-9,-8), (5,4,-8), (5,-9,8), (9,-4,-8), (9,-5,8), (-4,9,8), (-4,-5,-8), (-5,9,-8), (-5,-4,8) (-9,4,8), (-9,5,-8)
**Sapph**	**(1,8,2)**	**12.889**	**83.68**	**0.315**	**0.7**	**1.436**	**0.95**	**17.96**	**17.98**	(1,-9,-2), (8,1,-2), (8,-9,2), (9,-1,-2), (9,-8,2), (-1,9,2), (-1,-8,-2), (-8,9,-2), (-8,-1,2) (-9,1,2), (-9,8,-2)
**Sapph**	**(0,8,-8)**	**12.627**	**76.82**	**0.302**	**0.5**	**3.036**	**1.51**	**37.95**	**37.98**	(0,-8,8), (8,0,8), (8,-8,-8), (-8,0,-8), (-8,8,8)
**Quartz**	**(5,1,8)**	**12.248**	**70.82**	**0.294**	**0.4**	**4.512**	**1.95**	**56.4**	**56.43**	(5,-6,-8)
**Quartz**	**(1,5,-8)**	**12.248**	**70.82**	**0.292**	**0.4**	**4.512**	**1.94**	**56.4**	**56.43**	(1,-6,8)
**Quartz**	**(-6,1,-8)**	**12.248**	**70.82**	**0.291**	**0.4**	**4.512**	**1.94**	**56.4**	**56.43**	(-6,5,8)
**LiNbO3**	**(2,6,17)**	**12.583**	**76**	**0.288**	**0.8**	**3.233**	**2.66**	**40.41**	**40.5**	(2,-8,-17), (6,2,-17), (6,-8,17), (8,-2,-17), (8,-6,17), (-2,8,17), (-2,-6,-17), (-6,8,-17), (-6,-2,17) (-8,2,17), (-8,6,-17)
**Sapph**	**(1,3,-23)**	**12.245**	**70.77**	**0.28**	**0.4**	**4.523**	**1.93**	**56.53**	**56.57**	(1,-4,23), (3,1,23), (3,-4,-23), (4,-1,23), (4,-3,-23), (-1,4,-23), (-1,-3,23), (-3,4,23), (-3,-1,-23) (-4,1,-23), (-4,3,23)
**Quartz**	**(9,-1,-1)**	**12.501**	**74.58**	**0.274**	**0.5**	**3.577**	**1.65**	**44.71**	**44.74**	(9,-8,1), (-1,9,1), (-1,-8,-1), (-2,9,-3), (-2,-7,3), (-8,9,-1), (-8,-1,1)
**Quartz**	**(9,-2,3)**	**12.412**	**73.17**	**0.273**	**0.4**	**3.923**	**1.45**	**49.04**	**49.06**	(9,-7,-3), (-7,9,3), (-7,-2,-3)
**Sapph**	**(3,4,-19)**	**12.883**	**83.45**	**0.252**	**0.6**	**1.489**	**0.86**	**18.62**	**18.64**	(3,-7,19), (4,3,19), (4,-7,-19), (7,-3,19), (7,-4,-19), (-3,7,-19), (-3,-4,19), (-4,7,19), (-4,-3,-19) (-7,3,-19), (-7,4,19)
**Sapph**	**(0,3,24)**	**12.312**	**71.7**	**0.232**	**0.4**	**4.289**	**1.6**	**53.61**	**53.64**	(0,3,-24), (0,-3,24), (0,-3,-24), (3,0,24), (3,0,-24), (3,-3,24), (3,-3,-24), (-3,0,24), (-3,0,-24) (-3,3,24), (-3,3,-24)
**LiNbO3**	**(2,3,-25)**	**12.717**	**78.7**	**0.219**	**0.8**	**2.591**	**2.02**	**32.38**	**32.45**	(2,-5,25), (3,2,25), (3,-5,-25), (5,-2,25), (5,-3,-25), (-2,5,-25), (-2,-3,25), (-3,5,25), (-3,-2,-25) (-5,2,-25), (-5,3,25)
**Quartz**	**(2,7,-3)**	**12.412**	**73.17**	**0.218**	**0.4**	**3.923**	**1.45**	**49.04**	**49.06**	(2,-9,3), (7,2,3), (-9,2,-3), (-9,7,3)
**Quartz**	**(7,-9,-3)**	**12.412**	**73.17**	**0.217**	**0.4**	**3.923**	**1.44**	**49.04**	**49.06**	
**LiNbO3**	**(1,8,11)**	**12.858**	**82.55**	**0.215**	**0.9**	**1.697**	**1.6**	**21.21**	**21.27**	(1,-9,-11), (8,1,-11), (8,-9,11), (9,-1,-11), (9,-8,11), (-1,9,11), (-1,-8,-11), (-8,9,-11), (-8,-1,11) (-9,1,11), (-9,8,-11)
**Quartz**	**(0,5,9)**	**12.635**	**76.98**	**0.211**	**0.4**	**2.998**	**1.08**	**37.47**	**37.49**	(0,-5,-9)
**Quartz**	**(5,0,-9)**	**12.635**	**76.98**	**0.209**	**0.4**	**2.998**	**1.07**	**37.47**	**37.49**	(-5,0,9)
**Quartz**	**(5,-5,9)**	**12.635**	**76.98**	**0.208**	**0.4**	**2.998**	**1.07**	**37.47**	**37.49**	

	Equiv. Refl.: (-5,5,-9)								
**Sapph**	**(3,6,9)**	**12.69**	**78.12**	**0.198**	**0.4**	**2.729**	**1.08**	**34.11**	**34.13**
	Equiv. Refl.: (3,6,-9), (3,-9,9), (3,-9,-9), (6,3,9), (6,3,-9), (6,-9,9), (6,-9,-9), (9,-3,9), (9,-3,-9) (9,-6,9), (9,-6,-9), (-3,9,9), (-3,9,-9), (-3,-6,9), (-3,-6,-9), (-6,9,9), (-6,9,-9), (-6,-3,9) (-6,-3,-9), (-9,3,9), (-9,3,-9), (-9,6,9), (-9,6,-9)								
**LiNbO3**	**(1,7,15)**	**12.458**	**73.88**	**0.172**	**0.6**	**3.749**	**2.2**	**46.86**	**46.91**
	Equiv. Refl.: (1,7,-15), (1,-8,15), (1,-8,-15), (7,1,15), (7,1,-15), (7,-8,15), (7,-8,-15), (8,-1,15), (8,-1,-15) (8,-7,15), (8,-7,-15), (-1,8,15), (-1,8,-15), (-1,-7,15), (-1,-7,-15), (-7,8,15), (-7,8,-15), (-7,-1,15) (-7,-1,-15), (-8,1,15), (-8,1,-15), (-8,7,15), (-8,7,-15)								
**LiNbO3**	**(3,5,-17)**	**12.35**	**72.24**	**0.132**	**0.5**	**4.152**	**2.04**	**51.91**	**51.95**
	Equiv. Refl.: (3,-8,17), (5,3,17), (5,-8,-17), (8,-3,17), (8,-5,-17), (-3,8,-17), (-3,-5,17), (-5,8,17), (-5,-3,-17) (-8,3,-17), (-8,5,17)								
**Sapph**	**(3,4,17)**	**12.23**	**70.58**	**0.105**	**0.2**	**4.572**	**1.03**	**57.15**	**57.16**
	Equiv. Refl.: (3,-7,-17), (4,3,-17), (4,-7,17), (7,-3,-17), (7,-4,17), (-3,7,17), (-3,-4,-17), (-4,7,-17), (-4,-3,17) (-7,3,17), (-7,4,-17)								
**LiNbO3**	**(1,8,-7)**	**12.286**	**71.33**	**0.101**	**0.4**	**4.381**	**1.84**	**54.76**	**54.8**
	Equiv. Refl.: (1,-9,7), (8,1,7), (8,-9,-7), (9,-1,7), (9,-8,-7), (-1,9,-7), (-1,-8,7), (-8,9,7), (-8,-1,-7) (-9,1,-7), (-9,8,7)								
**Sapph**	**(1,7,12)**	**12.721**	**78.79**	**0.074**	**0.2**	**2.571**	**0.59**	**32.13**	**32.14**
	Equiv. Refl.: (1,7,-12), (1,-8,12), (1,-8,-12), (7,1,12), (7,1,-12), (7,-8,12), (7,-8,-12), (8,-1,12), (8,-1,-12) (8,-7,12), (8,-7,-12), (-1,8,12), (-1,8,-12), (-1,-7,12), (-1,-7,-12), (-7,8,12), (-7,8,-12), (-7,-1,12) (-7,-1,-12), (-8,1,12), (-8,1,-12), (-8,7,12), (-8,7,-12)								
**LiNbO3**	**(5,5,9)**	**12.697**	**78.26**	**0.01**	**0.2**	**2.695**	**0.48**	**33.69**	**33.69**
	Equiv. Refl.: (5,5,-9), (5,-10,9), (5,-10,-9), (10,-5,9), (10,-5,-9), (-5,10,9), (-5,10,-9), (-5,-5,9), (-5,-5,-9) (-10,5,9), (-10,5,-9)								

Ei = Cryst	12.385 Refl (h,k,l)	keV EB [keV]	ΘB [°]	∫ IR dΘ [µrad]	Width [µrad]	Ei cotΘ [meV/µrad]	ΔE [meV]	ΔEg 2m, 50µm [meV]	ΔEt 2m, 50µm [meV]
**Si**	**(3,5,9)**	**12.241**	**81.24**	**4.451**	**4**	**1.908**	**7.7**	**23.85**	**25.06**
**Ge**	**(2,4,10)**	**12.004**	**75.75**	**3.368**	**7**	**3.147**	**22.16**	**39.33**	**45.14**
**Si**	**(0,2,10)**	**11.64**	**70.03**	**3.304**	**3**	**4.5**	**13.51**	**56.25**	**57.85**
	Equiv. Refl.: (2,6,8)								
**Si**	**(1,5,9)**	**11.807**	**72.43**	**2.493**	**2.3**	**3.922**	**8.99**	**49.02**	**49.84**
	Equiv. Refl.: (3,7,7)								
**Ge**	**(1,1,11)**	**12.153**	**78.89**	**2.187**	**6.1**	**2.433**	**14.86**	**30.41**	**33.85**
	Equiv. Refl.: (5,7,7)								
**Ge**	**(3,5,9)**	**11.751**	**71.59**	**1.566**	**4.2**	**4.123**	**17.16**	**51.54**	**54.32**
**LiNbO3**	**(3,4,20)**	**12.31**	**83.67**	**22.932**	**20.9**	**1.373**	**28.74**	**17.17**	**33.47**
	Equiv. Refl.: (4,-7,20), (7,-4,20), (-3,7,20), (-4,-3,20), (-7,3,20)								
**LiNbO3**	**(3,-7,-20)**	**12.31**	**83.67**	**22.897**	**20.9**	**1.373**	**28.74**	**17.17**	**33.47**
	Equiv. Refl.: (4,3,-20), (7,-3,-20), (-3,-4,-20), (-4,7,-20), (-7,4,-20)								
**LiNbO3**	**(5,5,6)**	**12.337**	**84.97**	**21.057**	**19.7**	**1.091**	**21.51**	**13.63**	**25.47**
	Equiv. Refl.: (5,-10,6), (10,-5,6), (-5,10,6), (-5,-5,6), (-10,5,6)								
**LiNbO3**	**(2,6,-16)**	**12.318**	**84.03**	**20.445**	**18.7**	**1.294**	**24.22**	**16.18**	**29.13**
	Equiv. Refl.: (-2,8,-16), (-6,-2,-16), (-8,2,-16)								
**LiNbO3**	**(6,-8,-16)**	**12.318**	**84.03**	**20.444**	**18.7**	**1.294**	**24.22**	**16.18**	**29.13**
	Equiv. Refl.: (8,-6,-16)								
**LiNbO3**	**(5,5,-6)**	**12.337**	**84.97**	**20.143**	**19.7**	**1.091**	**21.51**	**13.63**	**25.47**
	Equiv. Refl.: (5,-10,-6), (10,-5,-6), (-5,10,-6), (-5,-5,-6), (-10,5,-6)								
**LiNbO3**	**(2,-8,16)**	**12.318**	**84.03**	**19.447**	**18.7**	**1.294**	**24.22**	**16.18**	**29.13**
	Equiv. Refl.: (6,2,16), (8,-2,16), (-2,-6,16), (-6,8,16), (-8,6,16)								
**LiNbO3**	**(3,6,12)**	**12.272**	**82.26**	**17.166**	**15.7**	**1.684**	**26.48**	**21.05**	**33.83**
	Equiv. Refl.: (6,-9,12), (9,-6,12), (-6,-3,12)								
**LiNbO3**	**(-3,9,12)**	**12.272**	**82.26**	**17.165**	**15.7**	**1.684**	**26.48**	**21.05**	**33.83**
	Equiv. Refl.: (-9,3,12)								
**LiNbO3**	**(3,-9,-12)**	**12.272**	**82.26**	**16.811**	**15.7**	**1.684**	**26.48**	**21.05**	**33.83**
	Equiv. Refl.: (6,3,-12), (9,-3,-12), (-3,-6,-12), (-6,9,-12), (-9,6,-12)								
**LiNbO3**	**(2,-9,-10)**	**12.229**	**80.88**	**15.455**	**13.8**	**1.987**	**27.39**	**24.84**	**36.98**
	Equiv. Refl.: (7,2,-10), (9,-2,-10), (-2,-7,-10), (-7,9,-10), (-9,7,-10)								
**LiNbO3**	**(3,-9,12)**	**12.272**	**82.26**	**14.836**	**13.9**	**1.684**	**23.4**	**21.05**	**31.48**
	Equiv. Refl.: (6,3,12), (9,-3,12), (-3,-6,12), (-6,9,12), (-9,6,12)								
**LiNbO3**	**(2,-6,-22)**	**12.285**	**82.71**	**14.786**	**14.1**	**1.584**	**22.27**	**19.8**	**29.8**
	Equiv. Refl.: (4,2,-22), (6,-2,-22), (-2,-4,-22), (-4,6,-22), (-6,4,-22)								
**LiNbO3**	**(4,-6,22)**	**12.285**	**82.71**	**14.734**	**14.1**	**1.584**	**22.27**	**19.8**	**29.8**
	Equiv. Refl.: (6,-4,22)								
**LiNbO3**	**(2,4,22)**	**12.285**	**82.71**	**14.733**	**14.1**	**1.584**	**22.27**	**19.8**	**29.8**
	Equiv. Refl.: (-2,6,22), (-4,-2,22), (-6,2,22)								
**LiNbO3**	**(3,6,-12)**	**12.272**	**82.26**	**14.57**	**13.9**	**1.684**	**23.4**	**21.05**	**31.48**
	Equiv. Refl.: (6,-9,-12), (9,-6,-12), (-3,9,-12), (-6,-3,-12), (-9,3,-12)								
**LiNbO3**	**(2,7,10)**	**12.229**	**80.88**	**14.45**	**13.8**	**1.987**	**27.39**	**24.84**	**36.98**
	Equiv. Refl.: (7,-9,10), (9,-7,10), (-2,9,10), (-7,-2,10), (-9,2,10)								
**LiNbO3**	**(0,5,22)**	**12.047**	**76.58**	**13.536**	**11.8**	**2.956**	**34.96**	**36.95**	**50.87**
	Equiv. Refl.: (5,-5,22), (-5,0,22)								
**LiNbO3**	**(4,6,4)**	**12.253**	**81.64**	**13.417**	**12.7**	**1.821**	**23.11**	**22.76**	**32.44**
	Equiv. Refl.: (6,-10,4), (10,-6,4), (-4,10,4), (-6,-4,4), (-10,4,4)								

Ei =	12.385	keV						ΔEg	ΔEt
Cryst	Refl	EB	ΘB	∫ IR dΘ	Width	Ei cotΘ	ΔE	2m, 50µm	2m, 50µm
	(h,k,l)	[keV]	[°]	[µrad]	[µrad]	[meV/µrad]	[meV]	[meV]	[meV]
**LiNbO3**	**(4,-10,-4)**	**12.253**	**81.64**	**13.357**	**12.7**	**1.821**	**23.11**	**22.76**	**32.43**
	Equiv. Refl.: (6,4,-4), (10,-4,-4), (-4,-6,-4), (-6,10,-4), (-10,6,-4)								
**LiNbO3**	**(0,-5,-22)**	**12.047**	**76.58**	**12.941**	**11.8**	**2.956**	**34.96**	**36.95**	**50.87**
	Equiv. Refl.: (5,0,-22), (-5,5,-22)								
**LiNbO3**	**(4,6,-2)**	**12.155**	**78.94**	**12.826**	**11.6**	**2.421**	**28.11**	**30.26**	**41.3**
	Equiv. Refl.: (6,-10,-2), (10,-6,-2), (-4,10,-2), (-6,-4,-2), (-10,4,-2)								
**LiNbO3**	**(1,2,26)**	**12.195**	**79.94**	**12.589**	**11.7**	**2.196**	**25.74**	**27.45**	**37.63**
	Equiv. Refl.: (2,-3,26), (3,-2,26), (-1,3,26), (-2,-1,26), (-3,1,26)								
**LiNbO3**	**(1,-3,-26)**	**12.195**	**79.94**	**12.565**	**11.7**	**2.196**	**25.74**	**27.45**	**37.63**
	Equiv. Refl.: (2,1,-26), (3,-1,-26), (-1,-2,-26), (-2,3,-26), (-3,2,-26)								
**LiNbO3**	**(4,-10,2)**	**12.155**	**78.94**	**12.422**	**11.6**	**2.421**	**28.11**	**30.26**	**41.3**
	Equiv. Refl.: (6,4,2), (10,-4,2), (-4,-6,2)								
**LiNbO3**	**(-6,10,2)**	**12.155**	**78.94**	**12.421**	**11.6**	**2.421**	**28.11**	**30.26**	**41.3**
	Equiv. Refl.: (-10,6,2)								
**LiNbO3**	**(5,5,0)**	**12.042**	**76.48**	**12.361**	**11.1**	**2.977**	**33.13**	**37.21**	**49.82**
	Equiv. Refl.: (5,-10,0), (10,-5,0), (-5,10,0), (-5,-5,0), (-10,5,0)								
**Quartz**	**(-5,8,-6)**	**12.304**	**83.43**	**11.832**	**9.7**	**1.426**	**13.86**	**17.83**	**22.58**
	Equiv. Refl.: (-5,-3,6)								
**Quartz**	**(8,-3,-6)**	**12.304**	**83.43**	**11.83**	**9.7**	**1.426**	**13.86**	**17.83**	**22.58**
	Equiv. Refl.: (8,-5,6), (-3,8,6)								
**Quartz**	**(-3,-5,-6)**	**12.304**	**83.43**	**11.829**	**9.7**	**1.426**	**13.86**	**17.83**	**22.58**
**Quartz**	**(5,3,-6)**	**12.304**	**83.43**	**11.742**	**9.7**	**1.426**	**13.86**	**17.83**	**22.58**
	Equiv. Refl.: (5,-8,6)								
**Quartz**	**(-8,3,6)**	**12.304**	**83.43**	**11.74**	**9.7**	**1.426**	**13.86**	**17.83**	**22.58**
	Equiv. Refl.: (-8,5,-6)								
**Quartz**	**(3,5,6)**	**12.304**	**83.43**	**11.739**	**9.7**	**1.426**	**13.86**	**17.83**	**22.58**
	Equiv. Refl.: (3,-8,-6)								
**LiNbO3**	**(0,-2,26)**	**11.955**	**74.85**	**10.257**	**9.3**	**3.353**	**31.2**	**41.92**	**52.26**
	Equiv. Refl.: (2,0,26), (-2,2,26)								
**LiNbO3**	**(3,-8,-16)**	**12.08**	**77.26**	**10.225**	**8.9**	**2.8**	**24.82**	**35**	**42.91**
	Equiv. Refl.: (5,3,-16), (8,-3,-16), (-3,-5,-16), (-5,8,-16), (-8,5,-16)								
**LiNbO3**	**(0,2,-26)**	**11.955**	**74.85**	**10.197**	**9.3**	**3.353**	**31.2**	**41.92**	**52.26**
	Equiv. Refl.: (2,-2,-26), (-2,0,-26)								
**LiNbO3**	**(1,-9,4)**	**12.014**	**75.95**	**10.112**	**8.9**	**3.101**	**27.74**	**38.76**	**47.66**
	Equiv. Refl.: (8,1,4), (9,-1,4), (-1,-8,4), (-8,9,4), (-9,8,4)								
**LiNbO3**	**(0,8,-8)**	**11.685**	**70.64**	**9.843**	**8.6**	**4.351**	**37.21**	**54.38**	**65.89**
	Equiv. Refl.: (8,-8,-8), (-8,0,-8)								
**LiNbO3**	**(1,8,-4)**	**12.014**	**75.95**	**9.728**	**8.9**	**3.101**	**27.74**	**38.76**	**47.66**
	Equiv. Refl.: (8,-9,-4), (9,-8,-4), (-1,9,-4), (-8,-1,-4), (-9,1,-4)								
**LiNbO3**	**(1,8,2)**	**11.914**	**74.15**	**9.4**	**8.4**	**3.517**	**29.38**	**43.96**	**52.88**
	Equiv. Refl.: (8,-9,2), (9,-8,2), (-1,9,2), (-8,-1,2), (-9,1,2)								
**LiNbO3**	**(0,-7,16)**	**12.08**	**77.26**	**9.378**	**8.6**	**2.8**	**23.94**	**35**	**42.4**
	Equiv. Refl.: (7,0,16), (-7,7,16)								
**LiNbO3**	**(0,-8,8)**	**11.685**	**70.64**	**9.305**	**8.6**	**4.351**	**37.21**	**54.38**	**65.89**
	Equiv. Refl.: (8,0,8), (-8,8,8)								
**LiNbO3**	**(1,-9,-2)**	**11.914**	**74.15**	**9.265**	**8.4**	**3.517**	**29.38**	**43.96**	**52.88**
	Equiv. Refl.: (8,1,-2), (9,-1,-2), (-1,-8,-2), (-8,9,-2), (-9,8,-2)								
**LiNbO3**	**(0,8,10)**	**11.989**	**75.47**	**9.246**	**8**	**3.209**	**25.61**	**40.11**	**47.59**
	Equiv. Refl.: (8,-8,10), (-8,0,10)								
**LiNbO3**	**(2,2,24)**	**11.764**	**71.77**	**9.205**	**8.1**	**4.078**	**33.02**	**50.98**	**60.74**
	Equiv. Refl.: (2,-4,24), (4,-2,24), (-2,-2,24)								

Ei =	12.385	keV						ΔEg	ΔEt
Cryst	Refl	EB	ΘB	∫ IR dΘ	Width	Ei cotΘ	ΔE	2m, 50µm	2m, 50µm
	(h,k,l)	[keV]	[°]	[µrad]	[µrad]	[meV/µrad]	[meV]	[meV]	[meV]
LiNbO3	(-2,4,24)	11.764	71.77	9.204	8.1	4.078	33.02	50.98	60.74
	Equiv. Refl.: (-4,2,24)								
LiNbO3	(2,2,-24)	11.764	71.77	9.184	8.1	4.078	33.02	50.98	60.74
	Equiv. Refl.: (2,-4,-24), (4,-2,-24), (-2,4,-24), (-2,-2,-24), (-4,2,-24)								
LiNbO3	(0,7,-16)	12.08	77.26	9.081	8.6	2.8	23.94	35	42.4
	Equiv. Refl.: (7,-7,-16), (-7,0,-16)								
LiNbO3	(3,5,16)	12.08	77.26	9.064	8.9	2.8	24.82	35	42.91
	Equiv. Refl.: (5,-8,16), (8,-5,16), (-3,8,16), (-5,-3,16), (-8,3,16)								
LiNbO3	(2,6,14)	11.821	72.64	9.004	7.9	3.872	30.54	48.4	57.23
	Equiv. Refl.: (6,-8,14), (8,-6,14), (-2,8,14), (-6,-2,14), (-8,2,14)								
LiNbO3	(2,5,18)	11.84	72.95	8.906	7.9	3.799	30.14	47.49	56.25
	Equiv. Refl.: (5,-7,18), (7,-5,18), (-2,7,18), (-5,-2,18), (-7,2,18)								
LiNbO3	(2,-7,-18)	11.84	72.95	8.857	7.9	3.799	30.14	47.49	56.25
	Equiv. Refl.: (5,2,-18), (7,-2,-18), (-2,-5,-18), (-5,7,-18), (-7,5,-18)								
LiNbO3	(2,-8,-14)	11.821	72.64	8.696	7.9	3.872	30.54	48.4	57.23
	Equiv. Refl.: (6,2,-14), (8,-2,-14), (-2,-6,-14), (-6,8,-14), (-8,6,-14)								
LiNbO3	(2,-7,18)	11.84	72.95	8.587	7.7	3.799	29.13	47.49	55.72
	Equiv. Refl.: (5,2,18), (7,-2,18), (-2,-5,18), (-5,7,18), (-7,5,18)								
LiNbO3	(2,5,-18)	11.84	72.95	8.477	7.7	3.799	29.13	47.49	55.72
	Equiv. Refl.: (5,-7,-18), (7,-5,-18), (-2,7,-18), (-5,-2,-18), (-7,2,-18)								
LiNbO3	(1,-8,12)	11.79	72.17	8.392	7.5	3.984	29.69	49.8	57.98
	Equiv. Refl.: (7,1,12), (8,-1,12), (-1,-7,12), (-7,8,12), (-8,7,12)								
LiNbO3	(1,7,-12)	11.79	72.17	8.223	7.5	3.984	29.69	49.8	57.98
	Equiv. Refl.: (7,-8,-12), (8,-7,-12), (-1,8,-12), (-7,-1,-12), (-8,1,-12)								
LiNbO3	(0,-8,-10)	11.989	75.47	8.173	8	3.209	25.61	40.11	47.59
	Equiv. Refl.: (8,0,-10), (-8,8,-10)								
LiNbO3	(4,5,-10)	11.745	71.5	8.045	7	4.145	29.11	51.81	59.43
	Equiv. Refl.: (5,-9,-10), (9,-5,-10), (-4,9,-10), (-5,-4,-10), (-9,4,-10)								
LiNbO3	(2,-9,8)	11.931	74.43	7.785	7	3.451	24.16	43.13	49.44
	Equiv. Refl.: (7,2,8), (9,-2,8), (-2,-7,8), (-7,9,8), (-9,7,8)								
LiNbO3	(1,5,20)	11.829	72.77	7.768	7.1	3.842	27.12	48.02	55.15
	Equiv. Refl.: (5,-6,20), (6,-5,20), (-1,6,20), (-5,-1,20), (-6,1,20)								
LiNbO3	(1,-6,-20)	11.829	72.77	7.726	7.1	3.842	27.12	48.02	55.15
	Equiv. Refl.: (5,1,-20), (6,-1,-20), (-1,-5,-20), (-5,6,-20), (-6,5,-20)								
Quartz	(5,1,-8)	12.248	81.46	7.643	6.2	1.859	11.56	23.24	25.95
Quartz	(5,-6,8)	12.248	81.46	7.642	6.2	1.859	11.56	23.24	25.95
Quartz	(-6,5,-8)	12.248	81.46	7.636	6.2	1.859	11.55	23.24	25.95
Quartz	(-6,1,8)	12.248	81.46	7.635	6.2	1.859	11.55	23.24	25.95
Quartz	(1,5,8)	12.248	81.46	7.633	6.2	1.859	11.55	23.24	25.95
Quartz	(1,-6,-8)	12.248	81.46	7.632	6.2	1.859	11.54	23.24	25.95
Quartz	(-5,6,-8)	12.248	81.46	7.628	6.2	1.859	11.56	23.24	25.95
	Equiv. Refl.: (-5,-1,8)								
Quartz	(6,-5,8)	12.248	81.46	7.622	6.2	1.859	11.55	23.24	25.95
Quartz	(6,-1,-8)	12.248	81.46	7.621	6.2	1.859	11.55	23.24	25.95
Quartz	(-1,-5,-8)	12.248	81.46	7.618	6.2	1.859	11.55	23.24	25.95
Quartz	(-1,6,8)	12.248	81.46	7.617	6.2	1.859	11.54	23.24	25.95
LiNbO3	(4,-9,10)	11.745	71.5	7.535	7	4.145	29.11	51.81	59.43
	Equiv. Refl.: (5,4,10), (9,-4,10), (-4,-5,10), (-5,9,10), (-9,5,10)								
LiNbO3	(1,7,12)	11.79	72.17	7.439	6.7	3.984	26.85	49.8	56.58
	Equiv. Refl.: (7,-8,12), (8,-7,12), (-1,8,12), (-7,-1,12), (-8,1,12)								

Ei = Cryst	12.385 Refl (h,k,l)	keV EB [keV]	ΘB [°]	∫ IR dΘ [µrad]	Width [µrad]	Ei cotΘ [meV/µrad]	ΔE [meV]	ΔEg 2m, 50µm [meV]	ΔEt 2m, 50µm [meV]
Quartz	(0,3,10)	12.275	82.35	7.355	6	1.663	9.98	20.79	23.06
	Equiv. Refl.: (0,-3,-10), (-3,0,10)								
Quartz	(3,0,-10)	12.275	82.35	7.354	6	1.663	9.98	20.79	23.06
	Equiv. Refl.: (3,-3,10), (-3,3,-10)								
LiNbO3	(-7,8,-12)	11.79	72.17	7.305	6.7	3.984	26.85	49.8	56.58
	Equiv. Refl.: (-8,7,-12)								
LiNbO3	(1,-8,-12)	11.79	72.17	7.304	6.7	3.984	26.85	49.8	56.58
	Equiv. Refl.: (7,1,-12), (8,-1,-12), (-1,-7,-12)								
LiNbO3	(2,7,-8)	11.931	74.43	7.282	7	3.451	24.16	43.13	49.44
	Equiv. Refl.: (7,-9,-8), (9,-7,-8), (-2,9,-8), (-7,-2,-8), (-9,2,-8)								
LiNbO3	(0,1,26)	11.709	70.99	7.095	6.4	4.267	27.44	53.34	59.98
	Equiv. Refl.: (1,-1,26), (-1,0,26)								
LiNbO3	(0,-1,-26)	11.709	70.99	6.992	6.4	4.267	27.44	53.34	59.98
	Equiv. Refl.: (1,0,-26), (-1,1,-26)								
Sapph	(0,8,4)	12.186	79.71	6.959	5.8	2.248	12.97	28.1	30.95
	Equiv. Refl.: (0,-8,-4), (8,0,-4), (8,-8,4), (-8,0,4), (-8,8,-4)								
Quartz	(-4,9,-4)	12.27	82.17	6.87	5.7	1.703	9.74	21.29	23.41
	Equiv. Refl.: (-4,-5,4), (-5,9,4), (-5,-4,-4)								
Quartz	(9,-4,4)	12.27	82.17	6.869	5.7	1.703	9.73	21.29	23.41
	Equiv. Refl.: (9,-5,-4)								
Quartz	(4,5,-4)	12.27	82.17	6.827	5.7	1.703	9.74	21.29	23.41
	Equiv. Refl.: (4,-9,4), (5,4,4), (5,-9,-4)								
Quartz	(-9,5,4)	12.27	82.17	6.826	5.7	1.703	9.74	21.29	23.41
Quartz	(-9,4,-4)	12.27	82.17	6.825	5.7	1.703	9.73	21.29	23.41
Sapph	(3,6,0)	11.941	74.61	6.631	5.3	3.409	18	42.61	46.26
	Equiv. Refl.: (3,-9,0), (6,3,0), (6,-9,0), (9,-3,0), (9,-6,0), (-3,9,0), (-3,-6,0), (-6,9,0), (-6,-3,0) (-9,3,0), (-9,6,0)								
Sapph	(2,3,-22)	12.38	88.35	6.485	7.7	0.357	2.75	4.46	5.24
	Equiv. Refl.: (2,-5,22), (3,2,22), (3,-5,-22), (5,-2,22), (5,-3,-22), (-2,5,-22), (-2,-3,22), (-3,5,22), (-3,-2,-22) (-5,2,-22), (-5,3,22)								
Quartz	(-5,7,-7)	12.135	78.47	6.033	4.8	2.527	12.06	31.59	33.81
Quartz	(-5,-2,7)	12.135	78.47	6.032	4.8	2.527	12.06	31.59	33.81
Quartz	(-2,-5,-7)	12.135	78.47	6.03	4.8	2.527	12.05	31.59	33.81
Quartz	(-2,7,7)	12.135	78.47	6.029	4.8	2.527	12.05	31.59	33.81
Quartz	(7,-2,-7)	12.135	78.47	6.027	4.8	2.527	12.05	31.59	33.81
	Equiv. Refl.: (7,-5,7)								
Quartz	(5,2,-7)	12.135	78.47	5.927	4.8	2.527	12.06	31.59	33.81
	Equiv. Refl.: (5,-7,7)								
Quartz	(2,5,7)	12.135	78.47	5.924	4.8	2.527	12.05	31.59	33.81
Quartz	(2,-7,-7)	12.135	78.47	5.923	4.8	2.527	12.05	31.59	33.81
Quartz	(-7,2,7)	12.135	78.47	5.922	4.8	2.527	12.05	31.59	33.81
	Equiv. Refl.: (-7,5,-7)								
Sapph	(1,1,24)	11.748	71.54	5.638	4.5	4.135	18.6	51.68	54.93
	Equiv. Refl.: (1,1,-24), (1,-2,24), (1,-2,-24), (2,-1,24), (2,-1,-24), (-1,2,24), (-1,2,-24), (-1,-1,24), (-1,-1,-24) (-2,1,24), (-2,1,-24)								
Sapph	(1,5,-19)	12.345	85.4	5.606	5.3	0.997	5.3	12.47	13.55
	Equiv. Refl.: (1,-6,19), (5,1,19), (5,-6,-19), (6,-1,19), (6,-5,-19), (-1,6,-19), (-1,-5,19), (-5,6,19), (-5,-1,-19) (-6,1,-19), (-6,5,19)								

Ei =	12.385	keV						ΔEg	ΔEt
Cryst	Refl	EB	ΘB	∫ IR dΘ	Width	Ei cotΘ	ΔE	2m, 50µm	2m, 50µm
	(h,k,l)	[keV]	[°]	[µrad]	[µrad]	[meV/µrad]	[meV]	[meV]	[meV]
**Sapph**	**(4,5,-4)**	**11.904**	**73.98**	**4.874**	**3.9**	**3.556**	**13.7**	**44.45**	**46.52**
	Equiv. Refl.: (4,-9,4), (5,4,4), (5,-9,-4), (9,-4,4), (9,-5,-4), (-4,9,-4), (-4,-5,4), (-5,9,4), (-5,-4,-4) (-9,4,-4), (-9,5,4)								
**Quartz**	**(0,7,-5)**	**11.701**	**70.87**	**4.497**	**3.5**	**4.297**	**15.14**	**53.71**	**55.8**
	Equiv. Refl.: (0,-7,5)								
**Quartz**	**(7,0,5)**	**11.701**	**70.87**	**4.495**	**3.5**	**4.297**	**15.13**	**53.71**	**55.8**
	Equiv. Refl.: (7,-7,-5), (-7,0,-5), (-7,7,5)								
**LiNbO3**	**(1,1,27)**	**12.312**	**83.76**	**4.39**	**5.9**	**1.354**	**8.03**	**16.93**	**18.74**
	Equiv. Refl.: (1,1,-27), (1,-2,27), (1,-2,-27), (2,-1,27), (2,-1,-27), (-1,2,27), (-1,2,-27), (-1,-1,27), (-1,-1,-27) (-2,1,27), (-2,1,-27)								
**Quartz**	**(-2,9,-2)**	**12.145**	**78.69**	**4.226**	**3.3**	**2.477**	**8.27**	**30.96**	**32.04**
	Equiv. Refl.: (-2,-7,2)								
**Quartz**	**(9,-2,2)**	**12.145**	**78.69**	**4.225**	**3.3**	**2.477**	**8.26**	**30.96**	**32.04**
	Equiv. Refl.: (9,-7,-2), (-7,9,2), (-7,-2,-2)								
**Quartz**	**(4,3,7)**	**11.959**	**74.92**	**4.051**	**3.1**	**3.336**	**10.41**	**41.7**	**42.98**
	Equiv. Refl.: (4,-7,-7)								
**Quartz**	**(3,4,-7)**	**11.959**	**74.92**	**4.047**	**3.1**	**3.336**	**10.4**	**41.7**	**42.98**
	Equiv. Refl.: (-7,3,-7), (-7,4,7)								
**Quartz**	**(3,-7,7)**	**11.959**	**74.92**	**4.046**	**3.1**	**3.336**	**10.4**	**41.7**	**42.98**
**Quartz**	**(2,7,-2)**	**12.145**	**78.69**	**4.02**	**3.3**	**2.477**	**8.27**	**30.96**	**32.04**
	Equiv. Refl.: (2,-9,2)								
**Quartz**	**(7,2,2)**	**12.145**	**78.69**	**4.019**	**3.3**	**2.477**	**8.26**	**30.96**	**32.04**
	Equiv. Refl.: (7,-9,-2), (-9,2,-2), (-9,7,2)								
**Quartz**	**(-4,7,7)**	**11.959**	**74.92**	**3.885**	**3.1**	**3.336**	**10.41**	**41.7**	**42.98**
	Equiv. Refl.: (-4,-3,-7)								
**Quartz**	**(7,-3,7)**	**11.959**	**74.92**	**3.882**	**3.1**	**3.336**	**10.4**	**41.7**	**42.98**
	Equiv. Refl.: (-3,-4,7)								
**Quartz**	**(7,-4,-7)**	**11.959**	**74.92**	**3.881**	**3.1**	**3.336**	**10.4**	**41.7**	**42.98**
	Equiv. Refl.: (-3,7,-7)								
**Quartz**	**(2,7,1)**	**11.981**	**75.32**	**3.866**	**3**	**3.243**	**9.67**	**40.54**	**41.68**
	Equiv. Refl.: (2,-9,-1), (-9,2,1), (-9,7,-1)								
**Quartz**	**(7,2,-1)**	**11.981**	**75.32**	**3.865**	**3**	**3.243**	**9.67**	**40.54**	**41.68**
	Equiv. Refl.: (7,-9,1)								
**Sapph**	**(1,7,6)**	**11.714**	**71.05**	**3.812**	**3.1**	**4.253**	**13**	**53.16**	**54.73**
	Equiv. Refl.: (1,7,-6), (1,-8,6), (1,-8,-6), (7,1,6), (7,1,-6), (7,-8,6), (7,-8,-6), (8,-1,6), (8,-1,-6) (8,-7,6), (8,-7,-6), (-1,8,6), (-1,8,-6), (-1,-7,6), (-1,-7,-6), (-7,8,6), (-7,8,-6), (-7,-1,6) (-7,-1,-6), (-8,1,6), (-8,1,-6), (-8,7,6), (-8,7,-6)								
**Quartz**	**(-5,2,9)**	**12.12**	**78.13**	**3.806**	**3.1**	**2.603**	**8.14**	**32.53**	**33.54**
	Equiv. Refl.: (-5,3,-9)								
**Quartz**	**(5,-2,-9)**	**12.12**	**78.13**	**3.805**	**3.1**	**2.603**	**8.14**	**32.53**	**33.54**
	Equiv. Refl.: (5,-3,9)								
**Quartz**	**(3,-5,9)**	**12.12**	**78.13**	**3.802**	**3.1**	**2.603**	**8.14**	**32.53**	**33.53**
**Quartz**	**(3,2,-9)**	**12.12**	**78.13**	**3.801**	**3.1**	**2.603**	**8.13**	**32.53**	**33.53**
	Equiv. Refl.: (-3,5,-9)								
**Quartz**	**(2,-5,-9)**	**12.12**	**78.13**	**3.8**	**3.1**	**2.603**	**8.13**	**32.53**	**33.53**
	Equiv. Refl.: (-3,-2,9)								
**Quartz**	**(2,3,9)**	**12.12**	**78.13**	**3.799**	**3.1**	**2.603**	**8.13**	**32.53**	**33.53**
	Equiv. Refl.: (-2,5,9)								
**Quartz**	**(-2,-3,-9)**	**12.12**	**78.13**	**3.798**	**3.1**	**2.603**	**8.13**	**32.53**	**33.53**

Ei =	12.385	keV						ΔEg	ΔEt
Cryst	Refl	EB	ΘB	∫ IR dΘ	Width	Ei cotΘ	ΔE	2m, 50µm	2m, 50µm
	(h,k,l)	[keV]	[°]	[µrad]	[µrad]	[meV/µrad]	[meV]	[meV]	[meV]
**Sapph**	**(1,4,21)**	**12.165**	**79.19**	**3.574**	**3.1**	**2.365**	**7.43**	**29.56**	**30.48**
	Equiv. Refl.: (1,4,-21), (1,-5,21), (1,-5,-21), (4,1,21), (4,1,-21), (4,-5,21), (4,-5,-21), (5,-1,21), (5,-1,-21) (5,-4,21), (5,-4,-21), (-1,5,21), (-1,5,-21), (-1,-4,21), (-1,-4,-21), (-4,5,21), (-4,5,-21), (-4,-1,21) (-4,-1,-21), (-5,1,21), (-5,1,-21), (-5,4,21), (-5,4,-21)								
**Quartz**	**(0,6,7)**	**11.87**	**73.41**	**3.527**	**2.8**	**3.689**	**10.36**	**46.11**	**47.26**
	Equiv. Refl.: (0,-6,-7)								
**Quartz**	**(6,0,-7)**	**11.87**	**73.41**	**3.522**	**2.8**	**3.689**	**10.35**	**46.11**	**47.26**
	Equiv. Refl.: (6,-6,7), (-6,0,7), (-6,6,-7)								
**Quartz**	**(2,3,-9)**	**12.12**	**78.13**	**3.515**	**2.9**	**2.603**	**7.59**	**32.53**	**33.41**
**Quartz**	**(2,-5,9)**	**12.12**	**78.13**	**3.514**	**2.9**	**2.603**	**7.58**	**32.53**	**33.4**
**Quartz**	**(3,2,9)**	**12.12**	**78.13**	**3.513**	**2.9**	**2.603**	**7.58**	**32.53**	**33.4**
**Quartz**	**(3,-5,-9)**	**12.12**	**78.13**	**3.512**	**2.9**	**2.603**	**7.58**	**32.53**	**33.4**
**Quartz**	**(-5,2,-9)**	**12.12**	**78.13**	**3.505**	**2.9**	**2.603**	**7.57**	**32.53**	**33.4**
	Equiv. Refl.: (-5,3,9)								
**Sapph**	**(0,5,-20)**	**12.153**	**78.9**	**3.493**	**3.3**	**2.429**	**7.99**	**30.37**	**31.4**
	Equiv. Refl.: (0,-5,20), (5,0,20), (5,-5,-20), (-5,0,-20), (-5,5,20)								
**Quartz**	**(-2,-3,9)**	**12.12**	**78.13**	**3.489**	**2.9**	**2.603**	**7.59**	**32.53**	**33.41**
**Quartz**	**(-2,5,-9)**	**12.12**	**78.13**	**3.488**	**2.9**	**2.603**	**7.58**	**32.53**	**33.4**
**Quartz**	**(-3,-2,-9)**	**12.12**	**78.13**	**3.487**	**2.9**	**2.603**	**7.58**	**32.53**	**33.4**
**Quartz**	**(-3,5,9)**	**12.12**	**78.13**	**3.486**	**2.9**	**2.603**	**7.58**	**32.53**	**33.4**
**Quartz**	**(5,-2,9)**	**12.12**	**78.13**	**3.479**	**2.9**	**2.603**	**7.57**	**32.53**	**33.4**
	Equiv. Refl.: (5,-3,-9)								
**Quartz**	**(4,5,4)**	**12.27**	**82.17**	**3.425**	**3.2**	**1.703**	**5.49**	**21.29**	**21.98**
	Equiv. Refl.: (4,-9,-4)								
**Quartz**	**(-9,4,4)**	**12.27**	**82.17**	**3.424**	**3.2**	**1.703**	**5.48**	**21.29**	**21.98**
	Equiv. Refl.: (-9,5,-4)								
**Quartz**	**(5,4,-4)**	**12.27**	**82.17**	**3.422**	**3.2**	**1.703**	**5.48**	**21.29**	**21.98**
	Equiv. Refl.: (5,-9,4)								
**Sapph**	**(4,5,-7)**	**12.216**	**80.51**	**3.413**	**3**	**2.069**	**6.18**	**25.87**	**26.59**
	Equiv. Refl.: (4,-9,7), (5,4,7), (5,-9,-7), (9,-4,7), (9,-5,-7), (-4,9,-7), (-4,-5,7), (-5,9,7), (-5,-4,-7) (-9,4,-7), (-9,5,7)								
**Quartz**	**(9,-5,4)**	**12.27**	**82.17**	**3.369**	**3.2**	**1.703**	**5.48**	**21.29**	**21.98**
	Equiv. Refl.: (-4,-5,-4)								
**Quartz**	**(9,-4,-4)**	**12.27**	**82.17**	**3.368**	**3.2**	**1.703**	**5.48**	**21.29**	**21.98**
	Equiv. Refl.: (-4,9,4)								
**Quartz**	**(-5,9,-4)**	**12.27**	**82.17**	**3.366**	**3.2**	**1.703**	**5.48**	**21.29**	**21.98**
	Equiv. Refl.: (-5,-4,4)								
**Quartz**	**(3,-1,-10)**	**12.101**	**77.7**	**3.322**	**2.6**	**2.701**	**7.05**	**33.76**	**34.49**
	Equiv. Refl.: (3,-2,10)								
**Quartz**	**(-2,3,-10)**	**12.101**	**77.7**	**3.318**	**2.6**	**2.701**	**7.04**	**33.76**	**34.49**
**Quartz**	**(-2,-1,10)**	**12.101**	**77.7**	**3.317**	**2.6**	**2.701**	**7.04**	**33.76**	**34.49**
**Quartz**	**(-1,-2,-10)**	**12.101**	**77.7**	**3.315**	**2.6**	**2.701**	**7.04**	**33.76**	**34.49**
**Quartz**	**(-1,3,10)**	**12.101**	**77.7**	**3.314**	**2.6**	**2.701**	**7.04**	**33.76**	**34.49**
**Quartz**	**(-2,9,1)**	**11.981**	**75.32**	**3.255**	**3**	**3.243**	**9.67**	**40.54**	**41.68**
	Equiv. Refl.: (-2,-7,-1)								
**Quartz**	**(9,-2,-1)**	**11.981**	**75.32**	**3.254**	**3**	**3.243**	**9.67**	**40.54**	**41.68**
	Equiv. Refl.: (9,-7,1), (-7,9,-1), (-7,-2,1)								
**Quartz**	**(-3,1,10)**	**12.101**	**77.7**	**3.21**	**2.6**	**2.701**	**7.05**	**33.76**	**34.49**
	Equiv. Refl.: (-3,2,-10)								
**Quartz**	**(2,1,-10)**	**12.101**	**77.7**	**3.206**	**2.6**	**2.701**	**7.04**	**33.76**	**34.49**
	Equiv. Refl.: (2,-3,10)								
**Quartz**	**(1,2,10)**	**12.101**	**77.7**	**3.204**	**2.6**	**2.701**	**7.04**	**33.76**	**34.49**

**Ei =**	**12.385**	**keV**						**ΔEg**	**ΔEt**
**Cryst**	**Refl**	**EB**	**ΘB**	**∫ IR dΘ**	**Width**	**Ei cotΘ**	**ΔE**	**2m, 50µm**	**2m, 50µm**
	**(h,k,l)**	**[keV]**	**[°]**	**[µrad]**	**[µrad]**	**[meV/µrad]**	**[meV]**	**[meV]**	**[meV]**
**Quartz**	**(1,-3,-10)**	**12.101**	**77.7**	**3.202**	**2.6**	**2.701**	**7.04**	**33.76**	**34.49**
**Quartz**	**(-8,4,-6)**	**12.217**	**80.56**	**3.199**	**2.6**	**2.06**	**5.42**	**25.75**	**26.32**
**Quartz**	**(-8,4,6)**	**12.217**	**80.56**	**3.198**	**2.6**	**2.06**	**5.42**	**25.75**	**26.32**
**Quartz**	**(4,4,6)**	**12.217**	**80.56**	**3.196**	**2.6**	**2.06**	**5.41**	**25.75**	**26.31**
	Equiv. Refl.: (4,4,-6), (4,-8,6), (4,-8,-6)								
**Quartz**	**(8,-1,4)**	**11.918**	**74.22**	**3.176**	**2.5**	**3.499**	**8.76**	**43.74**	**44.61**
	Equiv. Refl.: (8,-7,-4), (-1,8,-4), (-1,-7,4), (-7,8,4), (-7,-1,-4)								
**LiNbO3**	**(4,6,-5)**	**12.326**	**84.43**	**3.175**	**5.3**	**1.208**	**6.41**	**15.1**	**16.41**
	Equiv. Refl.: (4,-10,5), (6,4,5), (6,-10,-5), (10,-4,5), (10,-6,-5), (-4,10,-5), (-4,-6,5), (-6,10,5), (-6,-4,-5) (-10,4,-5), (-10,6,5)								
**Quartz**	**(9,-3,2)**	**11.79**	**72.16**	**3.155**	**2.4**	**3.985**	**9.57**	**49.81**	**50.72**
	Equiv. Refl.: (9,-6,-2), (-3,9,-2), (-3,-6,2), (-6,9,2), (-6,-3,-2)								
**Quartz**	**(3,6,-2)**	**11.79**	**72.16**	**3.132**	**2.4**	**3.985**	**9.57**	**49.81**	**50.72**
	Equiv. Refl.: (3,-9,2), (6,3,2), (6,-9,-2), (-9,3,-2), (-9,6,2)								
**Quartz**	**(-8,1,-4)**	**11.918**	**74.22**	**3.13**	**2.5**	**3.499**	**8.76**	**43.74**	**44.61**
**Quartz**	**(1,7,-4)**	**11.918**	**74.22**	**3.129**	**2.5**	**3.499**	**8.76**	**43.74**	**44.61**
	Equiv. Refl.: (1,-8,4), (7,1,4), (7,-8,-4), (-8,7,4)								
**Quartz**	**(8,-4,6)**	**12.217**	**80.56**	**3.067**	**2.6**	**2.06**	**5.42**	**25.75**	**26.32**
**Quartz**	**(8,-4,-6)**	**12.217**	**80.56**	**3.065**	**2.6**	**2.06**	**5.42**	**25.75**	**26.32**
**Quartz**	**(-4,8,6)**	**12.217**	**80.56**	**3.063**	**2.6**	**2.06**	**5.41**	**25.75**	**26.31**
	Equiv. Refl.: (-4,8,-6), (-4,-4,6), (-4,-4,-6)								
**Quartz**	**(1,4,9)**	**12.294**	**83.06**	**2.909**	**2.6**	**1.508**	**3.93**	**18.85**	**19.26**
**Quartz**	**(1,-5,-9)**	**12.294**	**83.06**	**2.908**	**2.6**	**1.508**	**3.92**	**18.85**	**19.26**
**Quartz**	**(4,1,-9)**	**12.294**	**83.06**	**2.906**	**2.6**	**1.508**	**3.92**	**18.85**	**19.26**
**Quartz**	**(-5,1,9)**	**12.294**	**83.06**	**2.905**	**2.6**	**1.508**	**3.92**	**18.85**	**19.26**
	Equiv. Refl.: (-5,4,-9)								
**Quartz**	**(4,-5,9)**	**12.294**	**83.06**	**2.904**	**2.6**	**1.508**	**3.92**	**18.85**	**19.26**
**Quartz**	**(-8,3,-6)**	**12.304**	**83.43**	**2.884**	**2.7**	**1.426**	**3.86**	**17.83**	**18.24**
	Equiv. Refl.: (-8,5,6)								
**Quartz**	**(5,3,6)**	**12.304**	**83.43**	**2.883**	**2.7**	**1.426**	**3.86**	**17.83**	**18.24**
	Equiv. Refl.: (5,-8,-6)								
**Quartz**	**(3,5,-6)**	**12.304**	**83.43**	**2.879**	**2.7**	**1.426**	**3.86**	**17.83**	**18.24**
**Quartz**	**(3,-8,6)**	**12.304**	**83.43**	**2.878**	**2.7**	**1.426**	**3.85**	**17.83**	**18.24**
**Quartz**	**(6,3,-2)**	**11.79**	**72.16**	**2.83**	**2.2**	**3.985**	**8.72**	**49.81**	**50.57**
	Equiv. Refl.: (6,-9,2), (-9,3,2), (-9,6,-2)								
**Quartz**	**(3,6,2)**	**11.79**	**72.16**	**2.829**	**2.2**	**3.985**	**8.71**	**49.81**	**50.57**
	Equiv. Refl.: (3,-9,-2)								
**Quartz**	**(-3,9,-3)**	**12.066**	**76.96**	**2.824**	**2.3**	**2.869**	**6.46**	**35.86**	**36.44**
	Equiv. Refl.: (-6,9,3), (-6,-3,-3)								
**Quartz**	**(2,7,-1)**	**11.981**	**75.32**	**2.823**	**2.2**	**3.243**	**7.27**	**40.54**	**41.19**
	Equiv. Refl.: (2,-9,1), (7,2,1), (7,-9,-1), (-3,-6,3), (-9,2,-1), (-9,7,1)								
**Quartz**	**(9,-3,3)**	**12.066**	**76.96**	**2.822**	**2.3**	**2.869**	**6.46**	**35.86**	**36.44**
	Equiv. Refl.: (9,-3,-2), (9,-6,2), (9,-6,-3), (-6,9,-2), (-6,-3,2)								
**Quartz**	**(-3,9,2)**	**11.79**	**72.16**	**2.821**	**2.2**	**3.985**	**8.71**	**49.81**	**50.57**
	Equiv. Refl.: (-3,-6,-2)								
**Quartz**	**(-2,9,-1)**	**11.981**	**75.32**	**2.794**	**2.2**	**3.243**	**7.27**	**40.54**	**41.19**
	Equiv. Refl.: (-2,-7,1), (-7,9,1), (-7,-2,-1)								
**Quartz**	**(9,-2,1)**	**11.981**	**75.32**	**2.793**	**2.2**	**3.243**	**7.27**	**40.54**	**41.19**
	Equiv. Refl.: (9,-7,-1)								
**Quartz**	**(0,8,-1)**	**11.712**	**71.03**	**2.782**	**2.4**	**4.258**	**10.43**	**53.22**	**54.23**
	Equiv. Refl.: (0,-8,1), (8,0,1), (8,-8,-1), (-8,0,-1), (-8,8,1)								

Ei =	12.385	keV						ΔEg	ΔEt
**Cryst**	**Refl**	**EB**	**ΘB**	**∫ IR dΘ**	**Width**	**Ei cotΘ**	**ΔE**	**2m, 50μm**	**2m, 50μm**
	**(h,k,l)**	**[keV]**	**[°]**	**[μrad]**	**[μrad]**	**[meV/μrad]**	**[meV]**	**[meV]**	**[meV]**
**Sapph**	**(2,7,1)**	**12.323**	**84.29**	**2.771**	**3.1**	**1.239**	**3.8**	**15.49**	**15.95**
	Equiv. Refl.: (2,-9,-1), (7,2,-1), (7,-9,1), (9,-2,-1), (9,-7,1), (-2,9,1), (-2,-7,-1), (-7,9,-1), (-7,-2,1) (-9,2,1), (-9,7,-1)								
**Quartz**	**(-3,1,-10)**	**12.101**	**77.7**	**2.758**	**2.5**	**2.701**	**6.78**	**33.76**	**34.43**
	Equiv. Refl.: (-3,2,10)								
**Quartz**	**(1,2,-10)**	**12.101**	**77.7**	**2.757**	**2.5**	**2.701**	**6.78**	**33.76**	**34.43**
**Quartz**	**(1,-3,10)**	**12.101**	**77.7**	**2.756**	**2.5**	**2.701**	**6.78**	**33.76**	**34.43**
**Quartz**	**(2,1,10)**	**12.101**	**77.7**	**2.755**	**2.5**	**2.701**	**6.77**	**33.76**	**34.43**
	Equiv. Refl.: (2,-3,-10)								
**Quartz**	**(2,6,5)**	**11.97**	**75.12**	**2.696**	**2.4**	**3.29**	**7.92**	**41.13**	**41.88**
	Equiv. Refl.: (2,-8,-5)								
**Quartz**	**(6,2,-5)**	**11.97**	**75.12**	**2.695**	**2.4**	**3.29**	**7.92**	**41.13**	**41.88**
	Equiv. Refl.: (-8,2,5), (-8,6,-5)								
**Quartz**	**(6,-8,5)**	**11.97**	**75.12**	**2.694**	**2.4**	**3.29**	**7.92**	**41.13**	**41.88**
**Quartz**	**(3,-1,10)**	**12.101**	**77.7**	**2.671**	**2.5**	**2.701**	**6.78**	**33.76**	**34.43**
	Equiv. Refl.: (3,-2,-10), (-1,-2,10)								
**Quartz**	**(-1,3,-10)**	**12.101**	**77.7**	**2.67**	**2.5**	**2.701**	**6.78**	**33.76**	**34.43**
**Quartz**	**(-2,3,10)**	**12.101**	**77.7**	**2.669**	**2.5**	**2.701**	**6.77**	**33.76**	**34.43**
	Equiv. Refl.: (-2,-1,-10)								
**Quartz**	**(-1,-4,-9)**	**12.294**	**83.06**	**2.621**	**2.6**	**1.508**	**3.93**	**18.85**	**19.26**
**Quartz**	**(-1,5,9)**	**12.294**	**83.06**	**2.62**	**2.6**	**1.508**	**3.92**	**18.85**	**19.26**
**Quartz**	**(-4,-1,9)**	**12.294**	**83.06**	**2.619**	**2.6**	**1.508**	**3.92**	**18.85**	**19.26**
**Quartz**	**(5,-1,-9)**	**12.294**	**83.06**	**2.618**	**2.6**	**1.508**	**3.92**	**18.85**	**19.26**
	Equiv. Refl.: (5,-4,9), (-4,5,-9)								
**Sapph**	**(1,6,-14)**	**11.915**	**74.17**	**2.585**	**2.4**	**3.512**	**8.54**	**43.9**	**44.72**
	Equiv. Refl.: (1,-7,14), (6,1,14), (6,-7,-14), (7,-1,14), (7,-6,-14), (-1,7,-14), (-1,-6,14), (-6,7,14), (-6,-1,-14) (-7,1,-14), (-7,6,14)								
**Quartz**	**(-2,8,5)**	**11.97**	**75.12**	**2.576**	**2.4**	**3.29**	**7.92**	**41.13**	**41.88**
	Equiv. Refl.: (-2,-6,-5)								
**Quartz**	**(8,-2,-5)**	**11.97**	**75.12**	**2.575**	**2.4**	**3.29**	**7.92**	**41.13**	**41.88**
	Equiv. Refl.: (8,-6,5), (-6,-2,5)								
**Quartz**	**(-6,8,-5)**	**11.97**	**75.12**	**2.574**	**2.4**	**3.29**	**7.92**	**41.13**	**41.88**
**Quartz**	**(-5,8,6)**	**12.304**	**83.43**	**2.484**	**2.7**	**1.426**	**3.86**	**17.83**	**18.24**
**Quartz**	**(8,-3,6)**	**12.304**	**83.43**	**2.483**	**2.7**	**1.426**	**3.86**	**17.83**	**18.24**
	Equiv. Refl.: (8,-5,-6), (-5,-3,-6)								
**Quartz**	**(-3,-5,6)**	**12.304**	**83.43**	**2.481**	**2.7**	**1.426**	**3.86**	**17.83**	**18.24**
**Quartz**	**(-3,8,-6)**	**12.304**	**83.43**	**2.479**	**2.7**	**1.426**	**3.85**	**17.83**	**18.24**
**Quartz**	**(2,5,-7)**	**12.135**	**78.47**	**2.461**	**2.3**	**2.527**	**5.75**	**31.59**	**32.11**
	Equiv. Refl.: (5,2,7), (5,-7,-7)								
**Quartz**	**(2,-7,7)**	**12.135**	**78.47**	**2.46**	**2.3**	**2.527**	**5.75**	**31.59**	**32.11**
	Equiv. Refl.: (-7,2,-7), (-7,5,7)								
**Quartz**	**(3,6,-3)**	**12.066**	**76.96**	**2.456**	**2.3**	**2.869**	**6.46**	**35.86**	**36.44**
	Equiv. Refl.: (3,-9,3), (6,3,3), (6,-9,-3)								
**Quartz**	**(-9,3,-3)**	**12.066**	**76.96**	**2.455**	**2.3**	**2.869**	**6.46**	**35.86**	**36.44**
	Equiv. Refl.: (-9,6,3)								
**Quartz**	**(-2,-5,7)**	**12.135**	**78.47**	**2.45**	**2.3**	**2.527**	**5.75**	**31.59**	**32.11**
	Equiv. Refl.: (-5,7,7), (-5,-2,-7)								
**Quartz**	**(7,-5,-7)**	**12.135**	**78.47**	**2.449**	**2.3**	**2.527**	**5.75**	**31.59**	**32.11**
	Equiv. Refl.: (-2,7,-7)								
**Quartz**	**(7,-2,7)**	**12.135**	**78.47**	**2.448**	**2.3**	**2.527**	**5.75**	**31.59**	**32.11**

Ei = Cryst	12.385 Refl (h,k,l)	keV EB [keV]	ΘB [°]	∫ IR dΘ [µrad]	Width [µrad]	Ei cotΘ [meV/µrad]	ΔE [meV]	ΔEg 2m, 50µm [meV]	ΔEt 2m, 50µm [meV]
**Quartz**	**(4,0,9)**	**11.855**	**73.17**	**2.349**	**2**	**3.746**	**7.62**	**46.82**	**47.44**
**Quartz**	**(4,-4,-9)**	**11.855**	**73.17**	**2.348**	**2**	**3.746**	**7.62**	**46.82**	**47.44**
	Equiv. Refl.: (-4,0,-9), (-4,4,9)								
**Quartz**	**(0,4,-9)**	**11.855**	**73.17**	**2.345**	**2**	**3.746**	**7.61**	**46.82**	**47.44**
	Equiv. Refl.: (0,-4,9)								
**Sapph**	**(2,6,-10)**	**11.852**	**73.13**	**2.344**	**2.2**	**3.756**	**8.29**	**46.95**	**47.67**
	Equiv. Refl.: (2,-8,10), (6,2,10), (6,-8,-10), (8,-2,10), (8,-6,-10), (-2,8,-10), (-2,-6,10), (-6,8,10), (-6,-2,-10) (-8,2,-10), (-8,6,10)								
**Sapph**	**(2,6,11)**	**12.052**	**76.69**	**2.311**	**2.2**	**2.931**	**6.56**	**36.63**	**37.21**
	Equiv. Refl.: (2,-8,-11), (6,2,-11), (6,-8,11), (8,-2,-11), (8,-6,11), (-2,8,11), (-2,-6,-11), (-6,8,-11), (-6,-2,11) (-8,2,11), (-8,6,-11)								
**Quartz**	**(9,-3,-3)**	**12.066**	**76.96**	**2.306**	**2**	**2.869**	**5.84**	**35.86**	**36.33**
	Equiv. Refl.: (9,-6,3), (-6,9,-3), (-6,-3,3)								
**Quartz**	**(-3,9,3)**	**12.066**	**76.96**	**2.305**	**2**	**2.869**	**5.83**	**35.86**	**36.33**
	Equiv. Refl.: (-3,-6,-3)								
**Sapph**	**(2,7,-2)**	**12.351**	**85.76**	**2.295**	**3.1**	**0.917**	**2.81**	**11.47**	**11.81**
	Equiv. Refl.: (2,-9,2), (7,2,2), (7,-9,-2), (9,-2,2), (9,-7,-2), (-2,9,-2), (-2,-7,2), (-7,9,2), (-7,-2,-2) (-9,2,-2), (-9,7,2)								
**LiNbO3**	**(3,7,-1)**	**12.367**	**86.91**	**2.283**	**5.4**	**0.668**	**3.62**	**8.35**	**9.1**
	Equiv. Refl.: (3,-10,1), (7,3,1), (7,-10,-1), (10,-3,1), (10,-7,-1), (-3,10,-1), (-3,-7,1), (-7,10,1), (-7,-3,-1) (-10,3,-1), (-10,7,1)								
**Quartz**	**(3,3,8)**	**11.896**	**73.85**	**1.92**	**2**	**3.587**	**7.09**	**44.84**	**45.39**
	Equiv. Refl.: (3,-6,-8)								
**Quartz**	**(-6,3,8)**	**11.896**	**73.85**	**1.919**	**2**	**3.587**	**7.08**	**44.84**	**45.39**
	Equiv. Refl.: (-6,3,-8)								
**Quartz**	**(3,3,-8)**	**11.896**	**73.85**	**1.918**	**2**	**3.587**	**7.08**	**44.84**	**45.39**
	Equiv. Refl.: (3,-6,8)								
**Sapph**	**(3,6,6)**	**12.28**	**82.52**	**1.873**	**2.3**	**1.626**	**3.74**	**20.33**	**20.67**
	Equiv. Refl.: (3,6,-6), (3,-9,6), (3,-9,-6), (6,3,6), (6,3,-6), (6,-9,6), (6,-9,-6), (9,-3,6), (9,-3,-6) (9,-6,6), (9,-6,-6), (-3,9,6), (-3,9,-6), (-3,-6,6), (-3,-6,-6), (-6,9,6), (-6,9,-6), (-6,-3,6) (-6,-3,-6), (-9,3,6), (-9,3,-6), (-9,6,6), (-9,6,-6)								
**Sapph**	**(4,5,5)**	**11.99**	**75.49**	**1.86**	**2.1**	**3.206**	**6.61**	**40.08**	**40.62**
	Equiv. Refl.: (4,-9,-5), (5,4,-5), (5,-9,5), (9,-4,-5), (9,-5,5), (-4,9,5), (-4,-5,-5), (-5,9,-5), (-5,-4,5) (-9,4,5), (-9,5,-5)								
**Quartz**	**(-3,6,8)**	**11.896**	**73.85**	**1.806**	**2**	**3.587**	**7.09**	**44.84**	**45.39**
	Equiv. Refl.: (-3,-3,-8)								
**Quartz**	**(6,-3,8)**	**11.896**	**73.85**	**1.805**	**2**	**3.587**	**7.08**	**44.84**	**45.39**
**Quartz**	**(6,-3,-8)**	**11.896**	**73.85**	**1.804**	**2**	**3.587**	**7.08**	**44.84**	**45.39**
**Quartz**	**(-3,6,-8)**	**11.896**	**73.85**	**1.803**	**2**	**3.587**	**7.08**	**44.84**	**45.39**
	Equiv. Refl.: (-3,-3,8)								
**Quartz**	**(-9,3,3)**	**12.066**	**76.96**	**1.795**	**2**	**2.869**	**5.84**	**35.86**	**36.33**
	Equiv. Refl.: (-9,6,-3)								
**Quartz**	**(3,6,3)**	**12.066**	**76.96**	**1.794**	**2**	**2.869**	**5.83**	**35.86**	**36.33**
	Equiv. Refl.: (3,-9,-3), (6,3,-3), (6,-9,3)								
**Quartz**	**(7,1,-4)**	**11.918**	**74.22**	**1.783**	**1.9**	**3.499**	**6.55**	**43.74**	**44.22**
	Equiv. Refl.: (7,-8,4)								
**Quartz**	**(1,7,4)**	**11.918**	**74.22**	**1.782**	**1.9**	**3.499**	**6.55**	**43.74**	**44.22**
	Equiv. Refl.: (1,-8,-4), (-8,1,4), (-8,7,-4)								
**Quartz**	**(-1,7,-6)**	**11.775**	**71.94**	**1.744**	**1.8**	**4.039**	**7.2**	**50.49**	**51**
	Equiv. Refl.: (-6,7,6), (-6,-1,-6)								

**Ei =**	**12.385**	**keV**						**ΔEg**	**ΔEt**
**Cryst**	**Refl**	**EB**	**ΘB**	**∫ IR dΘ**	**Width**	**Ei cotΘ**	**ΔE**	**2m, 50µm**	**2m, 50µm**
	**(h,k,l)**	**[keV]**	**[°]**	**[µrad]**	**[µrad]**	**[meV/µrad]**	**[meV]**	**[meV]**	**[meV]**
**Quartz**	**(-1,-6,6)**	**11.775**	**71.94**	**1.743**	**1.8**	**4.039**	**7.2**	**50.49**	**51**
**Quartz**	**(7,-1,6)**	**11.775**	**71.94**	**1.742**	**1.8**	**4.039**	**7.2**	**50.49**	**51**
	Equiv. Refl.: (7,-6,-6)								
**Quartz**	**(7,-3,-7)**	**11.959**	**74.92**	**1.735**	**1.8**	**3.336**	**6.14**	**41.7**	**42.15**
	Equiv. Refl.: (-3,7,7), (-3,-4,-7), (-4,7,-7), (-4,-3,7)								
**Sapph**	**(1,7,9)**	**12.143**	**78.66**	**1.734**	**2**	**2.483**	**4.99**	**31.04**	**31.44**
	Equiv. Refl.: (1,7,-9), (1,-8,9), (1,-8,-9), (7,1,9), (7,1,-9), (7,-8,9), (7,-8,-9), (8,-1,9), (8,-1,-9) (8,-7,9), (8,-7,-9), (-1,8,9), (-1,8,-9), (-1,-7,9), (-1,-7,-9), (-7,8,9), (-7,8,-9), (-7,-1,9) (-7,-1,-9), (-8,1,9), (-8,1,-9), (-8,7,9), (-8,7,-9), (7,-4,7)								
**Quartz**	**(-7,8,-4)**	**11.918**	**74.22**	**1.715**	**1.9**	**3.499**	**6.55**	**43.74**	**44.22**
	Equiv. Refl.: (-7,-1,4)								
**Quartz**	**(8,-1,-4)**	**11.918**	**74.22**	**1.714**	**1.9**	**3.499**	**6.55**	**43.74**	**44.22**
	Equiv. Refl.: (8,-7,4), (-1,8,4), (-1,-7,-4)								
**Quartz**	**(3,4,7)**	**11.959**	**74.92**	**1.677**	**1.8**	**3.336**	**6.14**	**41.7**	**42.15**
	Equiv. Refl.: (3,-7,-7), (4,3,-7)								
**Quartz**	**(4,-7,7)**	**11.959**	**74.92**	**1.676**	**1.8**	**3.336**	**6.14**	**41.7**	**42.15**
	Equiv. Refl.: (-7,3,7)								
**Quartz**	**(-7,4,-7)**	**11.959**	**74.92**	**1.675**	**1.8**	**3.336**	**6.13**	**41.7**	**42.15**
**Quartz**	**(2,4,-8)**	**11.985**	**75.4**	**1.627**	**1.7**	**3.226**	**5.51**	**40.33**	**40.7**
**Quartz**	**(2,-6,8)**	**11.985**	**75.4**	**1.626**	**1.7**	**3.226**	**5.51**	**40.33**	**40.7**
	Equiv. Refl.: (-6,2,-8), (-6,4,8)								
**Quartz**	**(4,2,8)**	**11.985**	**75.4**	**1.624**	**1.7**	**3.226**	**5.5**	**40.33**	**40.7**
	Equiv. Refl.: (4,-6,-8)								
**Quartz**	**(1,-7,6)**	**11.775**	**71.94**	**1.619**	**1.8**	**4.039**	**7.2**	**50.49**	**51**
	Equiv. Refl.: (1,-7,-6), (6,1,6), (6,-7,-6), (-7,6,-6)								
**Quartz**	**(1,6,6)**	**11.775**	**71.94**	**1.618**	**1.7**	**4.039**	**6.67**	**50.49**	**50.92**
	Equiv. Refl.: (1,6,-6), (-7,1,6)								
**Quartz**	**(6,1,-6)**	**11.775**	**71.94**	**1.617**	**1.6**	**4.039**	**6.66**	**50.49**	**50.92**
	Equiv. Refl.: (-7,1,-6), (-7,6,6)								
**Quartz**	**(6,-7,6)**	**11.775**	**71.94**	**1.616**	**1.6**	**4.039**	**6.66**	**50.49**	**50.92**
**LiNbO3**	**(4,5,-13)**	**12.318**	**84.04**	**1.608**	**3.4**	**1.293**	**4.35**	**16.16**	**16.73**
	Equiv. Refl.: (4,-9,13), (5,4,13), (5,-9,-13), (9,-4,13), (9,-5,-13), (-4,9,-13), (-4,-5,13), (-5,9,13), (-5,-4,-13) (-9,4,-13), (-9,5,13)								
**LiNbO3**	**(1,6,-17)**	**11.871**	**73.44**	**1.602**	**2.4**	**3.682**	**8.81**	**46.03**	**46.87**
	Equiv. Refl.: (1,-7,17), (6,1,17), (6,-7,-17), (7,-1,17), (7,-6,-17), (-1,7,-17), (-1,-6,17), (-6,7,17), (-6,-1,-17) (-7,1,-17), (-7,6,17)								
**LiNbO3**	**(4,6,1)**	**12.13**	**78.36**	**1.596**	**2.6**	**2.552**	**6.74**	**31.9**	**32.6**
	Equiv. Refl.: (4,-10,-1), (6,4,-1), (6,-10,1), (10,-4,-1), (10,-6,1), (-4,10,1), (-4,-6,-1), (-6,10,-1), (-6,-4,1) (-10,4,1), (-10,6,-1)								
**Sapph**	**(4,5,-1)**	**11.76**	**71.71**	**1.569**	**1.7**	**4.093**	**7.09**	**51.16**	**51.65**
	Equiv. Refl.: (4,-9,1), (5,4,1), (5,-9,-1), (9,-4,1), (9,-5,-1), (-4,9,-1), (-4,-5,1), (-5,9,1), (-5,-4,-1) (-9,4,-1), (-9,5,1)								
**Sapph**	**(0,4,-22)**	**12.103**	**77.74**	**1.524**	**1.8**	**2.691**	**4.8**	**33.64**	**33.98**
	Equiv. Refl.: (0,-4,22), (4,0,22), (4,-4,-22), (-4,0,-22), (-4,4,22)								
**Quartz**	**(0,8,0)**	**11.656**	**70.24**	**1.507**	**1.6**	**4.449**	**7.14**	**55.61**	**56.06**
	Equiv. Refl.: (0,-8,0), (8,0,0), (8,-8,0), (-8,0,0), (-8,8,0)								
**Sapph**	**(4,5,8)**	**12.355**	**85.99**	**1.504**	**2.3**	**0.867**	**1.98**	**10.84**	**11.02**
	Equiv. Refl.: (4,-9,-8), (5,4,-8), (5,-9,8), (9,-4,-8), (9,-5,8), (-4,9,8), (-4,-5,-8), (-5,9,-8), (-5,-4,8) (-9,4,8), (-9,5,-8)								
**Quartz**	**(-2,-4,8)**	**11.985**	**75.4**	**1.499**	**1.7**	**3.226**	**5.51**	**40.33**	**40.7**

Ei =	12.385	keV						ΔEg	ΔEt
**Cryst**	**Refl**	**EB**	**ΘB**	**∫ IR dΘ**	**Width**	**Ei cotΘ**	**ΔE**	**2m, 50µm**	**2m, 50µm**
	**(h,k,l)**	**[keV]**	**[°]**	**[µrad]**	**[µrad]**	**[meV/µrad]**	**[meV]**	**[meV]**	**[meV]**
**Quartz**	**(6,-2,8)**	**11.985**	**75.4**	**1.498**	**1.7**	**3.226**	**5.51**	**40.33**	**40.7**
	Equiv. Refl.: (6,-4,-8), (-2,6,-8)								
**Quartz**	**(-4,6,8)**	**11.985**	**75.4**	**1.497**	**1.7**	**3.226**	**5.5**	**40.33**	**40.7**
	Equiv. Refl.: (-4,-2,-8)								
**Quartz**	**(7,-1,-6)**	**11.775**	**71.94**	**1.467**	**1.7**	**4.039**	**6.67**	**50.49**	**50.92**
	Equiv. Refl.: (7,-6,6), (-1,7,6), (-1,-6,-6)								
**Quartz**	**(-6,7,-6)**	**11.775**	**71.94**	**1.465**	**1.6**	**4.039**	**6.66**	**50.49**	**50.92**
	Equiv. Refl.: (-6,-1,6)								
**Sapph**	**(2,5,15)**	**11.812**	**72.5**	**1.451**	**1.6**	**3.904**	**6.35**	**48.8**	**49.21**
	Equiv. Refl.: (2,5,-15), (2,-7,15), (2,-7,-15), (5,2,15), (5,2,-15), (5,-7,15), (5,-7,-15), (7,-2,15), (7,-2,-15) (7,-5,15), (7,-5,-15), (-2,7,15), (-2,7,-15), (-2,-5,15), (-2,-5,-15), (-5,7,15), (-5,7,-15), (-5,-2,15) (-5,-2,-15), (-7,2,15), (-7,2,-15), (-7,5,15), (-7,5,-15)								
**LiNbO3**	**(1,3,25)**	**12.252**	**81.6**	**1.386**	**2.7**	**1.829**	**4.93**	**22.86**	**23.39**
	Equiv. Refl.: (1,-4,-25), (3,1,-25), (3,-4,25), (4,-1,-25), (4,-3,25), (-1,4,25), (-1,-3,-25), (-3,4,-25), (-3,-1,25) (-4,1,25), (-4,3,-25)								
**LiNbO3**	**(2,7,7)**	**11.804**	**72.38**	**1.378**	**2.1**	**3.933**	**8.33**	**49.16**	**49.86**
	Equiv. Refl.: (2,-9,-7), (7,2,-7), (7,-9,7), (9,-2,-7), (9,-7,7), (-2,9,7), (-2,-7,-7), (-7,9,-7), (-7,-2,7) (-9,2,7), (-9,7,-7)								
**Quartz**	**(2,7,2)**	**12.145**	**78.69**	**1.372**	**1.6**	**2.477**	**3.87**	**30.96**	**31.2**
	Equiv. Refl.: (2,-9,-2), (7,2,-2), (7,-9,2), (-9,2,2), (-9,7,-2)								
**Quartz**	**(0,4,9)**	**11.855**	**73.17**	**1.351**	**1.5**	**3.746**	**5.54**	**46.82**	**47.15**
	Equiv. Refl.: (0,-4,-9)								
**Quartz**	**(4,-4,9)**	**11.855**	**73.17**	**1.348**	**1.5**	**3.746**	**5.53**	**46.82**	**47.15**
	Equiv. Refl.: (-4,0,9), (-4,4,-9)								
**Quartz**	**(4,0,-9)**	**11.855**	**73.17**	**1.347**	**1.5**	**3.746**	**5.53**	**46.82**	**47.15**
**Sapph**	**(3,5,13)**	**12.223**	**80.72**	**1.335**	**1.7**	**2.023**	**3.42**	**25.29**	**25.52**
	Equiv. Refl.: (3,-8,-13), (5,3,-13), (5,-8,13), (8,-3,-13), (8,-5,13), (-3,8,13), (-3,-5,-13), (-5,8,-13), (-5,-3,13) (-8,3,13), (-8,5,-13)								
**Quartz**	**(9,-2,-2)**	**12.145**	**78.69**	**1.33**	**1.6**	**2.477**	**3.87**	**30.96**	**31.2**
	Equiv. Refl.: (-2,9,2), (-2,-7,-2), (-7,9,-2), (-7,-2,2)								
**Quartz**	**(9,-7,2)**	**12.145**	**78.69**	**1.329**	**1.6**	**2.477**	**3.87**	**30.96**	**31.2**
**Sapph**	**(1,5,17)**	**11.662**	**70.32**	**1.308**	**1.5**	**4.429**	**6.5**	**55.36**	**55.74**
	Equiv. Refl.: (1,-6,-17), (5,1,-17), (5,-6,17), (6,-1,-17), (6,-5,17), (-1,6,17), (-1,-5,-17), (-5,6,-17), (-5,-1,17) (-6,1,17), (-6,5,-17)								
**Sapph**	**(2,4,19)**	**12.067**	**76.99**	**1.248**	**1.5**	**2.862**	**4.29**	**35.78**	**36.04**
	Equiv. Refl.: (2,-6,-19), (4,2,-19), (4,-6,19), (6,-2,-19), (6,-4,19), (-2,6,19), (-2,-4,-19), (-4,6,-19), (-4,-2,19) (-6,2,19), (-6,4,-19)								
**Quartz**	**(4,-9,-3)**	**11.888**	**73.72**	**1.206**	**1.3**	**3.617**	**4.81**	**45.22**	**45.47**
	Equiv. Refl.: (5,4,-3), (5,-9,3), (-9,4,3), (-9,5,-3)								
**Quartz**	**(4,5,3)**	**11.888**	**73.72**	**1.205**	**1.3**	**3.617**	**4.81**	**45.22**	**45.47**
**Quartz**	**(9,-4,-3)**	**11.888**	**73.72**	**1.172**	**1.3**	**3.617**	**4.81**	**45.22**	**45.47**
	Equiv. Refl.: (9,-5,3), (-4,9,3), (-4,-5,-3), (-5,9,-3), (-5,-4,3)								
**Quartz**	**(8,0,3)**	**12.153**	**78.9**	**1.156**	**1.4**	**2.43**	**3.35**	**30.38**	**30.56**
	Equiv. Refl.: (8,-8,-3), (-8,0,-3), (-8,8,3)								
**Quartz**	**(0,8,-3)**	**12.153**	**78.9**	**1.154**	**1.4**	**2.43**	**3.34**	**30.38**	**30.56**
	Equiv. Refl.: (0,-8,3)								
**Quartz**	**(2,0,10)**	**11.835**	**72.85**	**1.148**	**1.3**	**3.821**	**4.91**	**47.76**	**48.02**
	Equiv. Refl.: (2,-2,-10), (-2,0,-10), (-2,2,10)								
**Quartz**	**(0,2,-10)**	**11.835**	**72.85**	**1.145**	**1.3**	**3.821**	**4.89**	**47.76**	**48.01**
	Equiv. Refl.: (0,-2,10)								

Ei =	12.385	keV						ΔEg	ΔEt
**Cryst**	**Refl**	**EB**	**ΘB**	**∫ IR dΘ**	**Width**	**Ei cotΘ**	**ΔE**	**2m, 50µm**	**2m, 50µm**
	**(h,k,l)**	**[keV]**	**[°]**	**[µrad]**	**[µrad]**	**[meV/µrad]**	**[meV]**	**[meV]**	**[meV]**
**Quartz**	**(3,5,5)**	**11.701**	**70.87**	**1.091**	**1.2**	**4.297**	**5.16**	**53.71**	**53.96**
	Equiv. Refl.: (3,-8,-5), (-8,3,5), (-8,5,-5)								
**Quartz**	**(5,3,-5)**	**11.701**	**70.87**	**1.09**	**1.2**	**4.297**	**5.16**	**53.71**	**53.96**
	Equiv. Refl.: (5,-8,5)								
**Quartz**	**(8,-3,-5)**	**11.701**	**70.87**	**1.068**	**1.2**	**4.297**	**5.16**	**53.71**	**53.96**
	Equiv. Refl.: (8,-5,5), (-3,8,5), (-3,-5,-5)								
**Quartz**	**(-5,8,-5)**	**11.701**	**70.87**	**1.067**	**1.2**	**4.297**	**5.16**	**53.71**	**53.96**
	Equiv. Refl.: (-5,-3,5)								
**Quartz**	**(0,3,-10)**	**12.275**	**82.35**	**1.064**	**1.4**	**1.663**	**2.31**	**20.79**	**20.92**
	Equiv. Refl.: (0,-3,10)								
**Quartz**	**(-3,3,10)**	**12.275**	**82.35**	**1.057**	**1.4**	**1.663**	**2.3**	**20.79**	**20.92**
**Quartz**	**(3,-3,-10)**	**12.275**	**82.35**	**1.056**	**1.4**	**1.663**	**2.3**	**20.79**	**20.92**
	Equiv. Refl.: (-3,0,-10)								
**Quartz**	**(3,0,10)**	**12.275**	**82.35**	**1.055**	**1.4**	**1.663**	**2.3**	**20.79**	**20.92**
**Quartz**	**(-5,1,-9)**	**12.294**	**83.06**	**0.937**	**1.3**	**1.508**	**1.93**	**18.85**	**18.95**
	Equiv. Refl.: (-5,4,9)								
**Quartz**	**(1,4,-9)**	**12.294**	**83.06**	**0.936**	**1.3**	**1.508**	**1.92**	**18.85**	**18.95**
**Quartz**	**(1,-5,9)**	**12.294**	**83.06**	**0.935**	**1.3**	**1.508**	**1.92**	**18.85**	**18.95**
**Quartz**	**(4,1,9)**	**12.294**	**83.06**	**0.933**	**1.3**	**1.508**	**1.92**	**18.85**	**18.95**
**Quartz**	**(4,-5,-9)**	**12.294**	**83.06**	**0.932**	**1.3**	**1.508**	**1.92**	**18.85**	**18.95**
**Quartz**	**(-5,9,3)**	**11.888**	**73.72**	**0.927**	**1.1**	**3.617**	**3.82**	**45.22**	**45.38**
	Equiv. Refl.: (-5,-4,-3)								
**Quartz**	**(9,-4,3)**	**11.888**	**73.72**	**0.926**	**1.1**	**3.617**	**3.81**	**45.22**	**45.38**
	Equiv. Refl.: (9,-5,-3)								
**Quartz**	**(-4,9,-3)**	**11.888**	**73.72**	**0.925**	**1.1**	**3.617**	**3.81**	**45.22**	**45.38**
	Equiv. Refl.: (-4,-5,3)								
**Quartz**	**(5,-1,9)**	**12.294**	**83.06**	**0.911**	**1.3**	**1.508**	**1.93**	**18.85**	**18.95**
	Equiv. Refl.: (5,-4,-9)								
**Quartz**	**(-1,-4,9)**	**12.294**	**83.06**	**0.91**	**1.3**	**1.508**	**1.92**	**18.85**	**18.95**
**Quartz**	**(-1,5,-9)**	**12.294**	**83.06**	**0.909**	**1.3**	**1.508**	**1.92**	**18.85**	**18.95**
**Quartz**	**(-4,-1,-9)**	**12.294**	**83.06**	**0.906**	**1.3**	**1.508**	**1.92**	**18.85**	**18.95**
**Quartz**	**(-4,5,9)**	**12.294**	**83.06**	**0.905**	**1.3**	**1.508**	**1.92**	**18.85**	**18.95**
**Quartz**	**(5,4,3)**	**11.888**	**73.72**	**0.88**	**1.1**	**3.617**	**3.82**	**45.22**	**45.38**
	Equiv. Refl.: (5,-9,-3)								
**Quartz**	**(-9,4,-3)**	**11.888**	**73.72**	**0.879**	**1.1**	**3.617**	**3.81**	**45.22**	**45.38**
	Equiv. Refl.: (-9,5,3)								
**Quartz**	**(4,5,-3)**	**11.888**	**73.72**	**0.878**	**1.1**	**3.617**	**3.81**	**45.22**	**45.38**
	Equiv. Refl.: (4,-9,3)								
**Quartz**	**(0,8,2)**	**11.879**	**73.57**	**0.85**	**1**	**3.651**	**3.65**	**45.64**	**45.79**
	Equiv. Refl.: (0,-8,-2), (7,0,-5), (7,-7,5), (-7,0,5), (-7,7,-5)								
**Quartz**	**(0,7,5)**	**11.701**	**70.87**	**0.849**	**1**	**4.297**	**4.21**	**53.71**	**53.88**
	Equiv. Refl.: (0,-7,-5), (8,0,-2), (8,-8,2), (-8,0,2), (-8,8,-2)								
**LiNbO3**	**(3,4,-19)**	**11.989**	**75.46**	**0.834**	**1.6**	**3.211**	**5.19**	**40.14**	**40.47**
	Equiv. Refl.: (3,-7,19), (4,3,19), (4,-7,-19), (7,-3,19), (7,-4,-19), (-3,7,-19), (-3,-4,19), (-4,7,19), (-4,-3,-19) (-7,3,-19), (-7,4,19)								
**Quartz**	**(-5,8,5)**	**11.701**	**70.87**	**0.821**	**0.9**	**4.297**	**4.01**	**53.71**	**53.86**
	Equiv. Refl.: (-5,-3,-5)								
**Quartz**	**(-3,8,-5)**	**11.701**	**70.87**	**0.82**	**0.9**	**4.297**	**4**	**53.71**	**53.86**
	Equiv. Refl.: (-3,-5,5)								

Ei =	12.385	keV						ΔEg	ΔEt
Cryst	Refl	EB	ΘB	∫ IR dΘ	Width	Ei cotΘ	ΔE	2m, 50µm	2m, 50µm
	(h,k,l)	[keV]	[°]	[µrad]	[µrad]	[meV/µrad]	[meV]	[meV]	[meV]
**Quartz**	**(8,-3,5)**	**11.701**	**70.87**	**0.819**	**0.9**	**4.297**	**4**	**53.71**	**53.86**
	Equiv. Refl.: (8,-5,-5)								
**Quartz**	**(8,-2,5)**	**11.97**	**75.12**	**0.81**	**0.9**	**3.29**	**2.99**	**41.13**	**41.23**
	Equiv. Refl.: (8,-6,-5), (-2,8,-5), (-2,-6,5)								
**Quartz**	**(-6,8,5)**	**11.97**	**75.12**	**0.809**	**0.9**	**3.29**	**2.99**	**41.13**	**41.23**
	Equiv. Refl.: (-6,-2,-5)								
**Quartz**	**(5,3,5)**	**11.701**	**70.87**	**0.78**	**0.9**	**4.297**	**4.01**	**53.71**	**53.86**
	Equiv. Refl.: (5,-8,-5)								
**Quartz**	**(3,5,-5)**	**11.701**	**70.87**	**0.779**	**0.9**	**4.297**	**4**	**53.71**	**53.86**
	Equiv. Refl.: (3,-8,5)								
**Quartz**	**(-8,3,-5)**	**11.701**	**70.87**	**0.778**	**0.9**	**4.297**	**4**	**53.71**	**53.86**
	Equiv. Refl.: (-8,5,5)								
**Quartz**	**(-5,-1,-8)**	**12.248**	**81.46**	**0.777**	**1**	**1.859**	**1.86**	**23.24**	**23.31**
**Quartz**	**(-5,6,8)**	**12.248**	**81.46**	**0.776**	**1**	**1.859**	**1.86**	**23.24**	**23.31**
**Sapph**	**(3,5,-11)**	**11.767**	**71.83**	**0.774**	**1**	**4.066**	**3.92**	**50.82**	**50.97**
	Equiv. Refl.: (3,-8,11), (5,3,11), (5,-8,-11), (8,-3,11), (8,-5,-11), (-3,8,-11), (-3,-5,11), (-5,8,11), (-5,-3,-11) (-8,3,-11), (-8,5,11)								
**Quartz**	**(6,-5,-8)**	**12.248**	**81.46**	**0.771**	**1**	**1.859**	**1.85**	**23.24**	**23.31**
	Equiv. Refl.: (-1,6,-8), (-1,-5,8)								
**Quartz**	**(6,-1,8)**	**12.248**	**81.46**	**0.77**	**1**	**1.859**	**1.85**	**23.24**	**23.31**
**Sapph**	**(0,3,24)**	**12.312**	**83.78**	**0.691**	**1.1**	**1.35**	**1.55**	**16.88**	**16.95**
	Equiv. Refl.: (0,3,-24), (0,-3,24), (0,-3,-24), (3,0,24), (3,0,-24), (3,-3,24), (3,-3,-24), (-3,0,24), (-3,0,-24) (-3,3,24), (-3,3,-24)								
**LiNbO3**	**(4,5,11)**	**11.922**	**74.29**	**0.677**	**1.4**	**3.485**	**4.82**	**43.56**	**43.82**
	Equiv. Refl.: (4,-9,-11), (5,4,-11), (5,-9,11), (9,-4,-11), (9,-5,11), (-4,9,11), (-4,-5,-11), (-5,9,-11), (-5,-4,11) (-9,4,11), (-9,5,-11)								
**Quartz**	**(2,-8,5)**	**11.97**	**75.12**	**0.673**	**0.9**	**3.29**	**2.99**	**41.13**	**41.23**
**Quartz**	**(2,6,-5)**	**11.97**	**75.12**	**0.672**	**0.9**	**3.29**	**2.99**	**41.13**	**41.23**
	Equiv. Refl.: (-8,2,-5), (-8,6,5)								
**Quartz**	**(6,2,5)**	**11.97**	**75.12**	**0.671**	**0.9**	**3.29**	**2.99**	**41.13**	**41.23**
	Equiv. Refl.: (6,-8,-5)								
**Quartz**	**(5,1,8)**	**12.248**	**81.46**	**0.654**	**1**	**1.859**	**1.86**	**23.24**	**23.31**
**Quartz**	**(5,-6,-8)**	**12.248**	**81.46**	**0.653**	**1**	**1.859**	**1.86**	**23.24**	**23.31**
**Quartz**	**(1,5,-8)**	**12.248**	**81.46**	**0.648**	**1**	**1.859**	**1.85**	**23.24**	**23.31**
	Equiv. Refl.: (1,-6,8)								
**Quartz**	**(-6,1,-8)**	**12.248**	**81.46**	**0.647**	**1**	**1.859**	**1.85**	**23.24**	**23.31**
	Equiv. Refl.: (-6,5,8)								
**Quartz**	**(-6,2,8)**	**11.985**	**75.4**	**0.633**	**0.8**	**3.226**	**2.51**	**40.33**	**40.41**
	Equiv. Refl.: (-6,4,-8)								
**Quartz**	**(2,4,8)**	**11.985**	**75.4**	**0.632**	**0.8**	**3.226**	**2.51**	**40.33**	**40.41**
	Equiv. Refl.: (2,-6,-8), (4,2,-8), (4,-6,8)								
**Sapph**	**(1,3,-23)**	**12.245**	**81.37**	**0.618**	**1**	**1.88**	**1.84**	**23.5**	**23.57**
	Equiv. Refl.: (1,-4,23), (3,1,23), (3,-4,-23), (4,-1,23), (4,-3,-23), (-1,4,-23), (-1,-3,23), (-3,4,23), (-3,-1,-23) (-4,1,-23), (-4,3,23)								
**Quartz**	**(6,-6,-7)**	**11.87**	**73.41**	**0.604**	**0.8**	**3.689**	**2.79**	**46.11**	**46.2**
**Quartz**	**(0,6,-7)**	**11.87**	**73.41**	**0.603**	**0.8**	**3.689**	**2.79**	**46.11**	**46.2**
	Equiv. Refl.: (0,-6,7), (6,0,7), (-6,0,-7), (-6,6,7)								
**Quartz**	**(6,-2,-8)**	**11.985**	**75.4**	**0.575**	**0.8**	**3.226**	**2.51**	**40.33**	**40.41**
	Equiv. Refl.: (6,-4,8), (-4,-2,8)								
**Quartz**	**(-2,6,8)**	**11.985**	**75.4**	**0.574**	**0.8**	**3.226**	**2.51**	**40.33**	**40.41**
	Equiv. Refl.: (-2,-4,-8), (-4,6,-8)								

Ei =	12.385	keV						ΔEg	ΔEt
Cryst	Refl	EB	ΘB	∫ IR dΘ	Width	Ei cotΘ	ΔE	2m, 50μm	2m, 50μm
	(h,k,l)	[keV]	[°]	[μrad]	[μrad]	[meV/μrad]	[meV]	[meV]	[meV]
**Quartz**	**(0,8,1)**	**11.712**	**71.03**	**0.56**	**0.7**	**4.258**	**2.98**	**53.22**	**53.31**
	Equiv. Refl.: (0,-8,-1), (8,0,-1), (8,-8,1), (-8,0,1), (-8,8,-1)								
**Sapph**	**(1,2,23)**	**11.677**	**70.53**	**0.54**	**0.7**	**4.378**	**3.15**	**54.72**	**54.81**
	Equiv. Refl.: (1,-3,-23), (2,1,-23), (2,-3,23), (3,-1,-23), (3,-2,23), (-1,3,23), (-1,-2,-23), (-2,3,-23), (-2,-1,23) (-3,1,23), (-3,2,-23)								
**LiNbO3**	**(3,5,-17)**	**12.35**	**85.71**	**0.522**	**2.1**	**0.929**	**1.95**	**11.61**	**11.78**
	Equiv. Refl.: (3,-8,17), (5,3,17), (5,-8,-17), (8,-3,17), (8,-5,-17), (-3,8,-17), (-3,-5,17), (-5,8,17), (-5,-3,-17) (-8,3,-17), (-8,5,17)								
**Sapph**	**(1,3,22)**	**11.819**	**72.61**	**0.52**	**0.7**	**3.88**	**2.78**	**48.5**	**48.58**
	Equiv. Refl.: (1,-4,-22), (3,1,-22), (3,-4,22), (4,-1,-22), (4,-3,22), (-1,4,22), (-1,-3,-22), (-3,4,-22), (-3,-1,22) (-4,1,22), (-4,3,-22)								
**Quartz**	**(5,0,8)**	**11.716**	**71.09**	**0.517**	**0.7**	**4.244**	**2.77**	**53.05**	**53.12**
	Equiv. Refl.: (5,-5,-8), (-5,0,-8), (-5,5,8)								
**Quartz**	**(0,5,-8)**	**11.716**	**71.09**	**0.516**	**0.7**	**4.244**	**2.76**	**53.05**	**53.12**
	Equiv. Refl.: (0,-5,8)								
**Quartz**	**(1,1,10)**	**11.745**	**71.49**	**0.489**	**0.6**	**4.146**	**2.38**	**51.82**	**51.88**
	Equiv. Refl.: (1,1,-10), (1,-2,10), (1,-2,-10)								
**Quartz**	**(-2,1,10)**	**11.745**	**71.49**	**0.487**	**0.6**	**4.146**	**2.37**	**51.82**	**51.87**
	Equiv. Refl.: (-2,1,-10)								
**Sapph**	**(1,6,13)**	**11.654**	**70.22**	**0.466**	**0.6**	**4.454**	**2.86**	**55.68**	**55.75**
	Equiv. Refl.: (1,-7,-13), (6,1,-13), (6,-7,13), (7,-1,-13), (7,-6,13), (-1,7,13), (-1,-6,-13), (-6,7,-13), (-6,-1,13) (-7,1,13), (-7,6,-13)								
**LiNbO3**	**(1,2,-25)**	**11.769**	**71.86**	**0.445**	**1**	**4.059**	**4.16**	**50.73**	**50.9**
	Equiv. Refl.: (1,-3,25), (2,1,25), (2,-3,-25), (3,-1,25), (3,-2,-25), (-1,3,-25), (-1,-2,25), (-2,3,25), (-2,-1,-25) (-3,1,-25), (-3,2,25)								
**Sapph**	**(3,4,-16)**	**11.919**	**74.23**	**0.419**	**0.6**	**3.497**	**2.18**	**43.72**	**43.77**
	Equiv. Refl.: (3,-7,16), (4,3,16), (4,-7,-16), (7,-3,16), (7,-4,-16), (-3,7,-16), (-3,-4,16), (-4,7,16), (-4,-3,-16) (-7,3,-16), (-7,4,16)								
**Quartz**	**(-1,2,10)**	**11.745**	**71.49**	**0.384**	**0.6**	**4.146**	**2.38**	**51.82**	**51.88**
	Equiv. Refl.: (-1,2,-10), (-1,-1,10), (-1,-1,-10)								
**Quartz**	**(2,-1,10)**	**11.745**	**71.49**	**0.382**	**0.6**	**4.146**	**2.37**	**51.82**	**51.87**
	Equiv. Refl.: (2,-1,-10)								
**Quartz**	**(9,-2,0)**	**11.926**	**74.35**	**0.372**	**0.5**	**3.469**	**1.69**	**43.37**	**43.4**
	Equiv. Refl.: (9,-7,0), (-2,9,0), (-2,-7,0), (-7,9,0), (-7,-2,0)								
**LiNbO3**	**(3,6,9)**	**11.748**	**71.54**	**0.334**	**0.9**	**4.135**	**3.57**	**51.69**	**51.81**
	Equiv. Refl.: (3,6,-9), (3,-9,9), (3,-9,-9), (6,3,9), (6,3,-9), (6,-9,9), (6,-9,-9), (9,-3,9), (9,-3,-9) (9,-6,9), (9,-6,-9), (-3,9,9), (-3,9,-9), (-3,-6,9), (-3,-6,-9), (-6,9,9), (-6,9,-9), (-6,-3,9) (-6,-3,-9), (-9,3,9), (-9,3,-9), (-9,6,9), (-9,6,-9)								
**Quartz**	**(0,8,-2)**	**11.879**	**73.57**	**0.319**	**0.5**	**3.651**	**1.73**	**45.64**	**45.67**
	Equiv. Refl.: (0,-8,2), (8,0,2), (8,-8,-2), (-8,0,-2), (-8,8,2)								
**Quartz**	**(0,2,10)**	**11.835**	**72.85**	**0.286**	**0.4**	**3.821**	**1.65**	**47.76**	**47.79**
	Equiv. Refl.: (0,-2,-10), (2,7,0), (2,-9,0), (7,2,0), (7,-9,0), (-9,2,0), (-9,7,0)								
**Quartz**	**(2,0,-10)**	**11.835**	**72.85**	**0.281**	**0.4**	**3.821**	**1.63**	**47.76**	**47.79**
	Equiv. Refl.: (-2,0,10), (-2,2,-10)								
**Quartz**	**(2,-2,10)**	**11.835**	**72.85**	**0.28**	**0.4**	**3.821**	**1.63**	**47.76**	**47.79**
**LiNbO3**	**(1,8,-7)**	**12.286**	**82.74**	**0.247**	**1.1**	**1.577**	**1.76**	**19.71**	**19.79**
	Equiv. Refl.: (1,-9,7), (8,1,7), (8,-9,-7), (9,-1,7), (9,-8,-7), (-1,9,-7), (-1,-8,7), (-8,9,7), (-8,-1,-7) (-9,1,-7), (-9,8,7)								
**Sapph**	**(3,4,17)**	**12.23**	**80.93**	**0.217**	**0.5**	**1.977**	**0.98**	**24.71**	**24.73**
	Equiv. Refl.: (3,-7,-17), (4,3,-17), (4,-7,17), (7,-3,-17), (7,-4,17), (-3,7,17), (-3,-4,-17), (-4,7,-17), (-4,-3,17) (-7,3,17), (-7,4,-17)								

Ei =	12.385	keV						ΔEg	ΔEt
**Cryst**	**Refl**	**EB**	**ΘB**	**∫ IR dΘ**	**Width**	**Ei cotΘ**	**ΔE**	**2m, 50µm**	**2m, 50µm**
	**(h,k,l)**	**[keV]**	**[°]**	**[µrad]**	**[µrad]**	**[meV/µrad]**	**[meV]**	**[meV]**	**[meV]**
**Sapph**	**(3,6,3)**	**12.027**	**76.18**	**0.204**	**0.4**	**3.046**	**1.25**	**38.08**	**38.1**
	Equiv. Refl.: (3,6,-3), (3,-9,3), (3,-9,-3), (6,3,3), (6,3,-3), (6,-9,3), (6,-9,-3), (9,-3,3), (9,-3,-3) (9,-6,3), (9,-6,-3), (-3,9,3), (-3,9,-3), (-3,-6,3), (-3,-6,-3), (-6,9,3), (-6,9,-3), (-6,-3,3) (-6,-3,-3), (-9,3,3), (-9,3,-3), (-9,6,3), (-9,6,-3)								
**LiNbO3**	**(2,3,23)**	**11.938**	**74.56**	**0.191**	**0.7**	**3.42**	**2.35**	**42.75**	**42.82**
	Equiv. Refl.: (2,-5,-23), (3,2,-23), (3,-5,23), (5,-2,-23), (5,-3,23), (-2,5,23), (-2,-3,-23), (-3,5,-23), (-3,-2,23) (-5,2,23), (-5,3,-23)								
**LiNbO3**	**(1,8,5)**	**12.089**	**77.45**	**0.151**	**0.7**	**2.758**	**1.85**	**34.47**	**34.52**
	Equiv. Refl.: (1,-9,-5), (8,1,-5), (8,-9,5), (9,-1,-5), (9,-8,5), (-1,9,5), (-1,-8,-5), (-8,9,-5), (-8,-1,5) (-9,1,5), (-9,8,-5)								
**LiNbO3**	**(1,8,-1)**	**11.889**	**73.73**	**0.124**	**0.5**	**3.616**	**1.95**	**45.2**	**45.24**
	Equiv. Refl.: (1,-9,1), (8,1,1), (8,-9,-1), (9,-1,1), (9,-8,-1), (-1,9,-1), (-1,-8,1), (-8,9,1), (-8,-1,-1) (-9,1,-1), (-9,8,1)								
**LiNbO3**	**(3,3,21)**	**11.849**	**73.08**	**0.068**	**0.4**	**3.769**	**1.49**	**47.11**	**47.13**
	Equiv. Refl.: (3,3,-21), (3,-6,21), (3,-6,-21), (6,-3,21), (6,-3,-21), (-3,6,21), (-3,6,-21), (-3,-3,21), (-3,-3,-21) (-6,3,21), (-6,3,-21)								
**Sapph**	**(4,5,2)**	**11.789**	**72.15**	**0.052**	**0.2**	**3.989**	**0.67**	**49.86**	**49.87**
	Equiv. Refl.: (4,-9,-2), (5,4,-2), (5,-9,2), (9,-4,-2), (9,-5,2), (-4,9,2), (-4,-5,-2), (-5,9,-2), (-5,-4,2) (-9,4,2), (-9,5,-2)								
**Sapph**	**(4,4,12)**	**11.893**	**73.79**	**0.038**	**0.1**	**3.599**	**0.54**	**44.99**	**45**
	Equiv. Refl.: (4,4,-12), (4,-8,12), (4,-8,-12), (8,-4,12), (8,-4,-12), (-4,8,12), (-4,8,-12), (-4,-4,12), (-4,-4,-12) (-8,4,12), (-8,4,-12)								
**LiNbO3**	**(4,4,15)**	**11.739**	**71.41**	**0.012**	**0.2**	**4.166**	**0.71**	**52.07**	**52.08**
	Equiv. Refl.: (4,4,-15), (4,-8,15), (4,-8,-15), (8,-4,15), (8,-4,-15), (-4,8,15), (-4,8,-15), (-4,-4,15), (-4,-4,-15) (-8,4,15), (-8,4,-15)								
**LiNbO3**	**(5,5,3)**	**12.116**	**78.05**	**0.01**	**0.2**	**2.622**	**0.54**	**32.77**	**32.78**
	Equiv. Refl.: (5,5,-3), (5,-10,3), (5,-10,-3), (10,-5,3), (10,-5,-3), (-5,10,3), (-5,10,-3), (-5,-5,3), (-5,-5,-3) (-10,5,3), (-10,5,-3)								

Ei =	10.871	keV						ΔEg	ΔEt
Cryst	Refl	EB	ΘB	∫ IR dΘ	Width	Ei cotΘ	ΔE	2m, 50µm	2m, 50µm
	(h,k,l)	[keV]	[°]	[µrad]	[µrad]	[meV/µrad]	[meV]	[meV]	[meV]
**Ge**	**(4,4,8)**	**10.736**	**80.97**	**13.363**	**13.1**	**1.727**	**22.58**	**21.58**	**31.23**
**Si**	**(4,6,6)**	**10.708**	**80.06**	**10.34**	**8.9**	**1.906**	**16.93**	**23.82**	**29.23**
**Ge**	**(4,6,6)**	**10.279**	**71.01**	**7.733**	**7.4**	**3.741**	**27.52**	**46.76**	**54.26**
**Ge**	**(1,3,9)**	**10.453**	**74.06**	**5.495**	**5.8**	**3.105**	**18.04**	**38.81**	**42.8**
**Si**	**(1,1,9)**	**10.399**	**73.06**	**4.606**	**4.1**	**3.312**	**13.47**	**41.4**	**43.54**
	Equiv. Refl.: (3,5,7)								
**Quartz**	**(0,4,8)**	**10.87**	**89.42**	**182.338**	**154**	**0.11**	**16.9**	**1.37**	**16.96**
	Equiv. Refl.: (0,-4,-8)								
**Quartz**	**(-4,0,8)**	**10.87**	**89.42**	**182.275**	**154**	**0.11**	**16.9**	**1.37**	**16.95**
**Quartz**	**(4,0,-8)**	**10.87**	**89.42**	**182.272**	**154**	**0.11**	**16.9**	**1.37**	**16.95**
**Quartz**	**(4,-4,8)**	**10.87**	**89.42**	**182.25**	**153.9**	**0.11**	**16.9**	**1.37**	**16.95**
	Equiv. Refl.: (-4,4,-8)								
**LiNbO3**	**(1,-8,-6)**	**10.835**	**85.36**	**42.885**	**40**	**0.882**	**35.26**	**11.03**	**36.95**
	Equiv. Refl.: (7,1,-6), (8,-1,-6), (-1,-7,-6), (-7,8,-6), (-8,7,-6)								
**LiNbO3**	**(1,7,6)**	**10.835**	**85.36**	**42.606**	**40**	**0.882**	**35.26**	**11.03**	**36.95**
	Equiv. Refl.: (7,-8,6), (8,-7,6), (-1,8,6), (-7,-1,6), (-8,1,6)								
**LiNbO3**	**(1,7,-6)**	**10.835**	**85.36**	**40.654**	**37.7**	**0.882**	**33.27**	**11.03**	**35.05**
	Equiv. Refl.: (7,-8,-6), (8,-7,-6), (-1,8,-6), (-7,-1,-6), (-8,1,-6)								
**LiNbO3**	**(1,-8,6)**	**10.835**	**85.36**	**39.203**	**37.7**	**0.882**	**33.27**	**11.03**	**35.05**
	Equiv. Refl.: (7,1,6), (8,-1,6), (-1,-7,6), (-7,8,6), (-8,7,6)								
**LiNbO3**	**(3,3,18)**	**10.816**	**84.25**	**38.399**	**34.7**	**1.095**	**37.96**	**13.68**	**40.35**
	Equiv. Refl.: (3,-6,18), (6,-3,18), (-3,-3,18)								
**LiNbO3**	**(-3,6,18)**	**10.816**	**84.25**	**38.398**	**34.7**	**1.095**	**37.96**	**13.68**	**40.35**
	Equiv. Refl.: (-6,3,18)								
**LiNbO3**	**(3,3,-18)**	**10.816**	**84.25**	**36.602**	**34.7**	**1.095**	**37.96**	**13.68**	**40.35**
	Equiv. Refl.: (3,-6,-18), (6,-3,-18), (-3,6,-18), (-3,-3,-18), (-6,3,-18)								
**Quartz**	**(5,0,-7)**	**10.841**	**85.77**	**27.222**	**22.7**	**0.803**	**18.2**	**10.04**	**20.79**
	Equiv. Refl.: (5,-5,7), (-5,0,7)								
**Quartz**	**(-5,5,-7)**	**10.841**	**85.77**	**27.221**	**22.7**	**0.803**	**18.2**	**10.04**	**20.79**
**Quartz**	**(0,5,7)**	**10.841**	**85.77**	**27.216**	**22.7**	**0.803**	**18.2**	**10.04**	**20.78**
	Equiv. Refl.: (0,-5,-7)								
**LiNbO3**	**(2,-5,-20)**	**10.804**	**83.63**	**25.29**	**23.6**	**1.214**	**28.71**	**15.18**	**32.48**
	Equiv. Refl.: (3,2,-20), (5,-2,-20), (-2,-3,-20), (-3,5,-20), (-5,3,-20)								
**LiNbO3**	**(2,3,20)**	**10.804**	**83.63**	**23.38**	**23.6**	**1.214**	**28.71**	**15.18**	**32.48**
	Equiv. Refl.: (3,-5,20), (5,-3,20), (-2,5,20), (-3,-2,20), (-5,2,20)								
**LiNbO3**	**(0,7,-10)**	**10.711**	**80.17**	**22.859**	**21**	**1.883**	**39.47**	**23.54**	**45.96**
	Equiv. Refl.: (7,-7,-10), (-7,0,-10)								
**LiNbO3**	**(0,-7,10)**	**10.711**	**80.17**	**22.613**	**21**	**1.883**	**39.47**	**23.54**	**45.96**
	Equiv. Refl.: (7,0,10), (-7,7,10)								
**LiNbO3**	**(3,5,10)**	**10.711**	**80.17**	**19.223**	**17.4**	**1.883**	**32.71**	**23.54**	**40.3**
	Equiv. Refl.: (5,-8,10), (8,-5,10), (-3,8,10), (-5,-3,10), (-8,3,10)								
**LiNbO3**	**(2,-8,-8)**	**10.646**	**78.32**	**18.262**	**16.8**	**2.247**	**37.73**	**28.08**	**47.03**
	Equiv. Refl.: (6,2,-8), (8,-2,-8), (-2,-6,-8), (-6,8,-8), (-8,6,-8)								
**LiNbO3**	**(2,6,8)**	**10.646**	**78.32**	**17.831**	**16.8**	**2.247**	**37.73**	**28.08**	**47.03**
	Equiv. Refl.: (6,-8,8), (8,-6,8), (-2,8,8), (-6,-2,8), (-8,2,8)								
**LiNbO3**	**(3,-8,-10)**	**10.711**	**80.17**	**17.272**	**17.4**	**1.883**	**32.71**	**23.54**	**40.3**
	Equiv. Refl.: (5,3,-10), (8,-3,-10), (-3,-5,-10), (-5,8,-10), (-8,5,-10)								

Ei =	10.871	keV						ΔEg	ΔEt
Cryst	Refl	EB	ΘB	∫ IR dΘ	Width	Ei cotΘ	ΔE	2m, 50µm	2m, 50µm
	(h,k,l)	[keV]	[°]	[µrad]	[µrad]	[meV/µrad]	[meV]	[meV]	[meV]
Sapph	(2,6,-1)	10.859	87.31	16.941	16.4	0.511	8.4	6.38	10.55
	Equiv. Refl.: (2,-8,1), (6,2,1), (6,-8,-1), (8,-2,1), (8,-6,-1), (-2,8,-1), (-2,-6,1), (-6,8,1), (-6,-2,-1) (-8,2,-1), (-8,6,1)								
LiNbO3	(0,4,20)	10.532	75.65	16.224	14.9	2.781	41.41	34.76	54.06
	Equiv. Refl.: (4,-4,20), (-4,0,20)								
LiNbO3	(0,-4,-20)	10.532	75.65	16.159	14.9	2.781	41.41	34.76	54.06
	Equiv. Refl.: (4,0,-20), (-4,4,-20)								
Quartz	(-4,4,8)	10.87	89.42	15.816	21.4	0.11	2.35	1.37	2.72
Quartz	(-4,0,-8)	10.87	89.42	15.81	21.4	0.11	2.35	1.37	2.72
Quartz	(4,-4,-8)	10.87	89.42	15.805	21.4	0.11	2.35	1.37	2.72
Quartz	(4,0,8)	10.87	89.42	15.799	21.4	0.11	2.35	1.37	2.72
Quartz	(0,4,-8)	10.87	89.42	15.606	21.3	0.11	2.33	1.37	2.71
Quartz	(0,-4,8)	10.87	89.42	15.604	21.3	0.11	2.33	1.37	2.71
Sapph	(4,4,6)	10.809	83.89	15.354	13.1	1.164	15.28	14.55	21.1
	Equiv. Refl.: (4,4,-6), (4,-8,6), (4,-8,-6), (8,-4,6), (8,-4,-6), (-4,8,6), (-4,8,-6), (-4,-4,6), (-4,-4,-6) (-8,4,6), (-8,4,-6)								
LiNbO3	(3,5,-8)	10.37	72.54	15.234	13.5	3.42	46.16	42.75	62.91
	Equiv. Refl.: (-3,8,-8), (-5,-3,-8), (-8,3,-8)								
LiNbO3	(5,-8,-8)	10.37	72.54	15.233	13.5	3.42	46.16	42.75	62.91
	Equiv. Refl.: (8,-5,-8)								
Quartz	(-3,8,-3)	10.764	81.95	14.741	12.2	1.538	18.82	19.23	26.91
	Equiv. Refl.: (-3,-5,3)								
Quartz	(8,-5,-3)	10.764	81.95	14.74	12.2	1.538	18.82	19.23	26.91
Quartz	(8,-3,3)	10.764	81.95	14.739	12.2	1.538	18.82	19.23	26.91
	Equiv. Refl.: (-5,8,3), (-5,-3,-3)								
Quartz	(3,5,-3)	10.764	81.95	14.702	12.2	1.538	18.82	19.23	26.91
	Equiv. Refl.: (3,-8,3)								
Quartz	(-8,5,3)	10.764	81.95	14.701	12.2	1.538	18.82	19.23	26.91
Quartz	(5,3,3)	10.764	81.95	14.7	12.2	1.538	18.82	19.23	26.91
	Equiv. Refl.: (5,-8,-3), (-8,3,-3)								
LiNbO3	(1,5,-16)	10.542	75.86	14.683	13.7	2.738	37.42	34.23	50.72
	Equiv. Refl.: (-1,6,-16), (-5,-1,-16), (-6,1,-16)								
LiNbO3	(5,-6,-16)	10.542	75.86	14.682	13.7	2.738	37.42	34.23	50.72
	Equiv. Refl.: (6,-5,-16)								
LiNbO3	(1,-6,16)	10.542	75.86	14.564	13.7	2.738	37.42	34.23	50.72
	Equiv. Refl.: (5,1,16), (6,-1,16), (-1,-5,16), (-5,6,16), (-6,5,16)								
LiNbO3	(3,-8,8)	10.37	72.54	14.462	13.5	3.42	46.16	42.75	62.91
	Equiv. Refl.: (5,3,8), (8,-3,8), (-3,-5,8), (-5,8,8), (-8,5,8)								
LiNbO3	(4,5,-1)	10.869	88.97	13.791	29.4	0.195	5.72	2.43	6.21
	Equiv. Refl.: (4,-9,1), (5,4,1), (5,-9,-1), (9,-4,1), (9,-5,-1), (-4,9,-1), (-4,-5,1), (-5,9,1), (-5,-4,-1) (-9,4,-1), (-9,5,1)								
LiNbO3	(1,-3,22)	10.503	75.05	13.646	12.7	2.902	36.8	36.27	51.67
	Equiv. Refl.: (2,1,22), (3,-1,22), (-1,-2,22), (-2,3,22), (-3,2,22)								
LiNbO3	(0,0,-24)	10.732	80.84	13.64	12.9	1.754	22.61	21.92	31.49
LiNbO3	(1,-8,0)	10.498	74.95	13.629	12.7	2.924	37.04	36.55	52.03
	Equiv. Refl.: (7,1,0), (8,-1,0), (-1,-7,0), (-7,8,0), (-8,7,0)								
LiNbO3	(3,-7,-14)	10.523	75.46	13.416	12.4	2.819	34.89	35.24	49.59
	Equiv. Refl.: (4,3,-14), (7,-3,-14), (-3,-4,-14), (-4,7,-14), (-7,4,-14)								
LiNbO3	(1,7,0)	10.498	74.95	13.385	12.7	2.924	37.04	36.55	52.03
	Equiv. Refl.: (-1,8,0), (-7,-1,0), (-8,1,0)								

Ei =	10.871	keV						ΔEg	ΔEt
Cryst	Refl	EB	ΘB	∫ IR dΘ	Width	Ei cotΘ	ΔE	2m, 50µm	2m, 50µm
	(h,k,l)	[keV]	[°]	[µrad]	[µrad]	[meV/µrad]	[meV]	[meV]	[meV]
**LiNbO3**	**(7,-8,0)**	**10.498**	**74.95**	**13.384**	**12.7**	**2.924**	**37.04**	**36.55**	**52.03**
	Equiv. Refl.: (8,-7,0)								
**LiNbO3**	**(1,2,-22)**	**10.503**	**75.05**	**13.375**	**12.7**	**2.902**	**36.8**	**36.27**	**51.67**
	Equiv. Refl.: (2,-3,-22), (3,-2,-22), (-1,3,-22), (-2,-1,-22), (-3,1,-22)								
**LiNbO3**	**(0,7,8)**	**10.37**	**72.54**	**13.147**	**11.9**	**3.42**	**40.74**	**42.75**	**59.05**
	Equiv. Refl.: (7,-7,8), (-7,0,8)								
**LiNbO3**	**(3,4,14)**	**10.523**	**75.46**	**12.723**	**12.4**	**2.819**	**34.89**	**35.24**	**49.59**
	Equiv. Refl.: (4,-7,14), (7,-4,14), (-3,7,14), (-4,-3,14), (-7,3,14)								
**LiNbO3**	**(0,2,22)**	**10.223**	**70.12**	**12.665**	**11.4**	**3.93**	**44.83**	**49.13**	**66.51**
	Equiv. Refl.: (2,-2,22), (-2,0,22)								
**LiNbO3**	**(0,-7,-8)**	**10.37**	**72.54**	**12.562**	**11.9**	**3.42**	**40.74**	**42.75**	**59.05**
	Equiv. Refl.: (7,0,-8), (-7,7,-8)								
**LiNbO3**	**(1,-4,20)**	**10.253**	**70.59**	**12.453**	**11.4**	**3.831**	**43.61**	**47.89**	**64.77**
	Equiv. Refl.: (3,1,20), (4,-1,20), (-1,-3,20)								
**LiNbO3**	**(-3,4,20)**	**10.253**	**70.59**	**12.452**	**11.4**	**3.831**	**43.61**	**47.89**	**64.77**
	Equiv. Refl.: (-4,3,20)								
**Sapph**	**(1,5,14)**	**10.715**	**80.28**	**12.405**	**10.2**	**1.862**	**19.08**	**23.27**	**30.09**
	Equiv. Refl.: (1,-6,-14), (5,1,-14), (5,-6,14), (6,-1,-14), (6,-5,14), (-1,6,14), (-1,-5,-14), (-5,6,-14), (-5,-1,14) (-6,1,14), (-6,5,-14)								
**LiNbO3**	**(1,3,-20)**	**10.253**	**70.59**	**12.285**	**11.4**	**3.831**	**43.61**	**47.89**	**64.77**
	Equiv. Refl.: (3,-4,-20), (4,-3,-20), (-1,4,-20), (-3,-1,-20), (-4,1,-20)								
**LiNbO3**	**(0,-2,-22)**	**10.223**	**70.12**	**12.206**	**11.4**	**3.93**	**44.83**	**49.13**	**66.51**
	Equiv. Refl.: (2,0,-22), (-2,2,-22)								
**Sapph**	**(3,5,4)**	**10.703**	**79.9**	**12.045**	**10**	**1.936**	**19.29**	**24.2**	**30.95**
	Equiv. Refl.: (3,-8,-4), (5,3,-4), (5,-8,4), (8,-3,-4), (8,-5,4), (-3,8,4), (-3,-5,-4), (-5,8,-4), (-5,-3,4) (-8,3,4), (-8,5,-4)								
**LiNbO3**	**(2,4,16)**	**10.263**	**70.75**	**12.01**	**10.9**	**3.797**	**41.52**	**47.46**	**63.06**
	Equiv. Refl.: (4,-6,16), (6,-4,16), (-2,6,16), (-4,-2,16), (-6,2,16)								
**LiNbO3**	**(0,0,24)**	**10.732**	**80.84**	**11.824**	**12.9**	**1.754**	**22.61**	**21.92**	**31.49**
**LiNbO3**	**(2,-6,-16)**	**10.263**	**70.75**	**11.577**	**10.9**	**3.797**	**41.52**	**47.46**	**63.06**
	Equiv. Refl.: (4,2,-16), (6,-2,-16), (-2,-4,-16), (-4,6,-16), (-6,4,-16)								
**LiNbO3**	**(1,-5,-18)**	**10.266**	**70.8**	**11.368**	**10.2**	**3.787**	**38.79**	**47.33**	**61.19**
	Equiv. Refl.: (4,1,-18), (5,-1,-18), (-1,-4,-18), (-4,5,-18), (-5,4,-18)								
**LiNbO3**	**(1,4,-18)**	**10.266**	**70.8**	**11.038**	**10**	**3.787**	**37.9**	**47.33**	**60.63**
	Equiv. Refl.: (4,-5,-18), (5,-4,-18), (-1,5,-18), (-4,-1,-18), (-5,1,-18)								
**LiNbO3**	**(1,4,18)**	**10.266**	**70.8**	**10.987**	**10.2**	**3.787**	**38.79**	**47.33**	**61.19**
	Equiv. Refl.: (4,-5,18), (5,-4,18), (-1,5,18), (-4,-1,18), (-5,1,18)								
**LiNbO3**	**(1,-5,18)**	**10.266**	**70.8**	**10.704**	**10**	**3.787**	**37.9**	**47.33**	**60.63**
	Equiv. Refl.: (4,1,18), (5,-1,18), (-1,-4,18), (-4,5,18), (-5,4,18)								
**Sapph**	**(0,5,16)**	**10.719**	**80.41**	**9.988**	**8.6**	**1.836**	**15.7**	**22.95**	**27.81**
	Equiv. Refl.: (0,-5,-16), (5,0,-16), (5,-5,16), (-5,0,16), (-5,5,-16)								
**Sapph**	**(3,4,-10)**	**10.321**	**71.7**	**9.889**	**7.8**	**3.596**	**28.18**	**44.95**	**53.06**
	Equiv. Refl.: (3,-7,10), (4,3,10), (4,-7,-10), (7,-3,10), (7,-4,-10), (-3,7,-10), (-3,-4,10), (-4,7,10), (-4,-3,-10) (-7,3,-10), (-7,4,10)								
**Quartz**	**(0,0,9)**	**10.323**	**71.73**	**9.514**	**7.7**	**3.588**	**27.7**	**44.85**	**52.72**
	Equiv. Refl.: (0,0,-9)								
**Quartz**	**(8,-2,-2)**	**10.754**	**81.59**	**8.388**	**7.1**	**1.608**	**11.41**	**20.1**	**23.11**
	Equiv. Refl.: (8,-6,2)								
**Quartz**	**(-2,8,2)**	**10.754**	**81.59**	**8.387**	**7.1**	**1.608**	**11.4**	**20.1**	**23.11**
	Equiv. Refl.: (-2,-6,-2), (-6,-2,2)								
**Quartz**	**(-6,8,-2)**	**10.754**	**81.59**	**8.386**	**7.1**	**1.608**	**11.4**	**20.1**	**23.11**

Ei =	10.871	keV						ΔEg	ΔEt
**Cryst**	**Refl**	**EB**	**ΘB**	**∫ IR dΘ**	**Width**	**Ei cotΘ**	**ΔE**	**2m, 50µm**	**2m, 50µm**
	**(h,k,l)**	**[keV]**	**[°]**	**[µrad]**	**[µrad]**	**[meV/µrad]**	**[meV]**	**[meV]**	**[meV]**
**Quartz**	**(-8,2,2)**	**10.754**	**81.59**	**8.24**	**7.1**	**1.608**	**11.41**	**20.1**	**23.11**
**Quartz**	**(2,6,2)**	**10.754**	**81.59**	**8.239**	**7.1**	**1.608**	**11.4**	**20.1**	**23.11**
	Equiv. Refl.: (2,-8,-2), (6,2,-2), (-8,6,-2)								
**Quartz**	**(6,-8,2)**	**10.754**	**81.59**	**8.238**	**7.1**	**1.608**	**11.4**	**20.1**	**23.11**
**Quartz**	**(0,2,-9)**	**10.727**	**80.65**	**7.963**	**6.7**	**1.79**	**11.96**	**22.38**	**25.37**
	Equiv. Refl.: (0,-2,9)								
**Quartz**	**(-2,0,-9)**	**10.727**	**80.65**	**7.96**	**6.7**	**1.79**	**11.95**	**22.38**	**25.37**
	Equiv. Refl.: (-2,2,9)								
**Quartz**	**(2,0,9)**	**10.727**	**80.65**	**7.959**	**6.7**	**1.79**	**11.95**	**22.38**	**25.37**
**Quartz**	**(2,-2,-9)**	**10.727**	**80.65**	**7.958**	**6.7**	**1.79**	**11.95**	**22.38**	**25.37**
**Quartz**	**(6,-1,-6)**	**10.638**	**78.12**	**7.484**	**6.2**	**2.287**	**14.19**	**28.59**	**31.92**
**Quartz**	**(6,-5,6)**	**10.638**	**78.12**	**7.483**	**6.2**	**2.287**	**14.19**	**28.59**	**31.91**
	Equiv. Refl.: (-1,6,6), (-1,-5,-6)								
**Quartz**	**(-5,6,-6)**	**10.638**	**78.12**	**7.482**	**6.2**	**2.287**	**14.19**	**28.59**	**31.91**
	Equiv. Refl.: (-5,-1,6)								
**Quartz**	**(-6,1,6)**	**10.638**	**78.12**	**7.456**	**6.2**	**2.287**	**14.19**	**28.59**	**31.92**
**Quartz**	**(-6,5,-6)**	**10.638**	**78.12**	**7.455**	**6.2**	**2.287**	**14.19**	**28.59**	**31.91**
**Quartz**	**(1,5,6)**	**10.638**	**78.12**	**7.454**	**6.2**	**2.287**	**14.19**	**28.59**	**31.91**
	Equiv. Refl.: (1,-6,-6), (5,1,-6), (5,-6,6)								
**Sapph**	**(0,7,2)**	**10.574**	**76.58**	**7.338**	**6.3**	**2.594**	**16.38**	**32.43**	**36.33**
	Equiv. Refl.: (0,-7,-2), (7,0,-2), (7,-7,2), (-7,0,2), (-7,7,-2)								
**Sapph**	**(0,6,12)**	**10.69**	**79.54**	**6.956**	**6.2**	**2.007**	**12.54**	**25.09**	**28.05**
	Equiv. Refl.: (0,6,-12), (0,-6,12), (0,-6,-12), (6,0,12), (6,0,-12), (6,-6,12), (6,-6,-12), (-6,0,12), (-6,0,-12) (-6,6,12), (-6,6,-12)								
**LiNbO3**	**(1,6,13)**	**10.814**	**84.11**	**6.549**	**9.3**	**1.122**	**10.47**	**14.03**	**17.5**
	Equiv. Refl.: (1,-7,-13), (6,1,-13), (6,-7,13), (7,-1,-13), (7,-6,13), (-1,7,13), (-1,-6,-13), (-6,7,-13), (-6,-1,13) (-7,1,13), (-7,6,-13)								
**Quartz**	**(8,-2,-1)**	**10.569**	**76.46**	**6.216**	**5.2**	**2.618**	**13.69**	**32.72**	**35.47**
	Equiv. Refl.: (8,-6,1), (-2,8,1), (-2,-6,-1), (-6,8,-1), (-6,-2,1)								
**Sapph**	**(2,4,-14)**	**10.393**	**72.95**	**6.187**	**5.2**	**3.333**	**17.41**	**41.67**	**45.16**
	Equiv. Refl.: (2,-6,14), (4,2,14), (4,-6,-14), (6,-2,14), (6,-4,-14), (-2,6,-14), (-2,-4,14), (-4,6,14), (-4,-2,-14) (-6,2,-14), (-6,4,14)								
**Quartz**	**(3,-8,-3)**	**10.764**	**81.95**	**6.167**	**5.2**	**1.538**	**7.98**	**19.23**	**20.82**
**Quartz**	**(3,5,3)**	**10.764**	**81.95**	**6.166**	**5.2**	**1.538**	**7.98**	**19.23**	**20.82**
**Quartz**	**(5,3,-3)**	**10.764**	**81.95**	**6.164**	**5.2**	**1.538**	**7.98**	**19.23**	**20.82**
	Equiv. Refl.: (5,-8,3), (-8,3,3), (-8,5,-3)								
**Quartz**	**(2,6,1)**	**10.569**	**76.46**	**6.147**	**5.2**	**2.618**	**13.69**	**32.72**	**35.47**
	Equiv. Refl.: (2,-8,-1), (6,2,-1), (6,-8,1), (-8,2,1), (-8,6,-1)								
**Quartz**	**(4,4,3)**	**10.665**	**78.82**	**5.874**	**4.8**	**2.149**	**10.32**	**26.86**	**28.77**
	Equiv. Refl.: (4,-8,-3)								
**Quartz**	**(4,4,-3)**	**10.665**	**78.82**	**5.873**	**4.8**	**2.149**	**10.32**	**26.86**	**28.77**
	Equiv. Refl.: (4,-8,3), (-8,4,3), (-8,4,-3)								
**Sapph**	**(1,2,20)**	**10.342**	**72.06**	**5.719**	**4.8**	**3.52**	**17.03**	**44**	**47.18**
	Equiv. Refl.: (1,-3,-20), (2,1,-20), (2,-3,20), (3,-1,-20), (3,-2,20), (-1,3,20), (-1,-2,-20), (-2,3,-20), (-2,-1,20) (-3,1,20), (-3,2,-20)								
**Quartz**	**(-3,8,3)**	**10.764**	**81.95**	**5.705**	**5.2**	**1.538**	**7.98**	**19.23**	**20.82**
	Equiv. Refl.: (-3,-5,-3)								
**Quartz**	**(8,-3,-3)**	**10.764**	**81.95**	**5.704**	**5.2**	**1.538**	**7.98**	**19.23**	**20.82**
	Equiv. Refl.: (8,-5,3)								

Ei = Cryst	10.871 Refl (h,k,l)	keV EB [keV]	ΘB [°]	∫ IR dΘ [µrad]	Width [µrad]	Ei cotΘ [meV/µrad]	ΔE [meV]	ΔEg 2m, 50µm [meV]	ΔEt 2m, 50µm [meV]
**Quartz**	**(-5,8,-3)**	**10.764**	**81.95**	**5.703**	**5.2**	**1.538**	**7.98**	**19.23**	**20.82**
	Equiv. Refl.: (-5,-3,3)								
**Sapph**	**(4,4,0)**	**10.423**	**73.49**	**5.669**	**4.7**	**3.222**	**15.07**	**40.27**	**43**
	Equiv. Refl.: (4,-8,0), (8,-4,0), (-4,8,0), (-4,-4,0), (-8,4,0)								
**Quartz**	**(-4,8,3)**	**10.665**	**78.82**	**5.518**	**4.8**	**2.149**	**10.32**	**26.86**	**28.77**
	Equiv. Refl.: (-4,-4,-3)								
**Quartz**	**(8,-4,3)**	**10.665**	**78.82**	**5.517**	**4.8**	**2.149**	**10.32**	**26.86**	**28.77**
	Equiv. Refl.: (-4,8,-3), (-4,-4,3)								
**Quartz**	**(8,-4,-3)**	**10.665**	**78.82**	**5.516**	**4.8**	**2.149**	**10.32**	**26.86**	**28.77**
**Quartz**	**(0,6,5)**	**10.455**	**74.1**	**5.512**	**4.6**	**3.096**	**14.25**	**38.7**	**41.24**
	Equiv. Refl.: (0,-6,-5), (6,0,-5), (-6,0,5)								
**Quartz**	**(6,-6,5)**	**10.455**	**74.1**	**5.511**	**4.6**	**3.096**	**14.25**	**38.7**	**41.24**
	Equiv. Refl.: (-6,6,-5)								
**Quartz**	**(6,1,4)**	**10.599**	**77.15**	**5.41**	**4.4**	**2.48**	**10.82**	**31**	**32.84**
	Equiv. Refl.: (6,-7,-4)								
**Quartz**	**(1,6,-4)**	**10.599**	**77.15**	**5.408**	**4.4**	**2.48**	**10.82**	**31**	**32.84**
	Equiv. Refl.: (-7,1,-4)								
**Quartz**	**(1,-7,4)**	**10.599**	**77.15**	**5.407**	**4.4**	**2.48**	**10.82**	**31**	**32.84**
	Equiv. Refl.: (-7,6,4)								
**Quartz**	**(0,7,1)**	**10.263**	**70.75**	**5.342**	**4.3**	**3.796**	**16.47**	**47.45**	**50.23**
	Equiv. Refl.: (0,-7,-1), (7,0,-1), (7,-7,1), (-7,0,1), (-7,7,-1)								
**Quartz**	**(-6,7,4)**	**10.599**	**77.15**	**5.037**	**4.4**	**2.48**	**10.82**	**31**	**32.84**
	Equiv. Refl.: (-6,-1,-4)								
**Quartz**	**(-1,-6,4)**	**10.599**	**77.15**	**5.036**	**4.4**	**2.48**	**10.82**	**31**	**32.84**
**Quartz**	**(7,-1,4)**	**10.599**	**77.15**	**5.035**	**4.4**	**2.48**	**10.82**	**31**	**32.84**
	Equiv. Refl.: (-1,7,-4)								
**Quartz**	**(7,-6,-4)**	**10.599**	**77.15**	**5.034**	**4.4**	**2.48**	**10.82**	**31**	**32.84**
**Sapph**	**(1,6,-8)**	**10.578**	**76.67**	**4.981**	**4.4**	**2.575**	**11.28**	**32.19**	**34.11**
	Equiv. Refl.: (1,-7,8), (6,1,8), (6,-7,-8), (7,-1,8), (7,-6,-8), (-1,7,-8), (-1,-6,8), (-6,7,8), (-6,-1,-8) (-7,1,-8), (-7,6,8)								
**Quartz**	**(7,-3,-5)**	**10.556**	**76.18**	**4.879**	**4**	**2.674**	**10.59**	**33.43**	**35.07**
	Equiv. Refl.: (7,-4,5)								
**Quartz**	**(-4,7,-5)**	**10.556**	**76.18**	**4.878**	**4**	**2.674**	**10.59**	**33.43**	**35.07**
**Quartz**	**(-3,7,5)**	**10.556**	**76.18**	**4.877**	**4**	**2.674**	**10.59**	**33.43**	**35.06**
	Equiv. Refl.: (-3,-4,-5), (-4,-3,5)								
**Sapph**	**(1,1,21)**	**10.356**	**72.3**	**4.713**	**4**	**3.47**	**13.9**	**43.37**	**45.54**
	Equiv. Refl.: (1,1,-21), (1,-2,21), (1,-2,-21), (2,-1,21), (2,-1,-21), (-1,2,21), (-1,2,-21), (-1,-1,21), (-1,-1,-21) (-2,1,21), (-2,1,-21)								
**Quartz**	**(4,-7,5)**	**10.556**	**76.18**	**4.619**	**4**	**2.674**	**10.59**	**33.43**	**35.07**
**Quartz**	**(4,3,-5)**	**10.556**	**76.18**	**4.618**	**4**	**2.674**	**10.59**	**33.43**	**35.07**
	Equiv. Refl.: (-7,3,5), (-7,4,-5)								
**Quartz**	**(3,4,5)**	**10.556**	**76.18**	**4.616**	**4**	**2.674**	**10.59**	**33.43**	**35.06**
	Equiv. Refl.: (3,-7,-5)								
**Quartz**	**(4,4,2)**	**10.352**	**72.22**	**4.588**	**3.7**	**3.486**	**12.97**	**43.58**	**45.47**
	Equiv. Refl.: (4,4,-2)								
**Quartz**	**(4,-8,2)**	**10.352**	**72.22**	**4.587**	**3.7**	**3.486**	**12.97**	**43.58**	**45.47**
	Equiv. Refl.: (4,-8,-2), (-8,4,2), (-8,4,-2)								
**Quartz**	**(4,2,6)**	**10.335**	**71.92**	**4.583**	**3.6**	**3.548**	**12.91**	**44.35**	**46.19**
	Equiv. Refl.: (4,-6,-6)								
**Quartz**	**(-6,2,-6)**	**10.335**	**71.92**	**4.58**	**3.6**	**3.548**	**12.9**	**44.35**	**46.19**
**Quartz**	**(-6,4,6)**	**10.335**	**71.92**	**4.579**	**3.6**	**3.548**	**12.9**	**44.35**	**46.19**

Ei = Cryst	10.871 Refl (h,k,l)	keV EB [keV]	ΘB [°]	∫ IR dΘ [µrad]	Width [µrad]	Ei cotΘ [meV/µrad]	ΔE [meV]	ΔEg 2m, 50µm [meV]	ΔEt 2m, 50µm [meV]
Quartz	(2,4,-6)	10.335	71.92	4.578	3.6	3.548	12.9	44.35	46.19
	Equiv. Refl.: (2,-6,6)								
Quartz	(-4,8,2)	10.352	72.22	4.534	3.7	3.486	12.97	43.58	45.47
	Equiv. Refl.: (-4,8,-2), (-4,-4,2), (-4,-4,-2)								
Quartz	(8,-4,2)	10.352	72.22	4.533	3.7	3.486	12.97	43.58	45.47
	Equiv. Refl.: (8,-4,-2)								
Quartz	(4,-1,8)	10.573	76.56	4.517	3.8	2.597	9.74	32.46	33.89
	Equiv. Refl.: (4,-3,-8)								
Quartz	(-3,-1,-8)	10.573	76.56	4.515	3.8	2.597	9.74	32.46	33.89
Quartz	(-3,4,8)	10.573	76.56	4.513	3.7	2.597	9.74	32.46	33.89
Quartz	(-1,4,-8)	10.573	76.56	4.512	3.7	2.597	9.73	32.46	33.89
	Equiv. Refl.: (-1,-3,8)								
Quartz	(5,-7,-5)	10.756	81.64	4.481	3.9	1.597	6.3	19.97	20.94
Quartz	(5,2,5)	10.756	81.64	4.48	3.9	1.597	6.3	19.97	20.94
Quartz	(-7,2,-5)	10.756	81.64	4.478	3.9	1.597	6.29	19.97	20.94
Quartz	(-7,5,5)	10.756	81.64	4.477	3.9	1.597	6.29	19.97	20.94
Quartz	(2,5,-5)	10.756	81.64	4.474	3.9	1.597	6.29	19.97	20.93
	Equiv. Refl.: (2,-7,5)								
Quartz	(-4,6,6)	10.335	71.92	4.328	3.6	3.548	12.91	44.35	46.19
Quartz	(-4,-2,-6)	10.335	71.92	4.327	3.6	3.548	12.91	44.35	46.19
Quartz	(6,-2,6)	10.335	71.92	4.326	3.6	3.548	12.9	44.35	46.19
Quartz	(6,-4,-6)	10.335	71.92	4.324	3.6	3.548	12.9	44.35	46.19
Quartz	(-2,6,-6)	10.335	71.92	4.323	3.6	3.548	12.9	44.35	46.19
	Equiv. Refl.: (-2,-4,6)								
Quartz	(5,3,2)	10.454	74.07	4.264	3.6	3.102	11.08	38.78	40.33
	Equiv. Refl.: (5,-8,-2)								
Quartz	(3,-8,2)	10.454	74.07	4.263	3.6	3.102	11.08	38.78	40.33
Quartz	(3,5,-2)	10.454	74.07	4.262	3.6	3.102	11.08	38.78	40.33
	Equiv. Refl.: (-8,3,-2), (-8,5,2)								
Quartz	(-4,1,-8)	10.573	76.56	4.25	3.8	2.597	9.74	32.46	33.89
	Equiv. Refl.: (-4,3,8)								
Quartz	(3,1,8)	10.573	76.56	4.249	3.8	2.597	9.74	32.46	33.89
Quartz	(3,-4,-8)	10.573	76.56	4.247	3.7	2.597	9.74	32.46	33.89
Quartz	(1,3,-8)	10.573	76.56	4.246	3.7	2.597	9.73	32.46	33.89
	Equiv. Refl.: (1,-4,8)								
Quartz	(-5,8,2)	10.454	74.07	4.244	3.6	3.102	11.08	38.78	40.33
	Equiv. Refl.: (-5,-3,-2)								
Quartz	(8,-3,2)	10.454	74.07	4.242	3.6	3.102	11.08	38.78	40.33
	Equiv. Refl.: (8,-5,-2), (-3,8,-2), (-3,-5,2)								
Quartz	(-5,7,5)	10.756	81.64	3.832	3.9	1.597	6.3	19.97	20.94
	Equiv. Refl.: (-5,-2,-5)								
Quartz	(7,-2,5)	10.756	81.64	3.828	3.9	1.597	6.29	19.97	20.94
Quartz	(7,-5,-5)	10.756	81.64	3.827	3.9	1.597	6.29	19.97	20.94
Quartz	(-2,7,-5)	10.756	81.64	3.826	3.9	1.597	6.29	19.97	20.93
	Equiv. Refl.: (-2,-5,5)								
Quartz	(8,-6,-1)	10.569	76.46	3.613	3.4	2.618	8.82	32.72	33.89
Quartz	(8,-2,1)	10.569	76.46	3.612	3.4	2.618	8.82	32.72	33.89
	Equiv. Refl.: (-2,8,-1), (-2,-6,1), (-6,8,1), (-6,-2,-1)								
Quartz	(2,6,-2)	10.754	81.59	3.572	3.2	1.608	5.11	20.1	20.74
	Equiv. Refl.: (-8,2,-2), (-8,6,2)								

Ei =	10.871	keV						ΔEg	ΔEt
Cryst	Refl	EB	ΘB	∫ IR dΘ	Width	Ei cotΘ	ΔE	2m, 50µm	2m, 50µm
	(h,k,l)	[keV]	[°]	[µrad]	[µrad]	[meV/µrad]	[meV]	[meV]	[meV]
**Quartz**	**(2,-8,2)**	**10.754**	**81.59**	**3.571**	**3.2**	**1.608**	**5.11**	**20.1**	**20.74**
	Equiv. Refl.: (6,2,2), (6,-8,-2)								
**Quartz**	**(1,1,-9)**	**10.627**	**77.84**	**3.569**	**3.1**	**2.342**	**7.28**	**29.28**	**30.17**
**Quartz**	**(1,-2,9)**	**10.627**	**77.84**	**3.568**	**3.1**	**2.342**	**7.28**	**29.28**	**30.17**
**Quartz**	**(-2,1,-9)**	**10.627**	**77.84**	**3.559**	**3.1**	**2.342**	**7.26**	**29.28**	**30.16**
**Quartz**	**(-2,1,9)**	**10.627**	**77.84**	**3.558**	**3.1**	**2.342**	**7.26**	**29.28**	**30.16**
**Quartz**	**(1,1,9)**	**10.627**	**77.84**	**3.556**	**3.1**	**2.342**	**7.26**	**29.28**	**30.16**
	Equiv. Refl.: (1,-2,-9)								
**Quartz**	**(6,-2,-6)**	**10.335**	**71.92**	**3.514**	**2.8**	**3.548**	**10.07**	**44.35**	**45.48**
	Equiv. Refl.: (-2,6,6), (-2,-4,-6)								
**Quartz**	**(6,-4,6)**	**10.335**	**71.92**	**3.513**	**2.8**	**3.548**	**10.07**	**44.35**	**45.48**
**Quartz**	**(-4,6,-6)**	**10.335**	**71.92**	**3.511**	**2.8**	**3.548**	**10.07**	**44.35**	**45.48**
**Quartz**	**(-4,-2,6)**	**10.335**	**71.92**	**3.51**	**2.8**	**3.548**	**10.06**	**44.35**	**45.48**
**Quartz**	**(2,6,-1)**	**10.569**	**76.46**	**3.509**	**3.4**	**2.618**	**8.82**	**32.72**	**33.89**
	Equiv. Refl.: (2,-8,1), (6,2,1), (6,-8,-1), (-8,2,-1), (-8,6,1)								
**Quartz**	**(-1,-1,9)**	**10.627**	**77.84**	**3.424**	**3.1**	**2.342**	**7.28**	**29.28**	**30.17**
**Quartz**	**(-1,2,-9)**	**10.627**	**77.84**	**3.423**	**3.1**	**2.342**	**7.28**	**29.28**	**30.17**
**Quartz**	**(2,-1,9)**	**10.627**	**77.84**	**3.414**	**3.1**	**2.342**	**7.26**	**29.28**	**30.16**
**Quartz**	**(2,-1,-9)**	**10.627**	**77.84**	**3.412**	**3.1**	**2.342**	**7.26**	**29.28**	**30.16**
**Sapph**	**(4,4,3)**	**10.521**	**75.42**	**3.411**	**3.1**	**2.828**	**8.67**	**35.35**	**36.4**
	Equiv. Refl.: (4,4,-3), (4,-8,3), (4,-8,-3), (8,-4,3), (8,-4,-3), (-4,8,3), (-4,8,-3), (-4,-4,3), (-4,-4,-3) (-8,4,3), (-8,4,-3), (-1,2,9)								
**Quartz**	**(-1,-1,-9)**	**10.627**	**77.84**	**3.41**	**3.1**	**2.342**	**7.26**	**29.28**	**30.16**
**Quartz**	**(6,-5,-6)**	**10.638**	**78.12**	**3.357**	**3.2**	**2.287**	**7.38**	**28.59**	**29.52**
**Quartz**	**(6,-1,6)**	**10.638**	**78.12**	**3.356**	**3.2**	**2.287**	**7.38**	**28.59**	**29.52**
	Equiv. Refl.: (-5,6,6), (-5,-1,-6)								
**Quartz**	**(-1,-5,6)**	**10.638**	**78.12**	**3.355**	**3.2**	**2.287**	**7.37**	**28.59**	**29.52**
**Quartz**	**(-1,6,-6)**	**10.638**	**78.12**	**3.354**	**3.2**	**2.287**	**7.37**	**28.59**	**29.52**
**Quartz**	**(0,2,9)**	**10.727**	**80.65**	**3.35**	**3.3**	**1.79**	**5.94**	**22.38**	**23.15**
	Equiv. Refl.: (0,-2,-9)								
**Quartz**	**(2,-2,9)**	**10.727**	**80.65**	**3.349**	**3.3**	**1.79**	**5.94**	**22.38**	**23.15**
**Quartz**	**(2,0,-9)**	**10.727**	**80.65**	**3.348**	**3.3**	**1.79**	**5.94**	**22.38**	**23.15**
	Equiv. Refl.: (-2,2,-9)								
**Quartz**	**(-2,0,9)**	**10.727**	**80.65**	**3.347**	**3.3**	**1.79**	**5.94**	**22.38**	**23.15**
**Quartz**	**(2,4,6)**	**10.335**	**71.92**	**3.287**	**2.8**	**3.548**	**10.07**	**44.35**	**45.48**
	Equiv. Refl.: (-6,2,6)								
**Quartz**	**(2,-6,-6)**	**10.335**	**71.92**	**3.286**	**2.8**	**3.548**	**10.07**	**44.35**	**45.48**
	Equiv. Refl.: (-6,4,-6)								
**Quartz**	**(4,-6,6)**	**10.335**	**71.92**	**3.284**	**2.8**	**3.548**	**10.07**	**44.35**	**45.48**
**Quartz**	**(4,2,-6)**	**10.335**	**71.92**	**3.283**	**2.8**	**3.548**	**10.06**	**44.35**	**45.48**
**Quartz**	**(5,1,6)**	**10.638**	**78.12**	**3.259**	**3.2**	**2.287**	**7.38**	**28.59**	**29.52**
	Equiv. Refl.: (-6,5,6)								
**Quartz**	**(1,5,-6)**	**10.638**	**78.12**	**3.258**	**3.2**	**2.287**	**7.37**	**28.59**	**29.52**
	Equiv. Refl.: (5,-6,-6), (-6,1,-6)								
**Quartz**	**(1,-6,6)**	**10.638**	**78.12**	**3.257**	**3.2**	**2.287**	**7.37**	**28.59**	**29.52**
**Quartz**	**(0,1,9)**	**10.425**	**73.54**	**3.148**	**2.6**	**3.212**	**8.51**	**40.15**	**41.04**
	Equiv. Refl.: (0,-1,-9)								
**Sapph**	**(3,5,-5)**	**10.798**	**83.35**	**3.147**	**3.7**	**1.267**	**4.7**	**15.83**	**16.51**
	Equiv. Refl.: (3,-8,5), (5,3,5), (5,-8,-5), (8,-3,5), (8,-5,-5), (-3,8,-5), (-3,-5,5), (-5,8,5), (-5,-3,-5) (-8,3,-5), (-8,5,5)								

Ei = Cryst	10.871 Refl (h,k,l)	keV EB [keV]	ΘB [°]	∫ IR dΘ [µrad]	Width [µrad]	Ei cotΘ [meV/µrad]	ΔE [meV]	ΔEg 2m, 50µm [meV]	ΔEt 2m, 50µm [meV]
**Quartz**	**(1,0,-9)**	**10.425**	**73.54**	**3.137**	**2.6**	**3.212**	**8.49**	**40.15**	**41.04**
	Equiv. Refl.: (1,-1,9), (-1,0,9), (-1,1,-9)								
**Quartz**	**(5,-1,-7)**	**10.443**	**73.86**	**3.128**	**2.6**	**3.146**	**8.24**	**39.33**	**40.18**
	Equiv. Refl.: (5,-4,7)								
**Quartz**	**(3,3,6)**	**10.231**	**70.25**	**3.127**	**2.9**	**3.904**	**11.24**	**48.8**	**50.08**
	Equiv. Refl.: (3,-6,-6)								
**Quartz**	**(3,3,-6)**	**10.231**	**70.25**	**3.126**	**2.9**	**3.904**	**11.24**	**48.8**	**50.08**
	Equiv. Refl.: (3,-6,6), (-6,3,6), (-6,3,-6)								
**Quartz**	**(6,-3,-6)**	**10.231**	**70.25**	**3.122**	**2.9**	**3.904**	**11.24**	**48.8**	**50.08**
	Equiv. Refl.: (-3,6,6)								
**Quartz**	**(6,-3,6)**	**10.231**	**70.25**	**3.121**	**2.9**	**3.904**	**11.24**	**48.8**	**50.08**
	Equiv. Refl.: (-3,6,-6), (-3,-3,6), (-3,-3,-6)								
**Quartz**	**(-4,5,-7)**	**10.443**	**73.86**	**3.12**	**2.6**	**3.146**	**8.22**	**39.33**	**40.18**
	Equiv. Refl.: (-4,-1,7)								
**Quartz**	**(-1,-4,-7)**	**10.443**	**73.86**	**3.119**	**2.6**	**3.146**	**8.22**	**39.33**	**40.18**
**Quartz**	**(-1,5,7)**	**10.443**	**73.86**	**3.118**	**2.6**	**3.146**	**8.22**	**39.33**	**40.18**
**LiNbO3**	**(1,5,17)**	**10.85**	**86.46**	**3.085**	**7.5**	**0.673**	**5.04**	**8.41**	**9.8**
	Equiv. Refl.: (1,-6,-17), (5,1,-17), (5,-6,17), (6,-1,-17), (6,-5,17), (-1,6,17), (-1,-5,-17), (-5,6,-17), (-5,-1,17) (-6,1,17), (-6,5,-17)								
**Quartz**	**(8,-2,2)**	**10.754**	**81.59**	**3.081**	**3.2**	**1.608**	**5.11**	**20.1**	**20.74**
	Equiv. Refl.: (8,-6,-2), (-2,-6,2)								
**Quartz**	**(-2,8,-2)**	**10.754**	**81.59**	**3.08**	**3.2**	**1.608**	**5.11**	**20.1**	**20.74**
**Quartz**	**(-6,8,2)**	**10.754**	**81.59**	**3.079**	**3.2**	**1.608**	**5.11**	**20.1**	**20.74**
	Equiv. Refl.: (-6,-2,-2)								
**Quartz**	**(-5,1,7)**	**10.443**	**73.86**	**3.052**	**2.6**	**3.146**	**8.24**	**39.33**	**40.18**
	Equiv. Refl.: (-5,4,-7)								
**Quartz**	**(4,1,-7)**	**10.443**	**73.86**	**3.044**	**2.6**	**3.146**	**8.22**	**39.33**	**40.18**
	Equiv. Refl.: (4,-5,7)								
**Quartz**	**(1,4,7)**	**10.443**	**73.86**	**3.042**	**2.6**	**3.146**	**8.22**	**39.33**	**40.18**
	Equiv. Refl.: (1,-5,-7)								
**Quartz**	**(-5,8,-2)**	**10.454**	**74.07**	**2.951**	**2.5**	**3.102**	**7.82**	**38.78**	**39.56**
	Equiv. Refl.: (-5,-3,2)								
**Quartz**	**(8,-3,-2)**	**10.454**	**74.07**	**2.949**	**2.5**	**3.102**	**7.82**	**38.78**	**39.56**
	Equiv. Refl.: (8,-5,2), (-3,-5,-2)								
**Quartz**	**(-3,8,2)**	**10.454**	**74.07**	**2.948**	**2.5**	**3.102**	**7.82**	**38.78**	**39.56**
**Quartz**	**(5,3,-2)**	**10.454**	**74.07**	**2.931**	**2.5**	**3.102**	**7.82**	**38.78**	**39.56**
	Equiv. Refl.: (5,-8,2)								
**Quartz**	**(3,5,2)**	**10.454**	**74.07**	**2.929**	**2.5**	**3.102**	**7.82**	**38.78**	**39.56**
	Equiv. Refl.: (-8,3,2), (-8,5,-2)								
**Quartz**	**(3,-8,-2)**	**10.454**	**74.07**	**2.928**	**2.5**	**3.102**	**7.82**	**38.78**	**39.56**
**Sapph**	**(1,5,-13)**	**10.424**	**73.51**	**2.656**	**2.7**	**3.217**	**8.57**	**40.22**	**41.12**
	Equiv. Refl.: (1,-6,13), (5,1,13), (5,-6,-13), (6,-1,13), (6,-5,-13), (-1,6,-13), (-1,-5,13), (-5,6,13), (-5,-1,-13) (-6,1,-13), (-6,5,13)								
**Sapph**	**(2,5,9)**	**10.331**	**71.86**	**2.558**	**2.5**	**3.562**	**9.06**	**44.53**	**45.44**
	Equiv. Refl.: (2,5,-9), (2,-7,9), (2,-7,-9), (5,2,9), (5,2,-9), (5,-7,9), (5,-7,-9), (7,-2,9), (7,-2,-9) (7,-5,9), (7,-5,-9), (-2,7,9), (-2,7,-9), (-2,-5,9), (-2,-5,-9), (-5,7,9), (-5,7,-9), (-5,-2,9) (-5,-2,-9), (-7,2,9), (-7,2,-9), (-7,5,9), (-7,5,-9)								
**Quartz**	**(0,7,3)**	**10.764**	**81.95**	**2.53**	**2.8**	**1.538**	**4.31**	**19.23**	**19.71**
	Equiv. Refl.: (0,-7,-3), (-7,7,-3)								

Ei =	10.871	keV						ΔEg	ΔEt
Cryst	Refl	EB	ΘB	∫ IR dΘ	Width	Ei cotΘ	ΔE	2m, 50μm	2m, 50μm
	(h,k,l)	[keV]	[°]	[μrad]	[μrad]	[meV/μrad]	[meV]	[meV]	[meV]
Quartz	(7,0,-3)	10.764	81.95	2.529	2.8	1.538	4.3	19.23	19.71
	Equiv. Refl.: (7,-7,3), (-7,0,3)								
LiNbO3	(1,6,-11)	10.36	72.37	2.273	3.5	3.455	12.05	43.19	44.84
	Equiv. Refl.: (1,-7,11), (6,1,11), (6,-7,-11), (7,-1,11), (7,-6,-11), (-1,7,-11), (-1,-6,11), (-6,7,11), (-6,-1,-11) (-7,1,-11), (-7,6,11)								
LiNbO3	(2,4,-17)	10.58	76.7	2.233	3.7	2.569	9.6	32.11	33.52
	Equiv. Refl.: (2,-6,17), (4,2,17), (4,-6,-17), (6,-2,17), (6,-4,-17), (-2,6,-17), (-2,-4,17), (-4,6,17), (-4,-2,-17) (-6,2,-17), (-6,4,17)								
Quartz	(-5,5,7)	10.841	85.77	2.098	3.1	0.803	2.46	10.04	10.34
Quartz	(5,-5,-7)	10.841	85.77	2.097	3.1	0.803	2.46	10.04	10.34
Quartz	(-5,0,-7)	10.841	85.77	2.096	3.1	0.803	2.46	10.04	10.34
Quartz	(5,0,7)	10.841	85.77	2.095	3.1	0.803	2.46	10.04	10.34
Quartz	(0,7,-3)	10.764	81.95	2.088	2.6	1.538	4.01	19.23	19.64
	Equiv. Refl.: (0,-7,3), (7,0,3), (7,-7,-3), (-7,0,-3), (-7,7,3)								
Quartz	(0,5,-7)	10.841	85.77	2.074	3	0.803	2.44	10.04	10.33
	Equiv. Refl.: (0,-5,7)								
LiNbO3	(2,2,21)	10.554	76.13	2.013	3.4	2.684	9.25	33.55	34.81
	Equiv. Refl.: (2,2,-21), (2,-4,21), (2,-4,-21), (4,-2,21), (4,-2,-21), (-2,4,21), (-2,4,-21), (-2,-2,21), (-2,-2,-21) (-4,2,21), (-4,2,-21)								
Quartz	(7,0,2)	10.454	74.07	1.96	2.2	3.102	6.83	38.78	39.37
	Equiv. Refl.: (7,-7,-2), (-7,0,-2), (-7,7,2)								
Quartz	(0,7,-2)	10.454	74.07	1.959	2.2	3.102	6.83	38.78	39.37
	Equiv. Refl.: (0,-7,2)								
Quartz	(4,-1,-8)	10.573	76.56	1.913	2.2	2.597	5.69	32.46	32.96
	Equiv. Refl.: (4,-3,8), (-1,-3,-8)								
Quartz	(-1,4,8)	10.573	76.56	1.912	2.2	2.597	5.68	32.46	32.96
Quartz	(-3,-1,8)	10.573	76.56	1.91	2.2	2.597	5.68	32.46	32.96
Quartz	(-3,4,-8)	10.573	76.56	1.909	2.2	2.597	5.68	32.46	32.96
Quartz	(1,3,8)	10.573	76.56	1.865	2.2	2.597	5.69	32.46	32.96
	Equiv. Refl.: (-4,3,-8)								
Quartz	(1,-4,-8)	10.573	76.56	1.864	2.2	2.597	5.68	32.46	32.96
	Equiv. Refl.: (-4,1,8)								
Quartz	(2,6,0)	10.506	75.12	1.863	2	2.888	5.8	36.1	36.57
	Equiv. Refl.: (2,-8,0), (6,2,0), (6,-8,0), (-8,2,0), (-8,6,0)								
Quartz	(3,1,-8)	10.573	76.56	1.861	2.2	2.597	5.68	32.46	32.96
	Equiv. Refl.: (3,-4,8)								
Quartz	(5,-1,7)	10.443	73.86	1.807	2	3.146	6.3	39.33	39.83
	Equiv. Refl.: (5,-4,-7)								
Quartz	(-4,-1,-7)	10.443	73.86	1.806	2	3.146	6.3	39.33	39.83
Quartz	(-1,-4,7)	10.443	73.86	1.805	2	3.146	6.29	39.33	39.83
	Equiv. Refl.: (-4,5,7)								
Quartz	(-1,5,-7)	10.443	73.86	1.804	2	3.146	6.29	39.33	39.83
Quartz	(-5,1,-7)	10.443	73.86	1.702	2	3.146	6.3	39.33	39.83
	Equiv. Refl.: (-5,4,7)								
Quartz	(4,1,7)	10.443	73.86	1.701	2	3.146	6.3	39.33	39.83
Quartz	(1,4,-7)	10.443	73.86	1.7	2	3.146	6.29	39.33	39.83
	Equiv. Refl.: (1,-5,7), (4,-5,-7)								
Quartz	(8,-2,0)	10.506	75.12	1.611	2	2.888	5.8	36.1	36.57
	Equiv. Refl.: (8,-6,0), (-2,8,0), (-2,-6,0), (-6,8,0), (-6,-2,0)								
Quartz	(3,4,-5)	10.556	76.18	1.561	1.8	2.674	4.72	33.43	33.76
	Equiv. Refl.: (3,-7,5), (-7,3,-5)								

Ei =	10.871	keV						ΔEg	ΔEt
Cryst	Refl	EB	ΘB	∫ IR dΘ	Width	Ei cotΘ	ΔE	2m, 50μm	2m, 50μm
	(h,k,l)	[keV]	[°]	[μrad]	[μrad]	[meV/μrad]	[meV]	[meV]	[meV]
**Quartz**	**(4,3,5)**	**10.556**	**76.18**	**1.56**	**1.8**	**2.674**	**4.72**	**33.43**	**33.76**
	Equiv. Refl.: (4,-7,-5), (-7,4,5)								
**Quartz**	**(-2,4,-8)**	**10.473**	**74.44**	**1.558**	**1.8**	**3.027**	**5.31**	**37.84**	**38.21**
	Equiv. Refl.: (-2,-2,8)								
**Quartz**	**(4,-2,-8)**	**10.473**	**74.44**	**1.557**	**1.8**	**3.027**	**5.31**	**37.84**	**38.21**
**Quartz**	**(4,-2,8)**	**10.473**	**74.44**	**1.556**	**1.8**	**3.027**	**5.31**	**37.84**	**38.21**
**Quartz**	**(-2,4,8)**	**10.473**	**74.44**	**1.553**	**1.8**	**3.027**	**5.3**	**37.84**	**38.21**
	Equiv. Refl.: (-2,-2,-8)								
**Sapph**	**(3,5,1)**	**10.542**	**75.86**	**1.467**	**1.9**	**2.738**	**5.12**	**34.23**	**34.61**
	Equiv. Refl.: (3,-8,-1), (5,3,-1), (5,-8,1), (8,-3,-1), (8,-5,1), (-3,8,1), (-3,-5,-1), (-5,8,-1), (-5,-3,1) (-8,3,1), (-8,5,-1)								
**Quartz**	**(2,-4,8)**	**10.473**	**74.44**	**1.415**	**1.8**	**3.027**	**5.31**	**37.84**	**38.21**
**Quartz**	**(2,2,-8)**	**10.473**	**74.44**	**1.414**	**1.8**	**3.027**	**5.31**	**37.84**	**38.21**
**Quartz**	**(-4,2,8)**	**10.473**	**74.44**	**1.413**	**1.8**	**3.027**	**5.31**	**37.84**	**38.21**
**Quartz**	**(-4,2,-8)**	**10.473**	**74.44**	**1.412**	**1.8**	**3.027**	**5.31**	**37.84**	**38.21**
**Quartz**	**(2,2,8)**	**10.473**	**74.44**	**1.409**	**1.8**	**3.027**	**5.3**	**37.84**	**38.21**
	Equiv. Refl.: (2,-4,-8)								
**Quartz**	**(7,-3,5)**	**10.556**	**76.18**	**1.363**	**1.8**	**2.674**	**4.72**	**33.43**	**33.76**
**Quartz**	**(7,-4,-5)**	**10.556**	**76.18**	**1.362**	**1.8**	**2.674**	**4.72**	**33.43**	**33.76**
	Equiv. Refl.: (-3,7,-5), (-3,-4,5)								
**Quartz**	**(-4,7,5)**	**10.556**	**76.18**	**1.361**	**1.8**	**2.674**	**4.72**	**33.43**	**33.76**
	Equiv. Refl.: (-4,-3,-5)								
**Quartz**	**(-7,1,4)**	**10.599**	**77.15**	**1.349**	**1.7**	**2.48**	**4.14**	**31**	**31.28**
	Equiv. Refl.: (-7,6,-4)								
**Quartz**	**(6,1,-4)**	**10.599**	**77.15**	**1.348**	**1.7**	**2.48**	**4.14**	**31**	**31.28**
	Equiv. Refl.: (6,-7,4)								
**Quartz**	**(1,6,4)**	**10.599**	**77.15**	**1.347**	**1.7**	**2.48**	**4.13**	**31**	**31.28**
	Equiv. Refl.: (1,-7,-4)								
**Quartz**	**(7,-1,-4)**	**10.599**	**77.15**	**1.326**	**1.7**	**2.48**	**4.14**	**31**	**31.28**
	Equiv. Refl.: (7,-6,4)								
**Quartz**	**(-6,7,-4)**	**10.599**	**77.15**	**1.325**	**1.7**	**2.48**	**4.13**	**31**	**31.28**
	Equiv. Refl.: (-6,-1,4)								
**Quartz**	**(-1,7,4)**	**10.599**	**77.15**	**1.324**	**1.7**	**2.48**	**4.13**	**31**	**31.28**
	Equiv. Refl.: (-1,-6,-4)								
**Sapph**	**(2,3,17)**	**10.433**	**73.67**	**1.232**	**1.6**	**3.185**	**5.05**	**39.81**	**40.13**
	Equiv. Refl.: (2,-5,-17), (3,2,-17), (3,-5,17), (5,-2,-17), (5,-3,17), (-2,5,17), (-2,-3,-17), (-3,5,-17), (-3,-2,17) (-5,2,17), (-5,3,-17)								
**Quartz**	**(-2,7,5)**	**10.756**	**81.64**	**1.137**	**1.6**	**1.597**	**2.5**	**19.97**	**20.12**
	Equiv. Refl.: (-2,-5,-5)								
**Quartz**	**(-5,-2,5)**	**10.756**	**81.64**	**1.134**	**1.6**	**1.597**	**2.49**	**19.97**	**20.12**
**Quartz**	**(7,-2,-5)**	**10.756**	**81.64**	**1.133**	**1.6**	**1.597**	**2.49**	**19.97**	**20.12**
	Equiv. Refl.: (-5,7,-5)								
**Quartz**	**(7,-5,5)**	**10.756**	**81.64**	**1.132**	**1.6**	**1.597**	**2.49**	**19.97**	**20.12**
**Sapph**	**(0,7,-4)**	**10.703**	**79.9**	**1.12**	**1.6**	**1.936**	**3.18**	**24.2**	**24.41**
	Equiv. Refl.: (0,-7,4), (7,0,4), (7,-7,-4), (-7,0,-4), (-7,7,4)								
**Quartz**	**(6,0,5)**	**10.455**	**74.1**	**1.08**	**1.4**	**3.096**	**4.19**	**38.7**	**38.93**
	Equiv. Refl.: (6,-6,-5), (-6,0,-5), (-6,6,5)								
**Quartz**	**(0,6,-5)**	**10.455**	**74.1**	**1.077**	**1.4**	**3.096**	**4.18**	**38.7**	**38.93**
	Equiv. Refl.: (0,-6,5)								

Ei =	10.871	keV						ΔEg	ΔEt
Cryst	Refl	EB	ΘB	∫ IR dΘ	Width	Ei cotΘ	ΔE	2m, 50μm	2m, 50μm
	(h,k,l)	[keV]	[°]	[μrad]	[μrad]	[meV/μrad]	[meV]	[meV]	[meV]
**Quartz**	**(5,-2,-7)**	**10.237**	**70.34**	**1.035**	**1.2**	**3.884**	**4.7**	**48.55**	**48.78**
	Equiv. Refl.: (-2,-3,-7)								
**Quartz**	**(5,-3,7)**	**10.237**	**70.34**	**1.034**	**1.2**	**3.884**	**4.7**	**48.55**	**48.78**
	Equiv. Refl.: (-2,5,7)								
**Quartz**	**(-3,5,-7)**	**10.237**	**70.34**	**1.033**	**1.2**	**3.884**	**4.69**	**48.55**	**48.78**
	Equiv. Refl.: (-3,-2,7)								
**Quartz**	**(2,5,5)**	**10.756**	**81.64**	**1.009**	**1.6**	**1.597**	**2.5**	**19.97**	**20.12**
	Equiv. Refl.: (2,-7,-5)								
**Quartz**	**(5,2,-5)**	**10.756**	**81.64**	**1.005**	**1.6**	**1.597**	**2.49**	**19.97**	**20.12**
	Equiv. Refl.: (5,-7,5), (-7,2,5)								
**Quartz**	**(-7,5,-5)**	**10.756**	**81.64**	**1.004**	**1.6**	**1.597**	**2.49**	**19.97**	**20.12**
**LiNbO3**	**(3,4,-13)**	**10.263**	**70.75**	**1.002**	**2**	**3.796**	**7.55**	**47.46**	**48.05**
	Equiv. Refl.: (3,-7,13), (4,3,13), (4,-7,-13), (7,-3,13), (7,-4,-13), (-3,7,-13), (-3,-4,13), (-4,7,13), (-4,-3,-13) (-7,3,-13), (-7,4,13)								
**Quartz**	**(7,0,1)**	**10.263**	**70.75**	**0.998**	**1.2**	**3.796**	**4.66**	**47.45**	**47.68**
	Equiv. Refl.: (7,-7,-1), (-7,0,-1), (-7,7,1)								
**Quartz**	**(0,7,-1)**	**10.263**	**70.75**	**0.997**	**1.2**	**3.796**	**4.66**	**47.45**	**47.68**
	Equiv. Refl.: (0,-7,1)								
**Quartz**	**(2,3,7)**	**10.237**	**70.34**	**0.944**	**1.2**	**3.884**	**4.7**	**48.55**	**48.78**
	Equiv. Refl.: (2,-5,-7), (-5,2,7), (-5,3,-7)								
**Quartz**	**(3,2,-7)**	**10.237**	**70.34**	**0.943**	**1.2**	**3.884**	**4.69**	**48.55**	**48.78**
	Equiv. Refl.: (3,-5,7)								
**Sapph**	**(1,6,7)**	**10.416**	**73.36**	**0.817**	**1.2**	**3.25**	**3.77**	**40.62**	**40.8**
	Equiv. Refl.: (1,-7,-7), (6,1,-7), (6,-7,7), (7,-1,-7), (7,-6,7), (-1,7,7), (-1,-6,-7), (-6,7,-7), (-6,-1,7) (-7,1,7), (-7,6,-7)								
**Quartz**	**(1,0,9)**	**10.425**	**73.54**	**0.805**	**1.1**	**3.212**	**3.44**	**40.15**	**40.3**
	Equiv. Refl.: (1,-1,-9), (-1,0,-9), (-1,1,9)								
**Quartz**	**(0,1,-9)**	**10.425**	**73.54**	**0.802**	**1.1**	**3.212**	**3.43**	**40.15**	**40.3**
	Equiv. Refl.: (0,-1,9)								
**Sapph**	**(1,3,19)**	**10.567**	**76.42**	**0.653**	**1**	**2.626**	**2.75**	**32.83**	**32.95**
	Equiv. Refl.: (1,-4,-19), (3,1,-19), (3,-4,19), (4,-1,-19), (4,-3,19), (-1,4,19), (-1,-3,-19), (-3,4,-19), (-3,-1,19) (-4,1,19), (-4,3,-19)								
**Quartz**	**(3,2,7)**	**10.237**	**70.34**	**0.618**	**0.8**	**3.884**	**3.11**	**48.55**	**48.65**
	Equiv. Refl.: (3,-5,-7)								
**Quartz**	**(-5,2,-7)**	**10.237**	**70.34**	**0.617**	**0.8**	**3.884**	**3.11**	**48.55**	**48.65**
	Equiv. Refl.: (-5,3,7)								
**Quartz**	**(2,3,-7)**	**10.237**	**70.34**	**0.616**	**0.8**	**3.884**	**3.1**	**48.55**	**48.65**
	Equiv. Refl.: (2,-5,7)								
**Quartz**	**(0,7,2)**	**10.454**	**74.07**	**0.543**	**0.8**	**3.102**	**2.52**	**38.78**	**38.86**
	Equiv. Refl.: (0,-7,-2), (7,0,-2), (7,-7,2), (-7,0,2), (-7,7,-2)								
**Quartz**	**(5,-2,7)**	**10.237**	**70.34**	**0.532**	**0.8**	**3.884**	**3.11**	**48.55**	**48.65**
	Equiv. Refl.: (-3,5,7), (-3,-2,-7)								
**Quartz**	**(5,-3,-7)**	**10.237**	**70.34**	**0.531**	**0.8**	**3.884**	**3.11**	**48.55**	**48.65**
	Equiv. Refl.: (-2,5,-7)								
**Quartz**	**(-2,-3,7)**	**10.237**	**70.34**	**0.53**	**0.8**	**3.884**	**3.1**	**48.55**	**48.65**
**LiNbO3**	**(2,6,-7)**	**10.504**	**75.07**	**0.445**	**1.4**	**2.898**	**3.98**	**36.22**	**36.44**
	Equiv. Refl.: (2,-8,7), (6,2,7), (6,-8,-7), (8,-2,7), (8,-6,-7), (-2,8,-7), (-2,-6,7), (-6,8,7), (-6,-2,-7) (-8,2,-7), (-8,6,7)								
**Quartz**	**(3,5,1)**	**10.263**	**70.75**	**0.441**	**0.6**	**3.796**	**2.41**	**47.45**	**47.51**
	Equiv. Refl.: (3,-8,-1), (5,3,-1), (5,-8,1)								
**Quartz**	**(-8,3,1)**	**10.263**	**70.75**	**0.44**	**0.6**	**3.796**	**2.4**	**47.45**	**47.51**
	Equiv. Refl.: (-8,5,-1)								

Ei =	10.871	keV						ΔEg	ΔEt
**Cryst**	**Refl**	**EB**	**ΘB**	**∫ IR dΘ**	**Width**	**Ei cotΘ**	**ΔE**	**2m, 50μm**	**2m, 50μm**
	**(h,k,l)**	**[keV]**	**[°]**	**[μrad]**	**[μrad]**	**[meV/μrad]**	**[meV]**	**[meV]**	**[meV]**
**Quartz**	**(8,-3,-1)**	**10.263**	**70.75**	**0.383**	**0.6**	**3.796**	**2.4**	**47.45**	**47.51**
	Equiv. Refl.: (8,-5,1), (-3,8,1), (-3,-5,-1), (-5,8,-1), (-5,-3,1)								
**LiNbO3**	**(2,6,5)**	**10.273**	**70.91**	**0.378**	**1.1**	**3.762**	**4.28**	**47.03**	**47.22**
	Equiv. Refl.: (2,-8,-5), (6,2,-5), (6,-8,5), (8,-2,-5), (8,-6,5), (-2,8,5), (-2,-6,-5), (-6,8,-5), (-6,-2,5)<br>(-8,2,5), (-8,6,-5)								
**Sapph**	**(0,1,-22)**	**10.608**	**77.36**	**0.341**	**0.7**	**2.437**	**1.73**	**30.47**	**30.52**
	Equiv. Refl.: (0,-1,22), (1,0,22), (1,-1,-22), (-1,0,-22), (-1,1,22)								
**LiNbO3**	**(1,7,3)**	**10.583**	**76.79**	**0.322**	**1.2**	**2.552**	**3.12**	**31.9**	**32.05**
	Equiv. Refl.: (1,7,-3), (1,-8,3), (1,-8,-3), (7,1,3), (7,1,-3), (7,-8,3), (7,-8,-3), (8,-1,3), (8,-1,-3)<br>(8,-7,3), (8,-7,-3), (-1,8,3), (-1,8,-3), (-1,-7,3), (-1,-7,-3), (-7,8,3), (-7,8,-3), (-7,-1,3)<br>(-7,-1,-3), (-8,1,3), (-8,1,-3), (-8,7,3), (-8,7,-3)								
**LiNbO3**	**(2,3,-19)**	**10.437**	**73.75**	**0.264**	**1**	**3.169**	**3.19**	**39.61**	**39.74**
	Equiv. Refl.: (2,-5,19), (3,2,19), (3,-5,-19), (5,-2,19), (5,-3,-19), (-2,5,-19), (-2,-3,19), (-3,5,19), (-3,-2,-19)<br>(-5,2,-19), (-5,3,19)								
**Sapph**	**(3,4,11)**	**10.55**	**76.05**	**0.234**	**0.5**	**2.701**	**1.47**	**33.77**	**33.8**
	Equiv. Refl.: (3,-7,-11), (4,3,-11), (4,-7,11), (7,-3,-11), (7,-4,11), (-3,7,11), (-3,-4,-11), (-4,7,-11), (-4,-3,11)<br>(-7,3,11), (-7,4,-11)								
**Quartz**	**(8,-3,1)**	**10.263**	**70.75**	**0.232**	**0.4**	**3.796**	**1.53**	**47.45**	**47.48**
	Equiv. Refl.: (8,-5,-1), (-3,8,-1), (-3,-5,1)								
**Quartz**	**(-5,8,1)**	**10.263**	**70.75**	**0.231**	**0.4**	**3.796**	**1.53**	**47.45**	**47.48**
	Equiv. Refl.: (-5,-3,-1)								
**LiNbO3**	**(3,5,7)**	**10.224**	**70.14**	**0.2**	**0.8**	**3.927**	**3.15**	**49.09**	**49.19**
	Equiv. Refl.: (3,-8,-7), (5,3,-7), (5,-8,7), (8,-3,-7), (8,-5,7), (-3,8,7), (-3,-5,-7), (-5,8,-7), (-5,-3,7)<br>(-8,3,7), (-8,5,-7)								
**Quartz**	**(3,5,-1)**	**10.263**	**70.75**	**0.185**	**0.4**	**3.796**	**1.53**	**47.45**	**47.48**
	Equiv. Refl.: (3,-8,1), (-8,3,-1), (-8,5,1)								
**Quartz**	**(5,3,1)**	**10.263**	**70.75**	**0.184**	**0.4**	**3.796**	**1.53**	**47.45**	**47.48**
	Equiv. Refl.: (5,-8,-1)								
**Sapph**	**(3,3,15)**	**10.6**	**77.19**	**0.052**	**0.3**	**2.473**	**0.63**	**30.91**	**30.92**
	Equiv. Refl.: (3,3,-15), (3,-6,15), (3,-6,-15), (6,-3,15), (6,-3,-15), (-3,6,15), (-3,6,-15), (-3,-3,15), (-3,-3,-15)<br>(-6,3,15), (-6,3,-15)								
**LiNbO3**	**(4,4,9)**	**10.44**	**73.82**	**0.018**	**0.3**	**3.154**	**0.92**	**39.43**	**39.44**
	Equiv. Refl.: (4,4,-9), (4,-8,9), (4,-8,-9), (8,-4,9), (8,-4,-9), (-4,8,9), (-4,8,-9), (-4,-4,9), (-4,-4,-9)<br>(-8,4,9), (-8,4,-9)								

Ei =	13.419	keV						ΔEg	ΔEt
Cryst	Refl	EB	ΘB	∫ IR dΘ	Width	Ei cotΘ	ΔE	2m, 50µm	2m, 50µm
	(h,k,l)	[keV]	[°]	[µrad]	[µrad]	[meV/µrad]	[meV]	[meV]	[meV]
Si	(0,6,10)	13.311	82.74	5.595	4.7	1.71	8.1	21.37	22.85
	Equiv. Refl.: (6,6,8)								
Ge	(0,0,12)	13.149	78.49	3.011	6.5	2.731	17.8	34.14	38.5
	Equiv. Refl.: (4,4,8)								
Si	(0,8,8)	12.914	74.23	2.648	2.4	3.789	9.27	47.37	48.27
Ge	(0,6,10)	12.779	72.23	2.239	4.7	4.3	20.11	53.75	57.39
	Equiv. Refl.: (6,6,8)								
Ge	(7,7,7)	13.286	81.92	2.183	6.3	1.906	12.04	23.83	26.7
	Equiv. Refl.: (1,5,11)								
Si	(1,3,11)	13.064	76.8	1.786	2	3.148	6.23	39.34	39.83
	Equiv. Refl.: (1,7,9), (5,5,9)								
Si	(1,1,11)	12.659	70.63	1.386	1.5	4.718	7.16	58.98	59.41
	Equiv. Refl.: (5,7,7)								
Ge	(3,3,11)	12.919	74.31	1.305	3.6	3.769	13.57	47.11	49.03
	Equiv. Refl.: (3,7,9)								
LiNbO3	(1,-7,-22)	13.414	88.36	49.9	46.4	0.384	17.81	4.79	18.45
	Equiv. Refl.: (6,1,-22), (7,-1,-22), (-1,-6,-22), (-6,7,-22), (-7,6,-22)								
LiNbO3	(1,6,22)	13.414	88.36	47.8	46.4	0.384	17.81	4.79	18.45
	Equiv. Refl.: (6,-7,22), (7,-6,22), (-1,7,22), (-6,-1,22), (-7,1,22)								
LiNbO3	(4,-11,0)	13.409	87.83	40.1	37.4	0.508	19.01	6.36	20.04
	Equiv. Refl.: (7,4,0), (11,-4,0), (-4,-7,0), (-7,11,0), (-11,7,0)								
LiNbO3	(4,7,0)	13.409	87.83	39.4	37.4	0.508	19.01	6.36	20.04
	Equiv. Refl.: (7,-11,0), (11,-7,0), (-4,11,0), (-7,-4,0), (-11,4,0)								
LiNbO3	(5,6,-4)	13.384	85.89	27.2	24.4	0.965	23.5	12.06	26.41
	Equiv. Refl.: (6,-11,-4), (11,-6,-4), (-5,11,-4), (-6,-5,-4), (-11,5,-4)								
LiNbO3	(5,-11,4)	13.384	85.89	26.4	24.4	0.965	23.5	12.06	26.41
	Equiv. Refl.: (6,5,4), (11,-5,4), (-5,-6,4), (-6,11,4), (-11,6,4)								
LiNbO3	(1,9,4)	13.384	85.89	19.9	18.6	0.965	17.95	12.06	21.62
	Equiv. Refl.: (9,-10,4), (10,-9,4), (-1,10,4), (-9,-1,4), (-10,1,4)								
LiNbO3	(1,-10,-4)	13.384	85.89	19.3	18.6	0.965	17.95	12.06	21.62
	Equiv. Refl.: (9,1,-4), (10,-1,-4), (-1,-9,-4), (-9,10,-4), (-10,9,-4)								
Quartz	(3,7,3)	13.399	86.9	18.2	14.9	0.727	10.85	9.09	14.15
	Equiv. Refl.: (3,-10,-3), (7,3,-3), (7,-10,3), (-10,3,3), (-10,7,-3)								
Quartz	(10,-3,-3)	13.399	86.9	18.1	14.9	0.727	10.86	9.09	14.16
	Equiv. Refl.: (10,-7,3), (-3,10,3), (-3,-7,-3), (-7,10,-3), (-7,-3,3)								
Sapph	(3,7,2)	13.406	87.44	17.3	14.9	0.6	8.95	7.5	11.68
	Equiv. Refl.: (3,-10,-2), (7,3,-2), (7,-10,2), (10,-3,-2), (10,-7,2), (-3,10,2), (-3,-7,-2), (-7,10,-2), (-7,-3,2) (-10,3,2), (-10,7,-2)								
Quartz	(10,-2,1)	13.403	87.17	16.7	13.9	0.663	9.18	8.28	12.36
	Equiv. Refl.: (10,-8,-1), (-2,10,-1), (-2,-8,1), (-8,10,1), (-8,-2,-1)								
Quartz	(2,8,-1)	13.403	87.17	16.4	13.9	0.663	9.18	8.28	12.36
	Equiv. Refl.: (2,-10,1), (8,2,1), (8,-10,-1), (-10,2,-1), (-10,8,1)								
LiNbO3	(1,9,-2)	13.295	82.19	14.3	12.9	1.841	23.77	23.01	33.08
	Equiv. Refl.: (9,-10,-2), (10,-9,-2), (-1,10,-2), (-9,-1,-2), (-10,1,-2)								
LiNbO3	(1,-10,2)	13.295	82.19	14.1	12.9	1.841	23.77	23.01	33.08
	Equiv. Refl.: (9,1,2), (10,-1,2), (-1,-9,2), (-9,10,2), (-10,9,2)								

Ei =	13.419	keV						ΔEg	ΔEt
Cryst	Refl	EB	ΘB	∫ IR dΘ	Width	Ei cotΘ	ΔE	2m, 50µm	2m, 50µm
	(h,k,l)	[keV]	[°]	[µrad]	[µrad]	[meV/µrad]	[meV]	[meV]	[meV]
**Sapph**	**(1,4,24)**	**13.37**	**85.08**	**12**	**10.1**	**1.155**	**11.68**	**14.43**	**18.57**
	Equiv. Refl.: (1,4,-24), (1,-5,24), (1,-5,-24), (4,1,24), (4,1,-24), (4,-5,24), (4,-5,-24), (5,-1,24), (5,-1,-24) (5,-4,24), (5,-4,-24), (-1,5,24), (-1,5,-24), (-1,-4,24), (-1,-4,-24), (-4,5,24), (-4,5,-24), (-4,-1,24) (-4,-1,-24), (-5,1,24), (-5,1,-24), (-5,4,24), (-5,4,-24)								
**LiNbO3**	**(0,-7,-20)**	**13.218**	**80.08**	**11.2**	**10.1**	**2.347**	**23.65**	**29.33**	**37.68**
	Equiv. Refl.: (7,0,-20), (-7,7,-20)								
**LiNbO3**	**(0,7,20)**	**13.218**	**80.08**	**11**	**10.1**	**2.347**	**23.65**	**29.33**	**37.68**
	Equiv. Refl.: (7,-7,20), (-7,0,20)								
**LiNbO3**	**(1,-8,-18)**	**13.229**	**80.34**	**10.8**	**9.9**	**2.285**	**22.53**	**28.56**	**36.38**
	Equiv. Refl.: (7,1,-18), (8,-1,-18), (-1,-7,-18), (-7,8,-18), (-8,7,-18)								
**LiNbO3**	**(1,7,18)**	**13.229**	**80.34**	**10.7**	**9.9**	**2.285**	**22.53**	**28.56**	**36.38**
	Equiv. Refl.: (7,-8,18), (8,-7,18), (-1,8,18), (-7,-1,18), (-8,1,18)								
**LiNbO3**	**(0,-8,-16)**	**13.226**	**80.28**	**10.2**	**8.7**	**2.299**	**20**	**28.74**	**35.02**
	Equiv. Refl.: (8,0,-16), (-8,8,-16)								
**LiNbO3**	**(1,-8,18)**	**13.229**	**80.34**	**10**	**9**	**2.285**	**20.66**	**28.56**	**35.25**
	Equiv. Refl.: (5,5,-12), (5,-10,-12), (7,1,18), (8,-1,18), (10,-5,-12), (-1,-7,18), (-5,10,-12), (-5,-5,-12), (-7,8,18) (-8,7,18), (-10,5,-12)								
**LiNbO3**	**(1,7,-18)**	**13.229**	**80.34**	**9.9**	**9**	**2.285**	**20.66**	**28.56**	**35.25**
	Equiv. Refl.: (2,3,26), (2,-5,-26), (3,2,-26), (3,-5,26), (5,-2,-26), (5,-3,26), (5,-11,-2), (6,5,-2), (7,-8,-18) (8,-7,-18), (11,-5,-2), (-1,8,-18), (-2,5,26), (-2,-3,-26), (-3,5,-26), (-3,-2,26), (-5,2,26), (-5,3,-26) (-5,-6,-2), (-6,11,-2), (-7,-1,-18), (-8,1,-18), (-11,6,-2)								
**LiNbO3**	**(3,-10,10)**	**13.143**	**78.36**	**9.8**	**8.6**	**2.764**	**23.91**	**34.55**	**42.02**
	Equiv. Refl.: (5,6,2), (7,3,10), (10,-3,10), (-3,-7,10), (-6,-5,2), (-7,10,10), (-10,7,10)								
**LiNbO3**	**(3,7,-10)**	**13.143**	**78.36**	**9.7**	**8.6**	**2.764**	**23.91**	**34.55**	**42.02**
	Equiv. Refl.: (6,-11,2), (7,-10,-10), (10,-7,-10), (11,-6,2), (-3,10,-10), (-5,11,2), (-7,-3,-10), (-10,3,-10), (-11,5,2)								
**LiNbO3**	**(5,5,12)**	**13.183**	**79.25**	**9.5**	**8.7**	**2.548**	**22.24**	**31.85**	**38.84**
	Equiv. Refl.: (5,-10,12), (10,-5,12), (-5,10,12), (-5,-5,12), (-10,5,12)								
**Quartz**	**(10,-3,3)**	**13.399**	**86.9**	**9**	**7.4**	**0.727**	**5.37**	**9.09**	**10.55**
	Equiv. Refl.: (10,-7,-3), (-3,10,-3), (-3,-7,3), (-7,10,3), (-7,-3,-3)								
**LiNbO3**	**(0,8,16)**	**13.226**	**80.28**	**8.8**	**8.7**	**2.299**	**20**	**28.74**	**35.02**
	Equiv. Refl.: (8,-8,16), (-8,0,16)								
**LiNbO3**	**(3,5,-20)**	**13.218**	**80.08**	**8.5**	**7.5**	**2.347**	**17.49**	**29.33**	**34.15**
	Equiv. Refl.: (4,-9,16), (5,4,16), (5,-8,-20), (8,-5,-20), (9,-4,16), (-3,8,-20), (-4,-5,16), (-5,9,16), (-5,-3,-20) (-8,3,-20), (-9,5,16)								
**LiNbO3**	**(2,7,-14)**	**12.99**	**75.47**	**8.3**	**7.1**	**3.478**	**24.73**	**43.47**	**50.02**
	Equiv. Refl.: (4,5,-16), (5,-9,-16), (7,-9,-14), (9,-5,-16), (9,-7,-14), (-2,9,-14), (-4,9,-16), (-5,-4,-16), (-7,-2,-14) (-9,2,-14), (-9,4,-16)								
**Quartz**	**(7,3,3)**	**13.399**	**86.9**	**8.1**	**7.4**	**0.727**	**5.37**	**9.09**	**10.55**
	Equiv. Refl.: (7,-10,-3), (-10,3,-3), (-10,7,3)								
**LiNbO3**	**(1,2,-28)**	**13.05**	**76.54**	**8**	**7.1**	**3.213**	**22.95**	**40.16**	**46.25**
	Equiv. Refl.: (2,-3,-28), (3,-2,-28), (4,-10,-10), (6,4,-10), (10,-4,-10), (-1,3,-28), (-2,-1,-28), (-3,1,-28), (-4,-6,-10) (-6,10,-10), (-10,6,-10), (3,7,-3), (3,-10,3)								
**LiNbO3**	**(1,-3,28)**	**13.05**	**76.54**	**7.9**	**7.1**	**3.213**	**22.95**	**40.16**	**46.25**
	Equiv. Refl.: (2,1,28), (3,-1,28), (-1,-2,28), (-2,3,28), (-3,2,28), (5,5,6), (5,5,-6), (5,-10,6), (5,-10,-6) (10,-5,6), (10,-5,-6), (-5,10,6), (-5,10,-6), (-5,-5,6), (-5,-5,-6), (-10,5,6), (-10,5,-6)								
**LiNbO3**	**(2,-9,14)**	**12.99**	**75.47**	**7.8**	**7.1**	**3.478**	**24.73**	**43.47**	**50.02**
	Equiv. Refl.: (4,6,10), (6,-10,10), (7,2,14), (9,-2,14), (10,-6,10), (-2,-7,14), (-4,10,10), (-6,-4,10), (-7,9,14) (-9,7,14), (-10,4,10)								
**LiNbO3**	**(0,9,-6)**	**12.799**	**72.51**	**7.5**	**6.5**	**4.228**	**27.62**	**52.85**	**59.63**
	Equiv. Refl.: (0,-9,6), (9,0,6), (9,-9,-6), (-9,0,-6), (-9,9,6)								

Ei =	13.419	keV						ΔEg	ΔEt
Cryst	Refl	EB	ΘB	∫ IR dΘ	Width	Ei cotΘ	ΔE	2m, 50µm	2m, 50µm
	(h,k,l)	[keV]	[°]	[µrad]	[µrad]	[meV/µrad]	[meV]	[meV]	[meV]
**LiNbO3**	**(0,-8,14)**	**12.765**	**72.03**	**7.4**	**6.2**	**4.352**	**26.81**	**54.4**	**60.64**
	Equiv. Refl.: (2,-10,0), (3,-8,20), (5,3,20), (8,0,14), (8,2,0), (8,-3,20), (10,-2,0), (-2,-8,0), (-3,-5,20) (-5,8,20), (-8,5,20), (-8,8,14), (-8,10,0), (-10,8,0)								
**LiNbO3**	**(1,-7,20)**	**12.772**	**72.14**	**7.3**	**6.3**	**4.325**	**27.26**	**54.06**	**60.54**
	Equiv. Refl.: (2,8,0), (3,7,8), (6,1,20), (7,-1,20), (7,-10,8), (8,-10,0), (10,-7,8), (10,-8,0), (-1,-6,20) (-2,10,0), (-3,10,8), (-6,7,20), (-7,6,20), (-7,-3,8), (-8,-2,0), (-10,2,0), (-10,3,8), (3,4,20) (3,-7,-20), (4,3,-20), (4,-7,20), (7,-3,-20), (7,-4,20), (-3,7,20), (-3,-4,-20), (-4,7,-20), (-4,-3,20) (-7,3,20), (-7,4,-20)								
**LiNbO3**	**(1,6,-20)**	**12.772**	**72.14**	**7.1**	**6.3**	**4.325**	**27.26**	**54.06**	**60.54**
	Equiv. Refl.: (3,-10,-8), (6,-7,-20), (7,3,-8), (7,-6,-20), (10,-3,-8), (-1,7,-20), (-3,-7,-8), (-6,-1,-20), (-7,1,-20) (-7,10,-8), (-10,7,-8)								
**LiNbO3**	**(2,8,6)**	**13.023**	**76.05**	**7**	**6.3**	**3.333**	**20.94**	**41.66**	**46.62**
	Equiv. Refl.: (8,-10,6), (10,-8,6), (-2,10,6), (-8,-2,6), (-10,2,6)								
**LiNbO3**	**(3,4,-22)**	**12.974**	**75.2**	**6.9**	**6.1**	**3.545**	**21.7**	**44.31**	**49.34**
	Equiv. Refl.: (4,-7,-22), (7,-4,-22), (-3,7,-22), (-4,-3,-22), (-7,3,-22)								
**LiNbO3**	**(0,8,-14)**	**12.765**	**72.03**	**6.7**	**6.2**	**4.352**	**26.81**	**54.4**	**60.64**
	Equiv. Refl.: (2,-10,-6), (8,2,-6), (8,-8,-14), (10,-2,-6), (-2,-8,-6), (-8,0,-14), (-8,10,-6), (-10,8,-6)								
**LiNbO3**	**(3,-7,22)**	**12.974**	**75.2**	**6.6**	**6.1**	**3.545**	**21.7**	**44.31**	**49.34**
	Equiv. Refl.: (4,3,22), (7,-3,22), (-3,-4,22), (-4,7,22), (-7,4,22)								
**LiNbO3**	**(0,-9,-6)**	**12.799**	**72.51**	**6.4**	**5.6**	**4.228**	**23.58**	**52.85**	**57.87**
	Equiv. Refl.: (9,0,-6), (-9,9,-6)								
**LiNbO3**	**(2,8,-6)**	**13.023**	**76.05**	**6.2**	**5.7**	**3.333**	**19.14**	**41.66**	**45.84**
	Equiv. Refl.: (2,-10,6), (8,2,6), (8,-10,-6), (10,-2,6), (10,-8,-6), (-2,10,-6), (-2,-8,6), (-8,10,6), (-8,-2,-6) (-10,2,-6), (-10,8,6)								
**LiNbO3**	**(0,9,6)**	**12.799**	**72.51**	**6**	**5.6**	**4.228**	**23.58**	**52.85**	**57.87**
	Equiv. Refl.: (4,-10,8), (6,4,8), (9,-9,6), (10,-4,8), (-4,-6,8), (-6,10,8), (-9,0,6), (-10,6,8)								
**LiNbO3**	**(1,8,-10)**	**12.694**	**71.08**	**5.9**	**5.2**	**4.599**	**23.87**	**57.49**	**62.25**
	Equiv. Refl.: (3,3,-24), (3,-6,-24), (6,-3,-24), (8,-9,-10), (9,-8,-10), (-1,9,-10), (-3,6,-24), (-3,-3,-24), (-6,3,-24) (-8,-1,-10), (-9,1,-10)								
**LiNbO3**	**(0,-4,-26)**	**12.888**	**73.84**	**5.7**	**5.1**	**3.89**	**19.71**	**48.62**	**52.46**
	Equiv. Refl.: (1,-9,10), (3,3,24), (3,-6,24), (4,0,-26), (4,6,-8), (6,-3,24), (6,-10,-8), (8,1,10), (9,-1,10) (10,-6,-8), (-1,-8,10), (-3,6,24), (-3,-3,24), (-4,4,-26), (-4,10,-8), (-6,3,24), (-6,-4,-8), (-8,9,10) (-9,8,10), (-10,4,-8)								
**LiNbO3**	**(0,4,26)**	**12.888**	**73.84**	**5.6**	**5.1**	**3.89**	**19.71**	**48.62**	**52.46**
	Equiv. Refl.: (0,-2,-28), (2,0,-28), (4,-4,26), (-2,2,-28), (-4,0,26), (0,3,11), (0,-3,-11), (3,0,-11), (3,-3,11) (-3,0,11), (-3,3,-11)								
**LiNbO3**	**(0,2,28)**	**12.826**	**72.9**	**5.5**	**5.1**	**4.127**	**21**	**51.59**	**55.7**
	Equiv. Refl.: (2,-2,28), (-2,0,28)								
**Sapph**	**(2,6,-16)**	**13.267**	**81.36**	**5.4**	**4.6**	**2.038**	**9.35**	**25.48**	**27.14**
	Equiv. Refl.: (2,-8,16), (6,2,16), (6,-8,-16), (8,-2,16), (8,-6,-16), (-2,8,-16), (-2,-6,16), (-6,8,16), (-6,-2,-16) (-8,2,-16), (-8,6,16)								
**LiNbO3**	**(1,-4,26)**	**12.661**	**70.66**	**5.2**	**4.4**	**4.711**	**20.9**	**58.89**	**62.48**
	Equiv. Refl.: (3,1,26), (4,-1,26), (-1,-3,26), (-3,4,26), (-4,3,26), (1,4,-10), (1,-5,10), (4,1,10), (4,-5,-10) (-5,1,-10), (-5,4,10)								
**LiNbO3**	**(1,3,-26)**	**12.661**	**70.66**	**5.1**	**4.4**	**4.711**	**20.9**	**58.89**	**62.48**
	Equiv. Refl.: (3,-4,-26), (4,-3,-26), (-1,4,-26), (-3,-1,-26), (-4,1,-26), (5,-1,10), (5,-4,-10), (-1,5,-10), (-1,-4,10) (-4,5,10), (-4,-1,-10)								
**Quartz**	**(0,3,-11)**	**13.353**	**84.31**	**5**	**4.1**	**1.337**	**5.52**	**16.71**	**17.6**
	Equiv. Refl.: (0,-3,11), (3,0,11), (3,-3,-11), (-3,0,-11), (-3,3,11)								

Ei =	13.419	keV						ΔEg	ΔEt
**Cryst**	**Refl**	**EB**	**ΘB**	**∫ IR dΘ**	**Width**	**Ei cotΘ**	**ΔE**	**2m, 50µm**	**2m, 50µm**
	**(h,k,l)**	**[keV]**	**[°]**	**[µrad]**	**[µrad]**	**[meV/µrad]**	**[meV]**	**[meV]**	**[meV]**
**Sapph**	**(2,7,10)**	**13.207**	**79.8**	**4.6**	**3.7**	**2.415**	**8.94**	**30.19**	**31.48**
	Equiv. Refl.: (2,-9,-10), (7,2,-10), (7,-9,10), (9,-2,-10), (9,-7,10), (-2,9,10), (-2,-7,-10), (-7,9,-10), (-7,-2,10)<br>(-9,2,10), (-9,7,-10), (0,8,5), (0,-8,-5), (5,5,0), (5,-10,0), (8,0,-5), (8,-8,5), (10,-5,0)<br>(-5,10,0), (-5,-5,0), (-8,0,5), (-8,8,-5), (-10,5,0)								
**Sapph**	**(1,8,-4)**	**12.995**	**75.55**	**4.5**	**3.5**	**3.457**	**12.08**	**43.21**	**44.87**
	Equiv. Refl.: (1,-9,4), (8,1,4), (8,-9,-4), (9,-1,4), (9,-8,-4), (-1,9,-4), (-1,-8,4), (-8,9,4), (-8,-1,-4)<br>(-9,1,-4), (-9,8,4), (5,-2,-10), (5,-3,10), (-2,5,10), (-2,-3,-10), (-3,5,-10), (-3,-2,10)								
**Quartz**	**(2,3,10)**	**13.111**	**77.7**	**4.4**	**3.4**	**2.926**	**9.9**	**36.57**	**37.89**
	Equiv. Refl.: (2,5,-8), (2,-5,-10), (2,-7,8), (3,2,-10), (3,-5,10), (5,2,8), (5,-7,-8), (7,-2,8), (7,-5,-8)<br>(-2,7,-8), (-2,-5,8), (-5,2,10), (-5,3,-10), (-5,7,8), (-5,-2,-8), (-7,2,-8), (-7,5,8)								
**Quartz**	**(0,9,-2)**	**13.312**	**82.76**	**4.1**	**3.3**	**1.705**	**5.69**	**21.31**	**22.06**
	Equiv. Refl.: (0,-9,2), (2,7,-5), (2,-9,5), (7,2,5), (7,-9,-5), (9,0,2), (9,-2,5), (9,-7,-5), (9,-9,-2)<br>(-2,9,-5), (-2,-7,5), (-7,9,5), (-7,-2,-5), (-9,0,-2), (-9,2,-5), (-9,7,5), (-9,9,2)								
**Sapph**	**(2,6,14)**	**12.741**	**71.71**	**4**	**3**	**4.435**	**13.49**	**55.43**	**57.05**
	Equiv. Refl.: (2,-8,-14), (6,2,-14), (6,-8,14), (8,-2,-14), (8,-6,14), (-2,8,14), (-2,-6,-14), (-6,8,-14), (-6,-2,14)<br>(-8,2,14), (-8,6,-14)								
**LiNbO3**	**(2,4,25)**	**13.383**	**85.83**	**3.8**	**5.7**	**0.979**	**5.61**	**12.24**	**13.46**
	Equiv. Refl.: (2,-6,-25), (4,2,-25), (4,-6,25), (6,-2,-25), (6,-4,25), (-2,6,25), (-2,-4,-25), (-4,6,-25), (-4,-2,25)<br>(-6,2,25), (-6,4,-25)								
**Sapph**	**(1,8,-7)**	**13.281**	**81.77**	**3.7**	**3.2**	**1.94**	**6.22**	**24.25**	**25.04**
	Equiv. Refl.: (1,-9,7), (8,1,7), (8,-9,-7), (9,-1,7), (9,-8,-7), (-1,9,-7), (-1,-8,7), (-8,9,7), (-8,-1,-7)<br>(-9,1,-7), (-9,8,7)								
**Sapph**	**(3,5,16)**	**13.008**	**75.79**	**3.5**	**2.8**	**3.398**	**9.67**	**42.47**	**43.56**
	Equiv. Refl.: (3,-8,-16), (5,3,-16), (5,-8,16), (8,-3,-16), (8,-5,16), (-3,8,16), (-3,-5,-16), (-5,8,-16), (-5,3,16)<br>(-8,3,16), (-8,5,-16), (2,4,9), (2,8,0), (2,-6,-9), (2,-10,0), (4,2,-9), (4,-6,9), (8,2,0)<br>(8,-10,0), (-6,2,9), (-6,4,-9), (-10,2,0), (-10,8,0)								
**Quartz**	**(4,5,5)**	**12.743**	**71.74**	**3.4**	**2.6**	**4.429**	**11.53**	**55.36**	**56.55**
	Equiv. Refl.: (4,-9,-5), (5,4,-5), (5,-9,5), (9,-4,-5), (9,-5,5), (10,-2,0), (10,-8,0), (-2,10,0), (-2,-8,0)<br>(-4,9,5), (-4,-5,-5), (-5,9,-5), (-5,-4,5), (-8,10,0), (-8,-2,0), (-9,4,5), (-9,5,-5)								
**Sapph**	**(1,1,27)**	**13.148**	**78.46**	**3.3**	**2.6**	**2.74**	**7.25**	**34.25**	**35.01**
	Equiv. Refl.: (1,1,-27), (1,-2,27), (1,-2,-27), (2,7,-11), (2,-1,27), (2,-1,-27), (2,-9,11), (4,6,-2), (4,-10,2)<br>(6,4,2), (6,-10,-2), (7,2,11), (7,-9,-11), (9,-2,11), (9,-7,-11), (10,-4,2), (10,-6,-2), (-1,2,27)<br>(-1,2,-27), (-1,-1,27), (-1,-1,-27), (-2,1,27), (-2,1,-27), (-2,9,-11), (-2,-7,11), (-4,10,-2), (-4,-6,2)<br>(-6,10,2), (-6,-4,-2), (-7,9,11), (-7,-2,-11), (-9,2,-11), (-9,7,11), (-10,4,-2), (-10,6,2), (3,-1,-11)<br>(3,-2,11), (4,6,-1), (4,-10,1), (6,4,1), (6,-2,-9), (6,-4,9), (6,-10,-1), (8,-2,7), (8,-6,-7)<br>(-1,3,11), (-1,-2,-11), (-2,3,-11), (-2,6,9), (-2,8,-7), (-2,-1,11), (-2,-4,-9), (-2,-6,7), (-4,6,-9)<br>(-4,-2,9), (-6,8,7), (-6,-2,-7), (-10,4,-1), (-10,6,1)								
**Quartz**	**(1,2,11)**	**13.193**	**79.47**	**3.2**	**2.9**	**2.495**	**7.24**	**31.19**	**32.01**
	Equiv. Refl.: (1,8,-4), (1,-3,-11), (1,-9,4), (2,1,-11), (2,6,-7), (2,-3,11), (2,-8,7), (6,2,7), (6,-8,-7)<br>(8,1,4), (8,-9,-4), (10,-4,1), (10,-6,-1), (-3,1,11), (-3,2,-11), (-4,10,-1), (-4,-6,1), (-6,10,1)<br>(-6,-4,-1), (-8,2,-7), (-8,6,7), (-9,1,-4), (-9,8,4)								
**Quartz**	**(1,1,11)**	**12.867**	**73.51**	**3.1**	**2.3**	**3.972**	**9.19**	**49.65**	**50.5**
	Equiv. Refl.: (1,1,-11), (1,-2,11), (1,-2,-11), (-2,1,11), (-2,1,-11)								
**Sapph**	**(4,5,-13)**	**13.288**	**81.97**	**3**	**2.6**	**1.892**	**4.95**	**23.65**	**24.16**
	Equiv. Refl.: (4,-9,13), (5,4,13), (5,-9,-13), (9,-4,13), (9,-5,-13), (-4,9,-13), (-4,-5,13), (-5,9,13), (-5,-4,-13)<br>(-9,4,-13), (-9,5,13), (1,8,4), (1,-9,-4), (2,-1,11), (2,-1,-11), (8,1,-4), (8,-9,4), (9,-1,4)<br>(9,-1,-4), (9,-8,4), (9,-8,-4), (-1,2,11), (-1,2,-11), (-1,9,4), (-1,9,-4), (-1,-1,11), (-1,-1,-11)<br>(-1,-8,4), (-1,-8,-4), (-8,9,4), (-8,9,-4), (-8,-1,4), (-8,-1,-4), (-9,1,4), (-9,8,-4)								
**Quartz**	**(0,2,11)**	**12.949**	**74.8**	**2.9**	**2.2**	**3.647**	**7.88**	**45.59**	**46.26**
	Equiv. Refl.: (0,-2,-11)								

Ei =	13.419	keV						ΔEg	ΔEt
**Cryst**	**Refl**	**EB**	**ΘB**	**∫ IR dΘ**	**Width**	**Ei cotΘ**	**ΔE**	**2m, 50µm**	**2m, 50µm**
	**(h,k,l)**	**[keV]**	**[°]**	**[µrad]**	**[µrad]**	**[meV/µrad]**	**[meV]**	**[meV]**	**[meV]**
**Quartz**	**(0,8,-5)**	**12.99**	**75.48**	**2.8**	**2.1**	**3.475**	**7.44**	**43.44**	**44.07**
	Equiv. Refl.: (0,-8,5), (2,0,-11), (2,-2,11), (3,7,-2), (3,-10,2), (7,3,2), (7,-10,-2), (8,0,5), (8,-8,-5) (9,-3,-5), (9,-6,5), (10,-3,2), (10,-7,-2), (-2,0,11), (-2,2,-11), (-3,9,5), (-3,10,-2), (-3,-6,-5) (-3,-7,2), (-6,9,-5), (-6,-3,5), (-7,10,2), (-7,-3,-2), (-8,0,-5), (-8,8,5), (-10,3,-2), (-10,7,2)								
**Quartz**	**(2,4,-9)**	**12.884**	**73.77**	**2.7**	**2.1**	**3.906**	**8.01**	**48.82**	**49.47**
	Equiv. Refl.: (2,-6,9), (3,4,-8), (3,5,7), (3,6,5), (3,-7,8), (3,-8,-7), (3,-9,-5), (4,2,9), (4,3,8) (4,-6,-9), (4,-7,-8), (5,3,-7), (5,-8,7), (6,3,-5), (6,-2,9), (6,-4,-9), (6,-9,5), (7,-3,8) (7,-4,-8), (-2,6,-9), (-2,-4,9), (-3,7,-8), (-3,-4,8), (-4,6,9), (-4,7,8), (-4,-2,-9), (-4,-3,-8) (-6,2,-9), (-6,4,9), (-7,3,-8), (-7,4,8), (-8,3,7), (-8,5,-7), (-9,3,5), (-9,6,-5)								
**Sapph**	**(3,6,12)**	**13.244**	**80.72**	**2.6**	**2.5**	**2.192**	**5.38**	**27.4**	**27.92**
	Equiv. Refl.: (3,6,-12), (3,-9,12), (3,-9,-12), (6,3,12), (6,3,-12), (6,-9,12), (6,-9,-12), (9,-3,12), (9,-3,-12) (9,-6,12), (9,-6,-12), (-3,9,12), (-3,9,-12), (-3,-6,12), (-3,-6,-12), (-6,9,12), (-6,9,-12), (-6,-3,12) (-6,-3,-12), (-9,3,12), (-9,3,-12), (-9,6,12), (-9,6,-12)								
**Quartz**	**(0,9,1)**	**13.163**	**78.79**	**2.4**	**2.2**	**2.66**	**5.82**	**33.25**	**33.75**
	Equiv. Refl.: (0,-9,-1), (8,-1,6), (8,-3,-7), (8,-5,7), (8,-7,-6), (9,0,-1), (9,-9,1), (-1,8,-6), (-1,-7,6) (-3,8,7), (-3,-5,-7), (-5,8,-7), (-5,-3,7), (-7,8,6), (-7,-1,-6), (-9,0,1), (-9,9,-1)								
**Sapph**	**(1,8,5)**	**13.073**	**76.97**	**2.3**	**2.1**	**3.106**	**6.58**	**38.82**	**39.37**
	Equiv. Refl.: (1,-9,-5), (8,1,-5), (8,-9,5), (9,-1,-5), (9,-8,5), (-1,9,5), (-1,-8,-5), (-8,9,-5), (-8,-1,5) (-9,1,5), (-9,8,-5), (1,7,-6), (1,-8,6), (2,7,4), (2,-9,-4), (7,1,6), (7,2,-4), (7,-8,-6) (7,-9,4), (9,-2,-4), (9,-7,4), (-2,9,4), (-2,-7,-4), (-7,9,-4), (-7,-2,4), (-8,1,-6), (-8,7,6) (-9,2,4), (-9,7,-4)								
**LiNbO3**	**(1,9,1)**	**13.272**	**81.51**	**1.9**	**3**	**2.003**	**6**	**25.04**	**25.75**
	Equiv. Refl.: (1,-10,-1), (9,1,-1), (9,-10,1), (10,-1,-1), (10,-9,1), (-1,10,1), (-1,-9,-1), (-9,10,-1), (-9,-1,1) (-10,1,1), (-10,9,-1), (0,8,10), (0,-8,-10), (1,6,19), (1,-7,-19), (5,5,0), (5,-10,0), (6,1,-19) (6,-7,19), (7,-1,-19), (7,-6,19), (8,0,-10), (8,-8,10), (10,-5,0), (-1,7,19), (-1,-6,-19), (-5,10,0) (-5,-5,0), (-6,7,-19), (-6,-1,19), (-7,1,19), (-7,6,-19), (-8,0,10), (-8,8,-10), (-10,5,0)								
**Quartz**	**(0,4,-10)**	**12.866**	**73.49**	**1.8**	**1.8**	**3.977**	**7.22**	**49.71**	**50.23**
	Equiv. Refl.: (0,-4,10), (1,7,6), (1,8,-2), (1,-8,-6), (1,-9,2), (2,6,7), (2,-8,-7), (3,7,0), (3,-10,0) (4,0,10), (4,-4,-10), (6,2,-7), (6,-8,7), (7,1,-6), (7,3,0), (7,-8,6), (7,-10,0), (8,1,2) (8,-9,-2), (9,-1,2), (9,-8,-2), (-1,9,-2), (-1,-8,2), (-4,0,-10), (-4,4,10), (-8,1,6), (-8,2,7) (-8,6,-7), (-8,7,-6), (-8,9,2), (-8,-1,-2), (-9,1,-2), (-9,8,2), (-10,3,0), (-10,7,0)								
**Sapph**	**(1,2,26)**	**13.032**	**76.21**	**1.7**	**1.8**	**3.293**	**6.09**	**41.16**	**41.61**
	Equiv. Refl.: (1,-3,-26), (2,1,-26), (2,-3,26), (3,-1,-26), (3,-2,26), (4,5,-10), (4,-9,10), (5,4,10), (5,-9,-10) (9,-4,10), (9,-5,-10), (-1,3,26), (-1,-2,-26), (-2,3,-26), (-2,-1,26), (-3,1,26), (-3,2,-26), (-4,9,-10) (-4,-5,10), (-5,9,10), (-5,-4,-10), (-9,4,-10), (-9,5,10), (3,3,9), (3,3,-9), (3,-6,9), (3,-6,-9) (4,5,-6), (4,-9,6), (5,4,6), (5,-9,-6), (6,-3,9), (6,-3,-9), (7,-1,-8), (7,-6,8), (8,-1,-6) (8,-2,-7), (8,-6,7), (8,-7,6), (10,-3,0), (10,-7,0), (-1,7,8), (-1,8,6), (-1,-6,-8), (-1,-7,-6) (-2,8,7), (-2,-6,-7), (-3,6,9), (-3,6,-9), (-3,10,0), (-3,-3,9), (-3,-3,-9), (-3,-7,0), (-6,3,9) (-6,3,-9), (-6,7,-8), (-6,8,-7), (-6,-1,8), (-6,-2,7), (-7,8,-6), (-7,10,0), (-7,-1,6), (-7,-3,0) (-9,4,-6), (-9,5,6)								
**Sapph**	**(0,2,-26)**	**12.769**	**72.1**	**1.6**	**1.7**	**4.335**	**7.48**	**54.19**	**54.71**
	Equiv. Refl.: (0,-2,26), (1,5,20), (1,8,-1), (1,-6,-20), (1,-9,1), (2,0,26), (2,-2,-26), (5,1,-20), (5,-6,20) (6,-1,-20), (6,-5,20), (8,1,1), (8,-9,-1), (9,-1,1), (9,-8,-1), (-1,6,20), (-1,9,-1), (-1,-5,-20) (-1,-8,1), (-2,0,-26), (-2,2,26), (-5,6,-20), (-5,-1,20), (-6,1,20), (-6,5,-20), (-8,9,1), (-8,-1,-1) (-9,1,-1), (-9,8,1), (1,6,-8), (1,-7,8), (3,4,8), (3,-7,-8), (4,3,-8), (4,4,7), (4,4,-7) (4,-1,-10), (4,-3,10), (4,-7,8), (4,-8,7), (4,-8,-7), (6,1,8), (6,-7,-8), (7,-1,8), (7,-3,-8) (7,-4,8), (7,-6,-8), (8,-4,7), (8,-4,-7), (9,-4,6), (9,-5,-6), (10,-4,-2), (10,-6,2), (-1,4,10) (-1,7,-8), (-1,-3,-10), (-1,-6,8), (-3,4,-10), (-3,7,8), (-3,-1,10), (-3,-4,-8), (-4,7,-8), (-4,8,7) (-4,8,-7), (-4,9,-6), (-4,10,2), (-4,-3,8), (-4,-4,7), (-4,-4,-7), (-4,-5,6), (-4,-6,-2), (-5,9,6) (-5,-4,-6), (-6,7,8), (-6,10,-2), (-6,-1,-8), (-6,-4,2), (-7,1,-8), (-7,3,8), (-7,4,-8), (-7,6,8) (-8,4,7), (-8,4,-7)								

Ei =	13.419	keV						ΔEg	ΔEt
Cryst	Refl	EB	ΘB	∫ IR dΘ	Width	Ei cotΘ	ΔE	2m, 50μm	2m, 50μm
	(h,k,l)	[keV]	[°]	[μrad]	[μrad]	[meV/μrad]	[meV]	[meV]	[meV]
**Quartz**	**(1,3,10)**	**12.616**	**70.08**	**1.5**	**1.6**	**4.864**	**7.83**	**60.79**	**61.3**
	Equiv. Refl.: (1,5,-9), (1,6,8), (1,-4,-10), (1,-6,9), (1,-7,-8), (3,1,-10), (3,-4,10), (5,1,9), (5,-6,-9) (6,1,-8), (6,-7,8), (-4,1,10), (-4,3,-10), (-6,1,-9), (-6,5,9), (-7,1,8), (-7,6,-8)								
**Quartz**	**(0,6,8)**	**12.674**	**70.81**	**1.4**	**1.5**	**4.669**	**6.88**	**58.36**	**58.77**
	Equiv. Refl.: (0,-6,-8), (4,6,2), (4,6,-3), (4,-10,3), (4,-10,-2), (6,0,-8), (6,4,3), (6,4,-2), (6,-1,9) (6,-5,-9), (6,-6,8), (6,-10,2), (6,-10,-3), (10,-4,3), (10,-6,-3), (-1,6,-9), (-1,-5,9), (-4,10,-3) (-4,-6,3), (-5,6,9), (-5,-1,-9), (-6,0,8), (-6,6,-8), (-6,10,3), (-6,-4,-3), (-10,4,2), (-10,4,-3) (-10,6,3), (-10,6,-2)								
**LiNbO3**	**(3,4,23)**	**13.316**	**82.9**	**1.3**	**2.4**	**1.671**	**4.05**	**20.89**	**21.28**
	Equiv. Refl.: (3,-7,-23), (4,3,-23), (4,-7,23), (7,-3,-23), (7,-4,23), (-3,7,23), (-3,-4,-23), (-4,7,-23), (-4,-3,23) (-7,3,23), (-7,4,-23), (4,5,11), (4,6,4), (4,-9,-11), (4,-10,-4), (5,4,-11), (5,-9,11), (6,4,-4) (6,-10,4), (9,-4,-11), (9,-5,11), (10,-4,-4), (10,-6,4), (-4,9,11), (-4,10,4), (-4,-5,-11), (-4,-6,-4) (-5,9,-11), (-5,-4,11), (-6,10,-4), (-6,-4,4), (-9,4,11), (-9,5,-11), (-10,4,4), (-10,6,-4), (0,9,-1) (0,-9,1), (4,6,1), (4,-10,-1), (6,4,-1), (6,-10,1), (9,0,1), (9,-9,-1), (10,-4,-1), (10,-6,1) (-4,10,1), (-4,-6,-1), (-6,10,-1), (-6,-4,1), (-9,0,-1), (-9,9,1), (-10,4,1), (-10,6,-1)								
**LiNbO3**	**(4,6,-11)**	**13.082**	**77.13**	**1.2**	**1.9**	**3.065**	**5.77**	**38.32**	**38.75**
	Equiv. Refl.: (4,-10,11), (6,4,11), (6,-10,-11), (10,-4,11), (10,-6,-11), (-4,10,-11), (-4,-6,11), (-6,10,11), (-6,-4,-11) (-10,4,-11), (-10,6,11), (3,5,-17), (3,-8,17), (5,3,17), (5,-8,-17), (8,-3,17), (8,-5,-17), (-3,8,-17) (-3,-5,17), (-5,8,17), (-5,-3,-17), (-8,3,-17), (-8,5,17), (1,8,2), (1,-9,-2), (3,6,-5), (3,-9,5) (6,3,5), (6,-9,-5), (8,1,-2), (8,-9,2), (9,-3,5), (9,-4,5), (9,-4,-6), (9,-5,6), (9,-5,-5) (9,-6,-5), (10,-2,-1), (10,-8,1), (-2,10,1), (-2,-8,-1), (-3,9,-5), (-3,-6,5), (-4,9,6), (-4,9,-5) (-4,-5,5), (-4,-5,-6), (-5,9,5), (-5,9,-6), (-5,-4,6), (-5,-4,-5), (-6,9,5), (-6,-3,-5), (-8,10,-1) (-8,-2,1), (-9,1,2), (-9,3,-5), (-9,6,5), (-9,8,-2)								
**LiNbO3**	**(2,2,27)**	**12.999**	**75.63**	**1.1**	**1.7**	**3.438**	**5.86**	**42.98**	**43.37**
	Equiv. Refl.: (2,2,-27), (2,7,13), (2,-4,27), (2,-4,-27), (2,-9,-13), (4,-2,27), (4,-2,-27), (7,2,-13), (7,-9,13) (9,-2,-13), (9,-7,13), (-2,4,27), (-2,4,-27), (-2,9,13), (-2,-2,27), (-2,-2,-27), (-2,-7,-13), (-4,2,27) (-4,2,-27), (-7,9,-13), (-7,-2,13), (-9,2,13), (-9,7,-13), (1,8,3), (1,-9,-3), (4,5,6), (4,5,-5) (4,-9,5), (4,-9,-6), (5,4,5), (5,4,-6), (5,-9,6), (5,-9,-5), (8,1,-3), (8,-9,3), (9,-1,-2) (9,-8,2), (-1,9,2), (-1,-8,-2), (-8,9,-2), (-8,-1,2), (-9,1,3), (-9,4,6), (-9,4,-5), (-9,5,5) (-9,5,-6), (-9,8,-3)								
**Sapph**	**(4,4,15)**	**12.645**	**70.44**	**1**	**1.2**	**4.767**	**5.5**	**59.58**	**59.84**
	Equiv. Refl.: (4,4,-15), (4,-8,15), (4,-8,-15), (8,-4,15), (8,-4,-15), (-4,8,15), (-4,8,-15), (-4,-4,15), (-4,-4,-15) (-8,4,15), (-8,4,-15), (1,2,-11), (1,8,-3), (1,-3,11), (1,-9,3), (2,1,11), (2,8,1), (2,-3,-11) (2,-10,-1), (8,1,3), (8,2,-1), (8,-9,-3), (8,-10,1), (9,-1,3), (9,-1,-3), (9,-8,3), (9,-8,-3) (-1,9,3), (-1,9,-3), (-1,-8,3), (-1,-8,-3), (-3,1,-11), (-3,2,11), (-8,9,3), (-8,9,-3), (-8,-1,3) (-8,-1,-3), (-9,1,-3), (-9,8,3), (-10,2,1), (-10,8,-1)								
**LiNbO3**	**(2,4,-23)**	**12.646**	**70.46**	**0.9**	**1.4**	**4.763**	**6.67**	**59.54**	**59.91**
	Equiv. Refl.: (2,-6,23), (4,2,23), (4,5,17), (4,-6,-23), (4,-9,-17), (5,4,-17), (5,-9,17), (6,-2,23), (6,-4,-23) (9,-4,-17), (9,-5,17), (-2,6,-23), (-2,-4,23), (-4,6,23), (-4,9,17), (-4,-2,-23), (-4,-5,-17), (-5,9,-17) (-5,-4,17), (-6,2,-23), (-6,4,23), (-9,4,17), (-9,5,-17), (2,7,-8), (2,-9,8), (7,2,8), (7,-9,-8) (9,-2,8), (9,-7,-8), (-2,9,-8), (-2,-7,8), (-7,9,8), (-7,-2,-8), (-9,2,-8), (-9,7,8), (0,6,-8) (0,7,7), (0,-6,8), (0,-7,-7), (1,5,9), (1,-6,-9), (3,7,2), (3,-1,11), (3,-2,-11), (3,-10,-2) (5,1,-9), (5,5,3), (5,5,-3), (5,-6,9), (5,-10,3), (5,-10,-3), (6,0,8), (6,-6,-8), (7,0,-7) (7,3,-2), (7,-7,7), (7,-10,2), (9,-2,-5), (9,-7,5), (10,-3,-2), (10,-5,3), (10,-5,-3), (10,-7,2) (-1,3,-11), (-1,-2,11), (-2,3,11), (-2,9,5), (-2,-1,-11), (-2,-7,-5), (-3,10,2), (-3,-7,-2), (-5,10,3) (-5,10,-3), (-5,-5,3), (-5,-5,-3), (-6,0,-8), (-6,1,9), (-6,5,-9), (-6,6,8), (-7,0,7), (-7,7,-7) (-7,9,-5), (-7,10,-2), (-7,-2,5), (-7,-3,2), (-10,3,2), (-10,5,3), (-10,5,-3), (-10,7,-2)								

Ei =	13.419	keV						ΔEg	ΔEt
Cryst	Refl	EB	ΘB	∫ IR dΘ	Width	Ei cotΘ	ΔE	2m, 50µm	2m, 50µm
	(h,k,l)	[keV]	[°]	[µrad]	[µrad]	[meV/µrad]	[meV]	[meV]	[meV]
**LiNbO3**	**(2,8,9)**	**13.364**	**84.83**	**0.8**	**2**	**1.214**	**2.48**	**15.18**	**15.38**
	Equiv. Refl.: (2,8,-9), (2,-10,9), (2,-10,-9), (3,7,11), (3,-10,-11), (7,3,-11), (7,-10,11), (8,2,9), (8,2,-9) (8,-10,9), (8,-10,-9), (10,-2,9), (10,-2,-9), (10,-3,-11), (10,-7,11), (10,-8,9), (10,-8,-9), (-2,10,9) (-2,10,-9), (-2,-8,9), (-2,-8,-9), (-3,10,11), (-3,-7,-11), (-7,10,-11), (-7,-3,11), (-8,10,9), (-8,10,-9) (-8,-2,9), (-8,-2,-9), (-10,2,9), (-10,2,-9), (-10,3,11), (-10,7,-11), (-10,8,9), (-10,8,-9), (0,1,-11) (0,-1,11), (1,0,11), (1,4,10), (1,-1,-11), (1,-5,-10), (2,5,8), (2,7,5), (2,-7,-8), (2,-9,-5) (3,5,-7), (3,-8,7), (4,1,-10), (4,6,0), (4,-5,10), (4,-10,0), (5,2,-8), (5,3,7), (5,-7,8) (5,-8,-7), (6,4,0), (6,-1,-9), (6,-5,9), (6,-10,0), (7,2,-5), (7,-2,-8), (7,-5,8), (7,-9,5) (8,-3,7), (8,-5,-7), (10,-3,-1), (10,-4,0), (10,-6,0), (10,-7,1), (-1,0,-11), (-1,1,11), (-1,6,9) (-1,-5,-9), (-2,7,8), (-2,-5,-8), (-3,8,-7), (-3,10,1), (-3,-5,7), (-3,-7,-1), (-4,10,0), (-4,-6,0) (-5,1,10), (-5,4,-10), (-5,6,-9), (-5,7,-8), (-5,8,7), (-5,-1,9), (-5,-2,8), (-5,-3,-7), (-6,10,0) (-6,-4,0), (-7,2,8), (-7,5,-8), (-7,10,-1), (-7,-3,1), (-8,3,-7), (-8,5,7), (-9,2,5), (-9,7,-5) (-10,4,0), (-10,6,0)								
**Sapph**	**(2,7,7)**	**12.759**	**71.96**	**0.7**	**0.8**	**4.37**	**3.63**	**54.63**	**54.75**
	Equiv. Refl.: (2,-9,-7), (7,2,-7), (7,-9,7), (9,-2,-7), (9,-7,7), (-2,9,7), (-2,-7,-7), (-7,9,-7), (-7,-2,7) (-9,2,7), (-9,7,-7), (0,4,10), (0,-4,-10), (3,7,1), (3,-10,-1), (4,0,-10), (4,6,3), (4,-4,10) (4,-10,-3), (5,-1,-10), (5,-4,10), (6,4,-3), (6,-10,3), (7,3,-1), (7,-10,1), (10,-5,1), (10,-5,-1) (-1,5,10), (-1,-4,-10), (-4,0,10), (-4,4,-10), (-4,5,-10), (-4,-1,10), (-5,10,1), (-5,10,-1), (-5,-5,1) (-5,-5,-1), (-10,3,1), (-10,4,3), (-10,6,-3), (-10,7,-1)								
**LiNbO3**	**(5,6,-1)**	**13.272**	**81.51**	**0.6**	**1.5**	**2.003**	**2.92**	**25.04**	**25.21**
	Equiv. Refl.: (5,-11,1), (6,5,1), (6,-11,-1), (11,-5,1), (11,-6,-1), (-5,11,-1), (-5,-6,1), (-6,11,1), (-6,-5,-1) (-11,5,-1), (-11,6,1), (0,5,22), (0,-5,-22), (2,3,23), (2,-5,-23), (3,2,-23), (3,-5,23), (5,0,-22) (5,5,3), (5,5,-3), (5,-2,-23), (5,-3,23), (5,-5,22), (5,-10,3), (5,-10,-3), (10,-5,3), (10,-5,-3) (-2,5,23), (-2,-3,-23), (-3,5,-23), (-3,-2,23), (-5,0,22), (-5,2,23), (-5,3,-23), (-5,5,-22), (-5,10,3) (-5,10,-3), (-5,-5,3), (-5,-5,-3), (-10,5,3), (-10,5,-3), (1,3,-10), (1,-4,10), (2,7,-4), (2,-9,4) (3,1,10), (3,-4,-10), (5,5,1), (5,5,-1), (5,-10,1), (5,-10,-1), (7,2,4), (7,-9,-4), (9,-2,4) (9,-7,-4), (10,-4,-3), (10,-6,3), (-2,9,-4), (-2,-7,4), (-4,1,-10), (-4,3,10), (-4,10,3), (-4,-6,-3) (-6,10,-3), (-6,-4,3), (-7,9,4), (-7,-2,-4), (-9,2,-4), (-9,7,4), (-10,5,1), (-10,5,-1)								
**Quartz**	**(3,7,-1)**	**13.001**	**75.66**	**0.5**	**0.6**	**3.431**	**2.23**	**42.89**	**42.95**
	Equiv. Refl.: (3,-10,1), (4,-1,10), (4,-3,-10), (7,3,1), (7,-10,-1), (10,-3,1), (10,-7,-1), (-1,-4,-10), (-1,-3,10) (-3,4,10), (-3,10,-1), (-3,-1,-10), (-3,-7,1), (-7,10,1), (-7,-3,-1), (-10,3,-1), (-10,7,1)								
**LiNbO3**	**(1,5,23)**	**12.873**	**73.6**	**0.4**	**0.9**	**3.949**	**3.6**	**49.36**	**49.49**
	Equiv. Refl.: (1,-6,-23), (3,7,-7), (3,-10,7), (5,1,-23), (5,-6,23), (6,-1,-23), (6,-5,23), (7,3,7), (7,-10,-7) (10,-3,7), (10,-7,-7), (-1,6,23), (-1,-5,-23), (-3,10,-7), (-3,-7,7), (-5,6,-23), (-5,-1,23), (-6,1,23) (-6,5,-23), (-7,10,7), (-7,-3,-7), (-10,3,-7), (-10,7,7), (1,6,-17), (1,-7,17), (6,1,17), (6,-7,-17) (7,-1,17), (7,-6,-17), (-1,7,-17), (-1,-6,17), (-6,7,17), (-6,-1,-17), (-7,1,-17), (-7,6,17), (0,2,-11) (0,-2,11), (2,0,11), (2,-2,-11), (-2,0,-11), (-2,2,11)								
**LiNbO3**	**(2,6,-19)**	**13.143**	**78.35**	**0.3**	**0.9**	**2.767**	**2.4**	**34.58**	**34.67**
	Equiv. Refl.: (2,8,3), (2,8,-3), (2,-8,19), (2,-10,3), (2,-10,-3), (3,6,15), (3,6,-15), (3,-9,15), (3,-9,-15) (6,2,19), (6,3,15), (6,3,-15), (6,-8,-19), (6,-9,15), (6,-9,-15), (8,2,3), (8,2,-3), (8,-2,19) (8,-6,-19), (8,-10,3), (8,-10,-3), (9,-3,15), (9,-3,-15), (9,-6,15), (9,-6,-15), (10,-2,3), (10,-2,-3) (10,-8,3), (10,-8,-3), (-2,8,-19), (-2,10,3), (-2,10,-3), (-2,-6,19), (-2,-8,3), (-2,-8,-3), (-3,9,15) (-3,9,-15), (-3,-6,15), (-3,-6,-15), (-6,8,19), (-6,9,15), (-6,9,-15), (-6,-2,-19), (-6,-3,15), (-6,-3,-15) (-8,2,-19), (-8,6,19), (-8,10,3), (-8,10,-3), (-8,-2,3), (-8,-2,-3), (-9,3,15), (-9,3,-15), (-9,6,15) (-9,6,-15), (-10,2,3), (-10,2,-3), (-10,8,3), (-10,8,-3), (1,3,25), (1,-4,-25), (2,5,18), (2,5,-18) (2,-7,18), (2,-7,-18), (3,1,-25), (3,-4,25), (4,-1,-25), (4,-3,25), (5,2,18), (5,2,-18), (5,-7,18) (5,-7,-18), (7,-2,18), (7,-2,-18), (7,-5,18), (7,-5,-18), (-1,4,25), (-1,-3,-25), (-2,7,18), (-2,7,-18) (-2,-5,18), (-2,-5,-18), (-3,4,-25), (-3,-1,25), (-4,1,25), (-4,3,-25), (-5,7,18), (-5,7,-18), (-5,-2,18) (-5,-2,-18), (-7,2,18), (-7,2,-18), (-7,5,18), (-7,5,-18), (0,7,-7), (0,-7,7), (2,3,-10), (2,-5,10) (3,2,10), (3,-5,-10), (5,-2,10), (5,-3,-10), (7,0,7), (7,-7,-7), (-2,5,-10), (-2,-3,10), (-3,5,10) (-3,-2,-10), (-5,2,-10), (-5,3,10), (-7,0,-7), (-7,7,7)								
**LiNbO3**	**(1,8,-13)**	**13.226**	**80.28**	**0.2**	**0.7**	**2.299**	**1.51**	**28.73**	**28.77**

	Equiv. Refl.: (1,-9,13), (2,5,21), (2,5,-21), (2,-7,21), (2,-7,-21), (5,2,21), (5,2,-21), (5,-7,21), (5,-7,-21) (7,-2,21), (7,-2,-21), (7,-5,21), (7,-5,-21), (8,1,13), (8,-9,-13), (9,-1,13), (9,-8,-13), (-1,9,-13) (-1,-8,13), (-2,7,21), (-2,7,-21), (-2,-5,21), (-2,-5,-21), (-5,7,21), (-5,7,-21), (-5,-2,21), (-5,-2,-21) (-7,2,21), (-7,2,-21), (-7,5,21), (-7,5,-21), (-8,9,13), (-8,-1,-13), (-9,1,-13), (-9,8,13), (0,8,-8) (0,-8,8), (4,6,-5), (4,-10,5), (6,4,5), (6,-10,-5), (8,0,8), (8,-8,-8), (10,-4,5), (10,-6,-5) (-4,10,-5), (-4,-6,5), (-6,10,5), (-6,-4,-5), (-8,0,-8), (-8,8,8), (-10,4,-5), (-10,6,5)							
**LiNbO3**	**(1,8,11)**	**12.858**	**73.38**	**0.1**	**0.4**	**4.005**	**1.65**	**50.07**	**50.1**
	Equiv. Refl.: (1,-9,-11), (2,3,-25), (2,-5,25), (3,2,25), (3,5,19), (3,-5,-25), (3,-8,-19), (5,3,-19), (5,-2,25) (5,-3,-25), (5,-8,19), (8,1,-11), (8,-3,-19), (8,-5,19), (8,-9,11), (9,-1,-11), (9,-8,11), (-1,9,11) (-1,-8,-11), (-2,5,-25), (-2,-3,25), (-3,5,25), (-3,8,19), (-3,-2,-25), (-3,-5,-19), (-5,2,-25), (-5,3,25) (-5,8,-19), (-5,-3,19), (-8,3,19), (-8,5,-19), (-8,9,-11), (-8,-1,11), (-9,1,11), (-9,8,-11), (1,8,2) (1,-9,-2), (3,4,-19), (3,6,9), (3,6,-9), (3,-7,19), (3,-9,9), (3,-9,-9), (4,3,19), (4,6,1) (4,-7,-19), (4,-10,-1), (6,3,9), (6,3,-9), (6,4,-1), (6,-9,9), (6,-9,-9), (6,-10,1), (7,-3,19) (7,-4,-19), (8,1,-2), (8,-9,2), (9,-1,-2), (9,-3,9), (9,-3,-9), (9,-6,9), (9,-6,-9), (9,-8,2) (10,-4,-1), (10,-6,1), (-1,9,2), (-1,-8,-2), (-3,7,-19), (-3,9,9), (-3,9,-9), (-3,-4,19), (-3,-6,9) (-3,-6,-9), (-4,7,19), (-4,10,1), (-4,-3,-19), (-4,-6,-1), (-6,9,9), (-6,9,-9), (-6,10,-1), (-6,-3,9) (-6,-3,-9), (-6,-4,1), (-7,3,-19), (-7,4,19), (-8,9,-2), (-8,-1,2), (-9,1,2), (-9,3,9), (-9,3,-9) (-9,6,9), (-9,6,-9), (-9,8,-2), (-10,4,1), (-10,6,-1), (0,5,9), (0,-5,-9), (5,0,-9), (5,-5,9) (-5,0,9), (-5,5,-9)							
**LiNbO3**	**(5,5,9)**	**12.697**	**71.12**	**0**	**0.1**	**4.59**	**0.5**	**57.38**	**57.38**
	Equiv. Refl.: (5,5,-9), (5,-10,9), (5,-10,-9), (10,-5,9), (10,-5,-9), (-5,10,9), (-5,10,-9), (-5,-5,9), (-5,-5,-9) (-10,5,9), (-10,5,-9), (1,7,12), (1,7,-12), (1,-8,12), (1,-8,-12), (7,1,12), (7,1,-12), (7,-8,12) (7,-8,-12), (8,-1,12), (8,-1,-12), (8,-7,12), (8,-7,-12), (-1,8,12), (-1,8,-12), (-1,-7,12), (-1,-7,-12) (-7,8,12), (-7,8,-12), (-7,-1,12), (-7,-1,-12), (-8,1,12), (-8,1,-12), (-8,7,12), (-8,7,-12)							

Ei =	12.824	keV						ΔEg	ΔEt
**Cryst**	**Refl**	**EB**	**ΘB**	**∫IR dΘ**	**Width**	**Ei cotΘ**	**ΔE**	**2m, 50µm**	**2m, 50µm**
	**(h,k,l)**	**[keV]**	**[°]**	**[µrad]**	**[µrad]**	**[meV/µrad]**	**[meV]**	**[meV]**	**[meV]**
**Ge**	**(0,6,10)**	**12.779**	**85.19**	**7.834**	**17**	**1.079**	**18.39**	**13.48**	**22.8**
	Equiv. Refl.: (6,6,8)								
**Si**	**(2,4,10)**	**12.504**	**77.17**	**4.203**	**3.5**	**2.92**	**10.25**	**36.5**	**37.91**
**Si**	**(1,1,11)**	**12.659**	**80.81**	**3.634**	**3.3**	**2.076**	**6.86**	**25.95**	**26.84**
	Equiv. Refl.: (5,7,7)								
**Ge**	**(0,8,8)**	**12.397**	**75.18**	**2.937**	**6.2**	**3.393**	**20.94**	**42.42**	**47.3**
**Ge**	**(1,3,11)**	**12.542**	**77.96**	**1.843**	**5.2**	**2.736**	**14.09**	**34.2**	**36.99**
	Equiv. Refl.:(1,7,9), (5,5,9)								
**Si**	**(3,5,9)**	**12.241**	**72.65**	**1.811**	**2**	**4.006**	**7.95**	**50.08**	**50.7**
**Ge**	**(1,1,11)**	**12.153**	**71.38**	**1.401**	**3.7**	**4.321**	**16.12**	**54.01**	**56.36**
	Equiv. Refl.: (5,7,7)								
**LiNbO3**	**(0,-9,6)**	**12.799**	**86.41**	**36.4**	**33**	**0.805**	**26.6**	**10.07**	**28.45**
	Equiv. Refl.: (9,0,6), (-9,9,6)								
**LiNbO3**	**(0,9,-6)**	**12.799**	**86.41**	**36.2**	**33**	**0.805**	**26.6**	**10.07**	**28.45**
	Equiv. Refl.: (9,-9,-6), (-9,0,-6)								
**LiNbO3**	**(0,-9,-6)**	**12.799**	**86.41**	**31.4**	**28.2**	**0.805**	**22.73**	**10.07**	**24.86**
	Equiv. Refl.: (9,0,-6), (-9,9,-6)								
**LiNbO3**	**(0,9,6)**	**12.799**	**86.41**	**29.2**	**28.2**	**0.805**	**22.73**	**10.07**	**24.86**
	Equiv. Refl.: (9,-9,6), (-9,0,6)								
**LiNbO3**	**(1,-7,20)**	**12.772**	**84.84**	**25.2**	**22.7**	**1.158**	**26.26**	**14.47**	**29.98**
	Equiv. Refl.: (6,1,20), (7,-1,20), (-1,-6,20), (-6,7,20), (-7,6,20)								
**LiNbO3**	**(1,6,-20)**	**12.772**	**84.84**	**24.6**	**22.7**	**1.158**	**26.26**	**14.47**	**29.98**
	Equiv. Refl.: (6,-7,-20), (7,-6,-20), (-1,7,-20), (-6,-1,-20), (-7,1,-20)								
**LiNbO3**	**(0,-8,14)**	**12.765**	**84.48**	**24**	**20.8**	**1.239**	**25.82**	**15.48**	**30.11**
	Equiv. Refl.: (8,0,14), (-8,8,14)								
**LiNbO3**	**(0,8,-14)**	**12.765**	**84.48**	**21.6**	**20.8**	**1.239**	**25.82**	**15.48**	**30.11**
	Equiv. Refl.: (8,-8,-14), (-8,0,-14)								
**LiNbO3**	**(2,-10,0)**	**12.744**	**83.6**	**21.1**	**19**	**1.439**	**27.34**	**17.99**	**32.73**
	Equiv. Refl.: (8,2,0), (10,-2,0), (-2,-8,0), (-8,10,0), (-10,8,0)								
**LiNbO3**	**(2,8,0)**	**12.744**	**83.6**	**20.8**	**19**	**1.439**	**27.34**	**17.99**	**32.73**
	Equiv. Refl.: (8,-10,0), (10,-8,0), (-2,10,0), (-8,-2,0), (-10,2,0)								
**LiNbO3**	**(1,8,-10)**	**12.694**	**81.84**	**13.7**	**12.5**	**1.84**	**23.02**	**23**	**32.54**
	Equiv. Refl.: (8,-9,-10), (9,-8,-10), (-1,9,-10), (-8,-1,-10), (-9,1,-10)								
**LiNbO3**	**(1,-9,10)**	**12.694**	**81.84**	**13.2**	**12.5**	**1.84**	**23.02**	**23**	**32.54**
	Equiv. Refl.: (8,1,10), (9,-1,10), (-1,-8,10), (-8,9,10), (-9,8,10)								
**LiNbO3**	**(4,-10,8)**	**12.639**	**80.25**	**12.1**	**10.9**	**2.203**	**24.07**	**27.54**	**36.57**
	Equiv. Refl.: (6,4,8), (10,-4,8), (-4,-6,8), (-6,10,8), (-10,6,8)								
**LiNbO3**	**(0,1,-28)**	**12.598**	**79.23**	**11.8**	**10.8**	**2.44**	**26.33**	**30.51**	**40.3**
	Equiv. Refl.: (0,-1,28), (1,0,28), (1,-1,-28), (-1,0,-28), (-1,1,28)								
**LiNbO3**	**(4,6,-8)**	**12.639**	**80.25**	**11.5**	**10.9**	**2.203**	**24.07**	**27.54**	**36.57**
	Equiv. Refl.: (6,-10,-8), (10,-6,-8), (-4,10,-8), (-6,-4,-8), (-10,4,-8)								
**Quartz**	**(6,-3,9)**	**12.802**	**86.62**	**11.4**	**9.6**	**0.757**	**7.26**	**9.47**	**11.93**
	Equiv. Refl.: (6,-3,-9), (-3,6,9), (-3,6,-9), (-3,-3,9), (-3,-3,-9)								
**LiNbO3**	**(4,-9,-14)**	**12.535**	**77.82**	**11.3**	**10.1**	**2.768**	**27.92**	**34.6**	**44.46**
	Equiv. Refl.: (5,4,-14), (9,-4,-14), (-4,-5,-14), (-5,9,-14), (-9,5,-14), (3,3,9), (3,3,-9), (3,-6,9), (3,-6,-9) (-6,3,9), (-6,3,-9)								
**LiNbO3**	**(4,5,14)**	**12.535**	**77.82**	**10.9**	**10.1**	**2.768**	**27.92**	**34.6**	**44.46**
	Equiv. Refl.: (5,-9,14), (9,-5,14), (-4,9,14), (-5,-4,14), (-9,4,14)								

Ei =	12.824	keV						ΔEg	ΔEt
**Cryst**	**Refl**	**EB**	**ΘB**	**∫ IR dΘ**	**Width**	**Ei cotΘ**	**ΔE**	**2m, 50µm**	**2m, 50µm**
	**(h,k,l)**	**[keV]**	**[°]**	**[µrad]**	**[µrad]**	**[meV/µrad]**	**[meV]**	**[meV]**	**[meV]**
**Sapph**	**(2,6,14)**	**12.741**	**83.49**	**10.8**	**8.8**	**1.463**	**12.92**	**18.29**	**22.4**
	Equiv. Refl.: (2,-8,-14), (6,2,-14), (6,-8,14), (8,-2,-14), (8,-6,14), (-2,8,14), (-2,-6,-14), (-6,8,-14), (-6,-2,14) (-8,2,14), (-8,6,-14)								
**LiNbO3**	**(1,-4,26)**	**12.661**	**80.87**	**10.6**	**9.8**	**2.061**	**20.18**	**25.77**	**32.73**
	Equiv. Refl.: (3,1,26), (4,-1,26), (-1,-3,26), (-3,4,26), (-4,3,26)								
**LiNbO3**	**(1,3,-26)**	**12.661**	**80.87**	**10.4**	**9.8**	**2.061**	**20.18**	**25.77**	**32.73**
	Equiv. Refl.: (3,-4,-26), (4,-3,-26), (-1,4,-26), (-3,-1,-26), (-4,1,-26)								
**LiNbO3**	**(1,4,-24)**	**12.481**	**76.72**	**10.3**	**9.3**	**3.026**	**28.16**	**37.82**	**47.15**
	Equiv. Refl.: (4,-5,-24), (5,-4,-24), (-1,5,-24), (-4,-1,-24), (-5,1,-24)								
**LiNbO3**	**(1,4,24)**	**12.481**	**76.72**	**10.2**	**9.2**	**3.026**	**27.97**	**37.82**	**47.04**
	Equiv. Refl.: (1,-5,24), (4,1,24), (4,-5,24), (5,-1,24), (5,-4,24), (-1,5,24), (-1,-4,24), (-4,5,24), (-4,-1,24) (-5,1,24), (-5,4,24)								
**LiNbO3**	**(1,-5,-24)**	**12.481**	**76.72**	**10.1**	**9.2**	**3.026**	**27.97**	**37.82**	**47.04**
	Equiv. Refl.: (4,1,-24), (5,-1,-24), (-1,-4,-24), (-4,5,-24), (-5,4,-24)								
**LiNbO3**	**(4,4,-18)**	**12.554**	**78.22**	**9.9**	**8.9**	**2.676**	**23.84**	**33.44**	**41.07**
	Equiv. Refl.: (4,-8,-18), (8,-4,-18), (-4,8,-18), (-4,-4,-18), (-8,4,-18)								
**LiNbO3**	**(4,4,18)**	**12.554**	**78.22**	**9.7**	**8.9**	**2.676**	**23.84**	**33.44**	**41.07**
	Equiv. Refl.: (4,-8,18), (8,-4,18), (-4,8,18), (-4,-4,18), (-8,4,18)								
**Quartz**	**(4,6,-1)**	**12.753**	**83.98**	**9.6**	**7.7**	**1.351**	**10.37**	**16.89**	**19.82**
	Equiv. Refl.: (4,-10,1), (6,4,1), (6,-10,-1), (-10,4,-1), (-10,6,1)								
**LiNbO3**	**(1,-6,22)**	**12.519**	**77.48**	**9.5**	**8.5**	**2.849**	**24.15**	**35.61**	**43.03**
	Equiv. Refl.: (5,1,22), (6,-1,22), (-1,-5,22), (-5,6,22), (-6,5,22)								
**Quartz**	**(4,5,5)**	**12.743**	**83.55**	**9.4**	**7.6**	**1.449**	**11.04**	**18.11**	**21.21**
	Equiv. Refl.: (4,-9,-5), (5,4,-5), (5,-9,5), (9,-4,-5), (9,-5,5), (-4,9,5), (-4,-5,-5), (-5,9,-5), (-5,-4,5) (-9,4,5), (-9,5,-5)								
**LiNbO3**	**(0,9,0)**	**12.514**	**77.38**	**9.3**	**8.2**	**2.87**	**23.56**	**35.88**	**42.92**
	Equiv. Refl.: (3,7,-4), (7,-10,-4), (9,-9,0), (10,-7,-4), (-3,10,-4), (-7,-3,-4), (-9,0,0), (-10,3,-4), (10,-4,1) (10,-6,-1), (-4,10,-1), (-4,-6,1), (-6,10,1), (-6,-4,-1)								
**LiNbO3**	**(1,5,-22)**	**12.519**	**77.48**	**9.2**	**8.5**	**2.849**	**24.15**	**35.61**	**43.03**
	Equiv. Refl.: (5,-6,-22), (6,-5,-22), (-1,6,-22), (-5,-1,-22), (-6,1,-22)								
**LiNbO3**	**(3,-10,4)**	**12.488**	**76.85**	**9.1**	**8.3**	**2.996**	**24.82**	**37.45**	**44.93**
	Equiv. Refl.: (7,3,4), (10,-3,4), (-3,-7,4), (-7,10,4), (-10,7,4), (9,-2,-4), (9,-7,4), (-2,9,4), (-2,-7,-4) (-7,9,-4), (-7,-2,4)								
**LiNbO3**	**(3,4,20)**	**12.31**	**73.72**	**9**	**7.9**	**3.746**	**29.59**	**46.83**	**55.39**
	Equiv. Refl.: (3,-7,-20), (4,3,-20), (4,-7,20), (7,-3,-20), (7,-4,20), (-3,7,20), (-3,-4,-20), (-4,7,-20), (-4,-3,20) (-7,3,20), (-7,4,-20)								
**LiNbO3**	**(0,-9,0)**	**12.514**	**77.38**	**8.8**	**8.2**	**2.87**	**23.56**	**35.88**	**42.92**
	Equiv. Refl.: (9,0,0), (-9,9,0)								
**Quartz**	**(2,7,4)**	**12.778**	**85.15**	**8.7**	**7.4**	**1.089**	**8.05**	**13.61**	**15.81**
	Equiv. Refl.: (2,-9,-4), (7,2,-4), (7,-9,4), (-9,2,4), (-9,7,-4)								
**Quartz**	**(5,5,0)**	**12.618**	**79.71**	**8.5**	**6.9**	**2.327**	**16.06**	**29.09**	**33.23**
	Equiv. Refl.: (5,-10,0), (10,-5,0), (-5,10,0), (-5,-5,0), (-10,5,0)								
**LiNbO3**	**(2,-9,-10)**	**12.229**	**72.47**	**8.1**	**7**	**4.05**	**28.21**	**50.63**	**57.96**
	Equiv. Refl.: (7,2,-10), (9,-2,-10), (-2,-7,-10), (-7,9,-10), (-9,7,-10)								
**LiNbO3**	**(3,6,12)**	**12.272**	**73.13**	**7.9**	**7**	**3.889**	**27.26**	**48.62**	**55.74**
	Equiv. Refl.: (3,-10,-2), (6,-9,12), (7,3,-2), (9,-6,12), (10,-3,-2), (-3,9,12), (-3,-7,-2), (-6,-3,12), (-7,10,-2) (-9,3,12), (-10,7,-2)								
**LiNbO3**	**(3,-9,-12)**	**12.272**	**73.13**	**7.8**	**7**	**3.889**	**27.26**	**48.62**	**55.74**
	Equiv. Refl.: (6,3,-12), (9,-3,-12), (-3,-6,-12), (-6,9,-12), (-9,6,-12), (7,-3,8), (7,-4,-8), (-3,7,-8), (-3,-4,8) (-4,7,8), (-4,-3,-8)								

Ei = Cryst	12.824 Refl (h,k,l)	keV EB [keV]	ΘB [°]	∫ IR dΘ [μrad]	Width [μrad]	Ei cotΘ [meV/μrad]	ΔE [meV]	ΔEg 2m, 50μm [meV]	ΔEt 2m, 50μm [meV]
**LiNbO3**	**(4,6,-2)**	**12.155**	**71.41**	**7.7**	**6.7**	**4.313**	**28.94**	**53.91**	**61.19**
	Equiv. Refl.: (6,-10,-2), (10,-6,-2), (-4,10,-2), (-6,-4,-2), (-10,4,-2), (3,4,-8), (3,-7,8), (4,3,8), (4,-7,-8) (-7,3,-8), (-7,4,8)								
**LiNbO3**	**(2,6,-16)**	**12.318**	**73.85**	**7.6**	**6.7**	**3.714**	**24.92**	**46.42**	**52.69**
	Equiv. Refl.: (2,7,10), (3,7,2), (6,-8,-16), (7,-9,10), (7,-10,2), (8,-6,-16), (9,-7,10), (10,-7,2), (-2,8,-16) (-2,9,10), (-3,10,2), (-6,-2,-16), (-7,-2,10), (-7,-3,2), (-8,2,-16), (-9,2,10), (-10,3,2)								
**LiNbO3**	**(1,8,8)**	**12.407**	**75.35**	**7.5**	**6.9**	**3.352**	**23.08**	**41.89**	**47.83**
	Equiv. Refl.: (1,-9,-8), (4,-10,2), (6,4,2), (8,1,-8), (8,-9,8), (9,-1,-8), (9,-8,8), (10,-4,2), (-1,9,8) (-1,-8,-8), (-4,-6,2), (-6,10,2), (-8,9,-8), (-8,-1,8), (-9,1,8), (-9,8,-8), (-10,6,2)								
**LiNbO3**	**(2,-8,16)**	**12.318**	**73.85**	**7.2**	**6.7**	**3.714**	**24.92**	**46.42**	**52.69**
	Equiv. Refl.: (6,2,16), (8,-2,16), (-2,-6,16), (-6,8,16), (-8,6,16), (2,2,24), (2,2,-24), (2,-4,24), (2,-4,-24) (4,-2,24), (4,-2,-24), (-2,4,24), (-2,4,-24), (-2,-2,24), (-2,-2,-24), (-4,2,24), (-4,2,-24)								
**LiNbO3**	**(1,2,26)**	**12.195**	**71.98**	**7.1**	**6.3**	**4.173**	**26.49**	**52.16**	**58.5**
	Equiv. Refl.: (2,-3,26), (3,-2,26), (-1,3,26), (-2,-1,26), (-3,1,26)								
**LiNbO3**	**(1,-3,-26)**	**12.195**	**71.98**	**7**	**6.3**	**4.173**	**26.49**	**52.16**	**58.5**
	Equiv. Refl.: (2,1,-26), (3,-1,-26), (-1,-2,-26), (-2,3,-26), (-3,2,-26)								
**LiNbO3**	**(3,-9,12)**	**12.272**	**73.13**	**6.8**	**6.2**	**3.889**	**24.07**	**48.62**	**54.25**
	Equiv. Refl.: (6,3,12), (9,-3,12), (-3,-6,12), (-6,9,12), (-9,6,12)								
**LiNbO3**	**(3,6,-12)**	**12.272**	**73.13**	**6.7**	**6.2**	**3.889**	**24.07**	**48.62**	**54.25**
	Equiv. Refl.: (5,5,6), (5,-10,6), (6,-9,-12), (9,-6,-12), (10,-5,6), (-3,9,-12), (-5,10,6), (-5,-5,6), (-6,-3,-12) (-9,3,-12), (-10,5,6), (0,2,-26), (0,-2,26), (2,0,26), (2,-2,-26), (-2,0,-26), (-2,2,26)								
**LiNbO3**	**(4,6,4)**	**12.253**	**72.84**	**6.6**	**6**	**3.959**	**23.77**	**49.49**	**54.9**
	Equiv. Refl.: (6,-10,4), (10,-6,4), (-4,10,4), (-6,-4,4), (-10,4,4)								
**LiNbO3**	**(2,-6,-22)**	**12.285**	**73.33**	**6.5**	**6**	**3.84**	**22.91**	**48**	**53.19**
	Equiv. Refl.: (3,-8,-16), (4,2,-22), (4,-10,-4), (5,3,-16), (6,4,-4), (6,-2,-22), (8,-3,-16), (10,-4,-4), (-2,-4,-22) (-3,-5,-16), (-4,6,-22), (-4,-6,-4), (-5,8,-16), (-6,4,-22), (-6,10,-4), (-8,5,-16), (-10,6,-4)								
**LiNbO3**	**(2,4,22)**	**12.285**	**73.33**	**6.4**	**6**	**3.84**	**22.91**	**48**	**53.19**
	Equiv. Refl.: (4,-6,22), (5,5,-6), (5,-10,-6), (6,-4,22), (10,-5,-6), (-2,6,22), (-4,-2,22), (-5,10,-6), (-5,-5,-6) (-6,2,22), (-10,5,-6), (7,-3,-8), (7,-4,8), (-3,7,8), (-3,-4,-8), (-4,7,-8), (-4,-3,8)								
**Quartz**	**(3,4,8)**	**12.757**	**84.15**	**6.3**	**5.4**	**1.314**	**7.07**	**16.43**	**17.88**
	Equiv. Refl.: (3,-7,-8), (4,3,-8), (4,-7,8), (-7,3,8), (-7,4,-8)								
**LiNbO3**	**(0,-7,16)**	**12.08**	**70.39**	**6**	**5.4**	**4.569**	**24.63**	**57.11**	**62.2**
	Equiv. Refl.: (7,0,16), (-7,7,16)								
**LiNbO3**	**(0,7,-16)**	**12.08**	**70.39**	**5.8**	**5.4**	**4.569**	**24.63**	**57.11**	**62.2**
	Equiv. Refl.: (3,5,16), (5,-8,16), (7,-7,-16), (8,-5,16), (-3,8,16), (-5,-3,16), (-7,0,-16), (-8,3,16)								
**Sapph**	**(3,5,-14)**	**12.472**	**76.54**	**5.4**	**4.4**	**3.068**	**13.56**	**38.35**	**40.68**
	Equiv. Refl.: (3,-8,14), (5,3,14), (5,-8,-14), (8,-3,14), (8,-5,-14), (-3,8,-14), (-3,-5,14), (-5,8,14), (-5,-3,-14) (-8,3,-14), (-8,5,14)								
**Sapph**	**(0,6,18)**	**12.461**	**76.34**	**5.2**	**4.1**	**3.116**	**12.74**	**38.95**	**40.98**
	Equiv. Refl.: (0,6,-18), (0,-6,18), (0,-6,-18), (6,0,18), (6,0,-18), (6,-6,18), (6,-6,-18), (-6,0,18), (-6,0,-18) (-6,6,18), (-6,6,-18), (10,-4,-1), (10,-6,1), (-4,10,1), (-4,-6,-1), (-6,10,-1), (-6,-4,1)								
**Quartz**	**(0,8,-4)**	**12.526**	**77.63**	**5.1**	**4.1**	**2.812**	**11.46**	**35.15**	**36.97**
	Equiv. Refl.: (0,-8,4), (1,8,-2), (1,-9,2), (4,6,1), (4,-10,-1), (6,4,-1), (6,-10,1), (8,0,4), (8,1,2) (8,-8,-4), (8,-9,-2), (9,-1,2), (9,-8,-2), (-1,9,-2), (-1,-8,2), (-8,0,-4), (-8,8,4), (-8,9,2) (-8,-1,-2), (-9,1,-2), (-9,8,2), (-10,4,1), (-10,6,-1)								
**Sapph**	**(1,5,20)**	**12.7**	**82.02**	**4.9**	**4.1**	**1.798**	**7.46**	**22.47**	**23.68**
	Equiv. Refl.: (1,-6,-20), (4,5,-10), (4,-9,10), (5,1,-20), (5,4,10), (5,-6,20), (5,-9,-10), (6,-1,-20), (6,-5,20) (9,-4,10), (9,-5,-10), (-1,6,20), (-1,-5,-20), (-4,9,-10), (-4,-5,10), (-5,6,-20), (-5,9,10), (-5,-1,20) (-5,-4,-10), (-6,1,20), (-6,5,-20), (-9,4,-10), (-9,5,10), (3,5,6), (3,-8,-6), (5,3,-6), (5,-8,6) (8,-3,-6), (8,-5,6), (-3,8,6), (-3,-5,-6), (-5,8,-6), (-5,-3,6), (-8,3,6), (-8,5,-6)								

Ei =	12.824	keV						ΔEg	ΔEt
Cryst	Refl	EB	ΘB	∫ IR dΘ	Width	Ei cotΘ	ΔE	2m, 50µm	2m, 50µm
	(h,k,l)	[keV]	[°]	[µrad]	[µrad]	[meV/µrad]	[meV]	[meV]	[meV]
**Quartz**	**(7,-1,7)**	**12.48**	**76.7**	**4.6**	**3.5**	**3.032**	**10.64**	**37.9**	**39.37**
	Equiv. Refl.: (7,-6,-7), (-1,7,-7), (-1,-6,7), (-6,7,7), (-6,-1,-7)								
**LiNbO3**	**(2,7,13)**	**12.78**	**85.27**	**4.5**	**6.3**	**1.061**	**6.72**	**13.27**	**14.87**
	Equiv. Refl.: (2,-9,-13), (7,2,-13), (7,-9,13), (9,-2,-13), (9,-7,13), (-2,9,13), (-2,-7,-13), (-7,9,-13), (-7,-2,13) (-9,2,13), (-9,7,-13)								
**Quartz**	**(1,6,-7)**	**12.48**	**76.7**	**4.4**	**3.5**	**3.032**	**10.63**	**37.9**	**39.37**
	Equiv. Refl.: (1,-7,7), (6,1,7), (6,-7,-7), (-7,1,-7), (-7,6,7)								
**Quartz**	**(9,-4,5)**	**12.743**	**83.55**	**4.2**	**3.6**	**1.449**	**5.19**	**18.11**	**18.84**
	Equiv. Refl.: (9,-5,-5), (-4,9,-5), (-4,-5,5), (-5,9,5), (-5,-4,-5)								
**Sapph**	**(0,8,4)**	**12.186**	**71.85**	**4.1**	**3.2**	**4.204**	**13.41**	**52.55**	**54.24**
	Equiv. Refl.: (0,-8,-4), (8,0,-4), (8,-8,4), (-8,0,4), (-8,8,-4), (4,5,-5), (4,-9,5), (5,4,5), (5,-9,-5) (-9,4,-5), (-9,5,5)								
**Sapph**	**(2,4,-20)**	**12.43**	**75.75**	**4**	**3.2**	**3.256**	**10.52**	**40.7**	**42.04**
	Equiv. Refl.: (2,-6,20), (4,2,20), (4,-6,-20), (6,-2,20), (6,-4,-20), (-2,6,-20), (-2,-4,20), (-4,6,20), (-4,-2,-20) (-6,2,-20), (-6,4,20), (0,6,8), (0,8,4), (0,-6,-8), (0,-8,-4), (4,-1,-10), (4,-3,10), (6,0,-8) (6,-6,8), (8,0,-4), (8,-8,4), (9,-3,4), (9,-6,-4), (-1,4,10), (-1,-3,-10), (-3,4,-10), (-3,9,-4) (-3,-1,10), (-3,-6,4), (-6,0,8), (-6,6,-8), (-6,9,4), (-6,-3,-4), (-8,0,4), (-8,8,-4)								
**Quartz**	**(1,5,8)**	**12.248**	**72.76**	**3.9**	**3**	**3.98**	**11.94**	**49.75**	**51.16**
	Equiv. Refl.: (1,-6,-8), (5,1,-8), (5,-6,8), (6,-1,-8), (6,-5,8), (9,-1,1), (9,-8,-1), (-1,6,8), (-1,9,-1) (-1,-5,-8), (-1,-8,1), (-5,6,-8), (-5,-1,8), (-6,1,8), (-6,5,-8), (-8,9,1), (-8,-1,-1)								
**Sapph**	**(2,7,4)**	**12.461**	**76.34**	**3.8**	**3.1**	**3.116**	**9.67**	**38.95**	**40.14**
	Equiv. Refl.: (2,-9,-4), (7,2,-4), (7,-9,4), (9,-2,-4), (9,-7,4), (-2,9,4), (-2,-7,-4), (-7,9,-4), (-7,-2,4) (-9,2,4), (-9,7,-4), (1,3,10), (1,-4,-10), (3,1,-10), (3,6,-4), (3,-4,10), (3,-9,4), (6,3,4) (6,-9,-4), (7,-2,-7), (7,-5,7), (-2,7,7), (-2,-5,-7), (-4,1,10), (-4,3,-10), (-5,7,-7), (-5,-2,7) (-9,3,-4), (-9,6,4)								
**Quartz**	**(2,5,7)**	**12.135**	**71.13**	**3.7**	**2.8**	**4.383**	**12.46**	**54.78**	**56.18**
	Equiv. Refl.: (2,-7,-7), (5,2,-7), (5,-7,7), (-7,2,7), (-7,5,-7)								
**Quartz**	**(1,8,-1)**	**12.501**	**77.12**	**3.6**	**3**	**2.933**	**8.8**	**36.66**	**37.7**
	Equiv. Refl.: (1,-9,1), (2,6,6), (2,-8,-6), (6,2,-6), (6,-8,6), (8,1,1), (8,-9,-1), (-8,2,6), (-8,6,-6) (-9,1,-1), (-9,8,1)								
**Quartz**	**(8,-2,-6)**	**12.56**	**78.35**	**3.5**	**2.9**	**2.644**	**7.71**	**33.05**	**33.94**
	Equiv. Refl.: (8,-6,6), (9,-1,0), (9,-8,0), (-1,9,0), (-1,-8,0), (-2,8,6), (-2,-6,-6), (-6,8,-6), (-6,-2,6) (-8,9,0), (-8,-1,0)								
**Quartz**	**(1,8,0)**	**12.448**	**76.1**	**3.4**	**2.7**	**3.174**	**8.64**	**39.67**	**40.6**
	Equiv. Refl.: (1,8,2), (1,-9,0), (1,-9,-2), (2,7,3), (2,-9,-3), (7,2,-3), (7,-9,3), (8,1,0), (8,1,-2) (8,-9,0), (8,-9,2), (9,-2,-3), (9,-7,3), (-2,9,3), (-2,-7,-3), (-7,9,-3), (-7,-2,3), (-9,1,0) (-9,1,2), (-9,2,3), (-9,7,-3), (-9,8,0), (-9,8,-2)								
**Quartz**	**(4,5,-4)**	**12.27**	**73.09**	**3.3**	**2.6**	**3.899**	**10.07**	**48.73**	**49.76**
	Equiv. Refl.: (4,-9,4), (5,4,4), (5,-9,-4), (9,-4,4), (9,-5,-4), (-4,9,-4), (-4,-5,4), (-5,9,4), (-5,-4,-4) (-9,4,-4), (-9,5,4)								
**Quartz**	**(1,7,5)**	**12.405**	**75.32**	**3.2**	**2.4**	**3.36**	**8.16**	**42**	**42.78**
	Equiv. Refl.: (1,-8,-5), (7,1,-5), (7,-8,5), (-8,1,5), (-8,7,-5)								
**Quartz**	**(0,3,10)**	**12.275**	**73.17**	**3.1**	**2.7**	**3.879**	**10.31**	**48.49**	**49.57**
	Equiv. Refl.: (0,-3,-10), (1,7,-5), (1,-8,5), (2,2,10), (2,2,-10), (2,-4,10), (2,-4,-10), (3,0,-10), (3,-3,10) (4,-2,10), (4,-2,-10), (7,1,5), (7,-8,-5), (8,-1,-5), (8,-7,5), (9,-1,-2), (9,-8,2), (-1,8,5) (-1,9,2), (-1,-7,-5), (-1,-8,-2), (-2,4,10), (-2,4,-10), (-2,-2,10), (-2,-2,-10), (-3,0,10), (-3,3,-10) (-4,2,10), (-4,2,-10), (-7,8,-5), (-7,-1,5), (-8,1,-5), (-8,7,5), (-8,9,-2), (-8,-1,2)								
**Quartz**	**(3,6,4)**	**12.441**	**75.97**	**3**	**2.3**	**3.205**	**7.47**	**40.06**	**40.75**
	Equiv. Refl.: (3,-9,-4), (6,3,-4), (6,-9,4), (8,-1,5), (8,-7,-5), (9,-3,-4), (9,-6,4), (-1,8,-5), (-1,-7,5) (-3,9,4), (-3,-6,-4), (-6,9,-4), (-6,-3,4), (-7,8,5), (-7,-1,-5), (-9,3,4), (-9,6,-4)								

**Ei =**<br>**Cryst**	**12.824**<br>**Refl**<br>**(h,k,l)**	**keV**<br>**EB**<br>**[keV]**	**ΘB**<br>**[°]**	**∫ IR dΘ**<br>**[µrad]**	**Width**<br>**[µrad]**	**Ei cotΘ**<br>**[meV/µrad]**	**ΔE**<br>**[meV]**	**ΔEg**<br>**2m, 50µm**<br>**[meV]**	**ΔEt**<br>**2m, 50µm**<br>**[meV]**
**Quartz**	**(9,-2,4)**	**12.778**	**85.15**	**2.6**	**2.6**	**1.089**	**2.85**	**13.61**	**13.9**
	Equiv. Refl.: (9,-7,-4), (-2,9,-4), (-2,-7,4), (-7,9,4), (-7,-2,-4)								
**Sapph**	**(4,4,15)**	**12.645**	**80.41**	**2.5**	**2.4**	**2.166**	**5.26**	**27.07**	**27.58**
	Equiv. Refl.: (4,4,-15), (4,-8,15), (4,-8,-15), (8,-4,15), (8,-4,-15), (-4,8,15), (-4,8,-15), (-4,-4,15), (-4,-4,-15) (-8,4,15), (-8,4,-15)								
**Sapph**	**(2,7,7)**	**12.759**	**84.25**	**2.4**	**2.7**	**1.292**	**3.47**	**16.15**	**16.52**
	Equiv. Refl.: (2,-9,-7), (7,2,-7), (7,-9,7), (9,-2,-7), (9,-7,7), (-2,9,7), (-2,-7,-7), (-7,9,-7), (-7,-2,7) (-9,2,7), (-9,7,-7), (0,6,-8), (0,-6,8), (6,0,8), (6,-6,-8), (-6,0,-8), (-6,6,8)								
**Sapph**	**(2,3,23)**	**12.787**	**85.66**	**2.3**	**2.9**	**0.974**	**2.86**	**12.17**	**12.5**
	Equiv. Refl.: (2,-5,-23), (3,2,-23), (3,-5,23), (5,-2,-23), (5,-3,23), (-2,5,23), (-2,-3,-23), (-3,5,-23), (-3,-2,23) (-5,2,23), (-5,3,-23), (9,-2,2), (9,-7,-2), (-2,9,-2), (-2,-7,2), (-7,9,2), (-7,-2,-2)								
**Quartz**	**(2,7,-4)**	**12.778**	**85.15**	**2.1**	**2.6**	**1.089**	**2.85**	**13.61**	**13.9**
	Equiv. Refl.: (2,-9,4), (7,2,4), (7,-9,-4), (-9,2,-4), (-9,7,4)								
**LiNbO3**	**(2,8,3)**	**12.814**	**87.79**	**2**	**5.5**	**0.495**	**2.7**	**6.19**	**6.76**
	Equiv. Refl.: (2,8,-3), (2,-10,3), (2,-10,-3), (8,2,3), (8,2,-3), (8,-10,3), (8,-10,-3), (10,-2,3), (10,-2,-3) (10,-8,3), (10,-8,-3), (-2,10,3), (-2,10,-3), (-2,-8,3), (-2,-8,-3), (-8,10,3), (-8,10,-3), (-8,-2,3) (-8,-2,-3), (-10,2,3), (-10,2,-3), (-10,8,3), (-10,8,-3), (0,1,-11), (0,-1,11), (1,0,11), (1,-1,-11) (-1,0,-11), (-1,1,11)								
**Quartz**	**(2,7,-2)**	**12.145**	**71.27**	**1.9**	**2**	**4.349**	**8.54**	**54.36**	**55.03**
	Equiv. Refl.: (2,-9,2), (7,2,2), (7,-9,-2), (-9,2,-2), (-9,7,2)								
**LiNbO3**	**(2,4,-23)**	**12.646**	**80.44**	**1.8**	**3**	**2.16**	**6.38**	**27**	**27.74**
	Equiv. Refl.: (2,-6,23), (4,2,23), (4,-6,-23), (6,-2,23), (6,-4,-23), (-2,6,-23), (-2,-4,23), (-4,6,23), (-4,-2,-23) (-6,2,-23), (-6,4,23), (0,5,-20), (0,-5,20), (2,6,-13), (2,-8,13), (5,0,20), (5,-5,-20), (6,2,13) (6,-8,-13), (8,-2,13), (8,-6,-13), (-2,8,-13), (-2,-6,13), (-5,0,-20), (-5,5,20), (-6,8,13), (-6,-2,-13) (-8,2,-13), (-8,6,13), (2,3,9), (2,-5,-9), (3,2,-9), (3,-5,9), (4,6,0), (4,-10,0), (5,-2,-9) (5,-3,9), (6,4,0), (6,-10,0), (10,-4,0), (10,-6,0), (-2,5,9), (-2,-3,-9), (-3,5,-9), (-3,-2,9) (-4,10,0), (-4,-6,0), (-5,2,9), (-5,3,-9), (-6,10,0), (-6,-4,0), (-10,4,0), (-10,6,0)								
**LiNbO3**	**(1,6,19)**	**12.463**	**76.37**	**1.7**	**2.5**	**3.109**	**7.82**	**38.86**	**39.64**
	Equiv. Refl.: (1,-7,-19), (6,1,-19), (6,-7,19), (7,-1,-19), (7,-6,19), (-1,7,19), (-1,-6,-19), (-6,7,-19), (-6,-1,19) (-7,1,19), (-7,6,-19), (1,4,21), (1,4,-21), (1,-5,21), (1,-5,-21), (4,1,21), (4,1,-21), (4,-5,21) (4,-5,-21), (5,-1,21), (5,-1,-21), (5,-4,21), (5,-4,-21), (-1,5,21), (-1,5,-21), (-1,-4,21), (-1,-4,-21) (-4,5,21), (-4,5,-21), (-4,-1,21), (-4,-1,-21), (-5,1,21), (-5,1,-21), (-5,4,21), (-5,4,-21), (2,3,-9) (2,-5,9), (3,2,9), (3,-5,-9), (5,-2,9), (5,-3,-9), (-2,5,-9), (-2,-3,9), (-3,5,9), (-3,-2,-9) (-5,2,-9), (-5,3,9)								
**Quartz**	**(3,-1,-10)**	**12.101**	**70.66**	**1.6**	**1.6**	**4.5**	**7.29**	**56.25**	**56.72**
	Equiv. Refl.: (3,-2,10), (8,-2,6), (8,-6,-6), (-1,3,10), (-1,-2,-10), (-2,3,-10), (-2,8,-6), (-2,-1,10), (-2,-6,6) (-6,8,6), (-6,-2,-6)								
**LiNbO3**	**(1,1,27)**	**12.312**	**73.75**	**1.5**	**2.2**	**3.738**	**8.31**	**46.73**	**47.46**
	Equiv. Refl.: (1,1,-27), (1,-2,27), (1,-2,-27), (2,-1,27), (2,-1,-27), (-1,2,27), (-1,2,-27), (-1,-1,27), (-1,-1,-27) (-2,1,27), (-2,1,-27), (0,1,26), (0,-1,-26), (1,0,-26), (1,-1,26), (-1,0,26), (-1,1,-26), (1,2,10) (1,2,-10), (1,-3,10), (1,-3,-10), (2,1,10), (2,1,-10), (2,-3,10), (2,-3,-10), (3,-1,10), (3,-2,-10) (-1,3,-10), (-1,-2,10), (-2,3,10), (-2,-1,-10), (-3,1,10), (-3,1,-10), (-3,2,10), (-3,2,-10)								

Ei =	12.824	keV						ΔEg	ΔEt
Cryst	Refl	EB	ΘB	∫ IR dΘ	Width	Ei cotΘ	ΔE	2m, 50µm	2m, 50µm
	(h,k,l)	[keV]	[°]	[µrad]	[µrad]	[meV/µrad]	[meV]	[meV]	[meV]
**LiNbO3**	**(2,7,-11)**	**12.399**	**75.21**	**1.4**	**2.2**	**3.386**	**7.39**	**42.32**	**42.96**
	Equiv. Refl.: (2,-9,11), (4,6,7), (4,-10,-7), (6,4,-7), (6,-10,7), (7,2,11), (7,-9,-11), (9,-2,11), (9,-7,-11)<br>(10,-4,-7), (10,-6,7), (-2,9,-11), (-2,-7,11), (-4,10,7), (-4,-6,-7), (-6,10,-7), (-6,-4,7), (-7,9,11)<br>(-7,-2,-11), (-9,2,-11), (-9,7,11), (-10,4,7), (-10,6,-7), (1,5,-19), (1,-6,19), (4,5,-7), (4,-9,7)<br>(5,1,19), (5,4,7), (5,-6,-19), (5,-9,-7), (6,-1,19), (6,-5,-19), (9,-4,7), (9,-5,-7), (-1,6,-19)<br>(-1,-5,19), (-4,9,-7), (-4,-5,7), (-5,6,19), (-5,9,7), (-5,-1,-19), (-5,-4,-7), (-6,1,-19), (-6,5,19)<br>(-9,4,-7), (-9,5,7), (2,6,-6), (2,-8,6), (4,5,4), (4,-9,-4), (5,4,-4), (5,5,1), (5,5,-1)<br>(5,-9,4), (5,-10,1), (5,-10,-1), (6,2,6), (6,-8,-6), (9,-3,3), (9,-6,-3), (10,-5,1), (10,-5,-1)<br>(-3,9,-3), (-3,-6,3), (-5,10,1), (-5,10,-1), (-5,-5,1), (-5,-5,-1), (-6,9,3), (-6,-3,-3), (-8,2,-6)<br>(-8,6,6), (-9,4,4), (-9,5,-4), (-10,5,1), (-10,5,-1)								
**Sapph**	**(1,6,16)**	**12.476**	**76.61**	**1.3**	**1.5**	**3.052**	**4.67**	**38.15**	**38.43**
	Equiv. Refl.: (1,6,-17), (1,-7,17), (1,-7,-16), (2,6,11), (2,-8,-11), (6,1,17), (6,1,-16), (6,2,-11), (6,-7,16)<br>(6,-7,-17), (6,-8,11), (7,-1,17), (7,-1,-16), (7,-6,16), (7,-6,-17), (8,-2,-11), (8,-6,11), (-1,7,16)<br>(-1,7,-17), (-1,-6,17), (-1,-6,-16), (-2,8,11), (-2,-6,-11), (-6,7,17), (-6,7,-16), (-6,8,-11), (-6,-1,16)<br>(-6,-1,-17), (-6,-2,11), (-7,1,16), (-7,1,-17), (-7,6,17), (-7,6,-16), (-8,2,11), (-8,6,-11), (2,5,-7)<br>(2,-7,7), (3,6,-3), (3,-9,3), (4,4,6), (4,4,-6), (4,-8,6), (4,-8,-6), (5,2,7), (5,-7,-7)<br>(6,3,3), (6,-9,-3), (7,-2,7), (7,-5,-7), (9,-3,-3), (9,-4,-4), (9,-5,4), (9,-6,3), (-2,7,-7)<br>(-2,-5,7), (-3,9,3), (-3,-6,-3), (-4,9,4), (-4,-5,-4), (-5,7,7), (-5,9,-4), (-5,-2,-7), (-5,-4,4)<br>(-6,9,-3), (-6,-3,3), (-7,2,-7), (-7,5,7), (-8,4,6), (-8,4,-6), (-9,3,-3), (-9,6,3)								
**Quartz**	**(1,3,-10)**	**12.616**	**79.66**	**1.2**	**1.3**	**2.339**	**3**	**29.23**	**29.39**
	Equiv. Refl.: (1,-4,10), (3,1,10), (3,6,3), (3,-4,-10), (3,-9,-3), (6,3,-3), (6,-9,3), (8,-4,6), (8,-4,-6)<br>(-4,1,-10), (-4,3,10), (-4,8,6), (-4,8,-6), (-4,-4,6), (-4,-4,-6), (-9,3,3), (-9,6,-3)								
**LiNbO3**	**(3,7,-7)**	**12.749**	**83.81**	**1.1**	**2.4**	**1.391**	**3.4**	**17.39**	**17.72**
	Equiv. Refl.: (3,-10,7), (4,6,-5), (4,-10,5), (6,4,5), (6,-10,-5), (7,3,7), (7,-10,-7), (10,-3,7), (10,-4,5)<br>(10,-6,-5), (10,-7,-7), (-3,10,-7), (-3,-7,7), (-4,10,-5), (-4,-6,5), (-6,10,5), (-6,-4,-5), (-7,10,7)<br>(-7,-3,-7), (-10,3,-7), (-10,4,-5), (-10,6,5), (-10,7,7), (2,7,-5), (2,-9,5), (7,2,5), (7,-9,-5)<br>(9,-2,5), (9,-7,-5), (-2,9,-5), (-2,-7,5), (-7,9,5), (-7,-2,-5), (-9,2,-5), (-9,7,5), (1,6,7)<br>(1,-7,-7), (6,1,-7), (6,-7,7), (7,-1,-7), (7,-6,7), (-1,7,7), (-1,-6,-7), (-6,7,-7), (-6,-1,7)<br>(-7,1,7), (-7,6,-7)								
**LiNbO3**	**(2,5,21)**	**12.79**	**85.84**	**1**	**2.8**	**0.932**	**2.57**	**11.65**	**11.93**
	Equiv. Refl.: (2,5,-21), (2,-7,21), (2,-7,-21), (4,6,1), (4,-10,-1), (5,2,21), (5,2,-21), (5,-7,21), (5,-7,-21)<br>(6,4,-1), (6,-10,1), (7,-2,21), (7,-2,-21), (7,-5,21), (7,-5,-21), (10,-4,-1), (10,-6,1), (-2,7,21)<br>(-2,7,-21), (-2,-5,21), (-2,-5,-21), (-4,10,1), (-4,-6,-1), (-5,7,21), (-5,7,-21), (-5,-2,21), (-5,-2,-21)<br>(-6,10,-1), (-6,-4,1), (-7,2,21), (-7,2,-21), (-7,5,21), (-7,5,-21), (-10,4,1), (-10,6,-1), (0,4,-22)<br>(0,-4,22), (1,7,9), (1,7,-9), (1,-8,9), (1,-8,-9), (4,0,22), (4,-4,-22), (7,1,9), (7,1,-9)<br>(7,-8,9), (7,-8,-9), (8,-1,9), (8,-1,-9), (8,-7,9), (8,-7,-9), (-1,8,9), (-1,8,-9), (-1,-7,9)<br>(-1,-7,-9), (-4,0,-22), (-4,4,22), (-7,8,9), (-7,8,-9), (-7,-1,9), (-7,-1,-9), (-8,1,9), (-8,1,-9)<br>(-8,7,9), (-8,7,-9), (1,4,9), (1,-5,-9), (3,5,-6), (3,-8,6), (4,1,-9), (4,-1,10), (4,-3,-10)<br>(4,-5,9), (5,3,6), (5,-8,-6), (-1,-4,-10), (-1,-3,10), (-3,4,10), (-3,-1,-10), (-5,1,9), (-5,4,-9)<br>(-8,3,-6), (-8,5,6)								
**Sapph**	**(2,5,18)**	**12.731**	**83.09**	**0.9**	**1.3**	**1.553**	**2.01**	**19.41**	**19.52**
	Equiv. Refl.: (2,5,-18), (2,7,1), (2,-7,18), (2,-7,-18), (2,-9,-1), (5,2,18), (5,2,-18), (5,-7,18), (5,-7,-18)<br>(7,2,-1), (7,-2,18), (7,-2,-18), (7,-5,18), (7,-5,-18), (7,-9,1), (9,-2,-1), (9,-7,1), (-2,7,18)<br>(-2,7,-18), (-2,9,1), (-2,-5,18), (-2,-5,-18), (-2,-7,-1), (-5,7,18), (-5,7,-18), (-5,-2,18), (-5,-2,-18)<br>(-7,2,18), (-7,2,-18), (-7,5,18), (-7,5,-18), (-7,9,-1), (-7,-2,1), (-9,2,1), (-9,7,-1), (5,-1,-9)<br>(5,-4,9), (8,-3,6), (8,-5,-6), (-1,5,9), (-1,-4,-9), (-3,8,-6), (-3,-5,6), (-4,5,-9), (-4,-1,9)<br>(-5,8,6), (-5,-3,-6)								
**Sapph**	**(2,4,19)**	**12.067**	**70.21**	**0.8**	**1**	**4.613**	**4.44**	**57.67**	**57.84**
	Equiv. Refl.: (2,-6,-19), (3,6,6), (3,6,-6), (3,-9,6), (3,-9,-6), (4,2,-19), (4,-6,19), (6,3,6), (6,3,-6)<br>(6,-2,-19), (6,-4,19), (6,-9,6), (6,-9,-6), (9,-3,6), (9,-3,-6), (9,-6,6), (9,-6,-6), (-2,6,19)<br>(-2,-4,-19), (-3,9,6), (-3,9,-6), (-3,-6,6), (-3,-6,-6), (-4,6,-19), (-4,-2,19), (-6,2,19), (-6,4,-19)<br>(-6,9,6), (-6,9,-6), (-6,-3,6), (-6,-3,-6), (-9,3,6), (-9,3,-6), (-9,6,6), (-9,6,-6), (2,7,2)								

(2,-9,-2), (7,2,-2), (7,-9,2), (9,-2,-2), (9,-7,2), (-2,9,2), (-2,-7,-2), (-7,9,-2), (-7,-2,2)
(-9,2,2), (-9,7,-2)

Ei =<br>Cryst	12.824<br>Refl<br>(h,k,l)	keV<br>EB<br>[keV]	ΘB<br>[°]	∫ IR dΘ<br>[µrad]	Width<br>[µrad]	Ei cotΘ<br>[meV/µrad]	ΔE<br>[meV]	ΔEg<br>2m, 50µm<br>[meV]	ΔEt<br>2m, 50µm<br>[meV]
**LiNbO3**	**(1,3,25)**	**12.252**	**72.82**	**0.7**	**1.3**	**3.964**	**5.1**	**49.55**	**49.81**
	Equiv. Refl.: (1,-4,-25), (3,1,-25), (3,-4,25), (4,-1,-25), (4,-3,25), (-1,4,25), (-1,-3,-25), (-3,4,-25), (-3,-1,25) (-4,1,25), (-4,3,-25), (1,2,-25), (1,-3,25), (2,1,25), (2,-3,-25), (3,5,13), (3,-1,25), (3,-2,-25) (3,-8,-13), (5,3,-13), (5,-8,13), (8,-3,-13), (8,-5,13), (-1,3,-25), (-1,-2,25), (-2,3,25), (-2,-1,-25) (-3,1,-25), (-3,2,25), (-3,8,13), (-3,-5,-13), (-5,8,-13), (-5,-3,13), (-8,3,13), (-8,5,-13), (0,8,-3) (0,-8,3), (8,0,3), (8,-8,-3), (-8,0,-3), (-8,8,3)								
**LiNbO3**	**(3,7,5)**	**12.56**	**78.34**	**0.6**	**1.3**	**2.645**	**3.56**	**33.07**	**33.26**
	Equiv. Refl.: (3,-10,-5), (4,5,-13), (4,-9,13), (5,4,13), (5,-9,-13), (7,3,-5), (7,-10,5), (9,-4,13), (9,-5,-13) (10,-3,-5), (10,-7,5), (-3,10,5), (-3,-7,-5), (-4,9,-13), (-4,-5,13), (-5,9,13), (-5,-4,-13), (-7,10,-5) (-7,-3,5), (-9,4,-13), (-9,5,13), (-10,3,5), (-10,7,-5), (0,7,14), (0,-7,-14), (2,3,-22), (2,7,-2) (2,-5,22), (2,-9,2), (3,2,22), (3,-5,-22), (5,-2,22), (5,-3,-22), (7,0,-14), (7,2,2), (7,-7,14) (7,-9,-2), (9,-2,2), (9,-7,-2), (-2,5,-22), (-2,9,-2), (-2,-3,22), (-2,-7,2), (-3,5,22), (-3,-2,-22) (-5,2,-22), (-5,3,22), (-7,0,14), (-7,7,-14), (-7,9,2), (-7,-2,-2), (-9,2,-2), (-9,7,2)								
**LiNbO3**	**(3,7,-1)**	**12.367**	**74.66**	**0.5**	**1.1**	**3.518**	**3.74**	**43.98**	**44.14**
	Equiv. Refl.: (3,-10,1), (7,3,1), (7,-10,-1), (10,-3,1), (10,-7,-1), (-3,10,-1), (-3,-7,1), (-7,10,1), (-7,-3,-1) (-10,3,-1), (-10,7,1), (0,3,-10), (0,-3,10), (1,8,1), (1,-9,-1), (3,0,10), (3,-3,-10), (8,1,-1) (8,-9,1), (-3,0,-10), (-3,3,10), (-9,1,1), (-9,8,-1)								
**LiNbO3**	**(2,6,17)**	**12.583**	**78.87**	**0.4**	**1**	**2.522**	**2.63**	**31.53**	**31.64**
	Equiv. Refl.: (2,-8,-17), (6,2,-17), (6,-8,17), (8,-2,-17), (8,-6,17), (-2,8,17), (-2,-6,-17), (-6,8,-17), (-6,-2,17) (-8,2,17), (-8,6,-17), (0,8,-8), (0,-8,8), (4,5,8), (4,-9,-8), (5,4,-8), (5,-9,8), (8,0,8) (8,-8,-8), (9,-4,-8), (9,-5,8), (-4,9,8), (-4,-5,-8), (-5,9,-8), (-5,-4,8), (-8,0,-8), (-8,8,8) (-9,4,8), (-9,5,-8), (1,4,-9), (1,-5,9), (4,1,9), (4,-5,-9), (5,-1,9), (5,-4,-9), (6,-1,8) (6,-5,-8), (-1,5,-9), (-1,6,-8), (-1,-4,9), (-1,-5,8), (-4,5,9), (-4,-1,-9), (-5,1,-9), (-5,4,9) (-5,6,8), (-5,-1,-8)								
**LiNbO3**	**(2,3,-25)**	**12.717**	**82.58**	**0.3**	**1.2**	**1.669**	**2**	**20.87**	**20.96**
	Equiv. Refl.: (2,-5,25), (3,2,25), (3,-5,-25), (5,-2,25), (5,-3,-25), (-2,5,-25), (-2,-3,25), (-3,5,25), (-3,-2,-25) (-5,2,-25), (-5,3,25), (0,3,24), (0,3,-24), (0,-3,24), (0,-3,-24), (1,3,-23), (1,-4,23), (3,0,24) (3,0,-24), (3,1,23), (3,6,9), (3,6,-9), (3,-3,24), (3,-3,-24), (3,-4,-23), (3,-9,9), (3,-9,-9) (4,-1,23), (4,-3,-23), (6,3,9), (6,3,-9), (6,-9,9), (6,-9,-9), (9,-3,9), (9,-3,-9), (9,-6,9) (9,-6,-9), (-1,4,-23), (-1,-3,23), (-3,0,24), (-3,0,-24), (-3,3,24), (-3,3,-24), (-3,4,23), (-3,9,9) (-3,9,-9), (-3,-1,-23), (-3,-6,9), (-3,-6,-9), (-4,1,-23), (-4,3,23), (-6,9,9), (-6,9,-9), (-6,-3,9) (-6,-3,-9), (-9,3,9), (-9,3,-9), (-9,6,9), (-9,6,-9), (0,5,9), (0,-5,-9), (1,5,-8), (1,-6,8) (2,7,-3), (2,-9,3), (5,0,-9), (5,1,8), (5,-5,9), (5,-6,-8), (7,2,3), (7,-9,-3), (9,-1,-1) (9,-2,3), (9,-7,-3), (9,-8,1), (-1,9,1), (-1,-8,-1), (-2,9,-3), (-2,-7,3), (-5,0,9), (-5,5,-9) (-6,1,-8), (-6,5,8), (-7,9,3), (-7,-2,-3), (-8,9,-1), (-8,-1,1), (-9,2,-3), (-9,7,3)								
**LiNbO3**	**(1,7,15)**	**12.458**	**76.28**	**0.2**	**0.7**	**3.132**	**2.18**	**39.15**	**39.21**
	Equiv. Refl.: (1,7,-15), (1,-8,15), (1,-8,-15), (7,1,15), (7,1,-15), (7,-8,15), (7,-8,-15), (8,-1,15), (8,-1,-15) (8,-7,15), (8,-7,-15), (-1,8,15), (-1,8,-15), (-1,-7,15), (-1,-7,-15), (-7,8,15), (-7,8,-15), (-7,-1,15) (-7,-1,-15), (-8,1,15), (-8,1,-15), (-8,7,15), (-8,7,-15)								
**LiNbO3**	**(1,8,5)**	**12.089**	**70.51**	**0.1**	**0.4**	**4.54**	**1.91**	**56.75**	**56.78**
	Equiv. Refl.: (1,8,-7), (1,-9,7), (1,-9,-5), (3,5,-17), (3,-8,17), (5,3,17), (5,-8,-17), (8,1,7), (8,1,-5) (8,-3,17), (8,-5,-17), (8,-9,5), (8,-9,-7), (9,-1,7), (9,-1,-5), (9,-8,5), (9,-8,-7), (-1,9,5) (-1,9,-7), (-1,-8,7), (-1,-8,-5), (-3,8,-17), (-3,-5,17), (-5,8,17), (-5,-3,-17), (-8,3,-17), (-8,5,17) (-8,9,7), (-8,9,-5), (-8,-1,5), (-8,-1,-7), (-9,1,5), (-9,1,-7), (-9,8,7), (-9,8,-5), (1,7,12) (1,7,-12), (1,-8,12), (1,-8,-12), (3,4,17), (3,-7,-17), (4,3,-17), (4,-7,17), (7,1,12), (7,1,-12) (7,-3,-17), (7,-4,17), (7,-8,12), (7,-8,-12), (8,-1,12), (8,-1,-12), (8,-7,12), (8,-7,-12), (-1,8,12) (-1,8,-12), (-1,-7,12), (-1,-7,-12), (-3,7,17), (-3,-4,-17), (-4,7,-17), (-4,-3,17), (-7,3,17), (-7,4,-17) (-7,8,12), (-7,8,-12), (-7,-1,12), (-7,-1,-12), (-8,1,12), (-8,1,-12), (-8,7,12), (-8,7,-12)								
**LiNbO3**	**(5,5,3)**	**12.116**	**70.88**	**0**	**0.1**	**4.446**	**0.55**	**55.58**	**55.58**
	Equiv. Refl.: (5,5,9), (5,5,-3), (5,5,-9), (5,-10,3), (5,-10,9), (5,-10,-3), (5,-10,-9), (10,-5,3), (10,-5,9) (10,-5,-3), (10,-5,-9), (-5,10,3), (-5,10,9), (-5,10,-3), (-5,10,-9), (-5,-5,3), (-5,-5,9), (-5,-5,-3) (-5,-5,-9), (-10,5,3), (-10,5,9), (-10,5,-3), (-10,5,-9)								

Ei =	11.215	keV						ΔEg	ΔEt
Cryst	Refl	EB	ΘB	∫ IR dΘ	Width	Ei cotΘ	ΔE	2m, 50μm	2m, 50μm
	(h,k,l)	[keV]	[°]	[μrad]	[μrad]	[meV/μrad]	[meV]	[meV]	[meV]
**Si**	**(4,4,8)**	**11.184**	**85.73**	**19.513**	**17.4**	**0.838**	**14.57**	**10.47**	**17.95**
**Si**	**(4,6,6)**	**10.708**	**72.7**	**5.858**	**5**	**3.493**	**17.43**	**43.66**	**47.01**
**Si**	**(1,3,9)**	**10.889**	**76.14**	**4.672**	**4.2**	**2.766**	**11.51**	**34.58**	**36.44**
**Ge**	**(0,2,10)**	**11.175**	**85.15**	**4.134**	**9.9**	**0.952**	**9.43**	**11.9**	**15.19**
	Equiv. Refl.: (2,6,8)								
**Ge**	**(4,4,8)**	**10.736**	**73.2**	**1.589**	**3.6**	**3.386**	**12.18**	**42.32**	**44.04**
**Ge**	**(1,7,7)**	**10.903**	**76.45**	**0.83**	**2.9**	**2.703**	**7.82**	**33.78**	**34.68**
	Equiv. Refl.: (3,3,9), (5,5,7)								
**Sapph**	**(0,7,8)**	**11.202**	**87.22**	**33.1**	**28.6**	**0.545**	**15.58**	**6.81**	**17**
	Equiv. Refl.: (0,-7,-8), (7,0,-8), (7,-7,8), (-7,0,8), (-7,7,-8)								
**Quartz**	**(3,0,9)**	**11.21**	**88.36**	**23.6**	**21.9**	**0.321**	**7.04**	**4.02**	**8.1**
	Equiv. Refl.: (3,-3,-9), (-3,0,-9), (-3,3,9)								
**Quartz**	**(0,3,-9)**	**11.21**	**88.36**	**23.5**	**21.8**	**0.321**	**7.02**	**4.02**	**8.09**
	Equiv. Refl.: (0,-3,9)								
**Quartz**	**(0,7,4)**	**11.183**	**85.7**	**20.1**	**17**	**0.844**	**14.31**	**10.55**	**17.78**
	Equiv. Refl.: (0,-7,-4), (7,0,-4), (7,-7,4), (-7,0,4), (-7,7,-4)								
**LiNbO3**	**(1,1,24)**	**10.999**	**78.74**	**19.3**	**17.5**	**2.233**	**39.14**	**27.91**	**48.07**
	Equiv. Refl.: (1,6,-14), (1,-2,24), (2,-1,24), (6,-7,-14), (7,-6,-14), (-1,2,24), (-1,7,-14), (-1,-1,24), (-2,1,24) (-6,-1,-14), (-7,1,-14)								
**LiNbO3**	**(1,1,-24)**	**10.999**	**78.74**	**19.2**	**17.5**	**2.233**	**39.14**	**27.91**	**48.07**
	Equiv. Refl.: (1,-2,-24), (2,-1,-24), (-1,2,-24), (-1,-1,-24), (-2,1,-24)								
**LiNbO3**	**(3,-7,16)**	**11.078**	**81.05**	**18.6**	**17.2**	**1.767**	**30.33**	**22.09**	**37.52**
	Equiv. Refl.: (4,3,16), (7,-3,16), (-3,-4,16), (-4,7,16), (-7,4,16)								
**LiNbO3**	**(1,-7,14)**	**11.06**	**80.48**	**18.1**	**17.5**	**1.882**	**32.91**	**23.52**	**40.45**
	Equiv. Refl.: (6,1,14), (7,-1,14), (-1,-6,14), (-6,7,14), (-7,6,14)								
**LiNbO3**	**(0,-8,2)**	**11.16**	**84.31**	**17.6**	**17.6**	**1.117**	**19.66**	**13.96**	**24.11**
	Equiv. Refl.: (3,4,-16), (4,-7,-16), (7,-4,-16), (8,0,2), (-3,7,-16), (-4,-3,-16), (-7,3,-16), (-8,8,2)								
**LiNbO3**	**(3,6,0)**	**11.037**	**79.77**	**17.5**	**16.2**	**2.024**	**32.77**	**25.3**	**41.41**
	Equiv. Refl.: (6,-9,0), (9,-6,0), (-3,9,0), (-6,-3,0), (-9,3,0)								
**LiNbO3**	**(3,-9,0)**	**11.037**	**79.77**	**17.1**	**16.2**	**2.024**	**32.77**	**25.3**	**41.41**
	Equiv. Refl.: (6,3,0), (9,-3,0), (-3,-6,0), (-6,9,0), (-9,6,0)								
**LiNbO3**	**(0,8,-2)**	**11.16**	**84.31**	**16.7**	**17.6**	**1.117**	**19.66**	**13.96**	**24.11**
	Equiv. Refl.: (8,-8,-2), (-8,0,-2)								
**LiNbO3**	**(4,4,12)**	**11.027**	**79.5**	**16.6**	**15.2**	**2.078**	**31.67**	**25.98**	**40.96**
	Equiv. Refl.: (4,-8,12), (8,-4,12), (-4,8,12), (-4,-4,12), (-8,4,12)								
**LiNbO3**	**(4,-9,4)**	**11.006**	**78.93**	**16**	**14.6**	**2.194**	**31.96**	**27.42**	**42.11**
	Equiv. Refl.: (5,4,4), (9,-4,4), (-4,-5,4), (-5,9,4), (-9,5,4)								
**LiNbO3**	**(1,-4,-22)**	**11.042**	**79.91**	**15.9**	**14.9**	**1.995**	**29.76**	**24.94**	**38.82**
	Equiv. Refl.: (3,1,-22), (4,-1,-22), (-1,-3,-22), (-3,4,-22), (-4,3,-22)								
**LiNbO3**	**(4,4,-12)**	**11.027**	**79.5**	**15.7**	**15.2**	**2.078**	**31.67**	**25.98**	**40.96**
	Equiv. Refl.: (4,-8,-12), (8,-4,-12), (-4,8,-12), (-4,-4,-12), (-8,4,-12)								
**LiNbO3**	**(1,3,22)**	**11.042**	**79.91**	**15.4**	**14.9**	**1.995**	**29.76**	**24.94**	**38.82**
	Equiv. Refl.: (3,-4,22), (4,5,2), (4,-3,22), (5,-9,2), (9,-5,2), (-1,4,22), (-3,-1,22), (-4,1,22), (-4,9,2) (-5,-4,2), (-9,4,2)								
**LiNbO3**	**(4,-9,-2)**	**10.897**	**76.32**	**15.1**	**14**	**2.73**	**38.19**	**34.12**	**51.22**
	Equiv. Refl.: (5,4,-2), (9,-4,-2), (-4,-5,-2), (-5,9,-2), (-9,5,-2)								
**LiNbO3**	**(4,5,-4)**	**11.006**	**78.93**	**14.9**	**14.6**	**2.194**	**31.96**	**27.42**	**42.11**
	Equiv. Refl.: (5,-9,-4), (9,-5,-4), (-4,9,-4), (-5,-4,-4), (-9,4,-4)								

Ei =	11.215	keV						ΔEg	ΔEt
Cryst	Refl	EB	ΘB	∫ IR dΘ	Width	Ei cotΘ	ΔE	2m, 50µm	2m, 50µm
	(h,k,l)	[keV]	[°]	[µrad]	[µrad]	[meV/µrad]	[meV]	[meV]	[meV]
**LiNbO3**	**(2,-8,10)**	**10.979**	**78.22**	**14.7**	**13.6**	**2.338**	**31.86**	**29.23**	**43.24**
	Equiv. Refl.: (6,2,10), (8,-2,10), (-2,-6,10), (-6,8,10), (-8,6,10)								
**LiNbO3**	**(2,6,-10)**	**10.979**	**78.22**	**14.2**	**13.6**	**2.338**	**31.86**	**29.23**	**43.24**
	Equiv. Refl.: (3,3,18), (3,-6,18), (6,-3,18), (6,-8,-10), (8,-6,-10), (-2,8,-10), (-3,6,18), (-3,-3,18), (-6,3,18) (-6,-2,-10), (-8,2,-10)								
**LiNbO3**	**(3,3,-18)**	**10.816**	**74.68**	**13.5**	**12.7**	**3.073**	**39.02**	**38.41**	**54.76**
	Equiv. Refl.: (3,-6,-18), (6,-3,-18), (-3,6,-18), (-3,-3,-18), (-6,3,-18)								
**LiNbO3**	**(1,-8,-6)**	**10.835**	**75.05**	**13.1**	**12.1**	**2.995**	**36.24**	**37.43**	**52.1**
	Equiv. Refl.: (7,1,-6), (8,-1,-6), (-1,-7,-6), (-7,8,-6), (-8,7,-6)								
**LiNbO3**	**(1,7,6)**	**10.835**	**75.05**	**13**	**12.1**	**2.995**	**36.24**	**37.43**	**52.1**
	Equiv. Refl.: (7,-8,6), (8,-7,6), (-1,8,6), (-7,-1,6), (-8,1,6)								
**LiNbO3**	**(0,7,-10)**	**10.711**	**72.77**	**12.8**	**11.7**	**3.479**	**40.58**	**43.49**	**59.48**
	Equiv. Refl.: (7,-7,-10), (-7,0,-10)								
**LiNbO3**	**(0,-7,10)**	**10.711**	**72.77**	**12.7**	**11.7**	**3.479**	**40.58**	**43.49**	**59.48**
	Equiv. Refl.: (7,0,10), (-7,7,10)								
**LiNbO3**	**(1,7,-6)**	**10.835**	**75.05**	**12.4**	**11.4**	**2.995**	**34.19**	**37.43**	**50.69**
	Equiv. Refl.: (7,-8,-6), (8,-7,-6), (-1,8,-6), (-7,-1,-6), (-8,1,-6)								
**LiNbO3**	**(1,-8,6)**	**10.835**	**75.05**	**12**	**11.4**	**2.995**	**34.19**	**37.43**	**50.69**
	Equiv. Refl.: (7,1,6), (8,-1,6), (-1,-7,6), (-7,8,6), (-8,7,6)								
**Quartz**	**(0,6,-6)**	**11.126**	**82.77**	**11.5**	**9.5**	**1.423**	**13.5**	**17.78**	**22.33**
	Equiv. Refl.: (0,-6,6), (6,0,6), (6,-6,-6), (-6,0,-6), (-6,6,6)								
**LiNbO3**	**(2,-8,-8)**	**10.646**	**71.67**	**11.4**	**10.4**	**3.715**	**38.78**	**46.44**	**60.5**
	Equiv. Refl.: (6,2,-8), (8,-2,-8), (-2,-6,-8), (-6,8,-8), (-8,6,-8)								
**LiNbO3**	**(2,6,8)**	**10.646**	**71.67**	**11.2**	**10.4**	**3.715**	**38.78**	**46.44**	**60.5**
	Equiv. Refl.: (6,-8,8), (8,-6,8), (-2,8,8), (-6,-2,8), (-8,2,8)								
**LiNbO3**	**(3,5,10)**	**10.711**	**72.77**	**11**	**9.7**	**3.479**	**33.61**	**43.49**	**54.96**
	Equiv. Refl.: (5,-8,10), (8,-5,10), (-3,8,10), (-5,-3,10), (-8,3,10)								
**LiNbO3**	**(1,5,-16)**	**10.542**	**70.05**	**10.5**	**9.4**	**4.072**	**38.46**	**50.9**	**63.79**
	Equiv. Refl.: (5,-6,-16), (6,-5,-16), (-1,6,-16), (-5,-1,-16), (-6,1,-16), (2,6,-4), (2,-8,4), (6,2,4), (6,-8,-4) (8,-2,4), (8,-6,-4), (-2,8,-4), (-2,-6,4), (-6,8,4), (-6,-2,-4), (-8,2,-4), (-8,6,4)								
**LiNbO3**	**(1,-6,16)**	**10.542**	**70.05**	**10.4**	**9.4**	**4.072**	**38.46**	**50.9**	**63.79**
	Equiv. Refl.: (2,-5,-20), (3,2,-20), (5,1,16), (5,-2,-20), (6,-1,16), (-1,-5,16), (-2,-3,-20), (-3,5,-20), (-5,3,-20) (-5,6,16), (-6,5,16)								
**LiNbO3**	**(3,-8,-10)**	**10.711**	**72.77**	**9.9**	**9.7**	**3.479**	**33.61**	**43.49**	**54.96**
	Equiv. Refl.: (5,3,-10), (8,-3,-10), (-3,-5,-10), (-5,8,-10), (-8,5,-10)								
**Sapph**	**(1,6,10)**	**10.959**	**77.74**	**9.8**	**7.9**	**2.438**	**19.37**	**30.47**	**36.11**
	Equiv. Refl.: (1,-7,-10), (6,1,-10), (6,-7,10), (7,-1,-10), (7,-6,10), (-1,7,10), (-1,-6,-10), (-6,7,-10), (-6,-1,10) (-7,1,10), (-7,6,-10)								
**LiNbO3**	**(2,3,20)**	**10.804**	**74.44**	**9.6**	**9.4**	**3.123**	**29.49**	**39.04**	**48.93**
	Equiv. Refl.: (3,-5,20), (5,-3,20), (-2,5,20), (-3,-2,20), (-5,2,20)								
**Sapph**	**(1,3,-20)**	**10.979**	**78.23**	**9.5**	**7.8**	**2.336**	**18.27**	**29.2**	**34.45**
	Equiv. Refl.: (1,-4,20), (3,1,20), (3,-4,-20), (4,-1,20), (4,-3,-20), (-1,4,-20), (-1,-3,20), (-3,4,20), (-3,-1,-20) (-4,1,-20), (-4,3,20)								
**Quartz**	**(3,5,-4)**	**11.183**	**85.7**	**9.4**	**8.6**	**0.844**	**7.24**	**10.55**	**12.79**
	Equiv. Refl.: (3,-8,4), (5,3,4), (5,-8,-4), (-8,3,-4), (-8,5,4)								
**Quartz**	**(2,4,-7)**	**11.131**	**83**	**9.2**	**7.7**	**1.378**	**10.67**	**17.23**	**20.26**
	Equiv. Refl.: (2,-6,7), (4,-6,-7), (8,-3,4), (8,-5,-4), (-3,8,-4), (-3,-5,4), (-5,8,4), (-5,-3,-4), (-6,2,-7) (-6,4,7)								
**Quartz**	**(4,2,7)**	**11.131**	**83**	**9.1**	**7.7**	**1.378**	**10.66**	**17.23**	**20.26**

Ei = Cryst	11.215 Refl (h,k,l)	keV EB [keV]	ΘB [°]	∫ IR dΘ [µrad]	Width [µrad]	Ei cotΘ [meV/µrad]	ΔE [meV]	ΔEg 2m, 50µm [meV]	ΔEt 2m, 50µm [meV]
**Quartz**	**(5,-2,8)**	**11.16**	**84.3**	**9**	**7.6**	**1.12**	**8.53**	**14**	**16.39**
	Equiv. Refl.: (5,-3,-8), (-2,5,-8), (-2,-3,8), (-3,5,8), (-3,-2,-8)								
**Quartz**	**(6,-2,7)**	**11.131**	**83**	**8.9**	**7.7**	**1.378**	**10.67**	**17.23**	**20.26**
	Equiv. Refl.: (6,-4,-7), (-2,6,-7), (-2,-4,7), (-4,6,7), (-4,-2,-7)								
**Quartz**	**(2,3,-8)**	**11.16**	**84.3**	**8.5**	**7.6**	**1.12**	**8.53**	**14**	**16.39**
	Equiv. Refl.: (2,-5,8), (3,2,8), (3,-5,-8), (-5,2,-8), (-5,3,8)								
**Quartz**	**(1,6,5)**	**11.143**	**83.52**	**8.2**	**6.9**	**1.275**	**8.75**	**15.93**	**18.18**
	Equiv. Refl.: (1,-7,-5), (6,1,-5), (6,-7,5), (-7,1,5), (-7,6,-5)								
**Quartz**	**(0,5,7)**	**10.841**	**75.17**	**8.1**	**6.3**	**2.969**	**18.74**	**37.12**	**41.58**
	Equiv. Refl.: (0,-5,-7), (5,0,-7), (5,-5,7), (-5,0,7), (-5,5,-7)								
**Quartz**	**(0,4,8)**	**10.87**	**75.76**	**7.6**	**6.1**	**2.846**	**17.42**	**35.57**	**39.61**
	Equiv. Refl.: (0,-4,-8), (4,0,-8), (4,-4,8), (-4,0,8), (-4,4,-8)								
**Quartz**	**(3,5,-3)**	**10.764**	**73.69**	**7.5**	**5.9**	**3.281**	**19.39**	**41.02**	**45.37**
	Equiv. Refl.: (3,-8,3), (5,3,3), (5,-8,-3), (8,-3,3), (8,-5,-3), (-3,8,-3), (-3,-5,3), (-5,8,3), (-5,-3,-3) (-8,3,-3), (-8,5,3)								
**LiNbO3**	**(0,0,-24)**	**10.732**	**73.13**	**7.3**	**6.8**	**3.401**	**23.2**	**42.52**	**48.44**
	Equiv. Refl.: (7,-1,-5), (7,-6,5), (-1,7,5), (-1,-6,-5), (-6,7,-5), (-6,-1,5)								
**Sapph**	**(1,5,14)**	**10.715**	**72.83**	**6.9**	**5.7**	**3.466**	**19.65**	**43.32**	**47.57**
	Equiv. Refl.: (1,-6,-14), (3,5,4), (3,-8,-4), (5,1,-14), (5,3,-4), (5,-6,14), (5,-8,4), (6,-1,-14), (6,-5,14) (8,-3,-4), (8,-5,4), (-1,6,14), (-1,-5,-14), (-3,8,4), (-3,-5,-4), (-5,6,-14), (-5,8,-4), (-5,-1,14) (-5,-3,4), (-6,1,14), (-6,5,-14), (-8,3,4), (-8,5,-4)								
**LiNbO3**	**(0,0,24)**	**10.732**	**73.13**	**6.4**	**6.8**	**3.401**	**23.2**	**42.52**	**48.44**
	Equiv. Refl.: (8,-1,0), (8,-7,0), (-1,8,0), (-1,-7,0), (-7,8,0), (-7,-1,0)								
**Quartz**	**(4,4,4)**	**11.088**	**81.37**	**6.3**	**5.5**	**1.702**	**9.28**	**21.27**	**23.21**
	Equiv. Refl.: (4,4,-4), (4,-8,4), (4,-8,-4), (-8,4,4), (-8,4,-4)								
**Quartz**	**(1,7,0)**	**11**	**78.76**	**6.2**	**5.3**	**2.228**	**11.76**	**27.85**	**30.23**
	Equiv. Refl.: (1,-8,0), (2,6,-3), (2,-8,3), (6,2,3), (6,-8,-3), (7,1,0), (7,-8,0), (8,-2,3), (8,-4,4) (8,-4,-4), (8,-6,-3), (-2,8,-3), (-2,-6,3), (-4,8,4), (-4,8,-4), (-4,-4,4), (-4,-4,-4), (-6,8,3) (-6,-2,-3), (-8,1,0), (-8,2,-3), (-8,6,3), (-8,7,0)								
**Sapph**	**(4,4,6)**	**10.809**	**74.54**	**6**	**5.1**	**3.102**	**15.74**	**38.77**	**41.85**
	Equiv. Refl.: (4,4,-6), (4,-8,6), (4,-8,-6), (8,-4,6), (8,-4,-6), (-4,8,6), (-4,8,-6), (-4,-4,6), (-4,-4,-6) (-8,4,6), (-8,4,-6)								
**Sapph**	**(2,4,16)**	**11.031**	**79.62**	**5.9**	**5.1**	**2.055**	**10.58**	**25.69**	**27.78**
	Equiv. Refl.: (2,-6,-16), (4,2,-16), (4,-6,16), (6,-2,-16), (6,-4,16), (-2,6,16), (-2,-4,-16), (-4,6,-16), (-4,-2,16) (-6,2,16), (-6,4,-16)								
**Sapph**	**(0,5,16)**	**10.719**	**72.9**	**5.5**	**4.7**	**3.45**	**16.17**	**43.13**	**46.06**
	Equiv. Refl.: (0,-5,-16), (2,6,5), (2,-8,-5), (5,0,-16), (5,-5,16), (6,2,-5), (6,-8,5), (8,-2,-5), (8,-6,5) (-2,8,5), (-2,-6,-5), (-5,0,16), (-5,5,-16), (-6,8,-5), (-6,-2,5), (-8,2,5), (-8,6,-5)								
**Quartz**	**(8,-1,-1)**	**11.06**	**80.45**	**5.3**	**4.5**	**1.886**	**8.49**	**23.58**	**25.06**
	Equiv. Refl.: (8,-7,1), (-1,8,1), (-1,-7,-1), (-7,8,-1), (-7,-1,1)								
**Sapph**	**(0,7,2)**	**10.574**	**70.54**	**5.2**	**4.3**	**3.963**	**16.88**	**49.54**	**52.34**
	Equiv. Refl.: (0,-7,-2), (7,0,-2), (7,-7,2), (-7,0,2), (-7,7,-2), (1,7,1), (1,-8,-1), (7,1,-1), (7,-8,1) (-8,1,1), (-8,7,-1)								
**Quartz**	**(1,5,6)**	**10.638**	**71.54**	**5**	**3.9**	**3.743**	**14.61**	**46.79**	**49.02**
	Equiv. Refl.: (1,-6,-6), (5,1,-6), (5,-6,6), (6,-1,-6), (6,-5,6), (-1,6,6), (-1,-5,-6), (-5,6,-6), (-5,-1,6) (-6,1,6), (-6,5,-6)								
**Sapph**	**(2,3,-19)**	**11.191**	**86.26**	**4.9**	**5.5**	**0.733**	**4.04**	**9.17**	**10.02**
	Equiv. Refl.: (2,-5,19), (3,2,19), (3,-5,-19), (5,-2,19), (5,-3,-19), (-2,5,-19), (-2,-3,19), (-3,5,19), (-3,-2,-19) (-5,2,-19), (-5,3,19)								
**Quartz**	**(8,-2,-3)**	**11.056**	**80.33**	**4.6**	**3.9**	**1.911**	**7.43**	**23.89**	**25.02**
	Equiv. Refl.: (8,-6,3), (-2,8,3), (-2,-6,-3), (-6,8,-3), (-6,-2,3)								

Ei =	11.215	keV						ΔEg	ΔEt
Cryst	Refl	EB	ΘB	∫ IR dΘ	Width	Ei cotΘ	ΔE	2m, 50µm	2m, 50µm
	(h,k,l)	[keV]	[°]	[µrad]	[µrad]	[meV/µrad]	[meV]	[meV]	[meV]
**Quartz**	**(0,2,-9)**	**10.727**	**73.03**	**4.5**	**3.6**	**3.423**	**12.31**	**42.78**	**44.52**
	Equiv. Refl.: (0,-2,9), (2,0,9), (2,-2,-9), (8,-2,-1), (8,-6,1), (-2,0,-9), (-2,2,9), (-2,8,1), (-2,-6,-1)<br>(-6,8,-1), (-6,-2,1)								
**Quartz**	**(2,6,1)**	**10.569**	**70.46**	**4.4**	**3.5**	**3.981**	**14.11**	**49.76**	**51.72**
	Equiv. Refl.: (2,-8,-1), (6,2,-1), (6,-8,1), (8,-2,-2), (8,-6,2), (-2,8,2), (-2,-6,-2), (-6,8,-2), (-6,-2,2)<br>(-8,2,1), (-8,6,-1)								
**Sapph**	**(0,6,12)**	**10.69**	**72.4**	**4.3**	**3.6**	**3.557**	**12.92**	**44.46**	**46.3**
	Equiv. Refl.: (0,6,-12), (0,-6,12), (0,-6,-12), (6,0,12), (6,0,-12), (6,-6,12), (6,-6,-12), (-6,0,12), (-6,0,-12)<br>(-6,6,12), (-6,6,-12), (2,6,2), (2,-8,-2), (6,2,-2), (6,-8,2), (-8,2,2), (-8,6,-2)								
**Quartz**	**(0,3,9)**	**11.21**	**88.36**	**4.2**	**5.9**	**0.321**	**1.91**	**4.02**	**4.45**
	Equiv. Refl.: (0,-3,-9), (2,6,3), (2,-8,-3), (3,0,-9), (3,-3,9), (6,2,-3), (6,-8,3), (8,-1,1), (8,-7,-1)<br>(-1,8,-1), (-1,-7,1), (-3,0,9), (-3,3,-9), (-7,8,1), (-7,-1,-1), (-8,2,3), (-8,6,-3)								
**Quartz**	**(1,7,-1)**	**11.06**	**80.45**	**3.9**	**3.6**	**1.886**	**6.83**	**23.58**	**24.55**
	Equiv. Refl.: (1,-8,1), (7,1,1), (7,-8,-1), (-8,1,-1), (-8,7,1)								
**Quartz**	**(1,6,-4)**	**10.599**	**70.92**	**3.6**	**2.9**	**3.88**	**11.14**	**48.5**	**49.76**
	Equiv. Refl.: (1,-7,4), (4,4,3), (4,4,-3), (4,-8,3), (4,-8,-3), (6,1,4), (6,-7,-4), (-7,1,-4), (-7,6,4)<br>(-8,4,3), (-8,4,-3)								
**Sapph**	**(1,6,-8)**	**10.578**	**70.6**	**3.4**	**2.9**	**3.949**	**11.62**	**49.36**	**50.71**
	Equiv. Refl.: (1,-7,8), (2,6,-1), (2,-8,1), (6,1,8), (6,2,1), (6,-7,-8), (6,-8,-1), (7,-1,8), (7,-6,-8)<br>(8,-2,1), (8,-6,-1), (-1,7,-8), (-1,-6,8), (-2,8,-1), (-2,-6,1), (-6,7,8), (-6,8,1), (-6,-1,-8)<br>(-6,-2,-1), (-7,1,-8), (-7,6,8), (-8,2,-1), (-8,6,1), (7,-1,4), (7,-6,-4), (8,-4,3), (8,-4,-3)<br>(-1,7,-4), (-1,-6,4), (-4,8,3), (-4,8,-3), (-4,-4,3), (-4,-4,-3), (-6,7,4), (-6,-1,-4)								
**Quartz**	**(7,-1,5)**	**11.143**	**83.52**	**3.3**	**3.2**	**1.275**	**4.07**	**15.93**	**16.45**
	Equiv. Refl.: (7,-6,-5), (-1,7,-5), (-1,-6,5), (-6,7,5), (-6,-1,-5)								
**Quartz**	**(1,6,-5)**	**11.143**	**83.52**	**3.2**	**3.2**	**1.275**	**4.08**	**15.93**	**16.45**
	Equiv. Refl.: (1,-7,5), (4,-1,8), (4,-3,-8), (6,1,5), (6,-7,-5), (-1,4,-8), (-1,-3,8), (-3,4,8), (-3,-1,-8)<br>(-7,1,-5), (-7,6,5)								
**Quartz**	**(0,7,-4)**	**11.183**	**85.7**	**3.1**	**3.6**	**0.844**	**3.08**	**10.55**	**10.99**
	Equiv. Refl.: (0,-7,4), (1,3,-8), (1,-4,8), (3,1,8), (3,-4,-8), (7,0,4), (7,-7,-4), (-4,1,-8), (-4,3,8)<br>(-7,0,-4), (-7,7,4)								
**Quartz**	**(7,-3,-5)**	**10.556**	**70.27**	**3**	**2.7**	**4.023**	**10.9**	**50.29**	**51.46**
	Equiv. Refl.: (7,-4,5), (-3,7,5), (-3,-4,-5), (-4,7,-5), (-4,-3,5)								
**Quartz**	**(3,4,5)**	**10.556**	**70.27**	**2.9**	**2.7**	**4.023**	**10.9**	**50.29**	**51.46**
	Equiv. Refl.: (3,-7,-5), (4,3,-5), (4,-7,5), (8,-3,-4), (8,-5,4), (-3,8,4), (-3,-5,-4), (-5,8,-4), (-5,-3,4)<br>(-7,3,5), (-7,4,-5)								
**Quartz**	**(3,5,3)**	**10.764**	**73.69**	**2.8**	**2.5**	**3.281**	**8.21**	**41.02**	**41.83**
	Equiv. Refl.: (3,-8,-3), (5,3,-3), (5,-8,3), (-8,3,3), (-8,5,-3)								
**Quartz**	**(6,-2,-7)**	**11.131**	**83**	**2.7**	**2.8**	**1.378**	**3.89**	**17.23**	**17.66**
	Equiv. Refl.: (6,-4,7), (-2,6,7), (-2,-4,-7), (-4,6,-7), (-4,-2,7)								
**Quartz**	**(8,-3,-3)**	**10.764**	**73.69**	**2.6**	**2.5**	**3.281**	**8.21**	**41.02**	**41.83**
	Equiv. Refl.: (8,-5,3), (-3,8,3), (-3,-5,-3), (-5,8,-3), (-5,3,3)								
**Quartz**	**(2,4,7)**	**11.131**	**83**	**2.5**	**2.8**	**1.378**	**3.89**	**17.23**	**17.66**
	Equiv. Refl.: (2,-6,-7), (4,2,-7), (4,-6,7), (8,-2,1), (8,-6,-1), (-2,8,-1), (-2,-6,1), (-6,2,7), (-6,4,-7)<br>(-6,8,1), (-6,-2,-1)								
**Quartz**	**(2,6,-1)**	**10.569**	**70.46**	**2.4**	**2.3**	**3.981**	**9.08**	**49.76**	**50.58**
	Equiv. Refl.: (2,-8,1), (3,5,4), (3,-8,-4), (5,3,-4), (5,-8,4), (6,2,1), (6,-8,-1), (-8,2,-1), (-8,3,4)<br>(-8,5,-4), (-8,6,1)								
**LiNbO3**	**(1,6,13)**	**10.814**	**74.62**	**2.3**	**3.5**	**3.084**	**10.79**	**38.55**	**40.03**
	Equiv. Refl.: (1,-7,-13), (6,1,-13), (6,-7,13), (7,-1,-13), (7,-6,13), (-1,7,13), (-1,-6,-13), (-6,7,-13), (-6,-1,13)<br>(-7,1,13), (-7,6,-13)								

Ei =	11.215	keV						ΔEg	ΔEt
Cryst	Refl	EB	ΘB	∫ IR dΘ	Width	Ei cotΘ	ΔE	2m, 50µm	2m, 50µm
	(h,k,l)	[keV]	[°]	[µrad]	[µrad]	[meV/µrad]	[meV]	[meV]	[meV]
**Sapph**	**(1,6,-11)**	**11.175**	**85.17**	**2.2**	**3.2**	**0.947**	**3.02**	**11.84**	**12.22**
	Equiv. Refl.: (1,-7,11), (6,1,11), (6,-7,-11), (7,-1,11), (7,-6,-11), (-1,7,-11), (-1,-6,11), (-6,7,11), (-6,-1,-11)<br>(-7,1,-11), (-7,6,11)								
**Quartz**	**(1,1,9)**	**10.627**	**71.37**	**1.9**	**2**	**3.782**	**7.48**	**47.27**	**47.86**
	Equiv. Refl.: (1,1,-9), (1,-2,9), (1,-2,-9), (6,-1,6), (6,-5,-6), (-1,6,-6), (-1,-5,6), (-2,1,9), (-2,1,-9)<br>(-5,6,6), (-5,-1,-6)								
**Sapph**	**(3,5,7)**	**11.048**	**80.11**	**1.8**	**2.3**	**1.956**	**4.46**	**24.45**	**24.85**
	Equiv. Refl.: (3,-8,-7), (5,3,-7), (5,-8,7), (8,-3,-7), (8,-5,7), (-3,8,7), (-3,-5,-7), (-5,8,-7), (-5,-3,7)<br>(-8,3,7), (-8,5,-7), (1,5,-6), (1,-6,6), (2,5,-5), (2,-7,5), (5,1,6), (5,2,5), (5,-6,-6)<br>(5,-7,-5), (-6,1,-6), (-6,5,6), (-7,2,-5), (-7,5,5)								
**Quartz**	**(1,2,-9)**	**11.019**	**79.28**	**1.7**	**1.9**	**2.122**	**4.03**	**26.53**	**26.83**
	Equiv. Refl.: (1,-3,9), (2,1,9), (2,-1,9), (2,-1,-9), (2,-3,-9), (7,-2,5), (7,-5,-5), (-1,2,9), (-1,2,-9)<br>(-1,-1,9), (-1,-1,-9), (-2,7,-5), (-2,-5,5), (-3,1,-9), (-3,2,9), (-5,7,5), (-5,-2,-5)								
**Quartz**	**(0,2,9)**	**10.727**	**73.03**	**1.6**	**1.8**	**3.423**	**6.11**	**42.78**	**43.22**
	Equiv. Refl.: (0,-2,-9), (2,0,-9), (2,-2,9), (-2,0,9), (-2,2,-9)								
**LiNbO3**	**(2,4,-17)**	**10.58**	**70.62**	**1.5**	**2.5**	**3.945**	**9.9**	**49.31**	**50.3**
	Equiv. Refl.: (2,-6,17), (4,2,17), (4,5,5), (4,-6,-17), (4,-9,-5), (5,4,-5), (5,-9,5), (6,-2,17), (6,-4,-17)<br>(9,-4,-5), (9,-5,5), (-2,6,-17), (-2,-4,17), (-4,6,17), (-4,9,5), (-4,-2,-17), (-4,-5,-5), (-5,9,-5)<br>(-5,-4,5), (-6,2,-17), (-6,4,17), (-9,4,5), (-9,5,-5), (2,6,-2), (2,-8,2), (6,2,2), (6,-8,-2)<br>(-8,2,-2), (-8,6,2)								
**LiNbO3**	**(2,2,21)**	**10.554**	**70.23**	**1.4**	**2.4**	**4.031**	**9.53**	**50.39**	**51.28**
	Equiv. Refl.: (2,2,-21), (2,-4,21), (2,-4,-21), (4,-2,21), (4,-2,-21), (-2,4,21), (-2,4,-21), (-2,-2,21), (-2,-2,-21)<br>(-4,2,21), (-4,2,-21), (3,-1,9), (3,-2,-9), (-1,3,-9), (-1,-2,9), (-2,3,9), (-2,-1,-9)								
**Sapph**	**(3,5,-8)**	**11.202**	**87.22**	**1.3**	**2.7**	**0.545**	**1.49**	**6.81**	**6.97**
	Equiv. Refl.: (3,-8,8), (5,3,8), (5,-8,-8), (8,-3,8), (8,-5,-8), (-3,8,-8), (-3,-5,8), (-5,8,8), (-5,-3,-8)<br>(-8,3,-8), (-8,5,8), (0,6,6), (0,-6,-6), (1,3,8), (1,-4,-8), (3,1,-8), (3,-4,8), (4,-1,-8)<br>(4,-3,8), (6,0,-6), (6,-6,6), (8,-2,2), (8,-6,-2), (-1,4,8), (-1,-3,-8), (-2,8,-2), (-2,-6,2)<br>(-3,4,-8), (-3,-1,8), (-4,1,8), (-4,3,-8), (-6,0,6), (-6,6,-6), (-6,8,2), (-6,-2,-2)								
**Sapph**	**(3,5,-5)**	**10.798**	**74.33**	**1.2**	**1.5**	**3.147**	**4.84**	**39.34**	**39.63**
	Equiv. Refl.: (3,-8,5), (5,3,5), (5,-8,-5), (8,-3,5), (8,-5,-5), (-3,8,-5), (-3,-5,5), (-5,8,5), (-5,-3,-5)<br>(-8,3,-5), (-8,5,5)								
**Quartz**	**(0,7,3)**	**10.764**	**73.69**	**1.1**	**1.4**	**3.281**	**4.44**	**41.02**	**41.26**
	Equiv. Refl.: (0,-7,-3), (2,3,8), (2,-5,-8), (3,2,-8), (3,4,-5), (3,-5,8), (3,-7,5), (4,3,5), (4,-7,-5)<br>(7,0,-3), (7,-7,3), (-5,2,8), (-5,3,-8), (-7,0,3), (-7,3,-5), (-7,4,5), (-7,7,-3)								
**LiNbO3**	**(2,6,11)**	**11.169**	**84.78**	**1**	**3.3**	**1.024**	**3.36**	**12.8**	**13.24**
	Equiv. Refl.: (2,-8,-11), (4,5,-1), (4,-9,1), (5,4,1), (5,-9,-1), (6,2,-11), (6,-8,11), (8,-2,-11), (8,-6,11)<br>(9,-4,1), (9,-5,-1), (-2,8,11), (-2,-6,-11), (-4,9,-1), (-4,-5,1), (-5,9,1), (-5,-4,-1), (-6,8,-11)<br>(-6,-2,11), (-8,2,11), (-8,6,-11), (-9,4,-1), (-9,5,1), (3,5,1), (3,-8,-1), (5,3,-1), (5,-8,1)<br>(8,-3,-1), (8,-5,1), (-3,8,1), (-3,-5,-1), (-5,8,-1), (-5,-3,1), (-8,3,1), (-8,5,-1), (0,7,-3)<br>(0,-7,3), (5,-2,-8), (5,-3,8), (7,0,3), (7,-3,5), (7,-4,-5), (7,-7,-3), (-2,5,8), (-2,-3,-8)<br>(-3,5,-8), (-3,7,-5), (-3,-2,8), (-3,-4,5), (-4,7,5), (-4,-3,-5), (-7,0,-3), (-7,7,3)								
**LiNbO3**	**(3,6,3)**	**11.118**	**82.45**	**0.9**	**2.6**	**1.486**	**3.87**	**18.57**	**18.97**
	Equiv. Refl.: (3,6,-3), (3,-9,3), (3,-9,-3), (6,3,3), (6,3,-3), (6,-9,3), (6,-9,-3), (9,-3,3), (9,-3,-3)<br>(9,-6,3), (9,-6,-3), (-3,9,3), (-3,9,-3), (-3,-6,3), (-3,-6,-3), (-6,9,3), (-6,9,-3), (-6,-3,3)<br>(-6,-3,-3), (-9,3,3), (-9,3,-3), (-9,6,3), (-9,6,-3), (1,4,18), (1,4,-18), (1,-5,18), (1,-5,-18)<br>(4,1,18), (4,1,-18), (4,-5,18), (4,-5,-18), (5,-1,18), (5,-1,-18), (5,-4,18), (5,-4,-18), (-1,5,18)<br>(-1,5,-18), (-1,-4,18), (-1,-4,-18), (-4,5,18), (-4,5,-18), (-4,-1,18), (-4,-1,-18), (-5,1,18), (-5,1,-18)<br>(-5,4,18), (-5,4,-18), (1,2,9), (1,6,4), (1,-3,-9), (1,-7,-4), (2,1,-9), (2,-3,9), (3,-1,-9)<br>(3,-2,9), (6,1,-4), (6,-7,4), (7,-1,-4), (7,-6,4), (-1,3,9), (-1,7,4), (-1,-2,-9), (-1,-6,-4)<br>(-2,3,-9), (-2,-1,9), (-3,1,9), (-3,2,-9), (-6,7,-4), (-6,-1,4), (-7,1,4), (-7,6,-4)								

Ei =	11.215	keV						ΔEg	ΔEt
**Cryst**	**Refl**	**EB**	**ΘB**	**∫ IR dΘ**	**Width**	**Ei cotΘ**	**ΔE**	**2m, 50µm**	**2m, 50µm**
	**(h,k,l)**	**[keV]**	**[°]**	**[µrad]**	**[µrad]**	**[meV/µrad]**	**[meV]**	**[meV]**	**[meV]**
**LiNbO3**	**(1,2,23)**	**10.923**	**76.9**	**0.8**	**1.8**	**2.609**	**4.8**	**32.62**	**32.97**
	Equiv. Refl.: (1,5,17), (1,-3,-23), (1,-6,-17), (2,1,-23), (2,-3,23), (3,-1,-23), (3,-2,23), (5,1,-17), (5,-6,17)<br>(6,-1,-17), (6,-5,17), (-1,3,23), (-1,6,17), (-1,-2,-23), (-1,-5,-17), (-2,3,-23), (-2,-1,23), (-3,1,23)<br>(-3,2,-23), (-5,6,-17), (-5,-1,17), (-6,1,17), (-6,5,-17), (2,5,12), (2,5,-12), (2,-7,12), (2,-7,-12)<br>(5,2,12), (5,2,-12), (5,-7,12), (5,-7,-12), (7,-2,12), (7,-2,-12), (7,-5,12), (7,-5,-12), (-2,7,12)<br>(-2,7,-12), (-2,-5,12), (-2,-5,-12), (-5,7,12), (-5,7,-12), (-5,-2,12), (-5,-2,-12), (-7,2,12), (-7,2,-12)<br>(-7,5,12), (-7,5,-12)								
**Sapph**	**(0,7,-4)**	**10.703**	**72.61**	**0.6**	**0.9**	**3.512**	**3.26**	**43.9**	**44.02**
	Equiv. Refl.: (0,-7,4), (7,0,4), (7,-7,-4), (-7,0,-4), (-7,7,4), (0,4,-8), (0,5,-7), (0,-4,8), (0,-5,7)<br>(4,0,8), (4,-4,-8), (5,0,7), (5,-5,-7), (7,-2,-5), (7,-5,5), (-2,7,5), (-2,-5,-5), (-4,0,-8)<br>(-4,4,8), (-5,0,-7), (-5,5,7), (-5,7,-5), (-5,-2,5)								
**LiNbO3**	**(2,5,15)**	**10.973**	**78.07**	**0.5**	**1.5**	**2.37**	**3.66**	**29.63**	**29.86**
	Equiv. Refl.: (2,5,-15), (2,-7,15), (2,-7,-15), (5,2,15), (5,2,-15), (5,-7,15), (5,-7,-15), (7,-2,15), (7,-2,-15)<br>(7,-5,15), (7,-5,-15), (-2,7,15), (-2,7,-15), (-2,-5,15), (-2,-5,-15), (-5,7,15), (-5,7,-15), (-5,-2,15)<br>(-5,-2,-15), (-7,2,15), (-7,2,-15), (-7,5,15), (-7,5,-15), (0,2,22), (0,-2,-22), (1,3,19), (1,-4,-19)<br>(2,0,-22), (2,-2,22), (3,1,-19), (3,-4,19), (4,-1,-19), (4,-3,19), (-1,4,19), (-1,-3,-19), (-2,0,22)<br>(-2,2,-22), (-3,4,-19), (-3,-1,19), (-4,1,19), (-4,3,-19), (2,5,5), (2,-7,-5), (3,3,7), (3,3,-7)<br>(3,-6,7), (3,-6,-7), (5,2,-5), (5,-7,5), (-6,3,7), (-6,3,-7), (-7,2,5), (-7,5,-5)								
**Sapph**	**(3,4,-13)**	**11.056**	**80.35**	**0.3**	**0.7**	**1.907**	**1.28**	**23.84**	**23.88**
	Equiv. Refl.: (3,-7,13), (4,3,13), (4,-7,-13), (7,-3,13), (7,-4,-13), (-3,7,-13), (-3,-4,13), (-4,7,13), (-4,-3,-13)<br>(-7,3,-13), (-7,4,13), (6,-3,7), (6,-3,-7), (-3,6,7), (-3,6,-7), (-3,-3,7), (-3,-3,-7)								
**LiNbO3**	**(1,7,3)**	**10.583**	**70.68**	**0.2**	**0.8**	**3.932**	**3.22**	**49.15**	**49.26**
	Equiv. Refl.: (1,7,-3), (1,-8,3), (1,-8,-3), (3,5,-11), (3,-8,11), (5,3,11), (5,-8,-11), (7,1,3), (7,1,-3)<br>(7,-8,3), (7,-8,-3), (8,-1,3), (8,-1,-3), (8,-3,11), (8,-5,-11), (8,-7,3), (8,-7,-3), (-1,8,3)<br>(-1,8,-3), (-1,-7,3), (-1,-7,-3), (-3,8,-11), (-3,-5,11), (-5,8,11), (-5,-3,-11), (-7,8,3), (-7,8,-3)<br>(-7,-1,3), (-7,-1,-3), (-8,1,3), (-8,1,-3), (-8,3,-11), (-8,5,11), (-8,7,3), (-8,7,-3), (0,1,-22)<br>(0,-1,22), (1,0,22), (1,-1,-22), (3,4,11), (3,-7,-11), (4,3,-11), (4,-7,11), (7,-3,-11), (7,-4,11)<br>(-1,0,-22), (-1,1,22), (-3,7,11), (-3,-4,-11), (-4,7,-11), (-4,-3,11), (-7,3,11), (-7,4,-11)								
**Sapph**	**(2,6,2)**	**10.89**	**76.18**	**0.1**	**0.4**	**2.758**	**1.04**	**34.48**	**34.49**
	Equiv. Refl.: (2,-8,-2), (6,2,-2), (6,-8,2), (8,-2,-2), (8,-6,2), (-2,8,2), (-2,-6,-2), (-6,8,-2), (-6,-2,2)<br>(-8,2,2), (-8,6,-2)								
**Sapph**	**(3,3,15)**	**10.6**	**70.94**	**0**	**0.2**	**3.874**	**0.65**	**48.43**	**48.44**
	Equiv. Refl.: (3,3,-15), (3,-6,15), (3,-6,-15), (6,-3,15), (6,-3,-15), (-3,6,15), (-3,6,-15), (-3,-3,15), (-3,-3,-15)<br>(-6,3,15), (-6,3,-15)								

Ei =	13.88	keV						ΔEg	ΔEt
Cryst	Refl	EB	ΘB	∫IR dΘ	Width	Ei cotΘ	ΔE	2m, 50µm	2m, 50µm
	(h,k,l)	[keV]	[°]	[µrad]	[µrad]	[meV/µrad]	[meV]	[meV]	[meV]
**Ge**	**(0,4,12)**	**13.861**	**86.97**	**9.1178**	**20.49**	**0.7339**	**15.04**	**9.17**	**17.62**
	Equiv. Refl.: (0,12,4), (4,0,12), (4,12,0), (12,0,4), (12,4,0)								
**Si**	**(1,5,11)**	**13.839**	**85.61**	**5.0192**	**4.65**	**1.0655**	**4.96**	**13.32**	**14.21**
	Equiv. Refl.: (1,11,5), (5,1,11), (5,11,1), (7,7,7), (11,1,5), (11,5,1)								
**Si**	**(0,0,12)**	**13.697**	**80.69**	**3.806**	**3.23**	**2.2743**	**7.35**	**28.43**	**29.36**
	Equiv. Refl.: (0,12,0), (4,8,8), (8,4,8), (8,8,4), (12,0,0)								
**Ge**	**(2,2,12)**	**13.51**	**76.74**	**2.3667**	**5.14**	**3.2721**	**16.81**	**40.9**	**44.22**
	Equiv. Refl.: (2,12,2), (4,6,10), (4,10,6), (6,4,10), (6,10,4), (10,4,6), (10,6,4), (12,2,2)								
**Si**	**(0,6,10)**	**13.311**	**73.54**	**2.3307**	**2.04**	**4.0999**	**8.36**	**51.25**	**51.93**
	Equiv. Refl.: (0,10,6), (6,0,10), (6,6,8), (6,8,6), (6,10,0), (8,6,6), (10,0,6), (10,6,0)								
**Ge**	**(0,0,12)**	**13.149**	**71.33**	**1.9197**	**4.02**	**4.6909**	**18.88**	**58.64**	**61.6**
	Equiv. Refl.: (0,12,0), (4,8,8), (8,4,8), (8,8,4), (12,0,0)								
**Ge**	**(3,5,11)**	**13.642**	**79.38**	**1.5122**	**4.38**	**2.602**	**11.39**	**32.53**	**34.46**
	Equiv. Refl.: (3,11,5), (5,3,11), (5,7,9), (5,9,7), (5,11,3), (7,5,9), (7,9,5), (9,5,7), (9,7,5) (11,3,5), (11,5,3)								
**Si**	**(3,3,11)**	**13.457**	**75.83**	**1.447**	**1.6**	**3.5055**	**5.63**	**43.82**	**44.18**
	Equiv. Refl.: (3,7,9), (3,9,7), (3,11,3), (7,3,9), (7,9,3), (9,3,7), (9,7,3), (11,3,3)								
**Si**	**(1,3,11)**	**13.064**	**70.26**	**1.1763**	**1.29**	**4.9804**	**6.43**	**62.25**	**62.59**
	Equiv. Refl.: (1,7,9), (1,9,7), (1,11,3), (3,1,11), (3,11,1), (5,5,9), (5,9,5), (7,1,9), (7,9,1) (9,1,7), (9,5,5), (9,7,1), (11,1,3), (11,3,1)								
**Ge**	**(1,5,11)**	**13.286**	**73.17**	**1.0995**	**3.04**	**4.1981**	**12.77**	**52.48**	**54.01**
	Equiv. Refl.: (1,11,5), (5,1,11), (5,11,1), (7,7,7), (11,1,5), (11,5,1)								
**LiNbO3**	**(1,-9,16)**	**13.868**	**87.66**	**38.757**	**35.4**	**0.567**	**20.07**	**7.08**	**21.28**
	Equiv. Refl.: (8,1,16), (9,-1,16), (-1,-8,16)								
**LiNbO3**	**(-8,9,16)**	**13.868**	**87.66**	**38.756**	**35.4**	**0.567**	**20.07**	**7.08**	**21.28**
	Equiv. Refl.: (-9,8,16)								
**LiNbO3**	**(1,8,-16)**	**13.868**	**87.66**	**38.441**	**35.4**	**0.567**	**20.07**	**7.08**	**21.28**
	Equiv. Refl.: (-1,9,-16), (-8,-1,-16), (-9,1,-16)								
**LiNbO3**	**(8,-9,-16)**	**13.868**	**87.66**	**38.44**	**35.4**	**0.567**	**20.07**	**7.08**	**21.28**
	Equiv. Refl.: (9,-8,-16)								
**LiNbO3**	**(5,-8,22)**	**13.839**	**85.61**	**26.246**	**22.9**	**1.067**	**24.48**	**13.33**	**27.87**
	Equiv. Refl.: (8,-5,22)								
**LiNbO3**	**(3,5,22)**	**13.839**	**85.61**	**26.245**	**22.9**	**1.067**	**24.48**	**13.33**	**27.87**
	Equiv. Refl.: (-3,8,22), (-5,-3,22), (-8,3,22)								
**LiNbO3**	**(5,3,-22)**	**13.839**	**85.61**	**25.205**	**22.9**	**1.067**	**24.48**	**13.33**	**27.87**
	Equiv. Refl.: (-3,-5,-22), (-5,8,-22), (-8,5,-22)								
**LiNbO3**	**(3,-8,-22)**	**13.839**	**85.61**	**25.204**	**22.9**	**1.067**	**24.48**	**13.33**	**27.87**
	Equiv. Refl.: (8,-3,-22)								
**LiNbO3**	**(3,7,14)**	**13.854**	**86.5**	**22.328**	**20.9**	**0.849**	**17.7**	**10.61**	**20.64**
	Equiv. Refl.: (7,-10,14), (10,-7,14), (-7,-3,14)								
**LiNbO3**	**(-3,10,14)**	**13.854**	**86.5**	**22.327**	**20.9**	**0.849**	**17.7**	**10.61**	**20.64**
	Equiv. Refl.: (-10,3,14)								
**LiNbO3**	**(7,3,-14)**	**13.854**	**86.5**	**22.212**	**20.9**	**0.849**	**17.7**	**10.61**	**20.64**
	Equiv. Refl.: (-3,-7,-14), (-7,10,-14), (-10,7,-14)								
**LiNbO3**	**(3,-10,-14)**	**13.854**	**86.5**	**22.211**	**20.9**	**0.849**	**17.7**	**10.61**	**20.64**
	Equiv. Refl.: (10,-3,-14)								
**LiNbO3**	**(0,7,-22)**	**13.839**	**85.61**	**20.041**	**18.2**	**1.067**	**19.43**	**13.33**	**23.56**
	Equiv. Refl.: (7,-7,-22), (-7,0,-22)								

Ei =	13.88	keV						ΔEg	ΔEt
**Cryst**	**Refl**	**EB**	**ΘB**	**∫ IR dΘ**	**Width**	**Ei cotΘ**	**ΔE**	**2m, 50µm**	**2m, 50µm**
	**(h,k,l)**	**[keV]**	**[°]**	**[µrad]**	**[µrad]**	**[meV/µrad]**	**[meV]**	**[meV]**	**[meV]**
**LiNbO3**	**(3,-11,-4)**	**13.811**	**84.29**	**19.668**	**17.4**	**1.389**	**24.11**	**17.36**	**29.71**
	Equiv. Refl.: (8,3,-4), (11,-3,-4), (-3,-8,-4), (-8,11,-4), (-11,8,-4)								
**LiNbO3**	**(0,-7,22)**	**13.839**	**85.61**	**19.44**	**18.2**	**1.067**	**19.43**	**13.33**	**23.56**
	Equiv. Refl.: (7,0,22), (-7,7,22)								
**LiNbO3**	**(3,8,4)**	**13.811**	**84.29**	**19.147**	**17.4**	**1.389**	**24.11**	**17.36**	**29.71**
	Equiv. Refl.: (8,-11,4), (11,-8,4), (-3,11,4), (-8,-3,4), (-11,3,4)								
**LiNbO3**	**(2,8,-12)**	**13.828**	**85.03**	**18.645**	**16.8**	**1.208**	**20.29**	**15.1**	**25.3**
	Equiv. Refl.: (8,-10,-12), (10,-8,-12), (-2,10,-12), (-8,-2,-12), (-10,2,-12)								
**LiNbO3**	**(2,-10,12)**	**13.828**	**85.03**	**18.001**	**16.8**	**1.208**	**20.29**	**15.1**	**25.3**
	Equiv. Refl.: (8,2,12), (10,-2,12), (-2,-8,12)								
**LiNbO3**	**(-8,10,12)**	**13.828**	**85.03**	**18**	**16.8**	**1.208**	**20.29**	**15.1**	**25.3**
	Equiv. Refl.: (-10,8,12)								
**LiNbO3**	**(8,2,-12)**	**13.828**	**85.03**	**16.533**	**15**	**1.208**	**18.17**	**15.1**	**23.63**
	Equiv. Refl.: (-2,-8,-12), (-8,10,-12), (-10,8,-12)								
**LiNbO3**	**(2,-10,-12)**	**13.828**	**85.03**	**16.532**	**15**	**1.208**	**18.17**	**15.1**	**23.63**
	Equiv. Refl.: (10,-2,-12)								
**LiNbO3**	**(2,8,12)**	**13.828**	**85.03**	**15.708**	**15**	**1.208**	**18.17**	**15.1**	**23.63**
	Equiv. Refl.: (8,-10,12), (10,-8,12), (-2,10,12), (-8,-2,12), (-10,2,12)								
**LiNbO3**	**(2,5,-24)**	**13.805**	**84.05**	**14.471**	**13.3**	**1.446**	**19.19**	**18.08**	**26.36**
	Equiv. Refl.: (5,-7,-24), (7,-5,-24), (-2,7,-24), (-5,-2,-24), (-7,2,-24)								
**LiNbO3**	**(2,-7,24)**	**13.805**	**84.05**	**14.213**	**13.3**	**1.446**	**19.19**	**18.08**	**26.36**
	Equiv. Refl.: (5,2,24), (7,-2,24), (-2,-5,24), (-5,7,24), (-7,5,24)								
**LiNbO3**	**(-5,7,-24)**	**13.805**	**84.05**	**14.119**	**13.1**	**1.446**	**18.9**	**18.08**	**26.15**
	Equiv. Refl.: (-7,5,-24)								
**LiNbO3**	**(2,-7,-24)**	**13.805**	**84.05**	**14.118**	**13.1**	**1.446**	**18.9**	**18.08**	**26.15**
	Equiv. Refl.: (5,2,-24), (7,-2,-24), (-2,-5,-24)								
**LiNbO3**	**(2,5,24)**	**13.805**	**84.05**	**14.061**	**13.1**	**1.446**	**18.9**	**18.08**	**26.15**
	Equiv. Refl.: (5,-7,24), (7,-5,24), (-2,7,24), (-5,-2,24), (-7,2,24)								
**LiNbO3**	**(5,-11,-8)**	**13.738**	**81.81**	**12.388**	**11.1**	**1.998**	**22.24**	**24.98**	**33.44**
	Equiv. Refl.: (6,5,-8), (11,-5,-8), (-5,-6,-8), (-6,11,-8), (-11,6,-8)								
**LiNbO3**	**(5,6,8)**	**13.738**	**81.81**	**12.18**	**11.1**	**1.998**	**22.24**	**24.98**	**33.44**
	Equiv. Refl.: (6,-11,8), (11,-6,8), (-5,11,8), (-6,-5,8), (-11,5,8)								
**LiNbO3**	**(0,-4,28)**	**13.701**	**80.78**	**11.395**	**10.2**	**2.253**	**23.04**	**28.16**	**36.38**
	Equiv. Refl.: (4,0,28), (-4,4,28)								
**LiNbO3**	**(0,4,-28)**	**13.701**	**80.78**	**11.272**	**10.2**	**2.253**	**23.04**	**28.16**	**36.38**
	Equiv. Refl.: (4,-4,-28), (-4,0,-28)								
**LiNbO3**	**(1,-10,8)**	**13.738**	**81.81**	**10.347**	**9.1**	**1.998**	**18.25**	**24.98**	**30.93**
	Equiv. Refl.: (9,1,8), (10,-1,8), (-1,-9,8), (-9,10,8), (-10,9,8)								
**LiNbO3**	**(4,7,6)**	**13.675**	**80.14**	**10.111**	**9**	**2.411**	**21.75**	**30.14**	**37.17**
	Equiv. Refl.: (7,-11,6), (11,-7,6), (-4,11,6), (-7,-4,6), (-11,4,6)								
**LiNbO3**	**(4,-11,-6)**	**13.675**	**80.14**	**9.995**	**9**	**2.411**	**21.75**	**30.14**	**37.17**
	Equiv. Refl.: (7,4,-6), (11,-4,-6), (-4,-7,-6), (-7,11,-6), (-11,7,-6)								
**LiNbO3**	**(4,7,-6)**	**13.675**	**80.14**	**9.817**	**8.6**	**2.411**	**20.71**	**30.14**	**36.57**
	Equiv. Refl.: (7,-11,-6), (11,-7,-6), (-4,11,-6), (-7,-4,-6), (-11,4,-6)								
**LiNbO3**	**(0,-5,26)**	**13.547**	**77.42**	**9.797**	**8.6**	**3.098**	**26.78**	**38.72**	**47.08**
	Equiv. Refl.: (5,0,26)								
**LiNbO3**	**(-5,5,26)**	**13.547**	**77.42**	**9.796**	**8.6**	**3.098**	**26.78**	**38.72**	**47.08**
**LiNbO3**	**(0,5,-26)**	**13.547**	**77.42**	**9.711**	**8.6**	**3.098**	**26.78**	**38.72**	**47.08**
	Equiv. Refl.: (5,-5,-26), (-5,0,-26)								

Ei =	13.88	keV						ΔEg	ΔEt
Cryst	Refl	EB	ΘB	∫ IR dΘ	Width	Ei cotΘ	ΔE	2m, 50µm	2m, 50µm
	(h,k,l)	[keV]	[°]	[µrad]	[µrad]	[meV/µrad]	[meV]	[meV]	[meV]
**LiNbO3**	**(1,9,-8)**	**13.738**	**81.81**	**9.67**	**9.1**	**1.998**	**18.25**	**24.98**	**30.93**
	Equiv. Refl.: (9,-10,-8), (10,-9,-8), (-1,10,-8), (-9,-1,-8), (-10,1,-8)								
**LiNbO3**	**(2,4,-26)**	**13.759**	**82.43**	**9.646**	**8.9**	**1.844**	**16.44**	**23.05**	**28.31**
	Equiv. Refl.: (4,-6,-26), (6,-4,-26), (-2,6,-26), (-4,-2,-26), (-6,2,-26)								
**LiNbO3**	**(2,-6,26)**	**13.759**	**82.43**	**9.503**	**8.9**	**1.844**	**16.44**	**23.05**	**28.31**
	Equiv. Refl.: (4,2,26), (6,-2,26), (-2,-4,26), (-4,6,26), (-6,4,26)								
**LiNbO3**	**(3,6,-18)**	**13.66**	**79.79**	**9.497**	**8.4**	**2.501**	**21.01**	**31.26**	**37.66**
	Equiv. Refl.: (6,-9,-18), (9,-6,-18), (-3,9,-18), (-6,-3,-18), (-9,3,-18)								
**LiNbO3**	**(4,-11,6)**	**13.675**	**80.14**	**9.429**	**8.6**	**2.411**	**20.71**	**30.14**	**36.57**
	Equiv. Refl.: (7,4,6), (11,-4,6), (-4,-7,6)								
**LiNbO3**	**(0,9,12)**	**13.616**	**78.81**	**9.428**	**8.2**	**2.745**	**22.4**	**34.31**	**40.97**
	Equiv. Refl.: (9,-9,12), (-7,11,6), (-9,0,12), (-11,7,6)								
**LiNbO3**	**(3,-9,18)**	**13.66**	**79.79**	**9.365**	**8.4**	**2.501**	**21.01**	**31.26**	**37.66**
	Equiv. Refl.: (6,3,18), (9,-3,18), (-3,-6,18), (-6,9,18), (-9,6,18)								
**LiNbO3**	**(0,-9,-12)**	**13.616**	**78.81**	**9.089**	**8.2**	**2.745**	**22.4**	**34.31**	**40.97**
	Equiv. Refl.: (9,0,-12), (-9,9,-12)								
**LiNbO3**	**(0,0,-30)**	**13.415**	**75.13**	**8.957**	**7.8**	**3.685**	**28.69**	**46.06**	**54.27**
**LiNbO3**	**(0,0,30)**	**13.415**	**75.13**	**8.723**	**7.8**	**3.685**	**28.69**	**46.06**	**54.27**
**LiNbO3**	**(3,6,18)**	**13.66**	**79.79**	**8.319**	**7.5**	**2.501**	**18.85**	**31.26**	**36.5**
	Equiv. Refl.: (6,-9,18), (9,-6,18), (-6,-3,18)								
**LiNbO3**	**(-3,9,18)**	**13.66**	**79.79**	**8.318**	**7.5**	**2.501**	**18.85**	**31.26**	**36.5**
	Equiv. Refl.: (-9,3,18)								
**LiNbO3**	**(3,-9,-18)**	**13.66**	**79.79**	**8.262**	**7.5**	**2.501**	**18.85**	**31.26**	**36.5**
	Equiv. Refl.: (6,3,-18), (9,-3,-18), (-3,-6,-18), (-6,9,-18), (-9,6,-18)								
**LiNbO3**	**(4,6,-14)**	**13.643**	**79.4**	**8.225**	**7.3**	**2.597**	**19.01**	**32.47**	**37.62**
	Equiv. Refl.: (6,-10,-14), (10,-6,-14), (-4,10,-14), (-6,-4,-14), (-10,4,-14)								
**LiNbO3**	**(1,-4,-28)**	**13.487**	**76.34**	**8.192**	**7.2**	**3.373**	**24.12**	**42.17**	**48.58**
	Equiv. Refl.: (3,1,-28), (4,-1,-28), (-1,-3,-28), (-3,4,-28), (-4,3,-28)								
**LiNbO3**	**(1,3,28)**	**13.487**	**76.34**	**8.187**	**7.2**	**3.373**	**24.12**	**42.17**	**48.58**
	Equiv. Refl.: (3,-4,28), (4,-3,28), (-1,4,28), (-3,-1,28), (-4,1,28)								
**LiNbO3**	**(4,-10,14)**	**13.643**	**79.4**	**7.893**	**7.3**	**2.597**	**19.01**	**32.47**	**37.62**
	Equiv. Refl.: (6,4,14), (10,-4,14), (-4,-6,14), (-6,10,14), (-10,6,14)								
**LiNbO3**	**(2,7,16)**	**13.444**	**75.6**	**7.402**	**6.4**	**3.565**	**22.79**	**44.56**	**50.05**
	Equiv. Refl.: (7,-9,16), (9,-7,16), (-2,9,16), (-7,-2,16), (-9,2,16)								
**LiNbO3**	**(0,-6,-24)**	**13.594**	**78.34**	**7.376**	**6.6**	**2.864**	**18.81**	**35.8**	**40.44**
	Equiv. Refl.: (6,0,-24), (-6,6,-24)								
**LiNbO3**	**(5,6,-4)**	**13.384**	**74.64**	**7.333**	**6.3**	**3.812**	**24.14**	**47.65**	**53.41**
	Equiv. Refl.: (6,-11,-4), (11,-6,-4), (-5,11,-4), (-6,-5,-4), (-11,5,-4)								
**LiNbO3**	**(1,-9,-14)**	**13.429**	**75.35**	**7.296**	**6.4**	**3.628**	**23.1**	**45.35**	**50.89**
	Equiv. Refl.: (8,1,-14), (9,-1,-14), (-1,-8,-14), (-8,9,-14), (-9,8,-14)								
**LiNbO3**	**(1,8,14)**	**13.429**	**75.35**	**7.159**	**6.4**	**3.628**	**23.1**	**45.35**	**50.89**
	Equiv. Refl.: (8,-9,14), (9,-8,14), (-1,9,14), (-8,-1,14), (-9,1,14)								
**LiNbO3**	**(5,-11,4)**	**13.384**	**74.64**	**7.141**	**6.3**	**3.812**	**24.14**	**47.65**	**53.41**
	Equiv. Refl.: (6,5,4), (11,-5,4), (-5,-6,4), (-6,11,4), (-11,6,4)								
**LiNbO3**	**(2,-9,-16)**	**13.444**	**75.6**	**7.085**	**6.4**	**3.565**	**22.79**	**44.56**	**50.05**
	Equiv. Refl.: (7,2,-16), (9,-2,-16), (-2,-7,-16), (-7,9,-16), (-9,7,-16)								
**LiNbO3**	**(0,6,24)**	**13.594**	**78.34**	**7.039**	**6.6**	**2.864**	**18.81**	**35.8**	**40.44**
	Equiv. Refl.: (6,-6,24), (-6,0,24)								
**LiNbO3**	**(3,-11,2)**	**13.724**	**81.4**	**6.804**	**6.5**	**2.099**	**13.7**	**26.24**	**29.6**
	Equiv. Refl.: (8,3,2), (11,-3,2), (-3,-8,2), (-8,11,2), (-11,8,2)								

Ei =	13.88	keV						ΔEg	ΔEt
**Cryst**	**Refl**	**EB**	**ΘB**	**∫ IR dΘ**	**Width**	**Ei cotΘ**	**ΔE**	**2m, 50µm**	**2m, 50µm**
	**(h,k,l)**	**[keV]**	**[°]**	**[µrad]**	**[µrad]**	**[meV/µrad]**	**[meV]**	**[meV]**	**[meV]**
**LiNbO3**	**(0,6,-24)**	**13.594**	**78.34**	**6.782**	**6.2**	**2.864**	**17.86**	**35.8**	**40.01**
	Equiv. Refl.: (6,-6,-24), (-6,0,-24)								
**LiNbO3**	**(0,-6,24)**	**13.594**	**78.34**	**6.767**	**6.2**	**2.864**	**17.86**	**35.8**	**40.01**
	Equiv. Refl.: (6,0,24), (-6,6,24)								
**LiNbO3**	**(1,9,-2)**	**13.295**	**73.3**	**6.761**	**5.9**	**4.164**	**24.42**	**52.05**	**57.5**
	Equiv. Refl.: (9,-10,-2), (10,-9,-2), (-1,10,-2), (-9,-1,-2), (-10,1,-2)								
**LiNbO3**	**(3,8,-2)**	**13.724**	**81.4**	**6.655**	**6.5**	**2.099**	**13.7**	**26.24**	**29.6**
	Equiv. Refl.: (8,-11,-2), (11,-8,-2), (-3,11,-2), (-8,-3,-2), (-11,3,-2)								
**LiNbO3**	**(1,-10,2)**	**13.295**	**73.3**	**6.633**	**5.9**	**4.164**	**24.42**	**52.05**	**57.5**
	Equiv. Refl.: (9,1,2), (10,-1,2), (-1,-9,2), (-9,10,2), (-10,9,2)								
**Sapph**	**(0,8,-14)**	**13.766**	**82.65**	**6.624**	**5.4**	**1.791**	**9.74**	**22.38**	**24.41**
	Equiv. Refl.: (0,-8,14), (8,0,14), (8,-8,-14), (-8,0,-14), (-8,8,14)								
**LiNbO3**	**(0,-9,12)**	**13.616**	**78.81**	**6.541**	**6**	**2.745**	**16.44**	**34.31**	**38.04**
	Equiv. Refl.: (9,0,12), (-9,9,12)								
**LiNbO3**	**(1,1,30)**	**13.63**	**79.1**	**6.448**	**6**	**2.672**	**15.91**	**33.4**	**37**
	Equiv. Refl.: (1,-2,30), (2,-1,30), (-1,2,30), (-1,-1,30), (-2,1,30)								
**LiNbO3**	**(2,3,26)**	**13.112**	**70.85**	**6.423**	**5.6**	**4.821**	**26.76**	**60.26**	**65.94**
	Equiv. Refl.: (3,-5,26), (5,-3,26), (-2,5,26), (-3,-2,26), (-5,2,26)								
**LiNbO3**	**(2,-5,-26)**	**13.112**	**70.85**	**6.396**	**5.6**	**4.821**	**26.76**	**60.26**	**65.94**
	Equiv. Refl.: (3,2,-26), (5,-2,-26), (-2,-3,-26), (-3,5,-26), (-5,3,-26)								
**LiNbO3**	**(0,-7,-20)**	**13.218**	**72.24**	**6.278**	**5.5**	**4.446**	**24.29**	**55.58**	**60.65**
	Equiv. Refl.: (7,0,-20), (-7,7,-20)								
**LiNbO3**	**(0,9,-12)**	**13.616**	**78.81**	**6.267**	**6**	**2.745**	**16.44**	**34.31**	**38.04**
	Equiv. Refl.: (9,-9,-12), (-9,0,-12)								
**LiNbO3**	**(1,1,-30)**	**13.63**	**79.1**	**6.204**	**6**	**2.672**	**15.91**	**33.4**	**37**
	Equiv. Refl.: (1,-2,-30), (2,-1,-30), (-1,2,-30), (-1,-1,-30), (-2,1,-30)								
**LiNbO3**	**(0,7,20)**	**13.218**	**72.24**	**6.169**	**5.5**	**4.446**	**24.29**	**55.58**	**60.65**
	Equiv. Refl.: (7,-7,20), (-7,0,20)								
**LiNbO3**	**(1,-8,-18)**	**13.229**	**72.38**	**5.96**	**5.2**	**4.409**	**23.13**	**55.11**	**59.77**
	Equiv. Refl.: (7,1,-18), (8,-1,-18), (-1,-7,-18), (-7,8,-18), (-8,7,-18)								
**LiNbO3**	**(3,-10,10)**	**13.143**	**71.25**	**5.95**	**5.2**	**4.713**	**24.56**	**58.91**	**63.82**
	Equiv. Refl.: (7,3,10), (10,-3,10), (-3,-7,10), (-7,10,10), (-10,7,10)								
**LiNbO3**	**(3,7,-10)**	**13.143**	**71.25**	**5.916**	**5.2**	**4.713**	**24.56**	**58.91**	**63.82**
	Equiv. Refl.: (7,-10,-10), (10,-7,-10), (-3,10,-10), (-7,-3,-10), (-10,3,-10)								
**Sapph**	**(1,6,-20)**	**13.727**	**81.5**	**5.913**	**4.8**	**2.075**	**10.03**	**25.93**	**27.8**
	Equiv. Refl.: (1,-7,20), (6,1,20), (6,-7,-20), (7,-1,20), (7,-6,-20), (-1,7,-20), (-1,-6,20), (-6,7,20), (-6,-1,-20) (-7,1,-20), (-7,6,20)								
**LiNbO3**	**(1,7,18)**	**13.229**	**72.38**	**5.891**	**5.2**	**4.409**	**23.13**	**55.11**	**59.77**
	Equiv. Refl.: (7,-8,18), (8,-7,18), (-1,8,18), (-7,-1,18), (-8,1,18)								
**LiNbO3**	**(4,-11,0)**	**13.409**	**75.04**	**5.831**	**5.3**	**3.709**	**19.5**	**46.37**	**50.3**
	Equiv. Refl.: (7,4,0), (11,-4,0), (-4,-7,0), (-7,11,0), (-11,7,0)								
**LiNbO3**	**(0,-8,-16)**	**13.226**	**72.34**	**5.788**	**4.6**	**4.418**	**20.53**	**55.22**	**58.92**
	Equiv. Refl.: (8,0,-16), (-8,8,-16)								
**LiNbO3**	**(2,-8,-20)**	**13.436**	**75.47**	**5.787**	**5.2**	**3.598**	**18.66**	**44.97**	**48.69**
	Equiv. Refl.: (6,2,-20), (8,-2,-20), (-2,-6,-20), (-6,8,-20), (-8,6,-20)								
**LiNbO3**	**(5,5,-12)**	**13.183**	**71.77**	**5.771**	**5**	**4.571**	**22.83**	**57.14**	**61.53**
	Equiv. Refl.: (5,-10,-12), (10,-5,-12), (-5,10,-12), (-5,-5,-12), (-10,5,-12)								
**LiNbO3**	**(4,7,0)**	**13.409**	**75.04**	**5.729**	**5.3**	**3.709**	**19.5**	**46.37**	**50.3**
	Equiv. Refl.: (7,-11,0), (11,-7,0), (-4,11,0), (-7,-4,0), (-11,4,0)								
**LiNbO3**	**(1,6,22)**	**13.414**	**75.1**	**5.633**	**4.9**	**3.692**	**18.26**	**46.15**	**49.64**
	Equiv. Refl.: (6,-7,22), (7,-6,22), (-1,7,22), (-6,-1,22), (-7,1,22)								

Ei =	13.88	keV						ΔEg	ΔEt
Cryst	Refl	EB	ΘB	∫ IR dΘ	Width	Ei cotΘ	ΔE	2m, 50µm	2m, 50µm
	(h,k,l)	[keV]	[°]	[µrad]	[µrad]	[meV/µrad]	[meV]	[meV]	[meV]
**LiNbO3**	**(2,6,20)**	**13.436**	**75.47**	**5.532**	**5.2**	**3.598**	**18.66**	**44.97**	**48.69**
	Equiv. Refl.: (6,-8,20), (8,-6,20), (-2,8,20), (-6,-2,20), (-8,2,20)								
**LiNbO3**	**(1,-10,-4)**	**13.384**	**74.64**	**5.527**	**4.8**	**3.812**	**18.4**	**47.65**	**51.08**
	Equiv. Refl.: (9,1,-4), (10,-1,-4), (-1,-9,-4), (-9,10,-4), (-10,9,-4)								
**LiNbO3**	**(1,-7,-22)**	**13.414**	**75.1**	**5.478**	**4.9**	**3.692**	**18.26**	**46.15**	**49.64**
	Equiv. Refl.: (6,1,-22), (7,-1,-22), (-1,-6,-22), (-6,7,-22), (-7,6,-22)								
**LiNbO3**	**(5,5,12)**	**13.183**	**71.77**	**5.471**	**5**	**4.571**	**22.83**	**57.14**	**61.53**
	Equiv. Refl.: (5,-10,12), (10,-5,12), (-5,10,12), (-5,-5,12), (-10,5,12)								
**LiNbO3**	**(1,-8,18)**	**13.229**	**72.38**	**5.357**	**4.8**	**4.409**	**21.21**	**55.11**	**59.05**
	Equiv. Refl.: (7,1,18), (8,-1,18), (-1,-7,18), (-7,8,18), (-8,7,18)								
**LiNbO3**	**(1,7,-18)**	**13.229**	**72.38**	**5.334**	**4.8**	**4.409**	**21.21**	**55.11**	**59.05**
	Equiv. Refl.: (7,-8,-18), (8,-7,-18), (-1,8,-18), (-7,-1,-18), (-8,1,-18)								
**LiNbO3**	**(1,9,4)**	**13.384**	**74.64**	**5.326**	**4.8**	**3.812**	**18.4**	**47.65**	**51.08**
	Equiv. Refl.: (9,-10,4), (10,-9,4), (-1,10,4), (-9,-1,4), (-10,1,4)								
**LiNbO3**	**(1,2,-28)**	**13.05**	**70.09**	**5.314**	**4.7**	**5.028**	**23.57**	**62.85**	**67.12**
	Equiv. Refl.: (2,-3,-28), (3,-2,-28), (-1,3,-28), (-2,-1,-28), (-3,1,-28)								
**LiNbO3**	**(1,-3,28)**	**13.05**	**70.09**	**5.257**	**4.7**	**5.028**	**23.57**	**62.85**	**67.12**
	Equiv. Refl.: (2,1,28), (3,-1,28), (-1,-2,28), (-2,3,28), (-3,2,28)								
**LiNbO3**	**(0,8,16)**	**13.226**	**72.34**	**5.045**	**4.6**	**4.418**	**20.53**	**55.22**	**58.92**
	Equiv. Refl.: (8,-8,16), (-8,0,16)								
**LiNbO3**	**(3,5,-20)**	**13.218**	**72.24**	**4.884**	**4**	**4.446**	**17.94**	**55.58**	**58.4**
	Equiv. Refl.: (-3,8,-20), (-5,-3,-20), (-8,3,-20)								
**LiNbO3**	**(5,-8,-20)**	**13.218**	**72.24**	**4.883**	**4**	**4.446**	**17.94**	**55.58**	**58.4**
	Equiv. Refl.: (8,-5,-20)								
**LiNbO3**	**(5,-11,-2)**	**13.295**	**73.3**	**4.866**	**4.2**	**4.164**	**17.49**	**52.05**	**54.91**
	Equiv. Refl.: (6,5,-2), (11,-5,-2), (-5,-6,-2), (-6,11,-2), (-11,6,-2)								
**Sapph**	**(0,9,0)**	**13.54**	**77.29**	**4.857**	**3.7**	**3.131**	**11.65**	**39.14**	**40.83**
	Equiv. Refl.: (0,-9,0), (9,0,0), (9,-9,0), (-9,0,0), (-9,9,0), (-9,2,-6), (-9,7,6)								
**Quartz**	**(2,7,-6)**	**13.769**	**82.76**	**4.855**	**3.8**	**1.764**	**6.78**	**22.05**	**23.07**
	Equiv. Refl.: (2,-9,6), (7,-9,-6)								
**Quartz**	**(7,2,6)**	**13.769**	**82.76**	**4.854**	**3.8**	**1.764**	**6.78**	**22.05**	**23.07**
**LiNbO3**	**(5,6,2)**	**13.295**	**73.3**	**4.784**	**4.2**	**4.164**	**17.49**	**52.05**	**54.91**
	Equiv. Refl.: (6,-11,2), (11,-6,2), (-5,11,2), (-6,-5,2), (-11,5,2)								
**Quartz**	**(9,-2,6)**	**13.769**	**82.76**	**4.781**	**3.8**	**1.764**	**6.78**	**22.05**	**23.07**
	Equiv. Refl.: (9,-7,-6)								
**Quartz**	**(-2,9,-6)**	**13.769**	**82.76**	**4.779**	**3.8**	**1.764**	**6.78**	**22.05**	**23.07**
	Equiv. Refl.: (-2,-7,6), (-7,9,6)								
**Quartz**	**(-7,-2,-6)**	**13.769**	**82.76**	**4.778**	**3.8**	**1.764**	**6.78**	**22.05**	**23.07**
**Sapph**	**(2,8,0)**	**13.788**	**83.41**	**4.612**	**3.9**	**1.604**	**6.21**	**20.05**	**20.99**
	Equiv. Refl.: (2,-10,0), (8,2,0), (8,-10,0), (10,-2,0), (10,-8,0), (-2,10,0), (-2,-8,0), (-8,10,0), (-8,-2,0) (-10,2,0), (-10,8,0)								
**Quartz**	**(0,9,-3)**	**13.557**	**77.61**	**4.553**	**3.4**	**3.049**	**10.51**	**38.11**	**39.53**
	Equiv. Refl.: (0,-9,3), (9,0,3), (9,-9,-3), (-9,0,-3), (-9,9,3)								
**Quartz**	**(-6,3,-10)**	**13.743**	**81.95**	**4.382**	**3.5**	**1.962**	**6.78**	**24.52**	**25.44**
**Quartz**	**(-6,3,10)**	**13.743**	**81.95**	**4.381**	**3.5**	**1.962**	**6.78**	**24.52**	**25.44**
**Quartz**	**(3,-6,-10)**	**13.743**	**81.95**	**4.378**	**3.5**	**1.962**	**6.78**	**24.52**	**25.44**
**Quartz**	**(3,3,10)**	**13.743**	**81.95**	**4.377**	**3.5**	**1.962**	**6.78**	**24.52**	**25.44**
**Quartz**	**(3,3,-10)**	**13.743**	**81.95**	**4.375**	**3.5**	**1.962**	**6.77**	**24.52**	**25.44**
	Equiv. Refl.: (3,-6,10)								

Ei =	13.88	keV						ΔEg	ΔEt
Cryst	Refl	EB	ΘB	∫ IR dΘ	Width	Ei cotΘ	ΔE	2m, 50µm	2m, 50µm
	(h,k,l)	[keV]	[°]	[µrad]	[µrad]	[meV/µrad]	[meV]	[meV]	[meV]
**LiNbO3**	**(3,-8,20)**	**13.218**	**72.24**	**4.305**	**4**	**4.446**	**17.94**	**55.58**	**58.4**
	Equiv. Refl.: (5,3,20), (8,-3,20), (-3,-5,20)								
**LiNbO3**	**(-5,8,20)**	**13.218**	**72.24**	**4.304**	**4**	**4.446**	**17.94**	**55.58**	**58.4**
	Equiv. Refl.: (-8,5,20)								
**Quartz**	**(6,-3,10)**	**13.743**	**81.95**	**4.289**	**3.5**	**1.962**	**6.78**	**24.52**	**25.44**
**Quartz**	**(6,-3,-10)**	**13.743**	**81.95**	**4.288**	**3.5**	**1.962**	**6.78**	**24.52**	**25.44**
**Quartz**	**(-3,6,10)**	**13.743**	**81.95**	**4.285**	**3.5**	**1.962**	**6.78**	**24.52**	**25.44**
**Quartz**	**(-3,-3,-10)**	**13.743**	**81.95**	**4.284**	**3.5**	**1.962**	**6.78**	**24.52**	**25.44**
**Quartz**	**(-3,6,-10)**	**13.743**	**81.95**	**4.282**	**3.5**	**1.962**	**6.77**	**24.52**	**25.44**
	Equiv. Refl.: (-3,-3,10)								
**Sapph**	**(3,4,20)**	**13.224**	**72.31**	**4.191**	**3.2**	**4.427**	**13.99**	**55.34**	**57.08**
	Equiv. Refl.: (3,-7,-20), (4,3,-20), (4,-7,20), (7,-3,-20), (7,-4,20), (-3,7,20), (-3,-4,-20), (-4,7,-20), (-4,-3,20) (-7,3,20), (-7,4,-20)								
**Quartz**	**(-3,4,-11)**	**13.667**	**79.95**	**4.16**	**3.2**	**2.459**	**7.88**	**30.74**	**31.74**
	Equiv. Refl.: (-3,-1,11)								
**Quartz**	**(4,-1,-11)**	**13.667**	**79.95**	**4.159**	**3.2**	**2.459**	**7.88**	**30.74**	**31.74**
	Equiv. Refl.: (4,-3,11)								
**Quartz**	**(-1,-3,-11)**	**13.667**	**79.95**	**4.157**	**3.2**	**2.459**	**7.88**	**30.74**	**31.73**
**Quartz**	**(-1,4,11)**	**13.667**	**79.95**	**4.156**	**3.2**	**2.459**	**7.88**	**30.74**	**31.73**
**Quartz**	**(4,2,-10)**	**13.82**	**84.69**	**4.106**	**3.4**	**1.29**	**4.4**	**16.13**	**16.72**
	Equiv. Refl.: (4,-6,10)								
**Quartz**	**(2,-6,-10)**	**13.82**	**84.69**	**4.098**	**3.4**	**1.29**	**4.4**	**16.13**	**16.72**
**Quartz**	**(-6,2,10)**	**13.82**	**84.69**	**4.097**	**3.4**	**1.29**	**4.39**	**16.13**	**16.72**
	Equiv. Refl.: (-6,4,-10)								
**Quartz**	**(2,4,10)**	**13.82**	**84.69**	**4.095**	**3.4**	**1.29**	**4.39**	**16.13**	**16.72**
**Quartz**	**(3,1,-11)**	**13.667**	**79.95**	**4.07**	**3.2**	**2.459**	**7.88**	**30.74**	**31.74**
	Equiv. Refl.: (3,-4,11)								
**Quartz**	**(-4,1,11)**	**13.667**	**79.95**	**4.068**	**3.2**	**2.459**	**7.88**	**30.74**	**31.74**
	Equiv. Refl.: (-4,3,-11)								
**Sapph**	**(1,4,24)**	**13.37**	**74.41**	**4.066**	**3.1**	**3.872**	**12.06**	**48.4**	**49.88**
	Equiv. Refl.: (1,4,-24), (1,-5,24), (1,-5,-24), (4,1,24), (4,1,-24), (4,-5,24), (4,-5,-24), (5,-1,24), (5,-1,-24) (5,-4,24), (5,-4,-24), (-1,5,24), (-1,5,-24), (-1,-4,24), (-1,-4,-24), (-4,5,24), (-4,5,-24), (-4,-1,24) (-4,-1,-24), (-5,1,24), (-5,1,-24), (-5,4,24), (-5,4,-24), (1,3,11)								
**Quartz**	**(1,-4,-11)**	**13.667**	**79.95**	**4.065**	**3.2**	**2.459**	**7.88**	**30.74**	**31.73**
**Quartz**	**(7,3,-3)**	**13.399**	**74.88**	**4.041**	**3**	**3.751**	**11.21**	**46.89**	**48.21**
	Equiv. Refl.: (7,-10,3), (-10,3,3), (-10,7,-3)								
**Quartz**	**(3,7,3)**	**13.399**	**74.88**	**4.04**	**3**	**3.751**	**11.21**	**46.89**	**48.21**
	Equiv. Refl.: (3,-10,-3)								
**Quartz**	**(10,-3,-3)**	**13.399**	**74.88**	**4.024**	**3**	**3.751**	**11.21**	**46.89**	**48.21**
	Equiv. Refl.: (10,-7,3), (-7,10,-3), (-7,-3,3)								
**Quartz**	**(-3,10,3)**	**13.399**	**74.88**	**4.023**	**3**	**3.751**	**11.21**	**46.89**	**48.21**
	Equiv. Refl.: (-3,-7,-3)								
**Sapph**	**(4,5,14)**	**13.517**	**76.87**	**3.987**	**3.1**	**3.238**	**10.1**	**40.48**	**41.72**
	Equiv. Refl.: (4,-9,-14), (5,4,-14), (5,-9,14), (9,-4,-14), (9,-5,14), (-4,9,14), (-4,-5,-14), (-5,9,-14), (-5,-4,14) (-9,4,14), (-9,5,-14)								
**Quartz**	**(-4,6,-10)**	**13.82**	**84.69**	**3.846**	**3.4**	**1.29**	**4.4**	**16.13**	**16.72**
	Equiv. Refl.: (-4,-2,10)								
**Quartz**	**(-2,6,10)**	**13.82**	**84.69**	**3.838**	**3.4**	**1.29**	**4.4**	**16.13**	**16.72**
**Quartz**	**(6,-2,-10)**	**13.82**	**84.69**	**3.837**	**3.4**	**1.29**	**4.39**	**16.13**	**16.72**
	Equiv. Refl.: (6,-4,10)								
**Quartz**	**(-2,-4,-10)**	**13.82**	**84.69**	**3.835**	**3.4**	**1.29**	**4.39**	**16.13**	**16.72**

Ei =	13.88	keV						ΔEg	ΔEt
Cryst	Refl	EB	ΘB	∫ IR dΘ	Width	Ei cotΘ	ΔE	2m, 50μm	2m, 50μm
	(h,k,l)	[keV]	[°]	[μrad]	[μrad]	[meV/μrad]	[meV]	[meV]	[meV]
Sapph	(4,6,-8)	13.66	79.78	3.726	3.1	2.503	7.64	31.28	32.2
	Equiv. Refl.: (4,-10,8), (6,4,8), (6,-10,-8), (10,-4,8), (10,-6,-8), (-4,10,-8), (-4,-6,8), (-6,10,8), (-6,-4,-8) (-10,4,-8), (-10,6,8)								
Sapph	(0,9,6)	13.839	85.61	3.521	3.2	1.065	3.45	13.31	13.75
	Equiv. Refl.: (0,9,-6), (0,-9,6), (0,-9,-6), (9,0,6), (9,0,-6), (9,-9,6), (9,-9,-6), (-9,0,6), (-9,0,-6) (-9,9,6), (-9,9,-6)								
Quartz	(2,8,2)	13.549	77.46	3.268	2.8	3.086	8.55	38.58	39.51
	Equiv. Refl.: (2,-10,-2)								
Quartz	(8,2,-2)	13.549	77.46	3.267	2.8	3.086	8.55	38.58	39.51
	Equiv. Refl.: (8,-10,2), (-10,2,2), (-10,8,-2)								
Quartz	(7,0,8)	13.719	81.27	3.262	2.9	2.131	6.13	26.63	27.33
	Equiv. Refl.: (7,-7,-8), (-7,0,-8), (-7,7,8)								
Quartz	(0,7,-8)	13.719	81.27	3.258	2.9	2.131	6.13	26.63	27.33
	Equiv. Refl.: (0,-7,8)								
Sapph	(5,5,6)	13.34	73.96	3.156	2.5	3.99	9.94	49.88	50.86
	Equiv. Refl.: (5,5,-6), (5,-10,6), (5,-10,-6), (10,-5,6), (10,-5,-6), (-5,10,6), (-5,10,-6), (-5,-5,6), (-5,-5,-6) (-10,5,6), (-10,5,-6)								
Sapph	(3,3,24)	13.868	87.63	3.14	3.7	0.574	2.1	7.17	7.48
	Equiv. Refl.: (3,3,-24), (3,-6,24), (3,-6,-24), (6,-3,24), (6,-3,-24), (-3,6,24), (-3,6,-24), (-3,-3,24), (-3,-3,-24) (-6,3,24), (-6,3,-24), (-2,10,2), (-2,-8,-2)								
Quartz	(10,-2,-2)	13.549	77.46	3.139	2.8	3.086	8.55	38.58	39.51
	Equiv. Refl.: (10,-8,2), (-8,10,-2), (-8,-2,2)								
Sapph	(1,5,23)	13.808	84.18	3.131	2.8	1.415	3.97	17.69	18.13
	Equiv. Refl.: (1,-6,-23), (5,1,-23), (5,-6,23), (6,-1,-23), (6,-5,23), (-1,6,23), (-1,-5,-23), (-5,6,-23), (-5,-1,23) (-6,1,23), (-6,5,-23)								
Sapph	(3,7,2)	13.406	74.98	3.104	2.5	3.725	9.25	46.56	47.47
	Equiv. Refl.: (3,-10,-2), (7,3,-2), (7,-10,2), (10,-3,-2), (10,-7,2), (-3,10,2), (-3,-7,-2), (-7,10,-2), (-7,-3,2) (-10,3,2), (-10,7,-2)								
Sapph	(2,6,-16)	13.267	72.91	2.998	2.3	4.268	9.66	53.36	54.22
	Equiv. Refl.: (2,-8,16), (6,2,16), (6,-8,-16), (8,-2,16), (8,-6,-16), (-2,8,-16), (-2,-6,16), (-6,8,16), (-6,-2,-16) (-8,2,-16), (-8,6,16)								
Quartz	(10,-2,1)	13.403	74.93	2.983	2.5	3.737	9.48	46.72	47.67
	Equiv. Refl.: (10,-8,-1), (-2,10,-1), (-2,-8,1), (-8,10,1), (-8,-2,-1)								
Quartz	(-10,4,4)	13.505	76.65	2.967	2.2	3.294	7.31	41.17	41.82
	Equiv. Refl.: (-10,6,-4)								
Quartz	(6,4,-4)	13.505	76.65	2.966	2.2	3.294	7.31	41.17	41.82
	Equiv. Refl.: (6,-10,4)								
Quartz	(4,6,4)	13.505	76.65	2.965	2.2	3.294	7.31	41.17	41.82
	Equiv. Refl.: (4,-10,-4)								
Quartz	(2,8,-1)	13.403	74.93	2.943	2.5	3.737	9.48	46.72	47.67
	Equiv. Refl.: (2,-10,1), (-4,1,-11), (-10,8,1)								
Quartz	(8,2,1)	13.403	74.93	2.942	2.5	3.737	9.47	46.72	47.67
	Equiv. Refl.: (8,-10,-1), (-4,3,11), (-10,2,-1)								
Quartz	(3,-4,-11)	13.667	79.95	2.94	2.2	2.459	5.46	30.74	31.22
Quartz	(3,1,11)	13.667	79.95	2.939	2.2	2.459	5.46	30.74	31.22
Quartz	(10,-4,-4)	13.505	76.65	2.937	2.2	3.294	7.31	41.17	41.82
	Equiv. Refl.: (10,-6,4)								
Quartz	(-4,10,4)	13.505	76.65	2.936	2.2	3.294	7.31	41.17	41.82
	Equiv. Refl.: (-6,10,-4), (-6,-4,4)								

Ei = Cryst	13.88 Refl (h,k,l)	keV EB [keV]	ΘB [°]	∫ IR dΘ [µrad]	Width [µrad]	Ei cotΘ [meV/µrad]	ΔE [meV]	ΔEg 2m, 50µm [meV]	ΔEt 2m, 50µm [meV]
**Quartz**	**(1,3,-11)**	**13.667**	**79.95**	**2.935**	**2.2**	**2.459**	**5.46**	**30.74**	**31.22**
	Equiv. Refl.: (1,-4,11), (-4,-6,-4)								
**Quartz**	**(1,0,-12)**	**13.841**	**85.71**	**2.92**	**2.7**	**1.041**	**2.77**	**13.01**	**13.31**
	Equiv. Refl.: (1,-1,12), (-1,0,12), (-1,1,-12)								
**Quartz**	**(0,1,12)**	**13.841**	**85.71**	**2.913**	**2.7**	**1.041**	**2.77**	**13.01**	**13.31**
	Equiv. Refl.: (0,-1,-12)								
**Quartz**	**(4,1,10)**	**13.272**	**72.98**	**2.823**	**2.1**	**4.249**	**8.75**	**53.12**	**53.83**
	Equiv. Refl.: (4,-5,-10)								
**Quartz**	**(1,4,-10)**	**13.272**	**72.98**	**2.821**	**2.1**	**4.249**	**8.74**	**53.12**	**53.83**
**Quartz**	**(1,-5,10)**	**13.272**	**72.98**	**2.82**	**2.1**	**4.249**	**8.74**	**53.12**	**53.83**
**Quartz**	**(-5,1,-10)**	**13.272**	**72.98**	**2.818**	**2.1**	**4.249**	**8.73**	**53.12**	**53.83**
	Equiv. Refl.: (-5,4,10)								
**Quartz**	**(-4,5,10)**	**13.272**	**72.98**	**2.763**	**2.1**	**4.249**	**8.75**	**53.12**	**53.83**
	Equiv. Refl.: (-4,-1,-10)								
**Quartz**	**(-1,-4,10)**	**13.272**	**72.98**	**2.762**	**2.1**	**4.249**	**8.74**	**53.12**	**53.83**
**Quartz**	**(-1,5,-10)**	**13.272**	**72.98**	**2.761**	**2.1**	**4.249**	**8.74**	**53.12**	**53.83**
**Quartz**	**(5,-1,10)**	**13.272**	**72.98**	**2.759**	**2.1**	**4.249**	**8.73**	**53.12**	**53.83**
	Equiv. Refl.: (5,-4,-10)								
**Quartz**	**(-3,5,-10)**	**13.111**	**70.84**	**2.499**	**2.1**	**4.823**	**10.22**	**60.29**	**61.15**
	Equiv. Refl.: (-3,-2,10)								
**Quartz**	**(5,-2,-10)**	**13.111**	**70.84**	**2.498**	**2.1**	**4.823**	**10.22**	**60.29**	**61.15**
	Equiv. Refl.: (5,-3,10), (-2,5,10), (-2,-3,-10)								
**Quartz**	**(3,2,-10)**	**13.111**	**70.84**	**2.474**	**2.1**	**4.823**	**10.22**	**60.29**	**61.15**
**Quartz**	**(2,3,10)**	**13.111**	**70.84**	**2.473**	**2.1**	**4.823**	**10.22**	**60.29**	**61.15**
	Equiv. Refl.: (3,-5,10), (-5,2,10), (-5,3,-10)								
**Quartz**	**(2,-5,-10)**	**13.111**	**70.84**	**2.472**	**2.1**	**4.823**	**10.22**	**60.29**	**61.15**
**Sapph**	**(1,8,-10)**	**13.711**	**81.06**	**2.427**	**2.3**	**2.184**	**5.07**	**27.3**	**27.77**
	Equiv. Refl.: (1,-9,10), (8,1,10), (8,-9,-10), (9,-1,10), (9,-8,-10), (-1,9,-10), (-1,-8,10), (-8,9,10), (-8,-1,-10) (-9,1,-10), (-9,8,10)								
**Quartz**	**(4,-1,11)**	**13.667**	**79.95**	**2.422**	**2.2**	**2.459**	**5.47**	**30.74**	**31.22**
**Quartz**	**(4,-3,-11)**	**13.667**	**79.95**	**2.421**	**2.2**	**2.459**	**5.47**	**30.74**	**31.22**
**Quartz**	**(-3,4,11)**	**13.667**	**79.95**	**2.419**	**2.2**	**2.459**	**5.46**	**30.74**	**31.22**
**Quartz**	**(-3,-1,-11)**	**13.667**	**79.95**	**2.418**	**2.2**	**2.459**	**5.46**	**30.74**	**31.22**
**Quartz**	**(-1,4,-11)**	**13.667**	**79.95**	**2.415**	**2.2**	**2.459**	**5.46**	**30.74**	**31.22**
	Equiv. Refl.: (-1,-3,11)								
**Sapph**	**(2,7,10)**	**13.207**	**72.08**	**2.305**	**2.1**	**4.488**	**9.23**	**56.1**	**56.85**
	Equiv. Refl.: (2,-9,-10), (7,2,-10), (7,-9,10), (9,-2,-10), (9,-7,10), (-2,9,10), (-2,-7,-10), (-7,9,-10), (-7,-2,10) (-9,2,10), (-9,7,-10)								
**LiNbO3**	**(1,6,-23)**	**13.745**	**82**	**2.183**	**3.2**	**1.951**	**6.26**	**24.39**	**25.18**
	Equiv. Refl.: (1,-7,23), (6,1,23), (6,-7,-23), (7,-1,23), (7,-6,-23), (-1,7,-23), (-1,-6,23), (-6,7,23), (-6,-1,-23) (-7,1,-23), (-7,6,23)								
**Quartz**	**(1,8,5)**	**13.706**	**80.92**	**1.914**	**2**	**2.218**	**4.52**	**27.73**	**28.09**
	Equiv. Refl.: (1,-9,-5), (-9,1,5), (-9,8,-5)								
**Quartz**	**(8,1,-5)**	**13.706**	**80.92**	**1.912**	**2**	**2.218**	**4.51**	**27.73**	**28.09**
	Equiv. Refl.: (8,-9,5)								
**Quartz**	**(9,-1,-5)**	**13.706**	**80.92**	**1.902**	**2**	**2.218**	**4.52**	**27.73**	**28.09**
	Equiv. Refl.: (9,-8,5), (-1,9,5), (-1,-8,-5)								
**Quartz**	**(-8,9,-5)**	**13.706**	**80.92**	**1.901**	**2**	**2.218**	**4.51**	**27.73**	**28.09**
**Quartz**	**(-8,-1,5)**	**13.706**	**80.92**	**1.9**	**2**	**2.218**	**4.51**	**27.73**	**28.09**

Ei =<br>Cryst	13.88<br>Refl<br>(h,k,l)	keV<br>EB<br>[keV]	ΘB<br>[°]	∫ IR dΘ<br>[μrad]	Width<br>[μrad]	Ei cotΘ<br>[meV/μrad]	ΔE<br>[meV]	ΔEg<br>2m, 50μm<br>[meV]	ΔEt<br>2m, 50μm<br>[meV]
**Sapph**	**(2,5,21)**	**13.738**	**81.8**	**1.84**	**2.1**	**2.001**	**4.13**	**25.02**	**25.36**
	Equiv. Refl.: (2,5,-21), (2,8,3), (2,8,-3), (2,-7,21), (2,-7,-21), (2,-10,3), (2,-10,-3), (5,2,21), (5,2,-21) (5,-7,21), (5,-7,-21), (7,-2,21), (7,-2,-21), (7,-5,21), (7,-5,-21), (8,2,3), (8,2,-3), (8,-10,3) (8,-10,-3), (10,-2,3), (10,-2,-3), (10,-8,3), (10,-8,-3), (-2,7,21), (-2,7,-21), (-2,10,3), (-2,10,-3) (-2,-5,21), (-2,-5,-21), (-2,-8,3), (-2,-8,-3), (-5,7,21), (-5,7,-21), (-5,-2,21), (-5,-2,-21), (-7,2,21) (-7,2,-21), (-7,5,21), (-7,5,-21), (-8,10,3), (-8,10,-3), (-8,-2,3), (-8,-2,-3), (-10,2,3), (-10,2,-3) (-10,8,3), (-10,8,-3)								
**Quartz**	**(-8,10,-3)**	**13.79**	**83.46**	**1.823**	**2**	**1.591**	**3.16**	**19.88**	**20.13**
	Equiv. Refl.: (-8,-2,3)								
**Quartz**	**(10,-2,-3)**	**13.79**	**83.46**	**1.822**	**2**	**1.591**	**3.16**	**19.88**	**20.13**
	Equiv. Refl.: (10,-8,3), (-2,10,3), (-2,-8,-3)								
**Quartz**	**(-3,-7,-4)**	**13.739**	**81.82**	**1.787**	**1.9**	**1.996**	**3.84**	**24.95**	**25.24**
	Equiv. Refl.: (-7,10,-4), (-7,-3,4)								
**Quartz**	**(10,-3,-4)**	**13.739**	**81.82**	**1.786**	**1.9**	**1.996**	**3.84**	**24.95**	**25.24**
	Equiv. Refl.: (10,-7,4), (-3,10,4)								
**Sapph**	**(0,1,-28)**	**13.449**	**75.68**	**1.771**	**1.9**	**3.544**	**6.64**	**44.3**	**44.79**
	Equiv. Refl.: (0,-1,28), (1,0,28), (1,-1,-28), (-1,0,-28), (-1,1,28)								
**Quartz**	**(3,7,4)**	**13.739**	**81.82**	**1.748**	**1.9**	**1.996**	**3.84**	**24.95**	**25.24**
	Equiv. Refl.: (7,3,-4), (7,-10,4), (-10,3,4), (-10,7,-4)								
**Quartz**	**(3,-10,-4)**	**13.739**	**81.82**	**1.747**	**1.9**	**1.996**	**3.84**	**24.95**	**25.24**
**Quartz**	**(8,-10,3)**	**13.79**	**83.46**	**1.726**	**2**	**1.591**	**3.16**	**19.88**	**20.13**
**Quartz**	**(2,8,3)**	**13.79**	**83.46**	**1.725**	**2**	**1.591**	**3.16**	**19.88**	**20.13**
	Equiv. Refl.: (2,-10,-3), (8,2,-3), (-10,2,3), (-10,8,-3)								
**Quartz**	**(-10,5,5)**	**13.86**	**86.93**	**1.709**	**2.2**	**0.744**	**1.64**	**9.3**	**9.45**
**Quartz**	**(5,-10,-5)**	**13.86**	**86.93**	**1.708**	**2.2**	**0.744**	**1.64**	**9.3**	**9.45**
	Equiv. Refl.: (-10,5,-5)								
**Quartz**	**(5,5,5)**	**13.86**	**86.93**	**1.707**	**2.2**	**0.744**	**1.64**	**9.3**	**9.45**
**Quartz**	**(5,5,-5)**	**13.86**	**86.93**	**1.7**	**2.2**	**0.744**	**1.64**	**9.3**	**9.45**
	Equiv. Refl.: (5,-10,5)								
**Quartz**	**(-2,10,-2)**	**13.549**	**77.46**	**1.696**	**1.8**	**3.086**	**5.46**	**38.58**	**38.96**
	Equiv. Refl.: (-2,-8,2)								
**Quartz**	**(10,-2,2)**	**13.549**	**77.46**	**1.695**	**1.8**	**3.086**	**5.46**	**38.58**	**38.96**
	Equiv. Refl.: (10,-8,-2), (-8,10,2), (-8,-2,-2)								
**Quartz**	**(9,-2,5)**	**13.233**	**72.44**	**1.689**	**1.7**	**4.392**	**7.54**	**54.9**	**55.42**
	Equiv. Refl.: (9,-7,-5)								
**Quartz**	**(-2,9,-5)**	**13.233**	**72.44**	**1.688**	**1.7**	**4.392**	**7.53**	**54.9**	**55.42**
	Equiv. Refl.: (-2,-7,5), (-7,9,5), (-7,-2,-5)								
**Quartz**	**(10,-5,-5)**	**13.86**	**86.93**	**1.687**	**2.2**	**0.744**	**1.64**	**9.3**	**9.45**
**Quartz**	**(10,-5,5)**	**13.86**	**86.93**	**1.686**	**2.2**	**0.744**	**1.64**	**9.3**	**9.45**
	Equiv. Refl.: (-5,10,5)								
**Quartz**	**(-5,-5,-5)**	**13.86**	**86.93**	**1.685**	**2.2**	**0.744**	**1.64**	**9.3**	**9.45**
**Quartz**	**(2,8,-2)**	**13.549**	**77.46**	**1.683**	**1.8**	**3.086**	**5.46**	**38.58**	**38.96**
	Equiv. Refl.: (2,-10,2), (8,2,2), (8,-10,-2)								
**Quartz**	**(-10,2,-2)**	**13.549**	**77.46**	**1.682**	**1.8**	**3.086**	**5.46**	**38.58**	**38.96**
	Equiv. Refl.: (-10,8,2)								
**Quartz**	**(-5,10,-5)**	**13.86**	**86.93**	**1.678**	**2.2**	**0.744**	**1.64**	**9.3**	**9.45**
	Equiv. Refl.: (-5,-5,5)								
**Quartz**	**(-9,2,-5)**	**13.233**	**72.44**	**1.67**	**1.7**	**4.392**	**7.54**	**54.9**	**55.42**
	Equiv. Refl.: (-9,7,5)								
**Quartz**	**(2,-9,5)**	**13.233**	**72.44**	**1.669**	**1.7**	**4.392**	**7.53**	**54.9**	**55.42**
	Equiv. Refl.: (7,2,5), (7,-9,-5)								
**Quartz**	**(2,7,-5)**	**13.233**	**72.44**	**1.668**	**1.7**	**4.392**	**7.53**	**54.9**	**55.42**

Ei =	13.88	keV						ΔEg	ΔEt
Cryst	Refl	EB	ΘB	∫ IR dΘ	Width	Ei cotΘ	ΔE	2m, 50µm	2m, 50µm
	(h,k,l)	[keV]	[°]	[µrad]	[µrad]	[meV/µrad]	[meV]	[meV]	[meV]
**LiNbO3**	**(2,7,-17)**	**13.687**	**80.43**	**1.664**	**2.5**	**2.34**	**5.89**	**29.24**	**29.83**
	Equiv. Refl.: (2,-9,17), (7,2,17), (7,-9,-17), (9,-2,17), (9,-7,-17), (-2,9,-17), (-2,-7,17), (-7,9,17), (-7,-2,-17) (-9,2,-17), (-9,7,17)								
**Quartz**	**(3,0,-11)**	**13.353**	**74.16**	**1.624**	**1.7**	**3.938**	**6.6**	**49.23**	**49.67**
	Equiv. Refl.: (3,-3,11), (-3,0,11), (-3,3,-11)								
**Quartz**	**(3,-1,-11)**	**13.193**	**71.9**	**1.622**	**1.6**	**4.537**	**7.47**	**56.72**	**57.21**
	Equiv. Refl.: (3,-2,11), (-1,3,11), (-1,-2,-11), (-2,3,-11), (-2,-1,11)								
**Quartz**	**(0,3,11)**	**13.353**	**74.16**	**1.62**	**1.7**	**3.938**	**6.59**	**49.23**	**49.67**
	Equiv. Refl.: (0,-3,-11)								
**Sapph**	**(0,2,28)**	**13.699**	**80.73**	**1.598**	**1.8**	**2.266**	**4.08**	**28.32**	**28.61**
	Equiv. Refl.: (0,-2,-28), (2,0,-28), (2,-2,28), (-2,0,28), (-2,2,-28)								
**Quartz**	**(1,2,11)**	**13.193**	**71.9**	**1.596**	**1.6**	**4.537**	**7.47**	**56.72**	**57.21**
	Equiv. Refl.: (1,-3,-11), (-3,1,11), (-3,2,-11)								
**Quartz**	**(2,1,-11)**	**13.193**	**71.9**	**1.595**	**1.6**	**4.537**	**7.47**	**56.72**	**57.21**
	Equiv. Refl.: (2,-3,11)								
**Quartz**	**(-2,8,-7)**	**13.223**	**72.3**	**1.542**	**1.6**	**4.429**	**6.93**	**55.36**	**55.79**
	Equiv. Refl.: (-2,-6,7), (-6,8,7), (-6,-2,-7)								
**Quartz**	**(8,-2,7)**	**13.223**	**72.3**	**1.541**	**1.6**	**4.429**	**6.92**	**55.36**	**55.79**
	Equiv. Refl.: (8,-6,-7)								
**Sapph**	**(4,6,-2)**	**13.15**	**71.33**	**1.527**	**1.6**	**4.689**	**7.51**	**58.61**	**59.09**
	Equiv. Refl.: (4,-10,2), (6,4,2), (6,-10,-2), (10,-4,2), (10,-6,-2), (-4,10,-2), (-4,-6,2), (-6,10,2), (-6,-4,-2) (-10,4,-2), (-10,6,2)								
**Sapph**	**(1,1,27)**	**13.148**	**71.31**	**1.52**	**1.6**	**4.697**	**7.49**	**58.71**	**59.19**
	Equiv. Refl.: (1,1,-27), (1,-2,27), (1,-2,-27), (2,-1,27), (2,-1,-27), (-1,2,27), (-1,2,-27), (-1,-1,27), (-1,-1,-27) (-2,1,27), (-2,1,-27)								
**Quartz**	**(2,6,-7)**	**13.223**	**72.3**	**1.497**	**1.6**	**4.429**	**6.92**	**55.36**	**55.79**
	Equiv. Refl.: (2,-8,7), (6,2,7), (6,-8,-7)								
**Quartz**	**(-8,2,-7)**	**13.223**	**72.3**	**1.496**	**1.6**	**4.429**	**6.92**	**55.36**	**55.79**
	Equiv. Refl.: (-8,6,7)								
**Quartz**	**(10,-3,3)**	**13.399**	**74.88**	**1.483**	**1.5**	**3.751**	**5.54**	**46.89**	**47.21**
	Equiv. Refl.: (10,-7,-3), (-7,10,3), (-7,-3,-3)								
**Quartz**	**(-3,10,-3)**	**13.399**	**74.88**	**1.482**	**1.5**	**3.751**	**5.53**	**46.89**	**47.21**
	Equiv. Refl.: (-3,-7,3)								
**Sapph**	**(2,7,13)**	**13.789**	**83.44**	**1.471**	**1.8**	**1.596**	**2.84**	**19.95**	**20.16**
	Equiv. Refl.: (2,-9,-13), (7,2,-13), (7,-9,13), (9,-2,-13), (9,-7,13), (-2,9,13), (-2,-7,-13), (-7,9,-13), (-7,-2,13) (-9,2,13), (-9,7,-13)								
**Sapph**	**(1,8,-7)**	**13.281**	**73.1**	**1.43**	**1.5**	**4.216**	**6.42**	**52.7**	**53.09**
	Equiv. Refl.: (1,-9,7), (8,1,7), (8,-9,-7), (9,-1,7), (9,-8,-7), (-1,9,-7), (-1,-8,7), (-8,9,7), (-8,-1,-7) (-9,1,-7), (-9,8,7)								
**Sapph**	**(2,6,17)**	**13.547**	**77.43**	**1.415**	**1.6**	**3.095**	**4.82**	**38.69**	**38.99**
	Equiv. Refl.: (2,-8,-17), (6,2,-17), (6,-8,17), (8,-2,-17), (8,-6,17), (-2,8,17), (-2,-6,-17), (-6,8,-17), (-6,-2,17) (-8,2,17), (-8,6,-17)								
**LiNbO3**	**(1,9,7)**	**13.629**	**79.08**	**1.389**	**2.1**	**2.677**	**5.72**	**33.47**	**33.95**
	Equiv. Refl.: (1,-10,-7), (9,1,-7), (9,-10,7), (10,-1,-7), (10,-9,7), (-1,10,7), (-1,-9,-7), (-9,10,-7), (-9,-1,7) (-10,1,7), (-10,9,-7)								
**Quartz**	**(0,3,-11)**	**13.353**	**74.16**	**1.383**	**1.4**	**3.938**	**5.7**	**49.23**	**49.56**
	Equiv. Refl.: (0,-3,11), (3,0,11), (3,-3,-11), (-3,0,-11), (-3,3,11)								
**Quartz**	**(9,0,2)**	**13.312**	**73.55**	**1.375**	**1.4**	**4.098**	**5.88**	**51.22**	**51.56**
	Equiv. Refl.: (9,-9,-2), (-9,0,-2), (-9,9,2)								

Ei =	13.88	keV						ΔEg	ΔEt
**Cryst**	**Refl**	**EB**	**ΘB**	**∫ IR dΘ**	**Width**	**Ei cotΘ**	**ΔE**	**2m, 50µm**	**2m, 50µm**
	**(h,k,l)**	**[keV]**	**[°]**	**[µrad]**	**[µrad]**	**[meV/µrad]**	**[meV]**	**[meV]**	**[meV]**
**Quartz**	**(0,9,-2)**	**13.312**	**73.55**	**1.374**	**1.4**	**4.098**	**5.87**	**51.22**	**51.56**
	Equiv. Refl.: (0,-9,2)								
**Quartz**	**(3,7,-3)**	**13.399**	**74.88**	**1.337**	**1.5**	**3.751**	**5.53**	**46.89**	**47.21**
	Equiv. Refl.: (3,-10,3), (7,3,3), (7,-10,-3), (-10,3,-3), (-10,7,3)								
**Sapph**	**(1,8,5)**	**13.073**	**70.37**	**1.292**	**1.4**	**4.95**	**6.8**	**61.88**	**62.25**
	Equiv. Refl.: (1,-9,-5), (8,1,-5), (8,-9,5), (9,-1,-5), (9,-8,5), (-1,9,5), (-1,-8,-5), (-8,9,-5), (-8,-1,5) (-9,1,5), (-9,8,-5)								
**Quartz**	**(3,5,-8)**	**13.719**	**81.27**	**1.286**	**1.4**	**2.131**	**3.04**	**26.63**	**26.81**
	Equiv. Refl.: (3,-8,8), (-8,3,-8), (-10,3,-2), (-10,7,2)								
**Quartz**	**(3,7,-2)**	**13.152**	**71.36**	**1.285**	**1.3**	**4.683**	**6.21**	**58.54**	**58.87**
	Equiv. Refl.: (3,-10,2), (5,3,8), (5,-8,-8), (7,3,2), (7,-10,-2), (-8,5,8)								
**Quartz**	**(10,-3,2)**	**13.152**	**71.36**	**1.262**	**1.3**	**4.683**	**6.21**	**58.54**	**58.87**
	Equiv. Refl.: (10,-7,-2), (-3,10,-2), (-3,-7,2)								
**Quartz**	**(-7,10,2)**	**13.152**	**71.36**	**1.261**	**1.3**	**4.683**	**6.21**	**58.54**	**58.87**
	Equiv. Refl.: (-7,-3,-2)								
**Quartz**	**(3,6,6)**	**13.457**	**75.82**	**1.26**	**1.3**	**3.506**	**4.71**	**43.82**	**44.08**
	Equiv. Refl.: (3,-9,-6)								
**Quartz**	**(6,3,-6)**	**13.457**	**75.82**	**1.259**	**1.3**	**3.506**	**4.7**	**43.82**	**44.08**
	Equiv. Refl.: (6,-9,6), (-9,3,6), (-9,6,-6)								
**Quartz**	**(9,-3,-6)**	**13.457**	**75.82**	**1.258**	**1.3**	**3.506**	**4.71**	**43.82**	**44.08**
	Equiv. Refl.: (9,-6,6), (-3,9,6), (-3,-6,-6)								
**Quartz**	**(-6,9,-6)**	**13.457**	**75.82**	**1.257**	**1.3**	**3.506**	**4.7**	**43.82**	**44.08**
	Equiv. Refl.: (-6,-3,6)								
**Quartz**	**(8,-3,8)**	**13.719**	**81.27**	**1.243**	**1.4**	**2.131**	**3.04**	**26.63**	**26.81**
	Equiv. Refl.: (8,-5,-8), (-3,8,-8), (-3,-5,8)								
**Quartz**	**(-5,8,8)**	**13.719**	**81.27**	**1.242**	**1.4**	**2.131**	**3.04**	**26.63**	**26.81**
	Equiv. Refl.: (-5,-3,-8)								
**Quartz**	**(0,9,1)**	**13.163**	**71.5**	**1.24**	**1.3**	**4.643**	**6.01**	**58.04**	**58.35**
	Equiv. Refl.: (0,-9,-1), (9,0,-1), (9,-9,1), (-9,0,1), (-9,9,-1)								
**Quartz**	**(9,-1,5)**	**13.706**	**80.92**	**1.225**	**1.4**	**2.218**	**3.02**	**27.73**	**27.89**
	Equiv. Refl.: (9,-8,-5)								
**Quartz**	**(-1,9,-5)**	**13.706**	**80.92**	**1.224**	**1.4**	**2.218**	**3.01**	**27.73**	**27.89**
	Equiv. Refl.: (-1,-8,5), (-8,9,5), (-8,-1,-5)								
**Quartz**	**(1,8,-4)**	**13.267**	**72.91**	**1.187**	**1.2**	**4.268**	**5.2**	**53.34**	**53.6**
	Equiv. Refl.: (8,1,4), (8,-9,-4), (-9,1,-4), (-9,8,4)								
**Quartz**	**(1,-9,4)**	**13.267**	**72.91**	**1.186**	**1.2**	**4.268**	**5.2**	**53.34**	**53.6**
**Quartz**	**(-9,8,5)**	**13.706**	**80.92**	**1.18**	**1.4**	**2.218**	**3.02**	**27.73**	**27.89**
**Quartz**	**(1,8,-5)**	**13.706**	**80.92**	**1.179**	**1.4**	**2.218**	**3.02**	**27.73**	**27.89**
	Equiv. Refl.: (1,-9,5), (8,1,5), (8,-9,-5), (-9,1,-5)								
**Sapph**	**(3,6,12)**	**13.244**	**72.58**	**1.176**	**1.3**	**4.355**	**5.56**	**54.43**	**54.72**
	Equiv. Refl.: (3,6,-12), (3,-9,12), (3,-9,-12), (6,3,12), (6,3,-12), (6,-9,12), (6,-9,-12), (9,-3,12), (9,-3,-12) (9,-6,12), (9,-6,-12), (-3,9,12), (-3,9,-12), (-3,-6,12), (-3,-6,-12), (-6,9,12), (-6,9,-12), (-6,-3,12) (-6,-3,-12), (-9,3,12), (-9,3,-12), (-9,6,12), (-9,6,-12)								
**Quartz**	**(-4,7,9)**	**13.606**	**78.59**	**1.142**	**1.2**	**2.802**	**3.44**	**35.02**	**35.19**
**Quartz**	**(9,-1,-4)**	**13.267**	**72.91**	**1.141**	**1.2**	**4.268**	**5.1**	**53.34**	**53.59**
	Equiv. Refl.: (9,-8,4), (-1,9,4), (-1,-8,-4), (-4,-3,-9), (-8,9,-4), (-8,-1,4)								
**LiNbO3**	**(3,8,-5)**	**13.876**	**88.63**	**1.14**	**4.4**	**0.332**	**1.47**	**4.15**	**4.4**
	Equiv. Refl.: (3,-11,5), (8,3,5), (8,-11,-5), (11,-3,5), (11,-8,-5), (-3,11,-5), (-3,-8,5), (-8,11,5), (-8,-3,-5) (-11,3,-5), (-11,8,5), (-3,7,-9), (-3,-4,9)								
**Quartz**	**(7,-3,9)**	**13.606**	**78.59**	**1.139**	**1.2**	**2.802**	**3.43**	**35.02**	**35.19**
	Equiv. Refl.: (7,-4,-9)								

Ei =	13.88	keV						ΔEg	ΔEt
Cryst	Refl	EB	ΘB	∫ IR dΘ	Width	Ei cotΘ	ΔE	2m, 50µm	2m, 50µm
	(h,k,l)	[keV]	[°]	[µrad]	[µrad]	[meV/µrad]	[meV]	[meV]	[meV]
**Quartz**	**(-8,9,4)**	**13.267**	**72.91**	**1.121**	**1.2**	**4.268**	**5.21**	**53.34**	**53.6**
	Equiv. Refl.: (-8,-1,-4)								
**Quartz**	**(9,-1,4)**	**13.267**	**72.91**	**1.12**	**1.2**	**4.268**	**5.2**	**53.34**	**53.6**
	Equiv. Refl.: (9,-8,-4), (-1,9,-4), (-1,-8,4)								
**Quartz**	**(1,8,4)**	**13.267**	**72.91**	**1.114**	**1.2**	**4.268**	**5.1**	**53.34**	**53.59**
	Equiv. Refl.: (1,-9,-4), (8,1,-4), (8,-9,4), (-9,1,4), (-9,8,-4)								
**Sapph**	**(4,5,-13)**	**13.288**	**73.2**	**1.112**	**1.2**	**4.191**	**5.12**	**52.38**	**52.63**
	Equiv. Refl.: (4,-9,13), (5,4,13), (5,-9,-13), (9,-4,13), (9,-5,-13), (-4,9,-13), (-4,-5,13), (-5,9,13), (-5,-4,-13) (-9,4,-13), (-9,5,13)								
**LiNbO3**	**(1,9,-5)**	**13.452**	**75.73**	**1.103**	**1.7**	**3.531**	**5.95**	**44.14**	**44.54**
	Equiv. Refl.: (1,-10,5), (9,1,5), (9,-10,-5), (10,-1,5), (10,-9,-5), (-1,10,-5), (-1,-9,5), (-9,10,5), (-9,-1,-5) (-10,1,-5), (-10,9,5)								
**Quartz**	**(7,3,4)**	**13.739**	**81.82**	**1.096**	**1.2**	**1.996**	**2.46**	**24.95**	**25.07**
	Equiv. Refl.: (7,-10,-4), (-10,3,-4), (-10,7,4)								
**Quartz**	**(3,7,-4)**	**13.739**	**81.82**	**1.095**	**1.2**	**1.996**	**2.46**	**24.95**	**25.07**
	Equiv. Refl.: (3,-10,4)								
**Quartz**	**(0,8,6)**	**13.536**	**77.22**	**1.091**	**1.2**	**3.149**	**3.77**	**39.36**	**39.54**
	Equiv. Refl.: (0,-8,-6)								
**Quartz**	**(8,0,-6)**	**13.536**	**77.22**	**1.089**	**1.2**	**3.149**	**3.76**	**39.36**	**39.54**
	Equiv. Refl.: (8,-8,6), (-8,0,6), (-8,8,-6)								
**LiNbO3**	**(4,5,-19)**	**13.789**	**83.43**	**1.08**	**2.1**	**1.6**	**3.44**	**20**	**20.29**
	Equiv. Refl.: (4,-9,19), (5,4,19), (5,-9,-19), (9,-4,19), (9,-5,-19), (-4,9,-19), (-4,-5,19), (-5,9,19), (-5,-4,-19) (-9,4,-19), (-9,5,19)								
**Quartz**	**(4,3,9)**	**13.606**	**78.59**	**1.068**	**1.2**	**2.802**	**3.44**	**35.02**	**35.19**
	Equiv. Refl.: (4,-7,-9)								
**Quartz**	**(3,4,-9)**	**13.606**	**78.59**	**1.066**	**1.2**	**2.802**	**3.43**	**35.02**	**35.19**
	Equiv. Refl.: (3,-7,9)								
**Quartz**	**(-7,3,-9)**	**13.606**	**78.59**	**1.065**	**1.2**	**2.802**	**3.43**	**35.02**	**35.19**
	Equiv. Refl.: (-7,4,9)								
**Quartz**	**(-7,9,-6)**	**13.769**	**82.76**	**1.044**	**1.2**	**1.764**	**2.11**	**22.05**	**22.15**
**Quartz**	**(9,-2,-6)**	**13.769**	**82.76**	**1.043**	**1.2**	**1.764**	**2.11**	**22.05**	**22.15**
	Equiv. Refl.: (9,-7,6), (-7,-2,6)								
**Quartz**	**(-2,9,6)**	**13.769**	**82.76**	**1.041**	**1.2**	**1.764**	**2.1**	**22.05**	**22.15**
	Equiv. Refl.: (-2,-7,-6)								
**Quartz**	**(-10,2,-3)**	**13.79**	**83.46**	**1.011**	**1.2**	**1.591**	**1.91**	**19.88**	**19.98**
**Quartz**	**(-10,8,3)**	**13.79**	**83.46**	**1.01**	**1.2**	**1.591**	**1.91**	**19.88**	**19.98**
**Quartz**	**(2,8,-3)**	**13.79**	**83.46**	**1.009**	**1.2**	**1.591**	**1.9**	**19.88**	**19.98**
	Equiv. Refl.: (2,-10,3)								
**Quartz**	**(8,2,3)**	**13.79**	**83.46**	**1.008**	**1.2**	**1.591**	**1.9**	**19.88**	**19.97**
	Equiv. Refl.: (8,-10,-3)								
**Quartz**	**(10,-3,4)**	**13.739**	**81.82**	**1.004**	**1.2**	**1.996**	**2.46**	**24.95**	**25.07**
	Equiv. Refl.: (10,-7,-4), (-7,10,4), (-7,-3,-4)								
**Quartz**	**(-3,10,-4)**	**13.739**	**81.82**	**1.003**	**1.2**	**1.996**	**2.46**	**24.95**	**25.07**
	Equiv. Refl.: (-3,-7,4)								
**LiNbO3**	**(2,4,25)**	**13.383**	**74.63**	**0.977**	**1.5**	**3.816**	**5.79**	**47.7**	**48.05**
	Equiv. Refl.: (2,-6,-25), (4,2,-25), (4,-6,25), (6,-2,-25), (6,-4,25), (-2,6,25), (-2,-4,-25), (-4,6,-25), (-4,-2,25) (-6,2,25), (-6,4,-25)								
**LiNbO3**	**(4,6,13)**	**13.444**	**75.6**	**0.969**	**1.5**	**3.564**	**5.48**	**44.55**	**44.89**
	Equiv. Refl.: (4,-10,-13), (6,4,-13), (6,-10,13), (10,-4,-13), (10,-6,13), (-4,10,13), (-4,-6,-13), (-6,10,-13), (-6,-4,13) (-10,4,13), (-10,6,-13)								

Ei =	13.88	keV						ΔEg	ΔEt
Cryst	Refl	EB	ΘB	∫ IR dΘ	Width	Ei cotΘ	ΔE	2m, 50μm	2m, 50μm
	(h,k,l)	[keV]	[°]	[μrad]	[μrad]	[meV/μrad]	[meV]	[meV]	[meV]
Quartz	(6,2,-7)	13.223	72.3	0.967	1	4.429	4.47	55.36	55.54
	Equiv. Refl.: (6,-8,7), (-8,2,7), (-8,6,-7)								
Quartz	(2,-8,-7)	13.223	72.3	0.966	1	4.429	4.46	55.36	55.54
Quartz	(2,6,7)	13.223	72.3	0.965	1	4.429	4.46	55.36	55.54
LiNbO3	(1,9,1)	13.272	72.98	0.96	1.5	4.249	6.2	53.12	53.48
	Equiv. Refl.: (1,-10,-1), (9,1,-1), (9,-10,1), (10,-1,-1), (10,-9,1), (-1,10,1), (-1,-9,-1), (-9,10,-1), (-9,-1,1) (-10,1,1), (-10,9,-1)								
Quartz	(2,8,0)	13.353	74.17	0.949	1	3.936	4.05	49.2	49.37
	Equiv. Refl.: (2,-10,0), (8,2,0), (8,-10,0), (-10,2,0), (-10,8,0)								
Quartz	(6,0,-9)	13.527	77.06	0.944	1.1	3.19	3.36	39.88	40.02
	Equiv. Refl.: (6,-6,9), (-6,6,-9)								
Quartz	(0,6,9)	13.527	77.06	0.943	1.1	3.19	3.36	39.88	40.02
	Equiv. Refl.: (0,-6,-9), (-6,0,9)								
Quartz	(10,-2,0)	13.353	74.17	0.94	1	3.936	4.05	49.2	49.37
	Equiv. Refl.: (10,-8,0), (-2,10,0), (-2,-8,0), (-8,10,0), (-8,-2,0)								
Quartz	(5,-6,-9)	13.129	71.07	0.938	1	4.761	4.62	59.51	59.69
	Equiv. Refl.: (-6,1,-9), (-6,5,9)								
Quartz	(1,5,-9)	13.129	71.07	0.937	1	4.761	4.61	59.51	59.69
	Equiv. Refl.: (1,-6,9), (5,1,9)								
Sapph	(1,7,15)	13.426	75.31	0.936	1.1	3.639	3.9	45.49	45.65
	Equiv. Refl.: (1,7,-15), (1,-8,15), (1,-8,-15), (7,1,15), (7,1,-15), (7,-8,15), (7,-8,-15), (8,-1,15), (8,-1,-15) (8,-7,15), (8,-7,-15), (-1,8,15), (-1,8,-15), (-1,-7,15), (-1,-7,-15), (-7,8,15), (-7,8,-15), (-7,-1,15) (-7,-1,-15), (-8,1,15), (-8,1,-15), (-8,7,15), (-8,7,-15)								
Quartz	(7,-9,6)	13.769	82.76	0.922	1.2	1.764	2.11	22.05	22.15
Quartz	(7,2,-6)	13.769	82.76	0.921	1.2	1.764	2.11	22.05	22.15
	Equiv. Refl.: (-9,2,6), (-9,7,-6)								
Quartz	(2,7,6)	13.769	82.76	0.919	1.2	1.764	2.1	22.05	22.15
	Equiv. Refl.: (2,-9,-6)								
Quartz	(10,-2,3)	13.79	83.46	0.91	1.2	1.591	1.91	19.88	19.98
Quartz	(10,-8,-3)	13.79	83.46	0.909	1.2	1.591	1.91	19.88	19.98
Quartz	(-2,10,-3)	13.79	83.46	0.908	1.2	1.591	1.9	19.88	19.98
	Equiv. Refl.: (-2,-8,3)								
Quartz	(-8,10,3)	13.79	83.46	0.907	1.2	1.591	1.9	19.88	19.97
	Equiv. Refl.: (-8,-2,-3)								
Quartz	(-7,8,-7)	13.619	78.86	0.906	1	2.732	2.75	34.15	34.27
	Equiv. Refl.: (-7,-1,7)								
Quartz	(8,-2,-7)	13.223	72.3	0.905	1	4.429	4.47	55.36	55.54
	Equiv. Refl.: (8,-6,7), (-6,8,-7)								
Quartz	(8,-1,-7)	13.619	78.86	0.904	1	2.732	2.74	34.15	34.26
	Equiv. Refl.: (8,-7,7), (-1,8,7), (-1,-7,-7), (-6,-2,7)								
Quartz	(-2,8,7)	13.223	72.3	0.903	1	4.429	4.46	55.36	55.54
	Equiv. Refl.: (-2,-6,-7)								
Sapph	(2,4,-23)	13.56	77.68	0.877	1	3.032	3.16	37.9	38.03
	Equiv. Refl.: (2,-6,23), (4,2,23), (4,-6,-23), (6,-2,23), (6,-4,-23), (-2,6,-23), (-2,-4,23), (-4,6,23), (-4,-2,-23) (-6,2,-23), (-6,4,23)								
Quartz	(10,-4,4)	13.505	76.65	0.872	0.9	3.294	3.11	41.17	41.29
	Equiv. Refl.: (10,-6,-4), (-4,10,-4), (-4,-6,4), (-6,10,4), (-6,-4,-4)								
Quartz	(6,-1,9)	13.129	71.07	0.867	1	4.761	4.62	59.51	59.69
	Equiv. Refl.: (6,-5,-9), (-5,6,9), (-5,-1,-9)								

Ei =	13.88	keV						ΔEg	ΔEt
**Cryst**	**Refl**	**EB**	**ΘB**	**∫ IR dΘ**	**Width**	**Ei cotΘ**	**ΔE**	**2m, 50μm**	**2m, 50μm**
	**(h,k,l)**	**[keV]**	**[°]**	**[μrad]**	**[μrad]**	**[meV/μrad]**	**[meV]**	**[meV]**	**[meV]**
**Quartz**	**(-1,6,-9)**	**13.129**	**71.07**	**0.866**	**1**	**4.761**	**4.61**	**59.51**	**59.69**
	Equiv. Refl.: (-1,-5,9)								
**Quartz**	**(-1,7,8)**	**13.247**	**72.63**	**0.862**	**0.9**	**4.342**	**3.93**	**54.27**	**54.42**
	Equiv. Refl.: (-1,-6,-8), (-6,7,-8)								
**Quartz**	**(7,-1,-8)**	**13.247**	**72.63**	**0.861**	**0.9**	**4.342**	**3.93**	**54.27**	**54.42**
	Equiv. Refl.: (7,-6,8), (-6,-1,8)								
**Quartz**	**(10,-4,3)**	**13.16**	**71.46**	**0.854**	**0.9**	**4.655**	**4.23**	**58.19**	**58.34**
	Equiv. Refl.: (10,-6,-3), (-4,10,-3), (-4,-6,3), (-6,10,3), (-6,-4,-3)								
**Quartz**	**(7,-1,8)**	**13.247**	**72.63**	**0.844**	**0.9**	**4.342**	**3.95**	**54.27**	**54.42**
	Equiv. Refl.: (7,-6,-8), (-1,7,-8), (-1,-6,8), (-6,7,8), (-6,-1,-8)								
**Quartz**	**(7,1,-7)**	**13.619**	**78.86**	**0.834**	**1**	**2.732**	**2.74**	**34.15**	**34.26**
	Equiv. Refl.: (7,-8,7)								
**Quartz**	**(1,-8,-7)**	**13.619**	**78.86**	**0.833**	**1**	**2.732**	**2.74**	**34.15**	**34.26**
**Quartz**	**(1,7,7)**	**13.619**	**78.86**	**0.832**	**1**	**2.732**	**2.74**	**34.15**	**34.26**
	Equiv. Refl.: (-8,1,7), (-8,7,-7)								
**Quartz**	**(-3,9,-6)**	**13.457**	**75.82**	**0.83**	**0.9**	**3.506**	**3.12**	**43.82**	**43.93**
**Quartz**	**(9,-3,6)**	**13.457**	**75.82**	**0.829**	**0.9**	**3.506**	**3.12**	**43.82**	**43.93**
	Equiv. Refl.: (9,-6,-6), (-3,-6,6), (-6,9,6), (-6,-3,-6)								
**Quartz**	**(8,-4,8)**	**13.642**	**79.37**	**0.824**	**0.9**	**2.606**	**2.36**	**32.57**	**32.65**
	Equiv. Refl.: (8,-4,-8)								
**Quartz**	**(-4,8,8)**	**13.642**	**79.37**	**0.821**	**0.9**	**2.606**	**2.35**	**32.57**	**32.65**
	Equiv. Refl.: (-4,8,-8), (-4,-4,8), (-4,-4,-8)								
**Quartz**	**(4,6,-3)**	**13.16**	**71.46**	**0.819**	**0.9**	**4.655**	**4.23**	**58.19**	**58.34**
	Equiv. Refl.: (4,-10,3), (-10,4,-3), (-10,6,3)								
**Quartz**	**(1,6,-8)**	**13.247**	**72.63**	**0.818**	**0.9**	**4.342**	**3.95**	**54.27**	**54.42**
	Equiv. Refl.: (1,-7,8), (6,1,8), (6,4,3), (6,-7,-8), (6,-10,-3), (-7,1,-8), (-7,6,8)								
**Quartz**	**(9,0,1)**	**13.163**	**71.5**	**0.804**	**0.9**	**4.643**	**4.08**	**58.04**	**58.19**
	Equiv. Refl.: (9,-9,-1), (-9,0,-1), (-9,9,1)								
**Quartz**	**(0,9,-1)**	**13.163**	**71.5**	**0.803**	**0.9**	**4.643**	**4.08**	**58.04**	**58.19**
	Equiv. Refl.: (0,-9,1)								
**Sapph**	**(1,3,-26)**	**13.543**	**77.35**	**0.801**	**1**	**3.114**	**3**	**38.93**	**39.04**
	Equiv. Refl.: (1,-4,26), (3,1,26), (3,-4,-26), (4,-1,26), (4,-3,-26), (-1,4,-26), (-1,-3,26), (-3,4,26), (-3,-1,-26) (-4,1,-26), (-4,3,26)								
**Quartz**	**(6,4,4)**	**13.505**	**76.65**	**0.8**	**0.9**	**3.294**	**3.11**	**41.17**	**41.29**
	Equiv. Refl.: (6,-10,-4)								
**Quartz**	**(4,6,-4)**	**13.505**	**76.65**	**0.799**	**0.9**	**3.294**	**3.11**	**41.17**	**41.29**
	Equiv. Refl.: (4,-10,4), (-10,4,-4), (-10,6,4)								
**Quartz**	**(1,6,8)**	**13.247**	**72.63**	**0.791**	**0.9**	**4.342**	**3.93**	**54.27**	**54.42**
	Equiv. Refl.: (1,-7,-8), (6,-7,8)								
**Quartz**	**(6,1,-8)**	**13.247**	**72.63**	**0.79**	**0.9**	**4.342**	**3.93**	**54.27**	**54.42**
	Equiv. Refl.: (-7,1,8), (-7,6,-8)								
**LiNbO3**	**(4,6,-11)**	**13.082**	**70.48**	**0.773**	**1.2**	**4.921**	**5.96**	**61.52**	**61.81**
	Equiv. Refl.: (4,-10,11), (6,4,11), (6,-10,-11), (10,-4,11), (10,-6,-11), (-4,10,-11), (-4,-6,11), (-6,10,11), (-6,-4,-11) (-10,4,-11), (-10,6,11), (4,5,-6), (4,-9,6), (-9,4,-6), (-9,5,6)								
**Quartz**	**(5,4,6)**	**13.299**	**73.36**	**0.772**	**0.8**	**4.149**	**3.5**	**51.86**	**51.98**
	Equiv. Refl.: (5,-9,-6)								
**Sapph**	**(4,4,18)**	**13.507**	**76.69**	**0.757**	**0.9**	**3.283**	**3**	**41.04**	**41.15**
	Equiv. Refl.: (4,4,-18), (4,-8,18), (4,-8,-18), (8,-4,18), (8,-4,-18), (-4,8,18), (-4,8,-18), (-4,-4,18), (-4,-4,-18) (-8,4,18), (-8,4,-18)								
**Quartz**	**(3,6,-6)**	**13.457**	**75.82**	**0.745**	**0.9**	**3.506**	**3.12**	**43.82**	**43.93**
	Equiv. Refl.: (3,-9,6), (-9,3,-6), (-9,6,6)								

Ei = Cryst	13.88 Refl (h,k,l)	keV EB [keV]	ΘB [°]	∫ IR dΘ [µrad]	Width [µrad]	Ei cotΘ [meV/µrad]	ΔE [meV]	ΔEg 2m, 50µm [meV]	ΔEt 2m, 50µm [meV]
**Quartz**	**(6,3,6)**	**13.457**	**75.82**	**0.744**	**0.9**	**3.506**	**3.12**	**43.82**	**43.93**
	Equiv. Refl.: (6,-9,-6)								
**Quartz**	**(9,-4,6)**	**13.299**	**73.36**	**0.743**	**0.8**	**4.149**	**3.51**	**51.86**	**51.98**
	Equiv. Refl.: (9,-5,-6), (-4,9,-6), (-4,-5,6)								
**Quartz**	**(-5,9,6)**	**13.299**	**73.36**	**0.742**	**0.8**	**4.149**	**3.5**	**51.86**	**51.98**
	Equiv. Refl.: (-5,-4,-6)								
**LiNbO3**	**(4,7,3)**	**13.476**	**76.15**	**0.728**	**1.3**	**3.423**	**4.41**	**42.78**	**43.01**
	Equiv. Refl.: (4,7,-3), (4,-11,3), (4,-11,-3), (7,4,3), (7,4,-3), (7,-11,3), (7,-11,-3), (11,-4,3), (11,-4,-3) (11,-7,3), (11,-7,-3), (-4,11,3), (-4,11,-3), (-4,-7,3), (-4,-7,-3), (-7,11,3), (-7,11,-3), (-7,-4,3) (-7,-4,-3), (-11,4,3), (-11,4,-3), (-11,7,3), (-11,7,-3)								
**Sapph**	**(2,3,-25)**	**13.615**	**78.8**	**0.71**	**0.9**	**2.749**	**2.47**	**34.36**	**34.45**
	Equiv. Refl.: (2,-5,25), (3,2,25), (3,-5,-25), (5,-2,25), (5,-3,-25), (-2,5,-25), (-2,-3,25), (-3,5,25), (-3,-2,-25) (-5,2,-25), (-5,3,25)								
**Quartz**	**(-8,4,8)**	**13.642**	**79.37**	**0.701**	**0.9**	**2.606**	**2.36**	**32.57**	**32.66**
	Equiv. Refl.: (-8,4,-8)								
**Quartz**	**(4,4,8)**	**13.642**	**79.37**	**0.698**	**0.9**	**2.606**	**2.35**	**32.57**	**32.65**
	Equiv. Refl.: (4,4,-8), (4,-8,8), (4,-8,-8)								
**LiNbO3**	**(1,4,27)**	**13.652**	**79.6**	**0.694**	**1.4**	**2.547**	**3.52**	**31.84**	**32.03**
	Equiv. Refl.: (1,4,-27), (1,-5,27), (1,-5,-27), (4,1,27), (4,1,-27), (4,-5,27), (4,-5,-27), (5,-1,27), (5,-1,-27) (5,-4,27), (5,-4,-27), (-1,5,27), (-1,5,-27), (-1,-4,27), (-1,-4,-27), (-4,5,27), (-4,5,-27), (-4,-1,27) (-4,-1,-27), (-5,1,27), (-5,1,-27), (-5,4,27), (-5,4,-27), (2,7,-11), (2,-9,11), (7,2,11), (7,-9,-11) (9,-2,11), (9,-7,-11), (-2,9,-11), (-2,-7,11), (-7,9,11), (-7,-2,-11), (-9,2,-11), (-9,7,11)								
**Sapph**	**(5,5,9)**	**13.719**	**81.25**	**0.688**	**0.9**	**2.136**	**1.98**	**26.7**	**26.77**
	Equiv. Refl.: (5,5,-9), (5,-10,9), (5,-10,-9), (10,-5,9), (10,-5,-9), (-5,10,9), (-5,10,-9), (-5,-5,9), (-5,-5,-9) (-10,5,9), (-10,5,-9)								
**Sapph**	**(4,6,4)**	**13.253**	**72.72**	**0.657**	**0.8**	**4.318**	**3.36**	**53.98**	**54.08**
	Equiv. Refl.: (4,-10,-4), (6,4,-4), (6,-10,4), (10,-4,-4), (10,-6,4), (-4,10,4), (-4,-6,-4), (-6,10,-4), (-6,-4,4) (-10,4,4), (-10,6,-4)								
**Quartz**	**(10,-5,3)**	**13.079**	**70.44**	**0.612**	**0.7**	**4.933**	**3.38**	**61.66**	**61.75**
	Equiv. Refl.: (10,-5,-3), (-5,10,3), (-5,10,-3), (-5,-5,3), (-5,-5,-3)								
**Quartz**	**(5,5,3)**	**13.079**	**70.44**	**0.606**	**0.7**	**4.933**	**3.38**	**61.66**	**61.75**
	Equiv. Refl.: (5,-10,-3), (-10,5,3), (-10,5,-3)								
**Quartz**	**(5,5,-3)**	**13.079**	**70.44**	**0.605**	**0.7**	**4.933**	**3.38**	**61.66**	**61.75**
	Equiv. Refl.: (5,-10,3)								
**Quartz**	**(-2,6,-10)**	**13.82**	**84.69**	**0.58**	**0.7**	**1.29**	**0.9**	**16.13**	**16.15**
**Quartz**	**(-2,-4,10)**	**13.82**	**84.69**	**0.579**	**0.7**	**1.29**	**0.9**	**16.13**	**16.15**
**Quartz**	**(1,5,9)**	**13.129**	**71.07**	**0.578**	**0.6**	**4.761**	**2.9**	**59.51**	**59.58**
	Equiv. Refl.: (1,-6,-9)								
**Quartz**	**(5,1,-9)**	**13.129**	**71.07**	**0.577**	**0.6**	**4.761**	**2.9**	**59.51**	**59.58**
	Equiv. Refl.: (5,-6,9)								
**Quartz**	**(-4,-2,-10)**	**13.82**	**84.69**	**0.575**	**0.7**	**1.29**	**0.9**	**16.13**	**16.15**
	Equiv. Refl.: (-6,1,9), (-6,5,-9)								
**Quartz**	**(2,1,11)**	**13.193**	**71.9**	**0.574**	**0.6**	**4.537**	**2.82**	**56.72**	**56.79**
	Equiv. Refl.: (2,-3,-11), (6,-4,-10), (-4,6,10)								
**Quartz**	**(6,-2,10)**	**13.82**	**84.69**	**0.573**	**0.7**	**1.29**	**0.89**	**16.13**	**16.15**
**Quartz**	**(1,2,-11)**	**13.193**	**71.9**	**0.572**	**0.6**	**4.537**	**2.81**	**56.72**	**56.79**
	Equiv. Refl.: (1,-3,11), (-3,1,-11), (-3,2,11)								
**Quartz**	**(10,-3,-2)**	**13.152**	**71.36**	**0.559**	**0.6**	**4.683**	**2.98**	**58.54**	**58.61**
	Equiv. Refl.: (10,-7,2), (-7,10,-2), (-7,-3,2)								

Ei =	13.88	keV						ΔEg	ΔEt
Cryst	Refl	EB	ΘB	∫ IR dΘ	Width	Ei cotΘ	ΔE	2m, 50µm	2m, 50µm
	(h,k,l)	[keV]	[°]	[µrad]	[µrad]	[meV/µrad]	[meV]	[meV]	[meV]
Quartz	(-3,10,2)	13.152	71.36	0.558	0.6	4.683	2.98	58.54	58.61
	Equiv. Refl.: (-3,-7,-2)								
LiNbO3	(3,4,23)	13.316	73.61	0.557	1	4.082	4.19	51.02	51.19
	Equiv. Refl.: (3,-7,-23), (4,3,-23), (4,-7,23), (7,-3,-23), (7,-4,23), (-3,7,23), (-3,-4,-23), (-4,7,-23), (-4,-3,23) (-7,3,23), (-7,4,-23)								
Sapph	(3,5,-17)	13.294	73.3	0.551	0.7	4.165	2.83	52.07	52.14
	Equiv. Refl.: (3,-8,17), (5,3,17), (5,-8,-17), (8,-3,17), (8,-5,-17), (-3,8,-17), (-3,-5,17), (-5,8,17), (-5,-3,-17) (-8,3,-17), (-8,5,17)								
Quartz	(-10,3,2)	13.152	71.36	0.546	0.6	4.683	2.98	58.54	58.61
	Equiv. Refl.: (-10,7,-2)								
Quartz	(3,7,2)	13.152	71.36	0.545	0.6	4.683	2.98	58.54	58.61
	Equiv. Refl.: (3,-10,-2), (7,3,-2), (7,-10,2), (-4,9,6), (-4,-5,-6)								
Quartz	(9,-4,-6)	13.299	73.36	0.543	0.6	4.149	2.56	51.86	51.92
	Equiv. Refl.: (9,-5,6), (-5,9,-6)								
Quartz	(-5,-4,6)	13.299	73.36	0.542	0.6	4.149	2.56	51.86	51.92
LiNbO3	(3,7,-13)	13.658	79.74	0.534	1.2	2.513	2.98	31.42	31.56
	Equiv. Refl.: (3,-10,13), (7,3,13), (7,-10,-13), (10,-3,13), (10,-7,-13), (-3,10,-13), (-3,-7,13), (-7,10,13), (-7,-3,-13) (-10,3,-13), (-10,7,13)								
Quartz	(5,5,4)	13.426	75.31	0.529	0.6	3.639	2.31	45.49	45.55
	Equiv. Refl.: (5,-10,-4)								
Quartz	(5,5,-4)	13.426	75.31	0.528	0.6	3.639	2.31	45.49	45.55
Quartz	(5,-10,4)	13.426	75.31	0.527	0.6	3.639	2.31	45.49	45.55
	Equiv. Refl.: (-10,5,4), (-10,5,-4)								
Quartz	(-5,10,4)	13.426	75.31	0.525	0.6	3.639	2.31	45.49	45.55
	Equiv. Refl.: (-5,-5,-4)								
Quartz	(10,-5,4)	13.426	75.31	0.523	0.6	3.639	2.31	45.49	45.55
	Equiv. Refl.: (10,-5,-4), (-5,10,-4), (-5,-5,4)								
LiNbO3	(1,5,-25)	13.598	78.44	0.515	1.1	2.839	3.14	35.49	35.63
	Equiv. Refl.: (1,-6,25), (5,1,25), (5,-6,-25), (6,-1,25), (6,-5,-25), (-1,6,-25), (-1,-5,25), (-5,6,25), (-5,-1,-25) (-6,1,-25), (-6,5,25)								
Quartz	(4,5,6)	13.299	73.36	0.504	0.6	4.149	2.57	51.86	51.92
	Equiv. Refl.: (4,-9,-6)								
Quartz	(5,-9,6)	13.299	73.36	0.502	0.6	4.149	2.56	51.86	51.92
	Equiv. Refl.: (-9,4,6), (-9,5,-6)								
Quartz	(5,4,-6)	13.299	73.36	0.501	0.6	4.149	2.56	51.86	51.92
Quartz	(-2,3,11)	13.193	71.9	0.497	0.6	4.537	2.82	56.72	56.79
	Equiv. Refl.: (-2,-1,-11)								
Quartz	(3,-1,11)	13.193	71.9	0.496	0.6	4.537	2.81	56.72	56.79
Quartz	(3,-2,-11)	13.193	71.9	0.495	0.6	4.537	2.81	56.72	56.79
	Equiv. Refl.: (-1,3,-11), (-1,-2,11)								
Sapph	(0,4,26)	13.792	83.53	0.483	0.8	1.573	1.22	19.67	19.71
	Equiv. Refl.: (0,-4,-26), (4,0,-26), (4,-4,26), (-4,0,26), (-4,4,-26)								
Quartz	(-1,6,9)	13.129	71.07	0.481	0.6	4.761	2.91	59.51	59.58
	Equiv. Refl.: (-1,-5,-9)								
Quartz	(-5,6,-9)	13.129	71.07	0.48	0.6	4.761	2.9	59.51	59.58
	Equiv. Refl.: (-5,-1,9)								
Quartz	(6,-1,-9)	13.129	71.07	0.479	0.6	4.761	2.89	59.51	59.58
	Equiv. Refl.: (6,-5,9)								
Quartz	(9,-2,-5)	13.233	72.44	0.478	0.5	4.392	2.38	54.9	54.95
	Equiv. Refl.: (9,-7,5), (-2,-7,-5), (-7,9,-5), (-7,-2,5)								
Quartz	(-2,9,5)	13.233	72.44	0.477	0.5	4.392	2.37	54.9	54.95

Ei =	13.88	keV						ΔEg	ΔEt
**Cryst**	**Refl**	**EB**	**ΘB**	**∫ IR dΘ**	**Width**	**Ei cotΘ**	**ΔE**	**2m, 50µm**	**2m, 50µm**
	**(h,k,l)**	**[keV]**	**[°]**	**[µrad]**	**[µrad]**	**[meV/µrad]**	**[meV]**	**[meV]**	**[meV]**
**LiNbO3**	**(4,5,17)**	**13.256**	**72.76**	**0.464**	**0.9**	**4.307**	**3.88**	**53.84**	**53.98**
	Equiv. Refl.: (4,-9,-17), (5,4,-17), (5,-9,17), (9,-4,-17), (9,-5,17), (-4,9,17), (-4,-5,-17), (-5,9,-17), (-5,-4,17) (-9,4,17), (-9,5,-17)								
**Quartz**	**(2,5,-9)**	**13.761**	**82.48**	**0.461**	**0.7**	**1.832**	**1.21**	**22.89**	**22.93**
	Equiv. Refl.: (2,-7,9), (-7,2,-9), (-7,5,9)								
**Quartz**	**(5,2,9)**	**13.761**	**82.48**	**0.454**	**0.7**	**1.832**	**1.2**	**22.89**	**22.93**
	Equiv. Refl.: (5,-7,-9)								
**Quartz**	**(7,-2,9)**	**13.761**	**82.48**	**0.452**	**0.7**	**1.832**	**1.21**	**22.89**	**22.93**
	Equiv. Refl.: (7,-5,-9), (-2,7,-9), (-2,-5,9)								
**LiNbO3**	**(5,6,-7)**	**13.629**	**79.08**	**0.446**	**1**	**2.677**	**2.78**	**33.47**	**33.58**
	Equiv. Refl.: (5,-11,7), (6,5,7), (6,-11,-7), (11,-5,7), (11,-6,-7), (-5,11,-7), (-5,-6,7), (-6,11,7), (-6,-5,-7) (-11,5,-7), (-11,6,7)								
**Quartz**	**(-5,7,9)**	**13.761**	**82.48**	**0.445**	**0.7**	**1.832**	**1.2**	**22.89**	**22.93**
	Equiv. Refl.: (-5,-2,-9)								
**Quartz**	**(-10,4,3)**	**13.16**	**71.46**	**0.444**	**0.5**	**4.655**	**2.32**	**58.19**	**58.24**
	Equiv. Refl.: (-10,6,-3)								
**Quartz**	**(4,6,3)**	**13.16**	**71.46**	**0.443**	**0.5**	**4.655**	**2.32**	**58.19**	**58.24**
	Equiv. Refl.: (4,-10,-3), (6,4,-3), (6,-10,3)								
**Quartz**	**(-2,7,9)**	**13.761**	**82.48**	**0.441**	**0.6**	**1.832**	**1.12**	**22.89**	**22.92**
	Equiv. Refl.: (-2,-5,-9)								
**Sapph**	**(1,5,-22)**	**13.432**	**75.4**	**0.44**	**0.6**	**3.614**	**2.12**	**45.18**	**45.23**
	Equiv. Refl.: (1,-6,22), (5,1,22), (5,-6,-22), (6,-1,22), (6,-5,-22), (-1,6,-22), (-1,-5,22), (-5,6,22), (-5,-1,-22) (-6,1,-22), (-6,5,22)								
**Quartz**	**(7,-2,-9)**	**13.761**	**82.48**	**0.439**	**0.6**	**1.832**	**1.11**	**22.89**	**22.92**
	Equiv. Refl.: (7,-5,9), (-5,7,-9)								
**Quartz**	**(-5,-2,9)**	**13.761**	**82.48**	**0.438**	**0.6**	**1.832**	**1.11**	**22.89**	**22.92**
**Quartz**	**(8,-7,-7)**	**13.619**	**78.86**	**0.434**	**0.5**	**2.732**	**1.4**	**34.15**	**34.18**
	Equiv. Refl.: (-7,8,7), (-7,-1,-7)								
**Quartz**	**(8,-1,7)**	**13.619**	**78.86**	**0.433**	**0.5**	**2.732**	**1.4**	**34.15**	**34.18**
**Quartz**	**(-1,8,-7)**	**13.619**	**78.86**	**0.432**	**0.5**	**2.732**	**1.4**	**34.15**	**34.18**
	Equiv. Refl.: (-1,-7,7)								
**Quartz**	**(5,0,10)**	**13.588**	**78.23**	**0.429**	**0.6**	**2.893**	**1.63**	**36.16**	**36.19**
	Equiv. Refl.: (-5,0,-10)								
**Quartz**	**(5,-5,-10)**	**13.588**	**78.23**	**0.428**	**0.6**	**2.893**	**1.63**	**36.16**	**36.19**
	Equiv. Refl.: (-5,5,10)								
**Quartz**	**(0,5,-10)**	**13.588**	**78.23**	**0.426**	**0.6**	**2.893**	**1.62**	**36.16**	**36.19**
	Equiv. Refl.: (0,-5,10)								
**Quartz**	**(2,7,5)**	**13.233**	**72.44**	**0.421**	**0.5**	**4.392**	**2.38**	**54.9**	**54.95**
	Equiv. Refl.: (7,2,-5), (7,-9,5), (-9,2,5), (-9,7,-5)								
**Quartz**	**(2,-9,-5)**	**13.233**	**72.44**	**0.42**	**0.5**	**4.392**	**2.37**	**54.9**	**54.95**
**Quartz**	**(0,7,8)**	**13.719**	**81.27**	**0.414**	**0.6**	**2.131**	**1.26**	**26.63**	**26.66**
	Equiv. Refl.: (0,-7,-8)								
**Quartz**	**(7,0,-8)**	**13.719**	**81.27**	**0.409**	**0.6**	**2.131**	**1.25**	**26.63**	**26.66**
	Equiv. Refl.: (7,-7,8), (-7,0,8)								
**Quartz**	**(-7,7,-8)**	**13.719**	**81.27**	**0.408**	**0.6**	**2.131**	**1.25**	**26.63**	**26.66**
**LiNbO3**	**(1,2,29)**	**13.48**	**76.21**	**0.403**	**0.9**	**3.407**	**3.05**	**42.59**	**42.7**
	Equiv. Refl.: (1,-3,-29), (2,1,-29), (2,-3,29), (3,-1,-29), (3,-2,29), (-1,3,29), (-1,-2,-29), (-2,3,-29), (-2,-1,29) (-3,1,29), (-3,2,-29)								
**Quartz**	**(1,4,10)**	**13.272**	**72.98**	**0.381**	**0.5**	**4.249**	**2**	**53.12**	**53.16**

Ei =	13.88	keV						ΔEg	ΔEt
**Cryst**	**Refl**	**EB**	**ΘB**	**∫ IR dΘ**	**Width**	**Ei cotΘ**	**ΔE**	**2m, 50μm**	**2m, 50μm**
	**(h,k,l)**	**[keV]**	**[°]**	**[μrad]**	**[μrad]**	**[meV/μrad]**	**[meV]**	**[meV]**	**[meV]**
**Quartz**	**(1,-5,-10)**	**13.272**	**72.98**	**0.38**	**0.5**	**4.249**	**2**	**53.12**	**53.16**
	Equiv. Refl.: (4,1,-10), (4,-5,10), (10,-4,-3), (10,-6,3), (-4,10,3), (-4,-6,-3)								
**Quartz**	**(-6,10,-3)**	**13.16**	**71.46**	**0.379**	**0.5**	**4.655**	**2.32**	**58.19**	**58.24**
	Equiv. Refl.: (-6,-4,3)								
**Sapph**	**(1,6,19)**	**13.4**	**74.89**	**0.377**	**0.5**	**3.748**	**1.94**	**46.85**	**46.89**
	Equiv. Refl.: (1,-7,-19), (6,1,-19), (6,-7,19), (7,-1,-19), (7,-6,19), (-1,7,19), (-1,-6,-19), (-6,7,-19), (-6,-1,19) (-7,1,19), (-7,6,-19), (-5,1,10), (-5,4,-10)								
**Quartz**	**(2,5,9)**	**13.761**	**82.48**	**0.376**	**0.6**	**1.832**	**1.12**	**22.89**	**22.92**
	Equiv. Refl.: (2,-7,-9)								
**Quartz**	**(5,2,-9)**	**13.761**	**82.48**	**0.374**	**0.6**	**1.832**	**1.11**	**22.89**	**22.92**
	Equiv. Refl.: (5,-7,9), (-7,2,9), (-7,5,-9)								
**Quartz**	**(-1,5,10)**	**13.272**	**72.98**	**0.37**	**0.5**	**4.249**	**2**	**53.12**	**53.16**
	Equiv. Refl.: (-1,-4,-10)								
**Quartz**	**(-4,5,-10)**	**13.272**	**72.98**	**0.369**	**0.5**	**4.249**	**2**	**53.12**	**53.16**
	Equiv. Refl.: (-4,-1,10)								
**Sapph**	**(5,5,3)**	**13.107**	**70.79**	**0.366**	**0.5**	**4.836**	**2.33**	**60.45**	**60.5**
	Equiv. Refl.: (5,5,-3), (5,-10,3), (5,-10,-3), (10,-5,3), (10,-5,-3), (-5,10,3), (-5,10,-3), (-5,-5,3), (-5,-5,-3) (-10,5,3), (-10,5,-3), (5,-1,-10), (5,-4,10)								
**LiNbO3**	**(3,7,11)**	**13.302**	**73.4**	**0.358**	**0.8**	**4.137**	**3.23**	**51.71**	**51.81**
	Equiv. Refl.: (3,-10,-11), (7,3,-11), (7,-10,11), (10,-3,-11), (10,-7,11), (-3,10,11), (-3,-7,-11), (-7,10,-11), (-7,-3,11) (-10,3,11), (-10,7,-11)								
**LiNbO3**	**(5,6,5)**	**13.452**	**75.73**	**0.355**	**0.8**	**3.531**	**2.9**	**44.14**	**44.23**
	Equiv. Refl.: (5,-11,-5), (6,5,-5), (6,-11,5), (11,-5,-5), (11,-6,5), (-5,11,5), (-5,-6,-5), (-6,11,-5), (-6,-5,5) (-11,5,5), (-11,6,-5)								
**Quartz**	**(7,1,7)**	**13.619**	**78.86**	**0.316**	**0.5**	**2.732**	**1.4**	**34.15**	**34.18**
	Equiv. Refl.: (7,-8,-7), (-8,7,7)								
**Quartz**	**(-8,1,-7)**	**13.619**	**78.86**	**0.315**	**0.5**	**2.732**	**1.4**	**34.15**	**34.18**
**Quartz**	**(1,7,-7)**	**13.619**	**78.86**	**0.314**	**0.5**	**2.732**	**1.39**	**34.15**	**34.18**
	Equiv. Refl.: (1,-8,7)								
**Quartz**	**(2,-6,10)**	**13.82**	**84.69**	**0.312**	**0.7**	**1.29**	**0.9**	**16.13**	**16.15**
**LiNbO3**	**(5,6,-1)**	**13.272**	**72.98**	**0.311**	**0.7**	**4.249**	**3.02**	**53.12**	**53.2**
	Equiv. Refl.: (5,-11,1), (6,5,1), (6,-11,-1), (11,-5,1), (11,-6,-1), (-5,11,-1), (-5,-6,1), (-6,11,1), (-6,-5,-1) (-11,5,-1), (-11,6,1), (2,4,-10)								
**Quartz**	**(-3,8,8)**	**13.719**	**81.27**	**0.309**	**0.4**	**2.131**	**0.95**	**26.63**	**26.65**
	Equiv. Refl.: (-3,-5,-8), (-5,8,-8), (-5,-3,8)								
**Quartz**	**(4,2,10)**	**13.82**	**84.69**	**0.308**	**0.7**	**1.29**	**0.9**	**16.13**	**16.15**
	Equiv. Refl.: (8,-3,-8)								
**Quartz**	**(4,-6,-10)**	**13.82**	**84.69**	**0.307**	**0.7**	**1.29**	**0.89**	**16.13**	**16.15**
	Equiv. Refl.: (8,-5,8), (-6,4,10)								
**Quartz**	**(-6,2,-10)**	**13.82**	**84.69**	**0.306**	**0.7**	**1.29**	**0.89**	**16.13**	**16.15**
**LiNbO3**	**(2,8,9)**	**13.364**	**74.33**	**0.259**	**0.7**	**3.892**	**2.57**	**48.66**	**48.72**
	Equiv. Refl.: (2,8,-9), (2,-10,9), (2,-10,-9), (8,2,9), (8,2,-9), (8,-10,9), (8,-10,-9), (10,-2,9), (10,-2,-9) (10,-8,9), (10,-8,-9), (-2,10,9), (-2,10,-9), (-2,-8,9), (-2,-8,-9), (-8,10,9), (-8,10,-9), (-8,-2,9) (-8,-2,-9), (-10,2,9), (-10,2,-9), (-10,8,9), (-10,8,-9)								
**Quartz**	**(5,3,-8)**	**13.719**	**81.27**	**0.245**	**0.4**	**2.131**	**0.95**	**26.63**	**26.65**
	Equiv. Refl.: (5,-8,8)								
**Quartz**	**(3,5,8)**	**13.719**	**81.27**	**0.244**	**0.4**	**2.131**	**0.95**	**26.63**	**26.65**
	Equiv. Refl.: (3,-8,-8), (-8,3,8)								
**Quartz**	**(-8,5,-8)**	**13.719**	**81.27**	**0.243**	**0.4**	**2.131**	**0.94**	**26.63**	**26.65**
**Quartz**	**(10,-2,-1)**	**13.403**	**74.93**	**0.228**	**0.3**	**3.737**	**1.16**	**46.72**	**46.73**
	Equiv. Refl.: (10,-8,1), (-2,10,1), (-2,-8,-1), (-8,10,-1), (-8,-2,1)								
**Sapph**	**(1,3,25)**	**13.107**	**70.79**	**0.222**	**0.3**	**4.836**	**1.61**	**60.45**	**60.47**

	Equiv. Refl.: (1,-4,-25), (3,1,-25), (3,-4,25), (4,-1,-25), (4,-3,25), (-1,4,25), (-1,-3,-25), (-3,4,-25), (-3,-1,25) (-4,1,25), (-4,3,-25)								
**Quartz**	**(-2,-3,10)**	**13.111**	**70.84**	**0.219**	**0.3**	**4.823**	**1.43**	**60.29**	**60.31**
	Equiv. Refl.: (-3,-2,-10)								
**Quartz**	**(-2,5,-10)**	**13.111**	**70.84**	**0.218**	**0.3**	**4.823**	**1.43**	**60.29**	**60.31**
	Equiv. Refl.: (-3,5,10)								
**Quartz**	**(5,-2,10)**	**13.111**	**70.84**	**0.216**	**0.3**	**4.823**	**1.42**	**60.29**	**60.31**
	Equiv. Refl.: (5,-3,-10)								
**Quartz**	**(2,3,-10)**	**13.111**	**70.84**	**0.203**	**0.3**	**4.823**	**1.43**	**60.29**	**60.31**
	Equiv. Refl.: (2,-5,10), (3,2,10), (3,-5,-10)								
**Quartz**	**(-5,3,10)**	**13.111**	**70.84**	**0.201**	**0.3**	**4.823**	**1.42**	**60.29**	**60.31**
**Quartz**	**(-5,2,-10)**	**13.111**	**70.84**	**0.2**	**0.3**	**4.823**	**1.42**	**60.29**	**60.31**
**LiNbO3**	**(2,6,-19)**	**13.143**	**71.24**	**0.196**	**0.5**	**4.714**	**2.48**	**58.93**	**58.98**
	Equiv. Refl.: (2,-8,19), (6,2,19), (6,-8,-19), (8,-2,19), (8,-6,-19), (-2,8,-19), (-2,-6,19), (-6,8,19), (-6,-2,-19) (-8,2,-19), (-8,6,19)								
**Quartz**	**(2,8,1)**	**13.403**	**74.93**	**0.187**	**0.3**	**3.737**	**1.16**	**46.72**	**46.73**
	Equiv. Refl.: (2,-10,-1), (8,2,-1), (8,-10,1), (-10,2,1), (-10,8,-1)								
**LiNbO3**	**(3,8,1)**	**13.702**	**80.81**	**0.177**	**0.7**	**2.244**	**1.52**	**28.06**	**28.1**
	Equiv. Refl.: (3,-11,-1), (8,3,-1), (8,-11,1), (11,-3,-1), (11,-8,1), (-3,11,1), (-3,-8,-1), (-8,11,-1), (-8,-3,1) (-11,3,1), (-11,8,-1)								
**Sapph**	**(3,7,-4)**	**13.507**	**76.69**	**0.152**	**0.3**	**3.284**	**0.93**	**41.04**	**41.05**
	Equiv. Refl.: (3,-10,4), (7,3,4), (7,-10,-4), (10,-3,4), (10,-7,-4), (-3,10,-4), (-3,-7,4), (-7,10,4), (-7,-3,-4) (-10,3,-4), (-10,7,4)								
**LiNbO3**	**(1,8,-13)**	**13.226**	**72.35**	**0.088**	**0.4**	**4.417**	**1.56**	**55.22**	**55.24**
	Equiv. Refl.: (1,-9,13), (8,1,13), (8,-9,-13), (9,-1,13), (9,-8,-13), (-1,9,-13), (-1,-8,13), (-8,9,13), (-8,-1,-13) (-9,1,-13), (-9,8,13)								
**Sapph**	**(4,6,7)**	**13.534**	**77.18**	**0.087**	**0.2**	**3.158**	**0.66**	**39.48**	**39.48**
	Equiv. Refl.: (4,-10,-7), (6,4,-7), (6,-10,7), (10,-4,-7), (10,-6,7), (-4,10,7), (-4,-6,-7), (-6,10,-7), (-6,-4,7) (-10,4,7), (-10,6,-7)								
**Sapph**	**(4,6,-5)**	**13.331**	**73.83**	**0.073**	**0.2**	**4.026**	**0.7**	**50.32**	**50.33**
	Equiv. Refl.: (4,-10,5), (6,4,5), (6,-10,-5), (10,-4,5), (10,-6,-5), (-4,10,-5), (-4,-6,5), (-6,10,5), (-6,-4,-5) (-10,4,-5), (-10,6,5)								
**Sapph**	**(4,6,1)**	**13.124**	**71**	**0.067**	**0.2**	**4.779**	**0.74**	**59.74**	**59.74**
	Equiv. Refl.: (4,-10,-1), (6,4,-1), (6,-10,1), (10,-4,-1), (10,-6,1), (-4,10,1), (-4,-6,-1), (-6,10,-1), (-6,-4,1) (-10,4,1), (-10,6,-1)								
**LiNbO3**	**(5,5,15)**	**13.784**	**83.26**	**0.014**	**0.2**	**1.64**	**0.4**	**20.5**	**20.5**
	Equiv. Refl.: (5,5,-15), (5,-10,15), (5,-10,-15), (10,-5,15), (10,-5,-15), (-5,10,15), (-5,10,-15), (-5,-5,15), (-5,-5,-15) (-10,5,15), (-10,5,-15)								
**LiNbO3**	**(4,4,21)**	**13.453**	**75.76**	**0.011**	**0.1**	**3.523**	**0.52**	**44.04**	**44.05**
	Equiv. Refl.: (4,4,-21), (4,-8,21), (4,-8,-21), (8,-4,21), (8,-4,-21), (-4,8,21), (-4,8,-21), (-4,-4,21), (-4,-4,-21) (-8,4,21), (-8,4,-21)								

Ei = Cryst	13.273 Refl (h,k,l)	keV EB [keV]	ΘB [°]	∫ IR dΘ [μrad]	Width [μrad]	Ei cotΘ [meV/μrad]	ΔE [meV]	ΔEg 2m, 50μm [meV]	ΔEt 2m, 50μm [meV]
**Ge**	**(0,0,12)**	**13.149**	**82.17**	**4.3726**	**9.56**	**1.8245**	**17.43**	**22.81**	**28.71**
	Equiv. Refl.: (0,12,0), (4,8,8), (8,4,8), (8,8,4), (12,0,0)								
**Si**	**(0,8,8)**	**12.914**	**76.64**	**3.1436**	**2.91**	**3.1515**	**9.18**	**39.39**	**40.45**
	Equiv. Refl.: (8,0,8), (8,8,0)								
**Si**	**(1,3,11)**	**13.064**	**79.83**	**2.99**	**2.59**	**2.3812**	**6.16**	**29.76**	**30.4**
	Equiv. Refl.: (1,7,9), (1,9,7), (1,11,3), (3,1,11), (3,11,1), (5,5,9), (5,9,5), (7,1,9), (7,9,1) (9,1,7), (9,5,5), (9,7,1), (11,1,3), (11,3,1)								
**Ge**	**(0,6,10)**	**12.779**	**74.32**	**2.5064**	**5.29**	**3.7266**	**19.71**	**46.58**	**50.58**
	Equiv. Refl.: (0,10,6), (6,0,10), (6,6,8), (6,8,6), (6,10,0), (8,6,6), (10,0,6), (10,6,0)								
**Si**	**(2,4,10)**	**12.504**	**70.4**	**2.4441**	**2.24**	**4.7264**	**10.58**	**59.08**	**60.02**
	Equiv. Refl.: (2,10,4), (4,2,10), (4,10,2), (10,2,4), (10,4,2)								
**Si**	**(1,1,11)**	**12.659**	**72.51**	**1.5429**	**1.69**	**4.183**	**7.09**	**52.29**	**52.77**
	Equiv. Refl.: (1,11,1), (5,7,7), (7,5,7), (7,7,5), (11,1,1)								
**Ge**	**(3,3,11)**	**12.919**	**76.74**	**1.5183**	**4.25**	**3.1282**	**13.3**	**39.1**	**41.3**
	Equiv. Refl.: (3,7,9), (3,9,7), (3,11,3), (7,3,9), (7,9,3), (9,3,7), (9,7,3), (11,3,3)								
**Ge**	**(1,3,11)**	**12.542**	**70.89**	**1.2285**	**3.28**	**4.598**	**15.09**	**57.48**	**59.42**
	Equiv. Refl.: (1,7,9), (1,9,7), (1,11,3), (3,1,11), (3,11,1), (5,5,9), (5,9,5), (7,1,9), (7,9,1) (9,1,7), (9,5,5), (9,7,1), (11,1,3), (11,3,1)								
**Quartz**	**(4,1,10)**	**13.272**	**89.27**	**59.059**	**49.7**	**0.169**	**8.39**	**2.11**	**8.65**
**Quartz**	**(4,-5,-10)**	**13.272**	**89.27**	**59.055**	**49.7**	**0.169**	**8.38**	**2.11**	**8.65**
**Quartz**	**(1,4,-10)**	**13.272**	**89.27**	**59.014**	**49.7**	**0.169**	**8.38**	**2.11**	**8.64**
**Quartz**	**(1,-5,10)**	**13.272**	**89.27**	**59.001**	**49.7**	**0.169**	**8.38**	**2.11**	**8.64**
**Quartz**	**(-5,1,-10)**	**13.272**	**89.27**	**58.963**	**49.6**	**0.169**	**8.37**	**2.11**	**8.63**
**Quartz**	**(-5,4,10)**	**13.272**	**89.27**	**58.961**	**49.6**	**0.169**	**8.37**	**2.11**	**8.63**
**Quartz**	**(-4,-1,-10)**	**13.272**	**89.27**	**57.706**	**49.7**	**0.169**	**8.39**	**2.11**	**8.65**
**Quartz**	**(-4,5,10)**	**13.272**	**89.27**	**57.703**	**49.7**	**0.169**	**8.38**	**2.11**	**8.65**
**Quartz**	**(-1,-4,10)**	**13.272**	**89.27**	**57.675**	**49.7**	**0.169**	**8.38**	**2.11**	**8.64**
**Quartz**	**(-1,5,-10)**	**13.272**	**89.27**	**57.661**	**49.7**	**0.169**	**8.38**	**2.11**	**8.64**
**Quartz**	**(5,-1,10)**	**13.272**	**89.27**	**57.618**	**49.6**	**0.169**	**8.37**	**2.11**	**8.63**
**Quartz**	**(5,-4,-10)**	**13.272**	**89.27**	**57.615**	**49.6**	**0.169**	**8.37**	**2.11**	**8.63**
**Sapph**	**(2,6,-16)**	**13.267**	**88.26**	**26.494**	**22.9**	**0.404**	**9.26**	**5.05**	**10.55**
	Equiv. Refl.: (2,-8,16), (6,2,16), (6,-8,-16), (8,-2,16), (8,-6,-16), (-2,8,-16), (-2,-6,16), (-6,8,16), (-6,-2,-16) (-8,2,-16), (-8,6,16)								
**LiNbO3**	**(1,9,1)**	**13.272**	**89.28**	**24.195**	**35.4**	**0.168**	**5.93**	**2.09**	**6.29**
	Equiv. Refl.: (1,-10,-1), (9,1,-1), (9,-10,1), (10,-1,-1), (10,-9,1), (-1,10,1), (-1,-9,-1), (-9,10,-1), (-9,-1,1) (-10,1,1), (-10,9,-1)								
**LiNbO3**	**(-7,8,-18)**	**13.229**	**85.31**	**22.429**	**20.5**	**1.088**	**22.33**	**13.6**	**26.15**
	Equiv. Refl.: (-8,7,-18)								
**LiNbO3**	**(1,-8,-18)**	**13.229**	**85.31**	**22.428**	**20.5**	**1.088**	**22.33**	**13.6**	**26.15**
	Equiv. Refl.: (7,1,-18), (8,-1,-18), (-1,-7,-18)								
**LiNbO3**	**(1,7,18)**	**13.229**	**85.31**	**22.152**	**20.5**	**1.088**	**22.33**	**13.6**	**26.15**
	Equiv. Refl.: (7,-8,18), (8,-7,18), (-1,8,18), (-7,-1,18), (-8,1,18)								
**LiNbO3**	**(0,-7,-20)**	**13.218**	**84.8**	**21.439**	**19.4**	**1.208**	**23.45**	**15.1**	**27.89**
	Equiv. Refl.: (7,0,-20), (-7,7,-20)								
**LiNbO3**	**(0,7,20)**	**13.218**	**84.8**	**21.045**	**19.4**	**1.208**	**23.45**	**15.1**	**27.89**
	Equiv. Refl.: (7,-7,20), (-7,0,20)								
**LiNbO3**	**(0,-8,-16)**	**13.226**	**85.19**	**20.264**	**17.7**	**1.117**	**19.83**	**13.97**	**24.25**
	Equiv. Refl.: (8,0,-16), (-8,8,-16)								

Ei =	13.273	keV						ΔEg	ΔEt
Cryst	Refl	EB	ΘB	∫ IR dΘ	Width	Ei cotΘ	ΔE	2m, 50µm	2m, 50µm
	(h,k,l)	[keV]	[°]	[µrad]	[µrad]	[meV/µrad]	[meV]	[meV]	[meV]
LiNbO3	(1,-8,18)	13.229	85.31	20.211	18.8	1.088	20.49	13.6	24.59
	Equiv. Refl.: (7,1,18), (8,-1,18), (-1,-7,18)								
LiNbO3	(-7,8,18)	13.229	85.31	20.21	18.8	1.088	20.49	13.6	24.59
	Equiv. Refl.: (-8,7,18)								
LiNbO3	(1,7,-18)	13.229	85.31	20.119	18.8	1.088	20.49	13.6	24.59
	Equiv. Refl.: (7,-8,-18), (8,-7,-18), (-1,8,-18), (-7,-1,-18), (-8,1,-18)								
LiNbO3	(0,8,16)	13.226	85.19	17.518	17.7	1.117	19.83	13.97	24.25
	Equiv. Refl.: (8,-8,16), (-8,0,16)								
LiNbO3	(3,5,-20)	13.218	84.8	15.928	14.4	1.208	17.35	15.1	23
	Equiv. Refl.: (5,-8,-20), (8,-5,-20), (-3,8,-20), (-5,-3,-20), (-8,3,-20)								
LiNbO3	(5,5,-12)	13.183	83.34	15.838	14.2	1.549	22.04	19.37	29.34
	Equiv. Refl.: (5,-10,-12), (10,-5,-12), (-5,10,-12), (-5,-5,-12), (-10,5,-12)								
LiNbO3	(5,5,12)	13.183	83.34	14.969	14.2	1.549	22.04	19.37	29.34
	Equiv. Refl.: (5,-10,12), (10,-5,12), (-5,10,12), (-5,-5,12), (-10,5,12)								
Quartz	(8,1,4)	13.267	88.29	14.516	12.6	0.397	4.99	4.96	7.04
	Equiv. Refl.: (8,-9,-4)								
Quartz	(-9,1,-4)	13.267	88.29	14.506	12.6	0.397	4.99	4.96	7.03
Quartz	(1,8,-4)	13.267	88.29	14.505	12.6	0.397	4.99	4.96	7.03
Quartz	(-9,8,4)	13.267	88.29	14.504	12.6	0.397	4.99	4.96	7.03
Quartz	(1,-9,4)	13.267	88.29	14.503	12.6	0.397	4.99	4.96	7.03
Sapph	(3,4,20)	13.224	85.06	14.062	11.7	1.148	13.41	14.35	19.64
	Equiv. Refl.: (3,-7,-20), (4,3,-20), (4,-7,20), (7,-3,-20), (7,-4,20), (-3,7,20), (-3,-4,-20), (-4,7,-20), (-4,-3,20) (-7,3,20), (-7,4,-20)								
Quartz	(-1,9,4)	13.267	88.29	13.968	12.3	0.397	4.89	4.96	6.96
Quartz	(-1,-8,-4)	13.267	88.29	13.967	12.3	0.397	4.89	4.96	6.96
Quartz	(-8,-1,4)	13.267	88.29	13.965	12.3	0.397	4.89	4.96	6.96
Quartz	(9,-1,-4)	13.267	88.29	13.964	12.3	0.397	4.89	4.96	6.96
	Equiv. Refl.: (-8,9,-4)								
Quartz	(9,-8,4)	13.267	88.29	13.961	12.3	0.397	4.89	4.96	6.96
LiNbO3	(3,-8,20)	13.218	84.8	13.942	14.4	1.208	17.35	15.1	23
	Equiv. Refl.: (5,3,20), (8,-3,20), (-3,-5,20), (-5,8,20), (-8,5,20)								
LiNbO3	(3,-10,10)	13.143	81.98	13.895	12.7	1.871	23.7	23.39	33.3
	Equiv. Refl.: (7,3,10), (10,-3,10), (-3,-7,10), (-7,10,10), (-10,7,10)								
LiNbO3	(3,7,-10)	13.143	81.98	13.813	12.7	1.871	23.7	23.39	33.3
	Equiv. Refl.: (-3,10,-10), (-7,-3,-10), (-10,3,-10)								
LiNbO3	(7,-10,-10)	13.143	81.98	13.812	12.7	1.871	23.7	23.39	33.3
	Equiv. Refl.: (10,-7,-10)								
LiNbO3	(2,3,26)	13.112	81.05	13.709	12.4	2.089	25.81	26.11	36.72
	Equiv. Refl.: (3,-5,26), (5,-3,26), (-2,5,26), (-3,-2,26), (-5,2,26)								
LiNbO3	(2,-5,-26)	13.112	81.05	13.648	12.4	2.089	25.81	26.11	36.72
	Equiv. Refl.: (3,2,-26), (5,-2,-26), (-2,-3,-26), (-3,5,-26), (-5,3,-26)								
Quartz	(-8,9,4)	13.267	88.29	13.633	12.6	0.397	4.99	4.96	7.04
	Equiv. Refl.: (-8,-1,-4)								
Quartz	(9,-1,4)	13.267	88.29	13.623	12.6	0.397	4.99	4.96	7.03
	Equiv. Refl.: (-1,-8,4)								
Quartz	(9,-8,-4)	13.267	88.29	13.622	12.6	0.397	4.99	4.96	7.03
Quartz	(-1,9,-4)	13.267	88.29	13.621	12.6	0.397	4.99	4.96	7.03
Quartz	(1,-9,-4)	13.267	88.29	13.606	12.3	0.397	4.89	4.96	6.96
Quartz	(1,8,4)	13.267	88.29	13.605	12.3	0.397	4.89	4.96	6.96
	Equiv. Refl.: (8,1,-4), (8,-9,4)								

Ei = Cryst	13.273 Refl (h,k,l)	keV EB [keV]	ΘB [°]	∫ IR dΘ [μrad]	Width [μrad]	Ei cotΘ [meV/μrad]	ΔE [meV]	ΔEg 2m, 50μm [meV]	ΔEt 2m, 50μm [meV]
**Quartz**	**(-9,1,4)**	**13.267**	**88.29**	**13.604**	**12.3**	**0.397**	**4.89**	**4.96**	**6.96**
**Quartz**	**(-9,8,-4)**	**13.267**	**88.29**	**13.601**	**12.3**	**0.397**	**4.89**	**4.96**	**6.96**
**LiNbO3**	**(1,2,-28)**	**13.05**	**79.49**	**10.343**	**9.2**	**2.463**	**22.75**	**30.79**	**38.28**
	Equiv. Refl.: (2,-3,-28), (3,-2,-28), (-1,3,-28), (-2,-1,-28), (-3,1,-28)								
**LiNbO3**	**(4,-9,16)**	**13.005**	**78.47**	**10.332**	**9.3**	**2.708**	**25.12**	**33.85**	**42.15**
	Equiv. Refl.: (5,4,16), (9,-4,16), (-4,-5,16), (-5,9,16), (-9,5,16)								
**Quartz**	**(1,4,10)**	**13.272**	**89.27**	**10.268**	**11.4**	**0.169**	**1.92**	**2.11**	**2.85**
**Quartz**	**(1,-5,-10)**	**13.272**	**89.27**	**10.264**	**11.4**	**0.169**	**1.92**	**2.11**	**2.85**
**Quartz**	**(4,1,-10)**	**13.272**	**89.27**	**10.254**	**11.4**	**0.169**	**1.92**	**2.11**	**2.85**
**Quartz**	**(4,-5,10)**	**13.272**	**89.27**	**10.253**	**11.4**	**0.169**	**1.92**	**2.11**	**2.85**
**LiNbO3**	**(1,-3,28)**	**13.05**	**79.49**	**10.225**	**9.2**	**2.463**	**22.75**	**30.79**	**38.28**
	Equiv. Refl.: (2,1,28), (3,-1,28), (-1,-2,28), (-2,3,28), (-3,2,28)								
**LiNbO3**	**(2,7,-14)**	**12.99**	**78.14**	**10.189**	**8.8**	**2.787**	**24.52**	**34.83**	**42.59**
	Equiv. Refl.: (-2,9,-14), (-7,-2,-14), (-9,2,-14)								
**LiNbO3**	**(7,-9,-14)**	**12.99**	**78.14**	**10.188**	**8.8**	**2.787**	**24.51**	**34.83**	**42.59**
	Equiv. Refl.: (9,-7,-14)								
**Quartz**	**(-5,4,-10)**	**13.272**	**89.27**	**10.171**	**11.3**	**0.169**	**1.91**	**2.11**	**2.84**
**Quartz**	**(-5,1,10)**	**13.272**	**89.27**	**10.164**	**11.3**	**0.169**	**1.91**	**2.11**	**2.84**
**LiNbO3**	**(4,5,-16)**	**13.005**	**78.47**	**10.076**	**9.3**	**2.708**	**25.12**	**33.85**	**42.15**
	Equiv. Refl.: (5,-9,-16), (9,-5,-16), (-4,9,-16), (-5,-4,-16), (-9,4,-16)								
**Quartz**	**(-1,-4,-10)**	**13.272**	**89.27**	**9.949**	**11.4**	**0.169**	**1.92**	**2.11**	**2.85**
**Quartz**	**(-1,5,10)**	**13.272**	**89.27**	**9.945**	**11.4**	**0.169**	**1.92**	**2.11**	**2.85**
**Quartz**	**(-4,-1,10)**	**13.272**	**89.27**	**9.937**	**11.4**	**0.169**	**1.92**	**2.11**	**2.85**
**Quartz**	**(-4,5,-10)**	**13.272**	**89.27**	**9.936**	**11.4**	**0.169**	**1.92**	**2.11**	**2.85**
**Quartz**	**(5,-4,10)**	**13.272**	**89.27**	**9.851**	**11.3**	**0.169**	**1.91**	**2.11**	**2.84**
**Quartz**	**(5,-1,-10)**	**13.272**	**89.27**	**9.845**	**11.3**	**0.169**	**1.91**	**2.11**	**2.84**
**LiNbO3**	**(2,-9,14)**	**12.99**	**78.14**	**9.637**	**8.8**	**2.787**	**24.52**	**34.83**	**42.59**
	Equiv. Refl.: (7,2,14), (9,-2,14), (-2,-7,14)								
**LiNbO3**	**(-7,9,14)**	**12.99**	**78.14**	**9.636**	**8.8**	**2.787**	**24.51**	**34.83**	**42.59**
	Equiv. Refl.: (-9,7,14)								
**LiNbO3**	**(4,-10,-10)**	**12.921**	**76.77**	**9.504**	**8.3**	**3.122**	**25.8**	**39.02**	**46.78**
	Equiv. Refl.: (6,4,-10), (10,-4,-10), (-4,-6,-10), (-6,10,-10), (-10,6,-10)								
**LiNbO3**	**(4,6,10)**	**12.921**	**76.77**	**9.256**	**8.3**	**3.122**	**25.8**	**39.02**	**46.78**
	Equiv. Refl.: (6,-10,10), (10,-6,10), (-4,10,10), (-6,-4,10), (-10,4,10)								
**LiNbO3**	**(2,8,6)**	**13.023**	**78.87**	**8.873**	**8**	**2.611**	**20.76**	**32.64**	**38.68**
	Equiv. Refl.: (8,-10,6), (10,-8,6), (-2,10,6), (-8,-2,6), (-10,2,6)								
**LiNbO3**	**(0,-9,6)**	**12.799**	**74.64**	**8.602**	**7.5**	**3.647**	**27.37**	**45.58**	**53.17**
	Equiv. Refl.: (9,0,6), (-9,9,6)								
**LiNbO3**	**(0,9,-6)**	**12.799**	**74.64**	**8.553**	**7.5**	**3.647**	**27.37**	**45.58**	**53.17**
	Equiv. Refl.: (9,-9,-6), (-9,0,-6)								
**Quartz**	**(9,-2,5)**	**13.233**	**85.57**	**8.527**	**7**	**1.029**	**7.23**	**12.87**	**14.76**
**Quartz**	**(9,-7,-5)**	**13.233**	**85.57**	**8.526**	**7**	**1.029**	**7.23**	**12.87**	**14.76**
**LiNbO3**	**(3,7,8)**	**12.866**	**75.78**	**8.523**	**7.5**	**3.364**	**25.15**	**42.05**	**48.99**
	Equiv. Refl.: (7,-10,8), (10,-7,8), (-3,10,8), (-7,-3,8), (-10,3,8)								
**Quartz**	**(-7,9,5)**	**13.233**	**85.57**	**8.52**	**7**	**1.029**	**7.23**	**12.87**	**14.76**
	Equiv. Refl.: (-7,-2,-5)								
**Quartz**	**(-2,9,-5)**	**13.233**	**85.57**	**8.519**	**7**	**1.029**	**7.23**	**12.87**	**14.76**
	Equiv. Refl.: (-2,-7,5)								
**LiNbO3**	**(2,-10,-6)**	**13.023**	**78.87**	**8.507**	**8**	**2.611**	**20.76**	**32.64**	**38.68**
	Equiv. Refl.: (8,2,-6), (10,-2,-6), (-2,-8,-6), (-8,10,-6), (-10,8,-6)								

**Ei =**	**13.273**	**keV**						**ΔEg**	**ΔEt**
**Cryst**	**Refl**	**EB**	**ΘB**	**∫ IR dΘ**	**Width**	**Ei cotΘ**	**ΔE**	**2m, 50µm**	**2m, 50µm**
	**(h,k,l)**	**[keV]**	**[°]**	**[µrad]**	**[µrad]**	**[meV/µrad]**	**[meV]**	**[meV]**	**[meV]**
**LiNbO3**	**(3,4,-22)**	**12.974**	**77.81**	**8.421**	**7.5**	**2.867**	**21.52**	**35.83**	**41.79**
	Equiv. Refl.: (4,-7,-22), (7,-4,-22), (-3,7,-22), (-4,-3,-22), (-7,3,-22)								
**Quartz**	**(-9,2,-5)**	**13.233**	**85.57**	**8.42**	**7**	**1.029**	**7.23**	**12.87**	**14.76**
	Equiv. Refl.: (-9,7,5)								
**Quartz**	**(7,2,5)**	**13.233**	**85.57**	**8.415**	**7**	**1.029**	**7.23**	**12.87**	**14.76**
**Quartz**	**(2,-9,5)**	**13.233**	**85.57**	**8.414**	**7**	**1.029**	**7.23**	**12.87**	**14.76**
	Equiv. Refl.: (7,-9,-5)								
**Quartz**	**(2,7,-5)**	**13.233**	**85.57**	**8.413**	**7**	**1.029**	**7.23**	**12.87**	**14.76**
**LiNbO3**	**(2,-10,0)**	**12.744**	**73.77**	**8.405**	**7.3**	**3.864**	**28.13**	**48.3**	**55.89**
	Equiv. Refl.: (8,2,0), (10,-2,0), (-2,-8,0), (-8,10,0), (-10,8,0)								
**LiNbO3**	**(0,-8,14)**	**12.765**	**74.09**	**8.372**	**7**	**3.783**	**26.57**	**47.29**	**54.24**
	Equiv. Refl.: (8,0,14), (-8,8,14)								
**LiNbO3**	**(3,-10,-8)**	**12.866**	**75.78**	**8.346**	**7.5**	**3.364**	**25.15**	**42.05**	**48.99**
	Equiv. Refl.: (7,3,-8), (10,-3,-8), (-3,-7,-8), (-7,10,-8), (-10,7,-8)								
**LiNbO3**	**(1,-7,20)**	**12.772**	**74.21**	**8.292**	**7.2**	**3.754**	**27.01**	**46.92**	**54.14**
	Equiv. Refl.: (6,1,20), (7,-1,20), (-1,-6,20)								
**LiNbO3**	**(-6,7,20)**	**12.772**	**74.21**	**8.291**	**7.2**	**3.754**	**27.01**	**46.92**	**54.14**
	Equiv. Refl.: (-7,6,20)								
**LiNbO3**	**(2,8,0)**	**12.744**	**73.77**	**8.29**	**7.3**	**3.864**	**28.13**	**48.3**	**55.89**
	Equiv. Refl.: (8,-10,0), (10,-8,0), (-2,10,0), (-8,-2,0), (-10,2,0)								
**LiNbO3**	**(1,6,-20)**	**12.772**	**74.21**	**8.106**	**7.2**	**3.754**	**27.01**	**46.92**	**54.14**
	Equiv. Refl.: (6,-7,-20), (7,-6,-20), (-1,7,-20), (-6,-1,-20), (-7,1,-20)								
**LiNbO3**	**(3,-7,22)**	**12.974**	**77.81**	**8.076**	**7.5**	**2.867**	**21.52**	**35.83**	**41.79**
	Equiv. Refl.: (4,3,22), (7,-3,22), (-3,-4,22), (-4,7,22), (-7,4,22)								
**Sapph**	**(2,7,10)**	**13.207**	**84.28**	**7.894**	**6.7**	**1.33**	**8.85**	**16.63**	**18.84**
	Equiv. Refl.: (2,-9,-10), (7,2,-10), (7,-9,10), (9,-2,-10), (9,-7,10), (-2,9,10), (-2,-7,-10), (-7,9,-10), (-7,-2,10) (-9,2,10), (-9,7,-10)								
**LiNbO3**	**(2,-10,6)**	**13.023**	**78.87**	**7.839**	**7.3**	**2.611**	**18.98**	**32.64**	**37.76**
	Equiv. Refl.: (8,2,6), (10,-2,6), (-2,-8,6), (-8,10,6), (-10,8,6)								
**LiNbO3**	**(2,8,-6)**	**13.023**	**78.87**	**7.768**	**7.3**	**2.611**	**18.98**	**32.64**	**37.76**
	Equiv. Refl.: (8,-10,-6), (10,-8,-6), (-2,10,-6), (-8,-2,-6), (-10,2,-6)								
**LiNbO3**	**(0,8,-14)**	**12.765**	**74.09**	**7.59**	**7**	**3.783**	**26.57**	**47.29**	**54.24**
	Equiv. Refl.: (8,-8,-14), (-8,0,-14)								
**LiNbO3**	**(0,-9,-6)**	**12.799**	**74.64**	**7.365**	**6.4**	**3.647**	**23.37**	**45.58**	**51.22**
	Equiv. Refl.: (9,0,-6), (-9,9,-6)								
**LiNbO3**	**(4,-9,-14)**	**12.535**	**70.81**	**7.249**	**6.2**	**4.62**	**28.73**	**57.75**	**64.5**
	Equiv. Refl.: (5,4,-14), (9,-4,-14), (-4,-5,-14), (-5,9,-14), (-9,5,-14)								
**LiNbO3**	**(5,6,-1)**	**13.272**	**89.28**	**7.239**	**17.3**	**0.168**	**2.89**	**2.09**	**3.57**
	Equiv. Refl.: (5,-11,1), (6,5,1), (6,-11,-1), (11,-5,1), (11,-6,-1), (-5,11,-1), (-5,-6,1), (-6,11,1), (-6,-5,-1) (-11,5,-1), (-11,6,1)								
**LiNbO3**	**(3,3,-24)**	**12.938**	**77.09**	**7.067**	**6.4**	**3.041**	**19.58**	**38.02**	**42.76**
	Equiv. Refl.: (3,-6,-24), (6,-3,-24), (-3,6,-24), (-3,-3,-24), (-6,3,-24)								
**LiNbO3**	**(0,1,-28)**	**12.598**	**71.65**	**6.984**	**6.2**	**4.403**	**27.08**	**55.04**	**61.34**
	Equiv. Refl.: (1,-1,-28), (-1,0,-28)								
**LiNbO3**	**(4,5,14)**	**12.535**	**70.81**	**6.982**	**6.2**	**4.62**	**28.73**	**57.75**	**64.5**
	Equiv. Refl.: (5,-9,14), (9,-5,14), (-4,9,14), (-5,-4,14), (-9,4,14)								
**LiNbO3**	**(0,-1,28)**	**12.598**	**71.65**	**6.971**	**6.2**	**4.403**	**27.08**	**55.04**	**61.34**
	Equiv. Refl.: (1,0,28), (-1,1,28)								
**Quartz**	**(-6,8,7)**	**13.223**	**85.04**	**6.927**	**5.8**	**1.153**	**6.64**	**14.41**	**15.87**
	Equiv. Refl.: (-6,-2,-7)								

Ei =	13.273	keV						ΔEg	ΔEt
Cryst	Refl	EB	ΘB	∫ IR dΘ	Width	Ei cotΘ	ΔE	2m, 50µm	2m, 50µm
	(h,k,l)	[keV]	[°]	[µrad]	[µrad]	[meV/µrad]	[meV]	[meV]	[meV]
**Quartz**	**(-2,8,-7)**	**13.223**	**85.04**	**6.926**	**5.8**	**1.153**	**6.64**	**14.41**	**15.87**
**Quartz**	**(-2,-6,7)**	**13.223**	**85.04**	**6.925**	**5.8**	**1.153**	**6.64**	**14.41**	**15.87**
**Quartz**	**(8,-6,-7)**	**13.223**	**85.04**	**6.921**	**5.8**	**1.153**	**6.64**	**14.41**	**15.87**
**Quartz**	**(8,-2,7)**	**13.223**	**85.04**	**6.92**	**5.8**	**1.153**	**6.64**	**14.41**	**15.87**
**LiNbO3**	**(1,4,-24)**	**12.481**	**70.11**	**6.906**	**6**	**4.802**	**28.98**	**60.02**	**66.65**
	Equiv. Refl.: (4,-5,-24), (5,-4,-24), (-1,5,-24), (-4,-1,-24), (-5,1,-24)								
**LiNbO3**	**(1,-5,24)**	**12.481**	**70.11**	**6.901**	**6**	**4.802**	**28.98**	**60.02**	**66.65**
	Equiv. Refl.: (4,1,24), (5,-1,24), (-1,-4,24), (-4,5,24), (-5,4,24)								
**LiNbO3**	**(0,9,6)**	**12.799**	**74.64**	**6.875**	**6.4**	**3.647**	**23.37**	**45.58**	**51.22**
	Equiv. Refl.: (9,-9,6), (-9,0,6)								
**LiNbO3**	**(1,4,24)**	**12.481**	**70.11**	**6.865**	**6**	**4.802**	**28.77**	**60.02**	**66.56**
	Equiv. Refl.: (4,-5,24), (5,-4,24), (-1,5,24), (-4,-1,24), (-5,1,24)								
**LiNbO3**	**(1,-5,-24)**	**12.481**	**70.11**	**6.829**	**6**	**4.802**	**28.77**	**60.02**	**66.56**
	Equiv. Refl.: (4,1,-24), (5,-1,-24), (-1,-4,-24), (-4,5,-24), (-5,4,-24)								
**LiNbO3**	**(3,3,24)**	**12.938**	**77.09**	**6.801**	**6.4**	**3.041**	**19.58**	**38.02**	**42.76**
	Equiv. Refl.: (3,-6,24), (6,-3,24), (-3,6,24), (-3,-3,24), (-6,3,24)								
**Quartz**	**(2,-8,7)**	**13.223**	**85.04**	**6.709**	**5.8**	**1.153**	**6.64**	**14.41**	**15.87**
	Equiv. Refl.: (6,2,7), (6,-8,-7)								
**Quartz**	**(2,6,-7)**	**13.223**	**85.04**	**6.708**	**5.8**	**1.153**	**6.64**	**14.41**	**15.87**
**Quartz**	**(-8,6,7)**	**13.223**	**85.04**	**6.705**	**5.8**	**1.153**	**6.64**	**14.41**	**15.87**
**Quartz**	**(-8,2,-7)**	**13.223**	**85.04**	**6.704**	**5.8**	**1.153**	**6.64**	**14.41**	**15.87**
**LiNbO3**	**(4,-10,8)**	**12.639**	**72.22**	**6.664**	**5.8**	**4.257**	**24.75**	**53.21**	**58.69**
	Equiv. Refl.: (6,4,8), (10,-4,8), (-4,-6,8), (-6,10,8), (-10,6,8)								
**LiNbO3**	**(1,8,-10)**	**12.694**	**73.02**	**6.6**	**5.8**	**4.054**	**23.66**	**50.68**	**55.93**
	Equiv. Refl.: (8,-9,-10), (9,-8,-10), (-1,9,-10), (-8,-1,-10), (-9,1,-10)								
**LiNbO3**	**(0,4,26)**	**12.888**	**76.17**	**6.564**	**6**	**3.266**	**19.54**	**40.83**	**45.27**
	Equiv. Refl.: (4,-4,26), (-4,0,26)								
**LiNbO3**	**(0,-2,-28)**	**12.826**	**75.09**	**6.424**	**5.9**	**3.534**	**20.83**	**44.18**	**48.84**
	Equiv. Refl.: (2,0,-28), (-2,2,-28)								
**Sapph**	**(3,6,12)**	**13.244**	**86.18**	**6.404**	**6**	**0.886**	**5.32**	**11.08**	**12.29**
	Equiv. Refl.: (3,6,-12), (3,-9,12), (3,-9,-12), (6,3,12), (6,3,-12), (6,-9,12), (6,-9,-12), (9,-3,12), (9,-3,-12) (9,-6,12), (9,-6,-12), (-3,9,12), (-3,9,-12), (-3,-6,12), (-3,-6,-12), (-6,9,12), (-6,9,-12), (-6,-3,12) (-6,-3,-12), (-9,3,12), (-9,3,-12), (-9,6,12), (-9,6,-12)								
**LiNbO3**	**(2,-2,28)**	**12.826**	**75.09**	**6.386**	**5.9**	**3.534**	**20.83**	**44.18**	**48.84**
**LiNbO3**	**(0,2,28)**	**12.826**	**75.09**	**6.385**	**5.9**	**3.534**	**20.83**	**44.18**	**48.84**
	Equiv. Refl.: (-2,0,28)								
**LiNbO3**	**(1,-9,10)**	**12.694**	**73.02**	**6.356**	**5.8**	**4.054**	**23.66**	**50.68**	**55.93**
	Equiv. Refl.: (8,1,10), (9,-1,10), (-1,-8,10), (-8,9,10), (-9,8,10)								
**LiNbO3**	**(4,6,-8)**	**12.639**	**72.22**	**6.328**	**5.8**	**4.257**	**24.75**	**53.21**	**58.69**
	Equiv. Refl.: (6,-10,-8), (10,-6,-8), (-4,10,-8), (-6,-4,-8), (-10,4,-8)								
**LiNbO3**	**(0,-4,-26)**	**12.888**	**76.17**	**6.302**	**6**	**3.266**	**19.54**	**40.83**	**45.27**
	Equiv. Refl.: (4,0,-26), (-4,4,-26)								
**LiNbO3**	**(4,4,-18)**	**12.554**	**71.05**	**6.064**	**5.4**	**4.557**	**24.51**	**56.96**	**62.01**
	Equiv. Refl.: (4,-8,-18), (8,-4,-18), (-4,8,-18), (-4,-4,-18), (-8,4,-18)								
**LiNbO3**	**(3,7,-4)**	**12.488**	**70.19**	**6.033**	**5.3**	**4.781**	**25.53**	**59.76**	**64.98**
	Equiv. Refl.: (7,-10,-4), (10,-7,-4), (-3,10,-4), (-7,-3,-4), (-10,3,-4)								
**LiNbO3**	**(1,-6,22)**	**12.519**	**70.59**	**5.999**	**5.3**	**4.676**	**24.83**	**58.45**	**63.51**
	Equiv. Refl.: (5,1,22), (6,-1,22), (-1,-5,22), (-5,6,22), (-6,5,22)								
**Quartz**	**(3,-1,-11)**	**13.193**	**83.7**	**5.916**	**4.9**	**1.465**	**7.17**	**18.31**	**19.66**
	Equiv. Refl.: (-1,3,11)								

Ei =	13.273	keV						ΔEg	ΔEt
Cryst	Refl	EB	ΘB	∫ IR dΘ	Width	Ei cotΘ	ΔE	2m, 50µm	2m, 50µm
	(h,k,l)	[keV]	[°]	[µrad]	[µrad]	[meV/µrad]	[meV]	[meV]	[meV]
Quartz	(3,-2,11)	13.193	83.7	5.915	4.9	1.465	7.16	18.31	19.66
	Equiv. Refl.: (-1,-2,-11), (-2,3,-11)								
Quartz	(-2,-1,11)	13.193	83.7	5.914	4.9	1.465	7.16	18.31	19.66
LiNbO3	(3,-10,4)	12.488	70.19	5.913	5.3	4.781	25.53	59.76	64.98
	Equiv. Refl.: (7,3,4), (10,-3,4), (-3,-7,4), (-7,10,4), (-10,7,4)								
LiNbO3	(4,4,18)	12.554	71.05	5.903	5.4	4.557	24.51	56.96	62.01
	Equiv. Refl.: (4,-8,18), (8,-4,18), (-4,8,18), (-4,-4,18), (-8,4,18)								
Quartz	(-3,5,-10)	13.111	81.04	5.901	4.7	2.093	9.8	26.16	27.94
	Equiv. Refl.: (-3,-2,10)								
Quartz	(5,-2,-10)	13.111	81.04	5.9	4.7	2.093	9.8	26.16	27.94
	Equiv. Refl.: (5,-3,10), (-2,-3,-10)								
Quartz	(-2,5,10)	13.111	81.04	5.899	4.7	2.093	9.79	26.16	27.94
LiNbO3	(0,9,0)	12.514	70.54	5.873	5.2	4.691	24.22	58.64	63.44
	Equiv. Refl.: (9,-9,0), (-9,0,0)								
Quartz	(3,2,-10)	13.111	81.04	5.837	4.7	2.093	9.8	26.16	27.94
Quartz	(3,-5,10)	13.111	81.04	5.836	4.7	2.093	9.8	26.16	27.94
Quartz	(-5,2,10)	13.111	81.04	5.835	4.7	2.093	9.8	26.16	27.94
	Equiv. Refl.: (-5,3,-10)								
LiNbO3	(1,5,-22)	12.519	70.59	5.834	5.3	4.676	24.83	58.45	63.51
	Equiv. Refl.: (5,-6,-22), (6,-5,-22), (-1,6,-22), (-5,-1,-22), (-6,1,-22), (2,3,10), (2,-5,-10)								
Quartz	(-3,1,11)	13.193	83.7	5.813	4.9	1.465	7.17	18.31	19.66
Quartz	(1,-3,-11)	13.193	83.7	5.812	4.9	1.465	7.16	18.31	19.66
	Equiv. Refl.: (-3,2,-11)								
Quartz	(1,2,11)	13.193	83.7	5.811	4.9	1.465	7.16	18.31	19.66
Quartz	(2,-3,11)	13.193	83.7	5.81	4.9	1.465	7.16	18.31	19.66
Quartz	(2,1,-11)	13.193	83.7	5.809	4.9	1.465	7.16	18.31	19.66
LiNbO3	(1,3,-26)	12.661	72.54	5.692	5	4.175	20.73	52.19	56.15
	Equiv. Refl.: (3,-4,-26), (4,-3,-26), (-1,4,-26), (-3,-1,-26), (-4,1,-26)								
LiNbO3	(0,-9,0)	12.514	70.54	5.573	5.2	4.691	24.22	58.64	63.44
	Equiv. Refl.: (9,0,0), (-9,9,0)								
LiNbO3	(1,-4,26)	12.661	72.54	5.414	5	4.175	20.73	52.19	56.15
	Equiv. Refl.: (3,1,26), (4,-1,26), (-1,-3,26), (-3,4,26), (-4,3,26)								
Quartz	(8,0,-5)	12.99	78.16	5.351	4.2	2.784	11.7	34.79	36.71
	Equiv. Refl.: (8,-8,5), (-8,0,5), (-8,8,-5)								
Quartz	(0,8,5)	12.99	78.16	5.349	4.2	2.784	11.7	34.79	36.71
	Equiv. Refl.: (0,-8,-5)								
Sapph	(1,8,-4)	12.995	78.25	5.298	4.3	2.761	11.96	34.52	36.53
	Equiv. Refl.: (1,-9,4), (8,1,4), (8,-9,-4), (9,-1,4), (9,-8,-4), (-1,9,-4), (-1,-8,4), (-8,9,4), (-8,-1,-4) (-9,1,-4), (-9,8,4)								
Quartz	(-1,7,8)	13.247	86.41	5.215	4.5	0.832	3.77	10.4	11.07
	Equiv. Refl.: (-1,-6,-8)								
Quartz	(-6,7,-8)	13.247	86.41	5.212	4.5	0.832	3.77	10.4	11.07
Quartz	(-6,-1,8)	13.247	86.41	5.208	4.5	0.832	3.77	10.4	11.07
Quartz	(7,-1,-8)	13.247	86.41	5.206	4.5	0.832	3.77	10.4	11.07
Quartz	(7,-6,8)	13.247	86.41	5.205	4.5	0.832	3.77	10.4	11.07
Quartz	(5,5,0)	12.618	71.92	5.072	3.8	4.332	16.59	54.15	56.64
	Equiv. Refl.: (5,-10,0), (7,-6,-8), (-10,5,0)								
Quartz	(-6,7,8)	13.247	86.41	5.071	4.6	0.832	3.79	10.4	11.07
	Equiv. Refl.: (-6,-1,-8)								

Ei =	13.273	keV						ΔEg	ΔEt
Cryst	Refl	EB	ΘB	∫ IR dΘ	Width	Ei cotΘ	ΔE	2m, 50µm	2m, 50µm
	(h,k,l)	[keV]	[°]	[µrad]	[µrad]	[meV/µrad]	[meV]	[meV]	[meV]
**Quartz**	**(7,-1,8)**	**13.247**	**86.41**	**5.07**	**4.6**	**0.832**	**3.79**	**10.4**	**11.07**
Equiv. Refl.: (-1,-6,8)									
**Quartz**	**(-1,7,-8)**	**13.247**	**86.41**	**5.069**	**4.6**	**0.832**	**3.79**	**10.4**	**11.07**
**Quartz**	**(10,-5,0)**	**12.618**	**71.92**	**5.068**	**3.8**	**4.332**	**16.59**	**54.15**	**56.64**
Equiv. Refl.: (-5,10,0), (-5,-5,0)									
**Quartz**	**(2,5,-8)**	**12.923**	**76.8**	**5.028**	**3.9**	**3.112**	**12.19**	**38.9**	**40.77**
Equiv. Refl.: (2,-7,8)									
**Quartz**	**(-7,2,-8)**	**12.923**	**76.8**	**5.027**	**3.9**	**3.112**	**12.19**	**38.9**	**40.77**
Equiv. Refl.: (-7,5,8)									
**Quartz**	**(5,2,8)**	**12.923**	**76.8**	**5.025**	**3.9**	**3.112**	**12.19**	**38.9**	**40.77**
Equiv. Refl.: (5,-7,-8)									
**Quartz**	**(-2,7,-8)**	**12.923**	**76.8**	**5.024**	**3.9**	**3.112**	**12.19**	**38.9**	**40.77**
Equiv. Refl.: (-2,-5,8)									
**Quartz**	**(7,-2,8)**	**12.923**	**76.8**	**5.023**	**3.9**	**3.112**	**12.19**	**38.9**	**40.77**
**Quartz**	**(7,-5,-8)**	**12.923**	**76.8**	**5.022**	**3.9**	**3.112**	**12.19**	**38.9**	**40.77**
**Quartz**	**(-5,7,8)**	**12.923**	**76.8**	**5.021**	**3.9**	**3.112**	**12.19**	**38.9**	**40.77**
Equiv. Refl.: (-5,-2,-8)									
**Quartz**	**(-7,6,8)**	**13.247**	**86.41**	**4.906**	**4.6**	**0.832**	**3.79**	**10.4**	**11.07**
**Quartz**	**(6,1,8)**	**13.247**	**86.41**	**4.905**	**4.6**	**0.832**	**3.79**	**10.4**	**11.07**
Equiv. Refl.: (6,-7,-8)									
**Quartz**	**(-7,1,-8)**	**13.247**	**86.41**	**4.903**	**4.6**	**0.832**	**3.79**	**10.4**	**11.07**
**Quartz**	**(1,6,-8)**	**13.247**	**86.41**	**4.902**	**4.6**	**0.832**	**3.79**	**10.4**	**11.07**
**Quartz**	**(1,-7,8)**	**13.247**	**86.41**	**4.901**	**4.6**	**0.832**	**3.79**	**10.4**	**11.07**
**Quartz**	**(1,6,8)**	**13.247**	**86.41**	**4.751**	**4.5**	**0.832**	**3.77**	**10.4**	**11.07**
Equiv. Refl.: (1,-7,-8)									
**Quartz**	**(6,-7,8)**	**13.247**	**86.41**	**4.749**	**4.5**	**0.832**	**3.77**	**10.4**	**11.07**
**Quartz**	**(6,1,-8)**	**13.247**	**86.41**	**4.745**	**4.5**	**0.832**	**3.77**	**10.4**	**11.07**
**Quartz**	**(-7,1,8)**	**13.247**	**86.41**	**4.742**	**4.5**	**0.832**	**3.77**	**10.4**	**11.07**
**Quartz**	**(-7,6,-8)**	**13.247**	**86.41**	**4.741**	**4.5**	**0.832**	**3.77**	**10.4**	**11.07**
**Sapph**	**(4,6,-2)**	**13.15**	**82.19**	**4.672**	**4**	**1.82**	**7.2**	**22.75**	**23.86**
Equiv. Refl.: (4,-10,2), (6,4,2), (6,-10,-2), (10,-4,2), (10,-6,-2), (-4,10,-2), (-4,-6,2), (-6,10,2), (-6,-4,-2) (-10,4,-2), (-10,6,2)									
**Sapph**	**(1,1,27)**	**13.148**	**82.12**	**4.614**	**3.9**	**1.837**	**7.17**	**22.96**	**24.06**
Equiv. Refl.: (1,1,-27), (1,-2,27), (1,-2,-27), (2,-1,27), (2,-1,-27), (-1,2,27), (-1,2,-27), (-1,-1,27), (-1,-1,-27) (-2,1,27), (-2,1,-27)									
**Sapph**	**(2,6,14)**	**12.741**	**73.73**	**4.473**	**3.4**	**3.874**	**13.35**	**48.43**	**50.23**
Equiv. Refl.: (2,-8,-14), (6,2,-14), (6,-8,14), (8,-2,-14), (8,-6,14), (-2,8,14), (-2,-6,-14), (-6,8,-14), (-6,-2,14) (-8,2,14), (-8,6,-14)									
**Sapph**	**(2,2,24)**	**12.585**	**71.47**	**4.467**	**3.4**	**4.449**	**15.1**	**55.62**	**57.63**
Equiv. Refl.: (2,2,-24), (2,-4,24), (2,-4,-24), (4,-2,24), (4,-2,-24), (-2,4,24), (-2,4,-24), (-2,-2,24), (-2,-2,-24) (-4,2,24), (-4,2,-24)									
**Sapph**	**(3,5,16)**	**13.008**	**78.54**	**4.412**	**3.6**	**2.691**	**9.57**	**33.63**	**34.97**
Equiv. Refl.: (3,-8,-16), (5,3,-16), (5,-8,16), (8,-3,-16), (8,-5,16), (-3,8,16), (-3,-5,-16), (-5,8,-16), (-5,-3,16) (-8,3,16), (-8,5,-16)									
**Quartz**	**(6,2,-7)**	**13.223**	**85.04**	**4.331**	**3.7**	**1.153**	**4.28**	**14.41**	**15.04**
Equiv. Refl.: (6,-8,7), (-8,2,7), (-8,6,-7)									
**Quartz**	**(2,-8,-7)**	**13.223**	**85.04**	**4.326**	**3.7**	**1.153**	**4.28**	**14.41**	**15.03**
**Quartz**	**(2,6,7)**	**13.223**	**85.04**	**4.324**	**3.7**	**1.153**	**4.27**	**14.41**	**15.03**
**Sapph**	**(4,6,4)**	**13.253**	**86.89**	**4.136**	**4.5**	**0.721**	**3.23**	**9.01**	**9.57**
Equiv. Refl.: (4,-10,-4), (6,4,-4), (6,-10,4), (10,-4,-4), (10,-6,4), (-4,10,4), (-4,-6,-4), (-6,10,-4), (-6,-4,4) (-10,4,4), (-10,6,-4)									

Ei =	13.273	keV						ΔEg	ΔEt
Cryst	Refl	EB	ΘB	∫ IR dΘ	Width	Ei cotΘ	ΔE	2m, 50µm	2m, 50µm
	(h,k,l)	[keV]	[°]	[µrad]	[µrad]	[meV/µrad]	[meV]	[meV]	[meV]
**Quartz**	**(-6,4,-9)**	**12.884**	**76.1**	**4.057**	**3.1**	**3.285**	**10.1**	**41.06**	**42.28**
**Quartz**	**(2,-6,-9)**	**12.884**	**76.1**	**4.056**	**3.1**	**3.285**	**10.1**	**41.06**	**42.28**
	Equiv. Refl.: (-6,2,9)								
**Quartz**	**(2,4,9)**	**12.884**	**76.1**	**4.055**	**3.1**	**3.285**	**10.1**	**41.06**	**42.28**
	Equiv. Refl.: (4,2,-9), (4,-6,9)								
**Quartz**	**(9,0,-1)**	**13.163**	**82.62**	**4.051**	**3.4**	**1.72**	**5.77**	**21.5**	**22.26**
	Equiv. Refl.: (9,-9,1), (-9,0,1), (-9,9,-1)								
**Quartz**	**(0,9,1)**	**13.163**	**82.62**	**4.05**	**3.4**	**1.72**	**5.76**	**21.5**	**22.26**
	Equiv. Refl.: (0,-9,-1)								
**Quartz**	**(8,-2,-7)**	**13.223**	**85.04**	**4.029**	**3.7**	**1.153**	**4.28**	**14.41**	**15.04**
	Equiv. Refl.: (8,-6,7)								
**Quartz**	**(-6,8,-7)**	**13.223**	**85.04**	**4.028**	**3.7**	**1.153**	**4.28**	**14.41**	**15.04**
	Equiv. Refl.: (-6,-2,7)								
**Quartz**	**(-2,8,7)**	**13.223**	**85.04**	**4.023**	**3.7**	**1.153**	**4.28**	**14.41**	**15.03**
**Quartz**	**(-2,-6,-7)**	**13.223**	**85.04**	**4.021**	**3.7**	**1.153**	**4.27**	**14.41**	**15.03**
**Quartz**	**(-10,3,-2)**	**13.152**	**82.24**	**3.986**	**3.3**	**1.808**	**5.95**	**22.6**	**23.37**
	Equiv. Refl.: (-10,7,2)								
**Quartz**	**(3,7,-2)**	**13.152**	**82.24**	**3.984**	**3.3**	**1.808**	**5.95**	**22.6**	**23.36**
	Equiv. Refl.: (3,-10,2), (7,3,2), (7,-10,-2)								
**Quartz**	**(6,-4,9)**	**12.884**	**76.1**	**3.93**	**3.1**	**3.285**	**10.1**	**41.06**	**42.28**
**Quartz**	**(6,-2,-9)**	**12.884**	**76.1**	**3.929**	**3.1**	**3.285**	**10.1**	**41.06**	**42.28**
**Quartz**	**(-2,6,9)**	**12.884**	**76.1**	**3.928**	**3.1**	**3.285**	**10.1**	**41.06**	**42.28**
	Equiv. Refl.: (-2,-4,-9), (-4,6,-9), (-4,-2,9)								
**Quartz**	**(10,-3,2)**	**13.152**	**82.24**	**3.906**	**3.3**	**1.808**	**5.95**	**22.6**	**23.37**
	Equiv. Refl.: (10,-7,-2)								
**Quartz**	**(-3,10,-2)**	**13.152**	**82.24**	**3.905**	**3.3**	**1.808**	**5.95**	**22.6**	**23.36**
	Equiv. Refl.: (-3,-7,2)								
**Quartz**	**(-7,10,2)**	**13.152**	**82.24**	**3.904**	**3.3**	**1.808**	**5.95**	**22.6**	**23.36**
	Equiv. Refl.: (-7,-3,-2)								
**Quartz**	**(9,-4,-5)**	**12.743**	**73.75**	**3.87**	**3**	**3.868**	**11.42**	**48.35**	**49.68**
	Equiv. Refl.: (9,-5,5)								
**Quartz**	**(-4,9,5)**	**12.743**	**73.75**	**3.869**	**3**	**3.868**	**11.41**	**48.35**	**49.68**
	Equiv. Refl.: (-4,-5,-5), (-5,9,-5), (-5,-4,5)								
**Quartz**	**(-9,4,5)**	**12.743**	**73.75**	**3.857**	**3**	**3.868**	**11.42**	**48.35**	**49.68**
**Quartz**	**(4,-9,-5)**	**12.743**	**73.75**	**3.856**	**3**	**3.868**	**11.41**	**48.35**	**49.68**
	Equiv. Refl.: (5,4,-5), (5,-9,5), (-9,5,-5)								
**Quartz**	**(4,5,5)**	**12.743**	**73.75**	**3.855**	**3**	**3.868**	**11.41**	**48.35**	**49.68**
**Quartz**	**(6,4,1)**	**12.753**	**73.92**	**3.716**	**2.8**	**3.827**	**10.71**	**47.84**	**49.03**
	Equiv. Refl.: (6,-10,-1), (-10,4,-1), (-10,6,1)								
**Quartz**	**(4,6,-1)**	**12.753**	**73.92**	**3.715**	**2.8**	**3.827**	**10.71**	**47.84**	**49.03**
	Equiv. Refl.: (4,-10,1)								
**Quartz**	**(10,-4,1)**	**12.753**	**73.92**	**3.6**	**2.8**	**3.827**	**10.71**	**47.84**	**49.03**
	Equiv. Refl.: (10,-6,-1), (-6,10,1), (-6,-4,-1)								
**Quartz**	**(-4,10,-1)**	**12.753**	**73.92**	**3.599**	**2.8**	**3.827**	**10.71**	**47.84**	**49.03**
	Equiv. Refl.: (-4,-6,1)								
**Quartz**	**(-1,-1,11)**	**12.867**	**75.79**	**3.48**	**2.7**	**3.36**	**9.09**	**42**	**42.97**
**Quartz**	**(-1,2,-11)**	**12.867**	**75.79**	**3.479**	**2.7**	**3.36**	**9.09**	**42**	**42.97**
	Equiv. Refl.: (-1,-1,-11)								
**Quartz**	**(-1,2,11)**	**12.867**	**75.79**	**3.478**	**2.7**	**3.36**	**9.09**	**42**	**42.97**

Ei = Cryst	13.273 Refl (h,k,l)	keV EB [keV]	ΘB [°]	∫ IR dΘ [µrad]	Width [µrad]	Ei cotΘ [meV/µrad]	ΔE [meV]	ΔEg 2m, 50µm [meV]	ΔEt 2m, 50µm [meV]
**Quartz**	**(2,-1,11)**	**12.867**	**75.79**	**3.477**	**2.7**	**3.36**	**9.09**	**42**	**42.97**
	Equiv. Refl.: (2,-1,-11)								
**Quartz**	**(1,1,-11)**	**12.867**	**75.79**	**3.447**	**2.7**	**3.36**	**9.09**	**42**	**42.97**
**Quartz**	**(1,1,11)**	**12.867**	**75.79**	**3.446**	**2.7**	**3.36**	**9.09**	**42**	**42.97**
	Equiv. Refl.: (1,-2,11)								
**Quartz**	**(1,-2,-11)**	**12.867**	**75.79**	**3.445**	**2.7**	**3.36**	**9.09**	**42**	**42.97**
**Quartz**	**(-2,1,11)**	**12.867**	**75.79**	**3.444**	**2.7**	**3.36**	**9.09**	**42**	**42.97**
	Equiv. Refl.: (-2,1,-11)								
**Sapph**	**(1,8,5)**	**13.073**	**80.05**	**3.397**	**2.8**	**2.328**	**6.51**	**29.1**	**29.82**
	Equiv. Refl.: (1,-9,-5), (8,1,-5), (8,-9,5), (9,-1,-5), (9,-8,5), (-1,9,5), (-1,-8,-5), (-8,9,-5), (-8,-1,5) (-9,1,5), (-9,8,-5)								
**Quartz**	**(8,0,5)**	**12.99**	**78.16**	**3.275**	**2.6**	**2.784**	**7.37**	**34.79**	**35.56**
	Equiv. Refl.: (8,-8,-5), (-8,0,-5), (-8,8,5)								
**Quartz**	**(0,8,-5)**	**12.99**	**78.16**	**3.274**	**2.6**	**2.784**	**7.36**	**34.79**	**35.56**
	Equiv. Refl.: (0,-8,5)								
**Quartz**	**(0,2,11)**	**12.949**	**77.32**	**3.257**	**2.6**	**2.986**	**7.8**	**37.33**	**38.13**
	Equiv. Refl.: (0,-2,-11)								
**Quartz**	**(2,0,-11)**	**12.949**	**77.32**	**3.252**	**2.6**	**2.986**	**7.79**	**37.33**	**38.13**
	Equiv. Refl.: (-2,0,11)								
**Quartz**	**(2,-2,11)**	**12.949**	**77.32**	**3.251**	**2.6**	**2.986**	**7.79**	**37.33**	**38.13**
	Equiv. Refl.: (-2,2,-11)								
**Quartz**	**(-8,5,-7)**	**12.98**	**77.94**	**3.18**	**2.6**	**2.835**	**7.27**	**35.44**	**36.18**
**Quartz**	**(-8,3,7)**	**12.98**	**77.94**	**3.179**	**2.6**	**2.835**	**7.27**	**35.44**	**36.18**
**Quartz**	**(3,5,7)**	**12.98**	**77.94**	**3.178**	**2.6**	**2.835**	**7.27**	**35.44**	**36.18**
	Equiv. Refl.: (3,-8,-7)								
**Quartz**	**(5,3,-7)**	**12.98**	**77.94**	**3.174**	**2.6**	**2.835**	**7.26**	**35.44**	**36.18**
	Equiv. Refl.: (5,-8,7)								
**Quartz**	**(3,6,5)**	**12.908**	**76.54**	**3.157**	**2.5**	**3.177**	**7.8**	**39.71**	**40.47**
	Equiv. Refl.: (3,-9,-5), (6,3,-5), (6,-9,5), (8,-3,-7), (8,-5,7)								
**Quartz**	**(-3,8,7)**	**12.98**	**77.94**	**3.155**	**2.6**	**2.835**	**7.27**	**35.44**	**36.18**
	Equiv. Refl.: (-3,-5,-7), (-9,3,5)								
**Quartz**	**(-9,6,-5)**	**12.908**	**76.54**	**3.154**	**2.5**	**3.177**	**7.79**	**39.71**	**40.46**
**Quartz**	**(-5,8,-7)**	**12.98**	**77.94**	**3.152**	**2.6**	**2.835**	**7.26**	**35.44**	**36.18**
	Equiv. Refl.: (-5,-3,7)								
**Quartz**	**(-3,9,5)**	**12.908**	**76.54**	**3.148**	**2.5**	**3.177**	**7.8**	**39.71**	**40.47**
	Equiv. Refl.: (-3,-6,-5), (-6,9,-5), (-6,-3,5)								
**Quartz**	**(9,-3,-5)**	**12.908**	**76.54**	**3.145**	**2.5**	**3.177**	**7.79**	**39.71**	**40.46**
	Equiv. Refl.: (9,-6,5)								
**Quartz**	**(7,-4,-8)**	**12.757**	**73.97**	**3.092**	**2.3**	**3.812**	**8.79**	**47.66**	**48.46**
	Equiv. Refl.: (-4,7,8), (-4,-3,-8)								
**Quartz**	**(7,-3,8)**	**12.757**	**73.97**	**3.091**	**2.3**	**3.812**	**8.79**	**47.66**	**48.46**
	Equiv. Refl.: (-3,7,-8), (-3,-4,8)								
**Quartz**	**(-7,6,7)**	**12.48**	**70.09**	**3.059**	**2.3**	**4.807**	**10.99**	**60.08**	**61.08**
**Quartz**	**(-7,1,-7)**	**12.48**	**70.09**	**3.058**	**2.3**	**4.807**	**10.99**	**60.08**	**61.08**
**Quartz**	**(1,6,-7)**	**12.48**	**70.09**	**3.057**	**2.3**	**4.807**	**10.99**	**60.08**	**61.08**
	Equiv. Refl.: (1,-7,7), (4,-7,-8), (6,1,7)								
**Quartz**	**(4,3,8)**	**12.757**	**73.97**	**3.056**	**2.3**	**3.812**	**8.79**	**47.66**	**48.46**
	Equiv. Refl.: (6,-7,-7), (-7,4,8)								
**Quartz**	**(3,4,-8)**	**12.757**	**73.97**	**3.055**	**2.3**	**3.812**	**8.79**	**47.66**	**48.46**
	Equiv. Refl.: (3,-7,8), (-7,3,-8)								

Ei = Cryst	13.273 Refl (h,k,l)	keV EB [keV]	ΘB [°]	∫ IR dΘ [μrad]	Width [μrad]	Ei cotΘ [meV/μrad]	ΔE [meV]	ΔEg 2m, 50μm [meV]	ΔEt 2m, 50μm [meV]
**Quartz**	**(2,4,-9)**	**12.884**	**76.1**	**3.04**	**2.4**	**3.285**	**7.93**	**41.06**	**41.82**
	Equiv. Refl.: (2,-6,9)								
**Quartz**	**(-6,2,-9)**	**12.884**	**76.1**	**3.037**	**2.4**	**3.285**	**7.92**	**41.06**	**41.82**
**Quartz**	**(4,2,9)**	**12.884**	**76.1**	**3.036**	**2.4**	**3.285**	**7.92**	**41.06**	**41.82**
	Equiv. Refl.: (4,-6,-9), (-6,4,9)								
**Quartz**	**(7,-1,7)**	**12.48**	**70.09**	**3.032**	**2.3**	**4.807**	**10.99**	**60.08**	**61.08**
	Equiv. Refl.: (7,-6,-7)								
**Quartz**	**(-2,6,-9)**	**12.884**	**76.1**	**3.031**	**2.4**	**3.285**	**7.93**	**41.06**	**41.82**
	Equiv. Refl.: (-2,-4,9)								
**Quartz**	**(-1,7,-7)**	**12.48**	**70.09**	**3.03**	**2.3**	**4.807**	**10.99**	**60.08**	**61.08**
	Equiv. Refl.: (-1,-6,7), (-6,-1,-7)								
**Quartz**	**(-6,7,7)**	**12.48**	**70.09**	**3.029**	**2.3**	**4.807**	**10.99**	**60.08**	**61.08**
**Quartz**	**(6,-2,9)**	**12.884**	**76.1**	**3.028**	**2.4**	**3.285**	**7.92**	**41.06**	**41.82**
	Equiv. Refl.: (-4,-2,-9)								
**Quartz**	**(6,-4,-9)**	**12.884**	**76.1**	**3.027**	**2.4**	**3.285**	**7.92**	**41.06**	**41.82**
	Equiv. Refl.: (-4,6,9)								
**Quartz**	**(1,-8,-6)**	**12.975**	**77.85**	**2.989**	**2.2**	**2.859**	**6.41**	**35.73**	**36.3**
**Quartz**	**(1,7,6)**	**12.975**	**77.85**	**2.988**	**2.2**	**2.859**	**6.41**	**35.73**	**36.3**
	Equiv. Refl.: (-8,1,6), (-8,7,-6)								
**Quartz**	**(7,1,-6)**	**12.975**	**77.85**	**2.987**	**2.2**	**2.859**	**6.41**	**35.73**	**36.3**
**Quartz**	**(7,-8,6)**	**12.975**	**77.85**	**2.986**	**2.2**	**2.859**	**6.41**	**35.73**	**36.3**
**Quartz**	**(0,8,-4)**	**12.526**	**70.69**	**2.98**	**2.5**	**4.65**	**11.83**	**58.13**	**59.32**
	Equiv. Refl.: (0,-8,4)								
**Quartz**	**(8,0,4)**	**12.526**	**70.69**	**2.979**	**2.5**	**4.65**	**11.83**	**58.13**	**59.32**
	Equiv. Refl.: (8,-8,-4), (-8,0,-4), (-8,8,4)								
**Quartz**	**(8,-1,6)**	**12.975**	**77.85**	**2.945**	**2.6**	**2.859**	**7.36**	**35.73**	**36.48**
	Equiv. Refl.: (8,-7,-6)								
**Quartz**	**(-7,8,6)**	**12.975**	**77.85**	**2.944**	**2.6**	**2.859**	**7.36**	**35.73**	**36.48**
	Equiv. Refl.: (-7,-1,-6)								
**Quartz**	**(-1,-7,6)**	**12.975**	**77.85**	**2.943**	**2.6**	**2.859**	**7.36**	**35.73**	**36.48**
**Quartz**	**(-1,8,-6)**	**12.975**	**77.85**	**2.942**	**2.6**	**2.859**	**7.36**	**35.73**	**36.48**
**Sapph**	**(1,2,26)**	**13.032**	**79.07**	**2.818**	**2.4**	**2.563**	**6.02**	**32.04**	**32.6**
	Equiv. Refl.: (1,-3,-26), (2,1,-26), (2,-3,26), (3,-1,-26), (3,-2,26), (-1,3,26), (-1,-2,-26), (-2,3,-26), (-2,-1,26) (-3,1,26), (-3,2,-26)								
**Quartz**	**(-6,1,-9)**	**13.129**	**81.56**	**2.817**	**2.2**	**1.97**	**4.43**	**24.63**	**25.02**
	Equiv. Refl.: (-6,5,9)								
**Quartz**	**(5,-6,-9)**	**13.129**	**81.56**	**2.814**	**2.2**	**1.97**	**4.43**	**24.63**	**25.02**
**Quartz**	**(5,1,9)**	**13.129**	**81.56**	**2.813**	**2.2**	**1.97**	**4.43**	**24.63**	**25.02**
**Quartz**	**(1,5,-9)**	**13.129**	**81.56**	**2.812**	**2.2**	**1.97**	**4.43**	**24.63**	**25.02**
**Quartz**	**(1,-6,9)**	**13.129**	**81.56**	**2.811**	**2.2**	**1.97**	**4.43**	**24.63**	**25.02**
**Quartz**	**(-8,1,-6)**	**12.975**	**77.85**	**2.809**	**2.6**	**2.859**	**7.36**	**35.73**	**36.48**
	Equiv. Refl.: (-8,7,6)								
**Quartz**	**(7,1,6)**	**12.975**	**77.85**	**2.808**	**2.6**	**2.859**	**7.36**	**35.73**	**36.48**
	Equiv. Refl.: (7,-8,-6)								
**Quartz**	**(1,7,-6)**	**12.975**	**77.85**	**2.807**	**2.6**	**2.859**	**7.36**	**35.73**	**36.48**
	Equiv. Refl.: (1,-8,6)								
**Quartz**	**(9,-1,2)**	**12.658**	**72.49**	**2.784**	**2.1**	**4.187**	**8.74**	**52.34**	**53.06**
	Equiv. Refl.: (9,-8,-2), (-1,9,-2), (-1,-8,2), (-8,9,2), (-8,-1,-2)								
**Quartz**	**(8,1,2)**	**12.658**	**72.49**	**2.774**	**2.1**	**4.187**	**8.74**	**52.34**	**53.06**
	Equiv. Refl.: (8,-9,-2)								

Ei =	13.273	keV						ΔEg	ΔEt
Cryst	Refl	EB	ΘB	∫ IR dΘ	Width	Ei cotΘ	ΔE	2m, 50µm	2m, 50µm
	(h,k,l)	[keV]	[°]	[µrad]	[µrad]	[meV/µrad]	[meV]	[meV]	[meV]
**Quartz**	**(1,8,-2)**	**12.658**	**72.49**	**2.773**	**2.1**	**4.187**	**8.74**	**52.34**	**53.06**
	Equiv. Refl.: (1,-9,2), (-9,1,-2), (-9,8,2)								
**Quartz**	**(-1,8,6)**	**12.975**	**77.85**	**2.772**	**2.2**	**2.859**	**6.41**	**35.73**	**36.3**
**Quartz**	**(8,-1,-6)**	**12.975**	**77.85**	**2.771**	**2.2**	**2.859**	**6.41**	**35.73**	**36.3**
	Equiv. Refl.: (8,-7,6), (-1,-7,-6)								
**Quartz**	**(-7,8,-6)**	**12.975**	**77.85**	**2.77**	**2.2**	**2.859**	**6.41**	**35.73**	**36.3**
	Equiv. Refl.: (-7,-1,6)								
**Quartz**	**(4,-4,-10)**	**12.866**	**75.77**	**2.759**	**2.1**	**3.366**	**7.15**	**42.07**	**42.67**
**Quartz**	**(4,0,10)**	**12.866**	**75.77**	**2.758**	**2.1**	**3.366**	**7.15**	**42.07**	**42.67**
	Equiv. Refl.: (-4,0,-10), (-4,4,10)								
**Quartz**	**(0,4,-10)**	**12.866**	**75.77**	**2.757**	**2.1**	**3.366**	**7.15**	**42.07**	**42.67**
	Equiv. Refl.: (0,-4,10)								
**Sapph**	**(5,5,0)**	**13.029**	**78.99**	**2.755**	**2.6**	**2.583**	**6.66**	**32.28**	**32.96**
	Equiv. Refl.: (5,-10,0), (10,-5,0), (-5,10,0), (-5,-5,0), (-10,5,0)								
**LiNbO3**	**(4,5,17)**	**13.256**	**87.13**	**2.738**	**5.6**	**0.665**	**3.71**	**8.31**	**9.1**
	Equiv. Refl.: (4,-9,-17), (5,4,-17), (5,-9,17), (9,-4,-17), (9,-5,17), (-4,9,17), (-4,-5,-17), (-5,9,-17), (-5,-4,17) (-9,4,17), (-9,5,-17)								
**Quartz**	**(-2,9,4)**	**12.778**	**74.3**	**2.574**	**2.2**	**3.73**	**8.31**	**46.62**	**47.36**
	Equiv. Refl.: (-2,-7,-4), (-7,9,-4), (-7,-2,4)								
**Quartz**	**(9,-2,-4)**	**12.778**	**74.3**	**2.572**	**2.2**	**3.73**	**8.31**	**46.62**	**47.36**
	Equiv. Refl.: (9,-7,4)								
**Sapph**	**(0,8,10)**	**12.947**	**77.28**	**2.562**	**2.4**	**2.996**	**7.14**	**37.45**	**38.13**
	Equiv. Refl.: (0,-8,-10), (8,0,-10), (8,-8,10), (-8,0,10), (-8,8,-10)								
**Quartz**	**(10,-4,3)**	**13.16**	**82.5**	**2.523**	**2.3**	**1.746**	**4.06**	**21.83**	**22.2**
	Equiv. Refl.: (10,-6,-3), (-4,10,-3), (-4,-6,3)								
**Quartz**	**(-6,10,3)**	**13.16**	**82.5**	**2.522**	**2.3**	**1.746**	**4.06**	**21.83**	**22.2**
	Equiv. Refl.: (-6,-4,-3)								
**Quartz**	**(2,7,4)**	**12.778**	**74.3**	**2.487**	**2.2**	**3.73**	**8.31**	**46.62**	**47.36**
	Equiv. Refl.: (2,-9,-4), (7,2,-4), (7,-9,4)								
**Quartz**	**(-9,2,4)**	**12.778**	**74.3**	**2.485**	**2.2**	**3.73**	**8.31**	**46.62**	**47.36**
	Equiv. Refl.: (-9,7,-4)								
**Quartz**	**(3,7,0)**	**12.95**	**77.33**	**2.476**	**2.2**	**2.983**	**6.46**	**37.29**	**37.84**
	Equiv. Refl.: (3,-10,0), (7,3,0), (7,-10,0), (-10,3,0), (-10,7,0)								
**Quartz**	**(4,6,-3)**	**13.16**	**82.5**	**2.409**	**2.3**	**1.746**	**4.06**	**21.83**	**22.2**
	Equiv. Refl.: (4,-10,3), (-10,6,3)								
**Quartz**	**(6,4,3)**	**13.16**	**82.5**	**2.408**	**2.3**	**1.746**	**4.06**	**21.83**	**22.2**
	Equiv. Refl.: (-10,4,-3)								
**Quartz**	**(6,-10,-3)**	**13.16**	**82.5**	**2.407**	**2.3**	**1.746**	**4.06**	**21.83**	**22.2**
**Quartz**	**(0,9,-1)**	**13.163**	**82.62**	**2.39**	**2.3**	**1.72**	**3.91**	**21.5**	**21.85**
	Equiv. Refl.: (0,-9,1), (9,0,1), (9,-9,-1), (-9,0,-1), (-9,9,1)								
**Quartz**	**(6,-1,9)**	**13.129**	**81.56**	**2.375**	**2.2**	**1.97**	**4.43**	**24.63**	**25.02**
	Equiv. Refl.: (6,-5,-9)								
**Quartz**	**(-5,6,9)**	**13.129**	**81.56**	**2.374**	**2.2**	**1.97**	**4.43**	**24.63**	**25.02**
**Quartz**	**(-1,-5,9)**	**13.129**	**81.56**	**2.373**	**2.2**	**1.97**	**4.43**	**24.63**	**25.02**
	Equiv. Refl.: (-5,-1,-9)								
**Quartz**	**(-1,6,-9)**	**13.129**	**81.56**	**2.372**	**2.2**	**1.97**	**4.43**	**24.63**	**25.02**
**Quartz**	**(0,8,4)**	**12.526**	**70.69**	**2.343**	**2**	**4.65**	**9.27**	**58.13**	**58.87**
	Equiv. Refl.: (0,-8,-4)								
**Quartz**	**(8,0,-4)**	**12.526**	**70.69**	**2.342**	**2**	**4.65**	**9.26**	**58.13**	**58.86**
	Equiv. Refl.: (8,-8,4), (-8,0,4), (-8,8,-4)								

Ei =	13.273	keV						ΔEg	ΔEt
Cryst	Refl	EB	ΘB	∫ IR dΘ	Width	Ei cotΘ	ΔE	2m, 50µm	2m, 50µm
	(h,k,l)	[keV]	[°]	[µrad]	[µrad]	[meV/µrad]	[meV]	[meV]	[meV]
**Quartz**	**(10,-3,0)**	**12.95**	**77.33**	**2.331**	**2.2**	**2.983**	**6.46**	**37.29**	**37.84**
	Equiv. Refl.: (10,-7,0), (-3,10,0), (-3,-7,0), (-7,10,0), (-7,-3,0)								
**Quartz**	**(9,-1,1)**	**12.501**	**70.37**	**2.32**	**1.9**	**4.735**	**9.09**	**59.19**	**59.89**
	Equiv. Refl.: (9,-8,-1), (-1,9,-1), (-1,-8,1), (-8,9,1), (-8,-1,-1)								
**Quartz**	**(6,-3,-9)**	**12.802**	**74.69**	**2.309**	**2.1**	**3.635**	**7.5**	**45.43**	**46.05**
	Equiv. Refl.: (-3,6,9), (-3,6,-9), (-3,-3,9), (-3,-3,-9)								
**Quartz**	**(6,-3,9)**	**12.802**	**74.69**	**2.308**	**2.1**	**3.635**	**7.5**	**45.43**	**46.05**
**Quartz**	**(3,3,-9)**	**12.802**	**74.69**	**2.299**	**2.1**	**3.635**	**7.5**	**45.43**	**46.05**
	Equiv. Refl.: (3,-6,9), (3,-6,-9), (-6,3,9), (-6,3,-9)								
**Quartz**	**(3,3,9)**	**12.802**	**74.69**	**2.298**	**2.1**	**3.635**	**7.5**	**45.43**	**46.05**
**Quartz**	**(-7,9,-5)**	**13.233**	**85.57**	**1.92**	**2.2**	**1.029**	**2.27**	**12.87**	**13.07**
	Equiv. Refl.: (-7,-2,5)								
**Quartz**	**(9,-2,-5)**	**13.233**	**85.57**	**1.918**	**2.2**	**1.029**	**2.27**	**12.87**	**13.07**
	Equiv. Refl.: (9,-7,5)								
**Quartz**	**(-2,-7,-5)**	**13.233**	**85.57**	**1.917**	**2.2**	**1.029**	**2.27**	**12.87**	**13.07**
**Quartz**	**(-2,9,5)**	**13.233**	**85.57**	**1.915**	**2.2**	**1.029**	**2.27**	**12.87**	**13.06**
**Sapph**	**(1,8,-1)**	**12.863**	**75.72**	**1.906**	**2**	**3.379**	**6.9**	**42.24**	**42.8**
	Equiv. Refl.: (1,-9,1), (8,1,1), (8,-9,-1), (9,-1,1), (9,-8,-1), (-1,9,-1), (-1,-8,1), (-8,9,1), (-8,-1,-1) (-9,1,-1), (-9,8,1)								
**Sapph**	**(4,5,-10)**	**12.682**	**72.84**	**1.878**	**2**	**4.098**	**8.11**	**51.22**	**51.86**
	Equiv. Refl.: (4,-9,10), (5,4,10), (5,-9,-10), (9,-4,10), (9,-5,-10), (-4,9,-10), (-4,-5,10), (-5,9,10), (-5,-4,-10) (-9,4,-10), (-9,5,10)								
**Quartz**	**(4,-8,-7)**	**12.898**	**76.35**	**1.876**	**2**	**3.223**	**6.32**	**40.29**	**40.78**
	Equiv. Refl.: (-8,4,7), (-8,4,-7)								
**Quartz**	**(4,4,7)**	**12.898**	**76.35**	**1.875**	**2**	**3.223**	**6.32**	**40.29**	**40.78**
	Equiv. Refl.: (4,4,-7), (4,-8,7)								
**Quartz**	**(8,-4,7)**	**12.898**	**76.35**	**1.869**	**2**	**3.223**	**6.32**	**40.29**	**40.78**
	Equiv. Refl.: (8,-4,-7), (-4,8,7), (-4,8,-7), (-4,-4,-7)								
**Quartz**	**(-4,-4,7)**	**12.898**	**76.35**	**1.868**	**2**	**3.223**	**6.32**	**40.29**	**40.78**
**Quartz**	**(-3,7,8)**	**12.757**	**73.97**	**1.86**	**1.9**	**3.812**	**7.3**	**47.66**	**48.21**
**Quartz**	**(-3,-4,-8)**	**12.757**	**73.97**	**1.859**	**1.9**	**3.812**	**7.3**	**47.66**	**48.21**
**Quartz**	**(7,-4,8)**	**12.757**	**73.97**	**1.858**	**1.9**	**3.812**	**7.29**	**47.66**	**48.21**
	Equiv. Refl.: (-4,7,-8), (-4,-3,8)								
**Quartz**	**(7,-3,-8)**	**12.757**	**73.97**	**1.857**	**1.9**	**3.812**	**7.29**	**47.66**	**48.21**
**Quartz**	**(10,-4,-2)**	**12.907**	**76.52**	**1.851**	**1.9**	**3.182**	**5.93**	**39.78**	**40.22**
	Equiv. Refl.: (10,-6,2), (-4,10,2), (-4,-6,-2), (-6,10,-2), (-6,-4,2)								
**Sapph**	**(0,2,-26)**	**12.769**	**74.16**	**1.85**	**2**	**3.765**	**7.4**	**47.07**	**47.65**
	Equiv. Refl.: (0,-2,26), (2,0,26), (2,-2,-26), (-2,0,-26), (-2,2,26)								
**Quartz**	**(3,4,8)**	**12.757**	**73.97**	**1.84**	**1.9**	**3.812**	**7.3**	**47.66**	**48.21**
	Equiv. Refl.: (3,-7,-8)								
**Quartz**	**(4,3,-8)**	**12.757**	**73.97**	**1.838**	**1.9**	**3.812**	**7.29**	**47.66**	**48.21**
	Equiv. Refl.: (4,-7,8), (-7,3,8), (-7,4,-8)								
**Quartz**	**(1,8,-1)**	**12.501**	**70.37**	**1.832**	**1.9**	**4.735**	**9.09**	**59.19**	**59.89**
	Equiv. Refl.: (1,-9,1), (8,1,1), (8,-9,-1), (-9,1,-1), (-9,8,1)								
**Sapph**	**(1,5,20)**	**12.7**	**73.1**	**1.802**	**1.9**	**4.033**	**7.7**	**50.41**	**50.99**
	Equiv. Refl.: (1,-6,-20), (5,1,-20), (5,-6,20), (6,-1,-20), (6,-5,20), (-1,6,20), (-1,-5,-20), (-5,6,-20), (-5,-1,20) (-6,1,20), (-6,5,-20)								
**Quartz**	**(-1,4,10)**	**12.616**	**71.9**	**1.781**	**1.8**	**4.339**	**7.75**	**54.24**	**54.79**
	Equiv. Refl.: (-1,-3,-10)								
**Quartz**	**(-3,4,-10)**	**12.616**	**71.9**	**1.78**	**1.8**	**4.339**	**7.75**	**54.24**	**54.79**
**Quartz**	**(-3,-1,10)**	**12.616**	**71.9**	**1.779**	**1.8**	**4.339**	**7.74**	**54.24**	**54.79**

Ei =	13.273	keV						ΔEg	ΔEt
Cryst	Refl	EB	ΘB	∫ IR dΘ	Width	Ei cotΘ	ΔE	2m, 50µm	2m, 50µm
	(h,k,l)	[keV]	[°]	[µrad]	[µrad]	[meV/µrad]	[meV]	[meV]	[meV]
**Quartz**	**(4,-1,-10)**	**12.616**	**71.9**	**1.778**	**1.8**	**4.339**	**7.74**	**54.24**	**54.79**
	Equiv. Refl.: (4,-3,10)								
**Quartz**	**(6,2,-6)**	**12.56**	**71.13**	**1.725**	**1.8**	**4.536**	**7.97**	**56.7**	**57.26**
	Equiv. Refl.: (6,-8,6), (-8,6,-6)								
**Quartz**	**(-8,2,6)**	**12.56**	**71.13**	**1.724**	**1.8**	**4.536**	**7.97**	**56.7**	**57.26**
**Quartz**	**(2,6,6)**	**12.56**	**71.13**	**1.723**	**1.8**	**4.536**	**7.96**	**56.7**	**57.26**
	Equiv. Refl.: (2,-8,-6)								
**Quartz**	**(4,6,2)**	**12.907**	**76.52**	**1.691**	**1.9**	**3.182**	**5.94**	**39.78**	**40.22**
	Equiv. Refl.: (4,-10,-2), (6,4,-2), (6,-10,2), (-10,4,2), (-10,6,-2)								
**Quartz**	**(8,-2,-6)**	**12.56**	**71.13**	**1.685**	**1.8**	**4.536**	**7.97**	**56.7**	**57.26**
	Equiv. Refl.: (8,-6,6), (-6,8,-6), (-6,-2,6)								
**Quartz**	**(-2,8,6)**	**12.56**	**71.13**	**1.684**	**1.8**	**4.536**	**7.96**	**56.7**	**57.26**
	Equiv. Refl.: (-2,-6,-6)								
**Quartz**	**(2,1,11)**	**13.193**	**83.7**	**1.683**	**1.8**	**1.465**	**2.7**	**18.31**	**18.51**
	Equiv. Refl.: (2,-3,-11)								
**Quartz**	**(1,3,10)**	**12.616**	**71.9**	**1.682**	**1.8**	**4.339**	**7.75**	**54.24**	**54.79**
**Quartz**	**(1,-4,-10)**	**12.616**	**71.9**	**1.681**	**1.8**	**4.339**	**7.75**	**54.24**	**54.79**
	Equiv. Refl.: (3,-4,10)								
**Quartz**	**(3,1,-10)**	**12.616**	**71.9**	**1.68**	**1.8**	**4.339**	**7.74**	**54.24**	**54.79**
**Quartz**	**(-3,1,-11)**	**13.193**	**83.7**	**1.679**	**1.8**	**1.465**	**2.7**	**18.31**	**18.5**
	Equiv. Refl.: (-4,1,10), (-4,3,-10)								
**Quartz**	**(1,2,-11)**	**13.193**	**83.7**	**1.677**	**1.8**	**1.465**	**2.69**	**18.31**	**18.5**
	Equiv. Refl.: (1,-3,11)								
**Quartz**	**(-3,2,11)**	**13.193**	**83.7**	**1.676**	**1.8**	**1.465**	**2.69**	**18.31**	**18.5**
**Quartz**	**(7,2,-5)**	**13.233**	**85.57**	**1.671**	**2.2**	**1.029**	**2.27**	**12.87**	**13.07**
	Equiv. Refl.: (7,-9,5)								
**Quartz**	**(-9,2,5)**	**13.233**	**85.57**	**1.67**	**2.2**	**1.029**	**2.27**	**12.87**	**13.07**
	Equiv. Refl.: (-9,7,-5)								
**Quartz**	**(2,7,5)**	**13.233**	**85.57**	**1.668**	**2.2**	**1.029**	**2.27**	**12.87**	**13.07**
**Quartz**	**(2,-9,-5)**	**13.233**	**85.57**	**1.667**	**2.2**	**1.029**	**2.27**	**12.87**	**13.06**
**Quartz**	**(0,6,8)**	**12.674**	**72.72**	**1.581**	**1.6**	**4.13**	**6.81**	**51.62**	**52.07**
	Equiv. Refl.: (0,-6,-8), (6,0,-8), (6,-6,8), (-6,0,8), (-6,6,-8)								
**LiNbO3**	**(4,6,-11)**	**13.082**	**80.27**	**1.542**	**2.5**	**2.276**	**5.7**	**28.45**	**29.02**
	Equiv. Refl.: (4,-10,11), (6,4,11), (6,-10,-11), (10,-4,11), (10,-6,-11), (-4,10,-11), (-4,-6,11), (-6,10,11), (-6,-4,-11) (-10,4,-11), (-10,6,11)								
**Quartz**	**(10,-4,-1)**	**12.753**	**73.92**	**1.519**	**1.6**	**3.827**	**6.07**	**47.84**	**48.22**
	Equiv. Refl.: (10,-6,1), (-4,10,1), (-4,-6,-1), (-6,10,-1), (-6,-4,1)								
**Quartz**	**(4,6,1)**	**12.753**	**73.92**	**1.49**	**1.6**	**3.827**	**6.07**	**47.84**	**48.22**
	Equiv. Refl.: (4,-10,-1), (6,4,-1), (6,-10,1), (-10,4,1), (-10,6,-1)								
**Sapph**	**(4,5,11)**	**12.87**	**75.84**	**1.484**	**1.6**	**3.35**	**5.49**	**41.87**	**42.23**
	Equiv. Refl.: (4,-9,-11), (5,4,-11), (5,-9,11), (9,-4,-11), (9,-5,11), (-4,9,11), (-4,-5,-11), (-5,9,-11), (-5,-4,11) (-9,4,11), (-9,5,-11)								
**Quartz**	**(4,-2,10)**	**12.532**	**70.76**	**1.479**	**1.5**	**4.633**	**7.13**	**57.91**	**58.35**
	Equiv. Refl.: (4,-2,-10)								
**Quartz**	**(-2,4,-10)**	**12.532**	**70.76**	**1.478**	**1.5**	**4.633**	**7.12**	**57.91**	**58.35**
	Equiv. Refl.: (-2,-2,10)								
**Quartz**	**(-2,4,10)**	**12.532**	**70.76**	**1.477**	**1.5**	**4.633**	**7.12**	**57.91**	**58.35**
	Equiv. Refl.: (-2,-2,-10), (-4,2,10)								
**Quartz**	**(-4,2,-10)**	**12.532**	**70.76**	**1.476**	**1.5**	**4.633**	**7.13**	**57.91**	**58.35**

Ei = Cryst	13.273 Refl (h,k,l)	keV EB [keV]	ΘB [°]	∫ IR dΘ [µrad]	Width [µrad]	Ei cotΘ [meV/µrad]	ΔE [meV]	ΔEg 2m, 50µm [meV]	ΔEt 2m, 50µm [meV]
**Quartz**	**(2,2,-10)**	**12.532**	**70.76**	**1.475**	**1.5**	**4.633**	**7.12**	**57.91**	**58.35**
	Equiv. Refl.: (2,-4,10)								
**Quartz**	**(2,2,10)**	**12.532**	**70.76**	**1.474**	**1.5**	**4.633**	**7.12**	**57.91**	**58.35**
	Equiv. Refl.: (2,-4,-10)								
**Quartz**	**(6,3,5)**	**12.908**	**76.54**	**1.473**	**1.6**	**3.177**	**5.01**	**39.71**	**40.02**
	Equiv. Refl.: (6,-9,-5)								
**Quartz**	**(3,6,-5)**	**12.908**	**76.54**	**1.472**	**1.6**	**3.177**	**5.01**	**39.71**	**40.02**
	Equiv. Refl.: (3,-9,5), (-9,3,-5), (-9,6,5)								
**Quartz**	**(9,-3,5)**	**12.908**	**76.54**	**1.461**	**1.6**	**3.177**	**5.01**	**39.71**	**40.02**
	Equiv. Refl.: (9,-6,-5), (-6,9,5), (-6,-3,-5)								
**Quartz**	**(-3,9,-5)**	**12.908**	**76.54**	**1.46**	**1.6**	**3.177**	**5.01**	**39.71**	**40.02**
	Equiv. Refl.: (-3,-6,5)								
**Quartz**	**(-2,3,11)**	**13.193**	**83.7**	**1.439**	**1.8**	**1.465**	**2.7**	**18.31**	**18.51**
	Equiv. Refl.: (-2,-1,-11)								
**Quartz**	**(3,-1,11)**	**13.193**	**83.7**	**1.435**	**1.8**	**1.465**	**2.7**	**18.31**	**18.5**
**Quartz**	**(3,-2,-11)**	**13.193**	**83.7**	**1.433**	**1.8**	**1.465**	**2.69**	**18.31**	**18.5**
	Equiv. Refl.: (-1,3,-11), (-1,-2,11)								
**Quartz**	**(1,8,3)**	**12.915**	**76.67**	**1.366**	**1.4**	**3.145**	**4.45**	**39.32**	**39.57**
	Equiv. Refl.: (1,-9,-3), (8,1,-3), (10,-3,-2), (10,-7,2), (-9,1,3), (-9,8,-3)								
**Quartz**	**(8,-9,3)**	**12.915**	**76.67**	**1.365**	**1.4**	**3.145**	**4.45**	**39.32**	**39.57**
**Quartz**	**(-3,10,2)**	**13.152**	**82.24**	**1.364**	**1.6**	**1.808**	**2.85**	**22.6**	**22.78**
	Equiv. Refl.: (-3,-7,-2), (-7,10,-2), (-7,-3,2)								
**Quartz**	**(1,8,2)**	**12.658**	**72.49**	**1.36**	**1.4**	**4.187**	**5.76**	**52.34**	**52.65**
	Equiv. Refl.: (1,-9,-2), (8,1,-2), (8,-9,2)								
**Quartz**	**(-9,1,2)**	**12.658**	**72.49**	**1.359**	**1.4**	**4.187**	**5.76**	**52.34**	**52.65**
	Equiv. Refl.: (-9,8,-2)								
**Quartz**	**(-10,3,2)**	**13.152**	**82.24**	**1.33**	**1.6**	**1.808**	**2.86**	**22.6**	**22.78**
	Equiv. Refl.: (-10,7,-2)								
**Quartz**	**(1,-6,-9)**	**13.129**	**81.56**	**1.328**	**1.4**	**1.97**	**2.79**	**24.63**	**24.78**
	Equiv. Refl.: (3,7,2), (3,-10,-2), (7,3,-2), (7,-10,2)								
**Quartz**	**(1,5,9)**	**13.129**	**81.56**	**1.327**	**1.4**	**1.97**	**2.79**	**24.63**	**24.78**
**Quartz**	**(5,1,-9)**	**13.129**	**81.56**	**1.325**	**1.4**	**1.97**	**2.79**	**24.63**	**24.78**
	Equiv. Refl.: (5,-6,9)								
**Quartz**	**(-6,5,-9)**	**13.129**	**81.56**	**1.322**	**1.4**	**1.97**	**2.78**	**24.63**	**24.78**
**Quartz**	**(-6,1,9)**	**13.129**	**81.56**	**1.321**	**1.4**	**1.97**	**2.78**	**24.63**	**24.78**
**LiNbO3**	**(2,2,27)**	**12.999**	**78.34**	**1.304**	**2.1**	**2.739**	**5.79**	**34.24**	**34.72**
	Equiv. Refl.: (2,2,-27), (2,-4,27), (2,-4,-27), (4,-2,27), (4,-2,-27), (-2,4,27), (-2,4,-27), (-2,-2,27), (-2,-2,-27) (-4,2,27), (-4,2,-27)								
**Quartz**	**(9,-4,5)**	**12.743**	**73.75**	**1.302**	**1.4**	**3.868**	**5.35**	**48.35**	**48.64**
	Equiv. Refl.: (9,-5,-5), (-4,9,-5), (-4,-5,5), (-5,9,5), (-5,-4,-5)								
**Quartz**	**(4,5,-5)**	**12.743**	**73.75**	**1.285**	**1.4**	**3.868**	**5.35**	**48.35**	**48.64**
	Equiv. Refl.: (4,-9,5), (5,4,5), (5,-9,-5), (-9,4,-5), (-9,5,5)								
**Quartz**	**(-5,10,3)**	**13.079**	**80.18**	**1.247**	**1.4**	**2.297**	**3.25**	**28.71**	**28.89**
	Equiv. Refl.: (-5,-5,-3)								
**Quartz**	**(10,-5,3)**	**13.079**	**80.18**	**1.246**	**1.4**	**2.297**	**3.25**	**28.71**	**28.89**
	Equiv. Refl.: (10,-5,-3), (-5,10,-3), (-5,-5,3)								
**LiNbO3**	**(2,7,13)**	**12.78**	**74.34**	**1.233**	**1.9**	**3.721**	**6.95**	**46.51**	**47.03**
	Equiv. Refl.: (2,-9,-13), (7,2,-13), (7,-9,13), (9,-2,-13), (9,-7,13), (-2,9,13), (-2,-7,-13), (-7,9,-13), (-7,-2,13) (-9,2,13), (-9,7,-13), (5,5,3), (5,-10,-3), (-8,9,-2), (-8,-1,2)								
**Quartz**	**(5,5,-3)**	**13.079**	**80.18**	**1.232**	**1.4**	**2.297**	**3.25**	**28.71**	**28.89**
	Equiv. Refl.: (5,-10,3), (9,-1,-2), (9,-8,2), (-1,9,2), (-1,-8,-2), (-10,5,3), (-10,5,-3)								

Ei = Cryst	13.273 Refl (h,k,l)	keV EB [keV]	ΘB [°]	∫ IR dΘ [µrad]	Width [µrad]	Ei cotΘ [meV/µrad]	ΔE [meV]	ΔEg 2m, 50µm [meV]	ΔEt 2m, 50µm [meV]
**Quartz**	**(9,-1,-3)**	**12.915**	**76.67**	**1.227**	**1.4**	**3.145**	**4.45**	**39.32**	**39.57**
	Equiv. Refl.: (9,-8,3), (-1,9,3), (-1,-8,-3)								
**Quartz**	**(-8,9,-3)**	**12.915**	**76.67**	**1.226**	**1.4**	**3.145**	**4.45**	**39.32**	**39.57**
	Equiv. Refl.: (-8,-1,3)								
**Quartz**	**(-1,9,-3)**	**12.915**	**76.67**	**1.203**	**1.3**	**3.145**	**4.14**	**39.32**	**39.53**
	Equiv. Refl.: (-1,-8,3), (-8,9,3), (-8,-1,-3)								
**Quartz**	**(9,-1,3)**	**12.915**	**76.67**	**1.202**	**1.3**	**3.145**	**4.14**	**39.32**	**39.53**
	Equiv. Refl.: (9,-8,-3)								
**Sapph**	**(2,6,-13)**	**12.498**	**70.32**	**1.185**	**1.3**	**4.748**	**6.16**	**59.34**	**59.66**
	Equiv. Refl.: (2,-8,13), (6,2,13), (6,-8,-13), (8,-2,13), (8,-6,-13), (-2,8,-13), (-2,-6,13), (-6,8,13), (-6,-2,-13) (-8,2,-13), (-8,6,13)								
**Quartz**	**(8,1,3)**	**12.915**	**76.67**	**1.184**	**1.3**	**3.145**	**4.14**	**39.32**	**39.53**
	Equiv. Refl.: (8,-9,-3)								
**Quartz**	**(1,8,-3)**	**12.915**	**76.67**	**1.183**	**1.3**	**3.145**	**4.14**	**39.32**	**39.53**
	Equiv. Refl.: (1,-9,3), (-9,1,-3), (-9,8,3)								
**Sapph**	**(4,4,15)**	**12.645**	**72.3**	**1.156**	**1.3**	**4.235**	**5.44**	**52.94**	**53.22**
	Equiv. Refl.: (4,4,-15), (4,-8,15), (4,-8,-15), (8,-4,15), (8,-4,-15), (-4,8,15), (-4,8,-15), (-4,-4,15), (-4,-4,-15) (-8,4,15), (-8,4,-15)								
**Quartz**	**(-10,6,-3)**	**13.16**	**82.5**	**1.116**	**1.3**	**1.746**	**2.23**	**21.83**	**21.94**
**Quartz**	**(4,6,3)**	**13.16**	**82.5**	**1.115**	**1.3**	**1.746**	**2.23**	**21.83**	**21.94**
	Equiv. Refl.: (4,-10,-3), (-10,4,3)								
**Quartz**	**(6,4,-3)**	**13.16**	**82.5**	**1.114**	**1.3**	**1.746**	**2.22**	**21.83**	**21.94**
	Equiv. Refl.: (6,-10,3)								
**Quartz**	**(-1,6,9)**	**13.129**	**81.56**	**1.089**	**1.4**	**1.97**	**2.79**	**24.63**	**24.78**
**Quartz**	**(-1,-5,-9)**	**13.129**	**81.56**	**1.087**	**1.4**	**1.97**	**2.79**	**24.63**	**24.78**
	Equiv. Refl.: (-5,-1,9)								
**Quartz**	**(-5,6,-9)**	**13.129**	**81.56**	**1.086**	**1.4**	**1.97**	**2.79**	**24.63**	**24.78**
**Quartz**	**(6,-5,9)**	**13.129**	**81.56**	**1.084**	**1.4**	**1.97**	**2.78**	**24.63**	**24.78**
**Quartz**	**(6,-1,-9)**	**13.129**	**81.56**	**1.083**	**1.4**	**1.97**	**2.78**	**24.63**	**24.78**
**Quartz**	**(0,7,7)**	**12.98**	**77.94**	**1.062**	**1.2**	**2.835**	**3.42**	**35.44**	**35.61**
	Equiv. Refl.: (0,-7,-7)								
**Quartz**	**(-7,7,-7)**	**12.98**	**77.94**	**1.06**	**1.2**	**2.835**	**3.41**	**35.44**	**35.6**
**Quartz**	**(7,0,-7)**	**12.98**	**77.94**	**1.059**	**1.2**	**2.835**	**3.41**	**35.44**	**35.6**
	Equiv. Refl.: (7,-7,7), (-7,0,7)								
**Quartz**	**(0,6,-8)**	**12.674**	**72.72**	**1.04**	**1.1**	**4.13**	**4.68**	**51.62**	**51.83**
	Equiv. Refl.: (0,-6,8), (6,0,8), (6,-6,-8), (-6,0,-8), (-6,6,8)								
**Sapph**	**(2,7,-8)**	**12.893**	**76.25**	**1.009**	**1.2**	**3.248**	**3.85**	**40.6**	**40.78**
	Equiv. Refl.: (2,-9,8), (7,2,8), (7,-9,-8), (9,-2,8), (9,-7,-8), (-2,9,-8), (-2,-7,8), (-7,9,8), (-7,-2,-8) (-9,2,-8), (-9,7,8)								
**Quartz**	**(8,-3,7)**	**12.98**	**77.94**	**0.998**	**1.1**	**2.835**	**3.22**	**35.44**	**35.59**
**Quartz**	**(8,-5,-7)**	**12.98**	**77.94**	**0.997**	**1.1**	**2.835**	**3.22**	**35.44**	**35.59**
	Equiv. Refl.: (-3,8,-7), (-3,-5,7)								
**LiNbO3**	**(2,4,-23)**	**12.646**	**72.32**	**0.996**	**1.6**	**4.231**	**6.6**	**52.89**	**53.3**
	Equiv. Refl.: (2,-6,23), (4,2,23), (4,-6,-23), (6,-2,23), (6,-4,-23), (-2,6,-23), (-2,-4,23), (-4,6,23), (-4,-2,-23) (-6,2,-23), (-6,4,23)								
**Quartz**	**(-5,8,7)**	**12.98**	**77.94**	**0.994**	**1.1**	**2.835**	**3.21**	**35.44**	**35.59**
	Equiv. Refl.: (-5,-3,-7)								
**Sapph**	**(0,1,26)**	**12.5**	**70.36**	**0.976**	**1.1**	**4.738**	**5.2**	**59.23**	**59.45**
	Equiv. Refl.: (0,-1,-26), (1,0,-26), (1,-1,26), (-1,0,26), (-1,1,-26)								

Ei =	13.273	keV						ΔEg	ΔEt
Cryst	Refl	EB	ΘB	∫ IR dΘ	Width	Ei cotΘ	ΔE	2m, 50µm	2m, 50µm
	(h,k,l)	[keV]	[°]	[µrad]	[µrad]	[meV/µrad]	[meV]	[meV]	[meV]
**Quartz**	**(3,-8,7)**	**12.98**	**77.94**	**0.973**	**1.1**	**2.835**	**3.22**	**35.44**	**35.59**
	Equiv. Refl.: (-8,3,-7), (-8,5,7)								
**Quartz**	**(3,5,-7)**	**12.98**	**77.94**	**0.972**	**1.1**	**2.835**	**3.21**	**35.44**	**35.59**
**Quartz**	**(2,5,8)**	**12.923**	**76.8**	**0.969**	**1.1**	**3.112**	**3.38**	**38.9**	**39.05**
	Equiv. Refl.: (2,-7,-8), (5,3,7), (5,-8,-7), (-7,5,-8)								
**Quartz**	**(-7,2,8)**	**12.923**	**76.8**	**0.968**	**1.1**	**3.112**	**3.38**	**38.9**	**39.05**
**Quartz**	**(5,2,-8)**	**12.923**	**76.8**	**0.967**	**1.1**	**3.112**	**3.37**	**38.9**	**39.05**
	Equiv. Refl.: (5,-7,8)								
**Quartz**	**(-6,-2,-6)**	**12.56**	**71.13**	**0.947**	**1**	**4.536**	**4.46**	**56.7**	**56.87**
**Quartz**	**(8,-2,6)**	**12.56**	**71.13**	**0.946**	**1**	**4.536**	**4.45**	**56.7**	**56.87**
	Equiv. Refl.: (8,-6,-6), (-6,8,6)								
**Quartz**	**(-2,8,-6)**	**12.56**	**71.13**	**0.945**	**1**	**4.536**	**4.45**	**56.7**	**56.87**
	Equiv. Refl.: (-2,-6,6)								
**Quartz**	**(10,-6,3)**	**13.16**	**82.5**	**0.943**	**1.3**	**1.746**	**2.23**	**21.83**	**21.94**
**Quartz**	**(10,-4,-3)**	**13.16**	**82.5**	**0.942**	**1.3**	**1.746**	**2.23**	**21.83**	**21.94**
	Equiv. Refl.: (-4,10,3), (-4,-6,-3)								
**Quartz**	**(-6,10,-3)**	**13.16**	**82.5**	**0.941**	**1.3**	**1.746**	**2.22**	**21.83**	**21.94**
	Equiv. Refl.: (-6,-4,3)								
**Quartz**	**(10,-3,-1)**	**13.001**	**78.37**	**0.938**	**1.1**	**2.731**	**2.95**	**34.14**	**34.26**
	Equiv. Refl.: (10,-7,1), (-3,10,1), (-3,-7,-1), (-7,10,-1), (-7,-3,1)								
**Quartz**	**(0,1,-11)**	**12.701**	**73.12**	**0.935**	**1**	**4.028**	**4.17**	**50.35**	**50.52**
	Equiv. Refl.: (0,-1,11)								
**Quartz**	**(1,0,11)**	**12.701**	**73.12**	**0.934**	**1**	**4.028**	**4.17**	**50.35**	**50.52**
	Equiv. Refl.: (1,-1,-11), (-1,1,11)								
**Quartz**	**(-1,0,-11)**	**12.701**	**73.12**	**0.933**	**1**	**4.028**	**4.17**	**50.35**	**50.52**
**Quartz**	**(7,-5,8)**	**12.923**	**76.8**	**0.931**	**1.1**	**3.112**	**3.38**	**38.9**	**39.05**
	Equiv. Refl.: (-2,7,8), (-2,-5,-8)								
**Quartz**	**(7,-2,-8)**	**12.923**	**76.8**	**0.93**	**1.1**	**3.112**	**3.38**	**38.9**	**39.05**
**Quartz**	**(-5,7,-8)**	**12.923**	**76.8**	**0.929**	**1.1**	**3.112**	**3.37**	**38.9**	**39.05**
	Equiv. Refl.: (-5,-2,8)								
**Quartz**	**(3,7,1)**	**13.001**	**78.37**	**0.91**	**1.1**	**2.731**	**2.95**	**34.14**	**34.26**
	Equiv. Refl.: (3,-10,-1), (7,3,-1), (7,-10,1), (-10,3,1), (-10,7,-1)								
**LiNbO3**	**(4,6,7)**	**12.52**	**70.6**	**0.88**	**1.4**	**4.673**	**6.53**	**58.42**	**58.78**
	Equiv. Refl.: (4,-10,-7), (6,4,-7), (6,-10,7), (10,-4,-7), (10,-6,7), (-4,10,7), (-4,-6,-7), (-6,10,-7), (-6,-4,7) (-10,4,7), (-10,6,-7)								
**Sapph**	**(1,6,16)**	**12.476**	**70.04**	**0.878**	**1**	**4.821**	**4.82**	**60.26**	**60.45**
	Equiv. Refl.: (1,-7,-16), (6,1,-16), (6,-7,16), (7,-1,-16), (7,-6,16), (-1,7,16), (-1,-6,-16), (-6,7,-16), (-6,-1,16) (-7,1,16), (-7,6,-16)								
**Quartz**	**(10,-4,0)**	**12.702**	**73.13**	**0.852**	**1**	**4.025**	**3.85**	**50.32**	**50.46**
	Equiv. Refl.: (10,-6,0), (-4,10,0), (-4,-6,0), (-6,10,0), (-6,-4,0)								
**Quartz**	**(4,6,0)**	**12.702**	**73.13**	**0.848**	**1**	**4.025**	**3.85**	**50.32**	**50.46**
	Equiv. Refl.: (4,-10,0), (6,4,0), (6,-10,0), (-10,4,0), (-10,6,0)								
**Quartz**	**(0,4,10)**	**12.866**	**75.77**	**0.842**	**1**	**3.366**	**3.26**	**42.07**	**42.2**
	Equiv. Refl.: (0,-4,-10)								
**Quartz**	**(4,0,-10)**	**12.866**	**75.77**	**0.839**	**1**	**3.366**	**3.25**	**42.07**	**42.19**
	Equiv. Refl.: (4,-4,10), (6,2,6), (6,-8,-6), (-4,0,10), (-4,4,-10), (-8,2,-6), (-8,6,6)								
**Quartz**	**(2,6,-6)**	**12.56**	**71.13**	**0.838**	**1**	**4.536**	**4.45**	**56.7**	**56.87**
	Equiv. Refl.: (2,-8,6)								
**Sapph**	**(2,7,7)**	**12.759**	**74.01**	**0.786**	**0.9**	**3.804**	**3.59**	**47.55**	**47.68**
	Equiv. Refl.: (2,-9,-7), (7,2,-7), (7,-9,7), (9,-2,-7), (9,-7,7), (-2,9,7), (-2,-7,-7), (-7,9,-7), (-7,-2,7) (-9,2,7), (-9,7,-7)								

Ei =	13.273	keV						ΔEg	ΔEt
**Cryst**	**Refl**	**EB**	**ΘB**	**∫ IR dΘ**	**Width**	**Ei cotΘ**	**ΔE**	**2m, 50μm**	**2m, 50μm**
	**(h,k,l)**	**[keV]**	**[°]**	**[μrad]**	**[μrad]**	**[meV/μrad]**	**[meV]**	**[meV]**	**[meV]**
**Sapph**	**(5,5,3)**	**13.107**	**80.93**	**0.778**	**1.1**	**2.118**	**2.23**	**26.48**	**26.57**
	Equiv. Refl.: (5,5,-3), (5,-10,3), (5,-10,-3), (10,-5,3), (10,-5,-3), (-5,10,3), (-5,10,-3), (-5,-5,3), (-5,-5,-3) (-10,5,3), (-10,5,-3)								
**Quartz**	**(6,1,-7)**	**12.48**	**70.09**	**0.739**	**0.8**	**4.807**	**3.9**	**60.08**	**60.21**
	Equiv. Refl.: (6,-7,7)								
**Quartz**	**(1,6,7)**	**12.48**	**70.09**	**0.737**	**0.8**	**4.807**	**3.89**	**60.08**	**60.21**
	Equiv. Refl.: (1,-7,-7), (-7,1,7), (-7,6,-7)								
**Quartz**	**(10,-5,1)**	**12.67**	**72.66**	**0.724**	**0.8**	**4.144**	**3.44**	**51.8**	**51.91**
	Equiv. Refl.: (10,-5,-1), (-5,10,1), (-5,10,-1), (-5,-5,1), (-5,-5,-1)								
**Quartz**	**(5,5,1)**	**12.67**	**72.66**	**0.722**	**0.8**	**4.144**	**3.44**	**51.8**	**51.91**
	Equiv. Refl.: (5,-10,-1)								
**Quartz**	**(5,5,-1)**	**12.67**	**72.66**	**0.721**	**0.8**	**4.144**	**3.44**	**51.8**	**51.91**
	Equiv. Refl.: (5,-10,1), (-10,5,1), (-10,5,-1)								
**Quartz**	**(-2,9,-4)**	**12.778**	**74.3**	**0.706**	**0.8**	**3.73**	**2.95**	**46.62**	**46.72**
**Quartz**	**(9,-2,4)**	**12.778**	**74.3**	**0.705**	**0.8**	**3.73**	**2.95**	**46.62**	**46.72**
	Equiv. Refl.: (9,-7,-4), (-2,-7,4), (-7,9,4), (-7,-2,-4)								
**Sapph**	**(0,5,22)**	**12.917**	**76.69**	**0.704**	**0.9**	**3.139**	**2.81**	**39.24**	**39.34**
	Equiv. Refl.: (0,-5,-22), (5,0,-22), (5,-5,22), (-5,0,22), (-5,5,-22)								
**Quartz**	**(-6,7,-7)**	**12.48**	**70.09**	**0.699**	**0.8**	**4.807**	**3.9**	**60.08**	**60.21**
	Equiv. Refl.: (-6,-1,7)								
**Quartz**	**(7,-1,-7)**	**12.48**	**70.09**	**0.697**	**0.8**	**4.807**	**3.89**	**60.08**	**60.21**
	Equiv. Refl.: (7,-6,7), (-1,7,7), (-1,-6,-7)								
**Sapph**	**(2,7,-5)**	**12.543**	**70.91**	**0.696**	**0.8**	**4.593**	**3.81**	**57.41**	**57.54**
	Equiv. Refl.: (2,-9,5), (7,2,5), (7,-9,-5), (9,-2,5), (9,-7,-5), (-2,9,-5), (-2,-7,5), (-7,9,5), (-7,-2,-5) (-9,2,-5), (-9,7,5)								
**Quartz**	**(3,-4,-10)**	**12.616**	**71.9**	**0.676**	**0.7**	**4.339**	**3.1**	**54.24**	**54.33**
**Quartz**	**(3,1,10)**	**12.616**	**71.9**	**0.675**	**0.7**	**4.339**	**3.1**	**54.24**	**54.33**
**Quartz**	**(1,3,-10)**	**12.616**	**71.9**	**0.674**	**0.7**	**4.339**	**3.1**	**54.24**	**54.33**
**Quartz**	**(1,-4,10)**	**12.616**	**71.9**	**0.673**	**0.7**	**4.339**	**3.09**	**54.24**	**54.33**
	Equiv. Refl.: (-4,1,-10), (-4,3,10)								
**Quartz**	**(10,-3,1)**	**13.001**	**78.37**	**0.668**	**0.8**	**2.731**	**2.2**	**34.14**	**34.21**
	Equiv. Refl.: (10,-7,-1), (-3,10,-1), (-3,-7,1), (-7,10,1), (-7,-3,-1)								
**Quartz**	**(2,7,-4)**	**12.778**	**74.3**	**0.639**	**0.8**	**3.73**	**2.95**	**46.62**	**46.72**
	Equiv. Refl.: (2,-9,4), (7,2,4), (7,-9,-4)								
**Quartz**	**(-9,2,-4)**	**12.778**	**74.3**	**0.638**	**0.8**	**3.73**	**2.95**	**46.62**	**46.72**
	Equiv. Refl.: (-9,7,4)								
**Sapph**	**(2,3,23)**	**12.787**	**74.45**	**0.635**	**0.8**	**3.693**	**2.95**	**46.17**	**46.26**
	Equiv. Refl.: (2,-5,-23), (3,2,-23), (3,-5,23), (5,-2,-23), (5,-3,23), (-2,5,23), (-2,-3,-23), (-3,5,-23), (-3,-2,23) (-5,2,23), (-5,3,-23)								
**Quartz**	**(3,7,-1)**	**13.001**	**78.37**	**0.618**	**0.8**	**2.731**	**2.2**	**34.14**	**34.21**
	Equiv. Refl.: (3,-10,1), (7,3,1), (7,-10,-1), (-10,3,-1), (-10,7,1)								
**Quartz**	**(-3,4,10)**	**12.616**	**71.9**	**0.551**	**0.7**	**4.339**	**3.1**	**54.24**	**54.33**
**Quartz**	**(-3,-1,-10)**	**12.616**	**71.9**	**0.55**	**0.7**	**4.339**	**3.1**	**54.24**	**54.33**
**Quartz**	**(4,-1,10)**	**12.616**	**71.9**	**0.549**	**0.7**	**4.339**	**3.1**	**54.24**	**54.33**
	Equiv. Refl.: (-1,-3,10)								
**Quartz**	**(4,-3,-10)**	**12.616**	**71.9**	**0.548**	**0.7**	**4.339**	**3.09**	**54.24**	**54.33**
	Equiv. Refl.: (-1,4,-10)								
**LiNbO3**	**(1,5,23)**	**12.873**	**75.9**	**0.49**	**1.1**	**3.333**	**3.56**	**41.67**	**41.82**
	Equiv. Refl.: (1,-6,-23), (5,1,-23), (5,-6,23), (6,-1,-23), (6,-5,23), (-1,6,23), (-1,-5,-23), (-5,6,-23), (-5,-1,23) (-6,1,23), (-6,5,-23)								

Ei =	13.273	keV						ΔEg	ΔEt
Cryst	Refl	EB	ΘB	∫ IR dΘ	Width	Ei cotΘ	ΔE	2m, 50µm	2m, 50µm
	(h,k,l)	[keV]	[°]	[µrad]	[µrad]	[meV/µrad]	[meV]	[meV]	[meV]
**Quartz**	**(0,2,-11)**	**12.949**	**77.32**	**0.481**	**0.6**	**2.986**	**1.89**	**37.33**	**37.37**
	Equiv. Refl.: (0,-2,11)								
**Quartz**	**(2,0,11)**	**12.949**	**77.32**	**0.475**	**0.6**	**2.986**	**1.87**	**37.33**	**37.37**
	Equiv. Refl.: (2,-2,-11), (-2,0,-11), (-2,2,11)								
**Quartz**	**(-2,-3,10)**	**13.111**	**81.04**	**0.469**	**0.7**	**2.093**	**1.38**	**26.16**	**26.2**
	Equiv. Refl.: (-3,5,10), (-3,-2,-10)								
**Quartz**	**(-2,5,-10)**	**13.111**	**81.04**	**0.468**	**0.7**	**2.093**	**1.37**	**26.16**	**26.2**
**Sapph**	**(1,3,25)**	**13.107**	**80.94**	**0.467**	**0.7**	**2.117**	**1.54**	**26.46**	**26.51**
	Equiv. Refl.: (1,-4,-25), (3,1,-25), (3,-4,25), (4,-1,-25), (4,-3,25), (-1,4,25), (-1,-3,-25), (-3,4,-25), (-3,-1,25) (-4,1,25), (-4,3,-25)								
**Quartz**	**(5,-3,-10)**	**13.111**	**81.04**	**0.464**	**0.7**	**2.093**	**1.36**	**26.16**	**26.2**
**Quartz**	**(5,-2,10)**	**13.111**	**81.04**	**0.463**	**0.7**	**2.093**	**1.36**	**26.16**	**26.2**
**LiNbO3**	**(2,6,-19)**	**13.143**	**81.96**	**0.442**	**1.3**	**1.874**	**2.38**	**23.43**	**23.55**
	Equiv. Refl.: (2,-8,19), (6,2,19), (6,-8,-19), (8,-2,19), (8,-6,-19), (-2,8,-19), (-2,-6,19), (-6,8,19), (-6,-2,-19) (-8,2,-19), (-8,6,19)								
**Sapph**	**(1,2,-25)**	**12.579**	**71.39**	**0.437**	**0.6**	**4.471**	**2.58**	**55.88**	**55.94**
	Equiv. Refl.: (1,-3,25), (2,1,25), (2,-3,-25), (3,-1,25), (3,-2,-25), (-1,3,-25), (-1,-2,25), (-2,3,25), (-2,-1,-25) (-3,1,-25), (-3,2,25)								
**Quartz**	**(2,3,-10)**	**13.111**	**81.04**	**0.433**	**0.7**	**2.093**	**1.38**	**26.16**	**26.2**
	Equiv. Refl.: (3,2,10)								
**Quartz**	**(2,-5,10)**	**13.111**	**81.04**	**0.432**	**0.7**	**2.093**	**1.37**	**26.16**	**26.2**
	Equiv. Refl.: (3,-5,-10)								
**Quartz**	**(-5,2,-10)**	**13.111**	**81.04**	**0.427**	**0.7**	**2.093**	**1.36**	**26.16**	**26.2**
	Equiv. Refl.: (-5,3,10)								
**Sapph**	**(1,6,-17)**	**12.773**	**74.23**	**0.426**	**0.6**	**3.749**	**2.2**	**46.86**	**46.91**
	Equiv. Refl.: (1,-7,17), (6,1,17), (6,-7,-17), (7,-1,17), (7,-6,-17), (-1,7,-17), (-1,-6,17), (-6,7,17), (-6,-1,-17) (-7,1,-17), (-7,6,17)								
**LiNbO3**	**(3,7,-7)**	**12.749**	**73.85**	**0.411**	**0.9**	**3.844**	**3.52**	**48.05**	**48.18**
	Equiv. Refl.: (3,-10,7), (7,3,7), (7,-10,-7), (10,-3,7), (10,-7,-7), (-3,10,-7), (-3,-7,7), (-7,10,7), (-7,-3,-7) (-10,3,-7), (-10,7,7)								
**Sapph**	**(2,5,18)**	**12.731**	**73.57**	**0.376**	**0.5**	**3.914**	**2.07**	**48.93**	**48.97**
	Equiv. Refl.: (2,5,-18), (2,-7,18), (2,-7,-18), (5,2,18), (5,2,-18), (5,-7,18), (5,-7,-18), (7,-2,18), (7,-2,-18) (7,-5,18), (7,-5,-18), (-2,7,18), (-2,7,-18), (-2,-5,18), (-2,-5,-18), (-5,7,18), (-5,7,-18), (-5,-2,18) (-5,-2,-18), (-7,2,18), (-7,2,-18), (-7,5,18), (-7,5,-18)								
**Quartz**	**(0,7,-7)**	**12.98**	**77.94**	**0.375**	**0.5**	**2.835**	**1.51**	**35.44**	**35.47**
	Equiv. Refl.: (0,-7,7), (7,0,7), (7,-7,-7), (-7,0,-7), (-7,7,7)								
**LiNbO3**	**(3,7,5)**	**12.56**	**71.13**	**0.369**	**0.8**	**4.537**	**3.69**	**56.71**	**56.83**
	Equiv. Refl.: (3,-10,-5), (7,3,-5), (7,-10,5), (10,-3,-5), (10,-7,5), (-3,10,5), (-3,-7,-5), (-7,10,-5), (-7,-3,5) (-10,3,5), (-10,7,-5)								
**LiNbO3**	**(3,6,15)**	**12.915**	**76.66**	**0.353**	**0.9**	**3.146**	**2.84**	**39.33**	**39.43**
	Equiv. Refl.: (3,6,-15), (3,-9,15), (3,-9,-15), (6,3,15), (6,3,-15), (6,-9,15), (6,-9,-15), (9,-3,15), (9,-3,-15) (9,-6,15), (9,-6,-15), (-3,9,15), (-3,9,-15), (-3,-6,15), (-3,-6,-15), (-6,9,15), (-6,9,-15), (-6,-3,15) (-6,-3,-15), (-9,3,15), (-9,3,-15), (-9,6,15), (-9,6,-15)								
**LiNbO3**	**(1,8,-13)**	**13.226**	**85.2**	**0.31**	**1.3**	**1.116**	**1.5**	**13.94**	**14.03**
	Equiv. Refl.: (1,-9,13), (8,1,13), (8,-9,-13), (9,-1,13), (9,-8,-13), (-1,9,-13), (-1,-8,13), (-8,9,13), (-8,-1,-13) (-9,1,-13), (-9,8,13)								
**Quartz**	**(1,8,1)**	**12.501**	**70.37**	**0.3**	**0.4**	**4.735**	**1.69**	**59.19**	**59.22**
	Equiv. Refl.: (1,-9,-1), (8,1,-1), (8,-9,1), (-9,1,1), (-9,8,-1)								
**LiNbO3**	**(2,8,3)**	**12.814**	**74.9**	**0.298**	**0.8**	**3.582**	**2.8**	**44.78**	**44.87**
	Equiv. Refl.: (2,8,-3), (2,-10,3), (2,-10,-3), (8,2,3), (8,2,-3), (8,-10,3), (8,-10,-3), (10,-2,3), (10,-2,-3) (10,-8,3), (10,-8,-3), (-2,10,3), (-2,10,-3), (-2,-8,3), (-2,-8,-3), (-8,10,3), (-8,10,-3), (-8,2,3) (-8,-2,-3), (-10,2,3), (-10,2,-3), (-10,8,3), (-10,8,-3)								

**LiNbO3**	**(2,5,21)**	**12.79**	**74.5**	**0.264**	**0.7**	**3.681**	**2.66**	**46.01**	**46.09**
	Equiv. Refl.: (2,5,-21), (2,-7,21), (2,-7,-21), (5,2,21), (5,2,-21), (5,-7,21), (5,-7,-21), (7,-2,21), (7,-2,-21) (7,-5,21), (7,-5,-21), (-2,7,21), (-2,7,-21), (-2,-5,21), (-2,-5,-21), (-5,7,21), (-5,7,-21), (-5,2,21) (-5,-2,-21), (-7,2,21), (-7,2,-21), (-7,5,21), (-7,5,-21)								
**Sapph**	**(0,8,-8)**	**12.627**	**72.04**	**0.222**	**0.4**	**4.301**	**1.54**	**53.77**	**53.79**
	Equiv. Refl.: (0,-8,8), (8,0,8), (8,-8,-8), (-8,0,-8), (-8,8,8)								
**LiNbO3**	**(2,6,17)**	**12.583**	**71.44**	**0.221**	**0.6**	**4.456**	**2.72**	**55.7**	**55.76**
	Equiv. Refl.: (2,-8,-17), (6,2,-17), (6,-8,17), (8,-2,-17), (8,-6,17), (-2,8,17), (-2,-6,-17), (-6,8,-17), (-6,-2,17) (-8,2,17), (-8,6,-17)								
**Quartz**	**(-1,9,1)**	**12.501**	**70.37**	**0.217**	**0.4**	**4.735**	**1.69**	**59.19**	**59.22**
	Equiv. Refl.: (-1,-8,-1), (-8,9,-1), (-8,-1,1)								
**Quartz**	**(9,-1,-1)**	**12.501**	**70.37**	**0.216**	**0.4**	**4.735**	**1.69**	**59.19**	**59.22**
	Equiv. Refl.: (9,-8,1)								
**LiNbO3**	**(3,5,19)**	**12.92**	**76.76**	**0.155**	**0.6**	**3.124**	**1.81**	**39.05**	**39.09**
	Equiv. Refl.: (3,-8,-19), (5,3,-19), (5,-8,19), (8,-3,-19), (8,-5,19), (-3,8,19), (-3,-5,-19), (-5,8,-19), (-5,-3,19) (-8,3,19), (-8,5,-19), (0,5,9), (0,-5,-9)								
**Quartz**	**(5,0,-9)**	**12.635**	**72.16**	**0.154**	**0.3**	**4.272**	**1.1**	**53.4**	**53.41**
	Equiv. Refl.: (-5,0,9)								
**Quartz**	**(5,-5,9)**	**12.635**	**72.16**	**0.153**	**0.3**	**4.272**	**1.1**	**53.4**	**53.41**
	Equiv. Refl.: (-5,5,-9)								
**LiNbO3**	**(2,3,-25)**	**12.717**	**73.35**	**0.152**	**0.5**	**3.969**	**2.06**	**49.61**	**49.65**
	Equiv. Refl.: (2,-5,25), (3,2,25), (3,-5,-25), (5,-2,25), (5,-3,-25), (-2,5,-25), (-2,-3,25), (-3,5,25), (-3,-2,-25) (-5,2,-25), (-5,3,25)								
**Sapph**	**(1,8,2)**	**12.889**	**76.19**	**0.147**	**0.3**	**3.263**	**0.98**	**40.79**	**40.8**
	Equiv. Refl.: (1,-9,-2), (8,1,-2), (8,-9,2), (9,-1,-2), (9,-8,2), (-1,9,2), (-1,-8,-2), (-8,9,-2), (-8,-1,2) (-9,1,2), (-9,8,-2)								
**Sapph**	**(4,6,1)**	**13.124**	**81.4**	**0.142**	**0.4**	**2.006**	**0.71**	**25.08**	**25.09**
	Equiv. Refl.: (4,-10,-1), (6,4,-1), (6,-10,1), (10,-4,-1), (10,-6,1), (-4,10,1), (-4,-6,-1), (-6,10,-1), (-6,-4,1) (-10,4,1), (-10,6,-1)								
**Sapph**	**(3,6,9)**	**12.69**	**72.96**	**0.14**	**0.3**	**4.069**	**1.1**	**50.86**	**50.87**
	Equiv. Refl.: (3,6,-9), (3,-9,9), (3,-9,-9), (6,3,9), (6,3,-9), (6,-9,9), (6,-9,-9), (9,-3,9), (9,-3,-9) (9,-6,9), (9,-6,-9), (-3,9,9), (-3,9,-9), (-3,-6,9), (-3,-6,-9), (-6,9,9), (-6,9,-9), (-6,-3,9) (-6,-3,-9), (-9,3,9), (-9,3,-9), (-9,6,9), (-9,6,-9)								
**Sapph**	**(3,4,-19)**	**12.883**	**76.08**	**0.121**	**0.3**	**3.289**	**0.87**	**41.11**	**41.12**
	Equiv. Refl.: (3,-7,19), (4,3,19), (4,-7,-19), (7,-3,19), (7,-4,-19), (-3,7,-19), (-3,-4,19), (-4,7,19), (-4,-3,-19) (-7,3,-19), (-7,4,19)								
**LiNbO3**	**(1,8,11)**	**12.858**	**75.64**	**0.114**	**0.5**	**3.398**	**1.63**	**42.47**	**42.5**
	Equiv. Refl.: (1,-9,-11), (8,1,-11), (8,-9,11), (9,-1,-11), (9,-8,11), (-1,9,11), (-1,-8,-11), (-8,9,-11), (-8,-1,11) (-9,1,11), (-9,8,-11)								
**Sapph**	**(1,7,12)**	**12.721**	**73.41**	**0.051**	**0.2**	**3.954**	**0.6**	**49.43**	**49.43**
	Equiv. Refl.: (1,7,-12), (1,-8,12), (1,-8,-12), (7,1,12), (7,1,-12), (7,-8,12), (7,-8,-12), (8,-1,12), (8,-1,-12) (8,-7,12), (8,-7,-12), (-1,8,12), (-1,8,-12), (-1,-7,12), (-1,-7,-12), (-7,8,12), (-7,8,-12), (-7,-1,12) (-7,-1,-12), (-8,1,12), (-8,1,-12), (-8,7,12), (-8,7,-12)								
**LiNbO3**	**(5,5,9)**	**12.697**	**73.05**	**0.007**	**0.1**	**4.044**	**0.5**	**50.55**	**50.55**
	Equiv. Refl.: (5,5,-9), (5,-10,9), (5,-10,-9), (10,-5,9), (10,-5,-9), (-5,10,9), (-5,10,-9), (-5,-5,9), (-5,-5,-9) (-10,5,9), (-10,5,-9)								

Ei = Cryst	11.564 Refl (h,k,l)	keV EB [keV]	ΘB [°]	∫ IR dΘ [µrad]	Width [µrad]	Ei cotΘ [meV/µrad]	ΔE [meV]	ΔEg 2m, 50µm [meV]	ΔEt 2m, 50µm [meV]
**Si**	**(4,4,8)**	**11.184**	**75.27**	**5.7508**	**4.93**	**3.0407**	**15**	**38.01**	**40.86**
	Equiv. Refl.: (4,8,4), (8,4,4)								
**Si**	**(1,7,7)**	**11.357**	**79.15**	**4.9981**	**4.49**	**2.2167**	**9.94**	**27.71**	**29.44**
	Equiv. Refl.: (3,3,9), (3,9,3), (5,5,7), (5,7,5), (7,1,7), (7,5,5), (7,7,1), (9,3,3)								
**Ge**	**(0,2,10)**	**11.175**	**75.09**	**3.7294**	**7.68**	**3.0785**	**23.65**	**38.48**	**45.17**
	Equiv. Refl.: (0,10,2), (2,0,10), (2,6,8), (2,8,6), (2,10,0), (6,2,8), (6,8,2), (8,2,6), (8,6,2) (10,0,2), (10,2,0)								
**Si**	**(1,3,9)**	**10.889**	**70.32**	**3.4379**	**2.86**	**4.1354**	**11.84**	**51.69**	**53.03**
	Equiv. Refl.: (1,9,3), (3,1,9), (3,9,1), (9,1,3), (9,3,1)								
**Ge**	**(1,5,9)**	**11.335**	**78.57**	**2.3733**	**6.72**	**2.3371**	**15.7**	**29.21**	**33.17**
	Equiv. Refl.: (1,9,5), (3,7,7), (5,1,9), (5,9,1), (7,3,7), (7,7,3), (9,1,5), (9,5,1)								
**Ge**	**(1,7,7)**	**10.903**	**70.53**	**1.7032**	**4.56**	**4.0877**	**18.64**	**51.1**	**54.39**
	Equiv. Refl.: (3,3,9), (3,9,3), (5,5,7), (5,7,5), (7,1,7), (7,5,5), (7,7,1), (9,3,3)								
**Sapph**	**(0,7,-10)**	**11.562**	**88.95**	**125.417**	**104.2**	**0.211**	**22.03**	**2.64**	**22.18**
	Equiv. Refl.: (0,-7,10), (7,0,10), (7,-7,-10), (-7,0,-10), (-7,7,10)								
**LiNbO3**	**(2,-5,22)**	**11.555**	**87.75**	**83.255**	**74.6**	**0.455**	**33.92**	**5.68**	**34.39**
	Equiv. Refl.: (5,-2,22)								
**LiNbO3**	**(3,2,22)**	**11.555**	**87.75**	**83.254**	**74.6**	**0.455**	**33.92**	**5.68**	**34.39**
	Equiv. Refl.: (-2,-3,22), (-3,5,22), (-5,3,22)								
**LiNbO3**	**(2,3,-22)**	**11.555**	**87.75**	**79.782**	**74.6**	**0.455**	**33.92**	**5.68**	**34.39**
	Equiv. Refl.: (3,-5,-22), (5,-3,-22), (-2,5,-22), (-3,-2,-22), (-5,2,-22)								
**Quartz**	**(0,1,-10)**	**11.562**	**89.03**	**66.739**	**57.6**	**0.195**	**11.26**	**2.44**	**11.52**
	Equiv. Refl.: (0,-1,10)								
**Quartz**	**(1,0,10)**	**11.562**	**89.03**	**66.698**	**57.6**	**0.195**	**11.26**	**2.44**	**11.52**
**Quartz**	**(-1,0,-10)**	**11.562**	**89.03**	**66.695**	**57.6**	**0.195**	**11.26**	**2.44**	**11.52**
**Quartz**	**(1,-1,-10)**	**11.562**	**89.03**	**66.684**	**57.6**	**0.195**	**11.25**	**2.44**	**11.52**
**Quartz**	**(-1,1,10)**	**11.562**	**89.03**	**66.683**	**57.6**	**0.195**	**11.25**	**2.44**	**11.52**
**Quartz**	**(0,1,10)**	**11.562**	**89.03**	**64.682**	**55.9**	**0.195**	**10.93**	**2.44**	**11.2**
**Quartz**	**(0,-1,-10)**	**11.562**	**89.03**	**64.681**	**55.9**	**0.195**	**10.93**	**2.44**	**11.2**
**Quartz**	**(1,0,-10)**	**11.562**	**89.03**	**64.64**	**55.9**	**0.195**	**10.92**	**2.44**	**11.19**
**Quartz**	**(-1,0,10)**	**11.562**	**89.03**	**64.631**	**55.9**	**0.195**	**10.92**	**2.44**	**11.19**
**Quartz**	**(1,-1,10)**	**11.562**	**89.03**	**64.629**	**55.9**	**0.195**	**10.92**	**2.44**	**11.19**
**Quartz**	**(-1,1,-10)**	**11.562**	**89.03**	**64.619**	**55.9**	**0.195**	**10.92**	**2.44**	**11.19**
**Sapph**	**(3,5,10)**	**11.562**	**88.95**	**46.663**	**42.9**	**0.211**	**9.06**	**2.64**	**9.44**
	Equiv. Refl.: (3,-8,-10), (5,3,-10), (5,-8,10), (8,-3,-10), (8,-5,10), (-3,8,10), (-3,-5,-10), (-5,8,-10), (-5,-3,10) (-8,3,10), (-8,5,-10)								
**LiNbO3**	**(2,7,4)**	**11.521**	**85.08**	**28.835**	**26.6**	**0.996**	**26.51**	**12.45**	**29.29**
	Equiv. Refl.: (7,-9,4), (9,-7,4), (-2,9,4), (-7,-2,4), (-9,2,4)								
**LiNbO3**	**(2,-9,-4)**	**11.521**	**85.08**	**26.932**	**26.6**	**0.996**	**26.51**	**12.45**	**29.29**
	Equiv. Refl.: (7,2,-4), (9,-2,-4), (-2,-7,-4), (-7,9,-4), (-9,7,-4)								
**LiNbO3**	**(-3,3,-24)**	**11.514**	**84.69**	**21.287**	**20.2**	**1.074**	**21.67**	**13.43**	**25.49**
**LiNbO3**	**(0,-3,-24)**	**11.514**	**84.69**	**21.286**	**20.2**	**1.074**	**21.67**	**13.43**	**25.49**
	Equiv. Refl.: (3,0,-24)								
**LiNbO3**	**(0,3,-24)**	**11.514**	**84.69**	**20.898**	**19.9**	**1.074**	**21.42**	**13.43**	**25.28**
	Equiv. Refl.: (3,-3,-24), (-3,0,-24)								
**LiNbO3**	**(2,7,-2)**	**11.417**	**80.85**	**20.59**	**18.8**	**1.863**	**35.04**	**23.29**	**42.08**
	Equiv. Refl.: (7,-9,-2), (9,-7,-2), (-2,9,-2), (-7,-2,-2), (-9,2,-2)								

Ei =	11.564	keV						ΔEg	ΔEt
Cryst	Refl	EB	ΘB	∫ IR dΘ	Width	Ei cotΘ	ΔE	2m, 50µm	2m, 50µm
	(h,k,l)	[keV]	[°]	[µrad]	[µrad]	[meV/µrad]	[meV]	[meV]	[meV]
**LiNbO3**	**(2,-9,2)**	**11.417**	**80.85**	**20.565**	**18.8**	**1.863**	**35.04**	**23.29**	**42.08**
	Equiv. Refl.: (7,2,2), (9,-2,2), (-2,-7,2), (-7,9,2), (-9,7,2)								
**LiNbO3**	**(0,3,24)**	**11.514**	**84.69**	**19.358**	**20.2**	**1.074**	**21.67**	**13.43**	**25.49**
	Equiv. Refl.: (3,-3,24), (-3,0,24)								
**LiNbO3**	**(0,-3,24)**	**11.514**	**84.69**	**19.181**	**19.9**	**1.074**	**21.42**	**13.43**	**25.28**
	Equiv. Refl.: (3,0,24), (-3,3,24)								
**LiNbO3**	**(0,-8,-4)**	**11.267**	**76.98**	**18.159**	**16**	**2.674**	**42.71**	**33.42**	**54.23**
	Equiv. Refl.: (8,0,-4), (-8,8,-4)								
**LiNbO3**	**(0,8,4)**	**11.267**	**76.98**	**17.666**	**16**	**2.674**	**42.71**	**33.42**	**54.23**
	Equiv. Refl.: (8,-8,4), (-8,0,4)								
**LiNbO3**	**(3,6,-6)**	**11.358**	**79.17**	**15.872**	**14.7**	**2.212**	**32.47**	**27.65**	**42.65**
	Equiv. Refl.: (6,-9,-6), (9,-6,-6), (-3,9,-6), (-6,-3,-6), (-9,3,-6)								
**LiNbO3**	**(3,-9,6)**	**11.358**	**79.17**	**15.829**	**14.7**	**2.212**	**32.47**	**27.65**	**42.65**
	Equiv. Refl.: (6,3,6), (9,-3,6), (-3,-6,6), (-6,9,6), (-9,6,6)								
**LiNbO3**	**(4,5,8)**	**11.434**	**81.4**	**15.356**	**14.5**	**1.748**	**25.39**	**21.85**	**33.5**
	Equiv. Refl.: (5,-9,8), (9,-5,8), (-4,9,8), (-5,-4,8), (-9,4,8)								
**LiNbO3**	**(3,-9,-6)**	**11.358**	**79.17**	**14.852**	**13.7**	**2.212**	**30.22**	**27.65**	**40.96**
	Equiv. Refl.: (6,3,-6), (9,-3,-6), (-3,-6,-6), (-6,9,-6), (-9,6,-6)								
**LiNbO3**	**(4,-9,-8)**	**11.434**	**81.4**	**14.757**	**14.5**	**1.748**	**25.39**	**21.85**	**33.5**
	Equiv. Refl.: (5,4,-8), (9,-4,-8), (-4,-5,-8), (-5,9,-8), (-9,5,-8)								
**LiNbO3**	**(3,6,6)**	**11.358**	**79.17**	**14.304**	**13.7**	**2.212**	**30.22**	**27.65**	**40.96**
	Equiv. Refl.: (6,-9,6), (9,-6,6), (-3,9,6), (-6,-3,6), (-9,3,6)								
**LiNbO3**	**(0,4,-22)**	**11.301**	**77.76**	**12.641**	**11.7**	**2.508**	**29.32**	**31.35**	**42.93**
	Equiv. Refl.: (4,-4,-22), (-4,0,-22)								
**LiNbO3**	**(0,-4,22)**	**11.301**	**77.76**	**12.133**	**11.7**	**2.508**	**29.32**	**31.35**	**42.93**
	Equiv. Refl.: (4,0,22), (-4,4,22)								
**LiNbO3**	**(1,1,24)**	**10.999**	**72.02**	**11.871**	**10.7**	**3.753**	**40.21**	**46.92**	**61.79**
	Equiv. Refl.: (1,-2,24), (2,-1,24), (-1,2,24), (-1,-1,24), (-2,1,24)								
**LiNbO3**	**(1,1,-24)**	**10.999**	**72.02**	**11.796**	**10.7**	**3.753**	**40.21**	**46.92**	**61.79**
	Equiv. Refl.: (1,-2,-24), (2,-1,-24), (-1,2,-24), (-1,-1,-24), (-2,1,-24)								
**LiNbO3**	**(0,5,-20)**	**11.328**	**78.4**	**11.608**	**10.4**	**2.373**	**24.61**	**29.66**	**38.54**
	Equiv. Refl.: (5,-5,-20), (-5,0,-20)								
**LiNbO3**	**(1,6,-14)**	**11.06**	**73.03**	**10.91**	**9.6**	**3.529**	**33.8**	**44.11**	**55.57**
	Equiv. Refl.: (6,-7,-14), (7,-6,-14), (-1,7,-14), (-6,-1,-14), (-7,1,-14)								
**Quartz**	**(-1,8,3)**	**11.526**	**85.33**	**10.62**	**9**	**0.944**	**8.5**	**11.8**	**14.55**
	Equiv. Refl.: (-1,-7,-3)								
**Quartz**	**(-7,8,-3)**	**11.526**	**85.33**	**10.619**	**9**	**0.944**	**8.5**	**11.8**	**14.54**
**Quartz**	**(-7,-1,3)**	**11.526**	**85.33**	**10.618**	**9**	**0.944**	**8.5**	**11.8**	**14.54**
**Quartz**	**(8,-1,-3)**	**11.526**	**85.33**	**10.617**	**9**	**0.944**	**8.5**	**11.8**	**14.54**
	Equiv. Refl.: (8,-7,3)								
**LiNbO3**	**(4,5,2)**	**10.897**	**70.44**	**10.563**	**9.6**	**4.108**	**39.24**	**51.35**	**64.63**
	Equiv. Refl.: (5,-9,2), (9,-5,2), (-4,9,2), (-5,-4,2), (-9,4,2)								
**LiNbO3**	**(4,-9,-2)**	**10.897**	**70.44**	**10.401**	**9.6**	**4.108**	**39.24**	**51.35**	**64.63**
	Equiv. Refl.: (5,4,-2), (9,-4,-2), (-4,-5,-2), (-5,9,-2), (-9,5,-2)								
**LiNbO3**	**(3,6,0)**	**11.037**	**72.63**	**10.364**	**9.3**	**3.617**	**33.66**	**45.22**	**56.37**
	Equiv. Refl.: (6,-9,0), (9,-6,0), (-3,9,0), (-6,-3,0), (-9,3,0)								
**LiNbO3**	**(1,-7,14)**	**11.06**	**73.03**	**10.24**	**9.6**	**3.529**	**33.8**	**44.11**	**55.57**
	Equiv. Refl.: (6,1,14), (7,-1,14), (-1,-6,14), (-6,7,14), (-7,6,14)								
**Quartz**	**(1,7,3)**	**11.526**	**85.33**	**10.211**	**9**	**0.944**	**8.5**	**11.8**	**14.55**
	Equiv. Refl.: (1,-8,-3), (7,1,-3), (7,-8,3)								
**Quartz**	**(-8,1,3)**	**11.526**	**85.33**	**10.209**	**9**	**0.944**	**8.5**	**11.8**	**14.54**

Ei =	11.564	keV						ΔEg	ΔEt
Cryst	Refl	EB	ΘB	∫ IR dΘ	Width	Ei cotΘ	ΔE	2m, 50µm	2m, 50µm
	(h,k,l)	[keV]	[°]	[µrad]	[µrad]	[meV/µrad]	[meV]	[meV]	[meV]
**Quartz**	**(-8,7,-3)**	**11.526**	**85.33**	**10.208**	**9**	**0.944**	**8.5**	**11.8**	**14.54**
**LiNbO3**	**(3,-9,0)**	**11.037**	**72.63**	**10.147**	**9.3**	**3.617**	**33.66**	**45.22**	**56.37**
	Equiv. Refl.: (6,3,0), (9,-3,0), (-3,-6,0), (-6,9,0), (-9,6,0)								
**LiNbO3**	**(0,-5,20)**	**11.328**	**78.4**	**10.067**	**10.4**	**2.373**	**24.61**	**29.66**	**38.54**
	Equiv. Refl.: (5,0,20), (-5,5,20)								
**LiNbO3**	**(3,-7,16)**	**11.078**	**73.34**	**10.055**	**9**	**3.462**	**31.14**	**43.27**	**53.31**
	Equiv. Refl.: (4,3,16), (7,-3,16), (-3,-4,16), (-4,7,16), (-7,4,16)								
**Quartz**	**(-8,1,-3)**	**11.526**	**85.33**	**10.012**	**8.4**	**0.944**	**7.96**	**11.8**	**14.23**
**Quartz**	**(1,7,-3)**	**11.526**	**85.33**	**10.011**	**8.4**	**0.944**	**7.96**	**11.8**	**14.23**
	Equiv. Refl.: (7,1,3), (7,-8,-3), (-8,7,3)								
**Quartz**	**(1,-8,3)**	**11.526**	**85.33**	**10.009**	**8.4**	**0.944**	**7.95**	**11.8**	**14.23**
**LiNbO3**	**(4,4,12)**	**11.027**	**72.48**	**9.988**	**8.9**	**3.652**	**32.52**	**45.64**	**56.05**
	Equiv. Refl.: (4,-8,12), (8,-4,12), (-4,8,12), (-4,-4,12), (-8,4,12)								
**LiNbO3**	**(4,-9,4)**	**11.006**	**72.13**	**9.967**	**8.8**	**3.727**	**32.82**	**46.59**	**56.99**
	Equiv. Refl.: (5,4,4), (9,-4,4), (-4,-5,4), (-5,9,4), (-9,5,4)								
**LiNbO3**	**(4,4,-12)**	**11.027**	**72.48**	**9.512**	**8.9**	**3.652**	**32.52**	**45.64**	**56.05**
	Equiv. Refl.: (-4,8,-12), (-4,-4,-12), (-8,4,-12)								
**LiNbO3**	**(4,-8,-12)**	**11.027**	**72.48**	**9.511**	**8.9**	**3.652**	**32.52**	**45.64**	**56.05**
	Equiv. Refl.: (8,-4,-12)								
**LiNbO3**	**(3,4,-16)**	**11.078**	**73.34**	**9.507**	**9**	**3.462**	**31.14**	**43.27**	**53.31**
	Equiv. Refl.: (4,-7,-16), (7,-4,-16), (-3,7,-16), (-4,-3,-16), (-7,3,-16)								
**LiNbO3**	**(2,-8,10)**	**10.979**	**71.7**	**9.496**	**8.6**	**3.825**	**32.72**	**47.82**	**57.94**
	Equiv. Refl.: (6,2,10), (8,-2,10), (-2,-6,10), (-6,8,10), (-8,6,10)								
**Quartz**	**(4,-2,9)**	**11.491**	**83.56**	**9.492**	**7.8**	**1.306**	**10.21**	**16.33**	**19.26**
**Quartz**	**(4,-2,-9)**	**11.491**	**83.56**	**9.491**	**7.8**	**1.306**	**10.21**	**16.33**	**19.26**
**Quartz**	**(-2,4,-9)**	**11.491**	**83.56**	**9.488**	**7.8**	**1.306**	**10.21**	**16.33**	**19.26**
**Quartz**	**(-2,-2,9)**	**11.491**	**83.56**	**9.486**	**7.8**	**1.306**	**10.21**	**16.33**	**19.26**
**Quartz**	**(-2,-2,-9)**	**11.491**	**83.56**	**9.485**	**7.8**	**1.306**	**10.21**	**16.33**	**19.25**
**Quartz**	**(-2,4,9)**	**11.491**	**83.56**	**9.483**	**7.8**	**1.306**	**10.2**	**16.33**	**19.25**
**Sapph**	**(0,4,20)**	**11.284**	**77.37**	**9.444**	**7.6**	**2.591**	**19.7**	**32.38**	**37.9**
	Equiv. Refl.: (0,-4,-20), (4,0,-20), (4,-4,20), (-4,0,20), (-4,4,-20)								
**LiNbO3**	**(4,5,-4)**	**11.006**	**72.13**	**9.329**	**8.8**	**3.727**	**32.82**	**46.59**	**56.99**
	Equiv. Refl.: (5,-9,-4), (9,-5,-4), (-4,9,-4), (-5,-4,-4), (-9,4,-4)								
**LiNbO3**	**(1,-4,-22)**	**11.042**	**72.71**	**9.302**	**8.5**	**3.599**	**30.55**	**44.98**	**54.38**
	Equiv. Refl.: (3,1,-22), (4,-1,-22), (-1,-3,-22), (-3,4,-22), (-4,3,-22)								
**LiNbO3**	**(2,6,-10)**	**10.979**	**71.7**	**9.218**	**8.6**	**3.825**	**32.72**	**47.82**	**57.94**
	Equiv. Refl.: (6,-8,-10), (8,-6,-10), (-2,8,-10), (-6,-2,-10), (-8,2,-10)								
**Quartz**	**(8,-1,3)**	**11.526**	**85.33**	**9.19**	**8.4**	**0.944**	**7.96**	**11.8**	**14.23**
	Equiv. Refl.: (8,-7,-3), (-1,-7,3), (-7,8,3), (-7,-1,-3)								
**Quartz**	**(-1,8,-3)**	**11.526**	**85.33**	**9.189**	**8.4**	**0.944**	**7.95**	**11.8**	**14.23**
**Quartz**	**(-4,2,-9)**	**11.491**	**83.56**	**9.111**	**7.8**	**1.306**	**10.21**	**16.33**	**19.26**
**Quartz**	**(-4,2,9)**	**11.491**	**83.56**	**9.11**	**7.8**	**1.306**	**10.21**	**16.33**	**19.26**
**Quartz**	**(2,-4,9)**	**11.491**	**83.56**	**9.109**	**7.8**	**1.306**	**10.21**	**16.33**	**19.26**
**Quartz**	**(2,2,-9)**	**11.491**	**83.56**	**9.107**	**7.8**	**1.306**	**10.21**	**16.33**	**19.26**
**Quartz**	**(2,2,9)**	**11.491**	**83.56**	**9.104**	**7.8**	**1.306**	**10.21**	**16.33**	**19.25**
**Quartz**	**(2,-4,-9)**	**11.491**	**83.56**	**9.102**	**7.8**	**1.306**	**10.2**	**16.33**	**19.25**
**LiNbO3**	**(1,3,22)**	**11.042**	**72.71**	**9.058**	**8.5**	**3.599**	**30.55**	**44.98**	**54.38**
	Equiv. Refl.: (3,-4,22), (4,-3,22), (-1,4,22), (-3,-1,22), (-4,1,22)								
**Quartz**	**(-5,9,-1)**	**11.437**	**81.5**	**7.599**	**6.3**	**1.728**	**10.83**	**21.59**	**24.16**
	Equiv. Refl.: (-5,-4,1)								

Ei =	11.564	keV						ΔEg	ΔEt
Cryst	Refl	EB	ΘB	∫ IR dΘ	Width	Ei cotΘ	ΔE	2m, 50μm	2m, 50μm
	(h,k,l)	[keV]	[°]	[μrad]	[μrad]	[meV/μrad]	[meV]	[meV]	[meV]
**Quartz**	**(-4,-5,-1)**	**11.437**	**81.5**	**7.598**	**6.3**	**1.728**	**10.83**	**21.59**	**24.16**
**Quartz**	**(9,-4,-1)**	**11.437**	**81.5**	**7.597**	**6.3**	**1.728**	**10.83**	**21.59**	**24.16**
	Equiv. Refl.: (9,-5,1), (-4,9,1)								
**Quartz**	**(5,4,-1)**	**11.437**	**81.5**	**7.417**	**6.3**	**1.728**	**10.83**	**21.59**	**24.16**
	Equiv. Refl.: (5,-9,1)								
**Quartz**	**(4,5,1)**	**11.437**	**81.5**	**7.416**	**6.3**	**1.728**	**10.83**	**21.59**	**24.16**
	Equiv. Refl.: (4,-9,-1), (-9,4,1), (-9,5,-1)								
**Quartz**	**(6,-8,-4)**	**11.465**	**82.48**	**7.181**	**6**	**1.526**	**9.21**	**19.08**	**21.18**
**Quartz**	**(6,2,4)**	**11.465**	**82.48**	**7.18**	**6**	**1.526**	**9.21**	**19.08**	**21.18**
	Equiv. Refl.: (-8,2,-4), (-8,6,4)								
**Quartz**	**(2,6,-4)**	**11.465**	**82.48**	**7.177**	**6**	**1.526**	**9.2**	**19.08**	**21.18**
	Equiv. Refl.: (2,-8,4)								
**Quartz**	**(-6,8,4)**	**11.465**	**82.48**	**6.889**	**6**	**1.526**	**9.21**	**19.08**	**21.18**
**Quartz**	**(8,-2,4)**	**11.465**	**82.48**	**6.888**	**6**	**1.526**	**9.21**	**19.08**	**21.18**
	Equiv. Refl.: (8,-6,-4), (-6,-2,-4)								
**Quartz**	**(-2,8,-4)**	**11.465**	**82.48**	**6.885**	**6**	**1.526**	**9.2**	**19.08**	**21.18**
	Equiv. Refl.: (-2,-6,4)								
**LiNbO3**	**(0,-8,2)**	**11.16**	**74.81**	**6.553**	**6.4**	**3.14**	**20.15**	**39.26**	**44.12**
	Equiv. Refl.: (8,0,2), (-8,8,2)								
**Sapph**	**(0,7,8)**	**11.202**	**75.62**	**6.446**	**5.4**	**2.964**	**16.05**	**37.05**	**40.38**
	Equiv. Refl.: (0,-7,-8), (7,0,-8), (7,-7,8), (-7,0,8), (-7,7,-8)								
**Sapph**	**(1,5,-16)**	**11.335**	**78.58**	**6.418**	**5.5**	**2.337**	**12.89**	**29.21**	**31.93**
	Equiv. Refl.: (1,-6,16), (5,1,16), (5,-6,-16), (6,-1,16), (6,-5,-16), (-1,-6,-16), (-1,-5,16), (-5,6,16), (-5,-1,-16) (-6,1,-16), (-6,5,16)								
**Sapph**	**(1,6,10)**	**10.959**	**71.39**	**6.329**	**5.1**	**3.895**	**19.94**	**48.69**	**52.61**
	Equiv. Refl.: (1,-7,-10), (6,1,-10), (6,-7,10), (7,-1,-10), (7,-6,10), (-1,7,10), (-1,-6,-10), (-6,7,-10), (-6,-1,10) (-7,1,10), (-7,6,-10)								
**Sapph**	**(2,6,-4)**	**11.015**	**72.28**	**6.294**	**5.1**	**3.695**	**19**	**46.19**	**49.95**
	Equiv. Refl.: (2,-8,4), (6,2,4), (6,-8,-4), (8,-2,4), (8,-6,-4), (-2,8,-4), (-2,-6,4), (-6,8,4), (-6,-2,-4) (-8,2,-4), (-8,6,4)								
**LiNbO3**	**(0,8,-2)**	**11.16**	**74.81**	**6.156**	**6.4**	**3.14**	**20.15**	**39.26**	**44.12**
	Equiv. Refl.: (8,-8,-2), (-8,0,-2)								
**Quartz**	**(7,0,-4)**	**11.183**	**75.26**	**6.06**	**4.8**	**3.043**	**14.74**	**38.03**	**40.79**
	Equiv. Refl.: (7,-7,4)								
**Quartz**	**(-7,0,4)**	**11.183**	**75.26**	**6.059**	**4.8**	**3.043**	**14.74**	**38.03**	**40.79**
	Equiv. Refl.: (-7,7,-4)								
**Quartz**	**(0,7,4)**	**11.183**	**75.26**	**6.056**	**4.8**	**3.043**	**14.73**	**38.03**	**40.79**
	Equiv. Refl.: (0,-7,-4)								
**Sapph**	**(1,3,-20)**	**10.979**	**71.7**	**6.034**	**4.9**	**3.824**	**18.81**	**47.8**	**51.37**
	Equiv. Refl.: (1,-4,20), (3,1,20), (3,-4,-20), (4,-1,20), (4,-3,-20), (-1,4,-20), (-1,-3,20), (-3,4,20), (-3,-1,-20) (-4,1,-20), (-4,3,20)								
**Sapph**	**(1,7,0)**	**11.358**	**79.17**	**5.971**	**5.2**	**2.212**	**11.43**	**27.65**	**29.91**
	Equiv. Refl.: (1,-8,0), (7,1,0), (7,-8,0), (8,-1,0), (8,-7,0), (-1,8,0), (-1,-7,0), (-7,8,0), (-7,-1,0) (-8,1,0), (-8,7,0)								
**Quartz**	**(-1,6,-7)**	**11.414**	**80.75**	**5.947**	**5.1**	**1.882**	**9.59**	**23.53**	**25.41**
**Quartz**	**(6,-1,7)**	**11.414**	**80.75**	**5.946**	**5.1**	**1.882**	**9.59**	**23.53**	**25.41**
	Equiv. Refl.: (-1,-5,7)								
**Quartz**	**(6,-5,-7)**	**11.414**	**80.75**	**5.945**	**5.1**	**1.882**	**9.59**	**23.53**	**25.41**
**Quartz**	**(-5,6,7)**	**11.414**	**80.75**	**5.94**	**5.1**	**1.882**	**9.58**	**23.53**	**25.41**
	Equiv. Refl.: (-5,-1,-7)								

Ei =	11.564	keV						ΔEg	ΔEt
Cryst	Refl (h,k,l)	EB [keV]	ΘB [°]	∫ IR dΘ [μrad]	Width [μrad]	Ei cotΘ [meV/μrad]	ΔE [meV]	2m, 50μm [meV]	2m, 50μm [meV]
**Quartz**	**(1,-6,7)**	**11.414**	**80.75**	**5.912**	**5.1**	**1.882**	**9.59**	**23.53**	**25.41**
**Quartz**	**(1,5,-7)**	**11.414**	**80.75**	**5.911**	**5.1**	**1.882**	**9.59**	**23.53**	**25.41**
	Equiv. Refl.: (-6,1,-7)								
**Quartz**	**(-6,5,7)**	**11.414**	**80.75**	**5.91**	**5.1**	**1.882**	**9.59**	**23.53**	**25.41**
**Quartz**	**(5,1,7)**	**11.414**	**80.75**	**5.904**	**5.1**	**1.882**	**9.58**	**23.53**	**25.41**
	Equiv. Refl.: (5,-6,-7)								
**Quartz**	**(0,4,8)**	**10.87**	**70.06**	**5.315**	**4.3**	**4.196**	**17.93**	**52.45**	**55.43**
	Equiv. Refl.: (0,-4,-8)								
**Quartz**	**(4,0,-8)**	**10.87**	**70.06**	**5.313**	**4.3**	**4.196**	**17.93**	**52.45**	**55.43**
	Equiv. Refl.: (-4,0,8)								
**Quartz**	**(4,-4,8)**	**10.87**	**70.06**	**5.312**	**4.3**	**4.196**	**17.93**	**52.45**	**55.43**
	Equiv. Refl.: (-4,4,-8)								
**Quartz**	**(0,6,-6)**	**11.126**	**74.18**	**5.164**	**4.2**	**3.277**	**13.9**	**40.96**	**43.26**
	Equiv. Refl.: (0,-6,6)								
**Quartz**	**(6,0,6)**	**11.126**	**74.18**	**5.158**	**4.2**	**3.277**	**13.89**	**40.96**	**43.25**
	Equiv. Refl.: (6,-6,-6), (-6,0,-6), (-6,6,6)								
**Quartz**	**(-5,1,8)**	**11.348**	**78.91**	**5.141**	**4.2**	**2.266**	**9.42**	**28.33**	**29.85**
	Equiv. Refl.: (-5,4,-8)								
**Quartz**	**(1,-5,-8)**	**11.348**	**78.91**	**5.14**	**4.2**	**2.266**	**9.42**	**28.33**	**29.85**
**Quartz**	**(1,4,8)**	**11.348**	**78.91**	**5.139**	**4.2**	**2.266**	**9.42**	**28.33**	**29.85**
**Quartz**	**(4,1,-8)**	**11.348**	**78.91**	**5.134**	**4.2**	**2.266**	**9.41**	**28.33**	**29.85**
	Equiv. Refl.: (4,-5,8)								
**Quartz**	**(5,-1,-8)**	**11.348**	**78.91**	**4.974**	**4.2**	**2.266**	**9.42**	**28.33**	**29.85**
	Equiv. Refl.: (5,-4,8)								
**Quartz**	**(-1,5,8)**	**11.348**	**78.91**	**4.973**	**4.2**	**2.266**	**9.42**	**28.33**	**29.85**
**Quartz**	**(-1,-4,-8)**	**11.348**	**78.91**	**4.971**	**4.2**	**2.266**	**9.42**	**28.33**	**29.85**
**Quartz**	**(-4,5,-8)**	**11.348**	**78.91**	**4.968**	**4.2**	**2.266**	**9.41**	**28.33**	**29.85**
	Equiv. Refl.: (-4,-1,8)								
**Quartz**	**(4,5,-1)**	**11.437**	**81.5**	**4.796**	**4.2**	**1.728**	**7.18**	**21.59**	**22.76**
	Equiv. Refl.: (4,-9,1), (5,4,1), (5,-9,-1), (-9,4,-1), (-9,5,1)								
**Quartz**	**(9,-4,1)**	**11.437**	**81.5**	**4.747**	**4.2**	**1.728**	**7.18**	**21.59**	**22.76**
	Equiv. Refl.: (9,-5,-1), (-4,9,-1), (-4,-5,1), (-5,9,1), (-5,-4,-1)								
**Quartz**	**(-4,7,-6)**	**11.221**	**76.01**	**4.521**	**3.7**	**2.882**	**10.54**	**36.02**	**37.53**
	Equiv. Refl.: (-4,-3,6)								
**Quartz**	**(-3,-4,-6)**	**11.221**	**76.01**	**4.518**	**3.7**	**2.882**	**10.53**	**36.02**	**37.53**
**Quartz**	**(7,-3,-6)**	**11.221**	**76.01**	**4.517**	**3.7**	**2.882**	**10.53**	**36.02**	**37.53**
	Equiv. Refl.: (7,-4,6), (-3,7,6)								
**Sapph**	**(2,2,21)**	**11.297**	**77.66**	**4.445**	**3.9**	**2.529**	**9.77**	**31.61**	**33.09**
	Equiv. Refl.: (2,2,-21), (2,-4,21), (2,-4,-21), (4,-2,21), (4,-2,-21), (-2,4,21), (-2,4,-21), (-2,-2,21), (-2,-2,-21) (-4,2,21), (-4,2,-21)								
**Quartz**	**(4,3,-6)**	**11.221**	**76.01**	**4.437**	**3.7**	**2.882**	**10.54**	**36.02**	**37.53**
**Quartz**	**(4,-7,6)**	**11.221**	**76.01**	**4.436**	**3.7**	**2.882**	**10.54**	**36.02**	**37.53**
**Quartz**	**(3,4,6)**	**11.221**	**76.01**	**4.433**	**3.7**	**2.882**	**10.53**	**36.02**	**37.53**
**Quartz**	**(3,-7,-6)**	**11.221**	**76.01**	**4.432**	**3.7**	**2.882**	**10.53**	**36.02**	**37.53**
	Equiv. Refl.: (-7,3,6), (-7,4,-6)								
**Quartz**	**(2,-6,7)**	**11.131**	**74.28**	**4.21**	**3.4**	**3.256**	**10.98**	**40.69**	**42.15**
**Quartz**	**(2,4,-7)**	**11.131**	**74.28**	**4.209**	**3.4**	**3.256**	**10.98**	**40.69**	**42.15**
**Quartz**	**(4,-6,-7)**	**11.131**	**74.28**	**4.208**	**3.4**	**3.256**	**10.98**	**40.69**	**42.15**
	Equiv. Refl.: (-6,2,-7), (-6,4,7)								
**Quartz**	**(4,2,7)**	**11.131**	**74.28**	**4.207**	**3.4**	**3.256**	**10.98**	**40.69**	**42.15**

Ei =	11.564	keV						ΔEg	ΔEt
Cryst	Refl	EB	ΘB	∫ IR dΘ	Width	Ei cotΘ	ΔE	2m, 50µm	2m, 50µm
	(h,k,l)	[keV]	[°]	[µrad]	[µrad]	[meV/µrad]	[meV]	[meV]	[meV]
**Quartz**	**(-2,6,-7)**	**11.131**	**74.28**	**4.111**	**3.4**	**3.256**	**10.98**	**40.69**	**42.15**
	Equiv. Refl.: (-2,-4,7)								
**Quartz**	**(6,-2,7)**	**11.131**	**74.28**	**4.11**	**3.4**	**3.256**	**10.98**	**40.69**	**42.15**
	Equiv. Refl.: (6,-4,-7), (-4,6,7)								
**Quartz**	**(-4,-2,-7)**	**11.131**	**74.28**	**4.108**	**3.4**	**3.256**	**10.98**	**40.69**	**42.15**
**Quartz**	**(8,-1,0)**	**11**	**72.03**	**4.09**	**3.2**	**3.75**	**12.1**	**46.88**	**48.42**
	Equiv. Refl.: (8,-7,0), (-1,8,0), (-1,-7,0), (-7,8,0), (-7,-1,0)								
**Quartz**	**(1,7,0)**	**11**	**72.03**	**4.016**	**3.2**	**3.75**	**12.1**	**46.88**	**48.42**
	Equiv. Refl.: (1,-8,0), (7,1,0), (7,-8,0), (-8,1,0), (-8,7,0)								
**Sapph**	**(1,7,3)**	**11.448**	**81.88**	**3.688**	**3.5**	**1.65**	**5.77**	**20.63**	**21.42**
	Equiv. Refl.: (1,7,-3), (1,-8,3), (1,-8,-3), (7,1,3), (7,1,-3), (7,-8,3), (7,-8,-3), (8,-1,3), (8,-1,-3) (8,-7,3), (8,-7,-3), (-1,8,3), (-1,8,-3), (-1,-7,3), (-1,-7,-3), (-7,8,3), (-7,8,-3), (-7,-1,3) (-7,-1,-3), (-8,1,3), (-8,1,-3), (-8,7,3), (-8,7,-3)								
**Sapph**	**(2,6,-7)**	**11.351**	**79**	**3.679**	**3.4**	**2.249**	**7.6**	**28.11**	**29.12**
	Equiv. Refl.: (2,-8,7), (6,2,7), (6,-8,-7), (8,-2,7), (8,-6,-7), (-2,8,-7), (-2,-6,7), (-6,8,7), (-6,-2,-7) (-8,2,-7), (-8,6,7)								
**Quartz**	**(2,6,-3)**	**11.056**	**72.95**	**3.651**	**3**	**3.547**	**10.58**	**44.34**	**45.58**
	Equiv. Refl.: (2,-8,3)								
**Quartz**	**(-8,2,-3)**	**11.056**	**72.95**	**3.65**	**3**	**3.547**	**10.58**	**44.34**	**45.58**
**Quartz**	**(-8,6,3)**	**11.056**	**72.95**	**3.649**	**3**	**3.547**	**10.58**	**44.34**	**45.58**
**Quartz**	**(6,2,3)**	**11.056**	**72.95**	**3.648**	**3**	**3.547**	**10.57**	**44.34**	**45.58**
	Equiv. Refl.: (6,-8,-3)								
**Quartz**	**(-2,8,-3)**	**11.056**	**72.95**	**3.63**	**3**	**3.547**	**10.58**	**44.34**	**45.58**
	Equiv. Refl.: (-2,-6,3)								
**Quartz**	**(8,-2,3)**	**11.056**	**72.95**	**3.629**	**3**	**3.547**	**10.58**	**44.34**	**45.58**
	Equiv. Refl.: (8,-6,-3)								
**Quartz**	**(-6,8,3)**	**11.056**	**72.95**	**3.628**	**3**	**3.547**	**10.57**	**44.34**	**45.58**
	Equiv. Refl.: (-6,-2,-3)								
**Quartz**	**(1,-7,-5)**	**11.143**	**74.5**	**3.515**	**2.8**	**3.208**	**9.01**	**40.1**	**41.1**
**Quartz**	**(1,6,5)**	**11.143**	**74.5**	**3.514**	**2.8**	**3.208**	**9.01**	**40.1**	**41.1**
**Quartz**	**(-7,6,-5)**	**11.143**	**74.5**	**3.513**	**2.8**	**3.208**	**9.01**	**40.1**	**41.09**
**Quartz**	**(6,1,-5)**	**11.143**	**74.5**	**3.512**	**2.8**	**3.208**	**9**	**40.1**	**41.09**
	Equiv. Refl.: (6,-7,5), (-7,1,5)								
**Sapph**	**(2,4,16)**	**11.031**	**72.54**	**3.492**	**3**	**3.637**	**10.88**	**45.46**	**46.74**
	Equiv. Refl.: (2,-6,-16), (4,2,-16), (4,-6,16), (6,-2,-16), (6,-4,16), (-2,6,16), (-2,-4,-16), (-4,6,-16), (-4,-2,16) (-6,2,16), (-6,4,-16)								
**Quartz**	**(4,4,-4)**	**11.088**	**73.5**	**3.393**	**2.8**	**3.424**	**9.56**	**42.8**	**43.86**
	Equiv. Refl.: (4,-8,4)								
**Quartz**	**(-8,4,4)**	**11.088**	**73.5**	**3.392**	**2.8**	**3.424**	**9.56**	**42.8**	**43.86**
	Equiv. Refl.: (-8,4,-4)								
**Quartz**	**(4,-8,-4)**	**11.088**	**73.5**	**3.391**	**2.8**	**3.424**	**9.55**	**42.8**	**43.86**
**Quartz**	**(4,4,4)**	**11.088**	**73.5**	**3.39**	**2.8**	**3.424**	**9.55**	**42.8**	**43.86**
**Quartz**	**(-4,8,-4)**	**11.088**	**73.5**	**3.323**	**2.8**	**3.424**	**9.56**	**42.8**	**43.86**
	Equiv. Refl.: (-4,-4,4)								
**Quartz**	**(8,-4,4)**	**11.088**	**73.5**	**3.322**	**2.8**	**3.424**	**9.56**	**42.8**	**43.86**
	Equiv. Refl.: (8,-4,-4)								
**Quartz**	**(-4,8,4)**	**11.088**	**73.5**	**3.321**	**2.8**	**3.424**	**9.55**	**42.8**	**43.86**
**Quartz**	**(-4,-4,-4)**	**11.088**	**73.5**	**3.32**	**2.8**	**3.424**	**9.55**	**42.8**	**43.86**
**Quartz**	**(-1,7,5)**	**11.143**	**74.5**	**3.173**	**2.8**	**3.208**	**9.01**	**40.1**	**41.1**
**Quartz**	**(-1,-6,-5)**	**11.143**	**74.5**	**3.172**	**2.8**	**3.208**	**9.01**	**40.1**	**41.1**

Ei =	11.564	keV						ΔEg	ΔEt
Cryst	Refl	EB	ΘB	∫ IR dΘ	Width	Ei cotΘ	ΔE	2m, 50µm	2m, 50µm
	(h,k,l)	[keV]	[°]	[µrad]	[µrad]	[meV/µrad]	[meV]	[meV]	[meV]
**Quartz**	**(7,-1,-5)**	**11.143**	**74.5**	**3.171**	**2.8**	**3.208**	**9**	**40.1**	**41.09**
	Equiv. Refl.: (7,-6,5), (-6,-1,5)								
**Quartz**	**(-6,7,-5)**	**11.143**	**74.5**	**3.17**	**2.8**	**3.208**	**9**	**40.1**	**41.09**
**Quartz**	**(-3,5,8)**	**11.16**	**74.8**	**3.14**	**2.8**	**3.142**	**8.78**	**39.27**	**40.24**
	Equiv. Refl.: (-3,-2,-8)								
**Quartz**	**(5,-2,8)**	**11.16**	**74.8**	**3.138**	**2.8**	**3.142**	**8.77**	**39.27**	**40.24**
	Equiv. Refl.: (5,-3,-8), (-2,5,-8)								
**Quartz**	**(-2,-3,8)**	**11.16**	**74.8**	**3.137**	**2.8**	**3.142**	**8.77**	**39.27**	**40.24**
**Quartz**	**(8,-1,-1)**	**11.06**	**73.02**	**3.1**	**2.5**	**3.532**	**8.74**	**44.15**	**45.01**
	Equiv. Refl.: (8,-7,1), (-1,8,1), (-1,-7,-1), (-7,8,-1), (-7,-1,1)								
**Quartz**	**(5,2,-6)**	**11.408**	**80.59**	**3.075**	**2.7**	**1.916**	**5.23**	**23.95**	**24.51**
	Equiv. Refl.: (5,-7,6)								
**Quartz**	**(-7,2,6)**	**11.408**	**80.59**	**3.073**	**2.7**	**1.916**	**5.23**	**23.95**	**24.51**
**Quartz**	**(2,-7,-6)**	**11.408**	**80.59**	**3.072**	**2.7**	**1.916**	**5.22**	**23.95**	**24.51**
	Equiv. Refl.: (-7,5,-6)								
**Quartz**	**(2,5,6)**	**11.408**	**80.59**	**3.071**	**2.7**	**1.916**	**5.22**	**23.95**	**24.51**
**Quartz**	**(1,7,1)**	**11.06**	**73.02**	**3.049**	**2.5**	**3.532**	**8.74**	**44.15**	**45.01**
	Equiv. Refl.: (1,-8,-1), (7,1,-1), (7,-8,1), (-8,1,1), (-8,7,-1)								
**Sapph**	**(4,4,9)**	**11.273**	**77.13**	**3.034**	**2.7**	**2.642**	**7.18**	**33.03**	**33.8**
	Equiv. Refl.: (4,4,-9), (4,-8,9), (4,-8,-9), (8,-4,9), (8,-4,-9), (-4,8,9), (-4,8,-9), (-4,-4,9), (-4,-4,-9) (-8,4,9), (-8,4,-9)								
**Quartz**	**(-5,7,-6)**	**11.408**	**80.59**	**2.995**	**2.7**	**1.916**	**5.23**	**23.95**	**24.51**
	Equiv. Refl.: (-5,-2,6)								
**Quartz**	**(7,-2,-6)**	**11.408**	**80.59**	**2.993**	**2.7**	**1.916**	**5.23**	**23.95**	**24.51**
**Quartz**	**(7,-5,6)**	**11.408**	**80.59**	**2.992**	**2.7**	**1.916**	**5.23**	**23.95**	**24.51**
**Quartz**	**(-2,7,6)**	**11.408**	**80.59**	**2.991**	**2.7**	**1.916**	**5.22**	**23.95**	**24.51**
**Quartz**	**(-2,-5,-6)**	**11.408**	**80.59**	**2.99**	**2.7**	**1.916**	**5.22**	**23.95**	**24.51**
**Quartz**	**(3,5,-4)**	**11.183**	**75.26**	**2.976**	**2.4**	**3.043**	**7.45**	**38.03**	**38.76**
	Equiv. Refl.: (3,-8,4), (5,3,4), (5,-8,-4), (-8,3,-4), (-8,5,4)								
**Quartz**	**(3,2,8)**	**11.16**	**74.8**	**2.971**	**2.8**	**3.142**	**8.78**	**39.27**	**40.24**
	Equiv. Refl.: (3,-5,-8)								
**Quartz**	**(2,3,-8)**	**11.16**	**74.8**	**2.97**	**2.8**	**3.142**	**8.77**	**39.27**	**40.24**
	Equiv. Refl.: (2,-5,8), (-5,3,8)								
**Quartz**	**(-5,2,-8)**	**11.16**	**74.8**	**2.969**	**2.8**	**3.142**	**8.77**	**39.27**	**40.24**
**Quartz**	**(3,0,9)**	**11.21**	**75.79**	**2.967**	**2.5**	**2.927**	**7.24**	**36.59**	**37.3**
	Equiv. Refl.: (3,-3,-9), (-3,0,-9), (-3,3,9)								
**Quartz**	**(0,3,-9)**	**11.21**	**75.79**	**2.958**	**2.5**	**2.927**	**7.23**	**36.59**	**37.3**
	Equiv. Refl.: (0,-3,9)								
**Quartz**	**(-3,8,-4)**	**11.183**	**75.26**	**2.938**	**2.4**	**3.043**	**7.45**	**38.03**	**38.76**
**Quartz**	**(8,-3,4)**	**11.183**	**75.26**	**2.937**	**2.4**	**3.043**	**7.45**	**38.03**	**38.76**
	Equiv. Refl.: (8,-5,-4), (-3,-5,4), (-5,8,4), (-5,-3,-4)								
**LiNbO3**	**(2,7,1)**	**11.39**	**80.06**	**2.706**	**4.3**	**2.027**	**8.74**	**25.33**	**26.8**
	Equiv. Refl.: (2,-9,-1), (7,2,-1), (7,-9,1), (9,-2,-1), (9,-7,1), (-2,9,1), (-2,-7,-1), (-7,9,-1), (-7,-2,1) (-9,2,1), (-9,7,-1)								
**Quartz**	**(-1,5,-8)**	**11.348**	**78.91**	**2.578**	**2.5**	**2.266**	**5.57**	**28.33**	**28.87**
	Equiv. Refl.: (-1,-4,8)								
**Quartz**	**(5,-1,8)**	**11.348**	**78.91**	**2.577**	**2.5**	**2.266**	**5.57**	**28.33**	**28.87**
	Equiv. Refl.: (5,-4,-8), (-4,5,8)								
**Quartz**	**(-4,-1,-8)**	**11.348**	**78.91**	**2.576**	**2.5**	**2.266**	**5.57**	**28.33**	**28.87**

Ei =	11.564	keV						ΔEg	ΔEt
Cryst	Refl	EB	ΘB	∫ IR dΘ	Width	Ei cotΘ	ΔE	2m, 50µm	2m, 50µm
	(h,k,l)	[keV]	[°]	[µrad]	[µrad]	[meV/µrad]	[meV]	[meV]	[meV]
**Sapph**	**(2,6,5)**	**11.108**	**73.85**	**2.532**	**2.5**	**3.348**	**8.22**	**41.85**	**42.65**
	Equiv. Refl.: (2,-8,-5), (6,2,-5), (6,-8,5), (8,-2,-5), (8,-6,5), (-2,8,5), (-2,-6,-5), (-6,8,-5), (-6,-2,5) (-8,2,5), (-8,6,-5)								
**Quartz**	**(1,4,-8)**	**11.348**	**78.91**	**2.517**	**2.5**	**2.266**	**5.57**	**28.33**	**28.87**
	Equiv. Refl.: (1,-5,8)								
**Quartz**	**(4,-5,-8)**	**11.348**	**78.91**	**2.516**	**2.5**	**2.266**	**5.57**	**28.33**	**28.87**
	Equiv. Refl.: (-5,1,-8), (-5,4,8)								
**Sapph**	**(3,4,14)**	**11.331**	**78.48**	**2.515**	**2.6**	**2.357**	**6.1**	**29.46**	**30.09**
	Equiv. Refl.: (3,-7,-14), (4,3,-14), (4,-7,14), (7,-3,-14), (7,-4,14), (-3,7,14), (-3,-4,-14), (-4,7,-14), (-4,-3,14) (-7,3,14), (-7,4,-14), (4,1,8)								
**Quartz**	**(-2,8,3)**	**11.056**	**72.95**	**2.459**	**2.2**	**3.547**	**7.66**	**44.34**	**44.99**
	Equiv. Refl.: (-2,-6,-3), (-6,8,-3)								
**Quartz**	**(8,-2,-3)**	**11.056**	**72.95**	**2.458**	**2.2**	**3.547**	**7.66**	**44.34**	**44.99**
	Equiv. Refl.: (8,-6,3), (-6,-2,3)								
**Quartz**	**(2,6,3)**	**11.056**	**72.95**	**1.912**	**2.2**	**3.547**	**7.66**	**44.34**	**44.99**
	Equiv. Refl.: (2,-8,-3), (6,2,-3), (6,-8,3)								
**Quartz**	**(-8,2,3)**	**11.056**	**72.95**	**1.911**	**2.2**	**3.547**	**7.66**	**44.34**	**44.99**
	Equiv. Refl.: (-8,6,-3)								
**Sapph**	**(2,4,-17)**	**11.367**	**79.41**	**1.895**	**2.3**	**2.162**	**4.98**	**27.03**	**27.48**
	Equiv. Refl.: (2,-6,17), (4,2,17), (4,-6,-17), (6,-2,17), (6,-4,-17), (-2,6,-17), (-2,-4,17), (-4,6,17), (-4,-2,-17) (-6,2,-17), (-6,4,17)								
**Quartz**	**(8,-1,1)**	**11.06**	**73.02**	**1.891**	**2**	**3.532**	**7.03**	**44.15**	**44.71**
	Equiv. Refl.: (8,-7,-1), (-1,8,-1), (-1,-7,1), (-7,8,1), (-7,-1,-1)								
**LiNbO3**	**(2,4,19)**	**11.239**	**76.39**	**1.818**	**3**	**2.799**	**8.38**	**34.99**	**35.98**
	Equiv. Refl.: (2,-6,-19), (4,2,-19), (4,-6,19), (6,-2,-19), (6,-4,19), (-2,6,19), (-2,-4,-19), (-4,6,-19), (-4,2,19) (-6,2,19), (-6,4,-19)								
**Quartz**	**(1,7,-1)**	**11.06**	**73.02**	**1.758**	**2**	**3.532**	**7.03**	**44.15**	**44.71**
	Equiv. Refl.: (1,-8,1), (7,1,1), (7,-8,-1), (-8,1,-1), (-8,7,1)								
**LiNbO3**	**(1,3,-23)**	**11.442**	**81.67**	**1.695**	**3.4**	**1.694**	**5.73**	**21.17**	**21.93**
	Equiv. Refl.: (1,-4,23), (3,1,23), (3,-4,-23), (4,-1,23), (4,-3,-23), (-1,4,-23), (-1,-3,23), (-3,4,23), (-3,-1,-23) (-4,1,-23), (-4,3,23)								
**LiNbO3**	**(1,5,-19)**	**11.495**	**83.72**	**1.478**	**3.5**	**1.273**	**4.44**	**15.91**	**16.52**
	Equiv. Refl.: (1,-6,19), (5,1,19), (5,-6,-19), (6,-1,19), (6,-5,-19), (-1,6,-19), (-1,-5,19), (-5,6,19), (-5,-1,-19) (-6,1,-19), (-6,5,19)								
**LiNbO3**	**(3,4,17)**	**11.372**	**79.55**	**1.34**	**2.7**	**2.132**	**5.75**	**26.66**	**27.27**
	Equiv. Refl.: (3,-7,-17), (4,3,-17), (4,-7,17), (7,-3,-17), (7,-4,17), (-3,7,17), (-3,-4,-17), (-4,7,-17), (-4,-3,17) (-7,3,17), (-7,4,-17)								
**Quartz**	**(3,4,-6)**	**11.221**	**76.01**	**1.189**	**1.3**	**2.882**	**3.82**	**36.02**	**36.22**
**Quartz**	**(3,-7,6)**	**11.221**	**76.01**	**1.188**	**1.3**	**2.882**	**3.81**	**36.02**	**36.22**
	Equiv. Refl.: (4,3,6), (4,-7,-6)								
**Quartz**	**(-7,3,-6)**	**11.221**	**76.01**	**1.186**	**1.3**	**2.882**	**3.81**	**36.02**	**36.22**
	Equiv. Refl.: (-7,4,6)								
**Quartz**	**(-1,7,-5)**	**11.143**	**74.5**	**1.115**	**1.3**	**3.208**	**4.19**	**40.1**	**40.31**
	Equiv. Refl.: (-1,-6,5), (-6,7,5), (-6,-1,-5)								
**Quartz**	**(7,-1,5)**	**11.143**	**74.5**	**1.114**	**1.3**	**3.208**	**4.19**	**40.1**	**40.31**
	Equiv. Refl.: (7,-6,-5)								
**LiNbO3**	**(1,4,21)**	**11.348**	**78.92**	**1.08**	**2.3**	**2.264**	**5.24**	**28.3**	**28.79**
	Equiv. Refl.: (1,4,-21), (1,-5,21), (1,-5,-21), (4,1,21), (4,1,-21), (4,-5,21), (4,-5,-21), (5,-1,21), (5,-1,-21) (5,-4,21), (5,-4,-21), (-1,5,21), (-1,5,-21), (-1,-4,21), (-1,-4,-21), (-4,5,21), (-4,5,-21), (-4,-1,21) (-4,-1,-21), (-5,1,21), (-5,1,-21), (-5,4,21), (-5,4,-21)								

Ei =	11.564	keV						ΔEg	ΔEt
Cryst	Refl	EB	ΘB	∫ IR dΘ	Width	Ei cotΘ	ΔE	2m, 50µm	2m, 50µm
	(h,k,l)	[keV]	[°]	[µrad]	[µrad]	[meV/µrad]	[meV]	[meV]	[meV]
**Sapph**	**(2,3,-19)**	**11.191**	**75.41**	**1.078**	**1.4**	**3.01**	**4.16**	**37.63**	**37.86**
	Equiv. Refl.: (2,-5,19), (3,2,19), (3,-5,-19), (5,-2,19), (5,-3,-19), (-2,5,-19), (-2,-3,19), (-3,5,19), (-3,-2,-19) (-5,2,-19), (-5,3,19), (8,-1,2), (8,-7,-2), (-1,8,-2), (-1,-7,2)								
**Quartz**	**(-7,8,2)**	**11.237**	**76.34**	**1.077**	**1.3**	**2.812**	**3.62**	**35.14**	**35.33**
	Equiv. Refl.: (-7,-1,-2)								
**Quartz**	**(6,1,5)**	**11.143**	**74.5**	**1.074**	**1.3**	**3.208**	**4.19**	**40.1**	**40.31**
	Equiv. Refl.: (6,-7,-5)								
**Quartz**	**(1,6,-5)**	**11.143**	**74.5**	**1.073**	**1.3**	**3.208**	**4.19**	**40.1**	**40.31**
	Equiv. Refl.: (1,-7,5)								
**Quartz**	**(-7,1,-5)**	**11.143**	**74.5**	**1.072**	**1.3**	**3.208**	**4.19**	**40.1**	**40.31**
	Equiv. Refl.: (-7,6,5)								
**Quartz**	**(-4,6,-7)**	**11.131**	**74.28**	**1.055**	**1.2**	**3.256**	**4.01**	**40.69**	**40.89**
	Equiv. Refl.: (-4,-2,7)								
**Quartz**	**(6,-2,-7)**	**11.131**	**74.28**	**1.054**	**1.2**	**3.256**	**4.01**	**40.69**	**40.89**
**Quartz**	**(6,-4,7)**	**11.131**	**74.28**	**1.053**	**1.2**	**3.256**	**4**	**40.69**	**40.89**
**Quartz**	**(-2,6,7)**	**11.131**	**74.28**	**1.052**	**1.2**	**3.256**	**4**	**40.69**	**40.89**
	Equiv. Refl.: (-2,-4,-7)								
**Sapph**	**(3,5,7)**	**11.048**	**72.82**	**1.022**	**1.3**	**3.575**	**4.59**	**44.68**	**44.92**
	Equiv. Refl.: (3,-8,-7), (5,3,-7), (5,-8,7), (8,-3,-7), (8,-5,7), (-3,8,7), (-3,-5,-7), (-5,8,-7), (-5,-3,7) (-8,3,7), (-8,5,-7)								
**Quartz**	**(1,7,-2)**	**11.237**	**76.34**	**1.013**	**1.3**	**2.812**	**3.62**	**35.14**	**35.33**
	Equiv. Refl.: (1,-8,2), (7,1,2), (7,-8,-2), (-8,1,-2), (-8,7,2)								
**Quartz**	**(8,-2,-4)**	**11.465**	**82.48**	**1.008**	**1.3**	**1.526**	**1.99**	**19.08**	**19.18**
	Equiv. Refl.: (8,-6,4)								
**LiNbO3**	**(4,5,-7)**	**11.302**	**77.78**	**1.007**	**2.1**	**2.503**	**5.35**	**31.29**	**31.75**
	Equiv. Refl.: (4,-9,7), (5,4,7), (5,-9,-7), (9,-4,7), (9,-5,-7), (-4,9,-7), (-4,-5,7), (-5,9,7), (-5,-4,-7) (-9,4,-7), (-9,5,7), (1,2,-9), (1,-3,9), (2,-3,-9), (-6,8,-4), (-6,-2,4)								
**Quartz**	**(2,1,9)**	**11.019**	**72.35**	**1.006**	**1.1**	**3.68**	**4.14**	**46**	**46.19**
**Quartz**	**(-2,-6,-4)**	**11.465**	**82.48**	**1.005**	**1.3**	**1.526**	**1.99**	**19.08**	**19.18**
**Quartz**	**(-2,8,4)**	**11.465**	**82.48**	**1.004**	**1.3**	**1.526**	**1.99**	**19.08**	**19.18**
	Equiv. Refl.: (-3,1,-9), (-3,2,9)								
**Quartz**	**(-3,-4,6)**	**11.221**	**76.01**	**0.992**	**1.3**	**2.882**	**3.82**	**36.02**	**36.22**
	Equiv. Refl.: (-4,7,6)								
**Quartz**	**(-3,7,-6)**	**11.221**	**76.01**	**0.991**	**1.3**	**2.882**	**3.81**	**36.02**	**36.22**
	Equiv. Refl.: (-4,-3,-6)								
**Quartz**	**(7,-3,6)**	**11.221**	**76.01**	**0.989**	**1.3**	**2.882**	**3.81**	**36.02**	**36.22**
	Equiv. Refl.: (7,-4,-6)								
**Quartz**	**(4,-6,7)**	**11.131**	**74.28**	**0.985**	**1.2**	**3.256**	**4.01**	**40.69**	**40.89**
**Quartz**	**(4,2,-7)**	**11.131**	**74.28**	**0.984**	**1.2**	**3.256**	**4.01**	**40.69**	**40.89**
	Equiv. Refl.: (-6,2,7), (-6,4,-7)								
**Quartz**	**(2,4,7)**	**11.131**	**74.28**	**0.982**	**1.2**	**3.256**	**4**	**40.69**	**40.89**
	Equiv. Refl.: (2,-6,-7)								
**Quartz**	**(-1,3,-9)**	**11.019**	**72.35**	**0.868**	**1.1**	**3.68**	**4.15**	**46**	**46.19**
	Equiv. Refl.: (-1,-2,9), (-2,3,9)								
**Quartz**	**(-2,-1,-9)**	**11.019**	**72.35**	**0.866**	**1.1**	**3.68**	**4.14**	**46**	**46.19**
**Quartz**	**(3,-1,9)**	**11.019**	**72.35**	**0.865**	**1.1**	**3.68**	**4.14**	**46**	**46.19**
	Equiv. Refl.: (3,-2,-9)								
**Quartz**	**(0,7,-4)**	**11.183**	**75.26**	**0.812**	**1**	**3.043**	**3.17**	**38.03**	**38.16**
	Equiv. Refl.: (0,-7,4)								
**Quartz**	**(7,-7,-4)**	**11.183**	**75.26**	**0.81**	**1**	**3.043**	**3.16**	**38.03**	**38.16**
	Equiv. Refl.: (-7,7,4)								

Ei =	11.564	keV						ΔEg	ΔEt
Cryst	Refl	EB	ΘB	∫ IR dΘ	Width	Ei cotΘ	ΔE	2m, 50µm	2m, 50µm
	(h,k,l)	[keV]	[°]	[µrad]	[µrad]	[meV/µrad]	[meV]	[meV]	[meV]
**Quartz**	**(7,0,4)**	**11.183**	**75.26**	**0.809**	**1**	**3.043**	**3.16**	**38.03**	**38.16**
	Equiv. Refl.: (-7,0,-4)								
**LiNbO3**	**(4,5,5)**	**11.088**	**73.5**	**0.795**	**1.7**	**3.425**	**5.69**	**42.82**	**43.19**
	Equiv. Refl.: (4,-9,-5), (5,4,-5), (5,-9,5), (9,-4,-5), (9,-5,5), (-4,9,5), (-4,-5,-5), (-5,9,-5), (-5,-4,5) (-9,4,5), (-9,5,-5)								
**Quartz**	**(8,-3,-4)**	**11.183**	**75.26**	**0.782**	**1**	**3.043**	**2.94**	**38.03**	**38.15**
	Equiv. Refl.: (8,-5,4), (-3,8,4), (-3,-5,-4), (-5,8,-4)								
**Quartz**	**(-5,-3,4)**	**11.183**	**75.26**	**0.781**	**1**	**3.043**	**2.94**	**38.03**	**38.15**
**Quartz**	**(-8,2,4)**	**11.465**	**82.48**	**0.769**	**1.3**	**1.526**	**1.99**	**19.08**	**19.18**
	Equiv. Refl.: (-8,6,-4)								
**Quartz**	**(6,2,-4)**	**11.465**	**82.48**	**0.768**	**1.3**	**1.526**	**1.99**	**19.08**	**19.18**
	Equiv. Refl.: (6,-8,4)								
**Quartz**	**(2,6,4)**	**11.465**	**82.48**	**0.766**	**1.3**	**1.526**	**1.99**	**19.08**	**19.18**
**Quartz**	**(2,-8,-4)**	**11.465**	**82.48**	**0.765**	**1.3**	**1.526**	**1.99**	**19.08**	**19.18**
**Quartz**	**(7,-2,6)**	**11.408**	**80.59**	**0.763**	**1.1**	**1.916**	**2.01**	**23.95**	**24.03**
	Equiv. Refl.: (7,-5,-6)								
**Quartz**	**(-2,7,-6)**	**11.408**	**80.59**	**0.761**	**1**	**1.916**	**2.01**	**23.95**	**24.03**
	Equiv. Refl.: (-2,-5,6), (-5,-2,-6)								
**Quartz**	**(-5,7,6)**	**11.408**	**80.59**	**0.76**	**1**	**1.916**	**2.01**	**23.95**	**24.03**
**Sapph**	**(1,6,-11)**	**11.175**	**75.1**	**0.715**	**1**	**3.077**	**3.11**	**38.46**	**38.58**
	Equiv. Refl.: (1,-7,11), (6,1,11), (6,-7,-11), (7,-1,11), (7,-6,-11), (-1,7,-11), (-1,-6,11), (-6,7,11), (-6,-1,-11) (-7,1,-11), (-7,6,11)								
**Quartz**	**(-1,6,7)**	**11.414**	**80.75**	**0.704**	**0.9**	**1.882**	**1.77**	**23.53**	**23.6**
	Equiv. Refl.: (-1,-5,-7)								
**LiNbO3**	**(4,5,-1)**	**10.869**	**70.04**	**0.703**	**1.4**	**4.2**	**6.07**	**52.5**	**52.85**
	Equiv. Refl.: (4,-9,1), (5,4,1), (5,-9,-1), (9,-4,1), (9,-5,-1), (-4,9,-1), (-4,-5,1), (-5,9,1), (-5,-4,-1) (-9,4,-1), (-9,5,1), (-5,6,-7)								
**Quartz**	**(-5,-1,7)**	**11.414**	**80.75**	**0.702**	**0.9**	**1.882**	**1.77**	**23.53**	**23.6**
**Quartz**	**(6,-1,-7)**	**11.414**	**80.75**	**0.701**	**0.9**	**1.882**	**1.77**	**23.53**	**23.6**
	Equiv. Refl.: (6,-5,7)								
**Quartz**	**(3,5,4)**	**11.183**	**75.26**	**0.692**	**1**	**3.043**	**2.94**	**38.03**	**38.15**
	Equiv. Refl.: (3,-8,-4), (-8,3,4), (-8,5,-4)								
**Quartz**	**(5,3,-4)**	**11.183**	**75.26**	**0.691**	**1**	**3.043**	**2.94**	**38.03**	**38.15**
	Equiv. Refl.: (5,-8,4)								
**Quartz**	**(-7,2,-6)**	**11.408**	**80.59**	**0.656**	**1.1**	**1.916**	**2.01**	**23.95**	**24.03**
	Equiv. Refl.: (-7,5,6)								
**Quartz**	**(5,2,6)**	**11.408**	**80.59**	**0.654**	**1**	**1.916**	**2.01**	**23.95**	**24.03**
**Quartz**	**(2,5,-6)**	**11.408**	**80.59**	**0.653**	**1**	**1.916**	**2.01**	**23.95**	**24.03**
	Equiv. Refl.: (2,-7,6), (5,-7,-6)								
**Quartz**	**(0,6,6)**	**11.126**	**74.18**	**0.585**	**0.8**	**3.277**	**2.61**	**40.96**	**41.05**
	Equiv. Refl.: (0,-6,-6)								
**Quartz**	**(6,0,-6)**	**11.126**	**74.18**	**0.582**	**0.8**	**3.277**	**2.6**	**40.96**	**41.05**
	Equiv. Refl.: (6,-6,6), (-6,0,6), (-6,6,-6)								
**Sapph**	**(1,4,18)**	**11.015**	**72.28**	**0.578**	**0.8**	**3.695**	**3.06**	**46.19**	**46.29**
	Equiv. Refl.: (1,4,-18), (1,-5,18), (1,-5,-18), (4,1,18), (4,1,-18), (4,-5,18), (4,-5,-18), (5,-1,18), (5,-1,-18) (5,-4,18), (5,-4,-18), (-1,5,18), (-1,5,-18), (-1,-4,18), (-1,-4,-18), (-4,5,18), (-4,5,-18), (-4,1,18) (-4,-1,-18), (-5,1,18), (-5,1,-18), (-5,4,18), (-5,4,-18)								
**Quartz**	**(1,-3,-9)**	**11.019**	**72.35**	**0.545**	**0.7**	**3.68**	**2.69**	**46**	**46.08**
**Quartz**	**(1,2,9)**	**11.019**	**72.35**	**0.544**	**0.7**	**3.68**	**2.69**	**46**	**46.08**

Ei = Cryst	11.564 Refl (h,k,l)	keV EB [keV]	ΘB [°]	∫ IR dΘ [µrad]	Width [µrad]	Ei cotΘ [meV/µrad]	ΔE [meV]	ΔEg 2m, 50µm [meV]	ΔEt 2m, 50µm [meV]
**Quartz**	**(-3,1,9)**	**11.019**	**72.35**	**0.543**	**0.7**	**3.68**	**2.68**	**46**	**46.08**
	Equiv. Refl.: (-3,2,-9)								
**Quartz**	**(2,1,-9)**	**11.019**	**72.35**	**0.542**	**0.7**	**3.68**	**2.68**	**46**	**46.08**
	Equiv. Refl.: (2,-3,9)								
**Quartz**	**(-1,3,9)**	**11.019**	**72.35**	**0.528**	**0.7**	**3.68**	**2.69**	**46**	**46.08**
**Sapph**	**(2,5,12)**	**11.003**	**72.08**	**0.527**	**0.8**	**3.739**	**2.9**	**46.73**	**46.82**
	Equiv. Refl.: (2,5,-12), (2,-7,12), (2,-7,-12), (5,2,12), (5,2,-12), (5,-7,12), (5,-7,-12), (7,-2,12), (7,-2,-12) (7,-5,12), (7,-5,-12), (-2,7,12), (-2,7,-12), (-2,-5,12), (-2,-5,-12), (-5,7,12), (-5,7,-12), (-5,-2,12) (-5,-2,-12), (-7,2,12), (-7,2,-12), (-7,5,12), (-7,5,-12), (-1,-2,-9)								
**Quartz**	**(3,-1,-9)**	**11.019**	**72.35**	**0.526**	**0.7**	**3.68**	**2.68**	**46**	**46.08**
	Equiv. Refl.: (3,-2,9), (-2,3,-9)								
**Quartz**	**(-2,-1,9)**	**11.019**	**72.35**	**0.525**	**0.7**	**3.68**	**2.68**	**46**	**46.08**
**LiNbO3**	**(1,2,23)**	**10.923**	**70.84**	**0.523**	**1.2**	**4.019**	**4.94**	**50.23**	**50.48**
	Equiv. Refl.: (1,-3,-23), (2,1,-23), (2,-3,23), (3,-1,-23), (3,-2,23), (-1,3,23), (-1,-2,-23), (-2,3,-23), (-2,-1,23) (-3,1,23), (-3,2,-23)								
**Quartz**	**(4,5,0)**	**11.379**	**79.75**	**0.519**	**0.8**	**2.091**	**1.7**	**26.14**	**26.2**
	Equiv. Refl.: (4,-9,0), (5,4,0), (5,-9,0), (-9,4,0), (-9,5,0)								
**Quartz**	**(1,5,7)**	**11.414**	**80.75**	**0.513**	**0.9**	**1.882**	**1.77**	**23.53**	**23.6**
	Equiv. Refl.: (1,-6,-7)								
**Quartz**	**(5,-6,7)**	**11.414**	**80.75**	**0.512**	**0.9**	**1.882**	**1.77**	**23.53**	**23.6**
**Quartz**	**(5,1,-7)**	**11.414**	**80.75**	**0.511**	**0.9**	**1.882**	**1.77**	**23.53**	**23.6**
**Quartz**	**(-6,1,7)**	**11.414**	**80.75**	**0.51**	**0.9**	**1.882**	**1.77**	**23.53**	**23.6**
	Equiv. Refl.: (-6,5,-7)								
**Quartz**	**(9,-4,0)**	**11.379**	**79.75**	**0.501**	**0.8**	**2.091**	**1.7**	**26.14**	**26.2**
	Equiv. Refl.: (9,-5,0), (-4,9,0), (-4,-5,0), (-5,9,0), (-5,-4,0)								
**LiNbO3**	**(3,6,3)**	**11.118**	**74.03**	**0.446**	**1.2**	**3.309**	**3.99**	**41.36**	**41.55**
	Equiv. Refl.: (3,6,-3), (3,-9,3), (3,-9,-3), (6,3,3), (6,3,-3), (6,-9,3), (6,-9,-3), (9,-3,3), (9,-3,-3) (9,-6,3), (9,-6,-3), (-3,9,3), (-3,9,-3), (-3,-6,3), (-3,-6,-3), (-6,9,3), (-6,9,-3), (-6,-3,3) (-6,-3,-3), (-9,3,3), (-9,3,-3), (-9,6,3), (-9,6,-3)								
**Quartz**	**(0,3,9)**	**11.21**	**75.79**	**0.443**	**0.7**	**2.927**	**1.97**	**36.59**	**36.64**
	Equiv. Refl.: (0,-3,-9)								
**Quartz**	**(3,0,-9)**	**11.21**	**75.79**	**0.44**	**0.7**	**2.927**	**1.96**	**36.59**	**36.64**
	Equiv. Refl.: (3,-3,9), (-3,3,-9)								
**Quartz**	**(-3,0,9)**	**11.21**	**75.79**	**0.439**	**0.7**	**2.927**	**1.96**	**36.59**	**36.64**
**Quartz**	**(3,2,-8)**	**11.16**	**74.8**	**0.429**	**0.6**	**3.142**	**1.94**	**39.27**	**39.32**
	Equiv. Refl.: (3,-5,8)								
**Quartz**	**(2,3,8)**	**11.16**	**74.8**	**0.428**	**0.6**	**3.142**	**1.94**	**39.27**	**39.32**
	Equiv. Refl.: (2,-5,-8)								
**Quartz**	**(-5,3,-8)**	**11.16**	**74.8**	**0.424**	**0.6**	**3.142**	**1.93**	**39.27**	**39.32**
**Quartz**	**(-5,2,8)**	**11.16**	**74.8**	**0.423**	**0.6**	**3.142**	**1.92**	**39.27**	**39.32**
**Quartz**	**(4,-4,-8)**	**10.87**	**70.06**	**0.42**	**0.6**	**4.196**	**2.49**	**52.45**	**52.51**
	Equiv. Refl.: (-4,0,-8), (-4,4,8)								
**Quartz**	**(4,0,8)**	**10.87**	**70.06**	**0.419**	**0.6**	**4.196**	**2.49**	**52.45**	**52.51**
**Quartz**	**(0,4,-8)**	**10.87**	**70.06**	**0.415**	**0.6**	**4.196**	**2.47**	**52.45**	**52.51**
	Equiv. Refl.: (0,-4,8)								
**Quartz**	**(1,7,2)**	**11.237**	**76.34**	**0.388**	**0.6**	**2.812**	**1.71**	**35.14**	**35.19**
	Equiv. Refl.: (1,-8,-2)								
**Quartz**	**(7,1,-2)**	**11.237**	**76.34**	**0.387**	**0.6**	**2.812**	**1.71**	**35.14**	**35.19**
	Equiv. Refl.: (7,-8,2), (-8,1,2), (-8,7,-2)								
**Quartz**	**(-3,5,-8)**	**11.16**	**74.8**	**0.379**	**0.6**	**3.142**	**1.94**	**39.27**	**39.32**
	Equiv. Refl.: (-3,-2,8)								

Ei =	11.564	keV						ΔEg	ΔEt
**Cryst**	**Refl**	**EB**	**ΘB**	**∫ IR dΘ**	**Width**	**Ei cotΘ**	**ΔE**	**2m, 50μm**	**2m, 50μm**
	**(h,k,l)**	**[keV]**	**[°]**	**[μrad]**	**[μrad]**	**[meV/μrad]**	**[meV]**	**[meV]**	**[meV]**
**Quartz**	**(-2,5,8)**	**11.16**	**74.8**	**0.378**	**0.6**	**3.142**	**1.94**	**39.27**	**39.32**
	Equiv. Refl.: (-2,-3,-8)								
**Quartz**	**(5,-3,8)**	**11.16**	**74.8**	**0.374**	**0.6**	**3.142**	**1.93**	**39.27**	**39.32**
**Quartz**	**(5,-2,-8)**	**11.16**	**74.8**	**0.373**	**0.6**	**3.142**	**1.92**	**39.27**	**39.32**
	Equiv. Refl.: (-1,8,2), (-1,-7,-2)								
**Quartz**	**(8,-1,-2)**	**11.237**	**76.34**	**0.372**	**0.6**	**2.812**	**1.71**	**35.14**	**35.19**
	Equiv. Refl.: (8,-7,2), (-7,8,-2), (-7,-1,2)								
**LiNbO3**	**(2,6,11)**	**11.169**	**74.97**	**0.371**	**1.1**	**3.105**	**3.46**	**38.81**	**38.96**
	Equiv. Refl.: (2,-8,-11), (6,2,-11), (6,-8,11), (8,-2,-11), (8,-6,11), (-2,8,11), (-2,-6,-11), (-6,8,-11), (-6,-2,11) (-8,2,11), (-8,6,-11)								
**Sapph**	**(0,2,22)**	**10.923**	**70.83**	**0.358**	**0.6**	**4.019**	**2.34**	**50.24**	**50.3**
	Equiv. Refl.: (0,-2,-22), (2,0,-22), (2,-2,22), (-2,0,22), (-2,2,-22)								
**LiNbO3**	**(2,5,15)**	**10.973**	**71.6**	**0.34**	**1**	**3.848**	**3.78**	**48.1**	**48.24**
	Equiv. Refl.: (2,5,-15), (2,-7,15), (2,-7,-15), (5,2,15), (5,2,-15), (5,-7,15), (5,-7,-15), (7,-2,15), (7,-2,-15) (7,-5,15), (7,-5,-15), (-2,7,15), (-2,7,-15), (-2,-5,15), (-2,-5,-15), (-5,7,15), (-5,7,-15), (-5,-2,15) (-5,-2,-15), (-7,2,15), (-7,2,-15), (-7,5,15), (-7,5,-15)								
**Sapph**	**(0,0,24)**	**11.455**	**82.13**	**0.297**	**0.6**	**1.599**	**1.03**	**19.99**	**20.02**
	Equiv. Refl.: (0,0,-24)								
**Quartz**	**(3,3,-7)**	**11.036**	**72.61**	**0.275**	**0.4**	**3.621**	**1.45**	**45.27**	**45.29**
**Quartz**	**(3,-6,7)**	**11.036**	**72.61**	**0.274**	**0.4**	**3.621**	**1.45**	**45.27**	**45.29**
**Quartz**	**(-6,3,7)**	**11.036**	**72.61**	**0.273**	**0.4**	**3.621**	**1.45**	**45.27**	**45.29**
	Equiv. Refl.: (-6,3,-7)								
**Quartz**	**(3,3,7)**	**11.036**	**72.61**	**0.272**	**0.4**	**3.621**	**1.44**	**45.27**	**45.29**
	Equiv. Refl.: (3,-6,-7)								
**LiNbO3**	**(1,7,9)**	**11.243**	**76.47**	**0.266**	**1**	**2.783**	**2.73**	**34.79**	**34.9**
	Equiv. Refl.: (1,7,-9), (1,-8,9), (1,-8,-9), (7,1,9), (7,1,-9), (7,-8,9), (7,-8,-9), (8,-1,9), (8,-1,-9) (8,-7,9), (8,-7,-9), (-1,8,9), (-1,8,-9), (-1,-7,9), (-1,-7,-9), (-7,8,9), (-7,8,-9), (-7,-1,9) (-7,-1,-9), (-8,1,9), (-8,1,-9), (-8,7,9), (-8,7,-9)								
**Sapph**	**(3,5,-8)**	**11.202**	**75.62**	**0.26**	**0.5**	**2.964**	**1.53**	**37.05**	**37.09**
	Equiv. Refl.: (3,-8,8), (5,3,8), (5,-8,-8), (8,-3,8), (8,-5,-8), (-3,8,-8), (-3,-5,8), (-5,8,8), (-5,-3,-8) (-8,3,-8), (-8,5,8)								
**LiNbO3**	**(3,5,13)**	**11.337**	**78.64**	**0.251**	**1**	**2.324**	**2.39**	**29.05**	**29.15**
	Equiv. Refl.: (3,-8,-13), (5,3,-13), (5,-8,13), (8,-3,-13), (8,-5,13), (-3,8,13), (-3,-5,-13), (-5,8,-13), (-5,-3,13) (-8,3,13), (-8,5,-13)								
**Sapph**	**(2,6,8)**	**11.501**	**84.01**	**0.248**	**0.7**	**1.213**	**0.85**	**15.17**	**15.19**
	Equiv. Refl.: (2,-8,-8), (6,2,-8), (6,-8,8), (8,-2,-8), (8,-6,8), (-2,8,8), (-2,-6,-8), (-6,8,-8), (-6,-2,8) (-8,2,8), (-8,6,-8)								
**Quartz**	**(-3,6,-7)**	**11.036**	**72.61**	**0.184**	**0.4**	**3.621**	**1.45**	**45.27**	**45.29**
	Equiv. Refl.: (-3,-3,7)								
**Quartz**	**(6,-3,7)**	**11.036**	**72.61**	**0.183**	**0.4**	**3.621**	**1.45**	**45.27**	**45.29**
	Equiv. Refl.: (6,-3,-7)								
**Quartz**	**(-3,6,7)**	**11.036**	**72.61**	**0.182**	**0.4**	**3.621**	**1.44**	**45.27**	**45.29**
	Equiv. Refl.: (-3,-3,-7)								
**LiNbO3**	**(3,5,-11)**	**10.906**	**70.57**	**0.17**	**0.7**	**4.078**	**2.71**	**50.98**	**51.05**
	Equiv. Refl.: (3,-8,11), (5,3,11), (5,-8,-11), (8,-3,11), (8,-5,-11), (-3,8,-11), (-3,-5,11), (-5,8,11), (-5,-3,-11) (-8,3,-11), (-8,5,11)								
**Sapph**	**(3,4,-13)**	**11.056**	**72.96**	**0.165**	**0.4**	**3.545**	**1.32**	**44.31**	**44.33**
	Equiv. Refl.: (3,-7,13), (4,3,13), (4,-7,-13), (7,-3,13), (7,-4,-13), (-3,7,-13), (-3,-4,13), (-4,7,13), (-4,-3,-13) (-7,3,-13), (-7,4,13)								
**Sapph**	**(1,2,-22)**	**11.229**	**76.19**	**0.1**	**0.3**	**2.843**	**0.87**	**35.54**	**35.55**
	Equiv. Refl.: (1,-3,22), (2,1,22), (2,-3,-22), (3,-1,22), (3,-2,-22), (-1,3,-22), (-1,-2,22), (-2,3,22), (-2,-1,-22) (-3,1,-22), (-3,2,22)								

**Sapph**	**(2,6,2)**	**10.89**	**70.35**	**0.098**	**0.3**	**4.13**	**1.08**	**51.62**	**51.63**
	Equiv. Refl.: (2,-8,-2), (6,2,-2), (6,-8,2), (8,-2,-2), (8,-6,2), (-2,8,2), (-2,-6,-2), (-6,8,-2), (-6,-2,2) (-8,2,2), (-8,6,-2)								

Ei =<br>Cryst	14.353<br>Refl<br>(h,k,l)	keV<br>EB<br>[keV]	ΘB<br>[°]	∫ IR dΘ<br>[µrad]	Width<br>[µrad]	Ei cotΘ<br>[meV/µrad]	ΔE<br>[meV]	ΔEg<br>2m, 50µm<br>[meV]	ΔEt<br>2m, 50µm<br>[meV]
**Ge**	**(1,1,13)**	**14.329**	**86.7**	**3.8711**	**11.73**	**0.8279**	**9.71**	**10.35**	**14.19**
	Equiv. Refl.: (1,7,11), (1,11,7), (1,13,1), (3,9,9), (5,5,11), (5,11,5), (7,1,11), (7,11,1), (9,3,9) (9,9,3), (11,1,7), (11,5,5), (11,7,1), (13,1,1)								
**Ge**	**(2,8,10)**	**14.203**	**81.71**	**3.0301**	**6.83**	**2.092**	**14.28**	**26.15**	**29.79**
	Equiv. Refl.: (2,10,8), (8,2,10), (8,10,2), (10,2,8), (10,8,2)								
**Si**	**(2,2,12)**	**14.073**	**78.66**	**2.8876**	**2.33**	**2.8791**	**6.7**	**35.99**	**36.61**
	Equiv. Refl.: (2,12,2), (4,6,10), (4,10,6), (6,4,10), (6,10,4), (10,4,6), (10,6,4), (12,2,2)								
**Si**	**(3,5,11)**	**14.211**	**81.93**	**1.9823**	**2.23**	**2.035**	**4.53**	**25.44**	**25.84**
	Equiv. Refl.: (3,11,5), (5,3,11), (5,7,9), (5,9,7), (5,11,3), (7,5,9), (7,9,5), (9,5,7), (9,7,5) (11,3,5), (11,5,3)								
**Ge**	**(0,4,12)**	**13.861**	**74.95**	**1.891**	**4.11**	**3.8595**	**15.88**	**48.24**	**50.79**
	Equiv. Refl.: (0,12,4), (4,0,12), (4,12,0), (12,0,4), (12,4,0)								
**Ge**	**(2,2,12)**	**13.51**	**70.26**	**1.6384**	**3.44**	**5.15**	**17.74**	**64.37**	**66.77**
	Equiv. Refl.: (2,12,2), (4,6,10), (4,10,6), (6,4,10), (6,10,4), (10,4,6), (10,6,4), (12,2,2)								
**Si**	**(0,0,12)**	**13.697**	**72.62**	**1.5958**	**1.69**	**4.4938**	**7.58**	**56.17**	**56.68**
	Equiv. Refl.: (0,12,0), (4,8,8), (8,4,8), (8,8,4), (12,0,0)								
**Si**	**(1,5,11)**	**13.839**	**74.62**	**1.1666**	**1.3**	**3.9469**	**5.11**	**49.34**	**49.6**
	Equiv. Refl.: (1,11,5), (5,1,11), (5,11,1), (7,7,7), (11,1,5), (11,5,1)								
**Ge**	**(1,9,9)**	**13.99**	**77.09**	**1.1309**	**3.28**	**3.2909**	**10.78**	**41.14**	**42.53**
	Equiv. Refl.: (9,1,9), (9,9,1)								
**Ge**	**(3,5,11)**	**13.642**	**71.89**	**0.9245**	**2.56**	**4.6927**	**12.03**	**58.66**	**59.88**
	Equiv. Refl.: (3,11,5), (5,3,11), (5,7,9), (5,9,7), (5,11,3), (7,5,9), (7,9,5), (9,5,7), (9,7,5) (11,3,5), (11,5,3)								
**Sapph**	**(0,0,30)**	**14.319**	**86.04**	**16.669**	**13.6**	**0.993**	**13.48**	**12.42**	**18.33**
	Equiv. Refl.: (0,0,-30)								
**LiNbO3**	**(0,5,28)**	**14.322**	**86.21**	**12.89**	**12.5**	**0.95**	**11.86**	**11.87**	**16.78**
	Equiv. Refl.: (5,-5,28), (-5,0,28)								
**LiNbO3**	**(0,-5,-28)**	**14.322**	**86.21**	**12.686**	**12.5**	**0.95**	**11.86**	**11.87**	**16.78**
	Equiv. Refl.: (5,0,-28), (-5,5,-28)								
**LiNbO3**	**(3,-10,16)**	**14.281**	**84.24**	**11.563**	**10.7**	**1.447**	**15.44**	**18.09**	**23.78**
	Equiv. Refl.: (7,3,16), (10,-3,16), (-3,-7,16)								
**LiNbO3**	**(-7,10,16)**	**14.281**	**84.24**	**11.562**	**10.7**	**1.447**	**15.44**	**18.09**	**23.78**
	Equiv. Refl.: (-10,7,16)								
**LiNbO3**	**(3,7,-16)**	**14.281**	**84.24**	**11.515**	**10.7**	**1.447**	**15.44**	**18.09**	**23.78**
	Equiv. Refl.: (-3,10,-16), (-7,-3,-16), (-10,3,-16)								
**LiNbO3**	**(7,-10,-16)**	**14.281**	**84.24**	**11.514**	**10.7**	**1.447**	**15.44**	**18.09**	**23.78**
	Equiv. Refl.: (10,-7,-16)								
**Quartz**	**(11,-3,0)**	**14.35**	**88.76**	**10.586**	**9.5**	**0.31**	**2.94**	**3.88**	**4.86**
	Equiv. Refl.: (11,-8,0), (-3,11,0), (-3,-8,0), (-8,11,0), (-8,-3,0)								
**LiNbO3**	**(0,8,-20)**	**14.273**	**83.96**	**10.283**	**9**	**1.519**	**13.63**	**18.98**	**23.37**
	Equiv. Refl.: (8,-8,-20), (-8,0,-20)								
**LiNbO3**	**(3,8,-8)**	**14.154**	**80.45**	**10.171**	**8.9**	**2.414**	**21.59**	**30.17**	**37.1**
	Equiv. Refl.: (8,-11,-8), (11,-8,-8), (-3,11,-8), (-8,-3,-8), (-11,3,-8)								
**LiNbO3**	**(2,9,-4)**	**14.225**	**82.34**	**10.104**	**9.1**	**1.931**	**17.6**	**24.14**	**29.88**
	Equiv. Refl.: (9,-11,-4), (11,-9,-4), (-2,11,-4), (-9,-2,-4), (-11,2,-4)								
**LiNbO3**	**(2,-11,4)**	**14.225**	**82.34**	**10.103**	**9.1**	**1.931**	**17.6**	**24.14**	**29.88**
	Equiv. Refl.: (9,2,4), (11,-2,4), (-2,-9,4), (-9,11,4), (-11,9,4)								

Ei =	14.353	keV						ΔEg	ΔEt
Cryst	Refl	EB	ΘB	∫ IR dΘ	Width	Ei cotΘ	ΔE	2m, 50µm	2m, 50µm
	(h,k,l)	[keV]	[°]	[µrad]	[µrad]	[meV/µrad]	[meV]	[meV]	[meV]
**LiNbO3**	**(3,-11,8)**	**14.154**	**80.45**	**9.729**	**8.9**	**2.414**	**21.59**	**30.17**	**37.1**
	Equiv. Refl.: (8,3,8), (11,-3,8), (-3,-8,8), (-8,11,8), (-11,8,8)								
**LiNbO3**	**(2,2,30)**	**14.254**	**83.26**	**9.607**	**8.8**	**1.696**	**14.98**	**21.2**	**25.96**
	Equiv. Refl.: (2,-4,30), (4,-2,30), (-2,4,30), (-2,-2,30), (-4,2,30)								
**LiNbO3**	**(2,2,-30)**	**14.254**	**83.26**	**9.377**	**8.8**	**1.696**	**14.98**	**21.2**	**25.96**
	Equiv. Refl.: (2,-4,-30), (4,-2,-30), (-2,4,-30), (-2,-2,-30), (-4,2,-30)								
**Quartz**	**(3,8,0)**	**14.35**	**88.76**	**9.25**	**9.5**	**0.31**	**2.94**	**3.88**	**4.86**
	Equiv. Refl.: (3,-11,0), (8,3,0), (8,-11,0), (-11,3,0), (-11,8,0)								
**LiNbO3**	**(0,3,30)**	**14.049**	**78.18**	**9.111**	**7.9**	**3.003**	**23.78**	**37.53**	**44.43**
	Equiv. Refl.: (3,-3,30), (-3,0,30)								
**LiNbO3**	**(0,-3,30)**	**14.049**	**78.18**	**9.052**	**7.9**	**3.003**	**23.68**	**37.53**	**44.38**
	Equiv. Refl.: (3,0,30), (-3,3,30)								
**LiNbO3**	**(0,-3,-30)**	**14.049**	**78.18**	**9.009**	**7.9**	**3.003**	**23.78**	**37.53**	**44.43**
	Equiv. Refl.: (3,0,-30), (-3,3,-30)								
**LiNbO3**	**(0,3,-30)**	**14.049**	**78.18**	**8.985**	**7.9**	**3.003**	**23.68**	**37.53**	**44.38**
	Equiv. Refl.: (-3,0,-30)								
**LiNbO3**	**(3,-3,-30)**	**14.049**	**78.18**	**8.984**	**7.9**	**3.003**	**23.68**	**37.53**	**44.38**
**Quartz**	**(-10,1,3)**	**14.318**	**86.02**	**8.764**	**7**	**0.998**	**7.03**	**12.48**	**14.32**
	Equiv. Refl.: (-10,9,-3)								
**Quartz**	**(1,9,3)**	**14.318**	**86.02**	**8.761**	**7**	**0.998**	**7.02**	**12.48**	**14.32**
	Equiv. Refl.: (1,-10,-3), (9,-10,3)								
**Quartz**	**(9,1,-3)**	**14.318**	**86.02**	**8.76**	**7**	**0.998**	**7.02**	**12.48**	**14.32**
**LiNbO3**	**(0,-8,20)**	**14.273**	**83.96**	**8.651**	**9**	**1.519**	**13.63**	**18.98**	**23.37**
	Equiv. Refl.: (8,0,20)								
**LiNbO3**	**(-8,8,20)**	**14.273**	**83.96**	**8.65**	**9**	**1.519**	**13.63**	**18.98**	**23.37**
**Quartz**	**(10,-1,-3)**	**14.318**	**86.02**	**8.57**	**7**	**0.998**	**7.03**	**12.48**	**14.32**
	Equiv. Refl.: (10,-9,3)								
**Quartz**	**(-1,10,3)**	**14.318**	**86.02**	**8.568**	**7**	**0.998**	**7.02**	**12.48**	**14.32**
**Quartz**	**(-1,-9,-3)**	**14.318**	**86.02**	**8.567**	**7**	**0.998**	**7.02**	**12.48**	**14.32**
	Equiv. Refl.: (-9,10,-3), (-9,-1,3)								
**LiNbO3**	**(2,-11,-2)**	**14.14**	**80.12**	**7.939**	**7.1**	**2.499**	**17.77**	**31.24**	**35.94**
	Equiv. Refl.: (9,2,-2), (11,-2,-2), (-2,-9,-2)								
**LiNbO3**	**(-9,11,-2)**	**14.14**	**80.12**	**7.938**	**7.1**	**2.499**	**17.77**	**31.24**	**35.94**
	Equiv. Refl.: (-11,9,-2)								
**LiNbO3**	**(2,-8,22)**	**14.047**	**78.15**	**7.88**	**6.8**	**3.011**	**20.58**	**37.64**	**42.9**
	Equiv. Refl.: (6,2,22), (8,-2,22), (-2,-6,22)								
**LiNbO3**	**(-6,8,22)**	**14.047**	**78.15**	**7.879**	**6.8**	**3.011**	**20.58**	**37.64**	**42.9**
	Equiv. Refl.: (-8,6,22)								
**Sapph**	**(3,7,-10)**	**14.198**	**81.57**	**7.859**	**6.2**	**2.127**	**13.11**	**26.59**	**29.65**
	Equiv. Refl.: (3,-10,10), (7,3,10), (7,-10,-10), (10,-3,10), (10,-7,-10), (-3,10,-10), (-3,-7,10), (-7,10,10), (-7,-3,-10) (-10,3,-10), (-10,7,10)								
**LiNbO3**	**(2,9,2)**	**14.14**	**80.12**	**7.818**	**7.1**	**2.499**	**17.77**	**31.24**	**35.94**
	Equiv. Refl.: (9,-11,2), (11,-9,2), (-2,11,2), (-9,-2,2), (-11,2,2)								
**Sapph**	**(0,7,20)**	**14.214**	**82.01**	**7.817**	**6.2**	**2.016**	**12.43**	**25.2**	**28.1**
	Equiv. Refl.: (0,-7,-20), (7,0,-20), (7,-7,20), (-7,0,20), (-7,7,-20)								
**LiNbO3**	**(2,6,-22)**	**14.047**	**78.15**	**7.644**	**6.8**	**3.011**	**20.58**	**37.64**	**42.9**
	Equiv. Refl.: (6,-8,-22), (8,-6,-22), (-2,8,-22), (-6,-2,-22), (-8,2,-22)								
**LiNbO3**	**(3,5,22)**	**13.839**	**74.62**	**7.583**	**6.4**	**3.947**	**25.14**	**49.34**	**55.37**
	Equiv. Refl.: (5,-8,22), (8,-5,22), (-3,8,22), (-5,-3,22), (-8,3,22)								
**LiNbO3**	**(0,10,-4)**	**14.019**	**77.62**	**7.57**	**6.6**	**3.149**	**20.68**	**39.37**	**44.47**
	Equiv. Refl.: (10,-10,-4), (-10,0,-4)								

Ei =<br>Cryst	14.353<br>Refl<br>(h,k,l)	keV<br>EB<br>[keV]	ΘB<br>[°]	∫ IR dΘ<br>[µrad]	Width<br>[µrad]	Ei cotΘ<br>[meV/µrad]	ΔE<br>[meV]	ΔEg<br>2m, 50µm<br>[meV]	ΔEt<br>2m, 50µm<br>[meV]
**LiNbO3**	**(1,-10,-10)**	**13.998**	**77.23**	**7.431**	**6.4**	**3.253**	**20.81**	**40.67**	**45.68**
	Equiv. Refl.: (9,1,-10), (10,-1,-10), (-1,-9,-10), (-9,10,-10), (-10,9,-10)								
**LiNbO3**	**(4,5,20)**	**14.069**	**78.58**	**7.371**	**6.4**	**2.9**	**18.68**	**36.25**	**40.78**
	Equiv. Refl.: (5,-9,20), (9,-5,20), (-4,9,20), (-5,-4,20), (-9,4,20)								
**LiNbO3**	**(0,-10,4)**	**14.019**	**77.62**	**7.342**	**6.6**	**3.149**	**20.68**	**39.37**	**44.47**
	Equiv. Refl.: (10,0,4), (-10,10,4)								
**Quartz**	**(0,9,-5)**	**14.312**	**85.68**	**7.305**	**6**	**1.085**	**6.48**	**13.56**	**15.03**
	Equiv. Refl.: (0,-9,5)								
**Quartz**	**(9,0,5)**	**14.312**	**85.68**	**7.303**	**6**	**1.085**	**6.47**	**13.56**	**15.02**
	Equiv. Refl.: (-9,0,-5)								
**Quartz**	**(9,-9,-5)**	**14.312**	**85.68**	**7.302**	**6**	**1.085**	**6.47**	**13.56**	**15.02**
	Equiv. Refl.: (-9,9,5)								
**LiNbO3**	**(3,-8,-22)**	**13.839**	**74.62**	**7.295**	**6.4**	**3.947**	**25.14**	**49.34**	**55.37**
	Equiv. Refl.: (5,3,-22), (8,-3,-22), (-3,-5,-22), (-5,8,-22), (-8,5,-22)								
**LiNbO3**	**(3,-11,-4)**	**13.811**	**74.2**	**7.19**	**6.1**	**4.06**	**24.75**	**50.75**	**56.47**
	Equiv. Refl.: (8,3,-4), (11,-3,-4), (-3,-8,-4), (-8,11,-4), (-11,8,-4)								
**LiNbO3**	**(1,9,10)**	**13.998**	**77.23**	**7.103**	**6.4**	**3.253**	**20.81**	**40.67**	**45.68**
	Equiv. Refl.: (9,-10,10), (10,-9,10), (-1,10,10), (-9,-1,10), (-10,1,10)								
**LiNbO3**	**(4,-9,-20)**	**14.069**	**78.58**	**7.012**	**6.4**	**2.9**	**18.68**	**36.25**	**40.78**
	Equiv. Refl.: (5,4,-20), (9,-4,-20), (-4,-5,-20), (-5,9,-20), (-9,5,-20)								
**LiNbO3**	**(3,8,4)**	**13.811**	**74.2**	**7.008**	**6.1**	**4.06**	**24.75**	**50.75**	**56.47**
	Equiv. Refl.: (8,-11,4), (11,-8,4), (-3,11,4), (-8,-3,4), (-11,3,4)								
**LiNbO3**	**(4,6,16)**	**14.076**	**78.73**	**6.849**	**6.1**	**2.861**	**17.39**	**35.77**	**39.77**
	Equiv. Refl.: (6,-10,16), (10,-6,16), (-4,10,16), (-6,-4,16), (-10,4,16)								
**LiNbO3**	**(5,-11,10)**	**13.998**	**77.23**	**6.701**	**5.9**	**3.253**	**19.04**	**40.67**	**44.9**
	Equiv. Refl.: (6,5,10), (11,-5,10), (-5,-6,10), (-6,11,10), (-11,6,10)								
**LiNbO3**	**(4,-10,-16)**	**14.076**	**78.73**	**6.539**	**6.1**	**2.861**	**17.39**	**35.77**	**39.77**
	Equiv. Refl.: (6,4,-16), (10,-4,-16), (-4,-6,-16), (-6,10,-16), (-10,6,-16)								
**LiNbO3**	**(0,-5,26)**	**13.547**	**70.7**	**6.469**	**5.5**	**5.025**	**27.51**	**62.81**	**68.57**
	Equiv. Refl.: (5,0,26), (-5,5,26)								
**Quartz**	**(9,1,3)**	**14.318**	**86.02**	**6.441**	**5.3**	**0.998**	**5.29**	**12.48**	**13.56**
	Equiv. Refl.: (9,-10,-3)								
**Quartz**	**(1,9,-3)**	**14.318**	**86.02**	**6.438**	**5.3**	**0.998**	**5.29**	**12.48**	**13.56**
**Quartz**	**(1,-10,3)**	**14.318**	**86.02**	**6.437**	**5.3**	**0.998**	**5.29**	**12.48**	**13.56**
	Equiv. Refl.: (-10,1,-3), (-10,9,3)								
**LiNbO3**	**(0,5,-26)**	**13.547**	**70.7**	**6.415**	**5.5**	**5.025**	**27.51**	**62.81**	**68.57**
	Equiv. Refl.: (5,-5,-26), (-5,0,-26)								
**LiNbO3**	**(5,6,-10)**	**13.998**	**77.23**	**6.384**	**5.9**	**3.253**	**19.04**	**40.67**	**44.9**
	Equiv. Refl.: (6,-11,-10), (11,-6,-10), (-5,11,-10), (-6,-5,-10), (-11,5,-10)								
**LiNbO3**	**(-5,6,-26)**	**13.968**	**76.71**	**6.164**	**5.5**	**3.391**	**18.65**	**42.39**	**46.32**
	Equiv. Refl.: (-6,5,-26)								
**LiNbO3**	**(1,-6,-26)**	**13.968**	**76.71**	**6.163**	**5.5**	**3.391**	**18.65**	**42.39**	**46.32**
	Equiv. Refl.: (5,1,-26), (6,-1,-26), (-1,-5,-26)								
**Quartz**	**(-9,10,3)**	**14.318**	**86.02**	**6.117**	**5.3**	**0.998**	**5.29**	**12.48**	**13.56**
	Equiv. Refl.: (-9,-1,-3)								
**Quartz**	**(-1,-9,3)**	**14.318**	**86.02**	**6.115**	**5.3**	**0.998**	**5.29**	**12.48**	**13.56**
**Quartz**	**(10,-1,3)**	**14.318**	**86.02**	**6.114**	**5.3**	**0.998**	**5.29**	**12.48**	**13.56**
	Equiv. Refl.: (10,-9,-3), (-1,10,-3)								
**LiNbO3**	**(0,-4,28)**	**13.701**	**72.66**	**6.112**	**5.3**	**4.481**	**23.64**	**56.02**	**60.8**
	Equiv. Refl.: (4,0,28), (-4,4,28)								

**Ei =**	**14.353**	**keV**						**ΔEg**	**ΔEt**
**Cryst**	**Refl**	**EB**	**ΘB**	**∫ IR dΘ**	**Width**	**Ei cotΘ**	**ΔE**	**2m, 50µm**	**2m, 50µm**
	**(h,k,l)**	**[keV]**	**[°]**	**[µrad]**	**[µrad]**	**[meV/µrad]**	**[meV]**	**[meV]**	**[meV]**
**LiNbO3**	**(1,-9,16)**	**13.868**	**75.07**	**6.1**	**5.4**	**3.827**	**20.58**	**47.84**	**52.08**
	Equiv. Refl.: (8,1,16), (9,-1,16), (-1,-8,16), (-8,9,16), (-9,8,16)								
**LiNbO3**	**(5,-6,26)**	**13.968**	**76.71**	**6.081**	**5.5**	**3.391**	**18.65**	**42.39**	**46.32**
	Equiv. Refl.: (6,-5,26)								
**LiNbO3**	**(1,5,26)**	**13.968**	**76.71**	**6.08**	**5.5**	**3.391**	**18.65**	**42.39**	**46.32**
	Equiv. Refl.: (-1,6,26), (-5,-1,26), (-6,1,26)								
**LiNbO3**	**(5,-11,-8)**	**13.738**	**73.17**	**6.078**	**5.3**	**4.341**	**22.82**	**54.26**	**58.87**
	Equiv. Refl.: (6,5,-8), (11,-5,-8), (-5,-6,-8), (-6,11,-8), (-11,6,-8)								
**LiNbO3**	**(1,8,-16)**	**13.868**	**75.07**	**6.052**	**5.4**	**3.827**	**20.58**	**47.84**	**52.08**
	Equiv. Refl.: (8,-9,-16), (9,-8,-16), (-1,9,-16), (-8,-1,-16), (-9,1,-16)								
**LiNbO3**	**(0,4,-28)**	**13.701**	**72.66**	**6.049**	**5.3**	**4.481**	**23.64**	**56.02**	**60.8**
	Equiv. Refl.: (4,-4,-28), (-4,0,-28)								
**LiNbO3**	**(2,8,-12)**	**13.828**	**74.45**	**5.995**	**5.2**	**3.994**	**20.81**	**49.92**	**54.08**
	Equiv. Refl.: (8,-10,-12), (10,-8,-12), (-2,10,-12), (-8,-2,-12), (-10,2,-12)								
**LiNbO3**	**(5,6,8)**	**13.738**	**73.17**	**5.98**	**5.3**	**4.341**	**22.82**	**54.26**	**58.87**
	Equiv. Refl.: (6,-11,8), (11,-6,8), (-5,11,8), (-6,-5,8), (-11,5,8)								
**Quartz**	**(-2,3,12)**	**14.294**	**84.8**	**5.922**	**4.9**	**1.307**	**6.44**	**16.34**	**17.56**
**Quartz**	**(3,-1,12)**	**14.294**	**84.8**	**5.921**	**4.9**	**1.307**	**6.44**	**16.34**	**17.56**
	Equiv. Refl.: (3,-2,-12), (-2,-1,-12)								
**Quartz**	**(2,-3,-12)**	**14.294**	**84.8**	**5.917**	**4.9**	**1.307**	**6.44**	**16.34**	**17.56**
**Quartz**	**(2,1,12)**	**14.294**	**84.8**	**5.916**	**4.9**	**1.307**	**6.44**	**16.34**	**17.56**
	Equiv. Refl.: (-1,3,-12), (-3,1,-12), (-3,2,12)								
**Quartz**	**(-1,-2,12)**	**14.294**	**84.8**	**5.915**	**4.9**	**1.307**	**6.43**	**16.34**	**17.56**
**Quartz**	**(1,-3,12)**	**14.294**	**84.8**	**5.911**	**4.9**	**1.307**	**6.43**	**16.34**	**17.56**
**Quartz**	**(1,2,-12)**	**14.294**	**84.8**	**5.91**	**4.9**	**1.307**	**6.43**	**16.34**	**17.56**
**LiNbO3**	**(2,-10,12)**	**13.828**	**74.45**	**5.796**	**5.2**	**3.994**	**20.81**	**49.92**	**54.08**
	Equiv. Refl.: (8,2,12), (10,-2,12), (-2,-8,12), (-8,10,12), (-10,8,12)								
**LiNbO3**	**(0,7,-22)**	**13.839**	**74.62**	**5.748**	**5**	**3.947**	**19.92**	**49.34**	**53.21**
	Equiv. Refl.: (7,-7,-22), (-7,0,-22)								
**LiNbO3**	**(4,-11,-6)**	**13.675**	**72.32**	**5.609**	**4.9**	**4.575**	**22.31**	**57.18**	**61.38**
	Equiv. Refl.: (7,4,-6), (11,-4,-6), (-4,-7,-6), (-7,11,-6), (-11,7,-6)								
**LiNbO3**	**(0,9,12)**	**13.616**	**71.56**	**5.595**	**4.8**	**4.785**	**22.98**	**59.81**	**64.07**
	Equiv. Refl.: (9,-9,12), (-9,0,12)								
**LiNbO3**	**(0,-7,22)**	**13.839**	**74.62**	**5.583**	**5**	**3.947**	**19.92**	**49.34**	**53.21**
	Equiv. Refl.: (7,0,22)								
**LiNbO3**	**(-7,7,22)**	**13.839**	**74.62**	**5.582**	**5**	**3.947**	**19.92**	**49.34**	**53.21**
**LiNbO3**	**(4,7,6)**	**13.675**	**72.32**	**5.519**	**4.9**	**4.575**	**22.31**	**57.18**	**61.38**
	Equiv. Refl.: (7,-11,6), (11,-7,6), (-4,11,6), (-7,-4,6), (-11,4,6)								
**LiNbO3**	**(3,-10,-14)**	**13.854**	**74.85**	**5.51**	**4.7**	**3.886**	**18.14**	**48.58**	**51.85**
	Equiv. Refl.: (7,3,-14), (10,-3,-14), (-3,-7,-14), (-7,10,-14), (-10,7,-14)								
**LiNbO3**	**(2,5,-24)**	**13.805**	**74.12**	**5.438**	**4.8**	**4.083**	**19.67**	**51.04**	**54.7**
	Equiv. Refl.: (5,-7,-24), (7,-5,-24), (-2,7,-24), (-5,-2,-24), (-7,2,-24)								
**LiNbO3**	**(0,-9,-12)**	**13.616**	**71.56**	**5.402**	**4.8**	**4.785**	**22.98**	**59.81**	**64.07**
	Equiv. Refl.: (9,0,-12), (-9,9,-12)								
**LiNbO3**	**(2,8,12)**	**13.828**	**74.45**	**5.399**	**4.7**	**3.994**	**18.62**	**49.92**	**53.28**
	Equiv. Refl.: (8,-10,12), (10,-8,12), (-2,10,12), (-8,-2,12), (-10,2,12)								
**LiNbO3**	**(4,7,-6)**	**13.675**	**72.32**	**5.352**	**4.6**	**4.575**	**21.24**	**57.18**	**61**
	Equiv. Refl.: (7,-11,-6), (11,-7,-6), (-4,11,-6), (-7,-4,-6), (-11,4,-6)								
**LiNbO3**	**(2,-7,24)**	**13.805**	**74.12**	**5.345**	**4.8**	**4.083**	**19.67**	**51.04**	**54.7**
	Equiv. Refl.: (5,2,24), (7,-2,24), (-2,-5,24), (-5,7,24), (-7,5,24)								

Ei =	14.353	keV						ΔEg	ΔEt
Cryst	Refl	EB	ΘB	∫ IR dΘ	Width	Ei cotΘ	ΔE	2m, 50µm	2m, 50µm
	(h,k,l)	[keV]	[°]	[µrad]	[µrad]	[meV/µrad]	[meV]	[meV]	[meV]
**LiNbO3**	**(3,6,-18)**	**13.66**	**72.12**	**5.309**	**4.7**	**4.629**	**21.54**	**57.87**	**61.75**
	Equiv. Refl.: (6,-9,-18), (9,-6,-18), (-3,9,-18), (-6,-3,-18), (-9,3,-18)								
**LiNbO3**	**(2,-7,-24)**	**13.805**	**74.12**	**5.304**	**4.7**	**4.083**	**19.37**	**51.04**	**54.59**
	Equiv. Refl.: (5,2,-24), (7,-2,-24), (-2,-5,-24), (-5,7,-24), (-7,5,-24)								
**LiNbO3**	**(2,-10,-12)**	**13.828**	**74.45**	**5.295**	**4.7**	**3.994**	**18.62**	**49.92**	**53.28**
	Equiv. Refl.: (8,2,-12), (10,-2,-12), (-2,-8,-12), (-8,10,-12), (-10,8,-12)								
**LiNbO3**	**(2,5,24)**	**13.805**	**74.12**	**5.283**	**4.7**	**4.083**	**19.37**	**51.04**	**54.59**
	Equiv. Refl.: (5,-7,24), (7,-5,24), (-2,7,24), (-5,-2,24), (-7,2,24)								
**LiNbO3**	**(1,-10,8)**	**13.738**	**73.17**	**5.258**	**4.3**	**4.341**	**18.7**	**54.26**	**57.4**
	Equiv. Refl.: (9,1,8), (10,-1,8), (-1,-9,8), (-9,10,8), (-10,9,8)								
**LiNbO3**	**(3,-9,18)**	**13.66**	**72.12**	**5.238**	**4.7**	**4.629**	**21.54**	**57.87**	**61.75**
	Equiv. Refl.: (6,3,18), (9,-3,18), (-3,-6,18), (-6,9,18), (-9,6,18)								
**Sapph**	**(2,4,25)**	**14.344**	**87.97**	**5.176**	**5.3**	**0.508**	**2.67**	**6.34**	**6.88**
	Equiv. Refl.: (2,-6,-25), (4,2,-25), (4,-6,25), (6,-2,-25), (6,-4,25), (-2,6,25), (-2,-4,-25), (-4,6,-25), (-4,-2,25) (-6,2,25), (-6,4,-25)								
**LiNbO3**	**(0,-10,-2)**	**13.934**	**76.12**	**5.167**	**4.4**	**3.548**	**15.7**	**44.35**	**47.05**
	Equiv. Refl.: (10,0,-2), (-10,10,-2)								
**LiNbO3**	**(3,7,14)**	**13.854**	**74.85**	**5.163**	**4.7**	**3.886**	**18.14**	**48.58**	**51.85**
	Equiv. Refl.: (7,-10,14), (10,-7,14), (-3,10,14), (-7,-3,14), (-10,3,14)								
**LiNbO3**	**(4,-11,6)**	**13.675**	**72.32**	**5.148**	**4.6**	**4.575**	**21.24**	**57.18**	**61**
	Equiv. Refl.: (7,4,6), (11,-4,6), (-4,-7,6), (-7,11,6), (-11,7,6)								
**Sapph**	**(0,8,16)**	**14.254**	**83.26**	**5.069**	**4.1**	**1.697**	**6.99**	**21.21**	**22.34**
	Equiv. Refl.: (0,-8,-16), (8,0,-16), (8,-8,16), (-8,0,16), (-8,8,-16)								
**LiNbO3**	**(4,6,-14)**	**13.643**	**71.91**	**5.043**	**4.2**	**4.69**	**19.48**	**58.62**	**61.77**
	Equiv. Refl.: (6,-10,-14), (10,-6,-14), (-4,10,-14), (-6,-4,-14), (-10,4,-14)								
**LiNbO3**	**(0,10,2)**	**13.934**	**76.12**	**5.026**	**4.4**	**3.548**	**15.7**	**44.35**	**47.05**
	Equiv. Refl.: (10,-10,2), (-10,0,2)								
**LiNbO3**	**(3,6,18)**	**13.66**	**72.12**	**4.987**	**4.2**	**4.629**	**19.31**	**57.87**	**61**
	Equiv. Refl.: (6,-9,18), (9,-6,18), (-3,9,18), (-6,-3,18), (-9,3,18)								
**LiNbO3**	**(2,3,-28)**	**13.911**	**75.74**	**4.973**	**4.3**	**3.648**	**15.72**	**45.6**	**48.23**
	Equiv. Refl.: (3,-5,-28), (5,-3,-28), (-2,5,-28), (-3,-2,-28), (-5,2,-28)								
**LiNbO3**	**(3,-9,-18)**	**13.66**	**72.12**	**4.954**	**4.2**	**4.629**	**19.31**	**57.87**	**61**
	Equiv. Refl.: (6,3,-18), (9,-3,-18), (-3,-6,-18), (-6,9,-18), (-9,6,-18)								
**LiNbO3**	**(2,-5,28)**	**13.911**	**75.74**	**4.944**	**4.3**	**3.648**	**15.72**	**45.6**	**48.23**
	Equiv. Refl.: (5,-2,28)								
**LiNbO3**	**(3,2,28)**	**13.911**	**75.74**	**4.943**	**4.3**	**3.648**	**15.72**	**45.6**	**48.23**
	Equiv. Refl.: (-2,-3,28), (-3,5,28), (-5,3,28)								
**LiNbO3**	**(1,9,-8)**	**13.738**	**73.17**	**4.927**	**4.3**	**4.341**	**18.7**	**54.26**	**57.4**
	Equiv. Refl.: (9,-10,-8), (10,-9,-8), (-1,10,-8), (-9,-1,-8), (-10,1,-8)								
**LiNbO3**	**(4,-10,14)**	**13.643**	**71.91**	**4.848**	**4.2**	**4.69**	**19.48**	**58.62**	**61.77**
	Equiv. Refl.: (10,-4,14)								
**LiNbO3**	**(6,4,14)**	**13.643**	**71.91**	**4.847**	**4.2**	**4.69**	**19.48**	**58.62**	**61.77**
	Equiv. Refl.: (-4,-6,14), (-6,10,14), (-10,6,14)								
**Quartz**	**(5,6,3)**	**14.318**	**86.02**	**4.845**	**4.1**	**0.998**	**4.09**	**12.48**	**13.13**
	Equiv. Refl.: (5,-11,-3), (6,5,-3), (6,-11,3)								
**Quartz**	**(-11,5,3)**	**14.318**	**86.02**	**4.844**	**4.1**	**0.998**	**4.09**	**12.48**	**13.13**
	Equiv. Refl.: (-11,6,-3)								
**LiNbO3**	**(0,-6,-24)**	**13.594**	**71.28**	**4.806**	**4**	**4.864**	**19.27**	**60.8**	**63.78**
	Equiv. Refl.: (6,0,-24), (-6,6,-24)								

Ei =	14.353	keV						ΔEg	ΔEt
Cryst	Refl	EB	ΘB	∫ IR dΘ	Width	Ei cotΘ	ΔE	2m, 50µm	2m, 50µm
	(h,k,l)	[keV]	[°]	[µrad]	[µrad]	[meV/µrad]	[meV]	[meV]	[meV]
**Quartz**	**(-5,11,3)**	**14.318**	**86.02**	**4.761**	**4.1**	**0.998**	**4.09**	**12.48**	**13.13**
	Equiv. Refl.: (-5,-6,-3), (-6,11,-3), (-6,-5,3)								
**Quartz**	**(11,-5,-3)**	**14.318**	**86.02**	**4.76**	**4.1**	**0.998**	**4.09**	**12.48**	**13.13**
	Equiv. Refl.: (11,-6,3)								
**LiNbO3**	**(2,4,-26)**	**13.759**	**73.46**	**4.624**	**3.9**	**4.262**	**16.83**	**53.28**	**55.87**
	Equiv. Refl.: (4,-6,-26), (6,-4,-26), (-2,6,-26), (-4,-2,-26), (-6,2,-26)								
**LiNbO3**	**(0,6,24)**	**13.594**	**71.28**	**4.595**	**4**	**4.864**	**19.27**	**60.8**	**63.78**
	Equiv. Refl.: (6,-6,24), (-6,0,24)								
**Sapph**	**(4,6,10)**	**13.957**	**76.51**	**4.594**	**3.5**	**3.445**	**11.95**	**43.06**	**44.69**
	Equiv. Refl.: (4,-10,-10), (6,4,-10), (6,-10,10), (10,-4,-10), (10,-6,10), (-4,10,10), (-4,-6,-10), (-6,10,-10), (-6,-4,10) (-10,4,10), (-10,6,-10)								
**LiNbO3**	**(2,-6,26)**	**13.759**	**73.46**	**4.558**	**3.9**	**4.262**	**16.83**	**53.28**	**55.87**
	Equiv. Refl.: (4,2,26), (6,-2,26), (-2,-4,26), (-4,6,26), (-6,4,26)								
**Quartz**	**(8,-1,-8)**	**14.325**	**86.41**	**4.521**	**3.8**	**0.9**	**3.42**	**11.25**	**11.76**
**Quartz**	**(8,-7,8)**	**14.325**	**86.41**	**4.52**	**3.8**	**0.9**	**3.42**	**11.25**	**11.76**
**Quartz**	**(-1,-7,-8)**	**14.325**	**86.41**	**4.516**	**3.8**	**0.9**	**3.42**	**11.25**	**11.76**
**Quartz**	**(-1,8,8)**	**14.325**	**86.41**	**4.514**	**3.8**	**0.9**	**3.42**	**11.25**	**11.76**
**Quartz**	**(-7,8,-8)**	**14.325**	**86.41**	**4.511**	**3.8**	**0.9**	**3.42**	**11.25**	**11.76**
	Equiv. Refl.: (-7,-1,8)								
**Quartz**	**(0,-2,12)**	**14.069**	**78.59**	**4.503**	**3.3**	**2.897**	**9.67**	**36.21**	**37.48**
**Quartz**	**(0,2,-12)**	**14.069**	**78.59**	**4.502**	**3.3**	**2.897**	**9.67**	**36.21**	**37.48**
**Quartz**	**(2,0,12)**	**14.069**	**78.59**	**4.501**	**3.3**	**2.897**	**9.67**	**36.21**	**37.48**
	Equiv. Refl.: (2,-2,-12), (-2,0,-12), (-2,2,12)								
**LiNbO3**	**(0,6,-24)**	**13.594**	**71.28**	**4.429**	**3.8**	**4.864**	**18.29**	**60.8**	**63.49**
	Equiv. Refl.: (6,-6,-24), (-6,0,-24)								
**LiNbO3**	**(0,-6,24)**	**13.594**	**71.28**	**4.419**	**3.8**	**4.864**	**18.29**	**60.8**	**63.49**
	Equiv. Refl.: (6,0,24), (-6,6,24)								
**Quartz**	**(11,-4,-2)**	**14.237**	**82.7**	**4.263**	**3.4**	**1.838**	**6.19**	**22.98**	**23.8**
	Equiv. Refl.: (11,-7,2), (-4,11,2), (-4,-7,-2), (-7,11,-2), (-7,-4,2)								
**Quartz**	**(4,7,2)**	**14.237**	**82.7**	**4.215**	**3.4**	**1.838**	**6.19**	**22.98**	**23.8**
	Equiv. Refl.: (4,-11,-2), (7,4,-2), (7,-11,2), (-11,4,2), (-11,7,-2)								
**LiNbO3**	**(0,-9,12)**	**13.616**	**71.56**	**4.149**	**3.5**	**4.785**	**16.83**	**59.81**	**62.13**
	Equiv. Refl.: (9,0,12), (-9,9,12)								
**Quartz**	**(-7,-1,-8)**	**14.325**	**86.41**	**4.141**	**3.6**	**0.9**	**3.25**	**11.25**	**11.71**
**Quartz**	**(-7,8,8)**	**14.325**	**86.41**	**4.14**	**3.6**	**0.9**	**3.25**	**11.25**	**11.71**
**Quartz**	**(-1,8,-8)**	**14.325**	**86.41**	**4.138**	**3.6**	**0.9**	**3.25**	**11.25**	**11.71**
**Quartz**	**(-1,-7,8)**	**14.325**	**86.41**	**4.137**	**3.6**	**0.9**	**3.25**	**11.25**	**11.71**
**Quartz**	**(8,-1,8)**	**14.325**	**86.41**	**4.127**	**3.6**	**0.9**	**3.24**	**11.25**	**11.71**
	Equiv. Refl.: (8,-7,-8)								
**Quartz**	**(-8,1,8)**	**14.325**	**86.41**	**4.078**	**3.8**	**0.9**	**3.42**	**11.25**	**11.76**
**Quartz**	**(-8,7,-8)**	**14.325**	**86.41**	**4.077**	**3.8**	**0.9**	**3.42**	**11.25**	**11.76**
**Quartz**	**(1,7,8)**	**14.325**	**86.41**	**4.073**	**3.8**	**0.9**	**3.42**	**11.25**	**11.76**
**Quartz**	**(1,-8,-8)**	**14.325**	**86.41**	**4.071**	**3.8**	**0.9**	**3.42**	**11.25**	**11.76**
**Quartz**	**(7,1,-8)**	**14.325**	**86.41**	**4.068**	**3.8**	**0.9**	**3.42**	**11.25**	**11.76**
	Equiv. Refl.: (7,-8,8)								
**LiNbO3**	**(1,1,30)**	**13.63**	**71.73**	**4.022**	**3.4**	**4.737**	**16.28**	**59.22**	**61.41**
	Equiv. Refl.: (1,-2,30), (2,-1,30), (-1,2,30), (-1,-1,30), (-2,1,30)								
**LiNbO3**	**(0,9,-12)**	**13.616**	**71.56**	**3.982**	**3.5**	**4.785**	**16.83**	**59.81**	**62.13**
	Equiv. Refl.: (9,-9,-12), (-9,0,-12)								
**Quartz**	**(7,1,8)**	**14.325**	**86.41**	**3.928**	**3.6**	**0.9**	**3.25**	**11.25**	**11.71**
**Quartz**	**(7,-8,-8)**	**14.325**	**86.41**	**3.927**	**3.6**	**0.9**	**3.25**	**11.25**	**11.71**

Ei =	14.353	keV						ΔEg	ΔEt
Cryst	Refl	EB	ΘB	∫ IR dΘ	Width	Ei cotΘ	ΔE	2m, 50μm	2m, 50μm
	(h,k,l)	[keV]	[°]	[μrad]	[μrad]	[meV/μrad]	[meV]	[meV]	[meV]
**Quartz**	**(3,-1,-12)**	**14.294**	**84.8**	**3.926**	**3.3**	**1.307**	**4.26**	**16.34**	**16.89**
	Equiv. Refl.: (3,-2,12)								
**Quartz**	**(1,-8,8)**	**14.325**	**86.41**	**3.925**	**3.6**	**0.9**	**3.25**	**11.25**	**11.71**
**Quartz**	**(1,7,-8)**	**14.325**	**86.41**	**3.924**	**3.6**	**0.9**	**3.25**	**11.25**	**11.71**
**Quartz**	**(-1,-2,-12)**	**14.294**	**84.8**	**3.921**	**3.3**	**1.307**	**4.26**	**16.34**	**16.89**
**Quartz**	**(-1,3,12)**	**14.294**	**84.8**	**3.919**	**3.3**	**1.307**	**4.26**	**16.34**	**16.89**
**Quartz**	**(-2,3,-12)**	**14.294**	**84.8**	**3.914**	**3.3**	**1.307**	**4.25**	**16.34**	**16.89**
	Equiv. Refl.: (-2,-1,12), (-8,1,-8), (-8,7,8)								
**Sapph**	**(1,8,-13)**	**14.273**	**83.95**	**3.91**	**3.4**	**1.522**	**5.12**	**19.02**	**19.7**
	Equiv. Refl.: (1,-9,13), (8,1,13), (8,-9,-13), (9,-1,13), (9,-8,-13), (-1,9,-13), (-1,-8,13), (-8,9,13), (-8,-1,-13) (-9,1,-13), (-9,8,13)								
**LiNbO3**	**(1,1,-30)**	**13.63**	**71.73**	**3.875**	**3.4**	**4.737**	**16.28**	**59.22**	**61.41**
	Equiv. Refl.: (1,-2,-30), (2,-1,-30), (-1,2,-30), (-1,-1,-30), (-2,1,-30)								
**Quartz**	**(-3,1,12)**	**14.294**	**84.8**	**3.775**	**3.3**	**1.307**	**4.26**	**16.34**	**16.89**
	Equiv. Refl.: (-3,2,-12)								
**Quartz**	**(1,2,12)**	**14.294**	**84.8**	**3.77**	**3.3**	**1.307**	**4.26**	**16.34**	**16.89**
**Quartz**	**(1,-3,-12)**	**14.294**	**84.8**	**3.769**	**3.3**	**1.307**	**4.26**	**16.34**	**16.89**
**Quartz**	**(2,1,-12)**	**14.294**	**84.8**	**3.763**	**3.3**	**1.307**	**4.25**	**16.34**	**16.89**
	Equiv. Refl.: (2,-3,12)								
**Quartz**	**(11,-4,2)**	**14.237**	**82.7**	**3.744**	**3**	**1.838**	**5.5**	**22.98**	**23.63**
	Equiv. Refl.: (11,-7,-2), (-4,11,-2), (-4,-7,2)								
**Quartz**	**(-7,11,2)**	**14.237**	**82.7**	**3.742**	**3**	**1.838**	**5.49**	**22.98**	**23.63**
	Equiv. Refl.: (-7,-4,-2)								
**Quartz**	**(4,7,-2)**	**14.237**	**82.7**	**3.68**	**3**	**1.838**	**5.5**	**22.98**	**23.63**
	Equiv. Refl.: (4,-11,2), (-11,4,-2), (-11,7,2)								
**Quartz**	**(7,4,2)**	**14.237**	**82.7**	**3.679**	**3**	**1.838**	**5.49**	**22.98**	**23.63**
	Equiv. Refl.: (7,-11,-2)								
**Sapph**	**(2,8,6)**	**14.082**	**78.86**	**3.443**	**2.8**	**2.827**	**7.8**	**35.33**	**36.18**
	Equiv. Refl.: (2,8,-6), (2,-10,6), (2,-10,-6), (8,2,6), (8,2,-6), (8,-10,6), (8,-10,-6), (10,-2,6), (10,-2,-6) (10,-8,6), (10,-8,-6), (-2,10,6), (-2,10,-6), (-2,-8,6), (-2,-8,-6), (-8,10,6), (-8,10,-6), (-8,-2,6) (-8,-2,-6), (-10,2,6), (-10,2,-6), (-10,8,6), (-10,8,-6)								
**LiNbO3**	**(3,-11,2)**	**13.724**	**72.97**	**3.431**	**3.2**	**4.395**	**14**	**54.94**	**56.7**
	Equiv. Refl.: (8,3,2), (11,-3,2), (-3,-8,2), (-8,11,2), (-11,8,2)								
**Quartz**	**(11,-5,3)**	**14.318**	**86.02**	**3.42**	**2.9**	**0.998**	**2.94**	**12.48**	**12.82**
	Equiv. Refl.: (11,-6,-3)								
**Quartz**	**(-6,11,3)**	**14.318**	**86.02**	**3.418**	**2.9**	**0.998**	**2.93**	**12.48**	**12.82**
	Equiv. Refl.: (-6,-5,-3)								
**Quartz**	**(-5,11,-3)**	**14.318**	**86.02**	**3.415**	**2.9**	**0.998**	**2.93**	**12.48**	**12.82**
**Quartz**	**(-5,-6,3)**	**14.318**	**86.02**	**3.414**	**2.9**	**0.998**	**2.93**	**12.48**	**12.82**
**LiNbO3**	**(3,8,-2)**	**13.724**	**72.97**	**3.358**	**3.2**	**4.395**	**14**	**54.94**	**56.7**
	Equiv. Refl.: (8,-11,-2), (11,-8,-2), (-3,11,-2), (-8,-3,-2), (-11,3,-2)								
**Quartz**	**(7,-1,-9)**	**14.066**	**78.52**	**3.327**	**2.6**	**2.915**	**7.45**	**36.44**	**37.19**
	Equiv. Refl.: (7,-6,9)								
**Quartz**	**(-6,7,-9)**	**14.066**	**78.52**	**3.326**	**2.6**	**2.915**	**7.45**	**36.44**	**37.19**
	Equiv. Refl.: (-6,-1,9)								
**Quartz**	**(-1,7,9)**	**14.066**	**78.52**	**3.324**	**2.6**	**2.915**	**7.44**	**36.44**	**37.19**
	Equiv. Refl.: (-1,-6,-9)								
**Quartz**	**(-11,5,-3)**	**14.318**	**86.02**	**3.298**	**2.9**	**0.998**	**2.94**	**12.48**	**12.82**
	Equiv. Refl.: (-11,6,3)								

Ei = Cryst	14.353 Refl (h,k,l)	keV EB [keV]	ΘB [°]	∫ IR dΘ [μrad]	Width [μrad]	Ei cotΘ [meV/μrad]	ΔE [meV]	ΔEg 2m, 50μm [meV]	ΔEt 2m, 50μm [meV]
**Quartz**	**(6,5,3)**	**14.318**	**86.02**	**3.296**	**2.9**	**0.998**	**2.94**	**12.48**	**12.82**
	Equiv. Refl.: (6,-11,-3)								
**Quartz**	**(5,6,-3)**	**14.318**	**86.02**	**3.293**	**2.9**	**0.998**	**2.93**	**12.48**	**12.82**
	Equiv. Refl.: (5,-11,3)								
**Sapph**	**(3,7,8)**	**13.906**	**75.66**	**3.27**	**2.6**	**3.668**	**9.42**	**45.85**	**46.81**
	Equiv. Refl.: (3,-10,-8), (7,3,-8), (7,-10,8), (10,-3,-8), (10,-7,8), (-3,10,8), (-3,-7,-8), (-7,10,-8), (-7,-3,8) (-10,3,8), (-10,7,-8)								
**Quartz**	**(6,1,-9)**	**14.066**	**78.52**	**3.258**	**2.6**	**2.915**	**7.45**	**36.44**	**37.19**
	Equiv. Refl.: (6,-7,9), (-7,1,9), (-7,6,-9)								
**Quartz**	**(1,6,9)**	**14.066**	**78.52**	**3.256**	**2.6**	**2.915**	**7.44**	**36.44**	**37.19**
**Quartz**	**(1,-7,-9)**	**14.066**	**78.52**	**3.255**	**2.6**	**2.915**	**7.44**	**36.44**	**37.19**
**Sapph**	**(4,5,17)**	**14.279**	**84.19**	**3.233**	**2.8**	**1.461**	**4.08**	**18.26**	**18.71**
	Equiv. Refl.: (4,-9,-17), (5,4,-17), (5,-9,17), (9,-4,-17), (9,-5,17), (-4,9,17), (-4,-5,-17), (-5,9,-17), (-5,-4,17) (-9,4,17), (-9,5,-17)								
**Sapph**	**(0,9,0)**	**13.54**	**70.62**	**3.132**	**2.4**	**5.049**	**12.02**	**63.11**	**64.24**
	Equiv. Refl.: (0,-9,0), (9,0,0), (9,-9,0), (-9,0,0), (-9,9,0)								
**Sapph**	**(0,8,-14)**	**13.766**	**73.56**	**3.058**	**2.4**	**4.236**	**10.05**	**52.95**	**53.9**
	Equiv. Refl.: (0,-8,14), (8,0,14), (8,-8,-14), (-8,0,-14), (-8,8,14)								
**Sapph**	**(1,6,-20)**	**13.727**	**73.02**	**3.056**	**2.4**	**4.382**	**10.35**	**54.77**	**55.74**
	Equiv. Refl.: (1,-7,20), (6,1,20), (6,-7,-20), (7,-1,20), (7,-6,-20), (-1,7,-20), (-1,-6,20), (-6,7,20), (-6,-1,-20) (-7,1,-20), (-7,6,20)								
**Quartz**	**(11,-5,-1)**	**13.946**	**76.32**	**3.055**	**2.2**	**3.493**	**7.78**	**43.66**	**44.35**
	Equiv. Refl.: (11,-6,1)								
**Quartz**	**(-5,11,1)**	**13.946**	**76.32**	**3.054**	**2.2**	**3.493**	**7.78**	**43.66**	**44.35**
	Equiv. Refl.: (-5,-6,-1), (-6,11,-1), (-6,-5,1)								
**Sapph**	**(2,7,-14)**	**14.01**	**77.46**	**3.05**	**2.3**	**3.194**	**7.46**	**39.92**	**40.61**
	Equiv. Refl.: (2,-9,14), (7,2,14), (7,-9,-14), (9,-2,14), (9,-7,-14), (-2,9,-14), (-2,-7,14), (-7,9,14), (-7,-2,-14) (-9,2,-14), (-9,7,14)								
**Quartz**	**(-7,1,-9)**	**14.066**	**78.52**	**3.017**	**2.4**	**2.915**	**6.87**	**36.44**	**37.08**
	Equiv. Refl.: (-7,6,9)								
**Quartz**	**(1,-7,9)**	**14.066**	**78.52**	**3.016**	**2.4**	**2.915**	**6.87**	**36.44**	**37.08**
**Quartz**	**(1,6,-9)**	**14.066**	**78.52**	**3.015**	**2.4**	**2.915**	**6.87**	**36.44**	**37.08**
	Equiv. Refl.: (6,1,9), (6,-7,-9)								
**Quartz**	**(5,6,1)**	**13.946**	**76.32**	**2.998**	**2.2**	**3.493**	**7.78**	**43.66**	**44.35**
	Equiv. Refl.: (5,-11,-1), (6,5,-1), (6,-11,1), (-11,5,1), (-11,6,-1)								
**Quartz**	**(7,-1,9)**	**14.066**	**78.52**	**2.997**	**2.4**	**2.915**	**6.87**	**36.44**	**37.08**
**Quartz**	**(7,-6,-9)**	**14.066**	**78.52**	**2.996**	**2.4**	**2.915**	**6.87**	**36.44**	**37.08**
	Equiv. Refl.: (-1,7,-9)								
**Quartz**	**(4,6,-5)**	**13.936**	**76.16**	**2.995**	**2.3**	**3.535**	**8.13**	**44.19**	**44.93**
	Equiv. Refl.: (-1,-6,9), (-6,7,9)								
**Quartz**	**(4,-10,5)**	**13.936**	**76.16**	**2.994**	**2.3**	**3.535**	**8.13**	**44.19**	**44.93**
	Equiv. Refl.: (-6,-1,-9)								
**Quartz**	**(6,4,5)**	**13.936**	**76.16**	**2.993**	**2.3**	**3.535**	**8.13**	**44.19**	**44.93**
	Equiv. Refl.: (6,-10,-5)								
**Quartz**	**(-10,4,-5)**	**13.936**	**76.16**	**2.992**	**2.3**	**3.535**	**8.13**	**44.19**	**44.93**
	Equiv. Refl.: (-10,6,5)								
**Quartz**	**(-4,10,-5)**	**13.936**	**76.16**	**2.98**	**2.3**	**3.535**	**8.13**	**44.19**	**44.93**
	Equiv. Refl.: (-4,-6,5)								
**Quartz**	**(-6,-4,-5)**	**13.936**	**76.16**	**2.979**	**2.3**	**3.535**	**8.13**	**44.19**	**44.93**
**Quartz**	**(-6,10,5)**	**13.936**	**76.16**	**2.978**	**2.3**	**3.535**	**8.13**	**44.19**	**44.93**

Ei =	14.353	keV						ΔEg	ΔEt
Cryst	Refl	EB	ΘB	∫ IR dΘ	Width	Ei cotΘ	ΔE	2m, 50µm	2m, 50µm
	(h,k,l)	[keV]	[°]	[µrad]	[µrad]	[meV/µrad]	[meV]	[meV]	[meV]
**Quartz**	**(10,-4,5)**	**13.936**	**76.16**	**2.977**	**2.3**	**3.535**	**8.13**	**44.19**	**44.93**
	Equiv. Refl.: (10,-6,-5)								
**Sapph**	**(4,5,-16)**	**14.014**	**77.51**	**2.916**	**2.3**	**3.178**	**7.16**	**39.73**	**40.37**
	Equiv. Refl.: (4,-9,16), (5,4,16), (5,-9,-16), (9,-4,16), (9,-5,-16), (-4,9,-16), (-4,-5,16), (-5,9,16), (-5,-4,-16) (-9,4,-16), (-9,5,16)								
**Quartz**	**(0,9,-3)**	**13.557**	**70.83**	**2.892**	**2.2**	**4.991**	**10.85**	**62.38**	**63.32**
	Equiv. Refl.: (0,-9,3), (9,0,3), (9,-9,-3), (-9,0,-3), (-9,9,3)								
**Quartz**	**(10,-1,-1)**	**13.946**	**76.32**	**2.877**	**2.1**	**3.493**	**7.44**	**43.66**	**44.29**
	Equiv. Refl.: (10,-9,1), (-1,10,1), (-1,-9,-1), (-9,10,-1), (-9,-1,1)								
**Quartz**	**(1,9,1)**	**13.946**	**76.32**	**2.873**	**2.1**	**3.493**	**7.44**	**43.66**	**44.29**
	Equiv. Refl.: (9,1,-1), (9,-10,1), (-10,1,1), (-10,9,-1)								
**Quartz**	**(1,-10,-1)**	**13.946**	**76.32**	**2.872**	**2.1**	**3.493**	**7.44**	**43.66**	**44.29**
**Quartz**	**(-2,8,-8)**	**13.949**	**76.38**	**2.792**	**2.1**	**3.477**	**7.22**	**43.47**	**44.06**
	Equiv. Refl.: (-2,-6,8), (-6,8,8), (-6,-2,-8)								
**Quartz**	**(8,-2,8)**	**13.949**	**76.38**	**2.791**	**2.1**	**3.477**	**7.21**	**43.47**	**44.06**
	Equiv. Refl.: (8,-6,-8)								
**Quartz**	**(2,6,-8)**	**13.949**	**76.38**	**2.788**	**2.1**	**3.477**	**7.22**	**43.47**	**44.06**
	Equiv. Refl.: (2,-8,8), (6,2,8), (6,-8,-8)								
**Quartz**	**(-8,6,8)**	**13.949**	**76.38**	**2.787**	**2.1**	**3.477**	**7.21**	**43.47**	**44.06**
**Quartz**	**(-8,2,-8)**	**13.949**	**76.38**	**2.786**	**2.1**	**3.477**	**7.21**	**43.47**	**44.06**
**Sapph**	**(4,5,14)**	**13.517**	**70.35**	**2.751**	**2**	**5.125**	**10.43**	**64.07**	**64.91**
	Equiv. Refl.: (4,-9,-14), (5,4,-14), (5,-9,14), (9,-4,-14), (9,-5,14), (-4,9,14), (-4,-5,-14), (-5,9,-14), (-5,-4,14) (-9,4,14), (-9,5,-14)								
**Quartz**	**(9,-1,-6)**	**14.224**	**82.32**	**2.71**	**2.1**	**1.936**	**4.14**	**24.2**	**24.55**
	Equiv. Refl.: (9,-8,6)								
**Quartz**	**(-1,-8,-6)**	**14.224**	**82.32**	**2.706**	**2.1**	**1.936**	**4.13**	**24.2**	**24.55**
	Equiv. Refl.: (-8,9,-6), (-8,-1,6)								
**Quartz**	**(-1,9,6)**	**14.224**	**82.32**	**2.705**	**2.1**	**1.936**	**4.13**	**24.2**	**24.55**
**LiNbO3**	**(4,6,-17)**	**14.308**	**85.49**	**2.603**	**4.1**	**1.133**	**4.69**	**14.16**	**14.92**
	Equiv. Refl.: (4,-10,17), (6,4,17), (6,-10,-17), (10,-4,17), (10,-6,-17), (-4,10,-17), (-4,-6,17), (-6,10,17), (-6,-4,-17) (-10,4,-17), (-10,6,17)								
**Sapph**	**(3,5,-20)**	**14.214**	**82.01**	**2.501**	**2.4**	**2.016**	**4.8**	**25.2**	**25.65**
	Equiv. Refl.: (3,-8,20), (5,3,20), (5,-8,-20), (8,-3,20), (8,-5,-20), (-3,8,-20), (-3,-5,20), (-5,8,20), (-5,-3,-20) (-8,3,-20), (-8,5,20)								
**Quartz**	**(-1,5,11)**	**14.275**	**84.02**	**2.452**	**2.1**	**1.503**	**3.2**	**18.79**	**19.06**
	Equiv. Refl.: (-1,-4,-11)								
**Quartz**	**(-4,5,-11)**	**14.275**	**84.02**	**2.446**	**2.1**	**1.503**	**3.2**	**18.79**	**19.06**
**Quartz**	**(5,-1,-11)**	**14.275**	**84.02**	**2.444**	**2.1**	**1.503**	**3.19**	**18.79**	**19.06**
	Equiv. Refl.: (-4,-1,11)								
**Quartz**	**(5,-4,11)**	**14.275**	**84.02**	**2.443**	**2.1**	**1.503**	**3.19**	**18.79**	**19.06**
**Sapph**	**(2,3,26)**	**14.036**	**77.93**	**2.395**	**2.1**	**3.069**	**6.45**	**38.37**	**38.91**
	Equiv. Refl.: (2,-5,-26), (3,2,-26), (3,-5,26), (5,-2,-26), (5,-3,26), (-2,5,26), (-2,-3,-26), (-3,5,-26), (-3,-2,26) (-5,2,26), (-5,3,-26)								
**Quartz**	**(0,2,12)**	**14.069**	**78.59**	**2.387**	**2.1**	**2.897**	**6.07**	**36.21**	**36.72**
	Equiv. Refl.: (0,-2,-12)								
**Quartz**	**(2,-2,12)**	**14.069**	**78.59**	**2.386**	**2.1**	**2.897**	**6.07**	**36.21**	**36.72**
	Equiv. Refl.: (-2,0,12), (-2,2,-12)								
**Quartz**	**(2,0,-12)**	**14.069**	**78.59**	**2.385**	**2.1**	**2.897**	**6.07**	**36.21**	**36.72**
**Quartz**	**(-9,1,6)**	**14.224**	**82.32**	**2.362**	**2.1**	**1.936**	**4.14**	**24.2**	**24.55**
	Equiv. Refl.: (-9,8,-6)								
**Quartz**	**(1,8,6)**	**14.224**	**82.32**	**2.36**	**2.1**	**1.936**	**4.13**	**24.2**	**24.55**

Ei = Cryst	14.353 Refl (h,k,l)	keV EB [keV]	ΘB [°]	∫ IR dΘ [µrad]	Width [µrad]	Ei cotΘ [meV/µrad]	ΔE [meV]	ΔEg 2m, 50µm [meV]	ΔEt 2m, 50µm [meV]
**Quartz**	**(1,-9,-6)**	**14.224**	**82.32**	**2.359**	**2.1**	**1.936**	**4.13**	**24.2**	**24.55**
	Equiv. Refl.: (8,1,-6), (8,-9,6)								
**Quartz**	**(1,5,-10)**	**14.049**	**78.19**	**2.332**	**2**	**3.002**	**5.95**	**37.53**	**38**
	Equiv. Refl.: (1,-6,10)								
**Quartz**	**(-6,1,-10)**	**14.049**	**78.19**	**2.331**	**2**	**3.002**	**5.94**	**37.53**	**38**
	Equiv. Refl.: (-6,5,10)								
**Quartz**	**(5,1,10)**	**14.049**	**78.19**	**2.33**	**2**	**3.002**	**5.94**	**37.53**	**38**
	Equiv. Refl.: (5,-6,-10)								
**Quartz**	**(-1,6,-10)**	**14.049**	**78.19**	**2.316**	**2**	**3.002**	**5.95**	**37.53**	**38**
**Quartz**	**(0,9,4)**	**13.892**	**75.45**	**2.315**	**1.9**	**3.727**	**7.17**	**46.58**	**47.13**
	Equiv. Refl.: (0,-9,-4), (9,0,-4), (9,-9,4), (-1,-5,10), (-9,0,4), (-9,9,-4)								
**Quartz**	**(6,-1,10)**	**14.049**	**78.19**	**2.314**	**2**	**3.002**	**5.94**	**37.53**	**38**
	Equiv. Refl.: (6,-5,-10), (-5,6,10), (-5,-1,-10)								
**LiNbO3**	**(1,2,-31)**	**14.342**	**87.79**	**2.116**	**4.7**	**0.554**	**2.61**	**6.92**	**7.4**
	Equiv. Refl.: (1,-3,31), (2,1,31), (2,-3,-31), (3,-1,31), (3,-2,-31), (-1,3,-31), (-1,-2,31), (-2,3,31), (-2,-1,-31) (-3,1,-31), (-3,2,31)								
**Quartz**	**(8,1,6)**	**14.224**	**82.32**	**1.921**	**2**	**1.936**	**3.84**	**24.2**	**24.5**
	Equiv. Refl.: (8,-9,-6)								
**Quartz**	**(-9,1,-6)**	**14.224**	**82.32**	**1.92**	**2**	**1.936**	**3.84**	**24.2**	**24.5**
**Quartz**	**(1,8,-6)**	**14.224**	**82.32**	**1.919**	**2**	**1.936**	**3.84**	**24.2**	**24.5**
	Equiv. Refl.: (1,-9,6), (-9,8,6)								
**Quartz**	**(1,4,11)**	**14.275**	**84.02**	**1.834**	**2.1**	**1.503**	**3.2**	**18.79**	**19.06**
	Equiv. Refl.: (1,-5,-11)								
**Quartz**	**(4,-5,11)**	**14.275**	**84.02**	**1.83**	**2.1**	**1.503**	**3.2**	**18.79**	**19.06**
**Quartz**	**(4,1,-11)**	**14.275**	**84.02**	**1.829**	**2.1**	**1.503**	**3.19**	**18.79**	**19.06**
**Quartz**	**(-5,1,11)**	**14.275**	**84.02**	**1.828**	**2.1**	**1.503**	**3.19**	**18.79**	**19.06**
**Quartz**	**(-5,4,-11)**	**14.275**	**84.02**	**1.827**	**2.1**	**1.503**	**3.19**	**18.79**	**19.06**
**Quartz**	**(4,5,7)**	**13.927**	**76**	**1.81**	**1.8**	**3.578**	**6.56**	**44.72**	**45.2**
	Equiv. Refl.: (4,-9,-7), (-9,4,7), (-9,5,-7)								
**Quartz**	**(5,4,-7)**	**13.927**	**76**	**1.809**	**1.8**	**3.578**	**6.56**	**44.72**	**45.2**
	Equiv. Refl.: (5,-9,7)								
**Quartz**	**(2,8,2)**	**13.549**	**70.73**	**1.784**	**1.8**	**5.017**	**8.83**	**62.71**	**63.33**
	Equiv. Refl.: (2,-10,-2)								
**Quartz**	**(8,2,-2)**	**13.549**	**70.73**	**1.783**	**1.8**	**5.017**	**8.83**	**62.71**	**63.33**
	Equiv. Refl.: (8,-10,2), (-10,2,2), (-10,8,-2)								
**Quartz**	**(9,-5,7)**	**13.927**	**76**	**1.78**	**1.8**	**3.578**	**6.56**	**44.72**	**45.2**
**Quartz**	**(9,-4,-7)**	**13.927**	**76**	**1.779**	**1.8**	**3.578**	**6.56**	**44.72**	**45.2**
	Equiv. Refl.: (-4,9,7), (-4,-5,-7), (-5,9,-7), (-5,-4,7)								
**Quartz**	**(-8,9,6)**	**14.224**	**82.32**	**1.778**	**2**	**1.936**	**3.84**	**24.2**	**24.5**
	Equiv. Refl.: (-8,-1,-6)								
**Quartz**	**(9,-1,6)**	**14.224**	**82.32**	**1.777**	**2**	**1.936**	**3.84**	**24.2**	**24.5**
	Equiv. Refl.: (9,-8,-6)								
**Quartz**	**(-1,9,-6)**	**14.224**	**82.32**	**1.776**	**2**	**1.936**	**3.84**	**24.2**	**24.5**
	Equiv. Refl.: (-1,-8,6)								
**Quartz**	**(-3,-1,11)**	**13.667**	**72.22**	**1.77**	**1.8**	**4.604**	**8.14**	**57.55**	**58.12**
**Quartz**	**(4,-1,-11)**	**13.667**	**72.22**	**1.769**	**1.8**	**4.604**	**8.14**	**57.55**	**58.12**
	Equiv. Refl.: (4,-3,11), (-3,4,-11)								
**Quartz**	**(-1,4,11)**	**13.667**	**72.22**	**1.768**	**1.8**	**4.604**	**8.13**	**57.55**	**58.12**
	Equiv. Refl.: (-1,-3,-11)								

Ei =	14.353	keV						ΔEg	ΔEt
Cryst	Refl	EB	ΘB	∫ IR dΘ	Width	Ei cotΘ	ΔE	2m, 50µm	2m, 50µm
	(h,k,l)	[keV]	[°]	[µrad]	[µrad]	[meV/µrad]	[meV]	[meV]	[meV]
**LiNbO3**	**(2,7,19)**	**14.203**	**81.71**	**1.737**	**2.6**	**2.09**	**5.44**	**26.13**	**26.69**
	Equiv. Refl.: (2,-9,-19), (7,2,-19), (7,-9,19), (9,-2,-19), (9,-7,19), (-2,9,19), (-2,-7,-19), (-7,9,-19), (-7,-2,19) (-9,2,19), (-9,7,-19)								
**Quartz**	**(3,1,-11)**	**13.667**	**72.22**	**1.733**	**1.8**	**4.604**	**8.14**	**57.55**	**58.12**
	Equiv. Refl.: (3,-4,11), (-4,1,11), (-4,3,-11)								
**Quartz**	**(1,3,11)**	**13.667**	**72.22**	**1.732**	**1.8**	**4.604**	**8.13**	**57.55**	**58.12**
**Quartz**	**(1,-4,-11)**	**13.667**	**72.22**	**1.731**	**1.8**	**4.604**	**8.13**	**57.55**	**58.12**
**Quartz**	**(10,-2,-2)**	**13.549**	**70.73**	**1.718**	**1.8**	**5.017**	**8.83**	**62.71**	**63.33**
	Equiv. Refl.: (10,-8,2), (-2,10,2), (-2,-8,-2), (-8,-2,2)								
**Quartz**	**(-8,10,-2)**	**13.549**	**70.73**	**1.717**	**1.8**	**5.017**	**8.83**	**62.71**	**63.33**
**Sapph**	**(4,6,-8)**	**13.66**	**72.12**	**1.648**	**1.7**	**4.63**	**7.89**	**57.88**	**58.41**
	Equiv. Refl.: (4,-10,8), (6,4,8), (6,-10,-8), (10,-4,8), (10,-6,-8), (-4,10,-8), (-4,-6,8), (-6,10,8), (-6,-4,-8) (-10,4,-8), (-10,6,8)								
**Quartz**	**(3,6,-7)**	**14.078**	**78.78**	**1.646**	**1.7**	**2.848**	**4.81**	**35.6**	**35.93**
	Equiv. Refl.: (3,-9,7)								
**Quartz**	**(6,3,7)**	**14.078**	**78.78**	**1.644**	**1.7**	**2.848**	**4.8**	**35.6**	**35.92**
	Equiv. Refl.: (6,-9,-7), (-9,3,-7), (-9,6,7)								
**Quartz**	**(-9,2,-6)**	**13.769**	**73.6**	**1.64**	**1.7**	**4.224**	**7.01**	**52.79**	**53.26**
	Equiv. Refl.: (-9,7,6)								
**Quartz**	**(2,7,-6)**	**13.769**	**73.6**	**1.639**	**1.7**	**4.224**	**7**	**52.79**	**53.26**
	Equiv. Refl.: (2,-9,6), (7,2,6), (7,-9,-6)								
**Quartz**	**(9,-2,6)**	**13.769**	**73.6**	**1.616**	**1.7**	**4.224**	**7.01**	**52.79**	**53.26**
	Equiv. Refl.: (9,-7,-6)								
**Quartz**	**(-2,9,-6)**	**13.769**	**73.6**	**1.615**	**1.7**	**4.224**	**7**	**52.79**	**53.26**
	Equiv. Refl.: (-2,-7,6), (-7,9,6), (-7,-2,-6)								
**Quartz**	**(-6,3,-10)**	**13.743**	**73.24**	**1.606**	**1.6**	**4.322**	**7**	**54.03**	**54.48**
**Quartz**	**(-6,3,10)**	**13.743**	**73.24**	**1.605**	**1.6**	**4.322**	**7**	**54.03**	**54.48**
**Quartz**	**(3,3,10)**	**13.743**	**73.24**	**1.604**	**1.6**	**4.322**	**7**	**54.03**	**54.48**
	Equiv. Refl.: (3,-6,-10), (9,-3,-7), (-3,9,7), (-6,9,-7), (-6,-3,7)								
**Quartz**	**(3,3,-10)**	**13.743**	**73.24**	**1.603**	**1.6**	**4.322**	**6.99**	**54.03**	**54.48**
	Equiv. Refl.: (3,-6,10), (9,-6,7), (-3,-6,-7)								
**Quartz**	**(3,6,7)**	**14.078**	**78.78**	**1.588**	**1.7**	**2.848**	**4.77**	**35.6**	**35.92**
	Equiv. Refl.: (3,-9,-7), (6,3,-7), (6,-9,7), (-9,3,7), (-9,6,-7)								
**Quartz**	**(5,6,-1)**	**13.946**	**76.32**	**1.583**	**1.6**	**3.493**	**5.69**	**43.66**	**44.03**
	Equiv. Refl.: (5,-11,1), (6,5,1), (6,-11,-1), (-11,5,-1), (-11,6,1)								
**Quartz**	**(6,-3,10)**	**13.743**	**73.24**	**1.574**	**1.6**	**4.322**	**7**	**54.03**	**54.48**
**Quartz**	**(6,-3,-10)**	**13.743**	**73.24**	**1.573**	**1.6**	**4.322**	**7**	**54.03**	**54.48**
**Quartz**	**(-3,6,10)**	**13.743**	**73.24**	**1.572**	**1.6**	**4.322**	**7**	**54.03**	**54.48**
	Equiv. Refl.: (-3,-3,-10)								
**Quartz**	**(-3,6,-10)**	**13.743**	**73.24**	**1.571**	**1.6**	**4.322**	**6.99**	**54.03**	**54.48**
	Equiv. Refl.: (-3,9,-7), (-3,-3,10), (-3,-6,7)								
**Quartz**	**(-6,-3,-7)**	**14.078**	**78.78**	**1.57**	**1.7**	**2.848**	**4.8**	**35.6**	**35.92**
**Quartz**	**(9,-3,7)**	**14.078**	**78.78**	**1.569**	**1.7**	**2.848**	**4.8**	**35.6**	**35.92**
	Equiv. Refl.: (9,-6,-7), (11,-5,1), (11,-6,-1), (-5,11,-1), (-5,-6,1), (-6,9,7), (-6,-5,-1)								
**Quartz**	**(-6,11,1)**	**13.946**	**76.32**	**1.568**	**1.6**	**3.493**	**5.69**	**43.66**	**44.03**
**Sapph**	**(2,6,-19)**	**14.14**	**80.11**	**1.55**	**1.7**	**2.503**	**4.27**	**31.29**	**31.58**
	Equiv. Refl.: (2,-8,19), (6,2,19), (6,-8,-19), (8,-2,19), (8,-6,-19), (-2,8,-19), (-2,-6,19), (-6,8,19), (-6,-2,-19) (-8,2,-19), (-8,6,19)								
**Sapph**	**(2,8,0)**	**13.788**	**73.87**	**1.468**	**1.5**	**4.15**	**6.41**	**51.87**	**52.27**
	Equiv. Refl.: (2,-10,0), (8,2,0), (8,-10,0), (10,-2,0), (10,-8,0), (-2,10,0), (-2,-8,0), (-8,10,0), (-8,-2,0) (-10,2,0), (-10,8,0)								

Ei =	14.353	keV						ΔEg	ΔEt
Cryst	Refl (h,k,l)	EB [keV]	ΘB [°]	∫ IR dΘ [µrad]	Width [µrad]	Ei cotΘ [meV/µrad]	ΔE [meV]	2m, 50µm [meV]	2m, 50µm [meV]
**Sapph**	**(2,2,27)**	**13.901**	**75.58**	**1.463**	**1.6**	**3.691**	**5.74**	**46.14**	**46.49**
	Equiv. Refl.: (2,2,-27), (2,-4,27), (2,-4,-27), (4,-2,27), (4,-2,-27), (-2,4,27), (-2,4,-27), (-2,-2,27), (-2,-2,-27) (-4,2,27), (-4,2,-27)								
**Quartz**	**(6,-10,4)**	**13.505**	**70.21**	**1.444**	**1.5**	**5.166**	**7.54**	**64.57**	**65.01**
	Equiv. Refl.: (-10,4,4), (-10,6,-4)								
**Quartz**	**(4,6,4)**	**13.505**	**70.21**	**1.443**	**1.5**	**5.166**	**7.54**	**64.57**	**65.01**
	Equiv. Refl.: (4,-10,-4), (6,4,-4), (-1,5,-11)								
**Quartz**	**(-1,-4,11)**	**14.275**	**84.02**	**1.442**	**1.6**	**1.503**	**2.47**	**18.79**	**18.96**
	Equiv. Refl.: (-4,5,11)								
**Quartz**	**(-4,-1,-11)**	**14.275**	**84.02**	**1.441**	**1.6**	**1.503**	**2.47**	**18.79**	**18.96**
**Quartz**	**(5,-1,11)**	**14.275**	**84.02**	**1.434**	**1.6**	**1.503**	**2.46**	**18.79**	**18.95**
	Equiv. Refl.: (5,-4,-11)								
**Quartz**	**(10,-4,-4)**	**13.505**	**70.21**	**1.43**	**1.5**	**5.166**	**7.55**	**64.57**	**65.01**
	Equiv. Refl.: (10,-6,4), (-4,10,4), (-6,10,-4), (-6,-4,4)								
**Quartz**	**(-4,-6,-4)**	**13.505**	**70.21**	**1.429**	**1.5**	**5.166**	**7.54**	**64.57**	**65.01**
**Quartz**	**(1,-5,11)**	**14.275**	**84.02**	**1.421**	**1.6**	**1.503**	**2.47**	**18.79**	**18.96**
	Equiv. Refl.: (11,-4,-1), (11,-7,1), (-4,11,1), (-4,-7,-1), (-7,11,-1), (-7,-4,1)								
**Quartz**	**(1,4,-11)**	**14.275**	**84.02**	**1.42**	**1.6**	**1.503**	**2.47**	**18.79**	**18.96**
	Equiv. Refl.: (4,-5,-11)								
**Quartz**	**(4,1,11)**	**14.275**	**84.02**	**1.419**	**1.6**	**1.503**	**2.47**	**18.79**	**18.96**
**Sapph**	**(1,8,11)**	**13.885**	**75.32**	**1.413**	**1.5**	**3.759**	**5.66**	**46.99**	**47.33**
	Equiv. Refl.: (1,-9,-11), (8,1,-11), (8,-9,11), (9,-1,-11), (9,-8,11), (-1,9,11), (-1,-8,-11), (-8,9,-11), (-8,-1,11) (-9,1,11), (-9,8,-11)								
**Quartz**	**(-5,1,-11)**	**14.275**	**84.02**	**1.412**	**1.6**	**1.503**	**2.46**	**18.79**	**18.95**
	Equiv. Refl.: (-5,4,11)								
**LiNbO3**	**(1,9,-11)**	**14.147**	**80.28**	**1.409**	**2.1**	**2.458**	**5.28**	**30.72**	**31.17**
	Equiv. Refl.: (1,-10,11), (9,1,11), (9,-10,-11), (10,-1,11), (10,-9,-11), (-1,10,-11), (-1,-9,11), (-9,10,11), (-9,-1,-11) (-10,1,-11), (-10,9,11)								
**Quartz**	**(7,0,8)**	**13.719**	**72.91**	**1.396**	**1.4**	**4.413**	**6.33**	**55.16**	**55.52**
	Equiv. Refl.: (7,-7,-8)								
**Quartz**	**(-7,0,-8)**	**13.719**	**72.91**	**1.395**	**1.4**	**4.413**	**6.33**	**55.16**	**55.52**
	Equiv. Refl.: (-7,7,8)								
**Quartz**	**(0,7,-8)**	**13.719**	**72.91**	**1.394**	**1.4**	**4.413**	**6.32**	**55.16**	**55.52**
	Equiv. Refl.: (0,-7,8)								
**Quartz**	**(3,-5,-11)**	**14.125**	**79.78**	**1.377**	**1.4**	**2.587**	**3.75**	**32.34**	**32.55**
**Quartz**	**(3,2,11)**	**14.125**	**79.78**	**1.376**	**1.4**	**2.587**	**3.75**	**32.34**	**32.55**
**Quartz**	**(-5,2,-11)**	**14.125**	**79.78**	**1.375**	**1.4**	**2.587**	**3.74**	**32.34**	**32.55**
	Equiv. Refl.: (-5,3,11)								
**Quartz**	**(2,3,-11)**	**14.125**	**79.78**	**1.374**	**1.4**	**2.587**	**3.74**	**32.34**	**32.55**
	Equiv. Refl.: (2,-5,11)								
**Quartz**	**(5,6,-2)**	**14.087**	**78.95**	**1.346**	**1.4**	**2.803**	**3.92**	**35.04**	**35.26**
	Equiv. Refl.: (5,-11,2), (6,5,2), (6,-11,-2), (-11,5,-2), (-11,6,2)								
**Quartz**	**(-2,-6,-8)**	**13.949**	**76.38**	**1.325**	**1.4**	**3.477**	**4.74**	**43.47**	**43.72**
**Quartz**	**(8,-2,-8)**	**13.949**	**76.38**	**1.324**	**1.4**	**3.477**	**4.74**	**43.47**	**43.72**
	Equiv. Refl.: (8,-6,8), (-2,8,8), (-6,8,-8), (-6,-2,8)								
**Quartz**	**(10,-1,0)**	**13.899**	**75.55**	**1.318**	**1.3**	**3.699**	**4.87**	**46.24**	**46.5**
	Equiv. Refl.: (10,-9,0), (-1,10,0), (-1,-9,0), (-9,10,0), (-9,-1,0)								
**Quartz**	**(-1,2,-12)**	**13.994**	**77.15**	**1.315**	**1.4**	**3.274**	**4.43**	**40.92**	**41.16**
	Equiv. Refl.: (-1,-1,12)								
**Quartz**	**(2,-1,-12)**	**13.994**	**77.15**	**1.314**	**1.4**	**3.274**	**4.43**	**40.92**	**41.16**

Ei = Cryst	14.353 Refl (h,k,l)	keV EB [keV]	ΘB [°]	∫ IR dΘ [µrad]	Width [µrad]	Ei cotΘ [meV/µrad]	ΔE [meV]	ΔEg 2m, 50µm [meV]	ΔEt 2m, 50µm [meV]
**Quartz**	**(2,-1,12)**	**13.994**	**77.15**	**1.313**	**1.4**	**3.274**	**4.42**	**40.92**	**41.16**
	Equiv. Refl.: (-1,2,12)								
**Quartz**	**(-1,-1,-12)**	**13.994**	**77.15**	**1.312**	**1.4**	**3.274**	**4.42**	**40.92**	**41.16**
**Quartz**	**(-3,5,11)**	**14.125**	**79.78**	**1.31**	**1.4**	**2.587**	**3.75**	**32.34**	**32.55**
**Quartz**	**(-3,-2,-11)**	**14.125**	**79.78**	**1.309**	**1.4**	**2.587**	**3.75**	**32.34**	**32.55**
**Quartz**	**(5,-2,11)**	**14.125**	**79.78**	**1.308**	**1.4**	**2.587**	**3.74**	**32.34**	**32.55**
	Equiv. Refl.: (5,-3,-11)								
**Quartz**	**(-2,5,-11)**	**14.125**	**79.78**	**1.307**	**1.4**	**2.587**	**3.74**	**32.34**	**32.55**
	Equiv. Refl.: (-2,-3,11)								
**Quartz**	**(9,-10,-2)**	**14.087**	**78.95**	**1.29**	**1.4**	**2.803**	**3.83**	**35.04**	**35.25**
	Equiv. Refl.: (-10,1,-2), (-10,9,2)								
**Quartz**	**(1,9,-2)**	**14.087**	**78.95**	**1.289**	**1.4**	**2.803**	**3.83**	**35.04**	**35.25**
	Equiv. Refl.: (1,-10,2), (9,1,2)								
**Quartz**	**(2,6,8)**	**13.949**	**76.38**	**1.265**	**1.4**	**3.477**	**4.74**	**43.47**	**43.72**
	Equiv. Refl.: (2,-8,-8), (6,2,-8), (-8,2,8)								
**Quartz**	**(6,-8,8)**	**13.949**	**76.38**	**1.264**	**1.4**	**3.477**	**4.74**	**43.47**	**43.72**
	Equiv. Refl.: (-8,6,-8)								
**Quartz**	**(11,-5,2)**	**14.087**	**78.95**	**1.26**	**1.4**	**2.803**	**3.92**	**35.04**	**35.26**
	Equiv. Refl.: (11,-6,-2), (-5,11,-2), (-5,-6,2), (-6,11,2), (-6,-5,-2)								
**Quartz**	**(5,6,0)**	**13.899**	**75.55**	**1.251**	**1.3**	**3.699**	**4.86**	**46.24**	**46.5**
	Equiv. Refl.: (5,-11,0), (6,5,0), (6,-11,0), (-11,5,0), (-11,6,0)								
**Quartz**	**(11,-5,0)**	**13.899**	**75.55**	**1.25**	**1.3**	**3.699**	**4.86**	**46.24**	**46.5**
	Equiv. Refl.: (11,-6,0), (-5,11,0), (-5,-6,0), (-6,11,0), (-6,-5,0)								
**Quartz**	**(10,-1,2)**	**14.087**	**78.95**	**1.247**	**1.4**	**2.803**	**3.83**	**35.04**	**35.25**
	Equiv. Refl.: (10,-9,-2), (-1,10,-2), (-1,-9,2), (-9,10,2), (-9,-1,-2)								
**Quartz**	**(1,1,-12)**	**13.994**	**77.15**	**1.237**	**1.4**	**3.274**	**4.43**	**40.92**	**41.16**
	Equiv. Refl.: (1,-2,12)								
**Quartz**	**(-2,1,12)**	**13.994**	**77.15**	**1.236**	**1.4**	**3.274**	**4.43**	**40.92**	**41.16**
**Quartz**	**(-2,1,-12)**	**13.994**	**77.15**	**1.235**	**1.4**	**3.274**	**4.42**	**40.92**	**41.16**
**Quartz**	**(1,1,12)**	**13.994**	**77.15**	**1.234**	**1.4**	**3.274**	**4.42**	**40.92**	**41.16**
	Equiv. Refl.: (1,-2,-12)								
**Quartz**	**(-4,1,-11)**	**13.667**	**72.22**	**1.224**	**1.2**	**4.604**	**5.65**	**57.55**	**57.82**
	Equiv. Refl.: (-4,3,11)								
**Quartz**	**(3,-4,-11)**	**13.667**	**72.22**	**1.223**	**1.2**	**4.604**	**5.64**	**57.55**	**57.82**
**Quartz**	**(3,1,11)**	**13.667**	**72.22**	**1.222**	**1.2**	**4.604**	**5.64**	**57.55**	**57.82**
**Quartz**	**(1,3,-11)**	**13.667**	**72.22**	**1.221**	**1.2**	**4.604**	**5.63**	**57.55**	**57.82**
	Equiv. Refl.: (1,-4,11)								
**Quartz**	**(4,7,1)**	**14.097**	**79.17**	**1.22**	**1.4**	**2.746**	**3.89**	**34.32**	**34.54**
	Equiv. Refl.: (4,-11,-1), (7,4,-1), (7,-11,1), (-11,4,1), (-11,7,-1)								
**Sapph**	**(1,2,-28)**	**13.944**	**76.29**	**1.215**	**1.3**	**3.5**	**4.64**	**43.75**	**44**
	Equiv. Refl.: (1,-3,28), (2,1,28), (2,-3,-28), (3,-1,28), (3,-2,-28), (-1,3,-28), (-1,-2,28), (-2,3,28), (-2,-1,-28) (-3,1,-28), (-3,2,28)								
**Quartz**	**(1,9,0)**	**13.899**	**75.55**	**1.193**	**1.3**	**3.699**	**4.87**	**46.24**	**46.5**
	Equiv. Refl.: (1,-10,0), (9,1,0), (9,-10,0), (-10,1,0), (-10,9,0)								
**Quartz**	**(4,-1,11)**	**13.667**	**72.22**	**1.139**	**1.2**	**4.604**	**5.65**	**57.55**	**57.82**
**Quartz**	**(4,-3,-11)**	**13.667**	**72.22**	**1.138**	**1.2**	**4.604**	**5.65**	**57.55**	**57.82**
**Quartz**	**(-3,4,11)**	**13.667**	**72.22**	**1.137**	**1.2**	**4.604**	**5.64**	**57.55**	**57.82**
	Equiv. Refl.: (-3,-1,-11)								
**Quartz**	**(-1,-3,11)**	**13.667**	**72.22**	**1.136**	**1.2**	**4.604**	**5.63**	**57.55**	**57.82**
**Quartz**	**(-1,4,-11)**	**13.667**	**72.22**	**1.135**	**1.2**	**4.604**	**5.63**	**57.55**	**57.82**

Ei = Cryst	14.353 Refl (h,k,l)	keV EB [keV]	ΘB [°]	∫ IR dΘ [μrad]	Width [μrad]	Ei cotΘ [meV/μrad]	ΔE [meV]	ΔEg 2m, 50μm [meV]	ΔEt 2m, 50μm [meV]
**Quartz**	**(0,9,5)**	**14.312**	**85.68**	**1.125**	**1.4**	**1.085**	**1.56**	**13.56**	**13.65**
	Equiv. Refl.: (0,-9,-5)								
**Quartz**	**(9,0,-5)**	**14.312**	**85.68**	**1.121**	**1.4**	**1.085**	**1.56**	**13.56**	**13.65**
	Equiv. Refl.: (9,-9,5), (-9,0,5), (-9,9,-5)								
**Sapph**	**(1,7,18)**	**14.241**	**82.85**	**1.102**	**1.3**	**1.801**	**2.43**	**22.51**	**22.64**
	Equiv. Refl.: (1,7,-18), (1,-8,18), (1,-8,-18), (7,1,18), (7,1,-18), (7,-8,18), (7,-8,-18), (8,-1,18), (8,-1,-18) (8,-7,18), (8,-7,-18), (-1,8,18), (-1,8,-18), (-1,-7,18), (-1,-7,-18), (-7,8,18), (-7,8,-18), (-7,-1,18) (-7,-1,-18), (-8,1,18), (-8,1,-18), (-8,7,18), (-8,7,-18)								
**Quartz**	**(4,2,-10)**	**13.82**	**74.34**	**1.099**	**1.1**	**4.023**	**4.55**	**50.29**	**50.49**
	Equiv. Refl.: (4,-6,10)								
**Quartz**	**(2,-6,-10)**	**13.82**	**74.34**	**1.097**	**1.1**	**4.023**	**4.54**	**50.29**	**50.49**
	Equiv. Refl.: (-6,2,10), (-6,4,-10)								
**Quartz**	**(2,4,10)**	**13.82**	**74.34**	**1.096**	**1.1**	**4.023**	**4.53**	**50.29**	**50.49**
**Sapph**	**(1,8,-10)**	**13.711**	**72.8**	**1.091**	**1.2**	**4.442**	**5.22**	**55.53**	**55.77**
	Equiv. Refl.: (1,-9,10), (8,1,10), (8,-9,-10), (9,-1,10), (9,-8,-10), (-1,9,-10), (-1,-8,10), (-8,9,10), (-8,-1,-10) (-9,1,-10), (-9,8,10)								
**Quartz**	**(10,-2,2)**	**13.549**	**70.73**	**1.084**	**1.1**	**5.017**	**5.64**	**62.71**	**62.97**
	Equiv. Refl.: (10,-8,-2), (-2,10,-2), (-2,-8,2), (-8,10,2), (-8,-2,-2)								
**Quartz**	**(2,8,-2)**	**13.549**	**70.73**	**1.076**	**1.1**	**5.017**	**5.64**	**62.71**	**62.97**
	Equiv. Refl.: (2,-10,2), (8,2,2), (8,-10,-2), (-10,2,-2), (-10,8,2)								
**LiNbO3**	**(2,9,5)**	**14.288**	**84.54**	**1.064**	**2.2**	**1.371**	**2.99**	**17.14**	**17.4**
	Equiv. Refl.: (2,-11,-5), (9,2,-5), (9,-11,5), (11,-2,-5), (11,-9,5), (-2,11,5), (-2,-9,-5), (-9,11,-5), (-9,-2,5) (-11,2,5), (-11,9,-5)								
**Sapph**	**(3,4,-22)**	**13.928**	**76.03**	**1.057**	**1.2**	**3.571**	**4.19**	**44.64**	**44.83**
	Equiv. Refl.: (3,-7,22), (4,3,22), (4,-7,-22), (7,-3,22), (7,-4,-22), (-3,7,-22), (-3,-4,22), (-4,7,22), (-4,-3,-22) (-7,3,-22), (-7,4,22)								
**LiNbO3**	**(1,6,-23)**	**13.745**	**73.26**	**1.044**	**1.5**	**4.317**	**6.46**	**53.96**	**54.34**
	Equiv. Refl.: (1,-7,23), (6,1,23), (6,-7,-23), (7,-1,23), (7,-6,-23), (-1,7,-23), (-1,-6,23), (-6,7,23), (-6,-1,-23) (-7,1,-23), (-7,6,23)								
**Quartz**	**(-4,6,-10)**	**13.82**	**74.34**	**1.033**	**1.1**	**4.023**	**4.55**	**50.29**	**50.49**
	Equiv. Refl.: (-4,-2,10)								
**Quartz**	**(6,-2,-10)**	**13.82**	**74.34**	**1.031**	**1.1**	**4.023**	**4.54**	**50.29**	**50.49**
	Equiv. Refl.: (6,-4,10), (-2,6,10), (-2,-4,-10)								
**Quartz**	**(-7,10,5)**	**14.163**	**80.67**	**0.998**	**1.1**	**2.358**	**2.6**	**29.48**	**29.59**
	Equiv. Refl.: (-7,-3,-5)								
**Quartz**	**(10,-3,5)**	**14.163**	**80.67**	**0.997**	**1.1**	**2.358**	**2.6**	**29.48**	**29.59**
	Equiv. Refl.: (10,-7,-5), (-3,10,-5), (-3,-7,5)								
**Quartz**	**(4,5,-7)**	**13.927**	**76**	**0.995**	**1.1**	**3.578**	**3.82**	**44.72**	**44.88**
	Equiv. Refl.: (4,-9,7)								
**Quartz**	**(5,4,7)**	**13.927**	**76**	**0.994**	**1.1**	**3.578**	**3.82**	**44.72**	**44.88**
	Equiv. Refl.: (5,-9,-7), (-9,4,-7)								
**Quartz**	**(-9,5,7)**	**13.927**	**76**	**0.993**	**1.1**	**3.578**	**3.82**	**44.72**	**44.88**
**Quartz**	**(1,8,5)**	**13.706**	**72.73**	**0.989**	**1**	**4.462**	**4.66**	**55.77**	**55.97**
	Equiv. Refl.: (1,-9,-5), (-9,1,5), (-9,8,-5)								
**Quartz**	**(8,1,-5)**	**13.706**	**72.73**	**0.988**	**1**	**4.462**	**4.66**	**55.77**	**55.96**
	Equiv. Refl.: (8,-9,5)								
**Quartz**	**(9,-8,5)**	**13.706**	**72.73**	**0.984**	**1**	**4.462**	**4.66**	**55.77**	**55.97**
	Equiv. Refl.: (-1,9,5), (-1,-8,-5)								
**Quartz**	**(9,-1,-5)**	**13.706**	**72.73**	**0.983**	**1**	**4.462**	**4.66**	**55.77**	**55.97**
	Equiv. Refl.: (-8,9,-5), (-8,-1,5)								

Ei =	14.353	keV						ΔEg	ΔEt
**Cryst**	**Refl (h,k,l)**	**EB [keV]**	**ΘB [°]**	**∫ IR dΘ [μrad]**	**Width [μrad]**	**Ei cotΘ [meV/μrad]**	**ΔE [meV]**	**2m, 50μm [meV]**	**2m, 50μm [meV]**
**Quartz**	**(-4,9,-7)**	**13.927**	**76**	**0.981**	**1.1**	**3.578**	**3.82**	**44.72**	**44.88**
	Equiv. Refl.: (-4,-5,7), (-5,9,7), (-5,-4,-7)								
**Quartz**	**(9,-4,7)**	**13.927**	**76**	**0.98**	**1.1**	**3.578**	**3.82**	**44.72**	**44.88**
	Equiv. Refl.: (9,-5,-7)								
**Quartz**	**(4,6,5)**	**13.936**	**76.16**	**0.977**	**1**	**3.535**	**3.7**	**44.19**	**44.35**
**Quartz**	**(4,-10,-5)**	**13.936**	**76.16**	**0.976**	**1**	**3.535**	**3.7**	**44.19**	**44.35**
	Equiv. Refl.: (6,4,-5), (6,-10,5), (-10,4,5), (-10,6,-5)								
**Quartz**	**(-10,2,4)**	**14.12**	**79.66**	**0.97**	**1.1**	**2.62**	**2.79**	**32.75**	**32.87**
	Equiv. Refl.: (-10,8,-4)								
**Quartz**	**(2,8,4)**	**14.12**	**79.66**	**0.969**	**1.1**	**2.62**	**2.79**	**32.75**	**32.87**
	Equiv. Refl.: (2,-10,-4), (8,-10,4)								
**Quartz**	**(8,2,-4)**	**14.12**	**79.66**	**0.968**	**1.1**	**2.62**	**2.79**	**32.75**	**32.87**
**Quartz**	**(10,-4,-5)**	**13.936**	**76.16**	**0.951**	**1**	**3.535**	**3.69**	**44.19**	**44.35**
	Equiv. Refl.: (10,-6,5), (-4,10,5), (-4,-6,-5)								
**Quartz**	**(-6,10,-5)**	**13.936**	**76.16**	**0.95**	**1**	**3.535**	**3.69**	**44.19**	**44.35**
	Equiv. Refl.: (-6,-4,5)								
**Quartz**	**(7,3,5)**	**14.163**	**80.67**	**0.935**	**1.1**	**2.358**	**2.6**	**29.48**	**29.59**
	Equiv. Refl.: (7,-10,-5), (-10,3,-5), (-10,7,5)								
**Quartz**	**(3,7,-5)**	**14.163**	**80.67**	**0.934**	**1.1**	**2.358**	**2.6**	**29.48**	**29.59**
	Equiv. Refl.: (3,-10,5)								
**Quartz**	**(10,-2,-4)**	**14.12**	**79.66**	**0.917**	**1.1**	**2.62**	**2.79**	**32.75**	**32.87**
	Equiv. Refl.: (10,-8,4)								
**Quartz**	**(-2,10,4)**	**14.12**	**79.66**	**0.916**	**1.1**	**2.62**	**2.79**	**32.75**	**32.87**
	Equiv. Refl.: (-2,-8,-4)								
**Quartz**	**(-8,10,-4)**	**14.12**	**79.66**	**0.915**	**1.1**	**2.62**	**2.79**	**32.75**	**32.87**
	Equiv. Refl.: (-8,-2,4)								
**LiNbO3**	**(2,7,-17)**	**13.687**	**72.48**	**0.91**	**1.3**	**4.532**	**6.08**	**56.65**	**56.98**
	Equiv. Refl.: (2,-9,17), (7,2,17), (7,-9,-17), (9,-2,17), (9,-7,-17), (-2,9,-17), (-2,-7,17), (-7,9,17), (-7,-2,-17) (-9,2,-17), (-9,7,17), (2,6,17), (2,-8,-17), (6,2,-17), (6,-8,17), (8,-2,-17), (8,-6,17), (-2,8,17) (-2,-6,-17), (-6,8,-17), (-6,-2,17), (-8,2,17), (-8,6,-17)								
**Sapph**	**(1,5,23)**	**13.808**	**74.17**	**0.902**	**1**	**4.071**	**4.1**	**50.88**	**51.05**
	Equiv. Refl.: (1,-6,-23), (5,1,-23), (5,-6,23), (6,-1,-23), (6,-5,23), (-1,6,23), (-1,-5,-23), (-5,6,-23), (-5,-1,23) (-6,1,23), (-6,5,-23)								
**Sapph**	**(2,5,21)**	**13.738**	**73.17**	**0.886**	**1**	**4.343**	**4.27**	**54.28**	**54.45**
	Equiv. Refl.: (2,5,-21), (2,-7,21), (2,-7,-21), (5,2,21), (5,2,-21), (5,-7,21), (5,-7,-21), (7,-2,21), (7,-2,-21) (7,-5,21), (7,-5,-21), (-2,7,21), (-2,7,-21), (-2,-5,21), (-2,-5,-21), (-5,7,21), (-5,7,-21), (-5,-2,21) (-5,-2,-21), (-7,2,21), (-7,2,-21), (-7,5,21), (-7,5,-21)								
**Quartz**	**(10,-3,-4)**	**13.739**	**73.18**	**0.855**	**0.9**	**4.34**	**3.97**	**54.25**	**54.39**
	Equiv. Refl.: (10,-7,4), (-3,10,4), (-3,-7,-4), (-7,10,-4), (-7,-3,4)								
**Sapph**	**(0,2,28)**	**13.699**	**72.63**	**0.841**	**0.9**	**4.489**	**4.2**	**56.11**	**56.27**
	Equiv. Refl.: (0,-2,-28), (2,0,-28), (2,-2,28), (-2,0,28), (-2,2,-28)								
**Quartz**	**(3,7,4)**	**13.739**	**73.18**	**0.838**	**0.9**	**4.34**	**3.97**	**54.25**	**54.39**
	Equiv. Refl.: (3,-10,-4), (7,3,-4), (7,-10,4), (-10,3,4), (-10,7,-4)								
**LiNbO3**	**(1,9,7)**	**13.629**	**71.72**	**0.831**	**1.2**	**4.741**	**5.91**	**59.26**	**59.56**
	Equiv. Refl.: (1,-10,-7), (9,1,-7), (9,-10,7), (10,-1,-7), (10,-9,7), (-1,10,7), (-1,-9,-7), (-9,10,-7), (-9,-1,7) (-10,1,7), (-10,9,-7)								
**Quartz**	**(11,-4,0)**	**14.051**	**78.22**	**0.817**	**0.9**	**2.993**	**2.69**	**37.42**	**37.52**
	Equiv. Refl.: (11,-7,0), (-4,11,0), (-4,-7,0), (-7,11,0), (-7,-4,0), (-8,10,4), (-8,-2,-4)								
**Quartz**	**(10,-2,4)**	**14.12**	**79.66**	**0.816**	**0.9**	**2.62**	**2.23**	**32.75**	**32.82**
	Equiv. Refl.: (10,-8,-4), (-2,10,-4)								

Ei =	14.353	keV						ΔEg	ΔEt
**Cryst**	**Refl**	**EB**	**ΘB**	**∫ IR dΘ**	**Width**	**Ei cotΘ**	**ΔE**	**2m, 50µm**	**2m, 50µm**
	**(h,k,l)**	**[keV]**	**[°]**	**[µrad]**	**[µrad]**	**[meV/µrad]**	**[meV]**	**[meV]**	**[meV]**
**Quartz**	**(-2,-8,4)**	**14.12**	**79.66**	**0.815**	**0.9**	**2.62**	**2.23**	**32.75**	**32.82**
**Quartz**	**(3,7,5)**	**14.163**	**80.67**	**0.806**	**0.9**	**2.358**	**2.15**	**29.48**	**29.56**
	Equiv. Refl.: (3,-10,-5), (-10,7,-5)								
**Quartz**	**(7,3,-5)**	**14.163**	**80.67**	**0.805**	**0.9**	**2.358**	**2.15**	**29.48**	**29.56**
	Equiv. Refl.: (7,-10,5), (-10,3,5)								
**Sapph**	**(0,9,6)**	**13.839**	**74.62**	**0.788**	**0.9**	**3.947**	**3.55**	**49.33**	**49.46**
	Equiv. Refl.: (0,9,-6), (0,-9,6), (0,-9,-6), (9,0,6), (9,0,-6), (9,-9,6), (9,-9,-6), (-9,0,6), (-9,0,-6) (-9,9,6), (-9,9,-6)								
**Quartz**	**(4,7,0)**	**14.051**	**78.22**	**0.764**	**0.9**	**2.993**	**2.69**	**37.42**	**37.52**
	Equiv. Refl.: (4,-11,0), (7,4,0), (7,-11,0), (-11,4,0), (-11,7,0)								
**Quartz**	**(1,9,-1)**	**13.946**	**76.32**	**0.747**	**0.8**	**3.493**	**2.89**	**43.66**	**43.76**
	Equiv. Refl.: (1,-10,1), (9,1,1), (9,-10,-1), (-10,1,-1), (-10,9,1)								
**Quartz**	**(10,-3,-5)**	**14.163**	**80.67**	**0.733**	**0.9**	**2.358**	**2.15**	**29.48**	**29.56**
	Equiv. Refl.: (10,-7,5), (-3,10,5), (-3,-7,-5), (-7,10,-5), (-7,-3,5)								
**Quartz**	**(10,-2,-3)**	**13.79**	**73.9**	**0.729**	**0.8**	**4.144**	**3.27**	**51.8**	**51.9**
	Equiv. Refl.: (10,-8,3), (-2,10,3), (-2,-8,-3), (-8,10,-3), (-8,-2,3)								
**Quartz**	**(10,-1,1)**	**13.946**	**76.32**	**0.723**	**0.8**	**3.493**	**2.89**	**43.66**	**43.76**
	Equiv. Refl.: (10,-9,-1)								
**Quartz**	**(-1,10,-1)**	**13.946**	**76.32**	**0.722**	**0.8**	**3.493**	**2.89**	**43.66**	**43.76**
	Equiv. Refl.: (-1,-9,1), (-9,10,1), (-9,-1,-1)								
**LiNbO3**	**(4,7,9)**	**14**	**77.27**	**0.715**	**1.3**	**3.242**	**4.06**	**40.52**	**40.73**
	Equiv. Refl.: (4,7,-9), (4,-11,9), (4,-11,-9), (7,4,9), (7,4,-9), (7,-11,9), (7,-11,-9), (11,-4,9), (11,-4,-9) (11,-7,9), (11,-7,-9), (-4,11,9), (-4,11,-9), (-4,-7,9), (-4,-7,-9), (-7,11,9), (-7,11,-9), (-7,-4,9) (-7,-4,-9), (-11,4,9), (-11,4,-9), (-11,7,9), (-11,7,-9)								
**Quartz**	**(0,8,6)**	**13.536**	**70.58**	**0.707**	**0.8**	**5.062**	**3.89**	**63.27**	**63.39**
	Equiv. Refl.: (0,-8,-6)								
**Quartz**	**(8,0,-6)**	**13.536**	**70.58**	**0.706**	**0.8**	**5.062**	**3.88**	**63.27**	**63.39**
	Equiv. Refl.: (8,-8,6), (-8,0,6), (-8,8,-6)								
**Quartz**	**(2,8,3)**	**13.79**	**73.9**	**0.692**	**0.8**	**4.144**	**3.26**	**51.8**	**51.9**
	Equiv. Refl.: (2,-10,-3), (8,2,-3), (8,-10,3), (-10,2,3), (-10,8,-3)								
**Quartz**	**(-4,7,9)**	**13.606**	**71.43**	**0.69**	**0.7**	**4.822**	**3.55**	**60.28**	**60.38**
	Equiv. Refl.: (-4,-3,-9)								
**Quartz**	**(-3,7,-9)**	**13.606**	**71.43**	**0.689**	**0.7**	**4.822**	**3.54**	**60.28**	**60.38**
	Equiv. Refl.: (-3,-4,9)								
**Quartz**	**(7,-3,9)**	**13.606**	**71.43**	**0.688**	**0.7**	**4.822**	**3.54**	**60.28**	**60.38**
	Equiv. Refl.: (7,-4,-9)								
**Quartz**	**(3,-8,8)**	**13.719**	**72.91**	**0.648**	**0.7**	**4.413**	**3.14**	**55.16**	**55.25**
	Equiv. Refl.: (4,3,9), (4,-7,-9)								
**Quartz**	**(3,4,-9)**	**13.606**	**71.43**	**0.647**	**0.7**	**4.822**	**3.54**	**60.28**	**60.38**
	Equiv. Refl.: (3,5,-8), (3,-7,9), (5,3,8), (5,-8,-8), (-8,3,-8), (-8,5,8)								
**Quartz**	**(-7,3,-9)**	**13.606**	**71.43**	**0.646**	**0.7**	**4.822**	**3.54**	**60.28**	**60.38**
	Equiv. Refl.: (-7,4,9)								
**Quartz**	**(1,0,-12)**	**13.841**	**74.65**	**0.642**	**0.7**	**3.939**	**2.86**	**49.24**	**49.33**
	Equiv. Refl.: (1,-1,12), (-1,0,12), (-1,1,-12)								
**Quartz**	**(0,1,12)**	**13.841**	**74.65**	**0.641**	**0.7**	**3.939**	**2.86**	**49.24**	**49.33**
	Equiv. Refl.: (0,-1,-12), (8,2,4), (8,-10,-4), (-10,2,-4), (-10,8,4)								
**Quartz**	**(2,8,-4)**	**14.12**	**79.66**	**0.64**	**0.9**	**2.62**	**2.23**	**32.75**	**32.82**
	Equiv. Refl.: (2,-10,4)								

Ei =	14.353	keV						ΔEg	ΔEt
**Cryst**	**Refl**	**EB**	**ΘB**	**∫ IR dΘ**	**Width**	**Ei cotΘ**	**ΔE**	**2m, 50µm**	**2m, 50µm**
	**(h,k,l)**	**[keV]**	**[°]**	**[µrad]**	**[µrad]**	**[meV/µrad]**	**[meV]**	**[meV]**	**[meV]**
**LiNbO3**	**(3,4,-25)**	**14.018**	**77.61**	**0.639**	**1.2**	**3.154**	**3.7**	**39.43**	**39.6**
	Equiv. Refl.: (3,-7,25), (4,3,25), (4,-7,-25), (7,-3,25), (7,-4,-25), (-3,7,-25), (-3,-4,25), (-4,7,25), (-4,-3,-25) (-7,3,-25), (-7,4,25)								
**Quartz**	**(9,-1,5)**	**13.706**	**72.73**	**0.634**	**0.7**	**4.462**	**3.11**	**55.77**	**55.86**
	Equiv. Refl.: (9,-8,-5), (-1,9,-5), (-1,-8,5), (-8,9,5), (-8,-1,-5)								
**Quartz**	**(8,-3,8)**	**13.719**	**72.91**	**0.627**	**0.7**	**4.413**	**3.14**	**55.16**	**55.25**
	Equiv. Refl.: (8,-5,-8), (-3,8,-8), (-3,-5,8), (-5,8,8), (-5,-3,-8)								
**Quartz**	**(0,6,9)**	**13.527**	**70.47**	**0.616**	**0.7**	**5.091**	**3.46**	**63.63**	**63.73**
	Equiv. Refl.: (0,-6,-9), (6,0,-9), (6,-6,9), (-6,0,9), (-6,6,-9)								
**Quartz**	**(1,8,-5)**	**13.706**	**72.73**	**0.612**	**0.7**	**4.462**	**3.11**	**55.77**	**55.86**
	Equiv. Refl.: (1,-9,5), (8,1,5), (8,-9,-5), (-9,1,-5), (-9,8,5)								
**Quartz**	**(1,9,2)**	**14.087**	**78.95**	**0.601**	**0.7**	**2.803**	**1.96**	**35.04**	**35.1**
	Equiv. Refl.: (1,-10,-2), (9,1,-2), (9,-10,2), (-10,1,2), (-10,9,-2)								
**Sapph**	**(2,7,13)**	**13.789**	**73.89**	**0.594**	**0.7**	**4.147**	**2.93**	**51.83**	**51.91**
	Equiv. Refl.: (2,-9,-13), (7,2,-13), (7,-9,13), (9,-2,-13), (9,-7,13), (-2,9,13), (-2,-7,-13), (-7,9,-13), (-7,-2,13) (-9,2,13), (-9,7,-13)								
**LiNbO3**	**(2,9,-1)**	**14.119**	**79.64**	**0.581**	**1.2**	**2.624**	**3.1**	**32.8**	**32.95**
	Equiv. Refl.: (2,-11,1), (9,2,1), (9,-11,-1), (11,-2,1), (11,-9,-1), (-2,11,-1), (-2,-9,1), (-9,11,1), (-9,-2,-1) (-11,2,-1), (-11,9,1)								
**LiNbO3**	**(1,3,-29)**	**13.903**	**75.62**	**0.58**	**1**	**3.679**	**3.86**	**45.99**	**46.15**
	Equiv. Refl.: (1,-4,29), (3,1,29), (3,-4,-29), (4,-1,29), (4,-3,-29), (-1,4,-29), (-1,-3,29), (-3,4,29), (-3,-1,-29) (-4,1,-29), (-4,3,29)								
**Quartz**	**(10,-4,4)**	**13.505**	**70.21**	**0.578**	**0.6**	**5.166**	**3.2**	**64.57**	**64.65**
	Equiv. Refl.: (10,-6,-4), (-4,10,-4), (-4,-6,4), (-6,10,4), (-6,-4,-4)								
**Quartz**	**(2,3,11)**	**14.125**	**79.78**	**0.564**	**0.7**	**2.587**	**1.78**	**32.34**	**32.39**
	Equiv. Refl.: (2,-5,-11)								
**Quartz**	**(3,2,-11)**	**14.125**	**79.78**	**0.562**	**0.7**	**2.587**	**1.78**	**32.34**	**32.39**
	Equiv. Refl.: (3,-5,11)								
**Sapph**	**(2,4,-23)**	**13.56**	**70.87**	**0.56**	**0.7**	**4.979**	**3.27**	**62.24**	**62.32**
	Equiv. Refl.: (2,-6,23), (4,2,23), (4,-6,-23), (6,-2,23), (6,-4,-23), (-2,6,-23), (-2,-4,23), (-4,6,23), (-4,-2,-23) (-6,2,-23), (-6,4,23), (-5,3,-11)								
**Quartz**	**(-5,2,11)**	**14.125**	**79.78**	**0.559**	**0.7**	**2.587**	**1.77**	**32.34**	**32.38**
**Quartz**	**(10,-1,-2)**	**14.087**	**78.95**	**0.548**	**0.7**	**2.803**	**1.96**	**35.04**	**35.1**
	Equiv. Refl.: (10,-9,2), (-1,10,2), (-1,-9,-2), (-9,10,-2), (-9,-1,2)								
**Sapph**	**(3,5,19)**	**13.897**	**75.53**	**0.545**	**0.7**	**3.705**	**2.49**	**46.31**	**46.38**
	Equiv. Refl.: (3,-8,-19), (5,3,-19), (5,-8,19), (8,-3,-19), (8,-5,19), (-3,8,19), (-3,-5,-19), (-5,8,-19), (-5,-3,19) (-8,3,19), (-8,5,-19)								
**Quartz**	**(-2,5,11)**	**14.125**	**79.78**	**0.544**	**0.7**	**2.587**	**1.78**	**32.34**	**32.39**
	Equiv. Refl.: (-2,-3,-11)								
**Quartz**	**(-3,5,-11)**	**14.125**	**79.78**	**0.543**	**0.7**	**2.587**	**1.78**	**32.34**	**32.39**
**Quartz**	**(-3,-2,11)**	**14.125**	**79.78**	**0.542**	**0.7**	**2.587**	**1.78**	**32.34**	**32.39**
	Equiv. Refl.: (-7,8,-7)								
**Quartz**	**(-1,8,7)**	**13.619**	**71.59**	**0.541**	**0.6**	**4.777**	**2.83**	**59.71**	**59.78**
	Equiv. Refl.: (-1,-7,-7), (-7,-1,7)								
**Quartz**	**(5,-3,11)**	**14.125**	**79.78**	**0.54**	**0.7**	**2.587**	**1.77**	**32.34**	**32.38**
	Equiv. Refl.: (8,-1,-7), (8,-7,7)								
**Quartz**	**(5,-2,-11)**	**14.125**	**79.78**	**0.539**	**0.7**	**2.587**	**1.77**	**32.34**	**32.38**
**Quartz**	**(6,4,4)**	**13.505**	**70.21**	**0.533**	**0.6**	**5.166**	**3.21**	**64.57**	**64.65**
	Equiv. Refl.: (6,-10,-4)								
**Quartz**	**(4,6,-4)**	**13.505**	**70.21**	**0.532**	**0.6**	**5.166**	**3.2**	**64.57**	**64.65**
	Equiv. Refl.: (4,-10,4), (-10,4,-4), (-10,6,4)								

Ei =	14.353	keV						ΔEg	ΔEt
Cryst	Refl	EB	ΘB	∫ IR dΘ	Width	Ei cotΘ	ΔE	2m, 50µm	2m, 50µm
	(h,k,l)	[keV]	[°]	[µrad]	[µrad]	[meV/µrad]	[meV]	[meV]	[meV]
**Quartz**	**(7,3,4)**	**13.739**	**73.18**	**0.527**	**0.6**	**4.34**	**2.55**	**54.25**	**54.31**
	Equiv. Refl.: (7,-10,-4)								
**Quartz**	**(3,7,-4)**	**13.739**	**73.18**	**0.526**	**0.6**	**4.34**	**2.54**	**54.25**	**54.31**
	Equiv. Refl.: (3,-10,4), (-10,3,-4), (-10,7,4)								
**Sapph**	**(1,3,-26)**	**13.543**	**70.66**	**0.518**	**0.6**	**5.037**	**3.09**	**62.96**	**63.04**
	Equiv. Refl.: (1,-4,26), (3,1,26), (3,-4,-26), (4,-1,26), (4,-3,-26), (-1,4,-26), (-1,-3,26), (-3,4,26), (-3,-1,-26) (-4,1,-26), (-4,3,26)								
**Sapph**	**(4,4,18)**	**13.507**	**70.23**	**0.504**	**0.6**	**5.158**	**3.09**	**64.47**	**64.55**
	Equiv. Refl.: (4,4,-18), (4,-8,18), (4,-8,-18), (8,-4,18), (8,-4,-18), (-4,8,18), (-4,8,-18), (-4,-4,18), (-4,-4,-18) (-8,4,18), (-8,4,-18)								
**Quartz**	**(7,1,-7)**	**13.619**	**71.59**	**0.501**	**0.6**	**4.777**	**2.84**	**59.71**	**59.78**
	Equiv. Refl.: (7,-8,7)								
**Quartz**	**(1,7,7)**	**13.619**	**71.59**	**0.5**	**0.6**	**4.777**	**2.83**	**59.71**	**59.78**
	Equiv. Refl.: (1,-8,-7), (-8,1,7), (-8,7,-7)								
**Quartz**	**(8,-8,-7)**	**14.154**	**80.44**	**0.494**	**0.6**	**2.417**	**1.53**	**30.22**	**30.25**
**Quartz**	**(8,0,7)**	**14.154**	**80.44**	**0.493**	**0.6**	**2.417**	**1.53**	**30.22**	**30.25**
	Equiv. Refl.: (-8,0,-7), (-8,8,7)								
**Quartz**	**(0,8,-7)**	**14.154**	**80.44**	**0.492**	**0.6**	**2.417**	**1.53**	**30.22**	**30.25**
	Equiv. Refl.: (0,-8,7)								
**Quartz**	**(10,-3,4)**	**13.739**	**73.18**	**0.485**	**0.6**	**4.34**	**2.55**	**54.25**	**54.31**
	Equiv. Refl.: (10,-7,-4), (-7,10,4), (-7,-3,-4)								
**Quartz**	**(-3,10,-4)**	**13.739**	**73.18**	**0.484**	**0.6**	**4.34**	**2.54**	**54.25**	**54.31**
	Equiv. Refl.: (-3,-7,4)								
**Quartz**	**(8,-4,8)**	**13.642**	**71.89**	**0.476**	**0.5**	**4.695**	**2.43**	**58.69**	**58.74**
	Equiv. Refl.: (8,-4,-8)								
**Quartz**	**(-4,8,8)**	**13.642**	**71.89**	**0.474**	**0.5**	**4.695**	**2.42**	**58.69**	**58.74**
	Equiv. Refl.: (-4,8,-8), (-4,-4,8), (-4,-4,-8)								
**Quartz**	**(9,-2,-6)**	**13.769**	**73.6**	**0.456**	**0.5**	**4.224**	**2.18**	**52.79**	**52.84**
	Equiv. Refl.: (9,-7,6), (-7,9,-6), (-7,-2,6)								
**LiNbO3**	**(5,6,11)**	**14.147**	**80.28**	**0.455**	**1**	**2.458**	**2.57**	**30.72**	**30.83**
	Equiv. Refl.: (5,-11,-11), (6,5,-11), (6,-11,11), (11,-5,-11), (11,-6,11), (-5,11,11), (-5,-6,-11), (-6,11,-11), (-6,-5,11) (-11,5,11), (-11,6,-11), (-2,9,6), (-2,-7,-6)								
**Sapph**	**(5,5,12)**	**14.232**	**82.56**	**0.451**	**0.7**	**1.875**	**1.29**	**23.44**	**23.48**
	Equiv. Refl.: (5,5,-12), (5,-10,12), (5,-10,-12), (10,-5,12), (10,-5,-12), (-5,10,12), (-5,10,-12), (-5,-5,12), (-5,-5,-12) (-10,5,12), (-10,5,-12)								
**LiNbO3**	**(4,5,-19)**	**13.789**	**73.88**	**0.446**	**0.9**	**4.148**	**3.55**	**51.85**	**51.97**
	Equiv. Refl.: (4,-9,19), (5,4,19), (5,-9,-19), (9,-4,19), (9,-5,-19), (-4,9,-19), (-4,-5,19), (-5,9,19), (-5,-4,-19) (-9,4,-19), (-9,5,19)								
**Sapph**	**(3,3,24)**	**13.868**	**75.07**	**0.442**	**0.6**	**3.828**	**2.16**	**47.85**	**47.9**
	Equiv. Refl.: (3,3,-24), (3,-6,24), (3,-6,-24), (6,-3,24), (6,-3,-24), (-3,6,24), (-3,6,-24), (-3,-3,24), (-3,-3,-24) (-6,3,24), (-6,3,-24)								
**LiNbO3**	**(2,3,29)**	**14.315**	**85.81**	**0.436**	**1.5**	**1.052**	**1.55**	**13.15**	**13.24**
	Equiv. Refl.: (2,-5,-29), (3,2,-29), (3,-5,29), (5,-2,-29), (5,-3,29), (-2,5,29), (-2,-3,-29), (-3,5,-29), (-3,-2,29) (-5,2,29), (-5,3,-29)								
**Sapph**	**(2,3,-25)**	**13.615**	**71.55**	**0.429**	**0.5**	**4.788**	**2.55**	**59.85**	**59.9**
	Equiv. Refl.: (2,-5,25), (3,2,25), (3,-5,-25), (5,-2,25), (5,-3,-25), (-2,5,-25), (-2,-3,25), (-3,5,25), (-3,-2,-25) (-5,2,-25), (-5,3,25)								
**Quartz**	**(-8,4,8)**	**13.642**	**71.89**	**0.409**	**0.5**	**4.695**	**2.43**	**58.69**	**58.74**
	Equiv. Refl.: (-8,4,-8)								

Ei =	14.353	keV						ΔEg	ΔEt
**Cryst**	**Refl**	**EB**	**ΘB**	**∫ IR dΘ**	**Width**	**Ei cotΘ**	**ΔE**	**2m, 50µm**	**2m, 50µm**
	**(h,k,l)**	**[keV]**	**[°]**	**[µrad]**	**[µrad]**	**[meV/µrad]**	**[meV]**	**[meV]**	**[meV]**
**LiNbO3**	**(1,4,27)**	**13.652**	**72.02**	**0.407**	**0.8**	**4.658**	**3.63**	**58.23**	**58.34**
	Equiv. Refl.: (1,4,-27), (1,-5,27), (1,-5,-27), (4,1,27), (4,1,-27), (4,-5,27), (4,-5,-27), (5,-1,27), (5,-1,-27) (5,-4,27), (5,-4,-27), (-1,5,27), (-1,5,-27), (-1,-4,27), (-1,-4,-27), (-4,5,27), (-4,5,-27), (-4,-1,27) (-4,-1,-27), (-5,1,27), (-5,1,-27), (-5,4,27), (-5,4,-27), (4,4,8), (4,4,-8), (4,-8,8), (4,-8,-8) (-10,2,-3), (-10,8,3)								
**Quartz**	**(2,8,-3)**	**13.79**	**73.9**	**0.406**	**0.5**	**4.144**	**1.97**	**51.8**	**51.84**
	Equiv. Refl.: (2,-10,3), (7,2,-6), (7,-9,6), (8,2,3), (8,-10,-3), (-9,2,6), (-9,7,-6)								
**Quartz**	**(2,7,6)**	**13.769**	**73.6**	**0.405**	**0.5**	**4.224**	**2.18**	**52.79**	**52.84**
	Equiv. Refl.: (2,-9,-6)								
**Quartz**	**(10,-2,3)**	**13.79**	**73.9**	**0.369**	**0.5**	**4.144**	**1.97**	**51.8**	**51.84**
	Equiv. Refl.: (10,-8,-3)								
**Quartz**	**(-2,10,-3)**	**13.79**	**73.9**	**0.368**	**0.5**	**4.144**	**1.97**	**51.8**	**51.84**
	Equiv. Refl.: (-2,-8,3), (-8,10,3), (-8,-2,-3)								
**Sapph**	**(2,8,3)**	**13.862**	**74.98**	**0.354**	**0.5**	**3.852**	**1.84**	**48.15**	**48.19**
	Equiv. Refl.: (2,8,-3), (2,-10,3), (2,-10,-3), (8,2,3), (8,2,-3), (8,-10,3), (8,-10,-3), (10,-2,3), (10,-2,-3) (10,-8,3), (10,-8,-3), (-2,10,3), (-2,10,-3), (-2,-8,3), (-2,-8,-3), (-8,10,3), (-8,10,-3), (-8,-2,3) (-8,-2,-3), (-10,2,3), (-10,2,-3), (-10,8,3), (-10,8,-3)								
**Sapph**	**(5,5,9)**	**13.719**	**72.9**	**0.352**	**0.5**	**4.415**	**2.04**	**55.19**	**55.23**
	Equiv. Refl.: (5,5,-9), (5,-10,9), (5,-10,-9), (10,-5,9), (10,-5,-9), (-5,10,9), (-5,10,-9), (-5,-5,9), (-5,-5,-9) (-10,5,9), (-10,5,-9)								
**Quartz**	**(5,5,5)**	**13.86**	**74.94**	**0.347**	**0.4**	**3.862**	**1.7**	**48.27**	**48.3**
	Equiv. Refl.: (5,-10,-5), (-10,5,5), (-10,5,-5)								
**Quartz**	**(5,5,-5)**	**13.86**	**74.94**	**0.345**	**0.4**	**3.862**	**1.69**	**48.27**	**48.3**
	Equiv. Refl.: (5,-10,5)								
**Quartz**	**(10,-5,5)**	**13.86**	**74.94**	**0.343**	**0.4**	**3.862**	**1.7**	**48.27**	**48.3**
	Equiv. Refl.: (10,-5,-5), (-5,10,5), (-5,-5,-5)								
**Quartz**	**(-5,10,-5)**	**13.86**	**74.94**	**0.341**	**0.4**	**3.862**	**1.69**	**48.27**	**48.3**
	Equiv. Refl.: (-5,-5,5)								
**LiNbO3**	**(1,5,-25)**	**13.598**	**71.34**	**0.324**	**0.7**	**4.847**	**3.25**	**60.59**	**60.68**
	Equiv. Refl.: (1,-6,25), (5,1,25), (5,-6,-25), (6,-1,25), (6,-5,-25), (-1,6,-25), (-1,-5,25), (-5,6,25), (-5,-1,-25) (-6,1,-25), (-6,5,25)								
**Quartz**	**(-5,11,2)**	**14.087**	**78.95**	**0.317**	**0.4**	**2.803**	**1.11**	**35.04**	**35.06**
	Equiv. Refl.: (-5,-6,-2)								
**Quartz**	**(11,-5,-2)**	**14.087**	**78.95**	**0.316**	**0.4**	**2.803**	**1.11**	**35.04**	**35.06**
	Equiv. Refl.: (11,-6,2), (-6,11,-2), (-6,-5,2)								
**LiNbO3**	**(3,7,-13)**	**13.658**	**72.1**	**0.311**	**0.7**	**4.637**	**3.08**	**57.96**	**58.05**
	Equiv. Refl.: (3,-10,13), (7,3,13), (7,-10,-13), (10,-3,13), (10,-7,-13), (-3,10,-13), (-3,-7,13), (-7,10,13), (-7,-3,-13) (-10,3,-13), (-10,7,13)								
**Quartz**	**(0,8,7)**	**14.154**	**80.44**	**0.301**	**0.4**	**2.417**	**1.07**	**30.22**	**30.23**
	Equiv. Refl.: (0,-8,-7), (8,0,-7), (8,-8,7), (-8,0,7), (-8,8,-7)								
**Quartz**	**(5,1,-10)**	**14.049**	**78.19**	**0.294**	**0.4**	**3.002**	**1.22**	**37.53**	**37.55**
	Equiv. Refl.: (5,-6,10)								
**Quartz**	**(1,5,10)**	**14.049**	**78.19**	**0.291**	**0.4**	**3.002**	**1.21**	**37.53**	**37.55**
	Equiv. Refl.: (1,-6,-10), (-6,1,10), (-6,5,-10)								
**Quartz**	**(-5,6,-10)**	**14.049**	**78.19**	**0.287**	**0.4**	**3.002**	**1.22**	**37.53**	**37.55**
	Equiv. Refl.: (-5,-1,10)								
**Quartz**	**(6,-1,-10)**	**14.049**	**78.19**	**0.285**	**0.4**	**3.002**	**1.21**	**37.53**	**37.55**
	Equiv. Refl.: (6,-5,10), (-1,6,10), (-1,-5,-10)								
**Quartz**	**(0,4,-11)**	**13.898**	**75.54**	**0.274**	**0.4**	**3.702**	**1.38**	**46.27**	**46.3**
	Equiv. Refl.: (0,-4,11)								
**Quartz**	**(4,0,11)**	**13.898**	**75.54**	**0.272**	**0.4**	**3.702**	**1.37**	**46.27**	**46.3**
	Equiv. Refl.: (4,-4,-11), (-4,0,-11), (-4,4,11)								

Ei = Cryst	14.353 Refl (h,k,l)	keV EB [keV]	ΘB [°]	∫ IR dΘ [µrad]	Width [µrad]	Ei cotΘ [meV/µrad]	ΔE [meV]	ΔEg 2m, 50µm [meV]	ΔEt 2m, 50µm [meV]
**LiNbO3**	**(5,6,-7)**	**13.629**	**71.72**	**0.271**	**0.6**	**4.741**	**2.88**	**59.26**	**59.33**
	Equiv. Refl.: (5,-11,7), (6,5,7), (6,-11,-7), (11,-5,7), (11,-6,-7), (-5,11,-7), (-5,-6,7), (-6,11,7), (-6,-5,-7) (-11,5,-7), (-11,6,7)								
**Quartz**	**(5,0,10)**	**13.588**	**71.21**	**0.268**	**0.3**	**4.883**	**1.69**	**61.04**	**61.07**
	Equiv. Refl.: (5,-5,-10), (-5,0,-10)								
**Quartz**	**(-5,5,10)**	**13.588**	**71.21**	**0.267**	**0.3**	**4.883**	**1.69**	**61.04**	**61.07**
**Quartz**	**(0,5,-10)**	**13.588**	**71.21**	**0.266**	**0.3**	**4.883**	**1.68**	**61.04**	**61.07**
	Equiv. Refl.: (0,-5,10)								
**Quartz**	**(8,-1,7)**	**13.619**	**71.59**	**0.26**	**0.3**	**4.777**	**1.45**	**59.71**	**59.73**
	Equiv. Refl.: (8,-7,-7), (-7,8,7), (-7,-1,-7)								
**Quartz**	**(-1,8,-7)**	**13.619**	**71.59**	**0.259**	**0.3**	**4.777**	**1.44**	**59.71**	**59.73**
	Equiv. Refl.: (-1,-7,7)								
**Quartz**	**(11,-4,1)**	**14.097**	**79.17**	**0.254**	**0.3**	**2.746**	**0.9**	**34.32**	**34.33**
	Equiv. Refl.: (11,-7,-1), (-4,11,-1), (-4,-7,1), (-7,11,1), (-7,-4,-1)								
**Quartz**	**(5,6,2)**	**14.087**	**78.95**	**0.235**	**0.4**	**2.803**	**1.11**	**35.04**	**35.06**
	Equiv. Refl.: (5,-11,-2), (-11,5,2), (-11,6,-2)								
**Quartz**	**(6,5,-2)**	**14.087**	**78.95**	**0.234**	**0.4**	**2.803**	**1.1**	**35.04**	**35.06**
	Equiv. Refl.: (6,-11,2)								
**Sapph**	**(3,4,23)**	**14.292**	**84.7**	**0.221**	**0.5**	**1.331**	**0.65**	**16.64**	**16.65**
	Equiv. Refl.: (3,-7,-23), (4,3,-23), (4,-7,23), (7,-3,-23), (7,-4,23), (-3,7,23), (-3,-4,-23), (-4,7,-23), (-4,-3,23) (-7,3,23), (-7,4,-23)								
**Quartz**	**(0,7,8)**	**13.719**	**72.91**	**0.212**	**0.3**	**4.413**	**1.3**	**55.16**	**55.17**
	Equiv. Refl.: (0,-7,-8)								
**Quartz**	**(2,5,-9)**	**13.761**	**73.48**	**0.21**	**0.3**	**4.256**	**1.25**	**53.2**	**53.22**
	Equiv. Refl.: (2,-7,9), (-7,2,-9), (-7,5,9)								
**Quartz**	**(7,0,-8)**	**13.719**	**72.91**	**0.209**	**0.3**	**4.413**	**1.29**	**55.16**	**55.17**
	Equiv. Refl.: (7,-7,8), (-7,0,8), (-7,7,-8)								
**Quartz**	**(5,2,9)**	**13.761**	**73.48**	**0.207**	**0.3**	**4.256**	**1.24**	**53.2**	**53.22**
	Equiv. Refl.: (5,-7,-9)								
**Quartz**	**(7,-2,9)**	**13.761**	**73.48**	**0.206**	**0.3**	**4.256**	**1.25**	**53.2**	**53.22**
	Equiv. Refl.: (7,-5,-9), (-2,7,-9), (-2,-5,9)								
**Quartz**	**(-5,7,9)**	**13.761**	**73.48**	**0.203**	**0.3**	**4.256**	**1.24**	**53.2**	**53.22**
	Equiv. Refl.: (-5,-2,-9)								
**Quartz**	**(7,-2,-9)**	**13.761**	**73.48**	**0.2**	**0.3**	**4.256**	**1.15**	**53.2**	**53.22**
	Equiv. Refl.: (7,-5,9), (-2,7,9), (-2,-5,-9), (-5,7,-9)								
**Quartz**	**(-5,-2,9)**	**13.761**	**73.48**	**0.199**	**0.3**	**4.256**	**1.15**	**53.2**	**53.22**
**Quartz**	**(-2,6,-10)**	**13.82**	**74.34**	**0.195**	**0.2**	**4.023**	**0.93**	**50.29**	**50.29**
	Equiv. Refl.: (-2,-4,10)								
**Sapph**	**(0,4,26)**	**13.792**	**73.92**	**0.194**	**0.3**	**4.137**	**1.26**	**51.71**	**51.72**
	Equiv. Refl.: (0,-4,-26), (4,0,-26), (4,-4,26), (-4,0,26), (-4,4,-26)								
**Quartz**	**(6,-4,-10)**	**13.82**	**74.34**	**0.193**	**0.2**	**4.023**	**0.92**	**50.29**	**50.29**
	Equiv. Refl.: (7,1,7), (7,-8,-7), (9,0,4), (9,-9,-4), (-4,6,10), (-4,-2,-10), (-8,7,7), (-9,0,-4), (-9,9,4)								
**Quartz**	**(0,9,-4)**	**13.892**	**75.45**	**0.192**	**0.3**	**3.727**	**1.07**	**46.58**	**46.6**
	Equiv. Refl.: (0,-9,4), (1,7,-7), (1,-8,7), (6,-2,10), (-8,1,-7)								
**LiNbO3**	**(1,7,21)**	**14.085**	**78.91**	**0.181**	**0.6**	**2.812**	**1.67**	**35.15**	**35.19**
	Equiv. Refl.: (1,7,-21), (1,-8,21), (1,-8,-21), (7,1,21), (7,1,-21), (7,-8,21), (7,-8,-21), (8,-1,21), (8,-1,-21) (8,-7,21), (8,-7,-21), (-1,8,21), (-1,8,-21), (-1,-7,21), (-1,-7,-21), (-7,8,21), (-7,8,-21), (-7,-1,21) (-7,-1,-21), (-8,1,21), (-8,1,-21), (-8,7,21), (-8,7,-21)								
**LiNbO3**	**(3,5,-23)**	**14.161**	**80.61**	**0.173**	**0.6**	**2.374**	**1.48**	**29.68**	**29.71**
	Equiv. Refl.: (3,-8,23), (5,3,23), (5,-8,-23), (8,-3,23), (8,-5,-23), (-3,8,-23), (-3,-5,23), (-5,8,23), (-5,-3,-23) (-8,3,-23), (-8,5,23), (2,5,9), (2,-7,-9)								
**Quartz**	**(5,2,-9)**	**13.761**	**73.48**	**0.172**	**0.3**	**4.256**	**1.15**	**53.2**	**53.22**

	Equiv. Refl.: (5,-7,9), (-7,2,9), (-7,5,-9)								
**Quartz**	**(4,7,-1)**	**14.097**	**79.17**	**0.17**	**0.3**	**2.746**	**0.9**	**34.32**	**34.33**
	Equiv. Refl.: (4,-11,1), (7,4,1), (7,-11,-1), (-11,4,-1), (-11,7,1)								
**Quartz**	**(-3,8,8)**	**13.719**	**72.91**	**0.159**	**0.2**	**4.413**	**0.98**	**55.16**	**55.17**
	Equiv. Refl.: (-3,-5,-8), (-5,8,-8), (-5,-3,8)								
**Quartz**	**(8,-3,-8)**	**13.719**	**72.91**	**0.158**	**0.2**	**4.413**	**0.98**	**55.16**	**55.17**
	Equiv. Refl.: (8,-5,8)								
**LiNbO3**	**(3,8,7)**	**14.048**	**78.17**	**0.131**	**0.5**	**3.008**	**1.46**	**37.59**	**37.62**
	Equiv. Refl.: (3,-11,-7), (8,3,-7), (8,-11,7), (11,-3,-7), (11,-8,7), (-3,11,7), (-3,-8,-7), (-8,11,-7), (-8,-3,7) (-11,3,7), (-11,8,-7)								
**Quartz**	**(5,3,-8)**	**13.719**	**72.91**	**0.128**	**0.2**	**4.413**	**0.98**	**55.16**	**55.17**
	Equiv. Refl.: (5,-8,8)								
**Quartz**	**(3,5,8)**	**13.719**	**72.91**	**0.127**	**0.2**	**4.413**	**0.98**	**55.16**	**55.17**
	Equiv. Refl.: (3,-8,-8), (-8,3,8), (-8,5,-8)								
**Sapph**	**(3,6,15)**	**13.923**	**75.94**	**0.123**	**0.2**	**3.596**	**0.85**	**44.95**	**44.95**
	Equiv. Refl.: (3,6,-15), (3,-9,15), (3,-9,-15), (6,3,15), (6,3,-15), (6,-9,15), (6,-9,-15), (9,-3,15), (9,-3,-15) (9,-6,15), (9,-6,-15), (-3,9,15), (-3,9,-15), (-3,-6,15), (-3,-6,-15), (-6,9,15), (-6,9,-15), (-6,-3,15) (-6,-3,-15), (-9,3,15), (-9,3,-15), (-9,6,15), (-9,6,-15)								
**LiNbO3**	**(1,8,17)**	**14.104**	**79.32**	**0.122**	**0.5**	**2.707**	**1.33**	**33.84**	**33.86**
	Equiv. Refl.: (1,-9,-17), (8,1,-17), (8,-9,17), (9,-1,-17), (9,-8,17), (-1,9,17), (-1,-8,-17), (-8,9,-17), (-8,-1,17) (-9,1,17), (-9,8,-17)								
**LiNbO3**	**(3,8,-5)**	**13.876**	**75.19**	**0.109**	**0.4**	**3.795**	**1.52**	**47.44**	**47.47**
	Equiv. Refl.: (3,-11,5), (8,3,5), (8,-11,-5), (11,-3,5), (11,-8,-5), (-3,11,-5), (-3,-8,5), (-8,11,5), (-8,-3,-5) (-11,3,-5), (-11,8,5), (2,-6,10)								
**Quartz**	**(2,4,-10)**	**13.82**	**74.34**	**0.108**	**0.2**	**4.023**	**0.93**	**50.29**	**50.29**
**Quartz**	**(4,2,10)**	**13.82**	**74.34**	**0.107**	**0.2**	**4.023**	**0.93**	**50.29**	**50.29**
	Equiv. Refl.: (4,-6,-10), (-6,2,-10), (-6,4,10)								
**Sapph**	**(3,7,-4)**	**13.507**	**70.23**	**0.102**	**0.2**	**5.158**	**0.95**	**64.48**	**64.49**
	Equiv. Refl.: (3,-10,4), (7,3,4), (7,-10,-4), (10,-3,4), (10,-7,-4), (-3,10,-4), (-3,-7,4), (-7,10,4), (-7,-3,-4) (-10,3,-4), (-10,7,4)								
**LiNbO3**	**(3,8,1)**	**13.702**	**72.68**	**0.097**	**0.4**	**4.477**	**1.58**	**55.96**	**55.98**
	Equiv. Refl.: (3,-11,-1), (8,3,-1), (8,-11,1), (11,-3,-1), (11,-8,1), (-3,11,1), (-3,-8,-1), (-8,11,-1), (-8,-3,1) (-11,3,1), (-11,8,-1)								
**Sapph**	**(4,6,-11)**	**14.127**	**79.82**	**0.094**	**0.2**	**2.577**	**0.59**	**32.22**	**32.22**
	Equiv. Refl.: (4,-10,11), (6,4,11), (6,-10,-11), (10,-4,11), (10,-6,-11), (-4,10,-11), (-4,-6,11), (-6,10,11), (-6,-4,-11) (-10,4,-11), (-10,6,11)								
**LiNbO3**	**(3,3,27)**	**14.071**	**78.61**	**0.065**	**0.4**	**2.891**	**1.01**	**36.13**	**36.15**
	Equiv. Refl.: (3,3,-27), (3,-6,27), (3,-6,-27), (6,-3,27), (6,-3,-27), (-3,6,27), (-3,6,-27), (-3,-3,27), (-3,-3,-27) (-6,3,27), (-6,3,-27)								
**Sapph**	**(4,6,7)**	**13.534**	**70.55**	**0.059**	**0.1**	**5.068**	**0.69**	**63.35**	**63.35**
	Equiv. Refl.: (4,-10,-7), (6,4,-7), (6,-10,7), (10,-4,-7), (10,-6,7), (-4,10,7), (-4,-6,-7), (-6,10,-7), (-6,-4,7) (-10,4,7), (-10,6,-7)								
**LiNbO3**	**(5,5,15)**	**13.784**	**73.81**	**0.006**	**0.1**	**4.166**	**0.42**	**52.07**	**52.08**
	Equiv. Refl.: (5,5,-15), (5,-10,15), (5,-10,-15), (10,-5,15), (10,-5,-15), (-5,10,15), (-5,10,-15), (-5,-5,15), (-5,-5,-15) (-10,5,15), (-10,5,-15)								

Ei =	13.734	keV						ΔEg	ΔEt
Cryst	Refl	EB	ΘB	∫ IR dΘ	Width	Ei cotΘ	ΔE	2m, 50µm	2m, 50µm
	(h,k,l)	[keV]	[°]	[µrad]	[µrad]	[meV/µrad]	[meV]	[meV]	[meV]
**Si**	**(0,0,12)**	**13.697**	**85.81**	**8.1691**	**7.23**	**1.0056**	**7.27**	**12.57**	**14.52**
	Equiv. Refl.: (0,12,0), (4,8,8), (8,4,8), (8,8,4), (12,0,0)								
**Ge**	**(2,2,12)**	**13.51**	**79.63**	**2.9956**	**6.57**	**2.5132**	**16.51**	**31.41**	**35.49**
	Equiv. Refl.: (2,12,2), (4,6,10), (4,10,6), (6,4,10), (6,10,4), (10,4,6), (10,6,4), (12,2,2)								
**Si**	**(0,6,10)**	**13.311**	**75.75**	**2.9781**	**2.37**	**3.488**	**8.27**	**43.6**	**44.38**
	Equiv. Refl.: (0,10,6), (6,0,10), (6,6,8), (6,8,6), (6,10,0), (8,6,6), (10,0,6), (10,6,0)								
**Ge**	**(3,5,11)**	**13.642**	**83.38**	**2.3888**	**7.02**	**1.5947**	**11.19**	**19.93**	**22.86**
	Equiv. Refl.: (3,11,5), (5,3,11), (5,7,9), (5,9,7), (5,11,3), (7,5,9), (7,9,5), (9,5,7), (9,7,5) (11,3,5), (11,5,3)								
**Ge**	**(0,0,12)**	**13.149**	**73.22**	**2.1149**	**4.48**	**4.1408**	**18.54**	**51.76**	**54.98**
	Equiv. Refl.: (0,12,0), (4,8,8), (8,4,8), (8,8,4), (12,0,0)								
**Si**	**(0,8,8)**	**12.914**	**70.1**	**1.8159**	**1.91**	**4.9715**	**9.47**	**62.14**	**62.86**
	Equiv. Refl.: (8,0,8), (8,8,0)								
**Si**	**(3,3,11)**	**13.457**	**78.48**	**1.789**	**1.99**	**2.7987**	**5.57**	**34.98**	**35.42**
	Equiv. Refl.: (3,7,9), (3,9,7), (3,11,3), (7,3,9), (7,9,3), (9,3,7), (9,7,3), (11,3,3)								
**Si**	**(1,3,11)**	**13.064**	**72.04**	**1.2988**	**1.43**	**4.4531**	**6.36**	**55.66**	**56.03**
	Equiv. Refl.: (1,7,9), (1,9,7), (1,11,3), (3,1,11), (3,11,1), (5,5,9), (5,9,5), (7,1,9), (7,9,1) (9,1,7), (9,5,5), (9,7,1), (11,1,3), (11,3,1)								
**Ge**	**(1,5,11)**	**13.286**	**75.32**	**1.2427**	**3.49**	**3.5981**	**12.54**	**44.98**	**46.69**
	Equiv. Refl.: (1,11,5), (5,1,11), (5,11,1), (7,7,7), (11,1,5), (11,5,1)								
**Ge**	**(3,3,11)**	**12.919**	**70.16**	**1.0647**	**2.85**	**4.9546**	**14.14**	**61.93**	**63.53**
	Equiv. Refl.: (3,7,9), (3,9,7), (3,11,3), (7,3,9), (7,9,3), (9,3,7), (9,7,3), (11,3,3)								
**Sapph**	**(1,6,-20)**	**13.727**	**88.24**	**27.709**	**23.5**	**0.423**	**9.93**	**5.29**	**11.25**
	Equiv. Refl.: (1,-7,20), (6,1,20), (6,-7,-20), (7,-1,20), (7,-6,-20), (-1,7,-20), (-1,-6,20), (-6,7,20), (-6,-1,-20) (-7,1,-20), (-7,6,20)								
**LiNbO3**	**(0,-4,28)**	**13.701**	**86.01**	**26.512**	**23.8**	**0.958**	**22.84**	**11.98**	**25.79**
	Equiv. Refl.: (4,0,28), (-4,4,28)								
**LiNbO3**	**(3,-11,2)**	**13.724**	**87.8**	**26.411**	**25.8**	**0.527**	**13.6**	**6.59**	**15.12**
	Equiv. Refl.: (8,3,2), (11,-3,2), (-3,-8,2), (-8,11,2), (-11,8,2)								
**LiNbO3**	**(0,4,-28)**	**13.701**	**86.01**	**26.222**	**23.8**	**0.958**	**22.84**	**11.98**	**25.79**
	Equiv. Refl.: (-4,0,-28)								
**LiNbO3**	**(4,-4,-28)**	**13.701**	**86.01**	**26.221**	**23.8**	**0.958**	**22.84**	**11.98**	**25.79**
**LiNbO3**	**(3,8,-2)**	**13.724**	**87.8**	**25.826**	**25.8**	**0.527**	**13.6**	**6.59**	**15.12**
	Equiv. Refl.: (8,-11,-2), (11,-8,-2), (-3,11,-2), (-8,-3,-2), (-11,3,-2)								
**LiNbO3**	**(4,-11,-6)**	**13.675**	**84.69**	**18.699**	**16.9**	**1.275**	**21.57**	**15.94**	**26.82**
	Equiv. Refl.: (7,4,-6), (11,-4,-6), (-4,-7,-6), (-7,11,-6), (-11,7,-6)								
**LiNbO3**	**(4,7,6)**	**13.675**	**84.69**	**18.38**	**16.9**	**1.275**	**21.57**	**15.94**	**26.82**
	Equiv. Refl.: (7,-11,6), (11,-7,6), (-4,11,6), (-7,-4,6), (-11,4,6)								
**LiNbO3**	**(4,7,-6)**	**13.675**	**84.69**	**17.878**	**16.1**	**1.275**	**20.54**	**15.94**	**26**
	Equiv. Refl.: (7,-11,-6), (11,-7,-6), (-4,11,-6), (-7,-4,-6), (-11,4,-6)								
**LiNbO3**	**(4,-11,6)**	**13.675**	**84.69**	**17.161**	**16.1**	**1.275**	**20.54**	**15.94**	**26**
	Equiv. Refl.: (7,4,6), (11,-4,6), (-4,-7,6), (-7,11,6), (-11,7,6)								
**LiNbO3**	**(-3,9,-18)**	**13.66**	**84.05**	**15.979**	**14.6**	**1.431**	**20.84**	**17.89**	**27.46**
	Equiv. Refl.: (-9,3,-18)								
**LiNbO3**	**(3,6,-18)**	**13.66**	**84.05**	**15.978**	**14.6**	**1.431**	**20.84**	**17.89**	**27.46**
	Equiv. Refl.: (6,-9,-18), (9,-6,-18), (-6,-3,-18)								
**LiNbO3**	**(3,-9,18)**	**13.66**	**84.05**	**15.754**	**14.6**	**1.431**	**20.84**	**17.89**	**27.46**
	Equiv. Refl.: (6,3,18), (9,-3,18), (-3,-6,18)								

Ei =	13.734	keV						ΔEg	ΔEt
**Cryst**	**Refl**	**EB**	**ΘB**	**∫ IR dΘ**	**Width**	**Ei cotΘ**	**ΔE**	**2m, 50µm**	**2m, 50µm**
	**(h,k,l)**	**[keV]**	**[°]**	**[µrad]**	**[µrad]**	**[meV/µrad]**	**[meV]**	**[meV]**	**[meV]**
**LiNbO3**	**(-6,9,18)**	**13.66**	**84.05**	**15.753**	**14.6**	**1.431**	**20.84**	**17.89**	**27.46**
	Equiv. Refl.: (-9,6,18)								
**LiNbO3**	**(3,6,18)**	**13.66**	**84.05**	**14.041**	**13.1**	**1.431**	**18.7**	**17.89**	**25.88**
	Equiv. Refl.: (6,-9,18), (9,-6,18), (-3,9,18), (-6,-3,18), (-9,3,18)								
**LiNbO3**	**(3,-9,-18)**	**13.66**	**84.05**	**13.944**	**13.1**	**1.431**	**18.7**	**17.89**	**25.88**
	Equiv. Refl.: (6,3,-18), (9,-3,-18), (-3,-6,-18), (-6,9,-18), (-9,6,-18)								
**LiNbO3**	**(0,9,12)**	**13.616**	**82.5**	**13.747**	**12.3**	**1.809**	**22.21**	**22.62**	**31.7**
	Equiv. Refl.: (9,-9,12), (-9,0,12)								
**LiNbO3**	**(0,-9,-12)**	**13.616**	**82.5**	**13.245**	**12.3**	**1.809**	**22.21**	**22.62**	**31.7**
	Equiv. Refl.: (9,0,-12), (-9,9,-12)								
**LiNbO3**	**(0,-5,26)**	**13.547**	**80.53**	**13.102**	**11.6**	**2.291**	**26.55**	**28.64**	**39.06**
	Equiv. Refl.: (5,0,26), (-5,5,26)								
**LiNbO3**	**(4,6,-14)**	**13.643**	**83.41**	**12.998**	**11.9**	**1.587**	**18.85**	**19.84**	**27.37**
	Equiv. Refl.: (6,-10,-14), (10,-6,-14), (-4,10,-14), (-6,-4,-14), (-10,4,-14)								
**LiNbO3**	**(0,5,-26)**	**13.547**	**80.53**	**12.986**	**11.6**	**2.291**	**26.55**	**28.64**	**39.06**
	Equiv. Refl.: (5,-5,-26), (-5,0,-26)								
**LiNbO3**	**(4,-10,14)**	**13.643**	**83.41**	**12.467**	**11.9**	**1.587**	**18.85**	**19.84**	**27.37**
	Equiv. Refl.: (6,4,14), (10,-4,14), (-4,-6,14)								
**LiNbO3**	**(-6,10,14)**	**13.643**	**83.41**	**12.466**	**11.9**	**1.587**	**18.85**	**19.84**	**27.37**
	Equiv. Refl.: (-10,6,14)								
**Quartz**	**(7,0,8)**	**13.719**	**87.35**	**11.487**	**9.6**	**0.636**	**6.07**	**7.95**	**10**
**Quartz**	**(7,-7,-8)**	**13.719**	**87.35**	**11.486**	**9.6**	**0.636**	**6.07**	**7.95**	**10**
**Quartz**	**(-7,0,-8)**	**13.719**	**87.35**	**11.485**	**9.6**	**0.636**	**6.07**	**7.95**	**10**
**Quartz**	**(-7,7,8)**	**13.719**	**87.35**	**11.484**	**9.6**	**0.636**	**6.07**	**7.95**	**10**
**Quartz**	**(0,7,-8)**	**13.719**	**87.35**	**11.472**	**9.5**	**0.636**	**6.07**	**7.95**	**10**
	Equiv. Refl.: (0,-7,8)								
**LiNbO3**	**(0,0,-30)**	**13.415**	**77.63**	**10.844**	**9.4**	**3.011**	**28.44**	**37.64**	**47.18**
**LiNbO3**	**(0,-6,-24)**	**13.594**	**81.8**	**10.561**	**9.4**	**1.979**	**18.66**	**24.74**	**30.99**
	Equiv. Refl.: (6,0,-24), (-6,6,-24)								
**LiNbO3**	**(0,0,30)**	**13.415**	**77.63**	**10.557**	**9.4**	**3.011**	**28.44**	**37.64**	**47.18**
**LiNbO3**	**(0,6,24)**	**13.594**	**81.8**	**10.072**	**9.4**	**1.979**	**18.66**	**24.74**	**30.99**
	Equiv. Refl.: (6,-6,24), (-6,0,24)								
**LiNbO3**	**(1,-4,-28)**	**13.487**	**79.13**	**10.049**	**9.1**	**2.638**	**23.92**	**32.98**	**40.74**
	Equiv. Refl.: (3,1,-28), (4,-1,-28), (-1,-3,-28), (-3,4,-28), (-4,3,-28)								
**LiNbO3**	**(1,3,28)**	**13.487**	**79.13**	**10.042**	**9.1**	**2.638**	**23.92**	**32.98**	**40.74**
	Equiv. Refl.: (3,-4,28), (4,-3,28), (-1,4,28), (-3,-1,28), (-4,1,28)								
**LiNbO3**	**(1,1,30)**	**13.63**	**82.94**	**10.018**	**9.3**	**1.702**	**15.79**	**21.27**	**26.49**
	Equiv. Refl.: (1,-2,30), (2,-1,30), (-1,2,30), (-1,-1,30), (-2,1,30)								
**LiNbO3**	**(0,-9,12)**	**13.616**	**82.5**	**9.818**	**9**	**1.809**	**16.32**	**22.62**	**27.89**
	Equiv. Refl.: (9,0,12), (-9,9,12)								
**LiNbO3**	**(0,6,-24)**	**13.594**	**81.8**	**9.708**	**9**	**1.979**	**17.72**	**24.74**	**30.43**
	Equiv. Refl.: (6,-6,-24), (-6,0,-24)								
**LiNbO3**	**(0,-6,24)**	**13.594**	**81.8**	**9.687**	**9**	**1.979**	**17.72**	**24.74**	**30.43**
	Equiv. Refl.: (6,0,24)								
**LiNbO3**	**(-6,6,24)**	**13.594**	**81.8**	**9.686**	**9**	**1.979**	**17.72**	**24.74**	**30.43**
**LiNbO3**	**(1,1,-30)**	**13.63**	**82.94**	**9.634**	**9.3**	**1.702**	**15.79**	**21.27**	**26.49**
	Equiv. Refl.: (1,-2,-30), (2,-1,-30), (-1,2,-30), (-1,-1,-30), (-2,1,-30)								
**LiNbO3**	**(0,9,-12)**	**13.616**	**82.5**	**9.402**	**9**	**1.809**	**16.32**	**22.62**	**27.89**
	Equiv. Refl.: (9,-9,-12), (-9,0,-12)								

Ei = Cryst	13.734 Refl (h,k,l)	keV EB [keV]	ΘB [°]	∫ IR dΘ [µrad]	Width [µrad]	Ei cotΘ [meV/µrad]	ΔE [meV]	ΔEg 2m, 50µm [meV]	ΔEt 2m, 50µm [meV]
**LiNbO3**	**(2,7,16)**	**13.444**	**78.2**	**9.095**	**7.9**	**2.87**	**22.6**	**35.87**	**42.4**
	Equiv. Refl.: (7,-9,16), (9,-7,16), (-2,9,16), (-7,-2,16), (-9,2,16)								
**LiNbO3**	**(1,-9,-14)**	**13.429**	**77.9**	**8.89**	**7.8**	**2.944**	**22.91**	**36.8**	**43.35**
	Equiv. Refl.: (8,1,-14), (9,-1,-14), (-1,-8,-14), (-8,9,-14), (-9,8,-14)								
**LiNbO3**	**(5,6,-4)**	**13.384**	**77.05**	**8.75**	**7.6**	**3.159**	**23.94**	**39.49**	**46.18**
	Equiv. Refl.: (6,-11,-4), (11,-6,-4), (-5,11,-4), (-6,-5,-4), (-11,5,-4)								
**LiNbO3**	**(1,8,14)**	**13.429**	**77.9**	**8.721**	**7.8**	**2.944**	**22.91**	**36.8**	**43.35**
	Equiv. Refl.: (8,-9,14), (9,-8,14), (-1,9,14), (-8,-1,14), (-9,1,14)								
**LiNbO3**	**(2,-9,-16)**	**13.444**	**78.2**	**8.701**	**7.9**	**2.87**	**22.6**	**35.87**	**42.4**
	Equiv. Refl.: (7,2,-16), (9,-2,-16), (-2,-7,-16), (-7,9,-16), (-9,7,-16)								
**LiNbO3**	**(5,-11,4)**	**13.384**	**77.05**	**8.518**	**7.6**	**3.159**	**23.94**	**39.49**	**46.18**
	Equiv. Refl.: (6,5,4), (11,-5,4), (-5,-6,4)								
**LiNbO3**	**(-6,11,4)**	**13.384**	**77.05**	**8.517**	**7.6**	**3.159**	**23.94**	**39.49**	**46.18**
	Equiv. Refl.: (-11,6,4)								
**LiNbO3**	**(1,9,-2)**	**13.295**	**75.47**	**7.821**	**6.8**	**3.56**	**24.21**	**44.51**	**50.67**
	Equiv. Refl.: (9,-10,-2), (10,-9,-2), (-1,10,-2), (-9,-1,-2), (-10,1,-2)								
**LiNbO3**	**(1,-10,2)**	**13.295**	**75.47**	**7.67**	**6.8**	**3.56**	**24.21**	**44.51**	**50.67**
	Equiv. Refl.: (9,1,2), (10,-1,2), (-1,-9,2), (-9,10,2), (-10,9,2)								
**LiNbO3**	**(2,3,26)**	**13.112**	**72.68**	**7.151**	**6.2**	**4.282**	**26.54**	**53.52**	**59.74**
	Equiv. Refl.: (3,-5,26), (5,-3,26), (-2,5,26), (-3,-2,26), (-5,2,26)								
**LiNbO3**	**(0,-7,-20)**	**13.218**	**74.25**	**7.126**	**6.2**	**3.873**	**24.09**	**48.42**	**54.08**
	Equiv. Refl.: (7,0,-20), (-7,7,-20)								
**LiNbO3**	**(2,-5,-26)**	**13.112**	**72.68**	**7.121**	**6.2**	**4.282**	**26.54**	**53.52**	**59.74**
	Equiv. Refl.: (3,2,-26), (5,-2,-26), (-2,-3,-26), (-3,5,-26), (-5,3,-26)								
**LiNbO3**	**(-6,8,-20)**	**13.436**	**78.04**	**7.082**	**6.4**	**2.909**	**18.52**	**36.36**	**40.8**
	Equiv. Refl.: (-8,6,-20)								
**LiNbO3**	**(2,-8,-20)**	**13.436**	**78.04**	**7.081**	**6.4**	**2.909**	**18.52**	**36.36**	**40.8**
	Equiv. Refl.: (6,2,-20), (8,-2,-20), (-2,-6,-20)								
**Quartz**	**(-3,4,-11)**	**13.667**	**84.34**	**7.074**	**5.7**	**1.36**	**7.81**	**17**	**18.71**
	Equiv. Refl.: (-3,-1,11)								
**Quartz**	**(4,-3,11)**	**13.667**	**84.34**	**7.072**	**5.7**	**1.36**	**7.8**	**17**	**18.71**
**Quartz**	**(4,-1,-11)**	**13.667**	**84.34**	**7.071**	**5.7**	**1.36**	**7.8**	**17**	**18.71**
**Quartz**	**(-1,-3,-11)**	**13.667**	**84.34**	**7.068**	**5.7**	**1.36**	**7.8**	**17**	**18.71**
**Quartz**	**(-1,4,11)**	**13.667**	**84.34**	**7.066**	**5.7**	**1.36**	**7.8**	**17**	**18.71**
**LiNbO3**	**(4,-11,0)**	**13.409**	**77.52**	**7.037**	**6.4**	**3.04**	**19.35**	**38**	**42.64**
	Equiv. Refl.: (7,4,0), (11,-4,0), (-4,-7,0), (-7,11,0), (-11,7,0)								
**LiNbO3**	**(0,7,20)**	**13.218**	**74.25**	**7.001**	**6.2**	**3.873**	**24.09**	**48.42**	**54.08**
	Equiv. Refl.: (7,-7,20), (-7,0,20)								
**Sapph**	**(1,8,-10)**	**13.711**	**86.71**	**6.976**	**6.4**	**0.79**	**5.02**	**9.88**	**11.08**
	Equiv. Refl.: (1,-9,10), (8,1,10), (8,-9,-10), (9,-1,10), (9,-8,-10), (-1,9,-10), (-1,-8,10), (-8,9,10), (-8,-1,-10) (-9,1,-10), (-9,8,10)								
**Quartz**	**(3,1,-11)**	**13.667**	**84.34**	**6.917**	**5.7**	**1.36**	**7.81**	**17**	**18.71**
	Equiv. Refl.: (3,-4,11)								
**Quartz**	**(-4,1,11)**	**13.667**	**84.34**	**6.914**	**5.7**	**1.36**	**7.8**	**17**	**18.71**
	Equiv. Refl.: (-4,3,-11)								
**LiNbO3**	**(4,7,0)**	**13.409**	**77.52**	**6.912**	**6.4**	**3.04**	**19.35**	**38**	**42.64**
	Equiv. Refl.: (7,-11,0), (11,-7,0), (-4,11,0), (-7,-4,0), (-11,4,0)								
**Quartz**	**(1,3,11)**	**13.667**	**84.34**	**6.911**	**5.7**	**1.36**	**7.8**	**17**	**18.71**
**Quartz**	**(1,-4,-11)**	**13.667**	**84.34**	**6.909**	**5.7**	**1.36**	**7.8**	**17**	**18.71**
**LiNbO3**	**(1,-8,-18)**	**13.229**	**74.41**	**6.781**	**6**	**3.832**	**22.94**	**47.91**	**53.12**
	Equiv. Refl.: (7,1,-18), (8,-1,-18), (-1,-7,-18), (-7,8,-18), (-8,7,-18)								

Ei =	13.734	keV						ΔEg	ΔEt
**Cryst**	**Refl**	**EB**	**ΘB**	**∫ IR dΘ**	**Width**	**Ei cotΘ**	**ΔE**	**2m, 50µm**	**2m, 50µm**
	**(h,k,l)**	**[keV]**	**[°]**	**[µrad]**	**[µrad]**	**[meV/µrad]**	**[meV]**	**[meV]**	**[meV]**
**LiNbO3**	**(2,6,20)**	**13.436**	**78.04**	**6.766**	**6.4**	**2.909**	**18.52**	**36.36**	**40.8**
	Equiv. Refl.: (6,-8,20), (8,-6,20), (-2,8,20), (-6,-2,20), (-8,2,20)								
**LiNbO3**	**(1,7,18)**	**13.229**	**74.41**	**6.701**	**6**	**3.832**	**22.94**	**47.91**	**53.12**
	Equiv. Refl.: (7,-8,18), (8,-7,18), (-1,8,18), (-7,-1,18), (-8,1,18)								
**LiNbO3**	**(3,-10,10)**	**13.143**	**73.13**	**6.658**	**5.8**	**4.165**	**24.35**	**52.06**	**57.47**
	Equiv. Refl.: (7,3,10), (10,-3,10), (-3,-7,10), (-7,10,10), (-10,7,10)								
**LiNbO3**	**(-6,7,-22)**	**13.414**	**77.6**	**6.626**	**6**	**3.02**	**18.12**	**37.75**	**41.88**
	Equiv. Refl.: (-7,6,-22)								
**LiNbO3**	**(1,-7,-22)**	**13.414**	**77.6**	**6.625**	**6**	**3.02**	**18.12**	**37.75**	**41.88**
	Equiv. Refl.: (6,1,-22), (7,-1,-22), (-1,-6,-22)								
**LiNbO3**	**(3,7,-10)**	**13.143**	**73.13**	**6.62**	**5.8**	**4.165**	**24.35**	**52.06**	**57.47**
	Equiv. Refl.: (7,-10,-10), (10,-7,-10), (-3,10,-10), (-7,-3,-10), (-10,3,-10)								
**LiNbO3**	**(5,5,-12)**	**13.183**	**73.72**	**6.507**	**5.6**	**4.01**	**22.64**	**50.13**	**55**
	Equiv. Refl.: (5,-10,-12), (10,-5,-12), (-5,10,-12), (-5,-5,-12), (-10,5,-12)								
**LiNbO3**	**(4,-9,16)**	**13.005**	**71.25**	**6.405**	**5.5**	**4.662**	**25.82**	**58.28**	**63.74**
	Equiv. Refl.: (5,4,16), (9,-4,16), (-4,-5,16)								
**LiNbO3**	**(-5,9,16)**	**13.005**	**71.25**	**6.404**	**5.5**	**4.662**	**25.82**	**58.28**	**63.74**
	Equiv. Refl.: (-9,5,16)								
**LiNbO3**	**(1,6,22)**	**13.414**	**77.6**	**6.361**	**6**	**3.02**	**18.12**	**37.75**	**41.88**
	Equiv. Refl.: (6,-7,22), (7,-6,22), (-1,7,22), (-6,-1,22), (-7,1,22)								
**LiNbO3**	**(1,9,4)**	**13.384**	**77.05**	**6.357**	**5.8**	**3.159**	**18.26**	**39.49**	**43.51**
	Equiv. Refl.: (9,-10,4), (10,-9,4), (-1,10,4), (-9,-1,4), (-10,1,4)								
**LiNbO3**	**(4,5,-16)**	**13.005**	**71.25**	**6.253**	**5.5**	**4.662**	**25.82**	**58.28**	**63.74**
	Equiv. Refl.: (5,-9,-16), (9,-5,-16), (-4,9,-16), (-5,-4,-16), (-9,4,-16)								
**LiNbO3**	**(0,-8,-16)**	**13.226**	**74.37**	**6.247**	**5.3**	**3.842**	**20.37**	**48.03**	**52.17**
	Equiv. Refl.: (8,0,-16), (-8,8,-16)								
**LiNbO3**	**(2,7,-14)**	**12.99**	**71.05**	**6.241**	**5.3**	**4.715**	**25.2**	**58.94**	**64.1**
	Equiv. Refl.: (7,-9,-14), (9,-7,-14), (-2,9,-14), (-7,-2,-14), (-9,2,-14)								
**LiNbO3**	**(4,-10,-10)**	**12.921**	**70.18**	**6.216**	**5.4**	**4.95**	**26.52**	**61.87**	**67.32**
	Equiv. Refl.: (6,4,-10), (10,-4,-10), (-4,-6,-10), (-6,10,-10), (-10,6,-10)								
**LiNbO3**	**(5,5,12)**	**13.183**	**73.72**	**6.165**	**5.6**	**4.01**	**22.64**	**50.13**	**55**
	Equiv. Refl.: (5,-10,12), (10,-5,12), (-5,10,12), (-5,-5,12), (-10,5,12)								
**LiNbO3**	**(1,-10,-4)**	**13.384**	**77.05**	**6.158**	**5.8**	**3.159**	**18.26**	**39.49**	**43.51**
	Equiv. Refl.: (9,1,-4), (10,-1,-4), (-1,-9,-4), (-9,10,-4), (-10,9,-4)								
**Sapph**	**(0,9,0)**	**13.54**	**80.35**	**6.127**	**4.9**	**2.335**	**11.53**	**29.18**	**31.38**
	Equiv. Refl.: (0,-9,0), (9,0,0), (9,-9,0), (-9,0,0), (-9,9,0)								
**Sapph**	**(4,6,-8)**	**13.66**	**84.04**	**6.114**	**5.3**	**1.434**	**7.56**	**17.92**	**19.45**
	Equiv. Refl.: (4,-10,8), (6,4,8), (6,-10,-8), (10,-4,8), (10,-6,-8), (-4,10,-8), (-4,-6,8), (-6,10,8), (-6,-4,-8) (-10,4,-8), (-10,6,8)								
**LiNbO3**	**(1,-8,18)**	**13.229**	**74.41**	**6.095**	**5.5**	**3.832**	**21.04**	**47.91**	**52.32**
	Equiv. Refl.: (7,1,18), (8,-1,18), (-1,-7,18), (-7,8,18), (-8,7,18)								
**LiNbO3**	**(1,7,-18)**	**13.229**	**74.41**	**6.068**	**5.5**	**3.832**	**21.04**	**47.91**	**52.32**
	Equiv. Refl.: (7,-8,-18), (8,-7,-18), (-1,8,-18), (-7,-1,-18), (-8,1,-18)								
**LiNbO3**	**(4,6,10)**	**12.921**	**70.18**	**6.061**	**5.4**	**4.95**	**26.52**	**61.87**	**67.32**
	Equiv. Refl.: (6,-10,10), (10,-6,10), (-6,-4,10)								
**LiNbO3**	**(-4,10,10)**	**12.921**	**70.18**	**6.06**	**5.4**	**4.95**	**26.52**	**61.87**	**67.32**
	Equiv. Refl.: (-10,4,10)								
**Quartz**	**(1,-9,-5)**	**13.706**	**86.35**	**5.955**	**5.1**	**0.877**	**4.47**	**10.97**	**11.84**
**Quartz**	**(1,8,5)**	**13.706**	**86.35**	**5.954**	**5.1**	**0.877**	**4.47**	**10.97**	**11.84**
	Equiv. Refl.: (-9,1,5), (-9,8,-5)								

Ei =	13.734	keV						ΔEg	ΔEt
Cryst	Refl	EB	ΘB	∫ IR dΘ	Width	Ei cotΘ	ΔE	2m, 50μm	2m, 50μm
	(h,k,l)	[keV]	[°]	[μrad]	[μrad]	[meV/μrad]	[meV]	[meV]	[meV]
**Quartz**	**(8,-9,5)**	**13.706**	**86.35**	**5.949**	**5.1**	**0.877**	**4.47**	**10.97**	**11.84**
**Quartz**	**(8,1,-5)**	**13.706**	**86.35**	**5.948**	**5.1**	**0.877**	**4.47**	**10.97**	**11.84**
**LiNbO3**	**(2,-9,14)**	**12.99**	**71.05**	**5.918**	**5.3**	**4.715**	**25.2**	**58.94**	**64.1**
	Equiv. Refl.: (7,2,14), (9,-2,14), (-2,-7,14), (-7,9,14), (-9,7,14), (-1,9,5), (-1,-8,-5)								
**Quartz**	**(9,-1,-5)**	**13.706**	**86.35**	**5.917**	**5.1**	**0.877**	**4.47**	**10.97**	**11.84**
	Equiv. Refl.: (9,-8,5)								
**Quartz**	**(-8,9,-5)**	**13.706**	**86.35**	**5.913**	**5.1**	**0.877**	**4.47**	**10.97**	**11.84**
**Quartz**	**(-8,-1,5)**	**13.706**	**86.35**	**5.912**	**5.1**	**0.877**	**4.47**	**10.97**	**11.84**
**LiNbO3**	**(1,2,-28)**	**13.05**	**71.84**	**5.867**	**5.2**	**4.504**	**23.38**	**56.3**	**60.96**
	Equiv. Refl.: (2,-3,-28), (3,-2,-28), (-1,3,-28), (-2,-1,-28), (-3,1,-28)								
**Quartz**	**(-9,9,3)**	**13.557**	**80.79**	**5.84**	**4.7**	**2.227**	**10.4**	**27.84**	**29.72**
**Quartz**	**(0,9,-3)**	**13.557**	**80.79**	**5.839**	**4.7**	**2.227**	**10.4**	**27.84**	**29.72**
	Equiv. Refl.: (0,-9,3), (9,0,3), (9,-9,-3), (-9,0,-3)								
**LiNbO3**	**(1,-3,28)**	**13.05**	**71.84**	**5.803**	**5.2**	**4.504**	**23.38**	**56.3**	**60.96**
	Equiv. Refl.: (2,1,28), (3,-1,28), (-1,-2,28), (-2,3,28), (-3,2,28)								
**LiNbO3**	**(0,8,16)**	**13.226**	**74.37**	**5.73**	**5.3**	**3.842**	**20.37**	**48.03**	**52.17**
	Equiv. Refl.: (8,-8,16), (-8,0,16)								
**LiNbO3**	**(5,-11,-2)**	**13.295**	**75.47**	**5.631**	**4.9**	**3.56**	**17.36**	**44.51**	**47.77**
	Equiv. Refl.: (6,5,-2), (11,-5,-2), (-5,-6,-2), (-6,11,-2), (-11,6,-2)								
**LiNbO3**	**(3,5,-20)**	**13.218**	**74.25**	**5.549**	**4.6**	**3.873**	**17.8**	**48.42**	**51.58**
	Equiv. Refl.: (5,-8,-20), (8,-5,-20), (-3,8,-20), (-5,-3,-20), (-8,3,-20)								
**LiNbO3**	**(5,6,2)**	**13.295**	**75.47**	**5.535**	**4.9**	**3.56**	**17.36**	**44.51**	**47.77**
	Equiv. Refl.: (6,-11,2), (11,-6,2), (-5,11,2), (-6,-5,2), (-11,5,2)								
**LiNbO3**	**(3,-7,22)**	**12.974**	**70.85**	**5.397**	**4.6**	**4.77**	**22.09**	**59.62**	**63.58**
	Equiv. Refl.: (4,3,22), (7,-3,22), (-3,-4,22), (-4,7,22), (-7,4,22)								
**LiNbO3**	**(2,-10,-6)**	**13.023**	**71.49**	**5.371**	**4.6**	**4.598**	**21.31**	**57.48**	**61.3**
	Equiv. Refl.: (8,2,-6), (10,-2,-6), (-2,-8,-6), (-8,10,-6), (-10,8,-6)								
**LiNbO3**	**(3,4,-22)**	**12.974**	**70.85**	**5.245**	**4.6**	**4.77**	**22.09**	**59.62**	**63.58**
	Equiv. Refl.: (4,-7,-22), (7,-4,-22), (-3,7,-22), (-4,-3,-22), (-7,3,-22)								
**LiNbO3**	**(2,8,6)**	**13.023**	**71.49**	**5.222**	**4.6**	**4.598**	**21.31**	**57.48**	**61.3**
	Equiv. Refl.: (8,-10,6), (10,-8,6), (-2,10,6), (-8,-2,6), (-10,2,6)								
**Sapph**	**(4,5,14)**	**13.517**	**79.8**	**5.159**	**4**	**2.471**	**10**	**30.88**	**32.46**
	Equiv. Refl.: (4,-9,-14), (5,4,-14), (5,-9,14), (9,-4,-14), (9,-5,14), (-4,9,14), (-4,-5,-14), (-5,9,-14), (-5,-4,14) (-9,4,14), (-9,5,-14)								
**Quartz**	**(3,-8,8)**	**13.719**	**87.35**	**5.111**	**4.7**	**0.636**	**3.01**	**7.95**	**8.5**
**Quartz**	**(3,5,-8)**	**13.719**	**87.35**	**5.11**	**4.7**	**0.636**	**3.01**	**7.95**	**8.5**
**Quartz**	**(-8,3,-8)**	**13.719**	**87.35**	**5.109**	**4.7**	**0.636**	**3.01**	**7.95**	**8.5**
**Quartz**	**(-8,5,8)**	**13.719**	**87.35**	**5.108**	**4.7**	**0.636**	**3.01**	**7.95**	**8.5**
**Quartz**	**(5,-8,-8)**	**13.719**	**87.35**	**5.106**	**4.7**	**0.636**	**3.01**	**7.95**	**8.5**
**Quartz**	**(5,3,8)**	**13.719**	**87.35**	**5.105**	**4.7**	**0.636**	**3.01**	**7.95**	**8.5**
**Quartz**	**(-4,1,-11)**	**13.667**	**84.34**	**4.98**	**4**	**1.36**	**5.41**	**17**	**17.84**
**Quartz**	**(-4,3,11)**	**13.667**	**84.34**	**4.978**	**4**	**1.36**	**5.41**	**17**	**17.84**
**Quartz**	**(3,-4,-11)**	**13.667**	**84.34**	**4.974**	**4**	**1.36**	**5.41**	**17**	**17.84**
**Quartz**	**(3,1,11)**	**13.667**	**84.34**	**4.972**	**4**	**1.36**	**5.41**	**17**	**17.84**
**Quartz**	**(1,3,-11)**	**13.667**	**84.34**	**4.967**	**4**	**1.36**	**5.4**	**17**	**17.84**
**Quartz**	**(1,-4,11)**	**13.667**	**84.34**	**4.965**	**4**	**1.36**	**5.4**	**17**	**17.84**
**LiNbO3**	**(2,-10,6)**	**13.023**	**71.49**	**4.945**	**4.2**	**4.598**	**19.48**	**57.48**	**60.69**
	Equiv. Refl.: (8,2,6), (10,-2,6), (-2,-8,6), (-8,10,6), (-10,8,6)								
**Quartz**	**(2,8,2)**	**13.549**	**80.59**	**4.939**	**3.7**	**2.277**	**8.47**	**28.46**	**29.69**
	Equiv. Refl.: (2,-10,-2), (-3,8,-8)								

Ei =	13.734	keV						ΔEg	ΔEt
Cryst	Refl	EB	ΘB	∫ IR dΘ	Width	Ei cotΘ	ΔE	2m, 50µm	2m, 50µm
	(h,k,l)	[keV]	[°]	[µrad]	[µrad]	[meV/µrad]	[meV]	[meV]	[meV]
Quartz	(8,2,-2)	13.549	80.59	4.938	3.7	2.277	8.47	28.46	29.69
	Equiv. Refl.: (-3,-5,8), (-10,2,2), (-10,8,-2)								
Quartz	(8,-10,2)	13.549	80.59	4.937	3.7	2.277	8.47	28.46	29.69
Quartz	(8,-3,8)	13.719	87.35	4.936	4.7	0.636	3.01	7.95	8.5
Quartz	(8,-5,-8)	13.719	87.35	4.935	4.7	0.636	3.01	7.95	8.5
Quartz	(-5,8,8)	13.719	87.35	4.933	4.7	0.636	3.01	7.95	8.5
Quartz	(-5,-3,-8)	13.719	87.35	4.932	4.7	0.636	3.01	7.95	8.5
LiNbO3	(2,8,-6)	13.023	71.49	4.902	4.2	4.598	19.48	57.48	60.69
	Equiv. Refl.: (8,-10,-6), (10,-8,-6), (-2,10,-6), (-8,-2,-6), (-10,2,-6)								
LiNbO3	(3,-8,20)	13.218	74.25	4.883	4.6	3.873	17.8	48.42	51.58
	Equiv. Refl.: (5,3,20), (8,-3,20), (-3,-5,20), (-5,8,20), (-8,5,20)								
LiNbO3	(3,3,-24)	12.938	70.39	4.872	4.1	4.892	20.09	61.15	64.37
	Equiv. Refl.: (3,-6,-24), (6,-3,-24), (-3,6,-24), (-3,-3,-24), (-6,3,-24)								
Sapph	(1,4,24)	13.37	76.77	4.816	3.7	3.228	11.94	40.36	42.08
	Equiv. Refl.: (1,4,-24), (1,-5,24), (1,-5,-24), (4,1,24), (4,1,-24), (4,-5,24), (4,-5,-24), (5,-1,24), (5,-1,-24) (5,-4,24), (5,-4,-24), (-1,5,24), (-1,5,-24), (-1,-4,24), (-1,-4,-24), (-4,5,24), (-4,5,-24), (-4,-1,24) (-4,-1,-24), (-5,1,24), (-5,1,-24), (-5,4,24), (-5,4,-24)								
Sapph	(3,4,20)	13.224	74.33	4.757	3.6	3.852	13.85	48.16	50.11
	Equiv. Refl.: (3,-7,-20), (4,3,-20), (4,-7,20), (7,-3,-20), (7,-4,20), (-3,7,20), (-3,-4,-20), (-4,7,-20), (-4,-3,20) (-7,3,20), (-7,4,-20)								
Quartz	(10,-2,-2)	13.549	80.59	4.741	3.7	2.277	8.47	28.46	29.69
	Equiv. Refl.: (10,-8,2), (-2,10,2), (-2,-8,-2)								
Quartz	(-8,10,-2)	13.549	80.59	4.74	3.7	2.277	8.47	28.46	29.69
	Equiv. Refl.: (-8,-2,2)								
LiNbO3	(3,3,24)	12.938	70.39	4.696	4.1	4.892	20.09	61.15	64.37
	Equiv. Refl.: (3,-6,24), (6,-3,24), (-3,6,24), (-3,-3,24), (-6,3,24)								
Quartz	(7,3,-3)	13.399	77.33	4.616	3.6	3.089	11.1	38.61	40.17
	Equiv. Refl.: (7,-10,3), (-10,3,3), (-10,7,-3)								
Quartz	(3,7,3)	13.399	77.33	4.615	3.6	3.089	11.09	38.61	40.17
	Equiv. Refl.: (3,-10,-3)								
Quartz	(4,-1,11)	13.667	84.34	4.603	4	1.36	5.41	17	17.84
Quartz	(4,-3,-11)	13.667	84.34	4.602	4	1.36	5.41	17	17.84
Quartz	(-3,4,11)	13.667	84.34	4.598	4	1.36	5.41	17	17.84
Quartz	(10,-3,-3)	13.399	77.33	4.596	3.6	3.089	11.1	38.61	40.17
	Equiv. Refl.: (10,-7,3), (-3,-1,-11), (-7,10,-3), (-7,-3,3)								
Quartz	(-3,10,3)	13.399	77.33	4.595	3.6	3.089	11.09	38.61	40.17
	Equiv. Refl.: (-3,-7,-3)								
Quartz	(-1,-3,11)	13.667	84.34	4.59	4	1.36	5.4	17	17.84
Quartz	(-1,4,-11)	13.667	84.34	4.589	4	1.36	5.4	17	17.84
Sapph	(0,2,28)	13.699	85.89	4.369	4.1	0.987	4.04	12.34	12.98
	Equiv. Refl.: (0,-2,-28), (2,0,-28), (2,-2,28), (-2,0,28), (-2,2,-28)								
Quartz	(10,-2,1)	13.403	77.39	4.051	3.1	3.073	9.38	38.41	39.54
	Equiv. Refl.: (10,-8,-1), (-2,10,-1), (-2,-8,1), (-8,10,1), (-8,-2,-1)								
Quartz	(2,8,-1)	13.403	77.39	3.995	3.1	3.073	9.38	38.41	39.54
	Equiv. Refl.: (2,-10,1), (8,2,1), (8,-10,-1), (-10,2,-1), (-10,8,1)								
Quartz	(-9,8,5)	13.706	86.35	3.735	3.4	0.877	2.99	10.97	11.37
Quartz	(-9,1,-5)	13.706	86.35	3.733	3.4	0.877	2.99	10.97	11.37
Quartz	(1,8,-5)	13.706	86.35	3.731	3.4	0.877	2.99	10.97	11.37
	Equiv. Refl.: (8,-9,-5)								

Ei = Cryst	13.734 Refl (h,k,l)	keV EB [keV]	ΘB [°]	∫ IR dΘ [μrad]	Width [μrad]	Ei cotΘ [meV/μrad]	ΔE [meV]	ΔEg 2m, 50μm [meV]	ΔEt 2m, 50μm [meV]
**Sapph**	**(3,7,2)**	**13.406**	**77.45**	**3.73**	**3**	**3.058**	**9.16**	**38.23**	**39.31**
	Equiv. Refl.: (3,-10,-2), (7,3,-2), (7,-10,2), (10,-3,-2), (10,-7,2), (-3,10,2), (-3,-7,-2), (-7,10,-2), (-7,-3,2) (-10,3,2), (-10,7,-2), (1,-9,5), (8,1,5)								
**Sapph**	**(5,5,6)**	**13.34**	**76.24**	**3.695**	**2.9**	**3.364**	**9.84**	**42.05**	**43.19**
	Equiv. Refl.: (5,5,-6), (5,-10,6), (5,-10,-6), (10,-5,6), (10,-5,-6), (-5,10,6), (-5,10,-6), (-5,-5,6), (-5,-5,-6) (-10,5,6), (-10,5,-6)								
**Quartz**	**(9,-8,-5)**	**13.706**	**86.35**	**3.684**	**3.4**	**0.877**	**2.99**	**10.97**	**11.37**
**Quartz**	**(9,-1,5)**	**13.706**	**86.35**	**3.683**	**3.4**	**0.877**	**2.99**	**10.97**	**11.37**
**Quartz**	**(-8,9,5)**	**13.706**	**86.35**	**3.681**	**3.4**	**0.877**	**2.99**	**10.97**	**11.37**
	Equiv. Refl.: (-8,-1,-5)								
**Quartz**	**(-1,9,-5)**	**13.706**	**86.35**	**3.679**	**3.4**	**0.877**	**2.99**	**10.97**	**11.37**
	Equiv. Refl.: (-1,-8,5)								
**Quartz**	**(-10,4,4)**	**13.505**	**79.52**	**3.608**	**2.8**	**2.54**	**7.24**	**31.75**	**32.57**
	Equiv. Refl.: (-10,6,-4)								
**Quartz**	**(6,4,-4)**	**13.505**	**79.52**	**3.606**	**2.8**	**2.54**	**7.23**	**31.75**	**32.57**
	Equiv. Refl.: (6,-10,4)								
**Quartz**	**(4,6,4)**	**13.505**	**79.52**	**3.605**	**2.8**	**2.54**	**7.23**	**31.75**	**32.57**
	Equiv. Refl.: (4,-10,-4)								
**Quartz**	**(10,-4,-4)**	**13.505**	**79.52**	**3.571**	**2.8**	**2.54**	**7.24**	**31.75**	**32.57**
	Equiv. Refl.: (10,-6,4)								
**Quartz**	**(-6,10,-4)**	**13.505**	**79.52**	**3.569**	**2.8**	**2.54**	**7.23**	**31.75**	**32.57**
	Equiv. Refl.: (-6,-4,4)								
**Quartz**	**(-4,10,4)**	**13.505**	**79.52**	**3.568**	**2.8**	**2.54**	**7.23**	**31.75**	**32.57**
	Equiv. Refl.: (-4,-6,-4)								
**LiNbO3**	**(2,7,-17)**	**13.687**	**85.25**	**3.548**	**5.1**	**1.14**	**5.83**	**14.25**	**15.4**
	Equiv. Refl.: (2,-9,17), (7,2,17), (7,-9,-17), (9,-2,17), (9,-7,-17), (-2,9,-17), (-2,-7,17), (-7,9,17), (-7,-2,-17) (-9,2,-17), (-9,7,17)								
**Sapph**	**(1,8,-4)**	**12.995**	**71.12**	**3.406**	**2.6**	**4.698**	**12.35**	**58.72**	**60.01**
	Equiv. Refl.: (1,-9,4), (8,1,4), (8,-9,-4), (9,-1,4), (9,-8,-4), (-1,9,-4), (-1,-8,4), (-8,9,4), (-8,-1,-4) (-9,1,-4), (-9,8,4)								
**Sapph**	**(2,6,-16)**	**13.267**	**75.01**	**3.261**	**2.6**	**3.677**	**9.56**	**45.96**	**46.94**
	Equiv. Refl.: (2,-8,16), (6,2,16), (6,-8,-16), (8,-2,16), (8,-6,-16), (-2,8,-16), (-2,-6,16), (-6,8,16), (-6,-2,-16) (-8,2,-16), (-8,6,16)								
**Quartz**	**(4,1,10)**	**13.272**	**75.1**	**3.096**	**2.4**	**3.656**	**8.66**	**45.69**	**46.51**
	Equiv. Refl.: (4,-5,-10)								
**Quartz**	**(1,4,-10)**	**13.272**	**75.1**	**3.094**	**2.4**	**3.656**	**8.65**	**45.69**	**46.51**
**Quartz**	**(1,-5,10)**	**13.272**	**75.1**	**3.093**	**2.4**	**3.656**	**8.65**	**45.69**	**46.51**
**Quartz**	**(-5,1,-10)**	**13.272**	**75.1**	**3.091**	**2.4**	**3.656**	**8.65**	**45.69**	**46.5**
	Equiv. Refl.: (-5,4,10)								
**Quartz**	**(2,-7,8)**	**12.923**	**70.21**	**3.041**	**2.5**	**4.943**	**12.59**	**61.79**	**63.06**
**Quartz**	**(2,5,-8)**	**12.923**	**70.21**	**3.04**	**2.5**	**4.943**	**12.59**	**61.79**	**63.06**
	Equiv. Refl.: (-7,2,-8), (-7,5,8)								
**Quartz**	**(5,2,8)**	**12.923**	**70.21**	**3.039**	**2.5**	**4.943**	**12.59**	**61.79**	**63.06**
	Equiv. Refl.: (5,-7,-8)								
**Quartz**	**(-2,7,-8)**	**12.923**	**70.21**	**3.038**	**2.5**	**4.943**	**12.59**	**61.79**	**63.06**
	Equiv. Refl.: (-2,-5,8)								
**Quartz**	**(7,-2,8)**	**12.923**	**70.21**	**3.037**	**2.5**	**4.943**	**12.59**	**61.79**	**63.06**
	Equiv. Refl.: (7,-5,-8)								
**Quartz**	**(-5,7,8)**	**12.923**	**70.21**	**3.036**	**2.5**	**4.943**	**12.59**	**61.79**	**63.06**
	Equiv. Refl.: (-5,-2,-8)								

Ei = Cryst	13.734 Refl (h,k,l)	keV EB [keV]	ΘB [°]	∫ IR dΘ [μrad]	Width [μrad]	Ei cotΘ [meV/μrad]	ΔE [meV]	ΔEg 2m, 50μm [meV]	ΔEt 2m, 50μm [meV]
**Quartz**	**(8,0,-5)**	**12.99**	**71.06**	**3.035**	**2.6**	**4.713**	**12.09**	**58.91**	**60.14**
	Equiv. Refl.: (8,-8,5), (-8,0,5)								
**Quartz**	**(0,8,5)**	**12.99**	**71.06**	**3.034**	**2.6**	**4.713**	**12.08**	**58.91**	**60.14**
	Equiv. Refl.: (0,-8,-5), (-8,8,-5)								
**Quartz**	**(-4,-1,-10)**	**13.272**	**75.1**	**3.03**	**2.4**	**3.656**	**8.66**	**45.69**	**46.51**
**Quartz**	**(-4,5,10)**	**13.272**	**75.1**	**3.029**	**2.4**	**3.656**	**8.66**	**45.69**	**46.51**
**Quartz**	**(-1,-4,10)**	**13.272**	**75.1**	**3.028**	**2.4**	**3.656**	**8.65**	**45.69**	**46.51**
**Quartz**	**(-1,5,-10)**	**13.272**	**75.1**	**3.027**	**2.4**	**3.656**	**8.65**	**45.69**	**46.51**
**Quartz**	**(5,-1,10)**	**13.272**	**75.1**	**3.025**	**2.4**	**3.656**	**8.65**	**45.69**	**46.5**
	Equiv. Refl.: (5,-4,-10)								
**Quartz**	**(-2,-8,2)**	**13.549**	**80.59**	**3.018**	**2.4**	**2.277**	**5.41**	**28.46**	**28.97**
**Quartz**	**(-2,10,-2)**	**13.549**	**80.59**	**3.017**	**2.4**	**2.277**	**5.41**	**28.46**	**28.97**
	Equiv. Refl.: (-8,10,2), (-8,-2,-2)								
**Quartz**	**(10,-2,2)**	**13.549**	**80.59**	**3.016**	**2.4**	**2.277**	**5.41**	**28.46**	**28.97**
	Equiv. Refl.: (10,-8,-2)								
**Quartz**	**(2,8,-2)**	**13.549**	**80.59**	**2.995**	**2.4**	**2.277**	**5.41**	**28.46**	**28.97**
	Equiv. Refl.: (2,-10,2)								
**Quartz**	**(8,2,2)**	**13.549**	**80.59**	**2.994**	**2.4**	**2.277**	**5.41**	**28.46**	**28.97**
	Equiv. Refl.: (8,-10,-2)								
**Quartz**	**(-10,2,-2)**	**13.549**	**80.59**	**2.993**	**2.4**	**2.277**	**5.41**	**28.46**	**28.97**
	Equiv. Refl.: (-10,8,2)								
**Sapph**	**(0,1,-28)**	**13.449**	**78.3**	**2.882**	**2.3**	**2.845**	**6.57**	**35.56**	**36.16**
	Equiv. Refl.: (0,-1,28), (1,0,28), (1,-1,-28), (-1,0,-28), (-1,1,28)								
**Sapph**	**(3,5,16)**	**13.008**	**71.29**	**2.806**	**2.1**	**4.651**	**9.89**	**58.13**	**58.97**
	Equiv. Refl.: (3,-8,-16), (5,3,-16), (5,-8,16), (8,-3,-16), (8,-5,16), (-3,8,16), (-3,-5,-16), (-5,8,-16), (-5,-3,16) (-8,3,16), (-8,5,-16)								
**Quartz**	**(-3,5,-10)**	**13.111**	**72.68**	**2.782**	**2.4**	**4.284**	**10.12**	**53.55**	**54.5**
	Equiv. Refl.: (-3,-2,10)								
**Quartz**	**(5,-2,-10)**	**13.111**	**72.68**	**2.781**	**2.4**	**4.284**	**10.12**	**53.55**	**54.5**
	Equiv. Refl.: (5,-3,10), (-2,5,10), (-2,-3,-10)								
**Quartz**	**(3,2,-10)**	**13.111**	**72.68**	**2.753**	**2.4**	**4.284**	**10.12**	**53.55**	**54.5**
	Equiv. Refl.: (3,-5,10), (-5,2,10)								
**Quartz**	**(2,3,10)**	**13.111**	**72.68**	**2.752**	**2.4**	**4.284**	**10.12**	**53.55**	**54.5**
	Equiv. Refl.: (2,-5,-10), (-5,3,-10)								
**Sapph**	**(2,7,10)**	**13.207**	**74.07**	**2.608**	**2.3**	**3.919**	**9.14**	**48.99**	**49.83**
	Equiv. Refl.: (2,-9,-10), (7,2,-10), (7,-9,10), (9,-2,-10), (9,-7,10), (-2,9,10), (-2,-7,-10), (-7,9,-10), (-7,-2,10) (-9,2,10), (-9,7,-10)								
**Quartz**	**(-9,2,-5)**	**13.233**	**74.48**	**2.325**	**2**	**3.814**	**7.46**	**47.67**	**48.25**
	Equiv. Refl.: (-9,7,5)								
**Quartz**	**(2,-9,5)**	**13.233**	**74.48**	**2.324**	**2**	**3.814**	**7.46**	**47.67**	**48.25**
	Equiv. Refl.: (7,2,5), (7,-9,-5)								
**Quartz**	**(2,7,-5)**	**13.233**	**74.48**	**2.323**	**2**	**3.814**	**7.46**	**47.67**	**48.25**
**Quartz**	**(3,0,-11)**	**13.353**	**76.47**	**2.281**	**2**	**3.305**	**6.53**	**41.31**	**41.82**
	Equiv. Refl.: (3,-3,11), (-3,0,11), (-3,3,-11)								
**Quartz**	**(0,3,11)**	**13.353**	**76.47**	**2.275**	**2**	**3.305**	**6.52**	**41.31**	**41.82**
	Equiv. Refl.: (0,-3,-11)								
**Quartz**	**(9,-2,5)**	**13.233**	**74.48**	**2.239**	**2**	**3.814**	**7.46**	**47.67**	**48.25**
**Quartz**	**(9,-7,-5)**	**13.233**	**74.48**	**2.238**	**2**	**3.814**	**7.46**	**47.67**	**48.25**
**Quartz**	**(-2,9,-5)**	**13.233**	**74.48**	**2.237**	**2**	**3.814**	**7.46**	**47.67**	**48.25**
	Equiv. Refl.: (-2,-7,5), (-7,9,5), (-7,-2,-5)								

Ei =	13.734	keV						ΔEg	ΔEt
Cryst	Refl	EB	ΘB	∫ IR dΘ	Width	Ei cotΘ	ΔE	2m, 50µm	2m, 50µm
	(h,k,l)	[keV]	[°]	[µrad]	[µrad]	[meV/µrad]	[meV]	[meV]	[meV]
**Sapph**	**(5,5,9)**	**13.719**	**87.28**	**2.215**	**3**	**0.652**	**1.96**	**8.15**	**8.38**
	Equiv. Refl.: (5,5,-9), (5,-10,9), (5,-10,-9), (10,-5,9), (10,-5,-9), (-5,10,9), (-5,10,-9), (-5,-5,9), (-5,-5,-9) (-10,5,9), (-10,5,-9)								
**LiNbO3**	**(1,9,7)**	**13.629**	**82.9**	**2.135**	**3.3**	**1.71**	**5.66**	**21.38**	**22.11**
	Equiv. Refl.: (1,-10,-7), (9,1,-7), (9,-10,7), (10,-1,-7), (10,-9,7), (-1,10,7), (-1,-9,-7), (-9,10,-7), (-9,-1,7) (-10,1,7), (-10,9,-7)								
**Sapph**	**(2,6,17)**	**13.547**	**80.54**	**1.888**	**2.1**	**2.289**	**4.77**	**28.61**	**29**
	Equiv. Refl.: (2,-8,-17), (6,2,-17), (6,-8,17), (8,-2,-17), (8,-6,17), (-2,8,17), (-2,-6,-17), (-6,8,-17), (-6,-2,17) (-8,2,17), (-8,6,-17)								
**Quartz**	**(3,-1,-11)**	**13.193**	**73.86**	**1.832**	**1.9**	**3.974**	**7.4**	**49.67**	**50.22**
**Quartz**	**(3,-2,11)**	**13.193**	**73.86**	**1.831**	**1.9**	**3.974**	**7.4**	**49.67**	**50.22**
	Equiv. Refl.: (-1,3,11), (-1,-2,-11), (-2,3,-11), (-2,-1,11)								
**Quartz**	**(1,2,11)**	**13.193**	**73.86**	**1.801**	**1.9**	**3.974**	**7.4**	**49.67**	**50.22**
	Equiv. Refl.: (1,-3,-11), (2,-3,11), (-3,1,11), (-3,2,-11)								
**Quartz**	**(2,1,-11)**	**13.193**	**73.86**	**1.8**	**1.9**	**3.974**	**7.39**	**49.67**	**50.22**
**Quartz**	**(10,-3,3)**	**13.399**	**77.33**	**1.781**	**1.8**	**3.089**	**5.48**	**38.61**	**38.99**
	Equiv. Refl.: (10,-7,-3), (-7,10,3), (-7,-3,-3)								
**Quartz**	**(-3,10,-3)**	**13.399**	**77.33**	**1.78**	**1.8**	**3.089**	**5.48**	**38.61**	**38.99**
	Equiv. Refl.: (-3,-7,3)								
**Quartz**	**(-2,8,-7)**	**13.223**	**74.32**	**1.752**	**1.8**	**3.854**	**6.86**	**48.18**	**48.66**
	Equiv. Refl.: (-6,8,7), (-6,-2,-7)								
**Quartz**	**(8,-6,-7)**	**13.223**	**74.32**	**1.751**	**1.8**	**3.854**	**6.85**	**48.18**	**48.66**
	Equiv. Refl.: (-2,-6,7)								
**Quartz**	**(8,-2,7)**	**13.223**	**74.32**	**1.75**	**1.8**	**3.854**	**6.85**	**48.18**	**48.66**
**Sapph**	**(4,6,-2)**	**13.15**	**73.23**	**1.709**	**1.8**	**4.139**	**7.44**	**51.73**	**52.26**
	Equiv. Refl.: (4,-10,2), (6,4,2), (6,-10,-2), (10,-4,2), (10,-6,-2), (-4,10,-2), (-4,-6,2), (-6,10,2), (-6,-4,-2) (-10,4,-2), (-10,6,2)								
**Sapph**	**(1,1,27)**	**13.148**	**73.2**	**1.7**	**1.8**	**4.147**	**7.42**	**51.84**	**52.37**
	Equiv. Refl.: (1,1,-27), (1,-2,27), (1,-2,-27), (2,-1,27), (2,-1,-27), (-1,2,27), (-1,2,-27), (-1,-1,27), (-1,-1,-27) (-2,1,27), (-2,1,-27), (2,6,-7), (2,-8,7), (6,2,7), (6,-8,-7)								
**Quartz**	**(-8,2,-7)**	**13.223**	**74.32**	**1.699**	**1.8**	**3.854**	**6.85**	**48.18**	**48.66**
	Equiv. Refl.: (-8,6,7)								
**Quartz**	**(-4,7,9)**	**13.606**	**82.16**	**1.67**	**1.8**	**1.891**	**3.4**	**23.64**	**23.89**
**Quartz**	**(-4,-3,-9)**	**13.606**	**82.16**	**1.669**	**1.8**	**1.891**	**3.4**	**23.64**	**23.89**
**Quartz**	**(-3,7,-9)**	**13.606**	**82.16**	**1.668**	**1.8**	**1.891**	**3.4**	**23.64**	**23.89**
**Quartz**	**(-3,-4,9)**	**13.606**	**82.16**	**1.667**	**1.8**	**1.891**	**3.4**	**23.64**	**23.89**
**Quartz**	**(7,-3,9)**	**13.606**	**82.16**	**1.665**	**1.8**	**1.891**	**3.39**	**23.64**	**23.89**
	Equiv. Refl.: (7,-4,-9)								
**Sapph**	**(1,8,-7)**	**13.281**	**75.24**	**1.645**	**1.8**	**3.618**	**6.36**	**45.23**	**45.67**
	Equiv. Refl.: (1,-9,7), (8,1,7), (8,-9,-7), (9,-1,7), (9,-8,-7), (-1,9,-7), (-1,-8,7), (-8,9,7), (-8,-1,-7) (-9,1,-7), (-9,8,7)								
**Quartz**	**(0,3,-11)**	**13.353**	**76.47**	**1.629**	**1.7**	**3.305**	**5.64**	**41.31**	**41.69**
	Equiv. Refl.: (0,-3,11), (3,0,11), (3,-3,-11), (-3,0,-11), (-3,3,11)								
**Quartz**	**(0,2,11)**	**12.949**	**70.54**	**1.626**	**1.7**	**4.853**	**8.06**	**60.66**	**61.2**
	Equiv. Refl.: (0,-2,-11)								
[truncated: 1,708,672 more chars]
